# Medication errors in community pharmacies: a systematic review of the international literature

**DOI:** 10.1371/journal.pone.0322392

**Published:** 2025-05-20

**Authors:** Maguy Saffouh El Hajj, Rayah Asiri, Andy Husband, Adam Todd

**Affiliations:** 1 Clinical Pharmacy and Practice Department, College of Pharmacy, QU Health, Qatar University, Doha, Qatar; 2 School of Pharmacy, Newcastle University, Newcastle upon Tyne, United Kingdom; 3 School of Pharmacy, King Khalid University, Abha, Saudi Arabia; 4 Newcastle NIHR Patient Safety Research Collaborative (PSRC, School of Pharmacy, Newcastle University, Newcastle upon Tyne, United Kingdom; Lahore Medical and Dental College, PAKISTAN

## Abstract

**Introduction:**

Since the 1999 report by the Institute of Medicine (IOM) ‘To Err is Human.’, medication safety has become a public health concern due to its impact on preventable harm and healthcare costs. While formal systems in hospitals exist to address medication errors, there is less evidence in community pharmacies. The objective of this systematic review is to synthesise and critically appraise the international evidence about the prevalence, nature, and severity of medication errors in community pharmacies.

**Materials and methods:**

A systematic review was conducted for the literature published from January 1995 to December 2023. The choice of this date range was made to include a sufficient breadth of research conducted both preceding and subsequent to the publication of the IOM report. Various databases were used, supplemented by manual searches of bibliographies and grey literature. Studies were selected through a rigorous screening process. Data extraction and quality assessment were carried out using structured tools. Narrative and descriptive synthesis was conducted by geographical regions.

**Results:**

56898 potentially eligible studies were identified, yielding 73 included studies. Most studies were conducted in Europe and Central Asia and North America with few conducted in other regions. Most studies focused on prescribing and dispensing errors, with fewer studies addressing errors in other stages of the medication use process. Variations in error rates and types were observed across regions, which made calculating a global rate of errors challenging. Very few studies assessed the severity of medication errors with the majority of these being conducted in Europe and Central Asia and North America. Risk of bias varied, with selection and identification bias being common in all regions.

**Conclusions:**

This review underscores the need to assess medication safety in regions with limited pharmacy access and advocate for a standardised global reporting framework to streamline data analysis. Additionally, it implies that investigating the types and severity of medication errors is imperative. Addressing these gaps through rigorous quantitative and qualitative research could inform policy-making and implementation of strategies to enhance patient safety in community pharmacies.

## Introduction

In 1999, the Institute of Medicine (IOM) in United States (US) published the landmark report ‘To Err Is Human: Building a Safer Health System’ suggesting that medical errors account for an estimated 44,000–98,000 deaths each year [1]. Since the publication of this report, patient safety has emerged as an increasingly important public health issue. Patient safety initiatives aim to prevent and decrease risks, errors and harm that can occur to patients, while they receive healthcare [2]. Within the topic of patient safety, medication safety is regularly considered an important area [3].

According to the US National Coordinating Council for Medication Error Reporting and Prevention (NCC MERP), medication errors are defined as ‘any preventable event that may cause or lead to inappropriate medication use or patient harm while the medication is in the control of the healthcare professional, patient, or consumer. Such events may be related to professional practice, healthcare products, procedures, and systems’ [4]. Medication errors are one of the top causes of avoidable harm in healthcare systems worldwide. For example, in the US, they result in an estimated cost of around $42 billion United States Dollars (USD) annually [3], while in England, it has been estimated that 237 million medication errors occur yearly throughout the various stages of the medication use process [5]. When a medication error results in actual patient harm, it is considered an adverse drug event (ADE) [6]. Studies suggest around 1 in 100 medication errors results in an ADE, with 7 in 100 errors having the potential to cause actual patient harm [6]. In addition to harm from medication errors, medication overdose or medication failure, ADEs can also include adverse drug reactions (ADRs) [7]. An ADR is defined by the World Health Organisation (WHO) as ‘a response to a drug which is noxious and unintended, and which occurs at doses normally used in humans for the prophylaxis, diagnosis, or therapy of a disease’ [8]. ADEs are a significant cause of hospital admissions and emergency department (ED) visits; they are also associated with increased mortality and morbidity and higher costs of healthcare [9,10]. For instance, data from 60 nationally representative US EDs estimates there were 6.1 ED visits related to medication harms per 1000 population per year; 38.6% of these ED visits resulted in hospitalisation [9]. Another example from a different healthcare setting is the United Kingdom NHS (National Health Service) where avoidable ADRs have been estimated to cost £98.5 million annually, causing around 1,700 deaths [5].

The majority of the literature relating to medication errors originates from secondary care and this literature has encouraged the design and implementation of several effective programs for error prevention and mitigation [10–16]. Despite the design and implementation of these programs, medication errors are still a cause for concern in primary care, including community pharmacies, which are highly accessible healthcare facilities that the public can visit without the need for a referral or appointment [17]. Previous work has shown that medication errors can occur at any stage of the medication use process in a community pharmacy setting [18]. This multifaceted process, through which a medication travels from the pharmacy to the patient, consists of Prescribing: It Involves assessing the patient’s condition, selecting the most appropriate medication, and writing a prescription. 2) Transcribing and Documentation: Where applicable, the prescription is transcribed and recorded manually or electronically in the patient’s medical record 3) Dispensing: The pharmacist reviews and verifies the appropriateness of the prescribed medication before preparing and dispensing it, ensuring proper labelling and patient counselling. 4) Administration: The medication is administered while adhering to the Five Rights of Medication Administration (right patient, drug, dose, route, and time) 5) Monitoring – The patient is assessed for medication safety and effectiveness [18].

While formal systems exist in hospital settings to report, monitor and learn from medication errors, they may not always be available in community pharmacies, which are often considered the last check before a patient uses a medication in primary care [19]. Hence, the failure to detect or prevent medication errors in this setting can substantially increase the risk of harm to the patient.

To detect and execute strategies to optimise the medication-use process and prevent the occurrence of ADEs in community pharmacies, information about medication errors in this sector is needed. While there are many individual studies and a few systematic reviews published on medications errors in community pharmacies,[20–24] there is lack of synthesised information on the rate, types, nature and severity of medication errors in this setting globally. Moreover, information about where these errors occur in the medication use process in community pharmacies is also not available.

The aim of this study was, therefore, to review the existing international evidence in relation to medication safety problems and medication errors in community pharmacies. The study objective was to synthesise and critically appraise the available international evidence about the prevalence, nature, and severity of medication safety problems and medication errors in community pharmacies.

## Materials and methods

### Registration

The protocol was registered and is available on the International Prospective Register of Systematic Reviews (PROSPERO) at the Centre for Reviews and Dissemination, University of York, United Kingdom. [PROSPERO 2023 CRD42023390727).

### Study design

A systematic review of the international literature was conducted and reported as per the PRISMA (The Preferred Reporting Items for Systematic reviews and Meta-Analyses) 2020 Statement [25].

### Eligibility criteria

The inclusion criteria were kept deliberately broad to identify all relevant studies. The inclusion criteria were conceptualised using the PECOS (Population, Exposure, Comparison, Outcome, Study Design) framework, as follows:

– P: Adult and paediatric patients in a community pharmacy setting.
– E: Over the counter or prescription medicines
– C: Not applicable

O: Medication errors/safety problems, including those related to: (1) prescribing, (2) transcribing and documenting, (3) dispensing, (4) administering, and (5) monitoring.

S: Quantitative studies

Reviews, letters, editorials, commentaries, qualitative and intervention studies (*i.e.,* randomised controlled trials, non-randomised controlled trials, controlled before and after studies looking to report the effectiveness of an intervention to promote safety) were excluded. Articles were not excluded from the systematic review based on study quality. Language restrictions were not applied. Studies were excluded if published before January 1995. The choice of the ‘January 1995’ date was made to include a sufficient breadth of research conducted both preceding and after the publication of the 1999 IOM report.

A community pharmacy setting was defined as ‘a healthcare facility that provides pharmaceutical and cognitive services to the community’ [26].

Errors were categorised as per Table 1 below. The severity of medication error referred to the potential harm or actual harm associated with medication errors [30].

**Table 1:** Error type and categorisation.

Error Type	Error Category	Definition	Examples
Prescribing error[27]	Commission error	It involves inaccurately written information on the prescription	• Errors include: wrong strength, wrong drug name not spelling, drug dosage form and drug-drug interactions
	Omission error	It refers to the absence of essential information in a prescription	• Errors related to prescriber (including patient name, age, prescriber name, prescriber signature, patient visited department and diagnosis) • Errors related to drugs (including route, dose, frequency, dosage form and quantity to supply)
Dispensing error [28]	Labelling error	It is considered when the printed information on the dispensed medication label contains incorrect information	• Errors include: Incorrect - Patient name - Drug name - Drug strength - Drug quantity - Dosage form - Date - Instructions - Pharmacy address
	Content error	It pertains to situations where an incorrect medication is dispensed to the patient	• Errors include: - Incorrect drug - Incorrect strength - Incorrect dosage form - Omission of item - Expired medication - Dose added - Missed doses
Administration error [29]	Categorised according to the Institute for Healthcare Improvement (IHI) ‘The Five Rights of Medication Administration’[29]	Right patient	Errors include administration of the medication to the wrong patient
		Right drug	Errors include administration of the wrong medication to the patient
		Right time	Errors include administration of the medication to the patient at the wrong time
		Right dose	Errors include administration of the wrong medication dose to the patient
		Right route	Errors include administering the medication *via* the wrong route to the patient

### Data sources and search strategy

A systematic search was conducted through the following databases and search engines from 1 January 1995 until 31 December 2023. The following databases were searched: MEDLINE (Ovid), Embase (Ovid), Cochrane Central Register of Controlled Trials, ISI Web of Science, Scopus, Database of Abstracts of Reviews of Effects (DARE), Health System Evidence, Global Health Database, Joanna Briggs Institute Evidence-Based Practice Database, Academic Search Complete, ProQuest Dissertations, PROSPERO, Cumulative Index to Nursing and Allied Health Literature (CINAHL) (EBSCO), ScienceDirect (Elsevier), Health Management Information Consortium (HMIC), Eastern Mediterranean Regional Office of the World Health Organization (WHO) (EMRO), and Google Scholar. Manual searching of the bibliographies of key articles and other review articles was also undertaken. Unpublished studies (*i.e.,* grey literature) were identified through abstracts of conference proceedings and dissertation abstracts. The search also included theses.com and ProQuest as data sources as they provide access to doctoral dissertations, master’s theses, and other research works that may not be published in peer-reviewed journals.

Filters and advanced search strategies were applied according to specific databases. There were no restrictions imposed on the age or specific groups of patient populations that were included.

To locate relevant studies, search terms were chosen from different categories related to the systematic review PECOS. (Supplementary file 1 outlines the search strategy for each database).

### Study selection

Endnote^©^ was used to remove duplicate titles from the search. Titles and abstracts were then screened for eligibility by a single researcher (MH: Maguy El Hajj) using the inclusion criteria. Eligible studies were exported to Rayyan^©^ software where full-text screening was undertaken by a single researcher (MH). Rayyan^©^ is a free, user-friendly, web-based software designed to facilitate the screening and selection of studies. It enables comparison of inclusion and exclusion decisions, making the review process more efficient [31]. Studies were included if they satisfied the pre-specified inclusion criteria. Authors were contacted by email to request missing information. If no response was received after one week, the study was excluded from inclusion.

In case of doubts regarding the inclusion of articles, discussions were undertaken with the senior authors (AH: Andy Husband and AT: Adam Todd) who had consensus. The abstracts of articles published in languages other than English were translated into English using Google Translate® and screened for eligibility. Native speakers were consulted to translate studies in German, Dutch and Portuguese if considered relevant for a full-text review.

### Data extraction

A structured tool for extracting data was developed, incorporating the following areas: bibliographic details (author, year of publication, DOI), study design, study setting, ‘type’ of error in the medication use process (prescribing, transcribing and documenting, dispensing, administering, monitoring), year and duration of data collection, approach used for identifying error, main findings, limitations, and conclusions. The tool was piloted tested and refined on five randomly chosen included studies. The data were extracted by one researcher (MH) and checked in full by a second researcher (RA: Rayah Assiri) between March and May 2024. Any disagreements were discussed with the senior authors (AH and AT) who had consensus. Missing data was addressed by contacting the corresponding author of the study. If no response was received, the information was recorded as unavailable.

### Quality assessment

The quality of each included study was assessed by a single researcher (MH) using a risk of bias tool for medication errors previously designed and used by Campbell *et al.,* in a systematic review on medication errors in community pharmacies in the United States [22]. This tool assesses four sources of bias: 1) selection bias 2) identification bias 3) error categorisation bias and 4) conflict of interest bias [22].

### Data synthesis and analysis

Textual summaries and tables were created to align with the primary studies’ outcomes and characteristics, utilising the extracted data including country of the study, study design, number of included pharmacies, year of data collection, error detection method and responsible person, safety hazard, population studied, inclusion and exclusion criteria, rate of errors, type, nature and seriousness of errors, implicated medication(s) and limitations. The study results were systematically organised by geographical region in accordance with the 2023 World Bank classification system [32]. Narrative and descriptive synthesis of study results was used. The strategy for narrative synthesis was conducted focusing on four elements: ‘theory development, developing a preliminary synthesis, exploring relationships within and between studies, and assessing the robustness of the synthesis’ [33].

## Results

**Fig 1:**
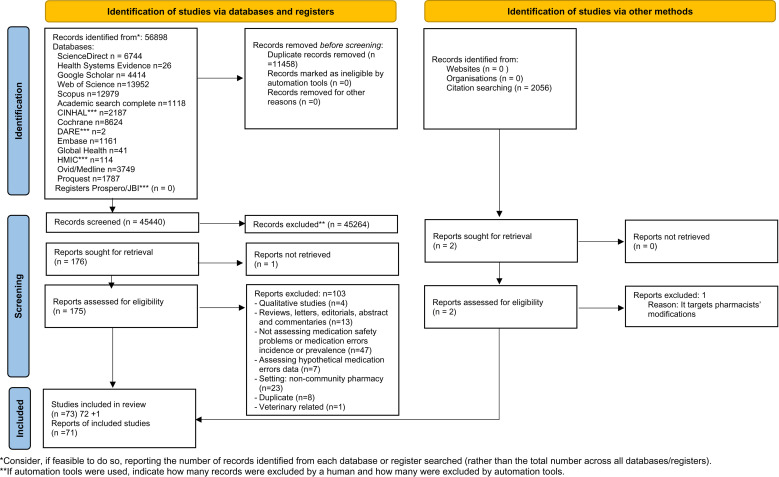
PRISMA 2020 flow diagram for new systematic reviews which included searches of databases, registers and other sources.

A total of 56898 potentially eligible studies were identified. After removal of duplicates, 45440 studies were screened of which 71 studies met the inclusion criteria. Two additional studies were identified through citation searching. Therefore, a total of individual 73 studies were included in the systematic review (Fig 1) (Supplementary file 2) [25].

### Studies characteristics

The characteristics of included studies are presented in Table 2. These studies were systematically organised by geographical region in accordance with the World Bank Classification System. [32].The predominant location of studies was in Europe and Central Asia (n = 27 studies) [24, 28,38–62], followed by North America (n = 20) [76–95]. Ten studies were conducted in the Middle East and North Africa (MENA) [66–75], while six studies were from South Asia [96–101]. Four studies were conducted in East Asia and Pacific [34–37]. Three studies were from Latin America and the Caribbean regions [63–65] and from the Sub-Saharan Africa region [102–104].

**Table 2:** Characteristics of Included Studies*.

Area[32]	Study Reference/ Year published	Country	Number of pharmacies	Year of data collection	Duration of data collection	Study Design	Approach Used for identifying error	Safety Hazard	Medication Type	Prescription Type	Population studied
**East** **Asia** **and Pacific**	Adie *et al.,*/2021[34]	Australia	30	2010-2011	30 months	Prospective	Self-report of errors	Near-miss + error	NM	NM	All
	Uzunbay *et al.,*/2023 [35]	Australia	100 patients who were using a community pharmacy-prepared DAA prior to hospital admission	2018/2019/2020	3 months in 2018 4 months in 2019 5 months in 2020	Prospective observational	Review of patient best possible medication history (BPMH) versus DAA	Near-miss	RX	NM	Age range = 73–89 years
	Han *et al.,*/2023 [36]	Republic of Korea	NM	2013- 2021 and 2018–2021	8 years and 3 years	Cross-sectional	Self-report of errors	Near-miss + error	RX	NM	NM
	Ho *et al.,*/2012 [37]	Taiwan	NM	2006	NM	Retrospective	Review of prescriptions	NM	RX	NM	All ages and genders as per study
**Europe and Central Asia**	Knudsen *et al.,*/2007 [38]	Denmark	40	2004	3-14 weeks	Retrospective + Prospective	Self-report of errors	Near-miss+ error	RX	Electronic Fax Telephone Typed Handwritten	NM
	Volmer *et al.,*/ 2012 [39]	Estonia Sweden Norway	Estonia (n = 4) Sweden (n = 7) Norway (n = 9)	Norway: 2004 Estonia: 2006 Sweden: 2007-2008	Norway: 5 weeks Estonia: 6 weeks Sweden: over 3 weeks	Observational study	Self-report of errors + direct observation	NM	RX	Handwritten + electronic	NM
	Teinilä *et al.,*/2009 [40]	Finland	599	2005	4 weeks	Cross-sectional	Survey of pharmacy owners and managers	NM	NM	NM	NM
	Timonen *et al.,*/2018 [41]	Finland	54	2017	3 days	NM	Self-report of errors	NM	RX	Electronic	NM
	Sayers *et al.,*/2009 [42]	Ireland	12	2003	3 days	Prospective	Review of prescriptions	Near-miss+ error	NM	Handwritten + electronic	Paediatrics+ adults
	Cassidy *et al.,*/ 2011 [43]	Ireland	NA/ NPIC	2007-2009	3 years	Prospective	Reported phone inquiries	Error	RX + OTC	NM	All ages+ genders
	Cheptanari-birta *et al.,*/2022 [44]	Republic of Moldova	22	NM	NM	NM	Review of prescriptions	Near-miss + error	RX	NM	NM
	Van Leeuwen *et al.,*/2001 [45]	Netherlands	1	1998-2001	3 years	Qualitative improvement + descriptive data	Self-report of errors	NM	NM	NM	NM
	Cheung *et al.,*/2011 [46]	Netherlands	331	2010-2011	1 year	NM	Self-report of errors	Near-miss + error	NM	NM	NM
	Cheung *et al.,*/2014 [47]	Netherlands	NM	2012-2013	13 months	Retrospective	Self-report of errors	Near-miss + error	RX	Electronic	NM
	Haavik *et al.,*/2006 [48]	Norway	9	2004	4 weeks	NM	Review of prescriptions	Near-miss + error	RX	NM	NM
	de Las Mercedes Martínez Sánchez *et al.,*/2013 [49]	Spain	1	2010-2011	13 months	NM	Self-report of errors	Near-miss + error	RX	NM	NM
	Jambrina *et al.,*/2023 [50]	Spain	75	2019-2021	3 years	Observational + prospective	Self-report of errors	Near-miss + error	NM	NM	NM
	Rios *et al.,*/2015 [51]	Spain	170	2011/ 2012	11 months	Prospective	Self-report of errors	NM	NM	NM	NM
	Serrano *et al.,*/2016 [52]	Spain	6	2016	2 weeks	Cross-sectional	Direct observation of patients and caregivers	Error	NM	NM	Patients using inhalers or their caregivers
	Drankowska *et al.,*/2021 [53]	Poland	2	2016	5 months	Retrospective	Review of prescriptions	Near-miss	RX	NM	Paediatrics (up to 18 years)
	Castel-Branco *et al.,*/2017 [54]	Portugal	4	2015	5 months	NM	Direct observation of patient	Error	Prescribed Inhalers for COPD and Asthma	Not applicable	Adult patients with asthma or COPD
	Greene/1995 [55]	UK	23	1986/1987	3 months	Cross-sectional	Self-report of errors	NM	RX + OTC	Handwritten + electronic	NM
	Kayne *et al.,*/1996 [56]	UK	4	NM	7 days	NM	Self-report of errors	NM	RX	Handwritten	NM
	Chen *et al.,*/2005 [57]	UK	9	2000/ 2001	8 months	NM	Self-report of errors	Near-miss	RX	NM	NM
	Ashcroft *et al.,*/2005 [58]	UK	35	2001	4 weeks	Prospective	Self-report of errors	Near-miss + error	RX	NM	NM
	Quinlan *et al.,*/2002 [59]	UK	35	2001	4 weeks	NM	Self-reports of errors	Near-miss+ error	NM	NM	NM
	Chua *et al.,*/2003 [60]	UK	4	2002	≈2 months	NM	Review of prescriptions +dispensed medications	Near-miss + error	RX	NM	NM
	Warner and Gerrett/2005 [61]	UK	107	2002-2003	1 year	NM	Self-report of errors	Near-miss + error	RX	NM	NM
	Lynskey et al.,/2007 [62]	UK	15	2004	3 months	NM	Self-report of errors	Near-miss + error	RX	NM	NM
	Franklin and O’Grady/2007 [28]	UK	11	2005/ 2006	7 months	NM	Review of prescriptions+ dispensed meds	Near-miss	RX	NM	NM
	Phipps *et al.,*/2017 [24]	UK	NM	2005 -2010	3 months	Retrospective	Self-report of errors	Error	NM	NM	NM
**Latin America and Caribbean**	de Souza *et al.,*/2006 [63]	Brazil	1	2004	3 months	Cross-sectional	Review of prescriptions	Near-miss	RX	NM	All patients regardless of age
	Diniz *et al.,*/2011 [64]	Brazil	30	2009	1 month	Retrospective	Review of prescriptions	Near-miss	RX (clonazepam)	Handwritten	NM
	da Silva *et al.,*/2012 [65]	Brazil	1	2009	2 months	Cross-sectional	Review of prescriptions	Near-miss	RX	Handwritten	NM
**Middle East and North Africa**	Abuelsoud *et al.,*/ 2018 [66]	Egypt	NM	2017	3 months	Retrospective+ Cross-sectional	Review of prescriptions	Near-miss +error	Rx	Handwritten	Adults and Elderly
	Kassem *et al.,*/2021 [67]	Egypt	10/region	2020	1.5 months	Cross-sectional	Review of prescriptions	Near-miss	RX	Handwritten/ computerized	Pediatrics
	Sarhangi *et al.,*/2021 [68]	Iran	10	2016	NM	Cross-sectional	Direct observation	NM	RX + OTC	NM	Patients <> 40 years
	Abdel-Qader *et al.,*/2021 [69]	Jordan	350	2020	5 months	Prospective	Direct observation	Near-miss	RX + OTC	Handwritten	NM
	Kamel *et al.,*/2018 [70]	KSA	8	2016	2 months	Cross-sectional	Review of prescriptions	NM	RX	NM	NM
	Soubra and Karout/2021 [71]	Lebanon	286	2017	2 months	Prospective+ Cross-sectional	Direct observation	Near-miss + error	RX	Handwritten	NM
	Mohamed Ibrahim *et al.,*/2020 [72]	UAE	350	2019/ 2020	5 months	Prospective	Direct observation+ interviews	Near-miss	RX	Electronic	NM
	Al-Worafi *et al.,*/2018 [73]	Yemen	23	2015/ 2016	8 months	Cross-sectional	Review of prescriptions	Near miss	RX	Handwritten	NM
	Al-Worafi *et al.,*/2018 [74]	Yemen	7	2016	4 months	Prospective	Self-report of errors	Near-miss	RX	Handwritten	NM
	Al-Worafi *et al.,*/2018 [75]	Yemen	5	2017	3 months	Prospective	Self-report of errors	Near-miss	RX	Handwritten	NM
**North America**	Makhinova *et al.,*/2020[76]	Canada	97	2016/ 2017	8 months	Cross-sectional	Direct observation	Error	NM	NM	Patients with asthma, COPD and respiratory conditions
	Ledlie *et al.,*/2023 [77]	Canada	2856	2018-2021	3 years 2 months	Retrospective	Self-report of errors	Near-miss+ error	NM	NM	Pediatrics, Adults + Geriatrics
	Sears *et al.,*/2016 [78]	Canada	1	NM	Over 1 month	Descriptive Quantitative	Self-report of errors	Error	NM	NM	NM
	Aubert *et al.,*/2023 [79]	Canada	NM	2019-2022	3 years	NM	Self-report of errors	Near-miss+ error	NM	NM	From ≤5 years to ≥ 80 years
	Lee *et al.,*/2023 [80]	Canada	NM	2009-2019	10 years	NM	Analysis of safety bulletin newsletters that highlighted QREs	Near-miss+ error	NM	NM	NM
	Boucher *et al.,*/2018 [81]	Canada	301	2010-2017	6 years 8 months	Retrospective	Self-report of errors	NM	NM	NM	NM
	Allan *et al.,*/1995 [82]	USA	100	1994	8 weeks	Cross-sectional	Direct observation	Near-miss	RX	Handwritten	NM
	Flynn *et al.,*/ 2003 [83]	USA	50	2000-2001	10 months	Cross-sectional	Direct observation	Near-miss+ error	RX	NM	NM
	Teagarden *et al.,*/2005 [84]	USA	3	2003	2 months	Descriptive	Review of prescriptions + dispensed medications	NM	RX	NM	NM
	Witte and Dundes/2007 [85]	USA	1	2003/ 2004	8 weeks	Prospective	Review of prescriptions	Near-miss+ error	RX	Handwritten	NM
	Hoxsie *et al.,*/ 2006 [86]	USA	18	2004/ 2005	5 months	Prospective	Direct observation	Near-miss+ error	RX	NM	NM
	Flynn *et al.,*/2009 [87]	USA	100	2007	8 weeks	Cross-sectional	Direct observation	Near-miss	RX	Handwritten	NM
	Khadem *et al.,*/2010 [88]	USA	8	2008	1 year	Prospective	Review of prescriptions +dispensed medications	Near-miss+ error	RX	Electronic+ Inter-pharmacy transfers+ telephone refill	Parkinson disease patients
	Nanji et al.,/ 2011 [89]	USA	NM	2008	4 weeks	Retrospective	Review of prescriptions	Near-miss+ error	RX	Electronic	NM
	Pervanas *et al.,*/2016 [90]	USA	NM	2007-2012	5 years 5 months	Retrospective	Self-report of errors	Near-miss	RX	NM	NM
	Hincapie *et al.,*/2019 [91]	USA	NM	2010-2015	5 years	Retrospective	Self-report of errors	Near-miss+ error	RX	Electronic	NM
	Lester *et al.,*/2017 [92]	USA	1660	2011-2014	4 years	Retrospective	Self-report of errors	Near-miss+ error	RX	Walk-in/ e-Rx/Fax/ Phone	NM
	Reed-Kane *et al.,*/2014 [93]	USA	1	2012	4 weeks	Quality improvement project with descriptive analysis	Review of prescriptions	NM	RX	Electronic	NM
	Odukoya *et al.,*/2014 [94]	USA	5	NM	45 hours	Retrospective +real time	Direct observation	Near-miss	RX	Electronic	NM
	Vo and Molitor/2022 [95]	USA	NM	2021/ 2022	13 days-6 months	NM	Review of prescriptions	NM	RX Schedule III to V	Electronic	NM
**South Asia**	Patel *et al.,*/2005 [96]	India	1	2003	7 days	Cross-sectional	Review of prescriptions	Near-miss	RX	NM	NM
	Joshi *et al.,*/2016 [97]	India	3	2008-2010	19 months	Cross-sectional	Review of prescriptions	NM	RX	Handwritten+ electronic	NM
	Marwaha *et al.,*/2010 [98]	India	2	NM	2 months	Retrospective	Review of prescriptions	Near-miss + error	RX	Handwritten	NM
	Rathi *et al.,*/2022 [99]	India	NM	NM	NM	Retrospective Cross-sectional	Review of prescriptions	Near-miss	RX + OTC	Handwritten+ electronic	NM
	Atif *et al.,*/2018 [100]	Pakistan	5	2015	4 weeks	Cross-sectional	Review of prescriptions	Near-miss	RX	NM	All ages and genders
	De Silva *et al.,*/2015 [101]	Sri Lanka	2	2013	4 months	NM	Review of prescriptions	Near-miss	RX	NM	NM
**Sub-Saharan Africa**	Anagaw *et al.,*/2023[102]	Ethiopia	10	2021	2 months	Retrospective+ Cross-sectional	Review of prescriptions	NM	RX	NM	Prescriptions by private sector except pregnant and psychiatry patients
	Simegn *et al.,*/2022 [103]	Ethiopia	19 pharmacies + 3 drug stores	2020	5 months	Cross-sectional	Self-administered cross-sectional survey of pharmacists	NM	RX	NM	Adults Children Elderly Teenagers
	Malangu *et al.,*/2012 [104]	South Africa	1	2006-2007	11 months	Cross-sectional	Review of prescriptions	Near-miss +error	RX	NM	Pediatric + adult patients on anti-retrovirals

*COPD: Chronic Obstructive Pulmonary Disease DAA: dose administration aids KSA: Kingdom of Saudi Arabia NB: Number NPIC : National Poison Information Center NM: Not Mentioned OTC: Over the counter RX: Prescription UAE: United Arab Emirates UK: United Kingdom USA: United States of America

The majority of the included studies (n = 67) were published in English [24,28,34–44,46,47,49,50,53–62,64,66–104]. Articles published in other languages included two studies published in Spanish [51,52], two in Portuguese [63,65], one in Dutch [45] and another in Danish [48]. These publications underwent translation into the English language.

The number of included community pharmacies varied across the studies and countries, ranging from one pharmacy in nine studies [45,49,78,85,93,96,63,65,104] to 2,856 pharmacies in a single study [77].

The studies varied in the duration of data collection, ranging from three days [41,42], to 10 years [80].

The predominant study design was cross-sectional (n = 20) [36,40,52,55,63,65,67,68,70,73,76,82,83,87,96,97,100,102–104]. Thirteen studies employed a retrospective design [24,47,53,77,81,89–92,94,98,37,64], while another thirteen studies were prospective [42,43,51,58,85,86,88,69,72,74,75,34,35]. Conversely, information on the study design was not provided in seventeen studies [41,44,46,48,49,54,56,57,28,59–62,79,80,95,101].

Various approaches were used to identify medication errors. Twenty-eight studies opted for a review of prescriptions [28,37,42,44,48,53,60,63–67,70,73,84,85,88,89,93,95–102,104]. Twenty-eight studies relied on self-reported errors or incidents [24,34,36,38,39,41,45–47,49–51,55–59,61,62,74,75,77–79,81,90–92]. Additionally,thirteen studies utilised direct observation techniques to detect medication errors [39,52,54,68,69,71,72,76,82,83,86,87,94]. In one study, patients’ best possible medication history (BPMH) were reviewed versus their community pharmacy-prepared dose administration aids [35]. While another study compiled Quality related events (QREs) occurring in community pharmacies and published in the Institute for Safe Medication Practices (ISMP) Canada safety bulletin newsletters [80].

Fifty-three studies did not specify the characteristics of the enrolled patients [24,38–41,44–51,28,55–62,78,81–87,89–95,80,69–75,96–99,101,36,64,65]. Six studies included patients without imposing any restrictions on age or sex [34,37,43,63,77,100]. Two studies exclusively focused on paediatric patients [53,67]. Additionally, three studies enrolled patients with respiratory diseases or those using inhalers[52,54,76]. Furthermore, one study specifically targeted patients with Parkinson’s disease who were treated with levodopa/carbidopa [88], while another study focused on patients receiving anti-retroviral agents [104]. One study only included geriatric patients using community pharmacy-prepared dose administration aids [35]. And another study targeted patients from the private health sector except pregnant and psychiatry patients [102].

Fifty- three studies focused exclusively on prescribed medications [28,35–39,41,44,47–49,53,54,56–58,60–67,70–76,82–98,100–104] while 16 studies did not specify whether they targeted over-the-counter or prescribed medications [24,34,40,42,45,46,50–52,59,76–81]. Additionally, five studies addressed both prescription and over-the-counter medications [43,55,68,69,99].

### Overall medication errors

This review categorises medication errors results based on the specific stage in the medication use process [18] (Table 3) and according to the geographical location of the included studies [32].

**Table 3:** Stages in the Medication Use Process targeted by Different Studies*.

			Medication Use Process Stages [18]								
**Area [32]**	**Study Reference/** **Year published**	**Country**	**Prescribing**	**Transcribing/** **Documenting**	**Dispensing**	**Administering**	**Monitoring**	**Supply/** **Ordering**	**Storage/** **Wastage**	**Compounding**	**Counseling**
**East** **Asia** **and Pacific**	Adie *et al.,*/2021[34]	Australia	X (most common)		X	X		X	X		
	Uzunbay *et al.,*/2023 [35]	Australia			X						
	Han *et al.,*/2023 [36]	Republic of Korea	X (most common)		X	X					
	Ho et al.,/2012 [37]	Taiwan	X								
**Europe and Central Asia**	Knudsen *et al.,*/2007 [38]	Denmark	X (most common)	X	X	X					X
	Volmer *et al.,*/ 2012[39]	Estonia Sweden Norway	X								
	Teinilä *et al.,*/2009 [40]	Finland			X						
	Timonen *et al.,*,/2018 [41]	Finland	X								
	Sayers *et al.,*/2009 [42]	Ireland	X								
	Cassidy *et al.,*/ 2011 [43]	Ireland	X		X	X (most common)					
	Cheptanari-birta *et al.,*/2022 [44]	Republic of Moldova	X								
	Van Leeuwen *et al.,*/2001 [45]	Netherlands			X (most common)					X	X
	Cheung *et al.,*/2011 [46]	Netherlands	X	X (most common-order entry)	X	X				X	
	Cheung *et al.,*/2014 [47]	Netherlands	X	X	X (most common)	X	X				
	Haavik *et al.,*/2006[48]	Norway	X								
	de Las Mercedes Martínez Sánchez *et al.,*/2013 [49]	Spain	X (most common)	X	X						
	Jambrina *et al.,*/2023 [50]	Spain	X (most common)		X	X					
	Rios *et al.,*/2015 [51]	Spain			X						
	Serrano *et al.,*/2016 [52]	Spain				X					
	Drankowska *et al.,*/2021[53]	Poland	X								
	Castel-Branco *et al.,*/2017 [54]	Portugal				X					
	Greene/1995 [55]	UK	X								
	Kayne *et al.,*/1996 [56]	UK	X (most common)		X						
	Chen *et al.,*/2005 [57]	UK	X								
	Ashcroft *et al.,*/2005 [58]	UK			X						
	Quinlan *et al.,*/2002 [59]	UK			X						
	Chua *et al.,*/2003 [60]	UK			X						
	Warner and Gerrett/2005 [61]	UK	X		X (most common)						
	Lynskey *et al.,*/2007 [62]	UK	X		X (most common)	X					
	Franklin and O’Grady/2007 [28]	UK			X						
	Phipps *et al.,*/2017 [24]	UK			X						
**Latin America and Caribbean**	de Souza *et al.,*/2006 [63]	Brazil	X								
	Diniz *et al.,*/2011 [64]	Brazil	X								
	da Silva *et al.,*/2012 [65]	Brazil	X								
**Middle East and North Africa**	Abuelsoud *et al.,*/ 2018[66]	Egypt	X								
	Kassem *et al.,*/2021 [67]	Egypt	X								
	Sarhangi *et al.,*/2021 [68]	Iran			X						
	Abdel-Qader *et al.,*/2021 [69]	Jordan			X						
	Kamel *et al.,*/2018[70]	KSA	X								
	Soubra and Karout/2021 [71]	Lebanon			X						
	Mohamed Ibrahim *et al.,*/2020 [72]	UAE			X						
	Al-Worafi *et al.,*/2018 [73]	Yemen	X								
	Al-Worafi *et al.,*/2018 [74]	Yemen			X						
	Al-Worafi *et al.,*/2018 [75]	Yemen			X						
**North America**	Makhinova *et al.,*/2020 [76]	Canada				X					
	Ledlie *et al.,*/2023 [77]	Canada	X	X (Most common-order entry)	X						
	Sears *et al.,*/2016 [78]	Canada	NM	NM	NM	NM	NM	NM	NM	NM	NM
	Aubert *et al.,*/2023 [79]	Canada	NM	NM	NM	NM	NM	NM	NM	NM	NM
	Lee *et al.,*/2023 [80]	Canada			X						
	Boucher *et al.,*/2018 [81]	Canada	X	X (Most common-order entry)	X	X	X				
	Allan *et al.,*/1995 [82]	USA			X						
	Flynn *et al.,*/2003 [83]	USA			X						
	Teagarden *et al.*/2005 [84]	USA			X						
	Witte and Dundes/ 2007 [85]	USA		X							
	Hoxsie *et al.,*/2006 [86]	USA			X						
	Flynn *et al.,*/2009 [87]	USA			X						
	Khadem *et al.,*/2010 [88]	USA			X (most common)						X
	Nanji *et al.,/* 2011 [89]	USA	X								
	Pervanas *et al.,*/2016 [90]	USA			X						
	Hincapie *et al.*,/2019 [91]	USA	X								
	Lester *et al.,*/2017 [92]	USA			X						
	Reed-Kane *et al.,*/2014 [93]	USA	X								
	Odukoya *et al.,*/2014 [94]	USA	X								
	Vo and Molitor/2022 [95]	USA	X								
**South Asia**	Patel *et al.,*/2005 [96]	India	X								
	Joshi *et al.,*/2016 [97]	India	X								
	Marwaha *et al.,*/2010 [98]	India	X								
	Rathi *et al.,*/2022 [99]	India	X								
	Atif *et al.,*/2018 [100]	Pakistan	X								
	De Silva *et al.,*/2015 [101]	Sri Lanka	X								
**Sub-Saharan Africa**	Anagaw *et al.,*/2023 [102]	Ethiopia	X								
	Simegn *et al.,*/2022 [103]	Ethiopia	X								
	Malangu *et al.,*/2012 [104]	South Africa	X (most common)		X						

* KSA: Kingdom of Saudi Arabia NM: Not Mentioned UAE: United Arab Emirates UK: United Kingdom USA: United States of America

In the Europe and Central Asia region, ten studies examined medication errors across multiple stages in the medication use process [38,43,45–47,49,50,56,61,62]. Prescribing errors were the exclusive focus of eight studies [39,41,42,44,48,53,55,57] while seven studies specifically examined dispensing errors [24,40,51,58–60,28]. Additionally, two studies addressed administration errors [52,54].

In North America, three studies assessed medication errors in several stages of the medication use process [77,81,88] while five studies mainly targeted prescribing errors [89,91,93–95], with eight studies examining specifically dispensing errors [80,82–84,86,87,90,92]. Only one study conducted in the US exclusively targeted transcribing errors [85]. In two studies, the specific stage at which the medication error occurs is not mentioned [78,79].

In the Middle East and North Africa (MENA) region, prescribing errors were the exclusive focus of four studies [66,67,70,73] while six studies concentrated solely on dispensing errors [68,69,71,72,74,75].

The included studies from South Asia, Latin America (n = 6) [96–101] and the Caribbean regions (n = 3) [63–65] assessed prescribing errors.

In the East Asia and Pacific region, two studies focused on multiple stages within the medication use process [34,36], one study concentrated on prescribing errors [37] and one study targeted dispensing errors [35].

Only two studies in all regions assessed monitoring errors: one study conducted in the Netherlands [47] and another in Canada [81].

#### Studies focusing on errors in multiple stages of the medication use process.

Among the studies conducted in the Europe and Central region investigating multiple stages of the medication use process, the following types of errors predominated: prescription errors in four studies [38,49,50,56], dispensing errors in four studies [45,47,61,62], administration errors in one study [43]. In North America, transcribing errors were identified as the most prevalent in two Canadian studies [77,81], while dispensing errors were predominant in another American study [88]. In the East Asia and Pacific region, prescribing errors were identified as the most prevalent in two studies [34,36], while in the Sub-Saharan region, prescribing errors were also found to be the most common [104].

### Prescribing errors

This section synthesises data on prescribing errors from studies that focused exclusively on prescribed errors, as well as studies that examined prescribing errors alongside other types of errors. Variations in the rate of prescribing errors were observed across different studies and geographic regions (Table 4). These variations stem from discrepancies in defining error rates and utilising different denominators. Denominators encompassed various metrics including the number of patient visits [37], total number of prescriptions [38,41,44,48,49,53,56,63–66,70,73,89,93,95–97,99–102,104] and total number of items [42,55,57,98]. Within the Europe and Central Asia region, reported error rates ranged from 0.062% in a UK study [55] to 32.84% in a Polish study [53]. The majority of studies (n = 12) addressed both commission and omission errors [38,39,41,42,44,48–50,53,55–57], with commission errors being the most frequently reported (n = 6) [38,39,44,55–57]. The most common commission error was incorrect medication (n = 6) [38,39,44,56,57,62]. Only four studies provided insights into the severity of errors [42,44,55,56], with three studies [42,44,56] reporting errors as ‘non-serious’, while one study highlighted ‘serious’ errors [55].

**Table 4:** Prescribing Errors*.

Area [32]	Study Reference	Country	Denominator for prescribing error	Numerator for prescribing error	Incidence (I)/Rate(R)/ Prevalence (P) of prescribing errors	Study targeted commission error	Study targeted omission error	Most common prescription error	Most common commission error	Most common omission error	Severity of errors	Top 2 implicated medication classes in prescribing errors
**East** **Asia** **and Pacific**	Adie *et al.,*/2021 [34]	Australia	NM	Prescribing errors: 619	NM	X	X	Commission	Incorrect/ wrong medication	Unclear/ incomplete prescription	NM	NM
	Han *et al.,*/2023 [36]	Republic of Korea	NM	Prescribing errors: 8098	NM	X	X	Commission	Incorrect/ wrong medication	NM	Near-miss: 86.4% No harm: 0.2% Mild harm: 0.3% Moderate harm: 0.2% Severe harm: 0%	NM
	Ho *et al.,*/2012 [37]	Taiwan	1003 patient visits 3065 prescriptions	Prescription errors:560	18.3% prescription errors identified from 350 (34.9%) ambulatory visits, ranging from 1 (59.1%) to 7 (0.3%) errors/ visit	X	X	Commission	Overdosing	Indication missing	NM	NM
**Europe and Central Asia**	Knudsen *et al.,*/2007 [38]	Denmark	Prescriptions processed during observation period: 421 809	Prescription incidents: 1015	Prescription corrections Error rate/10000 prescriptions: 23.1 (95%CI: 21.7 to 24.6)	X	X	Commission	Prescribing a medicine, strength, quantity or dosage that did not exist	No basic prescription data	NM	NM
	Volmer *et al.,*/2012 [39]	Estonia Sweden Norway	NM	RX errors Estonia (n = 222) Norway (n = 371) Sweden (n = 237)	% of errors/ prescription -Estonia: (1.1%) -Norway: (1.1%) -Sweden: (1.0%)	X	X	Estonia: Managerial error Norway: Commission error Sweden: Commission error	Estonia: Incorrect medication strength Norway and Sweden: Incorrect medication/ indication	Estonia and Norway: Prescriber information Sweden: Patient and prescriber information	NM	NM
	Timonen *et al.,*/2018 [41]	Finland	41170 e-Rx dispensed during study period	-2978 Rx contained anomalies - In total 3622 anomalies were recorded	7.2% contained anomalies	X	X	Omission	Incorrect dosage instructions	Dosage instructions written using abbreviation	NM	CNS: 22.9% CVD: 18.1%
	Sayers *et al.,*/2009 [42]	Ireland	8,686 drug items	Total number of items containing errors:546	Overall error rate: 6.2 per 100 items prescribed	X	X	Omission	Mix up in medications	No directions	Minor: 72.9% Major: 24.7% Serious: 2.4%	CVD
	Cassidy *et al.,*/2011 [43]	Ireland	NM	Prescribing errors: 9	NM	X	No	Commission	Incorrect dose	NA	NM	NM
	Cheptanari-birta *et al.*,/2022 [44]	Republic of Moldova	-754 prescriptions 1104 medicines were present in prescriptions	Prescribing errors: 1872	-Errors in medicine name (22.54% CI95:19.55–25.52) -Errors in pharmaceutical form (16.97% CI 95:14.29–19.65) - Lack of information about patient (10.74% CI95:8.53–12.95) -Lack of information about doctor (90.18% CI95: 87.87–92.48) -Failure to indicate the validity of prescription (87.93% CI95:85.60–90.25)	X	X	Commission	Error in the name of the medicine	Missing doctor phone number	4 cases reached patients and caused minor (2) and medium (2) damage	NM
	Cheung *et al.,*/2011 [46]	Netherlands	NM	Prescribing errors:698	NM	NM	NM	NM	NM	NM	NM	NM
	Haavik *et al.,*/2006 [48]	Norway	69,315 dispensed RX	1,696 errors recorded in 1,359 prescriptions	2%	X	X	Omission	Incorrect drug/ indication	Incomplete usage instructions	NM	NM
	de Las Mercedes Martínez Sánchez *et al.*,/2013 [49]	Spain	42,000 prescriptions	Prescribing errors: 1,127	NM	X	X	Omission	Missing or wrong patient ID	Prescription is illegible (illegible handwriting)	NM	NM
	Jambrina *et al.,*/2023 [50]	Spain	NM	NM	NM	X	X	NM	NM	NM	NM	NM
	Drankows-ka *et al.*,/2021 [53]	Poland	36262 prescriptions for ready-made medications analyzed	Prescribing errors -Szczecin: 111: -Lipiany: 154	Prescribing errors -Szczecin: 17.51% -Lipiany 32.84%	X	X	NM	Oral dosage forms incorrectly adapted to child age	Lack of dose specification	NM	Respiratory System -Szczecin: 34.38%% -Lipiany: 40.51%
	Greene/ 1995 [55]	UK	281,900 items monthly	340 incident report forms	0.062%	X	X self-evident	Commission	Changed dose	NM	Receptionist origin= 221 -Trivial: 10% -Not serious: 26% -Serious: 49% -Very serious: 15% Doctor originated = 96 -Trivial: 12% -Not serious: 26% -Serious: 48% -Very serious: 14%	CNS (14.4%)
	Kayne/1996 [56]	UK	5004 prescriptions dispensed	Prescriptions were queried on: 93	Prescription error: 1.86%	X	X	Commission	Wrong medicine on repeat prescription	Prescription illegible	20 serious prescription queries (0.4%)	NM
	Chen *et al.,*/2005 [57]	UK	32403 items	196 prescribing problems related to 194 prescriptions	Prescribing problems reporting rate:0.6%	X	X	Commission	Incorrect prescription, wrong information concerning medication, pack size/quantity or patient, violation of legal requirement		NM	NM
	Warner and Gerrett/ 2005 [61]	UK	NM	Prescribing errors: 233	NM	NM	NM	NM	NM	NM	NM	NM
	Lynskey *et al.,*/2007 [62]	UK	NM	Near-miss prescribing errors:23 Prescribing errors: 3	NM	X	No	Commission	Improper medicine	NA	NM	NM
**Latin America and Caribbean**	De Souza *et al.,*/2006 [63]	Brazil	200 prescriptions with 386 prescriptions	NM	NM	No	X	Omission	NA	Missing patient information prescription	NM	CNS: 22.5% Anti-infective: 17.4%
	Diniz *et al.,*/2011 [64]	Brazil	311 clonazepam prescriptions	Not fully eligible prescription:175	56.27% not fully eligible prescriptions	No	X	Omission or illegible	NA	Absent medication route on prescription	NM	NM
	da Silva *et al.,*/2012 [65]	Brazil	-98 prescriptions analysed, totalling 137 prescription drugs	NM	NM	No	X	Omission	NA	Missing route on prescription	NM	CNS: 59.2% CVD: 26.5%
**Middle East and North Africa**	Abuelsoud *et al.,*/ 2018 [66]	Egypt	810 prescriptions including 3262 medications	Prescribing errors: 19,405	NM	X	X	Omission	Drug-drug interactions	Missing generic name of medication from prescription	9% reached patients and may have caused harm or sub effect	CVD: 17.65% GIT: 13.08%
	Kassem *et al.,*/2021 [67]	Egypt	NM	NM	NM	X	X	Omission	Handwritten -Wrong duration of therapy Compute -contra-indication	Absence of storage information	NM	Abx
	Kamel *et al.*,/2018 [70]	KSA	117 prescriptions	NM	NM	X	X	Commission	Drug unrelated to diagnosis	Patient chronic condition not written on prescription	NM	NM
	Al-Worafi *et al.,*/2018 [73]	Yemen	2,178 prescriptions	2159 prescriptions of very poor quality (99.12%)	NM	No	X	Omission	NA	Patient weight is missing	NM	NM
**North America**	Ledlie *et al.,*/2023 [77]	Canada	NM	Prescribing errors: 943	NM	NM	NM	NM	NM	NM	NM	NM
	Boucher *et al.,*/2018 [81]	Canada	NM	Prescribing QRE: 10 658	NM	NM	NM	NM	NM	NM	Harmful QRE: 10.6%	NM
	Khadem *et al.,*/2010 [88]	USA	NM	NM	NM	NM	NM	NM	NM	NM	NM	Carbidopa Levodopa
	Nanji *et al.,*/2011 [89]	USA	3850 prescriptions	452 prescriptions (11.7%) contained 466 total errors, of which 163 (35.0%) were potential ADEs	Error rates varied by computerized prescribing system, from 5.1% to 37.5%	X	X	Omission	Improper abbreviation	Duration, omitted	Of potential ADEs: -58.3% significant - 41.7% serious -None life-threat-ening	-Anti-infect for systemic use: 40.3% -Nervous system: 13.9%)
	Hincapie *et al.,*/2019 [91]	USA		PEER report: 956 incidents PQC report: 550 incidents		X	X	Commission	Problem with patient directions	Prescription missing essential information	PEER report: 956 incidents -Reached patient: 7.9% -Near miss: 45.9% -Unsafe condition: 46.1%	NM
	Reed-Kane *et al.,*/2014 [93]	USA	111 electronic prescriptions	70 had errors	Electronic prescription rate: 63%	X	No	Commission	Drug name in wrong field	NA	NM	NM
	Odukoya *et al.,*/ 2014 [94]	USA	NM	75 e-prescription errors	NM	X	No	Commission	Wrong quantity	NA	NM	Anti-infect Hormones Hormone modifiers
	Vo and Molitor/ 2022 [95]	USA	Number of e-Scripts reviewed -1000 legend -500 CII 500 CIII-V	Number of e-Scripts errors -Legend: 40 -CII: 26 -CIII-V e-scripts:37	% of e-Scripts errors -Legend: 4% -CII: 5.2% -CIII-V: 7.4%	X	X	Omission	Competing instructions	Missing signature components	NM	-Legend -CII -CIII-V
**South Asia**	Patel *et al.*,/2005 [96]	India	990 prescriptions	–	–	No	X	Omission	NA	Missing doctor signature	NM	NM
	Joshi *et al.,*/2016 [97]	India	Total number of prescriptions: 749	Prescribing errors: 13334	–	No	X	Omission	NA	Drug item details	NM	NM
	Marwaha *et al.,*/2010 [98]	India	3,151 prescribed items	196 errors	Error rate: 6.09% (95% CI: 5.78‐6.41)	X	X	Omission	Prescribing two drugs of the same type	Directions not mentioned at all	NM	NM
	Rathi, K. M *et al.,*/2022 [99]	India	1500 prescriptions with 2750 drugs prescribed	–	–	No	X	Omission	NA	Absence of weight information	NM	NM
	Atif *et al.,*/2018 [100]	Pakistan	300 prescriptions	Total omission errors: 1218 Total commission errors: 510	–	–	X	Omission	NA	NM	NM	NM
	De Silva *et al.,*/2015 [101]	Sri Lanka	200 prescriptions	–	–	No	X	Omission	NA	Registration number of prescriber	NM	NM
**Sub-Saharan Africa**	Anagaw *et al.,*/2023 [102]	Ethiopia	1000 prescriptions containing 1770 medications			No	X	Omission	NA	Missing of card number Dosage form Diagnosis Prescriber full name and qualification	NM	NM
	Simegn *et al.,*/2022 [103]	Ethiopia	–	–	Prevalence of prescribing errors: 75.1% (95% CI 71.08–78.70)	X	X	Commission	Drug selection	Incomplete or unavailable form/ strength	NM	Abx: 63% Analgesics 59.5%
	Malanguet *et al.*,/2012 [104]	South Africa	713 prescriptions analyzed	181 had prescriptions errors	–	X	X	Commission	Incorrect regimen	NM	NM	Anti- retrovirals

*Abx: Antibiotics Anti-infect” Anti-infectives CNS: Central Nervous System, CVD: Cardiovascular disease GIT: Gastrointestinal Tract ID: Identification KSA: Kingdom of Saudi Arabia NM: Not Mentioned PEER (Pharmacy and Provider e-prescribing Experience Reporting) PRE: Prescription-related error PQC: Pharmacy Quality Commitment QRE: Quality Related Event Rx: Prescription UAE: United Arab Emirates UK: United Kingdom USA: United States of America

In North America, prescribing error rates were reported in three studies [89,93,95] ranging from 4% [95] to 63% [93]. Three studies in this region addressed both commission and omission errors [89,91,95], with omission errors prevailing in two studies [89,95]. Additionally, three studies reported the severity of medication errors [81,89,91], with 50% or more of errors causing harm.

In the MENA region, none of the studies provided data on prescribing error rates. Most studies in this region (n = 3) addressed both commission and omission errors [66,67,70], with omission errors being predominant in two studies [66,67]. Only one study assessed the severity of errors, with 9% of errors reaching patients judged to be likely to have caused harm or resulted in a subtherapeutic effect [66].

Within South Asia, a single study, conducted in India, reported a prescribing error rate of 6.09% (95% CI: 5.78‐6.41) [98]. Most studies in this region (n = 5) solely addressed omission errors [96,97,99–101]; the types of omission errors were variable between the studies. None of these studies documented the severity of errors.

In the East Asia and Pacific region, one study, conducted in Taiwan, reported a prescribing error rate of 18.3% [37]. All studies in this region addressed both commission and omission errors [34,36,37] with commission errors being the most common. Additionally, one study in the Republic of Korea assessed error severity, reporting that the majority of errors (86.4%) were near-miss errors [36].

In Latin America and the Caribbean, all studies focused on omission errors [63–65] with missing mode of administration being the most common error. None of the studies in this region evaluated error severity.

### Dispensing errors

This section examines the data on dispensing errors from studies that focused mainly on dispensing errors and studies that assessed both dispensing errors and other types of medication errors. Dispensing errors exhibited considerable variation across studies conducted within or outside the same geographical regions (Table 5). This variation stems from the use of different definitions to categorise a dispensing error and the adoption of different denominators. Denominators employed in the studies included the total number of prescriptions [38,49,56,74,75,82–84,87,92,104], the number of prescribed items [28,58–60], total number of dispensed medications [69,71,72], transactions [86] and total number of patients [35,88]. In 18 studies, the denominator was unspecified [24,40,43,45–47,50,51,61,62,77,81,85,90,80,68,34,36].

**Table 5:** Dispensing Errors*.

Area[32]	Study Reference	Country	Denominator for dispensing error	Numerator for dispensing error	Incidence (I)/Rate(R)/ Prevalence (P) of dispensing errors	Study targeted content error	Study targeted labelling error	Most common dispensing error	Most common content error	Most common labelling error	Severity of dispensing errors	Top two implicated medication classes in dispensing errors
**East** **Asia** **and Pacific**	Adie *et al.,*/2021 [34]	Australia	–	Dispensing errors:260	–	X	X	Content	Incorrect concentration/strength	NM Incorrect Label	NM	NM
	Uzunbay *et al.,*/2023 [35]	Australia	110 DAAs reviewed for 100 patients (6 patients had > 1 DAA); a total of 822 medications packed.	-4 patients had DAAs with no medication -\82 patients had ≥ 1 DAA label incidents.	Error rate: 85.4%	No	X	Label	NA	Illegible, ambiguous or missing medication details	NM	NM
	Han *et al.,*/2023 [36]	Republic of Korea	–	Dispensing errors:605	–	X	X	Content	Dosing error	NM	Near-miss: 1.2% No harm: 0.5% Mild harm: 2.1% Moderate harm: 1.1% Severe harm:0.1%	NM
**Europe and Central Asia**	Knudsen *et al.,*/2007 [38]	Denmark	1466043 RX during observation period	Dispensing near-miss:234 Dispensing errors: 209	Near-miss Error rate/10000 prescriptions 2.4 (95%CI: 2.1 to 2.7) Error rate/10000 prescriptions 1.4 (95%CI: 1.2 to 1.6)	X	X	Content	Wrong strength	NM	Seriousness score 1: 25.2% 2: 68.4% 3:6.4%	NM
	Teinilä *et al.,/*2009 [40]	Finland	–	–	14.4 documented dispensing error per 100,000 prescriptions dispensed (95% CI 13.8–15.1) -7.1 not documented dispensing errors per 100,000 prescriptions dispensed	NM	NM	NM	NM	NM	NM	NM
	Cassidy *et al.,*/ 2011 [43]	Ireland	–	Dispensing errors:18	–	X	NM	Content	Dispensing wrong dose	NM	NM	NM
	Van Leeuwen *et al.,*/2001 [45]	Netherlands	NM	Dispensing errors:18	NM	NM	NM	NM	NM	NM	No injury:9 No lasting injury:5 Hospital admission:1 Ultimate injury unknow: 2 Death:1	NM
	Cheung *et al.,*/2011 [46]	Netherlands		Dispensing errors: 452	NM	NM	NM	NM	NM	NM	NM	NM
	Cheung *et al.,*/2014 [47]	Netherlands	–	Dispensing errors: 23	–	X	NM	Content	Forgot to take out tablet of ADD bag	NA	NM	NM
	de Las Mercedes Martínez Sánchez *et al.,*/2013 [49]	Spain	42,000 prescriptions	Dispensing errors: 216	NM	X	No	Content	Wrong drug dispensed	NA	NM	NM
	Jambrina *et al.,*/2023 [50]	Spain	NM	NM	NM	X	X	Content	Similarity of packaging	Incorrect/misleading label	NM	NM
	Rios *et al.,*/2015 [51]	Spain	–	Dispensing errors:1012	–	X	No	Content	Patient record at the pharmacy	NA	NM	NM
	Kayne/1996 [56]	UK	5004 prescriptions were dispensed	Dispensing errors:50	Dispensing error: 0.99%	X	X	Content	Wrong medicine dispensed	Wrongly labeled	NM	NM
	Ashcroft *et al./,*2005 [58]	UK	125395 prescribed items dispensed	330 incidents recorded on 310 prescriptions 280 incidents classified as near miss 50 incidents classified as errors	-Total Rate of incidents per 10 000 items dispensed: 26.32 (95%CI 23.55–29.32) -Rate of near miss incidents: 84.8% (rate per 10 000 items dispensed 22.33: 19.79–25.10) -Rate of errors: 15.2% (rate per 10 000 items dispensed 3.99: 2.96–5.26)	X	X	Content	Wrong drug/form selected	Wrong directions on label	NM	NM
	Quinlan *et al.,*/2002 [59]	UK	125,395 items were dispensed	329 errors on 310 RX - 271 as near miss errors - 58 actual errors	Error rate 0.26% -Near miss errors (0.19%) -Actual errors (0.04%) -2 errors reported per 1000 items dispensed	X	X	Content	Wrong drug/form selected	Wrong direction on label	NM	NM
	Chua *et al.,*/2003 [60]	UK	Total number of items dispensed: 51 357	In 277 items: 39 dispensing errors 247 near misses	-0.08% dispensing errors -0.48% near-misses -For every 10 000 items dispensed, 56 dispensing errors or near-miss errors are reported CI95%:49–62 -Rate: 0.56%	X	X	Content	Incorrect strength	NM	NM	NM
	Warner and Gerrett/2005 [61]	UK	–	Dispensing errors:677	–	X	X	Content	Wrong strength	Wrong direction/ quantity	NM	NM
	Lynskey *et al.,*/2007 [62]	UK	–	Dispensing near-miss errors: 90 Dispensing errors: 28	–	X	X	Content	Incorrect drug/correct strength	Wrong drug name/quantity	NM	NM
	Franklin and O’Grady/2007 [28]	UK	-Total number of prescriptions assessed: 1391-Total prescribed Items: 2859	Dispensing errors:95	Dispensing errors: 3.3%	X	X	Content	-Dose added	- Incorrect instructions	-Minor errors: 67% -Moderate errors: 32% -Severe errors: 1%	NM
	Phipps *et al.,*/2017 [24]	UK		-14,709 incidents were retrieved -14675 incidents were due to medication [99.8%]		X	No	Content	Wrong/ unclear dose or strength	NA	-No harm: 92.3% -Low to moderate harm: 7.4% -Severe harm or death: 0.2%	
**Middle East and North Africa**	Sarhangi *et al.,*/2021 [68]	Iran		3968 errors recorded	Rate of errors: 36.7% totally	NM	NM	NM	NM	NM	NM	NM
	Abdel-Qader *et al.,*/ 2021 [69]	Jordan	Total 150,442 medications	Dispensing error Total 37,009 PREs 17,352 PCEs 19,657	Total 24.6% (CI 95%: 22.9–26.1) PREs 11.5% (CI 95%: 9.2–13.7) PCEs 13.1% (CI 95%: 10.1–14.9	X	X	Content	Wrong quantity error	NM	384 incidents randomly selected - Minor: 38.8% -Moderate: 52.6% - Serious: 8.6%	Antibiotics: 22.5% Analgesics: 21.3%
	Soubra and Karout/2021 [71]	Lebanon	2860 prescribed medications dispensed	376 errors	Error rate: 2.92%	X	No	Content	Incomplete/ incorrect directions for use	NA	NM	NM
	Mohamed Ibrahim e*t al.,*/2020 [72]	UAE	464222 dispensed medications	30912	6.7%; (CI 95%: 4.3–8.6)	X	X	Content	Wrong quantity	NM	Minor:44.5% Moderate: 46.8% Serious:8.7%	Analgesic: 17.0% Antibiotic: 17.2%
	Al-Worafi *et al.,*/2018 [74]	Yemen	4325 prescriptions	Dispensing errors: 35	Dispensing errors: 0.8%	X	No	Content	Wrong dosage form	NA	NM	NM
	Al-Worafi *et al.,*/2018 [75]	Yemen	5680 prescriptions	Dispensing errors: 47	0.82%	X	No	Content	Wrong dosage form	NA	NM	NM
**North America**	Ledlie *et al.,*/2023 [77]	Canada	–	Dispensing errors: 10,669	–	X	X	Content	Incorrect drug	Incorrect label	NM	NM
	Lee *et al.,*/2023 [80]	Canada	–	Dispensing errors: 34	–	X	X	Content	Wrong drug	Wrong instructions	Death: 6 Harm: 16 No harm: 8 Near miss: 4	NM
	Boucher *et al.,*/2018 [81]	Canada		Dispensing and preparing QRE: 34 859	NM	NM	NM	NM	NM	NM	Harmful QRE: 38.1%	NM
	Allan *et al.,*/1995 [82]	USA	100 prescriptions	Dispensing errors: 24	Dispensing rate: 24%	X	X	Labelling	Wrong quantity	NM	Auxiliary label omission	Four errors could have had ADEs (4% clinical significant errors)
	Flynn *et al.,*/ 2003 [83]	USA	# of RX filled Chain: 2335 Independe-nt: 1370	# of errors Chain:37 Independent: 21	% of errors Chain 1.6% Independent 1.5%	X	X	Labelling	Wrong quantity	Wrong label instructions	NM	NM
	Teagarden *et al.,*/2005 [84]	USA	21252 prescriptions	Dispensing errors: 16	Overall dispensing rate: 0.075% (95%:0.043–0.122)	X	X	Labelling	Incorrect quantity + omission	Difference between labels directions: and RX	NM	NM
	Witte and Dundes/ 2007 [85]	USA	–	–	–	–	–	–	–	–	–	–
	Hoxsie *et al.,*/2006[86]	USA	-550 transactions completed at low-risk stores -400 transactions at high-risk stores	-5 dispensing errors (3 in low-risk stores and 2 in high-risk stores)	-0.5% at low-risk pharmacies and 0.5% at high-risk pharmacies	NM	NM	NM	NM	NM	NM	NM
	Flynn *et al.,*/2009 [87]	USA	100 prescriptions	Dispensing errors: 22	Point prevalence of errors: 22%	X	NM	Content	Wrong instructions	NM	3 errors could pose harm	Divalproaex sodium extended release: 5 errors Aspart and aspart protamine: 2 errors
	Khadem *et al.,*/2010 [88]	USA	73 patients taking levodopa carbidopa	8 patients had pharmacy errors	11% dispensing errors	X	No	Content	Substitution of CR for IR carbidopa/ levodopa	NA	100% who took incorrect formulation had ADEs	Carbidopa levodopa
	Pervanas *et al.,*/2016 [90]	USA	–	68 reported errors	–	X	X	Content	Incorrect medication	NM	NM	NM
	Lester *et al.,*/2017 [92]	USA	Dispensed 216,500,000 prescriptions	531,555 error reports	–	X	NM	NM	Other errors Incorrect directions	NM	NM	0.05% of errors caused harm
**Sub-Saharan Africa**	Malanguet *et al.,*/ 2012 [104]	South Africa	713 prescriptions analyzed	Dispensing errors: 12	–	X	No	Content	Omission error	No	No	Anti-retrovirals

*ADD: Automated Dispensing Device ADE: Adverse Drug Event CR: Controlled Release DAA: Dose administration aid IR: Immediate Release NM: Not Mentioned RX: Prescription PRE: Prescription-related error PCE: Pharmacist counselling error UAE: United Arab Emirates UK: United Kingdom USA: United States of America

Within the Europe and Central Asia region, the dispensing error rate ranged from 7.1 undocumented dispensing errors per 100,000 dispensed prescriptions in a Finnish study [40] to 3.3% in a study conducted in the UK [28]. Most studies (n = 9) addressed both content and labelling errors [38,50,56,28,58–62] with content errors being the predominant type across all studies. Conversely, five studies focused solely on content errors [24,43,47,49,51]. Among content errors, incorrect dose or strength was the most frequently observed error in six studies [24,28,38,43,60,61], followed by incorrect drug or form in five studies [49,56,58,59,62]. On the other hand, incorrect directions constituted the most common labelling errors [58,59,61,28]. Four studies documented the severity of medication errors [24,38,45,28], with over 50% of errors causing either ‘no harm’ or ‘minor harm’ [24,45,28].

In North America, the range of dispensing error rates varied significantly, from 0.075% [84] to 24% [82]. Among the six studies that evaluated both content and labelling errors [77,82–84,90,80], the latter emerged as the most prevalent issue in three studies [82–84]. Conversely, three studies exclusively focused on content errors [87,88,92]. The most frequently observed content errors included incorrect medication [77,88,90,80] and incorrect quantity [82–84]. Wrong labelling instructions were consistently reported as the most common labelling error for four studies [77,83,84,80]. Additionally, four studies provided insights into the severity of errors using different definitions [81,87,88,80]. For instance, in one study the degree of harm was related to the patient outcome using the following categories: no error, no harm, mild harm, moderate harm, severe harm and death [81]. These studies revealed that the percentage of errors resulting in harm or ADEs varied from 13.6% [87] to 100% [88].

Within the MENA region, the range of dispensing error rates varied from 0.8% in two Yemen-based studies [74,75] to 36.7% in a study conducted in Iran [68]. Among the conducted studies, three exclusively concentrated on content errors [71,74,75], while two addressed both content and labelling errors [69,72]; content errors consistently emerged as the predominant type across all studies [69,71,72,74,75]. Within these studies, incorrect quantity was identified as the most common content error in two studies [69,72], while wrong dosage form prevailed in another two studies [74,75]. Notably, no information regarding the nature of labelling errors was available for the two studies that examined both labelling and content errors [69,72]. Furthermore, two studies provided insights into the severity of dispensing errors, indicating that over 50% of errors were classified as ‘moderate’ to ‘severe’ [69,72].

Only one of the three studies conducted in the East Asia and Pacific region provided a specific figure for the dispensing error rate of 85.4% [35]. Two studies focused on both content and labelling errors, with content errors being the most frequent [34,36]. In the Australian study, the most prevalent content error was identified as incorrect concentration/strength [34], whereas the Korean study highlighted dosing errors as the primary error [36]. No data concerning labelling errors was available for these studies. Only one study targeted labelling errors with illegible medication details being the most common [35]. Furthermore, only one of these studies evaluated the severity of errors, indicating that 3.2% of errors were categorized as ‘mild’ to ‘moderate’ [36].

### Administration errors

The data derived from studies that specifically focused on administration errors as well as studies that targeted administration alongside other types of errors were synthesised in this section. Administration errors are summarised in Table 6. Overall, only one study, conducted in Canada, provided data on the administration error rate, revealing that an error occurred in 78% of participants [76], with the incorrect use of inhalers identified as the most prevalent administration error.[76]

**Table 6:** Administration Errors*.

						Study targeted RIGHT									
**Area[32]**	**Study Reference**	**Country**	**Denominator** **for admin error**	**Numerator** **for** **admin** **error**	**Incidence (I)/** **Rate** **(R)/** **Prevalence (P)** of admin* errors	**Patient** **error**	**Drug error**	**Dose error**	**Time error**	**Route** **error**	**Admin tech** **error**	**Admin instruct-ions error**	Most common admin* **error**	**Severity of admin errors**	**Top two implicated medication classes in administration errors**
**East** **Asia** **and Pacific**	Adie *et al.,*/2021 [34]	Australia	–	Admin errors: 238	–	X	X	X	X	X	X	X	Not following Instructions	NM	NM
	Han *et al.,*/2023 [36]	Republic of Korea	–	Admin errors: 171	–	X	X	X		X			Dosing error	Near miss: 0.3% No harm: 0.4% Mild harm: 0.4% Moderate harm: 0.1% Severe harm: 0%	NM
**Europe and Central Asia**	Knudsen *et al.,*/2007 [38]	Denmark	–	Admin errors: 50	–	NM	NM	NM	NM	NM	NM	NM	NM	NM	NM
	Cassidy*et al.,*/ 2011 [43]	Ireland	–	Admin errors: 2279	–		X	X	X	X			Double dose	NM	NM
	Cheung *et al.,*/2011 [46]	Netherlands	–	–	–	NM	NM	NM	NM	NM	NM	NM	NM	NM	NM
	Cheung *et al.,*/2014 [47]	Netherlands	–	Admin errors: 4	–	X							NM	NM	NM
	Jambrina *et al.,*/2023 [50]	Spain	–	Admin errors: 148	–	NM	NM	NM	NM	NM	NM	NM	NM	NM	NM
	Serrano *et al.,*/2016 [52]	Spain	–	Admin errors: 876	–						X		Inhalation technique	NM	NM
	Castel-Branco *et al.,*/2017 [54]	Portugal		Inhalation techniques:95							X		Inhalation technique	NM	NM
	Lynskey *et al.,*/2007 [62]	UK	–	Admin error: 1	–	NM	NM	NM	NM	NM	NM	NM	NM	NM	NM
**North America**	Makhinova *et al.,*/2020 [76]	Canada	201 patients		At least 1 error was observed in 78% of patients						X		Inhalation technique	NM	NM
	Boucher *et al.,*/2018 [81]	Canada	–	Admin related events: 2167	–	NM	NM	NM	NM	NM	NM	NM	NM	% of harmful admin events: 10.5%	NM

Admin: Administration NM: Not Mentioned Tech: Technique UK: United Kingdom

Within the Europe and Central Asia region, an Irish study reported that ‘doubling the dose’ was the most frequent administration error [43]. Similarly, studies from Spain [52] and Portugal [54], reported the incorrect usage of inhalers as primary administration errors. In the Netherlands, a study found administration of a medication to an incorrect patient to be the most frequent error [47].

In the East Asia and Pacific region, an Australian study comprehensively examined all administration errors [34], with not following prescribed instructions identified as the most common issue[34]In the Republic of Korea, a study targeted errors related to the right patient, right drug, right dose, and right route, with dosing administration errors being the most common [36]. Notably, only this Korean study reported the seriousness of medication errors, revealing that 0.8% of errors resulted in ‘none to mild harm’ [36].

### Risk of bias

The risk of bias assessment in included studies are shown in Table 7.

**Table 7:** Critical Appraisal of Included Studies [22].

Study Reference/Year published	Selection Bias	Identification Bias	Error Categorization Bias	Conflict of Interest Bias
Adie *et al.,*/2021 [34]	High	High	Low	Low
Uzunbay *et al.,*/2023 [35]	Low	Low	Low	Low
Han *et al.*,/2023 [36]	Low	High	High	Low
Ho *et al.,/*2012 [37]	High	Low	Low	Low
Knudsen *et al.,*/2007 [38]	Low	High	Low	Low
Volmer *et al.*,/ 2012 [39]	High	Unclear	Low	Low
Teinilä *et al.,*/2009 [40]	Unclear	High	High	Low
Timonen *et al.,*/2018 [41]	Low	High	High	Low
Sayers *et al.*,/2009 [42]	High	High	Low	Low
Cassidy *et al.,*/ 2011 [43]	Low	High	High	High
Cheptanari-birta *et al.,*/2022 [44]	Unclear	High	High	Unclear
Van Leeuwen *et al.,*/2001 [45]	Low	High	High	Low
Cheung *et al.,*/2011 [46]	Low	High	High	Low
Cheung *et al.,*/2014 [47]	Low	High	Low	Low
Haavik *et al.,*/2006[48]	Low	High	High	Low
de Las Mercedes Martínez Sánchez *et al.,*/2013 [49]	Low	Low	Low	Low
Jambrina *et al.,*/2023 [50]	High	High	High	Low
Rios *et al.,*/2015 [51]	Low	High	High	Unclear
Serrano *et al.,*/2016 [52]	High	High	High	Unclear
Drankowska *et al.,*/2021 [53]	Unclear	High	High	Low
Castel-Branco *et al.,*/2017 [54]	Low	High	High	High
Greene/1995 [55]	Low	High	High	Low
Kayne *et al.,*/1996 [56]	Unclear	High	High	Low
Chen *et al.,*/2005 [57]	Unclear	High	High	Unclear
Ashcroft *et al.,*/2005 [58]	Unclear	High	High	Low
Quinlan *et al.,*/2002 [59]	Low	High	High	Low
Chua *et al.,*/2003 [60]	High	High	High	Low
Warner and Gerrett/2005 [61]	High	High	High	Unclear
Lynskey *et al.,*/2007 [62]	Low	High	High	High
Franklin and O’Grady/2007 [28]	High	High	High	Unclear
Phipps *et al.,*/2017[24]	Low	High	High	Low
de Souza *et al.,*/2006 [63]	Low	Unclear	Unclear	Low
Diniz *et al.,*/2011 [64]	High	Low	Low	Low
da Silva *et al.,*/2012 [65]	Low	High	High	Low
Abuelsoud *et al.,*/ 2018 [66]	Low	High	Unclear	High
Kassem *et al.*,/2021 [67]	High	Low	High	Low
Sarhangi *et al.,*/2021 [68]	Unclear	Low	High	Low
Abdel-Qader *et al.,*/2021 [69]	Low	Low	Low	Low
Kamel *et al.,*/2018 [70]	High	High	High	Low
Soubra and Karout/2021 [71]	High	High	High	Low
Mohamed Ibrahim *et al.,*/2020[72]	Low	Low	Low	Low
Al-Worafi *et al.,*/2018 [73]	Low	Low	High	Low
Al-Worafi *et al.,*/2018 [74]	Unclear	High	High	Low
Al-Worafi *et al.*,/2018 [75]	Unclear	High	High	Low
Makhinova *et al.,*/2020 [76]	High	High	High	Low
Ledlie *et al.,*/2023 [77]	Low	High	High	Low
Sears *et al.,*/2016 [78]	High	High	High	Unclear
Aubert *et al.,*/2023 [79]	Unclear	Unclear	Unclear	Low
Lee *et al.,*/2023 [80]	High	Unclear	Low	Low
Boucher *et al.,*/2018 [81]	Low	High	High	Low
Allan *et al.,*/1995 [82]	High	Low	Low	Low
Flynn *et al.,*/2003 [83]	Unclear	Low	Low	Low
Teagarden *et al.,*/2005 [84]	Low	Low	Unclear	Low
Witte and Dundes/2007 [85]	High	High	High	High
Hoxsie *et al.,* /2006 [86]	Low	Unclear	Low	Low
Flynn*et al.*,/2009 [87]	High	Low	Low	Low
Khadem *et al.,*/2010 [88]	High	Low	High	Low
Nanji *et al.,*/ 2011 [89]	Low	Low	Low	Low
Pervanas *et al.,*/2016 [90]	Low	High	High	Low
Hincapie *et al.,*/2019 [91]	High	High	High	Low
Lester *et al.*,/2017 [92]	Low	High	High	Low
Reed-Kane *et al.,*/2014 [93]	Low	High	High	Unclear
Odukoya *et al.,*/2014 [94]	Low	Low	Low	Low
Vo and Molitor/2022 [95]	Low	High	High	Low
Patel *et al.,*/2005 [96]	Unclear	Low	Low	High
Joshi *et al.,*/2016 [97]	Unclear	High	High	Low
Marwaha *et al.,*/2010 [98]	Low	High	High	Low
Rathi *et al.,*/2022 [99]	High	High	High	Low
Atif *et al.,*/2018 [100]	Low	Unclear	Low	Low
De Silva *et al.,*/2015 [101]	High	Low	High	Low
Anagaw *et al.*,/2023[102]	Low	Unclear	Low	Low
Simegn *et al.,*/2022 [103]	High	High	High	Unclear
Malangu *et al.,*/2012 [104]	Low	High	High	Low

The majority of studies (n = 36) had a low risk of selection bias

[24,35,36,38,41,43,45–49,51,54,55,59,62,63,65,66,69,72,73,77,81,84,86,89,90,92–95,98,100,102,104],

while 24 studies were deemed to have a high risk of selection bias [39,42,50,52,60,61,28,76,78,82,85,87,88,91,80,67,70,71,99,101,34,37,64,103].

Most studies (n = 48) were identified to have a high risk of identification bias [24,38,40–48,] [28,34,36,50–62,65,66,70,71,74–78,81,85,90–93,95,97–99,103,104] whereas 18 studies were classified as having low risk [49,82–84,87–89,94,67–69,72,73,96,101,37,35,64].

Similarly, the majority of studies (n = 48) were categorised as having a high risk of error categorisation bias [24,40,41,43–46,48, 28,36,48,50–62,65,67,68,70,71,73–78,81,85,88,90–93,95,97–99,101,103,104] while 21 studies were deemed to have a low risk [34,35,37–39,42,47,49,64,69,72,80,82,83,86,87,89,94,96,100,102]. Concerning conflict of interest bias, most studies were classified as having a low risk for bias (n = 58) [24,34–42,45–50,53,55,56,58–60,63–65,67–77,79–84,86–92,94,95,97–102,104] with only six studies considered at high risk [43,54,62,85,66,96].

## Discussion

This systematic review assessed medication errors reported in community pharmacies across the international literature, published between 1995 and 2023. The findings of this review indicated that most studies targeting medication errors in community pharmacies were primarily conducted in Europe and Central Asia and North America with relatively very few studies conducted in other regions. Moreover, most of the included studies focused exclusively on prescribed medications rather than over-the-counter medications.

In this review reported rates of errors demonstrated significant heterogeneity, displaying a wide range between studies. This result supports the findings from previous systematic reviews on medication errors [5,16,20,22,105,106]. For instance, Assiri *et al.,* undertook a systematic review of international literature to examine medication error epidemiology in community care settings, including community pharmacies[20] . The findings from this review revealed a wide range of reported or period prevalence rates for medication errors, ranging from 2% to 94% [20]. Furthermore, Alsulami *et al.,* conducted a systematic review on the incidence and types of medication errors in Middle Eastern nations. This study highlighted the considerable challenges of comparing medication error incidence between studies [105].

The variation in reported error rates in this review can be at least partially attributed to differences in the definition of medication errors, variations in denominator calculations, differences in study populations, and the diverse methodologies used for error identification.

This review illustrates that medication errors vary across geographical region. Consistent with previous research conducted across different populations and settings, prescribing errors were a common concern globally with dispensing errors gaining attention especially in Europe and Central Asia, North America, and the MENA region [20,22,105].

Concerning the type of prescribing errors, in Europe and Central Asia, as well as in the East Asia and Pacific regions, commission errors predominated as the most prevalent prescribing errors, while omission errors were most frequent in other regions. Assessment of error severity was predominantly conducted in Europe and Central Asia, showing that most errors were non-serious, whereas studies in North America demonstrated a higher rate of unsafe errors. Regarding dispensing errors, content errors were found to be the main errors across all regions. Content errors included incorrect dose or strength, incorrect drug or dosage form. The evaluation of dispensing error severity was only carried out in a limited number of studies and displayed variability among the different regions. It is noteworthy to mention that the systems used for identifying, classifying and assessing the severity of both dispensing and prescribing errors were often unclear. Moreover, the impact of errors on patient outcomes was also not captured in the majority of studies. These findings are similar to those of other previous systematic reviews [22,105]. For instance, in a systematic review of medication errors across Middle East countries, Alsulami *et al* noted that most of the included studies did not evaluate the clinical implications of documented medication errors and only 13% of studies categorised the severity of these errors [105].

Transcribing, administration, monitoring errors and errors occurring in other stages of the medication use process were the least targeted in all regions[20,105]. These findings align with those of other systematic reviews. For example, in their systematic review, Alsulami *et al.,* indicated that only one study in Iran assessed transcribing errors [105]. Furthermore, in Assiri *et al.’s* systematic re*view* monitoring errors were only measured in one study in Lebanon [20]. The limited research on these errors might skew the perception of the global error rate, distorting the true burden of medication errors in community pharmacies.

Overall, the risk of identification bias in included studies conducted in diverse regions was high. Identification bias pertains to the approach employed in detecting whether a prescription truly contains an error. The high identification bias can be attributed to the lack of a consensus process by more than one study investigator to assess prescriptions for errors [22]. Moreover, it can be related to the reliance on self-reporting mechanisms used in a high percentage of studies, which could underreport the rate of errors [22]. Ideally despite the potential influence of the Hawthorne Effect, where observation may significantly alter individuals’ behaviour, the use of a trained observer would likely enhance error detection and reduce the risk of identification bias [107]. However, the constraints of time and cost with this approach might have limited its use [16,23]. Categorisation of error bias was also high. Misclassification of errors can lead to artificially high or low error rates depending on the category assigned [22]. What system or approach used to classify errors in included studies was not evident. A standardised system for error categorisation or classification should have been employed to ensure comparability of errors across studies. Moreover, it would have been desirable to have two or more trained investigators in each study to categorise errors and resolve any disagreements through consensus.

### Future aspects

This review provides valuable worldwide insights on medication safety in community pharmacies that can help inform international policy initiatives focused on monitoring, decreasing and preventing medication errors. Moreover, the findings can assist in making informed decisions about where to allocate funding for medication safety initiatives, aimed at alleviating the burden caused by medication errors in different regions and for improving the patient safety culture in community pharmacy settings internationally. One of these initiatives could include the implementation of electronic systems as many reviews have highlighted their effectiveness in decreasing errors within hospital settings [108] and they may also prove effective in community pharmacy settings. Additional strategies encompass setting efficient systems among pharmacy personnel based on teamwork, communication and the no-blame culture [109]. Other strategies include the development of prescribing charts and guidelines [108] alongside process related interventions such as the adoption of the World Health Organisation medication safety guide for look-alike, sound-alike medicines [110]. This review also underscores the role of community pharmacists in adopting patient and medication safety measures. Community pharmacists should be encouraged to implement different strategies and systems to minimise medication errors, including but not limited to enhancing their vigilance in checking prescriptions to mitigate prescription errors, using technology, adopting operational flowcharts or tools, double verifying medications before dispensing, and improving public awareness of the importance of medication safety and reporting medication errors. In fact, there is strong evidence illustrating the impact of pharmacists on reducing medication errors in different healthcare settings. For instance, Gillani *et al.,* highlighted the important role pharmacists play in preventing and in raising awareness about medication errors and in implementing appropriate reporting policies [111]. Moreover, in a systematic review examining the impact of pharmacist interventions on medication errors in hospitalised paediatric patients, Naseralallah *et al.*, concluded that pharmacist involvement can lead to considerable reductions in the overall rate of medication errors [112]. Moreover, as recommended by the IOM, the International Pharmaceutical Federation (FIP) and the WHO [113], all pharmacists and undergraduate pharmacy students should receive comprehensive education and training in pharmacotherapy, and medication safety so that they would have the required knowledge, and skills to detect medication errors and be enabled to act accordingly to resolve issues. [114]Involving pharmacists who possess expertise in medication safety in this setting could also be considered, mirroring the practice observed in hospitals. Educational programs are also required for medical and non-medical prescribers in the outpatient sector to improve their prescribing competency with attention given to the different types of prescribing errors encountered in each region whether omission or commission errors [115].

The scarcity of research on medication errors in community pharmacies in the African and South-East Asian regions highlighted in this review necessitates an urgent attention by policy makers, healthcare professionals and researchers. In fact, according to the 2021 report by the FIP on community pharmacies, the pharmacist-to-pharmacy ratio in these regions is less than one, suggesting that certain pharmacies operate without a pharmacist [116]. Coupled with one of the highest rates of preventable medication harm in these regions, as indicated by the 2024 WHO report on the global burden of preventable medication harm [117], there are growing concerns about medication safety in community pharmacies within these areas. Furthermore, due to the scarcity of comprehensive international data concerning administration, transcribing and monitoring errors, as well as dispensing and prescribing errors in regions where these assessments have been lacking, there is a pressing need to conduct more well-designed studies in these areas.

This review showed that most studies targeted prescription medications with very few targeting over-the-counter or non-prescription medications. Non-prescription medications are accessible directly from pharmacies or other outlets without the need for a prescription. While promoting self-care, the accessibility of non-prescription medications has nurtured a common belief by the general public in relation to the safety of these medications often leading to insufficient awareness regarding their risks of misuse, dependency, and harm. Hence the gap in evidence on over-the-counter medication related errors also needs to be addressed in future studies [118,119].

The results of this review indicate a lack of high-quality research on medication errors in community pharmacies. Therefore, future studies should employ standardised error reporting methods that adhere to universal definitions or taxonomy of medication errors and classifications for error types and severity. Additionally, they should utilise specific denominators to enable the assessment of error rates across studies and should have a thorough analysis of the severity of these errors by assessing the potential harm on patients. Tools that could be used for this purpose include the NCC MERP Index for Categorising Medication Errors, which classifies harm into eight categories [120]. Moreover, the review indicated that policy guidelines, tools or frameworks should be designed for reporting and calculating community pharmacy error rates so that comprehensive data would be accurately and consistently captured. This is necessary for providing a more precise depiction of the safety levels associated with medication errors in community pharmacies globally and will help in identifying the causes of errors and in designing system level interventions for preventing and alleviating errors.

## Strengths and limitations

This systematic review had several strengths. It is one of the most comprehensive reviews of the literature on medication errors in community pharmacies across the world. The review used a robust methodology that included no language restrictions, screened multiple databases, and searched the grey literature, leading to identification of 73 included studies. The review also targeted all stages of the medication use process including prescribing, transcribing and documenting, dispensing, administration and monitoring [18]. Nevertheless, the review had some limitations. It did not assess the contributary factors and causes of medication errors or strategies to reduce errors; however, this was not an aim of the study. Moreover, the heterogeneity of included studies prevented conducting any meta-analysis. In addition, the heterogeneity presented difficulties in comparing error rates and types within studies conducted in the same geographical region and across different regions, which posed challenges for estimating a global error rate.

## Conclusions

This systematic review represents one of the few efforts to describe medication errors on a global scale within community pharmacies. The objective of this review was to synthesise and critically appraise the scientific literature that has documented or assessed medication errors in community pharmacies across the world.

The results of this review revealed significant variation in medication error rates among studies, which can be explained by differences in error definitions, denominators, methodologies, and other studies’ characteristics. This highlights the need to use standardised global definitions, taxonomy and denominators or frameworks to enable assembling, synthesis and comparison of data. Additionally, in this review, studies were mostly conducted in Europe, Central Asia, and North America. Therefore, it is imperative to assess medication safety in other regions of the world, especially those where pharmacy services are difficult to access. The review also indicated that most studies focused on prescribing and dispensing, while administering, monitoring and errors occurring in other stages of the medication use process received relatively less attention. In addition, very few studies examined the severity and potential for harm of the reported medication errors. This lack of this information may prevent policy makers and healthcare providers from understanding the true extent of this problem and implementing effective prevention and mitigation strategies.

## Supporting information

Supplementary File 1 Search strategies. (PDF)

Supplementary File 2 Excluded articles at first step. (PDF)

Supplementary File 3 Excluded full text articles and reasons for exclusion. (XLSX)
