# Supplementary material for: Medication errors in community pharmacies: a systematic review of the international literature
Source: PLoS One. 2025 May 20;20(5):e0322392. doi: 10.1371/journal.pone.0322392 (PMC12091811; doi:10.1371/journal.pone.0322392)
Supplement: Supplementary File 1 — (PDF) [file pone.0322392.s001.pdf]

Supplementary File 1. Search Strategies

Medline/Ovid

1. exp medication errors/ OR exp near miss, healthcare/
2. medic* error.mp.
3. medic* incident.mp.
4. medic* incidence.mp.
5. medic* harm.mp.
6. medic* risk.mp.
7. medic* event.mp.
8. medic* safe*.mp.
9. medic* problem.mp.
10. medic* discrepancy.mp.
11. medic* intervention.mp.
12. exp Medication Therapy Management/
13. medic* mistake.mp.
14. patient error.mp.
15. drug error.mp.
16. drug incident.mp.
17. drug incidence.mp
18. drug harm.mp.
19. drug risk.mp.
20. drug event.mp.
21. drug safe*.mp.
22. drug problem.mp.
23. drug discrepancy.mp.
24. drug intervention.mp.
25. drug manag*.mp.
26. drug mistake.mp.
27. Inappropriate Prescribing/
28. inappropriate prescribing.mp.
29. overprescribing.mp.
30. wrong drug.mp.
31. wrong medication.mp.
32. wrong route of administration.mp.
33. wrong dose.mp.
34. wrong calcul*.mp
35. 1 or 2 or 3 or 4 or 5 or 6 or 7 or 8 or 9 or 10 or 11 or 12 or 13 or 14 or 15 or 16 or 17 or 18 or 19 or 20 or 21 or 22 or 23 or 24 or 25 or 26 or 27 or 28 or 29 or 30 or 31 or 32 or 33 or 34

36. community pharmac*.mp.
37. community Pharmacy Services/
38. retail pharmacy.mp.
39. private pharmacy.mp.
40. outpatient pharmac*.mp.
41. exp Pharmacies/
42. Pharmacists/
43. ambulatory pharmacy.mp.
44. drug outlet.mp.
45. drug shop.mp.
46. medication outlet.mp.
47. medication shop.mp.
48. medicine outlet.mp.
49. medicine shop.mp.
50. 36 or 37 or 38 or 39 or 40 or 41 or 42 or 43 or 44 or 45 or 46 or 47 or 48 or 49
51. 35 AND 50

Embase

1. exp medication error/ or exp medical error/ or exp dispensing error/ or exp dosing error/ or exp inappropriate polypharmacy/ or exp potentially inappropriate medication/ or exp medication labelling device/
2. drug error.mp.
3. medic* harm.mp.
4. medic* incident.mp.
5. medic* incidence.mp.
6. medic* risk.mp.
7. medic* event.mp.
8. medic* safe*.mp.
9. medic* problem.mp.
10. exp medication therapy management/
11. medic* discrepancy.mp.
12. medic* intervention.mp.
13. medic* mistake.mp.
14. exp patient harm/ or exp patient risk/
15. exp medication error/ or exp prescribing error/
16. drug incident.mp.
17. drug incidence.mp.
18. drug harm.mp.
19. drug risk.mp.
20. drug event.mp.
21. drug problem.mp.

22. drug discrepancy.mp.
23. drug intervention.mp.
24. drug manag*.mp.
25. inappropriate prescribing/ or Inappropriate Prescrib*.mp.
26. overprescribing/ or overprescrib*.mp.
27. wrong drug.mp.
28. wrong medic*.mp.
29. wrong route of administration.mp.
30. wrong dose.mp.
31. wrong calculation*.mp.
32. 1 or 2 or 3 or 4 or 5 or 6 or 7 or 8 or 9 or 10 or 11 or 12 or 13 or 14 or 15 or 16 or 17 or 18 or 19 or 20 or 21 or 22 or 23 or 24 or 25 or 26 or 27 or 28 or 29 or 30 or 31
33. "pharmacy (shop)"/ or community pharmacist/ or pharmacist/ or community pharmacy/ or community pharmac*.mp. or pharmacy/
34. retail pharmac*.mp.
35. outpatient pharmac*.mp.
36. ambulatory pharmacy*.mp.
37. private pharmac*.mp.
38. drug outlet.mp.
39. medicine outlet.mp.
40. medication outlet.mp.
41. 33 or 34 or 35 or 36 or 37 or 38 or 39 or 40
42. 32 AND 41

Web of Science

#1	TS= (community OR private OR outpatient OR retail) AND (Pharmacy OR shop OR outlet)
#2	TS=((Drug OR medic* OR patient OR therapy) AND (Safe* OR err* OR harm OR risk OR event OR problem OR incident OR concern OR quality OR interaction or discrepancy Or inappropriate OR intervention OR manag* OR mistake))
#1 AND #2	

CINAHL

#1	Abstract= pharmacy or pharmacies or pharmacist or pharmacists
#2	Abstract= Medication Error (MESH)
#1 AND #2	

Scopus

#1	Title, abstract, keyword= (community OR private OR retail) AND (Pharmacy)
#2	Title, abstract, keyword =((Drug OR medic* OR patient OR therapy) AND (Safe* OR err* OR harm OR risk OR event OR problem OR incident OR quality OR interaction or discrepancy Or inappropriate OR intervention OR mistake))
#1 AND #2	

Google Scholar

1. ((community OR private OR outpatient OR ambulatory OR retail) **AND** Pharmacy) AND ((Drug OR medic* OR patient OR therapy) **AND** (Safe* OR err* OR harm OR risk OR event OR problem OR incident OR concern OR quality OR interaction or discrepancy Or inappropriate OR intervention OR manag* OR mistake)) (50 pages)
2. Community pharmacy and medication error (50 pages)
3. dispensing errors in community pharmacy (50 pages)
4. transcription error in community pharmacy (50 pages)
5. prescrib error in community pharmacy (50 pages)

ProQuest Dissertations & Theses Global

#1	All abstract and summary text - summary= (community OR private OR outpatient OR ambulatory OR retail OR drug OR med*) AND (Pharmacy OR shop OR outlet)
#2	All abstract and summary text - summary= ((Drug OR medic* OR patient OR therapy) AND (Safe* OR err* OR harm OR risk OR event OR problem OR incident OR concern OR quality OR interaction or discrepancy Or inappropriate OR intervention OR manag* OR mistake))
#1 AND #2	

ScienceDirect

Title, abstract or author-specified keywords**: (Safety OR error OR harm OR risk OR event OR problem OR incident) AND (pharmacy OR pharmacist) (no date limiting 1999 up)**

Global Health Database

#1	Abstract= (community OR private OR outpatient OR ambulatory OR retail) AND (Pharmacy)
#2	Abstract=(Safe* OR err* OR harm OR risk OR event OR problem OR incident OR concern OR quality OR interaction or discrepancy Or inappropriate OR intervention OR manag* OR mistake))
#1 AND #2	

Health Systems Evidence

((community OR private OR outpatient OR ambulatory OR retail) **AND** Pharmacy) AND ((Drug OR medic* OR patient OR therapy) **AND** (Safe* OR err* OR harm OR risk OR event OR problem OR incident OR concern OR quality OR interaction or discrepancy Or inappropriate OR intervention OR manag* OR mistake))

Academic search complete (EBSCO)

1. medication errors or medication safety

Health Management Information Consortium

1. medication error.mp. or exp Medication errors/
2. pharmacy.mp. or exp Community pharmacy/ or exp Pharmacy/ or exp Mail order pharmacy/
3. medication incident.mp.
4. medication harm.mp.
5. medication risk.mp.
6. medication event.mp.
7. medication safety.mp. or exp Errors/
8. medication problem.mp.
9. medication discrepancy.mp.
10. medication intervention.mp.
11. medication therapy management.mp.
12. medication mistake.mp.
13. drug error.mp.
14. drug incident.mp.
15. drug harm.mp.
16. drug risk.mp.
17. drug event.mp.
18. drug problem.mp.
19. drug safe.mp.
20. drug problem.mp.
21. inappropriate prescribing.mp.
22. drug intervention.mp.
23. wrong drug.mp.
24. wrong medication.mp.
25. exp "Appropriateness of care"/ or exp Over prescribing/
26. overprescribing.mp.
27. wrong route of administration.mp.
28. wrong dose.mp.
29. wrong calculation.mp.
30. 1 or 3 or 4 or 5 or 6 or 7 or 8 or 9 or 10 or 11 or 12 or 13 or 14 or 15 or 16 or 17 or 18 or 19 or 20 or 21 or 22 or 23 or 24 or 25 or 26 or 27 or 28 or 29
31. 2 and 30

Cochrane

#1	(Safe* OR err* OR harm OR risk OR event OR problem OR incident OR concern OR quality OR interaction or discrepancy Or inappropriate OR intervention OR manag* OR mistake)
#2	Pharmacy OR pharmacist
