# Supplementary material for: Medication errors in community pharmacies: a systematic review of the international literature
Source: PLoS One. 2025 May 20;20(5):e0322392. doi: 10.1371/journal.pone.0322392 (PMC12091811; doi:10.1371/journal.pone.0322392)
Supplement: Supplementary File 2 — (PDF) [file pone.0322392.s002.pdf]

Supplementary File 2.
Excluded Articles at First Step

1. Handled with care?: managing medication for residents of care homes and children's homes: a follow up study.

2. The health and care of older people in care homes: a comprehensive interdisciplinary approach: a report of a joint working party.

3. Impact assessment : rebalancing medicines legislation and pharmacy regulation programme: dispensing errors.

4. Impact assessment : rebalancing medicines legislation and pharmacy regulation programme: registered pharmacy standards and related matters.

5. Investigation into drug dependency services provided by Bolton, Salford and Trafford Mental Health NHS Trust.

6. NHS Discharge Medicines Service: essential service: toolkit for pharmacy staff in community, primary and secondary care.

7. Patient safety solutions.

8. The Pharmacy (Preparation and Dispensing Errors) Order 2015.

9. The prevention of intrathecal medication errors: a report to the Chief Medical Officer by Kent Woods.

10. Rebalancing medicines legislation and pharmacy regulation : dispensing errors and pharmacy standards and related matters: equality analysis.

11. Rebalancing medicines legislation and pharmacy regulation: consultation on draft orders under section 60 of the Health Act 1999. Pharmacy (Preparation and Dispensing Errors - Hospitals and Other Pharmacy Services) Order 2018 ; and Pharmacy (Responsible Pharmacists, Superintendent Pharmacists etc.) Order 2018.

12. Rebalancing medicines legislation and pharmacy regulation: draft orders under section 60 of the Health Act 1999: consultation document.

13. Rebalancing medicines legislation and professional regulation: draft orders under section 60 of the Health Act 1999 consultation: frequently asked questions.

14. [The report of the Short Life Working Group on reducing medication-related harm].

15. A spoonful of sugar: medicines management in NHS hospitals.

16. Events of pharmacy in 1933. The Journal of the American Pharmaceutical Association (1912). 1934;23(1):1-2. doi: https://doi.org/10.1002/jps.3080230101.

17. The Year in Review. Journal of the American Pharmaceutical Association (Practical Pharmacy ed). 1955;16(12):750-3. doi: https://doi.org/10.1016/S0095-9561(16)33892-0.

18. Mail-Order Prescription Schemes. Journal of the American Pharmaceutical Association (Practical Pharmacy ed). 1960;21(2):66-7. doi: https://doi.org/10.1016/S0095-9561(16)35893-5.

19. interprofessional problems ... national association of boards of pharmacy. Journal of the American Pharmaceutical Association (1961). 1961;1(6):370. doi: https://doi.org/10.1016/S0003-0465(15)33588-6.

20. The microbiological risk of mixing TPN-solutions at the ward compared with the standard pharmacy mixing: M.Suistomaa, A.Kari, J.Takala, M-L.Katila. Critical Care Research Program. Kuopio University Central Hospital. Findland. Clinical Nutrition. 1987;6:102. doi: https://doi.org/10.1016/0261-5614(87)90256-1.

21. Risk of venous thromboembolism and stroke associated with oral contraceptives containing lower oestrogen dosage: M.Pini∗, F.Caliumi∗∗, U.Scoditti°, G.Ciarlini+, T.Poli∗, R.Quintavalla∗, C.Pattacini∗. ∗ 5th Medical Division and ∗∗ Department of Pharmacy, General Hospital of Parma, °Department of Neurology and +Department of Obstetrics and Gynecology, University of Parma, Italy. Thrombosis Research. 1993;70:S37. doi: https://doi.org/10.1016/0049-3848(93)90321-E.

22. Short-term ibuprofen is safe for kids. Modern Medicine. 1995;63(5):11. PubMed PMID: 9506154074.

23. A reputation for quality care. Joicfp News. 1995;(248):4.

24. Childhood injuries and pediatric medical emergencies. Journal of Pharmacy Technology. 1995;11(4):191-2.

25. COGNITIVE AND FUNCTIONAL IMPAIRMENT. American Family Physician. 1995;51(3):633-6. PubMed PMID: WOS:A1995QJ94100004.

26. Parents should give medicine cautiously. USA Today Magazine. 1996;125(2617):7. PubMed PMID: 9610117766.

27. 081068 (E60) Intuitive toxicology: Slovic P., Malmfors T., Krewski D., Mertz C.K., Nell N., Decision Research, Oregon, Malmfors Consulting, Stockholm, Health Canada, Ottawa, University of Washington, School of Pharmacy, Washington, Risk Analysis, Vol. 15, Number 6, 1995, pp. 661–675. Insurance: Mathematics and Economics. 1996;18(2):151. doi: https://doi.org/10.1016/0167-6687(96)81449-1.

28. New class of asthma drug safely improves on usual care. Modern Medicine. 1997;65(1):41. PubMed PMID: 9702034233.

29. 'Real world' statin comparison finds fluvastatin the most cost effective. Formulary (Cleveland, Ohio). 1997;32(10):1019-. PubMed PMID: CN-01763762.

30. The One Minute Counselor: A Pharmacist's Guide to the Benefits and Risks of Estrogen Replacement Therapy. Journal of the American Pharmaceutical Association (1996). 1997;37(3):305-6. doi: https://doi.org/10.1016/S1086-5802(16)30222-4.

31. ASHP therapeutic position statement on strategies for identifying and preventing pneumococcal resistance. American Journal of Health-System Pharmacy. 1997;54(5):575-8. doi: 10.1093/ajhp/54.5.575.

32. Medicines in schools. Implementing good practice in mainstream schools: A guide for pharmacists. Pharmaceutical Journal. 1997;258(6925):69-72.

33. Alcoholism medication can be used in wide variety of clinical settings. Modern Medicine. 1998;66(3):21. PubMed PMID: 380027.

34. Healing Medications That Hurt Your Teeth. Heart & Soul. 1998;(29):28. PubMed PMID: 1149386.

35. Pain reliever taken off market due to possible liver damage. FDA Consumer. 1998;32(5):5. PubMed PMID: 1081878.

36. Foreword. In: Möbius D, Miller R, editors. Studies in Interface Science. 7: Elsevier; 1998. p. v-vii.

37. Better reporting, forms and procedures reduce medication errors. Executive solutions for healthcare management. 1998;1(8):16-8.

38. Automation is prescription for revamping pharmacy services at North Carolina hospital. Health care cost reengineering report. 1998;3(4):53-6.

39. Around Uganda. Africa health. 1998;20(5):38.

40. The impact of the Internet on pharmacy practice. International Pharmacy Journal; 1999.

41. Nicotine replacement therapy. MeReC Bulletin. 1999;10(3):9-12.

42. Liability for Errors in Expanded Pharmacy Practice: Issues for Technicians and Pharmacists: Medication errors should be viewed as•an opportunity to identify system problems and generate solutions-not to assign individual blame. Journal of the American Pharmaceutical Association (1996). 2000;40(5, Supplement 1):S56-S7. doi: https://doi.org/10.1016/S1086-5802(16)31196-2.

43. Preventing Dangerous Drug Interactions: Pharmacists need to determine which combinations of medications are potentially dangerous to avert potential problems. Journal of the American Pharmaceutical Association (1961). 2000;40(5, Supplement 1):S44-S5. doi: https://doi.org/10.1016/S0003-0465(15)33230-4.

44. Obesity: Managing Comorbidities of the Disease: Pharmacists can help patients lose weight and reduce health risks associated with obesity, the nation's second leading cause of preventable morbidity and mortality. Journal of the American Pharmaceutical Association (1961). 2000;40(5, Supplement 1):S42-S3. doi: https://doi.org/10.1016/S0003-0465(15)33229-8.

45. Medical error report galvanizes public, health care community. American journal of health-system pharmacy : AJHP : official journal of the American Society of Health-System Pharmacists. 2000;57(1):9,13-4. doi: 10.1093/ajhp/57.1.9.

46. Improving cancer care and treatments. Pharmaceutical journal. 2001;267(7169):510-. PubMed PMID: CN-01749351.

47. Medical abortion at 57 to 63 days' gestation with a lower dose of mifepristone and gemeprost. A randomized controlled trial. Acta obstetricia et gynecologica Scandinavica. 2001;80(5):447-51. PubMed PMID: CN-00347593.

48. The real world costs of pancreatic cancer. Oncology spectrums. 2001;2(3):204-. PubMed PMID: CN-01725739.

49. Understanding Medication Errors: While long-range system-wide changes are necessary, pharmacists can take many immediate steps to minimize the risk of medication errors. Journal of the American Pharmaceutical Association (1996). 2001;41(5, Supplement 1):S52-S3. doi: https://doi.org/10.1016/S1086-5802(16)31420-6.

50. How to Respond When a Medication Error Occurs: Effective communication skills can help pharmacists maximize resolution and minimize the negative impact of medication errors. Journal of the American Pharmaceutical Association (1961). 2001;41(5, Supplement 1):S54-S5. doi: https://doi.org/10.1016/S0003-0465(15)33364-4.

51. The Risk Revolution: Challenges and Opportunities for Pharmacists: The time is rightfor pharmacists to

help devise and implement a more effective risk management system. Journal of the American Pharmaceutical Association (1996). 2001;41(6, Supplement 1):S18-S20. doi: https://doi.org/10.1016/S1086-5802(16)31437-1.

52. Comprehensive Weight Management: With the prevalence of obesity rising at an alarming rate, pharmacists can help patients reduce their excess weight and risk of major comorbid disorders. Journal of the American Pharmaceutical Association (1961). 2001;41(5, Supplement 1):S34-S5. doi: https://doi.org/10.1016/S0003-0465(15)33354-1.

53. Hormone Replacement Therapy: Sorting Out the Facts: Pharmacists need to be well versed in the risks and benefits of hormone replacement therapy to adequately counsel postmenopausal women. Journal of the American Pharmaceutical Association (1996). 2001;41(5, Supplement 1):S38-S9. doi: https://doi.org/10.1016/S1086-5802(16)31413-9.

54. New Directions in the Management of Special Risk Medications: In a changing health care environment, pharmacy is poised to improve safe medication use. Journal of the American Pharmaceutical Association (1996). 2001;41(6, Supplement 1):S3-S4. doi: https://doi.org/10.1016/S1086-5802(16)31433-4.

55. The flu shot: A pharmacists' guide to promoting flu shots. Canadian Pharmaceutical Journal. 2001;134(7):41-6.

56. OTC medicines misuse and addiction: What can pharmacists do to help? Pharmaceutical Journal. 2001;267(7168):457.

57. Managing current risks in community pharmacy. Pharmaceutical Journal; 2001.

58. 'It's Hard for Parents To Understand' (cover story). Newsweek Publishing LLC; 2002. p. 60.

59. Care track. Time to tackle suboptimal medication prescribing for the elderly? Joint Commission Benchmark. 2002;4(3):8-9. PubMed PMID: 106961634. Language: English. Entry Date: 20020920. Revision Date: 20150711. Publication Type: Journal Article.

60. Intervention required to reduce inappropriate prescribing of psychotropics in the elderly. Drugs & Therapy Perspectives. 2002;18(10):17-20. PubMed PMID: 106847685. Language: English. Entry Date: 20030711. Revision Date: 20150711. Publication Type: Journal Article.

61. Debate centres on whether pharmacy practice research has enough legs. Pharmaceutical journal. 2002;268(7196):624-. PubMed PMID: CN-01767564.

62. Is alternate day dosing more cost-effective? Pharmaceutical journal. 2002;269(7224):706-. PubMed PMID: CN-01775491.

63. E-Pharmacy: Improving Patient Care and Managing Risk: As purveyors of information in a Web-based world, pharmacists are important elements in the brave new health care system. Journal of the American Pharmaceutical Association (1996). 2002;42(5, Supplement 1):S20-S1. doi: https://doi.org/10.1331/108658002764653657.

64. Trends and Events in American Pharmacy, 1852–2002: For 150 years, the progress of American pharmacy and the development of the American Pharmaceutical Association have been intertwined. Journal of the American Pharmaceutical Association (1996). 2002;42(5, Supplement 1):S24-S5. doi: https://doi.org/10.1331/108658002764653673.

65. JCAHO: Meeting the Standards for Patient Safety: A focus on medication safety is required under Joint

Commission standards, and tools are available to help pharmacists achieve the best possible outcomes. Journal of the American Pharmaceutical Association (1996). 2002;42(5, Supplement 1):S54-S5. doi: https://doi.org/10.1331/108658002764653800.

66. Drug-Related Problems in Older Adults: Pharmacists need to identify and recommend interventions to prevent medication mishaps in the elderly. Journal of the American Pharmaceutical Association (1996). 2002;42(5, Supplement 1):S32-S3. doi: https://doi.org/10.1331/108658002764653708.

67. Is It Legitimate? Strategies for Assessing Questionable Prescriptions: How can pharmacists balance the risk of dispensing a false prescription with the duty to provide needed medication to a legitimate patient? Journal of the American Pharmaceutical Association (1996). 2002;42(5, Supplement 1):S58-S9. doi: https://doi.org/10.1331/108658002764653817.

68. Homocysteine as a risk factor of atherosclerotic lesion: R.M. Santos1, J.P. Freitas1, M.E. Macedo2, A.A. Monteiro2, M.I. Rebelo3, E.M. Castro3, A.S. Silva3, L. Belo3, A.F. Freitas2. 1Centro Estudos Funcao Autonomica, Porto; 2Sao Joao Medical School, 3 Faculty of Pharmacy, Porto, Portugal. Atherosclerosis Supplements. 2002;3(2):200. doi: https://doi.org/10.1016/S1567-5688(02)80623-7.

69. Don't forget about yellow cards. Pharmaceutical Journal. 2002;269(7205):2.

70. Oseltamivir for infleunza. Drug and Therapeutics Bulletin. 2002;40(12):89-91. doi: 10.1136/dtb.2002.401289.

71. The challenges of shaping pharmacy practice in the future. Pharmaceutical Journal; 2002.

72. What it means for community pharmacy. Pharmaceutical Journal. 2002;268(7182):119.

73. Hospital achieves 100% usage of CPOE system. Healthcare benchmarks and quality improvement. 2002;9(11):54-6.

74. Changes in pharmacy discussed. Pharmaceutical Journal. 2002;269(7219):541.

75. Accrediting community pharmacies: A way of bringing in quality standards? Pharmaceutical Journal. 2002;269(7213):274-7.

76. Formal postgraduate courses for practising pharmacists. Pharmaceutical Journal. 2002;268(7199):729-36.

77. Drug misuse report excludes pharmacy. Pharmaceutical Journal. 2002;268(7189):350.

78. Osteoporosis scanning through community pharmacy. Pharmaceutical Journal. 2002;268(7191):419.

79. Error reporting to be piloted in community pharmacy setting. Pharmaceutical Journal. 2002;269(7227):800.

80. New ways of working in pharmacy. Pharmaceutical Journal; 2002.

81. NPA considers individual members. Pharmaceutical Journal. 2002;269(7205):5.

82. Concern over application of clinical governance in community practice. Pharmaceutical Journal. 2002;268(7190):415.

83. Medicines management services - Why are they so important? MeReC Bulletin. 2002;12(6):21-4.

84. Consumer Guide for Malaria Drug. FDA Consumer. 2003;37(5):7. PubMed PMID: 10824054.

85. Patient forums must give written notice of pharmacy inspections. Pharmaceutical Journal. 2003;271(7278):764.

86. What you should not miss at the community pharmacy conference. Pharmaceutical Journal; 2003.

87. Rising demands from new contract. Pharmaceutical Journal. 2003;271(7277):732.

88. Report examines changes in community pharmacy. Pharmaceutical Journal. 2003;271(7278):765.

89. Let's do it. Pharmaceutical Journal. 2003;270(7246):562.

90. Louisiana hospital reduces pneumonia pharmacy costs by 26%. Performance improvement advisor. 2003;7(7):101-3,93.

91. Gauteng Health calls for retrieval of unused medicines and drugs. South African Medical Journal. 2003;93(8):574.

92. London pharmacists secure training funds for supplementary prescribing. Pharmaceutical Journal. 2003;271(7268):399.

93. NPA fights pharmacy's corner in out-of-hours service planning. Pharmaceutical Journal. 2003;270(7248):639.

94. Patient safety: The next step for research in community pharmacy. Pharmaceutical Journal. 2003;270(7232):91.

95. A new health system for the 21st century - Crossing the quality chasm. Pharmaceutical Journal; 2003.

96. Medicines management services save money and improve patient care. Pharmaceutical Journal. 2003;270(7241):392.

97. Scanning medication barcodes improves accuracy at Lehigh Valley Hospital. Performance improvement advisor. 2003;7(10):132-4,29.

98. CHC Pharmacy: A developing partnership. Drug Benefit Trends. 2003;15(3):29.

99. Pharmaceutical care research - The next generation. Pharmaceutical Journal; 2003.

100. Carl Edelmann GmbH sales grew 14% in 2002. "Market leader in Europe with premium packaging". Deutscher Drucker Stuttgart. 2003;39(8):14.

101. Workforce pressures on the development of future hospital pharmacy services. Australian Journal of Pharmacy. 2003;84(1002):714.

102. £75,000 for research on pharmacy errors. Pharmaceutical Journal. 2003;270(7232):74.

103. Third agreement: Mid-term review. Australian Journal of Pharmacy. 2003;84(995):138-9.

104. Prescriptions from hospitals cause problems in the community. Pharmaceutical Journal. 2003;270(7244):505.

105. Research will look at community errors. Pharmaceutical Journal. 2003;270(7256):4.

106. Details of the proposed new pharmacy contract for Scotland are revealed. Pharmaceutical Journal.

2003;271(7261):165.

107. MPs say market must not control pharmacy services. Pharmaceutical Journal. 2003;270(7254):849.

108. Society to seek broader powers to regulate retail pharmacy premises. Pharmaceutical Journal. 2003;271(7262):217-20.

109. New Welsh quality control unit opened. Pharmaceutical Journal. 2003;270(7232):74.

110. WARNING TO SENIORS. Better Nutrition. 2004;66(6):32. PubMed PMID: 13114948.

111. Reducing medication errors requires a multifactorial approach. Drugs & Therapy Perspectives. 2004;20(8):22-6. doi: 10.2165/00042310-200420080-00008. PubMed PMID: 106598022. Language: English. Entry Date: 20050325. Revision Date: 20200624. Publication Type: Journal Article.

112. Ethinylestradiol + norelgestromin: new preparation. Transdermal contraception: no tangible progress. Prescrire international. 2004;13(72):123-6. PubMed PMID: CN-01743609.

113. Community pharmacy-based interventions can reduce risk behaviour and risk factors for coronary heart disease. Evidence-based Healthcare. 2004;8(1):21-3. doi: https://doi.org/10.1016/j.ehbc.2003.12.020.

114. President will urge every member to vote "yes" in Charter ballot. Pharmaceutical Journal. 2004;273(7308):95-7.

115. Clinical governance tool launched by society in Wales. Pharmaceutical Journal. 2004;273(7308):76.

116. Chief pharmacists: "State of the nation". Pharmaceutical Journal; 2004.

117. Are contractors ready for the contract? Pharmaceutical Journal. 2004;273(7325):710.

118. Standard operating procedures: Time to knuckle down and get writing. Pharmaceutical Journal. 2004;273(7319):460-1.

119. Limited benefit from pharmacists' interventions. Pharmaceutical Journal. 2004;273(7319):458.

120. Guide to community pharmacy services launched. Pharmaceutical Journal. 2004;272(7304):760.

121. Medicare law motivates hospitals to report quality data. American Journal of Health-System Pharmacy. 2004;61(19):1996+9-2000. doi: 10.1093/ajhp/61.19.1996.

122. Enhanced future cancer role for pharmacists. Pharmaceutical Journal. 2004;272(7304):758.

123. Pharmacists and veterinary surgeons: Cooperating on animal care. Veterinary Record. 2004;155(17):509-11.

124. ASHP, AMCP recommend criteria for Medicare formulary design, formulary decision-maker responsibilities. Formulary. 2004;39(11):559.

125. Clinical governance: How pharmacies in a local area can be compared. Pharmaceutical Journal; 2004.

126. Health minister impressed by Sheffield pharmacies. Pharmaceutical Journal. 2004;273(7307):42.

127. Technicians' ethics code has conflicting responsibilities. Pharmaceutical Journal. 2004;272(7300):634.

128. Teaching extemporaneous preparation is still important. Pharmaceutical Journal. 2004;272(7281):7.

129. Violence in pharmacies to be raised in talks with minister. Pharmaceutical Journal. 2004;272(7294):453.

130. Introduction. Studying the Organisation and Delivery of Health Services: A Reader. 2004:xiii-xv. doi: 10.4324/9780203337394-10.

131. In brief. Nature. 2005;435(7043):735. doi: 10.1038/435735a. PubMed PMID: 17265729.

132. Take the FDA Consumer QUIZ. FDA Consumer. 2005;39(4):38. PubMed PMID: 17513749.

133. Are you missing adverse drug events in the elderly? ED Nursing. 2005;8(8):88-9. PubMed PMID: 106374110. Language: English. Entry Date: 20060106. Revision Date: 20150818. Publication Type: Journal Article. Journal Subset: Nursing.

134. Stop dangerous practices for giving oral medications: using the wrong type of syringe can result in tragedy. ED Nursing. 2005;9(2):15-6. PubMed PMID: 106417037. Language: English. Entry Date: 20060331. Revision Date: 20150818. Publication Type: Journal Article. Journal Subset: Nursing.

135. Combining for lice effective. South African family practice. 2005;47(10):28-. PubMed PMID: CN-01752007.

136. Pharmacy project highlighted at NHS Live event. Pharmaceutical Journal. 2005;275(7381):766.

137. Many PCTs have no early diabetes detection plans. Pharmaceutical Journal. 2005;275(7381):767.

138. EHC purchase from pharmacies doubles in past year. Pharmaceutical Journal. 2005;275(7374):567.

139. Violence in primary care and the community should always be reported to PCTs. Pharmaceutical Journal. 2005;275(7374):568.

140. Minister backs careers campaign. Pharmaceutical Journal. 2005;275(7379):727-9.

141. Promotion of museum continues. Pharmaceutical Journal. 2005;275(7367):351.

142. New focus on long-term conditions for NHS Scotland. Pharmaceutical Journal. 2005;274(7352):667.

143. Up to £140,000 made available for research on the new contract. Pharmaceutical Journal. 2005;274(7350):627-8.

144. New oxygen contract is a risk to patients, says PSNC. Pharmaceutical Journal. 2005;274(7353):699.

145. Scottish Executive wants better use made of CHI data. Pharmaceutical Journal. 2005;275(7357):38.

146. Pharmacy project aims to reduce cancer risk. Pharmaceutical Journal. 2005;274(7338):224.

147. Erewash self-care study launches heart risk assessment through pharmacies. Pharmaceutical Journal. 2005;274(7337):200.

148. Pharmacy workload puts patients at risk. Pharmaceutical Journal. 2005;274(7337):200.

149. Preventing the accumulation of surplus controlled substances at long term care facilities. Final rule. Federal register. 2005;70(92):25462-6.

150. Pharmacy brings health care vision to community. Pharmaceutical Journal. 2005;275(7361):158.

151. Focus on core business. Pharmaceutical Journal. 2005;274(7344):408.

152. Competencies for advanced services announced. Pharmaceutical Journal. 2005;274(7332):41.

153. Intervention target: Diabetes support. Australian Journal of Pharmacy. 2005;86(1024):534-6.

154. Wary response to "new kind of health professional". Pharmaceutical Journal. 2005;275(7369):399.

155. Healthcare Commission calls for pharmacist input into prescribing. Pharmaceutical Journal. 2005;275(7359):103.

156. Error rates in community pharmacy leave room for improvement. Pharmaceutical Journal. 2005;274(7352):669.

157. Medicines and driving. Pharmaceutical Journal. 2005;275(7361):158.

158. Society wants fewer legal retrictions on pharmacists. Pharmaceutical Journal. 2005;274(7341):341.

159. Latebreakers. Drug Topics. 2005;149(16).

160. Radio-frequency identification: its potential in healthcare. Health devices. 2005;34(5):149-60.

161. 60 SECONDS. New Scientist. 2006;191(2562):5. doi: 10.1016/S0262-4079(06)60324-2. PubMed PMID: 21843558.

162. ADHD drug snag. New Scientist. 2006;191(2565):7. doi: 10.1016/S0262-4079(06)60130-9. PubMed PMID: 22115245.

163. ELDERLY AT RISK FROM OVERDOSE. Ecologist. 2006;36(9):13. PubMed PMID: 22697939.

164. green gazette. Mother Earth News. 2006;(217):21. PubMed PMID: 21559752.

165. Reforms on drug safety. Nature. 2006;443(7110):372. doi: 10.1038/443372a. PubMed PMID: 22543306.

166. ED nurses saved $100,000 and cut drug errors to almost zero: mistakes are 'practically eliminated' with cutting-edge interventions. ED Nursing. 2006;9(5):49-53. PubMed PMID: 106467384. Language: English. Entry Date: 20060707. Revision Date: 20150818. Publication Type: Journal Article.

167. EDs nurses are key to complying with new JCAHO medication goal: surveyors will look very closely at this in 2006. ED Nursing. 2006;9(6):61-4. PubMed PMID: 106311614. Language: English. Entry Date: 20060804. Revision Date: 20150711. Publication Type: Journal Article.

168. Non-medical prescribing. Drug & Therapeutics Bulletin. 2006;44(5):33-7. PubMed PMID: 106304101. Language: English. Entry Date: 20060714. Revision Date: 20191113. Publication Type: journal article. Journal Subset: Biomedical.

169. Strategies to reduce medication errors with reference to older adults. Nursing Standard. 2006;20(41):53-7. doi: 10.7748/ns2006.06.20.41.53.c6551. PubMed PMID: 106319228. Corporate Author: Joanna Briggs Institute. Language: English. Entry Date: 20060818. Revision Date: 20200518. Publication Type: Journal Article.

170. Prescriptions by non medical personnel. Assistenza infermieristica e ricerca. 2006;25(3):181-8. PubMed

PMID: CN-01750946.

171. Varenicline effective for smoking cessation. Journal of family practice. 2006;55(10):848-9. PubMed PMID: CN-01771287.

172. Prevention of chemotherapy- and radiotherapy-induced emesis: results of the 2004 Perugia International Antiemetic Consensus Conference. Annals of Oncology. 2006;17(1):20-8. doi: https://doi.org/10.1093/annonc/mdj078.

173. Not someone else's problem. Pharmaceutical Journal. 2006;277(7413):180.

174. Sound medication therapy management programs. Case Manager. 2006;17(4):47-50. doi: 10.1016/j.casemgr.2006.05.006.

175. Elderly people still given inappropriate drugs. Pharmaceutical Journal. 2006;276(7384):62.

176. Did Your Rx Get a Warning? Prevention. 2007;59(10):42. PubMed PMID: 26611620.

177. Flu drug fall. Nature. 2007;450(7168):335. doi: 10.1038/450335c. PubMed PMID: 27488798.

178. HEART HEALTH: ASK DR. ZIPES. Saturday Evening Post. 2007;279(2):32. PubMed PMID: 24200967.

179. In brief. Nature. 2007;447(7146):767. doi: 10.1038/447767a. PubMed PMID: 25384129.

180. Deaths from intravenous colchicine resulting from a compounding pharmacy error -- Oregon and Washington, 2007. MMWR: Morbidity & Mortality Weekly Report. 2007;56(40):1050-2. PubMed PMID: 106187130. Corporate Author: US Centers for Disease Control and Prevention (CDC). Language: English. Entry Date: 20071109. Revision Date: 20151015. Publication Type: Journal Article.

181. Outpatient surgery at risk for drug errors: report offers concrete steps you can take now. Same-Day Surgery. 2007;31(5):56-8. PubMed PMID: 105917793. Language: English. Entry Date: 20080104. Revision Date: 20150711. Publication Type: Journal Article. Journal Subset: Biomedical.

182. Pharmacist in ED slashes medication errors by 50%: turnaround accomplished in just six months. ED Management. 2007;19(3):29-30. PubMed PMID: 106104719. Language: English. Entry Date: 20070615. Revision Date: 20150711. Publication Type: Journal Article. Journal Subset: Nursing.

183. Study shows efficacy of bar-code usage: bar-coding can reduce errors, study says. Healthcare Benchmarks & Quality Improvement. 2007;14(1):7-8. PubMed PMID: 106180257. Language: English. Entry Date: 20071102. Revision Date: 20150711. Publication Type: Journal Article. Journal Subset: Health Services Administration.

184. The Joint Commission says the goal for medication reconciliation is unchanged. ED Management. 2007:1-3. PubMed PMID: 105931148. Language: English. Entry Date: 20080118. Revision Date: 20150711. Publication Type: Journal Article. Supplement Title: 2007 Aug Accreditation Update. Journal Subset: Nursing.

185. What surveyors want to see for review of med orders. ED Nursing. 2007;10(9):100-2. PubMed PMID: 106164056. Language: English. Entry Date: 20071005. Revision Date: 20150711. Publication Type: Journal Article. Journal Subset: Nursing.

186. What surveyors want to see for review of med orders: broader interpretation is now allowed. ED

Management. 2007;19(7):75-7. PubMed PMID: 106193004. Language: English. Entry Date: 20071116. Revision Date: 20150711. Publication Type: Journal Article. Journal Subset: Nursing.

187. The MEDMAN study: a randomized controlled trial of community pharmacy-led medicines management for patients with coronary heart disease. Family practice. 2007;24(2):189-200. doi: 10.1093/fampra/cml075. PubMed PMID: CN-00586725.

188. Expert Panel Report 3 (EPR-3): Guidelines for the Diagnosis and Management of Asthma-Summary Report 2007. The Journal of allergy and clinical immunology. 2007;120(5 Suppl):S94-138.

189. Chemotherapy medication errors with paediatric cancer drugs. Australian Journal of Pharmacy. 2007;88(1053):62.

190. Developments in mental health pharmacy practice. Pharmaceutical Journal; 2007.

191. Pharmacists will contribute to shifting care from the "middle ground" into community settings. Pharmaceutical Journal; 2007.

192. Control and supervision now separated. Pharmaceutical Journal; 2007.

193. Guidelines, editors, pharma and the biological paradigm shift. Mens Sana Monographs. 2007;5(1):27-145. doi: 10.4103/0973-1229.32176.

194. Monitoring quality standards in the provision of non-prescription medicines from Australian Community Pharmacies: results of a national programme (vol 16, pg 354, 2007). Quality & Safety in Health Care. 2007;16(6):461-. doi: 10.1136/qshc.2006.019463corr1. PubMed PMID: WOS:000251318100012.

195. National asthma education and prevention program - Expert panel report 3 (EPR-3): Guidelines for the diagnosis and management of asthma - Summary report 2007. Journal of Allergy and Clinical Immunology. 2007;120(5):S94-S138. doi: 10.1016/j.jaci.2007.09.029. PubMed PMID: WOS:000251247600002.

196. Too much haste in US drug approval? Nature. 2008;452(7187):516. doi: 10.1038/452516d. PubMed PMID: 31514353.

197. Clinical pharmacists in oncology practice. Journal of Oncology Practice. 2008;4(4):172-4. PubMed PMID: 105813035. Language: English. Entry Date: 20080912. Revision Date: 20150711. Publication Type: Journal Article.

198. Joint Commission warns of pediatric medication errors, urges action: most errors involve improper dose or quantity. Healthcare Risk Management. 2008;30(6):61-4. PubMed PMID: 105547102. Language: English. Entry Date: 20090417. Revision Date: 20150711. Publication Type: Journal Article. Journal Subset: Health Services Administration.

199. New recommendations for pediatric medication safety: EDs have greater chance of mistakes. ED Nursing. 2008;11(9):99-101. PubMed PMID: 105812733. Language: English. Entry Date: 20080912. Revision Date: 20150711. Publication Type: Journal Article.

200. Pediatric aspects of inpatient health information technology systems. Pediatrics. 2008;122(6):e1287-96. PubMed PMID: 105474777. Corporate Author: Council on Clinical Information Technology. Language: English. Entry Date: 20090515. Revision Date: 20150711. Publication Type: Journal Article.

201. Will a drug error harm the next child you treat? Study: it happens to 1 in 3: ED medication mistakes are surprisingly common. ED Nursing. 2008;11(9):97-9. PubMed PMID: 105812731. Language: English. Entry

Date: 20080912. Revision Date: 20150818. Publication Type: Journal Article. Journal Subset: Nursing.

202. Care management shows benefits. Disease management advisor. 2008;14(8):suppl 1-4, 1, following p. 6. PubMed PMID: CN-01714162.

203. Supervision of patients with hypertension via email improves their blood-pressure control. Nature clinical practice nephrology. 2008;4(10):523-. doi: 10.1038/ncpneph0903. PubMed PMID: CN-01773885.

204. OTC drug sales authorised in France. Prescrire International. 2008;17(98):261.

205. Can antenatal care be provided by community pharmacists? Pharmaceutical Journal; 2008.

206. Nova Scotia spearheads unique research into quality improvement and medication error management. Canadian Pharmacists Journal. 2008;141(4):212. doi: 10.3821/1913-701X(2008)141[212:NSSURI]2.0.CO;2.

207. Cause for celebration. Pharmaceutical Journal. 2008;281(7510):28.

208. News from the 36th European Symposium on Clinical Pharmacy; 25-27 October 2007; Istanbul. Drugs and Therapy Perspectives; 2008.

209. Medication therapy management in pharmacy practice: Core elements of an MTM service model (version 2.0). Journal of the American Pharmacists Association. 2008;48(3):341-53. doi: 10.1331/JAPhA.2008.08514.

210. Pain management model has safety advantages: developing inpatient pharmacy model is challenge. Healthcare Benchmarks & Quality Improvement. 2009;16(3):30-2. PubMed PMID: 105453105. Language: English. Entry Date: 20090529. Revision Date: 20150711. Publication Type: Journal Article. Journal Subset: Health Services Administration.

211. 20th Australasian Conference on Information Systems, ACIS 2009. ACIS 2009 Proceedings - 20th Australasian Conference on Information Systems; 2009.

212. AMCP guide to pharmaceutical payment methods, 2009 update. Journal of Managed Care Pharmacy. 2009;15(6 SUPPL. A):S1-S57.

213. AMCP Guide to Pharmaceutical Payment Methods, 2009 Update (Version 2.0). Journal of managed care pharmacy : JMCP. 2009;15(6 Suppl A):S3-57,quizS8.

214. Hard times ahead in hospital pharmacy. Pharmaceutical Journal. 2009;283(7560):51.

215. Medication Misadventures. Best Practices for Hospital & Health-System Pharmacy. 2010:172-207. PubMed PMID: 59619323. Language: English. Entry Date: 20110506. Revision Date: 20151001. Publication Type: Article.

216. MEDICATION SAFETY. Journal of Pharmacy Practice & Research. 2010;40(4):304-8. PubMed PMID: 57408645. Language: English. Entry Date: 20110301. Revision Date: 20110318. Publication Type: Article.

217. Cost-effectiveness of shared pharmaceutical care for older patients: RESPECT trial findings. British journal of general practice. 2010;60(570):e20-7. doi: 10.3399/bjgp09X482312. PubMed PMID: CN-00733058.

218. Effectiveness of shared pharmaceutical care for older patients: RESPECT trial findings. British journal of general practice. 2010;60(570):e10-9. doi: 10.3399/bjgp09X473295. PubMed PMID: CN-00733020.

219. Management of allergic rhinitis: advances in intranasal antihistamine therapy. Pharmacy times. 2010;76(3). PubMed PMID: CN-01728974.

220. Pharmacological treatment of frailty in the elderly. Australian journal of pharmacy. 2010;91(1076):56-8. PubMed PMID: CN-01736809.

221. Skill mix changes improves care. Australian Journal of Pharmacy. 2010;91(1077):71.

222. Effect of 6 Managed Care Pharmacy Tools: A Review of the Literature. Journal of Managed Care Pharmacy. 2010;16(6):S3-S20. PubMed PMID: WOS:000280456200008.

223. Highway To Health. Prevention. 2011;63(12):78. PubMed PMID: 67097976.

224. National Birth Defects Prevention Month. MMWR: Morbidity & Mortality Weekly Report. 2011;59(51/52):1687. PubMed PMID: 63230010.

225. Better drug-risk warnings needed. Consumer Reports on Health. 2011;23(9):6-. PubMed PMID: 65036205. Language: English. Entry Date: 20110907. Revision Date: 20110909. Publication Type: Article.

226. Document this about inpatient medications. ED Nursing. 2011;14(6):63-. PubMed PMID: 59699862. Language: English. Entry Date: 20110407. Revision Date: 20110407. Publication Type: Article.

227. Labelling remains a concern. Pharmacy News. 2011:15-. PubMed PMID: 58578903. Language: English. Entry Date: 20110304. Revision Date: 20110307. Publication Type: Article.

228. Medication errors in care homes. Nursing Times. 2011;107(24):14-5. PubMed PMID: 108232663. Language: English. Entry Date: 20110729. Revision Date: 20150819. Publication Type: Journal Article.

229. Medication Misadventures. Best Practices for Hospital & Health-System Pharmacy. 2011:193-229. PubMed PMID: 70244455. Language: English. Entry Date: 20120201. Revision Date: 20190617. Publication Type: Article.

230. Order scanning systems (and fax machines) may pull multiple pages through...Reprinted with permission from ISMP Medication Safety Alert! Nurse Advise-ERR (ISSN 1550-6304) November 2010 Volume 8 Issue 11 ©2010 Institute for Safe Medication Practices (ISMP). Alberta RN. 2011;67(1):24-5. PubMed PMID: 59267618. Language: English. Entry Date: 20110411. Revision Date: 20110411. Publication Type: Article.

231. Pharmacy students top of class for finding drug errors. Pharmacy News. 2011:7-. PubMed PMID: 60807467. Language: English. Entry Date: 20110527. Revision Date: 20110602. Publication Type: Article.

232. 'Quick kits' speed care of emergent conditions. ED Nursing. 2011;14(3):27-8. PubMed PMID: 104972177. Language: English. Entry Date: 20110118. Revision Date: 20150711. Publication Type: Journal Article. Journal Subset: Nursing.

233. Abstracts of the Neonatal and Paediatric Pharmacists Group 16th Annual Conference, NPPG 2010. Archives of disease in childhood. 2011;96(4). PubMed PMID: CN-01743623.

234. Late-Breaking Clinical Trial and Clinical Science: special Reports Abstracts from the American Heart Association's Scientific Sessions 2011. Circulation. 2011;124(21). PubMed PMID: CN-01007609.

235. Oral Session 1 - Managing Addiction in Community Pharmacy - Chairs: christine Bond and Richard Holland. International journal of pharmacy practice. 2011;19:4-6. doi: 10.1111/j.2042-7174.2011.00098.x.

PubMed PMID: CN-02128544.

236. APhA2011 abstracts of contributed papers. Journal of the American Pharmacists Association. 2011;51(2):213-308. doi: https://doi.org/10.1331/JAPhA.2011.11517.

237. Effective Pharmacist-Led Medication Therapy Management (MTM) Program for Patients of a Safety Net Program. Research in Social and Administrative Pharmacy. 2011;7(1):e12-e3. doi: https://doi.org/10.1016/j.sapharm.2010.09.031.

238. Management of acute infective conjunctivitis. Drug and Therapeutics Bulletin. 2011;49(7):78-81. doi: 10.1136/dtb.2011.02.0043.

239. Advising or referring patients on the basis of an interview: Diagnostic competence. Prescrire International. 2011;20(122):298-300.

240. A big bouncy baby business. Australian Journal of Pharmacy. 2011;92(1091):58-61.

241. Pharmacist-provided immunization compensation and recognition: White paper summarizing APhA/AMCP stakeholder meeting. Journal of the American Pharmacists Association. 2011;51(6):704-12. doi: 10.1331/JAPhA.2011.11544.

242. Authorized generics: An interim report. Pharmaceutical Industry: Innovation and Developments2011. p. 15-47.

243. MEDICATION ERRORS: Keep an eye out for this wart remover. Nursing. 2012;42(3):16-. PubMed PMID: 74217412. Language: English. Entry Date: 20120417. Revision Date: 20120610. Publication Type: Article.

244. Medication Misadventures. Bethesda, Maryland: American Society of Health System Pharmacists; 2012. p. 198-237.

245. Medication Safety and Reliability. FDA warns of Durezol/Durasal name confusion. Formulary. 2012;47(2):80-. PubMed PMID: 72133944. Language: English. Entry Date: 20120521. Revision Date: 20120611. Publication Type: Article.

246. MEDICATION SAFETY. Journal of Pharmacy Practice & Research. 2012;42(4):311-5. PubMed PMID: 84761430. Language: English. Entry Date: 20130122. Revision Date: 20210224. Publication Type: Article.

247. Methadone dispensed for methylphenidate. Nursing. 2012;42(11):12-. PubMed PMID: 83129803. Language: English. Entry Date: 20121106. Revision Date: 20121108. Publication Type: Article.

248. News Briefs. American Journal of Health-System Pharmacy. 2012;69(6):454-. PubMed PMID: 74198298. Language: English. Entry Date: 20120418. Revision Date: 20120608. Publication Type: Article.

249. Oral Session 6-Safe use of medicines in hospital and community settings. International Journal of Pharmacy Practice. 2012;20:25-30. doi: 10.1111/j.2042-7174.2012.00234_6.x. PubMed PMID: 79611829. Language: English. Entry Date: 20120913. Revision Date: 20131002. Publication Type: Article.

250. Late-Breaking Clinical Trials from the American Heart Association Scientific Sessions 2011. European heart journal. 2012;33(1). PubMed PMID: CN-01035082.

251. RSAP is pleased to announce the winning authors for its annual Best Paper Award among articles published in 2011, Todd Boyle, Thomas Mahaffey, Neil MacKinnon, Heidi Deal, Lars Hallstrom, and Holly

Morgan, for their paper titled Determinants of Medication Incident Reporting, Recovery, and Learning in Community Pharmacies: A Conceptual Model appearing in Volume 7, Issue 1 of the journal. The following is a description of the award. Research in Social and Administrative Pharmacy. 2012;8(2):101. doi: https://doi.org/10.1016/j.sapharm.2011.12.006.

252. Rural telepharmacies do not have higher error rates. Pharmacy Times. 2012;79(12).

253. No recycling. Drug and Therapeutics Bulletin. 2012;50(5):49. doi: 10.1136/dtb.2012.05.0100.

254. Seven days: 29 November-5 December 2013. Nature. 2013;504(7478):12. doi: 10.1038/504012a. PubMed PMID: 92707147.

255. Washington County Passes Drug Take-Back Program. Solid Waste Report. 2013;44(16):7. PubMed PMID: 90518247.

256. ASHP Statement on the Role of the Medication Safety Leader. American Journal of Health-System Pharmacy. 2013;70(5):448-52. doi: 10.2146/sp120010. PubMed PMID: 104236715. Language: English. Entry Date: 20130221. Revision Date: 20200708. Publication Type: Journal Article.

257. Avoid successful suits alleging prescribing errors. Same-Day Surgery. 2013;37(10):112-3. PubMed PMID: 90249113. Language: English. Entry Date: 20130920. Revision Date: 20180614. Publication Type: Article. Journal Subset: Biomedical.

258. Chain pharmacy "endangers patients". Pharmacy News. 2013:11-. PubMed PMID: 89936682. Language: English. Entry Date: 20130903. Revision Date: 20131002. Publication Type: Article.

259. Chapter 7: Participating in Interdisciplinary Care Planning. Journal of Pharmacy Practice & Research. 2013;43:S29-S. doi: 10.1002/j.2055-2335.2013.tb00901.x. PubMed PMID: 150678757. Language: English. Entry Date: 20210614. Revision Date: 20210614. Publication Type: Article.

260. E-prescribing is becoming the norm. Nursing. 2013;43(12):25-. doi: 10.1097/01.NURSE.0000437480.30251.a8. PubMed PMID: 92871601. Language: English. Entry Date: 20131213. Revision Date: 20180614. Publication Type: Article.

261. Glossary. Journal of Pharmacy Practice & Research. 2013;43:S68-S9. doi: 10.1002/j.2055-2335.2013.tb00910.x. PubMed PMID: 150678766. Language: English. Entry Date: 20210614. Revision Date: 20210614. Publication Type: Article.

262. LEGAL ADVISOR. Questioning the doctor's orders. Haymarket Media, Inc.; 2013. p. 61-2.

263. Lunch break: pro and con. Australian Doctor Group; 2013. p. 18-.

264. Managing your blood thinners. Harvard Heart Letter. 2013;23(9):6-. PubMed PMID: 88822346. Language: English. Entry Date: 20130703. Revision Date: 20130929. Publication Type: Article.

265. Medication errors and how to avoid them. Harvard Health Letter. 2013;39(2):6-. PubMed PMID: 95373681. Language: English. Entry Date: 20140403. Revision Date: 20140407. Publication Type: Article.

266. OF SPECIAL INTEREST. American Journal of Health-System Pharmacy. 2013;70(24):2159-. PubMed PMID: 92998500. Language: English. Entry Date: 20131219. Revision Date: 20191111. Publication Type: Article.

267. Pharmacists and skin problems: how we can help. Dermatological Nursing. 2013;12(2):56-7. PubMed

PMID: 88306677. Language: English. Entry Date: 20130628. Revision Date: 20191111. Publication Type: Article.

268. Wrong patient, wrong script. Australian Doctor Group; 2013. p. 29-.

269. Effectiveness of a group-based intervention to improve medication beliefs and adherence in non-adherent patients with rheumatoid arthritis: a randomized controlled trial. International journal of clinical pharmacy. 2013;35(6):1256. doi: 10.1007/s11096-013-9886-5. PubMed PMID: CN-02216551.

270. Acetaminophen safety: Be cautious but not afraid. Harvard Men's Health Watch. 2014;18(6):3. PubMed PMID: 94280323.

271. Hospital brings discharge meds to the bedside. Hospital Case Management. 2014;22(2):17-8. PubMed PMID: 104007883. Language: English. Entry Date: 20140123. Revision Date: 20150710. Publication Type: Journal Article. Journal Subset: Nursing.

272. Surgeon looks at dye label, 'sees' what he expects. Healthcare Risk Management. 2014;36(11):111-2. PubMed PMID: 102950345. Language: English. Entry Date: 20150810. Revision Date: 20170918. Publication Type: Article.

273. Think more about how technology affects quality. Detroit, Michigan: Crain Communications Inc. (MI); 2014. p. 32-.

274. Medicare star ratings: Stakeholder proceedings on community pharmacy and managed care partnerships in quality. Journal of the American Pharmacists Association. 2014;54(3):228-40. doi: 10.1331/JAPhA.2014.13180.

275. The consequences of nonprescription medication sales in Saudi Arabia's community pharmacies: Regulations without implementation. Therapeutic Advances in Drug Safety. 2014;5(4):173-4. doi: 10.1177/2042098614526770.

276. Disposal of controlled substances. Final rule. Federal register. 2014;79(174):53519-70.

277. ACA: Your ADVOCATE. W. Virginia OKs Lawsuits for Negligently Prescribed Pain Meds. ACA News (American Chiropractic Association). 2015;11(8):8-. PubMed PMID: 110454427. Language: English. Entry Date: 20160107. Revision Date: 20220624. Publication Type: Article.

278. Medication Safety. Journal of Pharmacy Practice & Research. 2015;45(4):450-8. doi: 10.1002/jppr.1170. PubMed PMID: 111999682. Language: English. Entry Date: 20170921. Revision Date: 20180828. Publication Type: Article.

279. Effect of a ward-based pharmacy team on preventable adverse drug events in surgical patients (SUREPILL study). British journal of surgery. 2015;102(10):1204-12. doi: 10.1002/bjs.9876. PubMed PMID: CN-01108877.

280. Feasibility of a Text Messaging Intervention to Promote Self-Management for Patients Prescribed Oral Anticancer Agents. Oncology nursing forum 42 (6) (pp 647-657), 2015 Date of publication: 01 nov 2015. 2015. doi: 10.1188/15.ONF.647-657. PubMed PMID: CN-01194310.

281. Pioglitazone in early Parkinson's disease: a phase 2, multicentre, double-blind, randomised trial. The lancet Neurology. 2015;14(8):795-803. doi: 10.1016/S1474-4422(15)00144-1. PubMed PMID: CN-01109006.

282. Changes are required to help community pharmacists become more involved in reporting medication safety issues. Drugs and Therapy Perspectives. 2015;31(5):165-6. doi: 10.1007/s40267-015-0186-0.

283. Studies in Health Technology and Informatics. Studies in Health Technology and Informatics. 2015;208.

284. Don't fall for hip fractures. Harvard Men's Health Watch. 2016;21(5):6. PubMed PMID: 119486679.

285. Put your heart in the right place. Harvard Men's Health Watch. 2016;20(9):5. PubMed PMID: 114087239.

286. Should you keep taking that heartburn medication? Harvard Health Letter. 2016;41(7):6. PubMed PMID: 114741838.

287. Avoid Medication Mix-Ups. Consumer Reports on Health. 2016;28(8):1-5. PubMed PMID: 116191641. Language: English. Entry Date: 20160617. Revision Date: 20160622. Publication Type: Article.

288. Impact of an emergency department pharmacist on prescribing errors is an Australian hospital. Journal of Pharmacy Practice & Research. 2016;46:25-7. doi: 10.1002/jppr.1275. PubMed PMID: 119575312. Language: English. Entry Date: 20161129. Revision Date: 20180808. Publication Type: Article.

289. 44th ESCP International Symposium on Clinical Pharmacy Medicines Information: making Better Decisions. International journal of clinical pharmacy. 2016;38(6). PubMed PMID: CN-01160006.

290. Evaluation of the influence of pharmacists and GPs on patient perceptions of long-term topical corticosteroid use. Journal of dermatological treatment (pp 1-7), 2016 Date of publication: 05 aug 2016. 2016. doi: 10.1080/09546634.2016.1213353. PubMed PMID: CN-01196887.

291. Study of the effect of an oral formulation of fig and olive on rheumatoid arthritis (RA) remission indicators: a randomized clinical trial. Iranian journal of pharmaceutical research. 2016;15(3):537-45. PubMed PMID: CN-01923792.

292. Corrigendum to: Uptake of the MedsCheck annual medication review service in Ontario community pharmacies between 2007 and 2013 (Can Pharm J (Ott), (2016), 149, (293-302), 10.1177/1715163516662670). Canadian Pharmacists Journal. 2016;149(6):319. doi: 10.1177/1715163516675591.

293. All Africa congress on pharmacology and pharmacy 2016 - Evidence in action. SA Pharmaceutical Journal; 2016.

294. Diabetes program focuses on the basics. Hospital case management : the monthly update on hospital-based care planning and critical paths. 2016;24(2):23-5.

295. Pharmacy Practice Research Abstracts: Canadian Pharmacists Conference 2016. Canadian Pharmacists Journal. 2016;149(4):S1-S46. doi: 10.1177/1715163516651630. PubMed PMID: WOS:000381005900001.

296. AIM TO HALVE MEDICATION ERRORS IN NEXT FIVE YEARS. Australian Nursing & Midwifery Journal. 2017;25(5):10-. PubMed PMID: 125905079. Language: English. Entry Date: 20171102. Revision Date: 20171102. Publication Type: Article.

297. Case study: Urinary incontinence -- the role of a pharmacist in an aged care Residential Medication Management Review. Cambridge Publishing; 2017. p. 30-4.

298. Development of a health-system palliative care clinical pharmacist. American Journal of Health-System

Pharmacy. 2017;74(1):e6-e8. doi: 10.2146/ajhp160055. PubMed PMID: 120561755. Language: English. Entry Date: 20170109. Revision Date: 20190712. Publication Type: Article.

299. Improved Nurse and Pharmacist Communications Optimizes the Medication Use Process. ONS Voice. 2017;32(6):32-. PubMed PMID: 124566345. Language: English. Entry Date: 20170824. Revision Date: 20200423. Publication Type: Article.

300. News briefs. American Journal of Health-System Pharmacy. 2017;74(23):1935-. PubMed PMID: 126511559. Language: English. Entry Date: 20171205. Revision Date: 20190603. Publication Type: Article.

301. Educating medical residents through podcasts developed by PharmD students. Currents in pharmacy teaching and learning. 2017;9(4):683-8. doi: 10.1016/j.cptl.2017.03.003. PubMed PMID: CN-01471286.

302. Effect of pharmaceutical care on clinical outcomes of outpatients with type 2 diabetes mellitus. Patient preference and adherence. 2017;11(pp 897-903). doi: 10.2147/PPA.S92533. PubMed PMID: CN-01411845.

303. The prophylactic antibiotic regimens in tumor surgery (PARITY) multicenter randomized controlled trial: international expansion of the collaborative network Michelle Ghert for. Journal of orthopaedic research. 2017;35. PubMed PMID: CN-01759860.

304. Randomized controlled trial of different aspirin regimens for reduction of niacin-induced flushing. American journal of health-system pharmacy. 2017;74(12):898-903. doi: 10.2146/ajhp160219. PubMed PMID: CN-01411745.

305. Erratum to: Revisiting the internal consistency and factorial validity of the 8-item Morisky Medication Adherence Scale (SAGE Open Medicine, (2016), 4, (1-17), 10.1177/2050312116674850). SAGE Open Medicine. 2017;5. doi: 10.1177/2050312117723969.

306. Corrections: Global antibiotic consumption 2000 to 2010: an analysis of national pharmaceutical sales data (The Lancet (2014) 14(8) (742–750)(S1473309914707807)(10.1016/S1473-3099(14)70780-7)). The Lancet Infectious Diseases. 2017;17(9):897. doi: 10.1016/S1473-3099(17)30241-4.

307. Retraction statement: Psychotropic drug usage characteristics amongst people with severe and profound disability undergoing home medicines review (Journal of Pharmacy Practice and Research, (2016) 46, 4, (312-315)). Journal of Pharmacy Practice and Research. 2017;47(5):411. doi: 10.1002/jppr.1220.

308. Corrigendum for Guenette L, Breton MC, Guillaumie L, Lauzier S, Gregoire JP, Moisan J. Psychosocial factors associated with adherence to non-insulin antidiabetes treatments. (Journal of Diabetes and its Complications (2016) 30(2) (335–342) (S1056872715004353) (10.1016/j.jdiacomp.2015.10.016)). Journal of Diabetes and its Complications. 2017;31(9):1475. doi: 10.1016/j.jdiacomp.2017.07.004.

309. Corrigendum to: Health behaviors and their correlates among participants in the Continuing to Confront COPD International Patient Survey (Int J Chron Obstruct Pulmon Dis, (2016) 11, (881-890), 10.2147/COPD.S102280). International Journal of COPD. 2017;12:859. doi: 10.2147/COPD.S133394.

310. Switzerland: Community pharmacists providing consultations for common medical conditions. Prescrire International. 2017;26(179):54-5.

311. STRATEGIES. Modern Healthcare. 2018;48(19):0030-. PubMed PMID: 129542019. Language: English. Entry Date: 20180514. Revision Date: 20191111. Publication Type: Article.

312. Abstracts 6th working symposium of the Pharmaceutical Care Network Europe, PCNE 2018. International journal of clinical pharmacy. 2018;40(2). PubMed PMID: CN-01608823.

313. Abstracts Presented at the 14th Annual Meeting for the Hematology/Oncology Pharmacy Association. Journal of oncology pharmacy practice. 2018;24(2). PubMed PMID: CN-01573442.

314. Pharmacy Practice Research Abstracts: canadian Pharmacists Conference 2018. Canadian pharmacists journal. 2018;151(5). PubMed PMID: CN-01653539.

315. Correction: Qualitative meta-synthesis of barriers and facilitators that influence the implementation of community pharmacy services: Perspectives of patients, nurses and general medical practitioners (BMJ Open (2017) 7 (e015471) DOI: 10.1136/bmjopen-2016-015471). BMJ Open. 2018;8(3). doi: 10.1136/bmjopen-2016-015471corr1.

316. ACOG COMMITTEE OPINION: Influenza Vaccination During Pregnancy. Obstetrics and Gynecology. 2018;131(4):E109-E14. doi: 10.1097/AOG.0000000000002588.

317. Community pharmacy and support for patients who take oral anticancer medication. Clinical Pharmacist. 2018;10(4). doi: 10.1211/CP.2018.20204570.

318. ACOG Committee Opinion No. 732 Summary: Influenza Vaccination During Pregnancy. Obstetrics and gynecology. 2018;131(4):752-3. doi: 10.1097/AOG.0000000000002586.

319. Erratum: Utility of the Morisky Medication Adherence Scale in gout: a prospective study (Psychological Science, (2016) 10, (2249-2457), 10.2147/PPA.S119719). Patient Preference and Adherence. 2018;12:527. doi: 10.2147/PPA.S130333.

320. Physicians' Preferences for Communication of Pharmacist-Provided Medication Therapy Management in Community Pharmacy. Pharmaceutical Care Espana. 2018;20(3):242-4. PubMed PMID: WOS:000435612800009.

321. An Evaluation of the Implementation of Pharmacist-Prescribed Hormonal Contraceptives in California. Obstetrical & Gynecological Survey. 2018;73(8):467-8. doi: 10.1097/01.ogx.0000541319.82632.52. PubMed PMID: WOS:000443243800017.

322. Sexual Harassment in the Work Place: Its Impact on Gynecologic Oncology and Women's Health. Obstetrical & Gynecological Survey. 2018;73(7):404-5. doi: 10.1097/ogx.0000000000000572. PubMed PMID: WOS:000440329200014.

323. Pilot Pharmaceutical Take-Back Program. New York State Conservationist. 2019;73(5):28. PubMed PMID: 136192492.

324. An Interdisciplinary Academic Detailing Approach to Decrease Inappropriate Medication Prescribing by Physician Residents for Older Veterans Treated in the Emergency Department. Journal of Pharmacy Practice. 2019;32(2):167-74. doi: 10.1177/0897190017747424. PubMed PMID: 136131412. Language: English. Entry Date: 20190507. Revision Date: 20190712. Publication Type: Article.

325. Executive summary of the 2019 ASHP Commission on Goals: Impact of artificial intelligence on healthcare and pharmacy practice. American Journal of Health-System Pharmacy. 2019;76(24):2087-92. doi: 10.1093/ajhp/zxz205. PubMed PMID: 140089879. Language: English. Entry Date: 20191207. Revision Date: 20201214. Publication Type: Article. Journal Subset: Biomedical.

326. Home health service gets assist from pharmacists. American Journal of Health-System Pharmacy. 2019;76(11):763-70. doi: 10.1093/ajhp/zxz050. PubMed PMID: 136525565. Language: English. Entry Date: 20190522. Revision Date: 20200601. Publication Type: Article. Journal Subset: Biomedical.

327. Professional policies approved by the 2019 ASHP March virtual House of Delegates. American Journal of Health-System Pharmacy. 2019;76(15):1165-. doi: 10.1093/ajhp/zxz103. PubMed PMID: 137628529. Language: English. Entry Date: 20190724. Revision Date: 20200731. Publication Type: Article. Journal Subset: Biomedical.

328. The Impact of Pharmaceutical Side Effects on Audiological and Vestibular Measurements. Seminars in Hearing. 2019;40(2):97-103. doi: 10.1055/s-0039-1684039. PubMed PMID: 136115617. Language: English. Entry Date: 20190430. Revision Date: 20190521. Publication Type: Article. Journal Subset: Allied Health.

329. 62nd NAF Annual Ataxia Conference. Contraception. 2019;99(5):265-320. doi: 10.1016/j.contraception.2019.02.008. PubMed PMID: CN-01936318.

330. Heart Failure Nurse Navigator Program Interventions Based on LACE Scores Reduces Inpatient Heart Failure Readmission Rates. Heart & Lung. 2019;48(5):470-1. doi: https://doi.org/10.1016/j.hrtlng.2019.08.007.

331. Correction: Comparisons of immunization records between a community pharmacy, a regional registry, and a health system (Journal of the American Pharmacists Association (2019)59(1)(30–34), (S1544319118304072), (10.1016/j.japh.2018.09.007)). Journal of the American Pharmacists Association. 2019;59(3):e1. doi: 10.1016/j.japh.2019.03.001.

332. Reducing opioid-related harms: practice in Australian hospitals in 2018. Journal of Pharmacy Practice and Research. 2019;49(1):69-77. doi: 10.1002/jppr.1538.

333. Erratum: Telemonitoring-based feedback improves adherence to non-Vitamin K antagonist oral anticoagulants intake in patients with atrial fibrillation (European Heart Journal (2018) 39 (1394-1403) DOI: 10.1093/eurheartj/ehx762). European Heart Journal. 2019;40(5):455. doi: 10.1093/eurheartj/ehy628.

334. Abstracts of 48th ESCP symposium on clinical pharmacy 23-25 October 2019, Ljubljana (Slovenia) : The digital revolution supporting clinical pharmacy through e-health, digital support systems, big data, and more. International Journal of Clinical Pharmacy. 2020;42(1):217-92. doi: 10.1007/s11096-019-00945-w. PubMed PMID: 142763551. Language: English. Entry Date: In Process. Revision Date: 20210201. Publication Type: journal article. Journal Subset: Biomedical.

335. ASHP Statement on the Role of the Medication Safety Leader. American Journal of Health-System Pharmacy. 2020;77(4):308-12. doi: 10.1093/ajhp/zxz292. PubMed PMID: 141628065. Language: English. Entry Date: 20200212. Revision Date: 20210215. Publication Type: Article.

336. Professional policies approved by the 2020 ASHP March virtual House of Delegates. American Journal of Health-System Pharmacy. 2020;77(13):1078-80. doi: 10.1093/ajhp/zxaa094. PubMed PMID: 144225338. Language: English. Entry Date: 20200627. Revision Date: 20210701. Publication Type: Article. Journal Subset: Biomedical.

337. Corrigendum to: children with poorly controlled asthma: randomized controlled trial of a home-based environmental control intervention (Pediatric Pulmonology, (2019), 54, 3, (245-256), 10.1002/ppul.24239). Pediatric pulmonology. 2020;55(5):1293-. doi: 10.1002/ppul.24731. PubMed PMID: CN-02146716.

338. CORRIGENDUM to:Increasing cardiovascular medication adherence: A medical research council complex mhealth intervention mixed-methods feasibility study to inform global practice (Journal of Advanced Nursing, (2020), 67, 10, (2670–2684), 10.1111/jan.14465). Journal of Advanced Nursing. 2020;76(12):3669. doi: 10.1111/jan.14595.

339. ASHP principles for COVID-19 vaccine distribution, allocation, and mass immunization. American

Journal of Health-System Pharmacy. 2020;77(24):2112-3. doi: 10.1093/ajhp/zxaa311.

340. What should primary care look like after the COVID-19 pandemic? Australian Journal of Primary Health. 2020;26(3):207-11. doi: 10.1071/PY20095.

341. Corrigendum to Examination of Barriers to Medication Adherence, Asthma Management, and Control Among Community Pharmacy Patients With Asthma (Journal of Pharmacy Practice, (2019), 10.1177/0897190019840117). Journal of Pharmacy Practice. 2020. doi: 10.1177/0897190020902889.

342. 14th International Conference on Algorithmic Aspects in Information and Management, AAIM 2020. Lecture Notes in Computer Science (including subseries Lecture Notes in Artificial Intelligence and Lecture Notes in Bioinformatics). 2020;12290 LNCS.

343. 2nd International Conference on Computer, Science, Engineering, and Technology, ICComSET 2019 - Health, Medical, Pharmacy and Technology. Journal of Physics: Conference Series; 2020.

344. Erratum: Community Pharmacist's Knowledge, Attitude, and Practices towards Vitamin Supplements in Al-Khobar Region, Saudi Arabia: A Descriptive Cross-Sectional Study (J Pharm Bioall Sci (2019)11 (333-40) DOI: 10.4103/jpbs.JPBS_99_19). Journal of Pharmacy and Bioallied Sciences. 2020;12(1):82. doi: 10.4103/0975-7406.277206.

345. corrigendum: Combined effect of coffee consumption and cigarette smoking on serum levels of vitamin b12, folic acid, and lipid profile in young male: A cross-sectional study (Int J Gen Med. (2019) 12, (421–432), 10.2147/IJGM.S213737). International Journal of General Medicine. 2020;13:27. doi: 10.2147/IJGM.S241320.

346. Feedback. New Scientist. 2021;249(3324):56. doi: 10.1016/s0262-4079(21)00391-2. PubMed PMID: 149093368.

347. Effects of pharmacist prescribing on patient outcomes in the hospital setting. Nursing & Health Sciences. 2021;23(4):978-81. doi: 10.1111/nhs.12846. PubMed PMID: 153893908. Language: English. Entry Date: 20211209. Revision Date: 20221201. Publication Type: Article.

348. Hospital Successfully Addresses Medication List Errors in ED. Healthcare Risk Management. 2021;43(11):1-2. PubMed PMID: 153372103. Language: English. Entry Date: 20211108. Revision Date: 20211108. Publication Type: Article. Journal Subset: Health Services Administration.

349. Professional Policies Approved by the 2020 ASHP November Virtual House of Delegates. American Journal of Health-System Pharmacy. 2021;78(7):646-7. doi: 10.1093/ajhp/zxab021. PubMed PMID: 149401395. Language: English. Entry Date: 20210326. Revision Date: 20220401. Publication Type: Article. Journal Subset: Biomedical.

350. Standards of practice for clinical pharmacy services – Chapter 16: My Health Record. Journal of Pharmacy Practice & Research. 2021;51(6):536-51. doi: 10.1002/jppr.1774. PubMed PMID: 154219260. Language: English. Entry Date: 20220103. Revision Date: 20221201. Publication Type: Article.

351. Emerging evidence in the dietary management of eczema with probiotics. Pharmacy times. 2021;89(5). PubMed PMID: CN-02325267.

352. Erratum: effect of a multifaceted clinical pharmacist intervention on medication safety after hospitalization in persons prescribed high-risk medications: a randomized clinical trial (JAMA Intern Med. (2021) 181: 5 (610-618) DOI: 10.1001/jamainternmed.2020.9285). JAMA internal medicine. 2021;181(5):727-. doi: 10.1001/jamainternmed.2021.2050. PubMed PMID: CN-02298459.

353. Help us fight joint pain in community pharmacy - become a 'PJ joint pain champion'. Pharmaceutical Journal. 2021;307(7953). doi: 10.1211/PJ.2021.1.107804.

354. COVID-19 antivirals to be tested in high-risk patients in the community. Pharmaceutical Journal. 2021;307(7954). doi: 10.1211/PJ.2021.1.112531.

355. Corrigendum to Examination of Barriers to Medication Adherence, Asthma Management, and Control Among Community Pharmacy Patients With Asthma. Journal of pharmacy practice. 2021;34(5):824-7. doi: 10.1177/0897190020902889.

356. Erratum: The evolving role of community pharmacists during COVID-19 in the UAE; Assessing preparedness and knowledge (International Journal of Pharmacy Practice (2020) DOI: 10.1093/ijpp/riaa003). International Journal of Pharmacy Practice. 2021;29(3):296. doi: 10.1093/ijpp/riab011.

357. NCPDP recommendations for standardizing dosing in metric units (mL) on prescription container labels of oral liquid medications, version 2.0. American Journal of Health-System Pharmacy. 2021;78(7):578-605. doi: 10.1093/ajhp/zxab023.

358. Erratum: (Anticancer Research). Anticancer Research. 2021;41(5):2733-5. doi: 10.21873/anticanres.41.5.2733.

359. Oral cancer drugs: Community pharmacists in France provide billable, structured care. Prescrire International. 2021;30(227):162-4.

360. Pharmacy teams have done brilliantly on COVID-19 vaccinations. Pharmaceutical Journal. 2021;306(7945). doi: 10.1211/PJ.2021.20208743.

361. Virtual Summit on Safe, Effective, and Accessible High-Quality Medicines as a Matter of National Security. American Journal of Health-System Pharmacy. 2021;78(6):511-20. doi: 10.1093/ajhp/zxaa392.

362. Effectiveness of a medication adherence management intervention in a community pharmacy setting: a cluster randomised controlled trial. Pharmaceutical Care Espana. 2021;23(2):196-200. PubMed PMID: WOS:000640215100009.

363. Challenges and opportunities relating to medication safety: learning from coroners' cases. Journal of Pharmacy Practice & Research. 2022;52(4):266-7. doi: 10.1002/jppr.1826. PubMed PMID: 158809793. Language: English. Entry Date: 20220909. Revision Date: 20220909. Publication Type: Editorial.

364. Corrigendum to: abstracts (CPT: pharmacometrics & Systems Pharmacology, (2022), 11, S1, 10.1002/psp4.12772). CPT: pharmacometrics and systems pharmacology. 2022. doi: 10.1002/psp4.12845. PubMed PMID: CN-02464744.

365. Efficacy and safety of two neutralising monoclonal antibody therapies, sotrovimab and BRII-196 plus BRII-198, for adults hospitalised with COVID-19 (TICO): a randomised controlled trial. The lancet Infectious diseases. 2022;22(5):622-35. doi: 10.1016/S1473-3099(21)00751-9. PubMed PMID: CN-02360420.

366. Errata to: CHEST 2021;160(6): 2247-2259 and CHEST 2021;160(6): e545-e608 (Chest (2021) 160(6) (e545–e608), (S0012369221015063), (10.1016/j.chest.2021.07.055)). Chest. 2022;162(1):269-. doi: 10.1016/j.chest.2022.05.028. PubMed PMID: CN-02462372.

367. Hyperimmune immunoglobulin for hospitalised patients with COVID-19 (ITAC): a double-blind, placebo-controlled, phase 3, randomised trial. Lancet (london, england). 2022;399(10324):530-40. doi:

10.1016/S0140-6736(22)00101-5. PubMed PMID: CN-02364357.

368. Retraction: a Potential Role of Ethosuximide and Pentoxifylline in Relieving Abdominal Pain in Irritable Bowel Syndrome Patients Treated with Mebeverine: a Randomized, Double-Blind, Placebo-Controlled Trial (J Inflamm Res., (2022), 15, (1159-1172), 10.2147/JIR.S346608). Journal of inflammation research. 2022;15:2381-. doi: 10.2147/JIR.S369945. PubMed PMID: CN-02407937.

369. Tixagevimab-cilgavimab for treatment of patients hospitalised with COVID-19: a randomised, double-blind, phase 3 trial. Lancet respiratory medicine. 2022;10(10):972-84. doi: 10.1016/S2213-2600(22)00215-6. PubMed PMID: CN-02426287.

370. Community pharmacies incentivised to refer frequent users of reliever inhalers to general practice. Pharmaceutical Journal. 2022;309(7966). doi: 10.1211/PJ.2022.1.161145.

371. Erratum: Pharmacist-driven penicillin skin-testing service for adults prescribed nonpreferred antibiotics in a community hospital (American Journal of Health-System Pharmacy (2019) (2060-2069) DOI: 10.1093/ajhp/zxz237). American Journal of Health-System Pharmacy. 2022;79(8):709. doi: 10.1093/ajhp/zxz343.

372. Corrections to “Quality of facility-based maternal and newborn care around the time of childbirth during the COVID-19 pandemic: Online survey investigating maternal perspectives in 12 countries of the WHO European Region” (The Lancet Regional Health - Europe (2022) 13, (S2666776221002544), (10.1016/j.lanepe.2021.100268)). The Lancet Regional Health - Europe. 2022;19. doi: 10.1016/j.lanepe.2022.100461.

373. In brief: Mifepristone by mail for pregnancy termination. The Medical letter on drugs and therapeutics. 2022;64(1642):11.

374. ASHP Standard for Certification as a Center of Excellence in Medication-Use Safety and Pharmacy Practice. American Journal of Health-System Pharmacy. 2022;79(7):564-99. doi: 10.1093/ajhp/zxab368.

375. The Quality of Counseling for Headache OTC Medications in German Community Pharmacies Using a Simulated Patient Approach: Are There Differences between Self-Purchase and Purchase for a Third Party? Pharmaceutical Care Espana. 2022;24(4):67-8. doi: 10.1155/2022/5851117. PubMed PMID: WOS:000888843100006.

376. Delivering specialised care to people ageing with HIV in the UK: experience and evolution of services from 2009 to 2019 Abstracts. Lancet Hiv. 2022;9:S1-S6. PubMed PMID: WOS:000771873300001.

377. Medication administration errors can induce fear, sadness and guilt among healthcare professionals. Pulse International. 2023;24(5):1-21. PubMed PMID: 162430529.

378. Duplication errors due to brand name confusion; It is not always the name—Short case series. Wiley-Blackwell; 2023. p. 1-3.

379. Acknowledgement to Referees. Drug Safety. 2023;46(12):1297-301. doi: 10.1007/s40264-023-01369-2. PubMed PMID: 173891516.

380. Medication Errors Surge in Kids On ADHD Drugs. Elder Law Report. 2023;35(3):7-. PubMed PMID: 172996053.

381. Correction. Pharmaceutical Biology. 2023;61(1):1413-. doi: 10.1080/13880209.2023.2254065. PubMed PMID: 174204248.

382. Amiodarone/diltiazem/liothyronine: Thyrotoxicosis, drug intolerance and worsening of thyrotoxicosis

following medication error: case report. Reactions Weekly. 2023;1985(1):45-. doi: 10.1007/s40278-023-50815-1. PubMed PMID: 173994590.

383. Paracetamol overdose: Acute liver failure and drug-toxicity followed by overdose and medication error: case report. Reactions Weekly. 2023;1982(1):350-. doi: 10.1007/s40278-023-49287-9. PubMed PMID: 173518226.

384. Lamivudine/nevirapine/zidovudine overdose: Various toxicities following medication error: case report. Reactions Weekly. 2023;1982(1):223-. doi: 10.1007/s40278-023-49160-0. PubMed PMID: 173518099.

385. Vincristine: Various toxicities following overdose or medication error. Reactions Weekly. 2023;1987(1):232-. doi: 10.1007/s40278-023-51976-3. PubMed PMID: 174257944.

386. Methotrexate: Leukoerythroblastosis following accidental overdose and medication error. Reactions Weekly. 2023;1987(1):142-. doi: 10.1007/s40278-023-51886-4. PubMed PMID: 174257854.

387. Artemether/lumefantrine: Treatment failure and medication error: case report. Reactions Weekly. 2023;1987(1):42-. doi: 10.1007/s40278-023-51786-6. PubMed PMID: 174257754.

388. Antineoplastics: Lack of efficacy, medication error and off label use: 3 case reports. Reactions Weekly. 2023;1985(1):65-. doi: 10.1007/s40278-023-50835-z. PubMed PMID: 173994610.

389. Mercaptopurine/scopolamine: Medication error leading to diplopia, cytopenias and drug overdose: 2 case reports. Reactions Weekly. 2023;1983(1):269-. doi: 10.1007/s40278-023-49618-8. PubMed PMID: 173823487.

390. Atovaquone/proguanil: Drug resistance, treatment noncompliance and medication error: case report. Reactions Weekly. 2023;1983(1):82-. doi: 10.1007/s40278-023-49431-x. PubMed PMID: 173823300.

391. Phenytoin: Overdose leading to drug-toxicity and medication error: case report. Reactions Weekly. 2023;1982(1):363-. doi: 10.1007/s40278-023-49300-2. PubMed PMID: 173518239.

392. Phenobarbital: Various toxicities following medication error: case report. Reactions Weekly. 2023;1982(1):359-. doi: 10.1007/s40278-023-49296-8. PubMed PMID: 173518235.

393. Risk of medication errors with use of UK-authorised potassium chloride. Reactions Weekly. 2023;1982(1):8-. doi: 10.1007/s40278-023-48945-2. PubMed PMID: 173517884.

394. Incorrect use of smart infusion pumps causes medication errors in NICU. Reactions Weekly. 2023;1981(1):10-. doi: 10.1007/s40278-023-48589-6. PubMed PMID: 173454411.

395. Trust but verify: A case report of unintended medication omission. Wiley-Blackwell; 2023. p. 1-3.

396. Paracetamol/Paracetamol/caffeine/salicylamide/promethazine/Paracetamol/ethenzamide/apronalide/caffeine: Idiosyncratic type of drug-induced liver injury and medication error. Reactions Weekly. 2023;1987(1):198-. doi: 10.1007/s40278-023-51942-6. PubMed PMID: 174257910.

397. Minoxidil: Various toxicities following medication error and poisoning of minoxidil. Reactions Weekly. 2023;1986(1):265-. doi: 10.1007/s40278-023-51570-y. PubMed PMID: 174180151.

398. Romosozumab/teriparatide: Various toxicity, drug-intolerance and medication error. Reactions Weekly. 2023;1985(1):469-. doi: 10.1007/s40278-023-51239-8. PubMed PMID: 173995014.

399. Glipizide/Hydrocortisone: Hypoglycaemia following medication error. Reactions Weekly. 2023;1984(1):254-. doi: 10.1007/s40278-023-50485-2. PubMed PMID: 173964902.

400. Clozapine: Medication errors and drug withdrawal: case report. Reactions Weekly. 2023;1983(1):149-. doi: 10.1007/s40278-023-49498-4. PubMed PMID: 173823367.

401. Calcium-gluconate/insulin/lanthanum-carbonate: Lack of efficacy along with ileus following drug misuse and medication error: case report. Reactions Weekly. 2023;1983(1):121-. doi: 10.1007/s40278-023-49470-6. PubMed PMID: 173823339.

402. Gabapentin/quetiapine: Akathisia, withdrawal symptom following misuse and medication error: 2 case reports. Reactions Weekly. 2023;1982(1):197-. doi: 10.1007/s40278-023-49134-3. PubMed PMID: 173518073.

403. Amoxicillin: Treatment non-compliance, medication error and penicillin allergy : case report. Reactions Weekly. 2023;1982(1):30-. doi: 10.1007/s40278-023-48967-0. PubMed PMID: 173517906.

404. Glucose/suxamethonium chloride: Elevated glucose level following medication error and rhabdomyolysis: 2 case reports. Reactions Weekly. 2023;1981(1):191-. doi: 10.1007/s40278-023-48770-3. PubMed PMID: 173454592.

405. Dulaglutide/semaglutide: Hypoglycaemia, intentional overdose, drug misuse and medication error : 4 case reports. Reactions Weekly. 2023;1979:173-. doi: 10.1007/s40278-023-47900-9. PubMed PMID: 173492247.

406. ADHD therapy medication errors in paediatric patients. Reactions Weekly. 2023;1975(1):10-. doi: 10.1007/s40278-023-46365-5. PubMed PMID: 172285833.

407. Fluorouracil: Myelosuppression following drug overdose secondary to medication error: case report. Reactions Weekly. 2023;1976(1):159-. doi: 10.1007/s40278-023-46888-6. PubMed PMID: 172444839.

408. Fenbendazole/hydroxychloroquine: Various toxicities, medication error, drug misuse and off-label use: case report. Reactions Weekly. 2023;1976(1):157-. doi: 10.1007/s40278-023-46886-6. PubMed PMID: 172444837.

409. Bupivacaine/epinephrine: Local anaesthetic systemic toxicity in the form of decreased blood pressure following medication error: case report. Reactions Weekly. 2023;1976(1):84-. doi: 10.1007/s40278-023-46813-0. PubMed PMID: 172444764.

410. Amlodipine: Various toxicities following overdose and medication error: 3 case reports. Reactions Weekly. 2023;1976(1):30-. doi: 10.1007/s40278-023-46759-y. PubMed PMID: 172444710.

411. Tranexamic acid: Polymyoclonus, ventricular fibrillation and Takotsubo syndrome following medication error: case report. Reactions Weekly. 2023;1975(1):364-. doi: 10.1007/s40278-023-46719-2. PubMed PMID: 172286187.

412. Warfarin: Coagulopathy secondary to drug toxicity and medication error: case report. Reactions Weekly. 2023;1973(1):282-. doi: 10.1007/s40278-023-46010-3. PubMed PMID: 171843395.

413. Methotrexate: Various toxicities following medication error: 3 case reports. Reactions Weekly. 2023;1973(1):172-. doi: 10.1007/s40278-023-45900-2. PubMed PMID: 171843285.

414. Carvedilol/spironolactone: BRASH syndrome and medication error: case report. Reactions Weekly.

2023;1973(1):83-. doi: 10.1007/s40278-023-45811-3. PubMed PMID: 171843196.

415. Etodolac/ofloxacin: Generalised bullous fixed-drug eruption and medication error: 2 case reports. Reactions Weekly. 2023;1972(1):165-. doi: 10.1007/s40278-023-45546-6. PubMed PMID: 171347556.

416. Medication errors with scopolamine and butylscopolamine. Reactions Weekly. 2023;1972(1):5-. doi: 10.1007/s40278-023-45386-6. PubMed PMID: 171347396.

417. Docetaxel/fluorouracil/oxaliplatin: Drug overdose, medication error and various toxicities: case report. Reactions Weekly. 2023;1970(1):120-. doi: 10.1007/s40278-023-44961-z. PubMed PMID: 170027863.

418. Ketorolac/tramadol: Status-epilepticus, lack of efficacy and medication error: case report. Reactions Weekly. 2023;1967(1):210-. doi: 10.1007/s40278-023-44017-9. PubMed PMID: 168596353.

419. Prednisone/ergocalciferol: Hypercalcaemia following rebound effect and kidney stone following medication error: case report. Reactions Weekly. 2023;1964(1):365-. doi: 10.1007/s40278-023-42923-9. PubMed PMID: 164784020.

420. Avoiding oxytocin medication errors: ISMP recommendations. Reactions Weekly. 2023;1961(1):3-. doi: 10.1007/s40278-023-41260-9. PubMed PMID: 164374415.

421. Semaglutide overdose: Various toxicities following medication error and drug overdose: 3 case reports. Reactions Weekly. 2023;1958(1):459-. doi: 10.1007/s40278-023-40133-4. PubMed PMID: 163940266.

422. ISMP guidance: Targeted Medication Safety Best Practices for Community Pharmacy. Reactions Weekly. 2023;1953(1):2-. doi: 10.1007/s40278-023-37437-7. PubMed PMID: 163256303.

423. Multiple drugs: Various toxicities and medication error: 9 case reports. Reactions Weekly. 2023;1950(1):306-. doi: 10.1007/s40278-023-36703-7. PubMed PMID: 162854037.

424. Multiple drugs: Pancytopenia, gastrointestinal toxicity following medication error and lack of efficacy: case report. Reactions Weekly. 2023;1961(1):303-. doi: 10.1007/s40278-023-41560-3. PubMed PMID: 164374715.

425. Azathioprine: Myelosuppression and alopecia following medication error: case report. Reactions Weekly. 2023;1961(1):82-. doi: 10.1007/s40278-023-41339-x. PubMed PMID: 164374494.

426. Loperamide/neostigmine: Ogilvie's syndrome following medication error and treatment failure: case report. Reactions Weekly. 2023;1959(1):377-. doi: 10.1007/s40278-023-40549-8. PubMed PMID: 164081221.

427. Vecuronium-bromide: Anaphylactic shock due to medication error: case report. Reactions Weekly. 2023;1958(1):493-. doi: 10.1007/s40278-023-40167-1. PubMed PMID: 163940303.

428. Rivastigmine: Acute cholinergic syndrome secondary to drug toxicity and medication error: case report. Reactions Weekly. 2023;1958(1):455-. doi: 10.1007/s40278-023-40129-5. PubMed PMID: 163940258.

429. Multiple drugs overdose: Bradycardic and unresponsive following medication error and overdose, lack of efficacy: case report. Reactions Weekly. 2023;1958(1):404-. doi: 10.1007/s40278-023-40078-2. PubMed PMID: 163940195.

430. Epinephrine overdose: Takotsubo-cardiomyopathy and epinephrine toxicity secondary to epinephrine overdose and medication error: case report. Reactions Weekly. 2023;1958(1):215-. doi: 10.1007/

s40278-023-39889-1. PubMed PMID: 163940006.

431. Urapidil: Various toxicities following medication error: case report. Reactions Weekly. 2023;1957(1):532-. doi: 10.1007/s40278-023-39657-8. PubMed PMID: 163799513.

432. Sibutramine: Rhabdomyolysis with multisystem injury following medication error: case report. Reactions Weekly. 2023;1957(1):486-. doi: 10.1007/s40278-023-39611-z. PubMed PMID: 163799467.

433. Rocuronium-bromide: Delayed recovery from anaesthesia following medication error: case report. Reactions Weekly. 2023;1957(1):479-. doi: 10.1007/s40278-023-39604-0. PubMed PMID: 163799460.

434. Diazoxide: Water and sodium retention, hypoglycaemia and medication error: case report. Reactions Weekly. 2023;1957(1):207-. doi: 10.1007/s40278-023-39332-3. PubMed PMID: 163799188.

435. Potassium/tacrolimus/trimethoprim: Lack of efficacy and drug toxicity following medication error and overdose : case report. Reactions Weekly. 2023;1956(1):433-. doi: 10.1007/s40278-023-39046-8. PubMed PMID: 163729765.

436. Celecoxib: Bullous symmetrical drug-related intertriginous and flexural exanthem following accidental administration of celecoxib (medication error): case report. Reactions Weekly. 2023;1956(1):135-. doi: 10.1007/s40278-023-38748-x. PubMed PMID: 163729467.

437. Bromocriptine/cabergoline: Hives and medication error: case report. Reactions Weekly. 2023;1956(1):115-. doi: 10.1007/s40278-023-38728-z. PubMed PMID: 163729447.

438. Sugammadex: Drug-induced myasthenic crisis and various toxicities secondary to medication error: 2 case reports. Reactions Weekly. 2023;1952(1):312-. doi: 10.1007/s40278-023-37405-x. PubMed PMID: 163100357.

439. Multiple drugs: Various toxicities, overdose, medication error and lack of efficacy: 5 case reports. Reactions Weekly. 2023;1952(1):274-. doi: 10.1007/s40278-023-37367-6. PubMed PMID: 163100319.

440. ISMP adds more drug name pairs to List of Confused Drug Names. Reactions Weekly. 2023;1970(1):8-. doi: 10.1007/s40278-023-44849-3. PubMed PMID: 170027751.

441. Biotin/levothyroxine-sodium: Severe hypothyroidism following drug titration error and interference in thyroid tests: case report. Reactions Weekly. 2023;1969(1):92-. doi: 10.1007/s40278-023-44558-8. PubMed PMID: 169912634.

442. Capecitabine: Various toxicities: 4 case reports. Reactions Weekly. 2023;1964(1):116-. doi: 10.1007/s40278-023-42674-x. PubMed PMID: 164783771.

443. Outpatient medication errors common among children with leukaemia or lymphoma. Reactions Weekly. 2023;1945(1):11-. doi: 10.1007/s40278-023-34092-5. PubMed PMID: 162078613.

444. Avoiding medication errors when diluting Bendamustine Accord concentrate. Reactions Weekly. 2023;1947(1):1-. doi: 10.1007/s40278-023-35141-5. PubMed PMID: 162358907.

445. Amoxicillin/clavulanic-acid: Anaphylaxis and medication error: case report. Reactions Weekly. 2023;1949(1):49-. doi: 10.1007/s40278-023-36029-6. PubMed PMID: 162683676.

446. Paracetamol: High anion gap metabolic acidosis and acute-kidney-injury following medication error and overdose: case report. Reactions Weekly. 2023;1948(1):353-. doi: 10.1007/s40278-023-35910-5. PubMed

PMID: 162507313.

447. Dexamethasone/sodium hypochlorite: Various toxicities following medication error: case report. Reactions Weekly. 2023;1948(1):163-. doi: 10.1007/s40278-023-35720-8. PubMed PMID: 162507123.

448. Lidocaine: Convulsions, unconsciousness, lidocaine toxicity following medication error and overdose: case report. Reactions Weekly. 2023;1946(1):322-. doi: 10.1007/s40278-023-34923-7. PubMed PMID: 162235603.

449. Voxelotor: Multi-organ dysfunction secondary to medication error: case report. Reactions Weekly. 2023;1947(1):412-. doi: 10.1007/s40278-023-35552-9. PubMed PMID: 162359318.

450. Betamethasone: Necrotising fasciitis following medication error: case report. Reactions Weekly. 2023;1947(1):104-. doi: 10.1007/s40278-023-35244-3. PubMed PMID: 162359010.

451. Multiple drugs: Various fatal drug toxicities, medication error and lack of efficacy: 45 case reports. Reactions Weekly. 2023;1945(1):393-. doi: 10.1007/s40278-023-34474-z. PubMed PMID: 162078995.

452. Cotrimoxazole: Purpura fulminans, disseminated-intravascular-coagulation following medication error: case report. Reactions Weekly. 2023;1945(1):190-. doi: 10.1007/s40278-023-34271-3. PubMed PMID: 162078792.

453. Etomidate: Decrease in blood pressure following medication error: case report. Reactions Weekly. 2023;1944(1):269-. doi: 10.1007/s40278-023-33788-8. PubMed PMID: 161958194.

454. Estradiol: Gynaecomastia and bone age acceleration following medication error: case report. Reactions Weekly. 2023;1944(1):268-. doi: 10.1007/s40278-023-33787-8. PubMed PMID: 161958193.

455. Multiple drugs: Various toxicities following off-label use, drug interaction, medication errors and drug overdose: 22 case reports. Reactions Weekly. 2023;1941(1):295-. doi: 10.1007/s40278-023-32558-5. PubMed PMID: 161581959.

456. Botulinum-toxin-A: Various toxicities and medication error: case report. Reactions Weekly. 2023;1940(1):117-. doi: 10.1007/s40278-023-31975-y. PubMed PMID: 161419026.

457. Iron-sucrose: Various toxicities due to drug overdose following medication error: case report. Reactions Weekly. 2023;1938(1):770-. doi: 10.1007/s40278-023-30911-7. PubMed PMID: 161299963.

458. Diclofenac: Acute limb ischaemia progressed to limb gangrene following medication error: case report. Reactions Weekly. 2023;1938(1):526-. doi: 10.1007/s40278-023-30667-8. PubMed PMID: 161299719.

459. Methadone/ondansetron: QTc prolongation and medication error: 2 case reports. Reactions Weekly. 2023;1939(1):295-. doi: 10.1007/s40278-023-31703-9. PubMed PMID: 161305689.

460. Digoxin: Various toxicities following medication error: case report. Reactions Weekly. 2023;1939(1):177-. doi: 10.1007/s40278-023-31585-5. PubMed PMID: 161305571.

461. Scientific Presentation - SP: Pharm (Pharmacology). Age & Ageing. 2023;52:i15-i. doi: 10.1093/ageing/afac322. PubMed PMID: 161457379.

462. Multiple drugs: Various toxicities: 7 case reports. Reactions Weekly. 2023;1942(1):219-. doi: 10.1007/s40278-023-32828-2. PubMed PMID: 161653917.

463. Prescribing Skills Assessment. Prescriber. 2023;34(1):16-7. doi: 10.1002/psb.2031. PubMed PMID: 161472323.

464. New guidelines to improve medication safety. Nursing. 2023;53(11):8-. doi: 10.1097/01.NURSE.0000978844.04554.69. PubMed PMID: 173262777.

465. Multiple drugs: Lack of efficacy and medication error: case report. Reactions Weekly. 2023;1980(1):269-. doi: 10.1007/s40278-023-48461-x. PubMed PMID: 173274743.

466. Naloxone/sodium-oxybate: Respiratory failure following medication error and no response to naloxone: case report. Reactions Weekly. 2023;1972(1):274-. doi: 10.1007/s40278-023-45655-3. PubMed PMID: 171347665.

467. Nirmatrelvir+ritonavir/tacrolimus interaction: Various toxicities following medication error: case report. Reactions Weekly. 2023;1958(1):413-. doi: 10.1007/s40278-023-40087-1. PubMed PMID: 163940204.

468. Ketamine/lithium/varenicline: Psychosis, medication error, drug-misuse and abuse: case report. Reactions Weekly. 2023;1955(1):220-. doi: 10.1007/s40278-023-38467-1. PubMed PMID: 163557615.

469. Dihydrocodeine/heparin: Gangrene following drug abuse and medication error and no improvement: case report. Reactions Weekly. 2023;1948(1):167-. doi: 10.1007/s40278-023-35724-8. PubMed PMID: 162507127.

470. Gabapentin/paclitaxel: Peripheral neuropathy, and overflow urinary incontinence secondary to drug overdose and medication error: case report. Reactions Weekly. 2023;1946(1):256-. doi: 10.1007/s40278-023-34857-6. PubMed PMID: 162235537.

471. Atropine/Ringers lactate: Autonomic instability, anticholinergic toxidrome and medication error: case report. Reactions Weekly. 2023;1946(1):86-. doi: 10.1007/s40278-023-34687-7. PubMed PMID: 162235367.

472. Tetrahydrocannabinol: Cerebral oedema, encephalopathy and drug-toxicity following medication error: case report. Reactions Weekly. 2023;1943(1):607-. doi: 10.1007/s40278-023-33490-0. PubMed PMID: 161821634.

473. Immunosuppressants/levofloxacin/lisinopril: Bullous pemphigoid, medication error and unintentional treatment non-compliance: case report. Reactions Weekly. 2023;1940(1):220-. doi: 10.1007/s40278-023-32078-0. PubMed PMID: 161419129.

474. Hydromorphone: Lack of efficacy: case report. Reactions Weekly. 2023;1976(1):171-. doi: 10.1007/s40278-023-46900-z. PubMed PMID: 172444851.

475. Nirmatrelvir+ritonavir: Various toxicities : 6 case reports. Reactions Weekly. 2023;1973(1):224-. doi: 10.1007/s40278-023-45952-x. PubMed PMID: 171843337.

476. Oxycodone: Death following administration error and accidental overdose: case report. Reactions Weekly. 2023;1972(1):288-. doi: 10.1007/s40278-023-45669-2. PubMed PMID: 171347679.

477. Fluphenazine: Nicolau syndrome: case report. Reactions Weekly. 2023;1972(1):168-. doi: 10.1007/s40278-023-45549-6. PubMed PMID: 171347559.

478. Triple therapy as safe as dual therapy for elephantiasis prevention. Reactions Weekly. 2023;1972(1):12-. doi: 10.1007/s40278-023-45393-5. PubMed PMID: 171347403.

479. Non-Intentional N-Acetylcysteine Overdose Associated with Cerebral Edema and Brain Death. Karger AG; 2023. p. 96-103.

480. Hypnosis in High-Intensity Focused Ultrasound for Thyroid Nodule Ablation. International Scientific Literature, Inc; 2023. p. 1-4.

481. Buprenorphine/fentanyl: Opioid withdrawal symptoms, prescription error and lack of efficacy: case report. Reactions Weekly. 2023;1962(1):103-. doi: 10.1007/s40278-023-41773-y. PubMed PMID: 164488543.

482. Fotemustine/temozolomide: Lack of efficacy: case report. Reactions Weekly. 2023;1957(1):260-. doi: 10.1007/s40278-023-39385-y. PubMed PMID: 163799241.

483. A Case of an Intraoperative Iatrogenic Methanol Exposure. International Scientific Literature, Inc; 2023. p. 1-4.

484. Accidental intrathecal injection of tranexamic acid: a case report. BioMed Central; 2023. p. 1-6.

485. Fluphenazine/levodopa/sodium valproate: Neurotoxicity in the form of parkinsonism, prescription error and drug ineffective: case report. Reactions Weekly. 2023;1947(1):193-. doi: 10.1007/s40278-023-35333-2. PubMed PMID: 162359099.

486. Q&A with Dan Sheridan, MS, RPh, CPPS. 2023. p. 52-.

487. Insulin abuse: Thrombophlebitis and necrotising fasciitis following drug abuse and medication error: case report. Reactions Weekly. 2023;1986(1):219-. doi: 10.1007/s40278-023-51524-3. PubMed PMID: 174180105.

488. Pharmacogenetic Testing in a 70-Year-Old Woman with Polypharmacy and Multiple Comorbidities: A Case Report. International Scientific Literature, Inc; 2023. p. 1-6.

489. Immunosuppressants/Varicella-zoster-virus-vaccine: Disseminated varicella zoster vaccine virus infection and medication error. Reactions Weekly. 2023;1987(1):127-. doi: 10.1007/s40278-023-51871-5. PubMed PMID: 174257839.

490. Dexamethasone/voriconazole: Invasive cerebral aspergillosis due to Aspergillus Niger, lack of efficacy, off label use and medication error: 3 case reports. Reactions Weekly. 2023;1976(1):131-. doi: 10.1007/s40278-023-46860-8. PubMed PMID: 172444811.

491. Is your home blood pressure monitor accurate? Harvard Health Letter. 2023;48(11):Cover-Cover. PubMed PMID: 171333490.

492. I/V Regular Insulin Use to Augment Cocaine Effect by Drug Abuser: New Dangerous Face of Substance Abuse. Journal of Medical Education & Research; 2023. p. 131-2.

493. 3 New Year's medication resolutions. Harvard Health Letter. 2023;48(3):1-7. PubMed PMID: 160925896.

494. Satellite Symposium. Epilepsia (Series 4). 2023;64:1-6. doi: 10.1111/epi.17786. PubMed PMID: 173925084.

495. Sintilimab: Immune-mediated adverse events: case report. Reactions Weekly. 2023;1984(1):482-. doi: 10.1007/s40278-023-50713-3. PubMed PMID: 173965130.

496. Iatrogenic Cushing syndrome in a child due to erroneous compounding of omeprazole containing glucocorticoid: A case report and literature review. 2023. p. 247-50.

497. Ibrutinib/venetoclax/voriconazole: CNS aspergillosis, fever and medication error: case report. Reactions Weekly. 2023;1938(1):700-. doi: 10.1007/s40278-023-30841-6. PubMed PMID: 161299893.

498. Norepinephrine: Skin necrosis following prolonged peripheral IV infusion of norepinephrine prepared at wrong concentration: case report. Reactions Weekly. 2023;1982(1):329-. doi: 10.1007/s40278-023-49266-y. PubMed PMID: 173518205.

499. Update on changing Australian medicine names. Australian Prescriber. 2023;46(3):64-. doi: 10.18773/austprescr.2023.016. PubMed PMID: 173252850.

500. Sorgfaltsanforderungen bei Medikamentengabe durch Krankenschwester. Care requirements when administering medication by a nurse. 2023;22(6):384-91. doi: 10.9785/gesr-2023-220614. PubMed PMID: 164227789.

501. Treatment and Management of Mental Health Conditions During Pregnancy and Postpartum: ACOG Clinical Practice Guideline No. 5. Obstetrics & Gynecology. 2023;141(6):1262-88. doi: 10.1097/AOG.0000000000005202. PubMed PMID: 163800043.

502. It's time to ease up. Economist. 2023;448(9364):49-. PubMed PMID: 172527066.

503. Nadolol/propranolol: Various toxicities : 2 case reports. Reactions Weekly. 2023;1973(1):223-. doi: 10.1007/s40278-023-45951-x. PubMed PMID: 171843336.

504. Other adverse reaction research. Reactions Weekly. 2023;1956(1):13-. doi: 10.1007/s40278-023-38626-1. PubMed PMID: 163729345.

505. Leflunomide/rifampicin/tocilizumab: Various toxicites, medication-error and lack of efficacy: case report. Reactions Weekly. 2023;1986(1):235-. doi: 10.1007/s40278-023-51540-1. PubMed PMID: 174180121.

506. Paracetamol: Aspartate aminotransferase elevation, drug overdose and dosing error: 3 case reports. Reactions Weekly. 2023;1976(1):274-. doi: 10.1007/s40278-023-47003-1. PubMed PMID: 172444954.

507. Methotrexate overdose: Acute kidney injury following inadvertent administration of incorrect dose: case report. Reactions Weekly. 2023;1957(1):361-. doi: 10.1007/s40278-023-39486-9. PubMed PMID: 163799342.

508. Why you keep waking up to go to the bathroom. Harvard Health Letter. 2023;48(5):4-. PubMed PMID: 162042658.

509. Multiple drugs: Various toxicities: 4 case reports. Reactions Weekly. 2023;1978(1):281-2. doi: 10.1007/s40278-023-47648-y. PubMed PMID: 172949392.

510. Betamethasone-sodium-phosphate: Increased intraocular pressure and medication-error: case report. Reactions Weekly. 2023;1962(1):95-. doi: 10.1007/s40278-023-41765-z. PubMed PMID: 164488535.

511. Hydrocortisone/levothyroxine-sodium: Osteopenia and osteoporosis following overtreatment: case report. Reactions Weekly. 2023;1974(1):169-. doi: 10.1007/s40278-023-46180-7. PubMed PMID: 171994740.

512. Dexmedetomidine (Precedex): risk of increased mortality in patients aged 65 years and younger. Reactions Weekly. 2023;1960(1):4-. doi: 10.1007/s40278-023-40832-3. PubMed PMID: 164225723.

513. Glucose/methylthioninium-chloride/paromomycin: Convulsions, loss of consciousness and loss of vision secondary to errors in infusion technique: 2 case report. Reactions Weekly. 2023;1948(1):218-. doi: 10.1007/s40278-023-35775-3. PubMed PMID: 162507178.

514. Multiple drugs: Neutropenia and lack of efficacy: case report. Reactions Weekly. 2023;1946(1):393-. doi: 10.1007/s40278-023-34994-0. PubMed PMID: 162235674.

515. ISMP updates list of drug names with mixed case lettering to prevent errors. Reactions Weekly. 2023;1943(1):3-. doi: 10.1007/s40278-023-32888-9. PubMed PMID: 161821032.

516. Nitroglycerin overdose: Methaemoglobinaemia and decreased oxygen saturation levels following off-label use and inadvertent exposure to high doses of drug: case report. Reactions Weekly. 2023;1942(1):229-. doi: 10.1007/s40278-023-32838-1. PubMed PMID: 161653927.

517. O.O.9.4 - A pharmacist communicating about medicines with carers of people with intellectual disabilities: Presenter(s): Bernadette Flood, Avista formerly Daughters of Charity, Ireland. Patient Education and Counseling. 2023;109:90. doi: https://doi.org/10.1016/j.pec.2022.10.212.

518. Society for Maternal-Fetal Medicine Statement: Response to the Food and Drug Administration's withdrawal of 17-alpha hydroxyprogesterone caproate. American Journal of Obstetrics and Gynecology. 2023;229(1):B2-B6. doi: https://doi.org/10.1016/j.ajog.2023.04.012.

519. Beyond 'safety of supply': a response to Harry McQuillan's lecture. Pharmaceutical Journal. 2023;311(7975). doi: 10.1211/PJ.2023.1.192552.

520. Correction to: 50th ESCP Symposium on Clinical Pharmacy, Polypharmacy and ageing - highly individualized, interprofessional, person-centered care: 19–21 October 2022, Prague, Czech Republic (International Journal of Clinical Pharmacy, (2022), 44, 6, (1464-1566), 10.1007/s11096-022-01521-5). International Journal of Clinical Pharmacy. 2023;45(2):545-6. doi: 10.1007/s11096-023-01552-6.

521. Pharmacy visits: Every third pharmacy is at risk: Stefan Schwartze (SPD) visits the market pharmacy in Vlotho. Nederlands Tijdschrift voor Geneeskunde. 2023;167(49).

522. Independent Community Pharmacy Association triumphs over Clicks. SA Pharmaceutical Journal. 2023;90(3):8-11. doi: 10.10520/ejc-mp_sapj_v90_n3_a3.

523. A T, Sinha S, Mohan P, Dubey S, Yadav V. A cross-sectional questionnaire based study to assess and compare knowledge of drug schedules amongst healthcare professionals in a medical college. Medical Journal Armed Forces India. 2022;78:S172-S8. doi: https://doi.org/10.1016/j.mjafi.2020.12.021.

524. Aabenhus R, Siersma V, Hansen MP, Bjerrum L. Antibiotic prescribing in Danish general practice 2004-13. Journal of Antimicrobial Chemotherapy. 2016;71(8):2286-94. doi: 10.1093/jac/dkw117.

525. Aag T, Garcia B, Viktil KK. Should the first step in a medication review: the medication reconciliation-be performed by nurses or by pharmacists? International journal of clinical pharmacy. 2014;36(4):852-. doi: 10.1007/s11096-014-9952-7. PubMed PMID: CN-01023430.

526. Aag T, Garcia BH, Viktil KK. Should nurses or clinical pharmacists perform medication reconciliation? A randomized controlled trial. European journal of clinical pharmacology. 2014;70(11):1325-32. doi: 10.1007/s00228-014-1741-7. PubMed PMID: CN-01040455.

527. Aagaard L, Nielsen LH, Hansen EH. Consumer reporting of adverse drug reactions: a retrospective analysis of the Danish adverse drug reaction database from 2004 to 2006. Drug safety. 2009;32:1067-74.

528. Aaltonen KJ, Turunen JH, Sokka T, Puolakka K, Valleala H. A survey on the medication adherence to methotrexate among rheumatoid arthritis patients treated with self-administered biologic drugs. Clinical and Experimental Rheumatology. 2016;34(4):694-7.

529. Aaltonen SE, Laine NP, Volmer D, Gharat MS, Muceniece R, Vitola A, et al. Barriers to medication counselling for people with mental health disorders: A six country study. Pharmacy Practice. 2010;8(2):122-31. doi: 10.4321/S1886-36552010000200007.

530. Aapro MS, Köhne C-H, Cohen HJ, Extermann M. Never too old? Age should not be a barrier to enrollment in cancer clinical trials. The oncologist. 2005;10(3):198-204.

531. Aarnes TK, Schmuhl KK, Summers K, Sandidge BR. Medication safety education: more than just a human concern? Journal of the American Veterinary Medical Association. 2023;261(4):597-600. doi: 10.2460/javma.22.09.0422. PubMed PMID: 163303111.

532. Aarnoudse A, Dieleman JP, Stricker BHC. Age- and gender-specific incidence of hospitalisation for digoxin intoxication. Drug Safety. 2007;30(5):431-6. doi: 10.2165/00002018-200730050-00006. PubMed PMID: WOS:000246827800006.

533. Aarnoudse ALHJ, Dieleman JP, Stricker BHC. Age- and gender-specific incidence of hospitalisation for digoxin intoxication. Drug Safety. 2007;30(5):431-6. doi: 10.2165/00002018-200730050-00006.

534. Aarsland D, Ballard C, Walker Z, Bostrom F, Alves G, Kossakowski K, et al. Memantine in patients with Parkinson's disease dementia or dementia with Lewy bodies: a double-blind, placebo-controlled, multicentre trial. The Lancet Neurology. 2009;8(7):613-8.

535. Aarts J, Ash J, Berg M. Extending the understanding of computerized physician order entry: implications for professional collaboration, workflow and quality of care. International journal of medical informatics. 2007;76:S4-S13.

536. Aarts J, Doorewaard H, Berg M. Understanding implementation: the case of a computerized physician order entry system in a large Dutch university medical center. Journal of the American Medical Informatics Association. 2004;11(3):207-16.

537. Aasen T, Culver M. Turning off the drip: a single center evidence-based cost savings model for high risk upper gastrointestinal bleeding. Gastroenterology. 2017;152(5):S475-. PubMed PMID: CN-01422310.

538. Ab Rahman AF. Monitoring adverse drug reactions in the community settings. Bulletin of the World Health Organization. 2019;97(11):730. PubMed PMID: 31673185.

539. Ab Rahman NS, Pakururazi FA, Zin CS, Abd Halim H. Prescribing patterns of benzodiazepine in Malaysia from 2014 to 2016. Pharmacoepidemiology and Drug Safety. 2018;27(Supplement 2):317. PubMed PMID: 623865698.

540. Ababneh M, Shamieh D, Al Demour S, Rababa'h A. Evaluation of the clinical pharmacist role in improving clinical outcomes in patients with lower urinary tract symptoms due to benign prostatic hyperplasia. International journal of clinical pharmacy. 2019;41(5):1373-8. doi: 10.1007/s11096-019-00896-2. PubMed PMID: CN-01989351.

541. Ababneh MA, Al-Azzam SI, Alzoubi KH, Rababa'h AM. Medication errors in outpatient pharmacies:

comparison of an electronic and a paper-based prescription system. Journal of Pharmaceutical Health Services Research. 2020;11(3):245-8. PubMed PMID: 2004808978.

542. Ababu A, Endashaw D, Fesseha H, Mathewos M. Antiprotozoal Drug Handling and Management Practices in Asella District, Central Oromia, Ethiopia. Veterinary Medicine International. 2021;2021. doi: 10.1155/2021/6648328.

543. Abad Sazatornil MR, Carcelén Andrés J, Aibar Remón C, Arrieta Navarro R, Gómez-Barrera M, Agustín Fernández MJ. Errores de medicación asociados al sistema de distribución de medicamentos en dosis unitarias. Concordancia entre observadores. Revista de Calidad Asistencial. 2007;22(6):310-6. doi: https://doi.org/10.1016/S1134-282X(07)71239-3.

544. Abahuje E, Diaz CM, Lin KA, Tesorero K, Bushara O, Yang S, et al. A qualitative study of how team characteristics and leadership are associated with information sharing in multidisciplinary intensive care units. Surgery. 2023;174(2):350-5. doi: https://doi.org/10.1016/j.surg.2023.03.017.

545. Abahussain E, Waheedi M, Koshy S. Practice, awareness and opinion of pharmacists toward disposal of unwanted medications in Kuwait. Saudi Pharmaceutical Journal. 2012;20(3):195-201. doi: https://doi.org/10.1016/j.jsps.2012.04.001.

546. Abahussain EA, Ball DE. Disposal of unwanted medicines from households in Kuwait. Pharmacy World and Science. 2007;29(4):368-73. doi: 10.1007/s11096-006-9082-y.

547. Abahussain NA, Abahussain EA, Al-Oumi FM. Pharmacists' attitudes and awareness towards the use and safety of herbs in Kuwait. Pharmacy Practice. 2007;5(3):125-9. doi: 10.4321/S1886-36552007000300005.

548. Abalahin AJ. Prostitution policy and the project of modernity: A comparative study of colonial Indonesia and the Philippines, 1850–1940 [Ph.D.]. United States -- New York: Cornell University; 2003.

549. Abaluck J, Gruber J. Choice inconsistencies among the elderly: evidence from plan choice in the Medicare Part D program. American Economic Review. 2011;101(4):1180-210.

550. Abar CC, Combs A, Miley A, Ruffino R, Abar B. Parenting, health, and use of medications among college youth: The PHARMACY survey study. Preventive Medicine Reports. 2021;24:101623. doi: https://doi.org/10.1016/j.pmedr.2021.101623.

551. Abarbanel J, Marcus EL. Impaired detrusor contractility in community-dwelling elderly presenting with lower urinary tract symptoms. Urology. 2007;69(3):436-40. doi: 10.1016/j.urology.2006.11.019. PubMed PMID: WOS:000245566500006.

552. Abarca J, Colon LR, Wang VS, Malone DC, Murphy JE, Armstrong EP. Evaluation of the performance of drug-drug interaction screening software in community and hospital pharmacies. Journal of Managed Care Pharmacy. 2006;12(5):383-9.

553. Abarca J, Malone DC, Armstrong EP, Grizzle AJ, Hansten PD, Van Bergen RC, et al. Concordance of severity ratings provided in four drug interaction compendia. Journal of the American Pharmacists Association. 2004;44(2):136-41.

554. Abarca J, Malone DC, Skrepnek GH, Rehfeld RA, Murphy JE, Grizzle AJ, et al. Community pharmacy managers' perception of computerized drug-drug interaction alerts. Journal of the American Pharmacists Association. 2006;46(2):148-53. doi: 10.1331/154434506776180676.

555. Abasaeed A, Vlcek J, Abuelkhair M, Kubena A. Self-medication with antibiotics by the community of Abu Dhabi Emirate, United Arab Emirates. Journal of Infection in Developing Countries. 2009;3(7):491-7. doi: 10.3855/jidc.466.

556. Abasaeed AE, Abuelkhair MA, Andrajati R, Elnour AA. A comparative study between prescribed and over-thecounter antibiotics. Saudi Medical Journal. 2013;34(10):1048-54.

557. Abasaeed AE, Vlcek J, Abuelkhair MA, Andrajati R, Elnour AA. A comparative study between prescribed and over-the-counter antibiotics. Saudi Med J. 2013;34(10):1048-54.

558. Abat C, Desboves G, Olaitan AO, Chaudet H, Roattino N, Fournier PE, et al. Increasing burden of urinary tract infections due to intrinsic colistin-resistant bacteria in hospitals in Marseille, France. International Journal of Antimicrobial Agents. 2015;45(2):144-50. doi: 10.1016/j.ijantimicag.2014.10.010.

559. Abate D, Aman MA, Bedru B, Gebremariam GT, Fentie AM. Assessment of Quality of Care Using Information on Patient Satisfaction at Adult Oncology Center of Tikur Anbessa Specialized Hospital, Ethiopia: A Cross-Sectional Study. Patient Preference and Adherence. 2020;14:847-58. doi: 10.2147/ppa.S253027. PubMed PMID: WOS:000535738200001.

560. Abaurre R, García-Delgado P, Maurandi MD, Arrebola C, Gastelurrutia MÁ, Martínez-Martínez F. Diseño y pilotaje de un proceso estructurado para el servicio de dispensación de medicamentos. Atención Primaria. 2015;47(1):7-14. doi: https://doi.org/10.1016/j.aprim.2014.01.005.

561. Abaurre R, García-Delgado P, Maurandi MD, Arrebola C, Gastelurrutia MÁ, Martínez-Martínez F. Design and piloting of a structured service medication dispensing process. Atencion Primaria. 2015;47(1):7-14. doi: 10.1016/j.aprim.2014.01.005.

562. Abaurre-Labrador R, Maurandi-Guillén MD, García-Delgado P, Moullin JC, Martínez-Martínez F, García-Corpas JP. Effectiveness of a protocolized dispensing service in community pharmacy for improving patient medication knowledge. International Journal of Clinical Pharmacy. 2016;38(5):1057-62. doi: 10.1007/s11096-016-0356-8.

563. Abaynew Y, Hussien M. A Qualitative Study on Barriers to Treatment and Control of Hypertension Among Patients at Dessie Referral Hospital, Northeast Ethiopia, Ethiopia: Healthcare Workers' Perspective. Integrated Blood Pressure Control. 2021;14:173-8. doi: 10.2147/ibpc.S339773. PubMed PMID: WOS:000729069800001.

564. Abazari MA, Rafieianzab D, Soltani M, Alimohammadi M. The effect of beta-blockers on hemodynamic parameters in patient-specific blood flow simulations of type-B aortic dissection: a virtual study. Scientific Reports. 2021;11(1). doi: 10.1038/s41598-021-95315-w. PubMed PMID: WOS:000684558900015.

565. Abba D, Inabo HI, Yakubu SE, Olonitola OS. CONTAMINATION OF HERBAL MEDICINAL PRODUCTS MARKETED IN KADUNA METROPOLIS WITH SELECTED PATHOGENIC BACTERIA. African Journal of Traditional Complementary and Alternative Medicines. 2009;6(1):70-7. PubMed PMID: WOS:000260922900010.

566. Abbas A, Al-Shaibi S, Sankaralingam S, Awaisu A, Kattezhathu VS, Wongwiwatthananukit S, et al. Determination of potential drug–drug interactions in prescription orders dispensed in a community pharmacy setting using Micromedex® and Lexicomp®: a retrospective observational study. International Journal of Clinical Pharmacy. 2022;44(2):348-56. doi: 10.1007/s11096-021-01346-8.

567. Abbas A, Al-Shaibi S, Sankaralingam S, Awaisu A, Kattezhathu VS, Wongwiwatthananukit S, et al.

Determination of potential drug-drug interactions in prescription orders dispensed in a community pharmacy setting using Micromedex (R) and Lexicomp (R): a retrospective observational study. International Journal of Clinical Pharmacy. 2022;44(2):348-56. doi: 10.1007/s11096-021-01346-8. PubMed PMID: WOS:000721390300001.

568. Abbas B, Marotta PL, Goddard-Eckrich D, Huang D, Schnaidt J, El-Bassel N, et al. Socio-ecological and pharmacy-level factors associated with naloxone stocking at standing-order naloxone pharmacies in New York City. Drug and Alcohol Dependence. 2021;218:108388. doi: https://doi.org/10.1016/j.drugalcdep.2020.108388.

569. Abbas H, Kurdi M, Watfa M, Karam R. Adherence to treatment and evaluation of disease and therapy knowledge in Lebanese hypertensive patients. Patient Preference and Adherence. 2017;11:1949-56. doi: 10.2147/PPA.S142453.

570. Abbasi K, Hazrati M, Mohammadbeigi A, Ansari J, Sajadi M, Hosseinnazzhad A, et al. Protection behaviors for cytotoxic drugs in oncology nurses of chemotherapy centers in Shiraz hospitals, South of Iran. Indian Journal of Medical and Paediatric Oncology. 2016;37(4):227-31. doi: 10.4103/0971-5851.195748. PubMed PMID: WOS:000391681100005.

571. Abbasi R, Alavi NM, Farzandipour M, Gong Y, Nabovati E. Using pharmacy surveillance information systems to monitor the dispensing practice of under-controlled drugs: A qualitative study on necessities, requirements, and implementation challenges. Informatics in Medicine Unlocked. 2023;38:101198. doi: https://doi.org/10.1016/j.imu.2023.101198.

572. Abbasi S, Sıcakyüz Ç, Erdebilli B. Designing the home healthcare supply chain during a health crisis. Journal of Engineering Research. 2023;11(4):447-52. doi: https://doi.org/10.1016/j.jer.2023.100098.

573. Abbasinazari M, Hajhossein Talasaz A, Eshraghi A, Sahraei Z. Detection and management of medication errors in internal wards of a teaching hospital by clinical pharmacists. Acta Medica Iranica. 2013;51(7):482-6. PubMed PMID: 23945894.

574. Abbasinazari M, Salamzadeh J, Eshraghi A, Belbasi V, Ashnagar A. Evaluating community pharmacists' dyspepsia knowledge: A crosssectional study in Tehran, Iran. Reviews on Recent Clinical Trials. 2017;12(1):34-7. doi: 10.2174/1574887111666160905122858.

575. Abbasinazari M, Salamzadeh J, Eshraghi A, Belbasi V, Ashnagar A. Evaluating Community Pharmacists' Dyspepsia Knowledge: A Cross-sectional Study in Tehran, Iran. Reviews on Recent Clinical Trials. 2017;12(1):34-7. doi: 10.2174/1574887111666160905122858. PubMed PMID: WOS:000397250500006.

576. Abbasinazari M, Tavana A. Identification and acceptance of suggested therapeutic alternatives of potentially inappropriate medications amongst hospitalised geriatric patients in Iran. Journal of Pharmacy Practice & Research. 2020;50(1):75-7. doi: 10.1002/jppr.1583. PubMed PMID: 141780250. Language: English. Entry Date: 20200225. Revision Date: 20210201. Publication Type: Article.

577. Abbasinazari M, Zareh-Toranposhti S, Hassani A, Sistanizad M, Azizian H, Panahi Y. The effect of information provision on reduction of errors in intravenous drug preparation and administration by nurses in ICU and surgical wards. Acta Medica Iranica. 2012:771-7.

578. Abbass I, Mhatre S, Sansgiry S, Tipton J, Frost C. Impact and determinants of commercial computerized prescriber order entry on the medication administration process. Hospital Pharmacy. 2011;46(5):341-8. doi: 10.1310/hpj4605-341.

579. Abbassi A, Cheikh Brahim AB, Ouahchi Z. Failure mode and effect analysis applied to improve the medication management process in a pharmacy of a teaching hospital and a proposal for a simplified rating system. European Journal of Hospital Pharmacy. 2023;30(E1):E55-E60. doi: https://dx.doi.org/10.1136/ejhpharm-2021-003013. PubMed PMID: 2021254668.

580. Abbassi A, Hasni N, Ben Hamida EB. Impact of computerized physician order entry system on parenteral nutrition medication errors in a teaching neonatal intensive care unit. Annales Pharmaceutiques Françaises. 2022;80(6):819-26. doi: https://doi.org/10.1016/j.pharma.2022.05.002.

581. Abbes M, Lo Presti C, Bambina E, Aghazarian V, Honoré S. Case report : contexte sanitaire Covid-19, un risque supplémentaire d'erreur médicamenteuse ? Le Pharmacien Clinicien. 2022;57(1):35-7. doi: https://doi.org/10.1016/j.phclin.2021.04.010.

582. Abbott A. Why we need a new guideline for preventing stroke associated with carotid arterial disease and why we need general medical practitioners to assist. Australian journal of primary health. 2020;26(4):iii-iv. doi: 10.1071/PYv26n4abs. PubMed PMID: CN-02179698.

583. Abbott AA, Fuji KT, Galt KA. A qualitative case study exploring nurse engagement with electronic health records and e-prescribing. Western Journal of Nursing Research. 2015;37(7):935-51.

584. Abbott R, Edwards S, Whelan M, Edwards J, Dranitsaris G. Are community pharmacists equipped to ensure the safe use of oral anticancer therapy in the community setting? Results of a cross-country survey of community pharmacists in Canada. Journal of Oncology Pharmacy Practice. 2014;20(1):29-39.

585. Abbott V, Creighton M, Hannam J, Vincent T, Coulter C. Access in Nnew Zealand to antidotes for accidental and intentional drug poisoni. Journal of Primary Health Care. 2012;4(2):100-5. doi: 10.1071/hc12100.

586. ABD EL RAHMAN A. Professional Development in Community Pharmacy Practice and Strategies for Implementation in Developing Countries: UOFK.

587. Abd Wahab MS. The relevance of educating doctors, pharmacists and older patients about potentially inappropriate medications. International Journal of Clinical Pharmacy. 2015;37(6):971-4. PubMed PMID: 26415737.

588. Abd Wahab MS, Sakthong P, Winit-Watjana W. Qualitative exploration of pharmacist care for herbal and dietary supplement users in Thai community pharmacies. Journal of Pharmaceutical Health Services Research. 2019;10(1):57-66. doi: 10.1111/jphs.12238. PubMed PMID: WOS:000458338900008.

589. Abd Wahab MSA, Jalani MM, Goh KW, Ming LC, Faller EM. Why Did I Consult My Pharmacist about Herbal and Dietary Supplements? An Online Survey Amid the COVID-19 Pandemic in Malaysia. International Journal of Environmental Research and Public Health. 2022;19(17). doi: 10.3390/ijerph191710994. PubMed PMID: WOS:000851879400001.

590. Abdallah O, Ageeb RA, Elkhalifa WHI, Zolezzi M, El-Awaisi A, Diab MI, et al. Evaluating prescribing competencies covered in a Canadian-accredited undergraduate pharmacy program in Qatar: a curriculum mapping process. Bmc Medical Education. 2020;20(1). doi: 10.1186/s12909-020-02109-1. PubMed PMID: WOS:000561111000001.

591. Abdallah S, Nizamuddin N. Blockchain-based solution for Pharma Supply Chain Industry. Computers & Industrial Engineering. 2023;177:108997. doi: https://doi.org/10.1016/j.cie.2023.108997.

592. Abdallah S, Nizamuddin N. Blockchain based solution for Pharma Supply Chain Industry. Computers &

Industrial Engineering. 2023:108997. doi: https://doi.org/10.1016/j.cie.2023.108997.

593. Abdallah W. Identifying Organizational Learning Dimensions that Promote Patient Safety Culture: A Study of Hospital Pharmacies in Kuwait [Ph.D.]. England: University of Bradford (United Kingdom); 2019.

594. Abdallah W, Johnson C, Nitzl C, Mohammed MA. Organizational learning and patient safety: hospital pharmacy settings. Journal of Health Organization and Management. 2019;33(6):695-713. doi: 10.1108/JHOM-11-2018-0319.

595. Abdallah W, Johnson C, Nitzl C, Mohammed MA. Arabic version of pharmacy survey on patient safety culture: Hospital pharmacy settings. SAGE Open Medicine. 2020;8. doi: 10.1177/2050312120951069.

596. Abdel Shaheed C, Maher CG, Mak W, Williams KA, McLachlan AJ. The effects of educational interventions on pharmacists' knowledge, attitudes and beliefs towards low back pain. International Journal of Clinical Pharmacy. 2015;37(4):616-25. doi: 10.1007/s11096-015-0112-5.

597. Abdel-Aziz SM, Abo Elsoud MM, Anise AAH. Chapter 2 - Microbial Biosynthesis: A Repertory of Vital Natural Products. In: Grumezescu AM, Holban AM, editors. Food Biosynthesis: Academic Press; 2017. p. 25-54.

598. Abdel-Qader DH, Al Meslamani AZ, Al Mazrouei N, El-Shara AA, El Sharu H, Merghani Ali E, et al. Virtual Coaching Delivered by Pharmacists to Prevent COVID-19 Transmission. Hospital Pharmacy. 2022;57(2):300-8. doi: 10.1177/00185787211032354.

599. Abdel-Qader DH, Al Meslamani AZ, Al Mazrouei N, Hamadi S, Ibrahim OM. The effect of educational interventions on medication dispensing errors: a randomised controlled trial in community pharmacies in Jordan. Drugs & Therapy Perspectives. 2021;37(8):383-91. doi: 10.1007/s40267-021-00846-x. PubMed PMID: WOS:000681660000001.

600. Abdel-Qader DH, Al Meslamani AZ, Al Mazrouei N, Hamadi S, Mohamed Ibrahim O. The effect of educational interventions on medication dispensing errors: a randomised controlled trial in community pharmacies in Jordan. Drugs & Therapy Perspectives. 2021;37(8):383-91. doi: 10.1007/s40267-021-00846-x. PubMed PMID: 151776929. Language: English. Entry Date: 20210811. Revision Date: 20210812. Publication Type: Article.

601. Abdel-Qader DH, Al Meslamani AZ, Albassam A, Al Mazrouei N, El-Shara AA, El Sharu H, et al. Assessment of A new Strategy to Prevent Prescribing Errors Involving COVID-19 Patients in Community Pharmacies. Hospital Pharmacy. 2022;57(5):654-65. doi: 10.1177/00185787211073506. PubMed PMID: 158865873. Language: English. Entry Date: 20220909. Revision Date: 20220909. Publication Type: Article.

602. Abdel-Qader DH, Harper L, Cantrill JA, Tully MP. Pharmacists' interventions in prescribing errors at hospital discharge: an observational study in the context of an electronic prescribing system in a UK teaching hospital. Drug safety. 2010;33:1027-44.

603. Abdel-Qader DH, Harper L, Cantrill JA, Tully MP, Abdel-Qader DH, Harper L, et al. Pharmacists' interventions in prescribing errors at hospital discharge: an observational study in the context of an electronic prescribing system in a UK teaching hospital. Drug Safety. 2010;33(11):1027-44. doi: 10.2165/11538310-000000000-00000. PubMed PMID: 104929023. Language: English. Entry Date: 20110204. Revision Date: 20211028. Publication Type: journal article.

604. Abdel-Qader DH, Hayajneh W, Albassam A, Obeidat NM, Belbeisi AM, Al Mazrouei N, et al. Pharmacists-physicians collaborative intervention to reduce vaccine hesitancy and resistance: a randomized controlled trial. Vaccine: x. 2022;10. doi: 10.1016/j.jvacx.2021.100135. PubMed PMID: CN-02357839.

605. Abdel-Qader DH, Saadi Ismael N, Al Meslamani AZ, Albassam A, El-Shara AA, Lewis PJ, et al. The Role of Clinical Pharmacy in Preventing Prescribing Errors in the Emergency Department of a Governmental Hospital in Jordan: A Pre-Post Study. Hospital Pharmacy. 2021;56(6):681-9. doi: 10.1177/0018578720942231. PubMed PMID: 153294027. Language: English. Entry Date: 20211109. Revision Date: 20211109. Publication Type: Article.

606. Abdel-Rahman SM, Ahlers N, Holmes A, Wright K, Harris A, Weigel J, et al. Validation of an improved pediatric weight estimation strategy. The Journal of Pediatric Pharmacology and Therapeutics. 2013;18(2):112-21.

607. Abdelaziz AI, Tawfik AG, Rabie KA, Omran M, Hussein M, Abou-Ali A, et al. Quality of community pharmacy practice in antibiotic self-medication encounters: a simulated patient study in upper Egypt. Antibiotics. 2019;8(2):35.

608. Abdelaziz AI, Tawfik AG, Rabie KA, Omran M, Hussein M, Abou-Ali A, et al. Quality of Community Pharmacy Practice in Antibiotic Self-Medication Encounters: A Simulated Patient Study in Upper Egypt. Antibiotics-Basel. 2019;8(2). doi: 10.3390/antibiotics8020035. PubMed PMID: WOS:000477005400002.

609. Abdelaziz AI, Tawfik AG, Rabie KA, Omran M, Hussein M, Abou-Ali A, et al. Quality of community pharmacy practice in antibiotic self-medication encounters: A simulated patient study in upper Egypt. Antibiotics. 2019;8(2). doi: 10.3390/antibiotics8020035.

610. Abdelaziz H, Al Anany R, Elmalik A, Saad M, Prabhu K, Al-Tamimi H, et al. Impact of clinical pharmacy services in a short stay unit of a hospital emergency department in Qatar. International Journal of Clinical Pharmacy. 2016;38(4):776-9. doi: 10.1007/s11096-016-0290-9. PubMed PMID: 116527012. Language: English. Entry Date: 20170512. Revision Date: 20180428. Publication Type: journal article.

611. Abdelaziz H, Richardson S, Walsh K, Nodzon J, Schwartz B. Evaluation of STAT medication ordering process in a community hospital. Pharmacy Practice. 2016;14(2). doi: 10.18549/PharmPract.2016.02.647.

612. Abdelaziz TA, Abdulraheem MA, Badi SA, Badawi MI, Saeed AO, Elobied MA, et al. Knowledge, attitude and practice of Sudanese pharmacist with regard to management of diabetes during Ramadan: A cross-sectional survey. Diabetes & Metabolic Syndrome: Clinical Research & Reviews. 2019;13(1):122-6. doi: https://doi.org/10.1016/j.dsx.2018.07.019.

613. Abdelhafiz AS, Fouda MA, El-Jaafary SI, Farghly MI, Salem M, Tammam A, et al. Targeting Future Customers: An Introductory Biobanking Course for Undergraduate Students of Life Sciences. Biopreservation and Biobanking. 2017;15(4):350-9. doi: 10.1089/bio.2016.0111. PubMed PMID: WOS:000407512500010.

614. Abdelhalim D, Mohundro BL, Evans JD. Role of student pharmacists in the identification and prevention of medication-related problems. Journal of the American Pharmacists Association. 2011;51(5):627-30. doi: https://doi.org/10.1331/JAPhA.2011.09101.

615. Abdelhamid E, Awad A, Gismallah A. Evaluation of a hospital pharmacy-based pharmaceutical care services for asthma patients. Pharmacy practice. 2008;6(1):25-32. PubMed PMID: CN-00707487.

616. Abdelkawy K, Kharouba M, Shendy K, Abdelmagged O, Galal N, Tarek M, et al. Prevalence of Drug-Drug Interactions in Primary Care Prescriptions in Egypt: A Cross-Sectional Retrospective Study. Pharmacy. 2023;11(3). doi: 10.3390/pharmacy11030106. PubMed PMID: WOS:001015597000001.

617. Abdelli N. Le cancer colorectal, du dépistage au diagnostic. Actualités Pharmaceutiques. 2023;62(625):20-4. doi: https://doi.org/10.1016/j.actpha.2023.02.011.

618. Abdelrahman Shehata ZH, Sabri NA, Elmelegy AA, Shehata ZHA. Descriptive analysis of medication errors reported to the Egyptian national online reporting system during six months. Journal of the American Medical Informatics Association. 2016;23(2):366-74. doi: 10.1093/jamia/ocv096. PubMed PMID: 113774246. Language: English. Entry Date: 20161110. Revision Date: 20180713. Publication Type: journal article.

619. Abdelsadig Mohammed H, Elamin SA, El-Awaisi A, El Hajj MS. Use of the job demands-resource model to understand community pharmacists' burnout during the COVID-19 pandemic. Research in Social and Administrative Pharmacy. 2022;18(9):3568-79. doi: https://doi.org/10.1016/j.sapharm.2022.03.011.

620. Abdelsalam Elshenawy R, Umaru N, Aslanpour Z. Five Rights of Antibiotic Safety: Antimicrobial Stewardship at One NHS Foundation Trust in England Before and During the COVID-19 Pandemic. International Journal of Pharmacy Practice. 2023;31(Supplement 2):ii2. PubMed PMID: 643188326.

621. Abdelshehid J, Guldner G. Errors in emergency department prescriptions resulting in pharmacy call backs. Annals of Emergency Medicine. 2004;44(4, Supplement):S10-S1. doi: https://doi.org/10.1016/j.annemergmed.2004.07.034.

622. Abdelwahab R, Hamad A, Al Okka R, Awahid M, Shibli KU, Saleem W, et al. Barriers and trends in cancer patients' pain management in Qatar: a randomized cohort prospective study. Supportive care in cancer. 2019;27(1):S46-S7. doi: 10.1007/s00520-019-04813-1. PubMed PMID: CN-01959980.

623. Abdelwahab R, Hamad A, Al Okka R, Wahid MA, Shibli KU, Saleem W, et al. Barriers and trends in cancer patients' pain management in Qatar: a randomized cohort prospective study. JNCCN journal of the national comprehensive cancer network. 2019;17(3-5). doi: 10.6004/jnccn.2018.7118. PubMed PMID: CN-01995451.

624. Abdi AM, Zarouri AT, Saloumi L, Basgut B. North Cyprus Pharmacist's Cognition and Practice of Pharmaceutical Care. Journal of Pharmaceutical Research International. 2018;21(3). doi: 10.9734/jpri/2018/39051. PubMed PMID: WOS:000454620500001.

625. Abdi S, Abbasinazari M, Valizadegan G, Kamarei M, Panahi Y, Sarafzadeh F, et al. Does the addition of melatonin to quadruple therapy increases the eradication rate of helicobacter pylori? A double-blind randomized clinical trial. Journal of clinical and diagnostic research. 2018;12(5):FC12-FC4. doi: 10.7860/JCDR/2018/35589.11543. PubMed PMID: CN-01610449.

626. Abdo MS, Hammad MA, Harun SN, Eusoff TM, Sheikh Ghadzi SM. Evaluation of knowledge, attitude and practice of healthcare providers towards life-threatening drug-drug interactions in Penang General Hospital, Malaysia. Clinical Epidemiology and Global Health. 2020;8(4):1253-8. doi: https://doi.org/10.1016/j.cegh.2020.04.023.

627. Abdolahi N, Kaheh E, Golsha R, Khodabakhshi B, Norouzi A, Khandashpoor M, et al. Letter to the editor: efficacy of different methods of combination regimen administrations including dexamethasone, intravenous immunoglobulin, and interferon-beta to treat critically ill COVID-19 patients: a structured summary of a study protocol for a randomized controlled trial. Trials. 2020;21(1):549. doi: 10.1186/s13063-020-04499-5. PubMed PMID: CN-02164605.

628. Abdu-Aguye SN, Labaran KS, Danjuma NM, Mohammed S. Hospital pharmacy outpatient medication dispensing and counselling practices in North-Western Nigeria: an observational study. International Journal of Pharmacy Practice. 2021;29(5):480-5. doi: 10.1093/ijpp/riab052. PubMed PMID: WOS:000736136400012.

629. Abdu-Aguye SN, Mohammed S, Danjuma NM, Labaran KS. Improving outpatient medication

counselling in hospital pharmacy settings: a behavioral analysis using the theoretical domains framework and behavior change wheel. Pharmacy Practice-Granada. 2021;19(2). doi: 10.18549/PharmPract.2021.2.2271. PubMed PMID: WOS:000669799600002.

630. Abdu-Aguye SN, Shehu A, Ahmad UI. Management of musculoskeletal pain in retail drug outlets within a Nigerian community: A descriptive study. Pharmacy Practice. 2017;15(1). doi: 10.18549/PharmPract.2017.01.873.

631. Abduelkarem AR. Towards optimising glycaemic control in patients with type 2 diabetes [Ph.D.]. England: University of Sunderland (United Kingdom); 2005.

632. Abduelkarem AR. Extending the role of pharmacists in patient care: are pharmacists in developing nations ready to change? Pharmacology & Pharmacy. 2014;2014.

633. Abduelkarem AR, Othman AM, Abuelkhair ZM, Ghazal MM, Alzouobi SB, El Zowalaty ME. Prevalence of self-medication with antibiotics among residents in United Arab Emirates. Infection and Drug Resistance. 2019;12:3445-53. doi: 10.2147/IDR.S224720.

634. Abduelkarem AR, Sackville M, Morgan RM, Hildreth AJ. An assessment of the level of type 2 diabetes patients' satisfaction with community pharmacists' services. Pharmaceutical Journal. 2003;270(7242):446-9.

635. Abduelkarem AR, Sackville MA. Changes of some health indicators in patients with type 2 diabetes: A prospective study in three community pharmacies in Sharjah, United Arab Emirates. Libyan Journal of Medicine. 2009;4(1):29-38. doi: 10.4176/080918.

636. Abduelkarem AR, Sackville MA, Morgan RM, Sackville MP, Hildreth AJ. Views and practices of community pharmacists regarding services for people with type 2 diabetes. International Journal of Pharmacy Practice. 2003;11(3):161-8. doi: 10.1211/0022357021990.

637. Abdul Aziz YH, Heydon SJ, Duffull SB, Marra CA. What free services do pharmacists offer? Investigating the provision of unfunded pharmacy services in community pharmacies. Research in Social and Administrative Pharmacy. 2021;17(3):588-94. doi: https://doi.org/10.1016/j.sapharm.2020.05.007.

638. Abdul Aziz YH, Heydon SJ, Duffull SB, Marra CA. What do pharmacy users think of free pharmacy services? Investigating pharmacy users' perceptions, attitudes and willingness to pay for free healthcare from pharmacies. Exploratory Research in Clinical and Social Pharmacy. 2023;11. doi: 10.1016/j.rcsop.2023.100288.

639. Abdul Halim Zaki I, Razali RM, Gnanasan S, Alias R, Karuppannan M. Medication discrepancies among elderly patients discharged from a tertiary hospital: prevalence and risk factors. Singapore Medical Journal. 2021;62(7):362-5. PubMed PMID: 34409484.

640. Abdul Kadir N, Wahab MSA, Mohd Suhaimi A, Othman N. Practices, barriers and facilitators to the delivery of pretravel health services by community pharmacists: A scoping review using the theoretical domains framework. Research in Social and Administrative Pharmacy. 2023;19(11):1412-23. doi: https://doi.org/10.1016/j.sapharm.2023.08.004.

641. Abdul Rahim AS. Mirror Mirror on the Wall: Escape a Remote Virtual Stereochemistry Lab Together. Journal of Chemical Education. 2022. doi: 10.1021/acs.jchemed.2c00050.

642. Abdul Rasool BK, Fahmy SA, Abu-Gharbieh EF, Ali HS. Professional practices and perception towards rational use of medicines according to WHO methodology in United Arab Emirates. Pharmacy Practice. 2010;8(1):70-6. doi: 10.4321/S1886-36552010000100009.

643. Abdul Salam M, Praveen D, Patel A, Tewari A, Webster R. Barriers to the Use of Cardiovascular Polypills In India: a Mixed-Methods Study. Global heart Conference: world congress of cardiology & cardiovascular health 2018 United arab emirates. 2018;13(4):418. doi: 10.1016/j.gheart.2018.09.150. PubMed PMID: CN-01670068.

644. Abdul-Latif M, Nagib R, El-Yazbi AF. Impact of a pharmacist-led intervention on glycemic control in diabetic patients in a setting with economic constraints. JACCP journal of the american college of clinical pharmacy. 2020;3(1):294-. doi: 10.1002/jac5.1204. PubMed PMID: CN-02120323.

645. Abdulah R, Barliana MI, Pradipta IS, Halimah E, Diantini A, Lestari K. Assessment of patient care indicators at community pharmacies in Bandung City, Indonesia. Southeast Asian Journal of Tropical Medicine and Public Health. 2014;45(5):1196.

646. Abdulah R, Khairinisa MA, Pratiwi AA, Barliana MI, Pradipta IS, Halimah E, et al. Off-label paediatric drug use in an Indonesian community setting. Journal of Clinical Pharmacy & Therapeutics. 2015;40(4):409-12. PubMed PMID: 25884353.

647. Abdulghani KH, Aseeri MA, Mahmoud A, Abulezz R. The impact of pharmacist-led medication reconciliation during admission at tertiary care hospital. International Journal of Clinical Pharmacy. 2018;40(1):196-201. doi: 10.1007/s11096-017-0568-6. PubMed PMID: 128333487. Language: English. Entry Date: 20180908. Revision Date: 20190201. Publication Type: journal article.

648. Abdulkadir SA, Wettermark B, Hammar T. Potential Drug-Related Problems in Pediatric Patients-Describing the Use of a Clinical Decision Support System at Pharmacies in Sweden. Pharmacy. 2023;11(1):35. doi: https://dx.doi.org/10.3390/pharmacy11010035. PubMed PMID: 2021751875.

649. Abdullah B, Ramli RR, Shukri NM, Mohamad S. Closing the knowledge gap in malaysian pharmacists: A virtual allergic rhinitis boot camp initiative. Multidisciplinary Respiratory Medicine. 2021;16. doi: 10.4081/MRM.2021.775.

650. Abdullah DC, Ibrahim NS, Ibrahim MIM. Medication errors among geriatrics at the outpatient pharmacy in a teaching hospital in Kelantan. Malaysian Journal of Medical Sciences. 2004;11(2):52-8. PubMed PMID: 41601102.

651. Abdullah MA. An Effective Design of the Rebate Calculation Process in a Rebate Management System Based on Data Warehousing [Ph.D.]. United States -- Colorado: Aspen University; 2018.

652. Abdullahi SU, Jibir BW, Bello-Manga H, Gambo S, Inuwa H, Tijjani AG, et al. Hydroxyurea for primary stroke prevention in children with sickle cell anaemia in Nigeria (SPRING): a double-blind, multicentre, randomised, phase 3 trial. The Lancet Haematology. 2022;9(1):e26-e37. doi: https://doi.org/10.1016/S2352-3026(21)00368-9.

653. Abdullahi SU, Jibir BW, Bello-Manga H, Gambo S, Inuwa H, Tijjani AG, et al. Hydroxyurea for primary stroke prevention in children with sickle cell anaemia in Nigeria (SPRING): a double-blind, multicentre, randomised, phase 3 trial. The Lancet Haematology. 2022;9(1):e26-e37. doi: 10.1016/S2352-3026(21)00368-9. PubMed PMID: CN-02361079.

654. Abdulraheem IS, Adegboye A, Fatiregun AA. Self-medication with Antibiotics: Empirical Evidence from a Nigerian Rural Population. British Journal of Pharmaceutical Research. 2016;11(5). doi: 10.9734/bjpr/2016/25268. PubMed PMID: WOS:000376276500001.

655. Abdulrasool Z. The development of a pharmacovigilance system in Bahrain. Saudi Pharmaceutical Journal. 2022;30(6):825-41. doi: https://doi.org/10.1016/j.jsps.2022.03.009.

656. Abdulrhim S, Sankaralingam S, Ibrahim MIM, Awaisu A. The impact of pharmacist care on diabetes outcomes in primary care settings: An umbrella review of published systematic reviews. Primary Care Diabetes. 2020;14(5):393-400. doi: 10.1016/j.pcd.2019.12.007.

657. Abdulsalim S, Farooqui M, Alshammari MS, Alotaibi M, Alhazmi A, Alqasomi A, et al. Evaluation of Knowledge, Attitudes, and Practices about Pharmacovigilance among Community Pharmacists in Qassim, Saudi Arabia. International Journal of Environmental Research and Public Health. 2023;20(4). doi: 10.3390/ijerph20043548.

658. Abdulsalim S, Unnikrishnan MK, Manu MK, Alrasheedy AA, Godman B, Morisky DE. Authors' response to the comments to "Structured pharmacist-led intervention programme to improve medication adherence in COPD patients: a randomized controlled study". Research in social & administrative pharmacy : RSAP. 2018;14(4):402-3. doi: 10.1016/j.sapharm.2017.12.008. PubMed PMID: CN-01967573.

659. Abdulsalim S, Unnikrishnan MK, Manu MK, Alrasheedy AA, Godman B, Morisky DE. Structured pharmacist-led intervention programme to improve medication adherence in COPD patients: a randomized controlled study. Research in social & administrative pharmacy : RSAP. 2018;14(10):909-14. doi: 10.1016/j.sapharm.2017.10.008. PubMed PMID: CN-01445591.

660. Abdulsalim S, Unnikrishnan MK, Manu MK, Alsahali S, Alrasheedy AA, Martin AP, et al. Impact of a Clinical Pharmacist Intervention on Medicine Costs in Patients with Chronic Obstructive Pulmonary Disease in India. PharmacoEconomics - open. 2020;4(2):331-42. doi: 10.1007/s41669-019-0172-x. PubMed PMID: CN-02130671.

661. Abdulwahab AA, Shomuyiwa DO, Augustine NC, Chinaza AM, Abdulwasiu MA, Chinecherem AE, et al. Challenges facing pharmaceutical care provision in Nigerian tertiary hospitals: A short review. Aims Medical Science. 2022;9(4):486-95. doi: 10.3934/medsci.2022026. PubMed PMID: WOS:000879555400001.

662. Abe M, Iwasa M, Sugimoto H, Watanabe M, Uchium F, Suzuki Y. The cooperation between pharmacists and nurses in outpatient cancer chemotherapy. Gan to kagaku ryoho Cancer & chemotherapy. 2006;33 Suppl 2:312-4.

663. Abe N, Kakamu T, Kumagai T, Hidaka T, Masuishi Y, Endo S, et al. Polypharmacy at admission prolongs length of hospitalization in gastrointestinal surgery patients. Geriatrics & Gerontology International. 2020;20(11):1085-90. doi: 10.1111/ggi.14044. PubMed PMID: WOS:000571713000001.

664. Abebe BT, Weiss M, Modess C, Roustom T, Tadken T, Wegner D, et al. Effects of the P-Glycoprotein Inhibitor Clarithromycin on the Pharmacokinetics of Intravenous and Oral Trospium Chloride: a 4-Way Crossover Drug-Drug Interaction Study in Healthy Subjects. Journal of clinical pharmacology. 2019;59(10):1319-30. doi: 10.1002/jcph.1421. PubMed PMID: CN-02417390.

665. Abebe E, Bao A, Kokkinias P, Russ-Jara AL, Degnan D. Maximizing student potential: Lessons for pharmacy programs from the patient safety movement. Exploratory Research in Clinical and Social Pharmacy. 2023;9:100216. doi: https://doi.org/10.1016/j.rcsop.2022.100216.

666. Abebe E, Bao A, Kokkinias P, Russ-Jara AL, Degnan D. Maximizing student potential: Lessons for pharmacy programs from the patient safety movement. Exploratory Research in Clinical and Social Pharmacy. 2023;9:100216. doi: https://doi.org/10.1016/j.rcsop.2022.100216.

667. Abebe E, Stone JA, Lester CA, Chui MA. Quality of Handoffs in Community Pharmacies. Journal of Patient Safety. 2021;17(6):405-11. doi: 10.1097/PTS.0000000000000382.

668. Abebe K, Beressa TB, Yimer BT. In-vitro evaluations of quality control parameters of paracetamol tablets marketed in Gondar City, Northwest Ethiopia. Drug, Healthcare and Patient Safety. 2020;12:273-9. doi: 10.2147/DHPS.S282420.

669. Abebe S, Ketema G, Kassahun H. In vitro comparative quality assessment of different brands of furosemide tablets marketed in northwest Ethiopia. Drug Design, Development and Therapy. 2020;14:5119-28. doi: 10.2147/DDDT.S280203.

670. Abebe TB, Erku DA, Gebresillassie BM, Haile KT, Mekuria AB. Expectation and satisfaction of HIV/ AIDS patients toward the pharmaceutical care provided at Gondar university referral hospital, northwestern Ethiopia: A cross-sectional study. Patient Preference and Adherence. 2016;10:2073-82. doi: 10.2147/ PPA.S114720.

671. Abedi G, Mohammadi A, Mohammadi F, Alizadeh A, Hosseini H, Rostami F. University students' personality profile based on casta & MaCrea five factor theory. International Journal of Collaborative Research on Internal Medicine and Public Health. 2012;4(6):1330-6.

672. Abedi M, Jafari F, Saber SSE, Fesharaki MG, Montazeri A. Effectiveness of Hefz-Al-Sehah on the number of visits to health care centers. Pakistan journal of medical and health sciences. 2021;15(3):1088-92. PubMed PMID: CN-02275029.

673. Abegaz TM, Belachew SA, Abebe TB, Gebresilassie BM, Teni FS, Woldie HG. Management of children’s acute diarrhea by community pharmacies in five towns of Ethiopia: Simulated client case study. Therapeutics and Clinical Risk Management. 2016;12:515-26. doi: 10.2147/TCRM.S98474.

674. Abegaz TM, Shegena EA, Gessie NF, Gebreyohanns EA, Seid MA. Barriers to and competency with the use of metered dose inhaler and its impact on disease control among adult asthmatic patients in Ethiopia. Bmc Pulmonary Medicine. 2020;20(1). doi: 10.1186/s12890-020-1081-6. PubMed PMID: WOS:000517543500001.

675. Abel C, Goldsmith CW, Marzerka M. Pharmacists can improve medication management in the vulnerable population of foster care youth. Journal of the American Pharmacists Association: JAPhA. 2019;59(5):629-32. PubMed PMID: 31255522.

676. Aberese-Ako M, Magnussen P, Gyapong M, Ampofo GD, Tagbor H. Managing intermittent preventive treatment of malaria in pregnancy challenges: An ethnographic study of two Ghanaian administrative regions. Malaria Journal. 2020;19(1). doi: 10.1186/s12936-020-03422-2.

677. Abeyaratne C, Bell JS, Dean L, White P, Maher-Sturgess S. Engaging older people as university-based instructors: A model to improve the empathy and attitudes of pharmacists in training. Currents in Pharmacy Teaching and Learning. 2020;12(1):58-64. doi: https://doi.org/10.1016/j.cptl.2019.10.011.

678. Abhinaya N, Thunga G, Muddukrishna BS, Pai R, Ravindra Shenoy U, Khan S, et al. A research on effective management of manufacturing defects to avoid product recalls: A challenge to pharmaceutical industry. Research Journal of Pharmacy and Technology. 2019;12(12):6124-32. doi: 10.5958/0974-360X.2019.01064.3.

679. Abili OD, Spevak C, Hodgson CDRJA. Safe Opioid Prescribing Project. The Journal of Pain. 2021;22(5):600. doi: https://doi.org/10.1016/j.jpain.2021.03.089.

680. Abili OD, Spevak C, Hodgson CDRJA. Opioid Safety: Complex Pain Case Conferences. The Journal of Pain. 2021;22(5):609. doi: https://doi.org/10.1016/j.jpain.2021.03.122.

681. Abili OD, Warusha S, Giunta R, Gobourne P, Brutcher R, Hodgson J, et al. Naloxone Education: Patient Perspectives. The Journal of Pain. 2021;22(5):600. doi: https://doi.org/10.1016/j.jpain.2021.03.090.

682. Abimbola S, Ukwaja KN, Onyedum CC, Negin J, Jan S, Martiniuk ALC. Transaction costs of access to health care: Implications of the care-seeking pathways of tuberculosis patients for health system governance in Nigeria. Global Public Health. 2015;10(9):1060-77. doi: 10.1080/17441692.2015.1007470.

683. Abla KK, Mehanna MM. Lipid-based nanocarriers challenging the ocular biological barriers: Current paradigm and future perspectives. Journal of Controlled Release. 2023;362:70-96. doi: 10.1016/j.jconrel.2023.08.018. PubMed PMID: 172871218.

684. Ables AZ, Baughman OL, 3rd. The clinical pharmacist as a preceptor in a family practice residency training program. Family Medicine. 2002;34(9):658-62. PubMed PMID: 12455249.

685. Abolhassanzadeh Z, Ansari S, Lorigooini Z, Anjomshoa M, Bijad E, Ramezannezhad P, et al. The nephrotoxicity of Aristolochia rotunda L. in rats: Mitochondrion as a target for renal toxicity of Aristolochic acids-containing plants. Heliyon. 2023;9(11):e21848. doi: https://doi.org/10.1016/j.heliyon.2023.e21848.

686. Aboneh EA, Look KA, Stone JA, Lester CA, Chui MA. Psychometric properties of the AHRQ Community Pharmacy Survey on Patient Safety Culture: a factor analysis. BMJ Quality & Safety. 2016;25(5):355-63. PubMed PMID: 26208535.

687. Aboneh EA, Stone JA, Lester CA, Chui MA. Evaluation of Patient Safety Culture in Community Pharmacies. Journal of patient safety. 2020;16(1):e18-e24. PubMed PMID: 29112024.

688. Abood EA, Scott J, Wazaify M. User Experiences of Prescription and Over-The-Counter Drug Abuse in Aden City, Yemen. Pharmacy. 2018;6(3). doi: 10.3390/pharmacy6030099. PubMed PMID: WOS:000448399800045.

689. Abood EA, Wazaify M. Abuse and Misuse of Prescription and Nonprescription Drugs from Community Pharmacies in Aden City—Yemen. Substance Use and Misuse. 2016;51(7):942-7. doi: 10.3109/10826084.2016.1155619.

690. Abood EA, Wazaify M. Abuse and Misuse of Prescription and Nonprescription Drugs from Community Pharmacies in Aden City-Yemen. Substance Use & Misuse. 2016;51(7):942-7. doi: 10.3109/10826084.2016.11556199. PubMed PMID: WOS:000376163500017.

691. Abood SJ, Abdulsahib WK, Al-Radeef MY. Prevalence of home storage of medicines and associated factors in iraq. Open Access Macedonian Journal of Medical Sciences. 2021;9(E):356-63. doi: 10.3889/oamjms.2021.5997.

692. Abookire SA, Teich JM, Sandige H, Paterno MD, Martin MT, Kuperman GJ, et al., editors. Improving allergy alerting in a computerized physician order entry system. Proceedings of the AMIA Symposium; 2000: American Medical Informatics Association.

693. Aborigo RA, Allotey P, Reidpath DD. The traditional healer in obstetric care: A persistent wasted opportunity in maternal health. Social Science & Medicine. 2015;133:59-66. doi: 10.1016/j.socscimed.2015.03.046. PubMed PMID: WOS:000354579800009.

694. Abosi OJ, Mascardo L, Marra AR, Diekema DJ, Edmond MB, Salinas JL. Determining the Risk of Septic Arthritis Following Intra-Articular Injection of Drugs Mixed At A Tertiary Care Center, 2012–2017. American Journal of Infection Control. 2019;47(6, Supplement):S49-S50. doi: https://doi.org/10.1016/j.ajic.2019.04.125.

695. Abou-Auda HS. An economic assessment of the extent of medication use and wastage among families in Saudi Arabia and Arabian Gulf countries. Clinical Therapeutics. 2003;25(4):1276-92. doi: 10.1016/s0149-2918(03)80083-8. PubMed PMID: WOS:000182628200018.

696. Abouassaly M. The student pharmacist compounding competition: creating problem solvers. International journal of pharmaceutical compounding. 2012;16(3):206-9.

697. Abouchouar I, Hindlet P, Ratsimbazafy C, Fernandez C, Schwab C. Medication reconciliation and hospital-community transition securisation: Survey of community pharmacists. Annales Pharmaceutiques Francaises. 2023;81(5):875-81. doi: https://dx.doi.org/10.1016/j.pharma.2023.01.005. PubMed PMID: 2022937143.

698. Abouchouar I, Hindlet P, Ratsimbazafy C, Fernandez C, Schwab C. Conciliation médicamenteuse et sécurisation de la transition hôpital–ville : enquête auprès des pharmaciens d'officine. Annales Pharmaceutiques Françaises. 2023;81(5):875-81. doi: https://doi.org/10.1016/j.pharma.2023.01.005.

699. Abouk R, Heydari B. The Immediate Effect of COVID-19 Policies on Social-Distancing Behavior in the United States. Public Health Reports. 2021;136(2):245-52. doi: 10.1177/0033354920976575.

700. Abouk R, Pacula RL, Powell D. Association between State Laws Facilitating Pharmacy Distribution of Naloxone and Risk of Fatal Overdose. JAMA Internal Medicine. 2019;179(6):805-11. doi: 10.1001/jamainternmed.2019.0272.

701. Aboukrat P, Maitre M, Hérisson C, Laffont I. Orthopedic shoes customized or not: Different kinds, indications and orders. Revue du Rhumatisme Monographies. 2014;81(3):209-14. doi: 10.1016/j.monrhu.2014.04.012.

702. Aboulatta L, Haidar L, Abou-Setta A, Askin N, Rabbani R, Lavu A, et al. Efficacy and Safety of MAO-B Inhibitors Safinamide and Zonisamide in Parkinson's Disease: A Systematic Review and Meta-Analysis of Randomized Controlled Trials. CNS Drugs. 2023;37(11):941-56. doi: 10.1007/s40263-023-01048-x. PubMed PMID: 173805769.

703. Aboumatar HJ, Winner L, Davis R, Peterson A, Hill R, Frank S, et al. Applying Lean Sigma solutions to mistake-proof the chemotherapy preparation process. Joint Commission Journal on Quality & Patient Safety. 2010;36(2):79-86. PubMed PMID: 105307641. Language: English. Entry Date: 20100319. Revision Date: 20150711. Publication Type: Journal Article.

704. Aboumatar HJ, Winner LE, Davis RO, Trovitch PB, Berg MM, Violette KM, et al. No time to waste: Decreasing patient wait times for chemotherapy administration using automated prioritization in an oncology pharmacy system. American Journal of Managed Care. 2008;14(5):309-16. PubMed PMID: WOS:000255965900006.

705. Abousheishaa AA, Sulaiman AH, Huri HZ, Zaini S, Othman NA, Aladdin ZB, et al. Global scope of hospital pharmacy practice: a scoping review. Healthcare (Switzerland). 2020;8(2). doi: 10.3390/healthcare8020143.

706. Abousheishaa AA, Sulaiman AH, Huri HZ, Zaini S, Othman NA, bin Aladdin Z, et al. Global Scope of Hospital Pharmacy Practice: A Scoping Review. Healthcare. 2020;8(2). doi: 10.3390/healthcare8020143. PubMed PMID: WOS:000548056900018.

707. Abraham J. Science, politics and the pharmaceutical industry: Controversy and bias in drug regulation: Routledge; 2023.

708. Abraham J, Kannampallil TG, Jarman A, Sharma S, Rash G, Schiff G, et al. Reasons for computerised provider order entry (CPOE)-based inpatient medication ordering errors: an observational study of voided orders. BMJ Quality & Safety. 2018;27(4):299-307. doi: 10.1136/bmjqs-2017-006606. PubMed PMID: 128652340. Language: English. Entry Date: 20180330. Revision Date: 20180330. Publication Type: Article.

709. Abraham J, Nguyen MAY, Zijlstra EMMA, Akula M, Traeger LEAH, Arora V, et al. USE OF TELEHEALTH VISITS TO ADDRESS GAPS IN DISCHARGE CARE TRANSITIONS OF PATIENTS WITH CHRONIC OBSTRUCTIVE PULMONARY DISEASE. Chest. 2023;164(4 Supplement):A4926-A7. doi: https://dx.doi.org/10.1016/j.chest.2023.07.3193. PubMed PMID: 2027228280.

710. Abraham K, Franken M. A SWOT analysis of the complex interdependencies of the Maltese reimbursement processes. Health Policy OPEN. 2023;4:100095. doi: https://doi.org/10.1016/j.hpopen.2023.100095.

711. Abraham NS, Cohen DC, Rivers B, Richardson P. Validation of administrative data used for the diagnosis of upper gastrointestinal events following nonsteroidal anti-inflammatory drug prescription. Alimentary pharmacology & therapeutics. 2006;24(2):299-306. doi: 10.1111/j.1365-2036.2006.02985.x. PubMed PMID: CN-01764479.

712. Abraham NS, Hartman C, Castillo D, Richardson P, Smalley W. Effectiveness of national provider prescription of PPI gastroprotection among elderly NSAID users. American Journal of Gastroenterology. 2008;103(2):323-32. doi: 10.1111/j.1572-0241.2007.01595.x. PubMed PMID: WOS:000253049700011.

713. Abraham NS, Hartman C, Hasche J. Reduced Hospitalization Cost for Upper Gastrointestinal Events That Occur Among Elderly Veterans Who Are Gastroprotected. Clinical Gastroenterology and Hepatology. 2010;8(4):350-6. doi: https://doi.org/10.1016/j.cgh.2010.01.002.

714. Abraham O, Alexander DS, Schleiden LJ, Carpenter DM. Identifying barriers and facilitators that affect community pharmacists' ability to engage children in medication counseling: A pilot study. Journal of Pediatric Pharmacology and Therapeutics. 2017;22(6):412-22. doi: 10.5863/1551-6776-22.6.412.

715. Abraham O, Brothers A, Alexander DS, Carpenter DM. Pediatric medication use experiences and patient counseling in community pharmacies: perspectives of children and parents. Journal of the American Pharmacists Association. 2017;57(1):38-46. e2.

716. Abraham O, Brothers A, Alexander DS, Carpenter DM, editors. Pediatric medication use experiences and patient counseling in community pharmacies: Perspectives of children and parents. Journal of the American Pharmacists Association; 2017.

717. Abraham O, Buechel M, Gay S, Szela L, Decker CA, Braun AT. Using A Work System Framework to Investigate Pharmacists' Roles in Cystic Fibrosis Management. Journal of Pediatric Pharmacology and Therapeutics. 2022;27(3):270-6. doi: 10.5863/1551-6776-27.3.270.

718. Abraham O, Feathers A, Mook H, Korenoski A. The perceived benefits of student pharmacists educating children about over-the-counter medication safety. Currents in Pharmacy Teaching and Learning. 2019;11(2):184-91. doi: https://doi.org/10.1016/j.cptl.2018.11.005.

719. Abraham O, LeMay S, Nixon G, Braun A, Decker C, Szela L. Characterizing medication management and the role of pharmacists in caring for people living with cystic fibrosis: A work system approach. Journal of the American Pharmacists Association. 2021;61(5):547-54.e2. doi: https://doi.org/10.1016/j.japh.2021.04.006.

720. Abraham O, Li JS, Monangai KE, Feathers AM, Weiner D. The pharmacist's role in supporting people

living with cystic fibrosis. Journal of the American Pharmacists Association. 2018;58(3):246-9. PubMed PMID: 2000600822.

721. Abraham O, McCarthy TJ, Zaborek J. Assessing the Impact of a Serious Game (MedSMARxT: Adventures in PharmaCity) in Improving Opioid Safety Awareness Among Adolescents and Parents: Quantitative Study. JMIR Formative Research. 2023;7:e51812. PubMed PMID: 38060287.

722. Abraham O, Morris A. Opportunities for outpatient pharmacy services for patients with cystic fibrosis: Perceptions of healthcare team members. Pharmacy. 2019;7(2) (no pagination). PubMed PMID: 627943130.

723. Abraham O, Myers MN, Brothers AL, Montgomery J, Norman BA, Fabian T. Assessing need for pharmacist involvement to improve care coordination for patients on LAI antipsychotics transitioning from hospital to home: A work system approach. Research in Social and Administrative Pharmacy. 2017;13(5):1004-13. doi: https://doi.org/10.1016/j.sapharm.2017.02.006.

724. Abraham O, Peters J, Peterson KA. Characterizing Pharmacist Perspectives on Utilizing a Personalized Family Medication Safety Plan for Opioid Education with Adolescents and Parents. Pharmacy. 2023;11(1):22. doi: https://dx.doi.org/10.3390/pharmacy11010022. PubMed PMID: 2021751893.

725. Abraham O, Peterson KA, Thao SS, McCarthy TJ. Adolescents' assessment of a Family Medication Safety Plan for opioid prescriptions: A qualitative usability study. Journal of the American Pharmacists Association. 2023;63(6):1768-75. e2.

726. Abraham O, Rosenberger CA, Poku VO. Implementing a youth advisory board to inform adolescent health and medication safety research. Research in Social and Administrative Pharmacy. 2022. doi: https://doi.org/10.1016/j.sapharm.2022.12.003.

727. Abraham O, Rosenberger CA, Poku VO. Implementing a youth advisory board to inform adolescent health and medication safety research. Research in social & administrative pharmacy : RSAP. 2023;19(4):681-5. doi: https://dx.doi.org/10.1016/j.sapharm.2022.12.003. PubMed PMID: 639811226.

728. Abraham O, Schleiden LJ, Brothers AL, Albert SM. Managing sleep problems using non-prescription medications and the role of community pharmacists: older adults' perspectives. International Journal of Pharmacy Practice. 2017;25(6):438-46.

729. Abraham O, Schleiden LJ, Chui MA. Errors related to outpatient e-prescribing. Safety of Health IT: Clinical Case Studies2016. p. 69-79.

730. Abraham O, Slonac E, Paulsen Z. Pharmacists' perspectives on MedSMAT: A serious game to educate youth about opioid safety. Journal of the American Pharmacists Association. 2023;63(4):1087-94.e1. doi: https://dx.doi.org/10.1016/j.japh.2023.04.020. PubMed PMID: 2024720669.

731. Abraham O, Slonac E, Paulsen Z. Pharmacists' Perspectives on MedSMAR T: A serious game to educate youth about opioid safety. Journal of the American Pharmacists Association. 2023;63(4):1087-94. e1.

732. Abraham O, Wytiaz RM, Feathers AM. Paediatric use of medications and adherence apps: a qualitative analysis of the perspectives of children and parents. Journal of Pharmacy Practice and Research. 2019;49(2):123-9. PubMed PMID: 624023646.

733. Abraham TG, Huggins C, Diaz-Fuentes G, Roglieri J. Combining pharmacy expertise with asthma educator certification: assessing the impact on inner-city asthma patients. Journal of Asthma. 2019;56(8):891-6. doi: 10.1080/02770903.2018.1501697.

734. Abrahamsen B, Burghle AH, Rossing C. Pharmaceutical care services available in Danish community pharmacies. International Journal of Clinical Pharmacy. 2020;42(2):315-20. doi: 10.1007/s11096-020-00985-7. PubMed PMID: 142999495. Language: English. Entry Date: 20210207. Revision Date: 20210921. Publication Type: journal article.

735. Abrahamsen B, Hansen RN, Bjorn-Christensen M, Druskeit T, Rossing C. Using Real-Life Data to Strengthen the Education of Pharmacy Technician Students: From Student to Research Assistant. Pharmacy. 2020;8(2). doi: 10.3390/pharmacy8020062. PubMed PMID: WOS:000552756500048.

736. Abrahamsen B, Hansen RN, Rossing C. For which patient subgroups are there positive outcomes from a medication review? A systematic review. Pharmacy Practice. 2020;18(4):1976. PubMed PMID: 33224322.

737. Abrahamsen B, Hansen RN, Skjott M, Sloth-Lisbjerg D, Rossing CV. Mapping of Danish Pharmacy Technician Students' Third-Year Projects in a Year with the COVID-19 Pandemic. Pharmacy. 2022;10(1). doi: 10.3390/pharmacy10010033. PubMed PMID: WOS:000765128900001.

738. Abrahamsen B, Rossing C, Hansen RN. Facilitating discontinuation of potentially inappropriate medication for the elderly with community pharmacy-led medication reviews. International Journal of Clinical Pharmacy. 2020;42(2):823-. PubMed PMID: WOS:000529692500086.

739. Abramochkin DV, Pustovit OB, Mironov NY, Filatova TS, Kuzmin VS. The Dependence of the Electrophysiological Effects of Class III Antiarrhythmic Drug Refralon on the Frequency of Myocardium Activation. Bulletin of Experimental Biology & Medicine. 2023;174(5):610-5. doi: 10.1007/s10517-023-05756-8. PubMed PMID: 163295209.

740. Abramowitz PW, Maroyka EM, Scheckelhoff DJ. Achieving the PAI 2030 vision: Leading and navigating transformative change. American Journal of Health-System Pharmacy. 2020;77(2):66-7. PubMed PMID: 31786598.

741. Abrams JA, Rutledge J, Opara I. Learning from community-based HIV prevention to inform control and mitigation of the COVID-19 pandemic. Preventive Medicine. 2023;169. doi: 10.1016/j.ypmed.2023.107445.

742. Abramson BL, Black DR, Christakis MK, Fortier M, Wolfman W. Guideline No. 422e: Menopause and Cardiovascular Disease. Journal of Obstetrics and Gynaecology Canada. 2021;43(12):1438-43.e1. doi: https://doi.org/10.1016/j.jogc.2021.09.010.

743. Abramson EL. Causes and consequences of e-prescribing errors in community pharmacies. Integrated Pharmacy Research and Practice. 2015;4:31-8. doi: 10.2147/iprp.S64927. PubMed PMID: WOS:000214676100004.

744. Abramson EL, Barrón Y, Quaresimo J, Kaushal R. Electronic prescribing within an electronic health record reduces ambulatory prescribing errors. The Joint Commission Journal on Quality and Patient Safety. 2011;37(10):470-8.

745. Abramson EL, Bates DW, Jenter C, Volk LA, Barrón Y, Quaresimo J, et al. Ambulatory prescribing errors among community-based providers in two states. Journal of the American Medical Informatics Association. 2012;19(4):644-8.

746. Abramson EL, Kaushal R. Computerized provider order entry and patient safety. Pediatric Clinics. 2012;59(6):1247-55.

747. Abramson EL, Malhotra S, Fischer K, Edwards A, Pfoh ER, Osorio SN, et al. Transitioning between electronic health records: effects on ambulatory prescribing safety. Journal of General Internal Medicine.

2011;26:868-74.

748. Abramson EL, Malhotra S, Osorio SN, Edwards A, Cheriff A, Cole C, et al. A long-term follow-up evaluation of electronic health record prescribing safety. Journal of the American Medical Informatics Association. 2013;20(e1):e52-e8.

749. Abramson EL, Patel V, Malhotra S, Pfoh ER, Osorio SN, Cheriff A, et al. Physician experiences transitioning between an older versus newer electronic health record for electronic prescribing. International journal of medical informatics. 2012;81(8):539-48.

750. Abramson EL, Patel V, Pfoh ER, Kaushal R. How Physician Perspectives on E-Prescribing Evolve over Time A Case Study Following the Transition between EHRs in an Out-patient Clinic. Applied Clinical Informatics. 2016;7(4):994-1006. doi: 10.4338/aci-2016-04-ra-0069. PubMed PMID: WOS:000391958400007.

751. Abramson ZH, Avni O, Levi O, Miskin IN. Randomized trial of a program to increase staff influenza vaccination in primary care clinics. Annals of family medicine. 2010;8(4):293-8. doi: 10.1370/afm.1132. PubMed PMID: CN-00768774.

752. Abramsson L, Gustafsson M. Prevalence of drug-related problems using STOPP/START and medication reviews in elderly patients with dementia. Research in Social & Administrative Pharmacy. 2020;16(3):308-14. doi: 10.1016/j.sapharm.2019.05.016. PubMed PMID: 142186570. Language: English. Entry Date: 20211108. Revision Date: 20211230. Publication Type: journal article.

753. Abreu M, Castel-Branco MM, Fernandez-Llimos F, Caramona MM, Figueiredo IV. Medication follow-up at community pharmacy to improve cardiovascular risk. International journal of clinical pharmacy. 2013;35(6):1281-. doi: 10.1007/s11096-013-9886-5. PubMed PMID: CN-01011806.

754. Abreu RM, Hori PCA, Rocha PA, Minari AB, Dias MA, Pinto VB, et al. Text message reminders increase antiviral therapy adherence in chronic hepatitis b patients. European journal of hospital pharmacy. 2016;23:A49-A50. doi: 10.1136/ejhpharm-2016-000875.113. PubMed PMID: CN-01334243.

755. Abrogoua DP, Koffi NO, Doffou E. Interventions pharmaceutiques sur les prescriptions d'antibiotiques en consultations externes de pédiatrie d'un centre hospitalier universitaire de Côte d'Ivoire. Annales Pharmaceutiques Françaises. 2016;74(5):380-8. doi: https://doi.org/10.1016/j.pharma.2015.12.004.

756. Abrogoua DP, N'Gbe Jean V, Allah Kouadio E, Doffou E. Incidence and factors related to neuropsychiatry side effects of interferon alpha in hepatitis B and C infected patients in Cote D'Ivoire. European Journal of Clinical Pharmacy. 2017;19(1):24-32.

757. Abrogoua DP, Sangaré AY, Doffou E, Aulagner G. Évaluation de la sécurisation de la dispensation médicamenteuse dans trois centres hospitaliers universitaires d'Abidjan (Côte d'Ivoire). Le Pharmacien Hospitalier et Clinicien. 2016;51(2):123-33. doi: https://doi.org/10.1016/j.phclin.2015.09.009.

758. Abrons J, Vadala T, Miller S, Cerulli J. Encouraging safe medication disposal through student pharmacist intervention. Journal of the American Pharmacists Association. 2010;50(2):169-73. doi: https://doi.org/10.1331/JAPhA.2010.09208.

759. Abrons JP, Jonkman LJ, Nonyel NP, Connor SE, Ombengi DN, Kahaleh AA. An ethics-based approach to global health research part 4: Scholarship and publications. Research in Social and Administrative Pharmacy. 2020;16(11):1597-601. doi: https://doi.org/10.1016/j.sapharm.2020.06.015.

760. Abrons JP, Smith M. Patient-centered medical homes: primer for pharmacists. Journal of the American

Pharmacists Association: JAPhA. 2011;51(3):e38-48; quiz e9-50. PubMed PMID: 21555279.

761. Absari DT, Djunaidy A, Susanto TD, editors. Design Science Research Methodology Concept and Its Application in the Development of Online Community Communication System for Selotapak Village. Proceedings - 2022 9th International Conference on Information Technology, Computer and Electrical Engineering, ICITACEE 2022; 2022.

762. Abstoss K, Shaw B, Owens T. Increasing medication error reporting rates while reducing harm through simultaneous cultural and system-level interventions in an intensive care unit.

763. Abstoss KM, Shaw BE, Owens TA, Juno JL, Commiskey EL, Niedner MF. Increasing medication error reporting rates while reducing harm through simultaneous cultural and system-level interventions in an intensive care unit. BMJ Quality & Safety. 2011;20(11):914-22. PubMed PMID: 104605000. Language: English. Entry Date: 20120127. Revision Date: 20150711. Publication Type: Journal Article.

764. Abtahi S, Pajouheshnia R, Durán CE, Riera-Arnau J, Gamba M, Alsina E, et al. Impact of 2018 EU risk minimisation measures and revised pregnancy prevention programme on utilisation and prescribing trends of medicinal products containing valproate: an interrupted time series study. Drug Safety. 2023;46(7):689-702.

765. Abu Al-Halawa D, Sarama R, Abdeen Z, Qasrawi R. Knowledge, attitudes, and practices relating to antibiotic resistance among pharmacists: a cross-sectional study in the West Bank, Palestine. The Lancet. 2019;393:S7. doi: https://doi.org/10.1016/S0140-6736(19)30593-8.

766. Abu Assab M, Jaber D, Albahar F, Alhamad H, Mukattash T, Mathani HA, et al. Willingness to pay for community pharmacies-based medication review service: perspectives of private health insurance firms. Pharmacy Practice-Granada. 2023;21(4). doi: 10.18549/PharmPract.2023.4.2863. PubMed PMID: WOS:001140765900001.

767. Abu Assab M, Jaber D, Basheer H, Abu Assab H, Al-Atram H. The COVID-19 Pandemic and Patient Safety Culture: A Cross-Sectional Study among Community Pharmacies in Jordan. Healthcare (Switzerland). 2022;10(8). doi: 10.3390/healthcare10081434.

768. Abu Bakar A, Ong SC, Ting CY, Ooi GS, Hassali MAA. Barriers for Implementation of E-pharmacy Policy: Views of Pharmacy Authorities, Public Institutions and Societal Groups. Pertanika Journal of Social Science and Humanities. 2022;30(1):41-56. doi: 10.47836/pjssh.30.1.03. PubMed PMID: WOS:000770275500003.

769. Abu El-Kass S, Ragheb MM, Hamed SM, Turkman AM, Zaki AT. Needs and Self-Care Efficacy for Cancer Patients Suffering from Side Effects of Chemotherapy. Journal of Oncology. 2021;2021. doi: 10.1155/2021/8880366. PubMed PMID: WOS:000664933900001.

770. Abu Esba LC, Yousef C, Ismail S, Halim M, Almodaimegh H, Al-Abdulkarim HA, et al. Fixed dose combinations: A formulary decision guide. Health Policy and Technology. 2021;10(2):100500. doi: https://doi.org/10.1016/j.hlpt.2021.02.006.

771. Abu Farha R, Abu Hammour K, Al-Jamei S, AlQudah R, Zawiah M. The prevalence and clinical seriousness of medication discrepancies identified upon hospital admission of pediatric patients. BMC Health Services Research. 2018;18(1):1-7. doi: 10.1186/s12913-018-3795-1. PubMed PMID: 133642524. Language: English. Entry Date: 20190405. Revision Date: 20190524. Publication Type: journal article.

772. Abu Farha R, Abu Hammour K, Alefishat E, Alsaeed H, Alma'aiah S. Drive-thru pharmacy service: Assessments of awareness, perception and barriers among pharmacists in Jordan. Saudi Pharmaceutical Journal. 2017;25(8):1231-6. doi: 10.1016/j.jsps.2017.09.008.

773. Abu Farha R, Abu Hammour K, Mukattash T, Alqudah R, Aljanabi R. Medication histories documentation at the community pharmacy setting: A study from Jordan. PLoS ONE [Electronic Resource]. 2019;14(10):e0224124. PubMed PMID: 31639171.

774. Abu Farha R, Alefishat E, Suyagh M, Elayeh E, Mayyas A. Evidence-based medicine use in pharmacy practice: A cross-sectional survey. Journal of Evaluation in Clinical Practice. 2014;20(6):786-92. doi: 10.1111/jep.12212.

775. Abu Farha R, Basheti I, Abu Al Ruz H, Alsaleh A, AbuRuz S. Assessment of drug-related problems and their impact on blood pressure control in patients with hypertension. European Journal of Hospital Pharmacy. 2016;23(3):126-30. doi: 10.1136/ejhpharm-2015-000712. PubMed PMID: WOS:000375092000002.

776. Abu Farha R, Gharaibeh L, Alzoubi KH, Alhamad H. Exploring Community Pharmacists' Perception and Readiness Toward Telepharmacy Implementation in Jordan: A Cross-Sectional Study. Telemedicine and E-Health. 2023. doi: 10.1089/tmj.2023.0264. PubMed PMID: WOS:001180223100001.

777. Abu Farha RK, Rashad M, Hasen E, Mukattash TL, Al-Hashar A, Basheti IA. Evaluation of the effect of video tutorial training on improving pharmacy students' knowledge and skills about medication reconciliation. Pharmacy Practice. 2020;18(1). doi: 10.18549/PharmPract.2020.1.1711.

778. Abu Hagar R, El-Dahiyat F, El Refae G. Risk management in community pharmacy practice in Abu Dhabi Region: a cross-sectional study. Journal of Pharmaceutical Health Services Research. 2020;11(3):275-85. doi: 10.1111/jphs.12364.

779. Abu Hajleh MN, Al-Samydai AM, Mare'i MN, Abd MM, Sibai OA, Mohammed AH, et al. The role of pharmacist in guiding the pharmacy clients towards pharmaceutical preparations use. Journal of Pharmaceutical Health Services Research. doi: 10.1093/jphsr/rmac055. PubMed PMID: WOS:000893871600001.

780. Abu Hajleh MN, Al-Samydai AM, Mare'I MN, Abd MM, Sibai OA, Mohammed AH, et al. The role of pharmacist in guiding the pharmacy clients towards pharmaceutical preparations use. Journal of Pharmaceutical Health Services Research. 2023;14(1):28-34. doi: 10.1093/jphsr/rmac055.

781. Abu Hammour K, Abu Farha R, Rizik M, Mukattash T, Alnan M, Alkhader A, et al. Pharmacy drive-thru service in Jordan: assessing customers' awareness, perceptions and factors affecting the use of this service. Journal of Pharmaceutical Health Services Research. 2019;10(1):141-7. doi: 10.1111/jphs.12245. PubMed PMID: WOS:000458338900017.

782. Abu Hammour K, Abu Farha R, Ya'acoub R, Salman Z, Basheti I. Impact of Pharmacist-Directed Medication Reconciliation in Reducing Medication Discrepancies: A Randomized Controlled Trial. Canadian Journal of Hospital Pharmacy. 2022;75(3):169-77. doi: 10.4212/cjhp.3143. PubMed PMID: 158648974. Language: English. Entry Date: 20220901. Revision Date: 20221228. Publication Type: Article.

783. Abu Mhadi A, Ong SC, Abed Y. Exploring medicines use patterns and practices among the public in the Gaza strip, Palestine: A qualitative study. Journal of Research in Pharmacy. 2023;27(1):469-80. doi: 10.29228/jrp.329.

784. Abu Shaqra QM, Shawaqfah MT, Al Momani W. Microbiological quality of blister pack tablets in community pharmacies in Jordan. Tropical Journal of Pharmaceutical Research. 2014;13(2):261-6. doi: 10.4314/tjpr.v13i2.15.

785. Abu-Dalbouh HM, Al-Matrouk M, Al-Zwaid N, Al-Handi A. Proposal of a standardized electronic health record for Kingdom of Saudi Arabia. Journal of Computer Science. 2019;15(4):566-81. doi: 10.3844/

jcssp.2019.566.581.

786. Abu-Farha R, Alzoubi KH, Alkhawaldeh R, Itani R, Karout S, Mukattash T, et al. Home delivery of medications: Community pharmacists' perspectives on the pros and cons of the service. Frontiers in Public Health. 2022;10. doi: 10.3389/fpubh.2022.966145.

787. Abu-Farha R, Alzoubi KH, Rizik M, Karout S, Itani R, Mukattash T, et al. Public Perceptions About Home Delivery of Medication Service and Factors Associated with the Utilization of This Service. Patient Preference and Adherence. 2022;16:2259-69. doi: 10.2147/PPA.S377558.

788. Abu-Gharbieh E, Khalidi DA, Baig MR, Khan SA. Refining knowledge, attitude and practice of evidence-based medicine (EBM) among pharmacy students for professional challenges. Saudi Pharmaceutical Journal. 2015;23(2):162-6. doi: https://doi.org/10.1016/j.jsps.2014.07.006.

789. Abu-Janb N, Jaana M. Facilitators and barriers to adherence to gluten-free diet among adults with celiac disease: a systematic review. Journal of Human Nutrition and Dietetics. 2020;33(6):786-810. doi: 10.1111/jhn.12754. PubMed PMID: WOS:000529241900001.

790. Abu-Naser D. Impact of Clinical Pharmacist Interventions in Prescribing Errors in Hospitalized Diabetic Patients with Major Polypharmacy. Hospital Pharmacy. 2021;56(4):392-9. doi: 10.1177/0018578720985428. PubMed PMID: 151666608. Language: English. Entry Date: 20210805. Revision Date: 20210805. Publication Type: Article.

791. Abu-Oliem AS, Al-Sharayri MG, AlJabra RJ, Hakuz NM. A clinical trial to investigate the role of clinical pharmacist in resolving/preventing drug related problems in ICU patients who receive anti-infective therapy. Jordan journal of pharmaceutical sciences. 2013;6(3):292-8. PubMed PMID: CN-00914027.

792. Abu-Ramaileh AM, Shane R, Churchill W, Steffenhagen A, Patka J, Rothschild JM. Evaluating and classifying pharmacists' quality interventions in the emergency department. American Journal of Health-System Pharmacy. 2011;68(23):2271-5. doi: 10.2146/ajhp110004. PubMed PMID: 104631760. Language: English. Entry Date: 20120221. Revision Date: 20200708. Publication Type: Journal Article.

793. Abu-Rish EY, Barakat NA. The impact of pharmacist-led educational intervention on pneumococcal vaccine awareness and acceptance among elderly in Jordan. Human vaccines & immunotherapeutics. 2021;17(4):1181-9. doi: 10.1080/21645515.2020.1802973. PubMed PMID: CN-02178420.

794. Abu-Sawwa R, Busque K, Cokley J. Parenteral medication considerations for the ketogenic diet. American Journal of Health-System Pharmacy. 2023;80(19):1357-63. doi: 10.1093/ajhp/zxad121. PubMed PMID: 172330912.

795. Abu-Sini MK, Maharmah RA, Abulebdah DH, Al-Sabi MNS. Isolation and Identification of Coliform Bacteria and Multidrug-Resistant Escherichia coli from Water Intended for Drug Compounding in Community Pharmacies in Jordan. Healthcare (Switzerland). 2023;11(3). doi: 10.3390/healthcare11030299.

796. Abu-Sini MK, Maharmah RA, Abulebdah DH, Al-Sabi MNS. Isolation and Identification of Coliform Bacteria and Multidrug-Resistant <i>Escherichia coli</i> from Water Intended for Drug Compounding in Community Pharmacies in Jordan. Healthcare. 2023;11(3). doi: 10.3390/healthcare11030299. PubMed PMID: WOS:000930418700001.

797. Abu-Zaid AM, Barakat M, Al-Qudah R, Abdalhafez A. The Role of Pharmacists in Patient Counselling for OTC Medication in Jordan: A Cross-Section Study. Jordan Journal of Pharmaceutical Sciences. 2021;14(4):445-55.

798. Abualhasan M, Dwaikat S, Ataya R, Ali A, Al-Atrash M. Quality evaluation of iron-containing food supplements in the Palestinian market. Food Science and Technology (Brazil). 2021;41:785-90. doi: 10.1590/fst.01621.

799. Abualhasan M, Tahan S, Nassar R, Damere M, Salameh H, Zyoud H. Pharmacists' knowledge of drug food administration and their appropriate patient counseling a cross-sectional study from Palestine. Journal of Health Population and Nutrition. 2023;42(1). doi: 10.1186/s41043-023-00444-9. PubMed PMID: WOS:001067100400001.

800. Abualhasan MN, Hamad VN, Abdulsamd M, Robe TY, Al Atrash M. A Pilot Study to Evaluation of the Level of Knowledge and Quality of Water Used in Drug Reconstitution among Community Pharmacist in Palestine. British Journal of Pharmaceutical Research. 2015;8(6). doi: 10.9734/bjpr/2015/20302. PubMed PMID: WOS:000370133700002.

801. Abuasbeh K, Qureshi S, Soomro A, Awadalla M. An Optimal Family of Block Techniques to Solve Models of Infectious Diseases: Fixed and Adaptive Stepsize Strategies. Mathematics. 2023;11(5). doi: 10.3390/math11051135.

802. Abubakar A, Bentley O. The role of the pharmacist in pharmacogenetics. Pharmacy Times. 2016;82(8).

803. Abubakar A, Sinclair J. The Emerging Role of Community Pharmacists in Remote Patient Monitoring Services. Pharmacy. 2020;8(3). doi: 10.3390/pharmacy8030166. PubMed PMID: WOS:000578861600001.

804. Abubakar M, Atif M. Impact of Pharmacist-Led Interventions on Diabetes Management at a Community Pharmacy in Pakistan: a Randomized Controlled Trial. Inquiry. 2021;58:469580211036283. doi: 10.1177/00469580211036283. PubMed PMID: CN-02326775.

805. Abubakar M, Ishaque A, Shahzad F, Atif M, Ashfaq T. Impact of pharmacist-led interventions on diabetes management at a community pharmacy in Pakistan: A quasi-experimental study. JPMA The Journal of the Pakistan Medical Association. 2021;71(8):1924-9. doi: 10.47391/JPMA.2220.

806. Abubakar U, Syed Sulaiman SA, Adesiyun AG. Utilization of surgical antibiotic prophylaxis for obstetrics and gynaecology surgeries in Northern Nigeria. International Journal of Clinical Pharmacy. 2018;40(5):1037-43. doi: 10.1007/s11096-018-0702-0. PubMed PMID: 132697483. Language: English. Entry Date: 20190313. Revision Date: 20191106. Publication Type: journal article.

807. Abubakar U, Tangiisuran B. Knowledge and practices of community pharmacists towards non-prescription dispensing of antibiotics in Northern Nigeria. International Journal of Clinical Pharmacy. 2020;42(2):756-64. doi: 10.1007/s11096-020-01019-y.

808. AbuBlan RS, Awad W, Agha R, Hejawi N, Srouji H, Hammoudeh S, et al. Impacts of a Mail-Order Service for Refilling Prescriptions on Patient Satisfaction and Operational Load at a Comprehensive Cancer Center in Jordan. Hospital Pharmacy. 2021;56(5):543-9. doi: 10.1177/0018578720928266. PubMed PMID: WOS:000539854600001.

809. Abudalo R, Abudalo R, Alqudah A, Abuqamar A, Abdelaziz A, Alshawabkeh M, et al. Pharmacy practitioners' practice, awareness and knowledge about herbal products and their potential interactions with cardiovascular drugs. F1000Research. 2022;11. doi: 10.12688/f1000research.121709.2.

810. Abuelhana ASA. Analysis of Clinical Pharmacy Services and Development of a Model to Inform the Appropriate Skill Mix for a Clinical Pharmacy Workforce to Achieve the Optimisation of Both Patient Outcomes and Cost-Effectiveness [Ph.D.]. Northern Ireland: University of Ulster (United Kingdom); 2020.

811. Abuelsoud NN. Drug-drug interaction management in internal medicine specialty. Asian Journal of Pharmaceutics. 2017;11(3):S566-S74.

812. Abuga K, Ongarora D, Karumbi J, Olulo M, Minnaard W, Kibwage I. Sub-Standard Pharmaceutical Services in Private Healthcare Facilities Serving Low-Income Settlements in Nairobi County, Kenya. Pharmacy. 2019;7(4). doi: 10.3390/pharmacy7040167. PubMed PMID: WOS:000505734200021.

813. Abughosh S, Wang X, Serna O, Esse T, Mann A, Masilamani S, et al. A Motivational Interviewing Intervention by Pharmacy Students to Improve Medication Adherence. Journal of managed care & specialty pharmacy. 2017;23(5):549-60. doi: 10.18553/jmcp.2017.23.5.549. PubMed PMID: CN-01443443.

814. Abughosh S, Wang X, Serna O, Esse T, Vadhariya A, Mann A, et al. A motivational interviewing intervention by pharmacy students to improve medication adherence. Journal of managed care and specialty pharmacy Conference: AMCP managed care and specialty pharmacy annual meeting 2017 United states. 2017;23(3-A SUPPL.):S69. PubMed PMID: CN-01669995.

815. Abughosh S, Wang X, Serna O, Esse TW, Fleming ML. Feasibility of a phone-based motivational interviewing intervention to improve medication adherence by pharmacy students on advanced pharmacy practice experience rotation in a medicare advantage with prescription drug plan. Value in health Conference: ISPOR 21st annual international meeting research Washington, DC united states Conference start: 20160521 Conference end: 20160525 Conference publication: (varpagings). 2016;19(3):A206. PubMed PMID: CN-01266604.

816. Abughosh SM, Vadhariya A, Johnson ML, Essien EJ, Esse TW, Serna O, et al. Enhancing statin adherence using a motivational interviewing intervention and past adherence trajectories in patients with suboptimal adherence. Journal of managed care & specialty pharmacy. 2019;25(10):1053-62. doi: 10.18553/jmcp.2019.25.10.1053. PubMed PMID: CN-02011524.

817. Abughosh SM, Wang X, Serna O, Esse T, Mann A, Essien EJ, et al. A motivational interviewing (MI) intervention by pharmacy students to prevent medication discontinuation. Value in health. 2017;20(5):A173-. PubMed PMID: CN-01407897.

818. Abughosh SM, Wang X, Serna O, Henges C, Masilamani S, Essien EJ, et al. A Pharmacist Telephone Intervention to Identify Adherence Barriers and Improve Adherence Among Nonadherent Patients with Comorbid Hypertension and Diabetes in a Medicare Advantage Plan. Journal of Managed Care & Specialty Pharmacy. 2016;22(1):63-73. PubMed PMID: 27015053.

819. Abuirmeileh A, Samara S, Alkhodari A, Bahnassi A, Talhouni A, Hayallah AM. Antibiotic dispensing without prescription in Jordanian community pharmacies: A pharmacist's perspective. Bulletin of Pharmaceutical Sciences Assiut. 2014;37:51-63. doi: 10.21608/bfsa.2014.62622.

820. Abujheisha KY, Ahmed N. PHARMACISTS' ATTITUDES AND PRACTICE TOWARDS DISPENSING ANTIBIOTICS WITHOUT A MEDICAL PRESCRIPTION (DAWMP). ARE PHARMACISTS DOING WELL? Indo American Journal of Pharmaceutical Sciences. 2018;5(2):1301-8. doi: 10.5281/zenodo.1195517. PubMed PMID: WOS:000434386200060.

821. Abujheisha KY, Al-Shdefat R, Ahmed N, Fouda MI. Public Knowledge and Behaviours Regarding Antibiotics Use: A Survey among the General Public. International Journal of Medical Research & Health Sciences. 2017;6(6):82-8. PubMed PMID: WOS:000408929500007.

822. Abukhalil AD, Shaloudi AY, Shamasneh NM, Aljamal AM. Awareness of Beers criteria and potentially inappropriate medications among physicians and pharmacists in Palestine. Journal of Pharmacy Practice & Research. 2021;51(5):381-9. doi: 10.1002/jppr.1728. PubMed PMID: 153560869. Language: English. Entry

Date: 20211129. Revision Date: 20221003. Publication Type: Article.

823. Abukres SH, Hoti K, Hughes JD. Patient attitudes towards a new role for pharmacists: Continued dispensing. Patient Preference and Adherence. 2014;8:1143-51. doi: 10.2147/PPA.S66719.

824. Abuloha S, Alabbadi I, Albsoul-Younes A, Younes N, Zayed A. The role of clinical pharmacist in initiation and/or dose adjustment of insulin therapy in diabetic patients in outpatient clinic in Jordan. Jordan journal of pharmaceutical sciences. 2016;9(1):33-50. PubMed PMID: CN-01132845.

825. AbuNaba'a Y, Basheti IA. Assessing the impact of medication management review service for females diagnosed with depression and anxiety: a randomized control trial. Journal of evaluation in clinical practice. 2020;26(5):1478-89. doi: 10.1111/jep.13314. PubMed PMID: CN-02005236.

826. Aburuz S, Jaber D, AlAhmad M, Basheti I, Arafat M, Sadeq A, et al. Drug-related problems and the value of a pharmaceutical care service in general surgery patients. International journal of clinical pharmacy. 2020;42(2):817-. doi: 10.1007/s11096-020-01009-0. PubMed PMID: CN-02205439.

827. Aburuz S, Jaber D, Basheti I, Sadeq A, Arafat M, Alahmad M, et al. Impact of pharmacist interventions on drug-related problems in general surgery patients: a randomised controlled trial. European journal of hospital pharmacy. 2020. doi: 10.1136/ejhpharm-2020-002206. PubMed PMID: CN-02195246.

828. AbuRuz S, Jaber D, Basheti I, Sadeq A, Arafat M, AlAhmad M, et al. Impact of pharmacist interventions on drug-related problems in general surgery patients: a randomised controlled trial. European journal of hospital pharmacy : science and practice. 2021;28(Suppl 2):e72-e8. doi: 10.1136/ejhpharm-2020-002206. PubMed PMID: CN-02391212.

829. Abusaksaka YA, Modeste RRM, Vember H. Perceived Occurrence of Medication Administration Errors among Nursing Students at a Higher Education Institution in Western Cape, South Africa. Africa Journal of Nursing & Midwifery. 2020;22(2):1-18. doi: 10.25159/2520-5293/6957. PubMed PMID: 148604749. Language: English. Entry Date: 20210215. Revision Date: 20210215. Publication Type: Article.

830. Abusara OH, Bishtawi S, Al-Qerem W, Jarrar W, Al-Khareisha L, Khdair SI. Pharmacists' knowledge, familiarity, and attitudes towards biosimilar drugs among practicing Jordanian pharmacists: A cross sectional study. Journal of King Saud University - Science. 2023;35(6):102767. doi: https://doi.org/10.1016/j.jksus.2023.102767.

831. Abushaiqa ME, Zaran FK, Bach DS, Smolarek RT, Farber MS. Educational interventions to reduce use of unsafe abbreviations. American Journal of Health-System Pharmacy. 2007;64(11):1170-3. doi: 10.2146/ajhp060173. PubMed PMID: 106166058. Language: English. Entry Date: 20071005. Revision Date: 20200708. Publication Type: Journal Article.

832. Abushanab D, Atchan M, Elajez R, Elshafei M, Abdelbari A, Al Hail M, et al. Economic impact of clinical pharmacist interventions in a general tertiary hospital in Qatar. PLoS One. 2023;18(6):e0286419.

833. Abushanab D, Gulied A, Hamad A, Abu-Tineh M, Abdul Rouf PV, Al Hail M, et al. Cost savings and cost avoidance with the inpatient clinical pharmacist interventions in a tertiary cancer care hospital. Journal of Oncology Pharmacy Practice. 2023;29(8):1935-43. PubMed PMID: 36946146.

834. Abushanab D, Mohammed R, Kaddoura R, AlMaraghi F, Farhat A, Mahfouz A, et al. EE431 Description of Clinical Pharmacists Reported Interventions to Prevent Adverse Drug Events Among Patients with Cardiovascular Disease. Value in Health. 2022;25(7, Supplement):S419. doi: https://doi.org/10.1016/j.jval.2022.04.679.

835. Abuya T, Fegan G, Rowa Y, Karisa B, Ochola S, Mutemi W, et al. Impact of Ministry of Health interventions on private medicine retailer knowledge and practices on anti-malarial treatment in Kenya. American Journal of Tropical Medicine and Hygiene. 2009;80(6):905-13. doi: 10.4269/ajtmh.2009.80.905.

836. Abuya TO. A comparative analysis of malaria control programmes targeting delivery of over-the-counter anti-malarial drugs in kenya [Ph.D.]. England: Open University (United Kingdom); 2009.

837. Abuya TO, Fegan G, Amin AA, Akhwale WS, Noor AM, Snow RW, et al. Evaluating Different Dimensions of Programme Effectiveness for Private Medicine Retailer Malaria Control Interventions in Kenya. Plos One. 2010;5(1). doi: 10.1371/journal.pone.0008937. PubMed PMID: WOS:000274138000015.

838. AbuYassin BH, Aljadhey H, Al-Sultan M, Al-Rashed S, Adam M, Bates DW. Accuracy of the medication history at admission to hospital in Saudi Arabia. Saudi Pharmaceutical Journal. 2011;19(4):263-7. doi: https://doi.org/10.1016/j.jsps.2011.04.006.

839. Abuye H, Abraham W, Kebede S, Tatiparthi R, Suleman S. Physicochemical quality assessment of antimalarial medicines: Chloroquine phosphate and quinine sulfate tablets from drug retail outlets of south-west ethiopia. Infection and Drug Resistance. 2020;13:691-701. doi: 10.2147/IDR.S234684.

840. Abuzour AS, Lewis PJ, Tully MP. Factors influencing secondary care pharmacist and nurse independent prescribers' clinical reasoning: An interprofessional analysis. Journal of Interprofessional Care. 2018;32(2):160-8.

841. Abuzour AS, Lewis PJ, Tully MP. Practice makes perfect: A systematic review of the expertise development of pharmacist and nurse independent prescribers in the United Kingdom. Research in Social and Administrative Pharmacy. 2018;14(1):6-17. doi: https://doi.org/10.1016/j.sapharm.2017.02.002.

842. Abuzour AS, Magola-Makina E, Dunlop J, O'Brien A, Khawagi WY, Ashcroft DM, et al. Implementing prescribing safety indicators in prisons: A mixed methods study. British Journal of Clinical Pharmacology. 2022;88(4):1866-84. PubMed PMID: 34625991.

843. Academy of Managed Care P, American Pharmacists A. Medicare star ratings: stakeholder proceedings on community pharmacy and managed care partnerships in quality. Journal of the American Pharmacists Association: JAPhA. 2014;54(3):228-40. PubMed PMID: 24770440.

844. Acar B. Konya Büyükşehir Belediyesi Koyunoğlu Müzesi ve Kütüphanesi'ndeki 13215 Numarali Güfte Mecmuasi ve 14717 Numarali Șiir Mecmuasinin "Mecmualarin Sistematik Tasnifi Projesi"ne (MESTAP) Göre Tasnifi [M.A.]. Turkey: Necmettin Erbakan University (Turkey); 2020.

845. Acevedo P, Camino JI, Plaza Ugalde A. Information system on the ambulatory use of dietetic products in the Basque Country. Pharmaceutical Care Espana. 2001;3(6):439-45.

846. Aceves-Hernandez JM, Nicolás-Vázquez I, Aceves FJ, Hinojosa-Torres J, Paz M, Castaño VM. Indomethacin polymorphs: Experimental and conformational analysis. Journal of Pharmaceutical Sciences. 2009;98(7):2448-63. doi: https://doi.org/10.1002/jps.21626.

847. Achankunju B, Thomas J, Alexander R, Varughese SA, Sam GK. Assessment and evaluation on knowledge, attitude, and practice towards medication therapy management among community of Davangere city. Asian Journal of Pharmaceutical and Clinical Research. 2017;10(4):380-3. doi: 10.22159/ajpcr.2017.v10i4.16889.

848. Acharya M. Opioid Trajectories, Safety and Potential Opioid Misuse among Individuals with Tramadol, Short-Acting Hydrocodone or Short-Acting Oxycodone Therapy [Ph.D.]. United States -- Arkansas:

University of Arkansas for Medical Sciences; 2022.

849. Acharya M, Chopra D, Hayes CJ, Teeter B, Martin BC. Cost-Effectiveness of Intranasal Naloxone Distribution to High-Risk Prescription Opioid Users. Value in Health. 2020;23(4):451-60. doi: https://doi.org/10.1016/j.jval.2019.12.002.

850. Acharya Y, Nepal P, Yang D, Karki K, Bajracharya D, Prentis T, et al. Economic and social drivers of antibiotic dispensing practices among community pharmacies in Nepal. Tropical Medicine & International Health. 2021;26(5):557-71. PubMed PMID: 33524230.

851. Acheampong F, Anto BP. Perceived barriers to pharmacist engagement in adverse drug event prevention activities in Ghana using semi-structured interview. BMC Health Services Research. 2015;15(1):361-. doi: 10.1186/s12913-015-1031-9. PubMed PMID: 109645355. Language: English. Entry Date: 20150923. Revision Date: 20160313. Publication Type: journal article. Journal Subset: Biomedical.

852. Acheampong F, Anto BP, Koffuor GA. Medication safety strategies in hospitals–a systematic review. International Journal of Risk & Safety in Medicine. 2014;26(3):117-31.

853. Acheampong F, Bruce E, Anto BP. Medication safety activities of hospital pharmacists in Ghana; challenges and perceived impact on patient care. International Journal of Risk & Safety in Medicine. 2015;27(1):1-10. PubMed PMID: 25766062.

854. Acheuk L, Mouchoux C, Lepetit A, Novais T. Représentations des pharmaciens et préparateurs en pharmacie vis-à-vis de la prise en soin de la dépression chez la personne âgée. Annales Pharmaceutiques Françaises. 2020;78(2):167-78. doi: https://doi.org/10.1016/j.pharma.2019.11.001.

855. Acheuk L, Mouchoux C, Lepetit A, Novais T. Community pharmacists' and pharmacy technicians' attitudes and beliefs about depression in elderly. Annales Pharmaceutiques Francaises. 2020;78(2):167-78. doi: 10.1016/j.pharma.2019.11.001.

856. Achhra CV, Gharat M. Upgradation of pharmacy practice in India: Need of the hour. Pharma Times. 2015;47(3):15-7.

857. Achieng L, Musangi H, Ong'uti S, Ombegoh E, Bryant L, Mwiindi J, et al. An observational cohort comparison of facilitators of retention in care and adherence to anti-eetroviral therapy at an HIV treatment center in Kenya. PLoS ONE. 2012;7(3). doi: 10.1371/journal.pone.0032727.

858. Achilleos M, McEwen J, Hoesly M, DeAngelo M, Jennings T. Pharmacist-led program to improve transitions from acute care to skilled nursing facility care. American Journal of Health-System Pharmacy. 2020;77(12):979-84. PubMed PMID: 32377682.

859. Acker CJ. Creating the American junkie: Addiction research in the classic era of narcotic control: JHU Press; 2002.

860. Ackerman SJ, Knight T, Wahl PM, Cartwright CP. Health care utilization and costs following amplified versus non-amplified molecular probe testing for symptomatic patients with suspected vulvovaginitis: a US commercial payer population. Clinicoeconomics and Outcomes Research. 2019;11:179-89. doi: 10.2147/ceor.S191831. PubMed PMID: WOS:000459488200001.

861. Ackerman SJ, Polly Jr DW, Knight T, Schneider K, Holt T, Cummings Jr J. Comparison of the costs of nonoperative care to minimally invasive surgery for sacroiliac joint disruption and degenerative sacroiliitis in a United States commercial payer population: Potential economic implications of a new minimally invasive technology. ClinicoEconomics and Outcomes Research. 2014;6(1):283-96. doi: 10.2147/CEOR.S63757.

862. Ackermann E. The Case Against. Journal of Pharmacy Practice & Research. 2018;48(3):277-80. doi: 10.1002/jppr.1465. PubMed PMID: 130228870. Language: English. Entry Date: 20180621. Revision Date: 20190603. Publication Type: Article. Journal Subset: Australia & New Zealand.

863. Ackermann RT, Rosenman MB, Downs SM, Holmes AM, Katz BP, Li J, et al. Telephonic case-finding of major depression in a Medicaid chronic disease management program for diabetes and heart failure. General Hospital Psychiatry. 2005;27(5):338-43. doi: https://doi.org/10.1016/j.genhosppsych.2005.05.009.

864. Ackert KE, Bauerle W, Pellegrino AN, Stoltzfus J, Pateman S, Graves D, et al. Implementation of an enhanced recovery after surgery (ERAS) protocol for total abdominal hysterectomies in the division of gynecologic oncology: A network-wide quality improvement initiative. Journal of Osteopathic Medicine. 2023;123(10):493-8. doi: 10.1515/jom-2022-0204.

865. Ackroyd-Stolarz S, Hartnell N, MacKinnon NJ. Approaches to improving the safety of the medication use system. Healthcare Quarterly. 2005;8:59-64.

866. Acosta A, Ciapponi A, Aaserud M, Vietto V, Austvoll-Dahlgren A, Kösters JP, et al. Pharmaceutical policies: effects of reference pricing, other pricing, and purchasing policies. Cochrane Database of Systematic Reviews. 2014;(10). doi: 10.1002/14651858.CD005979.pub2. PubMed PMID: CD005979.

867. Acosta de la Luz CLL. Growing medicinal plants in containers. Revista Cubana de Plantas Medicinales. 2014;19(1):4-13.

868. Acosta M, Darban A, Gitlin M. Logistical Considerations Related to Melphalan As a Conditioning Agent in Autologous Stem Cell Transplant for Multiple Myeloma: Results of a Survey of Oncology Pharmacists, Nurses and Pharmacy Technicians. Blood. 2019;134:5869. doi: https://doi.org/10.1182/blood-2019-124990.

869. Acquavita SP, Lewis MA, Aparicio E, Pecukonis E. Student perspectives on interprofessional education and experiences. Journal of Allied Health. 2014;43(2):e31-e6.

870. Acquavita SP, Richardson GB, Smith R, Van Loon RA, Brehm B, Kim K, et al. Outcomes of an interprofessional SBIRT training program: Knowledge attainment and perceived competence for practice. Substance Abuse. 2021;42(4):935-43. doi: 10.1080/08897077.2021.1900982.

871. Acquisto NM, Schult RF, Sarnoski-Roberts S, Wilmarth J, Jones CMC, McCann M, et al. Effect of pharmacist-led task force to reduce opioid prescribing in the emergency department. American Journal of Health-System Pharmacy. 2019;76(22):1853-61. PubMed PMID: 31557284.

872. Actrn. Analgesia after major thoracic or abdominal surgery: a comparison of patient controlled analgesia with morphine versus tramadol versus morphine/tramadol combination. http://wwwwhoint/trialsearch/Trial2aspx?TrialID=ACTRN12605000564651. 2005. PubMed PMID: CN-02432529.

873. Actrn. Bowel recovery after colorectal surgery: a comparison of patient contolled analgesia with tramadol versus morphine. http://wwwwhoint/trialsearch/Trial2aspx?TrialID=ACTRN12605000563662. 2005. PubMed PMID: CN-02442880.

874. Actrn. City Views. https://trialsearchwhoint/Trial2aspx?TrialID=ACTRN12605000637640. 2005. PubMed PMID: CN-01799996.

875. Actrn. A double blind randomised placebo-controlled intervention trial to determine the efficacy of the probiotic VSL #3 in reducing the incidence and or frequency of diarrhoea in enterally-fed critically ill patients. https://trialsearchwhoint/Trial2aspx?TrialID=ACTRN12605000167662. 2005. PubMed PMID:

CN-01830191.

876. Actrn. Improving evidence based prescribing in older adults moving from hospital to long-term facilities with a pharmacist transition coordinator: a randomised controlled trial. https://trialsearchwhoint/Trial2aspx?TrialID=ACTRN12605000376640. 2005. PubMed PMID: CN-01882920.

877. Actrn. Dose Administration Aids (DAAs) study. https://trialsearchwhoint/Trial2aspx?TrialID=ACTRN12606000247572. 2006. PubMed PMID: CN-01868587.

878. Actrn. Evaluation of an interactive audiovisual computer program educating patients with rheumatoid arthritis starting treatment with methotrexate: a randomized controlled trial. https://trialsearchwhoint/Trial2aspx?TrialID=ACTRN12606000072516. 2006. PubMed PMID: CN-01853729.

879. Actrn. Pilot of a randomised controlled trial of nutrition screening and appropriate support in hospitalised elderly. https://trialsearchwhoint/Trial2aspx?TrialID=ACTRN12606000237583. 2006. PubMed PMID: CN-01884028.

880. Actrn. Placebo controlled,randomised,crossover study of the efficacy of dipyridamole in the management of angina in patients with Coronary Slow Flow Phenomenon. https://trialsearchwhoint/Trial2aspx?TrialID=ACTRN12606000236594. 2006. PubMed PMID: CN-01878505.

881. Actrn. A research study for patients with acute myeloid leukemia (AML) in first relapse. https://trialsearchwhoint/Trial2aspx?TrialID=ACTRN12606000514505. 2006. PubMed PMID: CN-01866599.

882. Actrn. Selection of Nicotine Therapy to Increase Quitting. https://trialsearchwhoint/Trial2aspx?TrialID=ACTRN12606000451505. 2006. PubMed PMID: CN-01810807.

883. Actrn. Trial of nurse and GP partnership for care of COPD. https://trialsearchwhoint/Trial2aspx?TrialID=ACTRN12606000304538. 2006. PubMed PMID: CN-01852986.

884. Actrn. A collaborative disease management program for managing cholesterol in hospital patients. http://wwwwhoint/trialsearch/Trial2aspx?TrialID=ACTRN12607000313437. 2007. PubMed PMID: CN-02440004.

885. Actrn. Freedom study (Falls Risk Epidemiology: effect of Vitamin D on skeletal Outcomes and other Measures). https://trialsearchwhoint/Trial2aspx?TrialID=ACTRN12607000089437. 2007. PubMed PMID: CN-01835069.

886. Actrn. A prospective, randomized, placebo-controlled, double-blind, cross-over study of the usefulness of sustained-release opioids in the subjective sensation of dyspnoea secondary to advanced disease with no reversible components in opioid naive patients. https://trialsearchwhoint/Trial2aspx?TrialID=ACTRN12607000075482. 2007. PubMed PMID: CN-01817364.

887. Actrn. Which Heart failure Intervention is most Cost-effective & consumer friendly in reducing Hospital care: the WHICH? Study. https://trialsearchwhoint/Trial2aspx?TrialID=ACTRN12607000069459. 2007. PubMed PMID: CN-01863456.

888. Actrn. The efficacy of Hypericum perforatum extract (Ze117), nicotine patches and combination Hypericum perforatum/nicotine patches for smoking cessation. http://wwwwhoint/trialsearch/Trial2aspx?TrialID=ACTRN12608000500358. 2008. PubMed PMID: CN-02433000.

889. Actrn. Guidelines Adherence to the Polypill Study. A clinical trial of a fixed dose combination medication (Polypill) versus usual care for improved adherence to indicated pharmacotherapy among

individuals at high risk of a cardiovascular event. https://trialsearchwhoint/Trial2aspx?TrialID=ACTRN12608000584336. 2008. PubMed PMID: CN-01807993.

890. Actrn. The Kanyini Guidelines Adherence with the Polypill Study- A clinical trial to investigate whether a fixed dose combination 'Polypill' Medication is better than existing treatments for people at high risk of cardiovascular disease. https://trialsearchwhoint/Trial2aspx?TrialID=ACTRN12608000583347. 2008. PubMed PMID: CN-01830061.

891. Actrn. Pharmacy dispensing records to identify and educate patients with suboptimal asthma management. http://wwwwhoint/trialsearch/Trial2aspx?TrialID=ACTRN12608000119392. 2008. PubMed PMID: CN-02433123.

892. Actrn. Delivering a personalised smoking cessation intervention by community pharmacists in Western Australia: a pilot, randomised controlled trial. http://wwwwhoint/trialsearch/Trial2aspx?TrialID=ACTRN12609000885291. 2009. PubMed PMID: CN-02431979.

893. Actrn. Is Inspiratory Flow Rate (IFR) appropriate for the puffers patients use for their lung conditions, and will training them to achieve the appropriate IFR improve their shortness of breath and reduce their need for health services? http://wwwwhoint/trialsearch/Trial2aspx?TrialID=ACTRN12609000801213. 2009. PubMed PMID: CN-02441271.

894. Actrn. N-of-1 Trials of Stimulant (dexamphetamine or methylphenidate) vs Placebo for Paediatric Traumatic Brain Injury. http://wwwwhoint/trialsearch/Trial2aspx?TrialID=ACTRN12609000865213. 2009. PubMed PMID: CN-02443233.

895. Actrn. Pharmacist Prescribing in a Sexual Health and Human Immunodeficiency Virus (HIV) Outpatient Clinic. http://wwwwhoint/trialsearch/Trial2aspx?TrialID=ACTRN12609000427279. 2009. PubMed PMID: CN-02441600.

896. Actrn. Pharmacists Improving Patient Outcomes in the Perioperative Service Project: the effect of pharmacist prescribing and history taking on missed medications postoperatively. http://wwwwhoint/trialsearch/Trial2aspx?TrialID=ACTRN12609000868280. 2009. PubMed PMID: CN-02441017.

897. Actrn. A placebo-controlled, randomized clinical trial of Bioeffective A in the management of acute alcohol withdrawal and alcohol abstinence maintenance. https://trialsearchwhoint/Trial2aspx?TrialID=ACTRN12609000649213. 2009. PubMed PMID: CN-01840457.

898. Actrn. Randomized trial of homocysteine lowering treatment of depression in later life (B-VITAge). https://trialsearchwhoint/Trial2aspx?TrialID=ACTRN12609000256279. 2009. PubMed PMID: CN-01839453.

899. Actrn. Short Course Oncology Therapy. A study of adjuvant chemotherapy in colorectal cancer. http://wwwwhoint/trialsearch/Trial2aspx?TrialID=ACTRN12609000662268. 2009. PubMed PMID: CN-02432931.

900. Actrn. To Evaluate the Impact of a Doctor – Pharmacist Collaborative Prescribing Model in a Multi-Disciplinary Surgical Pre-Admission Clinic. http://wwwwhoint/trialsearch/Trial2aspx?TrialID=ACTRN12609000426280. 2009. PubMed PMID: CN-02432524.

901. Actrn. The effectiveness of alternative antiemetic therapies in patients with cancer and nausea not related to cancer therapy. https://trialsearchwhoint/Trial2aspx?TrialID=ACTRN12610000482066. 2010. PubMed PMID: CN-01811189.

902. Actrn. Evaluating the impact of a training-and-communication network program in nephrology to

facilitate the detection and management of drug-related problems by community pharmacists. https://trialsearchwhoint/Trial2aspx?TrialID=ACTRN12610001088033. 2010. PubMed PMID: CN-01810569.

903. Actrn. Evaluation of a training and communication-network nephrology program for community pharmacists. https://trialsearchwhoint/Trial2aspx?TrialID=ACTRN12610000613000. 2010. PubMed PMID: CN-01840684.

904. Actrn. To evaluate the impact of additional delivery strategies (involving the non-medical private sector) for low osmolarity Oral Rehydration Salts (ORS) and zinc supplements for the treatment of acute diarrhoea in under five children on Oral Rehydration Salts (ORS) solution use rate as well as zinc use rate when compared to standard delivery through health facilities. http://wwwwhoint/trialsearch/Trial2aspx?TrialID=ACTRN12610000413022. 2010. PubMed PMID: CN-02441278.

905. Actrn. Effect of solifenacin and oxybutynin patch on cognitive function in elderly women with overactive bladder: an open-labelled, randomised, cross-over pilot study. https://trialsearchwhoint/Trial2aspx?TrialID=ACTRN12611000796987. 2011. PubMed PMID: CN-01859792.

906. Actrn. The effects of Glucagon-Like Peptide 1 on gastric emptying in healthy volunteers with normal or low blood glucose levels. http://wwwwhoint/trialsearch/Trial2aspx?TrialID=ACTRN12611000973910. 2011. PubMed PMID: CN-02440111.

907. Actrn. Evaluation of Captopril and Nifedipine in Treatment of Hypertension in Children with Post-streptoccal Acute Glomerulonephritis with Hypertension- A Randomized Control Trial. https://trialsearchwhoint/Trial2aspx?TrialID=ACTRN12611000778987. 2011. PubMed PMID: CN-01804955.

908. Actrn. Evaluation of the effectiveness of a consumer pamphlet for low back pain. http://wwwwhoint/trialsearch/Trial2aspx?TrialID=ACTRN12611000053921. 2011. PubMed PMID: CN-02442554.

909. Actrn. Implementing and sustaining cultural and practice change to improve the nutritional status of patients in the acute care setting. http://wwwwhoint/trialsearch/Trial2aspx?TrialID=ACTRN12611000020987. 2011. PubMed PMID: CN-02440330.

910. Actrn. Improving medication risk management among residents of retirement villages. https://trialsearchwhoint/Trial2aspx?TrialID=ACTRN12611000109909. 2011. PubMed PMID: CN-01865990.

911. Actrn. Medication Reviews ReDirected (MedReDi): acute Coronary Syndrome as an Indication for Home Medicine Review, a Randomised Controlled Trial. http://wwwwhoint/trialsearch/Trial2aspx?TrialID=ACTRN12611000452998. 2011. PubMed PMID: CN-02432187.

912. Actrn. A pilot study to assess the feasibility of a prospective randomised controlled trial of a patient-centred medicines management approach to reduce the burden of iatrogenic symptoms in palliative care. https://trialsearchwhoint/Trial2aspx?TrialID=ACTRN12611001262998. 2011. PubMed PMID: CN-01830003.

913. Actrn. The role of a pharmacist supporting beliefs about medications in people who have experienced a heart attack. https://trialsearchwhoint/Trial2aspx?TrialID=ACTRN12611000322932. 2011. PubMed PMID: CN-01870995.

914. Actrn. Very High Intensity Users of Middlemore Hospital Emergency Department. https://trialsearchwhoint/Trial2aspx?TrialID=ACTRN12611000496910. 2011. PubMed PMID: CN-01868859.

915. Actrn. Addressing the needs of osteoporosis prevention in postmenopausal women attending a tertiary hospital in Malaysia. http://wwwwhoint/trialsearch/Trial2aspx?TrialID=ACTRN12612000900819. 2012.

PubMed PMID: CN-02441163.

916. Actrn. Antidepressants to maintain remission and improve quality of life and mental health in Crohn's disease (CD) patients: a pilot randomised controlled trial. https://trialsearchwhoint/Trial2aspx?TrialID=ACTRN12612001067864. 2012. PubMed PMID: CN-01847635.

917. Actrn. Comparison of oral antidiabetic drugs with insulin in the treatment of Gestational Diabetes Mellitus. https://trialsearchwhoint/Trial2aspx?TrialID=ACTRN12612001272886. 2012. PubMed PMID: CN-01850906.

918. Actrn. Comprehension of health education leaflets designed for patients with limited literacy – a randomized controlled trial. http://wwwwhoint/trialsearch/Trial2aspx?TrialID=ACTRN12612000761864. 2012. PubMed PMID: CN-02441689.

919. Actrn. Do blood glucose levels after meals relate to gut hormones and stomach emptying in young people with cystic fibrosis? http://wwwwhoint/trialsearch/Trial2aspx?TrialID=ACTRN12612000618853. 2012. PubMed PMID: CN-02440294.

920. Actrn. The effects of self-treatment on duration of exacerbations, health status and costs of health care in patients with Chronic Obstructive Pulmonary Disease (COPD) and common co-morbidities. http://wwwwhoint/trialsearch/Trial2aspx?TrialID=ACTRN12612000514808. 2012. PubMed PMID: CN-02440680.

921. Actrn. GIVE UP FOR GOOD- A study to evaluate the effectiveness of a pharmacist-led multidisciplinary smoking cessation intervention for smokers admitted to public hospitals. https://trialsearchwhoint/Trial2aspx?TrialID=ACTRN12612000368831. 2012. PubMed PMID: CN-01879760.

922. Actrn. Gout Treatment. Combined vs Allopurinol alone. http://wwwwhoint/trialsearch/Trial2aspx?TrialID=ACTRN12612000987864. 2012. PubMed PMID: CN-02440738.

923. Actrn. A maternal probiotic intervention for infant allergic disease prevention. https://trialsearchwhoint/Trial2aspx?TrialID=ACTRN12612000196842. 2012. PubMed PMID: CN-01840795.

924. Actrn. Multidisciplinary Approach to Management of Maternal Asthma (MAMMA [Copyright]). http://wwwwhoint/trialsearch/Trial2aspx?TrialID=ACTRN12612000681853. 2012. PubMed PMID: CN-02441057.

925. Actrn. Redefining pain management after cardiac surgery to improve intensive care and hospital length of stay: a randomised pilot trial. https://trialsearchwhoint/Trial2aspx?TrialID=ACTRN12612001243808. 2012. PubMed PMID: CN-01851461.

926. Actrn. A controlled trial of a health literacy education package for community pharmacists and pharmacy staff in Australia. http://wwwwhoint/trialsearch/Trial2aspx?TrialID=ACTRN12613000574741. 2013. PubMed PMID: CN-02432774.

927. Actrn. Does genetically guided antidepressant prescribing improve outcomes in depression? http://wwwwhoint/trialsearch/Trial2aspx?TrialID=ACTRN12613001135707. 2013. PubMed PMID: CN-02439861.

928. Actrn. Evaluation of a single iron infusion versus oral iron tablets in the treatment of pregnancy anaemia among Tasmanian women. https://trialsearchwhoint/Trial2aspx?TrialID=ACTRN12613000853741. 2013. PubMed PMID: CN-01809098.

929. Actrn. Is running re-education group more effective than high level balance group in reducing falls in the elderly living in the community? http://wwwwhoint/trialsearch/Trial2aspx?TrialID=ACTRN12613001314718. 2013. PubMed PMID: CN-02441171.

930. Actrn. Management of dry mouth following radiation therapy: investigation of a new saliva substitute product. https://trialsearchwhoint/Trial2aspx?TrialID=ACTRN12613000827730. 2013. PubMed PMID: CN-01835700.

931. Actrn. Melatonin and Cognitive-Behaviour Therapy for the treatment of insomnia in individuals with schizophrenia. http://wwwwhoint/trialsearch/Trial2aspx?TrialID=ACTRN12613000970741. 2013. PubMed PMID: CN-02441849.

932. Actrn. Opti-Med: a randomised controlled trial of deprescribing to optimise health outcomes for frail older people. https://trialsearchwhoint/Trial2aspx?TrialID=ACTRN12613001204730. 2013. PubMed PMID: CN-01857119.

933. Actrn. A Phase 1, Randomized, Double-Blind, Placebo-Controlled, Parallel Arm Study of the Safety, Tolerability and Pharmacokinetics of Oral BTD-001 in Healthy Volunteers. https://trialsearchwhoint/Trial2aspx?TrialID=ACTRN12613000919718. 2013. PubMed PMID: CN-01881728.

934. Actrn. Pilot trial of Aspirin versus Ticagrelor in Congestive Heart Failure. http://wwwwhoint/trialsearch/Trial2aspx?TrialID=ACTRN12613000826741. 2013. PubMed PMID: CN-02441247.

935. Actrn. A randomised controlled trial comparing daily (400 international units) and single bolus dosing (50,000 international units) of vitamin D in healthy breastfed infants of vitamin D deficient mothers. https://trialsearchwhoint/Trial2aspx?TrialID=ACTRN12613001234707. 2013. PubMed PMID: CN-01811821.

936. Actrn. A randomized, blinded, comparator-controlled trial investigating a 28-day course of Lyrica in participants with knee osteoarthritis who exhibit neuropathic pain, compared with a 28-day course of acetaminophen. https://trialsearchwhoint/Trial2aspx?TrialID=ACTRN12613000840785. 2013. PubMed PMID: CN-01801927.

937. Actrn. Salt and its effects on endothelial function in patients with type two diabetes. http://wwwwhoint/trialsearch/Trial2aspx?TrialID=ACTRN12613000127707. 2013. PubMed PMID: CN-02440063.

938. Actrn. Short-course Isoniazid and Rifapentine for Cost-effective Latent tuberculosis Eradication. https://trialsearchwhoint/Trial2aspx?TrialID=ACTRN12613000599774. 2013. PubMed PMID: CN-01816707.

939. Actrn. The SMART Study: can a Smartphone Application Improve Adherence to Antiretroviral Therapy? http://wwwwhoint/trialsearch/Trial2aspx?TrialID=ACTRN12613000265774. 2013. PubMed PMID: CN-02441114.

940. Actrn. Using a patient-focussed electronic health system for reducing heart disease risk in people with cardiovascular disease. https://trialsearchwhoint/Trial2aspx?TrialID=ACTRN12613000715774. 2013. PubMed PMID: CN-01876979.

941. Actrn. THE HEP573 STUDY: a randomised, double-blind, placebo-controlled clinical trial of silymarin alone, and silymarin combined with antioxidants in chronic hepatitis C. http://wwwwhoint/trialsearch/Trial2aspx?TrialID=ACTRN12614000966695. 2014. PubMed PMID: CN-02441702.

942. Actrn. Impact of standard communication and antimicrobial stewardship on time to appropriate antimicrobial therapy in blood stream infections. http://wwwwhoint/trialsearch/Trial2aspx?TrialID=ACTRN12614000258651. 2014. PubMed PMID: CN-02440581.

943. Actrn. An interdisciplinary model of care for early detection of lung damage, smoking cessation support and management of chronic obstructive pulmonary disease (COPD). https://trialsearchwhoint/Trial2aspx?TrialID=ACTRN12614001155684. 2014. PubMed PMID: CN-01854775.

944. Actrn. A new combination therapy for the treatment of type 2 diabetes mellitus. http://wwwwhoint/trialsearch/Trial2aspx?TrialID=ACTRN12614000836639. 2014. PubMed PMID: CN-02441804.

945. Actrn. Optimum Thiamine Intervention for Treatment and Prevention of Wernicke-Korsakoff Syndrome (WKS): a Randomised Controlled Trial. https://trialsearchwhoint/Trial2aspx?TrialID=ACTRN12614000327684. 2014. PubMed PMID: CN-01819020.

946. Actrn. A prospective randomised double-blind, double-dummy, placebo-controlled crossover study to determine whether Glucagon-like peptide-1 (GLP-1) stimulates or suppresses pancreatic exocrine function in health. https://trialsearchwhoint/Trial2aspx?TrialID=ACTRN12614000815662. 2014. PubMed PMID: CN-01799323.

947. Actrn. A Study to Compare Loperimide to Placebo for the Prevention of Excess Fluid Losses From the Stoma in Patients with New Loop Ileostomy. https://trialsearchwhoint/Trial2aspx?TrialID=ACTRN12614000180617. 2014. PubMed PMID: CN-01818443.

948. Actrn. 5% Aciclovir or Honevo(Trademark) as a treatment for cold sores. http://wwwwhoint/trialsearch/Trial2aspx?TrialID=ACTRN12615000648527. 2015. PubMed PMID: CN-02441416.

949. Actrn. The benefit of targeted pharmacist education in reducing prescribing errors by junior doctors – a controlled trial. http://wwwwhoint/trialsearch/Trial2aspx?TrialID=ACTRN12615000384550. 2015. PubMed PMID: CN-02441185.

950. Actrn. A clinical research study that is testing whether Penthrox (methoxyflurane) plus local anaesthetic reduces pain and discomfort for men having their first transrectal ultrasound-guided prostate biopsy compared to local anaesthetic alone. https://trialsearchwhoint/Trial2aspx?TrialID=ACTRN12615001105538. 2015. PubMed PMID: CN-01820054.

951. Actrn. A conversation with patients about medications after a stroke. http://wwwwhoint/trialsearch/Trial2aspx?TrialID=ACTRN12615000888561. 2015. PubMed PMID: CN-02433309.

952. Actrn. The effects of acute exercise on insulin sensitivity, bone metabolism and cardiovascular risk factors in healthy young men treated with a single dose of prednisolone. http://wwwwhoint/trialsearch/Trial2aspx?TrialID=ACTRN12615000755538. 2015. PubMed PMID: CN-02442329.

953. Actrn. The Effects of Regular Peak Flow Meter Utilisation on Asthma Self-Management. https://trialsearchwhoint/Trial2aspx?TrialID=ACTRN12615000109505. 2015. PubMed PMID: CN-01820228.

954. Actrn. Efficacy of Dexamethasone in reducing pain, nausea and vomiting, improve mobilisation and reduce hospital stay in hip and knee arthroplasty: a double blind controlled trial. http://wwwwhoint/trialsearch/Trial2aspx?TrialID=ACTRN12615000540516. 2015. PubMed PMID: CN-02432376.

955. Actrn. The Efficacy of Sodium Benzoate as an Adjunctive Treatment in Early Psychosis. http://wwwwhoint/trialsearch/Trial2aspx?TrialID=ACTRN12615000187549. 2015. PubMed PMID: CN-02441036.

956. Actrn. Evaluation of donepezil transdermal delivery system (TDS) formulations versus oral donezepil (aricept) in healthy volunteers. http://wwwanzctrorgau/ACTRN12615000925549aspx. 2015. PubMed PMID: CN-01308075.

957. Actrn. A phase II double-blind randomised, placebo-controlled clinical trial of oral nitazoxanide for the treatment of bronchiolitis in infants presenting to hospital emergency departments. https://trialsearchwhoint/Trial2aspx?TrialID=ACTRN12615000590561. 2015. PubMed PMID: CN-01837921.

958. Actrn. A pilot study of hypertonic saline in children with chest infections and disabilities. http://wwwwhoint/trialsearch/Trial2aspx?TrialID=ACTRN12615000766516. 2015. PubMed PMID: CN-02433219.

959. Actrn. Preventing falls in older people after discharge from hospital as a result of a fall. http://wwwwhoint/trialsearch/Trial2aspx?TrialID=ACTRN12615001326583. 2015. PubMed PMID: CN-02440864.

960. Actrn. A Randomised Phase II study of Veliparib, Radiotherapy and Temozolomide in patients with unmethylated O (6)-methylguanine-DNA methyltransferase (MGMT) Glioblastoma (brain cancer) (VERTU study). https://trialsearchwhoint/Trial2aspx?TrialID=ACTRN12615000407594. 2015. PubMed PMID: CN-01811973.

961. Actrn. Cannabidiol (CBD) for Cannabis and Mood Disorders in Adolescence (CCAMDA). http://wwwwhoint/trialsearch/Trial2aspx?TrialID=ACTRN12616001001482. 2016. PubMed PMID: CN-02432772.

962. Actrn. Cough Reflex Testing following Stroke: development of a clinical pathway. http://wwwwhoint/trialsearch/Trial2aspx?TrialID=ACTRN12616000724471. 2016. PubMed PMID: CN-02440379.

963. Actrn. DEFROST: a clinical trial testing metformin as a treatment for frozen shoulder or shoulder tendinopathy. http://wwwwhoint/trialsearch/Trial2aspx?TrialID=ACTRN12616000629437. 2016. PubMed PMID: CN-02441911.

964. Actrn. The effect of Colchicine on Cardiovascular Outcomes in Acute Coronary Syndrome Study (The COLCARDIO-ACS Study). http://wwwwhoint/trialsearch/Trial2aspx?TrialID=ACTRN12616000400460. 2016. PubMed PMID: CN-02443297.

965. Actrn. The effect of colloidal silver in chronic rhinosinusitis. http://wwwwhoint/trialsearch/Trial2aspx?TrialID=ACTRN12616001558415. 2016. PubMed PMID: CN-02441239.

966. Actrn. The effect of dexmedetomidine given as a premedication or intraoperatively on post-hospitalisation behavioural change in children: a randomised controlled trial. https://trialsearchwhoint/Trial2aspx?TrialID=ACTRN12616000096459. 2016. PubMed PMID: CN-01816724.

967. Actrn. Effect of extra intravenous (IV) nutrition compared to standard IV nutrition on growth in moderately preterm babies. https://trialsearchwhoint/Trial2aspx?TrialID=ACTRN12616000925448. 2016. PubMed PMID: CN-01851344.

968. Actrn. Efficacy of health promotion interventions in adults with lifestyle and health risk factors for dementia. http://wwwanzctrorgau/ACTRN12616000868482aspx. 2016. PubMed PMID: CN-01308100.

969. Actrn. Evaluation of Gold Coast Integrated Care for patients with chronic disease through a non-randomised controlled clinical trial. https://trialsearchwhoint/Trial2aspx?TrialID=ACTRN12616000821493. 2016. PubMed PMID: CN-01878533.

970. Actrn. Evaluation of Partnered Pharmacist Charting at Admission and Discharge. http://wwwwhoint/trialsearch/Trial2aspx?TrialID=ACTRN12616001034426. 2016. PubMed PMID: CN-02443595.

971. Actrn. A glass of beetroot juice a day beats the exercise blues? http://wwwwhoint/trialsearch/Trial2aspx?TrialID=ACTRN12616001608459. 2016. PubMed PMID: CN-02440941.

972. Actrn. Integrated Care for Atrial Fibrillation Management: a Randomised Controlled Trial. https://trialsearchwhoint/Trial2aspx?TrialID=ACTRN12616001109493. 2016. PubMed PMID: CN-01807577.

973. Actrn. INtegrated combination Therapy, Electronic General practice support tool, phaRmacy led

intervention And combination Therapy Evaluation (INTEGRATE): an integrated general practice and pharmacy-based intervention to promote the prescription and use of appropriate preventive medications among individuals at high cardiovascular risk. https://trialsearchwhoint/Trial2aspx?TrialID=ACTRN12616000233426. 2016. PubMed PMID: CN-01829811.

974. Actrn. Measuring the effectiveness and safety of electronic medication management systems in paediatric hospitals. https://trialsearchwhoint/Trial2aspx?TrialID=ACTRN12616001452482. 2016. PubMed PMID: CN-01841659.

975. Actrn. Medication Management and Medication-Related Problems in People with Chronic Liver Disease. http://wwwwhoint/trialsearch/Trial2aspx?TrialID=ACTRN12616000780459. 2016. PubMed PMID: CN-02440319.

976. Actrn. Older people in retirement villages: unidentified need & intervention research. http://wwwwhoint/trialsearch/Trial2aspx?TrialID=ACTRN12616000685415. 2016. PubMed PMID: CN-02441255.

977. Actrn. Pharmacist involvement in helping patients at discharge from hospital and the effect on their wellbeing and readmission to hospital. https://trialsearchwhoint/Trial2aspx?TrialID=ACTRN12616001474448. 2016. PubMed PMID: CN-01807893.

978. Actrn. The Pharmacy Diabetes Screening Trial: a comparison of three community pharmacy based approaches to screening for type 2 diabetes on proportions of newly diagnosed type 2 diabetes cases. http://wwwwhoint/trialsearch/Trial2aspx?TrialID=ACTRN12616001240437. 2016. PubMed PMID: CN-02439946.

979. Actrn. A phase I pharmacokinetic evaluation of oral arsenic trioxide in previously untreated patients with acute promyelocytic leukaemia. http://wwwwhoint/trialsearch/Trial2aspx?TrialID=ACTRN12616001022459. 2016. PubMed PMID: CN-02433300.

980. Actrn. Phase II of The Sildenafil during Coronary artery bypass graft Operations to Reduce Endpoints for patients with Coronary ARtery Disease (SCORECARD) Project. http://wwwwhoint/trialsearch/Trial2aspx?TrialID=ACTRN12616001133426. 2016. PubMed PMID: CN-02443537.

981. Actrn. PREVARID - PREVention of Acute Respiratory Infections with Vitamin D. https://trialsearchwhoint/Trial2aspx?TrialID=ACTRN12616000659404. 2016. PubMed PMID: CN-01881506.

982. Actrn. Quadruple UltrA-low-dose tReaTment for hypErTension - QUARTET. https://trialsearchwhoint/Trial2aspx?TrialID=ACTRN12616001144404. 2016. PubMed PMID: CN-01853736.

983. Actrn. A Randomised Phase III Double-Blind Placebo-Controlled Study of regorafenib in Refractory Advanced Gastro-Oesophageal Cancer (AGOC). https://trialsearchwhoint/Trial2aspx?TrialID=ACTRN12616000420448. 2016. PubMed PMID: CN-01874073.

984. Actrn. A randomized trial of varenicline (Champix) for the treatment of cognitive and affective symptoms in Huntington's Disease. https://trialsearchwhoint/Trial2aspx?TrialID=ACTRN12616001611415. 2016. PubMed PMID: CN-01851476.

985. Actrn. Safety and Efficacy study of CRD-102 in patients with Heart Failure with Preserved Ejection Fraction. http://wwwwhoint/trialsearch/Trial2aspx?TrialID=ACTRN12616000619448. 2016. PubMed PMID: CN-02441774.

986. Actrn. Short term effects of supplementing the amino acid, leucine during weight loss on improvements in body composition and insulin sensitivity in individuals at risk of the metabolic syndrome. http://wwwwhoint/trialsearch/Trial2aspx?TrialID=ACTRN12616001528448. 2016. PubMed PMID: CN-02432201.

987. Actrn. Topical Gabapentin Amitriptyline and Lignocaine (ToGA) ointment trial for post-haemorrhoidectomy analgesia. https://trialsearchwhoint/Trial2aspx?TrialID=ACTRN12616001477415. 2016. PubMed PMID: CN-01870723.

988. Actrn. Tranexamic acid use in Total Hip Replacement - beyond blood loss: a randomised clinical trial of efficacy of tranexamic acid in improving postoperative inpatient functional outcomes following anterior total hip replacement. https://trialsearchwhoint/Trial2aspx?TrialID=ACTRN12616000606482. 2016. PubMed PMID: CN-01844527.

989. Actrn. Treatment of Adolescents with Depression (TRACED): a pilot study on predictors for treatment response. http://wwwwhoint/trialsearch/Trial2aspx?TrialID=ACTRN12616001561471. 2016. PubMed PMID: CN-02443263.

990. Actrn. Treatment of Melasma with Oral Tranexamic Acid. https://trialsearchwhoint/Trial2aspx?TrialID=ACTRN12616000548437. 2016. PubMed PMID: CN-01815958.

991. Actrn. Varenicline alone or with nicotine e-cigarettes for smoking cessation in people with mental illness and drug and/or alcohol dependence. https://trialsearchwhoint/Trial2aspx?TrialID=ACTRN12616001355460. 2016. PubMed PMID: CN-01884207.

992. Actrn. VERBATIM: a randomised controlled trial of aripiprazole for the treatment of auditory verbal hallucinations in borderline personality disorder. https://trialsearchwhoint/Trial2aspx?TrialID=ACTRN12616001192471. 2016. PubMed PMID: CN-01859118.

993. Actrn. Can an allied health and nursing expanded scope Treatment Access Pathway (TAP) improve health outcomes for people with persistent pain? A pragmatic randomised controlled trial. https://trialsearchwhoint/Trial2aspx?TrialID=ACTRN12617001284358. 2017. PubMed PMID: CN-01884299.

994. Actrn. Comparing Hiprex tablets and Nitrofurantoin in women with recurrent urine infections. https://trialsearchwhoint/Trial2aspx?TrialID=ACTRN12617000585325. 2017. PubMed PMID: CN-01885035.

995. Actrn. Comparing home infusion devices for antibiotic treatment - CHID Study. https://trialsearchwhoint/Trial2aspx?TrialID=ACTRN12617000251325. 2017. PubMed PMID: CN-01857139.

996. Actrn. Comparison between ketamine and dexmedetomidine as adjuvants in Pectoral Nerves Regional Blocks for perioperative pain control for patients undergoing breast cancer surgery. https://trialsearchwhoint/Trial2aspx?TrialID=ACTRN12617000851369. 2017. PubMed PMID: CN-01891783.

997. Actrn. Does naltrexone reduce pain or disability after a whiplash? http://wwwwhoint/trialsearch/Trial2aspx?TrialID=ACTRN12617000274370. 2017. PubMed PMID: CN-02443264.

998. Actrn. Effect of iodine dye injected into long central venous catheter, on Thyroid Function in Premature Infants. http://wwwwhoint/trialsearch/Trial2aspx?TrialID=ACTRN12617000826347. 2017. PubMed PMID: CN-02441264.

999. Actrn. Effect of liraglutide on weight loss in patients with inadequate weight loss following bariatric surgery. https://trialsearchwhoint/Trial2aspx?TrialID=ACTRN12617001613392. 2017. PubMed PMID: CN-01886076.

1000. Actrn. Effectiveness of Education Training Program in Preventing Needle Stick Injury among Healthcare Workers in Health Clinics in Kuala Lumpur. http://wwwwhoint/trialsearch/Trial2aspx?TrialID=ACTRN12617001062314. 2017. PubMed PMID: CN-02443240.

1001. Actrn. Effectiveness of pharmacist based educational program on glycaemic control, self-care activities and disease knowledge in type 2 diabetes patients in Pakistan. https://trialsearchwhoint/Trial2aspx?TrialID=ACTRN12617001327370. 2017. PubMed PMID: CN-01886853.

1002. Actrn. Efficacy of implementation of the Goal-directed Medication review Electronic Decision Support System (G-MEDSS) into Home Medicines Review (HMR) to deprescribe medications in older adults. https://trialsearchwhoint/Trial2aspx?TrialID=ACTRN12617000895381. 2017. PubMed PMID: CN-01894029.

1003. Actrn. The efficacy of Metformin as an adjunctive treatment to attenuate weight gain and metabolic syndrome in patients with schizophrenia or schizoaffective disorder newly commenced on clozapine. http://wwwwhoint/trialsearch/Trial2aspx?TrialID=ACTRN12617001547336. 2017. PubMed PMID: CN-02439847.

1004. Actrn. A feasibility trial comparing a behavioural intervention, melatonin and usual care for people with dementia and sleep disturbance. http://wwwwhoint/trialsearch/Trial2aspx?TrialID=ACTRN12617000056392. 2017. PubMed PMID: CN-02441978.

1005. Actrn. Food 4 Health: using probiotics and prebiotic cereals to reduce blood sugar levels in adults at risk of developing type 2 diabetes He Oranga Kai. http://wwwwhoint/trialsearch/Trial2aspx?TrialID=ACTRN12617000990325. 2017. PubMed PMID: CN-02433480.

1006. Actrn. How a sleep clinical pathway in an inpatient rehabilitation centre may help with sleep and recovery. https://trialsearchwhoint/Trial2aspx?TrialID=ACTRN12617000353392. 2017. PubMed PMID: CN-01871667.

1007. Actrn. Impact of clinical pharmacist medication review on appropriate prescribing in elderly patients: a randomized, controlled trial. https://trialsearchwhoint/Trial2aspx?TrialID=ACTRN12617000665336. 2017. PubMed PMID: CN-01887226.

1008. Actrn. Intra-nasal fentanyl for breathlessness. https://trialsearchwhoint/Trial2aspx?TrialID=ACTRN12617001399381. 2017. PubMed PMID: CN-01894136.

1009. Actrn. Is shorter duration cardiac rehabilitation including progressive resistance training more effective than standard-care cardiac rehabilitation? http://wwwwhoint/trialsearch/Trial2aspx?TrialID=ACTRN12617000755336. 2017. PubMed PMID: CN-02440157.

1010. Actrn. Lidocaine (lignocaine) for Neuropathic Cancer Pain – Feasibility study (LiCPain). https://trialsearchwhoint/Trial2aspx?TrialID=ACTRN12617000747325. 2017. PubMed PMID: CN-01888763.

1011. Actrn. Lisdexamfetamine for the treatment of methamphetamine addiction. https://trialsearchwhoint/Trial2aspx?TrialID=ACTRN12617000657325. 2017. PubMed PMID: CN-01893339.

1012. Actrn. Mass media campaign material designed to prevent youth suicide: a randomised controlled trial. https://trialsearchwhoint/Trial2aspx?TrialID=ACTRN12617000043336. 2017. PubMed PMID: CN-01803806.

1013. Actrn. Optimising primary care management of knee osteoarthritis: the PARTNER project. http://wwwwhoint/trialsearch/Trial2aspx?TrialID=ACTRN12617001595303. 2017. PubMed PMID: CN-02443465.

1014. Actrn. A pilot study to investigate the impact of a new pharmacy service on new medicine adherence. http://wwwwhoint/trialsearch/Trial2aspx?TrialID=ACTRN12617000332325. 2017. PubMed PMID: CN-02433498.

1015. Actrn. Pregnancy outcomes when adding a progesterone pessary dose amongst women who are

receiving frozen embryo transfer with hormone replacement therapy, compared with placebo. https://trialsearchwhoint/Trial2aspx?TrialID=ACTRN12617001016325. 2017. PubMed PMID: CN-01892333.

1016. Actrn. Preventing Muscle Cramps in Individuals on Dialysis. http://wwwwhoint/trialsearch/Trial2aspx?TrialID=ACTRN12617000164392. 2017. PubMed PMID: CN-02442074.

1017. Actrn. “Quercetin as an augmentation agent in Treatment Resistant Schizophrenia”. http://wwwwhoint/trialsearch/Trial2aspx?TrialID=ACTRN12617000207314. 2017. PubMed PMID: CN-02443561.

1018. Actrn. Routes of Dexamethasone Administration during Wisdom Teeth Removal. http://wwwwhoint/trialsearch/Trial2aspx?TrialID=ACTRN12617001203347. 2017. PubMed PMID: CN-02442822.

1019. Actrn. SImplification of Medications Prescribed to Long tErm care Residents (SIMPLER). https://trialsearchwhoint/Trial2aspx?TrialID=ACTRN12617001060336. 2017. PubMed PMID: CN-01886034.

1020. Actrn. Tramadol/Paracetamol (Zaldiar) compared with oxycodone and paracetamol for the management of post-operative pain following major surgery in gynaecology oncology patients. http://wwwwhoint/trialsearch/Trial2aspx?TrialID=ACTRN12617001341314. 2017. PubMed PMID: CN-02442529.

1021. Actrn. 3% Kanuka Oil Cream for the Topical Treatment of Eczema. https://trialsearchwhoint/Trial2aspx?TrialID=ACTRN12618001754235. 2018. PubMed PMID: CN-01946992.

1022. Actrn. Adjuvant N Acetylcysteine for Post Traumatuic Stress Disorder. https://trialsearchwhoint/Trial2aspx?TrialID=ACTRN12618001784202. 2018. PubMed PMID: CN-01948034.

1023. Actrn. The Australian Team Approach to Polypharmacy Evaluation and Reduction (AusTAPER) study for older hospital inpatients. http://wwwwhoint/trialsearch/Trial2aspx?TrialID=ACTRN12618000979257. 2018. PubMed PMID: CN-02444911.

1024. Actrn. Comparing treatment outcomes of Adenoidectomy against medical therapies for mild obstructive sleep apnoea in children. https://trialsearchwhoint/Trial2aspx?TrialID=ACTRN12618001093279. 2018. PubMed PMID: CN-01909853.

1025. Actrn. Deprescribing in older community patients. https://trialsearchwhoint/Trial2aspx?TrialID=ACTRN12618000729224. 2018. PubMed PMID: CN-01896779.

1026. Actrn. Does a peer-supported lifestyle modification program improve health outcomes and satisfaction with care for obese patients. http://wwwwhoint/trialsearch/Trial2aspx?TrialID=ACTRN12618000624280. 2018. PubMed PMID: CN-02441180.

1027. Actrn. Does cannabidiol reduce severe behavioural problems in youth with intellectual disability? Feasibility and pilot randomised placebo-controlled trial. https://trialsearchwhoint/Trial2aspx?TrialID=ACTRN12618001852246. 2018. PubMed PMID: CN-01949171.

1028. Actrn. Does removing prescription charges reduce hospital stays for people with low incomes and high health needs? https://trialsearchwhoint/Trial2aspx?TrialID=ACTRN12618001486213. 2018. PubMed PMID: CN-01909343.

1029. Actrn. A double blind, randomized, placebo controlled, cross-over trial investigating the effect of High Amylose Maize Starch (HAMS) supplementation on fecal microbiological and inflammatory outcomes in individuals with Cystic Fibrosis and Healthy Volunteers. https://trialsearchwhoint/Trial2aspx?TrialID=ACTRN12618000283279. 2018. PubMed PMID: CN-01897308.

1030. Actrn. Economic evaluation and clinical impact of home medication review (HMR) by community pharmacists among patients with type 2 diabetes mellitus (T2DM). http://wwwwhoint/trialsearch/Trial2aspx?TrialID=ACTRN12618000839202. 2018. PubMed PMID: CN-02433040.

1031. Actrn. The effect of a common antibiotic (amoxicillin with clavulanate) on the microbiome of tonsil tissue in children with recurrent tonsillitis. https://trialsearchwhoint/Trial2aspx?TrialID=ACTRN12618001847202. 2018. PubMed PMID: CN-01946942.

1032. Actrn. Effect of electronic fetal monitoring method used during labour on emergency caesarean section rates: START (STan Australian Randomised Trial). https://trialsearchwhoint/Trial2aspx?TrialID=ACTRN12618000086268. 2018. PubMed PMID: CN-01898140.

1033. Actrn. Effectiveness of Activated B-vitamins compared with Standard B-vitamins in easing stress and fatigue in students. https://trialsearchwhoint/Trial2aspx?TrialID=ACTRN12618001400257. 2018. PubMed PMID: CN-01905328.

1034. Actrn. Enhanced intervention to improve medication adherence in a community pharmacy setting. https://trialsearchwhoint/Trial2aspx?TrialID=ACTRN12618001105235. 2018. PubMed PMID: CN-01908905.

1035. Actrn. Evaluation of a community pharmacist-led minor ailments service in collaboration with general practitioners: a cluster randomised controlled trial. https://trialsearchwhoint/Trial2aspx?TrialID=ACTRN12618000286246. 2018. PubMed PMID: CN-01900802.

1036. Actrn. An evaluation of an 'analgesia prescribing' education module for junior doctors and pharmacists: impact on opioid prescribing at discharge. http://wwwwhoint/trialsearch/Trial2aspx?TrialID=ACTRN12618000876291. 2018. PubMed PMID: CN-02443374.

1037. Actrn. Evaluation Of An Antiretroviral therapy adherence intervention by pharmacist among HIV Infected Patients. https://trialsearchwhoint/Trial2aspx?TrialID=ACTRN12618001882213. 2018. PubMed PMID: CN-01950284.

1038. Actrn. General Practitioner (GP) education and screening feedback for improving depression outcomes among primary care patients. http://wwwwhoint/trialsearch/Trial2aspx?TrialID=ACTRN12618001139268. 2018. PubMed PMID: CN-02445144.

1039. Actrn. Health and economic impacts associated with prescribing by expanded scope physiotherapists for patients with spinal pain? https://trialsearchwhoint/Trial2aspx?TrialID=ACTRN12618001443280. 2018. PubMed PMID: CN-01906828.

1040. Actrn. Improving quality of life, physical and psychological issue by using chemotherapy counselling among cancer patients in Malaysia. http://wwwwhoint/trialsearch/Trial2aspx?TrialID=ACTRN12618001345279. 2018. PubMed PMID: CN-02445012.

1041. Actrn. Medication adherence management in a community pharmacy setting (AdherenciaMED): a cluster randomized controlled trial. https://trialsearchwhoint/Trial2aspx?TrialID=ACTRN12618000410257. 2018. PubMed PMID: CN-01905516.

1042. Actrn. The Medicines Management Mapping Project: using risk stratification and care coordination to bridge the care continuum gap. http://wwwwhoint/trialsearch/Trial2aspx?TrialID=ACTRN12618001168246. 2018. PubMed PMID: CN-02444980.

1043. Actrn. Midodrine as an Adjunctive VasoprEssor for Refractory Hypotension in Intensive Care

(MAVERIC) Study. https://trialsearchwhoint/Trial2aspx?TrialID=ACTRN12618001158257. 2018. PubMed PMID: CN-01909378.

1044. Actrn. Mums on the Move Study: a pilot randomised controlled trial to evaluate the effectiveness of a physical activity program for women at risk of post-natal depression. https://trialsearchwhoint/Trial2aspx?TrialID=ACTRN12618001453279. 2018. PubMed PMID: CN-01905453.

1045. Actrn. Oral prednisolone for acute middle ear infection in children. https://trialsearchwhoint/Trial2aspx?TrialID=ACTRN12618000049279. 2018. PubMed PMID: CN-01900156.

1046. Actrn. Paramedic administered antibiotics for patients with severe sepsis: does this reduce the time to appropriate antibiotic therapy? http://wwwwhoint/trialsearch/Trial2aspx?TrialID=ACTRN12618000199213. 2018. PubMed PMID: CN-02443634.

1047. Actrn. PENtoxyphylline+TOcopherol+/-CLOdronate (PENTOCLO) vs Hyperbaric oxygen (HBO): a randomised assessor blinded pilot study comparing two therapies for Osteoradionecrosis of the mandible. https://trialsearchwhoint/Trial2aspx?TrialID=ACTRN12618001099213. 2018. PubMed PMID: CN-01905561.

1048. Actrn. Pharmacist service to reduce adverse events in residential aged care facilities. http://wwwwhoint/trialsearch/Trial2aspx?TrialID=ACTRN12618000766213. 2018. PubMed PMID: CN-02440912.

1049. Actrn. Pharmacy Trial Program (PTP)- Getting asthma under control using the skills of the community Pharmacist. http://wwwwhoint/trialsearch/Trial2aspx?TrialID=ACTRN12618000313235. 2018. PubMed PMID: CN-02442799.

1050. Actrn. A Phase 2, Randomized, Double Crossover, Open-Label, Active-Controlled Proof-of-Concept Study of CNSA-001 in Phenylketonuria Patients. https://trialsearchwhoint/Trial2aspx?TrialID=ACTRN12618001031257. 2018. PubMed PMID: CN-01904485.

1051. Actrn. A phase II randomised controlled trial to establish if LEE0011 (ribociclib) is effective in inhibiting the growth of prostate cancer cells. https://trialsearchwhoint/Trial2aspx?TrialID=ACTRN12618000354280. 2018. PubMed PMID: CN-01901206.

1052. Actrn. Randomised, controlled trial evaluating the effectiveness of Probiotic and prawn oral Immunotherapy at inducing desensitisation or tolerance in participants with Prawn Allergy compared with placebo (Probiotic and prawn Immunotherapy for treatment of Prawn Allergy: PIPA study). https://trialsearchwhoint/Trial2aspx?TrialID=ACTRN12618000235202. 2018. PubMed PMID: CN-01902222.

1053. Actrn. Safer Prescribing and Care for the Elderly (SPACE): cluster RCT in New Zealand General Practice. http://wwwwhoint/trialsearch/Trial2aspx?TrialID=ACTRN12618000034235. 2018. PubMed PMID: CN-02441565.

1054. Actrn. SAMSON: trial of a nurse and pharmacist-led mobile health management system. https://trialsearchwhoint/Trial2aspx?TrialID=ACTRN12618001798257. 2018. PubMed PMID: CN-01948084.

1055. Actrn. Single Ascending Dose Study to Evaluate the Safety and Tolerability of LPT99 Administered to Healthy Adult Subjects. https://trialsearchwhoint/Trial2aspx?TrialID=ACTRN12618001461280. 2018. PubMed PMID: CN-01909653.

1056. Actrn. Spray on skin for diabetic foot ulcers: an open label randomised controlled trial. https://trialsearchwhoint/Trial2aspx?TrialID=ACTRN12618000511235. 2018. PubMed PMID: CN-01901000.

1057. Actrn. A Study to Assess the Opioid-Sparing Activity of PAX-1 in Patients with Persistent Cancer Pain. https://trialsearchwhoint/Trial2aspx?TrialID=ACTRN12618001304224. 2018. PubMed PMID: CN-01909647.

1058. Actrn. A study to evaluate the Safety and Pharmacokinetics (Pharmacokintecus, the measure of how the human body processes a substance) of ETX0282 when administered orally to healthy participants. https://trialsearchwhoint/Trial2aspx?TrialID=ACTRN12618000350224. 2018. PubMed PMID: CN-01899246.

1059. Actrn. Targeted thromboprophylaxis in ambulatory patients receiving anticancer therapies (TARGET-TP). https://trialsearchwhoint/Trial2aspx?TrialID=ACTRN12618000811202. 2018. PubMed PMID: CN-01900501.

1060. Actrn. 'TEXT4myBACK' text message intervention to improve pain and disability in people with low back pain. http://wwwwhoint/trialsearch/Trial2aspx?TrialID=ACTRN12618001263280. 2018. PubMed PMID: CN-02445303.

1061. Actrn. A topical cold sore treatment containing St John's Wort, Calendula and Copper. https://trialsearchwhoint/Trial2aspx?TrialID=ACTRN12618000890235. 2018. PubMed PMID: CN-01900371.

1062. Actrn. 3% Kanuka Oil Serum for the Topical Treatment of Acne. https://trialsearchwhoint/Trial2aspx?TrialID=ACTRN12619001744145. 2019. PubMed PMID: CN-02065056.

1063. Actrn. Antiarrhythmic effects of phenytoin or dantrolene in patients with cardiomyopathy. https://trialsearchwhoint/Trial2aspx?TrialID=ACTRN12619000730101. 2019. PubMed PMID: CN-01972883.

1064. Actrn. Anticoagulant (Apixaban Vs Warfarin) in prevention of nephrotic syndrome associated thromboembolism. https://trialsearchwhoint/Trial2aspx?TrialID=ACTRN12619001193167. 2019. PubMed PMID: CN-01972539.

1065. Actrn. Assessment of narcotic administration to lead to analgesic effects and sedation in intensive care. https://trialsearchwhoint/Trial2aspx?TrialID=ACTRN12619000939190. 2019. PubMed PMID: CN-01972312.

1066. Actrn. ATOMIC Ears: a Phase IIB randomised controlled trial to assess safety, tolerability and acceptability of a 5-day Dornase alfa treatment as an adjunct therapy to ventilation tube insertion for otitis media in children. https://trialsearchwhoint/Trial2aspx?TrialID=ACTRN12619001306101. 2019. PubMed PMID: CN-02064800.

1067. Actrn. AusTAPER Dem: team Approach to Polypharmacy Evaluation and Reduction for General Practice patients with dementia: the Australian TAPER Dem study. https://trialsearchwhoint/Trial2aspx?TrialID=ACTRN12619000453189. 2019. PubMed PMID: CN-01972438.

1068. Actrn. Battle Field (Ear) Acupuncture as an adjunct to treat pain of the abdomen, limb trauma and lower back in the emergency department - a pilot study. https://trialsearchwhoint/Trial2aspx?TrialID=ACTRN12619000051145. 2019. PubMed PMID: CN-01947186.

1069. Actrn. Can loss of response to the gut hormone, glucose-dependent insulinotropic polypeptide (GIP), be reversed in type 2 diabetes? https://trialsearchwhoint/Trial2aspx?TrialID=ACTRN12619000305123. 2019. PubMed PMID: CN-01971272.

1070. Actrn. Can providing information about a generic medicine switch reduce side effect reporting? http://wwwwhoint/trialsearch/Trial2aspx?TrialID=ACTRN12619000654156. 2019. PubMed PMID: CN-02434134.

1071. Actrn. A cluster randomised controlled trial of a MedicineInsight educational quality improvement program to improve the diagnosis and treatment of chronic hepatitis C in Australian general practice - the EQUIP-HEPC trial. https://trialsearchwhoint/Trial2aspx?TrialID=ACTRN12619000508178. 2019. PubMed PMID: CN-01973289.

1072. Actrn. Community screening of participants across community pharmacies detecting asymptomatic clinical markers or risk factors associated with cardiovascular disease and chronic disease. https://trialsearchwhoint/Trial2aspx?TrialID=ACTRN12619000405112. 2019. PubMed PMID: CN-01975546.

1073. Actrn. Comparing post-sinus surgery symptoms of chronic sinusitis patients using Flo Sinus Care or Flo Kappa sinus rinses. https://trialsearchwhoint/Trial2aspx?TrialID=ACTRN12619000117112. 2019. PubMed PMID: CN-01951114.

1074. Actrn. DCMedsRec; Reducing hospital readmission by enhanced My Health Record use in Community Pharmacy. An ADHA Digital Test Beds project. https://trialsearchwhoint/Trial2aspx?TrialID=ACTRN12619000282189. 2019. PubMed PMID: CN-01972338.

1075. Actrn. Development and Implementation of Pharmacist-based Diabetic Intervention Model in type-2 diabetes patients in Pakistan: a randomized controlled trial. https://trialsearchwhoint/Trial2aspx?TrialID=ACTRN12619001618145. 2019. PubMed PMID: CN-02064980.

1076. Actrn. Effect of HMB and vitamin D supplementation on osteosarcopenia in older persons. https://trialsearchwhoint/Trial2aspx?TrialID=ACTRN12619000636156. 2019. PubMed PMID: CN-01972244.

1077. Actrn. The Effectiveness of a Personalised Digital Health Intervention (Naluri) on Self-Care Behaviours and Quality of Life Outcomes among Cardiac Patients at the Malaysian National Heart Institute: a Randomized Controlled Trial. https://trialsearchwhoint/Trial2aspx?TrialID=ACTRN12619000104156. 2019. PubMed PMID: CN-01946960.

1078. Actrn. Effects of acute and sub-acute treatment with metformin on cardiovascular responses to a glucose challenge in type 2 diabetes. https://trialsearchwhoint/Trial2aspx?TrialID=ACTRN12619001060134. 2019. PubMed PMID: CN-01972746.

1079. Actrn. Effects of topical and oral corticosteroids on sinonasal microbiome. https://trialsearchwhoint/Trial2aspx?TrialID=ACTRN12619001398190. 2019. PubMed PMID: CN-02064849.

1080. Actrn. Emergence characteristics comparing Endotrachael tube vs Laryngeal Mask airway during nasal surgery with general anaesthesia using intravenous anaesthetic agents like propofol and remifentanyl. https://trialsearchwhoint/Trial2aspx?TrialID=ACTRN12619000495123. 2019. PubMed PMID: CN-01973090.

1081. Actrn. Exploring the impact of attention training experienced by older adults with or without MCI MCI- Mild cognitive impairment. https://trialsearchwhoint/Trial2aspx?TrialID=ACTRN12619000820101. 2019. PubMed PMID: CN-01972716.

1082. Actrn. Impact of post-operative Lidocaine Infusion on GastroIntestinal Tract function in patients undergoing laparoscopic colorectal resections. https://trialsearchwhoint/Trial2aspx?TrialID=ACTRN12619001168145. 2019. PubMed PMID: CN-01972467.

1083. Actrn. Implementing a nurse-coordinated, integrated, shared-care model involving specialists and general practitioners in breast cancer post-treatment follow-up: a Phase II randomised controlled trial. https://trialsearchwhoint/Trial2aspx?TrialID=ACTRN12619001594112. 2019. PubMed PMID: CN-02064963.

1084. Actrn. Management of nausea in cancer patients â€“ Nausea study 4 pilot. https://trialsearchwhoint/

Trial2aspx?TrialID=ACTRN12619000744156. 2019. PubMed PMID: CN-01973830.

1085. Actrn. Preventing Adverse Drug Reactions in Older Australians. https://trialsearchwhoint/Trial2aspx?TrialID=ACTRN12619000729123. 2019. PubMed PMID: CN-01971689.

1086. Actrn. Randomised controlled trial: can topical timolol maleate prevent complications and reduce the need for further treatment for small superficial infantile haemangiomata in high risk areas? https://trialsearchwhoint/Trial2aspx?TrialID=ACTRN12619001685101. 2019. PubMed PMID: CN-02065019.

1087. Actrn. Randomized, Double-Blind, Placebo-Controlled, Single and Multiple Ascending Dose Study to Evaluate the Safety, Tolerability and Pharmacokinetics of KER-047 Administered to Healthy Male Volunteers and Postmenopausal Female Volunteers. https://trialsearchwhoint/Trial2aspx?TrialID=ACTRN12619000319178. 2019. PubMed PMID: CN-02241071.

1088. Actrn. Realising the benefits of clinical pharmacy in the bush: the efficacy and scalability of a virtual clinical pharmacy service (VCPS) in rural and remote New South Wales health facilities. https://trialsearchwhoint/Trial2aspx?TrialID=ACTRN12619001757101. 2019. PubMed PMID: CN-02065062.

1089. Actrn. Shared Team Efforts Leading to Adherence Results (STELAR)â€“ trial of a quality improvement program in acute stroke care. https://trialsearchwhoint/Trial2aspx?TrialID=ACTRN12619001072101. 2019. PubMed PMID: CN-01972310.

1090. Actrn. A Single-Center, Randomized, Double-Blind, Placebo-Controlled, Two-Part, Dose-Escalation Study to Evaluate the Safety, Tolerability, Pharmacokinetic, and Pharmacodynamic Effects of KER-050 Administered to Healthy, Postmenopausal Women. https://trialsearchwhoint/Trial2aspx?TrialID=ACTRN12619000318189. 2019. PubMed PMID: CN-01970145.

1091. Actrn. The STAR-PAD trial is testing a new treatment for Peripheral Arterial Disease (PAD). https://trialsearchwhoint/Trial2aspx?TrialID=ACTRN12619000423112. 2019. PubMed PMID: CN-01971613.

1092. Actrn. Akynzeo for post-operative nausea and vomiting after bariatric surgery. https://trialsearchwhoint/Trial2aspx?TrialID=ACTRN12620000455965. 2020. PubMed PMID: CN-02165270.

1093. Actrn. Bridging the Gap between Physical and Mental Illness in Community Pharmacy (PharMIbridge): randomised Controlled Trial. https://trialsearchwhoint/Trial2aspx?TrialID=ACTRN12620000577910. 2020. PubMed PMID: CN-02165337.

1094. Actrn. Does gabapentin decrease the incidence of postoperative pain in children undergoing tonsillectomy procedures? https://trialsearchwhoint/Trial2aspx?TrialID=ACTRN12620000396921. 2020. PubMed PMID: CN-02165236.

1095. Actrn. Early versus late parenteral nutrition in term and late preterm infants: a Randomized Controlled Trial. https://trialsearchwhoint/Trial2aspx?TrialID=ACTRN12620000324910. 2020. PubMed PMID: CN-02165191.

1096. Actrn. The effect of health information distributed to people with knee osteoarthritis through community pharmacies: a feasibility study. https://trialsearchwhoint/Trial2aspx?TrialID=ACTRN12620000020987. 2020. PubMed PMID: CN-02164998.

1097. Actrn. Effect of topical coconut oil on infection rates in extremely premature infants. https://trialsearchwhoint/Trial2aspx?TrialID=ACTRN12620001332910. 2020. PubMed PMID: CN-02241148.

1098. Actrn. The effects of the education program and other factors on the number of sedative medications

that older people with dementia regularly consume. https://trialsearchwhoint/Trial2aspx?TrialID=ACTRN12620000268943. 2020. PubMed PMID: CN-02165154.

1099. Actrn. Evaluating new, rapid point of care testing for hepatitis C in injecting drug users visiting a medically supervised injecting room. http://wwwwhoint/trialsearch/Trial2aspx?TrialID=ACTRN12620000526976. 2020. PubMed PMID: CN-02446608.

1100. Actrn. Evaluating the role of perhexiline (new medication treatment) in patients with abnormally thickened heart muscle (hypertrophic cardiomyopathy). https://trialsearchwhoint/Trial2aspx?TrialID=ACTRN12620000785909. 2020. PubMed PMID: CN-02184362.

1101. Actrn. Evaluation of the effect of tailored frailty interventions aimed at addressing frailty in older people: frailty in Older people, Rehabilitation Treatment, Research Examining Separate Settings - The FORTRESS study. https://trialsearchwhoint/Trial2aspx?TrialID=ACTRN12620000760976. 2020. PubMed PMID: CN-02184350.

1102. Actrn. Hydroxychloroquine for the Community-Based Treatment of COVID-19. https://trialsearchwhoint/Trial2aspx?TrialID=ACTRN12620000457943. 2020. PubMed PMID: CN-02165272.

1103. Actrn. Integrating pharmacists in residential aged care facilities to improve quality use of medicine. https://trialsearchwhoint/Trial2aspx?TrialID=ACTRN12620000430932. 2020. PubMed PMID: CN-02165256.

1104. Actrn. Paravertebral vs. Erector Spinae Blocks for lung function in those with rib fractures. https://trialsearchwhoint/Trial2aspx?TrialID=ACTRN12620001192976. 2020. PubMed PMID: CN-02238976.

1105. Actrn. PROtein dose and Muscle Synthesis in critically ill patients (PROMS). https://trialsearchwhoint/Trial2aspx?TrialID=ACTRN12620000776909. 2020. PubMed PMID: CN-02184357.

1106. Actrn. A Randomised, Double-Blind, Placebo Controlled Feasibility Study of Oral Lorazepam for Symptoms of Anxiety in Patients with Advanced Life-Limiting Disease. https://trialsearchwhoint/Trial2aspx?TrialID=ACTRN12620001143910. 2020. PubMed PMID: CN-02184563.

1107. Actrn. A randomized double-blind placebo-controlled trial of oral ivermectin for outpatient treatment of those at high risk for hospitalization due to COVID-19. https://trialsearchwhoint/Trial2aspx?TrialID=ACTRN12620000982910. 2020. PubMed PMID: CN-02184467.

1108. Actrn. Study of a vaginal treatment in preventing Recurrent Vulvovaginal Candidiasis (thrush). https://trialsearchwhoint/Trial2aspx?TrialID=ACTRN12620001084976. 2020. PubMed PMID: CN-02184529.

1109. Actrn. Team Approach to Polypharmacy Evaluation and Reduction for General Practice patients: the Australian TAPER (AusTAPER) study. https://trialsearchwhoint/Trial2aspx?TrialID=ACTRN12620001169932. 2020. PubMed PMID: CN-02184580.

1110. Actrn. A Telehealth Cancer-Related Fatigue Clinic Model for Cancer Survivors: a Pilot Randomised Controlled Trial (The T-CRF Trial). https://trialsearchwhoint/Trial2aspx?TrialID=ACTRN12620001334998. 2020. PubMed PMID: CN-02238210.

1111. Actrn. The Vietnam COPD Asthma and Prevention of Smoking (VCAPS) 4 Trial. https://trialsearchwhoint/Trial2aspx?TrialID=ACTRN12620000649910. 2020. PubMed PMID: CN-02165375.

1112. Actrn. Can Caffeine prevent the cessation of breathing in preterm newborn babies? https://trialsearchwhoint/Trial2aspx?TrialID=ACTRN12621001000897. 2021. PubMed PMID: CN-02327162.

1113. Actrn. Cannabidiol for At Risk for psychosis Youth. https://trialsearchwhoint/Trial2aspx?TrialID=ACTRN12621000349842. 2021. PubMed PMID: CN-02255599.

1114. Actrn. Cardiac Rehabilitation for transient ischaemic Attack and Mild-Stroke: the CRAMS randomised controlled trial. https://trialsearchwhoint/Trial2aspx?TrialID=ACTRN12621001586808. 2021. PubMed PMID: CN-02349114.

1115. Actrn. Care to Quit: an intervention for people with cancer who smoke. https://trialsearchwhoint/Trial2aspx?TrialID=ACTRN12621000154808. 2021. PubMed PMID: CN-02240843.

1116. Actrn. Clozapine Obesity and Semaglutide Treatment (COaST). A randomised controlled multi-centre trial of semaglutide versus placebo for people with schizophrenia on clozapine with obesity inadequately responsive to metformin. https://trialsearchwhoint/Trial2aspx?TrialID=ACTRN12621001539820. 2021. PubMed PMID: CN-02349090.

1117. Actrn. Comparing Oral and Sublingual Ketamine Lozenges as Rescue Analgesics in Adults with Acute Pain. https://trialsearchwhoint/Trial2aspx?TrialID=ACTRN12621000240842. 2021. PubMed PMID: CN-02257223.

1118. Actrn. Does vitamin C decrease post-operative pain in surgery to the foot and ankle? https://trialsearchwhoint/Trial2aspx?TrialID=ACTRN12621000812897. 2021. PubMed PMID: CN-02282943.

1119. Actrn. Dose finding clinical trial of sodium benzoate in people with treatment refractory schizophrenia. https://trialsearchwhoint/Trial2aspx?TrialID=ACTRN12621000327886. 2021. PubMed PMID: CN-02255798.

1120. Actrn. ECMT-154™ for the Topical Treatment of Eczema. https://trialsearchwhoint/Trial2aspx?TrialID=ACTRN12621001096842. 2021. PubMed PMID: CN-02327209.

1121. Actrn. The effect of a paracervical block prior to total laparoscopic hysterectomy on patient's quality of recovery. https://trialsearchwhoint/Trial2aspx?TrialID=ACTRN12621000584831. 2021. PubMed PMID: CN-02282632.

1122. Actrn. The effect of clonidine on pain post-tonsillectomy in children. https://trialsearchwhoint/Trial2aspx?TrialID=ACTRN12621001606875. 2021. PubMed PMID: CN-02349125.

1123. Actrn. Effect of Ivabradine use on exercise capacity in patients with transposition of the great arteries (TGA) post atrial switch repair. https://trialsearchwhoint/Trial2aspx?TrialID=ACTRN12621000196842. 2021. PubMed PMID: CN-02238018.

1124. Actrn. Effect of pharmacist involvement in Diabetes Medication Therapy Adherence Program (DMTAC) on the clinical outcomes of diabetes in two different hospitals of Kedah state Malaysia. https://trialsearchwhoint/Trial2aspx?TrialID=ACTRN12621001128886. 2021. PubMed PMID: CN-02327227.

1125. Actrn. Effect of Probiotic supplementation on gut bacteria in children with autism - a pilot randomized controlled trial. https://trialsearchwhoint/Trial2aspx?TrialID=ACTRN12621000029897. 2021. PubMed PMID: CN-02241654.

1126. Actrn. A Feasibility Trial of Tranexamic Acid for Necrotising Soft-tissue Infections. https://trialsearchwhoint/Trial2aspx?TrialID=ACTRN12621001423808. 2021. PubMed PMID: CN-02349018.

1127. Actrn. Home based Education and Learning Program for Gout. https://trialsearchwhoint/Trial2aspx?TrialID=ACTRN12621000761864. 2021. PubMed PMID: CN-02281096.

1128. Actrn. A home-based, multidisciplinary liver optimisation program for the first 28 days after an admission for acute-on-chronic liver failure (LivR Well). https://trialsearchwhoint/Trial2aspx?TrialID=ACTRN12621001703897. 2021. PubMed PMID: CN-02379303.

1129. Actrn. Idebenone versus placebo in dominant optic atrophy. https://trialsearchwhoint/Trial2aspx?TrialID=ACTRN12621000826842. 2021. PubMed PMID: CN-02281055.

1130. Actrn. Investigating the effectiveness of a patient-centred dispensing label on comprehension of medication dosing instructions. https://trialsearchwhoint/Trial2aspx?TrialID=ACTRN12621000083897. 2021. PubMed PMID: CN-02240685.

1131. Actrn. Is metformin better taken before or with meals? An acute evaluation of the impact of timing of metformin administration on intestinal glucose absorption, gut hormone secretion and the blood glucose response to an intraduodenal glucose infusion in type 2 diabetes. https://trialsearchwhoint/Trial2aspx?TrialID=ACTRN12621000878875. 2021. PubMed PMID: CN-02327091.

1132. Actrn. Ivermectin to prevent Coronavirus. https://trialsearchwhoint/Trial2aspx?TrialID=ACTRN12621001535864. 2021. PubMed PMID: CN-02349087.

1133. Actrn. Ketamine for Cancer Pain. https://trialsearchwhoint/Trial2aspx?TrialID=ACTRN12621000328875. 2021. PubMed PMID: CN-02256829.

1134. Actrn. More or less? Bile acids and glycaemic control in type 2 diabetes. https://trialsearchwhoint/Trial2aspx?TrialID=ACTRN12621000877886. 2021. PubMed PMID: CN-02327090.

1135. Actrn. NanaBis™ an Oro-Buccal Administered delta9-Tetrahydrocannabinol (d9-THC) and Cannabidiol (CBD) Medicine For The Management of Chronic Pain From Metastatic Bone Cancer. https://trialsearchwhoint/Trial2aspx?TrialID=ACTRN12621001302842. 2021. PubMed PMID: CN-02327334.

1136. Actrn. A pilot randomised controlled trial testing the efficacy of cannabidiol for anxiety in Tourette Syndrome. https://trialsearchwhoint/Trial2aspx?TrialID=ACTRN12621001659897. 2021. PubMed PMID: CN-02349159.

1137. Actrn. POMMS: pre-Operative Misoprostol in Myomectomy Surgery – A Pilot Study. https://trialsearchwhoint/Trial2aspx?TrialID=ACTRN12621000808842. 2021. PubMed PMID: CN-02281360.

1138. Actrn. Randomised trial of Empagliflozin and Left ventricular diastolic function in Acute Coronary Syndrome and Type 2 Diabetes (RELACS-T2D). https://trialsearchwhoint/Trial2aspx?TrialID=ACTRN12621000355875. 2021. PubMed PMID: CN-02257006.

1139. Actrn. Responsible opioid use before hip and knee replacement surgery. https://trialsearchwhoint/Trial2aspx?TrialID=ACTRN12621000919819. 2021. PubMed PMID: CN-02327120.

1140. Actrn. REstrictive vs StandarD FlUid Management in Mechanically Ventilated ChildrEn Admitted to Paediatric Intensive Care Unit (PICU) - a Pilot Randomised Controlled Trial. https://trialsearchwhoint/Trial2aspx?TrialID=ACTRN12621001311842. 2021. PubMed PMID: CN-02327341.

1141. Actrn. Sodium-glucose co-transporter 2 inhibition prior to atrial fibrillation ablation: a pilot randomised controlled trial. https://trialsearchwhoint/Trial2aspx?TrialID=ACTRN12621000685819. 2021. PubMed PMID: CN-02282111.

1142. Actrn. Sodium-Glucose Co-Transporter 2 inhibition prior to cardiac surgery and postoperative atrial fibrillation: a pilot randomised controlled trial. https://trialsearchwhoint/Trial2aspx?

TrialID=ACTRN12621000684820. 2021. PubMed PMID: CN-02281523.

1143. Actrn. A Study to Evaluate the Safety, Tolerability, Pharmacokinetic, and Pharmacodynamic Effects of KER-012 Administered to Healthy, Postmenopausal Women. https://trialsearchwhoint/Trial2aspx?TrialID=ACTRN12621001077853. 2021. PubMed PMID: CN-02327200.

1144. Actrn. Three versus five years of adjuvant Imatinib as treatment for patients with operable gastrointestinal stromal tumour (GIST) with a high risk for recurrence: SSGXXII: a Randomised phase III study. https://trialsearchwhoint/Trial2aspx?TrialID=ACTRN12621001756819. 2021. PubMed PMID: CN-02376270.

1145. Actrn. Time to analgesia with early involvement of the Emergency Medicine pharmacist in trauma callouts: a randomised trial (Study Protocol). https://trialsearchwhoint/Trial2aspx?TrialID=ACTRN12621000338864. 2021. PubMed PMID: CN-02255433.

1146. Actrn. Tripalma™ for the Topical Treatment of Impetigo. https://trialsearchwhoint/Trial2aspx?TrialID=ACTRN12621000403831. 2021. PubMed PMID: CN-02255522.

1147. Actrn. ULTRA Pain Study - Ursodeoxycholic acid in Low Phospholipid Associated Cholelithiasis (LPAC) Treating Recurrent Abdominal Pain Study. https://trialsearchwhoint/Trial2aspx?TrialID=ACTRN12621000450819. 2021. PubMed PMID: CN-02256956.

1148. Actrn. Audit and feedback for reducing long-term prescribing of benzodiazepines and opioids by general practitioners. https://trialsearchwhoint/Trial2aspx?TrialID=ACTRN12622000127707. 2022. PubMed PMID: CN-02376337.

1149. Actrn. Cannabidiol (CBD) for Clozapine Refractory Schizophrenia (CanCloz). https://trialsearchwhoint/Trial2aspx?TrialID=ACTRN12622001112752. 2022. PubMed PMID: CN-02451916.

1150. Actrn. ECMT-154™ for the Topical Treatment of Eczema in Children. https://trialsearchwhoint/Trial2aspx?TrialID=ACTRN12622000684729. 2022. PubMed PMID: CN-02408197.

1151. Actrn. The effect of an intervention comprising a coordinated and integrated medicine management service (Home Medicines Review (HMR)) provided for patients in primary care with osteoporosis (OP) and Minimal Trauma Fracture (MTF). https://trialsearchwhoint/Trial2aspx?TrialID=ACTRN12622000261718. 2022. PubMed PMID: CN-02379339.

1152. Actrn. Effect of an Online (Social Media) Nudge Campaign to improve PrEP use among overseas-born gay, bisexual and other men who have sex with men. https://trialsearchwhoint/Trial2aspx?TrialID=ACTRN12622000724774. 2022. PubMed PMID: CN-02408219.

1153. Actrn. The Effects of an Intervention to Optimise Quality Use Of Medicines In Older People in Hospital. https://trialsearchwhoint/Trial2aspx?TrialID=ACTRN12622000374763. 2022. PubMed PMID: CN-02408023.

1154. Actrn. Effects of cannabidiol (CBD) on chronic pain following spinal cord injury. https://trialsearchwhoint/Trial2aspx?TrialID=ACTRN12622000634774. 2022. PubMed PMID: CN-02408173.

1155. Actrn. The effects of glucagon-like peptide-1 on glycaemia in the critically ill. https://trialsearchwhoint/Trial2aspx?TrialID=ACTRN12622001409763. 2022. PubMed PMID: CN-02494435.

1156. Actrn. Enhancement of pharmacist role for the management of Chronic Obstructive Pulmonary Disease (COPD) in Pakistan. https://trialsearchwhoint/Trial2aspx?TrialID=ACTRN12622000234718. 2022. PubMed

PMID: CN-02376388.

1157. Actrn. Evaluating the feasibility and acceptability of a discharge medication literacy intervention (the CHAT study). https://trialsearchwhoint/Trial2aspx?TrialID=ACTRN12622001028796. 2022. PubMed PMID: CN-02428930.

1158. Actrn. Evaluating two doses of intravenous lidocaine infusion for relief of nerve-related pain that persists after trauma or surgery. https://trialsearchwhoint/Trial2aspx?TrialID=ACTRN12622000538741. 2022. PubMed PMID: CN-02408113.

1159. Actrn. Evaluation of text messaging service on management of post-operative pain in opioid-naïve surgical patients. https://trialsearchwhoint/Trial2aspx?TrialID=ACTRN12622000516785. 2022. PubMed PMID: CN-02408099.

1160. Actrn. Impact of probiotics and octacosanol supplementation on the regulation of obesity and associated diseases in overweight and obese women in the reproductive period. https://trialsearchwhoint/Trial2aspx?TrialID=ACTRN12622000696796. 2022. PubMed PMID: CN-02408205.

1161. Actrn. Investigating the effect of Activating pharmacists to reduce medication related problems: the ACTMed stepped wedge randomised controlled trial. https://trialsearchwhoint/Trial2aspx?TrialID=ACTRN12622000574741. 2022. PubMed PMID: CN-02408132.

1162. Actrn. Investigating the effect of medicinal cannabis on the symptoms and side effects of chemotherapy in people with cancer. https://trialsearchwhoint/Trial2aspx?TrialID=ACTRN12622000419763. 2022. PubMed PMID: CN-02408042.

1163. Actrn. Low-dose naltrexone as an adjunctive treatment in major depressive disorder. https://trialsearchwhoint/Trial2aspx?TrialID=ACTRN12622000881730. 2022. PubMed PMID: CN-02428846.

1164. Actrn. Managing Delirium with FLUvoxamine treatment in Non-Cardiac Surgical patients: a feasibility trial. https://trialsearchwhoint/Trial2aspx?TrialID=ACTRN12622000862741. 2022. PubMed PMID: CN-02428835.

1165. Actrn. Pharmacoeconomic evaluation of Pharmacist led diabetes education intervention in Type 2 diabetes patients Randomized Clinical Trial. https://trialsearchwhoint/Trial2aspx?TrialID=ACTRN12622000658718. 2022. PubMed PMID: CN-02408186.

1166. Actrn. Pharmacy-Based Screening and Quality Use of Medicines in Kidney Disease. https://trialsearchwhoint/Trial2aspx?TrialID=ACTRN12622000329763. 2022. PubMed PMID: CN-02376438.

1167. Actrn. Phase I clinical study to evaluate the safety and tolerability of SARS-CoV-2 spike protein (HexaPro) delivered intradermally by a high-density microarray patch (HD-MAP), in healthy adults aged 18 to 50 years. https://trialsearchwhoint/Trial2aspx?TrialID=ACTRN12622000597796. 2022. PubMed PMID: CN-02408147.

1168. Actrn. Quality family planning services and referrals in community pharmacy: expanding pharmacists' scope of practice (The ALLIANCE Trial). https://trialsearchwhoint/Trial2aspx?TrialID=ACTRN12622001024730. 2022. PubMed PMID: CN-02428927.

1169. Actrn. A Randomised trial assessing Efficacy and safety of Mineralocorticoid receptor Antagonist therapy compared to Standard antihypertensive Therapy in hypErtension with low Renin (REMASTER). https://trialsearchwhoint/Trial2aspx?TrialID=ACTRN12622000391774. 2022. PubMed PMID: CN-02408028.

1170. Actrn. Randomized clinical trial assessing efficacy of a novel Moisturising Skin Support versus standard care for management of chronic wounds. https://trialsearchwhoint/Trial2aspx?TrialID=ACTRN12622001229763. 2022. PubMed PMID: CN-02460876.

1171. Actrn. Restriction of Oxycodone in the Emergency Department (ROXY-ED): a Randomized Controlled Trial. https://trialsearchwhoint/Trial2aspx?TrialID=ACTRN12622001183774. 2022. PubMed PMID: CN-02458257.

1172. Actrn. Smoking cessation online program: a randomized controlled trial to test the effectiveness and the feasibility of Facebook platform to reduce smoking among Syrian young adults. https://trialsearchwhoint/Trial2aspx?TrialID=ACTRN12622000318785. 2022. PubMed PMID: CN-02376429.

1173. Actrn. Switching from Dose-Intensified intravenous to SubCutaneoUS infliximab in Inflammatory Bowel Disease: a randomised controlled trial - DISCUS-IBD. https://trialsearchwhoint/Trial2aspx?TrialID=ACTRN12622001458729. 2022. PubMed PMID: CN-02498088.

1174. Actrn. Te Tauwhiro Turi ma te Rongoa: knee Care for Arthritis through Pharmacy Study. https://trialsearchwhoint/Trial2aspx?TrialID=ACTRN12622000469718. 2022. PubMed PMID: CN-02408073.

1175. Actrn. Theta burst Transcranial Magnetic Stimulation for Methamphetamine use disorder (TARTAN). https://trialsearchwhoint/Trial2aspx?TrialID=ACTRN12622000762752. 2022. PubMed PMID: CN-02408245.

1176. Actrn. The VRESIST trial: evaluating the effectiveness of a hospital-based antimicrobial stewardship intervention upon antibiotic use in Vietnam. https://trialsearchwhoint/Trial2aspx?TrialID=ACTRN12622000715774. 2022. PubMed PMID: CN-02408214.

1177. Acuna P. Pharmacy Practice in Chile. Pharmacy Practice in Developing Countries: Achievements and Challenges2016. p. 403-47.

1178. Adah RO, John C, Uhunmwangho C, Adah GU, Okolo SN. Non-specialized care of skin disorders: a cross-sectional survey of new patients attending dermatology clinic in a tertiary hospital in Jos, Northcentral Nigeria. African Health Sciences. 2023;23(3):635-44. doi: 10.4314/ahs.v23i3.74. PubMed PMID: WOS:001085838300074.

1179. Adam J-P, Khazaka M, Charikhi F, Clervil M, Huot DD, Jebailey J, et al. Management of human resources of a pharmacy department during the COVID-19 pandemic: Take-aways from the first wave. Research in Social and Administrative Pharmacy. 2021;17(1):1990-6. doi: https://doi.org/10.1016/j.sapharm.2020.10.014.

1180. Adam TJ, Vang J. Content and Usability Evaluation of Patient Oriented Drug-Drug Interaction Websites. AMIA Annual Symposium proceedings AMIA Symposium. 2015;2015:287-96.

1181. Adams A, Blawatt S, MacDonald S, Finnick R, Lajeunesse J, Harrison S, et al. Provider experiences with relaxing restrictions on take-home medications for opioid use disorder during the COVID-19 pandemic: A qualitative systematic review. International Journal of Drug Policy. 2023;117:N.PAG-N.PAG. doi: 10.1016/j.drugpo.2023.104058. PubMed PMID: 164459452.

1182. Adams AJ. Medication synchronization: A regulatory approach to "short fills". Journal of the American Pharmacists Association: JAPhA. 2017;57(3):299-300. PubMed PMID: 28392160.

1183. Adams AJ. Advancing technician practice: Deliberations of a regulatory board. Research in Social and Administrative Pharmacy. 2018;14(1):1-5. doi: https://doi.org/10.1016/j.sapharm.2017.02.008.

1184. Adams AJ. Prescription Adaptation Services: A Regulatory and Practice Perspective. Annals of Pharmacotherapy. 2018;52(7):700-3. doi: 10.1177/1060028018763883.

1185. Adams AJ, Eid DD. Federal pharmacist Paxlovid prescribing authority: A model policy or impediment to optimal care? Exploratory Research in Clinical and Social Pharmacy. 2023;9:100244. doi: https://dx.doi.org/10.1016/j.rcsop.2023.100244. PubMed PMID: 2023427948.

1186. Adams AJ, Frost TP. The over-regulation of pharmacist services: Why over-the-counter naloxone should spark conversation about a "standard of care" regulatory approach. JACCP Journal of the American College of Clinical Pharmacy. 2023;6(6):615-20. doi: https://dx.doi.org/10.1002/jac5.1794. PubMed PMID: 2023081036.

1187. Adams AJ, Frost TP. The over-regulation of pharmacist services: Why over-the-counter naloxone should spark conversation about a “standard of care” regulatory approach. Journal of the American College of Clinical Pharmacy. 2023;6(6):615-20.

1188. Adams AJ, Frost TP, Eid D. The Basis for Elimination of the Jurisprudence Examination as a Condition of Pharmacist Licensure in Idaho. American Journal of Pharmaceutical Education. 2023;87(10):100119. doi: https://doi.org/10.1016/j.ajpe.2023.100119.

1189. Adams AJ, Martin SJ, Stolpe SF. “Tech-check-tech”: a review of the evidence on its safety and benefits. American Journal of Health-System Pharmacy. 2011;68(19):1824-33.

1190. Adams AL, Black MH, Zhang JL, Shi JM, Jacobsen SJ. Proton-pump inhibitor use and hip fractures in men: a population-based case-control study. Annals of Epidemiology. 2014;24(4):286-90. doi: https://doi.org/10.1016/j.annepidem.2014.01.004.

1191. Adams AM, Islam R, Yusuf SS, Panasci A, Crowell N. Healthcare seeking for chronic illness among adult slum dwellers in Bangladesh: A descriptive cross-sectional study in two urban settings. Plos One. 2020;15(6). doi: 10.1371/journal.pone.0233635. PubMed PMID: WOS:000542685100035.

1192. Adams AM, Islami R, Yusuf SS, Panasci A, Crowell N. Healthcare seeking for chronic illness among adult slum dwellers in Bangladesh: A descriptive cross-sectional study in two urban settings. PLoS ONE. 2020;15(6). doi: 10.1371/journal.pone.0233635.

1193. Adams D, Jubraj B. Prescribing for people with a learning disability. Prescriber. 2023;34:23-7. doi: 10.1002/psb.2087. PubMed PMID: 172046313.

1194. Adams D, Wilson AL. Structuring an indigent care pharmacy benefit program. American Journal of Health-System Pharmacy. 2002;59(17):1669-75. doi: 10.1093/ajhp/59.17.1669.

1195. Adams DJ, Eberly MD, Rajnik M, Nylund CM. Risk Factors for Community-Associated Clostridium difficile Infection in Children. Journal of Pediatrics. 2017;186:105-9. doi: 10.1016/j.jpeds.2017.03.032.

1196. Adams DW, Carroll NV. Changes in number and distribution of community and ambulatory pharmacies in Virginia from 1994 to 1999. Journal of the American Pharmaceutical Association (Washington, DC : 1996). 2000;40(6):780-4. doi: 10.1016/S1086-5802(16)31125-1.

1197. Adams IB, Martin BR. Cannabis: pharmacology and toxicology in animals and humans. Addiction. 1996;91(11):1585-614.

1198. Adams J, Cymbala AA, Delate T, Kurz D, Olson KL, Zadvorny E. Cluster-Randomized Trial of Clinical Pharmacist Tobacco Cessation Counseling Among Patients with Cardiovascular Disease. Population

health management. 2015;18(4):300-6. doi: 10.1089/pop.2014.0106. PubMed PMID: CN-01255485.

1199. Adams JL, O'Connor S, Seignemartin B, Shipman AJ, McConnell W, Dudman A, et al. Battling professional self-sabotage: Embracing standard of care as the future of pharmacy regulation. Journal of the American Pharmacists Association. 2023;63(6):1685-8.e1. doi: https://doi.org/10.1016/j.japh.2023.08.015.

1200. Adams KF, Patterson JH, Gattis WA, O'Connor CM, Lee CR, Schwartz TA, et al. Relationship of serum digoxin concentration to mortality and morbidity in women in the digitalis investigation group trial: a retrospective analysis. Journal of the American College of Cardiology. 2005;46(3):497-504.

1201. Adams OB. A Population Health Framework: Assessing Its Applicability for Primary Care Physicians [Ph.D.]. Canada -- Ontario, CA: University of Ottawa (Canada); 2013.

1202. Adams R. Development and feasibility testing of a supervised pharmacy student-led medication review of patients with diabetes in primary care [Ph.D.]. England: University of East Anglia (United Kingdom); 2014.

1203. Adams R, Bhattacharya D, Eddy G, Grassby P, Holland R, Howe A, et al. Stakeholder focus groups as part of the development phase of a pilot study of pharmacy student led medication reviews for patients with diabetes in primary care. International journal of pharmacy practice. 2012;20:45-6. doi: 10.1111/j.2042-7174.2012.00198.x. PubMed PMID: CN-01005307.

1204. Adams RP, Barton G, Bhattacharya D, Grassby PF, Holland R, Howe A, et al. Supervised pharmacy student-led medication review in primary care for patients with type 2 diabetes: a randomised controlled pilot study. BMJ open. 2015;5(11):e009246. doi: 10.1136/bmjopen-2015-009246. PubMed PMID: CN-01179898.

1205. Adams S, Boyd M, Carryer J, Bareham C, Tenbensel T. A survey of the NP workforce in primary healthcare settings in New Zealand. New Zealand Medical Journal. 2020;133(1523):29-40.

1206. Adams SV, Barrick B, Christopher EP, Shafer MM, Makar KW, Song X, et al. Genetic variation in metallothionein and metal-regulatory transcription factor 1 in relation to urinary cadmium, copper, and zinc. Toxicology and applied pharmacology. 2015;289(3):381-8.

1207. Adamsick ML, Gandhi RG, Steiger SN, Bidell MR, Nelson SB, Ard KL, et al. Impact of infectious diseases pharmacists as part of an interdisciplinary opat team managing vancomycin. Open forum infectious diseases. 2019;6:S337-S8. doi: 10.1093/ofid/ofz360.824. PubMed PMID: CN-02075075.

1208. Adamu AA, Gadanya MA, Jalo RI, Uthman OA, Wiysonge CS. Factors influencing non-prescription sales of antibiotics among patent and proprietary medicine vendors in Kano, Nigeria: a cross-sectional study. Health Policy and Planning. 2020;35(7):819-28. doi: 10.1093/heapol/czaa052. PubMed PMID: WOS:000582695300007.

1209. Adamu Y, Haque M. Pharmacogenovigilance in drug safety: Knowledge, attitudes and practice among final-year medical and pharmacy students in Peninsular Malaysia. International Journal of Pharmaceutical Research. 2016;8(1):57-71.

1210. Adan C, Molinero A, Castellano L, Peral V, Arano B, de Juan B, et al. Perceived effectiveness and safety of symptomatic slow action drugs for osteoarthritis (SYSADOA) for treating osteoarthrosis from community pharmacy. Farmaceuticos Comunitarios. 2016;8(4):5-17. doi: 10.5672/FC.2173-9218.(2016/Vol8).004.02. PubMed PMID: WOS:000417275700002.

1211. Adane M, Mengistie B, Mulat W, Kloos H, Medhin G. Utilization of health facilities and predictors of health-seeking behavior for under-five children with acute diarrhea in slums of Addis Ababa, Ethiopia: a

community-based cross-sectional study. Journal of health, population, and nutrition. 2017;36(1):9. doi: 10.1186/s41043-017-0085-1.

1212. Adane M, Teka B, Gismu Y, Halefom G, Ademe M. Food hygiene and safety measures among food handlers in street food shops and food establishments of Dessie town, Ethiopia: A community-based cross-sectional study. Plos One. 2018;13(5). doi: 10.1371/journal.pone.0196919. PubMed PMID: WOS:000431305100091.

1213. Adapa RM, Mani V, Murray LJ, Degnan BA, Ercole A, Cadman B, et al. Errors during the preparation of drug infusions: a randomized controlled trial. British journal of anaesthesia. 2012;109(5):729-34. doi: 10.1093/bja/aes257. PubMed PMID: CN-00879403.

1214. Adapa RM, Mani V, Murray LJ, Degnan BA, Ercole A, Cadman B, et al. Errors during the preparation of drug infusions: a randomized controlled trial. BJA: The British Journal of Anaesthesia. 2012;109(5):729-34. doi: 10.1093/bja/aes257. PubMed PMID: 104373994. Language: English. Entry Date: 20130201. Revision Date: 20200708. Publication Type: journal article.

1215. Adcock H. Is direct dispensing good for patients?

1216. Adcock H, editor How community pharmacists can help. Pharmaceutical Journal; 2003.

1217. Adcock H, editor Pharmacy is the way forward for direct-to-consumer cancer prevention and care. Pharmaceutical Journal; 2003.

1218. Adcock H. Clinical developments in 2006. Pharmaceutical Journal. 2007;278(7433):21-4.

1219. Adcock H. PSNC's challenge: To ensure potential of community pharmacy is recognised. Pharmaceutical Journal. 2008;280(7487):121.

1220. Adcock H, Pike H, Wang LN, editors. Pharmacists predicted to be emperors in the new world of poly-pharmacy. Pharmaceutical Journal; 2005.

1221. Adcock KG, Earl S. Interprofessional education tracks: One schools response to common IPE barriers. Currents in Pharmacy Teaching and Learning. 2023;15(5):528-33. doi: https://doi.org/10.1016/j.cptl.2023.05.007.

1222. Addison C, Taylor D. The pharmacist as safety net: an interview-based study of the intersecting dependencies between doctors and pharmacists. Journal of Pharmaceutical Policy and Practice. 2023;16(1):40.

1223. Addo-Atuah J. Making a case for a public health orientation in global pharmacy education and practice in the context of the Millennium Development Goals (MDGs). Currents in Pharmacy Teaching and Learning. 2014;6(5):723-9. doi: 10.1016/j.cptl.2014.05.019.

1224. Addo-Atuah J, Fuchs H, Tetenbaum-Novatt J, Jeger AM. Making a Case for Faculty Advisor–Advisee Concordant Pairs. American Journal of Pharmaceutical Education. 2023;87(12):100138. doi: https://doi.org/10.1016/j.ajpe.2023.100138.

1225. Adé A, Bourdon O, Bussières JF. A survey of pharmacists’ knowledge and views of biosimilars in Quebec and France. Annales Pharmaceutiques Françaises. 2017;75(4):267-75. doi: https://doi.org/10.1016/j.pharma.2017.01.003.

1226. Adebisi YA. Balancing the risks and benefits of antibiotic use in a globalized world: the ethics of

antimicrobial resistance. Globalization and Health. 2023;19(1). doi: 10.1186/s12992-023-00930-z.

1227. Adedeji AA, Sanusi B, Tella A, Akinsanya M, Ojo O, Akinwunmi MO, et al. Exposure to anti-malarial drugs and monitoring of adverse drug reactions using toll-free mobile phone calls in private retail sector in Sagamu, Nigeria: implications for pharmacovigilance. Malaria Journal. 2011;10. doi: 10.1186/1475-2875-10-230. PubMed PMID: WOS:000294505100001.

1228. Adedeji O. Assessing the extent and need for disability inclusion in the pharmacy education curriculum in Nigeria. Pharmacy Education. 2021;21:670-3. doi: 10.46542/PE.2021.211.670673.

1229. Adegbehingbe OO, Ayoola O, Soyoye D, Adegbehingbe A. Urinary bladder wall thickness in type 2 diabetes mellitus patients. Journal of Ultrasonography. 2022;22(88):E12-E20. doi: 10.15557/JoU.2022.0003. PubMed PMID: WOS:000754379200003.

1230. Adegbuyi TA, Fadare JO, Araromi EJ, Sijuade AO, Bankole I, Fasuba IK, et al. Assessment of Knowledge, Attitude and Practice of Adverse Drug Reaction Reporting Among Healthcare Professionals working in Primary, Secondary and Tertiary Healthcare Facilities in Ekiti State, South-West Nigeria. Hospital Pharmacy. 2021;56(6):751-9. doi: 10.1177/0018578720957968.

1231. Adejumo O, Ogundele O, Mamven M, Oyedepo D, Ntaji M, Mohammed A, et al. Assessment of hypertension service availability in some primary health centres in Nigeria: a mixed-methods study. Bmj Open. 2023;13(8). doi: 10.1136/bmjopen-2023-073833. PubMed PMID: WOS:001044964800008.

1232. Adeke AS, Umeokonkwo CD, Balogun MS, Odili AN. Essential medicines and technology for hypertension in primary healthcare facilities in Ebonyi State, Nigeria. PLoS ONE. 2022;17(2 February). doi: 10.1371/journal.pone.0263394.

1233. Adekunle OA, Olson AW, Schommer JC, Brown LM. Influence of patient-pharmacist relationship on willingness to accept pharmacist-provided services. Journal of the American Pharmacists Association. 2023;63(3):760-8.e1. doi: https://dx.doi.org/10.1016/j.japh.2022.12.016. PubMed PMID: 2022256627.

1234. Adekunle OA, Olson AW, Schommer JC, Brown LM. Investigation of predictors influencing patient-pharmacist relationship establishment. Journal of the American Pharmacists Association. 2023;63(3):853-62. doi: 10.1016/j.japh.2022.12.024.

1235. Adelglass J, Jones TM, Ruoff G, Kahn JB, Wiesinger BA, Rielly-Gauvin K, et al. A multicenter, investigator-blinded, randomized comparison of oral levofloxacin and oral clarithromycin in the treatment of acute bacterial sinusitis. Wiley Online Library; 1998.

1236. Adelia AL, Nurainiwati SA, Putra PYP, Hapsari AS. Efficacy, effectiveness, and safety of combination laser and tranexamic acid treatment for melasma: A meta-analysis. Chinese Journal of Plastic & Reconstructive Surgery. 2023;5(3):154-8. doi: 10.1016/j.cjprs.2023.06.001. PubMed PMID: 174187982.

1237. Ademi Y, Hoti K. Assessing the suitability of using the PCNE classification system to identify drug related problems in non-hospitalized patients with COVID-19. International Journal of Clinical Pharmacy. 2023;45(2):538-9. doi: https://dx.doi.org/10.1007/s11096-023-01576-y. PubMed PMID: 641388243.

1238. Ademola-Popoola DS, Olatunji VA, Obajolowo TS, Akande TM, Mokuolu OA. Outcome of vision screening by community health workers at immunization outlets in Nigeria to support access to early visual evaluation in children aged 0-2 years. Taiwan Journal of Ophthalmology. 2021;11(1):77-85. doi: 10.4103/tjo.tjo_39_20. PubMed PMID: WOS:000621020100010.

1239. Adeniji SE, Adalumo OB, Ekoja FO. Anti-tubercular modelling, molecular docking simulation and

insight toward computational design of novel compounds as potent antagonist against DNA gyrase receptor. Medicine in Microecology. 2020;5:100020. doi: https://doi.org/10.1016/j.medmic.2020.100020.

1240. Adeniji SE, Adamu Shallangwa G, Ebuka Arthur D, Abdullahi M, Mahmoud AY, Haruna A. Quantum modelling and molecular docking evaluation of some selected quinoline derivatives as anti-tubercular agents. Heliyon. 2020;6(3):e03639. doi: https://doi.org/10.1016/j.heliyon.2020.e03639.

1241. Adenot E, Agay L, Belon JP, Piraux A. Organisation of pharmacy students' initial training. Actualites Pharmaceutiques. 2019;58(583):25-8. doi: 10.1016/j.actpha.2018.12.006. PubMed PMID: WOS:000460926200019.

1242. Adenot I. The pharmaceutical record. Annales Pharmaceutiques Francaises. 2007;65(5):325-30. doi: 10.1016/S0003-4509(07)92595-X.

1243. Adeosun SO. Stigma by Association: To what Extent is the Attitude Toward Naloxone Affected by the Stigma of Opioid Use Disorder? Journal of Pharmacy Practice. doi: 10.1177/08971900221097173. PubMed PMID: WOS:000795717900001.

1244. Adeosun SO, Ahmed ZR. The impact of the COVID-19 pandemic on school-level scholarly outcomes and research focus of pharmacy practice faculty. JACCP Journal of the American College of Clinical Pharmacy. 2023;6(11):1191-202. doi: https://dx.doi.org/10.1002/jac5.1857. PubMed PMID: 2024495335.

1245. Adeoye OA, Lake LM, Lourens SG, Morris RE, Snyder ME. What predicts medication therapy management completion rates? The role of community pharmacy staff characteristics and beliefs about medication therapy management. Journal of the American Pharmacists Association: JAPhA. 2018;58(4S):S7-S15.e5. PubMed PMID: 29731422.

1246. Adeoye-Olatunde AO, Lake ML, Strohmier AC, Gourley KA, Ray RA, Zillich JA, et al. Positive deviants for medication therapy management: A mixed-methods comparative case study of community pharmacy practices. Research in Social & Administrative Pharmacy. 2021;17(8):1407-19. doi: 10.1016/j.sapharm.2020.10.006. PubMed PMID: WOS:000664077000004.

1247. Adeoye-Olatunde OA, Lake LM, Strohmier CA, Gourley AK, Ray AR, Zillich AJ, et al. Positive deviants for medication therapy management: A mixed-methods comparative case study of community pharmacy practices. Research in Social and Administrative Pharmacy. 2021;17(8):1407-19. doi: https://doi.org/10.1016/j.sapharm.2020.10.006.

1248. Adeoye-Olatunde OA, Pratt N, Kim DD, Schmidt E, Snyder ME. Adaptation and validation of the patient assessment of chronic illness care in United States community pharmacies. BMC Health Services Research. 2022;22(1). doi: 10.1186/s12913-022-07697-w.

1249. Adeoye-Olatunde OA, Vlashyn OO, Illingworth Plake KS, Woodyard JL, Weber ZA, Litzelman DK, et al. A mixed-methods study of pharmacy instructors' early experiences with a teaching electronic medical record. Currents in Pharmacy Teaching and Learning. 2021;13(9):1180-93. doi: https://doi.org/10.1016/j.cptl.2021.06.036.

1250. Adepoju OE, Kiaghadi A, Niaki DS, Karunwi A, Chen H, Woodard L. Rethinking access to care: A spatial-economic analysis of the potential impact of pharmacy closures in the United States. PLoS ONE. 2023;18(7 JULY). doi: 10.1371/journal.pone.0289284.

1251. Adepoju VA, Oladimeji O. A Comparative Analysis of Patient Profiles and Health Service Utilization between Patent Medicine Vendors and Community Pharmacists in Nigeria. Healthcare. 2023;11(18). doi: 10.3390/healthcare11182484. PubMed PMID: WOS:001078313400001.

1252. Adepu R, Ari SM. Influence of structured patient education on therapeutic outcomes in diabetes and hypertensive patients. Asian journal of pharmaceutical and clinical research. 2010;3(3):174-8. PubMed PMID: CN-00803115.

1253. Adepu R, Madhu S. Influence of post discharge counselling on health outcomes in diabetic and hypertensive patients. Asian journal of pharmaceutical and clinical research. 2011;4(3):28-33. PubMed PMID: CN-00868806.

1254. Adepu R, Nagavi B. General practitioners' perceptions about the extended roles of the community pharmacists in the state of Karnataka: A study. Indian Journal of Pharmaceutical Sciences. 2006;68(1):36-40. doi: 10.4103/0250-474X.22961.

1255. Adepu R, Rasheed A, Nagavi B. Effect of patient counseling on quality of life in type-2 diabetes mellitus patients in two selected South Indian community pharmacies: a study. Indian journal of pharmaceutical sciences. 2007;69(4):519-24. PubMed PMID: CN-00641091.

1256. Adepu SP, Ramanath KV, Bhat S, Sam S, Omanakuttan C. Assessment of pharmacist-mediated education on medication adherence behaviour in type 2 diabetes mellitus patients. Value in health Conference: ISPOR 21st annual international meeting research Washington, DC united states Conference start: 20160521 Conference end: 20160525 Conference publication: (varpagings). 2016;19(3):A18. PubMed PMID: CN-01266613.

1257. Adepu SPR, Ramanath KV, Bhat S, Sam S, Omanakuttan C. Assessment of pharmacist mediated education on health related quality of life in type 2 diabetes mellitus patients in rural india. Pharmacoepidemiology and drug safety. 2016;25:310-. doi: 10.1002/pds.4070. PubMed PMID: CN-01293648.

1258. Adepu SPR, Sam S, Omanakuttan C, Ramanath KV, Yashaswini Y. Assessment of pharmacist mediated education on medication adherence behavior in type 2 diabetes mellitus patients in South Indian rural population. International journal of pharmacy and pharmaceutical sciences. 2018;10(9):39-43. doi: 10.22159/ijpps.2018v10i9.27543. PubMed PMID: CN-01644495.

1259. Aderemi HO, Ojediran IA, Adegbite SA, Olasanmi OO. Effects of the use of ICT on pharmaceutical product marketing in Nigeria. International Journal of Management and Business Research. 2015;5(4):315-27.

1260. Aderemi-Williams RI. Patients' Adherence to Antiretroviral Therapy in Lagos State University Teaching Hospital (LASUTH), Ikeja, Lagos, Nigeria [Ph.D.]. Nigeria: University of Lagos (Nigeria); 2013.

1261. Aderemi-Williams RI, Nduaguba SO, Akoji EM, Ogbo PU, Abah IO. Drug therapy problems identified among patients receiving antiretroviral treatment in a HIV clinic: a prospective study in North Central, Nigeria. The Pan African medical journal. 2021;40:233. PubMed PMID: 35178144.

1262. Aderounmu AD. Development and validation of a new instrument to measure the socioeconomic impact of medication-related problems in community pharmacy setting [Ph.D.]. Wales: The University of Wales College of Cardiff (United Kingdom); 2008.

1263. Aderounmu AO. Development and validation of a new instrument to measure the socioeconomic impact of medication-related problems in community pharmacy setting [Ph.D.]. Wales: Cardiff University (United Kingdom); 2008.

1264. Ades S, Gilchrist S, Holm A, Libby K, Cushman M, Douce D, et al. Venous Thromboembolism Prevention in the Ambulatory Cancer Clinic (VTE-PACC): a Systems-Based, Personalized, Multidisciplinary

Program to Increase Venous Thromboembolism (VTE) Risk Assessment, Education and Anticoagulant Prophylaxis in Cancer Outpatients Initiating Therapy. Blood. 2017;130:217. doi: https://doi.org/10.1182/blood.V130.Suppl_1.217.217.

1265. Ades S, Herrera DA, Lahey T, Thomas AA, Jasra S, Barry M, et al. Cancer Care in the Wake of a Cyberattack: How to Prepare and What to Expect. JCO Oncology Practice. 2022;18(2):23-34. doi: 10.1200/OP.21.00116.

1266. Adetunji AA, Muyibi SA, Imhansoloeva M, Ibraheem OM, Sunmola A, Kolawole OO, et al. Mobile phone use for a social strategy to improve antiretroviral refill experience at a low-resource HIV clinic: patient responses from Nigeria. AIDS Care - Psychological and Socio-Medical Aspects of AIDS/HIV. 2017;29(5):575-8. doi: 10.1080/09540121.2016.1226476.

1267. Adeyemo G. The Effect of Education Through Text Messages to Reduce Systolic Blood Pressure in Adults with Uncontrolled Hypertension [D.N.P.]. United States -- California: Brandman University; 2020.

1268. Adgalanis MA, Davis BT, Hettinger KN, Thoma LM, Papineau JH, Meredith AH. Pharmacist-led hormonal contraceptive prescribing service in a Federally Qualified Health Center: Initial implementation outcomes. Journal of the American Pharmacists Association. 2023;63(4):1185-90.e1. doi: https://dx.doi.org/10.1016/j.japh.2023.04.027. PubMed PMID: 2024817979.

1269. Adham MA, Hamad BA. Drug dispensing systems in Gaza hospitals: a comparative study. Eastern Mediterranean Health Journal. 2011;17(10):722-9. doi: 10.26719/2011.17.10.722. PubMed PMID: 67233954. Language: English. Entry Date: 20120214. Revision Date: 20191111. Publication Type: Article.

1270. Adham MA, Hamad BA. Drug dispensing systems in Gaza hospitals: a comparative study...[corrected] [published erratum appears in EAST MEDITERRANEAN HEALTH J 2012; 17(2):948]. Eastern Mediterranean Health Journal. 2011;17(10):722-9. PubMed PMID: 104599425. Language: English. Entry Date: 20120214. Revision Date: 20150711. Publication Type: Journal Article.

1271. Adhien P, Van Dijk L, De Vegter M, Westein M, Nijpels G, Hugtenburg JG. Evaluation of a pilot study to influence medication adherence of patients with diabetes mellitus type-2 by the pharmacy. International Journal of Clinical Pharmacy. 2013;35(6):1113-9. doi: 10.1007/s11096-013-9834-4.

1272. Adhikari R, Tocher J, Smith P, Corcoran J, MacArthur J. A multi-disciplinary approach to medication safety and the implication for nursing education and practice. Nurse Education Today. 2014;34(2):185-90. doi: 10.1016/j.nedt.2013.10.008. PubMed PMID: 104130447. Language: English. Entry Date: 20140525. Revision Date: 20200708. Publication Type: Journal Article.

1273. Adhikari S, Mukhyopadhyay A, Kolzoff S, Li X, Nadel T, Fitchett C, et al. Cohort profile: a large EHR-based cohort with linked pharmacy refill and neighbourhood social determinants of health data to assess heart failure medication adherence. BMJ Open. 2023;13(12). doi: 10.1136/bmjopen-2023-076812.

1274. Adibe C. Impact of a Pharmacy-Led Medication History Program in the Emergency Center of a Large, Academic Hospital [M.S.]. United States -- Texas: University of Houston; 2018.

1275. Adibe MO, Igboeli NU, Ubaka CM, Udeogaranya PO, Onwudiwe NP, Ita OO. Evaluation of information contained in drug advertisement and promotion materials in Nigeria. Tropical Journal of Pharmaceutical Research. 2015;14(3):539-44. doi: 10.4314/tjpr.v14i3.25.

1276. Adie J, Graham W, Wallis M. Entry Points to the Health System: a review of the emerging community models for management of non-life threatening urgent conditions relevant to Australia. Asia Pacific Journal of Health Management. 2017;12(2):9-16. PubMed PMID: WOS:000417996900002.

1277. Adigwe OP. Policies and practices in Nigeria's pharmaceutical sector: A mixed methods exploration of stakeholders' perspectives on strategic reforms. Health Policy OPEN. 2023;4:100091. doi: https://doi.org/10.1016/j.hpopen.2023.100091.

1278. Adigwe OP. The role of pharmacists in eliminating counterfeit medicines in Nigeria. Frontiers in Public Health. 2023;11. doi: 10.3389/fpubh.2023.1170929. PubMed PMID: WOS:001058168600001.

1279. Adikusuma W, Qiyaam N. Adherence level and blood sugar control of type 2 diabetes mellitus patients who gets counseling and short messages service as reminder and motivation. Asian journal of pharmaceutical and clinical research. 2018;11(2):219-22. doi: 10.22159/ajpcr.2018.v11i2.22988. PubMed PMID: CN-01643961.

1280. Adil MS, Sultana R, Khulood D. PRIME study: Prescription review to impede medication errors. International Journal of Risk & Safety in Medicine. 2020;31(2):67-79. doi: 10.3233/JRS-191025. PubMed PMID: 149902828. Language: English. Entry Date: In Process. Revision Date: 20220904. Publication Type: Article. Journal Subset: Biomedical.

1281. Adinew A, Feyissa M, Tadesse B, Demeke B, Assefa T, Abdella M, et al. Assessment of patient satisfaction towards auditable pharmaceutical transactions and services implemented in outpatient hospital pharmacy in Ethiopia. Journal of Pharmaceutical Policy and Practice. 2021;14(1). doi: 10.1186/s40545-021-00372-1. PubMed PMID: WOS:000708860200001.

1282. Adisa R, Anifowose AT. Pharmacists' knowledge, attitude and involvement in palliative care in selected tertiary hospitals in southwestern Nigeria. Bmc Palliative Care. 2019;18(1). doi: 10.1186/s12904-019-0492-8. PubMed PMID: WOS:000499950900002.

1283. Adjei AA, Winch P, Laar A, Sullivan DJ, Sakyi KS, Stephens JK, et al. Insights into the Affordable Medicines Facility-malaria in Ghana: the role of caregivers and licensed chemical sellers in four regions. Malaria Journal. 2016;15. doi: 10.1186/s12936-016-1307-5. PubMed PMID: WOS:000375535100002.

1284. Adjie EA, Calista N, Muhtadiin RR, Handayani PW, Larasati PD. User switching intention from E-marketplace to E-pharmacy: The Influence of push, pull, and mooring factors. Informatics in Medicine Unlocked. 2023;43:101404. doi: https://doi.org/10.1016/j.imu.2023.101404.

1285. Adjuik M, Agnamey P, Babiker A, Borrmann S, Brasseur P, Cisse M, et al. Amodiaquine-artesunate versus amodiaquine for uncomplicated Plasmodium falciparum malaria in African children: a randomised, multicentre trial. The Lancet. 2002;359(9315):1365-72.

1286. Adkins K. Results from medication therapy management services for high-risk patients in an outpatient pharmacy. Journal of the American Pharmacists Association. 2011;51(5):650. PubMed PMID: 71331170.

1287. Adler DA, Bungay KM, Wilson IB, Pei Y, Supran S, Peckham E, et al. The impact of a pharmacist intervention on 6-month outcomes in depressed primary care patients. General hospital psychiatry. 2004;26(3):199-209. doi: 10.1016/j.genhosppsych.2003.08.005. PubMed PMID: CN-00481967.

1288. Adler NE, Stewart J. Reducing Obesity: Motivating Action While Not Blaming the Victim. Milbank Quarterly. 2009;87(1):49-70. doi: 10.1111/j.1468-0009.2009.00547.x. PubMed PMID: WOS:000264082300003.

1289. Adler-Milstein J, Salzberg C, Franz C, Orav EJ, Newhouse JP, Bates DW. Effect of electronic health records on health care costs: Longitudinal comparative evidence from community practices. Annals of Internal Medicine. 2013;159(2):97-104. doi: 10.7326/0003-4819-159-2-201307160-00004.

1290. Admire B, Yalkowsky SH. Predicting the Octanol Solubility of Organic Compounds. Journal of Pharmaceutical Sciences. 2013;102(7):2112-9. doi: https://doi.org/10.1002/jps.23561.

1291. Adnan M, Karim S, Khan S, Al-Wabel NA. Comparative evaluation of metered-dose inhaler technique demonstration among community pharmacists in Al Qassim and Al-Ahsa region, Saudi-Arabia. Saudi Pharmaceutical Journal. 2015;23(2):138-42.

1292. Adogu POU, Okechukwu R, Egenti NB, Emerole CO. Attitudes, Practice and Predictors of Rational Use of Medicines among Medicines Prescribers and Dispensers in Nnewi Nigeria. British Journal of Pharmaceutical Research. 2015;7(5):319-29. doi: 10.9734/bjpr/2015/18210. PubMed PMID: WOS:000219210500002.

1293. Adongo PB, Tabong PTN, Asampong E, Ansong J, Robalo M, Adanu RM. Preparing towards Preventing and Containing an Ebola Virus Disease Outbreak: What Sociocultural Practices May Affect Containment Efforts in Ghana? Plos Neglected Tropical Diseases. 2016;10(7). doi: 10.1371/journal.pntd.0004852. PubMed PMID: WOS:000381017800053.

1294. Adriano LS, Fonteles MMD, Azevedo MDM, Beserra MPP, Romero NR. Medication adherence in patients with juvenile idiopathic arthritis. Revista Brasileira De Reumatologia. 2017;57(1):23-9. doi: 10.1016/j.rbr.2015.11.004. PubMed PMID: WOS:000396445600004.

1295. Adsetts J. Aspects of the demographic profile and standard of pharmaceutical services in South Africa: North-West University; 2006.

1296. Adsuar-Meseguer GM, Santos-Cruz P, Pérez-Rodríguez RO, Granados-Flores J, de Ulierte EG, Alarcón MDL, et al. Strategy to optimize patient safety in the community pharmacy dispensing service. Proposed checklists. Farmaceuticos Comunitarios. 2023;15(2):29-40. doi: 10.33620/FC.2173-9218.(2023).15.

1297. Adu-Sarkodie Y, Steiner MJ, Attafuah J, Tweedy K. Syndromic management of urethral discharge in Ghanaian pharmacies. Sexually transmitted infections. 2000;76(6):439-42. doi: 10.1136/sti.76.6.439. PubMed PMID: CN-00399815.

1298. Adunlin G, Mahdavian S. The Effectiveness of Pharmacist Interventions on Asthma Management: A Systematic Review. Journal of Asthma and Allergy Educators. 2012;3(6):264-73. doi: 10.1177/2150129712464775.

1299. Adunlin G, Xiao H, Diaby V. Exploring the state of pharmacy profession: The perspective of CÔte d'Ivoire. Currents in Pharmacy Teaching and Learning. 2013;5(5):470-6. doi: 10.1016/j.cptl.2013.06.005.

1300. Advinha AM, de Oliveira-Martins S, Mateus V, Pajote SG, Lopes MJ. Medication regimen complexity in institutionalized elderly people in an aging society. International journal of clinical pharmacy. 2014;36:750-6.

1301. Advinha AM, Nunes C, de Barros CT, Lopes MJ, de Oliveira-Martins S. Key factors of the functional ability of older people to self-manage medications. Scientific Reports. 2021;11(1). doi: 10.1038/s41598-021-01434-9.

1302. Advinha AMM. Avaliação Da Capacidade Funcional Da população Idosa Na gestão Da Sua medicação [Ph.D.]. Portugal: Universidade de Lisboa (Portugal); 2017.

1303. Afanasjeva J, Gabay M, Poznanski T, Kerns S. Transdermal Patch Administration and Magnetic Resonance Imaging (MRI)—2020. Hospital Pharmacy. 2022;57(1):117-20. doi: 10.1177/0018578720987138.

PubMed PMID: 156651439. Language: English. Entry Date: 20220526. Revision Date: 20220526. Publication Type: Article.

1304. Afanasjeva J, Gruenberg K. Pharmacists as environmental stewards: Strategies for minimizing and managing drug waste. Sustainable Chemistry and Pharmacy. 2019;13. doi: 10.1016/j.scp.2019.100164.

1305. Afari-Asiedu S, Abdulai MA, Tostmann A, Boamah-Kaali E, Asante KP, Wertheim HFL, et al. Interventions to improve dispensing of antibiotics at the community level in low and middle income countries: a systematic review. Journal of Global Antimicrobial Resistance. 2022;29:259-74. doi: 10.1016/j.jgar.2022.03.009.

1306. Afari-Asiedu S, Boamah-Kaali E, Abdulai MA, Gyaase S, Oppong FB, Tostmann A, et al. Determinants of inappropriate antibiotics use in rural central Ghana: a mixed methods approach. American journal of tropical medicine and hygiene. 2019;101(5):343-. doi: 10.4269/ajtmh.abstract2019. PubMed PMID: CN-02076957.

1307. Afari-Asiedu S, Hulscher M, Abdulai MA, Boamah-Kaali E, Asante KP, Wertheim HFL. Every medicine is medicine; Exploring inappropriate antibiotic use at the community level in rural Ghana. BMC Public Health. 2020;20(1). doi: 10.1186/s12889-020-09204-4.

1308. Afari-Asiedu S, Hulscher M, Abdulai MA, Boamah-Kaali E, Wertheim HFL, Asante KP. Stakeholders' perspectives on training over the counter medicine sellers and Community-based Health Planning and Services facilities to dispense antibiotics in Ghana. Journal of Pharmaceutical Policy and Practice. 2021;14(1). doi: 10.1186/s40545-021-00349-0.

1309. Afari-Asiedu S, Oppong FB, Tostmann A, Abdulai MA, Boamah-Kaali E, Gyaase S, et al. Determinants of Inappropriate Antibiotics Use in Rural Central Ghana Using a Mixed Methods Approach. Frontiers in Public Health. 2020;8. doi: 10.3389/fpubh.2020.00090. PubMed PMID: WOS:000596899500001.

1310. Afari-Asiedu S, Oppong FB, Tostmann A, Ali Abdulai M, Boamah-Kaali E, Gyaase S, et al. Determinants of Inappropriate Antibiotics Use in Rural Central Ghana Using a Mixed Methods Approach. Frontiers in Public Health. 2020;8. doi: 10.3389/fpubh.2020.00090.

1311. Afezolli D, Austin VD, Flemig D, Easton E, Tortu E, Scarborough B. Narcan or Can't? Opioid Overdose Education for Patients with Cancer Pain. Journal of Pain and Symptom Management. 2022;63(5):873-4. doi: https://doi.org/10.1016/j.jpainsymman.2022.02.068.

1312. Afezolli D, Flemig D, Easton E, Austin V, Scarborough B, Smith CB. Standard Naloxone Prescribing for Palliative Care Cancer Patients on Opioid Therapy: A Single-Site Quality Improvement Pilot to Assess Attitudes and Access. Journal of Pain and Symptom Management. 2022. doi: https://doi.org/10.1016/j.jpainsymman.2022.12.014.

1313. Afezolli D, Flemig D, Easton E, Austin V, Scarborough B, Smith CB. Standard Naloxone Prescribing for Palliative Care Cancer Patients on Opioid Therapy: A Single-Site Quality Improvement Pilot to Assess Attitudes and Access. Journal of Pain and Symptom Management. 2023;65(4):e309-e14. doi: https://doi.org/10.1016/j.jpainsymman.2022.12.014.

1314. Affum AO, Lowor S, Osae SD, Dickson A, Gyan BA, Tulasi D. A pilot study on quality of artesunate and amodiaquine tablets used in the fishing community of Tema, Ghana. Malaria Journal. 2013;12. doi: 10.1186/1475-2875-12-220. PubMed PMID: WOS:000322204500001.

1315. Affum AO, Shiloh DO, Adomako D. Monitoring of arsenic levels in some ready-to-use anti-malaria

herbal products from drug sales outlets in the Madina area of Accra, Ghana. Food and Chemical Toxicology. 2013;56:131-5. doi: https://doi.org/10.1016/j.fct.2013.01.049.

1316. Afolabi A. Factors influencing the pattern of self-medication in an adult Nigerian population. Annals of African medicine. 2008;7(3):120-7.

1317. Afolabi MO, Afolabi ERI, Faleye BA. Construct validation of an instrument to measure patient satisfaction with pharmacy services in Nigerian hospitals. African Health Sciences. 2012;12(4):538-44. doi: 10.4314/ahs.v12i4.22. PubMed PMID: WOS:000316933800023.

1318. Afonso-Silva M, Laires PA. PCV24 REAL-WORLD COST-EFFECTIVENESS OF SACUBITRIL/VALSARTAN IN SYMPTOMATIC HEART FAILURE WITH REDUCED EJECTION FRACTION IN PORTUGAL. Value in health. 2019;22:S545-. doi: 10.1016/j.jval.2019.09.749. PubMed PMID: CN-02052769.

1319. Afriyie DK, Sefah IA, Sneddon J, Malcolm W, McKinney R, Cooper L, et al. Antimicrobial point prevalence surveys in two Ghanaian hospitals: opportunities for antimicrobial stewardship. Jac-Antimicrobial Resistance. 2020;2(1). doi: 10.1093/jacamr/dlaa001. PubMed PMID: WOS:000733828600001.

1320. Afshar M, Oguss M, Callaci TA, Gruenloh T, Gupta P, Sun C, et al. Creation of a data commons for substance misuse related health research through privacy-preserving patient record linkage between hospitals and state agencies. JAMIA Open. 2023;6(4). doi: 10.1093/jamiaopen/ooad092.

1321. Afzali M, Khorasani E, Alvandi M, Sabbagh-Bani-Azad M, Sharif Z, Saiyarsarai P, et al. Providing a framework for assessment of the access to medicine. Daru-Journal of Pharmaceutical Sciences. 2019;27(1):243-54. doi: 10.1007/s40199-019-00268-1. PubMed PMID: WOS:000472907200023.

1322. Agar R, Prendergast M, Maher V. Evaluation of lipid services in the Republic of Ireland. Irish Journal of Medical Science. 2020;189(3):925-31. doi: 10.1007/s11845-020-02187-6.

1323. Agarwal P. Longitudinal Patterns and Economic Consequences of Emergency Department Visits among Medicaid Enrollees [Ph.D.]. United States -- West Virginia: West Virginia University; 2015.

1324. Agarwal P, Frid I, Singer J, Zalatimo O, Schirmer CM, Kimmell KT, et al. Neurosurgery perception of Enhanced Recovery After Surgery (ERAS) protocols. Journal of Clinical Neuroscience. 2021;92:110-4. doi: https://doi.org/10.1016/j.jocn.2021.07.044.

1325. Agarwal P, Mukerji G, Laur C, Chandra S, Pimlott N, Heisey R, et al. Adoption, feasibility and safety of a family medicine-led remote monitoring program for patients with COVID-19: a descriptive study. CMAJ open. 2021;9(2):E324-E30. doi: 10.9778/cmajo.20200174.

1326. Agarwal P, Poeran J, Meyer J, Rogers L, Reich DL, Mazumdar M. Bedside medication delivery programs: suggestions for systematic evaluation and reporting. International Journal for Quality in Health Care. 2019;31(8):G53-G9. doi: 10.1093/intqhc/mzz014. PubMed PMID: 141945614. Language: English. Entry Date: 20200603. Revision Date: 20200930. Publication Type: journal article.

1327. Agarwala A, Satish P, Al Rifai M, Mehta A, Cainzos-Achirica M, Shah NS, et al. Identification and Management of Atherosclerotic Cardiovascular Disease Risk in South Asian Populations in the U.S. JACC: Advances. 2023;2(2):100258. doi: https://doi.org/10.1016/j.jacadv.2023.100258.

1328. Agashivala NV, Dastani HB, Carlton R, Sarnes E. Cost-effectiveness of fingolimod in the treatment of patients with relapsing remitting multiple sclerosis. Journal of managed care pharmacy. 2011;17(3):241. PubMed PMID: CN-01030099.

1329. Agashivala NV, Dastani HB, Carlton R, Sarnes E. Cost-effectiveness of oral fingolimod and intramuscular interferon-beta 1a for relapsing remitting multiple sclerosis (RRMS): results based on efficacy inputs from a head-to-head study. Journal of managed care pharmacy. 2011;17(3):272-. PubMed PMID: CN-01004215.

1330. Agbabiaka TB, Savović J, Ernst E. Methods for causality assessment of adverse drug reactions: a systematic review. Drug safety. 2008;31:21-37.

1331. Agboola F, Reddy P. Conversion from Filgrastim to Tbo-filgrastim: Experience of a Large Health Care System. Journal of Managed Care & Specialty Pharmacy. 2017;23(12):1214-8. doi: 10.18553/jmcp.2017.23.12.1214. PubMed PMID: WOS:000416249300001.

1332. Agbor MA, Azodo CC. Self medication for oral health problems in Cameroon. International Dental Journal. 2011;61(4):204-9. doi: https://doi.org/10.1111/j.1875-595X.2011.00058.x.

1333. Agee C, Coulter L, Hudson J. Effects of pharmacy resident led education on resident physician prescribing habits associated with stress ulcer prophylaxis in non-intensive care unit patients. American Journal of Health-System Pharmacy. 2015;72(11 Suppl 1):S48-52. PubMed PMID: 25991596.

1334. Aggarwal AM. An examination of the role of the community pharmacist in the pharmacovigilance of herbal medicines [Ph.D.]. England: University of London, University College London (United Kingdom); 2008.

1335. Aggarwal NR, Molina KC, Beaty LE, Bennett TD, Carlson NE, Mayer DA, et al. Real-world use of nirmatrelvir–ritonavir in outpatients with COVID-19 during the era of omicron variants including BA.4 and BA.5 in Colorado, USA: a retrospective cohort study. The Lancet Infectious Diseases. 2023;23(6):696-705. doi: https://doi.org/10.1016/S1473-3099(23)00011-7.

1336. Aggarwal NR, Molina KC, Beaty LE, Bennett TD, Carlson NE, Mayer DA, et al. Real-world use of nirmatrelvir-ritonavir in outpatients with COVID-19 during the era of omicron variants including BA.4 and BA.5 in Colorado, USA: a retrospective cohort study. Lancet Infectious Diseases. 2023;23(6):696-705. doi: 10.1016/s1473-3099(23)00011-7. PubMed PMID: WOS:001010916000001.

1337. Aggarwal SC. Materials management: The key is people management (five brief case histories). Journal of Operations Management. 1985;6(1):43-9. doi: https://doi.org/10.1016/0272-6963(85)90034-8.

1338. Aggarwal SP, Zinman L, Simpson E, McKinley J, Jackson KE, Pinto H, et al. Safety and efficacy of lithium in combination with riluzole for treatment of amyotrophic lateral sclerosis: a randomised, double-blind, placebo-controlled trial. The Lancet Neurology. 2010;9(5):481-8. doi: https://doi.org/10.1016/S1474-4422(10)70068-5.

1339. Aggarwal SP, Zinman L, Simpson E, McKinley J, Jackson KE, Pinto H, et al. Safety and efficacy of lithium in combination with riluzole for treatment of amyotrophic lateral sclerosis: a randomised, double-blind, placebo-controlled trial. The lancet Neurology. 2010;9(5):481-8. doi: 10.1016/S1474-4422(10)70068-5. PubMed PMID: CN-00742644.

1340. Agger JL, Schroder A, Gormsen LK, Jensen JS, Jensen TS, Fink PK. Imipramine versus placebo for multiple functional somatic syndromes (STreSS-3): a double-blind, randomised study. The lancet psychiatry. 2017;4(5):378-88. doi: 10.1016/S2215-0366(17)30126-8. PubMed PMID: CN-01366251.

1341. Agha S, Karim AM, Balal A, Sosler S. The impact of a reproductive health franchise on client satisfaction in rural Nepal. Health Policy and Planning. 2007;22(5):320-8. doi: 10.1093/heapol/czm025.

1342. Aghili M, Kasturirangan MN. A clinical pharmacist-led integrated approach for evaluation of medication errors among medical intensive care unit patients. International Journal of Evidence-Based Healthcare. 2021;19(1):21-30. doi: 10.1097/XEB.0000000000000228. PubMed PMID: 149643259. Language: English. Entry Date: 20210412. Revision Date: 20210412. Publication Type: Article.

1343. Aghili M, Neelathahalli Kasturirangan M. A clinical pharmacist-led integrated approach for evaluation of medication errors among medical intensive care unit patients. JBI Evidence Implementation. 2021;19(1):21-30. PubMed PMID: 33570331.

1344. Aghnatios L, Rofes GF, Sorli SC, Cestac P, Pages A, Eyvrard F. Covid-19: The impact of lockdown on the dispensing of hospital drugs to outpatients. Sante Publique. 2023;35(6):39-51. doi: 10.3917/spub.236.0039. PubMed PMID: WOS:001183773700005.

1345. Aghvami S. Techniques for the Initiation and Study of the Self-Assembly of Biopolymers [Ph.D.]. United States -- Massachusetts: Brandeis University; 2020.

1346. Agiro A, Dwyer JP, Oluwatosin Y, Desai P. Medical Costs in Patients with Hyperkalemia on Long-Term Sodium Zirconium Cyclosilicate Therapy: The RECOGNIZE II Study. Clinicoeconomics and Outcomes Research. 2023;15:691-702. doi: 10.2147/ceor.S420217. PubMed PMID: WOS:001077809500001.

1347. Agiro A, Gautam S, Wall E, Hackell J, Helm M, Barron J, et al. Variation in Outpatient Antibiotic Dispensing for Respiratory Infections in Children by Clinician Specialty and Treatment Setting. Pediatric Infectious Disease Journal. 2018;37(12):1248-54. doi: 10.1097/INF.0000000000002004.

1348. Agius A, Azzopardi LM, Serracino Inglott A. Faecal occult blood testing in community pharmacy. International journal of pharmacy practice. 2012;20:61-2. doi: 10.1111/j.2042-7174.2012.00235.x. PubMed PMID: CN-01030997.

1349. Agley J, Meyerson BE, Eldridge LA, Crosby RA, Bentele KG, Jun M, et al. Exploration of pharmacist comfort with harm reduction behaviors: Cross-sectional latent class analysis. Journal of the American Pharmacists Association. 2022;62(2):432-40. doi: https://doi.org/10.1016/j.japh.2021.10.015.

1350. Agley J, Meyerson BE, Eldridge LA, Crosby RA, Bentele KG, Jun MY, et al. Exploration of pharmacist comfort with harm reduction behaviors: Cross-sectional latent class analysis. Journal of the American Pharmacists Association. 2022;62(2):432-40. doi: 10.1016/j.japh.2021.10.015. PubMed PMID: WOS:000804552700009.

1351. Agner S, Groover S. On-line, real-time drug utilization review: Evolution toward decision support. Therapeutic Innovation & Regulatory Science. 1996;30(3):753-9. doi: 10.1177/009286159603000319.

1352. Agness CF, Huynh D, Brandt N. An introductory pharmacy practice experience based on a medication therapy management service model. American Journal of Pharmaceutical Education. 2011;75(5). doi: 10.5688/ajpe75582.

1353. Agomo C, Rowlands G, Ashworth M, Reid F. An investigation of nicotine replacement therapy provision by community pharmacists in Wandsworth Primary Care Trust, London. Pharmaceutical Journal. 2006;277(7427):609-11.

1354. Agomo C, Udoh A, Kpokiri E, Osuku-Opio J. Community pharmacists' contribution to public health: Assessing the global evidence base. Clinical Pharmacist. 2018;10(4). doi: 10.1211/CP.2018.20204556.

1355. Agomo CO. The role of community pharmacists in public health: a scoping review of the literature.

Journal of Pharmaceutical Health Services Research. 2012;3(1):25-33.

1356. Agomo CO, Ogunleye J. An investigation of strategies enhancing the public health role of community pharmacists: A review of knowledge and information. Journal of Pharmaceutical Health Services Research. 2014;5(2):135-45. doi: 10.1111/jphs.12056.

1357. Agomo CO, Ogunleye J, Portlock J. A survey to identify barriers in the public health role of community pharmacists. Journal of pharmaceutical health services research. 2016;7(4):253-61.

1358. Agomo CO, Ogunleye J, Portlock J. Strategies enhancing the public health role of community pharmacists: a qualitative study. Journal of Pharmaceutical Health Services Research. 2016;7(2):97-104. doi: 10.1111/jphs.12131.

1359. Agrawal A. Medication errors: prevention using information technology systems. British journal of clinical pharmacology. 2009;67(6):681.

1360. Agrawal A, Aronson JK, Britten N, Ferner RE, De Smet PA, Fialová D, et al. Medication errors: problems and recommendations from a consensus meeting. British journal of clinical pharmacology. 2009;67(6):592.

1361. Agrawal A, Wu WY. Reducing medication errors and improving systems reliability using an electronic medication reconciliation system. The Joint Commission Journal on Quality and Patient Safety. 2009;35(2):106-14.

1362. Agrawal AM, Pandey P. Scale Up of Pan Coating Process Using Quality by Design Principles. Journal of Pharmaceutical Sciences. 2015;104(11):3589-611. doi: https://doi.org/10.1002/jps.24582.

1363. Agrawal S, Wable R, Benison F, Mathew S, Pawar A, Deshpande PR. Knowledge, attitude, practice, and cost assessment of over the counter drugs in female athletes in Pune city, India. International Journal of Health and Allied Sciences. 2021;10(1):70-7. doi: 10.4103/ijhas.IJHAS_7_20. PubMed PMID: WOS:000621668100011.

1364. Agrawala PK, Adhikari J, Chaudhury N. Lymphocyte chromosomal aberration assay in radiation biodosimetry. Journal of pharmacy and bioallied sciences. 2010;2(3):197.

1365. Agu KA, Oqua D, Agada P, Ohiaeri SI, Adesina A, Abdulkareem MH, et al. Assessment of satisfaction with pharmaceutical services in patients receiving antiretroviral therapy in outpatient HIV treatment setting. International Journal of Clinical Pharmacy. 2014;36(3):636-47. doi: 10.1007/s11096-014-9948-3. PubMed PMID: WOS:000336222200021.

1366. Aguado-Lorenzo V, Weeks K, Tunstell P, Turnock K, Watts T, Arenas-Lopez S. Accuracy of the concentration of morphine infusions prepared for patients in a neonatal intensive care unit. Archives of Disease in Childhood. 2013;98(12):975-9. doi: 10.1136/archdischild-2013-304522. PubMed PMID: 104114340. Language: English. Entry Date: 20140124. Revision Date: 20200708. Publication Type: Journal Article.

1367. Aguas Compaired M, Pons Busom M, Delàs Amat J, Eguileor Partearroyo B. Implementing protocols in a university hospital. An assessment of various methods. Farmacia Hospitalaria. 2004;28(5):334-48.

1368. Agüera Ortiz LF, García-Ribas G, Jordano Luna F, Porras Álvarez MC, Sánchez Marcos N, Soler López B. Usefulness of Community Pharmacy for Early Detection of Cognitive Impairment in Older People Using the IQ-CODE Questionnaire. Journal of Prevention of Alzheimer's Disease. 2023;10(3):488-96. doi: 10.14283/jpad.2023.39.

1369. Aguiar JP, Costa LH, da Costa FA, Leufkens HGM, Martins AP. Identification of potentially inappropriate medications with risk of major adverse cardiac and cerebrovascular events among elderly patients in ambulatory setting and long-term care facilities. Clinical Interventions in Aging. 2019;14:535-47. doi: 10.2147/CIA.S192252.

1370. Aguiar JP, Heitor Costa L, Alves da Costa F, Leufkens HG, Martins AP. Identification of potentially inappropriate medications with risk of major adverse cardiac and cerebrovascular events among elderly patients in ambulatory setting and long-term care facilities. Clinical Interventions In Aging. 2019;14:535-47. PubMed PMID: 30880934.

1371. Aguiar PM, Balisa-Rocha BJ, Brito GC, Lyra DP. Pharmaceutical care program for elderly patients with uncontrolled hypertension. Journal of the American Pharmacists Association. 2012;52(4):515-8. doi: https://doi.org/10.1331/JAPhA.2012.11015.

1372. Aguiar PM, Balisa-Rocha BJ, Brito GC, Lyra Jr DP. Pharmaceutical care program for elderly patients with uncontrolled hypertension. Journal of the American Pharmacists Association. 2012;52(4):515-8. doi: 10.1331/JAPhA.2012.11015.

1373. Aguiar PM, Brito GD, Correr CJ, Lyra DP, Storpirtis S. Exploring the Quality of Systematic Reviews on Pharmacist Interventions in Patients With Diabetes: An Overview. Annals of Pharmacotherapy. 2014;48(7):887-96. doi: 10.1177/1060028014529411. PubMed PMID: WOS:000337650600007.

1374. Aguiar PM, da Silva CHP, Chiann C, Dórea EL, Lyra DP, Storpirtis S. Pharmacist-physician collaborative care model for patients with uncontrolled type 2 diabetes in Brazil: results from a randomized controlled trial. Journal of evaluation in clinical practice. 2018;24(1):22-30. doi: 10.1111/jep.12606. PubMed PMID: CN-01656524.

1375. Aguiar PM, da Silva CHP, Chiann C, Dorea EL, Lyra DP, Storpirtis S. Pharmacist–physician collaborative care model for patients with uncontrolled type 2 diabetes in Brazil: results from a randomized controlled trial. Journal of evaluation in clinical practice. 2018;24(1):22-30. doi: 10.1111/jep.12606. PubMed PMID: CN-01248504.

1376. Aguiar PM, Mori A, de Lima MGF, Rossi M, Nicoletti MA, Martins KOF, et al. The effects of pharmacist interventions on health outcomes in patients with advanced prostate cancer in Brazil. Brazilian Journal of Pharmaceutical Sciences. 2022;58. doi: 10.1590/s2175-97902021000219273. PubMed PMID: WOS:000789269400001.

1377. Aguiar PM, Mori ALPM, de Lima MGF, Rossi MSPN, Nicoletti MA, Martins KOF, et al. The effects of pharmacist interventionon health outcomes in patients with advanced prostate cancer in Brazil. Brazilian Journal of Pharmaceutical Sciences. 2022;58. doi: 10.1590/s2175-97902021000219273.

1378. Aguilar C, Chau C, Giridharan N, Huh Y, Cooley J, Warholak TL. How to plan workflow changes: A practical quality improvement tool used in an outpatient hospital pharmacy. Journal of Pharmacy Practice. 2013;26(3):214-9. PubMed PMID: 369098520.

1379. Aguilar Ferrandiz ME, Nijs J, Gidron Y, Roussel N, Vanderstraeten R, Van Dyck D, et al. Auto-Targeted Neurostimulation Is Not Superior to Placebo in Chronic Low Back Pain: a Fourfold Blind Randomized Clinical Trial. Pain physician. 2016;19(5):E707-E19. PubMed PMID: CN-01445625.

1380. Aguilar KM, Hou QJ, Miller RM. Impact of Employer-Sponsored Onsite Pharmacy and Condition Management Programs on Medication Adherence. Journal of Managed Care & Specialty Pharmacy. 2015;21(8):670-7. doi: 10.18553/jmcp.2015.21.8.670. PubMed PMID: WOS:000369191000005.

1381. Aguilar-Salmeron R, de Gamarra-Martinez EF, Garcia-Pelaez M, Broto-Sumalla A, Martinez-Sanchez L, Nogue-Xarau S. Creation of a virtual antidotes network between pharmacy departments of catalan hospitals. Farmacia Hospitalaria. 2017;41(3):317-33. doi: 10.7399/fh.2017.41.3.10507. PubMed PMID: WOS:000402581600001.

1382. Aguilar-Salmerón R, De Gamarra-Martínez EF, García-Peléez M, Broto-Sumalla A, Martínez-Sénchez L, Nogué-Xarau S. Creation of a virtual antidotes network between pharmacy Departments of Catalan Hospitals. Farmacia Hospitalaria. 2017;41(3):317-33. doi: 10.7399/fh.2017.41.3.10507.

1383. Aguiló Juanola MC. Introduction to quality in community pharmacy (II): The separate consultation room. Pharmaceutical Care Espana. 2007;9(2):91-6.

1384. Agus TP, Suratri MAL, Fajarwati T. Case Study in Indonesia: Compliance of Filling the Clinical Pathway of Ischemic Stroke and STEMI in Several Hospitals, 2019. Media Penelitian Dan Pengembangan Kesehatan. 2021;31(4):319-26. doi: 10.22435/mpk.v31i4.4995. PubMed PMID: WOS:000756913800006.

1385. Aguwa CN, Ukwe CV, Adibe MO. A comparative study of quinine and artemether in the treatment of severe malaria in Nigerian children. Tropical journal of pharmaceutical research. 2010;9(1):11-7. PubMed PMID: CN-00746684.

1386. Aguwa CN, Ukwe CV, Ekwunife OI. Effect of pharmaceutical care programme on blood pressure and quality of life in a Nigerian pharmacy. Pharmacy World and Science. 2008;30(1):107-10. doi: 10.1007/s11096-007-9151-x.

1387. Agyakwa W, Lubbe MS, Burger JR, Katende-Kyenda NL, Cockeran M, Malik M. Fluoroquinolone Prescribing Patterns in the Private Healthcare Sector of South Africa, 2005-2012. Latin American Journal of Pharmacy. 2015;34(9):1815-22. PubMed PMID: WOS:000368212000018.

1388. Agyemang REO, While A. Medication errors: types, causes and impact on nursing practice. British Journal of Nursing. 2010;19(6):380-5.

1389. Ah YM, Hwang S, Jun K, Choi SA, Yu YM, Kim E, et al. Real-world safety evaluation of topical corticosteroid use: A community pharmacy-based, prospective, observational study. Basic & Clinical Pharmacology & Toxicology. 2019;125(4):405-13. PubMed PMID: 30983117.

1390. Ah YM, Lee JY, Moon MR, Cho YS, Kim HS, Yi NJ, et al. Clinical and economic evaluation of pharmacists' contribution to patient care on a multi-disciplinary liver transplant team. International Journal of Clinical Pharmacology & Therapeutics. 2016;54(2):102-9. PubMed PMID: 26709599.

1391. Ahamad K, Hayashi K, Nguyen P, Dobrer S, Kerr T, Montaner JS, et al. Effect of low-threshold methadone maintenance therapy for people who inject drugs on HIV incidence in Vancouver, BC, Canada: An observational cohort study. The Lancet HIV. 2015;2(10):e445-e50. doi: 10.1016/S2352-3018(15)00129-0.

1392. Ahamad K, Hayashi K, Nguyen P, Dobrer S, Kerr T, Schutz CG, et al. Effect of low-threshold methadone maintenance therapy for people who inject drugs on HIV incidence in Vancouver, BC, Canada: an observational cohort study. Lancet Hiv. 2015;2(10):E445-E50. doi: 10.1016/s2352-3018(15)00129-0. PubMed PMID: WOS:000363794100014.

1393. Ahamad T, Ananda KN, Ghanashyam SD, Tekkalaki Bheemsain V. Effectiveness of clinical pharmacist led collaborative approach towards medication adherence in patients with schizophrenia receiving atypical antipsychotics at tertiary care hospital. International journal of research in ayurveda and pharmacy. 2019;10(3):87-91. doi: 10.7897/2277-4343.100369. PubMed PMID: CN-01988002.

1394. Ahamed TI, Vaishnavi K, Harini RR, Yalavarthi L, Jagadeesan M. Prescription Audit and Dispensing Errors in the Outpatient Pharmacy of Tertiary Care Multispecialty Teaching Hospital. Journal of Pharmaceutical Research International. 2021;33(54B):303-8. doi: 10.9734/JPRI/2021/v33i54B33790. PubMed PMID: WOS:000732676400033.

1395. Aharaz A, Henning Rasmussen J, Bach Olgaard Mcnulty H, Cyron A, Pedersen Bengaard AK, Lovig Simonsen RR, et al. Pharmacist led deprescribing and follow-up in a subacute multidisciplinary outpatient clinic: a pilot randomised controlled trial. European journal of hospital pharmacy. 2021;28(SUPPL 1):A171-. doi: 10.1136/ejhpharm-2021-eahpconf.357. PubMed PMID: CN-02262799.

1396. Aharaz A, Rasmussen JH, McNulty HBO, Cyron A, Fabricius PK, Bengaard AK, et al. A collaborative deprescribing intervention in a subacute medical outpatient clinic: a pilot randomized controlled trial. Metabolites. 2021;11(4). doi: 10.3390/metabo11040204. PubMed PMID: CN-02264595.

1397. Ahc M. Hospital Leverages Pharmacists, Trained Technicians to Reduce Medication History Errors. ED Management. 2018;30(2):5-N.PAG. PubMed PMID: 127162055. Language: English. Entry Date: 20180110. Revision Date: 20180530. Publication Type: Article. Journal Subset: Nursing.

1398. Ahc M. Incomplete Medication Lists Can Lead to Allegations of Negligence. ED Legal Letter. 2021;32(2):1-3. PubMed PMID: 148159949. Language: English. Entry Date: 20210121. Revision Date: 20210121. Publication Type: Article. Journal Subset: Biomedical.

1399. Ahearn EP, Chen P, Hertzberg M, Cornette M, Suvalsky L, Cooley-Olson D, et al. Suicide attempts in veterans with bipolar disorder during treatment with lithium, divalproex, and atypical antipsychotics. Journal of Affective Disorders. 2013;145(1):77-82. doi: 10.1016/j.jad.2012.07.015. PubMed PMID: WOS:000314091800011.

1400. Ahern TP. Estimating the impact of molecular profiles and prescription drugs on breast cancer outcomes [Ph.D.]. United States -- Massachusetts: Boston University; 2010.

1401. Ahiabu M-A, Magnussen P, Bygbjerg IC, Tersbøl BP. Treatment practices of households and antibiotic dispensing in medicine outlets in developing countries: The case of Ghana. Research in Social & Administrative Pharmacy. 2018;14(12):1180-8. doi: 10.1016/j.sapharm.2018.01.013. PubMed PMID: 132897639. Language: English. Entry Date: 20190406. Revision Date: 20190627. Publication Type: journal article.

1402. Ahiabu MA, Magnussen P, Bygbjerg IC, Tersbøl BP. Treatment practices of households and antibiotic dispensing in medicine outlets in developing countries: The case of Ghana. Research in Social and Administrative Pharmacy. 2018;14(12):1180-8. doi: 10.1016/j.sapharm.2018.01.013.

1403. Ahissou NCA, Benova L, Delvaux T, Gryseels C, Dossou JP, Goufodji S, et al. Modern contraceptive use among adolescent girls and young women in Benin: A mixed-methods study. BMJ Open. 2022;12(1). doi: 10.1136/bmjopen-2021-054188.

1404. Ahluwalia JS, Weisenberger ML, Bernard AM, McNagny SE. Changing physician prescribing behavior: A low-cost administrative policy that reduced the use of brand name nonsteroidal anti-inflammatory drugs. Preventive Medicine. 1996;25(6):668-72. doi: 10.1006/pmed.1996.0105.

1405. Ahmad A. Identifying Key Factors Associated with High Risk Asthma Patients to Reduce the Cost of Health Resources Utilization [M.Sc.]. United States -- Louisiana: Louisiana State University and Agricultural & Mechanical College; 2018.

1406. Ahmad A, Atique S, Balkrishnan R, Patel I. Pharmacy profession in India: Current scenario and

recommendations. Indian Journal of Pharmaceutical Education and Research. 2014;48(3):12-5. doi: 10.5530/ijper.48.3.3.

1407. Ahmad A, Dhanalekshmi UM, Koumaravelu K, Francis AP, Khan SA, Abuzinadah MF, et al. A Study on Pharmacokinetic Functionalities and Safety Margins of an Optimized Simvastatin Nanoformulation. Pharmaceuticals (14248247). 2023;16(3):380. doi: 10.3390/ph16030380. PubMed PMID: 162816046.

1408. Ahmad A, Hugtenburg J, Welschen L, Dekker JM, Nijpels G. Effect of medication review and cognitive behaviour treatment by community pharmacists of patients discharged from the hospital on drug related problems and compliance: design of a randomized controlled trial. BMC Public Health. 2010;10(1):1-10.

1409. Ahmad A, Hugtenburg J, Welschen LM, Dekker JM, Nijpels G. Effect of medication review and cognitive behaviour treatment by community pharmacists of patients discharged from the hospital on drug related problems and compliance: design of a randomized controlled trial. BMC public health. 2010;10:133. doi: 10.1186/1471-2458-10-133. PubMed PMID: CN-00813524.

1410. Ahmad A, Hugtenburg J, Welschen LMC, Dekker JM, Nijpels G. Effect of medication review and cognitive behaviour treatment by community pharmacists of patients discharged from the hospital on drug related problems and compliance: Design of a randomized controlled trial. BMC Public Health. 2010;10. doi: 10.1186/1471-2458-10-133.

1411. Ahmad A, Khan MU, Moorthy J, Jamshed SQ, Patel I. Comparison of knowledge and attitudes about antibiotics and resistance, and antibiotics selfpracticing between Bachelor of Pharmacy and Doctor of Pharmacy students in Southern India. Pharmacy Practice. 2015;13(1). doi: 10.18549/PharmPract.2015.01.523.

1412. Ahmad A, Khan MU, Moorthy J, Kumar BD, Kumar GS, Patel I. Comparison of knowledge, attitudes and perceived barriers towards adverse drug reactions reporting between Bachelor of Pharmacy and Doctor of Pharmacy students in Southern India. Journal of Pharmaceutical Health Services Research. 2016;7(1):63-9. doi: 10.1111/jphs.12128.

1413. Ahmad A, Khan MU, Patel I, Maharaj S, Pandey S, Dhingra S. Knowledge, attitude and practice of B.Sc. Pharmacy students about antibiotics in Trinidad and Tobago. Journal of Research in Pharmacy Practice. 2015;4(1):37-41. doi: 10.4103/2279-042x.150057. PubMed PMID: WOS:000218630600007.

1414. Ahmad A, Khan MU, Srikanth AB, Kumar B, Singh NK, Trivedi N, et al. Evaluation of knowledge, attitude and practice about self-medication among rural and urban north Indian population. International Journal of Pharmaceutical and Clinical Research. 2015;7(5):326-32.

1415. Ahmad A, Mast MR, Nijpels G, Elders PJ, Dekker JM, Hugtenburg JG. Identification of drug-related problems of elderly patients discharged from hospital. Patient preference and adherence. 2014:155-65.

1416. Ahmad A, Mast MR, Nijpels G, Elders PJM, Dekker JM, Hugtenburg JG. Identification of drug-related problems of elderly patients discharged from hospital. Patient Preference and Adherence. 2014;8:155-65. doi: 10.2147/PPA.S48357.

1417. Ahmad A, Patel I, Sanyal S, Balkrishnan R, Mohanta GP. Availability, cost and affordability of antimalarial medicines in India! International Journal of Pharmaceutical and Clinical Research. 2014;6(1):7-12.

1418. Ahmad F, Ismail SB, Yusof HM. Prescription errors in hospital Universiti Sains Malaysia, Kelantan, Malaysia. International Medical Journal. 2006;13(2):101-4. PubMed PMID: 106312967. Language: English.

Entry Date: 20060804. Revision Date: 20150711. Publication Type: Journal Article.

1419. Ahmad R, Naqvi AA, Al-Bukhaytan HM, Al-Nasser AH, Baqer Al-Ebrahim AH. Evaluation of aromatherapy with lavender oil on academic stress: a randomized placebo controlled clinical trial. Contemporary clinical trials communications. 2019;14. doi: 10.1016/j.conctc.2019.100346. PubMed PMID: CN-01915574.

1420. Ahmad S, Abdul Qadir M, Ahmed M, Imran M, Yousaf N, Wani TA, et al. New Acetamide-Sulfonamide-Containing Scaffolds: Antiurease Activity Screening, Structure-Activity Relationship, Kinetics Mechanism, Molecular Docking, and MD Simulation Studies. Molecules. 2023;28(14):5389. doi: 10.3390/molecules28145389. PubMed PMID: 169330641.

1421. Ahmad S, Alam MS, Alam N, Alqahtani SS, Sultan MH, Banji D, et al. In Vitro Bioequivalence Study and Quality Control Assessment of Different Brands of Omeprazole Capsules Available in Jazan, KSA. Latin American Journal of Pharmacy. 2023;42(3):524-9.

1422. Ahmad S, Alam MS, Alam N, Alqahtani SS, Sultan MH, Banji D, et al. <i>In</i><i> Vitro</i> Bioequivalence Study and Quality Control Assessment of Different Brands of Omeprazole Capsules Available in Jazan, KSA. Latin American Journal of Pharmacy. 2023;42(3):524-9. PubMed PMID: WOS:000955313900009.

1423. Ahmad S, Ali MS, Alam N, Alam MI, Alam MS, Ali MD. Drug interactions of H2receptor antagonists-ranitidine: A Review. Research Journal of Pharmacy and Technology. 2016;9(3):275-80. doi: 10.5958/0974-360X.2016.00051.2.

1424. Ahmad S, Idrees F, editors. Dispense of non-prescribed antibiotics without prescription from pharmacies in Jhelum: a cross-sectional survey. Population Medicine; 2023.

1425. Ahmad T, Khan FU, Ali S, Rahman AU, Khan SA. Assessment of without prescription antibiotic dispensing at community pharmacies in Hazara Division, Pakistan: A simulated client's study. PLoS ONE. 2022;17(2 February). doi: 10.1371/journal.pone.0263756.

1426. Ahmad T, Khan SA, Mallhi TH, Mannan A, Rahman AU, Salman M, et al. Assessing antibiotic dispensing without prescription through simulated client methodology in developing countries: A comprehensive literature review from 2009 to 2021. Journal of Public Health-Heidelberg. 2023. doi: 10.1007/s10389-023-02032-x. PubMed PMID: WOS:001049124100001.

1427. Ahmad W, Ijaz B, Javed FT, Jahan S, Shahid I, Khan FM, et al. HCV genotype distribution and possible transmission risks in Lahore, Pakistan. World Journal of Gastroenterology. 2010;16(34):4321-8. doi: 10.3748/wjg.v16.i34.4321. PubMed PMID: WOS:000281887800010.

1428. Ahmad ZM, Noel NL, Rudd TM, Nadelson CS, Ott MA, Wilkinson TA, et al. Implementation of a pharmacist-led hormonal contraceptive prescribing service in a campus community pharmacy in Indiana, United States. Contraception. 2022;113:113-8. doi: 10.1016/j.contraception.2022.05.004. PubMed PMID: WOS:000900078000020.

1429. Ahmadi M, Samadbeik M, Sadoughi F. Modeling of outpatient prescribing process in iran: a gateway toward electronic prescribing system. Iranian journal of pharmaceutical research: IJPR. 2014;13(2):725.

1430. Ahmadizar F, Soleymani F, Abdollahi M. Study of Drug-Drug Interactions in Prescriptions of General Practitioners and Specialists in Iran 2007-2009. Iranian Journal of Pharmaceutical Research. 2011;10(4):921-31. PubMed PMID: WOS:000299297400031.

1431. Ahmaro L, Lindsey L, Forrest S, Whittlesea C. Investigating community pharmacists' perceptions of delivering chlamydia screening to young people: a qualitative study using normalisation process theory to understand professional practice. International Journal of Pharmacy Practice. doi: 10.1093/ijpp/riac057. PubMed PMID: WOS:000828785900001.

1432. Ahmaro L, Lindsey L, Forrest S, Whittlesea C. Young people's perceptions of accessing a community pharmacy for a chlamydia testing kit: a qualitative study based in North East England. Bmj Open. 2021;11(9). doi: 10.1136/bmjopen-2021-052228. PubMed PMID: WOS:000773615100093.

1433. Ahmed A. Quality and outcomes of heart failure care in older adults: role of multidisciplinary disease-management programs. Journal of the American Geriatrics Society. 2002;50(9):1590-3. doi: 10.1046/j.1532-5415.2002.50418.x. PubMed PMID: CN-01774122.

1434. Ahmed A, Abdulelah Dujaili J, Rehman IU, Chuah LH, Hashmi FK, Awaisu A, et al. Effect of pharmacist care on clinical outcomes among people living with HIV/AIDS: A systematic review and meta-analysis. Research in Social and Administrative Pharmacy. 2022;18(6):2962-80. doi: https://doi.org/10.1016/j.sapharm.2021.07.020.

1435. Ahmed A, Blackburn DF, Evans C, Rosaasen N, Mansell H. The Saskatchewan Medication Assessment Program for Patients With Renal Failure: A Qualitative Study to Understand Health Care Provider Perspectives. Canadian Journal of Kidney Health and Disease. 2020;7. doi: 10.1177/2054358120954028.

1436. Ahmed A, Chaudhry AG, Farooq H. TB stigma, attitude and practices among urban dwellers. A descriptive study on TB. Medical Forum Monthly. 2014;25(7):22-6.

1437. Ahmed A, Dujaili JA, Jabeen M, Umair MM, Chuah LH, Hashmi FK, et al. Barriers and Enablers for Adherence to Antiretroviral Therapy Among People Living With HIV/AIDS in the Era of COVID-19: A Qualitative Study From Pakistan. Frontiers in Pharmacology. 2022;12. doi: 10.3389/fphar.2021.807446. PubMed PMID: WOS:000758926100001.

1438. Ahmed A, Guo P, Jalal Z. A systematic review investigating the role and impact of pharmacist interventions in cardiac rehabilitation. International Journal of Clinical Pharmacy. 2023;45(2):320-9. doi: https://dx.doi.org/10.1007/s11096-022-01517-1. PubMed PMID: 2020152600.

1439. Ahmed A, Mustafa G, Ul Haq N, Nasim A, Tahir M, Riaz S. ASSESSMENT OF PATIENT'S SATISFACTION WITH PHARMACEUTICAL SERVICES AND HEALTHCARE FACILITIES IN TERTIARY CARE HOSPITALS QUETTA, PAKISTAN. Indo American Journal of Pharmaceutical Sciences. 2017;4(11):4435-42. doi: 10.5281/zenodo.1065094. PubMed PMID: WOS:000434353100091.

1440. Ahmed A, Tanveer M, Dujaili J, Hashmi F. CO129 Pharmacist-Led Medication Review to Reduce Antiretroviral Therapy Errors in HIV/AIDS Patients: A Systematic Review. Value in Health. 2022;25(7, Supplement):S328. doi: https://doi.org/10.1016/j.jval.2022.04.224.

1441. Ahmed A, Tanveer M, Dujaili JA, Chuah LH, Hashmi FK, Awaisu A. Pharmacist-Involved Antiretroviral Stewardship Programs in People Living with HIV/AIDS: A Systematic Review. AIDS Patient Care & STDs. 2023;37(1):31-52. doi: 10.1089/apc.2022.0192. PubMed PMID: 161230981.

1442. Ahmed ASI, Dong K, Liu J, Wen T, Yu L, Xu F, et al. Long noncoding RNA NEAT1 (nuclear paraspeckle assembly transcript 1) is critical for phenotypic switching of vascular smooth muscle cells. Proceedings of the National Academy of Sciences. 2018;115(37):E8660-E7.

1443. Ahmed B, Nanji K, Mujeeb R, Patel MJ. Effects of Polypharmacy on Adverse Drug Reactions among

Geriatric Outpatients at a Tertiary Care Hospital in Karachi: A Prospective Cohort Study. Plos One. 2014;9(11). doi: 10.1371/journal.pone.0112133. PubMed PMID: WOS:000345158700029.

1444. Ahmed Casper E, Mohmed El Wakeel L, Ayman Saleh M, Hamed El-Hamamsy M. The impact of a comprehensive pharmaceutical care intervention in addition to cardiac rehabilitation program on outcomes of post-acute coronary syndrome patients: a pilot study. Patient education and counseling. 2022;105(10):3164-8. doi: 10.1016/j.pec.2022.06.004. PubMed PMID: CN-02415074.

1445. Ahmed H, Neuzil P, Skoda J, Petru J, Sediva L, Schejbalova M, et al. Renal sympathetic denervation using an irrigated radiofrequency ablation catheter for the management of drug-resistant hypertension. JACC: Cardiovascular Interventions. 2012;5(7):758-65.

1446. Ahmed I, King R, Akter S, Akter R, Aggarwal VR. Determinants of antibiotic self-medication: A systematic review and meta-analysis. Research in Social and Administrative Pharmacy. 2023;19(7):1007-17. doi: https://doi.org/10.1016/j.sapharm.2023.03.009.

1447. Ahmed K, Crook J, Tennant A, North-Lewis P, Abbasi T, Lloyd C, et al. TREATING CHILDREN WITH HCV CLOSE TO HOME THROUGH A VIRTUAL NATIONAL MULTIDISCIPLINARY NETWORK. Archives of Disease in Childhood. 2023;108(5):3-4. doi: https://dx.doi.org/10.1136/archdischild-2023-NPPG.5. PubMed PMID: 641345692.

1448. Ahmed MM. Evaluation of knowledge about antibiotics prescription during periodontal therapy among dental students: An observational study. Journal of Pharmacy and Bioallied Sciences. 2022;14(5):S829-S32. doi: 10.4103/jpbs.jpbs-11-22.

1449. Ahmed MM, Li P, Meece LE, Bian J, Shao H. A Varied Approach to LVAD Follow-Up Improves Cost-Effectiveness. The Journal of Heart and Lung Transplantation. 2021;40(4, Supplement):S94. doi: https://doi.org/10.1016/j.healun.2021.01.315.

1450. Ahmed NJ. The Pattern of Prescribing Diclofenac Gel in the Orthopedic Outpatient Clinic at a Tertiary Hospital. Latin American Journal of Pharmacy. 2023;42(1):86-9. PubMed PMID: WOS:000920231700013.

1451. Ahmed NJ, Khan MF. Frequency of Outpatient Inhalers Prescriptions in Riyadh. Journal of Pharmaceutical Research International. 2019;31(6). doi: 10.9734/JPRI/2019/v31i630345. PubMed PMID: WOS:000512909900020.

1452. Ahmed NJ, Khan MF. Community Pharmacists' Knowledge, Attitudes and Practices towards the Use of Herbal Remedies. Journal of Pharmaceutical Research International. 2019;31(6). doi: 10.9734/JPRI/2019/v31i630316. PubMed PMID: WOS:000497734600001.

1453. Ahmed NJ, Menshawy MA, Almalki ZS. Age-Related Prescribing Patterns of Antihypertensive Medications. Journal of Pharmaceutical Research International. 2020;32(30):80-5. doi: 10.9734/JPRI/2020/v32i3030908. PubMed PMID: WOS:000593071800012.

1454. Ahmed NO, Osman B, Abdelhai YM, El-Hadiyah TMH. Impact of clinical pharmacist intervention in anticoagulation clinic in Sudan. International journal of clinical pharmacy. 2017:1-5. doi: 10.1007/s11096-017-0475-x. PubMed PMID: CN-01373588.

1455. Ahmed R, Drouin M, Martin E, Mirro M, Ghahari RR, Toscos T. Perceptions on Tailored Educational Messaging to Patients with Atrial Fibrillation: towards Increased Patient Engagement and Medication Adherence. Heart & lung. 2020;49(2):216-. doi: 10.1016/j.hrtlng.2020.02.023. PubMed PMID: CN-02096229.

1456. Ahmed RR, Streimikiene D, Soomro RH, Streimikis J. Digital Transformation and Industry 4.0 Initiatives for Market Competitiveness: Business Integration Management Model in the Healthcare Industry. Journal of Competitiveness. 2022;14(4):6-24. doi: 10.7441/joc.2022.04.01. PubMed PMID: WOS:000908340400002.

1457. Ahmed S, Abd Manaf NH, Islam R. Measuring quality performance between public and private hospitals in Malaysia. International journal of quality and service sciences. 2017.

1458. Ahmed S, Abd Manaf NH, Islam R. Effects of Six Sigma initiatives in Malaysian private hospitals. International Journal of Lean Six Sigma. 2019;10(1):44-57.

1459. Ahmed SM, Hossain MA, Chowdhury MR. Informal sector providers in Bangladesh: how equipped are they to provide rational health care? Health Policy and Planning. 2009;24(6):467-78. doi: 10.1093/heapol/czp037. PubMed PMID: WOS:000271107100007.

1460. Ahmed SM, Hossain MA, RajaChowdhury AM, Bhuiya AU. The health workforce crisis in Bangladesh: shortage, inappropriate skill-mix and inequitable distribution. Human Resources for Health. 2011;9. doi: 10.1186/1478-4491-9-3. PubMed PMID: WOS:000287214400001.

1461. Ahmed SM, Naher N, Hossain T, Rawal LB. Exploring the status of retail private drug shops in Bangladesh and action points for developing an accredited drug shop model: A facility based cross-sectional study. Journal of Pharmaceutical Policy and Practice. 2017;10(1). doi: 10.1186/s40545-017-0108-8.

1462. Ahmed T, Haq N, Rehman M, Nasim A. PHS150 - The Impact of Pharmacist Intervention on Medication Errors In A Teaching Hospital Quetta, Pakistan. Value in Health. 2016;19(7):A630. doi: https://doi.org/10.1016/j.jval.2016.09.1632.

1463. Ahmed T, Iqbal Q, Ahmed S, Minhas M, Mehmood S. PHP34 - Prescription Writing Errors Detection by Pharmacist in A Pediatric Ward. Value in Health. 2017;20(9):A907. doi: https://doi.org/10.1016/j.jval.2017.08.2793.

1464. Ahmed Z, Ghoto MA, Mughal UUR, Dayo A, Arain MI, Metlo M, et al. Assessment of ADRs and patient's knowledge at government civil hospital Khairpur, Pakistan. Latin American Journal of Pharmacy. 2018;37(4):693-8.

1465. Ahmedani BK, Peterson EL, Wells KE, Rand CS, Williams LK. Asthma medication adherence: the role of God and other health locus of control factors. Annals of allergy, asthma & immunology. 2013;110(2):75-9.e2. doi: 10.1016/j.anai.2012.11.006. PubMed PMID: CN-00853587.

1466. Ahn H, Neumann L, Vazquez Novoa N. Measuring the relative balance of DMUs. European Journal of Operational Research. 2012;221(2):417-23. doi: https://doi.org/10.1016/j.ejor.2012.03.030.

1467. Ahn J, Kim G, Sun Suh H, Moo Lee S. Social values and healthcare priority setting in Korea. Journal of health organization and management. 2012;26(3):343-50.

1468. Ahn S, Park JS, Kim H, Heo M, Sung YC, Jeun SS. Compassionate use of recombinant human IL-7-hyFc as a salvage treatment for restoring lymphopenia in patients with recurrent glioblastoma. Cancer Medicine. 2023;12(6):6778-87. doi: 10.1002/cam4.5467. PubMed PMID: 162842220.

1469. Aho TF, Gilling PJ, Kennett KM, Westenberg AM, Fraundorfer MR, Frampton CM. Holmium laser bladder neck incision versus holmium enucleation of the prostate as outpatient procedures for prostates less than 40 grams: A randomized trial. Journal of Urology. 2005;174(1):210-4. doi: 10.1097/01.ju.0000161610.68204.ee. PubMed PMID: WOS:000229946400054.

1470. Ahorlu CK, Dunyo SK, Afari EA, Koram KA, Nkrumah FK. Malaria-related beliefs and behaviour in southern Ghana: Implications for treatment, prevention and control. Tropical Medicine & International Health. 1997;2(5):488-98. doi: 10.1046/j.1365-3156.1997.d01-298.x. PubMed PMID: WOS:A1997XE38700012.

1471. Ahrens RA, Hower M, Best AM. Effects of weight reduction interventions by community pharmacists. Journal of the American Pharmacists Association : JAPhA. 2003;43(5):583-9. doi: 10.1331/154434503322452210. PubMed PMID: CN-00459014.

1472. Ahrens RA, Hower M, Best AM. Effects of weight reduction interventions by community pharmacists. Journal of the American Pharmacists Association. 432003. p. 583-9.

1473. Ahuja S, Scypinski S. Handbook of modern pharmaceutical analysis: Academic press; 2001.

1474. Ahuja T, Merchan C, Arnouk S, Cirrone F, Dabestani A, Papadopoulos J. COVID-19 pandemic preparedness: A practical guide from clinical pharmacists' perspective. American Journal of Health-System Pharmacy. 2020;77(18):1510-5. PubMed PMID: 34279575.

1475. Ahumada-Canale A, Quirland C, Martinez-Mardones FJ, Plaza-Plaza JC, Benrimoj S, Garcia-Cardenas V. Economic evaluations of pharmacist-led medication review in outpatients with hypertension, type 2 diabetes mellitus, and dyslipidaemia: a systematic review. European Journal of Health Economics. 2019;20(7):1103-16. PubMed PMID: 31218580.

1476. Ahumada-Canale A, Vargas C, Martinez-Mardones F, Plaza JCP, Benrimoj S, Garcia-Cardenas V. Cost-utility analysis of medication review with follow-up for cardiovascular outcomes: A microsimulation model. Health Policy. 2021;125(11):1406-14. doi: 10.1016/j.healthpol.2021.09.004. PubMed PMID: WOS:000711028900002.

1477. Ahumada-Canale A, Vargas C, Martinez-Mardones F, Plaza-Plaza JC, Benrimoj S, Garcia-Cardenas V. Cost-utility analysis of medication review with follow-up for cardiovascular outcomes: a microsimulation model. Health policy (Amsterdam, Netherlands). 2021;125(11):1406-14. doi: 10.1016/j.healthpol.2021.09.004. PubMed PMID: CN-02365912.

1478. Ai AL, Carretta H, Beitsch LM, Watson L, Munn J, Mehriary S. Medication Therapy Management Programs: Promises and Pitfalls. Journal of Managed Care & Specialty Pharmacy. 2014;20(12):1162-82. doi: 10.18553/jmcp.2014.20.12.1162. PubMed PMID: WOS:000345802300002.

1479. Ai Y, Zhang F, Wang C, Xie R, Liang Q. Recent progress in lab-on-a-chip for pharmaceutical analysis and pharmacological/toxicological test. TrAC Trends in Analytical Chemistry. 2019;117:215-30. doi: https://doi.org/10.1016/j.trac.2019.06.026.

1480. Aiassa V, Bustos Fierro C, Gavelli ME, Toso GB, Bosio BE, Pirazzini MD, et al. Injectable antimicrobial use in the National Clinical Hospital of Córdoba (Argentina). Latin American Journal of Pharmacy. 2011;30(9):1682-8.

1481. Aicken CRH. Remote self-testing for sexually transmitted infections, within online care pathways: how could this intervention deliver public health benefit?: formative research using chlamydia as an exemplar [Ph.D.]. England: University of London, University College London (United Kingdom); 2018.

1482. Aicken CRH, Sutcliffe LJ, Gibbs J, Tickle LJ, Hone K, Harding-Esch EM, et al. Using the eSexual Health Clinic to access chlamydia treatment and care via the internet: a qualitative interview study. Sexually transmitted infections. 2018;94(4):241-7. doi: 10.1136/sextrans-2017-053227.

1483. Aierken G, Mutalifu Z, Dong Z, Shen M, Re Z. Curative Effects and Safety of Low Dose Paclitaxel Combined with Carboplatin on Patients with Advanced Cervical Cancer. Anti-tumor pharmacy. 2017;7(4):446-50. doi: 10.3969/j.issn.2095-1264.2017.04.13. PubMed PMID: CN-01471565.

1484. Aigbavboa SO. Pharmaceutical Supply Chains in Nigeria: A Framework for Outsourcing Outbound Value Chains [D.Phil.]. South Africa: University of Johannesburg (South Africa); 2019.

1485. Ailabouni N, Mangin D, Nishtala PS. Deprescribing anticholinergic and sedative medicines: protocol for a Feasibility Trial (DEFEAT-polypharmacy) in residential aged care facilities. BMJ Open. 2017;7(4):e013800. PubMed PMID: 28416498.

1486. Ailabouni NJ, Marcum ZA, Schmader KE, Gray SL. Medication Use Quality and Safety in Older Adults: 2018 Update. Journal of the American Geriatrics Society. 2019;67(12):2458-62. doi: 10.1111/jgs.16243. PubMed PMID: 140248967. Language: English. Entry Date: 20191212. Revision Date: 20201130. Publication Type: Article. Journal Subset: Biomedical.

1487. Ailawadhi S, Medhekar R, Princic N, Fowler R, Tran O, Bhowmik D, et al. Healthcare resource utilization and costs in patients with multiple myeloma with and without skeletal-related events. Journal of Oncology Pharmacy Practice. 2020;26(5):1070-9. doi: 10.1177/1078155219881489. PubMed PMID: WOS:000494132900001.

1488. Ailoje KO. Home Management of Uncomplicated Childhood Malaria by Mothers and Fathers in Lagos State, Nigeria [Ph.D.]. Nigeria: University of Lagos (Nigeria); 2014.

1489. Aimaurai S, Jumpated A, Krass I, Dhippayom T. Patient opinions on medicine-use review: Exploring an expanding role of community pharmacists. Patient Preference and Adherence. 2017;11:751-60. doi: 10.2147/PPA.S132054.

1490. Aimono Y, Kohyama N, Kamoshida T, Yamamoto T, Kogo M. Factors associated with a reduction in the quality of life of patients with chronic hepatitis C treated by ledipasvir/sofosbuvir therapy. European Journal of Hospital Pharmacy. 2021;28(E1):E29-E34. doi: 10.1136/ejhpharm-2020-002215. PubMed PMID: WOS:000726955800007.

1491. Ain KB, Pucino F, Csako G, Wesley RA, Drass JA, Clark C, et al. Effects of restricting levothyroxine dosage strength availability. Pharmacotherapy. 1996;16(6):1103-10. PubMed PMID: CN-00134429.

1492. Ainan S, Mfinanga S, Kivuyo S, Okebe J, Garrib A, Birungi J, et al. Integrating HIV, diabetes, and hypertension services in Africa: a cluster-randomised trial in Tanzania and Uganda. Tanzania journal of health research. 2022;23:34-5. doi: 10.4314/thrb.v23i1.1S. PubMed PMID: CN-02495076.

1493. Airaksinen M, Ahonen R, Enlund H. Customer feedback as a tool for improving pharmacy services. International Journal of Pharmacy Practice. 1995;3(4):219-26.

1494. Airaksinen M, Ahonen R, Enlund H. The "Questions to Ask about Your Medicines" Campaign: An Evaluation of Pharmacists and the Public's Response. Medical Care. 1998;36(3):422-7. doi: 10.1097/00005650-199803000-00017.

1495. Airaksinen M, Ahonen R, Enlund H. The "Questions to Ask About Your Medicines" campaign - An evaluation of pharmacists and the public's response. Medical Care. 1998;36(3):422-7. doi: 10.1097/00005650-199803000-00017. PubMed PMID: WOS:000072467300017.

1496. Airaksinen M, Toivo T, Jokinen L, Savela E, Parkkamaki S, Sandler C, et al. Policy and vision for community pharmacies in Finland: A roadmap towards enhanced integration and reduced costs. Pharmacy

Practice. 2021;19(1):2288. PubMed PMID: 33628348.

1497. Airaksinen MSA. Customer feedback as a tool for improving pharmacy services in Finland [Ph.D.]. Finland: Kuopion Yliopisto (Finland); 1997.

1498. Airee A, Guirguis AB, Mohammad RA. Clinical outcomes and pharmacists' acceptance of a community hospital's anticoagulation management service utilizing decentralized clinical staff pharmacists. Annals of Pharmacotherapy. 2009;43(4):621-8. doi: 10.1345/aph.1L460.

1499. Airiau C, Desbuquois AC, Blancher CH, Huon JF, Marson C, Ruspini E, et al. Du développement de la pharmacie clinique en chirurgie. Le Pharmacien Hospitalier et Clinicien. 2021;56(4):441-55. doi: https://doi.org/10.1016/j.phclin.2021.07.002.

1500. Aissa-Bokhtache A, Zegaoui A, Belmadani B, Bouchrit MS. Water Purification by a Lamp Discharge-Electronic Ballast System Using a Full Bridge Inverter. Energy Procedia. 2015;74:446-52. doi: https://doi.org/10.1016/j.egypro.2015.07.650.

1501. Aisuodionoe-Shadrach O, Kolade-Yunusa HO, Sadiq A. Ultrasound derived-parameters and symptom severity scores as noninvasive predictors of bladder outlet obstruction in patients with benign prostatic enlargement. West African Journal of Radiology. 2020;27(2):95-9. doi: 10.4103/wajr.wajr_25_19. PubMed PMID: WOS:000656717000004.

1502. Aitken LM, Bucknall T, Kent B, Mitchell M, Burmeister E, Keogh SJ. Protocol-directed sedation versus non-protocol-directed sedation in mechanically ventilated intensive care adults and children. Cochrane Database of Systematic Reviews. 2018;(11). doi: 10.1002/14651858.CD009771.pub3. PubMed PMID: CD009771.

1503. Aitken M, Gorokhovich L. Advancing the responsible use of medicines: applying levers for change. Available at SSRN 2222541. 2012.

1504. Aitken R, Manias E, Dunning T. Documentation of medication management by graduate nurses in patient progress notes: A way forward for patient safety. Collegian. 2006;13(4):5-11. doi: https://doi.org/10.1016/S1322-7696(08)60533-8.

1505. Aiudi CM, Oliver JJ, Chowatia PA, Dalia AA. Code Blue in the Operating Room—pharmacy is on the way! Journal of the American Pharmacists Association. 2021;61(5):e113-e8. doi: https://doi.org/10.1016/j.japh.2021.03.023.

1506. Aivalli PK, Elias MA, Pati MK, Bhanuprakash S, Munegowda C, Shroff ZC, et al. Perceptions of the quality of generic medicines: Implications for trust in public services within the local health system in Tumkur, India. BMJ Global Health. 2017;2. doi: 10.1136/bmjgh-2017-000644.

1507. Aizpurua-Arruti X, Malet-Larrea A, Onatibia-Astibia A, Del Pozo-Rodriguez A, Goyenechea E, Gastelurrutia MA. Home pharmaceutical care for frailty patients in Gipuzkoa. Farmaceuticos Comunitarios. 2021;13(3):17-24. doi: 10.33620/FC.2173-9218.(2021/Vol13).003.03. PubMed PMID: WOS:000669949300003.

1508. Ajala TO, Adebona AC, Bamiro OA. The pharmaceutical quality of brands of metformin tablets in Ogun-state, Nigeria. African Journal of Biomedical Research. 2014;17(1):43-8.

1509. Aje AA, Showande SJ, Adisa R, Fakeye TO, Olutayo OA, Adebusoye LA, et al. Effect of educational intervention on medication reconciliation practice of hospital pharmacists in a developing country - A non-randomised controlled trial. BMC medical education. 2023;23(1):867. doi: https://dx.doi.org/10.1186/

s12909-023-04844-7. PubMed PMID: 642758553.

1510. Ajiboye WT. Medication Counselling Practice in Community Pharmacies in Lagos State, Nigeria [Ph.D.]. Nigeria: University of Lagos (Nigeria); 2013.

1511. Ajie AAD, Andrajati R, Radji M. Factors affecting the sale of non-prescribed antibiotics in Jakarta, Indonesia: A cross-sectional study. International Journal of Applied Pharmaceutics. 2018;10(Special Issue 1):243-7. doi: 10.22159/ijap.2018.v10s1.54.

1512. Ajingdit DT, Karimi PN, Carasso KB, Niragire F. Cross-sectional study on the availability of essential medicines at public health facilities in Jur River County, South Sudan. South Sudan Medical Journal. 2023;16(2):45-9. doi: 10.4314/ssmj.v16i2.2.

1513. Ajjan RA, Hensor EMA, Del Galdo F, Shams K, Abbas A, Fairclough RJ, et al. Oral 11β-HSD1 inhibitor AZD4017 improves wound healing and skin integrity in adults with type 2 diabetes mellitus: a pilot randomized controlled trial. European journal of endocrinology. 2022;186(4):441-55. doi: 10.1530/EJE-21-1197. PubMed PMID: CN-02363329.

1514. Ajumobi OO. Physician-Initiated Buprenorphine Treatment for Opioid Use Disorder in Nevada Emergency Departments2023.

1515. Ajuwon AJ, Oshiname FO, Imaledo J, Ajayi O, Dipeolu IO. Delivery and utilisation of injectable contraceptive services in rural nigeria: Learning from the perspectives of patent medicine vendors and women of reproductive age. African Journal of Biomedical Research. 2016;19(2):79-87.

1516. Akaho E, MacLaughlin EJ, Takeuchi Y. Comparison of Prescription Reimbursement Methodologies in Japan and the United States. Journal of the American Pharmacists Association. 2003;43(4):519-26. doi: https://doi.org/10.1331/154434503322226275.

1517. Akalgan Aklar D, Ozcelikay G. Patient Experience in Community Pharmacies from an Experiential Marketing Perspective: Structural Equation Model. Journal of Research in Pharmacy. 2023;27(3):980-94. doi: 10.29228/jrp.391.

1518. Akande RO, Abodunrin OL, Olarewaju SO, Adeomi AA, Akande JO, Faramade IO. Health insurance in private and public health facilities in Southwestern Nigeria: what determines clients´ satisfaction with quality of service? Pan African Medical Journal. 2022;41. doi: 10.11604/pamj.2022.41.268.26875.

1519. Akande RO, Abodunrin OL, Olarewaju SO, Adeomi AA, Akande JO, Faramade IO. Health insurance in private and public health facilities in Southwestern Nigeria: what determines clients' satisfaction with quality of service? Pan African Medical Journal. 2022;41. doi: 10.11604/pamj.2022.41.268.26875. PubMed PMID: WOS:000813086700005.

1520. Akande-Sholabi W, Adebusoye LA, Olowookere OO. Potentially inappropriate medication use among older patients attending a geriatric centre in south-west Nigeria. Pharmacy Practice (1886-3655). 2018;16(3):1-7. doi: 10.18549/PharmPract.2018.03.1235. PubMed PMID: 132680592. Language: English. Entry Date: 20181101. Revision Date: 20181102. Publication Type: Article.

1521. Akande-Sholabi W, Ajamu AT. Antimicrobial stewardship: Assessment of knowledge, awareness of antimicrobial resistance and appropriate antibiotic use among healthcare students in a Nigerian University. BMC Medical Education. 2021;21(1). doi: 10.1186/s12909-021-02912-4.

1522. Akande-Sholabi W, Ajamu AT, Adisa R. Prevalence, knowledge and perception of self-medication practice among undergraduate healthcare students. Journal of Pharmaceutical Policy and Practice.

2021;14(1). doi: 10.1186/s40545-021-00331-w.

1523. Akande-Sholabi W, Akinyemi OO. Self-medication with over-the-counter drugs among consumers: A cross-sectional survey in a Southwestern State in Nigeria. BMJ Open. 2023;13(5). doi: 10.1136/bmjopen-2023-072059.

1524. Akande-Sholabi W, Bakare OE. Community Pharmacists' Perception about Mental Healthcare and Barriers to providing Pharmaceutical Care Services to Patients with Mental Disorder in Ibadan, Nigeria. African Journal of Biomedical Research. 2023;26(3):355-62. doi: 10.4314/ajbr.v26i3.8.

1525. Akande-Sholabi W, Ilesanmi OS, Ayanleke AS. Treatment of malaria in pregnancy: Knowledge of community pharmacists in Ibadan, Nigeria. Tropical Journal of Pharmaceutical Research. 2019;18(12):2679-86. doi: 10.4314/tjpr.v18i12.28.

1526. Akande-Sholabi W, Iluyomade A, Ilesanmi OS, Adisa R. Perception and use of herbal medicines among clients visiting selected community pharmacies in Ibadan, Nigeria. African Journal of Biomedical Research. 2020;23(2):147-53.

1527. Akande-Sholabi W, Oyesiji E. Antimicrobial stewardship: knowledge, perceptions, and factors associated with antibiotics misuse among consumer's visiting the community pharmacies in a Nigeria Southwestern State. Journal of Pharmaceutical Policy and Practice. 2023;16(1). doi: 10.1186/s40545-023-00629-x.

1528. Akash SR, Arnob MAJB, Uddin MJ. FDA Modernization Act 2.0: An insight from nondeveloping country. Drug Development Research. 2023;84(8):1572-7. doi: 10.1002/ddr.22108. PubMed PMID: 174272519.

1529. Akazawa M. Economic evaluation of treating chronic obstructive pulmonary disease: The effects of early initiation of inhaled corticosteroids on exacerbation risks, utilization and costs [Ph.D.]. United States -- North Carolina: The University of North Carolina at Chapel Hill; 2007.

1530. Akazawa M. Clinical Research in Communities and the Importance of Patient Information. Yakugaku Zasshi. 2022;142(3):215-8. doi: 10.1248/YAKUSHI.21-00174-3.

1531. Akazawa M, Biddle AK, Stearns SC. Economic assessment of early initiation of inhaled corticosteroids in chronic obstructive pulmonary disease using propensity score matching. Clinical Therapeutics. 2008;30:1003-16. doi: https://doi.org/10.1016/j.clinthera.2008.05.020.

1532. Akazawa M, Fukuoka K. Economic impact of switching to fixed-dose combination therapy for Japanese hypertensive patients: a retrospective cost analysis. Bmc Health Services Research. 2013;13. doi: 10.1186/1472-6963-13-124. PubMed PMID: WOS:000317459400001.

1533. Akazawa M, Imai H, Igarashi A, Tsutani K. Potentially inappropriate medication use in elderly Japanese patients. American Journal of Geriatric Pharmacotherapy. 2010;8(2):146-60. doi: 10.1016/j.amjopharm.2010.03.005. PubMed PMID: 105192171. Language: English. Entry Date: 20100806. Revision Date: 20200708. Publication Type: Journal Article.

1534. Akazawa M, Mikami A, Tamura Y, Yanagi N, Yamamura S, Ogata H. Establishing a Pharmacy-Based Patient Registry System: A Pilot Study for Evaluating Pharmacist Intervention for Patients with Long-Term Medication Use. Pharmacy. 2018;6(1). doi: 10.3390/pharmacy6010012. PubMed PMID: WOS:000428562300011.

1535. Akazawa M, Nomura K, Kusama M, Igarashi A. Drug Utilization Reviews by Community Pharmacists

in Japan: Identification of Potential Safety Concerns through the Brown Bag Program. Value in Health Regional Issues. 2012;1(1):98-104. doi: https://doi.org/10.1016/j.vhri.2012.03.001.

1536. Akbar FH, Maretta YA. The influence of quality of dental health services on trust in West Sulawesi Province, Indonesia. International Journal of Dentistry and Oral Science. 2020;7(7):770-5.

1537. Akbar S. Road less traveled. Journal of the American Pharmacists Association. 2022;62(6):1723-4. doi: 10.1016/j.japh.2022.08.008.

1538. Akbar Z, Saeed H, Saleem Z, Andleeb S. Dosing errors in total parenteral nutrition prescriptions at a specialized cancer care hospital of Lahore: The role of clinical pharmacist. Journal of Oncology Pharmacy Practice. 2021;27(3):531-40. doi: 10.1177/1078155220923014. PubMed PMID: 149555168. Language: English. Entry Date: 20210407. Revision Date: 20210423. Publication Type: Article.

1539. Akbarpour M, Ali Torabi S, Ghavamifar A. Designing an integrated pharmaceutical relief chain network under demand uncertainty. Transportation Research Part E: Logistics and Transportation Review. 2020;136:101867. doi: https://doi.org/10.1016/j.tre.2020.101867.

1540. Akca D. Operationalizing the Spatial Influence of the Risk Factors behind the Open-air Drug Markets in Durham Region [M.A.]. Canada -- Ontario, CA: University of Ontario Institute of Technology (Canada); 2015.

1541. Akers J, Seignemartin B, Anderson J, Richardson B. New interprofessional health sciences precepting legislation provides opportunity to assess student and preceptor knowledge, experience, and confidence. Journal of Interprofessional Education & Practice. 2022;27:100497. doi: https://doi.org/10.1016/j.xjep.2022.100497.

1542. Akers JL, Hansen RN, Oftebro RD. Implementing take-home naloxone in an urban community pharmacy. Journal of the American Pharmacists Association. 2017;57(2, Supplement):S161-S7. doi: https://doi.org/10.1016/j.japh.2017.01.006.

1543. Akers JL, Meer G, Kintner J, Shields A, Dillon-Sumner L, Bacci JL. Implementing a pharmacist-led in-home medication coaching service via community-based partnerships. Journal of the American Pharmacists Association. 2019;59(2):243-51. doi: https://doi.org/10.1016/j.japh.2018.11.008.

1544. Akesson C, Paulos C, Marín F, Salazar C, Del Campo CH. A description of patients presenting with minor illness in community pharmacies in southern Chile. International Journal of Pharmacy Practice. 2007;15(2):123-8. doi: 10.1211/ijpp.15.2.0007.

1545. Akhondzadeh S, Noroozian M, Mohammadi M, Ohadinia S, Jamshidi A, Khani M. Salvia officinalis extract in the treatment of patients with mild to moderate Alzheimer's disease: a double blind, randomized and placebo-controlled trial. Journal of clinical pharmacy and therapeutics. 2003;28(1):53-9.

1546. Akhtar S, Rutter P. Pharmacists thought processes in making a differential diagnosis using a gastro-intestinal case vignette. Research in Social and Administrative Pharmacy. 2015;11(3):472-9. doi: https://doi.org/10.1016/j.sapharm.2014.09.003.

1547. Akhtar T, Fratti JD, Mattumpuram J, Fugar S, Uprety A, Nwaichi C, et al. Factors associated with bleeding events in patients on rivaroxaban for non-valvular atrial fibrillation: A real-world experience. International Journal of Cardiology. 2020;320:78-82. doi: 10.1016/j.ijcard.2020.06.032. PubMed PMID: WOS:000580594200016.

1548. Akhtar T, Fratti JDC, Mattumpuram J, Fugar S, Uprety A, Nwaichi C, et al. Factors associated with

bleeding events in patients on rivaroxaban for non-valvular atrial fibrillation: A real-world experience. International Journal of Cardiology. 2020;320:78-82. doi: https://doi.org/10.1016/j.ijcard.2020.06.032.

1549. Akici A, Alp FI, Dülger GA, Elçioğlu K, Gümüşel B, Gündüz Ö, et al. Problems pharmacists face in the community pharmacies during the dispensing process - A perspective through the rational drug use. Hacettepe University Journal of the Faculty of Pharmacy. 2009;29(1):75-80.

1550. Akinbosoye OE, Taitel MS, Grana J, Hill J, Wade RL. Improving medication adherence and health care outcomes in a commercial population through a community pharmacy. Population health management. 2016;19(6):454-61.

1551. Akinboyo IC, Gerber JS. Principles, policy and practice of antibiotic stewardship. Seminars in Perinatology. 2020;44(8):151324. doi: https://doi.org/10.1016/j.semperi.2020.151324.

1552. Akincigil A, Bowblis JR, Levin C, Walkup JT, Jan S, Crystal S. Adherence to antidepressant treatment among privately insured patients diagnosed with depression. Medical Care. 2007;45(4):363-9. doi: 10.1097/01.mlr.0000254574.23418.f6.

1553. Akincigil A, Wilson IB, Walkup JT, Siegel MJ, Huang C, Crystal S. Antidepressant Treatment and Adherence to Antiretroviral Medications Among Privately Insured Persons with HIV/AIDS. Aids and Behavior. 2011;15(8):1819-28. doi: 10.1007/s10461-011-9938-6. PubMed PMID: WOS:000295735200023.

1554. Akindolire MA, Babalola OO, Ateba CN. Detection of Antibiotic Resistant Staphylococcus aureus from Milk: A Public Health Implication. International Journal of Environmental Research and Public Health. 2015;12(9):10254-75. doi: 10.3390/ijerph120910254. PubMed PMID: WOS:000361889000002.

1555. Akins RB, Tolson H, Cole BR. Stability of response characteristics of a Delphi panel: application of bootstrap data expansion. BMC medical research methodology. 2005;5(1):1-12.

1556. Akinsola OO. Evaluation of Antimalarial Chemotherapeutic Practices and Outcomes in the Southwest Region of Nigeria [Ph.D.]. England: University of Salford (United Kingdom); 2018.

1557. Akinsolu FT, Abodunrin OR, Olagunju MT, Adewole IE, Rahman NO, Dabar AM, et al. Community perception of school-based mass drug administration program for soil-transmitted helminths and Schistosomiasis in Ogun State, Nigeria. PLOS Neglected Tropical Diseases. 2023;17(7):e0011213.

1558. Akinwale TP, Adams AJ, Dering-Anderson AM, Klepser ME. Pharmacy-based point-of-care testing for infectious diseases: Considerations for the pharmacy curriculum. Currents in Pharmacy Teaching and Learning. 2015;7(1):131-6. doi: 10.1016/j.cptl.2014.09.005. PubMed PMID: WOS:000409724800019.

1559. Akinyemi AA. Precipitating Circumstances of Male Legal Intervention Deaths by Race/ethnicity, 17 NVDRS States, 2012–20172023.

1560. Akkad ZA, Alomar M, Palaian S. An exploratory survey on community pharmacists' service provision for pregnant and lactating women in Sharjah, United Arab Emirates. PLoS ONE. 2022;17(2 February). doi: 10.1371/journal.pone.0262254.

1561. Akkahadsee P, Sawangjit R, Phumart P, Chaiyakunapruk N, Sakloetsakun D. Systematic review and network meta-analysis of efficacy and safety of interventions for preventing anti-tuberculosis drug induced liver injury. Scientific Reports. 2023;13(1):19880.

1562. Akkawi ME. Pharmacist-led home medication review for older adults: A stepwise process and an algorithm for face-to-face or online approach. Research in social & administrative pharmacy : RSAP.

2023;19(1):1-4. doi: https://dx.doi.org/10.1016/j.sapharm.2022.09.012. PubMed PMID: 639447140.

1563. Akkawi ME. Pharmacist-led home medication review for older adults: A stepwise process and an algorithm for face-to-face or online approach. Research In Social & Administrative Pharmacy. 2023;19(1):1-4. PubMed PMID: 36333229.

1564. Akkoc N, Direskeneli H, Erdem H, Gul A, Kabasakal Y, Kiraz S, et al. Direct and indirect costs associated with ankylosing spondylitis and related disease activity scores in Turkey. Rheumatology International. 2015;35(9):1473-8. doi: 10.1007/s00296-015-3236-y. PubMed PMID: WOS:000359834200003.

1565. Aklar DA, Ozcelikay G. Patient Experience in Community Pharmacies from an Experiential Marketing Perspective: Structural Equation Model. Journal of Research in Pharmacy. 2023;27(3):980-94. doi: 10.29228/jrp.391. PubMed PMID: WOS:001011434600002.

1566. Aklilu AM, O'Connor KD, Wilson FP. Personalized Recommendations for AKI Using a Kidney Action Team (KAT-AKI): design, Rationale, and Early Data. Journal of the American Society of Nephrology : JASN. 2022;33:342-3. doi: 10.1681/asn.2021040439. PubMed PMID: CN-02500306.

1567. Akobundu UO. Short-term home-delivered meal intervention and the health, nutrition and functional status of hospital-discharged older adults [Ph.D.]. United States -- Maryland: University of Maryland, College Park; 2008.

1568. Akol A, Chin-Quee D, Wamala-Mucheri P, Namwebya JH, Mercer SJ, Stanback J. Getting closer to people: Family planning provision by drug shops in Uganda. Global Health Science and Practice. 2014;2(4):472-81. doi: 10.9745/GHSP-D-14-00085.

1569. Akoria OA, Arhuidese IJ. Progress toward elimination of malaria in Nigeria: Uptake of artemisinin-based combination therapies for the treatment of malaria in households in Benin City. Annals of African Medicine. 2014;13(3):104-13. doi: 10.4103/1596-3519.134383.

1570. Akour A, Bardaweel S, Awwad O, Al-Muhaissen S, Hussein R. Impact of a pharmacist-provided information booklet on knowledge and attitudes towards oral contraception among Jordanian women: an interventional study. European journal of contraception & reproductive health care. 2017;22(6):459-64. doi: 10.1080/13625187.2017.1412425. PubMed PMID: CN-01461524.

1571. Akour A, Elayeh E, Tubeileh R, Hammad A, Ya'Acoub R, Al-Tammemi AB. Role of community pharmacists in medication management during COVID-19 lockdown. Pathogens and Global Health. 2021;115(3):168-77. PubMed PMID: 33573528.

1572. Akour A, Elayeh E, Tubeileh R, Hammad A, Ya’Acoub R, Al-Tammemi AB. Role of community pharmacists in medication management during COVID-19 lockdown. Pathogens and Global Health. 2021;115(3):168-77. doi: 10.1080/20477724.2021.1884806.

1573. Akour A, Halloush S, Nusair MB, Barakat M, Abdulla F, Al Momani M. Gaps in pharmaceutical care for patients with mental health issues: A cross-sectional study. International Journal of Clinical Pharmacy. 2022;44(4):904-13. doi: 10.1007/s11096-022-01391-x.

1574. Akpan RM, Udoh EI, Akpan SE, Ozuluoha CC. Community pharmacists' management of self-limiting infections: a simulation study in Akwa Ibom State, South-South Nigeria. African Health Sciences. 2021;21(2):576-84. PubMed PMID: 34795710.

1575. Akpan RM, Udoh IE, Akpan ES, Ozuluoha CC. Community pharmacists' management of self-limiting

infections: a simulation study in Akwa Ibom State, South-South Nigeria. African Health Sciences. 2021;21(2):576-84. doi: 10.4314/ahs.v21i2.12. PubMed PMID: WOS:000719995100012.

1576. Akpoji U, Amos ME, McMillan K, Sims S, Rife K. Exercising empathy: Pharmacists possess skills to increase coronavirus vaccine confidence. Journal of the American Pharmacists Association. 2022;62(1):296-301. doi: https://doi.org/10.1016/j.japh.2021.07.016.

1577. Akram F, Huggan PJ, Lim V, Huang Y, Siddiqui FJ, Assam PN, et al. Medication discrepancies and associated risk factors identified among elderly patients discharged from a tertiary hospital in Singapore. Singapore medical journal. 2015;56(7):379.

1578. Akram W, Ijaz N, Ahmad H, Jamshaid M, Ismail NE. Barriers to the provision of asthma services and perceived practice towards asthma management among urban community pharmacists in Selangor, Malaysia. Brazilian Journal of Pharmaceutical Sciences. 2018;54(4). doi: 10.1590/s2175-97902018000417324. PubMed PMID: WOS:000464217400001.

1579. Akram W, Ijaz N, Butt MH, Ahmad A, Khalid W, Hamid H, et al. Extent of Asthma Knowledge among Community Pharmacists and its Relationship with Demographics and Professional Characteristics: a Regional Analysis. Latin American Journal of Pharmacy. 2020;39(5):862-8. PubMed PMID: WOS:000538168100002.

1580. Akré J. The pharmacist's role in promoting healthy infant feeding. International Pharmacy Journal. 1999;13(1):12-3.

1581. Akrom, Nurwijayanti A. Brief counseling and mobile phone short message service (SMS) increase patient compliance. International journal of pharma medicine and biological sciences. 2015;4(3):175-9. doi: 10.18178/ijpmbs.4.3.175-179. PubMed PMID: CN-01473500.

1582. Akrum V, Essenberg M-V, Veth N, Maat B. The effect of a customized medication verification tool on the amount of unintended clinically relevant medication discrepancies at hospital admission of psychiatric patients. Pharmaceutisch weekblad. 2020;155(1-2):29-32. PubMed PMID: CN-02086776.

1583. Akrum V, Maat B, Veth N. Using a customised medication verification tool on admission to hospital in psychiatric patients to reduce clinically relevant medication discrepancies. European journal of hospital pharmacy. 2019;26:A247-. doi: 10.1136/ejhpharm-2019-eahpconf.531. PubMed PMID: CN-01958451.

1584. Aksamit T, Wu J, Hassan M, Achter E, Chatterjee A. Impact of initiation of amikacin liposome inhalation suspension on hospitalizations and other healthcare resource utilization measures: a retrospective cohort study in real-world settings. Bmc Pulmonary Medicine. 2022;22(1). doi: 10.1186/s12890-022-02257-8. PubMed PMID: WOS:000893396600001.

1585. Akseli I, Stecuła A, He X, Ladyzhynsky N. Quantitative Correlation of the Effect of Process Conditions on the Capping Tendencies of Tablet Formulations. Journal of Pharmaceutical Sciences. 2014;103(6):1652-63. doi: https://doi.org/10.1002/jps.23951.

1586. Aksoy N. Addressing the gap between pharmacy education and practice. Pharmacists: Current Challenges and Perspectives2020. p. 1-37.

1587. Aksoy N, Demirel Ö. Knowledge and attitudes of community pharmacists about COVID-19. Journal of Research in Pharmacy. 2021;25(6):905-12. doi: 10.29228/JRP.87.

1588. Aksoy N, Ozturk N, Ulusoy S, Umar RM, Özaydin S. EVALUATION OF EDUCATIONAL NEEDS OF DIABETIC PATIENTS. Ankara Universitesi Eczacilik Fakultesi Dergisi. 2022;46(2):364-75. doi:

10.33483/jfpau.1079204.

1589. Aksu B, Yeğen G, Yeşilada A. A survey study in Istanbul on communication between pharmacist and patient. Ankara Universitesi Eczacilik Fakultesi Dergisi. 2019;43(1):28-43. doi: 10.33483/jfpau.519932.

1590. Akulayi L, Alum A, Andrada A, Archer J, Arogundade ED, Auko E, et al. Do anti-malarials in Africa meet quality standards? the market penetration of non quality-assured artemisinin combination therapy in eight African countries. Malaria Journal. 2017;16(1). doi: 10.1186/s12936-017-1818-8.

1591. Akulayi L, Alum A, Andrada A, Archer J, Arogundade ED, Auko E, et al. When it just won't go away: Oral artemisinin monotherapy in Nigeria, threatening lives, threatening progress. Malaria Journal. 2017;16(1). doi: 10.1186/s12936-017-2102-7.

1592. Akulayi L, Alum A, Andrada A, Archer J, Arogundade ED, Auko E, et al. The malaria testing and treatment landscape in Kenya: Results from a nationally representative survey among the public and private sector in 2016. Malaria Journal. 2017;16(1). doi: 10.1186/s12936-017-2089-0.

1593. Akulayi L, Alum A, Andrada A, Archer J, Arogundade ED, Auko E, et al. Anti-malarial landscape in Myanmar: Results from a nationally representative survey among community health workers and the private sector outlets in 2015/2016. Malaria Journal. 2017;16(1). doi: 10.1186/s12936-017-1761-8.

1594. Akura H, Nakano T, Hayashi T, Takahashi M, Egawa T. Effects of recommended consultation practice using fracture risk assessment tool and fracture evaluation questionnaire for early detection of osteoporosis at community pharmacies. Yakugaku Zasshi. 2021;141(2):263-72. doi: 10.1248/YAKUSHI.20-00187.

1595. Akura H, Takahashi M, Hayashi T, Egawa T. Evaluation of the collaboration between community interprofessionals and pharmacists for early detection of dementia. Yakugaku Zasshi. 2019;139(12):1583-90. doi: 10.1248/yakushi.19-00098.

1596. Akus M, Bartick M. Lactation safety recommendations and reliability compared in 10 medication resources. Annals of Pharmacotherapy. 2007;41(9):1352-60. PubMed PMID: 47311354.

1597. Akutey R, Der R, Owusu-Daaku F, Baiden F. Using community pharmacies to expand access to screening for noncommunicable diseases in suburban Ghana—A facility-based survey on client needs and acceptability. Health Science Reports. 2018;1(9). doi: 10.1002/hsr2.79.

1598. Akwagyriam I, Goodyer L, Harding L, Khakoo S, Millington H. Drug history taking and the identification of drug related problems in an accident and emergency department. Emergency Medicine Journal. 1996;13(3):166-8.

1599. Akyilmaz ECD. Evaluation of Pharmaceutical Care Services Provided to Hypertensive Patients by the Clinical Pharmacist at the Community Pharmacy2023.

1600. Al A, Prasetyo AKN, Eryando T, Gerke S. Users' Perception of the Hospital Information System in a Maternity Hospital in Lampung, Indonesia. Kesmas-National Public Health Journal. 2019;14(2):76-81. doi: 10.21109/kesmas.v14i2.2574. PubMed PMID: WOS:000500766000005.

1601. Al Adham M, Abu Hamad B. Drug dispensing systems in Gaza hospitals: a comparative study. EMHJ-Eastern Mediterranean Health Journal, 17 (10), 722-729, 2011. 2011.

1602. Al Akshar SA, Shamssain M, Metwaly Z. Pharmacists perceptions of community pharmacy practice in UAE: an Overview. IOSR J Pharm. 2014;4(6):47-56.

1603. Al Alawneh M, Nuaimi N, Abu-Gharbieh E, Basheti IA. A randomized control trial assessing the effect of a pharmaceutical care service on Syrian refugees' quality of life and anxiety. Pharmacy practice (1886-3655). 2020;18(1):1-9. doi: 10.18549/PharmPract.2020.1.1744. PubMed PMID: CN-02182083.

1604. Al Alawneh M, Nuaimi N, Basheti IA. Pharmacists in humanitarian crisis settings: assessing the impact of pharmacist-delivered home medication management review service to Syrian refugees in Jordan. Research in social & administrative pharmacy : RSAP. 2019;15(2):164-72. doi: 10.1016/j.sapharm.2018.04.008. PubMed PMID: CN-01792264.

1605. Al Aloola N, Alanazi M, Alotaibi N, Alwhaibi M. Pharmacists' communication skills with deaf and hard of hearing patients: A needs assessment. PLoS ONE. 2023;17(6):1-12. doi: 10.1371/journal.pone.0286537. PubMed PMID: 164619696.

1606. Al Anazi A. Medication reconciliation process: Assessing value, adoption, and the potential of information technology from pharmacists' perspective. Health Informatics Journal. 2021;27(1):1460458220987276. PubMed PMID: 33467954.

1607. Al Aqeel S, Abanmy N, AlShaya H, Almeshari A. Interventions for improving pharmacist-led patient counselling in the community setting: A systematic review. Systematic Reviews. 2018;7(1). doi: 10.1186/s13643-018-0727-4.

1608. Al Aqeel S, Hiligsmann M. Applying a best–worst scaling methodology to prioritise important attributes of counselling from community pharmacy users' perspective, Saudi Arabia. Journal of Pharmaceutical Health Services Research. 2018;9(2):153-9. doi: 10.1111/jphs.12225.

1609. Al Aqeel S, Hiligsmann M. Applying a best-worst scaling methodology to prioritise important attributes of counselling from community pharmacy users' perspective, Saudi Arabia. Journal of Pharmaceutical Health Services Research. 2018;9(2):153-9. doi: 10.1111/jphs.12225. PubMed PMID: WOS:000431657900011.

1610. Al Assaf S, Kleiner D, Zelkó R, Hankó B. Type 2 Diabetes Patients' Views of Local Pharmacists and Fulfilment with Pharmaceutical Diabetes Care in Syria's Latakia Governorate: An Online Survey Research. Healthcare. 2023;11(12). doi: 10.3390/healthcare11121720. PubMed PMID: WOS:001017505800001.

1611. Al Baz M, Law MR, Saadeh R. Antibiotics use among Palestine refugees attending UNRWA primary health care centers in Jordan - A cross-sectional study. Travel Medicine and Infectious Disease. 2018;22:25-9. doi: 10.1016/j.tmaid.2018.02.004. PubMed PMID: WOS:000432352200005.

1612. Al Damen L, Basheti I. Preventability analysis of adverse drug reactions in a Jordanian hospital: a prospective observational study. International Journal of Clinical Pharmacy. 2019;41(6):1599-610. doi: 10.1007/s11096-019-00925-0.

1613. Al Dulaijan B, Shah R, Davitkov P, Falck-Ytter YT, Macaron C. ONE WEEK POLYETHYLENE GYCOL (PEG) 3350 FOLLOWED BY SPLIT DOSE PEG ELECTOLYTE SOLUTION WITH OR WITHOUT ASCORBIC ACID IMPROVED COLONOSCOPY BOWEL PREPARATION IN PATIENTS WITH CONSTIPATION: a QUALITY IMPROVEMENT PROJECT AT A LARGE VETERANS AFFAIRS HOSPITAL. Gastrointestinal endoscopy. 2020;91(6):AB480-. doi: 10.1016/j.gie.2020.03.2941. PubMed PMID: CN-02130702.

1614. Al Essa M, Alshehri A, Alzahrani M, Bustami R, Adnan S, Alkeraidees A, et al. Practices, awareness and attitudes toward self-medication of analgesics among health sciences students in Riyadh, Saudi Arabia. Saudi Pharmaceutical Journal. 2019;27(2):235-9. doi: https://doi.org/10.1016/j.jsps.2018.11.004.

1615. Al Faleh W, Bin Mubayrik A, Al Dosary S, Almthen H, Alnnatrafi R. Public Perception and Viewpoints of Dental Radiograph Prescriptions and Dentists' Safety Protection Practice. Clinical Cosmetic and Investigational Dentistry. 2020;12:533-9. doi: 10.2147/ccide.S281851. PubMed PMID: WOS:000590452800001.

1616. Al Hagbani T, Alrdaian D, Alshammari RQ, Alshammary G, Ansari M. Assessment of the Quality of Saudi Patient Information Leaflets (PILs) Based on the Accuracy of Physical Description and Frequency of Solid Dosage Forms. Healthcare. 2022;10(3). doi: 10.3390/healthcare10030501. PubMed PMID: WOS:000775446500001.

1617. Al Hail M, Abdulrouf P, Stewart D, Elkassem W, Singh R, Al Enany R, et al. Behaviour and associated determinants of COVID-19 vaccine acceptance and advocacy: a nationwide survey of pharmacy professionals in Qatar. Journal of Pharmaceutical Policy and Practice. 2023;16(1). doi: 10.1186/s40545-023-00668-4.

1618. Al Hajje AH, Calop N, Bosson JL, Calop J, Allenet B. Quels facteurs associés à la survenue d'un événement iatrogène hémorragique chez les patients sous antivitamines K ? Annales Pharmaceutiques Françaises. 2010;68(1):36-43. doi: https://doi.org/10.1016/j.pharma.2009.11.003.

1619. Al Hajje AH, Calop N, Bosson JL, Calop J, Allenet B. [Which factors are associated to hemorrhagic adverse drug events related to antivitamin K?]. Annales Pharmaceutiques Francaises. 2010;68(1):36-43. PubMed PMID: 20176161.

1620. Al Hamarneh Y, Hemmelgarn B, Hassan I, Jones C, Tsuyuki R. The Alberta Vascular Risk Reduction Community Pharmacy Project in patients with CKD (RxEACH-CKD). Canadian pharmacists journal. 2017;150(4):S4-. doi: 10.1177/1715163517719855. PubMed PMID: CN-01408089.

1621. Al Hamarneh YN. Economic Evidence for Pharmacist Prescribing in Community Pharmacy. Encyclopedia of Evidence in Pharmaceutical Public Health and Health Services Research in Pharmacy: Springer; 2023. p. 246-58.

1622. Al Hamarneh YN, Charrois T, Lewanczuk R, Tsuyuki RT. Pharmacist intervention for glycaemic control in the community (the RxING study). BMJ Open. 2013;3(9). doi: 10.1136/bmjopen-2013-003154.

1623. Al Hamarneh YN, Crealey GE, McElnay JC. Coronary heart disease: Health knowledge and behaviour. International Journal of Clinical Pharmacy. 2011;33(1):111-23. doi: 10.1007/s11096-010-9467-9.

1624. Al Hamarneh YN, Hemmelgarn BR, Hassan I, Jones CA, Tsuyuki RT. The Effectiveness of Pharmacist Interventions on Cardiovascular Risk in Adult Patients with Type 2 Diabetes: the Multicentre Randomized Controlled RxEACH Trial. Canadian journal of diabetes. 2017;41(6):580-6. doi: 10.1016/j.jcjd.2017.08.244. PubMed PMID: CN-01621962.

1625. Al Hamarneh YN, Hemmelgarn BR, Hassan I, Jones CA, Tsuyuki RT. The Effectiveness of Pharmacist Interventions on Cardiovascular Risk in Adult Patients with Type 2 Diabetes: The Multicentre Randomized Controlled R_xEACH Trial. Canadian Journal of Diabetes. 2017;41(6):580-6. PubMed PMID: 28965748.

1626. Al Hamarneh YN, Johnston K, Marra C, Tsuyuki R. Cost-effectiveness of pharmacist care for managing patients at high risk for cardiovascular disease in Canada. International journal of clinical pharmacy. 2019;41(1):297-8. doi: 10.1007/s11096-018-0759-9. PubMed PMID: CN-01967031.

1627. Al hamarneh YN, Johnston K, Marra C, Tsuyuki RT. Cost-effectiveness of pharmacist care for managing patients at high risk for cardiovascular disease in Canada. Pharmacy practice. 2018;16. doi:

10.18549/PharmPract.2018.s1.1340. PubMed PMID: CN-02138126.

1628. Al Hamarneh YN, Johnston K, Marra CA, Tsuyuki RT. Pharmacist prescribing and care improves cardiovascular risk, but is it cost-effective? A cost-effectiveness analysis of the RxEACH study. Canadian pharmacists journal. 2019;152(4):257-66. doi: 10.1177/1715163519851822. PubMed PMID: CN-02117060.

1629. Al hamarneh YN, Lamb S, Donald M, Hemmelgarn B, King-Shier K, Jones C, et al. Pharmacist prescribing and care improves cardiovascular risk, but what do patients think? A sub-study of the RxEACH study. Pharmacy practice. 2018;16. doi: 10.18549/PharmPract.2018.s1.1340. PubMed PMID: CN-02164526.

1630. Al Hamarneh YN, Lamb S, Donald M, King-Shier K, Jones C, Hemmelgarn B, et al. Pharmacist prescribing and care improves cardiovascular risk, but what do patients think? A sub-study of the RxEACH study. International journal of clinical pharmacy. 2019;41(1):319-. doi: 10.1007/s11096-018-0759-9. PubMed PMID: CN-01967032.

1631. Al Hamarneh YN, Marra C, Gniadecki R, Keeling S, Morgan A, Tsuyuki R. R x IALTA: Evaluating the effect of a pharmacist-led intervention on CV risk in patients with chronic inflammatory diseases in a community pharmacy setting: A prospective pre-post intervention study. BMJ Open. 2021;11(3). doi: 10.1136/bmjopen-2020-043612.

1632. Al Hamarneh YN, Tsuyuki RT, Jones CA, Manns B, Tonelli M, Scott-Douglass N, et al. Effectiveness of Pharmacist Interventions on Cardiovascular Risk in Patients With CKD: a Subgroup Analysis of the Randomized Controlled RxEACH Trial. American journal of kidney diseases. 2017;(no pagination). doi: 10.1053/j.ajkd.2017.07.012. PubMed PMID: CN-01417558.

1633. Al Hamarneh YN, Tsuyuki RT, Jones CA, Manns B, Tonelli M, Scott-Douglass N, et al. Effectiveness of Pharmacist Interventions on Cardiovascular Risk in Patients With CKD: a Subgroup Analysis of the Randomized Controlled RxEACH Trial. American journal of kidney diseases. 2018;71(1):42-51. doi: 10.1053/j.ajkd.2017.07.012. PubMed PMID: CN-01952440.

1634. Al Hamid A, Malik A, Alyatama S. An exploration of patient safety culture in Kuwait hospitals: a qualitative study of healthcare professionals' perspectives. International Journal of Pharmacy Practice. 2020;28(6):617-25.

1635. Al Jalal ZSMA. Impact of motivational interviews within pharmacy care upon adherence to cardiovascular medicines : a feasibility pilot controlled trial [Ph.D.]. England: University of London, University College London (United Kingdom); 2016.

1636. Al Jeraisy M, Alshammari H, Albassam M, Al Aamer K, Abolfotouh MA. Utility of patient information leaflet and perceived impact of its use on medication adherence. BMC Public Health. 2023;23(1):488.

1637. Al Jomaa EE, Abdel-Qader DH, Silverthorne J, Hamadi S, Al Meslamani AZ. Pharmacy students' attitude towards providing pharmaceutical care to patients with anxiety and their knowledge of psychotropic medicines in Jordan. International Journal of Pharmacy Practice. 2021;29(2):196-9.

1638. Al Juffali L, Al-Aqeel S, Knapp P, Mearns K, Family H, Watson M. Using the human factors framework to understand the origins of medication safety problems in community pharmacy: a qualitative study. Research in Social and Administrative Pharmacy. 2019;15(5):558-67.

1639. Al Juma MA. Self care in Oman : public and healthcare professional perspectives [Ph.D.]. England: The University of Nottingham (United Kingdom); 2017.

1640. Al Jumah K. The impact of a decision aid on depressed patients' involvement in shared decision making: a pilot randomized controlled double-blinded study. Value in health Conference: ISPOR 21st annual international meeting research Washington, DC united states Conference start: 20160521 Conference end: 20160525 Conference publication: (varpagings). 2016;19(3):A19. PubMed PMID: CN-01266612.

1641. Al Khaja K, Sequeira R, Al-Ansari T, Damanhori A. Prescription writing skills of residents in a family practice residency programme in Bahrain. Postgraduate medical journal. 2008;84(990):198-204.

1642. Al Khaja KA, Al Ansari TM, Damanhori AH, Sequeira RP. Evaluation of drug utilization and prescribing errors in infants: a primary care prescription-based study. Health policy. 2007;81(2-3):350-7.

1643. Al Khames Aga QA, Haddad M, Bataineh YA, Dakkah AN, Shnoudeh AJ, Mansour RSH. Evaluation of pharmacy student and community pharmacist knowledge and impact on disease state management of patients with Asthma. Systematic reviews in pharmacy. 2020;11(8):472-80. doi: 10.31838/srp.2020.8.68. PubMed PMID: CN-02242670.

1644. Al Khayrallah Z, Al-Saeedy Z, Medriano RAA, Lee K, Sommerfield A, Sommerfield D, et al. Prescribing in a pediatric hospital setting - Lost in translation? Patient Education & Counseling. 2022;105(6):1614-9. doi: 10.1016/j.pec.2021.10.010. PubMed PMID: 157047674. Language: English. Entry Date: 20220601. Revision Date: 20220715. Publication Type: journal article.

1645. Al Khayrallah Z, Al-Saeedy Z, Medriano RAA, Lee K, Sommerfield A, Sommerfield D, et al. Prescribing in a pediatric hospital setting – Lost in translation? Patient Education and Counseling. 2022;105(6):1614-9. doi: https://doi.org/10.1016/j.pec.2021.10.010.

1646. Al Khoury A, Chao CY, Aruljothy A, Wyse J, Bessissow T. Intensified infliximab rescue therapy for acute severe ulcerative colitis does not improve long term colectomy-free survival. Journal of crohn's and colitis Conference: 12th congress of the european crohn's and colitis organisation, ECCO 2017 Spain. 2017;11(Supplement 1):S330-S1. doi: 10.1093/ecco-jcc/jjx002.619. PubMed PMID: CN-01469871.

1647. Al Kubaisi K, Hasan S, Hassan NA, Elnour AA. A pseudo-customer cross-sectional study to evaluate the community pharmacist?s management of migraine in pregnant women. Pharmacy Practice-Granada. 2022;20(4). doi: 10.18549/PharmPract.2022.4.2739. PubMed PMID: WOS:000896745700001.

1648. Al Kuwaiti A. APPLICATION OF SIX SIGMA METHODOLOGY TO REDUCE MEDICATION ERRORS IN THE OUTPATIENT PHARMACY UNIT: A CASE STUDY FROM THE KING FAHD UNIVERSITY HOSPITAL, SAUDI ARABIA. International Journal for Quality Research. 2016;10(2):267-78. doi: 10.18421/ijqr10.02-03. PubMed PMID: WOS:000410706800003.

1649. Al Kuwaiti A, Al Muhanna FA. Challenges facing healthcare leadership in attaining accreditation of teaching hospitals. Leadership in Health Services. 2019;32(2):170-81.

1650. Al Kuwaiti A, Nazer K, Al-Reedy A, Al-Shehri S, Al-Muhanna A, Subbarayalu AV, et al. A Review of the Role of Artificial Intelligence in Healthcare. Journal of Personalized Medicine. 2023;13(6):951. doi: 10.3390/jpm13060951. PubMed PMID: 164685252.

1651. Al Laif FZ, Ahmad R, Naqvi AA, Ahmad N. Pharmacist Perceived Barriers to Patient Counseling; A Study in Eastern Region of Saudi Arabia. British Journal of Pharmaceutical Research. 2017;19(6). doi: 10.9734/jpri/2017/37705. PubMed PMID: WOS:000417386400004.

1652. Al Manasra T, Shah SQ, Raj SR, Pollak PT. Amiodarone Thyrotoxicosis: A Case of Pharmacy Error Obscured by Amiodarone Suppression of Symptoms of Highly Elevated Thyroxine Concentration. CJC Open. 2022. doi: https://doi.org/10.1016/j.cjco.2022.11.019.

1653. Al Manasra T, Shah SQ, Raj SR, Pollak PT. Amiodarone Thyrotoxicosis: A Case of Pharmacy Error Obscured by Amiodarone Suppression of Symptoms of Highly Elevated Thyroxine Concentration. CJC Open. 2023;5(2):173-6. doi: https://doi.org/10.1016/j.cjco.2022.11.019.

1654. Al Mazrouei N, Ibrahim RM, Al Meslamani AZ, Abdel-Qader DH, Ibrahim OM. Virtual pharmacist interventions on abuse of over-the-counter medications during COVID-19 versus traditional pharmacist interventions. Journal of the American Pharmacists Association. 2021;61(3):331-9. doi: 10.1016/j.japh.2021.02.003. PubMed PMID: WOS:000651401000037.

1655. Al Mazrouei N, Ibrahim RM, Al Meslamani AZ, Abdel-Qader DH, Mohamed Ibrahim O. Virtual pharmacist interventions on abuse of over-the-counter medications during COVID-19 versus traditional pharmacist interventions. Journal of the American Pharmacists Association. 2021;61(3):331-9. doi: 10.1016/j.japh.2021.02.003.

1656. Al Mazrouei N, Ibrahim RM, Al Meslamani AZ, Abdel-Qader DH, Sadeq AS, Mohamed Ibrahim O. The evolving role of community pharmacists during COVID-19 in the UAE; assessing preparedness and knowledge. International Journal of Pharmacy Practice. 2021;29(2):137-44.

1657. Al Mazrouei N, Ibrahim RM, Al Meslamani AZ, Ibrahim OM. A novel educational approach for improving medication-related problems in community pharmacies. Research in Social & Administrative Pharmacy. 2022;18(3):2510-6. doi: 10.1016/j.sapharm.2021.04.017. PubMed PMID: WOS:000751828900017.

1658. Al Mazrouei N, Ibrahim RM, Al Meslamani AZ, Mohamed Ibrahim O. A novel educational approach for improving medication-related problems in community pharmacies. Research in social & administrative pharmacy : RSAP. 2022;18(3):2510-6. doi: 10.1016/j.sapharm.2021.04.017. PubMed PMID: CN-02286457.

1659. Al Mazroui NR, Kamal MM, Ghabash NM, Yacout TA, Kole PL, McElnay JC. Influence of pharmaceutical care on health outcomes in patients with Type 2 diabetes mellitus. British journal of clinical pharmacology. 2009;67(5):547-57. doi: 10.1111/j.1365-2125.2009.03391.x. PubMed PMID: CN-00729261.

1660. Al Meslamani AZ. Investigating Medication Prescribing Errors in the Emergency Department of a Governmental Hospital and Measuring Pharmacy Students' Knowledge and Attitude of Prescribing Errors in Jordan [M.S.]. Jordan: University of Petra (Jordan); 2019.

1661. Al Meslamani AZ. Applications of AI in pharmacy practice: a look at hospital and community settings. Journal of Medical Economics. 2023;26(1):1081-4. doi: 10.1080/13696998.2023.2249758.

1662. Al Meslamani AZ, Abdel-Qader DH. The Abuse and Misuse of Over-the-Counter Medicines During COVID-19. Hospital Pharmacy. 2023;58(5):437-40. doi: 10.1177/00185787231158777. PubMed PMID: 171848119.

1663. Al Meslamani AZ, Abu-Naser D, Abdel-Qader DH, Aljamal MS, Alsharif MA, Alshrahili MAM, et al. Assessment of inappropriate prescribing of QT interval-prolonging drugs in end-stage renal disease patients in Jordan. Drugs & Therapy Perspectives. 2021;37(2):87-93. doi: 10.1007/s40267-020-00806-x. PubMed PMID: 149031413. Language: English. Entry Date: 20210310. Revision Date: 20210312. Publication Type: Article.

1664. Al Meslamani AZ, Aldulaymi R, El Sharu H, Alwarawrah Z, Ibrahim OM, Al Mazrouei N. The patterns and determinants of telemedicine use during the COVID-19 crisis: A nationwide study. Journal of the American Pharmacists Association. 2022;62(6):1778-85. doi: https://doi.org/10.1016/j.japh.2022.05.020.

1665. Al Meslamani AZ, Jarab AS, Abdel-Qader DH, Ibrahim OM, Al Mazrouei N. Adoption of electronic

patient medication records in community pharmacies in the United Arab Emirates: A cross-sectional survey. Health Information Management Journal. 2023. doi: 10.1177/18333583231190744. PubMed PMID: WOS:001043068900001.

1666. Al Meslamani AZ, Jarab AS, Abdel-Qader DH, Mohamed Ibrahim O, Al Mazrouei N. Adoption of electronic patient medication records in community pharmacies in the United Arab Emirates: A cross-sectional survey. Health Information Management Journal. 2023:1. doi: 10.1177/18333583231190744. PubMed PMID: 169820757.

1667. Al Nemari M, Waterson J. The Introduction of Robotics to an Outpatient Dispensing and Medication Management Process in Saudi Arabia: Retrospective Review of a Pharmacy-led Multidisciplinary Six Sigma Performance Improvement Project. JMIR Human Factors. 2022;9(4):e37905. PubMed PMID: 36222805.

1668. Al Nuhait M, Al Harbi K, Al Jarboa A, Bustami R, Alharbi S, Masud N, et al. Sickness presenteeism among health care providers in an academic tertiary care center in Riyadh. Journal of Infection and Public Health. 2017;10(6):711-5. doi: https://doi.org/10.1016/j.jiph.2016.09.019.

1669. Al Omar S, Nazer L, Sarhan Z, Amireh A, Farraj R. Evaluating the level of knowledge of medicines and need for information among people caring for paediatric cancer patients. European Journal of Hospital Pharmacy. 2015;22(Supplement 1):A78. PubMed PMID: 614322494.

1670. Al Rahbi HAM, Al-Sabri RM, Chitme HR. Interventions by pharmacists in out-patient pharmaceutical care. Saudi pharmaceutical journal. 2014;22(2):101-6.

1671. Al Raiisi F, Stewart D, Fernandez-Llimos F, Salgado TM, Mohamed MF, Cunningham S. Clinical pharmacy practice in the care of Chronic Kidney Disease patients: a systematic review. International Journal of Clinical Pharmacy. 2019;41(3):630-66. doi: 10.1007/s11096-019-00816-4.

1672. Al Rawwad T, Schrader PK, Brooks A, Duong L, Thornton D. Do nursing and pharmacy students practice what they preach on safe drug storage and disposal? A cross-sectional study. Nurse Education Today. 2021;107:105143. doi: https://doi.org/10.1016/j.nedt.2021.105143.

1673. Al Rawwad T, Tata V, Wanat MA, Campbell D, Thornton D. Evaluating the feasibility of implementing a prescription drug misuse prevention intervention in the community: a mixed methods study. BMC Public Health. 2023;23(1). doi: 10.1186/s12889-023-15608-9.

1674. Al Rayees M, Mukhtar O, Al Yousef AR, Al Zahrani AB. REDUCING THE MEDICATION ERRORS USING ELECTRONIC PRESCRIPTIONS. Indo American Journal of Pharmaceutical Sciences. 2019;6(3):5817-24. doi: 10.5281/zenodo.2598062. PubMed PMID: WOS:000475330100167.

1675. Al Rifai LA, Soubra L, Hassanein M, Amin MEK. Comprehensive care for patients with diabetes in Ramadan: A module for pharmacy students and pharmacists. Saudi Pharmaceutical Journal. 2022;30(5):619-28. doi: 10.1016/j.jsps.2022.02.008.

1676. Al Rowily A, Aloudah N, Jalal Z, Abutaleb M, Baraka M, Paudyal V. Medication errors in relation to direct-acting oral anticoagulants: a qualitative study of pharmacists' views and experiences. International Journal of Clinical Pharmacy. 2023;45(3):681-8. doi: https://dx.doi.org/10.1007/s11096-023-01555-3. PubMed PMID: 2022312540.

1677. Al Rowily A, Aloudah N, Jalal Z, Abutaleb MH, Paudyal V. Views, experiences and contributory factors related to medication errors associated with direct oral anticoagulants: a qualitative study with physicians and nurses. International Journal of Clinical Pharmacy. 2022;44(4):1057-66. PubMed PMID: 35731467.

1678. Al Rowily A, Baraka MA, Abutaleb MH, Alhayyan AM, Aloudah N, Jalal Z, et al. Patients' views and experiences on the use and safety of directly acting oral anticoagulants: a qualitative study. Journal of Pharmaceutical Policy & Practice. 2023;16(1):1-8. doi: 10.1186/s40545-023-00563-y. PubMed PMID: 163414432.

1679. Al Ruwaili N, Al Balushi A, Alharf A, AlShaharani H, Eldali A. Do parents in Saudi Arabia store medications safely? International Journal of Pediatrics and Adolescent Medicine. 2014;1(1):21-5. doi: https://doi.org/10.1016/j.ijpam.2014.09.003.

1680. Al Saigh R, Herzallah R, Alhusban A. An assessment of the interaction between studying pharmacy, problematic use of social media and depression. American Journal of Pharmaceutical Education. 2021;85(9):1-8. doi: 10.5688/ajpe8625.

1681. Al Sawah S, Foster SA, Goldblum OM, Malatestinic WN, Zhu BJ, Shi NW, et al. Healthcare costs in psoriasis and psoriasis sub-groups over time following psoriasis diagnosis. Journal of Medical Economics. 2017;20(9):982-90. doi: 10.1080/13696998.2017.1345749. PubMed PMID: WOS:000407931300010.

1682. Al Shahaibi NM, Al Said LS, Chitme H, Kini T. Identifying errors in handwritten outpatient prescriptions in Oman. Journal of Young Pharmacists. 2012;4(4):267-72.

1683. Al Shemeili S, Klein S, Strath A, Fares S, Stewart D. An exploration of health professionals' experiences of medicines management in elderly, hospitalised patients in Abu Dhabi. International Journal of Clinical Pharmacy. 2016;38(1):107-18. PubMed PMID: 26499504.

1684. Al Shibly MNR, Hasan LA, Abdulsada AR. Journal of Population Therapeutics & Clinical Pharmacology. Journal of Population Therapeutics and Clinical Pharmacology. 2021;28(2):E62-E70. doi: 10.47750/jptcp.2022.863. PubMed PMID: WOS:000772156100007.

1685. Al Shibly MNR, Hasan LA, Abdulsada AR. Knowledge and practice of self-medication. Journal of population therapeutics and clinical pharmacology = Journal de la therapeutique des populations et de la pharmacologie clinique. 2022;28(2):e62-e70. doi: 10.47750/jptcp.2022.863.

1686. Al Souheil F, Chahine B. Statin Prescription Patterns Among Elderly Patients with Type 2 Diabetes: A Cross-Sectional Study in Lebanon. Drugs-Real World Outcomes. doi: 10.1007/s40801-022-00335-1. PubMed PMID: WOS:000887882600001.

1687. Al Souheil F, Chahine B. Statin Prescription Patterns Among Elderly Patients with Type 2 Diabetes: A Cross-Sectional Study in Lebanon. Drugs - Real World Outcomes. 2022. doi: 10.1007/s40801-022-00335-1.

1688. Al Souheil F, Chahine B. Statin Prescription Patterns Among Elderly Patients with Type 2 Diabetes: A Cross-Sectional Study in Lebanon. Drugs - Real World Outcomes. 2023;10(1):159-66. doi: 10.1007/s40801-022-00335-1.

1689. Al Wazzan AA, Tommelein E, Foubert K, Bonassi S, Onder G, Somers A, et al. Development and Application of the GheOP(3)S-Tool Addendum on Potentially Inappropriate Prescribing (PIP) of Renally Excreted Active Drugs (READs) in Older Adults with Polypharmacy. Drugs & Aging. 2018;35(4):343-64. doi: 10.1007/s40266-018-0530-x. PubMed PMID: WOS:000430197100007.

1690. Al Zabadi H, Shraim R, Sawalha R, Alkaiyat A. Outpatients satisfaction and perceptions toward pharmaceutical services in public and private hospitals in Palestine: a cross-sectional study. Journal of Pharmaceutical Policy and Practice. 2023;16(1):108. doi: https://dx.doi.org/10.1186/s40545-023-00608-2. PubMed PMID: 2025785981.

1691. Al-Abdelmuhsin L, Al-Ammari M, Babelghaith SD, Wajid S, Alwhaibi A, Alghadeer SM, et al. Assessment of Pharmacists' Knowledge and Practices towards Prescribed Medications for Dialysis Patients at a Tertiary Hospital in Riyadh Saudi Arabia. Healthcare. 2021;9(9). doi: 10.3390/healthcare9091098. PubMed PMID: WOS:000699735200001.

1692. Al-Abdelmuhsin L, Al-Ammari M, Babelghaith SD, Wajid S, Asiri YA, Almetawaz MS, et al. Pharmacist-led medication counseling for patients undergoing hemodialysis: A path to better adherence. International Journal of Environmental Research and Public Health. 2020;17(7). doi: 10.3390/ijerph17072399.

1693. Al-Ahmadi RF, Al-Juffali L, Al-Shanawani S, Ali S. Categorizing and understanding medication errors in hospital pharmacy in relation to human factors. Saudi Pharmaceutical Journal. 2020;28(12):1674-85. doi: https://doi.org/10.1016/j.jsps.2020.10.014.

1694. Al-Alhazmi NNN. Role of pharmacists in adverse drug reaction reporting in holy city of makkah, saudi arabia: characterisation of models for evaluating pharmacovigilance in the holy city of makkah and attitude and awareness of pharmacists towards adverse drug reaction reporting [Ph.D.]. England: University of Bradford (United Kingdom); 2013.

1695. Al-Amad SH, Ghebeh M, Saloum P, Maarouf TB, Moustafa YM, Younis Z. Pharmacists' clinical competency towards oral mucosal diseases: Results from a mystery shopper study. Oral Diseases. 2020;26(1):89-95. doi: 10.1111/odi.13208. PubMed PMID: WOS:000495562200001.

1696. Al-Ani OAS. Cardio-Renal Syndrome. Journal of Population Therapeutics and Clinical Pharmacology. 2023;30(3):E144-E54. doi: 10.47750/jptcp.2023.30.03.017. PubMed PMID: WOS:000992654400013.

1697. Al-Aqeel SA, Al-Sabhan JF, Sultan NY. Analysis of written advertising material distributed through Community Pharmacies in Riyadh, Saudi Arabia. Pharmacy Practice. 2013;11(3):138-43. doi: 10.4321/S1886-36552013000300003.

1698. Al-Araidah O, Momani A, Khasawneh M, Momani M. Lead-time reduction utilizing lean tools applied to healthcare: the inpatient pharmacy at a local hospital. Journal for Healthcare Quality: Promoting Excellence in Healthcare. 2010;32(1):59-66. doi: 10.1111/j.1945-1474.2009.00065.x. PubMed PMID: 105298123. Language: English. Entry Date: 20100305. Revision Date: 20200708. Publication Type: Journal Article.

1699. Al-Arifi M, Abu-Hashem H, Al-Meziny M, Said R, Aljadhey H. Emergency department visits and admissions due to drug related problems at Riyadh military hospital (RMH), Saudi Arabia. Saudi Pharmaceutical Journal. 2014;22(1):17-25. doi: https://doi.org/10.1016/j.jsps.2013.01.001.

1700. Al-Arifi MN. Prevalence of smoking and attitude toward smoking cessation among community pharmacists, Saudi Arabia. Journal of Pharmacy Technology. 2004;20(6):329-33. doi: 10.1177/875512250402000604.

1701. Al-Arifi MN. Community pharmacists' attitudes towards mental illness and providing pharmaceutical care for mentally ill patients. Neurosciences. 2008;13(4):412-20.

1702. Al-Arifi MN. Patients' perception, views and satisfaction with pharmacists' role as health care provider in community pharmacy setting at Riyadh, Saudi Arabia. Saudi Pharmaceutical Journal. 2012;20(4):323-30. doi: https://doi.org/10.1016/j.jsps.2012.05.007.

1703. Al-Arifi MN. Availability and needs of herbal medicinal information resources at community pharmacy, Riyadh region, Saudi Arabia. Saudi Pharmaceutical Journal. 2013;21(4):351-60. doi: 10.1016/

j.jsps.2012.11.004. PubMed PMID: WOS:000325516700004.

1704. Al-Arifi MN. Community pharmacist perception and attitude toward ethical issues at community pharmacy setting in central Saudi Arabia. Saudi Pharmaceutical Journal. 2014;22(4):315-25. doi: https://doi.org/10.1016/j.jsps.2013.08.003.

1705. Al-Arkee S, Mason J, Lindenmeyer A, Jalal Z. Pharmacist management of atrial fibrillation in UK primary care: a qualitative study. Journal of Pharmaceutical Policy and Practice. 2022;15(1). doi: 10.1186/s40545-022-00486-0.

1706. Al-Ashwal FY, Sulaiman SAS, Ghadzi SMS, Kubas MA, Halboup A. Knowledge, attitude, perceived barriers, and practices among pharmacists regarding risk assessment of cardiovascular disease: a cross-sectional study in Yemen. Current Medical Research and Opinion. 2022;38(3):451-9. doi: 10.1080/03007995.2021.1994380. PubMed PMID: WOS:000714268800001.

1707. Al-Ashwal FY, Sulaiman SAS, Ghadzi SMS, Kubas MA, Halboup A. Physicians and pharmacists' clinical knowledge of statin therapy and monitoring parameters, and the barriers to guideline implementation in clinical practice. PLoS ONE. 2023;18(1 January). doi: 10.1371/journal.pone.0280432.

1708. Al-Ashwal FY, Sulaiman SAS, Ghadzi SMS, Kubas MA, Halboup A. Physicians and pharmacists' clinical knowledge of statin therapy and monitoring parameters, and the barriers to guideline implementation in clinical practice. PLoS ONE. 2023;18(1 January). doi: 10.1371/journal.pone.0280432.

1709. Al-Ashwal FY, Sulaiman SAS, Sheikh Ghadzi SM, Kubas MA, Halboup A. Knowledge, attitude, perceived barriers, and practices among pharmacists regarding risk assessment of cardiovascular disease: a cross-sectional study in Yemen. Current Medical Research and Opinion. 2022;38(3):451-9. doi: 10.1080/03007995.2021.1994380.

1710. Al-Ashwal FY, Sulaiman SAS, Sheikh Ghadzi SM, Kubas MA, Halboup A. Physicians and pharmacists’ clinical knowledge of statin therapy and monitoring parameters, and the barriers to guideline implementation in clinical practice. PLoS One. 2023;18(1):e0280432.

1711. Al-Assaf AF, Bumpus LJ, Carter D, Dixon SB. Preventing errors in healthcare: a call for action. Hospital topics. 2003;81(3):5-13.

1712. Al-Asseri AA, Al-Achi A, Greenwood R. Counseling and post-discharge care. Saudi pharmaceutical journal. 2001;9(2):119-21. PubMed PMID: CN-00442482.

1713. Al-Aswad H, El-Medany WM, Balakrishna C, Ababneh N, Curran K. BZKP: Blockchain-based zero-knowledge proof model for enhancing healthcare security in Bahrain IoT smart cities and COVID-19 risk mitigation. Arab Journal of Basic and Applied Sciences. 2021;28(1):154-71. doi: 10.1080/25765299.2020.1870812.

1714. Al-Azayzih A, Al-Azzam SI, Alzoubi KH, Shawaqfeh M, Masadeh MM. Evaluation of drug-prescribing patterns based on the WHO prescribing indicators at outpatient clinics of five hospitals in Jordan: a cross-sectional study. International Journal of Clinical Pharmacology and Therapeutics. 2017;55(5):425-32. doi: 10.5414/cp202733. PubMed PMID: WOS:000402013100007.

1715. Al-Azayzih A, Al-Qerem W, Kharaba Z, Albiss L, Alawneh A, Abu-Dalhoum D, et al. Environmental and personal barriers encountered in community pharmacy settings based on public opinions. Future Science Oa. 2023;10(1). doi: 10.2144/fsoa-2023-0166. PubMed PMID: WOS:001108532200001.

1716. Al-Azzam SI, Alzoubi KH, AbuRuz S, Alefan Q. Drug-related problems in a sample of outpatients

with chronic diseases: a cross-sectional study from Jordan. Therapeutics and Clinical Risk Management. 2016;12:233-9. doi: 10.2147/tcrm.S98165. PubMed PMID: WOS:000370220700001.

1717. Al-Azzawi S, Masheta D. Impact of the COVID-19 pandemic on dispensing medicines in the community pharmacy. International Journal of Risk and Safety in Medicine. 2023;34(4):295-311. doi: 10.3233/JRS-220061.

1718. Al-babtain B, Cheema E, Hadi MA. Impact of community-pharmacist-led medication review programmes on patient outcomes: A systematic review and meta-analysis of randomised controlled trials. Research in Social & Administrative Pharmacy. 2022;18(4):2559-68. doi: 10.1016/j.sapharm.2021.04.022. PubMed PMID: WOS:000759248000002.

1719. Al-Badriyeh D, Kaddoura R, AlMaraghi F, Homosy A, Hail MA, El-Kassem W, et al. Impact of Clinical Pharmacist Interventions on Economic Outcomes in a Cardiology Setting in Qatar. Current Problems in Cardiology. 2023;48(9):101838. doi: https://doi.org/10.1016/j.cpcardiol.2023.101838.

1720. Al-Baghdadi H, Koca Al-Baghdadi Ç, Abdi A, Gültekin O, Meštrović A, Demirdamar R, et al. Introducing clinical pharmacy services to cardiovascular clinics at a university hospital in Northern Cyprus. International Journal of Clinical Pharmacy. 2017;39(6):1185-93. doi: 10.1007/s11096-017-0534-3. PubMed PMID: 126307421. Language: English. Entry Date: 20180706. Revision Date: 20191023. Publication Type: journal article.

1721. Al-Bakri AG, Bustanji Y, Yousef AM. Community consumption of antibacterial drugs within the Jordanian population: Sources, patterns and appropriateness. International Journal of Antimicrobial Agents. 2005;26(5):389-95. doi: 10.1016/j.ijantimicag.2005.07.014.

1722. Al-baloshi H, Al-Faleti N, Al-Ghalani I, Al-Baloshi W, Krishnan Nair U, Al-Salmani S, et al. Analysis of pharmacists' interventions on paediatric outpatient prescriptions in South Batinah Governorate, Oman. Pharmacy Education. 2023;23(3):291. doi: https://dx.doi.org/10.46542/pe.2023.233.262300. PubMed PMID: 642265829.

1723. Al-Bawardy R, Cheng-Lai A, Prlesi L, Assafin M, Xu S, Chen K, et al. Heart Failure Postdischarge Clinic: a Pharmacist-led Approach to Reduce Readmissions. Current problems in cardiology. 2019;44(10):100407. doi: 10.1016/j.cpcardiol.2018.12.004. PubMed PMID: CN-02078401.

1724. Al-Blowi AS. Pharmaceutical care for patients with type 2 diabetes in primary care [Ph.D.]. England: University of Bradford (United Kingdom); 2007.

1725. Al-Dahir S, Bryant K, Kennedy KB, Robinson DS. Online virtual-patient cases versus traditional problem-based learning in advanced pharmacy practice experiences. American journal of pharmaceutical education. 2014;78(4):76. doi: 10.5688/ajpe78476. PubMed PMID: CN-01047228.

1726. Al-Dewaissan F, Hill D, Evans L, Luz TCB. Suboptimal dosing of opioid agonist treatments - a literature review. Heroin Addiction and Related Clinical Problems. 2022;24(5):7-15. PubMed PMID: WOS:000877815800002.

1727. Al-Dewik NI, Morsi H, Ghasoub R, Yassin MA. Studying the Impact of Imatinib Mesylate (IM) Adherence in Chronic Myeloid Leukemia (CML) patients' Responses in the State of Qatar. Blood. 2014;124(21):5537. doi: https://doi.org/10.1182/blood.V124.21.5537.5537.

1728. Al-Dhawailie AA. Inpatient prescribing errors and pharmacist intervention at a teaching hospital in Saudi Arabia. Saudi Pharmaceutical Journal. 2011;19(3):193-6. doi: https://doi.org/10.1016/j.jsps.2011.03.001.

1729. Al-Diery T, Freeman H, Page AT, Cross AJ, Hawthorne D, Lee K. What types of information do pharmacists include in comprehensive medication management review reports? A qualitative content analysis. International Journal of Clinical Pharmacy. 2023;45(3):712-21. doi: https://dx.doi.org/10.1007/s11096-023-01561-5. PubMed PMID: 2022098530.

1730. Al-Diery T, Freeman H, Page AT, Cross AJ, Hawthorne D, Lee KN. What types of information do pharmacists include in comprehensive medication management review reports? A qualitative content analysis. International Journal of Clinical Pharmacy. 2023;45(3):712-21. doi: 10.1007/s11096-023-01561-5. PubMed PMID: WOS:000951283600001.

1731. Al-Dossari DS, Al-Zaagi IA, Bamogaddam RF, Alnajrani RH, Alnajrani NR, Alajami HN, et al. Hospital pharmacists' awareness and perspective toward the management of COVID-19 pandemic in the Kingdom of Saudi Arabia. Pharmacy Practice-Granada. 2022;20(3). doi: 10.18549/PharmPract.2022.3.2709. PubMed PMID: WOS:000860271500001.

1732. Al-Dossari DS, Laghbi YA, Almutairi AS, Alsupail MM, Alharbi FK, Alharbi MH, et al. Impact of telepharmacy on patients' outcome during COVID-19: a systematic literature review. Pharmacy Practice. 2023;21(4):2883. doi: https://dx.doi.org/10.18549/PharmPract.2023.4.2883. PubMed PMID: 2028198364.

1733. Al-Dujaili Z, Hallit S, Al Faraj A. Knowledge, attitude, and readiness of pharmacists toward medication therapy management for patients with attention deficit hyperactivity disorder: a cross-sectional quantitative study. International Journal of Clinical Pharmacy. 2023;45(5):1277-85. doi: https://dx.doi.org/10.1007/s11096-023-01628-3. PubMed PMID: 2025387037.

1734. Al-Dujaili Z, Omari S, Pillai J, Al Faraj A. Assessing the accuracy and consistency of ChatGPT in clinical pharmacy management: A preliminary analysis with clinical pharmacy experts worldwide. Research in Social and Administrative Pharmacy. 2023;19(12):1590-4. doi: https://doi.org/10.1016/j.sapharm.2023.08.012.

1735. Al-Eidan FA, McElnay JC, Scott MG, Kearney MP, Corrigan J, McConnell JB. Use of a treatment protocol in the management of community-acquired lower respiratory tract infection. Journal of Antimicrobial Chemotherapy. 2000;45(3):387-94. doi: 10.1093/jac/45.3.387.

1736. Al-Eidan FA, McElnay JC, Scott MG, McConnell JB. Management of Helicobacter pylori eradication--the influence of structured counselling and follow-up. British journal of clinical pharmacology. 2002;53(2):163-71. doi: 10.1046/j.0306-5251.2001.01531.x. PubMed PMID: CN-00378047.

1737. Al-Fadhel MA, Naylor IL. Pharmaceutical care provided by community pharmacists for diabetic patients in the city of Riyadh. Saudi Pharmaceutical Journal. 2002;10(4):196-203.

1738. Al-Ghadeer AA. Pharmacoeconomic evaluation of hospital -based therapies [Ph.D.]. Northern Ireland: Queen's University Belfast (United Kingdom); 1999.

1739. Al-Ghamdi MS. Continuing pharmaceutical education for community pharmacists in the eastern province of Saudi Arabia. Journal of Family and Community Medicine. 2001;8(3):45-52.

1740. Al-Ghamdi MS. Empirical treatment of uncomplicated urinary tract infection by community pharmacist in the Eastern province of Saudi Arabia. Saudi Medical Journal. 2001;22(12):1105-8. PubMed PMID: WOS:000173130300011.

1741. Al-Ghananeem AM, Malcom DR, Shammas S, Aburjai T. A Call to Action to Transform Pharmacy Education and Practice in the Arab World. American Journal of Pharmaceutical Education. 2018;82(9):1023-8. PubMed PMID: WOS:000452604500002.

1742. Al-Ghobashy MA, Mostafa MM, Abed HS, Fathalla FA, Salem MY. Correlation between Dynamic Light Scattering and Size Exclusion High Performance Liquid Chromatography for Monitoring the Effect of pH on Stability of Biopharmaceuticals. Journal of Chromatography B. 2017;1060:1-9. doi: https://doi.org/10.1016/j.jchromb.2017.05.029.

1743. Al-Hajje A, Atoui F, Awada S, Rachidi S, Zein S, Salameh P, editors. Drug-related problems identified by clinical pharmacist's students and pharmacist's interventions. Annales pharmaceutiques françaises; 2012: Elsevier.

1744. Al-Hajje AH, Atoui F, Awada S, Rachidi S, Zein S, Salameh P. Drug-related problems identified by clinical pharmacist's students and pharmacist's interventions. Annales Pharmaceutiques Françaises. 2012;70(3):169-76. doi: https://doi.org/10.1016/j.pharma.2012.02.004.

1745. Al-Hamaideh KD, Dmour IM. Diuretics misuse: A cross-sectional study to explore community pharmacist's perceptions of diuretics use and misuse. Journal of Applied Pharmaceutical Science. 2023;13(6):128-36. doi: 10.7324/JAPS.2023.104335.

1746. Al-Haqan A, Moreau P, Alghanem S, Shaaban J, Alsalem A, Hajjiah A, et al. Transforming pharmacy practice in Kuwait. International Journal of Health Planning and Management. 2023;38(4):879-88. doi: 10.1002/hpm.3631.

1747. Al-Harbi TF, Al-Harbi M, Asdaq SMB, Al-Yamani M. Behavior of community pharmacist of Buraydah City, Saudi Arabia, on dispensing antibiotics without prescription: A cross-sectional study. Latin American Journal of Pharmacy. 2017;36(9):1849-53.

1748. Al-Hasani NR, Kadhim DJ, Al-Jumaili AA. Exploring the role of community pharmacists in preventing the onsite infection during COVID-19 pandemic. Pharmaceutical Sciences Asia. 2022;49(2):169-79. doi: 10.29090/psa.2022.02.21.129.

1749. Al-Hashar A, Al-Zakwani I, Eriksson T, Al Za'abi M. Whose responsibility is medication reconciliation: Physicians, pharmacists or nurses? A survey in an academic tertiary care hospital. Saudi Pharmaceutical Journal. 2017;25(1):52-8. doi: https://doi.org/10.1016/j.jsps.2015.06.012.

1750. Al-Hashar A, Al-Zakwani I, Eriksson T, Sarakbi A, Al-Zadjali B, Al Mubaihsi S, et al. Impact of medication reconciliation and review and counselling, on adverse drug events and healthcare resource use. International journal of clinical pharmacy. 2018;40(5):1154-64. doi: 10.1007/s11096-018-0650-8. PubMed PMID: CN-01703593.

1751. Al-Hashar A, Al-Zakwani I, Eriksson T, Sarakbi A, Al-Zadjali B, Al Mubaihsi S, et al. Impact of medication reconciliation and review and counselling, on adverse drug events and healthcare resource use. International Journal of Clinical Pharmacy. 2018;40(5):1154-64. doi: 10.1007/s11096-018-0650-8. PubMed PMID: 132697448. Language: English. Entry Date: 20190313. Revision Date: 20191001. Publication Type: journal article.

1752. Al-Hassan MI. A survey on consumer need and opinion about the community pharmacists in Riyadh, Saudi Arabia. Journal of Medical Sciences. 2009;9(1):36-40. doi: 10.3923/jms.2009.36.40.

1753. Al-Hindi RR, Alharbi MG, Alotibi IA, Azhari SA, Ahmad A, Alseghayer MS, et al. MALDI-TOF MS-based identification and antibiotics profiling of <i>Salmonella</i> species isolated from retail chilled chicken in Saudi Arabia. Journal of King Saud University Science. 2023;35(5). doi: 10.1016/j.jksus.2023.102684. PubMed PMID: WOS:001055652300001.

1754. Al-Homrany MA, Irshaid YM. Pharmacoepidemiological study of prescription pattern of analgesics,

antipyretics, and nonsteroidal anti-inflammatory drugs at a tertiary health care center. Saudi Medical Journal. 2007;28(3):369-74. PubMed PMID: WOS:000246312100009.

1755. Al-Howiriny TA. Part I. Alkaloidal constituents of selected fractions of <i>Thalictrum angustifolium</i> L. and <i>Thalictrum wangii</i> Boivin (Ranunculaceae). Part II. A revision of the structure of thalprzewalskiinone. The synthesis of 2-methyl-6,7-dimethoxy-3'-methoxy-4'-hydroxyoxobenzylisoquinoline and 2-methyl-6,7-dimethoxy-3'-hydroxy-4'-methoxyoxobenzylisoquinoline [Ph.D.]. United States -- Pennsylvania: University of Pittsburgh; 2000.

1756. Al-Husseini A, Abu-Farha R, Van Hout MC, Wazaify M. Community pharmacists experience of pregabalin abuse and misuse: A quantitative study from Jordan. Journal of Substance Use. 2019;24(3):273-9. doi: 10.1080/14659891.2018.1554716. PubMed PMID: WOS:000460642300007.

1757. Al-Husseini A, Abu-Farha R, Wazaify M, Van Hout MC. Pregabalin dispensing patterns in Amman-Jordan: An observational study from community pharmacies. Saudi Pharmaceutical Journal. 2018;26(3):306-10. doi: 10.1016/j.jsps.2018.01.012. PubMed PMID: WOS:000427445500002.

1758. Al-Husseini A, Wazaify M, Van Hout MC. Pregabalin Misuse and Abuse in Jordan: a Qualitative Study of User Experiences. International Journal of Mental Health and Addiction. 2018;16(3):642-54. doi: 10.1007/s11469-017-9813-4.

1759. Al-Ibrahimy AS, Rabea IS. Evaluation of General Health Status in Diabetic Patients Using Short Form Health Survey (SF-36). Current Diabetes Reviews. 2023;19(9):135-40. doi: 10.2174/1573399816666200814133535.

1760. Al-Ibrahimya AS, Salah Rabea I. Evaluation of General Health Status in Diabetic Patients Using Short Form Health Survey (SF-36). Current diabetes reviews. 2020. doi: 10.2174/1573399816666200814133535. PubMed PMID: CN-02159293.

1761. Al-Jabr H, Twigg MJ, Katangwe-Chigamba T, Saadvandi R, Desborough JA. Patient feedback on hospital pharmacists' consultation skills: A feasibility study using the Interpersonal Skills Questionnaire (ISQ). PLoS ONE. 2022;17(7 July). doi: 10.1371/journal.pone.0268544.

1762. Al-Jabr H, Twigg MJ, Saadvandi RA, Desborough JA. Exploring what patients think when answering the Interpersonal Skills Questionnaire (ISQ): A 'think aloud' study. Research in Social and Administrative Pharmacy. 2019;15(5):619-22. doi: https://doi.org/10.1016/j.sapharm.2018.07.005.

1763. Al-Jamea R, Bossei A, Al Zhrani H, Bossei F, Faiz W, Alqurashi M, et al. Knowledge, Attitude and Practice of self-medication Among Undergraduate Medical Students in Jeddah city, Saudi Arabia. World Family Medicine. 2020;18(7):16-24. doi: 10.5742/mewfm.2020.93830. PubMed PMID: WOS:000548097500004.

1764. Al-Jazairi AS, Al-Ghamdi MA, Al-Suhaibani LK, Abu-Riash T, Assfoor Ab, Alsergani H, et al. The clinical and Practice Dilemma of Frequent Switching Among Generic Medications: Magnitude and Patient Safety Prospective. Hospital Pharmacy. 2023;58(5):476-83. doi: 10.1177/00185787231151858. PubMed PMID: 171848113.

1765. Al-Jedai A, Qaisi S, Al-Meman A. Pharmacy practice and the health care system in Saudi Arabia. The Canadian journal of hospital pharmacy. 2016;69(3):231.

1766. Al-Jedai AH, Khurshid F, Mayet AY, Al-Omar HA, Alghanem SS, Alsultan MS. Pharmacy practice in hospital settings in GCC countries: Prescribing and transcribing. Saudi Pharmaceutical Journal. 2021;29(9):1021-8. doi: 10.1016/j.jsps.2021.07.013. PubMed PMID: WOS:000701907400011.

1767. Al-Juffali L. Exploring medication safety problems in community pharmacy in saudi arabia [Ph.D.]. Scotland: University of Aberdeen (United Kingdom); 2017.

1768. Al-Jumaili AA, Al-Rekabi MD, Doucette W, Hussein AH, Abbas HK, Hussein FH. Factors influencing the degree of physician-pharmacist collaboration within Iraqi public healthcare settings. International Journal of Pharmacy Practice. 2017;25(6):411-7. PubMed PMID: 28181318.

1769. Al-Jumaili AA, Al-Rekabi MD, Sorofman B. Evaluation of instruments to assess health literacy in Arabic language among Iraqis. Research in Social and Administrative Pharmacy. 2015;11(6):803-13. doi: https://doi.org/10.1016/j.sapharm.2015.02.002.

1770. Al-Jumaili AA, Doucette WR. Comprehensive Literature Review of Factors Influencing Medication Safety in Nursing Homes: Using a Systems Model. Journal of the American Medical Directors Association. 2017;18(6):470-88. doi: 10.1016/j.jamda.2016.12.069. PubMed PMID: 123219042. Language: English. Entry Date: 20170605. Revision Date: 20170605. Publication Type: Article.

1771. Al-Jumaili AA, Elhiny R, Thomas D, Elbarbry F, Khdour M, Sherbeny F, et al. Factors impacting job satisfaction among pharmacists in the Arab world: A qualitative study. Saudi Pharmaceutical Journal. 2023;31(4):578-84. doi: 10.1016/j.jsps.2023.02.010.

1772. Al-Jumaili AA, Mohammed ZA, Yawuz MJ, Ahmed KK. Exploring Work Satisfaction and Characteristics of Iraqi Pharmacist Workforce. Brazilian Journal of Pharmaceutical Sciences. 2022;58. doi: 10.1590/s2175-97902022e19929. PubMed PMID: WOS:000828555000001.

1773. Al-Jumaili AA, Younus MM, Saleh MZ. The Epidemic of Substandard and Falsified Medications in Iraq: Evaluating the Effectiveness of National Pharmacovigilance Alerts to Community Pharmacies. Pharmaceutical Medicine. 2021;35(3):169-86. doi: 10.1007/s40290-021-00386-9.

1774. Al-Khalaileh W, Abu-Farha R, Wazaify M, Van Hout MC. Ophthalmic drug abuse: An observational study from community pharmacies. Research in Social and Administrative Pharmacy. 2019;15(8):943-8. doi: 10.1016/j.sapharm.2019.01.016.

1775. Al-Khalaileh W, Wazaify M, Van Hout MC. The Misuse and Abuse of Ophthalmic Preparations: a Scoping Review of Clinical Case Presentations and Extant Literature. International Journal of Mental Health and Addiction. 2018;16(4):1055-84. doi: 10.1007/s11469-017-9868-2.

1776. Al-Khani S, Moharram A, Aljadhey H. Factors contributing to the identification and prevention of incorrect drug prescribing errors in outpatient setting. Saudi Pharmaceutical Journal. 2014;22(5):429-32. doi: https://doi.org/10.1016/j.jsps.2013.11.003.

1777. Al-Khatib A, Andreski M, Pudlo A, Doucette WR. An evaluation of community pharmacies' actions under value-based payment. Journal of the American Pharmacists Association. 2020;60(6):899-+. doi: 10.1016/j.japh.2020.06.014. PubMed PMID: WOS:000585761700084.

1778. Al-Khatib AA. Evaluating the Influence of Facilitation in Community Pharmacy Practice Transformation: Identifying the Success Factors2023.

1779. Al-Khatib SM, Pokorney SD, Al-Khalidi HR, Haynes K, Garcia C, Martin D, et al. Underuse of oral anticoagulants in privately insured patients with atrial fibrillation: a population being targeted by the IMplementation of a randomized controlled trial to imProve treatment with oral AntiCoagulanTs in patients with Atrial Fibrillation (IMPACT-AFib). American heart journal. 2020;229:110-7. doi: 10.1016/j.ahj.2020.07.012. PubMed PMID: CN-02208735.

1780. Al-Khatib SM, Pokorney SD, Al-Khalidi HR, Haynes K, Garcia C, Martin D, et al. Underuse of oral anticoagulants in privately insured patients with atrial fibrillation: A population being targeted by the IMplementation of a randomized controlled trial to imProve treatment with oral AntiCoagulanTs in patients with Atrial Fibrillation (IMPACT-AFib). American Heart Journal. 2020;229:110-7. doi: https://doi.org/10.1016/j.ahj.2020.07.012.

1781. Al-Marwani S. Climate change impact on the healthcare provided to patients. Bulletin of the National Research Centre. 2023;47(1):1-5. doi: 10.1186/s42269-023-01026-9. PubMed PMID: 163023026.

1782. Al-Mashhadany DA. Occurrence and antibiogram of Listeria monocytogenes Isolates from Retail Meat Shops at Erbil City, Kurdistan Region, Iraq. Italian Journal of Food Safety. 2019;8(4):194-8. doi: 10.4081/ijfs.2019.8451. PubMed PMID: WOS:000500944700004.

1783. Al-Masri D, Wilbur K, Elazzazy S, Hassan AA, Wilby KJ. Law, Culture, and Fear: A Qualitative Study of Health Professionals' Perceptions of Narcotic Use Related to Cancer Pain. Journal of Pain & Palliative Care Pharmacotherapy. 2020;34(2):55-62. PubMed PMID: 32091944.

1784. Al-Mohammad MHS, Dawood SAI, Al-Mousawi HGK. The impact of spraying humic acid and gibberellic acid on growth, yield and chemical constituents of calyces of Hibiscus sabdariffa L. Planta medica. 2016;82(5). doi: 10.1055/s-0036-1578679. PubMed PMID: CN-01294810.

1785. Al-Momany NH, Al-Bakri AG, Makahleh ZM, Wazaify MMB. Adherence to International Antimicrobial Prophylaxis Guidelines in Cardiac Surgery: A Jordanian Study Demonstrates Need for Quality Improvement. Journal of Managed Care Pharmacy. 2009;15(3):262-71. doi: 10.18553/jmcp.2009.15.3.262. PubMed PMID: WOS:000265180400004.

1786. Al-Muallem N, Al-Surimi KM. Job satisfaction, work commitment and intention to leave among pharmacists: A cross-sectional study. BMJ open. 2019;9(9):e024448.

1787. Al-Musawe L. Patterns of Polypharmacy and Potential Overtreatment in Elderly People With Type 2 Diabetes Mellitus Using Real-world Data [Ph.D.]. Portugal: Universidade de Lisboa (Portugal); 2021.

1788. Al-Nagar A. An exploration of consultation skills in community pharmacists [Ph.D.]. England: University of East Anglia (United Kingdom); 2014.

1789. Al-Naqshbande M. Development of a predictive Antibiotic Burden Index for Primary Immunodeficiency – an explorative study [Master's]. Sweden: Uppsala Universitet (Sweden); 2020.

1790. Al-Nemrawi NK, AbuAlSamen MM, Alzoubi KH. Awareness about nanotechnology and its applications in drug industry among pharmacy students. Currents in Pharmacy Teaching and Learning. 2020;12(3):274-80. doi: https://doi.org/10.1016/j.cptl.2019.12.003.

1791. Al-Obaidy SSM. Nanocarrier-Formulated Antimicrobials and Microfluidics-Based Screening Assays [Ph.D.]. England: University of Hull (United Kingdom); 2018.

1792. Al-Omar BA. Patients' expectations, satisfaction and future behavior in hospitals in Riyadh city. Saudi Medical Journal. 2000;21(7):655-65.

1793. Al-Omar HA, Al-Sultan MS, Abu-Auda HS. Prescribing of potentially inappropriate medications among the elderly population in an ambulatory care setting in a Saudi military hospital: Trend and cost. Geriatrics and Gerontology International. 2013;13(3):616-21. PubMed PMID: 52248310.

1794. Al-Omar HA, Arafah AM, Barakat JM, Almutairi RD, Khurshid F, Alsultan MS. The impact of

perceived organizational support and resilience on pharmacists' engagement in their stressful and competitive workplaces in Saudi Arabia. Saudi Pharmaceutical Journal. 2019;27(7):1044-52.

1795. Al-Omar HA, Khurshid F, Sayed SK, Alotaibi WH, Almutairi RM, Arafah AM, et al. Work-Related Stress: Implications for Physical and Psychological Health among Female Pharmacists Working in Saudi Arabia. Archives of Pharmacy Practice. 2023;14(4):104-13. doi: 10.51847/UjhShie6aw. PubMed PMID: WOS:001121814500003.

1796. Al-Omrani H, Marwah MK, Al-Whaib R, Mekkawy M, Shokr H. Patterns of Drug Utilization and Self-Medication Practices: A Cross Sectional Study. Pharmacy. 2023;11(6):183.

1797. Al-Oudah RA, Abu Farha R, Al Ali MM, Jaradaneh NS, Ibrahim MIM. Evaluation of Community Pharmacists' Professional Practice and Management of Patient's Respiratory Conditions. Journal of Pharmacy and Bioallied Sciences. 2021;13(2):212-9. doi: 10.4103/jpbs.JPBS_534_20. PubMed PMID: WOS:000658801800010.

1798. Al-Qatamin S. The evaluation and analysis of counterfeit pharmaceuticals within jordan [Ph.D.]. England: Coventry University (United Kingdom); 2012.

1799. Al-Qerem W, Bargooth A, Jarab A, Akour A, Abu Heshmeh S. Information sources, attitudes, and practices of Self-Medication by Jordanian patients: A cross-sectional study. Saudi Pharmaceutical Journal. 2023;31(4):482-91. doi: 10.1016/j.jsps.2023.01.015. PubMed PMID: WOS:000993190100001.

1800. Al-Qerem W, Jarrar YB, Al-Sheikh I, Elmaadani A. The prevalence of drug-drug interactions and polypharmacy among elderly patients in Jordan. Biomedical Research (India). 2018;29(12):2561-9. doi: 10.4066/biomedicalresearch.29-18-618.

1801. Al-Qudah RA, Al-Badriyeh D, Al-Ali FM, Altawalbeh SM, Basheti IA. Cost-benefit analysis of clinical pharmacist intervention in preventing adverse drug events in the general chronic diseases outpatients. Journal of evaluation in clinical practice. 2020;26(1):115-24. doi: 10.1111/jep.13209. PubMed PMID: CN-01959253.

1802. Al-Qudah RA, Bulatova NR, Obeidat NM, Basheti IA. Impact of home medication management review on medication adherence among Jordanian patients. Journal of pharmaceutical health services research. 2018;9(3):227-35. doi: 10.1111/jphs.12243. PubMed PMID: CN-01630024.

1803. Al-Qudah RA, Farha RA, Al Ali MM, Jaradaneh NS, Mohamed Ibrahim MI. Evaluation of community pharmacists? professional practice and management of patient?s respiratory conditions. Journal of pharmacy & bioallied sciences. 2021;13(2):212-9. doi: 10.4103/jpbs.JPBS_534_20. PubMed PMID: CN-02286680.

1804. Al-Quteimat O, Siddiqui M, Hussein L, Al Emleh H, Shamieh IED. Analysis of pharmacist interventions in adult COVID-19 patients admitted to a tertiary care hospital. Journal of Pharmacy Practice. 2023;36(3):572-8.

1805. Al-Quteimat OM, Al-Badaineh MA. Intraperitoneal chemotherapy: rationale, applications, and limitations. Journal of Oncology Pharmacy Practice. 2014;20(5):369-80. PubMed PMID: 24166789.

1806. Al-Ramahi R. Patterns and attitudes of self-medication practices and possible role of community pharmacists in Palestine. International Journal of Clinical Pharmacology & Therapeutics. 2013;51(7):562-7. PubMed PMID: 23587151.

1807. Al-Ramahi R. Adherence to medications and associated factors: A cross-sectional study among Palestinian hypertensive patients. Journal of Epidemiology and Global Health. 2015;5(2):125-32. doi:

10.1016/j.jegh.2014.05.005.

1808. Al-Ramahi R, Jaradat N, Adawi D. Use of herbal medicines during pregnancy in a group of Palestinian women. Journal of Ethnopharmacology. 2013;150(1):79-84. doi: https://doi.org/10.1016/j.jep.2013.07.041.

1809. Al-Ramahi R, Zaid AN, Kettana N, Sweileh W, Al-Jabi D. Attitudes of consumers and healthcare professionals towards the patient package inserts - a study in Palestine. Pharmacy Practice. 2012;10(1):57-63. doi: 10.4321/S1886-36552012000100010.

1810. Al-Rashed SA, Wright DJ, Roebuck N, Sunter W, Chrystyn H. The value of inpatient pharmaceutical counselling to elderly patients prior to discharge. British journal of clinical pharmacology. 2002;54(6):657-64. doi: 10.1046/j.1365-2125.2002.01707.x. PubMed PMID: CN-00412342.

1811. Al-Rubeaan K, Banah F, Alruwaily FG, Sheshah E, Alnaqeb D, AlQahtani AM, et al. Longitudinal assessment of the quality of life and patterns of antidiabetic medication use in patients with type 2 diabetes, Saudi Arabia perspective, DISCOVER study. Current Medical Research & Opinion. 2023;39(1):27-35. doi: 10.1080/03007995.2022.2144052. PubMed PMID: 161082422.

1812. Al-Rusasi A. Patient-Centeredness in Pharmaceutical Care. Jordan Journal of Pharmaceutical Sciences. 2023;16(2):451. doi: 10.35516/jjps.v16i2.1490.

1813. Al-Ruthia YS, Mansy W, Barasin M, Ghawaa YM, AlSultan M, Alsenaidy MA, et al. Shortage of psychotropic medications in community pharmacies in Saudi Arabia: Causes and solutions. Saudi Pharmaceutical Journal. 2017;25(5):744-9. doi: 10.1016/j.jsps.2016.10.013. PubMed PMID: WOS:000406021100010.

1814. Al-Saadi A, Haridass S, Nouri A. Knowledge, attitude, perception, and practices of generic substitution of community pharmacists in the north al-Batinah governorate of Oman: a pilot study. Palestinian Medical and Pharmaceutical Journal. 2023;8(2):15.

1815. Al-Saadi A, Haridass S, Nouri A. Knowledge, attitude, perception, and practices of generic substitution of community pharmacists in the north al-Batinah governorate of Oman: a pilot study. Palestinian Medical and Pharmaceutical Journal. 2023;8(2):1-16.

1816. Al-Saffar H, Aljazzar R, Al-Saffar A. Pharmacy Practice in COVID-19 from a Middle Eastern and African Perspective: A Narrative Review. Ibnosina Journal of Medicine and Biomedical Sciences. 2021;13(4):173-82. doi: 10.4103/ijmbs.ijmbs_58_21. PubMed PMID: WOS:000754522600004.

1817. Al-Saffar N, Abdulkareem A, Abdulhakeem A, Salah AQ, Heba M. Depressed patients' preferences for education about medications by pharmacists in Kuwait. Patient education and counseling. 2008;72(1):94-101. doi: 10.1016/j.pec.2008.01.027. PubMed PMID: CN-00647841.

1818. Al-Saffar N, Deshmukh AA, Carter P, Adib SM. Effect of information leaflets and counselling on antidepressant adherence: open randomised controlled trial in a psychiatric hospital in Kuwait. International journal of pharmacy practice. 2005;13(2):123-31. doi: 10.1211/0022357056181. PubMed PMID: CN-00569387.

1819. Al-Safi SA, Aboul-Enein FH, Aboul-Enein BH, Manuel B. Influence of family history and lifestyle on blood pressure and heart rate in young adults in Jordan. Public Health. 2006;120(11):1027-32. doi: https://doi.org/10.1016/j.puhe.2006.06.009.

1820. Al-Safi SA, Ayoub NM, Aboul-Enein FH. Perception and knowledge of a common skin disorder among university students in Jordan. Journal of Public Health. 2008;16(2):123-8. doi: 10.1007/

s10389-007-0141-1.

1821. Al-Saheli Z, Donthireddy V. Implementing Quality Improvement to Morbidity and Mortality Conferences: Focusing on Action Plans By Closing the Loops with Timely Follow Ups. Blood. 2023;142:7194. doi: https://doi.org/10.1182/blood-2023-178609.

1822. Al-Saleh H, Al-Houtan T, Al-Odaill K, Al-Mutairi B, Al-Muaybid M, Al-Falah T, et al. Role of community pharmacists in providing oral health advice in the Eastern province of Saudi Arabia. The Saudi Dental Journal. 2017;29(3):123-8. doi: https://doi.org/10.1016/j.sdentj.2017.03.004.

1823. Al-Shami HA, Abubakar U, Hussein MSE, Hussin HFA, Al-Shami SA. Awareness, practices and perceptions of community pharmacists towards antimicrobial resistance and antimicrobial stewardship in Libya: a cross-sectional study. Journal of Pharmaceutical Policy and Practice. 2023;16(1). doi: 10.1186/s40545-023-00555-y.

1824. Al-Shaqha WMS. The role of clinical pharmacy in integrated healthcare delivery [Ph.D.]. England: University of Bradford (United Kingdom); 1999.

1825. Al-Sharayri MG, Aljbori TM, Migdadi QM, Al-Omoush MB, Jaarah AR. Do insulin cartridges really provide a lower risk of potential diabetes complications than traditional vials? Saudi Pharmaceutical Journal. 2014;22(4):373-5. doi: https://doi.org/10.1016/j.jsps.2013.12.008.

1826. Al-Shareef F, Abu El-Asrar S, Al-Bakr L, Al-Amro M, Alqahtani F, Aleanizy F, et al. Investigating the disposal of expired and unused medication in Riyadh, Saudi Arabia: a cross-sectional study. International Journal of Clinical Pharmacy. 2016;38(4):822-8. doi: 10.1007/s11096-016-0287-4. PubMed PMID: WOS:000379162900015.

1827. Al-Shehade SA. Prevalence and Determinants of Self-medication Practice Among Jordanian Households and Pharmacy Students [M.S.]. Jordan: University of Petra (Jordan); 2019.

1828. Al-Suhaimat R, Al-Halaseh LK, Al-Samydai A. Community pharmacists' perception and role in early diagnosis and health promotion of celiac disease. Journal of Pharmaceutical Health Services Research. 2024;15(1). doi: 10.1093/jphsr/rmad048. PubMed PMID: WOS:001110494800001.

1829. Al-Sulaiti F, Fares H, Awaisu A, Kheir N. Continuing Professional Development Needs of Community Pharmacists in Qatar: A Mixed-Methods Approach. International Quarterly of Community Health Education. 2021;41(3):285-92. PubMed PMID: 32487004.

1830. Al-Surimi K, Alwabel AM, Bawazir A, Shaheen NA. Road towards promoting patient safety practices among hospital pharmacists: Hospital-based baseline patient safety culture assessment cross-sectional survey. Medicine. 2021;100(2):e23670. PubMed PMID: 33466122.

1831. Al-Taani GM, Ayoub NM. A baseline survey of community pharmacies' workforce, premises, services and satisfaction with medical practitioners in Jordan. International Journal of Clinical Practice. 2021;75(9). doi: 10.1111/ijcp.14487. PubMed PMID: WOS:000664755800001.

1832. Al-Taani GM, Ayoub NM. Community pharmacists’ routine provision of drug-related problem-reduction services. PLoS ONE. 2022;17(5 May). doi: 10.1371/journal.pone.0267379.

1833. Al-Taie A. Reported patients' attitudes and practices for knowledge of prescribed medications with chronic disease conditions: A cross-sectional study. Biomedical and Biotechnology Research Journal. 2020;4(4):349-54. doi: 10.4103/bbrj.bbrj_174_20. PubMed PMID: WOS:000642265000016.

1834. Al-Taie A. Reported Patients' Practices, Knowledge and Attitudes Regarding Common Cold in the Community: A Cross-sectional Study. Advances in Human Biology. 2021;11(2):157-62. doi: 10.4103/aihb.aihb_142_20. PubMed PMID: WOS:000762240000006.

1835. Al-Taie A, Büyük AŞ, Sardas S. Considerations into pharmacogenomics of COVID-19 pharmacotherapy: Hope, hype and reality. Pulmonary Pharmacology & Therapeutics. 2022;77:102172. doi: https://doi.org/10.1016/j.pupt.2022.102172.

1836. Al-Taie A, Hussein AN, Albasry Z. Prescription pattern of non-steroidal anti-inflammatory drugs (nsaids) among community patients with musculoskeletal and co-morbid conditions: A crosssectional study from an iraqi province. Tropical Journal of Pharmaceutical Research. 2021;20(2):203-10. doi: 10.4314/tjpr.v20i1.29.

1837. Al-Taie A, Izzettin FV, Sancar M, Köseoğlu A. Impact of clinical pharmacy recommendations and patient counselling program among patients with diabetes and cancer in outpatient oncology setting. European journal of cancer care. 2020;29(5):e13261. doi: 10.1111/ecc.13261. PubMed PMID: CN-02138852.

1838. Al-Taie A, Yilmaz ZK. Evaluation of online counselling services based on Turkish web-based pharmacy care setting: A retrospective observational study. International Journal of Clinical Practice. 2021;75(3). doi: 10.1111/ijcp.13726.

1839. Al-Taie A, Yilmaz ZK, Dahman H, Yardimci T. Insights into disease and pharmacotherapy knowledge of Alzheimer’s disease among community pharmacists: a cross-sectional study. Current Medical Research and Opinion. 2022;38(12):2209-17. doi: 10.1080/03007995.2022.2129802.

1840. Al-Tameemi D, Al-Tukmagi H. The application of pharmaceutical care program on patients with dyslipidemia in iraqi community pharmacy. International journal of pharmaceutical sciences review and research. 2017;47(1):45-51. PubMed PMID: CN-01442649.

1841. Al-Tameemi NK, Sarriff A. The awareness of pharmacists regarding Medication Therapy Managment and their willingness to apply MTM in the future. Journal of Applied Pharmacy. 2018;10:45. PubMed PMID: 626579557.

1842. Al-Tameemi NK, Sarriff A. Towards Implementing Medication Therapy Management Service in Malaysia: Perceptions and Challenges. Value in Health. 2018;21(Supplement 3):S172. PubMed PMID: 2001400967.

1843. Al-Tameemi NK, Sarriff A. Knowledge, attitude and practice of pharmacists on medication therapy management: A survey in Hospital Pulau Pinang, Penang, Malaysia. Journal of Pharmaceutical Health Care and Sciences. 2019;5(1) (no pagination). PubMed PMID: 625841058.

1844. Al-Tannir M, Altannir Y, Altannir M, AlFayyad I. Community pharmacy sales of non-prescribed antibiotics in Riyadh, Saudi Arabia: a simulated patient study. International Journal of Clinical Pharmacy. 2020;42(2):341-6. doi: 10.1007/s11096-020-01033-0.

1845. Al-Taweel D, Al-Haqan A, Qabazard B, Abuzainah D, Koshy S, Alcienae F, et al. AVAILABILITY OF ANTIDOTES IN KUWAIT: A NATIONAL AUDIT. Journal of Emergency Medicine. 2020;58(2):305-12. doi: 10.1016/j.jemermed.2019.11.016. PubMed PMID: WOS:000531481300033.

1846. Al-Taweel D, Al-Haqan A, Qabazard B, Abuzainah D, Koshy S, Alqenae F, et al. Availability of Antidotes in Kuwait: A National Audit. Journal of Emergency Medicine. 2020;58(2):305-12. doi: 10.1016/j.jemermed.2019.11.016.

1847. Al-Taweel DM, Alsuwaidan M. A medication assessment tool to evaluate prescribers' adherence to evidence-based guidelines in bipolar disorder. International Journal of Clinical Pharmacy. 2017;39(4):897-905. PubMed PMID: 28653259.

1848. Al-Tawfiq JA, Momattin H, Hinedi K. Empiric Antibiotic Therapy in the Treatment of Community-acquired Pneumonia in a General Hospital in Saudi Arabia. Journal of Global Infectious Diseases. 2019;11(2):69-72. doi: 10.4103/jgid.jgid_84_18. PubMed PMID: WOS:000470794700005.

1849. Al-Tehewy M, Mohamed S, Ammar N. Patient safety culture among community pharmacists in Cairo. Journal of the Egyptian Public Health Association. 2023;98(1). doi: 10.1186/s42506-023-00136-6.

1850. Al-Theyab AA. Evaluation of pharmaceutical services provided by saudi arabia's ministry of interior and how they can be improved for optimisation of practice [Ph.D.]. Wales: Cardiff University (United Kingdom); 2003.

1851. Al-Wazaify M, Matowe L, Albsoul-Younes A, Al-Omran OA. Pharmacy education in jordan, saudi arabia, and kuwait. American journal of pharmaceutical education. 2006;70(1).

1852. Al-Wazaify MMB. Appropriate use of non-prescription drugs [Ph.D.]. Northern Ireland: Queen's University Belfast (United Kingdom); 2003.

1853. Al-Worafi YM. The challenges of pharmacy education in yemen. American Journal of Pharmaceutical Education. 2014;78(8). doi: 10.5688/ajpe788146.

1854. Al-worafi YM. Dispensing errors observed by community pharmacy dispensers in IBB–YEMEN. Asian J Pharm Clin Res. 2018;11(11).

1855. Al-Worafi YM. Drug safety in Yemen. Drug Safety in Developing Countries: Elsevier; 2020. p. 391-405.

1856. Al-Worafi YM. Drug Safety in Developing Countries: Achievements and Challenges. 2020.

1857. Al-Worafi YM. Chapter 7 - Self-medication. In: Al-Worafi Y, editor. Drug Safety in Developing Countries: Academic Press; 2020. p. 73-86.

1858. Al-Worafi YM. Chapter 2 - Medications safety-related terminology. In: Al-Worafi Y, editor. Drug Safety in Developing Countries: Academic Press; 2020. p. 7-19.

1859. Al-Worafi YM. Chapter 6 - Medication errors. In: Al-Worafi Y, editor. Drug Safety in Developing Countries: Academic Press; 2020. p. 59-71.

1860. Al-Worafi YM. 21 - Antibiotics case studies: Community pharmacies and primary care and public. In: Al-Worafi YM, editor. Clinical Case Studies on Medication Safety: Academic Press; 2023. p. 447-59.

1861. Al-Worafi YM. Antibiotics case studies: Community pharmacies and primary care and public. Clinical Case Studies on Medication Safety2023. p. 447-59.

1862. Al-Worafi YM. 21 - Antibiotics case studies: Community pharmacies and primary care and public. In: Al-Worafi YM, editor. Clinical Case Studies on Medication Safety: Academic Press; 2023. p. 447-59.

1863. Al-Worafi YM, Al-Moraya IS, Alqahtani HAM, Alnefaie MSM. 14 - Medications misuse and abuse case studies. In: Al-Worafi YM, editor. Clinical Case Studies on Medication Safety: Academic Press; 2023. p. 313-32.

1864. Al-Worafi YM, Al-Moraya IS, Alqahtani HAM, Alnefaie MSM. 14 - Medications misuse and abuse case studies. In: Al-Worafi YM, editor. Clinical Case Studies on Medication Safety: Academic Press; 2023. p. 313-32.

1865. Al-Worafi YM, Alseragi WM, Mahmoud MA. Competency of metered-dose inhaler use among community pharmacy dispensers in Ibb, Yemen: A simulation method study. Latin American Journal of Pharmacy. 2019;38(3):489-94.

1866. Al-Worafi YM, Kassab YW, Alseragi WM, Almutairi MS, Ahmed A, Ming LC, et al. Pharmacovigilance and adverse drug reaction reporting: A perspective of community pharmacists and pharmacy technicians in Sana'a, Yemen. Therapeutics and Clinical Risk Management. 2017;13:1175-81. doi: 10.2147/TCRM.S140674.

1867. Al-Worafi YM, Patel RP, Zaidi STR, Alseragi WM, Almutairi MS, Alkhoshaiban AS, et al. Completeness and Legibility of Handwritten Prescriptions in Sana'a, Yemen. Medical Principles & Practice. 2018;27(3):1-3. doi: 10.1159/000487307. PubMed PMID: 130596357. Language: English. Entry Date: 20181128. Revision Date: 20181128. Publication Type: journal article. Journal Subset: Biomedical.

1868. Al-Worafi YM, Patel RP, Zaidi STR, Alseragi WM, Almutairi MS, Alkhoshaiban AS, et al. Completeness and Legibility of Handwritten Prescriptions in Sana'a, Yemen. Medical Principles and Practice. 2018;27(3):290-2. doi: 10.1159/000487307. PubMed PMID: WOS:000438866800014.

1869. Al-Yahya KM. An evaluation of the impact of pharmacist intervention on clinical and patient outcomes in the management of diabetes mellitus in Saudi Arabia [Ph.D.]. Wales: The University of Wales College of Cardiff (United Kingdom); 2004.

1870. Al-Yousef HM, Alghadir AH, Iqbal A. Knowledge, attitude and dispensing practice of the pharmacists related to complementary alternative medicines in the Riyadh region of Saudi Arabia: a cross-sectional descriptive study. BMC Health Services Research. 2022;22(1). doi: 10.1186/s12913-022-08932-0.

1871. Al-Zakwani IS. Effect of higher prescription co -payments on clinical outcomes in patients with hypercholesterolemia in a managed care organization [Ph.D.]. United States -- Pennsylvania: University of the Sciences in Philadelphia; 2005.

1872. Al-Zidan RN, Saadallah AS, Abdulrazzaq GM. The public health dilemma of self-medication with antibiotics: The undergraduate students of the college of pharmacy in mosul as an example. International Journal of Research in Pharmaceutical Sciences. 2020;11(3):3743-51. doi: 10.26452/ijrps.v11i3.2542.

1873. Al-Shaqha WM, Zairi M. The role of quality in pharmaceutical care management. Managing Service Quality: An International Journal. 2001;11(1):32-9.

1874. Alaa Eddine N, Schreiber JB, Amin MEK. Translation and validation of the medication management patient satisfaction survey: The Lebanese Arabic version. Frontiers in Pharmacology. 2023;14:997103. doi: https://dx.doi.org/10.3389/fphar.2023.997103. PubMed PMID: 2021686032.

1875. Alabbadi I, Hammad EA. The impact of generic entry and price competition in Jordanian public health sector. Journal of Generic Medicines. 2014;11(3-4):123-8. doi: 10.1177/1741134315592945.

1876. Alabbas SA, Jiang R, Au A, Vu M, Moore VR, Cunningham FE, et al. Comparison of hospitalization costs for the same adverse reaction associated with different medications. American Journal of Health-System Pharmacy. 2023;80(12):750-5. doi: 10.1093/ajhp/zxad060. PubMed PMID: 164199119.

1877. Alabbasi A, Cohen S, Green M, Brammli S, Preis M, Khalaily R. P-143: breakthrough – a designated

pharmacist in the hemato-oncology department improves both quality and sequence of drug treatment and reduces ADR in Multiple Myeloma patients during Covid-19 pandemic. Clinical lymphoma, myeloma & leukemia. 2021;21:S113-S4. doi: 10.1016/S2152-2650(21)02270-9. PubMed PMID: CN-02342751.

1878. Alabi KO, Joshua F, Akinwumi AI, Adje DU, Awodele O. Perception of community pharmacists on abuse of psychotropic medications among the consumers. Tropical Journal of Pharmaceutical Research. 2022;21(12):2721-9. doi: 10.4314/tjpr.v21i12.30.

1879. Alabid A, Ibrahim MIM, Hassali MA, Palaian S. An Exploratory Study of Consumers' Social Behavior towards Role of Malaysian Community Pharmacists in Providing Healthcare. Journal of Pharmaceutical Research International. 2020;32(25):37-51. doi: 10.9734/JPRI/2020/v32i2530822. PubMed PMID: WOS:000581934900005.

1880. Alabkal RM, Medlinskiene K, Silcock J, Graham A. Impact of Pharmacist-Led Interventions to Improve Clinical Outcomes for Adults With Type 2 Diabetes at Risk of Developing Cardiovascular Disease: A Systematic Review and Meta-analysis. Journal of Pharmacy Practice. doi: 10.1177/08971900211064459. PubMed PMID: WOS:000799708200001.

1881. Alacreu M, Pardo J, Azorín M, Climent MT, Gasull V, Moreno L. Importance of increasing modifiable risk factors knowledge on Alzheimer's disease among community pharmacists and general practitioners in Spain. Frontiers in Pharmacology. 2019;10(JULY). doi: 10.3389/fphar.2019.00860.

1882. Alagha HZ. DISPENSING ANTIBIOTICS WITHOUT PRESCRIPTION IN THE ARAB WORLD: A NARRATIVE REVIEW OF THE PREVALENCE, APPROPRIATENESS, FACILITATORS AND PREVENTIVE INTERVENTIONS. Bulletin of Pharmaceutical Sciences Assiut. 2023;46(1):399-420. doi: https://dx.doi.org/10.21608/BFSA.2023.301131. PubMed PMID: 2025398871.

1883. Alagha HZ, Badary OA, Ibrahim HM, Sabri NA. Reducing prescribing errors in the paediatric intensive care unit: an experience from Egypt. Acta Paediatrica. 2011;100(10):e169-74. PubMed PMID: 21418100.

1884. Alagha HZI, Al Telbani MJ. Investigating antibiotic use in Gaza Strip hospitals: A retrospective cross-sectional analysis. Journal of Infection in Developing Countries. 2022;16(11):1739-+. doi: 10.3855/jidc.16985. PubMed PMID: WOS:000891836000010.

1885. Alahmad MM, Majed I, Sikh N, Alahmad K. The impact of community-pharmacist-led medication reconciliation process: Pharmacist-patient-centered medication reconciliation. Journal of Pharmacy and Bioallied Sciences. 2020;12(2):177-82. doi: 10.4103/jpbs.JPBS_55_20.

1886. Alahmad MM, Sadeq A, Zainalabdin S, Abdelsabour M, Muhaisen A, Fathelrahman A, et al. Physicians' attitudes toward expanding the role of community pharmacists in Al Ain, United Arab Emirates. Journal of Pharmaceutical Health Services Research. 2023;14(2):123-30. doi: 10.1093/jphsr/rmad030.

1887. Alahmadi YM. Perceptions and trends in the use of community pharmacy services: A cross sectional community based study from Saudi Arabia. Latin American Journal of Pharmacy. 2020;39(4):679-85.

1888. Alajmi AM, Alamoudi AA, Halwani AA, Almangour TA, Almozain NH, Al-Jedai A, et al. Antimicrobial Resistance Awareness, Antibiotics Prescription Errors and Dispensing Patterns by Community Pharmacists in Saudi Arabia. Journal of Infection and Public Health. 2023;16(1):34-41. doi: https://dx.doi.org/10.1016/j.jiph.2022.11.026. PubMed PMID: 2021389112.

1889. Alajmi AM, Alamoudi AA, Halwani AA, Almangour TA, Almozain NH, Al-Jedai A, et al. Antimicrobial Resistance Awareness, Antibiotics Prescription Errors and Dispensing Patterns by Community

Pharmacists in Saudi Arabia. Journal of Infection and Public Health. 2023;16(1):34-41. doi: https://doi.org/10.1016/j.jiph.2022.11.026.

1890. Alakhali KM. ASSESSMENT OF SELF MEDICATION PATTERN OF PHARMACIST IN ASEER REGION, SAUDI ARABIA. Bulletin of Pharmaceutical Sciences Assiut. 2016;39(1):87-93. doi: 10.21608/BFSA.2016.62611.

1891. Alakhali KM, Ansari SMA, Alavudeen SS, Ivhan NA. Medication errors at the outpatient pharmacy in a hospital in Aseer region, Kingdom of Saudi Arabia. Atencion Farmaceutica. 2014;16(2):144-6. PubMed PMID: 373286054.

1892. Alaki SM. Middle ear infections, systemic antibiotics and their relationship with early childhood caries [Dr.P.H.]. United States -- Michigan: University of Michigan, School of Public Health; 2006.

1893. Alalawneh M, Berardi A, Nuaimi N, Basheti IA. Improving Syrian refugees' knowledge of medications and adherence following a randomized control trial assessing the effect of a medication management review service. PloS one. 2022;17(10):e0276304. doi: 10.1371/journal.pone.0276304. PubMed PMID: CN-02474405.

1894. Alali KYAA. Development of National Drug Policy in the State of Kuwait [Ph.D.]. England: University of Bradford (United Kingdom); 2016.

1895. Alali N, Mahmoud MH, Alharbi MA, Ghazal SN. Appropriateness of dabigatran dosing in patients with nonvalvular atrial fibrillation (NVAF): A retrospective study conducted in a tertiary care university hospital in the eastern province of Saudi Arabia. Journal of the Saudi Heart Association. 2019;31(3):130-4. doi: https://doi.org/10.1016/j.jsha.2019.05.002.

1896. Alam AB, Lutsey PL, Chen LY, MacLehose RF, Shao IY, Alonso A. Risk Factors for Dementia in Patients With Atrial Fibrillation. The American Journal of Cardiology. 2022;174:48-52. doi: https://doi.org/10.1016/j.amjcard.2022.03.029.

1897. Alam GM, Shahjamal MM, Al-Amin AQ, Azam MN. State of pharmacy education in Bangladesh. Tropical Journal of Pharmaceutical Research. 2013;12(6):1107-12. doi: 10.4314/tjpr.v12i6.36.

1898. Alam K, Wagle S, Gupta AK, Sarraf DP, Karko B. Exploring community pharmacists' and drug retailers' knowledge and perception regarding generic medicine in Dharan, Nepal. Journal of Karnali Education Health Research. 2022;1(1):15-22.

1899. Alam M, Saleem Z, Haseeb A, Qamar MU, Sheikh A, Abuhussain SSA, et al. Tackling antimicrobial resistance in primary care facilities across Pakistan: Current challenges and implications for the future. Journal of Infection and Public Health. 2023;16:97-110. doi: 10.1016/j.jiph.2023.10.046. PubMed PMID: WOS:001133767800014.

1900. Alam M, Saleem Z, Haseeb A, Qamar MU, Sheikh A, Almarzoky Abuhussain SS, et al. Tackling antimicrobial resistance in primary care facilities across Pakistan: Current challenges and implications for the future. Journal of Infection and Public Health. 2023;16(Supplement 1):97-110. doi: https://dx.doi.org/10.1016/j.jiph.2023.10.046. PubMed PMID: 2028379334.

1901. Alam S, Khan F. Virtual screening, Docking, ADMET and System Pharmacology studies on Garcinia caged Xanthone derivatives for Anticancer activity. Scientific reports. 2018;8(1):1-16.

1902. Alam S, Osama M, Iqbal F, Sawar I. Reducing pharmacy patient waiting time. International journal of health care quality assurance. 2018;31(7):834-44.

1903. Alam SM, Larson M, Srinivasan P, Genz N, Fleer R, Sardiu M, et al. Evaluation of sarcopenia in patients receiving intravesical Bacillus Calmette-Guérin for non-muscle invasive bladder cancer. Urologic Oncology: Seminars and Original Investigations. 2023;41(10):431.e15-.e20. doi: https://doi.org/10.1016/j.urolonc.2023.05.021.

1904. Alam T, Ayaz S, Rehman H, Ali T, Sana A, Alam S, et al. Post marketing in vitro pharmaceutical equivalence of cefaclor 100 mg capsule available in retail pharmacies of Karachi, Pakistan. Latin American Journal of Pharmacy. 2019;38(7):1472-7.

1905. Alameddine M, Bou-Karroum K, Hijazi MA. A national study on the resilience of community pharmacists in Lebanon: a cross-sectional survey. Journal of Pharmaceutical Policy and Practice. 2022;15(1):1-10.

1906. Alameddine M, Bou-Karroum K, Kassas S, Hijazi MA. A profession in danger: Stakeholders' perspectives on supporting the pharmacy profession in Lebanon. PLoS ONE. 2020;15(11 November). doi: 10.1371/journal.pone.0242213.

1907. Alameddine M, Karroum KB, Hijazi MA. Upscaling the pharmacy profession in Lebanon: workforce distribution and key improvement opportunities. Human Resources for Health. 2019;17. doi: 10.1186/s12960-019-0386-0. PubMed PMID: WOS:000472876200002.

1908. Alamelu R, Amudha R, Cresenta Shakila Motha L, Nalini R. Online pharma retail is a promising/unpromising avenue: An Indian context. Asian Journal of Pharmaceutical and Clinical Research. 2016;9(2):26-9.

1909. Alamin Alabid AHM, Mohamed Ibrahim MI, Hassali MA, Palaian S. Community pharmacists' awareness toward their roles in healthcare and interaction with general practitioners: a cross-sectional study. Journal of pharmacy & bioallied sciences. 2021;13(2):220-9. doi: 10.4103/jpbs.JPBS_680_20. PubMed PMID: CN-02286681.

1910. Alamri SH, Kennedy CC, Marr S, Lohfeld L, Skidmore CJ, Papaioannou A. Strategies to overcome barriers to implementing osteoporosis and fracture prevention guidelines in long-term care: a qualitative analysis of action plans suggested by front line staff in Ontario, Canada. BMC geriatrics. 2015;15:94. doi: 10.1186/s12877-015-0099-8. PubMed PMID: CN-01170542.

1911. Alamyar S, Azzi E, Srour-Alphonse P, House R, Cvetkovski B, Kritikos V, et al. Uncovering the Burden of Rhinitis in Patients Purchasing Nonprescription Short-Acting β-Agonist (SABA) in the Community. Pharmacy. 2023;11(4). doi: 10.3390/pharmacy11040115. PubMed PMID: WOS:001056933400001.

1912. Alanazi AS, Alfadl AA, Hussain AS. Pharmaceutical care in the community pharmacies of Saudi Arabia: present status and possibilities for improvement. Saudi Journal of Medicine & Medical Sciences. 2016;4(1):9.

1913. Alanazi AS, Awwad S, Khan TM, Asdaq SMB, Mohzari Y, Alanazi F, et al. Medication reconciliation on discharge in a tertiary care Riyadh Hospital: An observational study. PLoS ONE [Electronic Resource]. 2022;17(3):e0265042. PubMed PMID: 35290378.

1914. Alanazi AS, Shah S, Abbas G, Hussain M, Saleem A, Khurram H, et al. Assessing Patient Satisfaction with Community Pharmacy Services: A Large Regional Study at Punjab, Pakistan. Patient Preference and Adherence. 2023;17:13-22. doi: 10.2147/PPA.S389053.

1915. Alanazi AS, Shah S, Abbas G, Hussain M, Saleem A, Khurram H, et al. Assessing Patient Satisfaction

with Community Pharmacy Services: A Large Regional Study at Punjab, Pakistan. Patient Preference and Adherence. 2023;17:13-22. doi: 10.2147/PPA.S389053.

1916. Alanazi AS, Shah SH, Abbas G, Hussain M, Saleem A, Khurram H, et al. Assessing Patient Satisfaction with Community Pharmacy Services: A Large Regional Study at Punjab, Pakistan. Patient Preference and Adherence. 2023;17:13-22. doi: 10.2147/ppa.S389053. PubMed PMID: WOS:000910682400001.

1917. Alanazi AS, Shah SH, Abbas G, Hussain M, Saleem A, Khurram H, et al. Assessing Patient Satisfaction with Community Pharmacy Services: A Large Regional Study at Punjab, Pakistan. Patient Preference and Adherence. 2023;17:13-22. doi: 10.2147/ppa.S389053. PubMed PMID: WOS:000910682400001.

1918. Alanazi MF, Shahein MI, Alsharif HM, Alotaibi SM, Alanazi AO, Alanazi AO, et al. Impact of automated drug dispensing system on patient safety. Pharmacy Practice (1886-3655). 2022;20(4):1-11. doi: 10.18549/PharmPract.2022.4.2744. PubMed PMID: 160816318. Language: English. Entry Date: 20230118. Revision Date: 20230118. Publication Type: Article.

1919. Alanazi MQ, Al-Jeraisy MI, Salam M. Prevalence and predictors of antibiotic prescription errors in an emergency department, Central Saudi Arabia. Drug, healthcare and patient safety. 2015:103-11.

1920. Alanazi S, Dekhaela S, Obaidy S, Mutairi F, Majid K, Mufrij H, et al. Assessment of employee engagement in pharmaceutical care service at King Abdulaziz Medical City – Central region (KAMC): A cross-sectional study. Saudi Pharmaceutical Journal. 2023;31(5):765-72. doi: https://doi.org/10.1016/j.jsps.2023.03.017.

1921. Alanber MN, Alharbi NS, Khaled JM. Evaluation of multidrug-resistant Bacillus strains causing public health risks in powdered infant milk formulas. Journal of Infection and Public Health. 2020;13(10):1462-8. doi: https://doi.org/10.1016/j.jiph.2019.11.013.

1922. Alaniz ML. Alcohol availability and targeted advertising in racial ethnic minority communities. Alcohol Health & Research World. 1998;22(4):286-9. PubMed PMID: WOS:000080652400009.

1923. Alanzi TM. Impact of ChatGPT on Teleconsultants in Healthcare: Perceptions of Healthcare Experts in Saudi Arabia. Journal of Multidisciplinary Healthcare. 2023;16:2309-21. doi: https://dx.doi.org/10.2147/JMDH.S419847. PubMed PMID: 2025373105.

1924. Alaqeel S, Abanmy NO. Counselling practices in community pharmacies in Riyadh, Saudi Arabia: a cross-sectional study. BMC health services research. 2015;15:1-9.

1925. Alaqeel S, Al Obaidi N. Patient Evaluation of Medication Package Leaflets in Al Kharj City, Saudi Arabia. Therapeutic Innovation and Regulatory Science. 2017;51(1):45-50. doi: 10.1177/2168479016659320.

1926. Alarcón ACR, Muñoz PAV, Vilema ERE, Aguagallo CNT, Barros AAM, Torres CA. Evaluation of the pharmaceutical care service in four private pharmacies of Riobamba, Ecuador. Revista Cubana de Farmacia. 2019;52(2).

1927. Alassaad A, Bertilsson M, Gillespie U, Sundström J, Hammarlund-Udenaes M, Melhus H. The effects of pharmacist intervention on emergency department visits in patients 80 years and older: subgroup analyses by number of prescribed drugs and appropriate prescribing. PloS one. 2014;9(11):e111797. doi: 10.1371/journal.pone.0111797. PubMed PMID: CN-01112714.

1928. Alassaad A, Melhus H, Hammarlund-Udenaes M, Bertilsson M, Gillespie U, Sundström J. A tool for

prediction of risk of rehospitalisation and mortality in the hospitalised elderly: secondary analysis of clinical trial data. BMJ open. 2015;5(2):e007259. doi: 10.1136/bmjopen-2014-007259. PubMed PMID: CN-01110496.

1929. Alastalo N, Siitonen P, Jyrkkä J, Hämeen-Anttila K. The quality of non-prescription medicine counselling in Finnish pharmacies – a simulated patient study. Exploratory Research in Clinical and Social Pharmacy. 2023;11. doi: 10.1016/j.rcsop.2023.100304.

1930. Alastalo N, Siitonen P, Jyrkkä J, Hämeen-Anttila K. The quality of non-prescription medicine counselling in Finnish pharmacies - a simulated patient study. Exploratory Research in Clinical and Social Pharmacy. 2023;11. doi: 10.1016/j.rcsop.2023.100304. PubMed PMID: WOS:001133190700001.

1931. Alatawi YM, Kavookjian J, Ekong G, Alrayees MM. The association between health beliefs and medication adherence among patients with type 2 diabetes. Research in Social & Administrative Pharmacy. 2016;12(6):914-25. doi: 10.1016/j.sapharm.2015.11.006. PubMed PMID: WOS:000386105800008.

1932. Alauzet F, Blanc S, Montaudié I, Piccini-Bailly C, Berlioz-Baudoin M, Bégassat M, et al. Recommandations de l'Agence nationale de sécurité du médicament et des produits de santé (ANSM) concernant la prise en charge de la toux aiguë du nourrisson : impact sur les familles et les pharmacies. Archives de Pédiatrie. 2014;21(5):469-75. doi: https://doi.org/10.1016/j.arcped.2014.02.026.

1933. Alauzet F, Blanc S, Montaudie I, Piccini-Bailly C, Berlioz-Baudoin M, Begassat M, et al. [Acute cough in infants: impact on families and pharmacists of contraindications of the Agence nationale de securite du medicament et des produits de sante (ANSM)]. Archives de Pediatrie. 2014;21(5):469-75. PubMed PMID: 24726669.

1934. Alavi M, Raffa JD, Deans GD, Lai C, Krajden M, Dore GJ, et al. Continued low uptake of treatment for hepatitis C virus infection in a large community-based cohort of inner city residents. Liver International. 2014;34(8):1198-206. doi: 10.1111/liv.12370.

1935. Alawneh MA, Nuaimi N, Abu-Gharbieh E, Basheti IA. A randomized control trial assessing the effect of a pharmaceutical care service on Syrian refugees' quality of life and anxiety. Pharmacy practice. 2020;18(1):1744. doi: 10.18549/PharmPract.2020.1.1744. PubMed PMID: CN-02094251.

1936. Alawneh MAL, Nuaimi N, Abu-Gharbieh E, Basheti IA. A randomized control trial assessing the effect of a pharmaceutical care service on syrian refugeesâ€™ quality of life and anxiety. Pharmacy practice. 2020;18(1). doi: 10.18549/PharmPract.2020.1.1744. PubMed PMID: CN-02096269.

1937. Alayoubi F, Al Shaban A, Elnahal I. Emergency Mobile and Point of Care Anticoagulation Clinic During (COVID-19) Curfew by Pharmacists in Saudi Arabia. Research and Practice in Thrombosis and Haemostasis. 2023;7(Supplement 2):100872. doi: https://dx.doi.org/10.1016/j.rpth.2023.100872. PubMed PMID: 2028294907.

1938. AlAzmi A, AlHamdan H, Abualezz R, Bahadig F, Abonofal N, Osman M. Patients' Knowledge and Attitude toward the Disposal of Medications. Journal of Pharmaceutics. 2017;2017. doi: 10.1155/2017/8516741. PubMed PMID: WOS:000413025800001.

1939. Alba S, Dillip A, Hetzel MW, Mayumana I, Mshana C, Makemba A, et al. Improvements in access to malaria treatment in Tanzania following community, retail sector and health facility interventions -- a user perspective. Malaria Journal. 2010;9. doi: 10.1186/1475-2875-9-163. PubMed PMID: WOS:000282407900001.

1940. Alba S, Hetzel MW, Goodman C, Dillip A, Liana J, Mshinda H, et al. Improvements in access to

malaria treatment in Tanzania after switch to artemisinin combination therapy and the introduction of accredited drug dispensing outlets - A provider perspective. Malaria Journal. 2010;9(1). doi: 10.1186/1475-2875-9-164.

1941. Albabtain B, Hadi MA, Bawazeer G, Alqahtani A, Bahatheq A, Alhossan A, et al. Evaluation of a community pharmacy-based medication therapy management programme: a study protocol of a pilot randomized controlled trial with an embedded qualitative study. Saudi pharmaceutical journal. 2021;29(7):706-12. doi: 10.1016/j.jsps.2021.05.001. PubMed PMID: CN-02276415.

1942. Albadr Y, Khan TM. Factors influencing community pharmacist decision to dispense generic or branded medicines; Eastern Province, Alahsa, Saudi Arabia. Saudi Pharmaceutical Journal. 2015;23(2):143-6. doi: 10.1016/j.jsps.2014.07.002.

1943. Albahar KARK, Abdullah NACB. The role of pharmacy in crisis management and disaster response in United Arab Emirates. International Journal of Entrepreneurship. 2021;25(Special Issue 1).

1944. Albahari A, Schultz CH, editors. A qualitative analysis of the spontaneous volunteer response to the 2013 Sudan floods: Changing the paradigm. Prehospital and Disaster Medicine; 2017.

1945. Albahari A, Schultz CH. A Qualitative Analysis of the Spontaneous Volunteer Response to the 2013 Sudan Floods: Changing the Paradigm. Prehospital and Disaster Medicine. 2017;32(3):240-8. doi: 10.1017/s1049023x17000164. PubMed PMID: WOS:000402845900003.

1946. Albals D, Yehya A, Issa R, Fawadleh A. Retrospective assessment of acute poisoning incidents by nonpharmaceutical agents in Jordan: Data from Pharmacy One™ Poison Call Center, 2014 to 2018—Part I. Pharmacology Research and Perspectives. 2020;8(2). doi: 10.1002/prp2.582.

1947. Albals D, Yehya A, Issa R, Fawadleh A. Retrospective assessment of acute poisoning incidents by nonpharmaceutical agents in Jordan: Data from Pharmacy One (TM) Poison Call Center, 2014 to 2018-Part I. Pharmacology Research & Perspectives. 2020;8(2). doi: 10.1002/prp2.582. PubMed PMID: WOS:000530342100025.

1948. Albanese NP, Rouse MJ, Schlaifer M, Pharmacy CoCi. Scope of contemporary pharmacy practice: roles, responsibilities, and functions of pharmacists and pharmacy technicians. Journal of the American Pharmacists Association. 2010;50(2):e35-e69.

1949. Albanna YKY. Awareness and Knowledge of Health Care Professionals and Obese Adults of Complementary and Alternative Medicine Used to Manage Obesity in Jordan [M.S.]. Jordan: University of Petra (Jordan); 2015.

1950. Albano ME, Bostwick JR, Ward KM, Fluent T, Choe HM. Discrepancies Identified Through a Telephone-Based, Student-Led Initiative for Medication Reconciliation in Ambulatory Psychiatry. Journal of Pharmacy Practice. 2018;31(3):304-11. doi: 10.1177/0897190017715391. PubMed PMID: 129686788. Language: English. Entry Date: 20180525. Revision Date: 20180525. Publication Type: Article.

1951. Albarrak AI, Al Rashidi EA, Fatani RK, Al Ageel SI, Mohammed R. Assessment of legibility and completeness of handwritten and electronic prescriptions. Saudi Pharmaceutical Journal. 2014;22(6):522-7. doi: https://doi.org/10.1016/j.jsps.2014.02.013.

1952. Albasri A. The Role of Community Pharmacists in the Management of Hypertension [Ph.D.]. England: University of Oxford (United Kingdom); 2020.

1953. Albasri A, Clark CE, Omboni S, McDonagh STJ, McManus RJ, Sheppard JP. Effective detection and

management of hypertension through community pharmacy in England. Pharmaceutical Journal. 2020;304(7935). doi: 10.1211/PJ.2020.20207532.

1954. Albasri A, O'Sullivan JW, Roberts NW, Prinjha S, McManus RJ, Sheppard JP. A comparison of blood pressure in community pharmacies with ambulatory, home and general practitioner office readings: Systematic review and meta-analysis. Journal of Hypertension. 2017;35(10):1919-28. doi: 10.1097/HJH.0000000000001443.

1955. Albasri A, Prinjha S, McManus RJ, Sheppard JP. Hypertension referrals from community pharmacy to general practice: Multivariate logistic regression analysis of 131 419 patients. British Journal of General Practice. 2018;68(673):e541-e50. doi: 10.3399/bjgp18X697925.

1956. Albasri A, Van Den Bruel A, Hayward G, McManus RJ, Sheppard JP, Verbakel JYJ. Impact of point-of-care tests in community pharmacies: A systematic review and meta-analysis. BMJ Open. 2020;10(5). doi: 10.1136/bmjopen-2019-034298.

1957. Albasry Z, Nafea LT. Study the perceptions, effectiveness and adherence pattern of anti-diabetic drugs prescribed for type 2 dm patients in some iraqi hospitals, specialized diabetic centers and private pharmacies. 20160737/5609. International Journal of Pharmaceutical Research. 2020;12(4):1281-90. doi: 10.31838/ijpr/2020.12.04.188.

1958. Albassam A, Almohammed H, Alhujaili M, Koshy S, Awad A. Perspectives of primary care physicians and pharmacists on interprofessional collaboration in Kuwait: a quantitative study. PloS one. 2020;15(7):e0236114. doi: 10.1371/journal.pone.0236114. PubMed PMID: CN-02138793.

1959. Albassam A, Awad A. Community pharmacists' services for women during pregnancy and breast feeding in Kuwait: a cross-sectional study. BMJ open. 2018;8(1):e018980.

1960. Albassam AA, Al-Saikhan FI. Pharmacists' Knowledge, Perception and Attitude towards Certain Medication Administration Time. Journal of Pharmaceutical Research International. 2020;32(21):79-85. doi: 10.9734/JPRI/2020/v32i2130757. PubMed PMID: WOS:000569119500010.

1961. Albassam AA, Alshehri F, Alarfaj M, Alharajin M, Althemery A, Alfaifi AA, et al. Knowledge, attitudes, and practices of antibiotics self-use among healthcare providers in Saudi Arabia. Latin American Journal of Pharmacy. 2021;40(3):586-93.

1962. Albassam AA, Iqbal MS, Ain MR, Alotaibi A, Althemery A, Alfaifi A. A cross-sectional assessment of health-related quality of life among healthcare providers in Alkharj, Saudi Arabia. Tropical Journal of Pharmaceutical Research. 2020;19(10):2171-7. doi: 10.4314/tjpr.v19i10.22. PubMed PMID: WOS:000585079600022.

1963. Albassam AA, Shahid Iqbal M, Ruhal Ain M, Alotaibi A, Althemery A, Alfaifi A. A cross-sectional assessment of health-related quality of life among healthcare providers in Alkharj, Saudi Arabia. Tropical Journal of Pharmaceutical Research. 2020;19(10):2171-7. doi: 10.4314/tjpr.v19i10.22.

1964. Albaum JM, Levesque LE, Gershon AS, Liu G, Cadarette SM. Glucocorticoid-induced osteoporosis management among seniors, by year, sex, and indication, 1996-2012. Osteoporosis International. 2015;26(12):2845-52. doi: 10.1007/s00198-015-3200-4. PubMed PMID: WOS:000365515600014.

1965. Albawani SM, Bin Hassan Y, Abd-Aziz N, Gnanasan S. Self-medication with antibiotics in Sana'a City, Yemen. Tropical Journal of Pharmaceutical Research. 2017;16(5):1195-9. doi: 10.4314/tjpr.v16i5.30. PubMed PMID: WOS:000406946600030.

1966. Albawani SM, Hassan YB, Abd-Aziz N, Gnanasan S. Self-medication with antibiotics in sana'a city, yemen. Tropical Journal of Pharmaceutical Research. 2017;16(5):1195-9. doi: 10.4314/tjpr.v16i5.30.

1967. Albawani SM, Hassan YB, Abd-Aziz N, Gnansan S. Self-medication practice among consumers in Sana'a City. International Journal of Pharmacy and Pharmaceutical Sciences. 2016;8(10):119-24. doi: 10.22159/ijpps.2016v8i10.12748.

1968. Albayrak A, Basgut B, Bikmaz GA, Karahalil B. Clinical pharmacist assessment of drug-related problems among intensive care unit patients in a Turkish university hospital. BMC Health Services Research. 2022;22(1):79. PubMed PMID: 35033079.

1969. Albayrak A, Demirbas H. Evaluation of potentially inappropriate medications use and medication complexity in elderly patients applying to community pharmacy in Turkey. BMC geriatrics. 2023;23(1):655. doi: https://dx.doi.org/10.1186/s12877-023-04381-4. PubMed PMID: 642500480.

1970. Albayrak A, Ozger HS, Basgut B, Aygencel Bikmaz G, Karahalil B. Impact of clinical pharmacist's interventions on clinical outcomes in appropriate use of colistin: a prospective pre-post intervention study. Journal of Chemotherapy. 2023;35(8):712-20. PubMed PMID: 37021512.

1971. Alberola Gómez-Escolar C, Vitores Picón P, Medina Bustillo B, Gaztelut San Felipe J, Sabadías López-Cañadilla C, Artola Manéndez S, et al. Tutelage practices in community pharmacy: Description of an experience. Pharmaceutical Care Espana. 2006;8(2):84-7.

1972. Albert E. The Teepokana Paradigm: A future for general practice and primary healthcare? Medical Journal of Australia. 2002;177(2):119-20. doi: 10.5694/j.1326-5377.2002.tb04689.x.

1973. Albert M, Baudouin A, Cerfon MA, Herledan C, Kimbidima R, Larbre V, et al. CLINICAL IMPACT OF PHARMACEUTICAL CARE IN A MULTIDISCIPLINARY PROGRAM TO SECURE MEDICATION THERAPY IN LYMPHOMA PATIENTS TREATED BY IMMUNOCHEMOTHERAPY. European Journal of Oncology Pharmacy. 2023;6(1 Supplement):19. doi: https://dx.doi.org/10.1097/OP9.0000000000000042. PubMed PMID: 640338928.

1974. Albert N, Buysman E, Chang C, Swindle J. Lower Hospitalization and Total Healthcare Costs Among Patients with Heart Failure When Treated with Sacubitril/Valsartan Versus Angiotensin-Converting Enzyme Inhibitor or Angiotensin-Receptor Blocker: A Retrospective Study of Managed Care Claims. Heart & Lung. 2020;49(2):211. doi: https://doi.org/10.1016/j.hrtlng.2020.02.012.

1975. Albert S, Brason FW, Sanford CK, Dasgupta N, Graham J, Lovette B. Project Lazarus: community-based overdose prevention in rural North Carolina. Pain medicine. 2011;12(suppl_2):S77-S85.

1976. Albert SB. Ask a question and perhaps you'll save a life. New York State Dental Journal. 1998;64(6):4-5. PubMed PMID: 9707975.

1977. Albert V, Lanz M, Imanidis G, Hersberger KE, Arnet I. Stability of medicines after repackaging into multicompartment compliance aids: eight criteria for detection of visual alteration. Drugs and Therapy Perspectives. 2017;33(10):487-96. doi: 10.1007/s40267-017-0431-9.

1978. Albert-Lorinez E, editor Need to Change the Optics in the Prevention of Addictive Behaviors. The Role of Local Communities in Prevention of Smoking. Lumen 3rd International Conference on Logos Universality Mentality Education Novelty (LUMEN) - Current Paradigms in Social Sciences; 2013 Apr 10-13; Iasi, ROMANIA2013.

1979. Alberti MP, Dusi G, Scroccaro G. Monitoring safety in clinical trials: The view of an Ethical

Committee. Giornale Italiano di Farmacia Clinica. 2000;14(4):166-71.

1980. Albertini A, Djalle D, Faye B, Gamboa D, Luchavez J, Mationg ML, et al. Preliminary enquiry into the availability, price and quality of malaria rapid diagnostic tests in the private health sector of six malaria-endemic countries. Tropical Medicine & International Health. 2012;17(2):147-52. doi: 10.1111/j.1365-3156.2011.02904.x. PubMed PMID: WOS:000299102500002.

1981. Albertson S, Murray T, Triboletti J, Pence L, Gonzalvo J, Meredith A, et al. Implementation of primary care clinical pharmacy services for adults experiencing homelessness. Journal of the American Pharmacists Association. 2021;61(1):e80-e4. doi: https://doi.org/10.1016/j.japh.2020.10.012.

1982. Alblooshi S, Thomas D, Zachariah S. Perceptions on continuity of Cardiovascular patient care by Institutional and Community Pharmacists. Research Journal of Pharmacy and Technology. 2023;16(7):3373-8. doi: 10.52711/0974-360X.2023.00558.

1983. Albrich S, Steetskamp J, Rommens K, Porta S, Battista M, Hoffmann G, et al. Detection of Subpubic Tumor Causing Bladder Outlet Obstruction by 3D Perineal Ultrasound. Geburtshilfe Und Frauenheilkunde. 2015;75(7):719-22. doi: 10.1055/s-0035-1546219. PubMed PMID: WOS:000360243100059.

1984. Albright T, Simonet R, Bollom E, Taylor S, Seckel E, Wilcox A, et al. Feasibility of a centralized specialty biologic medication management clinic at a VA hospital. Journal of the American Pharmacists Association. 2022;62(5):1694-9. doi: https://doi.org/10.1016/j.japh.2022.05.004.

1985. Albsoul-Younes A, Wazaify M, Yousef AM, Tahaineh L. Abuse and misuse of prescription and nonprescription drugs sold in community pharmacies in Jordan. Substance Use and Misuse. 2010;45(9):1319-29. doi: 10.3109/10826080802490683.

1986. Albsoul-Younes AM, Hammad EA, Yasein NA, Tahaineh LM. Pharmacist-physician collaboration improves blood pressure control. Saudi medical journal. 2011;32(3):288-92. PubMed PMID: CN-00786141.

1987. Albsoul-Younes AM, Jabateh SK, Abdel-Hafiz SM, Al-Safi SA. Awareness and frequency of potential side effects on nonsteroidal anti-inflammatory drugs among the Jordanian patient population. Saudi Medical Journal. 2004;25(7):907-11. PubMed PMID: WOS:000223075600017.

1988. Albulushi S, McIntosh T, Grant A, Fahmi M, Stewart D, Al Raisi F, et al. A qualitative exploration of key stakeholders' views and perceptions about organisational change for the implementation of polypharmacy management in Oman. Pharmacy Education. 2023;23(3):269. doi: https://dx.doi.org/10.46542/pe.2023.233.262300. PubMed PMID: 642266052.

1989. Alcalà M, Ropero J, Vázquez R, Romañach RJ. PHARMACEUTICAL TECHNOLOGY - Deconvolution of Chemical Physical Information from Intact Tablets NIR Spectra: Two-Three-Way Multivariate Calibration Strategies for Drug Quantitation. Journal of Pharmaceutical Sciences. 2009;98(8):2747-58. doi: https://doi.org/10.1002/jps.21634.

1990. Alcantara TD, Onozato T, Neto FDA, Dosea AS, Cunha LC, de Araujo D, et al. Perceptions of a group of hospital pharmacists and other professionals of the implementation of clinical pharmacy at a high complexity public hospital in Brazil. Bmc Health Services Research. 2018;18. doi: 10.1186/s12913-018-3036-7. PubMed PMID: WOS:000429272600003.

1991. Alcântara TdS, Carvalho GAC, Sanchez JM, Ramos SF, Cunha LC, Araújo-Neto FdC, et al. Quality indicators of hospitalized children influenced by clinical pharmacist services: A systematic review. Research in Social and Administrative Pharmacy. 2023;19(10):1315-30. doi: https://doi.org/10.1016/j.sapharm.2023.07.003.

1992. Alcaraz JC, Mas RP, Sanchis MCC, Mongars AG, Garcia EG, Ferrer FMB. Survey's Assessment of the Pharmaceutical Care Services Carried out by Alicante's Pharmacists. Pharmaceutical Care Espana. 2015;17(4):442-57. PubMed PMID: WOS:000420549300003.

1993. Alcocer Ve V, Peiró Jf M. Laxantive use in a community pharmacy: Opportunities for improvement. Atencion Farmaceutica. 2008;10(1):48-51.

1994. Alcocer VEV, Ribes C, Peiro JFM. Laxantive use in a community pharmacy: Opportunities. Atencion Farmaceutica. 2008;10(1):48-51. PubMed PMID: WOS:000256792500008.

1995. Alcorn KS, McCord SK, Seed SM, Gravel T, Morrill AM. The veteran-centered care conferences: interprofessional education and community involvement facilitated by the health sciences librarian. Journal of the Medical Library Association. 2022;110(3):365-71. doi: 10.5195/jmla.2022.1491.

1996. Alcover KC, Poltavskiy EA, Howard JT, Watrous JR, Janak JC, Walker LE, et al. Post-Combat-Injury Opioid Prescription and Alcohol Use Disorder in the Military. American Journal of Preventive Medicine. 2022;63(6):904-14. doi: https://doi.org/10.1016/j.amepre.2022.07.010.

1997. Alcuaz R, Nozzi E, Vivas N, Diaz G, Aiziczon D, Siga E. The preservation of residual renal function with high dose of furosemide. A pilot long term study. Nephrology dialysis transplantation. 2019;34:a279-a80. doi: 10.1093/ndt/gfz106.FP683. PubMed PMID: CN-02094855.

1998. Aldali S, Al-Badriyeh D, Gulied A, Hamad A, Al Hail M, Abdul Rouf P, et al. EE518 Characteristics of the Clinical Pharmacist Interventions at a Tertiary Cancer Care Hospital in Qatar. Value in Health. 2023;26(6 Supplement):S154. doi: https://dx.doi.org/10.1016/j.jval.2023.03.2497. PubMed PMID: 2025047862.

1999. Aldardeer NF, Shukairi ANAL, Nasser ME, Al Musawa M, Kalkatawi BS, Alsahli RM, et al. Continuation Versus De-escalation of Broad-Spectrum Antibiotic Therapy in Critically Ill COVID-19 Patients. Dr Sulaiman Al Habib Medical Journal. 2023;5(2):33-41. doi: 10.1007/s44229-023-00027-0. PubMed PMID: 171101124.

2000. Aldarij N, Gipmans S. Guidelines for ailments treatable by over-the-counter medications. Pharmacy Education. 2023;23(3):178-9. doi: https://dx.doi.org/10.46542/pe.2023.233.156186. PubMed PMID: 642265613.

2001. Alderman AK, Storey AF, Chung KC. Financial Impact of Emergency Hand Trauma on the Health Care System. Journal of the American College of Surgeons. 2008;206(2):233-8. doi: 10.1016/j.jamcollsurg.2007.07.043.

2002. Alderman C. Deprescribing: a great idea, but how to get traction? Journal of Pharmacy Practice & Research. 2018;48(5):399-401. doi: 10.1002/jppr.1496. PubMed PMID: 132515202. Language: English. Entry Date: 20181029. Revision Date: 20191121. Publication Type: Article.

2003. Alderman CP. Development and evaluation of an electronic drug and therapeutics bulletin. Annals of Pharmacotherapy. 2002;36(10):1637-41. PubMed PMID: WOS:000178341100020.

2004. Alderman CP, Farmer C. A brief analysis of clinical pharmacy interventions undertaken in an Australian teaching hospital. Journal of Quality in Clinical Practice. 2001;21(4):99-103.

2005. Alderman CP, Kong L, Kildea L. Medication-related problems identified in home medicines reviews conducted in an Australian rural setting. Consultant Pharmacist. 2013;28(7):432-42. PubMed PMID: 23835461.

2006. Alderman CP, Monsanto HA, Dion P. Development and evaluation of an electronic drug and therapeutics bulletin. Annals of Pharmacotherapy. 2002;36(10):1637-41. doi: 10.1345/aph.1A330.

2007. Aldeyab MA, Noble SC, Cuthbert M, Maxwell S, Dear J, Boyter A. Assessment of the impact of the Scottish public health campaign on patient reporting of adverse drug reactions. Drugs and Therapy Perspectives. 2016;32(5):209-18. doi: 10.1007/s40267-016-0280-y.

2008. Aldhwaihi K, Schifano F, Pezzolesi C, Umaru N. A systematic review of the nature of dispensing errors in hospital pharmacies. Integrated Pharmacy Research and Practice. 2016:1-10.

2009. Aldhwaihi KA. Types and contributing factors of dispensing errors in hospital pharmacies [Ph.D.]. England: University of Hertfordshire (United Kingdom); 2015.

2010. Aldila F, Walpola RL. Medicine self-administration errors in the older adult population: A systematic review. Research in Social & Administrative Pharmacy. 2021;17(11):1877-86. doi: 10.1016/j.sapharm.2021.03.008. PubMed PMID: 152604682. Language: English. Entry Date: 20211030. Revision Date: 20211118. Publication Type: journal article.

2011. Aldosari B. Rates, levels, and determinants of electronic health record system adoption: A study of hospitals in Riyadh, Saudi Arabia. International Journal of Medical Informatics. 2014;83(5):330-42. doi: 10.1016/j.ijmedinf.2014.01.006.

2012. Aldosari M. Medicines Adherence in Children [Ph.D.]. England: The University of Nottingham (United Kingdom); 2021.

2013. AlDossary S, Martin-Khan MG, Bradford NK, Smith AC. A systematic review of the methodologies used to evaluate telemedicine service initiatives in hospital facilities. International journal of medical informatics. 2017;97:171-94.

2014. Aldred R. 'A potential fifth column': conflicts and struggles for control in the context of local NHS privatization. Health. 2009;13(5):543-61. doi: 10.1177/1363459308336797. PubMed PMID: WOS:000269150700004.

2015. Aldridge VE, Park HK, Bounthavong M, Morreale AP. Implementing a comprehensive, 24-hour emergency department pharmacy program. American Journal of Health-System Pharmacy. 2009;66(21):1943-7. PubMed PMID: 19850790.

2016. Aldrigue RDFT, Correr CJ, Melchiors AC, Pontarolo R. Analysis of filling out of prescriptions dispensing in a community pharmacy of Fazenda Rio Grande - Paraná (Brazil). Acta Farmaceutica Bonaerense. 2006;25(3):454-9.

2017. Aldryhim AY, Alomair A, Alqhtani M, Mahmoud MA, Alshammari TM, Pont LG, et al. Factors that facilitate reporting of adverse drug reactions by pharmacists in Saudi Arabia. Expert Opinion on Drug Safety. 2019;18(8):745-52. doi: 10.1080/14740338.2019.1632287.

2018. Aldughayfiq B, Sampalli S. Digital health in physicians' and pharmacists' office: a comparative study of e-prescription systems' architecture and digital security in eight countries. Omics: a journal of integrative biology. 2021;25(2):102-22.

2019. Aldughayfiq B, Sampalli S. A framework to lower the risk of medication prescribing and dispensing errors: A usability study of an NFC-based mobile application. International Journal of Medical Informatics. 2021;153:104509. doi: https://doi.org/10.1016/j.ijmedinf.2021.104509.

2020. Aldughayfiq B, Sampalli S. Patients', pharmacists', and prescribers' attitude toward using blockchain and machine learning in a proposed ePrescription system: Online survey. JAMIA Open. 2022;5(1). doi: 10.1093/jamiaopen/ooab115.

2021. Alefan Q, Alshareef S, Al-Shatnawi S. Drug and therapeutics committees in jordanian hospitals: A nation-wide survey of organization, activities, and drug selection procedures. Pharmacy Practice. 2019;17(4). doi: 10.18549/PharmPract.2019.4.1590.

2022. Alefishat E, Jarab AS, Muflih S, Aqeel AW. Community pharmacists' attitudes toward practice-based research and their perceived utilization of scientific evidence. Plos One. 2022;17(3). doi: 10.1371/journal.pone.0264193. PubMed PMID: WOS:000799901400013.

2023. Alehyan A, Araujo-Santos JM, Martinez F, Faus MJ. Pharmacotherapeutical follow-up enhances HRQL and pharmacy sales to chronic patients. International Journal of Clinical Pharmacy. 2011;33(2):425-6. PubMed PMID: WOS:000289415500339.

2024. Alekhin AV, Erivantseva TN, Ryazhenov VV, Lyskov NB, Alekhina NA, Kuznetsova MM. New role of extemporaneous manufacturing in regulating drug products access onto the market. Farmatsiya i Farmakologiya. 2023;11(2):161-72. doi: 10.19163/2307-9266-2023-11-2-161-172.

2025. Alem G, Awuonda M, Haastrup D, Anyiwo A, Daftary M, Wingate LM, et al. Evaluation of knowledge of the new Pregnancy and Lactation Labeling Rule among pharmacists and physicians. Journal of the American Pharmacists Association. 2022;62(2):427-31. doi: https://doi.org/10.1016/j.japh.2021.10.012.

2026. Alemayehu C, Mitchell G, Aseffa A, Clavarino A, McGree J, Nikles J. A series of N-of-1 trials to assess the therapeutic interchangeability of two enalapril formulations in the treatment of hypertension in Addis Ababa, Ethiopia: study protocol for a randomized controlled trial. Trials. 2017;18(1):470. doi: 10.1186/s13063-017-2212-0. PubMed PMID: CN-01615311.

2027. Alemu BA, Hailemariam FH. Price, Availability and Affordability of Anti-Cancer Medicines in Addis Ababa, Ethiopia. Risk Management and Healthcare Policy. 2022;15:2421-33. doi: 10.2147/rmhp.s395456.

2028. Alemu S, Tadesse N, Mulugeta T, Assefa D. Generic substitution for prescribed brand medicines in Ethiopia: knowledge, attitude and practice among pharmacy professionals in community drug retail outlets. BMC Health Services Research. 2022;22(1). doi: 10.1186/s12913-022-08330-6.

2029. Alemu T, Jemal A, Gashe F, Suleman S, Sudhakar S, Fekadu G. Integrated pharmaceutical logistics system implementation in selected health facilities of Ethiopia: The case of four WOLLEGA ZONES. Research in Social and Administrative Pharmacy. 2021;17(5):956-68. doi: https://doi.org/10.1016/j.sapharm.2020.07.026.

2030. Alenezi A, Yahyouche A, Paudyal V. Roles, barriers and behavioral determinants related to community pharmacists' involvement in optimizing opioid therapy for chronic pain: a qualitative study. International Journal of Clinical Pharmacy. 2022;44(1):180-91. doi: 10.1007/s11096-021-01331-1.

2031. Alenezi S, Alanazi M, Aljazaeri R, Almuzaini M, Alrasheidi S, Shamlan WB, et al. Community Pharmacies in the Asian Countries of Developing Health System: Formation, Regulation, and Implication. Pharmacy. 2023;11(4). doi: 10.3390/pharmacy11040127. PubMed PMID: WOS:001055843700001.

2032. Aleo CL, Murchison AP, Dai Y, Hark LA, Mayro EL, Collymore B, et al. Improving eye care follow-up adherence in diabetic patients with ocular abnormalities: the effectiveness of patient contracts in a free, pharmacy-based eye screening. Public health. 2015;129(7):996-9. doi: 10.1016/j.puhe.2015.05.012. PubMed PMID: CN-01472934.

2033. Aleo CL, Murchison AP, Hark LA, Dai Y, Mayro E, Leiby B, et al. Improving eye care follow-up adherence in patients with Diabetes: the effectiveness of patient contracts in a community-based eye screening. Investigative ophthalmology & visual science. 2014;55(13):6094-. PubMed PMID: CN-01377422.

2034. Alex S, Adenew AB, Arundel C, Maron DD, Kerns JC. Medication Errors Despite Using Electronic Health Records: The Value of a Clinical Pharmacist Service in Reducing Discharge-Related Medication Errors. Quality Management in Health Care. 2016;25(1):32-7. doi: 10.1097/QMH.0000000000000080. PubMed PMID: 112808945. Language: English. Entry Date: 20180521. Revision Date: 20160307. Publication Type: Article.

2035. Alexa JM, Bertsche T. An online cross-sectional survey of community pharmacists to assess information needs for evidence-based self-medication counselling. International Journal of Clinical Pharmacy. 2023;45(6):1452-63. doi: 10.1007/s11096-023-01624-7.

2036. Alexa JM, Richter M, Bertsche T. Enhancing Evidence-Based Pharmacy by Comparing the Quality of Web-Based Information Sources to the EVInews Database: Randomized Controlled Trial With German Community Pharmacists. Journal of Medical Internet Research. 2023;25. doi: 10.2196/45582.

2037. Alexander A, editor BPC 2002 presentations previewed. Pharmaceutical Journal; 2002.

2038. Alexander AJ, Gatewood SB. Revealing undetected problems with medication therapy management services. Consultant Pharmacist. 2008;23(6):473-6. PubMed PMID: 18764677.

2039. Alexander AJ, Gatewood SB, Alexander AJ, Gatewood SBS. Revealing undetected problems with medication therapy management services. Consultant Pharmacist. 2008;23(6):473-6. PubMed PMID: 105555681. Language: English. Entry Date: 20090724. Revision Date: 20191116. Publication Type: journal article.

2040. Alexander AJ, Gatewood SBS. Revealing undetected problems with medication therapy management services. Consultant Pharmacist. 2008;23(6):473-6. doi: 10.4140/TCP.n.2008.473.

2041. Alexander D, Kurup U, Menon A, Mahgerefteh M, Warters A, Rigby M, et al. Affiliate Contributors to Primary Care for Children. Issues and Opportunities in Primary Health Care for Children in Europe: The Final Summarised Results of the Models of Child Health Appraised (MOCHA) Project2019. p. 303-30.

2042. Alexander DC, Bundy DG, Shore AD, Morlock L, Hicks RW, Miller MR. Cardiovascular medication errors in children. Pediatrics. 2009;124(1):324-32. doi: 10.1542/peds.2008-2073. PubMed PMID: 105377467. Language: English. Entry Date: 20090821. Revision Date: 20200708. Publication Type: Journal Article.

2043. Alexander K, Hamlin PA, Tew WP, Trevino K, Tin AL, Shahrokni A, et al. Development and implementation of an interdisciplinary telemedicine clinic for older patients with cancer-Preliminary data. Journal of the American Geriatrics Society. 2023;71(5):1638-49. doi: https://dx.doi.org/10.1111/jgs.18267. PubMed PMID: 2021353875.

2044. Alexander KM, Divine HS, Hanna CR, Gokun Y, Freeman PR. Implementation of personalized medicine services in community pharmacies: Perceptions of independent community pharmacists. Journal of the American Pharmacists Association. 2014;54(5):510-7. doi: 10.1331/JAPhA.2014.13041.

2045. Alexander KP, Chen AY, Newby LK, Schwartz JB, Redberg RF, Hochman JS, et al. Sex differences in major bleeding with glycoprotein IIb/IIIa inhibitors: results from the CRUSADE (Can Rapid risk stratification of Unstable angina patients Suppress ADverse outcomes with Early implementation of the ACC/AHA guidelines) initiative. Circulation. 2006;114(13):1380-7.

2046. Alexander KP, Chen AY, Roe MT, Newby LK, Gibson CM, Allen-LaPointe NM, et al. Excess dosing of antiplatelet and antithrombin agents in the treatment of non–ST-segment elevation acute coronary syndromes. Jama. 2005;294(24):3108-16.

2047. Alexander L, Weingarten B. A pharmacist's guide to the emerging abuse deterrence technology used in opioid analgesics. Pharmacy Times. 2014;80(8).

2048. Alexander M, Jupp J, Chazan G, O'Connor S, Chan A. Global oncology pharmacy response to COVID-19 pandemic: Medication access and safety. Journal of Oncology Pharmacy Practice. 2020;26(5):1225-9. PubMed PMID: 32408842.

2049. Alexander M, Kirsa S, Wolfe R, MacManus M, Ball D, Solomon B, et al. Thromboembolism in lung cancer – An area of urgent unmet need. Lung Cancer. 2014;84(3):275-80. doi: https://doi.org/10.1016/j.lungcan.2014.02.009.

2050. Alexander MD, Rao KV, Khan TS, Deal AM. Pharmacists' Impact in Hematopoietic Stem-Cell Transplantation: Economic and Humanistic Outcomes. Journal of Oncology Practice. 2016;12(2):147-+. doi: 10.1200/jop.2015.008797. PubMed PMID: WOS:000374325300018.

2051. Alexandraki KI, Syriou V, Ziakas PD, Apostolopoulos NV, Alexandrakis AI, Piperi C, et al. The knowledge of osteoporosis risk factors in a Greek female population. Maturitas. 2008;59(1):38-45. doi: https://doi.org/10.1016/j.maturitas.2007.10.008.

2052. Alexandrescu LG. Injecting ATS/NPS use and drug abjection in Romania. Drugs and Alcohol Today. 2016;16(2):142-9. doi: 10.1108/dat-09-2015-0056. PubMed PMID: WOS:000401789300004.

2053. Alexiou VG, Falagas ME. e-meducation.org: an open access medical education web portal. Bmc Medical Education. 2008;8. doi: 10.1186/1472-6920-8-6. PubMed PMID: WOS:000207735100006.

2054. Alexopoulou E, Demetzos C. Legal framework of clinical trials - Paediatric clinical trials. Pharmakeftiki. 2008;21(4):105-11.

2055. Alexxander, Puspitasari I, Kristina SA, Kristanto CS, Prihandiwati E. 3 Co-TEAM: A Logic Model for Pharmacy Health Coaching among Substance Use Disorders Patients. Indonesian Journal of Pharmacy. 2022;33(1):52-62. PubMed PMID: WOS:000826457000004.

2056. Alfa-Wali M, Mohammed I, Yusuph H. Under the counter, underground or unethical medicines? An African perspective. Tropical Doctor. 2014;44(1):21-4. PubMed PMID: 24265193.

2057. Alfadl AA, Alrasheedy AA, Alhassun MS. Evaluation of medication counseling practice at community pharmacies in Qassim region, Saudi Arabia. Saudi pharmaceutical journal. 2018;26(2):258-62.

2058. Alfadl AA, Anaam MS, Alhassun MS, Albuaymi YR, Alrehaili FM, Alsikhan RS. Evaluation of patients' knowledge about their medications: A cross-sectional study in Qassim region, Saudi Arabia. Journal of Medicine Access. 2023;7. doi: 10.1177/27550834231163003.

2059. Alfadl AA, Hassali MA, Ibrahim MIM. Counterfeit drug demand: Perceptions of policy makers and community pharmacists in Sudan. Research in Social and Administrative Pharmacy. 2013;9(3):302-10. doi: 10.1016/j.sapharm.2012.05.002.

2060. Alfadly S, Anaam M, Alshammari M, Alsahali S, Ahmed E, Mubarak AB, et al. Drug Information Sources for Patients with Chronic Conditions in the Qassim Region, Saudi Arabia. Pharmacy. 2023;11(2). doi: 10.3390/pharmacy11020057. PubMed PMID: WOS:000977405900001.

2061. Alfadly S, Ballaswad MR, Amra AS, Alghadeer SM, Wajid S, Al-Arifi MN, et al. Self-medication with antibiotic amongst adults attending community pharmacies in Mukalla district, Yemen. Latin American Journal of Pharmacy. 2017;36(2):224-8.

2062. Alfahmi AA, Curtain CM, Salahudeen MS. Assessment of Knowledge, Attitude and Practices of the Hospital and Community Pharmacists in Saudi Arabia (Jeddah) towards Inappropriate Medication Use in Older Adults. International Journal of Environmental Research and Public Health. 2023;20(2):1635. doi: https://dx.doi.org/10.3390/ijerph20021635. PubMed PMID: 2021152700.

2063. Alfahmi AA, Curtain CM, Salahudeen MS. Assessment of Knowledge, Attitude and Practices of the Hospital and Community Pharmacists in Saudi Arabia (Jeddah) towards Inappropriate Medication Use in Older Adults. International Journal of Environmental Research and Public Health. 2023;20(2). doi: 10.3390/ijerph20021635.

2064. Alfaifi JA, Alqarni SAM, Alqarni A, Alqahtani MM, Alshomrani RA. Parents' Knowledge, Attitude, and Practice Regarding Traditional Medicine on Their Children: A Community-Based Cross-Sectional Study in Bisha City, Saudi Arabia. Cureus Journal of Medical Science. 2023;15(8). doi: 10.7759/cureus.43136. PubMed PMID: WOS:001173889600016.

2065. Alfaro IG, Rodriguez JDC. Detection of therapeutic duplications in community pharmacy: Importance of the pharmaceutical follow-up service in patients cared by several specialists. Possible improvements. Pharmaceutical Care Espana. 2018;20(4):313-21. PubMed PMID: WOS:000441450900005.

2066. Alfaro IG, Rodriguez JDC. Electronic prescription: differences between autonomous communities affecting access to treatment and the quality of pharmaceutical care. Anales Del Sistema Sanitario De Navarra. 2020;43(3):297-306. doi: 10.23938/assn.0880. PubMed PMID: WOS:000681631300002.

2067. Alfaya FJF. Challenges and actions of the community pharmacist regarding vitamin D. Farmaceuticos Comunitarios. 2021;13(2):8-10. doi: 10.33620/FC.2173-9218.(2021/Vol13).002.03. PubMed PMID: WOS:000641969200004.

2068. Alfian SD, Abdulah R, Denig P, van Boven JFM, Hak E. Targeted and tailored pharmacist-led intervention to improve adherence to antihypertensive drugs among patients with type 2 diabetes in Indonesia: study protocol of a cluster randomised controlled trial. BMJ open. 2020;10(1):e034507. doi: 10.1136/bmjopen-2019-034507. PubMed PMID: CN-02076106.

2069. Alfian SD, Denig P, Abdulah R, Sukandar H, Van Boven JFM, Hak E. Effectiveness of a targeted and tailored pharmacist-led intervention to improve adherence to antihypertensive drugs among patients with type 2 diabetes in Indonesia: a cluster randomized controlled trial. Pharmacoepidemiology and drug safety. 2020;29(SUPPL 3):147-. doi: 10.1002/pds.5114. PubMed PMID: CN-02242682.

2070. Alfian SD, Khoiry QA, Pratama MAA, Wahyudin W, Puspitasari IM, Pradipta IS, et al. Awareness and beliefs of community pharmacists on disposal of unused and expired household medications in Indonesia: a cross-sectional study. Journal of Pharmaceutical Health Services Research. 2023;14(4):401-6. doi: 10.1093/jphsr/rmad043.

2071. Alfian SD, Sinuraya RK, Kautsar AP, Abdulah R. Consumer expectation on service quality provide by pharmacist in self medication practices and its associated factors in Bandung, Indonesia. Southeast Asian Journal of Tropical Medicine and Public Health. 2016;47(6):1379-84.

2072. Alfian SD, van Boven JFM, Abdulah R, Sukandar H, Denig P, Hak E. Effectiveness of a targeted and tailored pharmacist-led intervention to improve adherence to antihypertensive drugs among patients with type 2 diabetes in Indonesia: a cluster randomised controlled trial. British journal of clinical pharmacology.

2021;87(4):2032-42. doi: 10.1111/bcp.14610. PubMed PMID: CN-02213720.

2073. Alford K, Leake-Date H, Moore D, Ing K, Vera JH. A multicentred randomised controlled open study of the utility and acceptability of a medicines optimisation review (MOR) toolkit compared to standard pharmaceutical care in HIV outpatients. HIV medicine. 2019;20:39-40. doi: 10.1111/hiv.12815. PubMed PMID: CN-02119302.

2074. Alford K, Vera J, Date HL, Moore D, Ing K. Utility of a medicines optimisation review (MOR) compared with standard pharmaceutical care in people with HIV on cART. HIV medicine. 2019;20:49-. doi: 10.1111/hiv.12739. PubMed PMID: CN-01936515.

2075. Alford KF. Progressive Care Nurses' Attitudes, Beliefs, and Perceptions of Automated Medication Dispensing Cabinet Workarounds: A Mixed Methods Study. Progressive Care Nurses' Attitudes, Beliefs & Perceptions of Automated Medication Dispensing Cabinet Workarounds: A Mixed Methods Study. 2016:1-. PubMed PMID: 124665102. Language: English. Entry Date: 20180427. Revision Date: 20180621. Publication Type: Article.

2076. Algara CR, Modamio P, Marfa MC, Vilardell EV, Gonzalez MM, Catalina QM, et al. Health outcomes of patient-centered medication review interventions. Preliminary results. International Journal of Clinical Pharmacy. 2023;45(2):518. doi: https://dx.doi.org/10.1007/s11096-023-01576-y. PubMed PMID: 641388269.

2077. Algarni FS, Kachanathu SJ, Alabdulwahab SS. A Cross-Sectional Study on the Association of Patterns and Physical Risk Factors with Musculoskeletal Disorders among Academicians in Saudi Arabia. BioMed research international. 2020;2020:8930968. doi: 10.1155/2020/8930968. PubMed PMID: CN-02194522.

2078. Algarni M, Hadi MA, Yahyouche A, Mahmood S, Jalal Z. A mixed-methods systematic review of the prevalence, reasons, associated harms and risk-reduction interventions of over-the-counter (OTC) medicines misuse, abuse and dependence in adults. Journal of Pharmaceutical Policy and Practice. 2021;14(1). doi: 10.1186/s40545-021-00350-7.

2079. Algarni M, Jalal Z, Hadi MA, Alghamdi S. Community pharmacists' views and experiences toward over-the-counter medicines misuse and abuse in Saudi Arabia: A qualitative study. Frontiers in Pharmacology. 2022;13. doi: 10.3389/fphar.2022.997342.

2080. Algera-Van Der Schaaf MAW. Impressions from Scotland; a mirror? Pharmaceutisch Weekblad. 1996;131(13):383-9.

2081. Algethami AA, Alshamrani MA, AlHarbi AM, AlAzmi AA, Khan MA, AlHadidi RA. Evaluation of handling, storage, and disposal practices of oral anticancer medications among cancer patients and their caregivers at home setting in the Princess Noorah Oncology Center. Journal of Oncology Pharmacy Practice. 2021;27(1):20-5. doi: 10.1177/1078155220908937. PubMed PMID: WOS:000523801400001.

2082. Alghadeer S, Al-Arifi MN. Community pharmacists' practice, awareness, and beliefs about drug disposal in saudi arabia. Healthcare (Switzerland). 2021;9(7). doi: 10.3390/healthcare9070823.

2083. Alghadeer S, Babelghaith SD, Syed W, Al-Arifi MN. Community pharmacists' knowledge, prospective and practice towards health related illness at Hajj and Umrah: A cross sectional study. Saudi Pharmaceutical Journal. 2023;31(10):101786. doi: https://doi.org/10.1016/j.jsps.2023.101786.

2084. Alghamdi A, Alhulaylah F, Al-Qahtani F, Alsallal D, Alshabanat N, Alanazi H, et al. Evaluation of pharmacy intern-led transition of care service at an academic hospital in Saudi Arabia: A prospective pilot study. Saudi Pharmaceutical Journal. 2022;30(5):629-34. doi: https://doi.org/10.1016/j.jsps.2022.02.007.

2085. Alghamdi A, Seoane-Vazquez E, Rodriguez-Monguio R. ANALYSIS OF THE IMPACT OF AFFORDABLE CARE ACT AND THE 2011 FDA SAFETY COMMUNICATION ON THE UTILIZATION OF BIOLOGIC DISEASE-MODIFYING ANTI-RHEUMATIC DRUGS IN THE MEDICAID OUTPATIENT PHARMACY PROGRAM. Value in Health. 2017;20(5):A152-A. PubMed PMID: WOS:000405448002095.

2086. Alghamdi EA, Albalawi OM, Alshammari TM. Health outcomes and policy in pharmay curricula among Arab countries: An evaluation of 191 academic institutions. Saudi Pharmaceutical Journal. 2021;29(8):799-806. doi: https://doi.org/10.1016/j.jsps.2021.06.001.

2087. Alghamdi KS, Petzold M, Alsugoor MH, Makeen HA, Al Monif KH, Hussain-Alkhateeb L. Community pharmacists' perspectives towards automated pharmacy systems and extended community pharmacy services: An online cross-sectional study. Exploratory Research in Clinical and Social Pharmacy. 2023;12:100363. doi: https://dx.doi.org/10.1016/j.rcsop.2023.100363. PubMed PMID: 2028277647.

2088. Alghamdi KS, Petzold M, Alsugoor MH, Makeen HA, Monif KHA, Hussain-Alkhateeb L. Community pharmacists' perspectives towards automated pharmacy systems and extended community pharmacy services: An online cross-sectional study. Exploratory Research in Clinical and Social Pharmacy. 2023;12. doi: 10.1016/j.rcsop.2023.100363. PubMed PMID: WOS:001134090800001.

2089. Alghamdi KS, Petzold M, Ewis AA, Alsugoor MH, Saaban K, Hussain-Alkhateeb L. Public perspective toward extended community pharmacy services in sub-national Saudi Arabia: An online cross-sectional study. PLoS ONE. 2023;18(10 OCTOBER):e0280095. doi: https://dx.doi.org/10.1371/journal.pone.0280095. PubMed PMID: 2027751746.

2090. Alghamdi S. The adoption of Antimicrobial Stewardship Programmes in Ministry of Health hospitals in Saudi Arabia [Ph.D.]. England: University of Hertfordshire (United Kingdom); 2018.

2091. Alghanem SS, Bayoud T, Taher S, Al-Hazami M, Al-Kandari N, Al-Sharekh M. Introduction of an Ambulatory Care Medication Reconciliation Service in Dialysis Patients: Positive Impact on Medication Prescribing and Economic Benefit. Journal of patient safety. 2022;18(2):e489-e95. PubMed PMID: 34009876.

2092. Alghanem SS, Bayoud TP, Taher S, Al-Hazami M, Al-Kandari N, Al-Sharekh M. Introduction of an Ambulatory Care Medication Reconciliation Service in Dialysis Patients: Positive Impact on Medication Prescribing and Economic Benefit. Journal of Patient Safety. 2022;18(2):e489-e95. doi: 10.1097/PTS.0000000000000853. PubMed PMID: 155384549. Language: English. Entry Date: 20220307. Revision Date: 20220307. Publication Type: Article.

2093. Alghanim SA. Self-medication practice among patients in a public health care system. Eastern Mediterranean Health Journal. 2011;17(5):409-16. doi: 10.26719/2011.17.5.409.

2094. AlGhofaili F. Patterns of self-medication in Qassim Province, Saudi Arabia: A cross-sectional study. Annals of Medicine and Surgery. 2021;64:102207. doi: https://doi.org/10.1016/j.amsu.2021.102207.

2095. AlGhurair SA, Simpson SH, Guirguis LM. What elements of the patient-pharmacist relationship are associated with patient satisfaction? Patient Preference and Adherence. 2012;6:663-76. doi: 10.2147/PPA.S35688.

2096. Algouzi M, Alhawassi TM, Alburikan K, Adam M, Aljadhey H. Impact of discharge counseling by Pharm. D. Candidates on adverse drug events risk among hospitalized older patients. Pharmacoepidemiology and drug safety. 2017;26:597-8. doi: 10.1002/pds.4275. PubMed PMID: CN-01418409.

2097. Algra A, Pereira T, Coetzee M. Thriving by education: Shining the light on handling light sensitive medication. SA Pharmaceutical Journal Incorporating Pharmacy Management. 2023;90(5):56-8. PubMed PMID: 175493968.

2098. Alhaag MH, Aziz T, Alharkan IM, Ieee, editors. A Queuing Model for Health care Pharmacy Using Software Arena. 2015 International Conference on Industrial Engineering and Operations Management (IEOM); 2015 Mar 03-05; Dubai, U ARAB EMIRATES2015.

2099. Alhabardi S, Alhusayni L, Aljebreen M, Alzamil A, AlSwead M, Alrohaimi M, et al. EPH47 Active Surveillance for Safety Monitoring of COVID-19 Vaccines in Saudi Arabia. Value in Health. 2023;26(6):S172-S3.

2100. Alhabib S, Aldraimly M, Alfarhan A. An evolving role of clinical pharmacists in managing diabetes: Evidence from the literature. Saudi Pharmaceutical Journal. 2016;24(4):441-6. doi: https://doi.org/10.1016/j.jsps.2014.07.008.

2101. Alhaddad MS. Youth Experience With Community Pharmacy Services and Their Perceptions Toward Implementation of Medication Therapy Management Services by Community Pharmacists in the Western Region of Saudi Arabia. Therapeutic Innovation & Regulatory Science. 2019;53(1):95-9. PubMed PMID: 29714597.

2102. Alhamad H. Sustainability and medicines waste: investigating public attitudes towards the reuse of medicines returned to community pharmacies [Ph.D.]. England: The University of Reading (United Kingdom); 2018.

2103. Alhamad H, Patel N, Donyai P. How do people conceptualise the reuse of medicines? An interview study. International Journal of Pharmacy Practice. 2018;26(3):232-41. doi: 10.1111/ijpp.12391.

2104. Alhamad H, Patel N, Donyai P. Towards Medicines Reuse: A Narrative Review of the Different Therapeutic Classes and Dosage Forms of Medication Waste in Different Countries. Pharmacy. 2020;8(4). doi: 10.3390/pharmacy8040230. PubMed PMID: WOS:000601894500001.

2105. Alhamarneh YN. Coronary heart disease-public knowledge and the pharmacist's patient-centred role in prevention and management [Ph.D.]. Northern Ireland: Queen's University Belfast (United Kingdom); 2010.

2106. Alhanout K, Bun S-S, Retornaz K, Chiche L, Colombini N. Prescription errors related to the use of computerized provider order-entry system for pediatric patients. International Journal of Medical Informatics. 2017;103:15-9. doi: 10.1016/j.ijmedinf.2017.04.005. PubMed PMID: 123218472. Language: English. Entry Date: 20180129. Revision Date: 20190711. Publication Type: journal article.

2107. Alhanout K, Bun SS, Retornaz K, Chiche L, Colombini N. Prescription errors related to the use of computerized provider order-entry system for pediatric patients. International Journal of Medical Informatics. 2017;103:15-9. PubMed PMID: 28550997.

2108. Alharbi A, Umaru N, Webley S, Alsaleh F, Shebl N. Healthcare professionals' knowledge and implementation of valproate related safety communications in Kuwait. Pharmacy Education. 2023;23(3):294-5. doi: https://dx.doi.org/10.46542/pe.2023.233.262300. PubMed PMID: 642265945.

2109. Alharbi F, Aldayel A, Alrwisan A. Acceptance of real world data use in evidence-based medicine or decision making by committee members affiliated with the Saudi food and drug authority. Pharmacoepidemiology and drug safety. 2020;29(SUPPL 3):187-. doi: 10.1002/pds.5114. PubMed PMID: CN-02242687.

2110. Alharbi HM. Improving Pharmaceutical Compounding Skills Using Gagne's Instructional Design Model. Archives of Pharmacy Practice. 2023;14(4):32-8. doi: 10.51847/XbtnqShK6t. PubMed PMID: 174227839.

2111. Alharbi W, Cleland J, Morrison Z. Exploring healthcare professionals' perceptions of medication errors in an adult oncology department in Saudi Arabia: A qualitative study. Saudi Pharmaceutical Journal. 2019;27(2):176-81. doi: https://doi.org/10.1016/j.jsps.2018.10.001.

2112. Alharbi W, Cleland J, Morrison Z. Addressing medication errors in an adult oncology department in Saudi Arabia: A qualitative study. Saudi Pharmaceutical Journal. 2019;27(5):650-4. doi: https://doi.org/10.1016/j.jsps.2019.03.007.

2113. Alharthi H, Sultana N, Al-Amoudi A, Basudan A. An Analytic Hierarchy Process-based Method to Rank the Critical Success Factors of Implementing a Pharmacy Barcode System. Perspectives in Health Information Management. 2015;12:1g. PubMed PMID: 26807079.

2114. Alharthi M, Wright D, Scott S, Birt L. Barriers and enablers to deprescribing for older people in care homes: The theory-based perspectives of pharmacist independent prescribers. Research in social & administrative pharmacy : RSAP. 2023;19(5):746-52. doi: https://dx.doi.org/10.1016/j.sapharm.2023.01.013. PubMed PMID: 640245068.

2115. Alharthy A, Hosler AS, Leckman-Westin E, Kammer JR. Association Between Tobacco Retailer Density and Smoking Among Adults With Diabetes and Serious Mental Illness in New York State. Preventing Chronic Disease. 2022;19. doi: 10.5888/pcd19.210270. PubMed PMID: WOS:000742384800001.

2116. Alharthy AF. Evaluating the Impact of Tobacco Retailer Density on Smoking among Patients with Serious Mental Illness and Diabetes in New York State [Dr.P.H.]. United States -- New York: State University of New York at Albany; 2021.

2117. Alhashimi L, Cordwin DJ, Dandu C, Hummel SL, Dorsch MP. Patient self-assessment of urine dipsticks to estimate sodium intake in patients with hypertension. Clinical Nutrition ESPEN. 2022;51:295-300. doi: https://doi.org/10.1016/j.clnesp.2022.08.011.

2118. Alhawassi TM, Abuelizz HA, Almetwazi M, Mahmoud MA, Alghamdi A, Alruthia YS, et al. Advancing pharmaceuticals and patient safety in Saudi Arabia: A 2030 vision initiative. Saudi Pharmaceutical Journal. 2018;26(1):71-4. doi: https://doi.org/10.1016/j.jsps.2017.10.011.

2119. Alhmoud E, Al Khiyami D, Barazi R, Saad M, Al-Omari A, Awaisu A, et al. Perspectives of clinical pharmacists on the provision of pharmaceutical care through telepharmacy services during COVID-19 pandemic in Qatar: A focus group. PLoS ONE. 2022;17(10 October). doi: 10.1371/journal.pone.0275627.

2120. Alhnan MA, Okwuosa TC, Sadia M, Wan K-W, Ahmed W, Arafat B. Emergence of 3D printed dosage forms: opportunities and challenges. Pharmaceutical research. 2016;33:1817-32.

2121. Alhomoud F. Medication use and medicine-related problems (mrps) experienced by south asian (sa) and middle eastern (me) patients with chronic diseases in primary care in the uk [Ph.D.]. England: University of London, University College London (United Kingdom); 2014.

2122. Alhomoud F, Aljamea Z, Almahasnah R, Alkhalifah K, Basalelah L, Alhomoud FK. Self-medication and self-prescription with antibiotics in the Middle East—do they really happen? A systematic review of the prevalence, possible reasons, and outcomes. International Journal of Infectious Diseases. 2017;57:3-12. doi: https://doi.org/10.1016/j.ijid.2017.01.014.

2123. Alhomoud F, Aljamea Z, Almahasnah R, Alkhalifah K, Basalelah L, Alhomoud FK. Self-medication and self-prescription with antibiotics in the Middle East-do they really happen? A systematic review of the prevalence, possible reasons, and outcomes. International Journal of Infectious Diseases. 2017;57:3-12. doi: 10.1016/j.ijid.2017.01.014. PubMed PMID: WOS:000397947500002.

2124. Alhomoud F, Almahasnah R, Alhomoud FK. "You could lose when you misuse" - factors affecting over-the-counter sale of antibiotics in community pharmacies in Saudi Arabia: a qualitative study. BMC Health Services Research. 2018;18(1):N.PAG-N.PAG. doi: 10.1186/s12913-018-3753-y. PubMed PMID: 133368403. Language: English. Entry Date: In Process. Revision Date: 20190217. Publication Type: journal article. Journal Subset: Biomedical.

2125. Alhomoud FK. Act like a warrior to defeat medication counselling barriers: A cross sectional study. Saudi Pharmaceutical Journal. 2020;28(9):1084-92. doi: 10.1016/j.jsps.2020.07.009.

2126. Alhomoud FK, Alsadiq Y, Alghalawin L, Alhifany A, Alhomoud F. Pharmacy students' knowledge and practices concerning the storing and disposal of household medication in Saudi Arabia. Currents in Pharmacy Teaching and Learning. 2021;13(1):5-13. doi: 10.1016/j.cptl.2020.08.004. PubMed PMID: WOS:000583719800002.

2127. Alhomoud FK, Kunbus A, Ameer A, Alhomoud F. Quality Assessment of community pharmacy services provided in the United Arab Emirates: Patient experience and satisfaction. Journal of Applied Pharmaceutical Science. 2016;6(3):17-23. doi: 10.7324/JAPS.2016.60303.

2128. Alhossan A, Al Aloola N, Basoodan M, Alkathiri M, Alshahrani R, Mansy W, et al. COMMUNITY PHARMACIES SERVICES AND PREPAREDNESS DURING COVID-19 PANDEMIC IN SAUDI ARABIA: A CROSS- SECTIONAL STUDY. Pharmacophore. 2023;14(4):28-34. doi: 10.51847/iYOaj9kC0C. PubMed PMID: WOS:001073683200001.

2129. Alhossan A, Alazba A. Barriers interfering with establishment of Collaborative Drug Therapy Management (CDTM) agreements between clinical pharmacists and physicians. Saudi Pharmaceutical Journal. 2019;27(5):713-6. doi: https://doi.org/10.1016/j.jsps.2019.04.006.

2130. Alhossan A, Alhuqbani W, Abogosh A, Alkhezi O, Alessa M, Ahmad A. INPATIENT PHARMACIST INTERVENTIONS IN REDUCING PRESCRIPTION-RELATED MEDICATION ERRORS IN INTENSIVECARE UNIT (ICU) IN RIYADH, SAUDI ARABIA. Farmacia. 2023;71(2):404-10. doi: https://dx.doi.org/10.31925/farmacia.2023.2.22. PubMed PMID: 2023800568.

2131. Alhossan A, Alrabiah Z, Alghadeer S, Bablghaith S, Wajid S, Al-Arifi M. Attitude and knowledge of Saudi community pharmacists towards use of proton pump inhibitors. Saudi Pharmaceutical Journal. 2019;27(2):225-8. doi: https://doi.org/10.1016/j.jsps.2018.11.002.

2132. Alhrasen MNM. Assessment of adverse drug events in hospitalised patients [Ph.D.]. Northern Ireland: Queen's University Belfast (United Kingdom); 2012.

2133. Alhubaishi AA. Pancytopenia and Septic Infection Caused by Concurrent Use of Allopurinol and Mercaptopurine: A Case Report Illustrating the Importance of Clinical Pharmacist Consultation. The American Journal of Case Reports. 2019;20:1245-7. PubMed PMID: 31439827.

2134. Alhusayni L, Alorf N, Alhawas S, Al-Fadel N, Al-Harbi F. EPH254 Proactive Drug Safety Monitoring of Adverse Drug Events in Saudi Arabia: 2022 Experience. Value in Health. 2023;26(6):S209-S. doi: 10.1016/j.jval.2023.03.2599. PubMed PMID: 164252617.

2135. Alhusein N, Killick K, Macaden L, Smith A, Stoddart K, Taylor A, et al. “We're really not ready for

this”: A qualitative exploration of community pharmacy personnel's perspectives on the pharmaceutical care of older people with sensory impairment. Disability and Health Journal. 2019;12(2):242-8. doi: https://doi.org/10.1016/j.dhjo.2018.10.006.

2136. Alhusein N, Macaden L, Smith A, Stoddart KM, Taylor AJ, Killick K, et al. 'Has she seen me? ': A multiple methods study of the pharmaceutical care needs of older people with sensory impairment in Scotland. BMJ Open. 2018;8(8). doi: 10.1136/bmjopen-2018-023198.

2137. Alhusein N, Watson MC. Quality indicators and community pharmacy services: a scoping review. International Journal of Pharmacy Practice. 2019;27(6):490-500.

2138. Alhussain K, Almuayli DI, Aldaej SM. Factors associated with the use of over-the-counter sleep aids containing diphenhydramine: A cross-sectional study in Saudi Arabia. Saudi Pharmaceutical Journal. 2023;31(8):101679. doi: https://doi.org/10.1016/j.jsps.2023.06.012.

2139. Ali A. Role of Community Pharmacies and Assessment of Occupational Violence Against Him in Saudi Arabia: A Cross Sectional Study Design. Latin American Journal of Pharmacy. 2023;42(5):1067-73.

2140. Ali AK. Inventory management in pharmacy practice: a review of literature. Archives of pharmacy practice. 2011;2(4):151.

2141. Ali Al-Worafi YM. Pharmacy Practice in Yemen. Pharmacy Practice in Developing Countries: Achievements and Challenges2016. p. 267-87.

2142. Ali EM, Alqahtani SS, Syed NK, Banji OJF, Mashragi YA, Ageel MA, et al. Self-reported and the actual involvement of community pharmacy professionals in the management of acute childhood diarrhea in Jazan Province, Saudi Arabia: Simulated patient visits and survey study. Saudi Pharmaceutical Journal. 2022;30(10):1497-506. doi: 10.1016/j.jsps.2022.07.012. PubMed PMID: WOS:000885950600014.

2143. Ali Fathi TH, Miraj SA. Impact of knowledge, attitude and practice on medication errors and safety improvement in pharmacy Departments of King Saud University Medical City Riyadh Saudi Arabia. Biomedical and Pharmacology Journal. 2021;14(2):803-13. doi: 10.13005/bpj/2183.

2144. Ali FRM, Neff L, Wang X, Hu SS, Schecter A, Mahoney M, et al. Tobacco-Free Pharmacies and U.S. Adult Smoking Behavior: Evidence From CVS Health's Removal of Tobacco Sales. American Journal of Preventive Medicine. 2020;58(1):41-9. doi: https://doi.org/10.1016/j.amepre.2019.09.003.

2145. Ali H, Rafi SMT, Zafar F, Akram S, Zaheer E, Bushra R, et al. Method development and validation studies for gemifloxacin in vitro release profile: An application to quality attributes. Latin American Journal of Pharmacy. 2020;39(4):812-22.

2146. Ali H, Zafar F, Alam S, Mallick N, Hasnain H, Naqvi GR, et al. Integration of community setup in pharmaceutical care: Current challenges, perception, facts and opportunities in Pakistan. International Journal of Pharmacy and Pharmaceutical Sciences. 2016;8(10):138-43. doi: 10.22159/ijpps.2016v8i10.13169.

2147. Ali HS, Aldahab AS, Mohamed EB, Prajapati SK, Badullas WFS, Alshakka M, et al. Patients' Perspectives on Services Provided by Community Pharmacies in Terms of Patients' Perception and Satisfaction. Journal of Young Pharmacists. 2019;11(3):279-84. doi: 10.5530/jyp.2019.11.56. PubMed PMID: WOS:000478765500009.

2148. Ali I, Khan TM. Potential for community pharmacies to promote rational drug use in Pakistan. Research In Social & Administrative Pharmacy. 2016;12(1):167-8. PubMed PMID: 26443471.

2149. Ali K, Al-Quteimat O, Naseem R, Malhi SM, Wajdi M, Jahan N, et al. Incorporating a clinical oncology pharmacist into an ambulatory care pharmacy in pediatric hematology-oncology and transplant clinic: Assessment and significance. Journal of Oncology Pharmacy Practice. 2021;27(4):815-20. doi: 10.1177/1078155220934167. PubMed PMID: WOS:000546348200001.

2150. Ali K, Mensah EA, McDermott EA, Kirkham FA, Stevenson J, Hamer V, et al. Implementation of a medicine management plan (MMP) to reduce medication-related harm (MRH) in older people post-hospital discharge: a randomised controlled trial. BMC geriatrics. 2022;22(1):850. doi: 10.1186/s12877-022-03555-w. PubMed PMID: CN-02495712.

2151. Ali M, Dewy K, Phillips J, Quan D. Impact of Pre-Transplant Pharmacist Evaluations on Hepatitis B and Herpes Zoster Vaccination Rates in Solid Organ Transplant Recipients. American Journal of Transplantation. 2023;23(6 Supplement 1):S948. doi: https://dx.doi.org/10.1016/j.ajt.2023.05.014. PubMed PMID: 2027909743.

2152. Ali M, Schifano F, Robinson P, Phillips G, Doherty L, Melnick P, et al. Impact of community pharmacy diabetes monitoring and education programme on diabetes management: a randomized controlled study. Diabetic medicine. 2012;29(9):e326-33. doi: 10.1111/j.1464-5491.2012.03725.x. PubMed PMID: CN-00868468.

2153. Ali M, Schifano F, Robinson P, Phillips G, Doherty L, Melnick P, et al. Improvement in cardiovascular risk factors in patients with diabetes by a community pharmacy education and monitoring project. Journal of Human Hypertension. 2012;26(10):627-8. PubMed PMID: WOS:000308688700029.

2154. Ali MAS, Amirthalingam P, Alatawi Y, Aljabri A. Patient Satisfaction of Ambulatory Care Pharmacy Services. Journal of Patient Experience. 2022;9. doi: 10.1177/23743735221112631. PubMed PMID: WOS:000827524300001.

2155. Ali MAS, Khedr EMH, Ahmed FAH, Mohamed NNE. Clinical pharmacist interventions in managing drug-related problems in hospitalized patients with neurological diseases. International Journal of Clinical Pharmacy. 2018;40(5):1257-64. doi: 10.1007/s11096-018-0658-0. PubMed PMID: 132697454. Language: English. Entry Date: 20190313. Revision Date: 20210111. Publication Type: journal article.

2156. Ali MD, Ahmad A, Banu N, Kannan LS. Use of Proton Pump Inhibitors in Saudi Arabia: A Cross-Sectional Retrospective Drug Utilization Study. Journal of Pharmaceutical Research International. 2020;32(31):48-54. doi: 10.9734/JPRI/2020/v32i3130918. PubMed PMID: WOS:000595023800007.

2157. Ali MD, Al Mubarak FE, Ghosn SA, Ahmad A, Alhussini SH, Almozien SF, et al. Knowledge, Attitude, and the Practice of Community Pharmacists to Dispense Opioid-Related and Opioid-Containing Medication in Eastern Province, Saudi Arabia. Journal of Pharmacy and Bioallied Sciences. 2023;15(3):152-7. doi: 10.4103/jpbs.jpbs_349_23.

2158. Ali MD, Patel M, Banu N, Ahmad A, Hassan YA. A retrospective study on drug utilization pattern and cos utility analysis of antifungal drugs. Journal of Pharmaceutical Health Services Research. 2021;12(1):94-7. doi: 10.1093/jphsr/rmaa023. PubMed PMID: WOS:000651819800016.

2159. Ali MD, Patel M, Banu N, Ahmad A, Hassan YA. Drug utilization pattern and cost-effectiveness analysis of five most common antibiotics used in Saudi Arabia. Journal of Pharmaceutical Health Services Research. 2021;12(2):242-6. doi: 10.1093/jphsr/rmaa019. PubMed PMID: WOS:000661395600019.

2160. Ali MM, Al-Jumaili AA. Appraising the role of pharmacists in medication reconciliation at hospital discharge: A field-based study. Al-Rafidain Journal of Medical Sciences (ISSN 2789-3219). 2023;5(1S):S57-63.

2161. Ali N, Ashiru-Oredope D, Murdan S. Training university students as vaccination champions to promote vaccination in their multiple identities and help address vaccine hesitancy. Pharmacy Education. 2021;21(1):407-19. doi: 10.46542/pe.2021.211.407419. PubMed PMID: WOS:000691178800006.

2162. Ali NH, Arbab AH. Awareness and knowledge of autism spectrum disorders among community pharmacists in khartoum state(Sudan), 2018. Acta Pharmaceutica Sciencia. 2021;59(2):291-304. doi: 10.23893/1307-2080.APS.05917.

2163. Ali S, Aboheimed N, Al-Zaagi I, Al-Dossari D, Aboheimed NI, Al-Zaagi IA, et al. Analysis of medication errors at a large tertiary care hospital in Saudi Arabia: a retrospective analysis. International Journal of Clinical Pharmacy. 2017;39(5):1004-7. doi: 10.1007/s11096-017-0514-7. PubMed PMID: 126215645. Language: English. Entry Date: 20180723. Revision Date: 20181001. Publication Type: journal article.

2164. Ali S, Aboheimed NI, Al-Zaagi IA, Al-Dossari DS. Analysis of medication errors at a large tertiary care hospital in Saudi Arabia: a retrospective analysis. International Journal of Clinical Pharmacy. 2017;39(5):1004-7. PubMed PMID: 28748359.

2165. Ali S, Ali M, Paudyal V, Rasheed F, Ullah S, Haque S, et al. A randomized controlled trial to assess the impact of clinical pharmacy interventions on treatment outcomes, health related quality of life and medication adherence among Hepatitis C patients. Patient preference and adherence. 2019;13:2089-100. doi: 10.2147/PPA.S224937. PubMed PMID: CN-02076272.

2166. Ali S, Curtain CM, Peterson GM, Salahudeen MS. Exploring Australian pharmacists' views toward reducing the risk of medicines-related harm in aged care residents. Pharmacology research & perspectives. 2023;11(3):e01104. doi: https://dx.doi.org/10.1002/prp2.1104. PubMed PMID: 641398241.

2167. Ali S, Curtain CM, Peterson GM, Salahudeen MS. Exploring Australian pharmacists' perceptions and practices towards reducing the risk of medicines-related harm in aged care residents. Frontiers in Pharmacology. 2023;14:1131456. doi: https://dx.doi.org/10.3389/fphar.2023.1131456. PubMed PMID: 2022074128.

2168. Ali S, Shimels T, Bilal AI. Assessment of Patient Counseling on Dispensing of Medicines in Outpatient Pharmacy of Tikur-Anbessa Specialized Hospital, Ethiopia. Ethiopian Journal of Health Sciences. 2019;29(6):727-36. doi: 10.4314/ejhs.v29i6.9. PubMed PMID: WOS:000497960000009.

2169. Ali SE, Ibrahim MI, Palaian S. Medication storage and self-medication behaviour amongst female students in Malaysia. Pharmacy Practice. 2010;8(4):226-32.

2170. Ali U, Pasha SK, Patel R, Schmidt HM, Altaf A. Assessment of Acceptability of Pre-exposure Prophylaxis for HIV Prevention Among Men Who Sex with Men and Transgender Community in Pakistan. Jcpsp-Journal of the College of Physicians and Surgeons Pakistan. 2023;33(9):983-9. doi: 10.29271/jcpsp.2023.09.983. PubMed PMID: WOS:001128240600026.

2171. Ali ZT, Al Saadi MTA, Al Saadi NTA. Incidence of stroke with uncontrolled DM: effect of pharmaceutical care intervention to DM type-2 patients in Iraqi hospitals. Annals of tropical medicine and public health. 2019;22(8). doi: 10.36295/ASRO.2019.220823. PubMed PMID: CN-02086310.

2172. Alibaud R, Metadier De Saint-Denis A, Metadier De Saint-Denis D, Ruiz I, Lavaud K. Conciliation médicamenteuse et non médicamenteuse avec processus du parcours patient en consultation douleur chronique. Douleurs : Evaluation - Diagnostic - Traitement. 2016;17(4):205-10. doi: https://doi.org/10.1016/j.douler.2016.03.008.

2173. Alicic RZ, Short RA, Corbett CL, Neumiller JJ, Gates BJ, Daratha KB, et al. Medication Intervention for Chronic Kidney Disease Patients Transitioning from Hospital to Home: study Design and Baseline Characteristics. American journal of nephrology. 2016;44(2):122-9. doi: 10.1159/000447019. PubMed PMID: CN-01209864.

2174. Alicic RZ, Short RA, Corbett CL, Neumiller JJ, Gates BJ, Daratha KB, et al. Medication Intervention for Chronic Kidney Disease Patients Transitioning from Hospital to Home: Study Design and Baseline Characteristics. American Journal of Nephrology. 2016;44(2):122-9. PubMed PMID: 27487357.

2175. Alikhan R, Ahmad S, Groves T, Holroyd S, Dyer R, Holloway M, et al. Cardiff and Vale University Health Board Atrial Fibrillation Project "Stop A Stroke (SAS): who cares wins". British journal of haematology Conference: 57th annual scientific meeting of the british society for haematology United kingdom. 2017;176:124. doi: 10.1111/bjh.14613. PubMed PMID: CN-01474878.

2176. Alili-Idrizi E, Dauti M, Malaj L. Validation of the parental knowledge and attitude towards antibiotic usage and resistance among children in Tetovo, the Republic of Macedonia. Pharmacy Practice. 2014;12(4). doi: 10.4321/S1886-36552014000400003.

2177. Alipour F, Jamshidizadeh S, Bastani P, Mehralian G. The balanced scorecard as a strategic management tool in hospital pharmacies: an experimental study. Journal of Health Organization and Management. 2022;36(6):767-80. doi: 10.1108/JHOM-07-2021-0256.

2178. Alipour J, Mehdipour Y, Karimi A, Khorashadizadeh M, Akbarpour M. Security, confidentiality, privacy and patient safety in the hospital information systems from the users' perspective: A cross-sectional study. International Journal of Medical Informatics. 2023;175:105066. doi: https://doi.org/10.1016/j.ijmedinf.2023.105066.

2179. Alison C, Anselm S. The effectiveness of diabetes medication therapy adherence clinic to improve glycaemic control among patients with type 2 diabetes mellitus: a randomised controlled trial. Medical journal of Malaysia. 2020;75(3):246-53. PubMed PMID: CN-02130719.

2180. Alix L, Bajeux E, Hubert J, Cador B, Josselin JM, Tuffier S, et al. Medication reconciliation in hospital patients over the age of 65: How long does it take and how much does it cost? A time-motion study in an internal medicine ward. European Journal of Internal Medicine. 2020;73:100-2. PubMed PMID: 31859024.

2181. Alizadeh EA, Graf K, Schiwon J, Trautmann T, Krause F, Mayer W, et al. Thirty years of telemetry-based data acquisition for cardiovascular drug safety evaluation: Applications and optimization. Journal of Pharmacological & Toxicological Methods. 2023;122:N.PAG-N.PAG. doi: 10.1016/j.vascn.2023.107279. PubMed PMID: 164857434.

2182. Aljadhey H, Alhusan A, Alburikan K, Adam M, Murray MD, Bates DW. Medication safety practices in hospitals: A national survey in Saudi Arabia. Saudi Pharmaceutical Journal. 2013;21(2):159-64. doi: https://doi.org/10.1016/j.jsps.2012.07.005.

2183. Aljadhey H, Alkhani S, Khan TM. Medication safety officer preparatory course: Outcomes and experiences. Saudi Pharmaceutical Journal. 2017;25(7):1011-4. doi: https://doi.org/10.1016/j.jsps.2017.03.002.

2184. Aljadhey H, Assiri G, Adam M. Safety of Non-Prescription Medications: Knowledge and Attitude of Community Pharmacy Customers in Saudi Arabia. Pharmacoepidemiology and Drug Safety. 2012;21:227-. PubMed PMID: WOS:000308131701468.

2185. Aljadhey H, Mahmoud MA, Alshammari TM, Al-Dhaeefi M, Louet HL, Perez-Gutthann S, et al. A

qualitative exploration of the major challenges facing pharmacovigilance in Saudi Arabia. Saudi Medical Journal. 2015;36(9):1097-102. doi: 10.15537/smj.2015.9.12125.

2186. Aljadhey H, Mahmoud MA, Hassali MA, Alrasheedy A, Alahmad A, Saleem F, et al. Challenges to and the future of medication safety in Saudi Arabia: A qualitative study. Saudi Pharmaceutical Journal. 2014;22(4):326-32.

2187. Aljadhey H, Mahmoud MA, Mayet A, Alshaikh M, Ahmed Y, Murray MD, et al. Incidence of adverse drug events in an academic hospital: a prospective cohort study. International Journal for Quality in Health Care. 2013;25(6):648-55. doi: 10.1093/intqhc/mzt075. PubMed PMID: 104120196. Language: English. Entry Date: 20140829. Revision Date: 20200708. Publication Type: journal article.

2188. Aljamal MS, Ashcroft D, Tully MP. Development of indicators to assess the quality of medicines reconciliation at hospital admission: an e- Delphi study. International Journal of Pharmacy Practice. 2016;24(3):209-16. doi: 10.1111/ijpp.12234. PubMed PMID: 115295261. Language: English. Entry Date: 20160513. Revision Date: 20180629. Publication Type: Article.

2189. Aljauid AS, Al-Otaibi AO, Aldawood MM, Mohamed RN, Basha S, Al Thomali Y. Oral Health Behavior of Medical, Dental, and Pharmacology Students in Taif University: A Cross-sectional Study. Journal of Advanced Oral Research. 2020;11(1):83-92. doi: 10.1177/2320206820918746. PubMed PMID: WOS:000539261500012.

2190. Aljuffali LA, Alnaim L, Alfakhri A, Alrasheed HH, Alkherb Z, Almalag HM. Assessing Nontechnical Skills in Senior Pharmacy Students Using an Innovative Simulation Setting. American Journal of Pharmaceutical Education. 2023;87(5):100047. doi: https://doi.org/10.1016/j.ajpe.2022.12.008.

2191. Aljuffali LA, Alshabanah MO, Almalag HM. Cross-sectional study to evaluate burnout among pharmacy staff in Saudi Arabia during COVID-19 pandemic. Saudi Pharmaceutical Journal. 2022;30(4):440-53. doi: https://doi.org/10.1016/j.jsps.2022.01.017.

2192. Aljumah K. Impact of pharmacist intervention using shared decision making on adherence and measurable depressed patient outcomes. Value in health. 2016;19(3):A19-. PubMed PMID: CN-01167816.

2193. Aljumah K, Hassali M. Impact of pharmacist intervention using shared decision making on adherence and measurable depressed patient outcomes. European journal of hospital pharmacy. 2015;22:A6-A7. doi: 10.1136/ejhpharm-2015-000639.15. PubMed PMID: CN-01334317.

2194. Aljumah K, Hassali MA. Impact of pharmacist intervention on adherence and measurable patient outcomes among depressed patients: a randomised controlled study. BMC psychiatry. 2015;15:219. doi: 10.1186/s12888-015-0605-8. PubMed PMID: CN-01169822.

2195. Aljumah K, Nwaf A, Amed A, Alhoutan A, Aldhiab M. The impact of a decision aid on depressed patient's involvement in shared decision making: a pilot randomised controlled double blind study. European journal of hospital pharmacy. 2016;23:A9-A10. doi: 10.1136/ejhpharm-2016-000875.21. PubMed PMID: CN-01334031.

2196. Alkabbani W, Gamble JM. Active-comparator restricted disproportionality analysis for pharmacovigilance signal detection studies of chronic disease medications: An example using sodium/ glucose cotransporter 2 inhibitors. British Journal of Clinical Pharmacology. 2023;89(2):431-9. doi: 10.1111/ bcp.15178. PubMed PMID: 161395058.

2197. Alkadhimi A, Dawood OT, Hassali MA. Dispensing of antibiotics in community pharmacy in Iraq: a qualitative study. Pharmacy Practice. 2020;18(4):2095. PubMed PMID: 33343772.

2198. Alkadhimi A, Dawood OT, Hassali MAA. Dispensing of antibiotics in community pharmacy in Iraq: A qualitative study. Pharmacy Practice. 2020;18(4):1-9. doi: 10.18549/PharmPract.2020.4.2095.

2199. Alkayyal N, Cheema E, Hadi MA. Perspective of Saudi undergraduate pharmacy students on pharmacovigilance and adverse drug reaction reporting: A National Survey. Currents in Pharmacy Teaching and Learning. 2017;9(5):779-85. doi: 10.1016/j.cptl.2017.05.016.

2200. Alkema GE. Translating research into practice: A community-based medication management intervention [Ph.D.]. United States -- California: University of Southern California; 2007.

2201. Alkema GE, Enguidanos SM, Wilber KH, Trufasiu M, Simmons WJ, Frey D. The role of consultant pharmacists in reducing medication problems among older adults receiving Medicaid waiver services. Consultant Pharmacist. 2009;24(2):121-33. PubMed PMID: 19275454.

2202. Alkema GE, Enguidanos SM, Wilber KH, Trufasiu M, Simmons WJ, Frey D, et al. The role of consultant pharmacists in reducing medication problems among older adults receiving Medicaid waiver services. Consultant Pharmacist. 2009;24(2):121-33. PubMed PMID: 105472706. Language: English. Entry Date: 20090417. Revision Date: 20191116. Publication Type: journal article.

2203. Alkhalid Z, Birand N. Determination and comparison of potential drug-drug interactions using three different databases in northern cyprus community pharmacies. Nigerian Journal of Clinical Practice. 2022;25(12):2005-9. doi: 10.4103/njcp.njcp_448_22.

2204. Alkhalid ZN, Birand N. Determination and comparison of potential drug-drug interactions using three different databases in northern cyprus community pharmacies. Nigerian Journal of Clinical Practice. 2022;25(12):2005-9. doi: 10.4103/njcp.njcp_448_22. PubMed PMID: WOS:000904875900010.

2205. Alkhalidi DK, Jawad RM, Alsamak MJ, Ahmed MM, Aslam A. An exploration of individual knowledge and behavior for utilizing OTC drugs and dietary supplements for health enhancement: An empirical analysis from Dubai. Pharmacy Practice. 2021;19(4). doi: 10.18549/PharmPract.2021.4.2564.

2206. Alkhalili M, Ma J, Grenier S. Defining roles for pharmacy personnel in disaster response and emergency preparedness. Disaster Medicine and Public Health Preparedness. 2017;11(4):496-504. doi: 10.1017/dmp.2016.172.

2207. Alkhani S, Ahmed Y, Bin-Sabbar N, Almogirah H, Alturki A, Albanyan H, et al. Current practices for labeling medications in hospitals in Riyadh, Saudi Arabia. Saudi Pharmaceutical Journal. 2013;21(4):345-9. doi: 10.1016/j.jsps.2012.12.002. PubMed PMID: WOS:000325516700003.

2208. Alkharfy K. Community pharmacists' knowledge, attitudes and practices towards herbal remedies in Riyadh, Saudi Arabia. EMHJ-Eastern Mediterranean Health Journal, 16 (9), 988-993, 2010. 2010.

2209. Alkharfy KM. Community pharmacists' knowledge, attitudes and practices towards herbal remedies in Riyadh, Saudi Arabia. Eastern Mediterranean Health Journal. 2010;16(9). doi: 10.26719/2010.16.9.988.

2210. Alkhateeb FM, Clauson KA, McCafferty R, Latif DA. Physician attitudes toward pharmacist provision of medication therapy management services. Pharmacy World & Science. 2009;31(4):487-93. PubMed PMID: 19472070.

2211. Alkhateeb FM, Shields KM, Broedel-Zaugg K, Bryan A, Snell J. Credentialing of pharmacy technicians in the USA. International Journal of Pharmacy Practice. 2011;19(4):219-27.

2212. Alkhateeb FM, Unni E, Latif D, Shawaqfeh MS, Al-Rousan RM. Physician attitudes toward

collaborative agreements with pharmacists and their expectations of community pharmacists' responsibilities in West Virginia. Journal of the American Pharmacists Association. 2009;49(6):797-803a. doi: https://doi.org/10.1331/JAPhA.2009.08111.

2213. AlKhatib HS, Jalouqa S, Maraqa N, Ratka A, Elayeh E, Al Muhaissen S. Prevalence, determinants, and characteristics of extemporaneous compounding in Jordanian pharmacies. BMC health services research. 2019;19(1):816. doi: 10.1186/s12913-019-4684-y. PubMed PMID: CN-02005328.

2214. Alkhatib L, Parsons R, Czarniak P, Sunderland VB. An evaluation of the reclassification of ophthalmic chloramphenicol for the management of acute bacterial conjunctivitis in community pharmacies in Western Australia. International Journal of Pharmacy Practice. 2015;23(2):111-20. doi: 10.1111/ijpp.12119. PubMed PMID: WOS:000351178900004.

2215. Alkherat AM, Alkhalidi DK. Assessment of knowledge and counseling practice of warfarin among pharmacists in UAE: A cross-sectional study. Pharmacy Practice. 2022;20(4). doi: 10.18549/PharmPract.2022.4.2741.

2216. Alkhudair N, Alshamrani M, Samarkandi H, Almodaheem H, Alabdulkarim H, Alsaqaaby M, et al. Cancer Management in Saudi Arabia: Recommendations by the Saudi Oncology HeAlth Economics ExpeRt GrouP (SHARP). Saudi Pharmaceutical Journal. 2021;29(2):115-20. doi: https://doi.org/10.1016/j.jsps.2020.12.005.

2217. Alkhuzaee FS, Almalki HM, Attar AY, Althubiani SI, Almuallim WA, Cheema E, et al. Evaluating community pharmacists' perspectives and practices concerning generic medicines substitution in Saudi Arabia: A cross-sectional study. Health Policy. 2016;120(12):1412-9. doi: https://doi.org/10.1016/j.healthpol.2016.09.018.

2218. Alkoudmani R, Hassali MA, Allela O, Elkalmi R, Al-Essa RK. Acceptance of Pharmacist's Extended Roles by other Healthcare Providers in the Arab Region: Review Article. Archives of Pharmacy Practice. 2021;12(4):29-34. doi: 10.51847/iHO5cjE9Vn. PubMed PMID: WOS:000734027900005.

2219. Alkoudsi KT, Al-Qudah R, Basheti IA. Assessing the effectiveness of a pharmaceutical care service on the quality of life of women with polycystic ovarian syndrome living in war and non-war countries. Journal of evaluation in clinical practice. 2020;26(5):1467-77. doi: 10.1111/jep.13310. PubMed PMID: CN-02005331.

2220. Alkoudsi KT, Basheti IA. Prevalence of anxiety and depression among women with Polycystic Ovary Syndrome living in war versus non-war zone countries: a randomized controlled trial assessing a pharmacist intervention. Research in social & administrative pharmacy : RSAP. 2020;16(5):689-98. doi: 10.1016/j.sapharm.2019.08.027. PubMed PMID: CN-02228998.

2221. Allabi AC, Kpoviessi C, Kakpo-Zannou R. Contribution du pharmacien à l'optimisation de la pharmacothérapie dans un service de médecine interne au centre hospitalier et universitaire d'Abomey-Calavi/Sô-Ava. Le Pharmacien Hospitalier et Clinicien. 2018;53(1):38-43. doi: https://doi.org/10.1016/j.phclin.2017.09.001.

2222. Allaert F-A. Intérêt des compléments alimentaires dans les hyperlipidémies. Actualités Pharmaceutiques. 2016;55(554):46-9. doi: https://doi.org/10.1016/j.actpha.2016.01.010.

2223. Allaert FA, Villet S, Vincent S, Sauve L. Observational study on the dispensing of cough syrups to children with acute cough by community pharmacists in France. Minerva Pediatrica. 2018;70(2):117-26. doi: 10.23736/s0026-4946.17.04752-1. PubMed PMID: WOS:000432381500002.

2224. Allaertaert FA, Villet S, Vincent S, Sauve L. Observational study on the dispensing of cough syrups to children with acute cough by community pharmacists in France. Minerva Pediatrica. 2018;70(2):117-26. doi: 10.23736/S0026-4946.17.04752-1.

2225. Allaf MSK. Pharmacies and medication information system in Jeddah City, Saudi Arabia [Ph.D.]. England: Loughborough University (United Kingdom); 2009.

2226. Allan EL, Barker KN, Severson RW, Lane JM, Wilson JM, Mangum DA. Design and evaluation of a sterile compounding facility. American Journal of Health-System Pharmacy. 1995;52(13):1421-7. doi: 10.1093/ajhp/52.13.1421.

2227. Allan GM, Lexchin J, Wiebe N. Physician awareness of drug cost: a systematic review. PLoS medicine. 2007;4(9):e283.

2228. Allan J, Nott S, Chambers B, Hawthorn G, Munro A, Doran C, et al. A stepped wedge trial of efficacy and scalability of a virtual clinical pharmacy service (VCPS) in rural and remote NSW health facilities. BMC Health Services Research. 2020;20(1):1-8. doi: 10.1186/s12913-020-05229-y. PubMed PMID: 143038360. Language: English. Entry Date: In Process. Revision Date: 20201125. Publication Type: journal article.

2229. Allan JD, Stanley J, Crabtree MK, Werner KE, Swenson M. Clinical Prevention and Population Health Curriculum Framework: The nursing perspective. Journal of Professional Nursing. 2005;21(5):259-67. doi: 10.1016/j.profnurs.2005.07.006.

2230. Allard B, Grégoire M, Ferrandini C, Ollivier C, Vrtovsnik F, Lamure E, et al. Étude tEmPO : intérêt d'une érythropoïétine à injection mensuelle. Le Pharmacien Hospitalier. 2011;46(1):13-22. doi: https://doi.org/10.1016/j.phhp.2011.01.005.

2231. Allard J, Hébert R, Rioux M, Asselin J, Voyer L. Efficacy of a clinical medication review on the number of potentially inappropriate prescriptions prescribed for community-dwelling elderly people. CMAJ : Canadian Medical Association journal. 2001;164(9):1291-6. PubMed PMID: CN-00326776.

2232. Allard J, Hebert R, Rioux M, Asselin J, Voyer L. Efficacy of a clinical medication review on the number of potentially inapprrropriate prescriptions prescribed for community - dwelling elderly people. CMAJ : Canadian Medical Association journal. 2001;164(9):1291-6. PubMed PMID: CN-00354665.

2233. Allard N, Dev A, Dwyer J, Srivatsa G, Thompson A, Cowie B. Factors associated with poor adherence to antiviral treatment for hepatitis B. Journal of Viral Hepatitis. 2017;24(1):53-8. doi: 10.1111/jvh.12582. PubMed PMID: WOS:000391035200008.

2234. Allard NL, MacLachlan JH, Dev A, Dwyer J, Srivatsa G, Spelman T, et al. Adherence in chronic hepatitis B: associations between medication possession ratio and adverse viral outcomes. Bmc Gastroenterology. 2020;20(1). doi: 10.1186/s12876-020-01219-w. PubMed PMID: WOS:000533265200001.

2235. Alldred D. Prescription reviews can identify medicines-related problems. Pharmacy in Practice. 2006;16(4):103-6.

2236. Alldred DP, Kennedy MC, Hughes C, Chen TF, Miller P. Interventions to optimise prescribing for older people in care homes. Cochrane Database of Systematic Reviews. 2016;(2). doi: 10.1002/14651858.CD009095.pub3. PubMed PMID: CD009095.

2237. Alldred DP, Standage C, Fletcher O, Savage I, Carpenter J, Barber N, et al. The influence of formulation and medicine delivery system on medication administration errors in care homes for older people. BMJ Quality & Safety. 2011;20(5):397-401. doi: 10.1136/bmjqs.2010.046318. PubMed PMID:

104660730. Language: English. Entry Date: 20110812. Revision Date: 20200708. Publication Type: Journal Article.

2238. Alldred DP, Zermansky AG, Petty DR, Raynor DK, Freemantle N, Eastaugh J, et al. Clinical medication review by a pharmacist of elderly people living in care homes: pharmacist interventions. International journal of pharmacy practice. 2007;15(2):93-9. doi: 10.1211/ijpp.15.2.0003. PubMed PMID: CN-01412690.

2239. Allegaert K, Sherwin C. Neonates and medicines: a roadmap to further improve neonatal pharmaceutical care. European Journal of Pediatrics. 2016;175(6):743-6. doi: 10.1007/s00431-015-2686-2. PubMed PMID: 115376774. Language: English. Entry Date: 20170710. Revision Date: 20171007. Publication Type: editorial. Journal Subset: Biomedical.

2240. Allemann SS, Bornand D, Hug B, Hersberger KE, Arnet I. Issues around the Prescription of Half Tablets in Northern Switzerland: The Irrational Case of Quetiapine. BioMed Research International. 2015;2015. doi: 10.1155/2015/602021.

2241. Allen AS, Sequist TD. Pharmacy dispensing of electronically discontinued medications. Annals of internal medicine. 2012;157(10):700-5.

2242. Allen D. Managing medicines: 10 things to know: Safety in this area is key to becoming a competent nurse. Experts offer tips for students on gaining confidence and avoiding mistakes. Nursing Standard. 2023;38(7):41-2. doi: 10.7748/ns.38.7.41.s19. PubMed PMID: 164708393.

2243. Allen DD, Jaffe L, Pfleghaar A. Medication Management, Mild Cognitive Impairment and Occupational Therapy: A Scoping Review. Physical & Occupational Therapy in Geriatrics. 2023;41(1):31-45. doi: 10.1080/02703181.2022.2079800. PubMed PMID: 161832362.

2244. Allen EN, Wiyeh A, McCaul M. Adding rapid diagnostic tests to community-based programmes for treating malaria. Cochrane Database of Systematic Reviews. 2022;(9). doi: 10.1002/14651858.CD009527.pub3. PubMed PMID: CD009527.

2245. Allen EN, Wiyeh AB, McCaul M. Adding rapid diagnostic tests to community-based programmes for treating malaria. Cochrane Database of Systematic Reviews. 2022;2022(9). doi: 10.1002/14651858.CD009527.pub3.

2246. Allen FC. Temporal trends in prescribing of anti -inflammatory medications for asthma management [Ph.D.]. United States -- Maryland: The Johns Hopkins University; 2000.

2247. Allen GEK, Kim BSK, Smith TB, Hafoka O. Counseling Attitudes and Stigma Among Polynesian Americans. Counseling Psychologist. 2016;44(1):6-27. doi: 10.1177/0011000015618762. PubMed PMID: WOS:000367851300001.

2248. Allen JM, Cavanaugh TM, Hathorn T, Awunti M, Whitner C, Venugopalan V, et al. Exploring Trends in Social Vulnerability for Pharmacy Students at a Large Public University. American Journal of Pharmaceutical Education. 2023;87(12):100551. doi: https://doi.org/10.1016/j.ajpe.2023.100551.

2249. Allen L, Ansel HC. Ansel's pharmaceutical dosage forms and drug delivery systems: Lippincott Williams & Wilkins; 2013.

2250. Allen LV. The art, science, and technology of pharmaceutical compounding: American Pharmaceutical Association Washington, DC; 1998.

2251. Allen LV, Jr., Okeke CC. Basics of compounding: the responsibilities of compounding personnel in implementing United States Pharmacopeia Chapter 797 pharmaceutical compounding--sterile preparations, part 2. International Journal of Pharmaceutical Compounding. 2007;11(4):314-23. PubMed PMID: 105905581. Language: English. Entry Date: 20080502. Revision Date: 20150711. Publication Type: Journal Article.

2252. Allen M, Farris K. A practical approach to documenting pharmacists' interventions. Canadian Pharmaceutical Journal. 1999;132(10):36-42.

2253. Allen MB. Medical pluralism in operation in a squatter settlement (<i>gecekondu</i>) in Istanbul: Tradition, exchange and agency [Ph.D.]. United States -- Florida: University of Florida; 2002.

2254. Allen N, Downs GE. The Pharmacist as Physician Extender in Family Medicine Office Practice. Journal of the American Pharmacists Association. 2006;46(1):77-83. doi: https://doi.org/10.1331/154434506775268599.

2255. Allen RM. When peril responds to plague: predatory journal engagement with COVID-19. Library Hi Tech. 2021;39(3):746-60. doi: 10.1108/lht-01-2021-0011. PubMed PMID: WOS:000627045300001.

2256. Allen SJ, Christensen D, Clark TR, Eckel F, Gouveia WA, Hay JW, et al. Advancing pharmacy practice through research: A 2004 perspective. Journal of the American Pharmacists Association. 2004;44(5):621-8. doi: 10.1331/1544-3191.44.5.621.Allen.

2257. Allen T, Islam M, Abusyed M, Phalla S, Pokharel M, Watarai H, et al. Leveraging technology to advance patient care in the developing world: A new community platform for pharmacy workforce education. Pharmacy Education. 2023;23(3):47-8. doi: https://dx.doi.org/10.46542/pe.2023.233.154. PubMed PMID: 642265869.

2258. Allen V, Davis L, Wirawan J. Expanding clinical service of anticoagulation pharmacists to diabetes medication management Ashley. JACCP Journal of the American College of Clinical Pharmacy. 2018;1(2):299-300. PubMed PMID: 625879682.

2259. Allen-Ramey FC, Anstatt DT, Sajjan SG, Markson LE. Asthma-related health care resource use among patients starting fluticasone or montelukast therapy. Pharmacotherapy. 2005;25(12 I):1752-60. doi: 10.1592/phco.2005.25.12.1752.

2260. Allen-Ramey FC, Bukstein D, Luskin A, Sajjan SG, Markson LE. Administrative claims analysis of asthma-related health care utilization for patients who received inhaled corticosteroids with either montelukast or salmeterol as combination therapy. Journal of Managed Care Pharmacy. 2006;12(4):310-21. doi: 10.18553/jmcp.2006.12.4.310. PubMed PMID: WOS:000237832600003.

2261. Allen-Ramey FC, Markson LE, Riedel AA, Sajjan S, Weiss KB. Patterns of asthma-related health care resource use in children treated with montelukast or fluticasone. Current medical research and opinion. 2006;22(8):1453-61. PubMed PMID: CN-00591931.

2262. Allende Bandres MA, Arenere Mendoza M, Gutierrez Nicolas F, Calleja Hernandez MA, Ruiz La Iglesia F. Pharmacist-led medication reconciliation to reduce discrepancies in transitions of care in Spain. International Journal of Clinical Pharmacy. 2013;35(6):1083-90. PubMed PMID: 23881347.

2263. Allende Bandrés MA, Arenere Mendoza M, Gutiérrez Nicolás F, Calleja Hernández MA, Ruiz La Iglesia F, Allende Bandrés MÁ, et al. Pharmacist-led medication reconciliation to reduce discrepancies in transitions of care in Spain. International Journal of Clinical Pharmacy. 2013;35(6):1083-90. doi: 10.1007/s11096-013-9824-6. PubMed PMID: 104122475. Language: English. Entry Date: 20140912. Revision Date:

20211029. Publication Type: journal article.

2264. Allenet B. Pour que l'éducation thérapeutique du patient devienne pour le pharmacien une posture de travail. Annales Pharmaceutiques Françaises. 2012;70(1):53-7. doi: https://doi.org/10.1016/j.pharma.2011.11.006.

2265. Allenet B, Baudrant-Boga M, Bedouch P, Calop J, Foroni L. Le pharmacien a-t-il une place au sein de l'unité de soins ? Expérience du centre hospitalier universitaire de Grenoble: A pharmacist inside the care unit: Current data and experience of the Grenoble university hospital. Médecine des Maladies Métaboliques. 2009;3(4):442-7. doi: https://doi.org/10.1016/S1957-2557(09)72414-9.

2266. Allenet B, Bedouch P, Bourget S, Baudrant M, Foroni L, Calop J, et al. Physicians' perception of CPOE implementation. International Journal of Clinical Pharmacy. 2011;33(4):656-64. doi: 10.1007/s11096-011-9521-2. PubMed PMID: 104574261. Language: English. Entry Date: 20120608. Revision Date: 20211028. Publication Type: journal article.

2267. Allenet B, Bosson A, Baran N, Merle R, Bardet JD. Pharmaceutical interview training during COVID: Feedback from a remote experience with expert patients. Pharmacien Hospitalier et Clinicien. 2021. doi: 10.1016/j.phclin.2021.06.004.

2268. Allesø M, van den Berg F, Cornett C, Jørgensen FS, Halling-Sørensen B, de Diego HL, et al. Solvent Diversity in Polymorph Screening. Journal of Pharmaceutical Sciences. 2008;97(6):2145-59. doi: https://doi.org/10.1002/jps.21153.

2269. Alley A, Dorscheid H, Hentzen K. Pharmacist-Run Medication Reconciliation for Veterans Admitted to Non-Veterans Affairs Hospice Care. The Senior Care Pharmacist. 2021;36(1):42-8. PubMed PMID: 33384033.

2270. Alley L, Novak K, Havlin T, Irwin AN, Carson J, Johnston K, et al. Development and pilot of a prescription drug monitoring program and communication intervention for pharmacists. Research in Social and Administrative Pharmacy. 2020;16(10):1422-30. doi: https://doi.org/10.1016/j.sapharm.2019.12.023.

2271. Allibone L. Colour-coding can't beat critical thinking: Could what might seem like a safety measure in fact increase our reliance on assumption and, therefore, the risk of medication errors? Nursing Standard. 2023;38(4):13-. doi: 10.7748/ns.38.4.13.s10. PubMed PMID: 173147751.

2272. Allin S, Laporte A. Socioeconomic Status and the Use of Medicines in the Ontario Public Drug Program. Canadian Public Policy-Analyse De Politiques. 2011;37(4):563-76. PubMed PMID: WOS:000298709100007.

2273. Allinson TT, Szeinbach SL, Schneider PJ. Perceived accuracy of drug orders transmitted orally by telephone. American journal of health-system pharmacy. 2005;62(1):78-83.

2274. Allinson Y. Evaluate QUM performance indicators. Australian Journal of Pharmacy. 2008;89(1064):25.

2275. Allison DG. De-mystifying science: Engaging the community to make a difference. Pharmacy Education. 2016;16(1):118-21.

2276. Allison KC, Chao AM, Bruzas MB, McCuen-Wurst C, Jones E, McAllister C, et al. A pilot randomized controlled trial of liraglutide 3.0 mg for binge eating disorder. Obesity science and practice. 2022. doi: 10.1002/osp4.619. PubMed PMID: CN-02427735.

2277. Allison R, Chapman S, Howard P, Thornley T, Ashiru-Oredope D, Walker S, et al. Feasibility of a

community pharmacy antimicrobial stewardship intervention (PAMSI): An innovative approach to improve patients' understanding of their antibiotics. JAC-Antimicrobial Resistance. 2020;2(4). doi: 10.1093/jacamr/dlaa089.

2278. Allison RR, Downie GH, Cuenca R, Hu X-H, Childs CJ, Sibata CH. Photosensitizers in clinical PDT. Photodiagnosis and photodynamic therapy. 2004;1(1):27-42.

2279. Allred K, Rothschild C, Whitworth J, Cassisi JE. Interprofessional Pain Curriculum for an Academic Health Science Center. Pain Management Nursing. 2023;24(2):238. doi: https://doi.org/10.1016/j.pmn.2023.02.019.

2280. Allum L. National Minimum Standards in care homes: What role do pharmacists have? Pharmaceutical Journal. 2004;272(7292):387-90.

2281. Allwood MC. Problems with lines and the pharmacist's role. Nutrition. 1999;15(3):252-3. doi: https://doi.org/10.1016/S0899-9007(98)00185-3.

2282. Ally MZ, Persaud N, Umali N. Evaluation of Pharmacy Model in a Trial of Free Essential Medicine Access. Journal of Primary Care and Community Health. 2020;11. doi: 10.1177/2150132720923938. PubMed PMID: WOS:000537859200001.

2283. Almaawi A, Aloud W, Alshayhan F, Aljedia K, Alaskar F, Alsuleiman O. Awareness about total knee replacement among Saudi population. Journal of Family Medicine and Primary Care. 2022;11(4):1308-13. doi: 10.4103/jfmpc.jfmpc_1437_21. PubMed PMID: WOS:000805379200014.

2284. Almaghaslah D, Alsayari A, Alyahya SA, Alshehri R, Alqadi K, Alasmari S. Using Design Thinking Principles to Improve Outpatients' Experiences in Hospital Pharmacies: A Case Study of Two Hospitals in Asir Region, Saudi Arabia. Healthcare. 2021;9(7). doi: 10.3390/healthcare9070854. PubMed PMID: WOS:000676145300001.

2285. AlMahasis SO, Fox B, Ha D, Qian J, Wang C-h, Westrick SC. Pharmacy-based immunization in rural USA during the COVID-19 pandemic: A survey of community pharmacists from five southeastern states. Vaccine. 2023;41(15):2503-13. doi: https://doi.org/10.1016/j.vaccine.2023.03.002.

2286. Almalag HM, Mansy WH, Alwhaibi AM, Syed W, Babelghaith SD, Al Arifi MN. Community Pharmacists' Knowledge, Willingness, and Readiness to Prescribe Oral Contraceptives in Saudi Arabia. Healthcare. 2022;10(3). doi: 10.3390/healthcare10030503. PubMed PMID: WOS:000775314600001.

2287. Almalag HM, Mansy WH, Alwhaibi AM, Syed W, Babelghaith SD, Arifi MNA. Community Pharmacists' Knowledge, Willingness, and Readiness to Prescribe Oral Contraceptives in Saudi Arabia. Healthcare (Switzerland). 2022;10(3). doi: 10.3390/healthcare10030503.

2288. Almalak H, Albluwi AaI, Alkhelb DA, Alsaleh HM, Khan TM, Hassali MAA, et al. Students' attitude toward use of over the counter medicines during exams in Saudi Arabia. Saudi Pharmaceutical Journal. 2014;22(2):107-12. doi: https://doi.org/10.1016/j.jsps.2013.02.004.

2289. Almalki A, Jambi A, Elbehiry B, Albuti H. Improving Inpatient Medication Dispensing with an Automated System. Global Journal on Quality and Safety in Healthcare. 2023;6(4):117-25. doi: https://dx.doi.org/10.36401/JQSH-23-15. PubMed PMID: 2026758969.

2290. Almalki ZS, Alshehri AM, Alturki LA, Altamimi RA, Albassam AA, Alqurashi MS, et al. Exploring patient-safety culture in the community pharmacy setting: a national cross-sectional study. Postgraduate Medicine. 2021;133(1):57-65. doi: 10.1080/00325481.2020.1806593. PubMed PMID:

WOS:000572227400001.

2291. Almalki ZS, Alshehri AM, Alturki LA, Altamimi RA, Albassam AA, Alqurashi MS, et al. Exploring patient-safety culture in the community pharmacy setting: a national cross-sectional study. Postgraduate Medicine. 2021;133(1):57-65. doi: 10.1080/00325481.2020.1806593.

2292. Almalki ZS, Yue XM, Xia Y, Wigle PR, Guo JJ. Utilization, Spending, and Price Trends for Quinolones in the US Medicaid Programs: 25 Years' Experience 1991-2015. Pharmacoeconomics-Open. 2017;1(2):123-31. doi: 10.1007/s41669-016-0007-y. PubMed PMID: WOS:000464335300006.

2293. Almanasef M. Attitudes of the general public toward community pharmacy services in Saudi Arabia: A cross-sectional study. Frontiers in Public Health. 2023;11. doi: 10.3389/fpubh.2023.1092215. PubMed PMID: WOS:000950246500001.

2294. Almanasef M, Aloarni H. Self- care strategies for the management of primary dysmenorrhea among young women in Asir region, Saudi Arabia: a cross-sectional study. European Review for Medical and Pharmacological Sciences. 2023;27(1):172-8. PubMed PMID: WOS:000925590200023.

2295. Almanasef M, Alqarni H. Self-care strategies for the management of primary dysmenorrhea among young women in Asir region, Saudi Arabia: a cross-sectional study. European Review for Medical and Pharmacological Sciences. 2023;27(1):172-8. doi: 10.26355/eurrev_202301_30869.

2296. Almanasef M, Alqarni H. Self-care strategies for the management of primary dysmenorrhea among young women in Asir region, Saudi Arabia: a cross-sectional study. European Review for Medical and Pharmacological Sciences. 2023;27(1):172-8. doi: 10.26355/eurrev_202301_30869.

2297. Almanasreh E, Moles R, Chen TF. The medication reconciliation process and classification of discrepancies: a systematic review. British journal of clinical pharmacology. 2016;82(3):645-58.

2298. Almanasreh E, Moles R, Chen TF. The medication discrepancy taxonomy (MedTax): The development and validation of a classification system for medication discrepancies identified through medication reconciliation. Research in Social & Administrative Pharmacy. 2020;16(2):142-8. doi: 10.1016/j.sapharm.2019.04.005. PubMed PMID: 141237288. Language: English. Entry Date: In Process. Revision Date: 20210214. Publication Type: journal article. Journal Subset: Biomedical.

2299. Almangour TA, Alenazi B, Ghonem L, Alhifany AA, Aldakheel BA, Alruwaili A. Inhaled colistin for the treatment of nosocomial pneumonia due to multidrug-resistant Gram-negative bacteria: A real-life experience in tertiary care hospitals in Saudi Arabia. Saudi Pharmaceutical Journal. 2020;28(8):1009-13. doi: https://doi.org/10.1016/j.jsps.2020.06.023.

2300. Almansour HA, Aloudah NM, Alhawassi TM, Chaar B, Krass I, Saini B. Health consumer engagement in developing novel preventive health community pharmacy roles in cardiovascular disease in Saudi Arabia: A qualitative analysis. Saudi Pharmaceutical Journal. 2020;28(5):529-37. doi: https://doi.org/10.1016/j.jsps.2020.03.004.

2301. Almansour HA, Aloudah NM, Alhawassi TM, Chaar B, Krass I, Saini B. Physicians' views on cardiovascular disease risk prevention services by pharmacists and potential for collaboration. Exploratory Research in Clinical and Social Pharmacy. 2021;4:100077. doi: https://doi.org/10.1016/j.rcsop.2021.100077.

2302. Almansour HA, Aloudah NM, Alhawassi TM, Chaar B, Krass I, Saini B. Cardiovascular disease risk prevention services by pharmacists in Saudi Arabia: what do policymakers and opinion leaders think? Journal of Pharmaceutical Policy and Practice. 2021;14(1). doi: 10.1186/s40545-021-00319-6.

2303. Almansour HA, Aloudah NM, Alhawassi TM, Mekonnen AB, McNamara K, Chaar B, et al. Cardiovascular disease risk screening by pharmacists in Saudi Arabia: Feasibility and perspectives. Research in Social and Administrative Pharmacy. 2018;14(8):e30. doi: https://doi.org/10.1016/j.sapharm.2018.05.040.

2304. Almansour HA, Chaar B, Saini B. Pharmacists' perspectives about their role in care of patients with diabetes observing Ramadan. Research in Social & Administrative Pharmacy. 2017;13(1):109-22. doi: 10.1016/j.sapharm.2016.02.006. PubMed PMID: WOS:000389863700008.

2305. Almansour HA, Chaar B, Saini B. Perspectives and experiences of patients with type 2 diabetes observing the Ramadan fast. Ethnicity & Health. 2018;23(4):380-96. doi: 10.1080/13557858.2016.1269156. PubMed PMID: WOS:000427053800003.

2306. Almansour HA, Mekonnen AB, Aloudah NM, Alhawassi TM, Mc Namara K, Chaar B, et al. Cardiovascular disease risk screening by pharmacists: a behavior change wheel guided qualitative analysis. Research in Social and Administrative Pharmacy. 2020;16(2):149-59. doi: https://doi.org/10.1016/j.sapharm.2019.04.009.

2307. Almarsdóttir AB, Morgall JM. Technicians or patient advocates? - Still a valid question (results of focus group discussions with pharmacists). Pharmacy World and Science. 1999;21(3):127-31. doi: 10.1023/A:1008641618918.

2308. Almarsdottir AB, Morgall JM. Technicians or patient advocates? still a valid question. Pharmacy World & Science. 1999;21(3):127-31. doi: 10.1023/a:1008641618918. PubMed PMID: WOS:000081076600005.

2309. Almarsdottir AB, Morgall JM, Grimsson A. Cost containment of pharmaceutical use in Iceland: The impact of liberalisation and user charges. Journal of Health Services Research and Policy. 2000;5(2):109-13. doi: 10.1177/135581960000500209.

2310. Almatroudi A. An Investigation of Errors in the Preparation of Injectable Medicines in the Pharmacy Environment and on Hospital Wards [Ph.D.]. England: University of Bath (United Kingdom); 2018.

2311. Almazrou S, Alsahly H, Alwattar H, Alturki L, Alamri M. Ability of Saudi mothers to appropriately and accurately use dosing devices to administer oral liquid medications to their children. Drug, Healthcare and Patient Safety. 2015;7:1-6. PubMed PMID: 601019531.

2312. Almeida A, Caramona M, Figueiredo IV, Ramos F. Herbal products and their interactions with anti-hypertensive and lipid-lowering. Food-Drug Interactions: Pharmacokinetics, Prevention and Potential Side Effects2018. p. 227-50.

2313. Almeida CdS. Revisão da Terapêutica Para o Cancro da Próstata Avançado: Uma Meta-Análise Experiência Profissionalizante Na Vertente de Farmácia Comunitária, Hospitalar e Investigação [M.Sc.]. Portugal: Universidade da Beira Interior (Portugal); 2016.

2314. Almeida GPP. Canábis para Fins Terapêuticos e Respetivo Enquadramento Regulamentar: Experiência Profissionalizante na Vertente de Farmácia Comunitária e Investigação [M.Sc.]. Portugal: Universidade da Beira Interior (Portugal); 2020.

2315. Almeida PO. Perfil de Funções Mentais Globais e Específicas Numa População Idosa Institucionalizada na Região da Beira Interior Norte Pela Aplicação da CIF: Um Estudo Descritivo: Experiência Profissionalizante na Vertente de Farmácia Comunitária e Investigação [M.Sc.]. Portugal: Universidade da Beira Interior (Portugal); 2017.

2316. Almeida VB. Otimização de uma Metodologia Analítica para a Determinação de Pregabalina em

Plasma com Recurso à Microextração em Seringa: Experiência Profissionalizante na Vertente de Farmácia Comunitária e Investigação [M.Sc.]. Portugal: Universidade da Beira Interior (Portugal); 2014.

2317. Almeleebia TM, Alhifany AA, Almutairi F, Alshibani M, Alhossan AM. Regulating antimicrobial sales in Saudi Arabia: Achievements and challenges. International Journal of Clinical Practice. 2021;75(4):e13833.

2318. Almeman AA, Alrebish SA. Chapter 10 - Teaching Strategies Used in Pharmacy. In: Fathelrahman AI, Mohamed Ibrahim MI, Alrasheedy AA, Wertheimer AI, editors. Pharmacy Education in the Twenty First Century and Beyond: Academic Press; 2018. p. 125-45.

2319. Almeter PJ, Johnson GL, Schwieterman PA, Grantz DA, Pasala PP. Ambulatory care pharmacy: Realizing the potential for patient access and operational excellence. American Journal of Health-System Pharmacy. 2017;74(13):1013-9. doi: 10.2146/ajhp160802.

2320. Almodovar AS, "Kevin" Chang H-C, Matsunami M, Coleman A, Nahata MC. Confidence in skills applied to patient care among PharmD students in telehealth medication management programs versus other settings. Currents in Pharmacy Teaching and Learning. 2018;10(5):558-65. doi: https://doi.org/10.1016/j.cptl.2018.02.008.

2321. Almodovar AS, "Kevin" Chang HC, Matsunami M, Coleman A, Nahata MC. Confidence in skills applied to patient care among PharmD students in telehealth medication management programs versus other settings. Currents in Pharmacy Teaching and Learning. 2018;10(5):558-65. doi: 10.1016/j.cptl.2018.02.008.

2322. Almodovar AS, Ross E, Nahata MC, Bingham JM. Social needs of dual-enrolled Medicare-Medicaid patients with medication nonadherence in a telehealth medication therapy management program. Journal of managed care & specialty pharmacy. 2023;29(2):210-5. doi: https://dx.doi.org/10.18553/jmcp.2023.29.2.210. PubMed PMID: 640139584.

2323. Almohammadi AM, Al-Dhahri HM, Al-Harbi SH. Impact of Electronic Prescription on Prescribing Errors. Journal of Pharmaceutical Research International. 2021;33(32A):212-20. doi: 10.9734/JPRI/2021/v33i32A31733. PubMed PMID: WOS:000670069400024.

2324. Almohammed OA, Alnogaidan RA, Ghannam FO, Alqahtani RS, Aloraini SK, Abanmy NO. Public awareness of online pharmacies, consumers' motivating factors, experience and satisfaction with online pharmacy services, and current barriers and motivators for non-consumers: The case of Saudi Arabia. Saudi Pharmaceutical Journal. 2023;31(8). doi: 10.1016/j.jsps.2023.06.009.

2325. Almomani BA, Mayyas RK, Ekteish FA, Ayoub AM, Ababneh MA, Alzoubi SA. The effectiveness of clinical pharmacist's intervention in improving asthma care in children and adolescents: randomized controlled study in Jordan. Patient education and counseling (no pagination), 2016. 2016;Date of Publication: May 04. doi: 10.1016/j.pec.2016.11.002. PubMed PMID: CN-01287626.

2326. Almomani BA, Mayyas RK, Ekteish FA, Ayoub AM, Ababneh MA, Alzoubi SA. The effectiveness of clinical pharmacist's intervention in improving asthma care in children and adolescents: randomized controlled study in Jordan. Patient education and counseling. 2017;100(4):728-35. doi: 10.1016/j.pec.2016.11.002. PubMed PMID: CN-01215800.

2327. Almomani BA, Mokhemer E, Al-Sawalha NA, Momany SM. A novel approach of using educational pharmaceutical pictogram for improving inhaler techniques in patients with asthma. Respiratory medicine. 2018;143:103-8. doi: 10.1016/j.rmed.2018.09.004. PubMed PMID: CN-01644424.

2328. Almomani EY, Jarrar W, Alhadid A, Hamadneh L, Qablan A, Almomani HY. Shedding light on

pharmacists' knowledge of kidney stones' etiology and treatment. Pharmacy Practice. 2022;20(3). doi: 10.18549/PharmPract.2022.3.2712.

2329. Almomani HY, Pascual CR, Al-Azzam SI, Ahmadi K. Randomised controlled trial of pharmacist-led patient counselling in controlling hypoglycaemic attacks in older adults with type 2 diabetes mellitus (ROSE-ADAM): a study protocol of the SUGAR intervention. Research in social & administrative pharmacy : RSAP. 2021;17(5):885-93. doi: 10.1016/j.sapharm.2020.07.012. PubMed PMID: CN-02160159.

2330. Almomani HY, Pascual CR, Grassby P, Ahmadi K. Effectiveness of the SUGAR intervention on hypoglycaemia in elderly patients with type 2 diabetes: a pragmatic randomised controlled trial. Research in social & administrative pharmacy : RSAP. 2022. doi: 10.1016/j.sapharm.2022.09.017. PubMed PMID: CN-02487667.

2331. Almomani HY, Pascual CR, Grassby P, Ahmadi K. Effectiveness of the SUGAR intervention on hypoglycaemia in elderly patients with type 2 diabetes: A pragmatic randomised controlled trial. Research in Social and Administrative Pharmacy. 2023;19(2):322-31. doi: https://doi.org/10.1016/j.sapharm.2022.09.017.

2332. Almomani HY, Pascual CR, Grassby P, Ahmadi K. Effectiveness of the SUGAR intervention on hypoglycaemia in elderly patients with type 2 diabetes: A pragmatic randomised controlled trial. Research in Social and Administrative Pharmacy. 2023;19(2):322-31. doi: https://doi.org/10.1016/j.sapharm.2022.09.017.

2333. Almond M, Gordon K, Kent JR, Jones BW, Nice SW, Dhillon S. The effect of the controlled entry of electronic prescribing and medicines administration on the quality of prescribing, safety and success of administration on an acute medical ward. British Journal of Healthcare Computing & Information Management. 2002;19(2):41-6. PubMed PMID: 106946454. Language: English. Entry Date: 20020809. Revision Date: 20150820. Publication Type: Journal Article.

2334. Almubarak SH, Alshatti FA. The Government as Plaintiff: An Analysis of Medical Litigation Against Healthcare Providers in the Eastern Province of the Kingdom of Saudi Arabia. Journal of Patient Safety. 2023;19(2):E31-E7. doi: 10.1097/PTS.0000000000001097.

2335. Almubark RA, Aljadani RH, Alqahtani AS, Alshammari TM, BinDhim NF. National Cross-Sectional Study of Community-Based Adverse Drug Reactions in Saudi Arabia. Drugs Real World Outcomes. 2020;7(2):161-70. PubMed PMID: 32215839.

2336. AlMuhaissen SA, Elayeh E, Sharaydih R, Abdullah TM, AlShalalfeh A, AlKhatib HS. Doctor of pharmacy as a career option: a cross-sectional study exploring PharmD students and practitioners expectations in Jordan. Human Resources for Health. 2023;21(1):49.

2337. AlMukdad S, Zaghloul N, Awaisu A, Mahfoud ZR, Kheir N, El Hajj MS. Exploring the Role of Community Pharmacists in Obesity and Weight Management in Qatar: A Mixed-Methods Study. Risk Management and Healthcare Policy. 2021;14:2771-87. doi: 10.2147/rmhp.S309142. PubMed PMID: WOS:000668875200001.

2338. Almukdad S, Zaghloul N, Awaisu A, Mahfoud ZR, Kheir N, Hajj MSE. Exploring the role of community pharmacists in obesity and weight management in Qatar: A mixed-methods study. Risk Management and Healthcare Policy. 2021;14:2771-87. doi: 10.2147/RMHP.S309142.

2339. Almuqbil M, Alrojaie L, Alturki H, Alhammad A, Alsharawy Y, Alkoraishi A, et al. The role of drug information centers to improve medication safety in Saudi Arabia - a study from healthcare professionals' perspective. Saudi Pharmaceutical Journal. 2022;30(4):377-81. doi: https://doi.org/10.1016/j.jsps.2022.01.024.

2340. Almuqbil M, Alturki H, Al Juffali L, Al-Otaibi N, Awaad N, Alkhudair N, et al. Comparison of medical documentation between pharmacist-led anticoagulation clinics and physician-led anticoagulation clinics: A retrospective study. Saudi Pharmaceutical Journal. 2023;31(11):101795.

2341. Almuqbil M, Alturki H, Al Juffali L, Al-otaibi N, Awaad N, Alkhudair N, et al. Comparison of medical documentation between pharmacist-led anticoagulation clinics and physician-led anticoagulation clinics: A retrospective study. Saudi Pharmaceutical Journal. 2023;31(11):101795. doi: https://doi.org/10.1016/j.jsps.2023.101795.

2342. Almuqdadi A, Yousef AM, Masoud Z, Majdalawi K, Al-Hadeed H, Kalabani R. An Innovative Teaching Strategy for the Outpatient Clerkship Program at Jordan University Hospital A Comprehensive Approach to Faculty Development Program. Indian Journal of Pharmaceutical Education and Research. 2016;50(2):215-24. doi: 10.5530/ijper.50.2.1. PubMed PMID: WOS:000384814500001.

2343. Almutairi AM, Salonitis K, Al-Ashaab A. Assessing the leanness of a supply chain using multi-grade fuzzy logic: a health-care case study. International Journal of Lean Six Sigma. 2019;10(1):81-105.

2344. Almutairi H, Stafford A, Etherton-Beer C, Fitzgerald P, Flicker L. Impact of a Multifaceted, Pharmacist-Led Intervention on Psychotropic Medication Use for Residents of Aged Care Facilities: A Parallel Cluster Randomized Controlled Trial. Journal of the American Medical Directors Association. 2023;24(9):1311.e1-.e8. doi: https://dx.doi.org/10.1016/j.jamda.2023.06.037. PubMed PMID: 2026611748.

2345. Almutairi R, Sanogo V, Diaby K. CO51 Safety Profile of Approved Alzheimer's Medication By USFDA. Value in Health. 2023;26(12):S23-S. doi: 10.1016/j.jval.2023.09.123. PubMed PMID: 174417107.

2346. Alnafisah AS. Implementation of Solution and Solid State Nuclear Magnetic Resonance (NMR) Spectroscopic Techniques for Quantitative and Qualitative Analyses of Molecular Species [Ph.D.]. United States -- Missouri: University of Missouri - Kansas City; 2019.

2347. Alnahar SA, Gkountouras G, Darwish RM, Bates I. Community pharmacists workforce readiness to deliver vaccination services: A cross-sectional study from Jordan. Pharmacology Research and Perspectives. 2022;10(2). doi: 10.1002/prp2.943.

2348. Alnahas F, Yeboah P, Fliedel L, Abdin AY, Alhareth K. Expired Medication: Societal, Regulatory and Ethical Aspects of a Wasted Opportunity. International Journal of Environmental Research and Public Health. 2020;17(3). doi: 10.3390/ijerph17030787. PubMed PMID: WOS:000517783300112.

2349. Alnaim L, Almazrou S, Alsunbul M, Alhaji A, Alenazi A, Alboami S. Pharmacist’s knowledge and attitudes towards complementary and alternative medicine in Saudi Arabia: A cross-sectional study. Advances in Integrative Medicine. 2018;5(3):96-102. doi: https://doi.org/10.1016/j.aimed.2018.01.003.

2350. Alnaim LS, Almalki HM, Almutairi AM, Salamah HJ. The prevalence of drug–drug interactions in cancer therapy and the clinical outcomes. Life Sciences. 2022;310:121071. doi: https://doi.org/10.1016/j.lfs.2022.121071.

2351. Alnajjar MS, AlAbdin SZ, Arafat M, Skaik S, AbuRuz S. Pharmacists' knowledge, attitude and practice in the UAE toward the public health crisis of COVID-19: A cross-sectional study. Pharmacy Practice-Granada. 2022;20(1). doi: 10.18549/PharmPract.2022.1.2628. PubMed PMID: WOS:000780924400001.

2352. Alnajjar MS, Mohammed AA. Experiential community pharmacy training: Assessment of students’ perceptions in the united arab emirates. Pharmacy Education. 2020;20:225-35. doi: 10.46542/pe.2020.201.225235.

2353. Alnajjar MS, Zamzoum LM, Saeed DA. Barriers to adverse drug reaction reporting in community practice in the UAE. Journal of pharmaceutical health services research. 2019;10(3):373-80. doi: 10.1111/jphs.12314. PubMed PMID: CN-02002505.

2354. Alnuaimi A, Alshehhi A, Salah K, Jayaraman R, Omar IA, Battah A. Blockchain-Based Processing of Health Insurance Claims for Prescription Drugs. Ieee Access. 2022;10:118093-107. doi: 10.1109/access.2022.3219837. PubMed PMID: WOS:000886165400001.

2355. Alobaid N, Aboheimed H, Alhumsi A, Aboheimed G, Aloudah N, Aboheimed N. Evidence based practice in supplying of over the counter medicines in community pharmacies: a mixed methods study. Pharmacoepidemiology and drug safety. 2020;29(SUPPL 3):182-. doi: 10.1002/pds.5114. PubMed PMID: CN-02242698.

2356. Alodan A, Alalshaikh G, Alqasabi H, Alomran S, Abdelhadi A, Alkhayyal B. Studying the Efficiency of Waiting Time in Outpatient Pharmacy. MethodsX. 2020;7:100913. doi: https://doi.org/10.1016/j.mex.2020.100913.

2357. Aloe S, Filliter C, Salmasi S, Igweokpala S, Yu OHY, Tagalakis V, et al. Sodium-glucose cotransporter 2 inhibitors and the risk of venous thromboembolism: A population-based cohort study. British Journal of Clinical Pharmacology. 2023;89(9):2902-14. doi: 10.1111/bcp.15787. PubMed PMID: 169944237.

2358. Alohan DI, Evans G, Sanchez T, Harrington KRV, Quamina A, Young HN, et al. Examining pharmacies' ability to increase pre-exposure prophylaxis access for black men who have sex with men in the United States. Journal of the American Pharmacists Association. 2022. doi: https://doi.org/10.1016/j.japh.2022.11.004.

2359. Alohan DI, Evans G, Sanchez T, Harrington KRV, Quamina A, Young HN, et al. Examining pharmacies' ability to increase pre-exposure prophylaxis access for black men who have sex with men in the United States. Journal of the American Pharmacists Association. 2023;63(2):547-54. doi: https://doi.org/10.1016/j.japh.2022.11.004.

2360. Alolayyan MN, Hijazi B, Jalaileh RA. The association of patient safety culture with patient satisfaction: The role of the pharmacist's performance. Journal of Patient Safety and Risk Management. 2023;28(2):68-77. doi: 10.1177/25160435231160445.

2361. Alomar MJ. Factors affecting the development of adverse drug reactions. Saudi pharmaceutical journal. 2014;22(2):83-94.

2362. Alomar MJ, Mellal AA. Patients' preferences toward the gender of pharmacist in community pharmacy in Al Ain, UAE. International Journal of Pharmacy and Pharmaceutical Sciences. 2015;7(9):281-5.

2363. Alomar MJ, Qandil S, Al-Hilwani HM, Malkat DM, Caroline C. Evaluation of the community pharmacist's behavior towards a prescription of antidiabetic and antiasthma drugs. Pharmacy practice. 2011;9(1):37.

2364. Alomar MJ, Qandil S, Al-Hilwani HMA, Malkat DM, Caroline C. Evaluation of the community pharmacist's behavior towards a prescription of antidiabetic and antiasthma drugs. Pharmacy Practice. 2011;9(1):37-43. doi: 10.4321/S1886-36552011000100006.

2365. Alomari A, Wilson V, Davidson PM, Lewis J. Families, nurses and organisations contributing factors to medication administration error in paediatrics: a literature review. International Practice Development Journal. 2015;5(1):1-14. doi: 10.19043/ipdj.51.007. PubMed PMID: 103282091. Language: English. Entry Date: 20180117. Revision Date: 20200123. Publication Type: Article.

2366. Alomi YA, Al-Shubbar NA, Lubad NA. PHP223 - Inpatient Medication Errors And Pharmacist Intervention At Ministry Of Health Public Hospital In Riyadh, Saudi Arabia. Value in Health. 2017;20(9):A690. doi: https://doi.org/10.1016/j.jval.2017.08.1758.

2367. Alomi YA, Alghamdi SJ, Alattyh RA. Saudi Managed Care Pharmacy (SMCP): New initiative system of MOH prescriptions dispensed through community pharmacies. Journal of Pharmacy Practice and Community Medicine. 2017;3(3).

2368. Alomi YA, Alghamdi SJ, Alattyh RA. National medication errors reporting system at ministry of health in Saudi Arabia. Pharmacology, Toxicology and Biomedical Reports. 2019;5(1).

2369. Alomi YA, Almudaiheem HY. Clinical outcomes of pharmacokinetics services managed by pharmacist at ministry of health hospital in Saudi Arabia. Value in health Conference: ISPOR 22nd annual international meeting United states. 2017;20(5):A38. PubMed PMID: CN-01407776.

2370. Alomi YA, Almudaiheem HY. Clinical outcomes of vancomycin therapeutic monitoring services at ministry of health hospital in Saudi Arabia. Value in health Conference: ISPOR 22nd annual international meeting United states. 2017;20(5):A42. PubMed PMID: CN-01407839.

2371. Alonso MJO, Rodicio LAB. Pharmaceutical indication for influenza and common cold in the context of COVID-19. Pharmaceutical Care Espana. 2021;23(1):40-72. PubMed PMID: WOS:000618816200005.

2372. Alonso V. Drug consumption and health equity in the Metropolitan Area of Buenos Aires, Argentina. Revista Panamericana de Salud Publica/Pan American Journal of Public Health. 2003;13(6):400-6. doi: 10.1590/s1020-49892003000500009.

2373. Alonso-Moreno C, Alonso-Moreno R, Arroyo-Jimenez MM, Artigao MM, Bravo I, de la Casa-Esperon E, et al., editors. FRATER: A MENTORING INNOVATIVE PROJECT FOR FRESHMEN. 7th International Technology, Education and Development Conference (INTED); 2013 Mar 04-06; Valencia, SPAIN2013.

2374. Alonso-Perales MD, Lasheras B, Beitia G, Beltran I, Marcos B, Nunez-Cordoba JM. Barriers to promote cardiovascular health in community pharmacies: a systematic review. Health Promotion International. 2017;32(3):535-48. doi: 10.1093/heapro/dav098. PubMed PMID: WOS:000404035400013.

2375. Aloo N, Nyachae P, Mbugua N, Sirera M, Owino K, Kagwe P, et al. Improving access to family planning services through community pharmacies: Experience from The Challenge Initiative in three counties in Kenya. Frontiers in Global Women's Health. 2023;4. doi: 10.3389/fgwh.2023.1060832.

2376. Alookaran S, O'Sullivan F. Polypharmacy in older adults: The role of the advanced nurse practitioner. Age and Ageing. 2023;52(Supplement 3):iii26. doi: https://dx.doi.org/10.1093/ageing/afad156.108. PubMed PMID: 642656664.

2377. Alor SK, Kretchy IMA, Glozah FN, Adongo PB. Factors associated with glycaemic control among patients with type 2 diabetes mellitus in Ho, Ghana: A cross-sectional study. Metabolism Open. 2023;20:100265. doi: https://doi.org/10.1016/j.metop.2023.100265.

2378. Alorfi NM. PRESCRIBING PATTERNS AND PHARMACOLOGICAL KNOWLEDGE OF ANALGESICS AMONG COMMUNITY PHARMACISTS IN JEDDAH, SAUDI ARABIA. Bulletin of Pharmaceutical Sciences Assiut. 2023;46(1):647-57. doi: https://dx.doi.org/10.21608/bfsa.2023.301293. PubMed PMID: 2025285895.

2379. Alorfi NM, Alqurashi RS, Algarni AS. Assessment of community pharmacists' knowledge about drug-drug interactions in Jeddah, Saudi Arabia. Frontiers in Pharmacology. 2023;14. doi: 10.3389/

fphar.2023.1209318.

2380. Alorfi NM, Ashour AM, Algarni AS, Alsolami FA, Alansari AM, Tobaiqy M. Assessment of the Community Pharmacists Knowledge and Attitudes Toward Pain and Pain Management in Saudi Arabia. International Journal of General Medicine. 2022;15:8527-37. doi: 10.2147/IJGM.S387066InternationalJournalofGeneral. PubMed PMID: WOS:000893275900001.

2381. Alosaimi FD, Alruwais FS, Alanazi FA, Alabidi GA, Aljomah NA, Alsalameh NS. Patients reasons for obtaining psychotropic medications without a prescription at retail pharmacies in Central Saudi Arabia. Neurosciences. 2016;21(4):338-44. doi: 10.17712/nsj.2016.4.20160245. PubMed PMID: WOS:000393113900007.

2382. Alosaimy S, Vaidya A, Day K, Stern G. Effect of a Pharmacist-Driven Medication Management Intervention Among Older Adults in an Inpatient Setting. Drugs & Aging. 2019;36(4):371-8. doi: 10.1007/s40266-018-00634-9. PubMed PMID: 135555705. Language: English. Entry Date: 20190329. Revision Date: 20190408. Publication Type: Article.

2383. Alotaibi FM, Bukhamsin ZM, Alsharafaa AN, Asiri IM, Kurdi SM, Alshayban DM, et al. Knowledge, Attitude, and Perception of Health Care Providers Providing Medication Therapy Management (MTM) Services to Older Adults in Saudi Arabia. Healthcare (Switzerland). 2023;11(22). doi: 10.3390/healthcare11222936.

2384. Alotaibi HS, Abdelkarim MA. Consumers' perceptions on the contribution of community pharmacists in the dispensing process at Dawadmi. Saudi Pharmaceutical Journal. 2015;23(3):230-4. doi: https://doi.org/10.1016/j.jsps.2014.11.002.

2385. Alotaibi HS, Shivanandappa TB, Nagarethinam S. Contribution of community pharmacists in educating the asthma patients. Saudi Pharmaceutical Journal. 2016;24(6):685-8. doi: https://doi.org/10.1016/j.jsps.2015.06.002.

2386. Alotaibi M. Limitations and Potential Facilitators and Benefits of Managing Chronic Conditions in Community Pharmacy Settings [Ph.D.]. Wales: Cardiff University (United Kingdom); 2021.

2387. Alotaibi MM, Hughes L, Bowen JL, Ford WR. Stakeholders' Views about the Management of Stable Chronic Conditions in Community Pharmacies. Pharmacy. 2022;10(3). doi: 10.3390/pharmacy10030059. PubMed PMID: WOS:000817460400001.

2388. Alotaibi NH, Alzarea AI, Alotaibi AM, Khan YH, Mallhi TH, Alharbi KS, et al. Exploring satisfaction level among outpatients regarding pharmacy facilities and services in the Kingdom of Saudi Arabia; a large regional analysis. Plos One. 2021;16(4). doi: 10.1371/journal.pone.0247912. PubMed PMID: WOS:000636467000011.

2389. Alotaibi NM. Pharmaceutical Consultation in Hospitals at Northern Border Region, Saudi Arabia. Journal of Pharmaceutical Research International. 2021;33(24A):9-14. doi: 10.9734/JPRI/2021/v33i24A31426. PubMed PMID: WOS:000669864500002.

2390. Alotaibi YK, Federico F. The impact of health information technology on patient safety. Saudi medical journal. 2017;38(12):1173.

2391. Alothaim R, Almatroudi A, Ahmed MU, Khan MA, Joseph RJ, Alharbi A, et al. Public health risk assessment of the door handles of the community pharmacies in Qassim Region, saudi Arabia. Journal of Pure and Applied Microbiology. 2020;14(4):2649-54. doi: 10.22207/JPAM.14.4.41.

2392. Aloudah NM, Almanea H, Alotaibi K, Al Rubeaan KA. Quality of diabetes care among patients with schizophrenia. A mixed-methods study. Exploratory Research in Clinical and Social Pharmacy. 2021;4:100070. doi: https://doi.org/10.1016/j.rcsop.2021.100070.

2393. Aloy B, Launay-Vacher V, Bleibtreu A, Bortolotti P, Faure E, Filali A, et al. Antibiotics and chronic kidney disease: Dose adjustment update for infectious disease clinical practice. Médecine et Maladies Infectieuses. 2020;50(4):323-31. doi: https://doi.org/10.1016/j.medmal.2019.06.010.

2394. Alpert JM. Assessing the Communication Immediacy of an Online Health Portal: Analysis and Recommendations to Create a Communicatively Competent Health Information System [Ph.D.]. United States -- Virginia: George Mason University; 2015.

2395. Alpuente Climent M, D'Ocón P, Forner MJ, Moreno L. Approaching the problem of hypertension from a pharmacy: Detection, health education and monitoring. Which activity shows more efficiency? Pharmaceutical Care Espana. 2013;15(2):44-50.

2396. Alqadi AA. Studies of antihypertensive drug persistence and adherence in the glasgow blood pressure clinic [Ph.D.]. Scotland: University of Glasgow (United Kingdom); 2017.

2397. Alqahtani FY, Alattas SH, Almangour TA, Aleanizy FS. Status of infectious disease content in the professional pharmacy curriculum in Saudi Arabia: Results of a national survey. Saudi Pharmaceutical Journal. 2021;29(12):1492-7. doi: https://doi.org/10.1016/j.jsps.2021.11.009.

2398. AlQahtani HA, Ghiasi FS, Zahiri AN, Rahmani NI, Abdullah N, Al Kawas S. Self-medication for oral health problems among adults attending the University Dental Hospital, Sharjah. Journal of Taibah University Medical Sciences. 2019;14(4):370-5. doi: https://doi.org/10.1016/j.jtumed.2019.06.006.

2399. Alqahtani S, Alhawassi T, Balkhi B, Alrasheed M, Mahmoud M, Aljadhey H. Public willingness to receive vaccination in community pharmacies in Saudi Arabia. Pharmacoepidemiology and drug safety. 2017;26:619-20. doi: 10.1002/pds.4275. PubMed PMID: CN-01418423.

2400. Alqahtani SS, Ahmad S, Alam N, Kashan Syed N, Syed MH, Khardali A, et al. Healthcare professionals' awareness, attitudes and practices towards pharmacovigilance and spontaneous adverse drug reaction reporting in Jazan Province, Saudi Arabia: A survey study. Saudi Pharmaceutical Journal. 2023;31(6):979-88. doi: https://doi.org/10.1016/j.jsps.2023.04.021.

2401. Alqahtani SS, Banji D, Banji OJF. A survey assessing sleep efficiency among Saudis during COVID-19 home confinement using the Pittsburgh sleep quality index: A call for health education. Saudi Pharmaceutical Journal. 2021;29(7):692-8. doi: 10.1016/j.jsps.2021.04.031.

2402. Alqenae FA, Steinke D, Keers RN. Prevalence and nature of medication errors and medication-related harm following discharge from hospital to community settings: a systematic review. Drug safety. 2020;43:517-37.

2403. Alqifari SF. Assessment of students' knowledge and perception of pain and anti-inflammatory drug therapy in dental schools in Saudi Arabia. Future Dental Journal. 2018;4(2):314-7. doi: https://doi.org/10.1016/j.fdj.2018.04.007.

2404. Alqubaisi M, Tonna A, Strath A, Stewart D. Quantifying behavioural determinants relating to health professional reporting of medication errors: a cross-sectional survey using the Theoretical Domains Framework. European Journal of Clinical Pharmacology. 2016;72(11):1401-11. doi: 10.1007/s00228-016-2124-z. PubMed PMID: 118671718. Language: English. Entry Date: 20170704. Revision Date: 20190308. Publication Type: Article.

2405. Alqudah S, Jarab AS, Alefishat EA, Mayyas F, Khdour M, Pinto S. Factors Associated with Poor Hemoglobin A1c Control in Patients with Type 2 Diabetes. Current Diabetes Reviews. 2019;15(2):164-70. doi: 10.2174/1573399814666180510144858. PubMed PMID: WOS:000457685200012.

2406. Alrabiah Z, Al-Arifi MN, Alghadeer SM, Wajid S, AlQahtani A, Almotari N, et al. Knowledge of community pharmacists about the risks of medication use during pregnancy in central region of Saudi Arabia. Saudi Pharmaceutical Journal. 2017;25(7):1093-6.

2407. Alrabiah Z, Alhossan A, Alghadeer SM, Wajid S, Babelghaith SD, Al-Arifi MN. Evaluation of community pharmacists' knowledge about drug–drug interaction in Central Saudi Arabia. Saudi Pharmaceutical Journal. 2019;27(4):463-6. doi: https://doi.org/10.1016/j.jsps.2019.01.008.

2408. Alrabiah Z, Alhossan A, Alghadeer SM, Wajid S, Babelghaith SD, Al-Arifi MN. Evaluation of community pharmacists' knowledge about drug-drug interaction in Central Saudi Arabia. Saudi Pharmaceutical Journal. 2019;27(4):463-6. doi: 10.1016/j.jsps.2019.01.008. PubMed PMID: WOS:000466099300002.

2409. Alrabiah Z, Alhossan A, Yun S, MacDonald K, Abraham I. Adherence to Tyrosine Kinase Inhibitor Therapy in Patients with Chronic Myeloid Leukemia: Meta-Analyses of Prevalence Rates By Measurement Method. Blood. 2016;128(22):3610. doi: https://doi.org/10.1182/blood.V128.22.3610.3610.

2410. Alraie NA, Saad AA, Sabry NA, Farid SF. Adverse drug reactions reporting: a questionnaire-based study on Egyptian pharmacists' attitudes following an awareness workshop. Journal of Evaluation in Clinical Practice. 2016;22(3):349-55. PubMed PMID: 26638825.

2411. Alrasheed AA. The impact of waiting time in primary care clinics on self-medication with antibiotics: A hospital based study in Saudi Arabia. Biomedical Research (India). 2017;28(7):3119-24.

2412. Alrasheedy AA, Alsalloum MA, Almuqbil FA, Almuzaini MA, Aba Alkhayl BS, Albishri AS, et al. The impact of law enforcement on dispensing antibiotics without prescription: a multi-methods study from Saudi Arabia. Expert Review of Anti-Infective Therapy. 2020;18(1):87-97. doi: 10.1080/14787210.2020.1705156.

2413. Alridha AMA, Al-Gburi KMH, Abbood SK, Yasir A, Hussam D. Practices, knowledge, and attitude toward dispensing antibiotics without a prescription in Iraqi pharmacies. Journal of Public Health and Development. 2023;21(1):15-31. doi: 10.55131/jphd/2023/210102.

2414. Alridha AMA, Al-Gburi KMH, Abbood SK, Yasir A, Hussam D. Practices, knowledge, and attitude toward dispensing antibiotics without a prescription in Iraqi pharmacies. Journal of Public Health and Development. 2023;21(1):15-31. doi: 10.55131/jphd/2023/210102.

2415. Alrukban M, Alruthia Y, Almasaoud M, Al-Owairdhi M, Alsouan A, Alrabiah A, et al. Community pharmacists' views of the enforced antibiotics dispensing law and its impact on oral antibiotics sales in Saudi Arabia. Risk Management and Healthcare Policy. 2020;13:2899-907. doi: 10.2147/RMHP.S278404.

2416. Alruthea S, Bowman P, Tariq A, Hinchcliff R. Interventions to Enhance Medication Safety in Residential Aged-care Settings: An Umbrella Review. British Journal of Clinical Pharmacology. 2022;88(4):1630-43. PubMed PMID: 34652833.

2417. AlRuthia Y, Alkofide H, Alosaimi FD, Sales I, Alnasser A, Aldahash A, et al. Drug-drug interactions and pharmacists' interventions among psychiatric patients in outpatient clinics of a teaching hospital in Saudi Arabia. Saudi Pharmaceutical Journal. 2019;27(6):798-802. doi: https://doi.org/10.1016/j.jsps.2019.05.001.

2418. AlRuthia Y, Bashatah A, Batis AA, Alradhi SA, Almohammed O, Sales I, et al. Exploring the opportunities and challenges to implementing interprofessional education in Saudi Arabia: a qualitative study among faculty. Journal of Interprofessional Care. 2023;37(1):47-57. doi: 10.1080/13561820.2021.2004097. PubMed PMID: WOS:000738394700001.

2419. Alruthia YS, Alwhaibi M, Alotaibi MF, Asiri SA, Alghamdi BM, Almuaythir GS, et al. Drug shortages in Saudi Arabia: Root causes and recommendations. Saudi Pharmaceutical Journal. 2018;26(7):947-51. doi: https://doi.org/10.1016/j.jsps.2018.05.002.

2420. AlRuwaili N, Zaidan F, AlFattani A, Alenazi S. Urgent need to standardize labelling of acetaminophen-paediatric liquid drug products in Saudi Arabia. International Journal of Pediatrics and Adolescent Medicine. 2022;9(1):62-5. doi: https://doi.org/10.1016/j.ijpam.2021.09.001.

2421. Alrwisan A. Azithromycin: An Evaluation of use and Safety [Ph.D.]. United States -- Florida: University of Florida; 2017.

2422. Alrwisan A, Ross J, Williams D. Medication incidents reported to an online incident reporting system. European journal of clinical pharmacology. 2011;67:527-32.

2423. Alrwisan AA, Alshammari TM, Tahir KW, Aleissa FM, Aljadhey HS. Community pharmacists' knowledge of isotretinoin safety. Saudi Medical Journal. 2014;35(1):81-4.

2424. AlSaad D, Awaisu A, AlHail M, Ahmed A, Moursi A, Abdulrouf PV, et al. Chapter 2 - Clinical pharmacy service innovation in an obstetrics and gynecology setting: A case study from Qatar. In: Babar Z-U-D, editor. Pharmacy Practice Research Case Studies: Academic Press; 2021. p. 25-39.

2425. Alsabbagh MW, Church D, Wenger L, Papastergiou J, Raman-Wilms L, Schneider E, et al. Pharmacy patron perspectives of community pharmacist administered influenza vaccinations. Research in Social & Administrative Pharmacy. 2019;15(2):202-6. doi: 10.1016/j.sapharm.2018.04.015. PubMed PMID: WOS:000456442700009.

2426. Alsabbagh MW, Lemstra M, Eurich D, Wilson TW, Robertson P, Blackburn DF. Pharmacist intervention in cardiac rehabilitation: a randomized controlled trial. Journal of cardiopulmonary rehabilitation and prevention. 2012;32(6):394-9. doi: 10.1097/HCR.0b013e318272bbf2. PubMed PMID: CN-00966885.

2427. Alsahali S, Almutairi G, Aedh R, Alanezi S, Almutairi H, Anaam M, et al. Perceptions of Community Pharmacists toward the National E-Prescribing Service (Wasfaty) and Exploring the Benefits and Challenges of the Service: A Descriptive Study from Qassim Region, Saudi Arabia. Pharmacy. 2023;11(5):152. doi: https://dx.doi.org/10.3390/pharmacy11050152. PubMed PMID: 2026378685.

2428. Alsaid A, Mohamed MS, Mallhi TH, Panda DS, Khan YH, Butt MH. Knowledge, Attitude and Practice of Community Pharmacists towards Food-Drug Interactions; A Prospective Cross-Sectional Analysis from Sudan. Latin American Journal of Pharmacy. 2022;41(3):506-14. PubMed PMID: WOS:000764900200002.

2429. Alsaid AMAH, Mohamed MS, Mallhi TH, Panda DS, Khan YH, Butt MH. Knowledge, Attitude and Practice of Community Pharmacists towards Food-Drug Interactions; A Prospective Cross-Sectional Analysis from Sudan. Latin American Journal of Pharmacy. 2022;41(3):506-14.

2430. Alsaidan J, Portlock J, Aljadhey HS, Shebl NA, Franklin BD. Systematic review of the safety of medication use in inpatient, outpatient and primary care settings in the Gulf Cooperation Council countries. Saudi Pharmaceutical Journal. 2018;26(7):977-1011.

2431. Alsairafi Z, Mason J, Davies N, Dennis M, Pilgrim G, Goel N, et al. Community pharmacy advanced adherence services for children and young people with long-term conditions: A cross-sectional survey study. Pharmacy Practice. 2020;18(1). doi: 10.18549/PharmPract.2020.1.1720.

2432. Alsairafi Z, Waheedi M, Alsaleh F. The perspectives of patients and physicians on the role of pharmacists in improving medication adherence in type 2 diabetes: a qualitative study. Patient Preference and Adherence. 2019;13:1527-43. doi: 10.2147/ppa.S218068. PubMed PMID: WOS:000484764900002.

2433. Alsaleh F, Smith FJ, Rigby E, Gray NJ. Applying the 'You're Welcome' youth-friendly service criteria to community pharmacy in the UK. Journal of Pharmaceutical Health Services Research. 2016;7(1):71-9. doi: 10.1111/jphs.12125.

2434. Alsaleh FM, Abahussain EA, Altabaa HH, Al-Bazzaz MF, Almandil NB. Assessment of patient safety culture: a nationwide survey of community pharmacists in Kuwait. BMC Health Services Research. 2018;18(1):N.PAG-N.PAG. doi: 10.1186/s12913-018-3662-0. PubMed PMID: 133148393. Language: English. Entry Date: In Process. Revision Date: 20190126. Publication Type: journal article.

2435. Alsaleh NA. Pharmacist-led flu vaccination services in community pharmacy: Experiences and Benefits. Journal of Advanced Pharmacy Education and Research. 2020;10(1):181-5.

2436. Alsalem A. To Stay or Not to Stay: Perceived Organisational and Individual Determinants of Voluntary Turnover of Medical Representative Role Among Pharmacists Working for Pharmaceutical Companies in Iraq2023.

2437. Alsaloumi L, Abdi A, Tosun O, Basgut B. Pharmacogenomics-based practice in North Cyprus: its adoption by pharmacists and their attitudes and knowledge. International Journal of Clinical Pharmacy. 2019;41(5):1299-306. doi: 10.1007/s11096-019-00868-6. PubMed PMID: WOS:000491548500024.

2438. AlSamanhodi H, Almeshary M, Amoh K, Aldekhael S, Alkatheri A, Alharbi S, et al. Evaluation of the causes and cost impact of returned intravenous medications at a tertiary care hospital in Riyadh, Saudi Arabia. Tropical Journal of Pharmaceutical Research. 2017;16(1):231-7. doi: 10.4314/tjpr.v16i1.31. PubMed PMID: WOS:000397812000031.

2439. Alsammarraie HJ, Abdulwahed AM, Abdulrahman MA, Alkanaani MIM, Ahmed UA, editors. Effects of Slimming Herbal Product (Lipo 6)® on Body Mass Index and Selected Biochemical Parameters in Obese Healthy People. AIP Conference Proceedings; 2022.

2440. Alsayari A, Almghaslah D, Khaled A, Annadurai S, Alkhairy MA, Alqahtani HA, et al. Community pharmacists' knowledge, attitudes, and practice of herbal medicines in Asir region, Kingdom of Saudi Arabia. Evidence-Based Complementary and Alternative Medicine. 2018;2018.

2441. Alsayed AR, Al-Dulaimi A, Alnatour D, Awajan D, Alshammari B. Validation of an assessment, medical problem-oriented plan, and care plan tools for demonstrating the clinical pharmacist's activities. Saudi Pharmaceutical Journal. 2022;30(10):1464-72. doi: https://doi.org/10.1016/j.jsps.2022.07.007.

2442. Alsayed AR, Darwish El Hajji F, Al-Najjar MAA, Abazid H, Al-Dulaimi A. Patterns of antibiotic use, knowledge, and perceptions among different population categories: A comprehensive study based in Arabic countries. Saudi Pharmaceutical Journal. 2022;30(3):317-28. doi: https://doi.org/10.1016/j.jsps.2022.01.013.

2443. Alsayed AR, El Hajji FD, Al-Najjar MAA, Abazid H, Al-Dulaimi A. Patterns of antibiotic use, knowledge, and perceptions among different population categories: A comprehensive study based in Arabic countries. Saudi Pharmaceutical Journal. 2022;30(3):317-28. doi: 10.1016/j.jsps.2022.01.013. PubMed PMID: WOS:000820483200016.

2444. Alsayed AR, Halloush S, Hasoun L, Alnatour D, Al-Dulaimi A, Alnajjar MS, et al. Perspectives of the community in the developing countries toward telemedicine and pharmaceutical care during the COVID-19 pandemic. Pharmacy Practice. 2022;20(1). doi: 10.18549/PharmPract.2022.1.2618.

2445. Alsayed AR, Hasoun L, Al-Dulaimi A, AbuAwad A, Basheti I, Khader HA, et al. Evaluation of the effectiveness of educational medical informatics tutorial on improving pharmacy students' knowledge and skills about the clinical problem-solving process. Pharmacy Practice-Granada. 2022;20(2). doi: 10.18549/PharmPract.2022.2.2652. PubMed PMID: WOS:000822925900001.

2446. Alshabi AM, Shaikh MAK, Shaikh IA, Alkahtani SA, Aljadaan A. Knowledge, attitude and practice of hospital pharmacists towards pharmacovigilance and adverse drug reaction reporting in Najran, Saudi Arabia. Saudi Pharmaceutical Journal. 2022;30(7):1018-26. doi: https://doi.org/10.1016/j.jsps.2022.04.014.

2447. Alshagga MA, Nasir NZM, Behzadnia A, Jasamai M, Al-Absi AM, Al-Dubai SAR. Perceived stress and sources of stress among pharmacy students in Malaysian public and private universities: a comparative study. Pharmacy Education. 2015;15(1):64-8. PubMed PMID: WOS:000378481400012.

2448. Alshahrani AF, Dighriri IM. Patients' Satisfaction With Medication Delivery Pharmacy Services in a Tertiary Hospital in Asir, Saudi Arabia: A Cross-Sectional Study. Cureus Journal of Medical Science. 2023;15(11). doi: 10.7759/cureus.48903. PubMed PMID: WOS:001109814300013.

2449. Alshahrani AM, Fathelrahman AI, Ashour AM, Alqahtani SS. Pharmacists Analysis of the Negative Impacts of Smoking and Quality of Glycaemic Control Following Smoking Cessation in Patients with Type II Diabetes. Latin american journal of pharmacy. 2022;41(12):2446-52. PubMed PMID: CN-02508810.

2450. Alshahrani SM. Assessment of the quality of outpatient prescriptions from various clinical setting in a tertiary hospital, Saudi Arabia. International Journal of Applied Pharmaceutics. 2019;11(Special Issue 5):5-8. doi: 10.22159/ijap.2019.v11s5.T0021.

2451. Alshahrani SM. Perspectives Role of Pharmacists in the Provision of COVID-19 Vaccine at Community Pharmacies in Asser Region, Saudi Arabia. Latin American Journal of Pharmacy. 2022;41(1):261-5.

2452. Alshahrani SM, Orayj K, Alqahtani AM, Alfatease A, Alshahrani A, Ibrahim ARN. Attitude and Willingness to Get COVID-19 Vaccines by a Community Pharmacist in Saudi Arabia: A Cross-Sectional Study. Patient Preference and Adherence. 2022;16:2821-34. doi: 10.2147/ppa.S379309. PubMed PMID: WOS:000888023000001.

2453. Alshaiban A, Alavudeen SS, Alshahrani I, Kardam AM, Alhasan IM, Alasiri SA, et al. Impact of Clinical Pharmacist Running Anticoagulation Clinic in Saudi Arabia. Journal of Clinical Medicine. 2023;12(12):3887.

2454. Alshakka M, Badulla W, Ibrahim MIM. Dispensing of Banned Phenolphthalein and Ketoconazole: Simulated Patients Method. Journal of Pharmaceutical Research International. 2021;33(9):46-50. doi: 10.9734/JPRI/2021/v33i931226. PubMed PMID: WOS:000627856700007.

2455. Alshakka M, Badulla W, Ibrahim MIM. Knowledge, Attitudes and Practices Survey of Medication Safety among Community Pharmacists in Aden-Yemen. Journal of Pharmaceutical Research International. 2021;33(23A):13-27. doi: 10.9734/JPRI/2021/v33i23A31405. PubMed PMID: WOS:000669859600002.

2456. Alshakka M, Badullah W, Al-Dhuraibi A, Alshagga S, Ibrahim MIM. Teaching pharmacovigilance to undergraduate students: Our experience in poor-resource setting. Journal of Pharmacy and Bioallied Sciences. 2022;14(1):31-7. doi: 10.4103/jpbs.JPBS_532_20. PubMed PMID: WOS:000805249500005.

2457. Alshakka M, Bahattab A, Shankar PR, Ansari M, Ali HS, Ibrahim MIM. Perception of Community Pharmacy Personnel towards Patient Counseling and Continuing Pharmacy Education Programs in Aden, Yemen. Journal of Clinical and Diagnostic Research. 2018;12(7):LC18-LC22. doi: 10.7860/jcdr/2018/34106.11820. PubMed PMID: WOS:000441802000048.

2458. Alshakrah MA, Steinke DT, Lewis PJ. Patient prioritization for pharmaceutical care in hospital: A systematic review of assessment tools. Research in Social and Administrative Pharmacy. 2019;15(6):767-79. doi: https://doi.org/10.1016/j.sapharm.2018.09.009.

2459. Alshakrah MA, Steinke DT, Tully MP, Abuzour AS, Williams SD, Lewis PJ. Development of the adult complexity tool for pharmaceutical care (ACTPC) in hospital: A modified Delphi study. Research in Social and Administrative Pharmacy. 2021;17(11):1907-22. doi: https://doi.org/10.1016/j.sapharm.2021.02.009.

2460. Alshammari MK, Alotaibi NM, Al Suroor SN, Al Saed RS, Al-Hamoud AA, Alluwaif MA, et al., editors. Global advancement in pharmacy services for mental health: A review for evidence-based practices. Healthcare; 2023: MDPI.

2461. Alshammari MK, Alotaibi NM, Al Suroor SN, Al Saed RS, Al-hamoud AA, Alluwaif MA, et al. Global Advancement in Pharmacy Services for Mental Health: A Review for Evidence-Based Practices. Healthcare (Switzerland). 2023;11(8). doi: 10.3390/healthcare11081082.

2462. Alshammari T, Alseraye S, Alqasim R, Rogowska A, Alrasheed N, Alshammari M. Examining anxiety and stress regarding virtual learning in colleges of health sciences: A cross-sectional study in the era of the COVID-19 pandemic in Saudi Arabia. Saudi Pharmaceutical Journal. 2022;30(3):256-64. doi: https://doi.org/10.1016/j.jsps.2022.01.010.

2463. Alshammari TM, Alhindi SA, Alrashdi AM, Benmerzouga I, Aljofan M. Pharmacy Malpractice: The rate and prevalence of dispensing high-risk prescription-only medications at community pharmacies in Saudi Arabia. Saudi Pharmaceutical Journal. 2017;25(5):709-14. doi: https://doi.org/10.1016/j.jsps.2016.10.001.

2464. AlShammari TM, Almoslem MJ. Knowledge, attitudes & practices of healthcare professionals in hospitals towards the reporting of adverse drug reactions in Saudi Arabia: A multi-centre cross sectional study. Saudi Pharmaceutical Journal. 2018;26(7):925-31. doi: https://doi.org/10.1016/j.jsps.2018.04.012.

2465. Alshammari TM, Alsubait SI, Alenzi KA, Almalki ZS. Estimating the potential economic impact of the Wasfaty program on costs of antidiabetic treatment: An initiative for the digital transformation of health. Saudi Pharmaceutical Journal. 2023;31(6):1029-35. doi: https://doi.org/10.1016/j.jsps.2023.04.026.

2466. Alshamrani M, Almalki A, Qureshi M, Yusuf O, Ismail S. Polypharmacy and Medication-Related Problems in Hemodialysis Patients: A Call for Deprescribing. Pharmacy. 2018;6(3). doi: 10.3390/pharmacy6030076. PubMed PMID: WOS:000448399800022.

2467. AlShanableh Z, Rehman MEU, Shimko K, Rosenthal J, Viswanathan V, DeSilva RN. Improving Medication Errors in Hospitalized Hemodialysis Patients. Journal of the American Society of Nephrology. 2023;34:896. PubMed PMID: 642701531.

2468. Alshargi O, Albalagi N. Role of drug information centre in detecting medication errors in a tertiary care hospital, central region, Saudi Arabia. Journal of the Pakistan Medical Association. 2023;73(4):755-8. doi: https://dx.doi.org/10.47391/JPMA.922. PubMed PMID: 2023472154; PubMed Central PMCID: PMCLexicomp, Micromedex, IBM.

2469. Alsharif WM, Alyami JH, Qurashi AA, Aljuhani RA, Alqrafi RK, Alraddadi RS, et al. The Motivational Factors of Choosing Diagnostic Radiology as a Profession Among Saudi Radiography Students.

Advances in Medical Education and Practice. 2022;13:955-67. doi: 10.2147/amep.S374194. PubMed PMID: WOS:000849944800001.

2470. Alshatti FA, AlMubarak SaH. The prevalence of medical violation claims and associated predictors at the Eastern Province in Saudi Arabia: A logistic regression analysis. Journal of Forensic and Legal Medicine. 2022;85:102300. doi: https://doi.org/10.1016/j.jflm.2021.102300.

2471. Alshatti FA, AlMubarak SH. The prevalence of medical violation claims and associated predictors at the Eastern Province in Saudi Arabia: A logistic regression analysis. Journal of Forensic and Legal Medicine. 2022;85. doi: 10.1016/j.jflm.2021.102300.

2472. AlShayban DM, Naqvi AA, Alhumaid O, AlQahtani AS, Islam MA, Ghori SA, et al. Association of Disease Knowledge and Medication Adherence Among Out-Patients With Type 2 Diabetes Mellitus in Khobar, Saudi Arabia. Frontiers in Pharmacology. 2020;11. doi: 10.3389/fphar.2020.00060. PubMed PMID: WOS:000519085900001.

2473. AlShayban DM, Naqvi AA, Islam MA, Almaskeen M, Almulla A, Alali M, et al. Patient satisfaction and their willingness to pay for a pharmacist counseling session in hospital and community pharmacies in Saudi healthcare settings. Frontiers in Pharmacology. 2020;11:138.

2474. Alshdaifat E, Sindiani A, Amarin Z, Absy N, AlOsta N, Abuhayyeh HA, et al. Awareness of polycystic ovary syndrome: A university students' perspective. Annals of Medicine and Surgery. 2021;72:103123. doi: https://doi.org/10.1016/j.amsu.2021.103123.

2475. Alshehri A. Regulatory Review, Prices and Safety Communications of Human Immunodeficiency Virus-1 Antiretroviral Drugs in the United States [Ph.D.]. United States -- Massachusetts: Massachusetts College of Pharmacy and Health Sciences; 2017.

2476. Alshehri AA, Cheema E, Yahyouche A, Haque MS, Jalal Z. Evaluating the role and integration of general practice pharmacists in England: a cross-sectional study. International Journal of Clinical Pharmacy. 2021;43(6):1609-18. doi: 10.1007/s11096-021-01291-6.

2477. Alshehri AA, Jalal Z, Cheema E, Haque MS, Jenkins D, Yahyouche A. Impact of the pharmacist-led intervention on the control of medical cardiovascular risk factors for the primary prevention of cardiovascular disease in general practice: A systematic review and meta-analysis of randomised controlled trials. British Journal of Clinical Pharmacology. 2020;86(1):29-38. PubMed PMID: 31777082.

2478. Alshehri AM, Alenazi OS, Almutairi SA, Alali AZ, Almogbel YS, Alonazi RE, et al. Pharmacist Intention to Provide Medication Therapy Management Services in Saudi Arabia: A Study Using the Theory of Planned Behaviour. International Journal of Environmental Research & Public Health [Electronic Resource]. 2022;19(9):26. PubMed PMID: 35564673.

2479. Alshehri AM, Almogbel YS, Alonazi RE, Alshehri WM, Alkhelaifi HA, Almutairi SA, et al. Pharmacists' Knowledge and Intention to Provide Palliative Care Services in Saudi Arabia: Using the Theory of Planned Behaviour. Healthcare. 2023;11(15). doi: 10.3390/healthcare11152173. PubMed PMID: WOS:001045398200001.

2480. Alshehri AM, Barner JC, Brown CM, Rush SK. Medication and health problems and recommendations from MTM services provided by third-year pharmacy students in community pharmacies. Currents in Pharmacy Teaching and Learning. 2015;7(1):70-7. doi: https://doi.org/10.1016/j.cptl.2014.09.020.

2481. Alshehri AM, Barner JC, Brown CM, Rush SK. Medication and health problems and recommendations from MTM services provided by third-year pharmacy students in community phatmacies. Currents in

Pharmacy Teaching and Learning. 2015;7(1):70-7. doi: 10.1016/j.cptl.2014.09.020. PubMed PMID: WOS:000409724800010.

2482. Alshehri AM, Barner JC, Rush S. The impact of a required course on third year pharmacy students' perceived abilities and intentions in providing medication therapy management. Currents in Pharmacy Teaching and Learning. 2019;11(1):94-105. doi: https://doi.org/10.1016/j.cptl.2018.09.014.

2483. Alshehri GH, Keers RN, Ashcroft DM. Frequency and nature of medication errors and adverse drug events in mental health hospitals: a systematic review. Drug Safety. 2017;40:871-86.

2484. Alshehri N, Alanazi A. Pharmacists' Perceptions on Safety Alerts of the Drug Utilization Review (DUR) in Electronic Health Records in a Tertiary Healthcare Hospital. Pharmacy. 2023;11(4):119.

2485. AlShehri SS, Asiri MAM, Marouf MAY, Hawsawi AOA, Alshamrani NA, Alqahtani GAJ, et al. DIAGNOSIS AND MANAGEMENT OF ACUTE COUGH. Indo American Journal of Pharmaceutical Sciences. 2019;6(2):3646-50. doi: 10.5281/zenodo.2562679. PubMed PMID: WOS:000458607000120.

2486. Alsheikh MY, Alasmari MM. A National Survey of Community Pharmacists' Viewpoints About Pharmacovigilance and Adverse Drug Reaction Reporting in Saudi Arabia. Frontiers in Pharmacology. 2022;13:819551. PubMed PMID: 35721182.

2487. Alsheikh MY, Alzahrani MA, Alsharif NA, Altowairqi HM, Asiri SA, Althubaiti BM, et al. Community Pharmacy Staff Knowledge, Opinion and Practice toward Drug Shortages in Saudi Arabia. Saudi Pharmaceutical Journal. 2021;29(12):1383-91. doi: https://doi.org/10.1016/j.jsps.2021.09.001.

2488. Alshogran OY, Alzoubi KH, Khabour OF, Farah S. Patterns of self-medication among medical and nonmedical University students in Jordan. Risk Management and Healthcare Policy. 2018;11:169-76. doi: 10.2147/rmhp.S170181. PubMed PMID: WOS:000444399700002.

2489. Alshogran OY, Hajjar MH, Muflih SM, Alzoubi KH. The role of clinical pharmacist in enhancing hemodialysis patients' adherence and clinical outcomes: a randomized-controlled study. International journal of clinical pharmacy. 2022;44(5):1169-78. doi: 10.1007/s11096-022-01453-0. PubMed PMID: CN-02493928.

2490. Alslubi H, El-Dahiyat F. Patient safety practices among community pharmacists in Abu Dhabi, United Arab Emirates. Journal of Pharmaceutical Health Services Research. 2019;10(2):203-10. doi: 10.1111/jphs.12300.

2491. Alsolmi MS, Mulla HH, Sindi STA, Nazer NWM, Almarzooq MMH, Alhamood MA, et al. AWARENESS LEVEL OF PARENTS TOWARD ANTIBIOTICS THOSE ARE PRESCRIBED TO THEIR CHILDREN IN AL-DAMMAM CITY. Indo American Journal of Pharmaceutical Sciences. 2018;5(12):15969-72. doi: 10.5281/zenodo.2205473. PubMed PMID: WOS:000454558500237.

2492. Alssageer MA, Sherif FM, Mohammed ES, Abd Alsalm SA. Pattern of drug prescribed and drug related problems among hospitalized elderly patients. Mediterranean Journal of Pharmacy and Pharmaceutical Sciences. 2023;2(2):66-78.

2493. Alston G, Hanrahan C. Can a pharmacist reduce annual costs for medicare part D enrollees? Consultant Pharmacist. 2011;26(3):182-9. doi: 10.4140/TCP.n.2011.182.

2494. Alston GL, Blizzard JC. The value prescription: Relative value theorem as a call to action. Research in Social & Administrative Pharmacy. 2012;8(4):338-48. doi: 10.1016/j.sapharm.2011.06.004. PubMed PMID: WOS:000307326100009.

2495. Alston GL, Lane D, Wright NJD. The methodology for the early identification of students at risk for failure in a professional degree program. Currents in Pharmacy Teaching and Learning. 2014;6(6):798-806. doi: https://doi.org/10.1016/j.cptl.2014.07.006.

2496. Alsulami Z. Double checking the administration of medicines : what is the evidence? A systematic review.

2497. Alsulami Z, Conroy S, Choonara I. Double checking the administration of medicines: what is the evidence? A systematic review. Archives of Disease in Childhood. 2012;97(9):833-7. doi: 10.1136/archdischild-2011-301093. PubMed PMID: 104501432. Language: English. Entry Date: 20121130. Revision Date: 20200708. Publication Type: Journal Article.

2498. Alsulami Z, Conroy S, Choonara I. Medication errors in the Middle East countries: A systematic review of the literature. European Journal of Clinical Pharmacology. 2013;69(4):995-1008. doi: 10.1007/s00228-012-1435-y. PubMed PMID: 128418547. Language: English. Entry Date: 20180314. Revision Date: 20180612. Publication Type: Article.

2499. Alsultan MS, Khurshid F, Mayet AY, Al-jedai AH. Hospital pharmacy practice in Saudi Arabia: Dispensing and administration in the Riyadh region. Saudi Pharmaceutical Journal. 2012;20(4):307-15. doi: https://doi.org/10.1016/j.jsps.2012.05.003.

2500. Alsultan MS, Mayet AY, Khurshid F, Al-jedai AH. Hospital pharmacy practice in Saudi Arabia: Drug monitoring and patient education in the Riyadh region. Saudi Pharmaceutical Journal. 2013;21(4):361-70. doi: https://doi.org/10.1016/j.jsps.2012.12.006.

2501. Alsuwaidan S, Malone DC, Billups SJ, Carter BL, Investigators I. Characteristics of ambulatory care clinics and pharmacists in Veterans Affairs medical centers. American Journal of Health-System Pharmacy. 1998;55(1):68-72. doi: 10.1093/ajhp/55.1.68. PubMed PMID: WOS:000071389000015.

2502. Alsuwayni B, Alhossan A. Impact of clinical pharmacist-led diabetes management clinic on health outcomes at an academic hospital in Riyadh, Saudi Arabia: A prospective cohort study. Saudi Pharmaceutical Journal. 2020;28(12):1756-9. doi: 10.1016/j.jsps.2020.11.002. PubMed PMID: WOS:000605479700001.

2503. Altagracia M M, Kravzov J J, Moreno S R, Ríos C C, Vázquez M E. Self-medication in rural and urban communities in the state of Guerrero, Mexico. Revista Mexicana de Ciencias Farmaceuticas. 2003;34(2):27-35.

2504. Altalhi F, Altalhi A, Magliah Z, Abushal Z, Althaqafi A, Falemban A, et al. Development and evaluation of clinical reasoning using ‘think aloud’ approach in pharmacy undergraduates – A mixed-methods study. Saudi Pharmaceutical Journal. 2021;29(11):1250-7. doi: https://doi.org/10.1016/j.jsps.2021.10.003.

2505. Altalib HH, Galluzzo D, Argraves S, Goulet J, Bornovski Y, Cheung KH, et al. Managing Functional Neurological Disorders: Protocol of a Cohort Study on Psychogenic Non-Epileptic Seizures Study. Neuropsychiatric Disease and Treatment. 2019;15:3557-68. doi: 10.2147/ndt.S234852. PubMed PMID: WOS:000505023900001.

2506. Altan A, Ozkan O, Demirtunc R, Okuyan B, Izzettin FV, Sancar M. Importance of medication dose adjustment in hospitalized patients with impaired renal function at internal medicine ward: the possible impact of clinical pharmacist. International journal of clinical pharmacy Conference: 46th ESCP symposium on clinical pharmacy Germany. 2018;40(1):275. doi: 10.1007/s11096-017-0565-9. PubMed PMID: CN-01468254.

2507. Altavela JL, Dorward KM, Sorrento TA, Diehl KM, Wyman CA. Population health management: An independent physician organization approach. American Journal of Health-System Pharmacy. 2017;74(18):1477-85. doi: 10.2146/ajhp161009.

2508. Altavela JL, Jones MK, Ritter M. A prospective trial of a clinical pharmacy intervention in a primary care practice in a capitated payment system. Journal of Managed Care Pharmacy. 2008;14(9):831-43. doi: 10.18553/jmcp.2008.14.9.831.

2509. Altawalbeh SM, Alshogran OY, Al-Sawalha NA, Al-Saleem MM. Clinical Outcomes and Direct Medical Expenditures Associated With Intensive Care Unit Admission for Inpatients With COVID-19 in Jordan: A Retrospective Cohort Study. Value in Health Regional Issues. 2023;33:76-82. doi: https://doi.org/10.1016/j.vhri.2022.09.002.

2510. Altawalbeh SM, Alshogran OY, Al-Sawalha NA, Al-Saleem MM. Clinical Outcomes and Direct Medical Expenditures Associated With Intensive Care Unit Admission for Inpatients With COVID-19 in Jordan: A Retrospective Cohort Study. Value in Health Regional Issues. 2023;33:76-82. doi: https://doi.org/10.1016/j.vhri.2022.09.002.

2511. Altawalbeh SM, Hijazi B, Kufoof L, Basheti IA. Health expenditures of asthma-COPD overlap in Northern Jordan. Plos One. 2021;16(9). doi: 10.1371/journal.pone.0257566. PubMed PMID: WOS:000746527400018.

2512. Altawalbeh SM, Ibrahim IA, Al-Shatnawi SF. Influence of pharmaceutical promotion on prescribers in Jordan. International Journal of Clinical Pharmacy. 2020;42(2):744-55. doi: 10.1007/s11096-020-01006-3. PubMed PMID: WOS:000529692500051.

2513. Altawalbeh SM, Manoon NA, Ababneh MA, Basheti IA. Respiratory tract infection-induced asthma exacerbations in adults with asthma: assessing predictors and outcomes. Journal of asthma. 2019. doi: 10.1080/02770903.2019.1568454. PubMed PMID: CN-01792904.

2514. Altawalbeh SM, Manoon NA, Ababneh MA, Basheti IA. Respiratory tract infection-induced asthma exacerbations in adults with asthma: assessing predictors and outcomes. Journal of Asthma. 2020;57(3):231-40. doi: 10.1080/02770903.2019.1568454. PubMed PMID: WOS:000516747900001.

2515. Altaweel ABH, Abusalah L, Qato DM. Near Field Communication Detection System for Drug-Drug Interactions. Procedia Computer Science. 2018;140:314-23. doi: https://doi.org/10.1016/j.procs.2018.10.312.

2516. Alte D, Weitschies W, Ritter CA. Evaluation of consultation in community pharmacies with mystery shoppers. Annals of Pharmacotherapy. 2007;41(6):1023-30. doi: 10.1345/aph.1H565.

2517. Altenau B, Crisp CC, Devaiah CG, Lambers DS. Randomized controlled trial of intravenous acetaminophen for postcesarean delivery pain control. American journal of obstetrics and gynecology. 2017;217(3):362.e1-.e6. doi: 10.1016/j.ajog.2017.04.030. PubMed PMID: CN-01410489.

2518. Althagafi A, Alshibani M, Alshehri S, Alqarni A, Baharith M, Alqurashi S. Evaluation of Pharmacy-Led Post-Discharge Follow-Up on Transitional Care Management in a Tertiary Academic Hospital: An Observational Study. Cureus. 2023;15(8).

2519. Althausen PL, Bray TJ, Hill AD. Reno orthopaedic trauma fellowship business curriculum. Journal of Orthopaedic Trauma. 2014;28(7 SUPPL.):S3-S11. doi: 10.1097/BOT.0000000000000144.

2520. Altice FL, Mezger JA, Hodges J, Bruce RD, Marinovich A, Walton M, et al. Developing a directly administered antiretroviral therapy intervention for HIV-infected drug users: implications for program

replication. Clinical infectious diseases. 2004;38 Suppl 5:S376-87. doi: 10.1086/421400. PubMed PMID: CN-00468426.

2521. Altinbas A, Baser E, Burkan A, Ertugay E, Kariburyo MF, Baser O. Retrospective analysis of total direct medical costs associated with hepatitis B patients with oral antiviral versus pegylated interferon therapy in Turkey. Journal of Viral Hepatitis. 2014;21(11):794-801. doi: 10.1111/jvh.12206. PubMed PMID: WOS:000342844900009.

2522. Altinel S. Gelişmiş ülkelerde Uygulanmakta Olan ilaç güvenliği (Farmakovijilans) Sistemlerinin Incelenmesi, Türkiye'Deki Mevcut yapılanma Ile karşılaştırılması Ve ülkemizde oluşturulması amaçlanan Denetim Sisteminin araştırılması [Master's]. Turkey: Marmara Universitesi (Turkey); 2010.

2523. Altiner A, Bell J, Duerden M, Essack S, Kozlov R, Noonan L, et al. More action, less resistance: report of the 2014 summit of the Global Respiratory Infection Partnership. International Journal of Pharmacy Practice. 2015;23(5):370-7. PubMed PMID: 25711969.

2524. Altiner A, Bell J, Duerden M, Essack S, Kozlov R, Noonan L, et al., editors. More action, less resistance: Report of the 2014 summit of the Global Respiratory Infection Partnership. International Journal of Pharmacy Practice; 2015.

2525. Altiner A, Bell J, Duerden M, Essack S, Kozlov R, Noonan L, et al. More action, less resistance: report of the 2014 summit of the Global Respiratory Infection Partnership. International Journal of Pharmacy Practice. 2015;23(5):370-7. doi: 10.1111/ijpp.12177. PubMed PMID: 109539972. Language: English. Entry Date: 20150923. Revision Date: 20191106. Publication Type: Article.

2526. Altman DG. Poor-quality medical research: what can journals do? Jama. 2002;287(21):2765-7.

2527. Altman DJ. THE ROLES OF THE PHARMACEUTICAL INDUSTRY AND DRUG DEVELOPMENT IN DERMATOLOGY AND DERMATOLOGIC HEALTH CARE. Dermatologic Clinics. 2000;18(2):287-96. doi: https://doi.org/10.1016/S0733-8635(05)70174-1.

2528. Altok M, Kim B, Patel BB, Shih YCT, Ward JF, McRae SE, et al. Cost and efficacy comparison of five prostate biopsy modalities: a platform for integrating cost into novel-platform comparative research. Prostate Cancer and Prostatic Diseases. 2018;21(4):524-32. doi: 10.1038/s41391-018-0056-7. PubMed PMID: WOS:000452400900009.

2529. Alton S, Farndon L. The impact of community pharmacy-led medicines management support for people with COPD. British Journal of Community Nursing. 2018;23(6):214-9. doi: 10.12968/bjcn.2018.23.6.214.

2530. Altowaijri A, Phillips CJ, Fitzsimmons D. A systematic review of the clinical and economic effectiveness of clinical pharmacist intervention in secondary prevention of cardiovascular disease. Journal of Managed Care Pharmacy. 2013;19(5):408-16. doi: 10.18553/jmcp.2013.19.5.408.

2531. Altuwaijri MM, Bahanshal A, Almehaid M. Implementation of computerized physician order entry in National Guard Hospitals: assessment of critical success factors. Journal of Family and Community Medicine. 2011;18(3):143.

2532. Altyar A, Sadoun SA, Aljohani SS, Alradadi RS. Evaluating Pharmacy Practice in Hospital Settings in Jeddah City, Saudi Arabia: Dispensing and Administration—2019. Hospital Pharmacy. 2022;57(1):32-7. doi: 10.1177/0018578720970470. PubMed PMID: 156651422. Language: English. Entry Date: 20220526. Revision Date: 20220526. Publication Type: Article.

2533. Alucema A, ChavarrÃ-A N, Valdés M. Patterns of self-medication in customers of a community pharmacy in the Antofagasta city. Journal of Pharmacy and Pharmacognosy Research. 2013;1(2):54-63.

2534. Alucema A, González SC, Valenzuela I, Valdés M. Pharmaceutical intervention in menopausal patients with hormone replacement therapy in a community pharmacy from Antofagasta. Journal of Pharmacy and Pharmacognosy Research. 2015;3(1):24-36.

2535. Aluga D, Nnyanzi LA, King N, Okolie EA, Raby P. Effect of Electronic Prescribing Compared to Paper-Based (Handwritten) Prescribing on Primary Medication Adherence in an Outpatient Setting: A Systematic Review. Applied Clinical Informatics. 2021;12(04):845-55. doi: 10.1055/s-0041-1735182. PubMed PMID: WOS:000688207900003.

2536. Aluh DO, Anyachebelu OC, Ajaraonye CI. Comparison of pharmacists' mental health literacy: Developed versus developing countries. Journal of the American Pharmacists Association. 2020;60(5):S64-S72. doi: 10.1016/j.japh.2020.05.003. PubMed PMID: WOS:000580037500009.

2537. Alvarado A C, Ossa G X, Bustos M L. Errors in prescriptions and their preparation at the outpatient pharmacy of a regional hospital. Revista Medica de Chile. 2017;145(1):33-40. doi: 10.4067/S0034-98872017000100005.

2538. Alvarado AC, Ossa GX, Bustos ML. Errors in prescriptions and their preparation at the outpatient pharmacy of a regional hospital. [Spanish]. Revista medica de Chile. 2017;145(1):33-40. PubMed PMID: 617775338.

2539. Alvarado AC, Ossa GX, Bustos ML. [Errors in prescriptions and their preparation at the outpatient pharmacy of a regional hospital]. Revista Medica de Chile. 2017;145(1):33-40. PubMed PMID: 28393967.

2540. Alvarado C, Ossa X, Bustos L. Adaptation and validation of checklists to detect medication prescription and dispensing errors. Revista Medica De Chile. 2014;142(12):1547-52. doi: 10.4067/s0034-98872014001200008. PubMed PMID: WOS:000348090900008.

2541. Alvarez de Toledo F, Arcos González P, Eyaralar Riera T, Abal Ferrer FF, Dago Martínez A, Cabiedes Miragaya L, et al. Pharmaceutical care in people who have had acute coronary episodes (TOMCOR study). Revista espanola de salud publica. 2001;75(4):375-87. PubMed PMID: CN-00375037.

2542. Alvarez OP, Core BV, Fernandez JCR, Gonzalez MIB, Gonzalez LB. 'KAIROS' project: ambulatory monitoring of blood pressure in community pharmacies. Monitoring and follow-up of blood pressure in hypertensive elderly patients under treatment. Farmaceuticos Comunitarios. 2018;10(2):21-6. doi: 10.5672/FC.2173-9218.(2018/Vol10).002.04. PubMed PMID: WOS:000437662000004.

2543. Álvarez Otero S, Cid Fariñas C, Martínez Ques Á A, Varela Correa JJ, López Otero MJ. Conciliation in medication in a short stay medical unit. Metas de Enfermería. 2009;12(6):70-4. PubMed PMID: 105397701. Language: Spanish. Entry Date: 20090904. Revision Date: 20150820. Publication Type: Journal Article.

2544. Alvarez SJ, Larmour I, Tapley N, Buick M, Greene L, Hines L, et al. Education Outreach to the Community: Does Hospital Pharmacy Have a Role? Australian Journal of Hospital Pharmacy. 2001;31(1):13-7. doi: 10.1002/jppr200131113.

2545. Alvarez-Risco A, Del-Aguila-Arcentales S. Prescription errors as a barrier to pharmaceutical care in public health facilities: Experience Peru. Pharmaceutical Care Espana. 2015;17(6):725-31. PubMed PMID: WOS:000420554400002.

2546. Alvarez-Risco A, Mil JFv. Pharmaceutical care in community pharmacies: practice and research in

Peru. Annals of Pharmacotherapy. 2007;41(12):2032-7.

2547. Alvarez-Risco A, van Mil JW. Pharmaceutical care in community pharmacies: practice and research in Peru. Annals of Pharmacotherapy. 2007;41(12):2032-7. PubMed PMID: 17940126.

2548. Alvarez-Risco A, van Mil JWF. Pharmaceutical care in community pharmacies: Practice and researchin Peru. Annals of Pharmacotherapy. 2007;41(12):2032-7. doi: 10.1345/aph.1K117. PubMed PMID: WOS:000251280500014.

2549. Alvarez-Risco A, Yi-Fang L, Del-Aguila-Arcentales S, Pei-Wen Y. Barriers to pharmaceutical care practice in pharmacies in Tainan, Taiwan. Pharmaceutical Care Espana. 2017;19(2):58-68. PubMed PMID: WOS:000404292300002.

2550. Alvaro-Alonso EA, Aldeyab M, Ashfield L, Gilmore F, Perez-Encinas M. "International Centres of Excellence in Hospital Pharmacy"; a SEFH new initiative; the role of the clinical pharmacist in the hospital antibiotic stewardship in Northern Ireland. Farmacia Hospitalaria. 2016;40(4):233-6. PubMed PMID: 27571491.

2551. Álvaro-Alonso EA, Tejedor-Prado P, Aguilar-Ros A, Escobar-Rodríguez I. Centralization of the methadone maintenance plan in a hospital pharmacy department in the Community of Madrid. Farmacia hospitalaria : organo oficial de expresion cientifica de la Sociedad Espanola de Farmacia Hospitalaria. 2020;44(5):185-91. doi: 10.7399/fh.11384.

2552. Alvaro-Gracia JM, Arredondo M, Daudén E, Meca-Lallana V, Morell A, Gisbert JP, et al. Launch and preliminary analysis of Hospital de La Princesa's inter-specialists biological therapies unit. Reumatología Clínica. 2019;15(2):63-8. doi: https://doi.org/10.1016/j.reuma.2018.11.008.

2553. Alverson SP. Pharmacists' implementation and response to drug utilization review requirements of the 1990 Omnibus Budget Reconciliation Act [D.P.A.]. United States -- California: University of Southern California; 1995.

2554. Alves AL. Efficacy and Safety of Biological Treatments of Multiple Sclerosis [Master's]. Portugal: Universidade da Beira Interior (Portugal); 2013.

2555. Alves ASM. Identificação de Opções Terapêuticas não Satisfeitas Pelos Medicamentos Comercializados com Autorização de Introdução no Mercado em Portugal: Experiência Profissionalizante na Vertente de Farmácia Comunitária, Hospitalar e Investigação [M.Sc.]. Portugal: Universidade da Beira Interior (Portugal); 2016.

2556. Alves Carneiro VL, González-Méijome JM. Prevalence of refractive error in Portugal estimated from ophthalmic lens manufacturing data: Ten-years analysis. PLoS ONE. 2023;17(4):1-12. doi: 10.1371/journal.pone.0284703. PubMed PMID: 163251045.

2557. Alves CEN. Análise da Terapêutica de Antipsicóticos no Centro Hospitalar Universitário Cova da Beira Experiência Profissionalizante na Vertente de Investigação e Farmácia Comunitária [M.Sc.]. Portugal: Universidade da Beira Interior (Portugal); 2019.

2558. Alves da Costa F, Paulino E. Chapter 8 - Pharmacist-led interventions to promote cardiovascular health: A review of studies from Portugal. In: Babar Z-U-D, editor. Pharmacy Practice Research Case Studies: Academic Press; 2021. p. 155-86.

2559. Alves Da Costa F, Paulino E. Pharmacist-led interventions to promote cardiovascular health: A review of studies from Portugal. Pharmacy Practice Research Case Studies2021. p. 155-86.

2560. Alves de Souza CM, Paranhos J, Hasenclever L. Comparison between maximum consumer prices for medicines and prices practiced on the internet in Brazil: Misalignments and regulatory distortions. Ciencia e Saude Coletiva. 2021;26(11):5463-80. doi: 10.1590/1413-812320212611.44082020.

2561. Alves DFF. Os Extratos de Plantas Medicinais Na Prevenção e No Tratamento da Hiperplasia Benigna da Próstata [M.Sc.]. Portugal: Universidade da Beira Interior (Portugal); 2020.

2562. Alves EA. Krokodil: The Resurface of an Old Drug [Ph.D.]. Portugal: Universidade do Porto (Portugal); 2017.

2563. Alves EA, Grund JPC, Afonso CM, Netto ADP, Carvalho F, Dinis-Oliveira RJ. The harmful chemistry behind krokodil (desomorphine) synthesis and mechanisms of toxicity. Forensic Science International. 2015;249:207-13. doi: 10.1016/j.forsciint.2015.02.001. PubMed PMID: WOS:000351947300035.

2564. Alvesson HM, Lindelow M, Khanthaphat B, Laflamme L. Coping with uncertainty during healthcare-seeking in Lao PDR. Bmc International Health and Human Rights. 2013;13. doi: 10.1186/1472-698x-13-28. PubMed PMID: WOS:000320872300001.

2565. Alvin M, Picco L, Wood P, Mnatzaganian G, Nielsen S. Community pharmacists' preparedness to intervene with concerns around prescription opioids: findings from a nationally representative survey. International Journal of Clinical Pharmacy. 2021;43(2):411-9. doi: 10.1007/s11096-020-01152-8.

2566. Alwabel AM, Bawazir AA, Al-Surimi K. Assessment of patient safety culture among pharmacists in Riyadh hospitals, Saudi Arabia. Journal of Infection and Public Health. 2015;8(4):402. doi: https://doi.org/10.1016/j.jiph.2015.04.016.

2567. Alwan AM, Cockburn A, Coll A, Kinnear M. Evaluation of a pharmacist-led diabetic cardiovascular risk (DCVR) clinic: a feasibility study. International journal of clinical pharmacy. 2013;35(5):872-. doi: 10.1007/s11096-013-9801-0. PubMed PMID: CN-01006237.

2568. Alwan MA. Evaluation of pharmacist work activities before and after implementation of computerized provider order entry [M.S.]. United States -- Utah: The University of Utah; 2010.

2569. Alwhaibi A, Alenazi M, Almadi B, Alotaibi A, Alshehri SM, Shakeel F. A practical method for oral administration of isotretinoin in pediatric oncology patient: A case study of neuroblastoma. Journal of Oncology Pharmacy Practice. 2023;29(3):755-9. doi: https://dx.doi.org/10.1177/10781552221117814. PubMed PMID: 2018411858.

2570. Alwhaibi A, Alrwaished A, Binobydaan SM, Alawwad S, Wajid S, Bablghaith S, et al. Role of pharmacist during COVID-19 pandemic: A retrospective study focused on critically ill COVID-19 patients. Saudi Pharmaceutical Journal. 2021;29(9):1050-5. doi: https://doi.org/10.1016/j.jsps.2021.07.010.

2571. Alwhaibi M. Inappropriate Medications Use and Polypharmacy among Older Adults with Anxiety Disorder. Journal of Clinical Medicine. 2023;12(13):4195. doi: 10.3390/jcm12134195. PubMed PMID: 164924912.

2572. Alwhaibi M, Al Aloola NA. Associations between Stress, Anxiety, Depression and Sleep Quality among Healthcare Students. Journal of Clinical Medicine. 2023;12(13):4340. doi: 10.3390/jcm12134340. PubMed PMID: 164925057.

2573. Alwhaibi M, Alkofide H. Potentially Inappropriate Medications Use among Older Adults with Dyslipidaemia. Journal of Clinical Medicine. 2023;12(12):4063. doi: 10.3390/jcm12124063. PubMed PMID: 164652056.

2574. Alwhaibi M, AlRuthia Y, Alhawassi TM, Almalag H, Alsalloum H, Balkhi B. Polypharmacy and comorbidities among ambulatory cancer patients: A cross-sectional retrospective study. Journal of Oncology Pharmacy Practice. 2020;26(5):1052-9. PubMed PMID: 31619132.

2575. Alwhaibi M, Asser WM, A. Al Aloola N, Alsalem N, Almomen A, Alhawassi TM. Evaluating the frequency, consumers' motivation and perception of online medicinal, herbal, and health products purchase safety in Saudi Arabia. Saudi Pharmaceutical Journal. 2021;29(2):166-72. doi: https://doi.org/10.1016/j.jsps.2020.12.017.

2576. Alwhaibi M, Asser WM, Al Aloola NA, Alsalem N, Almomen A, Alhawassi TM. Evaluating the frequency, consumers' motivation and perception of online medicinal, herbal, and health products purchase safety in Saudi Arabia. Saudi Pharmaceutical Journal. 2021;29(2):166-72. doi: 10.1016/j.jsps.2020.12.017. PubMed PMID: WOS:000620997000005.

2577. Alwhaibi M, Balkhi B. Gender Differences in Potentially Inappropriate Medication Use among Older Adults. Pharmaceuticals (14248247). 2023;16(6):869. doi: 10.3390/ph16060869. PubMed PMID: 164702485.

2578. Alwidyan T, McCorry NK, Parsons C. Healthcare professionals' perspectives of deprescribing in older patients at the end of life in hospice care: a qualitative study using the Theoretical Domains Framework. International Journal of Pharmacy Practice. 2023;31(3):305-13. doi: https://dx.doi.org/10.1093/ijpp/riad005. PubMed PMID: 2026231217.

2579. Alwon B. Developing a national strategy for combating counterfeit medicines [Ph.D.]. England: University of East Anglia (United Kingdom); 2015.

2580. Aly M, Garcia-Cardenas V, Williams KA, Benrimoj SI. A qualitative study of stakeholder views and experiences of minor ailment services in the United Kingdom. Research in Social & Administrative Pharmacy. 2019;15(5):496-504. doi: 10.1016/j.sapharm.2018.06.014. PubMed PMID: WOS:000468482900069.

2581. Aly M, Schneider CR, Sukkar MB, Lucas C. Educational needs of community pharmacy staff in minor ailment service delivery: A systematic scoping review. Currents in Pharmacy Teaching and Learning. 2020;12(10):1269-87. doi: 10.1016/j.cptl.2020.05.013.

2582. Aly M, Schneider CR, Sukkar MB, Lucas C. Exploration of health professional stakeholders' views and experiences regarding minor ailments services' education, training and assessment. International Journal of Clinical Pharmacy. 2021;43(3):654-65. doi: 10.1007/s11096-020-01177-z. PubMed PMID: WOS:000587051500001.

2583. Alyami AA. Smart e-health system for real-time tracking and monitoring of patients, staff and assets for healthcare decision support in saudi arabia [Ph.D.]. England: Staffordshire University (United Kingdom); 2018.

2584. Alyami HS, Naser AY, Alyami MH, Garsan MA, Al-Nahdi AY, Alalhareth IS, et al. Drug shortages in Najran region, Saudi Arabia: A cross-sectional survey. Clinical Epidemiology and Global Health. 2023;21:101305. doi: https://doi.org/10.1016/j.cegh.2023.101305.

2585. Alyami MH, Naser AY, Alswar HS, Alyami HS, Alyami AH, Al Sulayyim HJ. Medication errors in Najran, Saudi Arabia: Reporting, responsibility, and characteristics: A cross-sectional study. Saudi Pharmaceutical Journal. 2022;30(4):329-36. doi: https://doi.org/10.1016/j.jsps.2022.02.005.

2586. Alyami SH, Al Rizq HMM, Lasloom NMN, Alamri HMM, MaraiHadiAlAmer M.

COMPREHENSIVE REVIEW ON THE IMPACT OF PHARMACISTS ON MEDICATION SAFETY AND ADHERENCE. Chelonian Research Foundation. 2023;18(1):478-91.

2587. Alzaabi F, Almheiri K, Al-Saeedy DYM, Zachariah S. Value of pharmacist interventions: patient medication adherence measured with clinical outcomes. International Journal of Pharmacy Practice. 2020;28(4):413-4. PubMed PMID: 32215975.

2588. Alzahrani AA, Alwhaibi MM, Asiri YA, Kamal KM, Alhawassi TM. Description of pharmacists' reported interventions to prevent prescribing errors among in hospital inpatients: a cross sectional retrospective study. BMC Health Services Research. 2021;21(1):1-7. doi: 10.1186/s12913-021-06418-z. PubMed PMID: 150249781. Language: English. Entry Date: In Process. Revision Date: 20210923. Publication Type: journal article. Journal Subset: Biomedical.

2589. Alzahrani F, Alahmadi YM, Al Thagfan SS, Alolayan S, Elbadawy HM. Migraine Management in Community Pharmacies: Knowledge, Attitude and Practice Patterns of Pharmacists in Saudi Arabia. Pharmacy. 2023;11(5). doi: 10.3390/pharmacy11050155. PubMed PMID: WOS:001093492500001.

2590. Alzahrani F, Sandaqji Y, Alharrah A, Alblowi R, Alrehaili S, Mohammed-Saeid W. Community Pharmacies’ Promotion of Smoking Cessation Support Services in Saudi Arabia: Examining Current Practice and Barriers. Healthcare (Switzerland). 2023;11(13). doi: 10.3390/healthcare11131841.

2591. Alzahrani M, Alhindi T, Almutairi A, Aldajani M, Sami W. Frequency of using non-prescribed medication in Majmaah city, Saudi Arabia—A cross sectional study. Journal of the Pakistan Medical Association. 2015;65(8):825-8.

2592. Alzahrani M, Alhindi T, Almutairi A, Aldajani M, Sami W. Frequency of using non-prescribed medication in Majmaah city, Saudi Arabia- A cross sectional study. Journal of the Pakistan Medical Association. 2015;65(8):825-8. PubMed PMID: WOS:000360444600006.

2593. Alzahrany MS. Pulmonary gas transport and drug delivery in a patient specific lung model during invasive high frequency oscillatory ventilation [Ph.D.]. United States -- Pennsylvania: Lehigh University; 2014.

2594. Alzayer R, Almansour HA, Basheti I, Chaar B, Al Aloola N, Saini B. Asthma patients in Saudi Arabia - preferences, health beliefs and experiences that shape asthma management. Ethnicity & Health. 2022;27(4):877-93. doi: 10.1080/13557858.2020.1817868. PubMed PMID: WOS:000569426100001.

2595. Alzayer R, Svedin E, Rizvi SA, Basheti I, Chaar B, Saini B. Pharmacists' experience of asthma management in culturally and linguistically diverse (CALD) patients. Research in Social & Administrative Pharmacy. 2021;17(2):315-25. doi: 10.1016/j.sapharm.2020.04.009. PubMed PMID: WOS:000604732400006.

2596. Alzeer AA, Alfantoukh LA, Theneyan A, Bin Eid F, Almangour TA, Alshememry AK, et al. The influence of demographics on influenza vaccine awareness and hesitancy among adults visiting educational hospital in Saudi Arabia. Saudi Pharmaceutical Journal. 2021;29(2):188-93. doi: https://doi.org/10.1016/j.jsps.2021.01.001.

2597. Alzubaidi FA, Al-Saigh RJ, Oubaid EN, Liapi C, Al-Humadi HW. In vitro Quality Assessment and Antibacterial Efficacy of Ciprofloxacin Tablets from Different Pharmaceutical Companies in Community Pharmacies in Iraq. Journal of Pharmaceutical Negative Results. 2022;13(2):113-8. doi: 10.47750/pnr.2022.13.02.018.

2598. Alzubaidi H, Austin Z, Saidawi W, Rees VW. Exploring the quality of smoking cessation in

community pharmacies: A simulated patient study. Research in Social and Administrative Pharmacy. 2022;18(6):2997-3003. doi: https://doi.org/10.1016/j.sapharm.2021.07.013.

2599. Alzubaidi H, Hasan S, Saidawi W, Mc Namara K, Chandir S, Krass I. Outcomes of a novel pharmacy screening intervention to address the burden of type 2 diabetes and cardiovascular disease in an Arabic-speaking country. Diabetic Medicine. 2021;38(8). doi: 10.1111/dme.14598.

2600. Alzubaidi H, Mc Namara K, Browning C. Time to question diabetes self-management support for Arabic-speaking migrants: exploring a new model of care. Diabetic medicine. 2017;34(3):348-55.

2601. Alzubaidi H, Mc Namara K, Saidawi W, Hasan S, Krass I. Pharmacists' experiences and views on providing screening services: An international comparison. Research in Social & Administrative Pharmacy. 2020;16(11):1558-68. doi: 10.1016/j.sapharm.2020.07.019. PubMed PMID: WOS:000587607700008.

2602. Alzubaidi H, Mc Namara K, Versace VL. Predictors of effective therapeutic relationships between pharmacists and patients with type 2 diabetes: Comparison between Arabic-speaking and Caucasian English-speaking patients. Research in Social and Administrative Pharmacy. 2018;14(11):1064-71. doi: https://doi.org/10.1016/j.sapharm.2017.11.013.

2603. Alzubaidi H, Namara KM, Saidawi W, Hasan S, Krass I. Pharmacists' experiences and views on providing screening services: An international comparison. Research in Social and Administrative Pharmacy. 2020;16(11):1558-68. doi: https://doi.org/10.1016/j.sapharm.2020.07.019.

2604. Alzubaidi H, Saidawi W, Hussein A, Hasan S. Critical Role of Pharmacists in Caring for Alzheimer's Disease Patients. Journal of Alzheimers Disease. 2020;73(3):1253-64. doi: 10.3233/jad-190804. PubMed PMID: WOS:000512319400035.

2605. Alzubaidi H, Saidawi W, Mc Namara K. Pharmacist views and pharmacy capacity to deliver professional services in the United Arab Emirates. International Journal of Clinical Pharmacy. 2018;40(5):1106-15. doi: 10.1007/s11096-018-0662-4.

2606. Alzubaidi HT, Chandir S, Hasan S, McNamara K, Cox R, Krass I. Diabetes and cardiovascular disease risk screening model in community pharmacies in a developing primary healthcare system: A feasibility study. BMJ Open. 2019;9(11). doi: 10.1136/bmjopen-2019-031246.

2607. Ama PFM, Ama VJ, Kamga JP, Sato G, Laure P. Dopage et sport : connaissances et attitudes des pharmaciens de la ville de Yaoundé. Science & Sports. 2002;17(3):135-9. doi: https://doi.org/10.1016/S0765-1597(02)00132-6.

2608. Amadi C, Tsui EK. How the quality of essential medicines is perceived and maintained through the pharmaceutical supply chain: A perspective from stakeholders in Nigeria. Research in Social and Administrative Pharmacy. 2019;15(11):1344-57. doi: 10.1016/j.sapharm.2018.11.011.

2609. Amado AY, Frye C, Holland W, Holland CR, Rhodes LA, Marciniak MW. Development of a process to improve medication adherence in patients with rheumatoid arthritis in the specialty pharmacy setting. Journal of the American Pharmacists Association. 2020;60(3, Supplement):S61-S4.e1. doi: https://doi.org/10.1016/j.japh.2020.03.022.

2610. Amado AY, Frye C, Holland W, Holland CR, Rhodes LA, Marciniak MW, editors. Development of a process to improve medication adherence in patients with rheumatoid arthritis in the specialty pharmacy setting. Journal of the American Pharmacists Association; 2020.

2611. Amado I, Brenugat-Herne L, Orriols E, Desombre C, Dos Santos M, Prost Z, et al. A Serious Game to

Improve Cognitive Functions in Schizophrenia: A Pilot Study. Frontiers in Psychiatry. 2016;7. doi: 10.3389/fpsyt.2016.00064. PubMed PMID: WOS:000374449100001.

2612. Amador-Fernández N, Baechler T, Quintana-Bárcena P, Berger J. Documentation of drug related problems and their management in community pharmacy: Data evolution over six years. Research in Social and Administrative Pharmacy. 2023;19(11):1480-5. doi: https://doi.org/10.1016/j.sapharm.2023.07.001.

2613. Amador-Fernández N, Benrimoj SI, de Labry Lima AO, García-Cárdenas V, Gastelurrutia MÁ, Berger J, et al. Strengthening patients' triage in community pharmacies: A cluster randomised controlled trial to evaluate the clinical impact of a minor ailment service. PLoS ONE. 2022;17(10 October). doi: 10.1371/journal.pone.0275252.

2614. Amador-Fernandez N, Benrimoj SI, Fernandez VJB, Catala MTC, Molina VC, Jimenez OE, et al. Collaborative care protocols for the management of minor ailments agreed between pharmacists and medical practitioners: INDICA+PRO Study. Farmaceuticos Comunitarios. 2019;11(4):21-31. doi: 10.5672/FC.2173-9218.(2019/Vol11).004.03. PubMed PMID: WOS:000504905600003.

2615. Amador-Fernández N, Benrimoj SI, García-Cárdenas V, Gastelurrutia MÁ, Graham EL, Palomo-Llinares R, et al. Identification of high-risk patients for referral through machine learning assisting the decision making to manage minor ailments in community pharmacies. Frontiers in Pharmacology. 2023;14:1105434.

2616. Amador-Fernández N, Benrimoj SI, García-Cárdenas V, Gastelurrutia MÁ, Graham EL, Palomo-Llinares R, et al. Identification of high-risk patients for referral through machine learning assisting the decision making to manage minor ailments in community pharmacies. Frontiers in Pharmacology. 2023;14. doi: 10.3389/fphar.2023.1105434.

2617. Amador-Fernández N, Benrimoj SI, García-Cárdenas V, Gastelurrutia MA, Graham EL, Palomo-Llinares R, et al. Identification of high-risk patients for referral through machine learning assisting the decision making to manage minor ailments in community pharmacies. Frontiers in Pharmacology. 2023;14. doi: 10.3389/fphar.2023.1105434. PubMed PMID: WOS:001036238400001.

2618. Amador-Fernández N, Benrimoj SI, García-Mochón L, García-Cárdenas V, Dineen-Griffin S, Gastelurrutia M, et al. A cost utility analysis alongside a cluster-randomised trial evaluating a minor ailment service compared to usual care in community pharmacy. BMC health services research. 2021;21(1):1253. doi: 10.1186/s12913-021-07188-4. PubMed PMID: CN-02347218.

2619. Amador-Fernandez N, Benrimoj SI, Garcia-Mochon L, Garcia-Cardenas V, Dineen-Griffin S, Gastelurrutia MA, et al. A cost utility analysis alongside a cluster-randomised trial evaluating a minor ailment service compared to usual care in community pharmacy. BMC Health Services Research. 2021;21(1):1253. PubMed PMID: 34798895.

2620. Amador-Fernández N, Benrimoj SI, Olry de Labry Lima A, García-Cárdenas V, Gastelurrutia M, Berger J, et al. Strengthening patients' triage in community pharmacies: a cluster randomised controlled trial to evaluate the clinical impact of a minor ailment service. PloS one. 2022;17(10):e0275252. doi: 10.1371/journal.pone.0275252. PubMed PMID: CN-02486647.

2621. Amador-Fernández N, Botnaru I, Allemann SS, Kälin V, Berger J. Clinical relevance and implementation into daily practice of pharmacist-prescribed medication for the management of minor ailments. Frontiers in Pharmacology. 2023;14. doi: 10.3389/fphar.2023.1256172.

2622. Amador-Fernández N, Escaith M, Simi E, Quintana-Bárcena P, Berger J. Evaluation of an enhanced service for medication review with follow up in Swiss community pharmacies: Pre-post study protocol. PLoS

ONE. 2023;18(10 October). doi: 10.1371/journal.pone.0292037.

2623. Amador-Fernandez N, Garcia-Cardenas V, Baixauli-Fernandez VJ, Colomer-Molina V, Benrimoj SI, Martinez-Martinez F. Pilot study of a minor ailment service in Community Pharmacy in the Province of Valencia; INDICA + PRO. International journal of clinical pharmacy. 2018;40(2):503-. doi: 10.1007/s11096-018-0609-9. PubMed PMID: CN-01605165.

2624. Amador-Fernandez N, Garcia-Cardenas V, Garcia-Agudo O, Baixauli-fernandez VJ, Colomer-Molina V, Climent-catala MT, et al. INDICA+PRO: study of a minor ailment service in community pharmacy in a Spanish province. Preliminary data, referral rates. Pharmacy practice. 2018;16. doi: 10.18549/PharmPract.2018.s1.1340. PubMed PMID: CN-02138128.

2625. Amador-Fernandez N, Gastelurrutia MA, Garcia-Cardenas V. Development of self-care in Spanish community pharmacies. Exploratory Research in Clinical and Social Pharmacy. 2023;12:100337. doi: https://dx.doi.org/10.1016/j.rcsop.2023.100337. PubMed PMID: 2027357444.

2626. Amador-Fernández N, Jenkinson SP, Berger J. Vision and practice of self-care for community pharmacy in Switzerland. Exploratory Research in Clinical and Social Pharmacy. 2023;9:100253. doi: https://doi.org/10.1016/j.rcsop.2023.100253.

2627. Amador-Fernández N, Matthey-de-l'Endroit J, Berger J. Factors Influencing the Implementation of a New Pharmacist Prescribing Service in Community Pharmacies. Pharmacy. 2023;11(6). doi: 10.3390/pharmacy11060173. PubMed PMID: WOS:001131287700001.

2628. Amaha ND, Weldemariam DG, Abdu N, Tesfamariam EH. Prescribing practices using WHO prescribing indicators and factors associated with antibiotic prescribing in six community pharmacies in Asmara, Eritrea: A cross-sectional study. Antimicrobial Resistance and Infection Control. 2019;8(1). doi: 10.1186/s13756-019-0620-5.

2629. Amalberti R, Auroy Y, Berwick D, Barach P. Five system barriers to achieving ultrasafe health care. Annals of internal medicine. 2005;142(9):756-64.

2630. Aman A, Hasen G, Usman H, Suleman S. Regulatory Compliance and Associated Quality of Amoxicillin in Drug Retail Outlets of Southwestern Ethiopia. Drug Healthcare and Patient Safety. 2021;13:241-9. doi: 10.2147/dhps.S337791. PubMed PMID: WOS:000735669800001.

2631. Amani A, Amini MA, Ali HSM, York P. Alternatives to conventional suspensions for pulmonary drug delivery by nebulisers: A review. Journal of Pharmaceutical Sciences. 2011;100(11):4563-70. doi: https://doi.org/10.1002/jps.22665.

2632. Amankwa CE, Bonful HA, Agyabeng K, Nortey PA. Dispensing practices for anti-malarials in the La Nkwantanang-Madina municipality, Greater Accra, Ghana: a cross-sectional study. Malaria journal. 2019;18(1):260. doi: 10.1186/s12936-019-2897-5. PubMed PMID: CN-01980036.

2633. Amann S, Kantelhardt P. Bericht aus der Praxis: Medikationsfehlererfassung und Medication Reconciliation aus Sicht der Krankenhausapotheker. Zeitschrift für Evidenz, Fortbildung und Qualität im Gesundheitswesen. 2012;106(10):717-22. doi: https://doi.org/10.1016/j.zefq.2012.11.015.

2634. Amany S-k, אמאני ס-חא. Examining the Relation Between Knowledge and Attitudes Towards Medical Cannabis Among Israeli Community Pharmacists at Haifa and the Krayot [Master's]. Israel: University of Haifa (Israel); 2020.

2635. Amara P. New protocol for treating paracetamol overdose: The Scottish and Newcastle Acetylcysteine

Protocol reduces risk of anaphylactoid reactions and drug errors. Emergency Nurse. 2023;31(6):10-1. doi: 10.7748/en.31.6.10.s4. PubMed PMID: 174978115.

2636. Amaral PMR. Estudo da Eficácia do Ácido Tranexâmico Como Agente Hemostático em Doentes Submetidos a Artroplastia Total Primária de Joelho e Anca: Experiência Profissionalizante na Vertente de Farmácia Comunitária, Farmácia Hospitalar e Investigação [M.Sc.]. Portugal: Universidade da Beira Interior (Portugal); 2020.

2637. Amaral S, Foster N, Dashefsky H, Kim J, Furth S. Peers4path: challenges in measuring medication adherence in adolescent transplant recipients. Transplantation. 2014;98:238. doi: 10.1097/01.tp.0000452129.50834.32. PubMed PMID: CN-01065948.

2638. Amaral S, Rogers R, Paterno C, Foster N, Shults J. Peers4path (promoting adolescent transplant health): a pilot study to test peer mentoring as an adherence intervention in adolescent transplant recipients. Pediatric transplantation. 2017;21:33-. doi: 10.1111/petr.12966. PubMed PMID: CN-01657920.

2639. Amaratunge S, Harrison M, Clifford R, Seubert L, Page A, Bond C. Developing a checklist for reporting research using simulated patient methodology(CRISP): a consensus study. International Journal of Pharmacy Practice. 2021;29(3):218-27. doi: 10.1093/ijpp/riaa002. PubMed PMID: WOS:000661386900004.

2640. Amaratunge S, Harrison M, Perry D, Bond C, Ceulemans M, Foulon V, et al. Assessing the reporting quality of simulated patient studies in pharmacy research using a novel checklist (CRiSP). Research in Social and Administrative Pharmacy. 2022;18(2):2301-7. doi: 10.1016/j.sapharm.2021.04.007.

2641. Amariles P, Baena MI, Faus MJ, Machuca M, Tudela J, Barris D, et al. Knowledge and cardiovascular disease risk among patients are taking cardiovascular related medications. Ars Pharmaceutica. 2005;46(3):279-300.

2642. Amariles P, Ceballos M, González-Giraldo C. Primary health care policy and vision for community pharmacy and pharmacists in colombia. Pharmacy Practice. 2020;18(4):1-7. doi: 10.18549/PharmPract.2020.4.2159.

2643. Amariles P, Faus MJ, Machuca M, González-Zapata L, Baena MI, Quesada F. Evaluation of a knowledge questionnaire about cardiovascular disease in Spanish community pharmacies. Atencion Farmaceutica. 2009;11(2):101-9.

2644. Amariles P, Ledezma-Morales M, Salazar-Ospina A, Hincapié-García JA. Pharmacist's Role and Pharmaceutical Care During the COVID-19 Pandemic. Advances in Experimental Medicine and Biology. 13182021. p. 605-22.

2645. Amariles P, Ledezma-Morales M, Salazar-Ospina A, Hincapie-Garcia JA. How to link patients with suspicious COVID-19 to health system from the community pharmacies? A route proposal. Research in Social & Administrative Pharmacy. 2021;17(1):1988-9. doi: 10.1016/j.sapharm.2020.03.007. PubMed PMID: WOS:000615350400032.

2646. Amariles P, MacHuca M, Faus MJ, Baena MI, Martinez-Martinez F, Jimenez-Martin J. Application of the SCORE and Wilson-Grundy methods for the assessment of cardiovascular risk in community pharmacies. Journal of Clinical Pharmacy and Therapeutics. 2008;33(5):475-81. doi: 10.1111/j.1365-2710.2008.00953.x.

2647. Amariles P, Monsalve M, Rivera-Cadavid M, Ceballos M, Ledezma-Morales M, Salazar-Ospina A. Processes implemented and activities carried out by the pharmacists during the COVID-19 pandemic: A scoping review. Farmacia Hospitalaria. 2022;46(6):350-8. doi: 10.7399/fh.13146. PubMed PMID: WOS:000918618200007.

2648. Amariles P, Monsalve M, Rivera-Cadavid M, Ceballos M, Ledezma-Morales M, Salazar-Ospina A. Processes implemented and activities carried out by the pharmacists during the COVID-19 pandemic: A scoping review. Farmacia Hospitalaria. 2022;46(6):350-8. doi: 10.7399/fh.13146.

2649. Amariles P, Pino-Marín D, Sabater-Hernández D, García-Jiménez E, Roig-Sánchez I, Faus MJ. Fiabilidad y validez externa de un cuestionario de conocimiento sobre riesgo y enfermedad cardiovascular en pacientes que acuden a farmacias comunitarias de España. Atención Primaria. 2016;48(9):586-95. doi: https://doi.org/10.1016/j.aprim.2016.01.005.

2650. Amariles P, Pino-Marín D, Sabater-Hernández D, García-Jiménez E, Roig-Sánchez I, Faus MJ. Reliability and external validity of a questionnaire to assess the knowledge about risk and cardiovascular disease and in patients attending Spanish community pharmacies. Atencion Primaria. 2016;48(9):586-95. doi: 10.1016/j.aprim.2016.01.005.

2651. Amariles P, Sabater-Hernández D, García-Jiménez E, Rodríguez-Chamorro M, Prats-Más R, Marín-Magán F, et al. Effectiveness of Dader Method for pharmaceutical care on control of blood pressure and total cholesterol in outpatients with cardiovascular disease or cardiovascular risk: EMDADER-CV randomized controlled trial. Journal of managed care pharmacy. 2012;18(4):311-23. doi: 10.18553/jmcp.2012.18.4.311. PubMed PMID: CN-00837181.

2652. Amariles P, Sabater-Hernandez D, Garcia-Jimenez E, Rodriguez-Chamorro MA, Prats-Mas R, Marin-Magan F, et al. Effectiveness of Dader Method for Pharmaceutical Care on Control of Blood Pressure and Total Cholesterol in Outpatients with Cardiovascular Disease or Cardiovascular Risk: EMDADER-CV Randomized Controlled Trial. Journal of Managed Care Pharmacy. 2012;18(4):311-23. doi: 10.18553/jmcp.2012.18.4.311. PubMed PMID: WOS:000304131700002.

2653. Amariles P, Sabater-Hernández D, García-Jiménez E, Rodríguez-Chamorro MÁ, Prats-Más R, Marín-Magán F, et al. Effectiveness of dader method for pharmaceutical care on control of blood pressure and total cholesterol in outpatients with cardiovascular disease or cardiovascular risk: EMDADER-CV randomized controlled trial. Journal of Managed Care Pharmacy. 2012;18(4):311-23. doi: 10.18553/jmcp.2012.18.4.311.

2654. Amarshi R. Establishing Standards of Care for Amiodarone Monitoring in an Outpatient Setting. 2017.

2655. Amat M, Fernandez L, Anderson T. IMPROVING POST-DISCHARGE CARE FOR PATIENTS WITH LIMITED ENGLISH PROFICIENCY: A MULTIDISCIPLINARY STUDY TO STRENGTHEN THE CONNECTION BETWEEN PATIENTS AND THEIR PRIMARY CARE TEAM AFTER HOSPITALIZATION. Journal of General Internal Medicine. 2023;38(Supplement 2):S666-S7. doi: https://dx.doi.org/10.1007/s11606-023-08226-z. PubMed PMID: 641880593.

2656. Amato MG, Salazar A, Hickman T-TT, Quist AJ, Volk LA, Wright A, et al. Computerized prescriber order entry–related patient safety reports: analysis of 2522 medication errors. Journal of the American Medical Informatics Association. 2017;24(2):316-22.

2657. Amato MG, Salazar A, Hickman T-TT, Quist AJL, Volk LA, Wright A, et al. Computerized prescriber order entry-related patient safety reports: analysis of 2522 medication errors. Journal of the American Medical Informatics Association. 2017;24(2):316-22. doi: 10.1093/jamia/ocw125. PubMed PMID: 121346953. Language: English. Entry Date: 20180723. Revision Date: 20201203. Publication Type: journal article.

2658. Ambery P, Parker VE, Stumvoll M, Posch MG, Heise T, Plum-Moerschel L, et al. MEDI0382, a GLP-1 and glucagon receptor dual agonist, in obese or overweight patients with type 2 diabetes: a randomised, controlled, double-blind, ascending dose and phase 2a study. The Lancet. 2018;391(10140):2607-18. doi:

https://doi.org/10.1016/S0140-6736(18)30726-8.

2659. Ambery P, Parker VE, Stumvoll M, Posch MG, Heise T, Plum-Moerschel L, et al. MEDI0382, a GLP-1 and glucagon receptor dual agonist, in obese or overweight patients with type 2 diabetes: a randomised, controlled, double-blind, ascending dose and phase 2a study. Lancet (london, england). 2018;391(10140):2607-18. doi: 10.1016/S0140-6736(18)30726-8. PubMed PMID: CN-01664895.

2660. Ambiga N, Nagarajan A. Possibilities of using nano particles in human urine for transient biometrics. Materials Today: Proceedings. 2021. doi: https://doi.org/10.1016/j.matpr.2021.07.034.

2661. Ambiga N, Nagarajan A. Possibilities of using nano particles in human urine for transient biometrics. Materials Today: Proceedings. 2023;80:2765-8. doi: https://doi.org/10.1016/j.matpr.2021.07.034.

2662. Ambler S. General practitioners and community pharmacists: Times they are a-changing. British Journal of General Practice. 2003;53(493):594-5.

2663. Ambrose CS, Chen X, Kumar VR. A population-weighted, condition-adjusted estimate of palivizumab efficacy in preventing RSV-related hospitalizations among US high-risk children. Human vaccines & immunotherapeutics. 2014;10(10):2785-8. doi: 10.4161/hv.32082. PubMed PMID: CN-01111803.

2664. Ambrose PG, Lomovskaya O, Griffith DC, Dudley MN, VanScoy B. β-Lactamase inhibitors: what you really need to know. Current Opinion in Pharmacology. 2017;36:86-93. doi: https://doi.org/10.1016/j.coph.2017.09.001.

2665. Ambrose PJ. Drug use in sports: a veritable arena for pharmacists. Journal of the American Pharmacists Association. 2004;44(4):501-16.

2666. Ambrose PJ, Saya FG, Lovett LT, Tan S, Adams DW, Shane R. Evaluating the accuracy of technicians and pharmacists in checking unit dose medication cassettes. American journal of health-system pharmacy. 2002;59(12):1183-8.

2667. Ambrose PJ, Tsourounis C, Uryasz FD, Patterson E. Characteristics and trends of drug and dietary supplement inquiries by college athletes. Journal of the American Pharmacists Association. 2013;53(3):297-303. doi: https://doi.org/10.1331/JAPhA.2013.12154.

2668. Ambrose T, De Silva A, Naghibi M, Saunders J, Smith TR, Coleman RL, et al. Refeeding risks in patients requiring intravenous nutrition support: results of a two-centre, prospective, double-blind, randomised controlled trial. Clinical nutrition ESPEN. 2021;41:143-52. doi: 10.1016/j.clnesp.2020.11.028. PubMed PMID: CN-02231512.

2669. Ambrosetti L, Jordan Von Gunten V, Beney J, Bridevaux PO. Overview of the inpatient management of acute exacerbations of COPD - A case-series. Respiration; international review of thoracic diseases. 2017;94(1):92-. doi: 10.1159/000474251. PubMed PMID: CN-01647776.

2670. Ambroziak K, Ibrahim N, Marshall VD, Kelling SE. Virtual simulation to personalize student learning in a required pharmacy course. Currents in Pharmacy Teaching and Learning. 2018;10(6):750-6. doi: 10.1016/j.cptl.2018.03.017. PubMed PMID: WOS:000438935600013.

2671. Ameixa AMS. Classificação ABC e XYZ do Grupo de Fármacos Anti-Infeciosos e do Sistema Cardiovascular do CHUCB: Análise Individual e Comparativa das Duas Classes: Experiência Profissionalizante na Vertente de Farmácia Comunitária e Investigação [M.Sc.]. Portugal: Universidade da Beira Interior (Portugal); 2019.

2672. Amekyeh H, Mensah A, Saaka Y, Orman E. Comparative assessment of the physicochemical properties of antacid suspensions available in the Ho Municipality of the Volta Region of Ghana. Scientific African. 2021;13. doi: 10.1016/j.sciaf.2021.e00955.

2673. Amer M, Rahman N, Nazir SR, Raza A, Riaz H, Sultana M, et al. Impact of pharmacist's intervention on disease related knowledge, medication adherence, HRQoL and control of blood pressure among hypertensive patients. Pakistan journal of pharmaceutical sciences. 2018;31(6 (Supplementary):2607-16. PubMed PMID: CN-01751422.

2674. Ameri A, Khajouei R, Ameri A, Jahani Y. LabSafety, the Pharmaceutical Laboratory Android Application, for Improving the Knowledge of Pharmacy Students. Biochemistry and molecular biology education. 2020;48(1):44-53. doi: 10.1002/bmb.21311. PubMed PMID: CN-02005354.

2675. American College of Clinical P, Hume AL, Kirwin J, Bieber HL, Couchenour RL, Hall DL, et al. Improving care transitions: current practice and future opportunities for pharmacists. Pharmacotherapy:The Journal of Human Pharmacology & Drug Therapy. 2012;32(11):e326-37. PubMed PMID: 23108810.

2676. American College of Clinical P, Kirwin J, Canales AE, Bentley ML, Bungay K, Chan T, et al. Process indicators of quality clinical pharmacy services during transitions of care. Pharmacotherapy:The Journal of Human Pharmacology & Drug Therapy. 2012;32(11):e338-47. PubMed PMID: 23108762.

2677. American Hospital A, American Society of Health-System P, Hospitals, Health N. Medication Safety Issue Brief. Small and rural hospitals--unique challenges, unique solutions. Hospitals & Health Networks. 2005;79(11):45-6. PubMed PMID: 16396133.

2678. American Pharmacists A. Pharmacists' role in addressing opioid abuse, addiction, and diversion. Journal of the American Pharmacists Association. 2014;54(1):e5-e15. doi: https://doi.org/10.1331/JAPhA.2014.13101.

2679. American Pharmacists A, American Society of Health-System P. Improving care transitions: Optimizing medication reconciliation. Journal of the American Pharmacists Association. 2012;52(4):e43-e52. doi: https://doi.org/10.1331/JAPhA.2012.12527.

2680. American Pharmacists A, American Society of Health-System P, Steeb D, Webster L. Improving care transitions: optimizing medication reconciliation. Journal of the American Pharmacists Association: JAPhA. 2012;52(4):e43-52. PubMed PMID: 22825240.

2681. American Pharmacists A, National Association of Chain Drug Stores F. Medication therapy management in pharmacy practice: core elements of an MTM service model (version 2.0). Journal of the American Pharmacists Association: JAPhA. 2008;48(3):341-53. PubMed PMID: 18595820.

2682. American Society of Health-System P. ASHP statement on bar-code verification during inventory, preparation, and dispensing of medications. American Journal of Health-System Pharmacy. 2011;68(5):442-5. PubMed PMID: 21330687.

2683. Américo P, Rocha R. Subsidizing access to prescription drugs and health outcomes: The case of diabetes. Journal of Health Economics. 2020;72:102347. doi: https://doi.org/10.1016/j.jhealeco.2020.102347.

2684. Amerine JP, Khan T, Crisp B. Improvement of patient wait times in an outpatient pharmacy. American Journal of Health-System Pharmacy. 2017;74(13):958-61. doi: 10.2146/ajhp160843.

2685. Amerongen HM, LeGros TA, Cooley JH, Schloss EP, Theodorou A. Constructive contact: Design of a successful introductory interprofessional education experience. Currents in Pharmacy Teaching and Learning.

2015;7(5):565-74. doi: https://doi.org/10.1016/j.cptl.2015.06.013.

2686. Amesty S, Blaney S, Crawford ND, Rivera AV, Fuller C. Pharmacy staff characteristics associated with support for pharmacy-based HIV testing. Journal of the American Pharmacists Association : JAPhA. 2012;52(4):472-9, 1-9. doi: 10.1331/JAPhA.2012.10194. PubMed PMID: CN-00859670.

2687. Amesty S, Crawford ND, Nandi V, Perez-Figueroa R, Rivera A, Sutton M, et al. Evaluation of pharmacy-based HIV testing in a high-risk New York city community. AIDS Patient Care and STDs. 2015;29(8):437-44. doi: 10.1089/apc.2015.0017.

2688. Amico KR, Marcus JL, McMahan V, Liu A, Koester KA, Goicochea P, et al. Study product adherence measurement in the iPrEx placebo-controlled trial: concordance with drug detection. Journal of acquired immune deficiency syndromes (1999). 2014;66(5):530-7. doi: 10.1097/QAI.0000000000000216. PubMed PMID: CN-01053772.

2689. Amien F, Myburgh NG, Butler N. Location of community pharmacies and prevalence of oral conditions in the Western Cape Province. Health SA Gesondheid. 2013;18(1). doi: 10.4102/hsag.v18i1.687.

2690. Amin A, Boulanger L, Gatt E, Makenbaeva D, Stokes M, Wiederkehr D, et al. Exposure to warfarin and the risks of stroke and bleeding events among patients with non-valvular atrial fibrillation: real-world vs. Clinical trial. Circulation: cardiovascular quality and outcomes. 2012;5(3 SUPPL. 1). PubMed PMID: CN-01023993.

2691. Amin A, Bruno A, Trocio J, Lin J, Lingohr-Smith M. Incremental Health Care Burden of Bleeding Among Patients with Venous Thromboembolism in the United States. Journal of Managed Care & Specialty Pharmacy. 2015;21(10):965-72. doi: 10.18553/jmcp.2015.21.10.965. PubMed PMID: WOS:000369189800010.

2692. Amin A, Lin J, Lenhart G, Schulman K. Economic Outcomes in Patients at Risk for Venous Thromboembolism Receiving Appropriate Enoxaparin or Unfractionated Heparin Prophylaxis. Blood. 2008;112(11):1285. doi: https://doi.org/10.1182/blood.V112.11.1285.1285.

2693. Amin A, Stokes M, Wu N, Gatt E, Makenbaeva D, Wiederkehr D, et al. Application of randomized clinical trial data to actual practice: apixaban therapy for reduction of stroke risk in non-valvular atrial fibrillation patients. Current medical research and opinion. 2013;29(10):1253-61. doi: 10.1185/03007995.2013.818967. PubMed PMID: CN-00916222.

2694. Amin A, Stokes M, Wu N, Gatt E, Makenbaeva D, Wiederkehr D, et al. Applying aristotle trial to actual practice. Stroke. 2013;44(2). PubMed PMID: CN-01007036.

2695. Amin AA, Snow RW. Brands, costs and registration status of antimalarial drugs in the Kenyan retail sector. Malaria Journal. 2005;4. doi: 10.1186/1475-2875-4-36.

2696. Amin AA, Snow RW, Kokwaro GO. The quality of sulphadoxine-pyrimethamine and amodiaquine products in the Kenyan retail sector. Journal of Clinical Pharmacy and Therapeutics. 2005;30(6):559-65. doi: 10.1111/j.1365-2710.2005.00685.x.

2697. Amin FA, Harrison S, Heywood P. Testing interventions for improving adherence to diabetic guideline: a randomised controlled study in Bahrain. Journal of the bahrain medical society. 2004;16(4):156-65. PubMed PMID: CN-01771265.

2698. Amin K, Bethel G, Jackson LR, II, Essien UR, Sloan CE. Eliminating Health Disparities in Atrial Fibrillation, Heart Failure, and Dyslipidemia: A Path Toward Achieving Pharmacoequity. Current

Atherosclerosis Reports. 2023;25(12):1113-27. doi: 10.1007/s11883-023-01180-5. PubMed PMID: WOS:001126162400001.

2699. Amin K, Dannenfelser R-M. In vitro hemolysis: Guidance for the pharmaceutical scientist. Journal of Pharmaceutical Sciences. 2006;95(6):1173-6. doi: https://doi.org/10.1002/jps.20627.

2700. Amin ME. Pharmacists' Role in Adjusting Medication Regimens for Muslim Patients in Ramadan [Ph.D.]. United States -- Wisconsin: The University of Wisconsin - Madison; 2013.

2701. Amin ME, Chewning B. Community pharmacists' knowledge of diabetes management during Ramadan in Egypt. International journal of clinical pharmacy. 2014;36(6):1213-21. doi: 10.1007/s11096-014-0019-6. PubMed PMID: CN-01113541.

2702. Amin MEK. Pharmacists' knowledge and interest in developing counseling skills relating to oral contraceptives. International Journal of Clinical Pharmacy. 2016;38(2):395-403. doi: 10.1007/s11096-016-0264-y.

2703. Amin MEK, Amine A, Newegy MS. Perspectives of pharmacy staff on dispensing subtherapeutic doses of antibiotics: a theory informed qualitative study. International Journal of Clinical Pharmacy. 2017;39(5):1110-8. doi: 10.1007/s11096-017-0510-y.

2704. Amin MEK, Chewning B. Community pharmacists' knowledge of diabetes management during Ramadan in Egypt. International Journal of Clinical Pharmacy. 2014;36(6):1213-21. doi: 10.1007/s11096-014-0019-6.

2705. Amin MEK, Chewning B. Predicting pharmacists' adjustment of medication regimens in Ramadan using the Theory of Planned Behavior. Research in Social and Administrative Pharmacy. 2015;11(1):e1-e15. doi: 10.1016/j.sapharm.2014.04.004.

2706. Amin MEK, Chewning B. Pharmacist–patient communication about medication regimen adjustment during Ramadan. International Journal of Pharmacy Practice. 2016;24(6):419-27. doi: 10.1111/ijpp.12282.

2707. Amin MEK, Chewning B. Pharmacists' counseling on oral contraceptives: A theory informed analysis. Research in Social and Administrative Pharmacy. 2016;12(5):669-81. doi: 10.1016/j.sapharm.2015.08.009.

2708. Amin MEK, Chewning B. Pharmacist-patient communication about medication regimen adjustment during Ramadan. International Journal of Pharmacy Practice. 2016;24(6):419-27. doi: 10.1111/ijpp.12282. PubMed PMID: WOS:000387807400006.

2709. Amin MEK, Qudah BA, Kaur A, Rawy M, Chewning B. Construction and evaluation of an online module addressing counseling patients with diabetes observing religious fasting. Currents in Pharmacy Teaching and Learning. 2021;13(12):1602-10. doi: 10.1016/j.cptl.2021.09.022. PubMed PMID: WOS:000731102100009.

2710. Amin MR, Gentile JP, Edwards B, Davis M. Evaluation of Health Care Disparities for Individuals with Intellectual and Developmental Disabilities in Ohio. Community Mental Health Journal. 2021;57(3):482-9. doi: 10.1007/s10597-020-00669-6. PubMed PMID: WOS:000543585400001.

2711. Amin R. RPS community support hub will support your day-to-day practice. Pharmaceutical Journal. 2014;292(7806-7807):435-6.

2712. Amin R. Everything you need in one place: The RPS community pharmacy online hub. Pharmaceutical Journal. 2014;292(7797):S20.

2713. Amin S, McKeirnan KC. The physician perspective on pharmacist-physician collaboration and trust. Journal of the American Pharmacists Association. 2022;62(4):1304-12. doi: https://doi.org/10.1016/j.japh.2022.02.014.

2714. Amin-Esmaeili M, Rahimi-Movaghar A, Sharifi V, Hajebi A, Radgoodarzi R, Mojtabai R, et al. Epidemiology of illicit drug use disorders in Iran: prevalence, correlates, comorbidity and service utilization results from the Iranian Mental Health Survey. Addiction. 2016;111(10):1836-47. doi: 10.1111/add.13453. PubMed PMID: WOS:000383713500028.

2715. Amini S, Gholami K, Bakhshandeh H, Farsad BF. Effect of oral anticoagulant therapy on coagulation activity and inflammatory markers in patients with atrial fibrillation undergoing ablation: a randomized comparison between dabigatran and warfarin. Iranian journal of pharmaceutical research. 2013;12(4):945-53. PubMed PMID: CN-00911016.

2716. Amiraj-Surujparsad S. The Effect of a Homeoepathic Remedy, Passiflora incarnata 0 on Insomnia [M.Tech.]. South Africa: University of Johannesburg (South Africa); 2008.

2717. Amirfar S, Anane S, Buck M, Cohen R, Di Lonardo S, Maa P, et al. Study of electronic prescribing rates and barriers identified among providers using electronic health records in New York City. Informatics in Primary Care. 2011;19(2):91-7. PubMed PMID: 104528831. Language: English. Entry Date: 20120309. Revision Date: 20150820. Publication Type: Journal Article.

2718. Amiri Jabalbarez F, Dabaghzadeh F, Oghabian Z. Role of pharmacists in reducing antibiotic prescribing errors in an emergency department. Journal of Pharmacy Practice & Research. 2020;50(1):42-7. doi: 10.1002/jppr.1586. PubMed PMID: 141780252. Language: English. Entry Date: 20200225. Revision Date: 20210201. Publication Type: Article.

2719. Amiri S, Monsivais P, McDonell MG, Amram O. Availability of licensed cannabis businesses in relation to area deprivation in Washington state: A spatiotemporal analysis of cannabis business presence between 2014 and 2017. Drug and Alcohol Review. 2019;38(7):790-7. doi: 10.1111/dar.12987. PubMed PMID: WOS:000492068800001.

2720. Amirian I, Huston S, Ha D, Garza K, Hohmann L, Hastings T, et al. Results of immunization delivery enhancement intervention on pneumococcal and herpes zoster immunization planning in alabama and california community pharmacies. Journal of the american pharmacists association. 2017;57(3). doi: 10.1016/j.japh.2017.04.011. PubMed PMID: CN-01608601.

2721. Amirsadri A, Chapman T, Breen M, Drake W, Arfken CL. Experience with mandated use of generic medications for patients covered by the mental health safety net. Psychiatric Services. 2014;65(7):850-2. doi: 10.1176/appi.ps.201400075).

2722. Amirsadri M, Torkpour E. Cost-effectiveness and cost-utility analysis of type-2 diabetes screening in pharmacies in Iran. Research in Pharmaceutical Sciences. 2023;18(2):210-8. doi: 10.4103/1735-5362.367799.

2723. Amirsadri M, Torkpour E. Cost-effectiveness and cost-utility analysis of type-2 diabetes screening in pharmacies in Iran. Research in Pharmaceutical Sciences. 2023;18(2):210-8. doi: 10.4103/1735-5362.367799.

2724. Amirthalingam ARS. An evaluation of a community pharmacy based, pharmacist-led intervention package targeted to the patients' adherence status, to achieve and maintain target blood pressure (BP) control by optimising antihypertensive medicine adherence [D.Pharm.]. England: University of Birmingham (United Kingdom); 2017.

2725. Amirthalingam S, Verma RK, Appalasamy JR, Barura A, Yi KS. Pharmacists' knowledge and perception of topical antibacterial drug dispensing in community pharmacy setting in Kedah, Malaysia. Tropical Journal of Pharmaceutical Research. 2016;15(4):855-60. doi: 10.4314/tjpr.v15i4.27.

2726. Amit, Kumari S, Jamwal R. Use of FTIR spectroscopy integrated with multivariate chemometrics as a swift, and non-destructive technique to detect various adulterants in virgin coconut oil: A comprehensive review. Food Chemistry Advances. 2023;2:100203. doi: https://doi.org/10.1016/j.focha.2023.100203.

2727. Amitai Y, Degani Y. Treatment of phenobarbital poisoning with multiple dose activated charcoal in an infant. The Journal of Emergency Medicine. 1990;8(4):449-50. doi: https://doi.org/10.1016/0736-4679(90)90174-T.

2728. Amkreutz J, Lenssen R, Marx G, Deisz R, Eisert A. Medication safety in a German telemedicine centre: Implementation of a telepharmaceutical expert consultation in addition to existing tele-intensive care unit services. Journal of Telemedicine & Telecare. 2020;26(1-2):105-12. PubMed PMID: 30253681.

2729. Ammar R, Song JC, Kluger J, White CM. Evaluation of electrocardiographic and hemodynamic effects of caffeine with acute dosing in healthy volunteers. Pharmacotherapy. 2001;21(4):437-42. doi: 10.1592/phco.21.5.437.34502. PubMed PMID: CN-00347307.

2730. Ammari WG, Chrystyn H. Optimizing the inhalation flow and technique through metered dose inhalers of asthmatic adults and children attending a community pharmacy. Journal of asthma. 2013;50(5):505-13. doi: 10.3109/02770903.2013.783064. PubMed PMID: CN-00862184.

2731. Ammenwerth E, Duftschmid G, Gall W, Hackl WO, Hoerbst A, Janzek-Hawlat S, et al. A nationwide computerized patient medication history: Evaluation of the Austrian pilot project "e-Medikation". International Journal of Medical Informatics. 2014;83(9):655-69. doi: https://doi.org/10.1016/j.ijmedinf.2014.06.004.

2732. Ammerman CA, Simpkins BA, Warman N, Downs TN. Potentially Inappropriate Medications in Older Adults: Deprescribing with a Clinical Pharmacist. Journal of the American Geriatrics Society. 2019;67(1):115-8. doi: 10.1111/jgs.15623. PubMed PMID: 133988352. Language: English. Entry Date: 20190110. Revision Date: 20200424. Publication Type: Article.

2733. Ammeter T, Lang P, Czock A. Overview of the influenza vaccination activities and legal frameworks in 26 Swiss cantons during the influenza season 2019/20. Vaccine. 2022;40(12):1702-6. doi: https://doi.org/10.1016/j.vaccine.2022.02.009.

2734. Amoabeng IA, Otoo BA, Darko G, Borquaye LS. Disposal of Unused and Expired Medicines within the Sunyani Municipality of Ghana: A Cross-Sectional Survey. Journal of Environmental and Public Health. 2022;2022. doi: 10.1155/2022/6113346.

2735. Amodeo SJ, Kowalkowski HF, Brantley HL, Jones NW, Bangerter LR, Cook DJ. Temporal Patterns of High-Spend Subgroups Can Inform Service Strategy for Medicare Advantage Enrollees. Journal of General Internal Medicine. 2022;37(8):1853-61. doi: 10.1007/s11606-021-06912-4. PubMed PMID: WOS:000658592400003.

2736. Amorha KC, Idoko KE, Okonta MJ, Ukwe CV. Knowledge and practice of the global initiative for asthma report among community pharmacists in a Nigerian state. Pan African Medical Journal. 2020;37(83):1-11. doi: 10.11604/pamj.2020.37.83.18897.

2737. Amorha KC, Okonta MJ, Ukwe CV. Impact of pharmacist-led educational interventions on asthma control and adherence: single-blind, randomised clinical trial. International journal of clinical pharmacy.

2021;43(3):689-97. doi: 10.1007/s11096-020-01187-x. PubMed PMID: CN-02204726.

2738. Amorha KC, Okonta MJ, Ukwe CV. Impact of pharmacist-led interventions on asthma patients' knowledge, attitudes and self-efficacy: single-blind randomised controlled trial. Journal of pharmaceutical health services research. 2021;12(1):90-3. doi: 10.1093/jphsr/rmaa020. PubMed PMID: CN-02503431.

2739. Amorha KC, Ugwu CV, Ubaka CM, Okonta MJ, Ukwe CV. Community pharmacists' competence for educating asthmatic patients on the use of inhalers and the peak flow meter. African Journal of Biomedical Research. 2020;23(2):221-6.

2740. Amori RE, Pittas AG, Siegel RD, Kumar S, Chen JS, Karnam S, et al. Inpatient medical errors involving glucose-lowering medications and their impact on patients: review of 2,598 incidents from a voluntary electronic error-reporting database. Endocrine Practice. 2008;14(5):535-42.

2741. Amorim AA. O Processo de Desprescrição em Idosos Polimedicados Revisitado: Experiência Profissionalizante na Vertente de Farmácia Comunitária e Investigação [M.Sc.]. Portugal: Universidade da Beira Interior (Portugal); 2018.

2742. Amoroso TCN. Análise, Caracterização e Estudo da Toxicidade de um Hidrogel Depilatório Com Tioglicolato de Cálcio: Experiência Profissionalizante na Vertente de Farmácia Comunitária e Investigaçãol [Master's]. Portugal: Universidade da Beira Interior (Portugal); 2012.

2743. Ampe E, Spinewine A, Tulkens PM. Clinical Pharmacy, a useful evolution in pharmacists' activities for a better care of patients in Belgian hospitals. Tijdschrift voor Geneeskunde. 2006;62(18):1273-84. doi: 10.2143/TVG.62.18.2021584.

2744. Ampe E, Spinewine A, Wilmotte L, Hecq JD, Tulkens PM. Clinical pharmacy: A recent development of pharmacists' activity for an optimized management of patients from the medicinal point of view. Louvain Medical. 2006;125(8):275-90.

2745. Amponsah AO, Vosper H, Marfo AFA. Patient related factors affecting adherence to antimalarial medication in an urban estate in ghana. Malaria Research and Treatment. 2015;2015. doi: 10.1155/2015/452539.

2746. Amruso NA. Ability of Clinical Pharmacists in a Community Pharmacy Setting to Manage Anticoagulation Therapy. Journal of the American Pharmacists Association. 2004;44(4):467-71. doi: https://doi.org/10.1331/1544345041475751.

2747. Amsallem A, Berthou-Contreras J, Joret N, Koeberlé S, Limat S, Clairet AL. Utilisations des statines chez le sujet âgé selon le type de prise en charge : un exemple de l'intérêt des groupements hospitaliers de territoire. Annales Pharmaceutiques Françaises. 2023;81(2):346-53. doi: https://doi.org/10.1016/j.pharma.2022.06.004.

2748. Amstislavski P. Access to urban food outlets as a predictor of diabetes [Ph.D.]. United States -- New York: City University of New York; 2012.

2749. Amstislavski P, Matthews A, Sheffield S, Maroko AR, Weedon J. Medication deserts: survey of neighborhood disparities in availability of prescription medications. International journal of health geographics. 2012;11(1):1-13.

2750. Amuasi JH. The Affordable Medicines Facility-malaria in Ghana: Factors Accounting for Antimalarial Availability and Pricing Outcomes [Ph.D.]. United States -- Minnesota: University of Minnesota; 2014.

2751. Amundson C, Marupuru S, Kliethermes MA, Axon DR, Warholak TL, Turgeon J, et al. Comprehensive medication management services provided via telehealth or hybrid models: A scoping review. JACCP Journal of the American College of Clinical Pharmacy. 2023;6(1):53-72. doi: https://dx.doi.org/10.1002/jac5.1742. PubMed PMID: 2020606145.

2752. Amundson CJ, Sias JJ, Frietze GA. Perceptions of COVID-19 vaccines in a predominantly Hispanic patient population from the Texas–Mexico border. Journal of the American Pharmacists Association. 2022;62(4, Supplement):S17-S21.e2. doi: https://doi.org/10.1016/j.japh.2021.11.003.

2753. Amundstuen Reppe L, Spigset O, Schjott J. Drug Information Services Today: Current Role and Future Perspectives in Rational Drug Therapy. Clinical Therapeutics. 2016;38(2):414-21. PubMed PMID: 26831829.

2754. Amutah-Onukagha N, Mahadevan M, Opara I, Rodriguez M, Trusdell M, Kelly J. Project THANKS: Examining HIV/AIDS-Related Barriers and Facilitators to Care in African American Women: A Community Perspective. Aids Patient Care and Stds. 2018;32(4):119-28. doi: 10.1089/apc.2017.0215. PubMed PMID: WOS:000429445400001.

2755. Amvgn M, Jayamanne SF, Coombes J, De Silva A, Lynch CB, Wickramasinghe NDD. The impact of pharmacist counselling on glycaemic control among patients with diabetes mellitus: data from a Sri Lankan Teaching Hospital. Indian journal of endocrinology and metabolism. 2017;21(7):23-. PubMed PMID: CN-01437632.

2756. An J, Wei R, Zhou H, Luong TQ, Gould MK, Mefford MT, et al. Angiotensin-Converting Enzyme Inhibitors or Angiotensin Receptor Blockers Use and COVID-19 Infection Among 824 650 Patients With Hypertension From a US Integrated Healthcare System. Journal of the American Heart Association. 2021;10(3). doi: 10.1161/jaha.120.019669. PubMed PMID: WOS:000637752200039.

2757. An S, Malhotra K, Dilley C, Han-Burgess E, Valdez JN, Robertson J, et al. Predicting drug-resistant epilepsy — A machine learning approach based on administrative claims data. Epilepsy & Behavior. 2018;89:118-25. doi: https://doi.org/10.1016/j.yebeh.2018.10.013.

2758. An X, Li X, An H, Wang Y. Establishment and practice of the management model of prescription refills for chronic diseases during the COVID-19 epidemic. Journal of Xi'an Jiaotong University (Medical Sciences). 2022;43(5):666-70. doi: 10.7652/jdyxb202205006.

2759. An X, Shi C, Han Y, Li X, Dong L, Li Y, et al. The rational dose for MaXingShiGan decoction is crucial for its clinical effectiveness in treating bronchial pneumonia: three randomized, double-blind, dose-parallel controlled clinical studies. Frontiers in Pharmacology. 2023;14:1279519.

2760. Anacleto TA, Perini E, Rosa MB, César CC. Medication errors and drug-dispensing systems in a hospital pharmacy. Clinics. 2005;60:325-32.

2761. Anacleto TA, Perini E, Rosa MB, César CC. DRUG-DISPENSING ERRORS IN THE HOSPITAL PHARMACY. Clinics. 2007;62(3):243-50. doi: https://doi.org/10.1590/S1807-59322007000300007.

2762. Anadi I, Havrda DE, Owens-Mosby D, Shelton CM. Evaluation of Academic and Nonacademic Factors of First-Generation Students Transitioning to a Pharmacy Program. American Journal of Pharmaceutical Education. 2023;87(12):100598. doi: https://doi.org/10.1016/j.ajpe.2023.100598.

2763. Anaf S, Sheppard LA. Describing physiotherapy interventions in an emergency department setting: An observational pilot study. Accident and Emergency Nursing. 2007;15(1):34-9. doi: 10.1016/j.aaen.2006.09.005.

2764. Anagnostiadis E, Chatterjee SV. The Dangers of Buying Prescription Drugs from Rogue Wholesale Distributors. Journal of Medical Regulation. 2018;104(1):13-6. doi: 10.30770/2572-1852-104.1.13.

2765. Anand T, Ingle GK, Kishore J, Kumar R. ABC-VED analysis of a drug store in the Department of Community Medicine of a Medical College in Delhi. Indian Journal of Pharmaceutical Sciences. 2013;75(1):113-7. doi: 10.4103/0250-474X.113543.

2766. Ananda KN, Ghanashyam SD, Ahamad T, Bheemsain TV. Pharmacist-led collaborative approach in quality of life through 36-item short form survey questionnaire in patients on atypical antipsychotics. International journal of research in ayurveda and pharmacy. 2019;10(3):74-6. doi: 10.7897/2277-4343.100366. PubMed PMID: CN-01988001.

2767. Ananda-Rajah MR, Fitchett S, Ayton D, Peleg AY, Fleming S, Watson E, et al. Ushering in Antifungal Stewardship: Perspectives of the Hematology Multidisciplinary Team Navigating Competing Demands, Constraints, and Uncertainty. Open Forum Infectious Diseases. 2020;7(6). doi: 10.1093/ofid/ofaa168. PubMed PMID: WOS:000565181000010.

2768. Anandaraj M, Venkatesan G, Briskilla P, Sumathi V, editors. A Holistic Approach to Prevent Drug Misuse: Strategies and Framework for Comprehensive Support. 2023 Intelligent Computing and Control for Engineering and Business Systems (ICCEBS); 2023: IEEE.

2769. Anastassopoulos KP, Chow W, Tapia CI, Baik R, Ackerman SJ, Biondi D, et al. Economic Study on the Impact of Side Effects in Patients Taking Oxycodone Controlled-Release for Noncancer Pain. Journal of Managed Care Pharmacy. 2012;18(8):615-26. doi: 10.18553/jmcp.2012.18.8.615. PubMed PMID: WOS:000311065100003.

2770. Anazi AA. Medication reconciliation process: Assessing value, adoption, and the potential of information technology from pharmacists' perspective. Health Informatics Journal. 2021;27(1):1-7. doi: 10.1177/1460458220987276. PubMed PMID: 150395748. Language: English. Entry Date: 20210526. Revision Date: 20210526. Publication Type: Article.

2771. Ancell CD, Phipps J, Young L. Thymosin alpha-1. American journal of health-system pharmacy. 2001;58(10):879-85; quiz 86-8. doi: 10.1093/ajhp/58.10.886. PubMed PMID: CN-01766138.

2772. Anchah L, Hassali MA, Lim MS, Ibrahim MI, Sim KH, Ong TK. Health related quality of life assessment in acute coronary syndrome patients: the effectiveness of early phase I cardiac rehabilitation. Health and quality of life outcomes. 2017;15(1):10. doi: 10.1186/s12955-016-0583-7. PubMed PMID: CN-01327338.

2773. Anchah L, Hassali MA, Lim MSH, Ibrahim MIM, Sim KH, Ong TK. Health related quality of life assessment in acute coronary syndrome patients: the effectiveness of early phase I cardiac rehabilitation. Health and Quality of Life Outcomes. 2017;15. doi: 10.1186/s12955-016-0583-7. PubMed PMID: WOS:000394818200001.

2774. Anchordoquy TJ, Barenholz Y, Boraschi D, Chorny M, Decuzzi P, Dobrovolskaia MA, et al. Mechanisms and barriers in cancer nanomedicine: addressing challenges, looking for solutions. ACS Publications; 2017.

2775. Ancker JS, Edwards A, Nosal S, Hauser D, Mauer E, Kaushal R, et al. Effects of workload, work complexity, and repeated alerts on alert fatigue in a clinical decision support system. BMC medical informatics and decision making. 2017;17:1-9.

2776. Andaç A, Yaşayan G, Alarçin E, Okuyan B, Şahbaz S. Evaluation of pharmacy manager's knowledge

and attitude on extemporaneous preparation. Marmara Pharmaceutical Journal. 2015;19(3):283-9. doi: 10.12991/mpj.201519399879.

2777. Andalo D. Pharmacy denied a level playing-field. Pharmaceutical Journal. 2008;280(7507):744.

2778. Andalo D. Opening of 100-hour pharmacies has upsides and downsides for recruitment. Pharmaceutical Journal. 2008;280(7508):791-2.

2779. Andalo D. Using apps in community pharmacy. Pharmaceutical Journal. 2015;295(7876-7877):152-3.

2780. Andayi F, Emukule GO, Osoro E, Ndegwa LK, Otiato F, Muturi P, et al. Knowledge and attitude of Kenyan healthcare workers towards pandemic influenza disease and vaccination: 9 years after the last influenza pandemic. Vaccine. 2021;39(29):3991-6. doi: https://doi.org/10.1016/j.vaccine.2021.05.057.

2781. Andel C, Davidow SL, Hollander M, Moreno DA. The economics of health care quality and medical errors. Journal of health care finance. 2012;39(1):39.

2782. Anderegg MD, Gums TH, Uribe L, Coffey CS, James PA, Carter BL. Physician-Pharmacist Collaborative Management: narrowing the Socioeconomic Blood Pressure Gap. Hypertension (dallas, tex : 1979). 2016;68(5):1314-20. doi: 10.1161/HYPERTENSIONAHA.116.08043. PubMed PMID: CN-01401754.

2783. Anderegg MD, Gums TH, Uribe L, MacLaughlin EJ, Hoehns J, Bazaldua OV, et al. Pharmacist Intervention for Blood Pressure Control in Patients with Diabetes and/or Chronic Kidney Disease. Pharmacotherapy. 2018;38(3):309-18. doi: 10.1002/phar.2083. PubMed PMID: CN-01460727.

2784. Anderegg SV, Demik DE, Carter BL, Dawson JD, Farris K, Shelsky C, et al. Acceptance of recommendations by inpatient pharmacy case managers: unintended consequences of hospitalist and specialist care. Pharmacotherapy. 2013;33(1):11-21. doi: 10.1002/phar.1164. PubMed PMID: CN-00908072.

2785. Andersen AL, Houlind MB, Nielsen RL, Jorgensen LM, Treldal C, Damgaard M, et al. Optimization of Nutrition And Medication (OptiNAM) for acutely admitted older patients: protocol for a randomized single-blinded controlled trial. Trials. 2021;22(1):616. doi: 10.1186/s13063-021-05456-6. PubMed PMID: CN-02321447.

2786. Andersen CU, Hansen S, Dalhoff KP, Nielsen LP, Møller JM, Olesen AE. Clinical characteristics of hospitalized patients with paracetamol poisoning before and after restrictions of over-the-counter sale of paracetamol. Clinical Toxicology (15563650). 2023;61(4):228-33. doi: 10.1080/15563650.2023.2181104. PubMed PMID: 163520304.

2787. Andersen EM, Patrick DL, Carter WB, Malmgren JA. COMPARING THE PERFORMANCE OF HEALTH-STATUS MEASURES FOR HEALTHY OLDER ADMITS. Journal of the American Geriatrics Society. 1995;43(9):1030-4. doi: 10.1111/j.1532-5415.1995.tb05569.x. PubMed PMID: WOS:A1995RT90800013.

2788. Andersen G, Hedermann G, Witting N, Duno M, Andersen H, Vissing J. The antimyotonic effect of lamotrigine in non-dystrophic myotonias: a double-blind randomized study. Brain. 2017;140(9):2295-305. doi: 10.1093/brain/awx192. PubMed PMID: CN-01419887.

2789. Andersen M. The danish database oped. European Journal of Clinical Pharmacology. 2001;57(5):A8.

2790. Andersen S. Implementing a new drug record system: a qualitative study of difficulties perceived by physicians and nurses. BMJ Quality & Safety. 2002;11(1):19-24.

2791. Andersen S, Pedersen A, Bach K. Medication history on internal medicine wards: assessment of extra information collected from second drug interviews and GP lists. Pharmacoepidemiology and drug safety. 2003;12(6):491-8.

2792. Andersen SO, Beurling C. Prescriptions, prescription-writing errors and patient safety. [Danish]. Ugeskrift for laeger. 1997;159(10):1454-8. PubMed PMID: 127238815.

2793. Andersen SO, Beurling C. Recepter, receptfejl og patientsikkerhed prescription-writing errors. Ugeskrift for Laeger. 1997;159(10):1454-8.

2794. Andersen SR, St Peter JV, Macres MG, St Peter WL. Accuracy of technicians versus pharmacists in checking syringes prepared for a dialysis program. American Journal of Health-System Pharmacy. 1997;54(14):1611-3. PubMed PMID: 105819548. Language: English. Entry Date: 20080307. Revision Date: 20150711. Publication Type: Journal Article.

2795. Andersen SR, St. Peter JV, Macres MG, St. Peter WL. Accuracy of technicians versus pharmacists in checking syringes prepared for a dialysis program. American journal of health-system pharmacy. 1997;54(14):1611-3.

2796. Anderson A, Cooper J, Oyston C, Miles C, Sowden K, Peiris-John R, et al. Audit of waiting time-to-treatment of atypical endometrial hyperplasia. Australian & New Zealand Journal of Obstetrics & Gynaecology. 2022;62(5):707-13. doi: 10.1111/ajo.13551. PubMed PMID: WOS:000814127000001.

2797. Anderson A, Eisold JF. Anthrax attack at the United States Capitol. Front line thoughts. AAOHN journal : official journal of the American Association of Occupational Health Nurses. 2002;50(4):170-3. doi: 10.1177/216507990205000410.

2798. Anderson AS, Goode JVR. Engaging students in wellness and disease prevention services. American Journal of Pharmaceutical Education. 2006;70(2). doi: 10.5688/aj700240. PubMed PMID: WOS:000237246400017.

2799. Anderson B. Specialty Drug and Drug-Related Products Management across the Pharmacy and Medical Benefit: Experience of a Self-Insured Employer from 2006 to 2014 [Ph.D.]. United States -- Minnesota: University of Minnesota; 2021.

2800. Anderson B, Townsend T, CCRN-CMC C-B. Medication errors. American nurse today. 2010;44.

2801. Anderson BJ, Carroll ME, Taylor SE, Chow A. Perceptions of hospital pharmacists and pharmacy technicians towards expanding roles for hospital pharmacy technicians: a cross-sectional survey. Journal of Pharmacy Practice and Research. 2021;51(3):216-24. doi: 10.1002/jppr.1697.

2802. Anderson BJ, Ellis JF. Common errors of drug administration in infants: causes and avoidance. Pediatric Drugs. 1999;1(2):93-107.

2803. Anderson BJ, Merry AF. Paperless anesthesia: Uses and abuses of these data. Paediatric Anaesthesia. 2015;25(12):1184-92. doi: 10.1111/pan.12782.

2804. Anderson BJ, Taylor SE, Mitchell SM, Carroll ME, Verde A, Sepe D, et al. Evaluation of a novel advanced pharmacy technician role: discharge Medication Education Technician. Journal of pharmacy practice and research. 2021;51(5):396-405. doi: 10.1002/jppr.1752. PubMed PMID: CN-02341876.

2805. Anderson C. Global trends in pharmacy practice. Remington: The Science and Practice of Pharmacy2020. p. 769-80.

2806. Anderson C. Chapter 42 - Global trends in pharmacy practice. In: Adejare A, editor. Remington (Twenty-third Edition): Academic Press; 2021. p. 769-80.

2807. Anderson C, Bates I, Futter B, Gal D, Rouse M, Whitmarsh S. Global perspectives of pharmacy education and practice. World Medical & Health Policy. 2010;2(1):5-18.

2808. Anderson C, Bissell P. Using semi covert research to evaluate an emergency hormonal contraception service. Pharmacy World & Science. 2004;26(2):102-6. doi: 10.1023/B:PHAR.0000018598.83883.bc. PubMed PMID: WOS:000220804400009.

2809. Anderson C, Blenkinsopp A, Armstrong M. Pharmacists' perceptions regarding their contribution to improving the public's health: A systematic review of the United Kingdom and international literature 1990-2001. International Journal of Pharmacy Practice. 2003;11(2):111-20. doi: 10.1211/0022357021297.

2810. Anderson C, Blenkinsopp A, Armstrong M. Feedback from community pharmacy users on the contribution of community pharmacy to improving the public's health: A systematic review of the peer reviewed and non-peer reviewed literature 1990-2002. Health Expectations. 2004;7(3):191-202. doi: 10.1111/j.1369-7625.2004.00274.x.

2811. Anderson C, Blenkinsopp A, Armstrong M. Feedback from community pharmacy users on the contribution of community pharmacy to improving the public's health: a systematic review of the peer reviewed and non-peer reviewed titerature 1990-2002. Health Expectations. 2004;7(3):191-202. doi: 10.1111/j.1369-7625.2004.00274.x. PubMed PMID: WOS:000225715400003.

2812. Anderson C, Checkoway LH, Franklin GM, Beresford S, Smith-Weller T, Swanson PD. Dietary factors in Parkinson's disease: The role of food groups and specific foods. Movement Disorders. 1999;14(1):21-7. doi: 10.1002/1531-8257(199901)14:1<21::Aid-mds1006>3.3.Co;2-p. PubMed PMID: WOS:000082999200005.

2813. Anderson C, Sharma R. Primary health care policy and vision for community pharmacy and pharmacists in England. Pharmacy Practice (Granada). 2020;18(1).

2814. Anderson C, Thornley T. "it's easier in pharmacy": Why some patients prefer to pay for flu jabs rather than use the National Health Service. BMC Health Services Research. 2014;14. doi: 10.1186/1472-6963-14-35.

2815. Anderson C, Thornley T. Who uses pharmacy for flu vaccinations? Population profiling through a UK pharmacy chain. International Journal of Clinical Pharmacy. 2016;38(2):218-22. doi: 10.1007/s11096-016-0255-z.

2816. Anderson CM, Cummins SE, Kohatsu ND, Gamst AC, Zhu SH. Incentives and Patches for Medicaid Smokers: an RCT. American journal of preventive medicine. 2018;55(6 Suppl 2):S138-S47. doi: 10.1016/j.amepre.2018.07.015. PubMed PMID: CN-01666377.

2817. Anderson CM, Hultgren KE, Warholak T, Frye J, Levine J. Association Report: Changing the Conversation; How Every Pharmacist Can Help Decrease Medication Errors and Sleep Better at Night; Policy, Community Service at Forefront of Student Pharmacists' Patient Safety Activity. Journal of the American Pharmacists Association: JAPhA. 2016;56(1):7-10. doi: 10.1016/j.japh.2015.12.004. PubMed PMID: 114187680. Language: English. Entry Date: 20170601. Revision Date: 20191111. Publication Type: journal article. Journal Subset: Biomedical.

2818. Anderson CM, Hultgren KE, Warholak T, Frye J, Levine J. Association report. Journal of the American Pharmacists Association. 2016;56(1):7-8. doi: 10.1016/j.japh.2015.12.004.

2819. Anderson D, Villagra V, Coman EN, Zlateva I, Hutchinson A, Villagra J, et al. A Cost-Effectiveness Analysis of Cardiology eConsults for Medicaid Patients. American Journal of Managed Care. 2018;24(1):E9-E16. PubMed PMID: WOS:000423223900002.

2820. Anderson DJ, Watson S, Moehring RW, Komarow L, Finnemeyer M, Arias R, et al. Feasibility assessment of stewardship interventions in community hospitals: a multicenter, 3-stage cluster-randomized historically controlled crossover trial. Open forum infectious diseases. 2016;3. doi: 10.1093/ofid/ofw194.100. PubMed PMID: CN-01956401.

2821. Anderson DJ, Watson S, Moehring RW, Komarow L, Finnemeyer M, Arias RM, et al. Feasibility of Core Antimicrobial Stewardship Interventions in Community Hospitals. JAMA Network Open. 2019;2(8):e199369-e. doi: 10.1001/jamanetworkopen.2019.9369. PubMed PMID: 138094947. Language: English. Entry Date: 20190821. Revision Date: 20190916. Publication Type: Article.

2822. Anderson EJ, Axon DR, Taylor AM, Towers V, Warholak T, Johnson M, et al. Impact evaluation of a four-year academic-community partnership in provision of medication management and tertiary prevention services for rural patients with diabetes and/or hypertension. Preventive Medicine Reports. 2020;17:101038. doi: https://doi.org/10.1016/j.pmedr.2019.101038.

2823. Anderson EJ, Dhatt H, Vaffis S, Nelson ML, Warholak T, Campbell PJ, et al. Key informant perspectives about telephonic comprehensive medication review services in the United States. Journal of the American Pharmacists Association: JAPhA. 2022;62(3):817-25.e1. PubMed PMID: 35067476.

2824. Anderson GM, Lexchin J. Strategies for improving prescribing practice. CMAJ: Canadian Medical Association Journal. 1996;154(7):1013.

2825. Anderson H, Patterson VP, Wright G, Rawlings JE, Moore G, Leonard J, et al. Evaluating the Effect of COVID-19 on Outpatient Opioid Utilization Among Health First Colorado Members and a National Non-Medicaid Cohort: An Interrupted Time Series Analysis. Therapeutics and Clinical Risk Management. 2023;19:745-53. doi: 10.2147/tcrm.S424961. PubMed PMID: WOS:001076524100001.

2826. Anderson I, Nowling A, Meyers A, Nguyen D, Johanson E. Time is money: reimbursement for pharmacist provision of point-of-care testing. Journal of the american pharmacists association. 2018;58(3):e171-. doi: 10.1016/j.japh.2018.04.004. PubMed PMID: CN-01612155.

2827. Anderson JG. Clearing the way for physicians' use of clinical information systems. Communications of the ACM. 1997;40(8):83-90.

2828. Anderson JG, Jay SJ, Anderson M, Hunt TJ. Evaluating the capability of information technology to prevent adverse drug events: a computer simulation approach. Journal of the American Medical Informatics Association. 2002;9(5):479-90.

2829. Anderson JG, Ramanujam R, Hensel D, Anderson MM, Sirio CA. The need for organizational change in patient safety initiatives. International Journal of Medical Informatics. 2006;75(12):809-17. doi: https://doi.org/10.1016/j.ijmedinf.2006.05.043.

2830. Anderson JL, Horne BD, Stevens SM, Grove AS, Barton S, Nicholas ZP, et al. Randomized trial of genotype-guided versus standard warfarin dosing in patients initiating oral anticoagulation. Circulation. 2007;116(22):2563-70. doi: 10.1161/CIRCULATIONAHA.107.737312. PubMed PMID: CN-00620796.

2831. Anderson JP, Tchetgen EJT, Lo Re V, Tate JP, Williams PL, Seage GR, et al. Antiretroviral Therapy Reduces the Rate of Hepatic Decompensation Among HIV- and Hepatitis C Virus-Coinfected Veterans. Clinical Infectious Diseases. 2014;58(5):719-27. doi: 10.1093/cid/cit779. PubMed PMID:

WOS:000331846700023.

2832. Anderson JR, Hunter T, Dinallo JM, Glaser D, Roybal LK, Segovia A, et al. Population screening for atrial fibrillation by student pharmacists at health fairs. Journal of the American Pharmacists Association. 2020;60(4):e52-e7. doi: https://doi.org/10.1016/j.japh.2019.12.021.

2833. Anderson K, Foster M, Freeman C, Luetsch K, Scott I. Negotiating "Unmeasurable Harm and Benefit": Perspectives of General Practitioners and Consultant Pharmacists on Deprescribing in the Primary Care Setting. Qualitative Health Research. 2017;27(13):1936-47. doi: 10.1177/1049732316687732. PubMed PMID: 126067292. Language: English. Entry Date: 20171116. Revision Date: 20190712. Publication Type: Article.

2834. Anderson K, Freeman C, Foster M, Scott I. GP-Led Deprescribing in Community-Living Older Australians: An Exploratory Controlled Trial. Journal of the American Geriatrics Society. 2020;68(2):403-10. doi: 10.1111/jgs.16273. PubMed PMID: 141541393. Language: English. Entry Date: 20200207. Revision Date: 20210201. Publication Type: Article.

2835. Anderson K, Freeman C, Foster M, Scott I. GP-Led Deprescribing in Community-Living Older Australians: an Exploratory Controlled Trial. Journal of the American Geriatrics Society. 2020;68(2):403-10. doi: 10.1111/jgs.16273. PubMed PMID: CN-02180094.

2836. Anderson L, Borgelt LM, Clark P, Moore G. Encouraging comprehensive and consistent pharmacist education for provision of contraception. Journal of the American Pharmacists Association. 2018;58(1):113-6. doi: https://doi.org/10.1016/j.japh.2017.10.017.

2837. Anderson L, Hartung DM, Middleton L, Rodriguez MI. Pharmacist provision of hormonal contraception in the Oregon Medicaid population. Obstetrics & Gynecology. 2019;133(6):1231-7.

2838. Anderson LJ, Emswiller CF. Resolving Role Conflict: Suggestions from Practitioners: Two well-known community pharmacists were asked by American Pharmacy to discuss the problems in role conflict with physicians encountered in their day-to-day patient counseling programs and what they suggest for overcoming them. Here's what they said. American Pharmacy. 1981;21(7):40. doi: https://doi.org/10.1016/S0160-3450(16)31382-4.

2839. Anderson LJ, Schnipper JL, Nuckols TK, Shane R, Le MM, Robbins K, et al. Effect of medication reconciliation interventions on outcomes: A systematic overview of systematic reviews. American Journal of Health-System Pharmacy. 2019;76(24):2028-40. PubMed PMID: 31789354.

2840. Anderson LM. Adherence and Quality of Life in Pediatric Sickle Cell Disease: A Pilot Mobile Health Intervention [Ph.D.]. United States -- North Carolina: Duke University; 2017.

2841. Anderson M, O'Neill C, Clark JM, Street A, Woods M, Johnston-Webber C, et al. Securing a sustainable and fit-for-purpose UK health and care workforce. The Lancet. 2021;397(10288):1992-2011.

2842. Anderson p. Improving safety for people taking medicines.

2843. Anderson R, Doucette W, Veach S, Witry M, McDonough R. Retrospective analysis of medication therapy problems identified during medication synchronization. Journal of the american pharmacists association. 2018;58(3):e86-. doi: 10.1016/j.japh.2018.04.004. PubMed PMID: CN-01612164.

2844. Anderson R, Groundwater PW, Todd A, Worsley A. Antibacterial agents: chemistry, mode of action, mechanisms of resistance and clinical applications: John Wiley & Sons; 2012.

2845. Anderson RJ, Nykamp D, Miyahara RK. Documentation of Pharmaceutical Care Activities in Community Pharmacies by Doctor of Pharmacy Students. Journal of Pharmacy Practice. 1995;8(2):83-8. doi: 10.1177/089719009500800206.

2846. Anderson RW. The evolution and assessment of pharmaceutical care: Structure, processes, and outcomes [Dr.P.H.]. United States -- Texas: The University of Texas School of Public Health; 1997.

2847. Anderson S. Community pharmacy in Great Britain: mediation at the boundary between professional and lay care, 1920 to 1995. Biographies of Remedies: Brill; 2002. p. 75-97.

2848. Anderson S. The state of the world's pharmacy: a portrait of the pharmacy profession. Journal of interprofessional care. 2002;16(4):391-404.

2849. Anderson S. Making medicines: a brief history of pharmacy and pharmaceuticals: Pharmaceutical Press; 2005.

2850. Anderson S. Community pharmacy and public health in Great Britain, 1936 to 2006: How a phoenix rose from the ashes. Journal of Epidemiology and Community Health. 2007;61(10):844-8. doi: 10.1136/jech.2006.055442.

2851. Anderson SL, Marrs JC. A Review of the Role of the Pharmacist in Heart Failure Transition of Care. Advances in Therapy. 2018;35(3):311-23. PubMed PMID: 29488151.

2852. Anderson SL, Marrs JC, Chachas CR, Cichon BS, Cizmic AD, Calderon BB, et al. Evaluation of a Pharmacist-Led Intervention to Improve Statin Use in Persons with Diabetes. Journal of Managed Care & Specialty Pharmacy. 2020;26(7):910-7. PubMed PMID: 32584671.

2853. Anderson SM, Geyer S, Cailor SM, Chen AMH. Impact of a team-based learning drug misuse education training program on student pharmacists' confidence. Currents in Pharmacy Teaching and Learning. 2019;11(1):58-65. doi: 10.1016/j.cptl.2018.09.016.

2854. Anderson SP, Dunn C, Laughter A, Yoon L, Swanson C, Stulnig TM, et al. Overlapping transcriptional programs regulated by the nuclear receptors peroxisome proliferator-activated receptor α, retinoid X receptor, and liver X receptor in mouse liver. Molecular pharmacology. 2004;66(6):1440-52.

2855. Anderson TS, Jing B, Wray CM, Ngo S, Xu E, Fung K, et al. Comparison of Pharmacy Database Methods for Determining Prevalent Chronic Medication Use. Medical care. 2019;27. PubMed PMID: 629174915.

2856. Anderson TS, Jing BC, Wray CM, Ngo S, Xu E, Fung K, et al. Comparison of Pharmacy Database Methods for Determining Prevalent Chronic Medication Use. Medical Care. 2019;57(10):836-42. doi: 10.1097/mlr.0000000000001188. PubMed PMID: WOS:000487642400015.

2857. Andersson A, Ström K, Brodin H, Alton M, Boman G, Jakobsson P, et al. Domiciliary liquid oxygen versus concentrator treatment in chronic hypoxaemia: a cost-utility analysis. The european respiratory journal. 1998;12(6):1284-9. doi: 10.1183/09031936.98.12061284. PubMed PMID: CN-00158583.

2858. Andersson AC, Brodin H, Nilsson JLG. Pharmacy representatives: A non-pharmacy outlet of prescribed medicines in Sweden. Pharmacy World and Science. 2002;24(4):139-43. doi: 10.1023/A:1019525202489.

2859. Andersson ÅC, Brodin H, Nilsson JLG. Pharmacist interventions in relation to patient drug-related problems. Journal of Social and Administrative Pharmacy. 2003;20(3):82-91.

2860. Andersson K, Jörgensen T, Carlsten A. Physicians' opinions and experiences of the Pharmaceutical Benefits Reform. Scandinavian Journal of Public Health. 2006;34(6):654-9. doi: 10.1080/14034940600551111.

2861. Andersson ML, Bottiger Y, Bastholm-Rahmner P, Ovesjo ML, Veg A, Eiermann B. Evaluation of usage patterns and user perception of the drug-drug interaction database SFINX. International Journal of Medical Informatics. 2015;84(5):327-33. PubMed PMID: 25670228.

2862. Andia T, Mantilla C, Morales Á, Ortiz S, Rodríguez-Lesmes P. Extracting Low-Cost Signals of Perceived Quality Control in Community Pharmacies: A Simulated Client Study of Contraceptive Dispensing in Bogotá. Lecturas de Economia. 2022;(96):9-30. doi: 10.17533/udea.le.n96a344243.

2863. Andolina KL, Tolosa JE, Monzo JM, Roberts NS, Daly S. Oral misoprostol is rapidly absorbed in postpartum women at term. Journal of maternal-fetal & neonatal medicine. 2003;14(4):229-32. doi: 10.1080/jmf.14.4.229.232. PubMed PMID: CN-00559532.

2864. Andrade SE, Gurwitz JH. Drug benefit plans under managed care: To what extent do older subscribers selecting less drug coverage put themselves at increased financial risk? Journal of the American Geriatrics Society. 2002;50(1):178-81. doi: 10.1046/j.1532-5415.2002.50026.x.

2865. Andrade SE, Kahler KH, Frech F, Chan KA. Methods for evaluation of medication adherence and persistence using automated databases. Pharmacoepidemiology and drug safety. 2006;15(8):565-74.

2866. Andrade SE, Lo JC, Roblin D, Fouayzi H, Connor DF, Penfold RB, et al. Antipsychotic Medication Use Among Children and Risk of Diabetes Mellitus. Pediatrics. 2011;128(6):1135-41. doi: 10.1542/peds.2011-0855. PubMed PMID: WOS:000298131400054.

2867. Andrawis M, Carmichael J, Collins CD, Dopp AL, Ellison LTCC, Mahan K, et al. Improving patient care and demonstrating value during a global pandemic: Recommendations from leaders of the Pharmacy Accountability Measures Work Group. American Journal of Health-System Pharmacy. 2020;77(23):2003-5. doi: 10.1093/ajhp/zxaa291.

2868. Andrawis M, Ellison L, Riddle S, Mahan KC, Collins CD, Brummond P, et al. Recommended quality measures for health-system pharmacy: 2019 update from the Pharmacy Accountability Measures Work Group. American Journal of Health-System Pharmacy. 2019;76(12):874-87. PubMed PMID: 31361855.

2869. André D, Chatain C, Chaumais MC, Rieutord A, Roy S. Pharmaceutical cares as means of prevention against drug iatrogenic: Case of oral anticoagulant. Annales Pharmaceutiques Francaises. 2022;80(4):494-506. doi: 10.1016/j.pharma.2021.08.009.

2870. Andrea B, Anna C, Jurg K, Andree F, Franz E, Martin N, et al. Management of hepatitis C in decentralised versus centralised drug substitution programmes and minimally invasive point-of-care tests to close gaps in the HCV cascade. Swiss Medical Weekly. 2017;147. doi: 10.4414/smw.2017.14544. PubMed PMID: WOS:000417853500008.

2871. Andrea B, Bicanic LA, Orehovacki H, De Oliveira DR, Mucalo I. Impact of collaborative practice between pharmacists and general practitioners as a key for medication mismanagement of elderly cardiovascular patients. International Journal of Clinical Pharmacy. 2023;45(2):536. doi: https://dx.doi.org/10.1007/s11096-023-01576-y. PubMed PMID: 641388408.

2872. Andreadis, II, Gioumouxouzis CI, Eleftheriadis GK, Fatouros DG. The Advent of a New Era in Digital Healthcare: A Role for 3D Printing Technologies in Drug Manufacturing? Pharmaceutics. 2022;14(3). doi: 10.3390/pharmaceutics14030609. PubMed PMID: WOS:000774329500001.

2873. Andreadis II, Gioumouxouzis CI, Eleftheriadis GK, Fatouros DG. The Advent of a New Era in Digital Healthcare: A Role for 3D Printing Technologies in Drug Manufacturing? Pharmaceutics. 2022;14(3). doi: 10.3390/pharmaceutics14030609.

2874. Andreassen L, Houghton J, Kjome R, Sandberg S, Sølvik U, Kristensen G, et al. A descriptive study of care home patients with diabetes - Prevalence, comorbidities, drug treatment and health care resource use. International journal of pharmacy practice. 2014;22:3-4. PubMed PMID: CN-01008998.

2875. Andreatta G, De Giovanni L, Serafini P. Optimal shift coloring of trees. Operations Research Letters. 2014;42(4):251-6. doi: https://doi.org/10.1016/j.orl.2014.04.004.

2876. Andreatta G, De Giovanni L, Serafini P. Optimal shift partitioning of pharmacies. Computers & Operations Research. 2015;55:88-98. doi: https://doi.org/10.1016/j.cor.2014.09.009.

2877. Andrés J. From evidence to practice: Aplication to pharmaceutical care in asthma. Ars Pharmaceutica. 1999;40(4):179-86.

2878. Andres J, Lott S, Qureshi K. Eight-Week Outcomes of Ledipasvir/Sofosbuvir in Noncirrhotic Treatment-Naive Patients with Hepatitis C: Analysis of Pharmacy-Based Data. Journal of Managed Care & Specialty Pharmacy. 2018;24(1):23-8. doi: 10.18553/jmcp.2018.24.1.23. PubMed PMID: WOS:000423386600004.

2879. Andres J, Ruchalski C, Katz P, Linares G. Drug therapy management in a pharmacist-run stroke prevention clinic. American Journal of Health-System Pharmacy. 2016;73(18):1388-90. PubMed PMID: 27605317.

2880. Andres Jacome J, Inesta Garcia A. Prospective study about the impact of a community pharmaceutical care service in patients with asthma. Revista espanola de salud publica. 2003;77(3):393-403. PubMed PMID: CN-00451504.

2881. Andrés Jácome J, Iñesta García A. Drug-related problems identified in asthmatic patients. Pharmaceutical Care Espana. 2004;6(4):210-7.

2882. Andres RB, Castillon EM, Dandan N, Sarabosing J. A Review on Hospital Prescription Errors in Asia.

2883. Andres Rodriguez NF, Fornos Perez JA, Andres Iglesias JC. Assessment of knowledgwe/compliance in a drug therapy follow-up program involving type 2 diabetic patients in community pharmacy: a randomized study. Pharmaceutical care espana. 2007;9(1):2-9. PubMed PMID: CN-00754602.

2884. Andres-Rodriguez NF, Fornos-Perez JA, Mera-Gallego I, Todo MI, Tous S, Molinero A. Blood pressure screening campaign "May Measurement Month 2017": An analysis of screening in Spanish community pharmacies. Farmaceuticos Comunitarios. 2019;11(2):5-13. doi: 10.5672/FC.2173-9218.(2019/Vol11).002.02. PubMed PMID: WOS:000473306400002.

2885. Andres-Rodriguez NF, Mera-Gallego R, Pineiro-Abad A, Acuna-Ferradanes A, Mera-Gallego I, Garcia-Rodriguez P, et al. Influenza vaccination in the community pharmacy: opinion of patients and pharmacists. Farmaceuticos Comunitarios. 2018;10(3):15-24. doi: 10.5672/FC.2173-9218.(2018/Vol10).003.03. PubMed PMID: WOS:000445964800003.

2886. Andresen EM, Patrick DL, Carter WB, Malmgren JA. Comparing the Performance of Health Status Measures for Healthy Older Adults. Journal of the American Geriatrics Society. 1995;43(9):1030-3. doi: 10.1111/j.1532-5415.1995.tb05569.x.

2887. Andreski M, Friedrich-Myers M, Pudlo A. Baseline description of seven pharmacies involved in evaluation of a new practice model. Journal of the American Pharmacists Association. 2015;55(2):e201-e2. PubMed PMID: 71971018.

2888. Andreski M, Meyers M, Gainer K, Pudlo A. The iowa new practice model: expansion of an initiative to enable pharmacists to provide more patient care services. Journal of the american pharmacists association. 2018;58(3):e86-e7. doi: 10.1016/j.japh.2018.04.004. PubMed PMID: CN-01612162.

2889. Andreski M, Myers M, Gainer K, Pudlo A. An initiative to enable pharmacists to provide more patient care services by using technician verification of prescription filling: the iowa new practice model. Journal of the american pharmacists association. 2017;57(3). doi: 10.1016/j.japh.2017.04.011. PubMed PMID: CN-01608605.

2890. Andreski M, Myers M, Gainer K, Pudlo A. The Iowa new practice model: Advancing technician roles to increase pharmacists' time to provide patient care services. Journal of the American Pharmacists Association. 2018;58(3):268-74. e1.

2891. Andreski MT. Development of a model of the Pharmacy and Therapeutics Committee to predict the level of prescriber adoption of its' decisions [Ph.D.]. United States -- Iowa: The University of Iowa; 2009.

2892. Andreu G, Morel P, Forestier F, Debeir J, Rebibo D, Janvier G, et al. Hemovigilance network in France: organization and analysis of immediate transfusion incident reports from 1994 to 1998. Transfusion. 2002;42(10):1356-64.

2893. Andreu-March M, Compaired MA, Busom MP, Marino EL, Modamio P. Development and Validation of the Hospital Outpatients' Information Needs Questionnaire (HOINQ). Patient Preference and Adherence. 2021;15:653-64. doi: 10.2147/ppa.S280816. PubMed PMID: WOS:000638104900001.

2894. Andreu-March M, Fernandez-Pellicer F, Modamio P, Marino EL. Turn back crime: illicit products and the role of the community pharmacist. Farmaceuticos Comunitarios. 2016;8(4):26-33. doi: 10.5672/FC.2173-9218.(2016/Vol8).004.04. PubMed PMID: WOS:000417275700004.

2895. Andrews AW, Chinworth SA, Bourassa M, Garvin M, Benton D, Tanner S. Update on distance and velocity requirements for community ambulation. Journal of geriatric physical therapy. 2010;33(3):128-34.

2896. Andrews GP, Laverty TP, Jones DS. Mucoadhesive polymeric platforms for controlled drug delivery. European journal of pharmaceutics and biopharmaceutics. 2009;71(3):505-18.

2897. Andrews J. Perhaps "retail pharmacy" is a better descriptor than community pharmacy. Pharmaceutical Journal. 2010;284(7606):604.

2898. Andrews LB, Candelario DM, Jensen SM. Optimizing inpatient pharmacotherapy using a single clinical policy streamlining pharmacy protocols. Journal of Clinical Outcomes Management. 2015;22(4):169-77.

2899. Andrews LB, Patel TA, Wasylyk P, Mundhenk K, St John F, Desai S, et al. Elevating critical care pharmacy services in a resource-limited environment through establishment of a pharmacist team. Journal of Clinical Outcomes Management. 2019;26(4):159-67.

2900. Andrews SB, Marcy TR, Osborn B, Planas LG. The impact of an appointment-based medication synchronization programme on chronic medication adherence in an adult community pharmacy population. Journal of Clinical Pharmacy and Therapeutics. 2017;42(4):461-6. doi: 10.1111/jcpt.12533.

2901. Andreyev J, Ross P, Donnellan C, Lennan E, Leonard P, Waters C, et al. Guidance on the management of diarrhoea during cancer chemotherapy. The Lancet Oncology. 2014;15(10):e447-e60. doi: https://doi.org/10.1016/S1470-2045(14)70006-3.

2902. Andrick B, Mathur A, Maiers T, Graham J, Vadakara J. Pharmacist Versus Physician Provider Management of Hydroxyurea Therapy for Polycythemia Vera (PERMANACE). Blood. 2020;136:44-5. doi: https://doi.org/10.1182/blood-2020-142864.

2903. Andrick B, Tusing L, Jones LK, Hu Y, Sneidman R, Gregor C, et al. The Impact of a Hematopoietic Cellular Therapy Pharmacist on Clinical and Humanistic Outcomes: A RE-AIM Framework Analysis. Transplantation and Cellular Therapy. 2022;28(6):334.e1-.e9. doi: https://doi.org/10.1016/j.jtct.2022.02.015.

2904. Andrikyan W, Jung-Poppe L, Altenbuchner A, Nicolaus HF, Pfistermeister B, Dormann H, et al. Documentation of Drug-Related Problems with ICD-11: Application of the New WHO Code-Set to Clinical Routine Data. Journal of Clinical Medicine. 2023;12(1):315. doi: 10.3390/jcm12010315. PubMed PMID: 161186582.

2905. Andrikyan W, Then MI, Durr P, Jung-Poppe L, Schuster AK, Weisbach L, et al. Comprehensibility and clinical relevance of absolute contraindications - the perspective of German pharmacists and physicians. Naunyn-Schmiedeberg's Archives of Pharmacology. 2023;396(Supplement 1):S48. doi: https://dx.doi.org/10.1007/s00210-023-02397-6. PubMed PMID: 640831532.

2906. Andrikyan W, Then MI, Gasmann KG, Tumena T, Durr P, Fromm MF, et al. Use of medication data alone to identify diagnoses and related contraindications: Application of algorithms to close a common documentation gap. British Journal of Clinical Pharmacology. 2022;88(12):5399-411. PubMed PMID: 35877931.

2907. Andrikyan W, Then MI, Gaßmann KG, Tümena T, Dürr P, Fromm MF, et al. Use of medication data alone to identify diagnoses and related contraindications: Application of algorithms to close a common documentation gap. British Journal of Clinical Pharmacology. 2022;88(12):5399-411. doi: 10.1111/bcp.15469.

2908. Andronicou AM, Krska J, Hackett A, Richards J. Supply of over-the-counter weight-loss products from community pharmacies. International Journal of Pharmacy Practice. 2009;17(6):333-7. doi: 10.1211/ijpp.17.06.0003.

2909. Andrulis DP, Brach C. Integrating literacy, culture, and language to improve health care quality for diverse populations. American journal of health behavior. 2007;31(1):S122-S33.

2910. Andrus MR. Student pharmacist initiated medication reconciliation in the outpatient setting. Pharmacy Practice (1886-3655). 2012;10(2):78-82. PubMed PMID: 104355732. Language: English. Entry Date: 20130109. Revision Date: 20180305. Publication Type: Journal Article.

2911. Andrus MR, Forrester JB, Germain KE, Eiland LS. Accuracy of Pharmacy Benefit Manager Medication Formularies in an Electronic Health Record System and the Epocrates Mobile Application. Journal of Managed Care & Specialty Pharmacy. 2015;21(4):281-6. doi: 10.18553/jmcp.2015.21.4.281. PubMed PMID: WOS:000351787200002.

2912. Andrus MR, Roth MT. Health literacy: a review. Pharmacotherapy: The Journal of Human Pharmacology and Drug Therapy. 2002;22(3):282-302.

2913. Andrus MR, Stevenson TL. Three-year review of pharmacy students' interventions and activities in an outpatient teaching family medicine center. Currents in Pharmacy Teaching and Learning. 2015;7(2):192-8.

doi: 10.1016/j.cptl.2014.11.016. PubMed PMID: WOS:000409734800008.

2914. Andrusko D. "How Painful is Medication Abortion?" Plenty! National Right to Life News. 2023:46-. PubMed PMID: 162985790.

2915. Andrzejewski C, McCreary EK, Khadem T, Abdel-Massih RC, Bariola JR. Tele-antimicrobial stewardship programs: A review of the literature and the role of the pharmacist. JACCP Journal of the American College of Clinical Pharmacy. 2021;4(8):1016-33. doi: 10.1002/jac5.1489.

2916. Anell A. The public-private pendulum-patient choice and equity in Sweden. New England Journal of Medicine. 2015;372(1):1-4. doi: 10.1056/NEJMp1411430.

2917. Ang F, Pau JE, Koh EW, Loh NKL, Yeoh W, Liang JW, et al. Drug-related problems associated with community-dwelling older persons living alone in Singapore. International Journal of Clinical Pharmacy. 2019. doi: 10.1007/s11096-019-00813-7.

2918. Ang JW, Chia C, Koh CJ, Chua BW, Narayanaswamy S, Wijaya L, et al. Healthcare-seeking behaviour, barriers and mental health of non-domestic migrant workers in Singapore. BMJ Global Health. 2017;2(2):e000213.

2919. Ang KTM, Saini B, Wong K. Sleep health awareness in pharmacy undergraduates and practising community pharmacists. Journal of Clinical Pharmacy and Therapeutics. 2008;33(6):641-52. doi: 10.1111/j.1365-2710.2008.00963.x. PubMed PMID: WOS:000260823600009.

2920. Ang WC, Fadzil MS, Ishak FN, Adenan NN, Mohamed MHN. Readiness and willingness of Malaysian community pharmacists in providing vaccination services. Journal of Pharmaceutical Policy and Practice. 2022;15(1). doi: 10.1186/s40545-022-00478-0. PubMed PMID: WOS:000882376600001.

2921. Ang WC, Fadzil MS, Ishak FN, Adenan NN, Nik Mohamed MH. Readiness and willingness of Malaysian community pharmacists in providing vaccination services. Journal of Pharmaceutical Policy and Practice. 2022;15(1). doi: 10.1186/s40545-022-00478-0.

2922. Angaran DM, Bonal J, Eide G, Koda-Kimble MA, Lake KD, Leufkens HG. Clinical pharmacy: looking 20 years back... looking 20 years forward. Pharmacotherapy:The Journal of Human Pharmacology & Drug Therapy. 2000;20(10 Pt 2):235S-42S. PubMed PMID: 11034049.

2923. Ângelo A, Barata J, Da Cunha PR, Almeida V, editors. Digital transformation in the pharmaceutical compounds supply chain: Design of a service ecosystem with e-labeling. Lecture Notes in Business Information Processing; 2017.

2924. Ângelo A, Barata J, Santos APM, editors. Projecting the community pharmacy into home health care: An IS perspective. Proceedings of the International Conference on Electronic Business (ICEB); 2020.

2925. Angelo LB. Engaging students in self-care activities during an advanced community pharmacy practice experience. American journal of pharmaceutical education. 2006;70(6):143. doi: 10.5688/aj7006143.

2926. Angelo LB. Shared decision-making for vaccines. Journal of the American Pharmacists Association. 2020;60(6):e55-e9. doi: https://doi.org/10.1016/j.japh.2020.05.010.

2927. Angelo LB, Christensen DB, Ferreri SP. Impact of community pharmacy automation on workflow, workload, and patient interaction. Journal of the American Pharmacists Association. 2005;45(2):138-44. doi: 10.1331/1544345053623537.

2928. Angelo LB, Ferreri SP. Assessment of workflow redesign in community pharmacy. Journal of the American Pharmacists Association. 2005;45(2):145-50. doi: 10.1331/1544345053623474.

2929. Angelo LB, Maffeo CM. Local and global volunteer opportunities for pharmacists to contribute to public health. International Journal of Pharmacy Practice. 2011;19(3):206-13. doi: 10.1111/j.2042-7174.2011.00123.x.

2930. Angelova S. Attitudes Towards Development of Innovative Pharmaceutical Products in Bulgaria [Ph.D.]. Bulgaria: Medical University of Varna (Bulgaria); 2018.

2931. Angelucci E, Deconinck E, Manteigas D, Zuurman M, Herling M. Durable Outcomes with Manageable Safety Leading to Prolonged Survival with Tagraxofusp for Treatment-Naive Patients with Blastic Plasmacytoid Dendritic Cell Neoplasm: Updated Results from a European Named Patient Program. Blood. 2023;142:547. doi: https://doi.org/10.1182/blood-2023-178734.

2932. Anggraini S, Utami W, Zairina E. Factors affecting community pharmacist's service for women with chronic diseases during pregnancy and breastfeeding: Application of the Health Belief Model. Journal of Basic and Clinical Physiology and Pharmacology. 2020;30(6). doi: 10.1515/jbcpp-2019-0347.

2933. Angione SL, Inde Z, Beck CM, Artenstein AW, Opal SM, Tripathi A. Microdroplet Sandwich Real-Time RT-PCR for Detection of Pandemic and Seasonal Influenza Subtypes. PLoS ONE. 2013;8(9). doi: 10.1371/journal.pone.0073497.

2934. Angira CH, Otieno OA, Muga RO, Abong'o BO. Factors contributing to antimalarial drug resistance in Rachuonyo district, Kenya. East African journal of public health. 2010;7(1):11-5.

2935. Angkana S, Sunicha C, Varavoot S, Somsajee S, Walaiporn P, Shunmay Y, et al. Antibiotic distribution channels in Thailand: results of key-informant interviews, reviews of drug regulations and database searches. Bulletin of the World Health Organization. 2018;96(2):101-9. doi: 10.2471/BLT.17.199679. PubMed PMID: 132310764. Language: English. Entry Date: 20181015. Revision Date: 20220624. Publication Type: Article.

2936. Angkasekwinai N, Werarak P, Tongsai S, Thamlikitkul V. Effectiveness and safety of generic formulation of meropenem (Penem) for treatment of infections at Siriraj Hospital. Journal of the Medical Association of Thailand = Chotmaihet thangphaet. 2012;95 Suppl 2:S34-41.

2937. Anglade C, Le Dorze G, Croteau C. Service encounter interactions of people living with moderate-to-severe post-stroke aphasia in their community. Aphasiology. 2019;33(9):1061-82. doi: 10.1080/02687038.2018.1532068. PubMed PMID: WOS:000473211600002.

2938. Angonesi D, Renno MUP. Dispensation of Pharmaceuticals: proposal of a model for dispensation. Ciencia & Saude Coletiva. 2011;16(9):3883-91. doi: 10.1590/s1413-81232011001000024. PubMed PMID: WOS:000295723000027.

2939. Angoulvant F, Rouault A, Prot-Labarthe S, Boizeau P, Skurnik D, Morin L, et al. Randomized controlled trial of parent therapeutic education on antibiotics to improve parent satisfaction and attitudes in a pediatric emergency department. PloS one. 2013;8(9):e75590. doi: 10.1371/journal.pone.0075590. PubMed PMID: CN-01121226.

2940. Angstman G. Getting physician and organization buy-in: A disease management strategy. Joint Commission Journal on Quality Improvement. 1996;22(8):551-6. doi: 10.1016/s1070-3241(16)30255-3. PubMed PMID: WOS:A1996VF43100003.

2941. Anhorn R, Ashcraft K, Beattie M, Berliner E, Bordenave K, Bourne P, et al. AMCP Partnership Forum:

Managing Care in the Wave of Precision Medicine. Journal of Managed Care & Specialty Pharmacy. 2018;24(7):583-8. doi: 10.18553/jmcp.2018.24.7.583. PubMed PMID: WOS:000439907600001.

2942. Anifowoshe RA, Morrison IO, Isaac AA. Microbial quality of some non-sterile pharmaceutical products sourced from some retail pharmacies in Lagos, Nigeria. African Journal of Microbiology Research. 2012;6(23):4903-7. doi: 10.5897/ajmr11.1158. PubMed PMID: WOS:000307503400009.

2943. Anis MS, Tan ML. Exploring OTC drug consumers' perception towards online shopping and digital marketing through qualitative interviews: A sample from Malaysia. International Journal of Healthcare Management. doi: 10.1080/20479700.2022.2163865. PubMed PMID: WOS:000907861500001.

2944. Anis MS, Tan ML. Exploring OTC drug consumers' perception towards online shopping and digital marketing through qualitative interviews: A sample from Malaysia. International Journal of Healthcare Management. 2024;17(1):168-76. doi: 10.1080/20479700.2022.2163865. PubMed PMID: WOS:000907861500001.

2945. Aniteye P, Mayhew SH. Shaping legal abortion provision in Ghana: Using policy theory to understand provider-related obstacles to policy implementation. Health Research Policy and Systems. 2013;11(1). doi: 10.1186/1478-4505-11-23.

2946. Anjalee JAL, Rutter V, Samaranayake NR. Application of Failure Mode and Effect Analysis (FMEA) to improve medication safety: a systematic review. Postgraduate Medical Journal. 2021;97(1145):168-74.

2947. Anjorin O, Okpala O, Adeyemi O. Coordinating Nigeria's micronutrient deficiency control programs is necessary to prevent deficiencies and toxicity risks. Annals of the New York Academy of Sciences. 2019;1446(1):153-69. doi: 10.1111/nyas.14055.

2948. Anna SV, Bernadett N, Gyorgy VR, Andrea L, Lajos B. Comprehensive evaluation of drug interaction screening programs: discrepancies and concordances. Orvosi Hetilap. 2015;156(18):720-30. doi: 10.1556/oh.2015.30134. PubMed PMID: WOS:000353978400002.

2949. Annabel B. Message with a difference. Australian Journal of Pharmacy. 2012;93(1111):59.

2950. Annable D. Developing a targeted pharmacy oncology guide. Journal of Oncology Pharmacy Practice. 2004;10(4):211-5. doi: 10.1191/1078155204jp139oa.

2951. Annaciri-Donkers A, Ng S. Improving oral drug administration in patients with swallowing problems and feeding tubes. Pharmacy Education. 2023;23(3):165. doi: https://dx.doi.org/10.46542/pe.2023.233.156186. PubMed PMID: 642266097.

2952. Annane D, Lerolle N, Meuris S, Sibilla J, Olsen KM. Academic conflict of interest. Intensive Care Medicine. 2019;45(1):13-20. doi: 10.1007/s00134-018-5458-4.

2953. Annisa AT, Yasin NM, Kristina SA. Medication errors analysis in Asia and Australia: A systematic review. Pharmaceutical Sciences Asia. 2023;50(2):100-12. doi: 10.29090/psa.2023.02.22.291. PubMed PMID: 164975162.

2954. Anokbonggo WW, Ogwal-Okeng JW, Obua C, Aupont O, Ross-Degnan D. Impact of decentralization on health services in Uganda: a look at facility utilization, prescribing and availability of essential drugs. East African medical journal. 2004;Suppl:S2-7.

2955. Anonymous. ASHP strategic-planning report for 1998-99. American Journal of Health-System Pharmacy. 1998;55(16):1717-21. PubMed PMID: 9740910.

2956. Anonymous. Relief for the Rx blues: long waits at the drugstore? Incomplete advice? High prices? We prescribe this report. Consumer Reports. 1999;64(10):38-46. PubMed PMID: 10848095.

2957. Anonymous. Jury finds pharmacy's misfilling of prescription caused child's brain damage. Journal of Child Neurology. 1999;14(4):263-4. PubMed PMID: 10334402.

2958. Anonymous. Sending PharmD candidates to patients' homes can avert potential medication problems. Clinical Resource Management. 2000;1(12):186-8, 77. PubMed PMID: 11195434.

2959. Anonymous. System uses pharmacists to take on medication errors. Clinical Resource Management. 2000;1(6):81-5. PubMed PMID: 11014793.

2960. Anonymous. Cut medication errors in half with ED pharmacist. Hospital Case Management. 2002;10(9):139-41. PubMed PMID: 12355610.

2961. Anonymous. Society to seek broader powers to regulate retail pharmacy premises. Pharmaceutical Journal. 2003;271(7262):217-20. PubMed PMID: 37063479.

2962. Anonymous. Nurses and pharmacists partner for patient safety. Healthcare Benchmarks & Quality Improvement. 2003;10(8):92-3. PubMed PMID: 12901320.

2963. Anonymous. Are pediatric drug errors occurring in your ED? Act now before tragedy strikes. ED Management. 2003;15(3):25-7. PubMed PMID: 12655893.

2964. Anonymous. Peak expiratory flow rate does not predict asthma exacerbations. Journal of family practice. 2004;53(8):608. PubMed PMID: CN-00496633.

2965. Anonymous. ASHP Guidelines on the Pharmacist's Role in the Development, Implementation, and Assessment of Critical Pathways. American Journal of Health-System Pharmacy. 2004;61(9):939-45. PubMed PMID: 15156971.

2966. Anonymous. Help pharmacists meet new medication standards. Hospital Peer Review. 2004;29(3):33-4, 9. PubMed PMID: 15015432.

2967. Anonymous. Medication safety guidelines offer minimum and best practice standards. Performance Improvement Advisor. 2005;9(3):33-5, 25. PubMed PMID: 15881712.

2968. Anonymous. Hospitalists, pharmacists partner to cut errors. Hospital Case Management. 2005;13(3):39-40. PubMed PMID: 15754605.

2969. Anonymous. Pharmacist in ED slashes medication errors by 50%. ED Management. 2007;19(3):29-30. PubMed PMID: 19848031.

2970. Anonymous. Experts discuss pain management issues. Healthcare Benchmarks & Quality Improvement. 2009;16(3):32-4. PubMed PMID: 19239077.

2971. Anonymous. Pain management model has safety advantages. Healthcare Benchmarks & Quality Improvement. 2009;16(3):30-2. PubMed PMID: 19239076.

2972. Anonymous. Pharmacists conduct med rec at admission. Healthcare Benchmarks & Quality Improvement. 2010;17(6):70-2. PubMed PMID: 20491202.

2973. Anonymous. Best drugstores: 43,739 readers rate pharmacies for accuracy, knowledge, and speed.

Consumer Reports. 2011;76(5):24-9. PubMed PMID: 21510434.

2974. Anonymous. Clinical pharmacists with EM training slash medication errors, help to optimize therapies in the ED. ED Management. 2012;24(2):13-6. PubMed PMID: 22413733.

2975. Anonymous. A new opportunity for pharmacists: accountable care organizations. Consultant Pharmacist. 2014;29(9):570. PubMed PMID: 25203406.

2976. Anonymous. ED-based pharmacists make a big dent in medication errors. ED Management. 2014;26(8):91-4. PubMed PMID: 25076611.

2977. Anonymous. Pharmacists see patients through discharge. Hospital Case Management. 2014;22(2):16-7. PubMed PMID: 24505833.

2978. Anonymous. Making pharmacists part of the multidisciplinary team. Hospital Case Management. 2014;22(2):13-6. PubMed PMID: 24505832.

2979. Anonymous. CPNP 2015 Annual Meeting. Journal of pharmacy practice Conference: 18th annual meeting of the college of psychiatric and neurologic pharmacists, CPNP United states. 2015;28(3) (no pagination). PubMed PMID: CN-01371980.

2980. Anonymous. NCPDP recommendations for dose accumulation monitoring in the inpatient setting: Acetaminophen case model, version 1.0. American Journal of Health-System Pharmacy. 2016;73(15):1144-65. PubMed PMID: 27267535.

2981. Anonymous. Electronic prescribing: the risk of errors and adverse effects. Prescrire International. 2016;25(167):24-7. PubMed PMID: 26942261.

2982. Anonymous. Erratum: linifanib Versus Sorafenib in Patients With Advanced Hepatocellular Carcinoma: results of a Randomized Phase III Trial (Journal of Clinical Oncology (2015) 33 (172-179) DOI: 10.1200/JCO.2013.54.3298). Journal of clinical oncology. 2017;35(22):2590. doi: 10.1200/JCO.2017.74.5729. PubMed PMID: CN-01602791.

2983. Anonymous. Pharmacist Role in Transitions of Care. Consultant Pharmacist. 2017;32(10):645-9. PubMed PMID: 28992825.

2984. Anonymous. Neda Leonard: Filling a Void. Consultant Pharmacist. 2017;32(5):256-7. PubMed PMID: 28483005.

2985. Anonymous. Impact of Pharmacist-Driven Heart Failure in-Home Counseling on 30-Day Readmission Rates. Professional Case Management. 2019;24(4):E3-E4. PubMed PMID: 31145239.

2986. Anonymous. ASHP long-range vision for the pharmacy workforce in hospitals and health systems. American Journal of Health-System Pharmacy. 2020;77(5):386-400. PubMed PMID: 31799998.

2987. Anonymous. NCPDP recommendations for standardizing dosing in metric units (mL) on prescription container labels of oral liquid medications, version 2.0. American Journal of Health-System Pharmacy. 2021;78(7):578-605. PubMed PMID: 33647100.

2988. Anonymous. Information about undesirable effects. Nederlands Tijdschrift voor Geneeskunde. 2023;2023(51-52). PubMed PMID: 2029057233.

2989. Anonymous. Congratulations ARMIN! Deutsche Apotheker Zeitung. 2023;2023(21). PubMed PMID:

2029041270.

2990. Anonymous. Win customers for medication analysis. Deutsche Apotheker Zeitung. 2023;2023(19). PubMed PMID: 2029032599.

2991. Anonymous. ARMIN is a win for everyone involved. Deutsche Apotheker Zeitung. 2023;2023(16). PubMed PMID: 2029032237.

2992. Anonymous. Abstracts from The Royal Pharmaceutical Society Annual Conference. International Journal of Pharmacy Practice. 2023;31(Supplement 2). PubMed PMID: 643188390.

2993. Anonymous. Canadian Pharmacy Conference 2022. Canadian Pharmacists Journal. 2023;156(5). PubMed PMID: 642577101.

2994. Anonymous. Beyond 'safety of supply': a response to Harry McQuillan's lecture. Pharmaceutical Journal. 2023;311(7975). doi: https://dx.doi.org/10.1211/PJ.2023.1.192552. PubMed PMID: 2027241919.

2995. Anonymous. FIP Pharmacy Practice Research Summer Meeting 2023. Pharmacy Education. 2023;23(5). PubMed PMID: 642369375.

2996. Anonymous. 27th EAHP Congress. European Journal of Hospital Pharmacy. 2023;30(Supplement 1). PubMed PMID: 640966608.

2997. Anosike C, Adibe MO, Isah A, Ukoha-Kalu OB. Willingness to pay for pharmacist-provided home telemonitoring among patients with chronic diseases in Enugu metropolis. Health Informatics Journal. 2020;26(2):829-40. doi: 10.1177/1460458219852534. PubMed PMID: WOS:000549435300009.

2998. Anosike C, Chu-Madu CN, Igboeli NU, Adibe MO, Ukwe CV. The participation of community pharmacists in the case management of malaria in Enugu metropolis, Nigeria: a cross-sectional survey. Malawi Medical Journal. 2020;32(4):218-25. doi: 10.4314/mmj.v32i4.7. PubMed PMID: WOS:000618842600006.

2999. Anrys P, Petit A-E, Thevelin S, Sallevelt B, Drenth C, Soiza RL, et al. An International Consensus List of Potentially Clinically Significant Drug-Drug Interactions in Older People. Journal of the American Medical Directors Association. 2021;22(10):2121-33.e24. doi: https://doi.org/10.1016/j.jamda.2021.03.019.

3000. Anrys P, Strauven G, Boland B, Dalleur O, Declercq A, Degryse J-M, et al. Collaborative approach to Optimise MEdication use for Older people in Nursing homes (COME-ON): study protocol of a cluster controlled trial. Implementation Science. 2016;11(1):1-11. doi: 10.1186/s13012-016-0394-6. PubMed PMID: 113735343. Language: English. Entry Date: 20180731. Revision Date: 20220819. Publication Type: journal article.

3001. Anrys P, Strauven G, Boland B, Dalleur O, Declercq A, Degryse JM, et al. Collaborative approach to Optimise MEdication use for Older people in Nursing homes (COME-ON): study protocol of a cluster controlled trial. Implementation science. 2016;11:35. doi: 10.1186/s13012-016-0394-6. PubMed PMID: CN-01200210.

3002. Anrys P, Strauven G, Roussel S, Vande Ginste M, De Lepeleire J, Foulon V, et al. Process evaluation of a complex intervention to optimize quality of prescribing in nursing homes (COME-ON study). Implementation science. 2019;14(1):104. doi: 10.1186/s13012-019-0945-8. PubMed PMID: CN-02093175.

3003. Anrys P, Strauven G, Spinewine A, Foulon V. The optimization of medication use of Belgian nursing home residents based on a multidisciplinary collaboration (the Come-On study): study protocol. International

journal of clinical pharmacy. 2015;37(2):418. doi: 10.1007/s11096-015-0080-9. PubMed PMID: CN-01130694.

3004. Ansah D, Weaver J, Munoz B, Bloch EM, Coles CL, Lietman T, et al. A Cross-Sectional Study of the Availability of Azithromycin in Local Pharmacies and Associated Antibiotic Resistance in Communities in Kilosa District, Tanzania. American Journal of Tropical Medicine and Hygiene. 2019;100(5):1105-9. doi: 10.4269/ajtmh.18-0747. PubMed PMID: WOS:000476671900019.

3005. Ansah EK, Gyapong M, Narh-Bana S, Bart-Plange C, Whitty CJM. Factors influencing choice of care-seeking for acute fever comparing private chemical shops with health centres and hospitals in Ghana: A study using case-control methodology. Malaria Journal. 2016;15(1). doi: 10.1186/s12936-016-1351-1.

3006. Ansah EK, Narh-Bana S, Affran-Bonful H, Bart-Plange C, Cundill B, Gyapong M, et al. The impact of providing rapid diagnostic malaria tests on fever management in the private retail sector in Ghana: a cluster randomized trial. BMJ (Clinical research ed). 2015;350:h1019. doi: 10.1136/bmj.h1019. PubMed PMID: CN-01110298.

3007. Ansah EK, Narh-Bana S, Affran-Bonful H, Bart-Plange C, Cundill B, Gyapong M, et al. The impact of providing rapid diagnostic malaria tests on fever management in the private retail sector in Ghana: a cluster randomized trial. BMJ (online). 2015;350. doi: 10.1136/bmj.h1019. PubMed PMID: CN-01068754.

3008. Ansah EK, Whitty CJM, Bart-Plange C, Gyapong M. Changes in the availability and affordability of subsidised artemisinin combination therapy in the private drug retail sector in rural Ghana: Before and after the introduction of the AMFm subsidy. International Health. 2016;8(6):427-32. doi: 10.1093/inthealth/ihw041.

3009. Ansari F, Gray K, Nathwani D, Phillips G, Ogston S, Ramsay C, et al. Outcomes of an intervention to improve hospital antibiotic prescribing: interrupted time series with segmented regression analysis. Journal of antimicrobial chemotherapy. 2003;52(5):842-8. PubMed PMID: CN-00597841.

3010. Ansari J. Drug interaction and pharmacist. Journal of young pharmacists. 2010;2(3):326-31.

3011. Ansari JA. Drug Interaction and Pharmacist. Journal of Young Pharmacists. 2010;2(3):326-31. doi: https://doi.org/10.4103/0975-1483.66807.

3012. Ansari M. Evaluation of community pharmacies regarding dispensing practices of antibiotics in two districts of central Nepal. PLoS One. 2017;12(9):e0183907.

3013. Ansari M. Chapter 2 - Sociobehavioral Aspects of Medicines Use in Developing Countries. In: Ibrahim MIM, Wertheimer AI, Babar Z-U-D, editors. Social and Administrative Aspects of Pharmacy in Low- and Middle-Income Countries: Academic Press; 2018. p. 15-33.

3014. Ansari M, Alam K. Pharmacy Practice in Nepal. Pharmacy Practice in Developing Countries: Achievements and Challenges2016. p. 147-68.

3015. Ansari M, Alanazi A, Moin A. Consumers' awareness, attitude and associated factors towards self-medication in Hail, Saudi Arabia. Plos One. 2020;15(4). doi: 10.1371/journal.pone.0232322. PubMed PMID: WOS:000536661300052.

3016. Ansari M, Neupane D. Study on determination of errors in prescription writing: A semi-electronic perspective. Kathmandu University Medical Journal. 2009;7(3):238-41.

3017. Ansari MS, Nunia SK, Bansal A, Singh P, Sekhon V, Singh D, et al. Bladder contractility index in

posterior urethral valve: A new marker for early prediction of progression to renal failure. Journal of Pediatric Urology. 2018;14(2). doi: 10.1016/j.jpurol.2017.09.029. PubMed PMID: WOS:000433000600026.

3018. Anselmo AC, Mitragotri S. An overview of clinical and commercial impact of drug delivery systems. Journal of Controlled Release. 2014;190:15-28.

3019. Anshari M, Almunawar M, Low P, Al-Mudimigh A. Empowering clients through e-health in healthcare services: case Brunei. International quarterly of community health education. 2013;33(2):189-219.

3020. Anson A, Ramay B, de Esparza AR, Bero L. Availability, prices and affordability of the World Health Organization's essential medicines for children in Guatemala. Globalization and Health. 2012;8. doi: 10.1186/1744-8603-8-22. PubMed PMID: WOS:000311836100001.

3021. Anson C, Zhong HMJ, Wilby KJ. Advancing the conversation: A review of scholarly activity for curricular interventions for sexually- and gender-diverse patients in pharmacy education. Currents in Pharmacy Teaching and Learning. 2021;13(12):1718-23. doi: https://doi.org/10.1016/j.cptl.2021.09.045.

3022. Antaky E, Lalonde L, Schnitzer ME, Martin É, Berbiche D, Perreault S, et al. Identifying heavy health care users among primary care patients with chronic non-cancer pain. Canadian Journal of Pain. 2017;1(1):22-36. doi: 10.1080/24740527.2017.1326088.

3023. Antepohl W, Herzig S. Problem-based learning versus lecture-based learning in a course of basic pharmacology: a controlled, randomized study. Medical education. 1999;33(2):106-13. doi: 10.1046/j.1365-2923.1999.00289.x. PubMed PMID: CN-00162223.

3024. Anthenelli RM, Heffner JL, Wong E, Tibbs J, Russell K, Isgro M, et al. A Randomized Trial Evaluating Whether Topiramate Aids Smoking Cessation and Prevents Alcohol Relapse in Recovering Alcohol-Dependent Men. Alcoholism, clinical and experimental research. 2017;41(1):197-206. doi: 10.1111/acer.13279. PubMed PMID: CN-01300152.

3025. Anthierens S, Grypdonck M, De Pauw L, Christiaens T. Perceptions of nurses in nursing homes on the usage of benzodiazepines. Journal of clinical nursing. 2009;18(22):3098-106.

3026. Anthony BF, Surgey A, Hiscock J, Williams NH, Charles JM. General medical services by non-medical health professionals: A systematic quantitative review of economic evaluations in primary care. British Journal of General Practice. 2019;69(682):E304-E13. doi: 10.3399/bjgp19X702425.

3027. Anthony C. Reducing medication errors. JAMA. 2001;286(17):2091-2; author reply 2. PubMed PMID: 11694136.

3028. Antman E, Weiss S, Loscalzo J. Systems pharmacology, pharmacogenetics, and clinical trial design in network medicine. Wiley Interdisciplinary Reviews: Systems Biology and Medicine. 2012;4(4):367-83.

3029. Anto B, Barlow D, Oborne A, Cape A, Vlassoff A, Whittlesea C. Dispensing-label errors in hospital: types and potential causes. International Journal of Pharmacy Practice. 2010;18(2):122-4. PubMed PMID: 20441122.

3030. Anto B, Barlow D, Oborne CA, Whittlesea C. Incorrect drug selection at the point of dispensing: a study of potential predisposing factors. International Journal of Pharmacy Practice. 2011;19(1):51-60.

3031. Anto B, James KL, Barlow D, Brinklow N, Oborne CA, Whittlesea C. Exploratory study to identify the process used by pharmacy staff to verify the accuracy of dispensed medicines. International Journal of Pharmacy Practice. 2013;21(4):233-42. PubMed PMID: 23419159.

3032. Anto BP. Dispensing errors in hospital : potential causes of label and drug specification errors and evaluation of final dispensing accuracy check process [Ph.D.]. England: University of London, King's College (United Kingdom); 2010.

3033. Antofie I, Iorga M, Pop LM, Jaradat-Hadi M. EXPLORING THE OPINION ABOUT JOB SATISFACTION, LIFELONG LEARNING OPORTUNITIES AND INSTITUTIONAL SUPPORT AMONG ROMANIAN PHARMACISTS. Farmacia. 2023;71(2):419-27. doi: 10.31925/farmacia.2023.2.24. PubMed PMID: WOS:000982169600019.

3034. Antoine S, Glewis S, Alexander M, Lingaratnam MS, Cherie C, Luetsch K. Cancer patients' perspectives on participating in a community pharmacy-based hyperglycaemia screening service – A qualitative exploration of enablers and barriers. Research in Social and Administrative Pharmacy. 2021;17(3):613-8. doi: https://doi.org/10.1016/j.sapharm.2020.05.023.

3035. Antonelli RC, Antonelli DM. Providing a medical home: the cost of care coordination services in a community-based, general pediatric practice. Pediatrics. 2004;113(Supplement_4):1522-8.

3036. Antonenko O, Puldová K, Halačová M, Grešáková S, Brkic J, Fialová D. PRESCRIBING OF HYPNOSEDATIVES IN SENIORS IN AMBULATORY CARE IN THE CZECH REPUBLIC: INOMED AND EUROAGEISM PROJECT FINDINGS...British Geriatrics Society Autumn Meeting, November 25-27 2020 (Virtual). Age & Ageing. 2021;50:i1-i. doi: 10.1093/ageing/afab030.58. PubMed PMID: 149478780. Language: English. Entry Date: 20210329. Revision Date: 20220302. Publication Type: Article.

3037. Antoni MH, Carrico AW, Durán RE, Spitzer S, Penedo F, Ironson G, et al. Randomized clinical trial of cognitive behavioral stress management on human immunodeficiency virus viral load in gay men treated with highly active antiretroviral therapy. Psychosomatic medicine. 2006;68(1):143-51. doi: 10.1097/01.psy.0000195749.60049.63. PubMed PMID: CN-00562403.

3038. Antoniello AA, Pauls P, Awad NI, Sobolewski K, Fernández D, Bridgeman P. Optimization of antidote stocking, availability, and administration practices for a large multihospital organization. American Journal of Health-System Pharmacy. 2023;80:S1-S10. doi: 10.1093/ajhp/zxac191. PubMed PMID: 162060438.

3039. Antoniou T, McCormack D, Campbell T, Sutradhar R, Tadrous M, Lum-Wilson N, et al. Geographic variation in the provision of naloxone by pharmacies in Ontario, Canada: A population-based small area variation analysis. Drug and Alcohol Dependence. 2020;216:108238. doi: https://doi.org/10.1016/j.drugalcdep.2020.108238.

3040. Antoniou T, Men S, Tadrous M, Leece P, Munro C, Gomes T. Impact of a publicly funded pharmacy-dispensed naloxone program on fatal opioid overdose rates: A population-based study. Drug and Alcohol Dependence. 2022;236:109473. doi: https://doi.org/10.1016/j.drugalcdep.2022.109473.

3041. Antoniou T, Pritlove C, Shearer D, Martins D, Tadrous M, Munro C, et al. A qualitative study of a publicly funded pharmacy-dispensed naloxone program. International Journal of Drug Policy. 2021;92:103146. doi: https://doi.org/10.1016/j.drugpo.2021.103146.

3042. Antoon JW, Feinstein JA, Goldman JL, Kyler KE, Shah SS, Hall M, et al. Advancing pediatric medication safety using real-world data: Current problems and potential solutions. Journal of Hospital Medicine. 2023;18(9):865-9. doi: https://dx.doi.org/10.1002/jhm.13068. PubMed PMID: 2021892905.

3043. Antrim A. Benchmark does not represent retail prices, research finds. Pharmacy Times. 2020;88(9).

3044. Antunes LP, Gomes JJ, Cavaco AM. How pharmacist-patient communication determines pharmacy loyalty? Modeling relevant factors. Research in Social & Administrative Pharmacy. 2015;11(4):560-70. doi:

10.1016/j.sapharm.2014.11.003. PubMed PMID: WOS:000355339200007.

3045. Antunes MN. Anticancer Activity of Ginger Professional Experience in Research and Community Pharmacy2023.

3046. Anuwong W. Community pharmacist-child medication communication: Magnitude, influences, content, and determinants of the pharmacist's decision to communicate with children [Ph.D.]. United States -- Indiana: Purdue University; 2000.

3047. Anwar I, Minimol K, Narasimhaiah M. Self-Medication Practices among Medical and Non-Medical Students. Journal of Evolution of Medical and Dental Sciences-Jemds. 2020;9(40):2976-80. doi: 10.14260/jemds/2020/652. PubMed PMID: WOS:000578051900009.

3048. Anyakora C, Oni Y, Ezedinachi U, Adekoya A, Ali I, Nwachukwu C, et al. Quality medicines in maternal health: Results of oxytocin, misoprostol, magnesium sulfate and calcium gluconate quality audits. BMC Pregnancy and Childbirth. 2018;18(1). doi: 10.1186/s12884-018-1671-y.

3049. Anyanwu JC. Demand for health care institutions' services: Evidence from malaria fever treatment in Nigeria. African Development Review-Revue Africaine De Developpement. 2007;19(2):304-34. doi: 10.1111/j.1467-8268.2007.00163.x. PubMed PMID: WOS:000250434900003.

3050. Anyira CIE. Preservation of Medical information in Healthcare facilities in Delta State, Nigeria: a Survey. Library Philosophy and Practice. 2020;2020:1-14.

3051. Anyolitho MK, Nyakato VN, Huyse T, Poels K, Masquillier C. Health-seeking behaviour regarding schistosomiasis treatment in the absence of a mass drug administration (MDA) program: the case of endemic communities along Lake Albert in Western Uganda. Bmc Public Health. 2023;23(1). doi: 10.1186/s12889-023-16020-z. PubMed PMID: WOS:001000971100016.

3052. Anzenberger P, Popov SB, Ostermann H. Factors that motivate young pharmacists to work in rural communities in the Ukraine. Rural and Remote Health. 2011;11(4).

3053. Ao X, Zhou T, Jiang M, Han G, He W, Li H. Analysis of the Curative Effects of DP, Gefitinib Combined with Endostar in the Treatment of Non-small Cell Lung Cancer. Anti-tumor pharmacy. 2017;7(3):350-4. doi: 10.3969/j.issn.2095-1264.2017.03.20. PubMed PMID: CN-01406007.

3054. Aoki T, Matsushima M. The Ecology of Medical Care During the COVID-19 Pandemic in Japan: a Nationwide Survey. Journal of General Internal Medicine. 2022;37(5):1211-7. doi: 10.1007/s11606-022-07422-7. PubMed PMID: WOS:000752205000004.

3055. Aoki Y, Yaju Y, Utsumi T, Sanyaolu L, Storm M, Takaesu Y, et al. Shared decision-making interventions for people with mental health conditions. Cochrane Database of Systematic Reviews. 2022;(11). doi: 10.1002/14651858.CD007297.pub3. PubMed PMID: CD007297.

3056. Aouifi A, Charbonnel JF, Lapeyre E, Frebault V, Pasquié M, Martel A, et al. First open label study to investigate the use, safety and tolerability of octaplaslg® in patient blood management in two cardiac surgery centres in France. Journal of Cardiothoracic and Vascular Anesthesia. 2020;34:S44-S5. doi: https://doi.org/10.1053/j.jvca.2020.09.062.

3057. Aoun MH, Ben Soussia R, Brahim S, Betbout I, Bouali W, Hadj-Mohamed A, et al. Pandémie COVID-19 : impact psychosocial sur le personnel de santé en Tunisie. L'Encéphale. 2023;49(1):27-33. doi: https://doi.org/10.1016/j.encep.2021.08.012.

3058. Aoun MH, Ben Soussia R, Brahim S, Betbout I, Bouali W, Hadj-Mohamed A, et al. Pandémie COVID-19 : impact psychosocial sur le personnel de santé en Tunisie. L'Encéphale. 2023;49(1):27-33. doi: https://doi.org/10.1016/j.encep.2021.08.012.

3059. Aparasu R, Baer R, Aparasu A. Clinically important potential drug-drug interactions in outpatient settings. Research in Social and Administrative Pharmacy. 2007;3(4):426-37.

3060. Aparasu RR, Mort JR, Brandt H. Psychotropic prescription use by community-dwelling elderly in the United States. Journal of the American Geriatrics Society. 2003;51(5):671-7.

3061. Aparecida Reis D, da Silva Winter J, Milani Martins VL, Pilger D. CONCILIAÇÃO MEDICAMENTOSA NA ADMISSÃO DE PACIENTES TRANSPLANTADOS RENAIS EM UM HOSPITAL UNIVERSITÁRIO. MEDICATION RECONCILIATION IN THE ADMISSION OF KIDNEY TRANSPLANT PATIENTS TO A UNIVERSITY HOSPITAL. 2023;43(1):30-8. doi: 10.22491/2357-9730.122368. PubMed PMID: 164674904.

3062. Aparicio Jabalquinto G, Castro Toro J, Rodríguez Escolar C, Iglesias García J, Gutiérrez Muñoz L. Coordinación de servicios sociosanitarios para la mejora de la prescripción y dispensación farmacológica en una residencia de ancianos. Revista Española de Geriatría y Gerontología. 2007;42(6):333-41. doi: https://doi.org/10.1016/S0211-139X(07)73572-2.

3063. Aparna Y, Aruru M, Mohammed Z, Pabba D, Akkanolla R, Duggaraju V, et al. Assessment of Community Pharmacists' Knowledge and Role in Promoting Rational Use of Antibiotics in Southern India. JACCP journal of the american college of clinical pharmacy. 2019;2(5):576-7. doi: 10.1002/jac5.1154. PubMed PMID: CN-02260473.

3064. Apassa MA. Development and Evaluation of a Medication Adherence Program in an Outpatient Mental Health Clinic [D.N.P.]. United States -- Delaware: Wilmington University (Delaware); 2021.

3065. Apeland SG. [Lack of safety in medicine supply]. Tidsskrift for den Norske lægeforening : tidsskrift for praktisk medicin, ny række. 2012;132(9):1062.

3066. Apidi NA, Murugiah MK, Muthuveloo R, Soh YC, Caruso V, Patel R, et al. Mobile medical applications for dosage recommendation, drug adverse reaction, and drug interaction: review and comparison. Therapeutic innovation & regulatory science. 2017;51(4):480-5.

3067. Apikoglu S, Selcuk A, Ozcan V, Balta E, Turker M, Albayrak OD, et al. The first nationwide implementation of pharmaceutical care practices through a continuing professional development approach for community pharmacists. International Journal of Clinical Pharmacy. 2022;44(6):1223-31. doi: 10.1007/s11096-022-01413-8.

3068. Apikoglu S, Selcuk A, Ozcan V, Balta E, Turker M, Albayrak OD, et al. The first nationwide implementation of pharmaceutical care practices through a continuous professional development approach for community pharmacists. International Journal of Clinical Pharmacy. doi: 10.1007/s11096-022-01413-8. PubMed PMID: WOS:000810829900001.

3069. Apikoglu-Rabus S, Kahya I, Serin M, Yildiz P. Assessment of attitudes and practice of Turkish community pharmacy staff regarding skin problems using virtual patient profiles. International Journal of Clinical Pharmacy. 2019;41(1):320-. PubMed PMID: WOS:000458652000117.

3070. Apikoglu-Rabus S, Ozcan V, Balta E, Turker M, Uney A. SMART pharmacy program: guiding the nationwide implementation of pharmaceutical care practices-preliminary results of the asthma/COPD module. International journal of clinical pharmacy Conference: 46th ESCP symposium on clinical pharmacy

Germany. 2018;40(1):205. doi: 10.1007/s11096-017-0565-9. PubMed PMID: CN-01468271.

3071. Apikoglu-Rabus S, Sancar M, Okuyan B, Izzettin FV. Turkish pharmacy technicians' counseling practices and attitudes regarding emergency contraceptive pills. African Journal of Pharmacy and Pharmacology. 2011;5(1):60-6. doi: 10.5897/ajpp10.192.

3072. Apikoglu-Rabus S, Yesilyaprak G, Izzettin FV. Drug-related problems and pharmacist interventions in a cohort of patients with asthma and chronic obstructive pulmonary disease. Respiratory Medicine. 2016;120:109-15. doi: https://doi.org/10.1016/j.rmed.2016.10.006.

3073. Apisarnthanarak A, Lapcharoen P, Vanichkul P, Srisaeng-Ngoen T, Mundy LM. Design and analysis of a pharmacist-enhanced antimicrobial stewardship program in Thailand. American Journal of Infection Control. 2015;43(9):956-9. doi: 10.1016/j.ajic.2015.05.011. PubMed PMID: 109835381. Language: English. Entry Date: 20150911. Revision Date: 20200708. Publication Type: Journal Article.

3074. Apkon M, Leonard J, Probst L, DeLizio L, Vitale R. Design of a safer approach to intravenous drug infusions: failure mode effects analysis. Quality & Safety in Health Care. 2004:265-71. doi: 10.1136/qshc.2003.007443. PubMed PMID: 106600910. Language: English. Entry Date: 20050401. Revision Date: 20200708. Publication Type: Journal Article.

3075. Apkon M, Mattera JA, Lin Z, Herrin J, Bradley EH, Carbone M, et al. A randomized outpatient trial of a decision-support information technology tool. Archives of internal medicine. 2005;165(20):2388-94. doi: 10.1001/archinte.165.20.2388. PubMed PMID: CN-00531948.

3076. Apkon M, Mattera JA, Lin ZQ, Herrin J, Bradley EH, Carbone M, et al. A randomized outpatient trial of a decision-support information technology tool. Archives of Internal Medicine. 2005;165(20):2388-94. doi: 10.1001/archinte.165.20.2388. PubMed PMID: WOS:000233251900010.

3077. Aponte-González J, Brown P, Eslava-Schmalbach J. Preferences based interventions to address the use of antibiotics without prescription: A discrete choice experiment. Pharmacy Practice. 2021;19(3). doi: 10.18549/PHARMPRACT.2021.3.2401.

3078. Aponte-González J, González-Acuña A, Lopez J, Brown P, Eslava-Schmalbach J. Perceptions in the community about the use of antibiotics without a prescription: Exploring ideas behind this practice. Pharmacy Practice. 2019;17(1). doi: 10.18549/PharmPract.2019.1.1394.

3079. Apoola A, Brunt L. A randomised controlled study of mouth swab testing versus same day blood tests for HIV infection in young people attending a community drug service. Drug and alcohol review. 2011;30(1):101-3. doi: 10.1111/j.1465-3362.2010.00208.x. PubMed PMID: CN-00784990.

3080. Apoorva Dev M, Kishore G. Awareness of breast cancer and breast self-examination among female pharmacy professionals in Bengaluru. International Journal of Pharmaceutical Research. 2020;13(1):881-6. doi: 10.31838/ijpr/2021.13.01.087.

3081. Appalasamy JR, Sariff A. A survey of drug-related-problems among patients treated for allergy symptoms in community pharmacies at Negeri Sembilan, Malaysia. International Journal of Pharmacy and Pharmaceutical Sciences. 2014;6(10):313-6.

3082. Appaneal HJ, Caffrey AR, Lopes V, Dosa D, LaPlante KL. Antibiotic Prescribing in Outpatient Settings: Rural Patients Are More Likely to Receive Fluoroquinolones and Longer Antibiotic Courses. Antibiotics. 2023;12(2):224.

3083. Appaneal HJ, Lesniak K, LaPlante KL, Feret B. Student pharmacists delivering academic detailing on

adult pneumococcal vaccination to community pharmacists. Journal of the American Pharmacists Association. 2023;63(4):1064-9.e2. doi: 10.1016/j.japh.2023.04.003.

3084. Appari A, Johnson ME. Information security and privacy in healthcare: current state of research. International journal of Internet and enterprise management. 2010;6(4):279-314.

3085. Apple FS, Chung AY, Kogut ME, Bubany S, Murakami MM. Decreased patient charges following implementation of point-of-care cardiac troponin monitoring in acute coronary syndrome patients in a community hospital cardiology unit. Clinica Chimica Acta. 2006;370(1-2):191-5. doi: 10.1016/j.cca.2006.02.011.

3086. Appleford G, Mumbia D, Emmart P. Incentives and disincentives to private sector reporting on family planning in Kenya: why these matter, and how they may change over time. Gates Open Research. 2023;6. doi: 10.12688/gatesopenres.13909.2.

3087. Aprahamian I, Borges MK, Hanssen DJC, Jeuring HW, Voshaar RCO. The Frail Depressed Patient: A Narrative Review on Treatment. Clinical Interventions in Aging. 2022;17:979-90. doi: 10.2147/cia.S328432. PubMed PMID: WOS:000817163100001.

3088. Apriyanto D, Setiawati MCN. Prescription of medicine for outpatients of gynaecology obstetric poly at a private hospital in Semarang, Indonesia. Pharmacy Education. 2022;22(2):184-7. doi: 10.46542/pe.2022.222.184187.

3089. Aquino RP, Barile S, Grasso A, Saviano M. Envisioning smart and sustainable healthcare: 3D Printing technologies for personalized medication. Futures. 2018;103:35-50. doi: https://doi.org/10.1016/j.futures.2018.03.002.

3090. Ara S. A literature review of cardiovascular disease management programs in managed care populations. Journal of managed care pharmacy. 2004;10(4):326-44. doi: 10.18553/jmcp.2004.10.4.326. PubMed PMID: CN-01714824.

3091. Arab OO, Alshaya OA, Almeleebia TM, Shorog EM, Alzuman S, Alqahtani A, et al. Current developments of ambulatory pharmaceutical care in Saudi Arabia. Journal of the American College of Clinical Pharmacy. 2023;6(4):363-9. doi: 10.1002/jac5.1755. PubMed PMID: WOS:000916701700001.

3092. Arabkhazaeli A, Vijverberg SJH, van der Ent CK, Raaijmakers JAM, Maitland-van der Zee AH. High incidence of oral corticosteroids prescriptions in children with asthma in early childhood. Journal of Asthma. 2016;53(10):1012-7. doi: 10.1080/02770903.2016.1185439.

3093. Arabkhazaeli A, Vijverberg SJH, van der Lee M, van der Ent CK, Bruijnzeel-Koomen CA, de Bruin-Weller MS, et al. Patterns of topical corticosteroids prescriptions in children with asthma. Pediatric Dermatology. 2018;35(3):378-83. doi: 10.1111/pde.13455.

3094. Arabyat RM, Martinez AS, Nusair MB. Adverse drug event reporting by pharmacists: a systematic literature review. Journal of Pharmaceutical Health Services Research. 2020;11(1):5-23. doi: 10.1111/jphs.12332. PubMed PMID: WOS:000501725600001.

3095. Arabyat RM, Raisch DW. PHP78 - Factors That Impact Reporting Of Adverse Drug Events By Pharmacists: A Systematic Literature Review. Value in Health. 2014;17(3):A23. doi: https://doi.org/10.1016/j.jval.2014.03.142.

3096. Arabyat RM, Raisch DW, Bakhireva L. Influenza vaccination for patients with chronic obstructive pulmonary disease: Implications for pharmacists. Research in Social and Administrative Pharmacy.

2018;14(2):162-9. doi: https://doi.org/10.1016/j.sapharm.2017.02.010.

3097. Arabyat RM, Sanchez Martinez A, Nusair MB. Adverse drug event reporting by pharmacists: a systematic literature review. Journal of Pharmaceutical Health Services Research. 2020;11(1):5-23. doi: 10.1111/jphs.12332.

3098. Aradhya PJ, Ravi R, Chandra BJS, Ramesh M, Chalasani SH. Assessment of Medication Safety Incidents Associated with High-alert Medication Use in Intensive Care Setting: A Clinical Pharmacist Approach. Indian Journal of Critical Care Medicine. 2023;27(12):917-22. doi: 10.5005/jp-journals-10071-24588. PubMed PMID: 175434026.

3099. Aradottir HAE, Kinnear M. Design of an algorithm to support community pharmacy dyspepsia management. Pharmacy World & Science. 2008;30(5):515-25. doi: 10.1007/s11096-008-9201-z. PubMed PMID: WOS:000259443000005.

3100. Aragon BR, Pierce RA, Jones WN. VA CMOPs: Producing a pattern of quality and efficiency in government. Journal of the American Pharmacists Association. 2012;52(6):810-5. doi: 10.1331/JAPhA.2012.11075. PubMed PMID: WOS:000314401500034.

3101. Aragon-Herrera A, Feijoo-Bandin S, Santiago MO, Barral L, Campos-Toimil M, Gil-Longo J, et al. Empagliflozin reduces the levels of CD36 and cardiotoxic lipids while improving autophagy in the hearts of Zucker diabetic fatty rats. Biochemical Pharmacology. 2019;170:113677.

3102. Aragona SE, Ciprandi G. Gastric reflux management in pharmacy. Journal of Biological Regulators and Homeostatic Agents. 2022;36(2):41-5. PubMed PMID: WOS:000822960600001.

3103. Arai A, Takahashi Y, Nakamura A, Tanabe M. Public Awareness and Educational Activities to Improve Knowledge and Understanding of Antimicrobial Resistance (AMR) in Japan. American Journal of Infection Control. 2019;47(6, Supplement):S21-S2. doi: https://doi.org/10.1016/j.ajic.2019.04.034.

3104. Arain S, Thalapparambath R, Al Ghamdi FH. COVID-19 pandemic: Response plan by the Johns Hopkins Aramco Healthcare inpatient pharmacy department. Research in Social and Administrative Pharmacy. 2021;17(1):2009-11. doi: https://doi.org/10.1016/j.sapharm.2020.05.016.

3105. Arakawa N, Nomura K, Duggan C, Bates I. A report from the Japanese Society of Drug Informatics Forum: The role of pharmacists providing self-care. Pharmacy Education. 2015;15(1):182-8. PubMed PMID: WOS:000378481400032.

3106. Arakawa T, Ejima D, Li T, Philo JS. The critical role of mobile phase composition in size exclusion chromatography of protein pharmaceuticals. Journal of Pharmaceutical Sciences. 2010;99(4):1674-92. doi: https://doi.org/10.1002/jps.21974.

3107. Aramwit P, Assawawitoontip S. Evaluation of patient counseling on blood pressure control of out-patients with hypertension at Chulalongkorn Hospital. Chotmaihet thangphaet [Journal of the Medical Association of Thailand]. 2003;86 Suppl 2:S496-500. PubMed PMID: CN-00440081.

3108. Arancon-Monge JM, De-Castro-Cuenca A, Serrano-Vázquez Á, Campos-Díaz L, Díaz-Eraso M, Del Cura-González I, et al. Study CAMBIMED: Effects of changes in medication appearance on safety of antihypertensive and hypolipidemic treatments in chronic patients older than 65 years in primary health care. BMC Public Health. 2015;15(1). doi: 10.1186/s12889-014-1342-5.

3109. Arancon-Monge JM, de-Castro-Cuenca A, Serrano-Vazquez A, Campos-Diaz L, Diaz-Eraso M, del Cura-Gonzalez I, et al. Study CAMBIMED: Effects of changes in medication appearance on safety of

antihypertensive and hypolipidemic treatments in chronic patients older than 65 years in primary health care. Bmc Public Health. 2015;15. doi: 10.1186/s12889-014-1342-5. PubMed PMID: WOS:000350953600001.

3110. Araújo AC, Fernandes E, Ruivo IF, Machado MD, Vaz AF, Furtado C. Prevalence of Outpatient Use of Medicines by the Elderly Population in Portugal: A Cross-Sectional Study. Acta Medica Portuguesa. 2023;36(12):792-801. doi: 10.20344/amp.19254. PubMed PMID: WOS:001026867500001.

3111. Araujo JLO, Pereira MD, De Cássia Bergamaschi C, De Sá Del Fiol F, Lopes LC, De Toledo MI, et al. Access to medicines for diabetes treatment in Brazil: Evaluation of "health has no price" program. Diabetology and Metabolic Syndrome. 2016;8(1). doi: 10.1186/s13098-016-0150-8.

3112. Araujo JLO, Pereira MD, De Sá Del Fiol F, Barberato-Filho S. Access to antihypertensive agents in Brazil: Evaluation of the "health has no price" program. Clinical Therapeutics. 2014;36(8):1191-5. doi: 10.1016/j.clinthera.2014.06.003.

3113. Araujo L, Montagne M. Trade agreements and drug access: Assessment of the impact of the 2009 Peruvian new drug policy on anti-infectives registration and availability. Journal of Pharmaceutical Policy and Practice. 2018;11(1). doi: 10.1186/s40545-018-0151-0.

3114. Araujo LE. Effect of the 2009 US-Peru Free Trade Agreement on Peruvian new drug policies and the registration and quality of pharmaceutical products [Ph.D.]. United States -- Massachusetts: Massachusetts College of Pharmacy and Health Sciences; 2015.

3115. Aravind Krishnan U, Mathew R, Jesse I, Joseph S, Shyam Sundar P. A study on clinical pharmacist interventions in the management of sepsis in a tertiary care hospital. International journal of pharmaceutical sciences review and research. 2016;40(2):109-14. PubMed PMID: CN-01247973.

3116. Arbaeen A, Wheate NJ, Brown JA, Cairns R. Over-the-counter cough and cold medicines: reported poisonings of children before and after the 2012 and 2020 labelling changes in Australia. Medical Journal of Australia. 2023;218(9):410-1. doi: https://dx.doi.org/10.5694/mja2.51865. PubMed PMID: 2021705231.

3117. Arbanas D, Vrbanec J. Medication Errors in the Treatment of Pain in Cancer Patients. Libri Oncologici. 2022;50(Supplement 2):5. PubMed PMID: 638542718.

3118. Arca KN, Smith JH, Chiang CC, Starling AJ, Robertson CE, Singh RBH, et al. COVID-19 and Headache Medicine: A Narrative Review of Non-Steroidal Anti-Inflammatory Drug (NSAID) and Corticosteroid Use. Headache. 2020;60(8):1558-68. doi: 10.1111/head.13903. PubMed PMID: WOS:000546630400001.

3119. Arca MA. The professional biochemical practice in Latin America and Argentina. Acta Bioquimica Clinica Latinoamericana. 2008;42(3):339-59.

3120. Arcangelo VP, Peterson AM. Pharmacotherapeutics for advanced practice: a practical approach: Lippincott Williams & Wilkins; 2006.

3121. Arcas MJR. Hypercholesterolemia resulted from the usage of a contraceptive ring. It is detected in a community pharmacy, through a pharmaceutical care service. Pharmaceutical Care Espana. 2016;18(4):181-6. PubMed PMID: WOS:000382705500005.

3122. Arcas MJR. Pharmacovigilance of biological medicines in the community pharmacy: report of a case. Pharmaceutical Care Espana. 2019;21(2):134-41. PubMed PMID: WOS:000464569600004.

3123. Arcas MJR, Mata MC, Fernandez VL. Monitored Dosage System: the path to the safety and efficacy of

medication. Farmaceuticos Comunitarios. 2020;12(2):23-5. doi: 10.33620/FC.2173-9218.(2020/Vol12).002.04. PubMed PMID: WOS:000532767200004.

3124. Archer DF, Merkatz RB, Bahamondes L, Westhoff CL, Darney P, Apter D, et al. Efficacy of the 1-year (13-cycle) segesterone acetate and ethinylestradiol contraceptive vaginal system: results of two multicentre, open-label, single-arm, phase 3 trials. The Lancet Global Health. 2019;7(8):e1054-e64. doi: 10.1016/S2214-109X(19)30265-7.

3125. Ardabili AR, van Esser D, Wintjens D, Cilissen M, Deben D, Mujagic Z, et al. Development and Validation of a Remote Monitoring Tool for Assessment of Mild, Moderate, and Severe Infections in Inflammatory Bowel Disease. Journal of Crohns & Colitis. 2023;17(7):1079-88. doi: 10.1093/ecco-jcc/jjad023. PubMed PMID: WOS:000963902100001.

3126. Ardavani A, Curtis F, Khunti K, Wilkinson TJ, Highton P. The need for standardised outcomes in pharmacist interventions in chronic kidney disease: a systematic review of randomised controlled trials. International Journal of Pharmacy Practice. 2023;31(Supplement 2):ii5-ii6. PubMed PMID: 643188337.

3127. Ardery G, Carter BL, Milchak JL, Bergus GR, Dawson JD, James PA, et al. Explicit and implicit evaluation of physician adherence to hypertension guidelines. The Journal of Clinical Hypertension. 2007;9(2):113-9.

3128. Arditti J, Bourdon JH, Spadari M, de Haro L, Richard N, Valli M. Ma- Huang, from dietary supplement to abuse. Acta Clinica Belgica. 2002;57:34-6. doi: 10.1179/acb.2002.074. PubMed PMID: WOS:000174603600009.

3129. Are A, Hauser R, Spencer R, Satterfield J, Nguyen E. States' pharmacist immunization authority and the impact on adult influenza vaccination rates. Journal of the American Pharmacists Association. 2022;62(5):1666-70.e1. doi: https://doi.org/10.1016/j.japh.2022.04.017.

3130. Áreas del Águila VL. Cost accounting: Use in a pharmacy service. Farmacia Hospitalaria. 2001;25(1):25-30.

3131. Areej J, Rabia J. Eliciting Time Motion study with involved pharmacist interventions in an emergency department of a Tertiary care hospital. Journal of Medical Sciences (Faisalabad). 2013;13(7):555-62. doi: 10.3923/jms.2013.555.562.

3132. Aref HAT, Makowsky MJ, Kung JY, Guirguis LM. Mapping the implementation of pharmacogenomic testing in community pharmacies 2003-2021 using the Theoretical Domains Framework: A scoping review. Journal of the American Pharmacists Association. 2023;63(2):459-76.e6. doi: 10.1016/j.japh.2022.09.019.

3133. Aref HAT, Witry M, Olufemi-Yusuf D, Guirguis LM. Ensuring quality qualitative research reporting in community pharmacy: a systematic literature review. International Journal of Pharmacy Practice. 2021;29(5):416-27. doi: 10.1093/ijpp/riab027. PubMed PMID: WOS:000736136400004.

3134. Aref HT, Makowsky MJ, Kung JY, Guirguis LM. Mapping the implementation of pharmacogenomic testing in community pharmacies 2002-2021 using the Theoretical Domains Framework: A scoping review. Journal of the American Pharmacists Association. 2022. doi: 10.1016/j.japh.2022.09.019.

3135. Aremu O, Lawoko S, Moradi T, Dalal K. Socio-economic determinants in selecting childhood diarrhoea treatment options in Sub-Saharan Africa: A multilevel model. Italian Journal of Pediatrics. 2011;37(1). doi: 10.1186/1824-7288-37-13.

3136. Arenas Villafranca JJ, GÓMez SÁNchez A, Nieto Guindo M, Faus Felipe V. Using failure mode and

effects analysis to improve the safety of neonatal parenteral nutrition. American Journal of Health-System Pharmacy. 2014;71(14):1210-8. doi: 10.2146/ajhp130640. PubMed PMID: 103971743. Language: English. Entry Date: 20140711. Revision Date: 20200708. Publication Type: Journal Article.

3137. Arend RC, O'Malley DM, Banerjee S, McLaurin K, Davidson R, Long GH. Utilization of Poly(ADP-Ribose) Polymerase Inhibitors in Ovarian Cancer: A Retrospective Cohort Study of US Healthcare Claims Data. Advances in Therapy. 2022;39(1):328-45. doi: 10.1007/s12325-021-01959-5. PubMed PMID: WOS:000714008100002.

3138. Arendt DD, Boyle JA. When resiliency is not enough: Addressing the structure of a residency program and its contribution to pharmacy resident burnout. American Journal of Health-System Pharmacy. 2021;78(11):949-52. doi: 10.1093/ajhp/zxab067.

3139. Arenz BJ, Diez HL, Bostwick JR, Kales HC, Zivin K, Dalack GW, et al. Effectively implementing FDA medication alerts utilizing patient centered medical home clinical pharmacists. Healthcare. 2016;4(1):69-73. doi: https://doi.org/10.1016/j.hjdsi.2015.07.001.

3140. Argay M, Koós I, Takács I, Dormaeva I, Meskó A, Zelkó R, et al. Pharmaceutical care for patients with acute myocardial infarction in Hungary. International Journal of Clinical Pharmacology and Therapeutics. 2013;51(2):91-5. doi: 10.5414/CP201592.

3141. Argeg GM. The problems of translating medical terms from English into Arabic [Ph.D.]. England: University of Durham (United Kingdom); 2015.

3142. Argoff CE, Stanos SP, Wieman MS. Validity testing of patient objections to acceptance of tamper-resistant opioid formulations. Journal of Pain Research. 2013;6:367-73. doi: 10.2147/JPR.S37343.

3143. Argüelles RAF, Marante MC, Orozco DE, Michelena MAA. Validation of a proposal of survey on the regulation of pharmaceutical services in mexico. Revista Cubana de Farmacia. 2015;49(1).

3144. Ari M, Murray J, Dickson S, Kerins A, Weyer G. A Model to Improve Care and Enhance the Physician-Patient Relationship for Hospitalized Patients with Opioid Use Disorder. Academic Medicine. 2023;98(6):S25-S7. doi: 10.1097/ACM.0000000000005184.

3145. Ariaga A, Balzan D, Falzon S, Sultana J. A scoping review of legibility of hand-written prescriptions and drug-orders: the writing on the wall. Expert Review of Clinical Pharmacology. 2023;16(7):617-21. doi: 10.1080/17512433.2023.2223972. PubMed PMID: WOS:001019164200001.

3146. Arias Constanti V, Domingo Garau A, Rodríguez Marrodán B, Villalobos Pinto E, Riaza Gómez M, García Soto L, et al. Do not do recommendations in different paediatric care settings. Anales de Pediatría (English Edition). 2023;98(4):291-300. doi: https://doi.org/10.1016/j.anpede.2023.02.019.

3147. Arias Constanti V, Domingo Garau A, Rodríguez Marrodán B, Villalobos Pinto E, Riaza Gómez M, García Soto L, et al. Recomendaciones de no hacer en distintos ámbitos de la atención pediátrica. Anales de Pediatría. 2023;98(4):291-300. doi: https://doi.org/10.1016/j.anpedi.2023.02.001.

3148. Arias JL, Santamaría-López JM. Improvement of the drug therapy results in patients with high blood pressure at a community pharmacy. Ars Pharmaceutica. 2008;49(1):39-50.

3149. Arias LHM, Lobato CT, Garcia SP, Ortega PG, Gil MS, Fadrique RS, et al. Impact of regulatory measures on antipsychotics drug consumption in Castilla y Leon, Spain. Public Health. 2016;141:113-9. doi: 10.1016/j.puhe.2016.08.011. PubMed PMID: WOS:000390639900018.

3150. Arias S, Monestel Z, Fernandez JMC, Garcia C, Bermudez G, Madriz JPD, et al. Impact of the clinical care provided by pharmacists to patients treated in the Onco-Hematology service in a private hospital in Costa Rica. Pharmaceutical Care Espana. 2021;23(3):253-68. PubMed PMID: WOS:000661261600005.

3151. Arias-Núñez D, Chavarría-Viquez C, Quesada-Morúa MS. Proposal of an active dispensation protocol of oral antineoplasics for newly diagnosed patients for an outpatient hospital pharmacy service. Pharmaceutical Care Espana. 2023;25(5):5-19. doi: 10.60103/phc.v25i5.821. PubMed PMID: WOS:001099681700001.

3152. Aricanli S. Diversifying the center: Authority and representation within the context of multiplicity in eighteenth century Qing imperial medicine [Ph.D.]. United States -- New Jersey: Princeton University; 2016.

3153. Arief NN, Karlinah S, Setianti Y, Susilawati S. Counterfeit vaccines in Indonesia: managing the issue through media. Journal of Communication Management. 2018;22(4):510-20. doi: 10.1108/jcom-09-2018-0081. PubMed PMID: WOS:000448128300010.

3154. Arif R, Khan AZ, Hammad M, Ghani U, Vaddepalli R, Sanker V. Current Practices and Perceived Role of Community Pharmacists in Type 2 Diabetes Services in Pakistan. Cureus Journal of Medical Science. 2023;15(4). doi: 10.7759/cureus.37311. PubMed PMID: WOS:001032254700027.

3155. Arif S, Wang S, Lakada IY, Lee JY. An elective course to train student pharmacists to provide culturally sensitive health care. American Journal of Pharmaceutical Education. 2019;83(8):1742-8.

3156. Arif SA, Gill TK, Reutzel TJ. Barriers to offering international experiences to pharmacy students by U.S. colleges of pharmacy. Currents in Pharmacy Teaching and Learning. 2013;5(5):387-93. doi: https://doi.org/10.1016/j.cptl.2013.06.009.

3157. Arikan P, Gopinath V, Molino J. Evaluating the effects of pharmacist visits on diabetic control in a residency clinic. Journal of general internal medicine. 2021;36(SUPPL 1):S20-. doi: 10.1007/s11606-021-06830-5. PubMed PMID: CN-02305984.

3158. Arikawe O, Morrissey H, Ball P. Community pharmacy brief screening intervention to improve health outcomes for patients diagnosed with chronic diseases. Journal of Advanced Pharmacy Education and Research. 2022;12(3):1-8. doi: 10.51847/bmamIaRVB8.

3159. Arikian SR, Binder G, Gibson C, Hu XH, Nagarwala Y, Hussein MA, et al. Patterns of Total Costs of Care over Time for Patients with Multiple Myeloma (MM). Blood. 2015;126(23):3294. doi: https://doi.org/10.1182/blood.V126.23.3294.3294.

3160. Aris E, Montourcy M, Esterberg E, Kurosky SK, Poston S, Hogea C. The adult vaccination landscape in the United States during the Affordable Care Act era: Results from a large retrospective database analysis. Vaccine. 2020;38(14):2984-94. doi: https://doi.org/10.1016/j.vaccine.2020.02.057.

3161. Arita E. Why Pharmacists Need Education in the Humanities: A Medical Professional Educator's Viewpoint. Yakugaku Zasshi-Journal of the Pharmaceutical Society of Japan. 2020;140(3):411-4. PubMed PMID: WOS:000517669700013.

3162. Ariyo O, Kinney O, Brookhart A, Nadpara P, Goode J-VKR. Medication therapy problems and vaccine needs identified during initial appointment-based medication synchronization visits. Journal of the American Pharmacists Association. 2019;59(4, Supplement):S67-S71. doi: https://doi.org/10.1016/j.japh.2019.04.019.

3163. Ariyo O, Kinney O, Brookhart A, Nadpara P, Goode JKR. Medication therapy problems and vaccine needs identified during initial appointment-based medication synchronization visits. Journal of the American

Pharmacists Association: JAPhA. 2019;59(4S):S67-S71. PubMed PMID: 31153823.

3164. Ariyo O, Kinney O, Brookhart A, Nadpara P, Goode JVR. Medication therapy problems and vaccine needs identified during initial appointment-based medication synchronization visits. Journal of the American Pharmacists Association. 2019;59(4):S67-S71. doi: 10.1016/j.japh.2019.04.019.

3165. Arkaravichien W, Wongpratat A, Lertsinudom S. Quality indicators to compare accredited independent pharmacies and accredited chain pharmacies in Thailand. International Journal of Clinical Pharmacy. 2016;38(4):899-907. doi: 10.1007/s11096-016-0307-4.

3166. Arkin L, Schuermann AA, Loerzel V, Penoyer D. Original Research: Exploring Medication Safety Practices from the Nurse's Perspective. AJN American Journal of Nursing. 2023;123(12):18-28. doi: 10.1097/01.NAJ.0000996552.02491.7d. PubMed PMID: 175571074.

3167. Arlt S, Lindner R, Rösler A, von Renteln-Kruse W. Adherence to medication in patients with dementia: predictors and strategies for improvement. Drugs & aging. 2008;25:1033-47.

3168. Armada ER, Villamañán E, López-de-Sá E, Rosillo S, Rey-Blas JR, Testillano ML, et al. Computerized physician order entry in the cardiac intensive care unit: Effects on prescription errors and workflow conditions. Journal of Critical Care. 2014;29(2):188-93. doi: 10.1016/j.jcrc.2013.10.016. PubMed PMID: 104041180. Language: English. Entry Date: 20140307. Revision Date: 20200708. Publication Type: Journal Article.

3169. Armando Carle PD, Uema SAN, Martínez Pérez SR, Pallarés MM, Solá Uthurry NH, Faus Dáder MJ. Validation of a questionnaire on patient satisfaction with the dispensing service in community pharmacies. Atencion Primaria. 2007;39(11):591-6. doi: 10.1157/13112195.

3170. Armando P, Semería N, Tenllado M, Sola N. Seguimiento farmacoterapéutico de pacientes en farmacias comunitarias. Atención Primaria. 2005;36(3):129-34. doi: https://doi.org/10.1157/13077478.

3171. Armando P, Semería N, Tenllado M, Sola N. Pharmacotherapeutic follow-up of patients in community pharmacies. Atencion Primaria. 2005;36(3):129-34. doi: 10.1157/13077478.

3172. Armando P, Uema S, Correa Salde V, Fontana D, Vega EM, Paraje MG. Guidelines for utilizing protocols in pharmaceutical consultation or indication. Acta Farmaceutica Bonaerense. 2006;25(2):302-9.

3173. Armando PD, Martinez Perez SR, Marti Pallares M, Sola Uthurry NH, Faus Dader MJ. Development and validation of a Spanish language patient satisfaction questionnaire with drug dispensing. Pharmacy World & Science. 2008;30:169-74.

3174. Armando PD, Perez SRM, Pallares MM, Uthurry NHS, Dader MJF. Development and validation of a Spanish language patient satisfaction questionnaire with drug dispensing. Pharmacy World & Science. 2008;30(2):169-74. doi: 10.1007/s11096-007-9158-3. PubMed PMID: WOS:000253529400006.

3175. Armando PD, Semería N, Tenllado MI, Sola N. Dáder program in Argentina: Results of the first trimester activities. Pharmaceutical Care Espana. 2001;3(3):196-203.

3176. Armando PD, Sonzini LJ, Tenllado MI, Uema SAN, Faccor C. EFFECTS OF PHARMACEUTICAL INTERVENTION IN PATIENTS WITH HIGH BLOOD PRESSURE AND NO PHARMACOLOGICAL TREATMENT AT COMMUNITY PHARMACIES IN THE PROVINCE OF CORDOBA. Revista De Salud Publica-Cordoba. 2017;21(3):35-44. doi: 10.31052/1853.1180.v21.n3.17739. PubMed PMID: WOS:000438090400005.

3177. Armando PD, Uema SA, Vega EM. Integration of community pharmacy and pharmacists in primary health care policies in Argentina. Pharmacy Practice. 2020;18(4):1-7. doi: 10.18549/PharmPract.2020.4.2173.

3178. Armando PD, Vega EM, Martínez-Pérez SR, Martí-Pallarés M, Solá NH, Faus-Dáder MJ. Validating a patient-satisfaction questionnaire about professional advice received for minor illness in community pharmacies. Revista de Salud Publica. 2009;11(5):784-93.

3179. Armas DA, Ayala JRS, Castillo YR, de la Torre AH, Armendáriz CR. Quality of Life Assessment in Patients Using Benzodiazepines during the COVID-19 Pandemic in a Community Pharmacy Using EuroQol 5D-3L. Pharmacy. 2023;11(1). doi: 10.3390/pharmacy11010019. PubMed PMID: WOS:000941691000001.

3180. Armato III SG, McLennan G, McNitt-Gray MF, Meyer CR, Yankelevitz D, Aberle DR, et al. Lung image database consortium: developing a resource for the medical imaging research community. Radiology. 2004;232(3):739-48.

3181. Armero AM, Hernandez MAC, Perez-Vicente SA, Martinez-Martinez F. Pharmaceutical care in smoking cessation. Patient Preference and Adherence. 2015;9. doi: 10.2147/ppa.S67707. PubMed PMID: WOS:000348680800001.

3182. Armero AMíN, Hernandez MAC, Perez-Vicente S, Martinez-Martinez F. Pharmaceutical care in smoking cessation. Patient Preference and Adherence. 2015;9:209-15. doi: 10.2147/PPA.S67707.

3183. Armistead LT, Ferreri SP. Improving Value Through Community Pharmacy Partnerships. Population Health Management. 2019;22(1):5-8. doi: 10.1089/pop.2018.0040.

3184. Armistead LT, Hughes TD, Larson CK, Busby-Whitehead J, Ferreri SP. Integrating targeted consultant pharmacists into a new collaborative care model to reduce the risk of falls in older adults owing to the overuse of opioids and benzodiazepines. Journal of the American Pharmacists Association. 2021;61(1):e16-e8. doi: https://doi.org/10.1016/j.japh.2020.08.038.

3185. Armistead LT, Sanders KA, Larson CK, Busby-Whitehead J, Ferreri SP. A-TAPER: A Framework for Deprescribing Medications effectively. Research in Social and Administrative Pharmacy. 2022;18(8):3358-61. doi: https://doi.org/10.1016/j.sapharm.2021.11.013.

3186. Armitstead J, Loyd L, Pane F, Rough S, Shane R. Implications of the specialty drug marketplace for health-system pharmacy: a roundtable discussion. American Journal of Health-System Pharmacy. 2007;64(22):2364-72. PubMed PMID: 17989447.

3187. Armitstead JA, Burton DC. Enhanced pharmacy training for counter-terrorism and disaster response. Journal of Telemedicine and Telecare. 2006;12(SUPPL. 1):S1:3-5. doi: 10.1258/135763306777978498.

3188. Armoiry X, Aulagner G, Aullen JP, Auray JP, Baume MO, Latour JF, et al. Evaluation of hospital pharmacy activity: Survey conducted on Rhone-Alpes by a multidisciplinary group of health professionals. Journal d'Economie Medicale. 2006;24(1):3-18.

3189. Armor BL, Wight AJ, Carter SM. Evaluation of Adverse Drug Events and Medication Discrepancies in Transitions of Care Between Hospital Discharge and Primary Care Follow-Up. Journal of Pharmacy Practice. 2016;29(2):132-7. doi: 10.1177/0897190014549836. PubMed PMID: 113847310. Language: English. Entry Date: 20160324. Revision Date: 20160328. Publication Type: Article.

3190. Armour C, Bosnic-Anticevich S, Brillant M, Burton D, Emmerton L, Krass I, et al. Pharmacy Asthma Care Program (PACP) improves outcomes for patients in the community. Thorax. 2007;62(6):496-502. doi:

10.1136/thx.2006.064709. PubMed PMID: CN-00586650.

3191. Armour CL, Lemay K, Saini B, Reddel HK, Bosnic-Anticevich SZ, Smith LD, et al. Using the community pharmacy to identify patients at risk of poor asthma control and factors which contribute to this poor control. Journal of Asthma. 2011;48(9):914-22. doi: 10.3109/02770903.2011.615431.

3192. Armour CL, Reddel HK, LeMay KS, Saini B, Smith LD, Bosnic-Anticevich SZ, et al. Feasibility and effectiveness of an evidence-based asthma service in Australian community pharmacies: a pragmatic cluster randomized trial. Journal of asthma. 2013;50(3):302-9. doi: 10.3109/02770903.2012.754463. PubMed PMID: CN-00839702.

3193. Armour CL, Reddel HK, Lemay KS, Saini B, Smith LD, Bosnic-Anticevich SZ, et al. Feasibility and Effectiveness of an Evidence-Based Asthma Service in Australian Community Pharmacies: A Pragmatic Cluster Randomized Trial. Journal of Asthma. 2013;50(3):302-9. doi: 10.3109/02770903.2012.754463.

3194. Armour CL, Smith L, Krass I. Community pharmacy, disease state management, and adherence to medication: A review. Disease Management and Health Outcomes. 2008;16(4):245-54. doi: 10.2165/00115677-200816040-00005.

3195. Armour CL, Smith L, Krass I. Community pharmacy, disease state management, and adherence to medication - A review. Disease Management & Health Outcomes. 2008;16(4):245-54. doi: 10.2165/00115677-200816040-00005. PubMed PMID: WOS:000259137200005.

3196. Armour CL, Taylor SJ, Hourihan F, Smith C, Krass I. Implementation and Evaluation of Australian Pharmacists' Diabetes Care Services. Journal of the American Pharmacists Association. 2004;44(4):455-66. doi: https://doi.org/10.1331/1544345041475625.

3197. Armstrong Cook J, Pouliot J, Parker R. Out With the Old, in With the New: What Rising Pharmacists Need to Know About Vancomycin Therapeutic Drug Monitoring in Adults. Journal of pharmacy practice. 2023:8971900221150321. doi: https://dx.doi.org/10.1177/08971900221150321. PubMed PMID: 639973668.

3198. Armstrong D, Nakhla N. Non-prescription proton-pump inhibitors for self-treating frequent heartburn: The role of the Canadian pharmacist. Pharmacy Practice. 2016;14(4). doi: 10.18549/PharmPract.2016.04.868.

3199. Armstrong EP. DUE Software Highlights Therapeutic Issues: Drug use evaluation systems concurrently review prescribing criteria and patient medication history to identify potential drug therapy problems. American Pharmacy. 1992;32(10):42-6. doi: https://doi.org/10.1016/S0160-3450(15)30970-3.

3200. Armstrong EP, Cherrick RE. Epoetin alfa drug use evaluation using a software system: lessons learned. Disease management and health outcomes. 2000;8(5):287-95. PubMed PMID: CN-01716029.

3201. Armstrong EP, Chrischilles EA. Electronic prescribing and monitoring are needed to improve drug use. Archives of Internal Medicine. 2000;160(18):2713-4.

3202. Armstrong EP, Markson TJ. On-line DUR messages: pharmacists' attitudes and actions in response. Journal of the American Pharmaceutical Association (Washington,DC : 1996). 1997;NS37(3):315-20. doi: 10.1016/s1086-5802(16)30215-7.

3203. Armstrong ML, Duncan CL, Stokes JO, Pereira D. Association of caregiver health beliefs and parenting stress with medication adherence in preschoolers with asthma. Journal of asthma. 2014;51(4):366-72. doi: 10.3109/02770903.2013.876431. PubMed PMID: CN-00991794.

3204. Armutlu M, Foley M-L, Surette J, Belzile E, McCusker J. Survey of nursing perceptions of medication

administration practices, perceived sources of errors and reporting behaviours. Healthcare Quarterly. 2008;11(3):58-65.

3205. Arnal J, Gonzalez-Alvarez I, Bermejo M, Amidon GL, Junginger HE, Kopp S, et al. Biowaiver Monographs for Immediate Release Solid Oral Dosage Forms: Aciclovir. Journal of Pharmaceutical Sciences. 2008;97(12):5061-73. doi: https://doi.org/10.1002/jps.21392.

3206. Arnau-Sánchez J, Jiménez-Guillén C, Alcaraz-Quiñonero M, Vigueras-Abellán JJ, Garnica-Martínez B, Soriano-Ibarra JF, et al. Factors Influencing Inappropriate Use of Antibiotics in Infants under 3 Years of Age in Primary Care: A Qualitative Study of the Paediatricians' Perceptions. Antibiotics. 2023;12(4). doi: 10.3390/antibiotics12040727.

3207. Arnaud P. Similar biological medicinal products in oncology. Is the same drug the same final product? Are they super similar drugs? Oncologie. 2005;7(2):120-3. doi: 10.1007/s10269-005-0169-1.

3208. Arnaud P. Le broyage des comprimés, l'ouverture des capsules, quelles responsabilités ? Le Pharmacien Hospitalier et Clinicien. 2015;50(3):309-11. doi: https://doi.org/10.1016/j.phclin.2015.03.001.

3209. Arnaud S, Jeannin A, Dubois-Arber F. Estimating national-level syringe availability to injecting drug users and injection coverage: Switzerland, 1996–2006. International Journal of Drug Policy. 2011;22(3):226-32. doi: https://doi.org/10.1016/j.drugpo.2011.03.008.

3210. Arnet I, Bernhardt V, Hersberger KE. Methotrexate intoxication: the Pharmaceutical Care process reveals a critical error. Journal of Clinical Pharmacy & Therapeutics. 2012;37(2):242-4. doi: 10.1111/j.1365-2710.2011.01263.x. PubMed PMID: 104527767. Language: English. Entry Date: 20120703. Revision Date: 20200708. Publication Type: Journal Article.

3211. Arnet I, Holden M, Antoniou S. Evaluation of a prompt card for community pharmacists performing consultations with patients on anticoagulation – lessons learned. Pharmacy Practice. 2018;16(3). doi: 10.18549/PharmPract.2018.03.1244.

3212. Arnet I, Holden M, Antoniou S, i PGIPA. Evaluation of a prompt card for community pharmacists performing consultations with patients on anticoagulation - lessons learned. Pharmacy Practice-Granada. 2018;16(3). doi: 10.18549/PharmPract.2018.03.1244. PubMed PMID: WOS:000508117400008.

3213. Arnet I, Messerli M, Oezvegyi J, Hersberger K, Sahm L. Translation to English, cross-cultural adaptation, and pilot testing of the self-report questionnaire on swallowing difficulties with medication intake and coping strategies (SWAMECO) for adults with polypharmacy. BMJ open. 2020;10(8):e036761. doi: 10.1136/bmjopen-2020-036761.

3214. Arnet I, Seidling HM, Hersberger KE. [Adverse drug effects in the community pharmacy]. Therapeutische Umschau. 2015;72(11-12):687-92. PubMed PMID: 26654812.

3215. Arnet I, Walter PN, Hersberger KE. Polymedication Electronic Monitoring System (POEMS) - a new technology for measuring adherence. Frontiers in Pharmacology. 2013;4:26. PubMed PMID: 23493650.

3216. Arney J, Gregg LP, Wydermyer S, Herrera MA, Richardson PA, Matheny ME, et al. Understanding Prescribing Practices and Patient Experiences with Renin Angiotensin System Inhibitors Use in Chronic Kidney Disease: A Qualitative Study. CardioRenal Medicine. 2023;14(1):34-44. doi: https://dx.doi.org/10.1159/000535829. PubMed PMID: 2032341470.

3217. Arnholt AM, Duval-Arnould JM, McNamara LM, Rosen MA, Singh K, Hunt EA. Comparatively Evaluating Medication Preparation Sequences for Treatment of Hyperkalemia in Pediatric Cardiac Arrest: a

Prospective, Randomized, Simulation-Based Study. Pediatric critical care medicine. 2015;16(7):e224-30. doi: 10.1097/PCC.0000000000000497. PubMed PMID: CN-01259330.

3218. Arnold A, Bentley JP, Patel A, Holmes E. Predictors of pharmacists' likelihood to query prescription drug monitoring program databases. Journal of the American Pharmacists Association. 2021;61(5):614-22.e3. doi: https://doi.org/10.1016/j.japh.2021.04.019.

3219. Arnold A, Holmes E, Rosenthal M. Building a Patient-Centered Weight Management Program: A Mixed Methods Project to Obtain Patients' Information Needs and Ideas for Program Structure. Pharmacy. 2019;7(4). doi: 10.3390/pharmacy7040165. PubMed PMID: WOS:000505734200017.

3220. Arnold ME, Buys L, Fullas F. Impact of pharmacist intervention in conjunction with outpatient physician follow-up visits after hospital discharge on readmission rate. American Journal of Health-System Pharmacy. 2015;72(11):S36-S42.

3221. Arnold ME, Buys L, Fullas F. Impact of pharmacist intervention in conjunction with outpatient physician follow-up visits after hospital discharge on readmission rate (Reprinted from Journal of Health-System Pharmacy Residents JHPR, vol 2, issue 4, 2014). American Journal of Health-System Pharmacy. 2015;72(11):S36-S42. doi: 10.2146/sp150011. PubMed PMID: WOS:000218309000010.

3222. Arnold SR, Straus SE. Interventions to improve antibiotic prescribing practices in ambulatory care. Cochrane database of systematic reviews. 2005;(4).

3223. Arnwine CC, Nielsen SL, Davis MK, Galdo JA. Community and Specialty Pharmacy at a Crossroads. Us Pharmacist. 2018;43(4):33-6. PubMed PMID: WOS:000436191700008.

3224. Aroke H, Buchanan A, Wen X, Ragosta P, Koziol J, Kogut S. Estimating the direct costs of outpatient opioid prescriptions: A retrospective analysis of data from the Rhode Island prescription drug monitoring program. Journal of Managed Care and Specialty Pharmacy. 2018;24(3):214-24. doi: 10.18553/jmcp.2018.24.3.214.

3225. Aroke H, Buchanan A, Wen XR, Ragosta P, Koziol J, Kogut S. Estimating the Direct Costs of Outpatient opioid Prescriptions: A Retrospective Analysis of Data from the Rhode Island Prescription Drug Monitoring Program. Journal of Managed Care & Specialty Pharmacy. 2018;24(3):214-+. PubMed PMID: WOS:000428254700003.

3226. Aroke HA. An Evaluation of Provider-Sharing Networks of Patients Using Prescription Opioids [M.S.]. United States -- Rhode Island: University of Rhode Island; 2018.

3227. Aronson J. Medication errors. Side Effects of Drugs Annual. 32: Elsevier; 2010. p. 903-22.

3228. Aronson JK. Medication errors: what they are, how they happen, and how to avoid them. QJM: An International Journal of Medicine. 2009;102(8):513-21.

3229. Aronson JK. From prescription-only to over-the-counter medicines ('PoM to P'): Time for an intermediate category. British Medical Bulletin. 2009;90(1):63-9. doi: 10.1093/bmb/ldp015.

3230. Aronson JK. Meyler's side effects of drugs: the international encyclopedia of adverse drug reactions and interactions: Elsevier; 2015.

3231. Aronson JK, Henderson G, Webb DJ, Rawlins MD. A prescription for better prescribing. British Medical Journal Publishing Group; 2006.

3232. Aronson JK, Heneghan C, Ferner RE. Drug shortages. Part 1. Definitions and harms. British Journal of Clinical Pharmacology. 2023;89(10):2950-6. doi: 10.1111/bcp.15842. PubMed PMID: 171961570.

3233. Aronson JK, Heneghan C, Ferner RE. Drug shortages. Part 2: Trends, causes and solutions. British Journal of Clinical Pharmacology. 2023;89(10):2957-63. doi: 10.1111/bcp.15853. PubMed PMID: 171961572.

3234. Arora R. Hyperspectral spontaneous and nonlinear Raman imaging for biomedical applications [Ph.D.]. United States -- Wisconsin: The University of Wisconsin - Milwaukee; 2011.

3235. Arparsrithongsagul S, Kulsomboon V, Zuckerman IH. Multidisciplinary perspective intervention with community involvement to decrease antibiotic sales in village groceries in Thailand. Asia-pacific journal of public health. 2015;27(2):NP2480-8. doi: 10.1177/1010539513479968. PubMed PMID: CN-01157694.

3236. Arques-Armoiry E, Cabelguenne D, Stamm C, Janoly-Dumenil A, Grosset-Grange I, Vantard N, et al. Problèmes médicamenteux les plus fréquemment détectés par l’analyse pharmacothérapeutique des prescriptions dans un centre hospitalier universitaire. La Revue de Médecine Interne. 2010;31(12):804-11. doi: https://doi.org/10.1016/j.revmed.2010.08.001.

3237. Arques-Armoiry E, Cabelguenne D, Stamm C, Janoly-Dumenil A, Grosset-Grange I, Vantard N, et al. [Most frequent drug-related events detected by pharmacists during prescription analysis in a university hospital]. Revue de Medecine Interne. 2010;31(12):804-11. PubMed PMID: 20843588.

3238. Arrais PSD, Barreto ML, Coelho HLL. Drug prescription and dispensing from the patient's perspective: A community-based study in Fortaleza, Ceará State, Brazil. Cadernos de Saude Publica. 2007;23(4):927-37. doi: 10.1590/S0102-311X2007000400020.

3239. Arrasmith K. Physical Therapy Management of a Person with Subacromial Impingement Syndrome2023.

3240. Arrebola C, Garcia-Delgado P, Barba EL, Pena MLO, Martinez-Martinez F. Pharmacist perception of the use of analgesics and their practice on mild-moderate pain. DOLOR-OFF survey. Atencion Primaria. 2016;48(6):366-73. doi: 10.1016/j.aprim.2015.05.010. PubMed PMID: WOS:000378978300005.

3241. Arrebola C, García-Delgado P, Labrador Barba E, Orera Peña ML, Martínez-Martínez F. Pharmacist perception of the use of analgesics and their practice on mild-moderate pain. DOLOR-OFF survey. Atencion Primaria. 2016;48(6):366-73. doi: 10.1016/j.aprim.2015.05.010.

3242. Arrebola Pascual I, Hernández Martí F, Cremades Alcaraz J. Results from the application of a quality management system in the community pharmacy. Ars Pharmaceutica. 2010;51(SUPPL. 3):277-83.

3243. Arredondo-García JL, Bondani-Guasti A, Burgos-Vargas R, Castelazo-Morales E, De La Garza-Salazar J, Jerjes-Sánchez-Días C, et al. Open formulary propossal for a managed care program in Mexico. Salud Publica de Mexico. 1999;41(1):27-41. doi: 10.1590/s0036-36341999000100005.

3244. Arredondo-Garcia JL, Bondani-Guasti A, Burgos-Vargas R, Castelazo-Morales E, de la Garza-Salazar J, Jerjes-Sanchez-Dias C, et al. Open formulary proposal for a managed care program in Mexico. Salud Publica De Mexico. 1999;41(1):27-41. PubMed PMID: WOS:000079005000005.

3245. Arrieta-Loitegui M, Caro-Teller JM, Ortiz-Perez S, Lopez-Medrano F, San Juan-Garrido R, Ferrari-Piquero JM. Effectiveness, safety and cost analysis of dalbavancin in clinical practice. European Journal of Hospital Pharmacy. 2022;29(1):55-8. doi: 10.1136/ejhpharm-2020-002315. PubMed PMID: WOS:000737292300011.

3246. Arrison W, Merritt E, Powell A. Comparing medication histories obtained by pharmacy technicians and nursing staff in the emergency department. Research in Social and Administrative Pharmacy. 2020;16(10):1398-400. doi: https://doi.org/10.1016/j.sapharm.2020.01.009.

3247. Arrowsmith CH, Audia JE, Austin C, Baell J, Bennett J, Blagg J, et al. The promise and peril of chemical probes. Nature chemical biology. 2015;11(8):536-41.

3248. Arroyo A, Perez-Vicente F, Serrano P, Sanchez A, Miranda E, Navarro JM, et al. Evaluation of the stapled transanal rectal resection technique with two staplers in the treatment of obstructive defecation syndrome. Journal of the American College of Surgeons. 2007;204(1):56-63. doi: 10.1016/j.jamcollsurg.2006.09.017. PubMed PMID: WOS:000243351200008.

3249. Arroyo Conde C, Aquerreta I, Ortega Eslava A, Goñi Zamarbide O, Giráldez Deiró J. Clinical and economic impact of the pharmacy resident incorporation into the healthcare team. Farmacia Hospitalaria (1130-6343). 2006;30(5):284-90. PubMed PMID: 106164570. Language: Spanish. Entry Date: 20071005. Revision Date: 20150711. Publication Type: Journal Article.

3250. Arroyo Conde C, Aquerreta I, Ortega Eslava A, Goñi Zamarbide O, Giráldez Deiró J. Impacto clínico y económico de la incorporación del farmacéutico residente en el equipo asistencial. Farmacia Hospitalaria. 2006;30(5):284-90. doi: https://doi.org/10.1016/S1130-6343(06)73992-7.

3251. Arroyo-Jimenez MM, Minguez S, Carbajal JA, Castro-Vazquez L, Gonzalez-Fuentes J, Blazquez-Abellan G, editors. II WORKSHOP OF PHARMACEUTICAL CARE OF PARKINSON'S DISEASE PATIENTS. 11th Annual International Conference of Education, Research and Innovation (ICERI); 2018 Nov 12-14; Seville, SPAIN2018.

3252. Arroyo-Laguna J, Villanueva PLC. Factors Associated with Changes in Healthcare Demand in Peru, 2007-2021: A Discrete Model of Privatization and Informalization. Revista Gerencia y Politicas de Salud. 2023;22. doi: 10.11144/Javeriana.rgps22.fadd.

3253. Arruñada B. Quality safeguards and regulation of online pharmacies. Health Economics. 2004;13(4):329-44. doi: 10.1002/hec.827.

3254. Arsalan A, Naqvi SBS, Habib S, Hussain M, Shakeel O. Storage of vaccines in different health centers and pharmacies at Karachi, Pakistan: The handling errors. Pakistan Journal of Pharmaceutical Sciences. 2019;32(5):2051-8.

3255. Arsalan A, Qureshi S, Alam M, Ahmed S, Shakeel O, Ali A, et al. Errors in patients' information leaflets of marketed medicines in Pakistan. Journal of Applied Pharmaceutical Science. 2015;5(5):68-74. doi: 10.7324/JAPS.2015.50513.

3256. Arsenault-Lapierre G, Bui TX, Le Berre M, Bergman H, Vedel I. Rural and urban differences in quality of dementia care of persons with dementia and caregivers across all domains: a systematic review. BMC Health Services Research. 2023;23:1-15. doi: 10.1186/s12913-023-09100-8. PubMed PMID: 161654456.

3257. Arshad F, Schuemie MJ, Bu F, Minty EP, Alshammari TM, Lai LYH, et al. Serially Combining Epidemiological Designs Does Not Improve Overall Signal Detection in Vaccine Safety Surveillance. Drug Safety. 2023;46(8):797-807. doi: 10.1007/s40264-023-01324-1. PubMed PMID: 164900024.

3258. Arshad HM, Shoaib MH, Ghayas S, Yousuf RI, Hanif M, Khan A. Pharmaceutical quality control studies on gatifloxacin 200 mg tablets available in the Pakistani market. Latin American Journal of Pharmacy. 2011;30(10):1922.

3259. Arsic J, Krajnovic D. Portable medicine chests and supply of medicines in Serbia from the 1830s to the mid-20th century: analysis of medicines list. Pharmazie. 2020;75(9):443-54. doi: 10.1691/ph.2020.0039. PubMed PMID: WOS:000588348700007.

3260. Arslan M, Akbal Dagistan O, Sar S. Pharmaceutical Care Services During the COVID-19 Pandemic in Turkey: Proposal of a Holistic Approach. Bezmialem Science. 2022;10(2):242-6. doi: 10.14235/bas.galenos.2021.6186. PubMed PMID: WOS:000789045900020.

3261. Arslan M, Kaganoglu I. Identifying and Prioritising the Causes of Medication Errors in the Pharmacy. International Journal of Pharmacy Practice. 2023;31(Supplement 2):ii6-ii7. doi: https://dx.doi.org/10.1093/ijpp/riad074.007. PubMed PMID: 643188341.

3262. Artemi A. The Effect of the Homoeopathic Similimum in the Treatment of Climacteric Symptoms [Master's]. South Africa: University of Johannesburg (South Africa); 2008.

3263. Artes M, Martin Oliveros A, Iracheta Todo M, de Diego Colilla V, Gil Saenz E, Catala Cerdan A, et al. CLINICAL IMPACT OF A PHARMACEUTICAL PROFESIONAL SERVICE INTERVENTION WITH OR WITHOUT A MULTI-COMPARTMENT MEDICATION IN NON-ADHERENT, UNCONTROLLED, CHRONIC HYPERTENSIVE, POLYMEDICATED PATIENTS IN SPANISH COMMUNITY PHARMACIES. SEFAC-SPD- VALOR STUDY. Value in health. 2018;21:S270-S1. doi: 10.1016/j.jval.2018.09.1610. PubMed PMID: CN-01742324.

3264. Artes M, Oliveros AM, Todo MI, Colilla VD, Saenz EG, Cerdan AC, et al. CLINICAL IMPACT OF A PHARMACEUTICAL PROFESIONAL SERVICE INTERVENTION WITH OR WITHOUT A MULTI-COMPARTMENT MEDICATION IN NON-ADHERENT, UNCONTROLLED, CHRONIC HYPERTENSIVE, POLYMEDICATED PATIENTS IN SPANISH COMMUNITY PHARMACIES. SEFAC-SPD- VALOR STUDY. Value in Health. 2018;21:S270-S1. doi: 10.1016/j.jval.2018.09.1610. PubMed PMID: WOS:000459985602369.

3265. Artetxe Aranzamendi E. A study of antibiotics use at a community pharmacy office. Pharmaceutical Care Espana. 2003;5(6):253-60.

3266. Artoisenet C, Ausselet N, Delaere B, Spinewine A. Evaluation of the appropriateness of intravenous amoxicillin/clavulanate prescription in a teaching hospital. Acta Clinica Belgica. 2013;68(2):81-6. doi: 10.2143/ACB.2980.

3267. Arulogun OS, Oluwole SK, Titiloye MA. Prescription errors prevalent in four units of a University Teaching Hospital in Nigeria. Journal of Public Health and Epidemiology. 2011;3(11):513-9.

3268. Arumugam K. The Role of the Community Pharmacists in the Management of Acute Pain in Adults: A Scoping Review2023.

3269. Arunmanakul P, Chaiyakunapruk N, Phrommintikul A, Ruengorn C, Permsuwan U. Cost-effectiveness analysis of pharmacist interventions in patients with heart failure in Thailand. Journal of the American Pharmacists Association. 2022;62(1):71-8. doi: https://doi.org/10.1016/j.japh.2021.10.005.

3270. Aruru M, Truong H-A, Clark S. Pharmacy Emergency Preparedness and Response (PEPR): a proposed framework for expanding pharmacy professionals' roles and contributions to emergency preparedness and response during the COVID-19 pandemic and beyond. Research in Social and Administrative Pharmacy. 2021;17(1):1967-77. doi: https://doi.org/10.1016/j.sapharm.2020.04.002.

3271. Aruru M, Truong HA, Clark S. Pharmacy Emergency Preparedness and Response (PEPR): a proposed framework for expanding pharmacy professionals' roles and contributions to emergency preparedness and

response during the COVID-19 pandemic and beyond. Research In Social & Administrative Pharmacy. 2021;17(1):1967-77. PubMed PMID: 32389631.

3272. Arvidsson PI. On the bridge over the translational valley of death: interview with Per I Arvidsson. Future Science Oa. 2017;3(2). doi: 10.4155/fsoa-2017-0019. PubMed PMID: WOS:000405987900017.

3273. Arvind RK, Beerwala FA, Wali SC, Parihar AS, Ganachari MS, Bhandari R. A Randomized, Single Centered, Parallel and Open Labelled Interventional Study on Effectiveness of Clinical Pharmacists on Adverse Event Following Immunization (AEFI) in Pediatric Population. Current drug safety. 2022;17(4):357-65. doi: 10.2174/1574886317666220103092844. PubMed PMID: CN-02363376.

3274. Arwood MJ, McDonough CW, Cavallari LH, Elsey AR, Frye RF, Gong Y, et al. Evaluating an interactive teaching approach with personal genotyping to provide pharmacy students with a knowledge base for clinical pharmacogenetics. JACCP journal of the american college of clinical pharmacy. 2021;4(3):343-51. doi: 10.1002/jac5.1335. PubMed PMID: CN-02250301.

3275. Arya H, Nimesh S. Chapter 21 - An overview of IND, NDA, approval agencies and FDA post-marketing surveillance. In: Bhatt TK, Nimesh S, editors. The Design & Development of Novel Drugs and Vaccines: Academic Press; 2021. p. 267-73.

3276. Arya SC, Agarwal N. Antimicrobial storage and antibiotic resistance. Journal of the American Board of Family Medicine. 2008;21(2):168. doi: 10.3122/jabfm.2008.02.070233.

3277. Arya V, Pinto S, Singer J. Understanding awareness of pharmacist-led medication therapy management among primary care physicians in New York City, Part II: Findings and implications. Journal of Medical Practice Management. 2013;29(3):187-94. PubMed PMID: 24765739.

3278. Arya V, Pinto S, Singer J, Khan T. Understanding awareness of pharmacist-led medication therapy management among primary care physicians in New York City using qualitative methods: part I. Journal of Medical Practice Management. 2013;29(2):84-8. PubMed PMID: 24228367.

3279. Asahina Y, Hori S, Sawada Y. Understanding of definition and safety of oral health products among patients, physicians and pharmacists. Yakugaku Zasshi. 2010;130(7):961-9. doi: 10.1248/yakushi.130.961.

3280. Asahina Y, Hori S, Sawada Y. Community pharmacists' attitudes relating to patients' use of health products in Japan. International Journal of Clinical Pharmacy. 2012;34(4):529-37. doi: 10.1007/s11096-012-9640-4.

3281. Asante KP, Abokyi L, Zandoh C, Owusu R, Awini E, Sulemana A, et al. Community perceptions of malaria and malaria treatment behaviour in a rural district of Ghana: implications for artemisinin combination therapy. Bmc Public Health. 2010;10. doi: 10.1186/1471-2458-10-409. PubMed PMID: WOS:000281862200003.

3282. Asare AF. Pharmacist-Led Hypertension detection and management services in the Ghanaian community pharmacy: an exploratory study 2017.

3283. Asare KK, Boampong JN, Afoakwah R, Ameyaw EO, Sehgal R, Quashie NB. Use of proscribed chloroquine is associated with an increased risk of pfcrt T76 mutation in some parts of Ghana. Malaria Journal. 2014;13(1). doi: 10.1186/1475-2875-13-246.

3284. Asavakarn S, Sirivatanauksorn Y, Promraj R, Ruenrom A, Limsrichamrern S, Kositamongkol P, et al. Systematic Pharmaceutical Educational Approach to Enhance Drug Adherence in Liver Transplant Recipients. Transplantation Proceedings. 2016;48(4):1202-7. doi: https://doi.org/10.1016/

j.transproceed.2015.12.100.

3285. Asayut N, Olson PS, Kanjanasilp J, Thanarat P, Senkraigul B, Sittisarn C, et al. A community pharmacist-led smoking cessation intervention using a smartphone app (PharmQuit): a randomized controlled trial. PloS one. 2022;17(3):e0265483. doi: 10.1371/journal.pone.0265483. PubMed PMID: CN-02389148.

3286. Asayut N, Sookaneknun P, Chaiyasong S, Saramunee K. Outcomes, costs and stakeholders' perspectives associated with the incorporation of community pharmacy services into the National Health Insurance System in Thailand: a systematic review. International Journal of Pharmacy Practice. 2018;26(1):16-27.

3287. Ascar GI, Hespe CB, Hernández MM. Relationship between polypharmacy and number of specialists consulted by elderly patients. Revista Cubana de Farmacia. 2015;49(3).

3288. Asche C, LaFleur J, Conner C. A Review of Diabetes Treatment Adherence and the Association with Clinical and Economic Outcomes. Clinical Therapeutics. 2011;33(1):74-109. doi: https://doi.org/10.1016/j.clinthera.2011.01.019.

3289. Asche C, McAdam-Marx C, Seal B, Crookston B, Mullins CD. Treatment costs associated with community-acquired pneumonia by community level of antimicrobial resistance. Journal of Antimicrobial Chemotherapy. 2008;61(5):1162-8. doi: 10.1093/jac/dkn073. PubMed PMID: WOS:000254955300032.

3290. Asche CV, Luo WL, Aagren M. Differences in rates of hypoglycemia and health care costs in patients treated with insulin aspart in pens versus vials. Current Medical Research and Opinion. 2013;29(10):1287-96. doi: 10.1185/03007995.2013.825590. PubMed PMID: WOS:000324619300008.

3291. Asche CV, Singer ME, Jhaveri M, Chung HW, Miller A. All-Cause Health Care Utilization and Costs Associated with Newly Diagnosed Multiple Sclerosis in the United States. Journal of Managed Care Pharmacy. 2010;16(9):703-12. doi: 10.18553/jmcp.2010.16.9.703. PubMed PMID: WOS:000285270400004.

3292. Asche S, Bergdall A, Dehmer S, Green B, Maciosek M, Nyboer R. A successful trial multifaceted randomized trial of home blood pressure telemonitoring plus pharmacist management: what makes it work and who benefits the most? Circulation. 2014. PubMed PMID: CN-01042701.

3293. Asche SE, O'Connor PJ, Dehmer SP, Green BB, Bergdall AR, Maciosek MV, et al. Patient characteristics associated with greater blood pressure control in a randomized trial of home blood pressure telemonitoring and pharmacist management. Journal of the American Society of Hypertension : JASH. 2016;10(11):873-80. doi: 10.1016/j.jash.2016.09.004. PubMed PMID: CN-01444252.

3294. Aschenbrenner D. Incidents of Prematurely Infused Medication with Smart Pumps. AJN American Journal of Nursing. 2023;123(12):17-. doi: 10.1097/01.NAJ.0000997212.89790.22. PubMed PMID: 175571073.

3295. Aschenbrenner DS. Smart Use of Smart Infusion Pumps. AJN American Journal of Nursing. 2023;123(6):18-9. doi: 10.1097/01.NAJ.0000938716.49846.e8. PubMed PMID: 163882983.

3296. Aschenbrenner DS. Safe Practices for Medication Administration via Enteral Tubes. AJN American Journal of Nursing. 2023;123(5):22-3. doi: 10.1097/01.NAJ.0000933928.23150.59. PubMed PMID: 163213628.

3297. Aschenbrenner DS. Nurse Using Barcode Workaround Leads to Patient Injury. AJN American Journal of Nursing. 2023;123(9):21-. doi: 10.1097/01.NAJ.0000978136.01694.31. PubMed PMID: 172981043.

3298. Aschenbrenner DS. New Treatment to Prevent Recurring Clostridioides Difficile Infections. AJN American Journal of Nursing. 2023;123(7):16-7. doi: 10.1097/01.NAJ.0000944904.20242.ae. PubMed PMID: 164442331.

3299. Aschenbrenner DS. Automated Dispensing Cabinet Overrides Should be Considered Risky, Not Routine. AJN American Journal of Nursing. 2023;123(7):17-. doi: 10.1097/01.NAJ.0000944908.02445.0f. PubMed PMID: 164442332.

3300. Ascierto PA, Del Vecchio M, Mandalá M, Gogas H, Arance AM, Dalle S, et al. Adjuvant nivolumab versus ipilimumab in resected stage IIIB–C and stage IV melanoma (CheckMate 238): 4-year results from a multicentre, double-blind, randomised, controlled, phase 3 trial. The lancet oncology. 2020;21(11):1465-77.

3301. Ascione FJ, Brown GH, Kirking DM. Evaluation of a medication refill reminder system for a community pharmacy. Patient Education and Counseling. 1985;7(2):157-65. doi: https://doi.org/10.1016/0738-3991(85)90006-0.

3302. Asdaq SMB, Alshammari MK, Alsirhani AB, Alsirhani JB, Alshammari AM, Imran M, et al. Perceived Stress and its Triggers among Pharmacy University Students in Comparison to Student of Other Faculties: A Cross Sectional Study. Indian Journal of Pharmaceutical Education and Research. 2022;56(1):S98-S104. doi: 10.5530/ijper.56.1s.48. PubMed PMID: WOS:000750590300012.

3303. Ased S, Wells J, Morrow LE, Malesker MA. Clinically Significant Food-Drug Interactions. Consultant Pharmacist. 2018;33(11):649-57. PubMed PMID: 30458907.

3304. Aseeva I, Volokhova N. The offset of the value accents in business, medicine and pharmacy in modern socio-economic conditions. Economic Annals-XXI. 2016;157(3-4):75-8. doi: 10.21003/ea.V157-0023.

3305. Asgari MM, Arron ST, Warton EM, Quesenberry CP, Weisshaar D. Sirolimus use and risk of cutaneous squamous cell carcinoma (SCC) in solid organ transplant recipients (SOTRs). Journal of the American Academy of Dermatology. 2015;73(3):444-50. doi: https://doi.org/10.1016/j.jaad.2015.05.029.

3306. Asgari MM, Chien AJ, Tsai AL, Fireman B, Quesenberry CP. Association between Lithium Use and Melanoma Risk and Mortality: A Population-Based Study. Journal of Investigative Dermatology. 2017;137(10):2087-91. doi: https://doi.org/10.1016/j.jid.2017.06.002.

3307. Asgari MM, Tang J, Epstein EH, Chren M-M, Warton EM, Quesenberry CP, et al. Statin use and risk of basal cell carcinoma. Journal of the American Academy of Dermatology. 2009;61(1):66-72. doi: https://doi.org/10.1016/j.jaad.2009.02.011.

3308. Asgarian M, Kooshyar M-M, Elyasi S, Pakdel AF, Aledavood SA. Adherence to a Standardized Order Form for Gastric Cancer in a Referral Chemotherapy Teaching Hospital, Mashhad, Iran. Middle East Journal of Cancer. 2017;8(4):187-93. PubMed PMID: 125251597. Language: English. Entry Date: 20170927. Revision Date: 20170927. Publication Type: Article.

3309. Asghar S, Atif M, Mushtaq I, Malik I, Hayat K. Factors associated with inappropriate dispensing of antibiotics among non-pharmacist pharmacy workers. Research in Social and Administrative Pharmacy. 2020;16(6):805-11.

3310. Asghar S, Atif M, Mushtaq I, Malik I, Hayat K, Babar Z-U-D. Factors associated with inappropriate dispensing of antibiotics among non-pharmacist pharmacy workers. Research in Social and Administrative Pharmacy. 2020;16(6):805-11. doi: https://doi.org/10.1016/j.sapharm.2019.09.003.

3311. Asghar S, Atif M, Mushtaq I, Malik I, Hayat K, Babar ZUD. Factors associated with inappropriate

dispensing of antibiotics among non-pharmacist pharmacy workers. Research in Social and Administrative Pharmacy. 2020;16(6):805-11. doi: 10.1016/j.sapharm.2019.09.003.

3312. Asgharzadeh M, Mahdavipoor B, Asgharzadeh V, Pourostadi M, Kafil HS, Vegari A, et al. Why the COVID-19 Is Not Significantly Reduced in Iran? Iranian Journal of Public Health. 2021;50(7):1303-10. doi: 10.18502/ijph.v50i7.6616. PubMed PMID: WOS:000670985000002.

3313. Ash JS, Gorman P, Lavelle M, Stavri P, Lyman J, Fournier L, et al. Perceptions of physician order entry: results of a cross-site qualitative study. Methods of information in medicine. 2003;42(04):313-23.

3314. Ash JS, Gorman PN, Lavelle M, Payne TH, Massaro TA, Frantz GL, et al. A cross-site qualitative study of physician order entry. Journal of the American Medical Informatics Association. 2003;10(2):188-200.

3315. Ash JS, Sittig DF, Campbell EM, Guappone KP, Dykstra RH, editors. Some unintended consequences of clinical decision support systems. Amia annual Symposium proceedings; 2007: American Medical Informatics Association.

3316. Ash JS, Sittig DF, Dykstra R, Campbell E, Guappone K. The unintended consequences of computerized provider order entry: findings from a mixed methods exploration. International journal of medical informatics. 2009;78:S69-S76.

3317. Ash JS, Sittig DF, Poon EG, Guappone K, Campbell E, Dykstra RH. The extent and importance of unintended consequences related to computerized provider order entry. Journal of the American Medical Informatics Association. 2007;14(4):415-23.

3318. Ash N, Tuten J, Bohenek W, Latham B. A comprehensive approach to addressing the opioid epidemic in a large health system. American Journal of Health-System Pharmacy. 2021;78(4):320-6. doi: 10.1093/ajhp/zxaa388. PubMed PMID: WOS:000649434700013.

3319. Ashames A, Bhandare R, AlAbdin SZ, Alhalabi T, Jassem F. Public perception toward e-commerce of medicines and comparative pharmaceutical quality assessment study of two different products of furosemide tablets from community and illicit online pharmacies. Journal of Pharmacy and Bioallied Sciences. 2019;11(3):284-91. doi: 10.4103/jpbs.JPBS_66_19.

3320. Ashburn K, Antelman G, N'Goran MK, Jahanpour O, Yemaneberhan A, N'Guessan Kouakou B, et al. Willingness to use HIV self-test kits and willingness to pay among urban antenatal clients in Cote d'Ivoire and Tanzania: a cross-sectional study. Tropical Medicine and International Health. 2020;25(9):1155-65. doi: 10.1111/tmi.13456.

3321. Ashburn TT, Thor KB. Drug repositioning: identifying and developing new uses for existing drugs. Nature reviews Drug discovery. 2004;3(8):673-83.

3322. Ashcraft AM, Farjo S, Ponte CD, Dotson S, Sambamoorthi U, Murray PJ. Harder to get than you think: Levonorgestrel emergency contraception access in West Virginia community pharmacies. Journal of the American Pharmacists Association. 2020;60(6):969-77. doi: https://doi.org/10.1016/j.japh.2020.07.027.

3323. Ashcroft D, Morecroft C, Parker D, Noyce P. Safety culture assessment in community pharmacy: development, face validity, and feasibility of the Manchester Patient Safety Assessment Framework. BMJ Quality & Safety. 2005;14(6):417-21.

3324. Ashcroft D, Morecroft C, Parker D, Noyce P. Likelihood of reporting adverse events in community pharmacy: an experimental study. BMJ Quality & Safety. 2006;15(1):48-52.

3325. Ashcroft DM, Cooke J. Retrospective analysis of medication incidents reported using an on-line reporting system. Pharmacy World and Science. 2006;28:359-65.

3326. Ashcroft DM, Lewis PJ, Tully MP, Farragher TM, Taylor D, Wass V, et al. Prevalence, Nature, Severity and Risk Factors for Prescribing Errors in Hospital Inpatients: Prospective Study in 20 UK Hospitals. Drug Safety. 2015;38(9):833-43. PubMed PMID: 26115701.

3327. Ashcroft DM, Morecroft C, Parker D, Noyce P. Reporting, reflecting on and learning from adverse events in community pharmacy: Development and evaluation of an incident reporting form. Pharmaceutical Journal. 2005;274(7350):615-7.

3328. Ashcroft DM, Morecroft C, Parker D, Noyce PR. Safety culture assessment in community pharmacy: Development, face validity, and feasibility of the Manchester Patient Safety Assessment Framework. Quality and Safety in Health Care. 2005;14(6):417-21. doi: 10.1136/qshc.2005.014332.

3329. Ashcroft DM, Morecroft C, Parker D, Noyce PR. Likelihood of reporting adverse events in community pharmacy: an experimental study. Quality & Safety in Health Care. 2006:48-52. doi: 10.1136/qshc.2005.014639. PubMed PMID: 106369989. Language: English. Entry Date: 20061208. Revision Date: 20200708. Publication Type: Journal Article.

3330. Ashcroft DM, Parker D. Development of the Pharmacy Safety Climate Questionnaire: a principal components analysis. Quality and Safety in Health Care. 2009;18(1):28-31. doi: 10.1136/qshc.2006.022129.

3331. Ashcroft R, Mathers A, Gin A, Lam S, Donnelly C, Brown JB, et al. Pharmacists' role and experiences with delivering mental health care within team-based primary care settings during the COVID-19 pandemic. International Journal of Pharmacy Practice. 2024;32(2):156-63. doi: 10.1093/ijpp/riad086. PubMed PMID: WOS:001118064900001.

3332. Åsheim H, Nilsen KB, Stordahl H, Johansen K. Prescription of central nervous system stimulants. Tidsskrift for den Norske Laegeforening. 2001;121(8):908-10.

3333. Ashigbie PG, Azameti D, Wirtz VJ. Challenges of medicines management in the public and private sector under Ghana's National Health Insurance Scheme - a qualitative study. Journal of Pharmaceutical Policy and Practice. 2016;9(1). doi: 10.1186/s40545-016-0055-9.

3334. Ashiq K, Bajwa MA, Khokhar R, Abid F, Tanveer S, Oayyum M, et al. A Cross-Sectional Analysis of Knowledge, Beliefs and Practices Regarding Traditional Medicine Use among Pharmacy Students of Different Private and Government Universities of Lahore, Pakistan. Bangladesh Journal of Medical Science. 2022;21(2):323-34. doi: 10.3329/bjms.v21i2.58065. PubMed PMID: WOS:000762328800013.

3335. Ashiq K, Bajwa MA, Khokhar R, Abid F, Tanveer S, Qayyum M, et al. A Cross-Sectional Analysis of Knowledge, Beliefs and Practices Regarding Traditional Medicine Use among Pharmacy Students of Different Private and Government Universities of Lahore, Pakistan. Bangladesh Journal of Medical Science. 2022;21(2):323-34. doi: 10.3329/bjms.v21i2.58065.

3336. Ashiru-Oredope D, Doble A, Thornley T, Saei A, Gold N, Sallis A, et al. Improving management of respiratory tract infections in community pharmacies and promoting antimicrobial stewardship: a cluster randomised control trial with a self-report behavioural questionnaire and process evaluation. Pharmacy. 2020;8(1). doi: 10.3390/pharmacy8010044. PubMed PMID: CN-02096442.

3337. Ashiru-Oredope D, Harrison T, Pyper C. Rapid evidence review of pharmaceutical public health in high and upper-middle-income countries. Pharmacy Education. 2023;23(5):35. doi: https://dx.doi.org/10.46542/pe.2023.235.138. PubMed PMID: 642369358.

3338. Ashiru-Oredope D, Hopkins S, Vasandani S, Umoh E, Oloyede O, Nilsson A, et al. Healthcare workers' knowledge, attitudes and behaviours with respect to antibiotics, antibiotic use and antibiotic resistance across 30 EU/EEA countries in 2019. Eurosurveillance. 2021;26(12). doi: 10.2807/1560-7917.ES.2021.26.12.1900633.

3339. Ashizawa R, Yamashita K, Take K, Okawara K, Mochizuki E, Sakamoto A, et al. Nonleisure-Time Physical Activity Guidance Following Minor Ischemic Stroke: A Randomized Clinical Trial. Adapted Physical Activity Quarterly. 2021;38(2):329-47. doi: 10.1123/apaq.2020-0029. PubMed PMID: WOS:000631677600009.

3340. Ashizawa T. Primary Care Requirements for Pharmacists-Clinical Reasoning Education at Schools of Pharmaceutical Sciences. Yakugaku Zasshi - Journal of the Pharmaceutical Society of Japan. 2016;136(7):939-44. PubMed PMID: 27374954.

3341. Ashjian E, Kurtz B, Renner E, Yeshe R, Barnes GD. Evaluation of a pharmacist-led outpatient direct oral anticoagulant service. American Journal of Health-System Pharmacy. 2017;74(7):483-9. doi: 10.2146/ajhp151026. PubMed PMID: 122330966. Language: English. Entry Date: 20170411. Revision Date: 20170411. Publication Type: Article.

3342. Ashjian E, Salamin LB, Eschenburg K, Kraft S, Mackler E. Evaluation of outpatient medication reconciliation involving student pharmacists at a comprehensive cancer center. Journal of the American Pharmacists Association. 2015;55(5):540-5. doi: https://doi.org/10.1331/JAPhA.2015.14214.

3343. Ashjian EJ, Yoo A, Piette JD, Choe HM, Thompson AN. Implementation and barriers to uptake of interactive voice response technology aimed to improve blood pressure control at a large academic medical center. Journal of the American Pharmacists Association: JAPhA. 2019;59(2S):S104-S9.e1. PubMed PMID: 30660451.

3344. Ashkanani FZ, Lindsey L, Rathbone AP. A systematic review and thematic synthesis exploring the role of pharmacists in supporting better sleep health and managing sleep disorders. International Journal of Pharmacy Practice. 2023;31(2):153-64. doi: 10.1093/ijpp/riac102.

3345. Ashkanani FZ, Rathbone AP, Lindsey L. The role of pharmacists in deprescribing benzodiazepines: A scoping review. Exploratory Research in Clinical and Social Pharmacy. 2023:100328.

3346. Ashley MJ, Brewster JM, Victor JC. Pharmacists' smoking cessation practices: Relationship to their knowledge and skills, attitudes, and perceptions of roles. Journal of the American Pharmacists Association. 2006;46(6):729-37.

3347. Ashok Kumar M, Elayaraja J, Shailaja K, Ramasamy C. Improving medication adherence and clinical outcomes of hypertensive patients through patient counseling. Research journal of pharmaceutical, biological and chemical sciences. 2011;2(3):231-41. PubMed PMID: CN-00868805.

3348. Ashokkumar R, Srinivasamurthy S, Kelly JJ, Howard SC, Parasuraman S, Uppugunduri CRS. Frequency of chemotherapy medication errors: a systematic review. Journal of Pharmacology and Pharmacotherapeutics. 2018;9(2):86-91.

3349. Ashour A. Non-Technical Skills and the Role of the Community Pharmacist [Ph.D.]. England: The University of Manchester (United Kingdom); 2021.

3350. Ashour A, Ashcroft DM, Phipps DL. Mind the gap: Examining work-as-imagined and work-as-done when dispensing medication in the community pharmacy setting. Applied Ergonomics. 2021;93:103372. doi: https://doi.org/10.1016/j.apergo.2021.103372.

3351. Ashour A, Phipps DL, Ashcroft DM. The role of non-technical skills in community pharmacy practice: An exploratory review of the literature. International Journal of Pharmacy Practice. 2021;29(3):203-9. doi: 10.1093/ijpp/riaa014.

3352. Ashraf Z, Bashir S, Kahkashan I, Majeed M, Farhat S. A Study on Evaluation of Drug Package Inserts in Kashmir, India. Research Journal of Pharmacy and Technology. 2023;16(12):5744-7. doi: https://dx.doi.org/10.52711/0974-360X.2023.00929. PubMed PMID: 2028175911.

3353. Ashwood D, Farris KB, Campo S, Aquilino ML, Losch M. Unlocking the condoms: The effect on sales and theft. Pharmacy Practice. 2011;9(1):44-7. doi: 10.4321/S1886-36552011000100007.

3354. Ashworth N, Kain N, Wiebe D, Hernandez-Ceron N, Jess E, Mazurek K. Reducing prescribing of benzodiazepines in older adults: a comparison of four physician-focused interventions by a medical regulatory authority. BMC family practice. 2021;22(1):68. doi: 10.1186/s12875-021-01415-x. PubMed PMID: CN-02263373.

3355. Asias-Dinh BD, Garey KW. Factors associated with abnormal glucose readings in a pharmacy-led community health fair using the ADA risk assessment tool. Journal of the American Pharmacists Association. 2021;61(2):174-80. doi: https://doi.org/10.1016/j.japh.2020.11.002.

3356. Asif U, Saleem A, Masood I, Nawaz M. Consumers' expectations and experiences with chain community pharmacies in Lahore, Pakistan: a qualitative exploratory study. International journal of clinical pharmacy. 2019. doi: 10.1007/s11096-019-00841-3. PubMed PMID: CN-01936702.

3357. Asim M, Batool SA, Chaudhry MN. Scavengers and their role in the recycling of waste in Southwestern Lahore. Resources Conservation and Recycling. 2012;58:152-62. doi: 10.1016/j.resconrec.2011.10.013. PubMed PMID: WOS:000300127400017.

3358. Asiri IM, Alrastal DY, Alaqeel RK, Alotaibi FM, Kurdi SM, Alshayban DM, et al. A survey of drive-thru pharmacy services: Evaluating the acceptance and perspectives of community pharmacists in Saudi Arabia. Saudi Pharmaceutical Journal. 2024;32(1). doi: 10.1016/j.jsps.2023.101924. PubMed PMID: WOS:001148497700001.

3359. Asiri MAA, Assiri SAA. Prevalence and Outcomes of Rheumatoid Arthritis with Pregnancy: A Systematic Review Article. Egyptian Journal of Hospital Medicine. 2023;92(1):5662-6. doi: 10.21608/ejhm.2023.306898. PubMed PMID: 172015168.

3360. Askarian M, Reza Moravveji A, Assadian O. Prescription of prophylactic antibiotics for neurosurgical procedures in teaching hospitals in Iran. American Journal of Infection Control. 2007;35(4):260-2. doi: https://doi.org/10.1016/j.ajic.2006.04.214.

3361. Askarieh A, Morrison I, Ross K, McCowan C, Matthews K, Kidd B, et al. Medication adherence, utilization of healthcare services, and mortality of patients with epilepsy on opiate replacement therapy: A retrospective cohort study. Epilepsy & Behavior. 2021;117:107829. doi: https://doi.org/10.1016/j.yebeh.2021.107829.

3362. Aslam A, Zin CS, Ab Rahman NS, Gajdács M, Ahmed SI, Jamshed S. Self-medication practices with antibiotics and associated factors among the public of malaysia: A cross-sectional study. Drug, Healthcare and Patient Safety. 2021;13:171-81. doi: 10.2147/DHPS.S331427.

3363. Aslam H, Shah SFA, Khokhar A, Arslaan A, Zahid S, Hakim Z. Role of Corticosteroids in Dengue Fever in Pakistan. Pakistan journal of medical and health sciences. 2021;15(11):3014-6. doi: 10.53350/pjmhs2115113014. PubMed PMID: CN-02358837.

3364. Aslam N, Bushra R, Khan MU. Community pharmacy practice in Pakistan. Arch Pharma Pract 2012; 3: 297. 2012;302.

3365. Aslan I, Özen U. Getting Strategic Advantage by Measuring Resistance Developed against E-Prescription in Turkey. Procedia - Social and Behavioral Sciences. 2014;150:465-74. doi: https://doi.org/10.1016/j.sbspro.2014.09.055.

3366. Aslan K, Kiliç Ö, Kiral E, Bozan G, Bör Ö, Dinleyici EÇ. Clinical and laboratory responses to tigecycline in children. Journal of Clinical Pharmacy and Therapeutics. 2022;47(10):1585-90. doi: 10.1111/jcpt.13708.

3367. Aslani P, Benrimoj SI, Krass I. Development and evaluation of a training program to foster the use of written drug information in community pharmacies. Part 2: Evaluation. Pharmacy Education. 2007;7(2):141-9. doi: 10.1080/15602210701406477.

3368. Aslani P, Krass I. Adherence: A review of education, research, practice and policy in Australia. Pharmacy Practice. 2009;7(1):1-10. doi: 10.4321/S1886-36552009000100001.

3369. Aslani P, Rose G, Chen TF, Whitehead PA, Krass I. A community pharmacist delivered adherence support service for dyslipidaemia. European journal of public health. 2011;21(5):567-72. doi: 10.1093/eurpub/ckq118. PubMed PMID: CN-00813166.

3370. Aslanpour Z. A study of oral counselling and audit processes for dispensed medications in community pharmacy [Ph.D.]. England: University of London, University College London (United Kingdom); 2001.

3371. Aslanyan M, Bobrytska L, Hrytsenko V, Shpychak O, Popova N, Germanyuk T, et al. Technological Aspects of Development of A New Drug In Tablets Called "Lavaflam" And Its Pharmacoeconomic Evaluation. Research Journal of Pharmaceutical Biological and Chemical Sciences. 2017;8(4):808-14. PubMed PMID: WOS:000410676300108.

3372. Asmamaw G, Agedew T, Tesfaye B, Sasamo S, Gena S, Argeta M, et al. Prevalence of leftover medicines, disposal practices, and associated factors in Arba Minch Town, Southern Ethiopia. SAGE Open Medicine. 2023;11. doi: 10.1177/20503121231158214.

3373. Asmelashe Gelayee D, Binega Mekonnen G, Asrade Atnafe S, Birarra MK, Asrie AB. Herbal Medicines: Personal Use, Knowledge, Attitude, Dispensing Practice, and the Barriers among Community Pharmacists in Gondar, Northwest Ethiopia. Evidence-based Complementary and Alternative Medicine. 2017;2017. doi: 10.1155/2017/6480142.

3374. Asmelashe Gelayee D, Binega Mekonnen G, Birarra MK. The Needs and Resources of Drug Information at Community Pharmacies in Gondar Town, Northwest Ethiopia. BioMed Research International. 2017;2017. doi: 10.1155/2017/8310636.

3375. Aspden P, Aspden P. Preventing medication errors: National Acad. Press; 2007.

3376. Asperti F, Ferrario L, Foglia E, Pirovano G, Pozzi R, Rossi T, et al., editors. The optimization of drug distribution processes for citizens and users during pandemics. Proceedings of the Summer School Francesco Turco; 2023.

3377. Aspinall MB, Whittle J, Aspinall SL, Maher RL, Good CB. Improving adverse-drug-reaction reporting in ambulatory care clinics at a Veterans Affairs hospital. American Journal of Health-System Pharmacy. 2002;59(9):841-5. doi: 10.1093/ajhp/59.9.841. PubMed PMID: WOS:000175368000014.

3378. Aspinall S, Sevick MA, Donohue J, Maher R, Hanlon JT. Medication errors in older adults: A review of recent publications. The American Journal of Geriatric Pharmacotherapy. 2007;5(1):75-84. doi: https://doi.org/10.1016/j.amjopharm.2007.03.009.

3379. Aspinall SL, Berlin JA, Zhang YW, Metlay JP. Facility-level variation in antibiotic prescriptions for veterans with upper respiratory infections. Clinical Therapeutics. 2005;27(2):258-62. doi: 10.1016/j.clinthera.2005.02.002. PubMed PMID: WOS:000227691400015.

3380. Aspinall SL, Cunningham FE, Zhao X, Boresi JS, Tonnu-Mihara IQ, Smith KJ, et al. Impact of Pharmacist-Managed Erythropoiesis-Stimulating Agents Clinics for Patients With Non–Dialysis-Dependent CKD. American Journal of Kidney Diseases. 2012;60(3):371-9. doi: https://doi.org/10.1053/j.ajkd.2012.04.013.

3381. Aspinall SL, Metlay JP, Maselli JH, Gonzales R. Impact of hospital formularies on fluoroquinolone prescribing in emergency departments. American Journal of Managed Care. 2007;13(5):241-8. PubMed PMID: WOS:000246564700005.

3382. Aspinall SL, Sales MM, Good CB, Calabrese V, Glassman PA, Burk M, et al. Pharmacy Benefits Management in the Veterans Health Administration Revisited: A Decade of Advancements, 2004-2014. Journal of Managed Care & Specialty Pharmacy. 2016;22(9):1058-63. PubMed PMID: 27579828.

3383. Aspinall SL, Zhao X, Good CB, Stone RA, Boresi J, Cox S, et al. Intervention to Decrease Glyburide Use in Elderly Patients With Renal Insufficiency⁎⁎The views expressed in this paper are those of the authors, and no official endorsement by the Department of Veteran Affairs is intended or should be inferred. The American Journal of Geriatric Pharmacotherapy. 2011;9(1):58-68. doi: https://doi.org/10.1016/j.amjopharm.2011.02.001.

3384. Aspinall SL, Zhao XH, Good CB, Stone RA, Boresi J, Cox S, et al. Intervention to Decrease Glyburide Use in Elderly Patients With Renal Insufficiency. American Journal of Geriatric Pharmacotherapy. 2011;9(1):58-68. doi: 10.1016/j.amjopharm.2011.02.001. PubMed PMID: WOS:000289866600006.

3385. Aspinall TV, Marlee D, Hyde JE, Sims PFG. Prevalence of Toxoplasma gondii in commercial meat products as monitored by polymerase chain reaction - food for thought? International Journal for Parasitology. 2002;32(9):1193-9. doi: 10.1016/s0020-7519(02)00070-x. PubMed PMID: WOS:000177414600013.

3386. Asrade B, Tessema E, Tarekegn A. In vitro comparative quality evaluation of different brands of carbamazepine tablets commercially available in Dessie town, Northeast Ethiopia. BMC Pharmacology and Toxicology. 2023;24(1). doi: 10.1186/s40360-023-00670-1.

3387. Asrade SA, Mishra B. Assessment of dispensing practice and associated factors towards patient medication counseling in dessie town, north east ethiopia. Biotechnol Res Int. 2016;9:111-21.

3388. Asres M, Gedefaw M, Kahsay A, Weldu Y. Patients' Delay in Seeking Health Care for Tuberculosis Diagnosis in East Gojjam Zone, Northwest Ethiopia. American Journal of Tropical Medicine and Hygiene. 2017;96(5):1071-5. doi: 10.4269/ajtmh.16-0892. PubMed PMID: WOS:000401764600013.

3389. Assa MT. An interpersonal perception approach to understanding communication between pharmacists and patients [Ph.D.]. United States -- Florida: University of Florida; 1997.

3390. Assab MA, Jaber D, Albahar F, Alhamad H, Mukattash T, Al-Mathani H, et al. Willingness to pay for community pharmacies-based medication review service: perspectives of private health insurance firms. Pharmacy Practice. 2023;21(4). doi: 10.18549/PharmPract.2023.4.2863.

3391. Assabahi A, Driggers R, Keeys CA, Mader A, Norris P. Evaluation of appropriate vancomycin prescribing for the prevention of newborn group B streptococcal infections in a community hospital obstetrics service. Journal of Perinatal Medicine. 2022;50(9):1218-24. doi: 10.1515/jpm-2021-0558. PubMed PMID: 160092747. Language: English. Entry Date: 20221122. Revision Date: 20221125. Publication Type: Article.

3392. Assaf AM. Stress-induced immune-related diseases and health outcomes of pharmacy students: A pilot study. Saudi Pharmaceutical Journal. 2013;21(1):35-44. doi: https://doi.org/10.1016/j.jsps.2012.02.006.

3393. Assanee J, Sorofman BA, Sirisinsuk Y, Kitisopee T. Factors influencing patient intention to report adverse drug reaction to community pharmacists: A structural equation modeling approach. Research in Social and Administrative Pharmacy. 2022;18(4):2643-50. doi: https://doi.org/10.1016/j.sapharm.2021.05.010.

3394. Asscher A, Parr G, Whitmarsh V. Towards the safer use of medicines. BMJ. 1995;311(7011):1003-5.

3395. Assefa D, Paulos G, Kebebe D, Alemu S, Reta W, Mulugeta T, et al. Investigating the knowledge, perception, and practice of healthcare practitioners toward rational use of compounded medications and its contribution to antimicrobial resistance: a cross-sectional study. BMC Health Services Research. 2022;22(1). doi: 10.1186/s12913-022-07649-4.

3396. Assefa D, Tekassa T, Alemu S. Extent and barriers to providing effective pharmaceutical compounding services in hospital and community pharmacies of a low-income country: Case of Southwest Ethiopia. Journal of Medicine Access. 2023;7. doi: 10.1177/27550834231183753.

3397. Assimon MM. Investigating the Longitudinal Patterns of Use and Comparative Effectiveness of Beta Blocker Therapy in the Hemodialysis Population [Ph.D.]. United States -- North Carolina: The University of North Carolina at Chapel Hill; 2017.

3398. Assiri GA, Shebl NA, Mahmoud MA. Correction: What is the epidemiology of medication errors, error-related adverse events and risk factors for errors in adults managed in community care contexts? A systematic review of the international literature (BMJ Open (2018) 8 (e019101) DOI: 10.1136/bmjopen-2017-019101). BMJ Open. 2019;9(5). doi: 10.1136/bmjopen-2017-019101corr1.

3399. Assiri GA, Shebl NA, Mahmoud MA, Aloudah N, Grant E, Aljadhey H, et al. What is the epidemiology of medication errors, error-related adverse events and risk factors for errors in adults managed in community care contexts? A systematic review of the international literature. BMJ open. 2018;8(5):e019101.

3400. Assuncao-Costa L, Pinto CR, Machado JFF, Valli CG, de Souza L, Franklin BD. Validation of a method to assess the severity of medication administration errors in Brazil. BMJ Open Quality. 2023;12(4):30. PubMed PMID: 38160020.

3401. Assunção-Costa L, Pinto CR, Machado JFF, Valli CG, de Souza LEPF. Assessing the severity of medication administration errors identified in an observational study using a valid and reliable method. Journal of Pharmaceutical Policy & Practice. 2023;16(1):1-5. doi: 10.1186/s40545-023-00653-x. PubMed PMID: 173624310.

3402. Assuncao-costa L, Pinto CR, Machado JFF, Valli CG, De Souza LEPF. Severity of medication administration errors in a teaching hospital in Brazil. Pharmacy Education. 2023;23(3):206. doi: https://dx.doi.org/10.46542/pe.2023.233.187239. PubMed PMID: 642265769.

3403. Assuncao-Costa L, Pinto CR, Machado JFF, Valli CG, de Souza LEPF, Franklin BD. Validation of a method to assess the severity of medication administration errors in Brazil. BMJ Open Quality.

2023;12(4):e002510. doi: https://dx.doi.org/10.1136/bmjoq-2023-002510. PubMed PMID: 2029516125.

3404. Astashkina A, Mann B, Grainger DW. A critical evaluation of in vitro cell culture models for high-throughput drug screening and toxicity. Pharmacology & therapeutics. 2012;134(1):82-106.

3405. Astbury JL, Gallagher CT. Development and validation of a questionnaire to measure moral distress in community pharmacists. International journal of clinical pharmacy. 2017;39:156-64.

3406. Astbury JL, Gallagher CT, O'Neill RC. The issue of moral distress in community pharmacy practice: Background and research agenda. International Journal of Pharmacy Practice. 2015;23(5):361-6. doi: 10.1111/ijpp.12174.

3407. Asti E, Lovece A, Bonavina L. Thoracoscopic Implant of Neurostimulator for Delayed Gastric Conduit Emptying After Esophagectomy. Journal of Laparoendoscopic & Advanced Surgical Techniques. 2016;26(4):299-301. doi: 10.1089/lap.2016.0089. PubMed PMID: WOS:000374838200012.

3408. Astibia AO, Malet-Larrea A, Aizpurua-Arruti X, Gastelurrutia MA, Goyenechea E. Medication discrepancy identification service in community setting. Pharmaceutical Care Espana. 2021;23(2):134-48. PubMed PMID: WOS:000640215100004.

3409. Aston J, Black P. Does participation in formal postgraduate studies have a positive impact on pharmacists' professional activities? Pharmaceutical Journal. 2006;276(7387):175-9.

3410. Aston J, Wilson KA, Sinclair A, Terry D. A telephone survey to determine the experiences of children and their parents/carers, following the initiation of a new medicine. European Journal of Hospital Pharmacy. 2017;24(5):266-71. doi: 10.1136/ejhpharm-2016-000925. PubMed PMID: WOS:000412654900004.

3411. Åstrand B, Åstrand E, Antonov K, Petersson G. Detection of potential drug interactions - A model for a national pharmacy register. European Journal of Clinical Pharmacology. 2006;62(9):749-56. doi: 10.1007/s00228-006-0143-x.

3412. Astrand B, Hovstadius B, Antonov K, Petersson G. The Swedish National Pharmacy Register. Studies in Health Technology & Informatics. 2007;129(Pt 1):345-9. PubMed PMID: 17911736.

3413. Åstrand B, Montelius E, Petersson G, Ekedahl A. Assessment of ePrescription quality: an observational study at three mail-order pharmacies. BMC medical informatics and decision making. 2009;9:1-8.

3414. Astrand B, Montelius E, Petersson G, Ekedahl A, Astrand B, Montelius E, et al. Assessment of ePrescription quality: an observational study at three mail-order pharmacies. BMC Medical Informatics & Decision Making. 2009;9(1):8-. doi: 10.1186/1472-6947-9-8. PubMed PMID: 105531047. Language: English. Entry Date: 20100115. Revision Date: 20211027. Publication Type: journal article.

3415. Astuti EKA, Sriatmi A, Agushybana F. Implementation of Patient Drug Prescription Services in Hospital Pharmacy Installations: Has It Been Managed Properly to Reduce Waiting Time? Contagion: Scientific Periodical Journal of Public Health and Coastal Health. 2023;5(1):189-203.

3416. Asyyed Z, MacDonald T, Price VE, Fernandez C, Bernstein M, Kulkarni K. Assessing Incidence of and Risk Predictors Ascertained at Diagnosis for Symptomatic Venous Thrombotic Events in Pediatric Cancer Patients: A 20-Year Population Based Study from the Maritimes, Canada. Blood. 2015;126(23):1117. doi: https://doi.org/10.1182/blood.V126.23.1117.1117.

3417. Atal S, Jhaj R, Mathur A, Rai N, Misra S, Sadasivam B. Outpatient prescribing trends, rational use of

medicine and impact of prescription audit with feedback at a tertiary care centre in India. International Journal of Health Planning and Management. 2021;36(3):738-53. doi: 10.1002/hpm.3116. PubMed PMID: WOS:000611086700001.

3418. Atanasov AG, Waltenberger B, Pferschy-Wenzig E-M, Linder T, Wawrosch C, Uhrin P, et al. Discovery and resupply of pharmacologically active plant-derived natural products: A review. Biotechnology advances. 2015;33(8):1582-614.

3419. Atance Martínez JC, Yusta Izquierdo A, Grupeli Gardel BE. Costs study in Alzheimer's disease. Revista Clinica Espanola. 2004;204(2):64-9. doi: 10.1157/13058799.

3420. Atanda A, Shapiro NL, Stubbings J, Groo V. Implementation of a New Clinic-Based, Pharmacist-Managed PCSK9 Inhibitor Consultation Service. Journal of Managed Care & Specialty Pharmacy. 2017;23(9):918-25. doi: 10.18553/jmcp.2017.23.9.918. PubMed PMID: WOS:000410718800001.

3421. Atayee RS, Awdishu L, Namba J. Using simulation to improve first-year pharmacy students' ability to identify medication errors involving the top 100 prescription medications. American journal of pharmaceutical education. 2016;80(5).

3422. Atayee RS, Best BM, Daniels CE. Development of an ambulatory palliative care pharmacist practice. Journal of Palliative Medicine. 2008;11(8):1077-82. PubMed PMID: 18980446.

3423. Atayee RS, Sam AM, Edmonds KP. Patterns of Palliative Care Pharmacist Interventions and Outcomes as Part of Inpatient Palliative Care Consult Service. Journal of Palliative Medicine. 2018;21(12):1761-7. doi: 10.1089/jpm.2018.0093. PubMed PMID: WOS:000437073500001.

3424. Atchison A, Bachyrycz A, O'Hara L. 2013 patient care provider of the year finalists. Pharmacy Times. 2013;79(7).

3425. Atehortua NA, Patino S. COVID-19, a tale of two pandemics: novel coronavirus and fake news messaging. Health Promotion International. 2021;36(2):524-34. doi: 10.1093/heapro/daaa140. PubMed PMID: WOS:000647175000020.

3426. Atembina L, Boehmer L, Terrell K, Koka R, Janakiram M, Grothey A, et al. Multidisciplinary Provider Insights to Promote Adoption of Bispecific Antibodies to Treat Cancer in the Community. Blood. 2021;138:4033. doi: https://doi.org/10.1182/blood-2021-153794.

3427. Atencio V, Avey S, Blando P, Campo J, Cannon E, Clewell J, et al. AMCP partnership forum: Biosimilars-ready, set, launch. Journal of Managed Care and Specialty Pharmacy. 2016;22(4):434-40. doi: 10.18553/jmcp.2016.22.4.434.

3428. Ateshim Y, Bereket B, Major F, Emun Y, Woldai B, Pasha I, et al. Prevalence of self-medication with antibiotics and associated factors in the community of Asmara, Eritrea: a descriptive cross sectional survey. Bmc Public Health. 2019;19. doi: 10.1186/s12889-019-7020-x. PubMed PMID: WOS:000471001000003.

3429. Atey TM, Peterson GM, Salahudeen MS, Bereznicki LR, Simpson T, Boland CM, et al. Impact of partnered pharmacist medication charting (PPMC) on medication discrepancies and errors: a pragmatic evaluation of an emergency department-based process redesign. International Journal of Environmental Research and Public Health. 2023;20(2):1452.

3430. Atey TM, Peterson GM, Salahudeen MS, Bereznicki LR, Simpson T, Boland CM, et al. Impact of Partnered Pharmacist Medication Charting (PPMC) on Medication Discrepancies and Errors: A Pragmatic Evaluation of an Emergency Department-Based Process Redesign. International Journal of Environmental

Research and Public Health. 2023;20(2):1452. doi: https://dx.doi.org/10.3390/ijerph20021452. PubMed PMID: 2021152577.

3431. Atey TM, Peterson GM, Salahudeen MS, Bereznicki LR, Wimmer BC. Impact of pharmacist interventions provided in the emergency department on quality use of medicines: a systematic review and meta-analysis. Emergency Medicine Journal. 2023;40(2):120-7.

3432. Atey TM, Peterson GM, Salahudeen MS, Simpson T, Boland CM, Anderson E, et al. Clinical and economic impact of partnered pharmacist medication charting in the emergency department. Frontiers in Pharmacology. 2023;14:1273657. doi: https://dx.doi.org/10.3389/fphar.2023.1273657. PubMed PMID: 2027278744; PubMed Central PMCID: PMCR Foundation [Austria].

3433. Atey TM, Peterson GM, Salahudeen MS, Wimmer BC. The impact of partnered pharmacist medication charting in the emergency department on the use of potentially inappropriate medications in older people. Frontiers in Pharmacology. 2023;14:1273655. doi: https://dx.doi.org/10.3389/fphar.2023.1273655. PubMed PMID: 2026684603.

3434. Athanasopoulos A, Gyftopoulos K, Giannitsas K, Fisfis J, Perimenis P, Barbalias G. Combination treatment with an alpha-blocker plus an anticholinergic for bladder outlet obstruction: A prospective, randomized, controlled study. Journal of Urology. 2003;169(6):2253-6. doi: 10.1097/01.ju.0000067541.73285.eb. PubMed PMID: WOS:000182929500068.

3435. Athavale AS, Banahan I, Benjamin F, Bentley JP, West-Strum DS. Antecedents and consequences of pharmacy loyalty behavior. International Journal of Pharmaceutical and Healthcare Marketing. 2015;9(1):36-55.

3436. Athavale AS, Bentley JP, Banahan BF, McCaffrey DJ, Pace PF. Preliminary development of the Medication Nonpersistence Scale. Journal of the American Pharmacists Association. 2017;57(3):389-94.e1. doi: https://doi.org/10.1016/j.japh.2017.01.014.

3437. Athavale AS, Bentley JP, Banahan BF, McCaffrey DJ, Pace PF, Vorhies DW. Development of the medication adherence estimation and differentiation scale (MEDS). Current Medical Research and Opinion. 2019;35(4):577-85. doi: 10.1080/03007995.2018.1512478.

3438. Athiyah U, Rahem A, Setiawan CD, Hermansyah A. Role of pharmacist in providing drug information and education for patients with chronic diseases during Transition of Care. Pharmacy Education. 2021;21(2):275-80. doi: 10.46542/pe.2021.212.275280. PubMed PMID: WOS:000691189400047.

3439. Athiyah U, Setiawan CD, Nugraheni G, Zairina E, Utami W, Hermansyah A. Assessment of pharmacists' knowledge, attitude and practice in chain community pharmacies towards their current function and performance in Indonesia. Pharmacy Practice (Granada). 2019;17(3).

3440. Athuraliya N, Sarunac J, Robertson J. Medication reconciliation at two teaching hospitals in Australia: a missed opportunity? Internal Medicine Journal. 2017;47(12):1440-4. doi: 10.1111/imj.13634. PubMed PMID: 126656845. Language: English. Entry Date: 20171221. Revision Date: 20200508. Publication Type: Article.

3441. Atia A. PHYSICIAN TRENDS OF DRUG PRESCRIPTION IN LIBYA: A PHARMACOEPIDEMIOLOGICAL STUDY. Pharmacophore. 2019;10(3):33-8. PubMed PMID: WOS:000483370300006.

3442. Atia A. Information provided to customers about over-the-counter medications dispensed in community pharmacies in Libya: A crosssectional study. Eastern Mediterranean Health Journal.

2020;26(7):828-33. doi: 10.26719/emhj.20.030.

3443. Atia A. Information provided to customers about over-the-counter medications dispensed in community pharmacies in Libya: a cross-sectional study. Eastern Mediterranean Health Journal. 2020;26(7):828-33. doi: 10.26719/emhj.20.030. PubMed PMID: WOS:000552313600012.

3444. Atif M, Asghar S, Mushtaq I, Malik I. Community pharmacists as antibiotic stewards: A qualitative study exploring the current status of Antibiotic Stewardship Program in Bahawalpur, Pakistan. Journal of Infection and Public Health. 2020;13(1):118-24.

3445. Atif M, Asghar S, Mushtaq I, Malik I, Amin A, Babar Z-U-D, et al. What drives inappropriate use of antibiotics? A mixed methods study from Bahawalpur, Pakistan. Infection and drug resistance. 2019:687-99.

3446. Atif M, Asghar S, Mushtaq I, Malik I, Amin A, Babar ZUD, et al. What drives inappropriate use of antibiotics? A mixed methods study from Bahawalpur, Pakistan. Infection and Drug Resistance. 2019;12:687-99. doi: 10.2147/IDR.S189114.

3447. Atif M, Hasan S, Mushtaq I, Javaid S, Asghar N, Scahill S. A qualitative study to explore the role of pharmacists in healthy weight management in adults in Pakistan: Current scenario and future perspectives. BMC Health Services Research. 2020;20(1). doi: 10.1186/s12913-020-05419-8.

3448. Atif M, Malik I. COVID-19 and community pharmacy services in Pakistan: challenges, barriers and solution for progress. Journal of pharmaceutical policy and practice. 2020;13(1):1-4.

3449. Atif M, Rasheed W, Mushtaq I, Malik I, Kanwal S, Qamar-Uz-Zaman M. Medication Related Knowledge and Practices Among Patients Attending Pharmacies in Bahawalpur, Pakistan: a Cross-Sectional Study. Latin American Journal of Pharmacy. 2019;38(7):1404-15. PubMed PMID: WOS:000478794500020.

3450. Atif M, Razzaq W, Mushtaq I, Malik I, Razzaq M, Scahill S, et al. Pharmacy services beyond the basics: A qualitative study to explore perspectives of pharmacists towards basic and enhanced pharmacy services in Pakistan. International Journal of Environmental Research and Public Health. 2020;17(7). doi: 10.3390/ijerph17072379.

3451. Atif M, Sarwar MR, Azeem M, Umer D, Rauf A, Rasool A, et al. Assessment of WHO/INRUD core drug use indicators in two tertiary care hospitals of Bahawalpur, Punjab, Pakistan. Journal of Pharmaceutical Policy and Practice. 2016;9(1). doi: 10.1186/s40545-016-0076-4.

3452. Atkin K, Madden M, Morris S, Gough B, McCambridge J. Community pharmacy and public health: preserving professionalism by extending the pharmacy gaze? Sociology of Health and Illness. 2021;43(2):336-52. doi: 10.1111/1467-9566.13221.

3453. Atkin PA, Stringer RS, Duffy JB, Elion C, Ferraris CS, Misrachi SR, et al. The influence of information provided by patients on the accuracy of medication records. Medical journal of Australia. 1998;169(2):85-8. PubMed PMID: CN-00369245.

3454. Atkins K, Van Hoek AJ, Watson C, Baguelin M, Choga L, Patel A, et al. Seasonal influenza vaccination delivery through community pharmacists in England: evaluation of the London pilot. BMJ open. 2016;6(2):e009739.

3455. Atkins L, Chadborn T, Bondaronek P, Ashiru-Oredope D, Beech E, Herd N, et al. Content and mechanism of action of national antimicrobial stewardship interventions on management of respiratory tract infections in primary and community care. Antibiotics. 2020;9(8):1-36. doi: 10.3390/antibiotics9080512.

3456. Atkins PM, Barone LD. Case Study on Measuring and Improving the Medication Use System. The Joint Commission Journal on Quality Improvement. 1999;25(1):40-9. doi: https://doi.org/10.1016/S1070-3241(16)30425-4.

3457. Atkins R, Smith L. Impact of pharmacy intervention on the use of proton-pump inhibitors in the hospital setting. Consultant Pharmacist. 2013;28(12):786-92. doi: 10.4140/TCP.n.2013.786.

3458. Atkinson J. Advances in Pharmacy Practice: A Look towards the Future. Pharmacy. 2022;10(5). doi: 10.3390/pharmacy10050125. PubMed PMID: WOS:000875269900001.

3459. Atkinson J, De Paepe K, Pozo AS, Rekkas D, Volmer D, Hirvonen J, et al. How Do European Pharmacy Students Rank Competences for Practice? Pharmacy. 2016;4(1). doi: 10.3390/pharmacy4010008. PubMed PMID: WOS:000374049100007.

3460. Atkinson J, de Paepe K, Pozo AS, Rekkas D, Volmer D, Hirvonen J, et al. What is a Pharmacist: Opinions of Pharmacy Department Academics and Community Pharmacists on Competences Required for Pharmacy Practice. Pharmacy. 2016;4(1). doi: 10.3390/pharmacy4010012. PubMed PMID: WOS:000374049100011.

3461. Atkinson J, Pozo AS, Rekkas D, Volmer D, Hirvonen J, Bozic B, et al. Hospital and Community Pharmacists' Perceptions of Which Competences Are Important for Their Practice. Pharmacy. 2016;4(2). doi: 10.3390/pharmacy4020021. PubMed PMID: WOS:000380254100006.

3462. Atkinson SD. Increasing the detection of adverse drug events in pediatric patients [M.S.]. United States -- Utah: The University of Utah; 2010.

3463. Atkinson TJ, Gulum AH, Forkum WG. The future of pain pharmacy: driven by need. Integrated Pharmacy Research and Practice. 2016;5:33-42. doi: 10.2147/iprp.S63824. PubMed PMID: WOS:000386587800002.

3464. Atkinson TJ, Pisansky AJB, Miller KL, Jason Yong R. Common elements in opioid use disorder guidelines for buprenorphine prescribing. American Journal of Managed Care. 2019;25(3):E88-E97.

3465. Atkinson TJ, Pisansky AJB, Miller KL, Yong RJ. Common Elements in Opioid Use Disorder Guidelines for Buprenorphine Prescribing. American Journal of Managed Care. 2019;25(3):E88-+. PubMed PMID: WOS:000471690100006.

3466. Atlas RF. The role of PBMS in implementing the medicare prescription drug benefit. Health Affairs. 2004;23(SUPPL. 2):W4-504. doi: 10.1377/hlthaff.W4.504.

3467. Atletiko FJ. Development of Android Application for Courier Monitoring System. Procedia Computer Science. 2017;124:759-66. doi: https://doi.org/10.1016/j.procs.2017.12.215.

3468. Atsbeha BW, Kebede BT, Birhanu BS, Yimenu DK, Belay WS, Demeke CA. The Weekend Drug; Recreational Use of Sildenafil Citrate and Concomitant Factors: A Cross-Sectional Study. Frontiers in Medicine. 2021;8. doi: 10.3389/fmed.2021.665247.

3469. Atsmon J, Cherniakov I, Izgelov D, Hoffman A, Domb AJ, Deutsch L, et al. PTL401, a New Formulation Based on Pro-Nano Dispersion Technology, Improves Oral Cannabinoids Bioavailability in Healthy Volunteers. Journal of pharmaceutical sciences. 2018;107(5):1423-9. doi: 10.1016/j.xphs.2017.12.020. PubMed PMID: CN-01570349.

3470. Attaei MW, Khatib R, McKee M, Lear S, Dagenais G, Igumbor EU, et al. Availability and affordability

of blood pressure-lowering medicines and the effect on blood pressure control in high-income, middle-income, and low-income countries: an analysis of the PURE study data. The Lancet Public Health. 2017;2(9):e411-e9. doi: 10.1016/S2468-2667(17)30141-X.

3471. Attard E. History, definition, and legislation. Nonvitamin and Nonmineral Nutritional Supplements2018. p. 3-8.

3472. Attard MC, Loupis Y, Togher L, Rose ML. Experiences of people with severe aphasia and spouses attending an Interdisciplinary Community Aphasia Group. Disability and Rehabilitation. 2020;42(10):1382-96. doi: 10.1080/09638288.2018.1526336. PubMed PMID: WOS:000533029800005.

3473. Atthobari J, Monster TB, de Jong PE, de Jong-van den Berg LT. The effect of hypertension and hypercholesterolemia screening with subsequent intervention letter on the use of blood pressure and lipid lowering drugs. British journal of clinical pharmacology. 2004;57(3):328-36. doi: 10.1046/j.1365-2125.2003.02011.x. PubMed PMID: CN-00471645.

3474. Atthobari J, Monster TBM, De Jong PE, De Jong-Van Den Berg LTW. The effect of hypertension and hypercholesterolemia screening with subsequent intervention letter on the use of blood pressure and lipid lowering drugs. British Journal of Clinical Pharmacology. 2004;57(3):328-36. doi: 10.1046/j.1365-2125.2003.02011.x.

3475. Atthobari J, Monster TBM, de Jong PE, de Jong-van den Berg LTW, Prevend Study G. The effect of hypertension and hypercholesterolemia screening with subsequent intervention letter on the use of blood pressure and lipid lowering drugs. British Journal of Clinical Pharmacology. 2004;57(3):328-36. doi: 10.1111/j.1365-2125.2003.02011.x. PubMed PMID: WOS:000188988800013.

3476. Attia E, Fuentes A, Vassallo M, Dobbs S, Nguyen P, Baker K. Establishing a multidisciplinary taskforce to improve anticoagulation safety at a large health system. American Journal of Health-System Pharmacy. 2022;79(4):297-305. doi: 10.1093/ajhp/zxab411. PubMed PMID: 155145621. Language: English. Entry Date: 20220216. Revision Date: 20220217. Publication Type: Article.

3477. Attipoe-Dorcoo S, Yang P, Sperling L, Loustalot F, Thompson-Paul AM, Gray EB, et al. Characteristics and trends of PCSK9 inhibitor prescription fills in the United States. Journal of Clinical Lipidology. 2021;15(2):332-8. doi: https://doi.org/10.1016/j.jacl.2021.01.010.

3478. Attwood LO, McKechnie M, Vujovic O, Higgs P, Doyle JS, Stewardson AJ. Review of management priorities for invasive infections in people who inject drugs: highlighting the need for patient-centred multidisciplinary care. Medical Journal of Australia. 2022;217(2):102-9. doi: 10.5694/mja2.51623. PubMed PMID: WOS:000815728700001.

3479. Atzema CL, Wong A, Masood S, Zia A, Al-bulushi S, Sohail QZ, et al. The Characteristics and Outcomes of Patients Who Make an Emergency Department Visit for Hypertension After Use of a Home or Pharmacy Blood Pressure Device. Annals of Emergency Medicine. 2018;72(5):534-43. doi: https://doi.org/10.1016/j.annemergmed.2018.06.002.

3480. Atzinger CB. Impact of Ultrasonography on Use of Biologics in Patients with Rheumatoid Arthritis [Ph.D.]. United States -- Ohio: University of Cincinnati; 2018.

3481. Atzinger CB, Guo JJ. Biologic Disease-Modifying Antirheumatic Drugs in a National, Privately Insured Population: Utilization, Expenditures, and Price Trends. American Health and Drug Benefits. 2017;10(1):27-35. PubMed PMID: WOS:000397306800005.

3482. Au B, Dersch-Mills D, Ghosh S, Jupp J, Chambers C, Cusano F, et al. Implementation of additional

prescribing authorization among oncology pharmacists in Alberta. Journal of Oncology Pharmacy Practice. 2019;25(3):584-98. PubMed PMID: 29338572.

3483. Au DH, Curtis JR, Every NR, McDonell MB, Fihn SD. Association between inhaled beta-agonists and the risk of unstable angina and myocardial infarction. Chest. 2002;121(3):846-51. doi: 10.1378/chest.121.3.846. PubMed PMID: WOS:000174446000032.

3484. Au NT. Genetic and Environmental Factors Affecting Hemostasis in Yup'ik People [Ph.D.]. United States -- Washington: University of Washington; 2014.

3485. Au TH, Castillo SL, Morrow LE, Malesker MA. Obstructive sleep apnea and continuous positive airway pressure: A primer for pharmacists. US Pharmacist. 2015;40(1):30-3.

3486. Au YK, Baker L, Hindmarsh J. The Impact of an Electronic Prescribing Template with Decision Support upon the Prescribing of Subcutaneous Infusions at the End of Life in a Community Setting: A Future Vision for Community Palliative Care. Pharmacy. 2022;10(5). doi: 10.3390/pharmacy10050112. PubMed PMID: WOS:000873689300001.

3487. Auclair JR, Rathore AS. Analytical Methods to Determine the Stability of Biopharmaceutical Products. LC-GC North America. 2023;41(1):23-7. doi: 10.56530/lcgc.na.qc1477t9. PubMed PMID: 161451191.

3488. Audax AB, Muro EP. Evaluation of Prescriptions Dispensed in the Outpatient Pharmacies of a University Teaching Hospital in Moshi, Kilimanjaro Region, Tanzania: A Cross-Sectional Study. East African Health Research Journal. 2019;3(2):166-71. PubMed PMID: 34308210.

3489. Audet AM, Hartman EE. A 40-year-old woman who noticed a medication error, 1 year later. JAMA. 2002;287(24):3258. PubMed PMID: 12076223.

3490. Audet CM, Pettapiece-Phillips M, Tian Y, Shepherd BE, Vermund SH, Salato J. “If it weren't for my traditional healer, I would be dead”: Engaging traditional healers to support people living with HIV in rural Mozambique. PLoS ONE. 2022;17(6 6). doi: 10.1371/journal.pone.0270565.

3491. Audet CM, Pettapiece-Phillips M, Tian YQ, Shepherd BE, Vermund SH, Salato J. "If it weren't for my traditional healer, I would be dead": Engaging traditional healers to support people living with HIV in rural Mozambique. Plos One. 2022;17(6). doi: 10.1371/journal.pone.0270565. PubMed PMID: WOS:000892027900188.

3492. Audu B. The contribution of pharmacy to the management of hiv patients at maitama district hospital, abuja, nigeria [Ph.D.]. England: University of Wolverhampton (United Kingdom); 2013.

3493. Audu R, Anto BP, Koffuor GA, Abruquah AA, Buabeng KO. Malaria rapid diagnostic test evaluation at private retail pharmacies in Kumasi, Ghana. Journal of Research in Pharmacy Practice. 2016;5(3):175-80. doi: 10.4103/2279-042x.185723. PubMed PMID: WOS:000384204700006.

3494. Audurier Y, Chapet N, Renaudin P, Bons C, Mathieu B, Theret S, et al. Collaboration between cardiologist and clinical pharmacist on prescription quality: What is the potential clinical impact for cardiology patients? International Journal of Clinical Practice. 2020;74(9):1-8. doi: 10.1111/ijcp.13531. PubMed PMID: 145340646. Language: English. Entry Date: 20210123. Revision Date: 20210903. Publication Type: journal article.

3495. Audurier Y, Chapet N, Renaudin P, Bons C, Mathieu B, Theret S, et al. Collaboration between cardiologist and clinical pharmacist on prescription quality: What is the potential clinical impact for cardiology patients? International Journal of Clinical Practice. 2020;74(9):e13531. PubMed PMID:

32459398.

3496. Audurier Y, Roubille C, Manna F, Zerkowski L, Faucanie M, Macioce V, et al. Development and validation of a score to assess risk of medication errors detected during medication reconciliation process at admission in internal medicine unit: SCOREM study. International Journal of Clinical Practice. 2021;75(2):e13663. PubMed PMID: 32770845.

3497. Auerbach M, Adamson JW. How we diagnose and treat iron deficiency anemia. American journal of hematology. 2016;91(1):31-8.

3498. Auffret M, Rolland B, Behal H, Labreuche J, Jouanjus E, Bordet R, et al. A capture-recapture method or estimating the incidence of off-label prescriptions: the example of baclofen or alcohol use disorder in France. Therapie. 2019;74(6):645-50. doi: 10.1016/j.therap.2019.06.002. PubMed PMID: WOS:000497125000009.

3499. Auffret M, Rolland B, Deheul S, Lecomte L, Cottencin O, Bordet R, et al. CAMTEA, un dispositif original de prescription encadrée hors-AMM de baclofène dans le Nord–Pas-de-Calais : quels rôles pour le pharmacien ? Annales Pharmaceutiques Françaises. 2014;72(1):28-32. doi: https://doi.org/10.1016/j.pharma.2013.09.005.

3500. Auffret M, Rolland B, Deheul S, Lecomte L, Cottencin O, Bordet R, et al. [Involvement of pharmacists in systems for supervising off-label medications: example of the CAMTEA system for the prescription of baclofen in alcohol use disorder in Northern France]. Annales Pharmaceutiques Francaises. 2014;72(1):28-32. PubMed PMID: 24438666.

3501. August GP, Caprio S, Fennoy I, Freemark M, Kaufman FR, Lustig RH, et al. Prevention and Treatment of Pediatric Obesity: An Endocrine Society Clinical Practice Guideline Based on Expert Opinion. Journal of Clinical Endocrinology & Metabolism. 2008;93(12):4576-99. doi: 10.1210/jc.2007-2458. PubMed PMID: WOS:000261396900002.

3502. Augustine JM, Winkles CL, Thurston MM. Description of a novel co-curricular professional engagement program for student pharmacists. Currents in Pharmacy Teaching and Learning. 2020;12(9):1062-71. doi: 10.1016/j.cptl.2020.04.033.

3503. Augustine SC, Norenberg JP, Colcher DM, Vose JM, Gobar LS, Dukat VJ, et al. Combination Therapy for Non-Hodgkin's Lymphoma: An Opportunity for Pharmaceutical Care in a Specialty Practice. Journal of the American Pharmaceutical Association (1996). 2002;42(1):93-100. doi: https://doi.org/10.1331/108658002763538125.

3504. Augustine X, Aravind RS, Karthikeyan M, Belsy B, Aswathy KS, Athira BM. Prescription adalath: A novel interventional approach of clinical pharmacy services. European Journal of Clinical Pharmacy. 2016;18(4):273-6.

3505. Aujesky D, Smith KJ, Cornuz J, Roberts MS. Cost-effectiveness of low-molecular-weight heparin for treatment of pulmonary embolism. Chest. 2005;128(3):1601-10. doi: 10.1378/chest.128.3.1601. PubMed PMID: WOS:000231917000081.

3506. Auliya AI, Wijaya IN, Setiawan CD, Nugraheni G. Bpjs kesehatan patients satisfaction on pharmaceutical services in community health center (Puskesmas)—south surabaya area. Indian Journal of Public Health Research and Development. 2019;10(9):1482-7. doi: 10.5958/0976-5506.2019.02658.5.

3507. Aung AK, Zubrinich CM, Goh MSY, Snyder B, Tang MJ, Khu CYL, et al. Development and application of Adverse drug reactions reports QUality Algorithm (AQUA-12) score: a single-centre quality

improvement initiative. European Journal of Clinical Pharmacology. 2023;79(4):513-22. doi: 10.1007/s00228-023-03457-9. PubMed PMID: 162682726.

3508. Aung HL, Siefried KJ, Gates TM, Brew BJ, Mao L, Carr A, et al. Meaningful cognitive decline is uncommon in virally suppressed HIV, but sustained impairment, subtle decline and abnormal cognitive aging are not. eClinicalMedicine. 2023;56. doi: 10.1016/j.eclinm.2022.101792.

3509. Aungst TD, Patel R, Pugliese R, Patel I, Boutari C. From ideation to practice: How pharmacists and students can leverage hackathons and innovation labs to accelerate innovation in pharmacy. Journal of the American Pharmacists Association. 2019;59(2, Supplement):S25-S9. doi: https://doi.org/10.1016/j.japh.2018.12.009.

3510. Austin D, Shivji A, Offei D. Analysis of a novel enrichment strategy for an integrated medicinal chemistry and pharmacology course. Currents in Pharmacy Teaching and Learning. 2020;12(10):1201-7. doi: https://doi.org/10.1016/j.cptl.2020.05.016.

3511. Austin EJ, Gojic AJ, Bhatraju EP, Pierce KA, Pickering EI, Tung EL, et al. Barriers and facilitators to implementing a Pharmacist, Physician, and Patient Navigator-Collaborative Care Model (PPP-CCM) to treat hepatitis C among people who inject drugs. International Journal of Drug Policy. 2023;111. doi: 10.1016/j.drugpo.2022.103924.

3512. Austin JA, Smith IR, Tariq A. The impact of closed-loop electronic medication management on time to first dose: a comparative study between paper and digital hospital environments. International Journal of Pharmacy Practice. 2018;26(6):526-33. doi: 10.1111/ijpp.12432.

3513. Austin JP, Gunden S, Hoffner W, Ismail L, Mendez S, Alvarez F. The value of pediatricians on pharmacy and therapeutics committees. P and T. 2019;44(1):2-4.

3514. Austin M, Fanovich T, Joseph S, Ryan D, Ramdath DD, Pinto Pereira LM. Assessment of risk for Type 2 diabetes mellitus in a Caribbean population with high diabetes-related morbidity. West Indian medical journal. 2004;53(6):387-91. PubMed PMID: CN-01714123.

3515. Austin Z. Development of a curriculum for foreign-trained pharmacists seeking licensure in Canada. Pharmacy education. 2004;4(4).

3516. Austin Z, Ensom MHH. Education of Pharmacists in Canada. American Journal of Pharmaceutical Education. 2008;72(6). doi: 10.5688/aj7206128. PubMed PMID: WOS:000261853000003.

3517. Austin Z, Gregory P. Resilience in the time of pandemic: the experience of community pharmacists during COVID-19. Research in Social and Administrative Pharmacy. 2021;17(1):1867-75.

3518. Austin Z, Gregory PAM. The role of disengagement in the psychology of competence drift. Research in Social and Administrative Pharmacy. 2019;15(1):45-52. doi: https://doi.org/10.1016/j.sapharm.2018.02.011.

3519. Austin Z, Gregory PAM, Martin JC. Characterizing the professional relationships of community pharmacists. Research in Social and Administrative Pharmacy. 2006;2(4):533-46. doi: 10.1016/j.sapharm.2006.02.007.

3520. Austin Z, Marini A, Croteau D, Violato C. Assessment of pharmacist's patient care competencies: Validity evidence from Ontario (Canada)'s quality assurance and Peer Review process. Pharmacy Education. 2004;4(1):23-32. doi: 10.1080/1560221042000208593.

3521. Austin Z, Tsuyuki RT. Health human resources problems are everyone's problems. Canadian Pharmacists Journal. 2023;156(5):229-30. doi: 10.1177/17151635231189226.

3522. Auta A, Bala ET, Shalkur D. Generic medicine substitution: A cross-sectional survey of the perception of pharmacists in north-central, Nigeria. Medical Principles and Practice. 2013;23(1):53-8. doi: 10.1159/000355473.

3523. Auta A, Bala ET, Shalkur D. Generic Medicine Substitution: A Cross-Sectional Survey of the Perception of Pharmacists in North-Central, Nigeria. Medical Principles and Practice. 2014;23(1):53-8. doi: 10.1159/000355473. PubMed PMID: WOS:000329057800010.

3524. Auta A, Banwat SB, Sariem CN, Shalkur D, Nasara B, Atuluku MO. Medicines in Pharmacy Students' Residence Self-medication Practices. Journal of Young Pharmacists. 2012;4(2):119-23. doi: https://doi.org/10.4103/0975-1483.96627.

3525. Auta A, Hadi MA, Oga E, Adewuyi EO, Abdu-Aguye SN, Adeloye D, et al. Global access to antibiotics without prescription in community pharmacies: A systematic review and meta-analysis. Journal of Infection. 2019;78(1):8-18. doi: 10.1016/j.jinf.2018.07.001. PubMed PMID: WOS:000454534000002.

3526. Auta A, Omale S, Folorunsho TJ, David S, Banwat SB. Medicine vendors: Self-medication practices and medicine knowledge. North American journal of medical sciences. 2012;4(1):24.

3527. Auyeung V, Patel G, McRobbie D, Weinman J, Davies G. Information about medicines to cardiac in-patients: Patient satisfaction alongside the role perceptions and practices of doctors, nurses and pharmacists. Patient Education and Counseling. 2011;83(3):360-6. doi: https://doi.org/10.1016/j.pec.2011.04.028.

3528. Auzéric M, Bellemère J, Conort O, Roubille R, Allenet B, Bedouch P, et al. Développement d'un outil d'aide à la description des interactions et des effets indésirables médicamenteux pour la formation et la routine clinique. Annales Pharmaceutiques Françaises. 2009;67(6):433-41. doi: https://doi.org/10.1016/j.pharma.2009.09.003.

3529. Avant ND, McGee HY. The past, present, and futurist role of the pharmacy profession to achieve black health equity. American Journal of Pharmaceutical Education. 2021;85(9):899-907. doi: 10.5688/ajpe8610.

3530. Avant ND, Penm J, Hincapie AL, Huynh VW, Gillespie GL. "Not to exclude you, but…": Characterization of pharmacy student microaggressions and recommendations for academic pharmacy. Currents in Pharmacy Teaching and Learning. 2020;12(10):1171-9. doi: https://doi.org/10.1016/j.cptl.2020.05.007.

3531. Avant ND, Penm J, Hincapie AL, Huynh VW, Gillespie GL. "Not to exclude you, but ...": Characterization of pharmacy student microaggressions and recommendations for academic pharmacy. Currents in Pharmacy Teaching and Learning. 2020;12(10):1171-9. doi: 10.1016/j.cptl.2020.05.007. PubMed PMID: WOS:000562909800002.

3532. Avdic E, Cushinotto LA, Hughes AH, Hansen AR, Efird LE, Bartlett JG, et al. Impact of an antimicrobial stewardship intervention on shortening the duration of therapy for community-acquired pneumonia. Clinical Infectious Diseases. 2012;54(11):1581-7. doi: 10.1093/cid/cis242.

3533. Avedillo-Salas A, Corral-Cativiela S, Fanlo-Villacampa A, Vicente-Romero J. The Efficacy and Safety of Biologic Drugs in the Treatment of Moderate–Severe Crohn's Disease: A Systematic Review. Pharmaceuticals (14248247). 2023;16(11):1581. doi: 10.3390/ph16111581. PubMed PMID: 173864739.

3534. Avent ML, Fejzic J, van Driel ML. An underutilised resource for Antimicrobial Stewardship: a

‘snapshot’ of the community pharmacists’ role in delayed or ‘wait and see’ antibiotic prescribing. International Journal of Pharmacy Practice. 2018;26(4):373-5. doi: 10.1111/ijpp.12431.

3535. Avery AA, Barber N, Ghaleb M, Dean Franklin B, Armstrong S, Crowe S, et al. Investigating the prevalence and causes of prescribing errors in general practice: the PRACtICe study. 2012.

3536. Avery AJ, Anderson C, Bond C, Fortnum H, Gifford A, Hannaford PC, et al. Evaluation of patient reporting of adverse drug reactions to the UK ‘Yellow Card Scheme’: literature review, descriptive and qualitative analyses, and questionnaire surveys. Health Technology Assessment. 2011.

3537. Avery AJ, Ghaleb M, Barber N, Franklin BD, Armstrong SJ, Serumaga B, et al. The prevalence and nature of prescribing and monitoring errors in English general practice: a retrospective case note review. British Journal of General Practice. 2013;63(613):e543-e53.

3538. Avery AJ, Rodgers S, Cantrill JA, Armstrong S, Boyd M, Cresswell K. PINCER trial: a cluster randomised trial comparing the effectiveness and cost-effectiveness of a pharmacist-led IT-based intervention with simple feedback in reducing rates of clinically important errors in medicines management in general practices. A report for the Department of Health Patient Safety Research Portfolio. A report for the department of health patient safety research portfolio. 2010. PubMed PMID: CN-01089582.

3539. Avery AJ, Rodgers S, Cantrill JA, Armstrong S, Cresswell K, Eden M, et al. A pharmacist-led information technology intervention for medication errors (PINCER): a multicentre, cluster randomised, controlled trial and cost-effectiveness analysis. The Lancet. 2012;379(9823):1310-9.

3540. Avery AJ, Rodgers S, Cantrill JA, Armstrong S, Cresswell K, Eden M, et al. A pharmacist-led information technology intervention for medication errors (PINCER): a multicentre, cluster randomised, controlled trial and cost-eff ectiveness analysis. Lancet. 2012;379(9823):1310-9. doi: 10.1016/S0140-6736(11)61817-5. PubMed PMID: CN-00814726.

3541. Avery AJ, Rodgers S, Cantrill JA, Armstrong S, Cresswell K, Eden M, et al. A pharmacist-led information technology intervention for medication errors (PINCER): a multicentre, cluster randomised, controlled trial and cost-effectiveness analysis. The Lancet. 2012;379(9823):1310-9. doi: https://doi.org/10.1016/S0140-6736(11)61817-5.

3542. Avery AJ, Rodgers S, Cantrill JA, Armstrong S, Cresswell K, Eden M, et al. A pharmacist-led information technology intervention for medication errors (PINCER): a multicentre, cluster randomised, controlled trial and cost-effectiveness analysis. Lancet. 2012;379(9823):1310-9. doi: 10.1016/S0140-6736(11)61817-5. PubMed PMID: 108175354. Language: English. Entry Date: 20120511. Revision Date: 20211028. Publication Type: journal article.

3543. Avery AJ, Rodgers S, Cantrill JA, Armstrong S, Eden M, Kendrick D, et al. Assessing the effectiveness of an IT-based pharmacist-led intervention aimed at reducing prortions of patients at risk of medication errors in family practice: the PINCER trial. Pharmacoepidemiology and drug safety Conference: 26th international conference on pharmacoepidemiology and therapeutic risk management brighton united kingdom Conference start: 20100819 conference end: 20100822 Conference publication: (varpagings). 2010;19(Suppl S1):S117. doi: 10.1002/pds.2019. PubMed PMID: CN-00833539.

3544. Avery AJ, Rodgers S, Cantrill JA, Armstrong S, Elliott R, Howard R, et al. Protocol for the PINCER trial: a cluster randomised trial comparing the effectiveness of a pharmacist-led IT-based intervention with simple feedback in reducing rates of clinically important errors in medicines management in general practices. Trials [Electronic Resource]. 2009;10:28. PubMed PMID: 19409095.

3545. Avery AJ, Rodgers S, Cantrill JA, Armstrong S, Elliott R, Howard R, et al. Protocol for the PINCER

trial: a cluster randomised trial comparing the effectiveness of a pharmacist-led IT-based intervention with simple feedback in reducing rates of clinically important errors in medicines management in general practices. Trials. 2009;10:28-. doi: 10.1186/1745-6215-10-28. PubMed PMID: 105536772. Language: English. Entry Date: 20100730. Revision Date: 20211027. Publication Type: journal article.

3546. Avery AJ, Rodgers S, Cantrill JA, Armstrong S, Elliott R, Howard R, et al. Protocol for the PINCER trial: a cluster randomised trial comparing the effectiveness of a pharmacist-led IT-based intervention with simple feedback in reducing rates of clinically important errors in medicines management in general practices. Trials. 2009;10:28. doi: 10.1186/1745-6215-10-28. PubMed PMID: CN-00706927.

3547. Avery AJ, Rodgers S, Cantrill JA, Armstrong S, Elliott R, Howard R, et al. Correction: protocol for the PINCER trial: a cluster randomised trial comparing the effectiveness of a pharmacist-led IT-based intervention with simple feedback in reducing rates of clinically important errors in medicines management in general practices. Trials. 2010;11:23. doi: 10.1186/1745-6215-11-23. PubMed PMID: CN-00789806.

3548. Avery AJ, Sheikh A, Hurwitz B, Smeaton L, Chen Y-F, Howard R, et al. Safer medicines management in primary care. British Journal of General Practice. 2002;52(Suppl):S17-S22.

3549. Avery AP. Folklore and alternative masculinities in a rave scene [Ph.D.]. United States -- New Mexico: The University of New Mexico; 2005.

3550. Avery EJ, Lariscy RW. Preventable Disease Practices Among a Lower SES, Multicultural, Nonurban, US Community: The Roles of Vaccination Efficacy and Personal Constraints. Health Communication. 2014;29(8):826-36. doi: 10.1080/10410236.2013.804486. PubMed PMID: WOS:000335847200008.

3551. Avery LM, Chen IH, Reyes S, Nicolau DP, Kuti JL. Assessment of the Physical Compatibility of Eravacycline and Common Parenteral Drugs During Simulated Y-site Administration. Clinical Therapeutics. 2019;41(10):2162-70. doi: https://doi.org/10.1016/j.clinthera.2019.08.005.

3552. Avery LM, Zempel M, Weiss E. Case of antibiotic-associated diarrhea caused by Staphylococcus aureus enterocolitis. American Journal of Health-System Pharmacy. 2015;72(11):943-51. doi: 10.2146/ajhp140672. PubMed PMID: 103806771. Language: English. Entry Date: 20150603. Revision Date: 20200708. Publication Type: Journal Article.

3553. Avery M, Williams F. The importance of pharmacist providing patient education in oncology. Journal of Pharmacy Practice. 2015;28(1):26-30. PubMed PMID: 25540194.

3554. Avery RL, Castellarin AA, Dhoot DS, Pieramici DJ, Nasir MA, Steinle NC, et al. LARGE SILICONE DROPLETS after INTRAVITREAL BEVACIZUMAB (AVASTIN). Retinal Cases and Brief Reports. 2019;13(2):130-4. doi: 10.1097/ICB.0000000000000570.

3555. Avondstondt AM, Salamon C. The impact of pre-procedure topical lidocaine on pain scores after complex urodynamic testing in women: a randomized control trial. International urogynecology journal. 2019;30(1):S221-. doi: 10.1007/s00192-019-04116-3. PubMed PMID: CN-02119334.

3556. Avong YK, Aliyu GG, Jatau B, Gurumnaan R, Danat N, Kayode GA, et al. Integrating community pharmacy into community based anti-retroviral therapy program: A pilot implementation in Abuja, Nigeria. Plos One. 2018;13(1). doi: 10.1371/journal.pone.0190286. PubMed PMID: WOS:000419689600043.

3557. Avorn J. Powerful medicines: the benefits, risks, and costs of prescription drugs: Vintage; 2008.

3558. Awad A, Abahussain E. Health promotion and education activities of community pharmacists in Kuwait. Pharmacy World and Science. 2010;32(2):146-53. doi: 10.1007/s11096-009-9360-6.

3559. Awad A, Al-Ebrahim S, Abahussain E. Pharmaceutical care services in hospitals of Kuwait. Journal of pharmacy & pharmaceutical sciences : a publication of the Canadian Society for Pharmaceutical Sciences, Société canadienne des sciences pharmaceutiques. 2006;9(2):149-57.

3560. Awad A, Al-Haqan A, Moreau P. Motivations, career aspiration, and learning experience of students in the pharmacy program at Kuwait University: A tool to guide curriculum development. Currents in Pharmacy Teaching and Learning. 2017;9(2):332-8. doi: https://doi.org/10.1016/j.cptl.2016.11.018.

3561. Awad A, Ball D, Eltayeb I. Improving rational drug use in Africa: the example of Sudan. EMHJ-Eastern Mediterranean Health Journal, 13 (5), 1202-1211, 2007. 2007.

3562. Awad A, Eltayeb I, Matowe L, Thalib L. Self-medication with antibiotics and antimalarials in the community of Khartoum State, Sudan. Journal of Pharmacy and Pharmaceutical Sciences. 2005;8(2):326-31.

3563. Awad A, Matowe L, Capps P. Medical doctors' perceptions and expectations of the role of hospital pharmacists in Sudan. Pharmacy World & Science. 2007;29:557-64.

3564. Awad A, Waheedi M. Community Pharmacists role in obesity treatment in Kuwait: A cross-sectional study. BMC Public Health. 2012;12(1). doi: 10.1186/1471-2458-12-863.

3565. Awad AI, Aboud EA. Knowledge, attitude and practice towards antibiotic use among the public in Kuwait. PloS one. 2015;10(2):e0117910.

3566. Awad AI, Al-Rasheedi A, Lemay J. Public perceptions, expectations, and views of community pharmacy practice in Kuwait. Medical Principles and Practice. 2017;26(5):438-46.

3567. Awad AI, Eltayeb IB. Self-medication practices with antibiotics and antimalarials among Sudanese undergraduate university students. Annals of Pharmacotherapy. 2007;41(7-8):1249-55. doi: 10.1345/aph.1K068.

3568. Awad C, Canneva A, Chiasson CO, Galarneau A, Schnitzer ME, Sheehan NL, et al. PHIRST Trial - pharmacist consults: prioritization of HIV-patients with a referral screening tool. AIDS Care. 2017;29(11):1463-72. PubMed PMID: 28614952.

3569. Awad C, Canneva A, Chiasson CO, Galarneau A, Schnitzer ME, Sheehan NL, et al. PHIRST Trial - pharmacist consults: prioritization of HIV-patients with a referral screening tool. Aids Care-Psychological and Socio-Medical Aspects of Aids/Hiv. 2017;29(11):1463-72. doi: 10.1080/09540121.2017.1338657. PubMed PMID: WOS:000413808300021.

3570. Awad E, Obeid S, Sacre H, Salameh P, Strahler J, Hallit S. Association between impulsivity and orthorexia nervosa: any moderating role of maladaptive personality traits? Eating and weight disorders. 2022;27(2):483-93. doi: 10.1007/s40519-021-01186-5. PubMed PMID: CN-02266676.

3571. Awad M, Furdich K, Webb D. Patient satisfaction with pharmacist-led chronic care management services combined with medication synchronization. American Journal of Health-System Pharmacy. 2023;80(19):1350-6. doi: https://dx.doi.org/10.1093/ajhp/zxad148. PubMed PMID: 2031904494.

3572. Awad MH, Ulbrich TR, Furdich KM, Schneider SR, Gothard MD. The Effect of Pharmacy-Led, Small-Group Academic Detailing on Prescribing Patterns in an Ambulatory Care Clinic. Journal of Pharmacy Technology. 2019;35(2):56-63. doi: 10.1177/8755122518818826.

3573. Awad NI, Cocchio C. Assessment of hospital pharmacy preparedness for mass casualty events. P and T. 2015;40(4):264-7.

3574. Awadh AA, Hilleman DE, Knezevich E, Malesker MA, Gallagher JC. Vitamin D supplements: The pharmacists' perspective. Journal of the American Pharmacists Association. 2021;61(4):e191-e201. doi: https://doi.org/10.1016/j.japh.2021.02.002.

3575. Awais YJ. It Has Always Been the "Business Model": Racism and Sexism in Creative Arts Therapy Higher Education2023.

3576. Awaisu A, Alsalimy N. Pharmacists' involvement in and attitudes toward pharmacy practice research: A systematic review of the literature. Research in Social and Administrative Pharmacy. 2015;11(6):725-48. doi: 10.1016/j.sapharm.2014.12.008.

3577. Awaisu A, Katoue MG, Al-Taweel D, Bacha R, El-Gargawi A, Kheir N. Self-reported attitudes and perceived preparedness to provide pharmaceutical care among final year pharmacy students in Qatar and Kuwait. Pharmacy Education. 2018;18(1):284-91.

3578. Awaisu A, Kheir N, Ibrahim MIM, El-Hajj M, Hazi H, Khudair N, et al. Knowledge, attitudes, and practices of community pharmacists on generic medicines in Qatar. International journal of clinical pharmacy. 2014;36:394-404.

3579. Awaisu A, Owusu Y, Sankaralingam S, Al-Ismail M, Abdallah O. Management of Coronary Artery Disease & Dyslipidemia and Pharmacist's Role. In: Babar Z-U-D, editor. Encyclopedia of Pharmacy Practice and Clinical Pharmacy. Oxford: Elsevier; 2019. p. 169-87.

3580. Awan SE, Shamim R, Awais M, Irum S, Shoaib M, Raja MAZ. Convective flow dynamics with suspended carbon nanotubes in the presence of magnetic dipole: Intelligent solution predicted Bayesian regularization networks. Tribology International. 2023;187:108685. doi: https://doi.org/10.1016/j.triboint.2023.108685.

3581. Awang Sayang H, Haji Abdul Mumin K, Md Sofian HN, Abdul Rahman H, Haji Hashim S. Knowledge of Cervical Cancer and Awareness of Screening Services Among Female Undergraduate Health Sciences Students in Brunei. Indian Journal of Gynecologic Oncology. 2021;19(4). doi: 10.1007/s40944-021-00578-w.

3582. Awaritoma O, Oyekanmi N, Erah PO, Isokpehi RD. Small and medium enterprises and biopharmaceutical innovations in Africa: Challenges and prospects. Tropical Journal of Pharmaceutical Research. 2011;10(4):523-9. doi: 10.4314/tjpr.v10i4.19.

3583. Awasthi V, Singh S, Kumar N, Kumar M, Sachan A, Garg R, et al. Evaluation of medical adherence, adverse drug reactions, and quality of life in post tubercular obstructive airway disease. Perspectives in Clinical Research. 2023;14(1):20-5. doi: 10.4103/picr.PICR_55_21. PubMed PMID: 161328616.

3584. Awé C, Lin S-J. A patient empowerment model to prevent medication errors. Journal of Medical Systems. 2003;27:503-17.

3585. Awofisayo SO, Jimmy UA, Eyen NO. Procurement of prescription only medicine (pom); sources, pattern and appropriateness. Journal of Applied Pharmaceutical Science. 2011;1(7):46-9.

3586. Awor P, Wamani H, Bwire G, Jagoe G, Peterson S. Private sector drug shops in integrated community case management of malaria, pneumonia, and diarrhea in children in Uganda. American Journal of Tropical Medicine and Hygiene. 2012;87(SUPPL.5):92-6. doi: 10.4269/ajtmh.2012.11-0791.

3587. Awor P, Wamani H, Tylleskar T, Jagoe G, Peterson S. Increased access to care and appropriateness of treatment at private sector drug shops with integrated management of malaria, pneumonia and diarrhoea: A

quasi-experimental study in Uganda. PLoS ONE. 2014;9(12). doi: 10.1371/journal.pone.0115440.

3588. Awor P, Wamani H, Tylleskar T, Peterson S. Drug seller adherence to clinical protocols with integrated management of malaria, pneumonia and diarrhoea at drug shops in Uganda. Malaria Journal. 2015;14(1). doi: 10.1186/s12936-015-0798-9.

3589. Ax AK, Husberg M, Johansson B, Demmelmaier I, Berntsen S, Sjövall K, et al. Long-term resource utilisation and associated costs of exercise during (neo)adjuvant oncological treatment: the Phys-Can project. Acta oncologica (Stockholm, Sweden). 2022;61(7):888-96. doi: 10.1080/0284186X.2022.2075238. PubMed PMID: CN-02400550.

3590. Ax F, Branstad JO, Westerlund T. Pharmacy counselling models: a means to improve drug use. Journal of clinical pharmacy and therapeutics. 2010;35(4):439-51. doi: 10.1111/j.1365-2710.2009.01138.x. PubMed PMID: CN-00789491.

3591. Ax F, Branstad JO, Westerlund T. Pharmacy counselling models: a means to improve drug usea. Journal of Clinical Pharmacy and Therapeutics. 2010;35(4):439-51. doi: 10.1111/j.1365-2710.2009.01138.x. PubMed PMID: WOS:000279732200009.

3592. Ax F, Ekedahl A. Electronically transmitted prescriptions not picked up at pharmacies in Sweden. Research in Social and Administrative Pharmacy. 2010;6(1):70-7.

3593. Axelrod DA, Schnitzler MA, Alhamad T, Gordon F, Bloom RD, Hess GP, et al. The impact of direct-acting antiviral agents on liver and kidney transplant costs and outcomes. American Journal of Transplantation. 2018;18(10):2473-82. doi: 10.1111/ajt.14895. PubMed PMID: WOS:000446654000015.

3594. Axelsson M. [Proposal on drug reviews lack structure and evidence]. Lakartidningen. 2012;109(26-28):1302. PubMed PMID: 22852262.

3595. Axon DR, Aljadeed R, Potisarach P, Forbes S, DiLeo J, Warholak T. Pilot study of focus groups exploring student pharmacists' perceptions of a medication management center internship. Currents in Pharmacy Teaching & Learning. 2020;12(9):1123-8. PubMed PMID: 32624142.

3596. Axon DR, Chinthammit C, Tate J, Taylor AM, Leal S, Pickering M, et al. Current Procedural Terminology Codes for Medication Therapy Management in Administrative Data. Journal of Managed Care & Specialty Pharmacy. 2020;26(10):1297-300. PubMed PMID: 32996390.

3597. Axon DR, Taylor AM, Vo D, Bingham J. Initial assessment of an interprofessional team-delivered telehealth program for patients with epilepsy. Epilepsy Research. 2019;158:106235. doi: https://doi.org/10.1016/j.eplepsyres.2019.106235.

3598. Axon DR, Vaffis S, Chinthammit C, Lott BE, Taylor AM, Pickering M, et al. Assessing the association between medication adherence, as defined in quality measures, and disease-state control, health care utilization, and costs in a retrospective database analysis of Medicare supplemental beneficiaries using statin medications. Journal of Managed Care & Specialty Pharmacy. 2020;26(12):1529-37. PubMed PMID: WOS:000609241600004.

3599. Axtell S, Haines S, Fairclough J. Effectiveness of Various Methods of Teaching Proper Inhaler Technique. Journal of pharmacy practice. 2017;30(2):195-201. doi: 10.1177/0897190016628961. PubMed PMID: CN-01135230.

3600. Axworthy S, Mackinnon NJ. Perceived importance and self-assessment of the skills of Canada's health-system pharmacy managers. American Journal of Health-System Pharmacy. 2002;59(11):1090-7. doi:

10.1093/ajhp/59.11.1090.

3601. Ayadi F, Ghedira D, Hassairi A. Étude des interventions pharmaceutiques dans deux services cliniques à l'hôpital tunisien universitaire Sahloul. Le Pharmacien Hospitalier et Clinicien. 2016;51(2):116-22. doi: https://doi.org/10.1016/j.phclin.2015.08.009.

3602. Ayadurai S, Sunderland B, Tee LB, Hattingh HL. A training program incorporating a diabetes tool to facilitate delivery of quality diabetes care by community pharmacists in Malaysia and Australia. Pharmacy Practice-Granada. 2019;17(2). doi: 10.18549/PharmPract.2019.2.1457. PubMed PMID: WOS:000508118700011.

3603. Ayadurai S, Sunderland B, Tee LBG, Hattingh HL. A training program incorporating a diabetes tool to facilitate delivery of quality diabetes care by community pharmacists in Malaysia and Australia. Pharmacy Practice. 2019;17(2). doi: 10.18549/PharmPract.2019.2.1457.

3604. Ayadurai S, Sunderland VB, Tee LBG, Md Said SN, Hattingh HL. Structured tool to improve clinical outcomes of type 2 diabetes mellitus patients: a randomized controlled trial. Journal of diabetes. 2018;10(12):965-76. doi: 10.1111/1753-0407.12799. PubMed PMID: CN-01617670.

3605. Ayadurai S, Sunderland VB, Tee LBG, Norlina S, Hattingh HL. Evaluating pharmacist interventions using the Simplertool in Malaysian patients with type 2 diabetes. Pharmaceutical journal. 2020;305(7940). doi: 10.1211/PJ.2020.20208228. PubMed PMID: CN-02466570.

3606. Ayala L, Ayala L. Active medical device cyber-attacks. Cybersecurity for Hospitals and Healthcare Facilities: A Guide to Detection and Prevention. 2016:19-37.

3607. Ayalew MB. Self-medication practice in Ethiopia: a systematic review. Patient Preference and Adherence. 2017;11. doi: 10.2147/ppa.S131496. PubMed PMID: WOS:000395234000001.

3608. Ayalew MB, Dieberg G, Quirk F, Spark MJ. Development and validation of explicit criteria to identify potentially inappropriate prescribing for adults with type 2 diabetes mellitus. Research in Social & Administrative Pharmacy. 2022;18(6):2989-96. doi: 10.1016/j.sapharm.2021.07.014. PubMed PMID: 156373555. Language: English. Entry Date: 20220426. Revision Date: 20220512. Publication Type: journal article. Journal Subset: Biomedical.

3609. Ayalew MB, Solomon L, Abay S. Pharmaceutical Care Practice in Community and Institutional Drug Retail Outlets of Gondar Town, North West Ethiopia. Global Advances In Health and Medicine. 2019;8. doi: 10.1177/2164956119868777.

3610. Ayalon L, Tesch-Römer C. Contemporary perspectives on ageism: Springer Nature; 2018.

3611. Ayana H, Sileshi T, Bule MH, Chaka EE. Non-Prescription Antibiotics Use and Associated Factors Among Drug Retail Outlets in Ambo, Ethiopia: A Cross-Sectional Study. Patient Preference and Adherence. 2021;15:2739-47. doi: 10.2147/PPA.S337364.

3612. Ayandeh A, Farrell N, Sheng AY. Requirement for Discharge in the Care of a Responsible Adult in Procedural Sedation in the Emergency Department: Necessity or Potential Barrier to Health Equity? Journal of Emergency Medicine (0736-4679). 2023;65(4):e272-e9. doi: 10.1016/j.jemermed.2023.05.010. PubMed PMID: 172775386.

3613. Ayati S, Vahidroodsari F, Farshidi F, Shahabian M, Aghaeed MA. Vaginal versus sublingual misoprostol for labor induction at term and post term: a randomized prospective study. Iranian journal of pharmaceutical research. 2014;13(1):299-304. PubMed PMID: CN-00995197.

3614. Aydinkarahaliloglu ND, Aykac E, Kasap Y, Durmus N, Babacanoglu C, Basgut CE, et al. Pharmaceutical risk management in Turkey: The first national overview. Regulatory Toxicology and Pharmacology. 2013;67(3):344-50. doi: https://doi.org/10.1016/j.yrtph.2013.08.012.

3615. Aydoğmuş S. Anadolu Halk Resimleri Figürlerinin Resim Sanatı Açısından İncelenmesi [Master's]. Turkey: Necmettin Erbakan University (Turkey); 2019.

3616. Ayele AA, Cosh S, Islam MS, East L. Role of community pharmacy professionals in child health service provision in Ethiopia: a cross-sectional survey in six cities of Amhara regional state. Bmc Health Services Research. 2022;22(1). doi: 10.1186/s12913-022-08641-8. PubMed PMID: WOS:000869827100004.

3617. Ayele AA, Islam MS, Cosh S, East L. Involvement and practice of community pharmacists in maternal and child health services: A systematic review. Research in Social and Administrative Pharmacy. 2021;17(4):643-52. doi: https://doi.org/10.1016/j.sapharm.2020.07.035.

3618. Ayele AA, Islam MS, Cosh S, East L. Community pharmacy professionals' practice in responding to minor symptoms experienced by pregnant women in Ethiopia: results from sequential mixed methods. Journal of Pharmaceutical Policy and Practice. 2022;15(1). doi: 10.1186/s40545-022-00427-x.

3619. Ayele AA, Islam MS, Cosh S, East L. Involvement of community pharmacy professionals in maternal health service provision in Ethiopia: A multi-centre cross-sectional survey. Journal of Pharmaceutical Health Services Research. 2023;14(1):20-7. doi: 10.1093/jphsr/rmad001.

3620. Ayele AA, Mekuria AB, Tegegn HG, Gebresillassie BM, Mekonnen AB, Erku DA. Management of minor ailments in a community pharmacy setting: Findings from simulated visits and qualitative study in Gondar town, Ethiopia. PloS one. 2018;13(1):e0190583.

3621. Aygun M, Murat M. Assessment of Health Sciences Students' Knowledge and Awareness of the Covid-19 Pandemic: Sample of One University from Turkey. Yuksekogretim Dergisi. 2022;12(1):61-73. doi: 10.2399/yod.21.811926. PubMed PMID: WOS:000812032000001.

3622. Ayhan YE, Enver C, Okuyan B, Boran P, Sancar M. Genel Pediatri Servisinde Kullanılan Pediatrik Dozaj Formu Olmayan İlaçların İncelenmesi: Tanımlayıcı Bir Çalışma. Investigation of Medications Without Pediatric Dosage Forms Used in General Pediatric Service: A Descriptive Study. 2023;32(1):36-41. doi: 10.5336/pediatr.2022-92519. PubMed PMID: 161725448.

3623. Ayhan YE, Karakurt S, Sancar M. The effect of the clinical pharmacist in minimizing drug-related problems and related costs in the intensive care unit in Turkey: a non-randomized controlled study. Journal of clinical pharmacy and therapeutics. 2022;47(11):1867-74. doi: 10.1111/jcpt.13784. PubMed PMID: CN-02495984.

3624. Ayisa A, Getahun Y, Yesuf N. Patient safety culture and associated factors among health-care providers in the university of gondar comprehensive specialized hospital, northwest ethiopia. Drug, Healthcare and Patient Safety. 2021;13:141-50. PubMed PMID: 2007829868.

3625. Ayogu EE, Yahaya RI, Isah A, Ubaka CM. Effectiveness of a pharmacist-led educational intervention on health outcomes in hypertension management at community pharmacies in Nigeria: a two-arm parallel single-blind randomized controlled trial. British journal of clinical pharmacology. 2022. doi: 10.1111/bcp.15514. PubMed PMID: CN-02463654.

3626. Ayogu EE, Yahaya RI, Isah A, Ubaka CM. Effectiveness of Pharmacist-led Educational Intervention on Health Outcomes in Hypertension Management at Community Pharmacies in Nigeria: a Two-Arm Parallel Single-Blind Randomized Controlled Trial. British journal of clinical pharmacology. 2022. doi: 10.1111/

bcp.15514. PubMed PMID: CN-02460058.

3627. Ayogu EE, Yahaya RI, Isah A, Ubaka CM. Effectiveness of a pharmacist-led educational intervention on health outcomes in hypertension management at community pharmacies in Nigeria: A two-arm parallel single-blind randomized controlled trial. British Journal of Clinical Pharmacology. 2023;89(2):649-59. doi: 10.1111/bcp.15514.

3628. Ayogu EE, Yahaya RI, Isah A, Ubaka CM. Effectiveness of a pharmacist-led educational intervention on health outcomes in hypertension management at community pharmacies in Nigeria: A two-arm parallel single-blind randomized controlled trial. British Journal of Clinical Pharmacology. 2023;89(2):649-59. doi: 10.1111/bcp.15514.

3629. Ayorinde AA, Porteous T, Sharma P. Screening for major diseases in community pharmacies: A systematic review. International Journal of Pharmacy Practice. 2013;21(6):349-61. doi: 10.1111/ijpp.12041.

3630. Ayoub J, McGregor JC, Castner RM, Singh H. Opportunities for successful de-escalation of proton pump inhibitors at a federally qualified health center. BMC Pharmacology and Toxicology. 2021;22(1). doi: 10.1186/s40360-021-00486-x.

3631. Ayoub NM, Alhamdan YR, Jaradat SK, Jibreel M. Knowledge of and willingness for dispensing oral anticancer drug therapy among community pharmacists: a cross-sectional study from Jordan. Journal of Pharmaceutical Health Services Research. 2023;14(3):306-16.

3632. Ayoub NM, Nuseir KQ, Othman AK, Abu Alkishik S. Knowledge, attitudes and barriers towards breast cancer health education among community pharmacists. Journal of Pharmaceutical Health Services Research. 2016;7(3):189-98.

3633. Ayre MJ, Lewis PJ, Keers RN. Understanding the medication safety challenges for patients with mental illness in primary care: a scoping review. BMC Psychiatry. 2023;23(1):1-17. doi: 10.1186/s12888-023-04850-5. PubMed PMID: 164490664.

3634. Ayre MJ, Lewis PJ, Phipps DL, Keers RN. Understanding the causes of preventable medication incidents for patients with mental illness in primary care. International Journal of Pharmacy Practice. 2023;31(Supplement 2):ii7. doi: https://dx.doi.org/10.1093/ijpp/riad074.008. PubMed PMID: 643188344.

3635. Ayre MJ, Lewis PJ, Phipps DL, Keers RN. unDerstandIng the cauSes of mediCation errOrs and adVerse drug evEnts for patients with mental illness in community caRe (DISCOVER): a qualitative study. Frontiers in Psychiatry. 2023;14:1241445. doi: https://dx.doi.org/10.3389/fpsyt.2023.1241445. PubMed PMID: 2027270406.

3636. Aytar BA. Türkiye"de Ilaç Sektörünün Ekonomik Yapısı [Master's]. Turkey: Marmara Universitesi (Turkey); 2000.

3637. Ayukekbong JA, Ntemgwa M, Atabe AN. The threat of antimicrobial resistance in developing countries: causes and control strategies. Antimicrobial Resistance and Infection Control. 2017;6. doi: 10.1186/s13756-017-0208-x. PubMed PMID: WOS:000401747300002.

3638. Ayvaz S, Horn J, Hassanzadeh O, Zhu Q, Stan J, Tatonetti NP, et al. Toward a complete dataset of drug–drug interaction information from publicly available sources. Journal of biomedical informatics. 2015;55:206-17.

3639. Azad A, Levy J, Kefer E, Loubani L, Brinton M, Wollen J. The impact of a pharmacy student-led community naloxone education program in high school, college, and pharmacy students on knowledge,

preparedness, and responsiveness in the event of an opioid overdose. JACCP Journal of the American College of Clinical Pharmacy. 2022;5(3):324-31. doi: 10.1002/jac5.1590.

3640. Azad AK, Islam MA, Azizi WSWM. Appraisement of ranitidine hydrochloride tablet (USP150mg) preparations from few selected companies in Bangladesh. Pakistan Journal of Nutrition. 2014;12(11):966-72. doi: 10.3923/pjn.2013.966.972.

3641. Azad AK, Sunzida NK, Islam MA. Unused medication waste: Respondent's opinion from current study in Malaysia. Annals of Tropical Medicine and Public Health. 2018;2(Special Issue):3-7.

3642. Azad MA, Olawuni D, Kimbell G, Badruddoza AZM, Hossain MS, Sultana T. Polymers for extrusion-based 3D printing of pharmaceuticals: A holistic materials–process perspective. Pharmaceutics. 2020;12(2):124.

3643. Azad N, Molnar F, Byszewski A. Lessons learned from a multidisciplinary heart failure clinic for older women: a randomised controlled trial. Age and ageing. 2008;37(3):282-7. doi: 10.1093/ageing/afn013. PubMed PMID: CN-00637914.

3644. Azad N, Tierney M, Victor G, Kumar P. Adverse drug events in the elderly population admitted to a tertiary care hospital. Journal of Healthcare Management. 2002;47(5):295-305.

3645. Azadmehr A, Ziaee A, Ghanei L, Huseini HF, Hajiaghaee R, Tavakoli-Far B, et al. A randomized clinical trial study: anti-oxidant, anti-hyperglycemic and anti-hyperlipidemic effects of olibanum gum in type 2 diabetic patients. Iranian journal of pharmaceutical research. 2014;13(3):1003-10. PubMed PMID: CN-00998571.

3646. Azar C, Raffoul P, Rizk R, Boutros C, Saleh N, Maison P. Prevalence of medication administration errors in hospitalized adults: A systematic review and meta-analysis up to 2017 to explore sources of heterogeneity. Fundamental & Clinical Pharmacology. 2023;37(3):531-48. doi: 10.1111/fcp.12873. PubMed PMID: 163669333.

3647. Azarnoff DL, Lee JC, Lee C, Chandler J, Karlin D. Quality of extemporaneously compounded nitroglycerin ointment. Diseases of the Colon and Rectum. 2007;50(4):509-16. doi: 10.1007/s10350-006-0818-5.

3648. Azeem M, Naureen H, Nasiri MI, Saleem MU. Analysis of cost-of-illness due to typhoid fever in tertiary health care hospitals and pharmaceutical evaluation of different brands used in the treatment. Bulletin of Pharmaceutical Sciences Assiut. 2020;43(1):27-38. doi: 10.21608/BFSA.2020.93568.

3649. Azeez S, Panakkal LM, Meenpidiyil SS, Sulaiman N. Impact of clinical pharmacist intervention in promoting rational antibiotic use in pediatric patients. Research journal of pharmacy and technology. 2020;13(11):5077-82. doi: 10.5958/0974-360X.2020.00889.6. PubMed PMID: CN-02212014.

3650. Azhar S, Hassali MA, Ibrahim MIM. Doctors' Perception and Expectations of the Role of the Pharmacist in Punjab, Pakistan. Tropical Journal of Pharmaceutical Research. 2010;9(3):215-22. PubMed PMID: WOS:000279771900001.

3651. Azhar S, Hassali MA, Ibrahim MIM, Ahmad M, Masood I, Shafie AA. The role of pharmacists in developing countries: the current scenario in Pakistan. Human Resources for Health. 2009;7(1):1-6.

3652. Azhar S, Hassali MA, Ibrahim MMI. Perceptions of hospital pharmacist's role in Pakistan's healthcare system: A cross-sectional survey. Tropical Journal of Pharmaceutical Research. 2011;10(1):11-7. doi: 10.4314/tjpr.v10i1.66535.

3653. Azhar S, Hassali MA, Taha A, Khan SA, Murtaza G, Hussain I. Evaluation of the perception of community pharmacists regarding their role in Pakistan's healthcare system: A qualitative approach. Tropical Journal of Pharmaceutical Research. 2013;12(4):635-9. doi: 10.4314/tjpr.v12i4.29.

3654. Azhar S, Kousar R, Khan SA, Murtaza G. Evolving scenario of pharmaceutical care in Pakistan and other countries: Health impact assessment in public health practice. Acta Poloniae Pharmaceutica - Drug Research. 2016;73(5):1101-9.

3655. Azhar S, Murtaza G, Kousar R, Khan SA. What do the doctors perceive about the quality of pharmaceutical care services in Khyber Pakhtunkhwa, Pakistan: A qualitative study. Latin American Journal of Pharmacy. 2015;34(4):835-88.

3656. Aziz M, Rehman T, Qureshi S, Andleeb S, Aziz MT, Rehman TU. Effects of multidisciplinary teams and an integrated follow-up electronic system on clinical pharmacist interventions in a cancer hospital. International Journal of Clinical Pharmacy. 2017;39(6):1175-84. doi: 10.1007/s11096-017-0530-7. PubMed PMID: 126307425. Language: English. Entry Date: 20180706. Revision Date: 20190708. Publication Type: journal article.

3657. Aziz MM, Alboghdadly A, Rasool MF, Shaalan MS, Goresh HK, Najjar MF, et al. A discursive presentation of community pharmacies: Premises, storage, staff, documentation and legal compliance. Heliyon. 2023;9(12):e23112. doi: https://doi.org/10.1016/j.heliyon.2023.e23112.

3658. Aziz MM, Haider F, Rasool MF, Hashmi FK, Bahsir S, Li P, et al. Dispensing of non-prescribed antibiotics from community pharmacies of Pakistan: A cross-sectional survey of pharmacy staff's opinion. Antibiotics. 2021;10(5). doi: 10.3390/antibiotics10050482.

3659. Aziz MM, Haider F, Rasool MF, Hashmi FK, Bahsir S, Li PC, et al. Dispensing of Non-Prescribed Antibiotics from Community Pharmacies of Pakistan: A Cross-Sectional Survey of Pharmacy Staff's Opinion. Antibiotics-Basel. 2021;10(5). doi: 10.3390/antibiotics10050482. PubMed PMID: WOS:000653473900001.

3660. Aziz MM, Ji W, Masood I, Farooq M, Malik MZ, Chang J, et al. Patient satisfaction with community pharmacies services: a cross-sectional survey from Punjab; Pakistan. International journal of environmental research and public health. 2018;15(12):2914.

3661. Aziz MM, Ji WJ, Masood I, Farooq M, Malik MZ, Chang J, et al. Patient Satisfaction with Community Pharmacies Services: A Cross-Sectional Survey from Punjab; Pakistan. International Journal of Environmental Research and Public Health. 2018;15(12). doi: 10.3390/ijerph15122914. PubMed PMID: WOS:000456527000305.

3662. Aziz MM, Jiang M, Masood I, Chang J, Shan Z, Raza MA, et al. Patients' anticipation for the pharmacies of rural communities: A qualitative study from Pakistan. International Journal of Environmental Research and Public Health. 2019;16(1). doi: 10.3390/ijerph16010143.

3663. Aziz MM, Rasool MF, Alanazi M, Alharby TN, Alanazi J, Huwaimel B. Evaluating the Counseling Standards and Ability of Pharmacy Staff to Detect Antibiotic-Drugs Interactions: A Simulated Client Study from Pakistan. Antibiotics. 2023;12(5). doi: 10.3390/antibiotics12050931.

3664. Aziz MM, Yang S, Masood I, Zhu S, Raza MA, Ji W, et al. An assessment of counseling quality provided by community pharmacies to type 2 diabetic adult patients for oral therapy: A simulated patient study from Pakistan. Endocrine Journal. 2019;66(3):233-9. doi: 10.1507/endocrj.EJ18-0429.

3665. Aziz MM, Yang SM, Masood I, Zhu S, Raza MA, Ji WJ, et al. An assessment of counseling quality

provided by community pharmacies to type 2 diabetic adult patients for oral therapy: a simulated patient study from Pakistan. Endocrine Journal. 2019;66(3):233-9. doi: 10.1507/endocrj.EJ18-0429. PubMed PMID: WOS:000466708900005.

3666. Aziz MT, Ur-Rehman T, Qureshi S, Bukhari NI. Reduction in chemotherapy order errors with computerised physician order entry and clinical decision support systems. Health Information Management Journal. 2015;44(3):13-32. doi: 10.1177/183335831504400303. PubMed PMID: 110412639. Language: English. Entry Date: 20151023. Revision Date: 20190103. Publication Type: Article.

3667. Aziz YHA, Heydon SJ, Duffull SB, Marra CA. Are professional pharmacy services being offered for free in pharmacies? A feasibility study exploring the use of a time motion study in New Zealand. Pharmacy Practice-Granada. 2021;19(3). doi: 10.18549/PharmPract.2021.3.2422. PubMed PMID: WOS:000680679500002.

3668. Aziz YHA, Heydon SJ, Duffull SB, Marra CA. What free services do pharmacists offer? Investigating the provision of unfunded pharmacy services in community pharmacies. Research in Social & Administrative Pharmacy. 2021;17(3):588-94. doi: 10.1016/j.sapharm.2020.05.007. PubMed PMID: WOS:000617989800013.

3669. Aziz YHA, Heydon SJ, Duffull SB, Marra CA. What do pharmacy users think of free pharmacy services? Investigating pharmacy users' perceptions, attitudes and willingness to pay for free healthcare from pharmacies. Exploratory Research in Clinical and Social Pharmacy. 2023;11. doi: 10.1016/j.rcsop.2023.100288. PubMed PMID: WOS:001133364700001.

3670. Azizi M, Dodhia V, Wan H, Ng W, Bhuva N, Ahmed R. Pharmacogenomic testing for dihydropyrimidine dehydrogenase polymorphisms in patients receiving fluoropyrimidine therapies at Mount Vernon Cancer Centre. Journal of Oncology Pharmacy Practice. 2023;29(1 Supplement):5-6. doi: https://dx.doi.org/10.1177/10781552231153542. PubMed PMID: 641008818.

3671. Azman AS, Lessler J, Satter SM, McKay MV, Khan A, Ahmed D, et al. Tracking Cholera through Surveillance of Oral Rehydration Solution Sales at Pharmacies: Insights from Urban Bangladesh. Plos Neglected Tropical Diseases. 2015;9(12). doi: 10.1371/journal.pntd.0004230. PubMed PMID: WOS:000368345100020.

3672. Azuma T, Akashi Y, Kanno M, Kobayashi S, Koyama H, Yamada Y. O2-12-2 - Identification of Issues on Chemotherapy by Multidisciplinary Workshop: New Attempt for Standardization in Rural Area. Annals of Oncology. 2014;25:v64. doi: https://doi.org/10.1093/annonc/mdu435.84.

3673. Azzi E, Kritikos V, Peters M, Price D, Cvetkovski B, Alphonse PS, et al. Perceptions, attitudes, and behaviors of short-acting beta2 agonist users: an Australian cross-sectional community pharmacy-based study. Journal of Asthma. 2022;59(1):178-88. doi: 10.1080/02770903.2020.1841223.

3674. Azzi E, Srour P, Armour C, Rand C, Bosnic-Anticevich S. Practice makes perfect: self-reported adherence a positive marker of inhaler technique maintenance. Npj Primary Care Respiratory Medicine. 2017;27. doi: 10.1038/s41533-017-0031-0. PubMed PMID: WOS:000401022000001.

3675. Azzi EA, Kritikos V, Peters MJ, Price DB, Srour P, Cvetkovski B, et al. Understanding reliever overuse in patients purchasing over-the-counter short-acting beta2 agonists: an Australian community pharmacy-based survey. BMJ open. 2019;9(8):e028995.

3676. Azziz-Baumgartner E, Wolkin A, Sanchez C, Bayleyegn T, Young S, Kieszak S, et al. Impact of Hurricane Ivan on pharmacies in Baldwin County, Alabama. Journal of the American Pharmacists Association. 2005;45(6):670-5. doi: 10.1331/154434505774909634.

3677. Azzopardi LM. Validation instruments for community pharmacy: pharmaceutical care for the third millennium: CRC Press; 2000.

3678. Azzopardi LM. Medications management in older persons: what can be achieved in the international community? Home Health Care Services Quarterly. 2005;24(1/2):137-46. PubMed PMID: 106379818. Language: English. Entry Date: 20060120. Revision Date: 20150820. Publication Type: Journal Article. Journal Subset: Blind Peer Reviewed.

3679. Azzopardi LM. Pharmacy practice in western Europe. Encyclopedia of Pharmacy Practice and Clinical Pharmacy2019. p. 478-87.

3680. Azzopardi LM. Chapter 16: How to Become a Successful Hospital and Community Pharmacist. Career Options in the Pharmaceutical and Biomedical Industry: An Insider's Guide2023. p. 263-87.

3681. Azzopardi LM, Bason L. Comparison of patient knowledge regarding diabetes between health centre patients and the central Diabetic Clinic at Mater Dei Hospital. International journal of clinical pharmacy Conference: nordic social pharmacy and health services research conference and the nordic networking group of clinical pharmacy Estonia. 2015;37(1 Supplement 1):8. doi: 10.1007/s11096-015-0121-4. PubMed PMID: CN-01375657.

3682. Azzopardi LM, Grech L, Rogers M. Pharmaceutical care issues of rheumatoid arthritis patients. Pharmaceutical Care Issues of Patients with Rheumatoid Arthritis: From Hospital to Community2016. p. 53-69.

3683. Azzopardi LM, Salek S, Inglott AS, Adami MZ, Buhagiar A. Validating tools for the monitoring of community pharmacy services. Pharmaceutical Journal. 2001;267(7163):303-5.

3684. Azzopardi LM, Serracino-Inglott A, Salek S, Zarb-Adami M. Development of External Methods to Evaluate the Quality of Pharmacy Services Offered by Community Pharmacists. Quality Assurance Journal. 2003;7(4):248-57. doi: 10.1002/qaj.242.

3685. Azzopardi LM, Serracino-Inglott A, Zarb-Adami M, Zerafa N. Pharmacist intervention in diabetic patient care in the community pharmacy. Pharmacy World & Science. 2007;29(5):474-. PubMed PMID: WOS:000249408200103.

3686. B.B. Med use widens in kids with ADHD. Science News. 2002;161(22):350. doi: 10.2307/4013334. PubMed PMID: 6750779.

3687. Ba SBYSY. Research on Construction and Performance of New Rotary Vane Pump [M.Eng.]. Peoples Rep. of China: Northeastern University (People's Republic of China); 2005.

3688. Ba YSBYS. Research on construction and performance of new rotary vane pump [Master]. Peoples Rep. of China: Northeastern University (People's Republic of China); 2008.

3689. Baalmann A, Crowl A, Coffey C, Jernigan S, Kalender-Rich J, Sabata D, et al. Interprofessional Medication Error Disclosure Training Using a Telehealth Consultation Simulation. American journal of pharmaceutical education. 2023;87(1):ajpe8799. doi: https://dx.doi.org/10.5688/ajpe8799. PubMed PMID: 637343732.

3690. Baba R, Nakachi I, Masaki K, Amagai T, Ohno R, Takaoka H, et al. Repetitive instructions at short intervals contribute to the improvement of inhalation technique. Asia Pacific Allergy. 2020;10(2). doi: 10.5415/apallergy.2020.10.e19. PubMed PMID: WOS:000531000400008.

3691. Babadagli H, Koshman S, Graham M, Wang M, Pearson G. PHARMACIST-LED SHORT TERM FOLLOW-UP PILOT PROGRAM FOR ACUTE CORONARY SYNDROME PATIENTS: A RETROSPECTIVE COHORT STUDY. Canadian Journal of Diabetes. 2023;47(7 Supplement):S67. doi: https://dx.doi.org/10.1016/j.jcjd.2023.10.132. PubMed PMID: 2027989876.

3692. Babaei K, Khatiban M, Khazaei M, Tapak L, Shamsaei F. Self-Management and Its Predictors in adult Patients with Epilepsy: A Cross-Sectional Study. Ethiopian Journal of Health Sciences. 2023;33(3):507-14. doi: 10.4314/ejhs.v33i3.14. PubMed PMID: 164270707.

3693. Babamir SM, Arabfard M. Improving Service Accessibility in Service-Oriented HIS. Journal of Medical Systems. 2012;36(6):4021-30. doi: 10.1007/s10916-012-9874-7. PubMed PMID: WOS:000309348200057.

3694. Babar Z-U-D, Grover P, Stewart J, Hogg M, Short L, Seo HG, et al. Evaluating pharmacists’ views, knowledge, and perception regarding generic medicines in New Zealand. Research in Social and Administrative Pharmacy. 2011;7(3):294-305. doi: https://doi.org/10.1016/j.sapharm.2010.06.004.

3695. Babar Z-U-D, Jamshed S, Yeong SW, Ibrahim MIM. Examination of students' interest in a public health pharmacy course in Malaysia. Currents in Pharmacy Teaching and Learning. 2011;3(3):199-207. doi: https://doi.org/10.1016/j.cptl.2011.04.011.

3696. Babar ZU, Polwin A, Kan SW, Amerasinghe N, McCarthy S, Rasheed F, et al. Exploring pharmacists' opinions regarding PHARMAC's interventions in promoting brand changes. Research In Social & Administrative Pharmacy. 2015;11(1):96-110. PubMed PMID: 24811426.

3697. Babar ZUD. The need for an evidence-based encyclopaedia in health services research in pharmacy. International Journal of Environmental Research and Public Health. 2020;17(7). doi: 10.3390/ijerph17072549.

3698. Babar ZUD. Ten recommendations to improve pharmacy practice in low and middle-income countries (LMICs). Journal of Pharmaceutical Policy and Practice. 2021;14(1). doi: 10.1186/s40545-020-00288-2.

3699. Babar ZUD, Grover P, Stewart J, Hogg M, Short L, Seo HG, et al. Evaluating pharmacists' views, knowledge, and perception regarding generic medicines in New Zealand. Research in Social and Administrative Pharmacy. 2011;7(3):294-305. doi: 10.1016/j.sapharm.2010.06.004.

3700. Babar ZUD, Hassali MAA, Shyong TL, Hin TK, Cien CS, Bin LS, et al. An evaluation of consumers' perceptions regarding "modern medicines" in Penang, Malaysia. Journal of Young Pharmacists. 2012;4(2):108-13. doi: 10.4103/0975-1483.96625.

3701. Babar ZUD, Ibrahim MIM, Singh H, Bukahri NI, Creese A. Evaluating drug prices, availability, affordability, and price components: Implications for access to drugs in Malaysia. PLoS Medicine. 2007;4(3):466-75. doi: 10.1371/journal.pmed.0040082.

3702. Babar ZUD, Kousar R, Murtaza G, Azhar S, Khan SA, Curley L. Randomized controlled trials covering pharmaceutical care and medicines management: A systematic review of literature. Research in Social and Administrative Pharmacy. 2018;14(6):521-39. doi: 10.1016/j.sapharm.2017.06.008.

3703. Babar ZUD, Lessing C, Mace C, Bissell K. The availability, pricing and affordability of three essential asthma medicines in 52 low- and middle-income countries. PharmacoEconomics. 2013;31(11):1063-82. doi: 10.1007/s40273-013-0095-9.

3704. Babar ZUD, Pengelly K, Scahill SL, Garg S, Shaw J. Migrant health in New Zealand: Exploring issues

concerning medicines access and use. Journal of Pharmaceutical Health Services Research. 2013;4(1):41-9. doi: 10.1111/j.1759-8893.2012.00105.x.

3705. Babar ZUD, Scahill S, Nagaria RA, Curley LE. The future of pharmacy practice research - Perspectives of academics and practitioners from Australia, NZ, United Kingdom, Canada and USA. Research in Social & Administrative Pharmacy. 2018;14(12):1163-71. doi: 10.1016/j.sapharm.2018.01.004. PubMed PMID: WOS:000449715400008.

3706. Babazono A, Kuwabara K, Hagiihara A, Nagano J, Ishihara R. Do interventions to prevent lifestyle-related diseases reduce healthcare expenditures? A randomized controlled clinical trial. Journal of epidemiology. 2011;21(1):75-80. doi: 10.2188/jea.je20100095. PubMed PMID: CN-00770814.

3707. Babb VJ, Babb J. Pharmacist Involvement in Healthy People 2010. Journal of the American Pharmaceutical Association (1996). 2003;43(1):56-60. doi: https://doi.org/10.1331/10865800360467051.

3708. Babbrah P, Solomon MR, Stember LA, Hill JW, Weiker M. Formulary & benefit and real-time pharmacy Benefit: Electronic standards delivering value to prescribers and pharmacists. Journal of the American Pharmacists Association. 2023;63(3):725-30. doi: https://dx.doi.org/10.1016/j.japh.2023.01.016. PubMed PMID: 2023027404.

3709. Babeaux HPF, Hall LE, Seifert JL. Charitable pharmacy services: Impact on patient-reported hospital use, medication access, and health status. Journal of the American Pharmacists Association. 2015;55(1):59-66. doi: 10.1331/JAPhA.2015.14010.

3710. Babic R, Babic D, Martinac M, Pavlovic M, Vasilj I, Miljko M, et al. ADDICTIONS WITHOUT DRUGS: CONTEMPORARY ADDICTIONS OR WAY OF LIFE? Psychiatria Danubina. 2018;30:S371-S9. PubMed PMID: WOS:000445620800006.

3711. Babić T. Sources of drug information used by pharmacists in croatia. Medicus. 2017;26(1):95-102.

3712. Babiker AH, Carson L, Awaisu A. Medication use review in Qatar: Are community pharmacists prepared for the extended professional role? International Journal of Clinical Pharmacy. 2014;36(6):1241-50. doi: 10.1007/s11096-014-0025-8.

3713. Babiker HM, Davis L, Larson K, Placencia C, Swensen C, Tenneti P, et al. A Multidisciplinary Evaluation of Barriers to Enrolling Cancer Patients into Early Phase Clinical Trials: Challenges and Patient-centric Recommendations. Expert Opinion on Investigational Drugs. 2019;28(8):675-86. doi: 10.1080/13543784.2019.1646726. PubMed PMID: WOS:000479628800001.

3714. Babiker HM, Davis L, Larson K, Placencia C, Swensen C, Tenneti P, et al. A Multidisciplinary Evaluation of Barriers to Enrolling Cancer Patients into Early Phase Clinical Trials: Challenges and Patient-centric Recommendations. Expert Opinion on Investigational Drugs. 2019;28(8):675-86. doi: 10.1080/13543784.2019.1646726.

3715. Babiker HM, McBride A, Newton M, Boehmer LM, Drucker AG, Gowan M, et al. Cardiotoxic effects of chemotherapy: A review of both cytotoxic and molecular targeted oncology therapies and their effect on the cardiovascular system. Critical Reviews in Oncology/Hematology. 2018;126:186-200. doi: https://doi.org/10.1016/j.critrevonc.2018.03.014.

3716. Babirye M, Yadesa TM, Tamukong R, Obwoya PS. Prevalence and factors associated with drug therapy problems among hypertensive patients at hypertension clinic of Mbarara Regional Referral Hospital, Uganda: a| cross-sectional study. Therapeutic Advances in Cardiovascular Disease. 2023;17:17539447231160319.

3717. Babonji A, Darwesh B, Al-alwai M. Implementation of pharmacist-managed early switch from intravenous to oral therapy using electronic identification at a tertiary academic hospital. Saudi Pharmaceutical Journal. 2021;29(4):324-36. doi: https://doi.org/10.1016/j.jsps.2021.03.006.

3718. Babu SSRS, Subbaiah S, Paniot S, Ramesh S, Nagasubramanian VR. Impact of practice settings on knowledge, attitude, and practice of disposal of unwanted pharmaceuticals among pharmacists in a selected city of Tamil Nadu. Journal of Applied Pharmaceutical Science. 2021;11(12):151-7. doi: 10.7324/JAPS.2021.1101214.

3719. Babu T, Mathew Panachiyil G, Sebastian J, Dhati Ravi M. Prescribing patterns and drug-related problems (DRPs) in transfusion-dependent paediatric thalassemia patients: a prospective interventional study from a tertiary care hospital in India: prescribing Patterns and DRPs in Transfusion-Dependent Paediatric Thalassemia Patients. International journal of pediatrics and adolescent medicine. 2021;8(1):35-8. doi: 10.1016/j.ijpam.2020.01.001. PubMed PMID: CN-02263864.

3720. Bacchelli M, Ajolfi C, Sangiorgi Cellini G, Guerra G, Pastorelli MC, Apollonio R, et al. Monitoring drug distribution at discharge to increase quality and decrease costs. Giornale Italiano di Farmacia Clinica. 2003;17(4):89-95.

3721. Bacchini M, Bonometti S, Del Zotti F, Lechi A, Realdon F, Fava C, et al. Opportunistic Screening for Atrial Fibrillation in the Pharmacies: A Population-Based Cross-Sectional Study. High Blood Pressure and Cardiovascular Prevention. 2019;26(4):339-44. doi: 10.1007/s40292-019-00334-4.

3722. Bacci JL, Akinwale TP, Adams AJ, McGivney MS. An analysis of community pharmacy shared faculty members' contributions to teaching, service, and scholarship. American Journal of Pharmaceutical Education. 2016;80(7).

3723. Bacci JL, Berenbrok LA. Innovative Advances in Connectivity and Community Pharmacist Patient Care Services: Implications for Patient Safety. Pharmacotherapy. 2018;38(8):867-74. doi: 10.1002/phar.2148.

3724. Bacci JL, Coley KC, McGrath K, Abraham O, Adams AJ, McGivney MS. Strategies to facilitate the implementation of collaborative practice agreements in chain community pharmacies. Journal of the American Pharmacists Association. 2016;56(3):257-65.e2. doi: https://doi.org/10.1016/j.japh.2016.02.014.

3725. Bacci JL, Coley KC, McGrath K, Abraham O, Adams AJ, McGivney MS, editors. Strategies to facilitate the implementation of collaborative practice agreements in chain community pharmacies. Journal of the American Pharmacists Association; 2016.

3726. Bacci JL, Ferreri SP, Coley KC, Daly CJ, Hake KL, Herbert SMC, et al. Qualitative analysis of a community pharmacy practice transformation initiative. Journal of the American College of Clinical Pharmacy. doi: 10.1002/jac5.1728. PubMed PMID: WOS:000888620300001.

3727. Bacci JL, Ferreri SP, Coley KC, Daly CJ, Hake KL, Herbert SMC, et al. Qualitative analysis of a community pharmacy practice transformation initiative. JACCP Journal of the American College of Clinical Pharmacy. 2022;5(12):1236-52. doi: 10.1002/jac5.1728.

3728. Bacci JL, Hansen R, Ree C, Reynolds MJ, Stergachis A, Odegard PS. The effects of vaccination forecasts and value-based payment on adult immunizations by community pharmacists. Vaccine. 2019;37(1):152-9. doi: 10.1016/j.vaccine.2018.11.018.

3729. Bacci JL, Marcum ZA, Rodriguez P, Hansen RN, Bansal A, Pfund T, et al. Community pharmacist intervention to optimize statin adherence in diabetes care: The GuIDE-S study. Journal of the American Pharmacists Association. 2023;63(3):946-51. doi: https://doi.org/10.1016/j.japh.2023.03.002.

3730. Bacci JL, McGrath SH, Pringle JL, Maguire MA, McGivney MS. Implementation of targeted medication adherence interventions within a community chain pharmacy practice: The Pennsylvania Project. Journal of the American Pharmacists Association: JAPhA. 2014;54(6):584-93. PubMed PMID: 25379980.

3731. Bacci JL, Odegard P, Arnold J, Stergachis A. Strengthening pandemic preparedness through pharmacy and public health collaborations: Findings from a facilitated discussion exercise. Journal of the American Pharmacists Association. 2021;61(3):e99-e106. doi: https://doi.org/10.1016/j.japh.2020.12.024.

3732. Bacci JL, Odegard P, Arnold J, Stergachis A, editors. Strengthening pandemic preparedness through pharmacy and public health collaborations: Findings from a facilitated discussion exercise. Journal of the American Pharmacists Association; 2021.

3733. Bacci JL, Rodriguez T, Hansen R, Marcum ZA, Bansal A, Pfund T, et al. Community pharmacist intervention to close statin gaps in diabetes care: The GuIDE-S study. Journal of the American Pharmacists Association. 2023;63(1):108-17. doi: https://doi.org/10.1016/j.japh.2022.08.025.

3734. Bacci JL, Rodriguez T, Hansen R, Marcum ZA, Bansal A, Pfund T, et al. Community pharmacist intervention to close statin gaps in diabetes care: The GuIDE-S study. Journal of the American Pharmacists Association. 2023;63(1):108-17. doi: https://doi.org/10.1016/j.japh.2022.08.025.

3735. Bacci JL, Zaraa S, Stergachis A, Simic G, White HS. Community pharmacists' role in caring for people living with epilepsy: A scoping review. Epilepsy and Behavior. 2021;117 (no pagination). PubMed PMID: 2011107143.

3736. Bacci JL, Zaraa S, Stergachis A, Simic G, White HS. Stakeholder perceptions of community pharmacist population health management of people living with epilepsy. Epilepsy & Behavior. 2021;125. doi: 10.1016/j.yebeh.2021.108389. PubMed PMID: WOS:000719409400014.

3737. Bach P, Hartung D. Leveraging the role of community pharmacists in the prevention, surveillance, and treatment of opioid use disorders. Addiction Science & Clinical Practice. 2019;14(1):30. PubMed PMID: 31474225.

3738. Bach QN, Peasah SK, Barber E. Review of the Role of the Pharmacist in Reducing Hospital Readmissions. Journal of Pharmacy Practice. 2019;32(6):617-24. PubMed PMID: 29618283.

3739. Bachyrycz A, Shrestha S, Bleske BE, Tinker D, Bakhireva LN. Opioid overdose prevention through pharmacy-based naloxone prescription program: Innovations in health care delivery. Substance Abuse. 2017;38(1):55-60. doi: 10.1080/08897077.2016.1184739.

3740. Bachyrycz A, Takeda MY, Wittstrom K, Bleske B. Opioid overdose response training in pharmacy education: An analysis of students' perception of naloxone use for opioid overdose prevention. Currents in Pharmacy Teaching and Learning. 2019;11(2):166-71. doi: https://doi.org/10.1016/j.cptl.2018.11.007.

3741. Bacic-Vrca V, Marusic S, Erdeljic V, Falamic S, Gojo-Tomic N, Rahelic D. The incidence of potential drug-drug interactions in elderly patients with arterial hypertension. Pharmacy World and Science. 2010;32(6):815-21. doi: 10.1007/s11096-010-9442-5.

3742. Back SE, Gros DF, McCauley JL, Flanagan JC, Cox E, Barth KS, et al. Laboratory-induced cue reactivity among individuals with prescription opioid dependence. Addictive Behaviors. 2014;39(8):1217-23. doi: https://doi.org/10.1016/j.addbeh.2014.04.007.

3743. Backes A, Rizos A, Pricor L. Primary medication adherence among patients transitioning from hospital to home care. Journal of the american pharmacists association. 2012;52(2):205-6. doi: 10.1331/

JAPhA.2012.12510. PubMed PMID: CN-01011670.

3744. Backes AC, Cash P, Jordan J. Transitional Care Units: Expanding the Role of Pharmacists Providing Patient Care. Consultant Pharmacist. 2016;31(1):13-24. PubMed PMID: 26803087.

3745. Backes AC, Kuo GM. The association between functional health literacy and patient-reported recall of medications at outpatient pharmacies. Research in Social and Administrative Pharmacy. 2012;8(4):349-54. PubMed PMID: 51744750.

3746. Backes G, Rose VJ. Primary and secondary analysis of local elected officials' decisions to support or oppose pharmacy sale of syringes in California. Journal of Urban Health. 2010;87(4):553-60. doi: 10.1007/s11524-010-9442-8.

3747. Backes J, Padley R, Ruisinger J, Acree L, Luoma J, Moriarty P. Dietary supplement versus prescription niacin products: differentiation and important considerations for use in patients with dyslipidemia. Journal of the american pharmacists association. 2012;52(2):207-. doi: 10.1331/JAPhA.2012.12510. PubMed PMID: CN-01011669.

3748. Backes K, Griesbach S, BCACP A-C, Wilhelm S. Identification of drug therapy opportunities with oral chemotherapy. Specialty Pharmacy Times. 2013;4(3).

3749. Backman C, Cho-Young D. Engaging patients and informal caregivers to improve safety and facilitate person-and family-centered care during transitions from hospital to home–a qualitative descriptive study. Patient preference and adherence. 2019:617-26.

3750. Backman R, Foy R, Diggle PJ, Kneen R, Easton A, Defres S, et al. A pragmatic cluster randomised controlled trial of a tailored intervention to improve the initial management of suspected encephalitis. PloS one. 2018;13(12):e0202257. doi: 10.1371/journal.pone.0202257. PubMed PMID: CN-01736897.

3751. Backman R, Weber P, Turner AM, Lee M, Litchfield I. Assessing the extent of drug interactions among patients with multimorbidity in primary and secondary care in the West Midlands (UK): A study protocol for the Mixed Methods Multimorbidity Study (MiMMS). BMJ Open. 2017;7(9). doi: 10.1136/bmjopen-2017-016713.

3752. Backran D, Ahmad S, Hansen J, Almarsdottir AB, Jacobsen R. Pregnancy prevention while using oral retinoids: a questionnaire survey in Denmark. International Journal of Clinical Pharmacy. 2023;45(2):521. doi: https://dx.doi.org/10.1007/s11096-023-01576-y. PubMed PMID: 641388313.

3753. Backstrom M, Hagg S, Mjorndal T, Dahlqvist R. Utilization pattern of metamizole in northern Sweden and risk estimates of agranulocytosis. Pharmacoepidemiology and Drug Safety. 2002;11(3):239-45. doi: 10.1002/pds.697. PubMed PMID: WOS:000175637000009.

3754. Bacon B, Curry M, Dieterich D, Flamm S, Kowdley K, Milligan S, et al. Real-world use of elbasvir/grazoprevir and outcomes in patients with chronic hepatitis C: retrospective data analyses from the TRIO Network. Journal of hepatology Conference: international liver congress 2017 - 52nd annual meeting of the european association for the study of the liver Netherlands. 2017;66(1 Supplement 1):S295. PubMed PMID: CN-01469203.

3755. Bacon L, Savage I, Cook S, Taylor B. Training and supporting pharmacists to supply progestogen-only emergency contraception. Journal of Family Planning and Reproductive Health Care. 2003;29(2):17-22. doi: 10.1783/147118903101197476. PubMed PMID: WOS:000182671000004.

3756. Bacon M, Kematick B, Scullion B, Lally K, Suliman I. Development of PharmPAL: A Collaborative

Practice Pharmacy Clinic in an Ambulatory Palliative Care Clinic. Journal of Pain and Symptom Management. 2022;63(5):860. doi: https://doi.org/10.1016/j.jpainsymman.2022.02.044.

3757. Bacon M, Kematick B, Scullion BC, Lally K. We Can Be Your PAL, Too: A Deeper Look into a Collaborative Practice Pharmacist-Led Clinic in Ambulatory Palliative Care (CS367). Journal of Pain and Symptom Management. 2023;65(5):e574. doi: https://dx.doi.org/10.1016/j.jpainsymman.2023.02.129. PubMed PMID: 2023950223.

3758. Bacon M, Kematick B, Suliman I, Scullion B, Chua I, Given S, et al. Development of PharmPAL: A Collaborative Practice Pharmacy Clinic in Ambulatory Palliative Care. American Journal of Hospice & Palliative Medicine. doi: 10.1177/10499091221120593. PubMed PMID: WOS:000849431200001.

3759. Bacon S, Khan N. Integration of patients, caregivers and healthcare providers into a hypertension management research priorities process. Trials. 2017;18. doi: 10.1186/s13063-017-1902-y. PubMed PMID: CN-01470734.

3760. Badcott D. Professional values in community and public health pharmacy. Medicine, Health Care & Philosophy. 2011;14(2):187-94. PubMed PMID: 20803257.

3761. Badenhorst J, Todd A, Lindsey L, Ling J, Husband A. Widening the scope for early cancer detection: identification of alarm symptoms by community pharmacies. International Journal of Clinical Pharmacy. 2015;37(3):465-70. doi: 10.1007/s11096-015-0078-3.

3762. Bader L, Kusynová Z, Duggan C. FIP Perspectives: Realising global patient safety goals requires an integrated approach with pharmacy at the core. Research in Social & Administrative Pharmacy. 2019;15(7):815-7. doi: 10.1016/j.sapharm.2019.02.012. PubMed PMID: 136980151. Language: English. Entry Date: In Process. Revision Date: 20200325. Publication Type: journal article. Journal Subset: Biomedical.

3763. Bader MD, Ailshire JA, Morenoff JD, House JS. Measurement of the local food environment: a comparison of existing data sources. American journal of epidemiology. 2010;171(5):609-17.

3764. Badger F, Kingscote-Davies T, Nolan P. The pharmacist's role in the medicinal management of depression. Nursing standard (Royal College of Nursing (Great Britain) : 1987). 2002;16(47):33-40. doi: 10.7748/ns2002.08.16.47.33.c3243.

3765. Badger N, Mullis S, Butler K, Tucker D. Pharmacist's intervention for older hospitalized patients. American Journal of Health-System Pharmacy. 2007;64(17):1794-6. doi: 10.2146/ajhp070074.

3766. Badgwell B, Krouse R, Klimberg SV, Bruera E. Outcome Measures Other than Morbidity and Mortality for Patients with Incurable Cancer and Gastrointestinal Obstruction. Journal of Palliative Medicine. 2014;17(1):18-26. doi: 10.1089/jpm.2013.0240. PubMed PMID: WOS:000330634100008.

3767. Badiu R, Bucsa C, Mogosan C, Dumitrascu D. Statin drug-drug interactions in a romanian community pharmacy. Clujul Medical. 2016;89(2):273-8. doi: 10.15386/cjmed-569.

3768. Badoum G, Toloba Y, Thiombiano R, Ouedraogo G, Ouedraogo M. Attitude of private pharmacists in the dispensation of asthma drugs. Revue Des Maladies Respiratoires. 2015;32(1):18-23. doi: 10.1016/j.rmr.2013.09.019. PubMed PMID: WOS:000351718400003.

3769. Badowski M. Examining the implications of analytical and remote monitoring in pharmacy practice.

3770. Badowski M, Mazur JE, Lam SW, Miyares M, Schulz L, Michienzi S. Engaging in collaborative

research: Focus on the pharmacy practitioner. Hospital Pharmacy. 2017;52(1):33-43. doi: 10.1310/hpj5201-33.

3771. Badowski ME, Walker S, Bacchus S, Bartlett S, Chan J, Cochran KA, et al. Providing Comprehensive Medication Management in Telehealth. Pharmacotherapy:The Journal of Human Pharmacology & Drug Therapy. 2018;38(2):e7-e16. PubMed PMID: 29239004.

3772. Badr AF, Humedi RA, Alfarsi NA, Alghamdi HA. Rapid antigen detection test (RADT) for pharyngitis diagnosis in children: Public and pharmacist perception. Saudi Pharmaceutical Journal. 2021;29(7):677-81. doi: 10.1016/j.jsps.2021.04.029. PubMed PMID: WOS:000681733900005.

3773. Badr AF, Kurdi S, Alshehri S, McManus C, Lee J. Pharmacists' interventions to reduce sedative/hypnotic use for insomnia in hospitalized patients. Saudi pharmaceutical journal. 2018;(no pagination). doi: 10.1016/j.jsps.2018.07.010. PubMed PMID: CN-01618107.

3774. Badreldin HA, Alreshoud L, Altoukhi R, Vasudevan S, Isamil W, Mohamed MSA. Prevalence and predictors of inappropriate apixaban dosing in patients with non-valvular atrial fibrillation at a large tertiary academic medical institution. Drugs & Therapy Perspectives. 2020;36(2):83-8. doi: 10.1007/s40267-019-00696-8. PubMed PMID: 141414631. Language: English. Entry Date: 20200130. Revision Date: 20200201. Publication Type: Article.

3775. Badreldin HA, Atallah B. Global drug shortages due to COVID-19: impact on patient care and mitigation strategies. Research in Social and Administrative Pharmacy. 2021;17(1):1946-9.

3776. Badro DA, Sacre H, Hallit S, Amhaz A, Salameh P. Good pharmacy practice assessment among community pharmacies in Lebanon. Pharmacy Practice (Granada). 2020;18(1).

3777. Badyal DK, Desai C. Animal use in pharmacology education and research: The changing scenario. Indian journal of pharmacology. 2014;46(3):257.

3778. Bae SJ, Paltiel AD, Fuhlbrigge AL, Weiss ST, Kuntz KM. Modeling the Potential Impact of a Prescription Drug Copayment Increase on the Adult Asthmatic Medicaid Population. Value in Health. 2008;11(1):110-8. doi: https://doi.org/10.1111/j.1524-4733.2007.00219.x.

3779. Bae Y, Worley M, Law A. Use of the modified drug adherence work-up tool to identify and address barriers to medication adherence. Journal of the american pharmacists association Conference: apha2016 Baltimore, MD united states Conference start: 20160304 Conference end: 20160307 Conference publication: (varpagings). 2016;56(3):e15-e6. doi: 10.1016/j.japh.2016.03.014. PubMed PMID: CN-01472414.

3780. Bae YHJ, Mullins CD. Do Value Thresholds for Oncology Drugs Differ from Nononcology Drugs? Journal of Managed Care & Specialty Pharmacy. 2014;20(11):1086-92. doi: 10.18553/jmcp.2014.20.11.1086. PubMed PMID: WOS:000344515200002.

3781. Baek H. A spectral element method for fluid -structure interaction: New algorithm and applications to intracranial aneurysms [Ph.D.]. United States -- Rhode Island: Brown University; 2010.

3782. Baena Parejo MI, Juanes Borrego AM, Ruiz JA, Monjó MC, García-Peláez M, Hernanz BC, et al. Medication List Assessment in Spanish Hospital Emergency Departments. The Journal of Emergency Medicine. 2015;48(4):416-23. doi: https://doi.org/10.1016/j.jemermed.2014.06.063.

3783. Baergen R, Owens C. Revisiting pharmacists' refusals to dispense emergency contraception. Obstetrics and Gynecology. 2006;108(5):1277-82. doi: 10.1097/01.AOG.0000240136.60598.b8.

3784. Baeyens A. European Court of Justice. European Journal of Health Law. 2023;30(5):578-84. doi: 10.1163/15718093-12423559.

3785. Baftiu A, Feet SA, Larsson PG, Burns ML, Henning O, Sætre E, et al. Utilisation and polypharmacy aspects of antiepileptic drugs in elderly versus younger patients with epilepsy: A pharmacoepidemiological study of CNS-active drugs in Norway, 2004-2015. Epilepsy Research. 2018;139:35-42. doi: https://doi.org/10.1016/j.eplepsyres.2017.11.001.

3786. Bagattini ÂM, Borges JLA, Riera R, de Carvalho DCMF. Automation of a tertiary hospital pharmacy drug dispensing system in a lower-middle-income country: A case study and preliminary results. Exploratory Research in Clinical and Social Pharmacy. 2022;6:100151. doi: https://doi.org/10.1016/j.rcsop.2022.100151.

3787. Bagga SK. An Exploration of Healthcare Professionals' Attitudes and Perceptions Towards a Local Hospital Drug Formulary and Their Impact on Prescribing Practice [Ph.D.]. England: University of Derby (United Kingdom); 2021.

3788. Bagga SK, Town C. Is money really being devolved? (multiple letters) [1]. Pharmaceutical Journal. 2004;273(7329):849.

3789. Baggarly SA, Jenkins TL, Biglane GC, Smith GW, Smith CM, Blaylock BL. Implementing a referral to telephone tobacco cessation services in Louisiana community pharmacies: A pilot study. Annals of Pharmacotherapy. 2010;44(9):1395-402. doi: 10.1345/aph.1P226.

3790. Baggett ACL, Dorval E, Ballou JM, Dalton E, Rhodes LA. Barriers and best practices related to documentation of electronic care plans: A survey of community-based pharmacies in 4 states. Journal of the American Pharmacists Association. 2022;62(4):S11-+. doi: 10.1016/j.japh.2021.11.016. PubMed PMID: WOS:000824834300003.

3791. Baggs J, Gee J, Lewis E, Fowler G, Benson P, Lieu T, et al. The Vaccine Safety Datalink: A Model for Monitoring Immunization Safety. Pediatrics. 2011;127:S45-S53. doi: 10.1542/peds.2010-1722H. PubMed PMID: WOS:000296918100008.

3792. Baggs J, Yousey-Hindes K, Ashley ED, Meek J, Dumyati G, Cohen J, et al. Identification of population at risk for future Clostridium difficile infection following hospital discharge to be targeted for vaccine trials. Vaccine. 2015;33(46):6241-9. doi: https://doi.org/10.1016/j.vaccine.2015.09.078.

3793. Bagher AM, Neamatallah T, Balto G, Almikhy L, Almutairi SS, Abushal MO, et al. Knowledge, perception, and confidence of hospital pharmacists toward pharmacogenetics in Jeddah, Kingdom of Saudi Arabia. Saudi Pharmaceutical Journal. 2021;29(1):53-8. doi: https://doi.org/10.1016/j.jsps.2020.12.006.

3794. Bagheri H, Giroud JP. Automédication et mésusage. Bulletin de l'Académie Nationale de Médecine. 2023;207(2):178-85. doi: https://doi.org/10.1016/j.banm.2022.12.010.

3795. Bagheri H, Giroud JP. Automédication et mésusage. Bulletin de l'Académie Nationale de Médecine. 2023;207(2):178-85. doi: https://doi.org/10.1016/j.banm.2022.12.010.

3796. Bagheri I, Shan Y, Klaassen Z, Kamat AM, Konety B, Mehta HB, et al. Comparing Costs of Radical Versus Partial Cystectomy for Patients Diagnosed With Localized Muscle-Invasive Bladder Cancer: Understanding the Value of Surgical Care. Urology. 2021;147:127-34. doi: https://doi.org/10.1016/j.urology.2020.08.058.

3797. Bagley WP, Smith AA, Hebert JD, Snider CC, Sega GA, Piller MD, et al. A randomized, placebo controlled, trial of preoperative sustained release betamethasone plus non-controlled intraoperative ketorolac

or fentanyl on pain after diagnostic laparoscopy or laparoscopic tubal ligation. BMC anesthesiology. 2003;3. doi: 10.1186/1471-2253-3-3. PubMed PMID: CN-01752849.

3798. Bagonza A, Kitutu FE, Peterson S, Mårtensson A, Mutto M, Awor P, et al. Effectiveness of peer-supervision on pediatric fever illness treatment among registered private drug sellers in East-Central Uganda: An interrupted time series analysis. Health Science Reports. 2021;4(2). doi: 10.1002/hsr2.284.

3799. Bagonza A, Peterson S, Martensson A, Wamani H, Awor P, Mutto M, et al. Regulatory inspection of registered private drug shops in East-Central Uganda-what it is versus what it should be: a qualitative study. Journal of Pharmaceutical Policy and Practice. 2020;13(1). doi: 10.1186/s40545-020-00265-9. PubMed PMID: WOS:000570491600001.

3800. Bagonza A, Wamani H, Peterson S, Mårtensson A, Mutto M, Musoke D, et al. Peer supervision experiences of drug sellers in a rural district in East-Central Uganda: A qualitative study. Malaria Journal. 2020;19(1). doi: 10.1186/s12936-020-03343-0.

3801. Bagshaw SM, Stelfox HT, McDermid RC, Rolfson DB, Tsuyuki RT, Baig N, et al. Association between frailty and short-and long-term outcomes among critically ill patients: a multicentre prospective cohort study. Cmaj. 2014;186(2):E95-E102.

3802. Bagwell A, Kelley T, Carver A, Lee JB, Newman B. Advancing Patient Care Through Specialty Pharmacy Services in an Academic Health System. Journal of Managed Care & Specialty Pharmacy. 2017;23(8):815-20. PubMed PMID: 28737983.

3803. Bahadori M, Mohammadnejhad SM, Ravangard R, Teymourzadeh E. Using Queuing Theory and Simulation Model to Optimize Hospital Pharmacy Performance. Iranian Red Crescent Medical Journal. 2014;16(3). doi: 10.5812/ircmj.16807. PubMed PMID: WOS:000333961800027.

3804. Bahar MA, Hak E, Bos JHJ, Borgsteede SD, Wilffert B. The burden and management of cytochrome P450 2D6 (CYP2D6)-mediated drug–drug interaction (DDI): co-medication of metoprolol and paroxetine or fluoxetine in the elderly. Pharmacoepidemiology and Drug Safety. 2017;26(7):752-65. doi: 10.1002/pds.4200.

3805. Bahar MA, Hak E, Bos JHJ, Borgsteede SD, Wilffert B. The burden and management of cytochrome P450 2D6 (CYP2D6)-mediated drug-drug interaction (DDI): co-medication of metoprolol and paroxetine or fluoxetine in the elderly. Pharmacoepidemiology and Drug Safety. 2017;26(7):752-65. doi: 10.1002/pds.4200. PubMed PMID: WOS:000404723300003.

3806. Bahar MA, Wang YY, Bos JHJ, Wilffert B, Hak E. Discontinuation and dose adjustment of metoprolol after metoprolol-paroxetine/fluoxetine co-prescription in Dutch elderly. Pharmacoepidemiology and Drug Safety. 2018;27(6):621-9. doi: 10.1002/pds.4422. PubMed PMID: WOS:000434160600009.

3807. Baharuddin KA, Rahman NHN, Wahab SFA, Halim NA, Ahmad R. Intravenous parecoxib sodium as an analgesic alternative to morphine in acute trauma pain in the emergency department. International journal of emergency medicine. 2014;7(1). doi: 10.1186/1865-1380-7-2. PubMed PMID: CN-00959085.

3808. Bahkali NM, Ghamri KA, Aladwani LF, Alzaydi TH, Aljarid FA, Alamoudi NA, et al. Awareness of Pre-eclampsia and Measurement of Risk Factors in Women from the Western Region of Saudi Arabia: A Cross-sectional Study. International Journal of Womens Health and Reproduction Sciences. 2024;12(1):8-15. doi: 10.15296/ijwhr.2024.6002. PubMed PMID: WOS:001122476400001.

3809. Bähler C, Huber CA, Brüngger B, Reich O. Multimorbidity, health care utilization and costs in an elderly community-dwelling population: a claims data based observational study. BMC health services

research. 2015;15:1-12.

3810. Bahler C, Schoepfer AM, Vavricka SR, Brungger B, Reich O. Chronic comorbidities associated with inflammatory bowel disease: prevalence and impact on healthcare costs in Switzerland. European Journal of Gastroenterology & Hepatology. 2017;29(8):916-25. doi: 10.1097/meg.0000000000000891. PubMed PMID: WOS:000405092100008.

3811. Bahlol M, Bushell M, Khojah HMJ, Dewey RS. Spontaneous adverse drug reaction reporting by community pharmacists: preparedness and barriers. Saudi Pharmaceutical Journal. 2022;30(7):1052-9. doi: https://doi.org/10.1016/j.jsps.2022.04.006.

3812. Bahlol M, Dewey RS. Pandemic preparedness of community pharmacies for COVID-19. Research in Social and Administrative Pharmacy. 2021;17(1):1888-96. doi: 10.1016/j.sapharm.2020.05.009.

3813. Bahlol M, Sum ZZ, Dewey RS. Barriers to Community Pharmacists Referring Patients With Suspected COVID-19 Symptoms. Journal of Pharmacy Practice. doi: 10.1177/08971900221074952. PubMed PMID: WOS:000776097200001.

3814. Bahlol M, Sum ZZ, Dewey RS. Barriers to Community Pharmacists Referring Patients With Suspected COVID-19 Symptoms. Journal of Pharmacy Practice. 2023;36(4):783-94. doi: https://dx.doi.org/10.1177/08971900221074952. PubMed PMID: 2015412019.

3815. Bahnasawy S, El Beblawy N, El Wakeel L, El Hamamsy M. Clinical pharmacist-provided services in iron overloaded beta-thalassemia major children. A new insight to patient care. British journal of haematology. 2016;173:27-. doi: 10.1111/bjh.14019. PubMed PMID: CN-01360306.

3816. Bahnasawy SM, El Wakeel LM, Beblawy NE, El-Hamamsy M. Clinical Pharmacist-Provided Services In Iron-Overloaded Beta-Thalassaemia Major Children: a New Insight Into Patient Care. Basic & clinical pharmacology & toxicology. 2017;120(4):354-9. doi: 10.1111/bcpt.12695. PubMed PMID: CN-01335799.

3817. Bahnassi A. The effect of integration of pharmaceutical care components in pharmacy schools curricula on medication adherence in Syria. Pharmacotherapy. 2011;31(10):441e. PubMed PMID: CN-01035316.

3818. Bahnassi A. A qualitative analysis of pharmacists' attitudes and practices regarding the sale of antibiotics without prescription in Syria. Journal of Taibah University Medical Sciences. 2015;10(2):227-33.

3819. Bahnassi A. Pharmacists views and practices in regard to sales of antibiotics without a prescription in Madinah, Saudi Arabia. Journal of Patient Safety. 2016;12(3):159-64. doi: 10.1097/PTS.0000000000000087.

3820. Bahnassi A. Do no harm: The role of community pharmacists in regulating public access to prescription drugs in Saudi Arabia. International Journal of Pharmacy Practice. 2016;24(2):142-5. doi: 10.1111/ijpp.12224.

3821. Bahrami S, Chang C, Alvarez KS, Lutek K, Nguyen S, Hegde A. Pharmacist impact on health outcomes in a homeless population. Journal of the American Pharmacists Association. 2020;60(3):485-90. doi: 10.1016/j.japh.2019.11.013. PubMed PMID: WOS:000581116500017.

3822. Bahrani L, Eriksson T, Hoglund P, Midlov P. The rate and nature of medication errors among elderly upon admission to hospital after implementation of clinical pharmacist-led medication reconciliation. European Journal of Hospital Pharmacy-Science and Practice. 2014;21(3):156-60. doi: 10.1136/ejhpharm-2013-000403. PubMed PMID: WOS:000337695600006.

3823. Bahri P, Bowring G, Edwards BD, Anton C, Aronson JK, Caro-Rojas A, et al. Communicating for the Safe Use of Medicines: Progress and Directions for the 2020s Promoted by the Special Interest Group of the International Society of Pharmacovigilance. Drug Safety. 2023;46(6):517-32. doi: 10.1007/s40264-023-01285-5. PubMed PMID: 163942540.

3824. BAHS MHGM. The use of interprofessional simulation to improve collaboration and problem solving among undergraduate BHSc medical laboratory science and BScN nursing students. Canadian Journal of Medical Laboratory Science. 2020;82(2):25-33.

3825. Bahta M, Weldemariam DG, Tesfamariam S, Tesfamariam EH, Russom M. Determinants of dispensing antibiotics without prescription in Eritrea: A mixed-method qualitative study on pharmacy professionals' perspective. BMJ Open. 2021;11(8). doi: 10.1136/bmjopen-2021-049000.

3826. Bai G. Cell Permeability Studies of AApeptides and Novel Molecular Probes for AD [Ph.D.]. United States -- Florida: University of South Florida; 2016.

3827. Bai G, Armenante PM, Plank RV, Gentzler M, Ford K, Harmon P. Hydrodynamic Investigation of USP Dissolution Test Apparatus II. Journal of Pharmaceutical Sciences. 2007;96(9):2327-49. doi: https://doi.org/10.1002/jps.20818.

3828. Bai I, Isenor JE, Reeve E, Whelan AM, Martin-Misener R, Burgess S, et al. Using the behavior change wheel to link published deprescribing strategies to identified local primary healthcare needs. Research in Social and Administrative Pharmacy. 2022;18(8):3350-7. doi: https://doi.org/10.1016/j.sapharm.2021.12.001.

3829. Bai J. Interdisciplinary Research in Operations Management: Applications in Healthcare, Retailing and On-demand Service Platforms [Ph.D.]. United States -- California: University of California, Irvine; 2017.

3830. Bai X. Research on key technologies of pharmacy automation [Master]. Peoples Rep. of China: Beijing University of Aeronautics and Astronautics (People's Republic of China); 2010.

3831. Bai Y, Wu B, Gou L, Fang Z, Xu T, Zhang T, et al. Cardiovascular Safety Evaluation of Febuxostat and Allopurinol: Findings from the FDA Adverse Event Reporting System. Journal of Clinical Medicine. 2023;12(18):6089. doi: 10.3390/jcm12186089. PubMed PMID: 172415362.

3832. Bailey A, Geraghty L, Mason M, Weant K, Atchison L, Dewitt K, et al. Dosing strategies of N-acetylcysteine for acetaminophen overdose in patients greater than 100 kg. An evaluation of dosage capping. Clinical toxicology Conference: 2017 annual meeting of the north american congress of clinical toxicology, NACCT 2017 Canada. 2017;55(7):698-9. doi: 10.1080/15563650.2017.1348043. PubMed PMID: CN-01407009.

3833. Bailey A, Graham B, Harps M, Sedore G. Vancouver's Alcohol Knowledge Exchange: lessons learned from creating a peer-involved alcohol harm reduction strategy in Vancouver's Downtown Eastside. Harm Reduction Journal. 2023;20(1). doi: 10.1186/s12954-023-00838-2. PubMed PMID: WOS:001033871000001.

3834. Bailey AL, Moe G, Moe J, Oland R. Implementation and evaluation of a community-based medication reconciliation (CMR) system at the hospital-community interface of care. Healthcare quarterly (Toronto, Ont). 2009;13 Spec No:91-7. doi: 10.12927/hcq.2009.21105. PubMed PMID: CN-01754751.

3835. Bailey J, Rudman W. The expanding role of the HIM professional: where research and HIM roles intersect. Perspectives in Health Information Management. 2004;1:6p-p. PubMed PMID: 106458377. Language: English. Entry Date: 20060616. Revision Date: 20150818. Publication Type: Journal Article. Journal Subset: Allied Health.

3836. Bailey JE, Hajjar M, Shoib B, Tang J, Ray MM, Wan JY. Risk Factors Associated With Antihypertensive Medication Nonadherence in a Statewide Medicaid Population. American Journal of the Medical Sciences. 2014;348(5):410-5. doi: 10.1097/MAJ.0b013e31825ce50f. PubMed PMID: WOS:000344608400011.

3837. Bailey JE, Lee MD, Somes GW, Graham RL. Risk factors for antihypertensive medication refill failure by patients under Medicaid managed care. Clinical Therapeutics. 1996;18(6):1252-62. doi: https://doi.org/10.1016/S0149-2918(96)80080-4.

3838. Bailey JE, Surbhi S, Bell PC, Jones AM, Rashed S, Ugwueke MO. SafeMed: Using pharmacy technicians in a novel role as community health workers to improve transitions of care. Journal of the American Pharmacists Association. 2016;56(1):73-81. doi: https://doi.org/10.1016/j.japh.2015.11.011.

3839. Bailey JE, Wan JY, Tang J, Ghani MA, Cushman WC. Antihypertensive medication adherence, ambulatory visits, and risk of stroke and death. Journal of General Internal Medicine. 2010;25(6):495-503. doi: 10.1007/s11606-009-1240-1.

3840. Bailey L, Brewer L, Carroll J, Seebah S. SPECIALIST MEDICATION REVIEW AS PART OF THE COMPREHENSIVE GERIATRIC ASSESSMENT IN THE DAY HOSPITAL SETTING...British Geriatrics Society Autumn Meeting, November 25-27 2020 (Virtual). Age & Ageing. 2021;50:i1-i. doi: 10.1093/ageing/afab030.98. PubMed PMID: 149478820. Language: English. Entry Date: 20210329. Revision Date: 20220302. Publication Type: Article.

3841. Bailey L, Curington R, Brown B, Hegener M, Espel M. Motivational interviewing education: Creation and assessment of a learning module implemented among advanced pharmacy practice students. Currents in Pharmacy Teaching and Learning. 2017;9(5):786-93. doi: 10.1016/j.cptl.2017.06.007.

3842. Bailey LC, Barrett NR, Thorne M, Ford FM, Elizabeth W, Psevdos G. Successful Drive-thru Point-of-Distribution Influenza Vaccination Program for Veterans Affairs Medical Center Employees. American Journal of Infection Control. 2020;48(8, Supplement):S31. doi: https://doi.org/10.1016/j.ajic.2020.06.201.

3843. Bailey LC, Papamanoli A, Thorne M, Barrett NR, Ford FM, Elizabeth W, et al. Influenza Vaccination Challenges for Hospital Employees in the COVID Era: Experience of a Veterans Affairs Medical Center. American Journal of Infection Control. 2021;49(6, Supplement):S17. doi: https://doi.org/10.1016/j.ajic.2021.04.069.

3844. Bailey N, Kaarto P, Burkey J, Bright D, Sohn M. Evaluation of an American Sign Language co-curricular training for pharmacy students. Currents in Pharmacy Teaching and Learning. 2021;13(1):68-72. doi: 10.1016/j.cptl.2020.08.002.

3845. Bailey S, Usa T. Suits say placement of homeopathic items is misleading.

3846. Bailey SC. The Effect of Improved Labeling on Medication Understanding Among Limited English Proficient Adults [Ph.D.]. United States -- Illinois: University of Illinois at Chicago, Health Sciences Center; 2011.

3847. Bailey SC, Agarwal N, Sleath B, Gumusoglu S, Wolf MS. Improving Drug Labeling and Counseling for Limited English Proficient Adults. Journal of Health Care for the Poor and Underserved. 2011;22(4):1131-43. PubMed PMID: WOS:000297232600003.

3848. Bailey SC, Belter LT, Pandit AU, Carpenter DM, Carlos E, Wolf MS. The availability, functionality, and quality of mobile applications supporting medication self-management. Journal of the American Medical Informatics Association. 2014;21(3):542-6. doi: 10.1136/amiajnl-2013-002232. PubMed PMID:

WOS:000334611600022.

3849. Bailey SC, Pandit AU, Curtis L, Wolf MS. Availability of Spanish prescription labels: A multi-state pharmacy survey. Medical Care. 2009;47(6):707-10. doi: 10.1097/MLR.0b013e318195fd02.

3850. Bailey SC, Pandit AU, Yin S, Federman A, Davis TC, Parker RM, et al. Predictors of misunderstanding pediatric liquid medication instructions. Fam Med. 2009;41(10):715-21.

3851. Bailey SC, Sarkar U, Chen AH, Schillinger D, Wolf MS. Evaluation of language concordant, patient-centered drug label instructions. Journal of general internal medicine. 2012;27(12):1707-13. doi: 10.1007/s11606-012-2035-3. PubMed PMID: CN-00880761.

3852. Bailey SC, Sarkar U, Chen AH, Schillinger D, Wolf MS, Bailey SC, et al. Evaluation of language concordant, patient-centered drug label instructions. JGIM: Journal of General Internal Medicine. 2012;27(12):1707-13. doi: 10.1007/s11606-012-2035-3. PubMed PMID: 104389147. Language: English. Entry Date: 20130621. Revision Date: 20211028. Publication Type: journal article.

3853. Bailey SC, Schillinger D, Chen A, Sarkar U, Larsen E, Wolf M. Improving understanding of rx instructions among patients with limited English proficiency. Journal of general internal medicine. 2011;26:S352. doi: 10.1007/s11606-011-1730-9. PubMed PMID: CN-01035227.

3854. Bailey TC, Noirot LA, Blickensderfer A, Rachmiel E, Schaiff R, Kessels A, et al. An intervention to improve secondary prevention of coronary heart disease. Archives of internal medicine. 2007;167(6):586-90. doi: 10.1001/archinte.167.6.586. PubMed PMID: CN-00579538.

3855. Bailey TC, Noirot LA, Gage BF, Li X, Shannon WD, Waterman B, et al. Improving adherence to coronary heart disease secondary prevention medication guidelines at a community hospital. Amia Annual symposium proceedings AMIA symposium. 2006;2006:850. PubMed PMID: CN-00609135.

3856. Bailey TC, Ritchie DJ, McMullin ST, Kahn M, Reichley RM, Casabar E, et al. A randomized, prospective evaluation of an interventional program to discontinue intravenous antibiotics at two tertiary care teaching institutions. Pharmacotherapy. 1997;17(2):277-81. PubMed PMID: CN-00138097.

3857. Bailie GR, Romeo B. New York State primary care physicians' attitudes to community pharmacists' clinical services. Archives of Internal Medicine. 1996;156(13):1437-41. doi: 10.1001/archinte.156.13.1437.

3858. Bailin AG, Lai LL. Are Pharmacists in Mail Order Pharmacy Performing Pharmaceutical Care? Journal of Managed Pharmaceutical Care. 2001;1(3):61-9.

3859. Bailkin S. Simple solution to pharmacy's problems [3]. Drug Topics. 2007;151(9).

3860. Bailly A, Fau L, Lourioux C, Gramaje N, Mechain A, Arnaud A, et al. Therapeutic patient education: A solution to the treatment of obesity and metabolic syndrome in psychiatry. European Psychiatry. 2017;41:S471-S2. doi: https://doi.org/10.1016/j.eurpsy.2017.01.540.

3861. Bain A, Hasan SS, Kavanagh S, Babar ZU. Use and validation of a survey tool to measure the perceived effectiveness of insulin prescribing safety interventions in UK hospitals. Diabetic Medicine. 2020;37(12):2027-34. PubMed PMID: 32592220.

3862. Bain A, Hasan SS, Kavanagh S, Babar ZU. Strategies to reduce insulin prescribing errors in UK hospitals: results from a national survey. Diabetic Medicine. 2020;37(7):1176-84. PubMed PMID: 31845373.

3863. Bain A, Hasan SS, Kavanagh S, Babar ZUD. Strategies to reduce insulin prescribing errors in UK

hospitals: results from a national survey. Diabetic Medicine. 2020;37(7):1176-84. doi: 10.1111/dme.14209. PubMed PMID: 143852627. Language: English. Entry Date: 20200623. Revision Date: 20210701. Publication Type: Article.

3864. Bain A, Hasan SS, Kavanagh S, Babar ZUD. Use and validation of a survey tool to measure the perceived effectiveness of insulin prescribing safety interventions in UK hospitals. Diabetic Medicine. 2020;37(12):2027-34. doi: 10.1111/dme.14351. PubMed PMID: 146973856. Language: English. Entry Date: 20201118. Revision Date: 20211201. Publication Type: Article.

3865. Bain A, Kavanagh S, Babar Z-U-D. Prescribing Insulin for People With Diabetes in Secondary Care: Recommendations and Future Direction. In: Babar Z-U-D, editor. Encyclopedia of Pharmacy Practice and Clinical Pharmacy. Oxford: Elsevier; 2019. p. 107-16.

3866. Bain E, Pierides KL, Clifton VL, Hodyl NA, Stark MJ, Crowther CA, et al. Interventions for managing asthma in pregnancy. Cochrane Database of Systematic Reviews. 2014;(10). doi: 10.1002/14651858.CD010660.pub2. PubMed PMID: CD010660.

3867. Bain KT, Knowlton CH, Matos A. Cost avoidance related to a pharmacist-led pharmacogenomics service for the Program of All-inclusive Care for the Elderly. Pharmacogenomics. 2020;21(10):651-61. PubMed PMID: 32515286.

3868. Bain KT, Knowlton CH, Turgeon J. Medication Risk Mitigation: Coordinating and Collaborating with Health Care Systems, Universities, and Researchers to Facilitate the Design and Execution of Practice-Based Research. Clinics in Geriatric Medicine. 2017;33(2):257-81. doi: https://doi.org/10.1016/j.cger.2017.01.009.

3869. Bain KT, Schwartz EJ, Knowlton OV, Knowlton CH, Turgeon J. Implementation of a pharmacist-led pharmacogenomics service for the Program of All-Inclusive Care for the Elderly (PHARM-GENOME-PACE). Journal of the American Pharmacists Association. 2018;58(3):281-9.e1. doi: https://doi.org/10.1016/j.japh.2018.02.011.

3870. Baines D. Has pharmacy run out of time? Pharmaceutical Journal. 2008;281(7509):21-3.

3871. Baines D, Gahir IK, Hussain A, Khan AJ, Schneider P, Hasan SS, et al. A scoping review of the quality and the design of evaluations of mobile health, telehealth, smart pump and monitoring technologies performed in a pharmacy-related setting. Frontiers in Pharmacology. 2018;9(JUL). doi: 10.3389/fphar.2018.00678.

3872. Baines D, Hale C. How should community pharmacists be paid under the new contract? Pharmaceutical Journal. 2004;273(7309):119-20.

3873. Baines DL. Community pharmacy needs magical thinkers to help secure its future? Pharmaceutical Journal. 2005;275(7365):285-6.

3874. Bairami F, Soleymani F, Rashidian A. Improving Injectable Medicines Prescription in Outpatient Services: A Path Towards Rational Use of Medicines in Iran. International Journal of Health Policy and Management. 2016;5(5):321-4. doi: 10.15171/ijhpm.2016.24. PubMed PMID: WOS:000379823700005.

3875. Baird AB, Abode AM, Holland CR, Rhodes LA, Marciniak MW. Using pharmacy technicians to maximize pharmacist intervention with home health service patients. Journal of the American Pharmacists Association. 2020;60(3, Supplement):S103-S7. doi: https://doi.org/10.1016/j.japh.2020.01.006.

3876. Baird JRJ, Benjamin K, Qing M, Gionfriddo MR, McConaha J, Schoen R. Medication affordability discussions with older adults in primary care. Exploratory Research in Clinical and Social Pharmacy.

2023:100230. doi: https://doi.org/10.1016/j.rcsop.2023.100230.

3877. Bairu M. Combating a global pandemic of weak, adulterated, and fake drugs. Journal of Commercial Biotechnology. 2015;21(3):3-4. doi: 10.5912/jcb712.

3878. Baixauli Fernández VJ, Moreno Royo L, Salar Ibáñez L. Opinion of the community pharmacists of the province of Valencia about consultation and resolution of pharmacotherapeutics consultations of drug information. Pharmaceutical Care Espana. 2014;16(1):2-10.

3879. Baixauli VJ, Jimenez EG, Molinero A. Specifications from the vascular risk measuring and control service. Farmaceuticos Comunitarios. 2016;8(3):18-22. doi: 10.5672/FC.2173-9218.(2016/Vol8).003.04. PubMed PMID: WOS:000417274800004.

3880. Baiyeri PK, Ugese FD, Nwobodo CE. Diversity, food, nutrition and medicinal importance of some selected less-known African indigenous tropical plant species: Advocacy for global research attention. CAB Reviews: Perspectives in Agriculture, Veterinary Science, Nutrition and Natural Resources. 2019;14. doi: 10.1079/PAVSNNR201914012.

3881. Bajaj N, Phelan J, McConnell EE, Reed SM. A narrative medicine intervention in pediatric residents led to sustained improvements in resident well-being. Annals of Medicine. 2023;55(1):849-59. doi: 10.1080/07853890.2023.2185674. PubMed PMID: WOS:000943336100001.

3882. Bajaria S. The sooner we stop moaning the better [4]. Pharmaceutical Journal. 2006;277(7430):688.

3883. Bajcar JM, Kennie N, Einarson TR. Collaborative medication management in a team-based primary care practice: an explanatory conceptual framework. Research in Social and Administrative Pharmacy. 2005;1(3):408-29.

3884. Bajema KL, Berry K, Streja E, Rajeevan N, Li Y, Mutalik P, et al. Effectiveness of COVID-19 Treatment With Nirmatrelvir–Ritonavir or Molnupiravir Among U.S. Veterans: Target Trial Emulation Studies With One-Month and Six-Month Outcomes. Annals of Internal Medicine. 2023;176(6):807-16. doi: 10.7326/M22-3565. PubMed PMID: 164395017.

3885. Bajeux E, Alix L, Cornee L, Barbazan C, Mercerolle M, Howlett J, et al. Pharmacist-led medication reconciliation at patient discharge: a tool to reduce healthcare utilization? an observational study in patients 65 years or older. BMC Geriatrics. 2022;22(1):576. PubMed PMID: 35831783.

3886. Bajis D, Chaar B, Basheti I, Moles R. Teaching asthma first aid to pharmacy students: a comparative study between an online course and simulation by role-play. Pharmacy education. 2021;21(1):92-104. doi: 10.46542/pe.2021.211.92104. PubMed PMID: CN-02303522.

3887. Bajis D, Chaar B, Basheti IA, Moles R. Pharmacy students' medication history taking competency: Simulation and feedback learning intervention. Currents in Pharmacy Teaching and Learning. 2019;11(10):1002-15. doi: https://doi.org/10.1016/j.cptl.2019.06.007.

3888. Bajis D, Chaar B, Penm J, Moles R. Competency-based pharmacy education in the Eastern Mediterranean Region-A scoping review. Currents in Pharmacy Teaching and Learning. 2016;8(3):401-28. doi: 10.1016/j.cptl.2016.02.003. PubMed PMID: WOS:000409330700019.

3889. Bajorek B, Lemay K, Magin P, Roberts C, Krass I, Armour C. Patients' Attitudes and Approaches to the Self-Management of Hypertension: Perspectives from an Australian Qualitative Study in Community Pharmacy. High Blood Pressure and Cardiovascular Prevention. 2017;24(2):149-55. doi: 10.1007/s40292-017-0181-8.

3890. Bajorek B, Lemay KS, Magin P, Roberts C, Armour CL. Implementation and evaluation of a pharmacist-led hypertension management service in primary care: outcomes and methodological challenges. Pharmacy practice. 2016;14(2) (no pagination). doi: 10.18549/PharmPract.2016.02.723. PubMed PMID: CN-01471932.

3891. Bajorek B, Lemay KS, Magin P, Roberts C, Krass I. Implementation and evaluation of a pharmacist-led hypertension management service in primary care: Outcomes and methodological challenges. Pharmacy Practice. 2016;14(2). doi: 10.18549/PharmPract.2016.02.723.

3892. Bajorek BV, Bakshi R, MacPherson RD, Chow C, Elliott P. Pharmacist charting in the preadmission clinic of a Sydney teaching hospital: a pilot study. Journal of Pharmacy Practice & Research. 2017;47(5):375-82. doi: 10.1002/jppr.1268. PubMed PMID: 125725366. Language: English. Entry Date: 20171028. Revision Date: 20181001. Publication Type: Article.

3893. Bajorek BV, Lemay KS, Magin PJ, Roberts C, Krass I, Armour CL. Preparing pharmacists to deliver a targeted service in hypertension management: evaluation of an interprofessional training program. Bmc Medical Education. 2015;15. doi: 10.1186/s12909-015-0434-y. PubMed PMID: WOS:000361812900001.

3894. Bajorek BV, Lemay KS, Magin PJ, Roberts C, Krass I, Armour CL. Management of hypertension in an Australian community pharmacy setting - patients' beliefs and perspectives. International Journal of Pharmacy Practice. 2017;25(4):263-73. doi: 10.1111/ijpp.12301. PubMed PMID: WOS:000407071800003.

3895. Bajos N, Goulard H, Job-Spira N. Emergency contraception: from accessibility to counseling. Contraception. 2003;67(1):39-40. doi: https://doi.org/10.1016/S0010-7824(02)00435-3.

3896. Bajwah S, Oluyase AO, Yi D, Gao W, Evans CJ, Grande G, et al. The effectiveness and cost-effectiveness of hospital-based specialist palliative care for adults with advanced illness and their caregivers. Cochrane Database of Systematic Reviews. 2020;(9). doi: 10.1002/14651858.CD012780.pub2. PubMed PMID: CD012780.

3897. Bakar AA, Ong SC, Ting CY, Ooi GS, Hassali MAA. Barriers for Implementation of E-pharmacy Policy: Views of Pharmacy Authorities, Public Institutions and Societal Groups. Pertanika Journal of Social Sciences and Humanities. 2022;30(1):41-56. doi: 10.47836/pjssh.30.1.03.

3898. Baker A, Anderson M, Rooms L, Gonzalez C, Taylor K, McNall-Knapp R. DOUBLE BLIND, PLACEBO CONTROLLED TRIAL OF APREPITANT WITH CROSSOVER DESIGN: NO EVIDENCE OF BENEFIT. Pediatric blood & cancer. 2022;69(SUPPL 2):S61-. doi: 10.1002/pbc.29735. PubMed PMID: CN-02402937.

3899. Baker C, Whisler R, Brandon K, Fu D, Canfield S, Ejaz N, et al. The impact of individualized adherence interventions for solid-organ transplant patients with low medication possession ratio. Journal of the american pharmacists association. 2017;57(3). doi: 10.1016/j.japh.2017.04.011. PubMed PMID: CN-01608606.

3900. Baker CM, Crabtree G, Anderson K. Student pharmacist perceptions of learning after strengths-based leadership skills lab and escape room in pharmacy practice skills laboratory. Currents in Pharmacy Teaching and Learning. 2020;12(6):724-7. doi: https://doi.org/10.1016/j.cptl.2020.01.021.

3901. Baker CN. History and Politics of Medication Abortion in the United States and the Rise of Telemedicine and Self-Managed Abortion. Journal of Health Politics, Policy & Law. 2023;48(4):485-510. doi: 10.1215/03616878-10449941. PubMed PMID: 165130477.

3902. Baker DE. Omadacycline. Hospital Pharmacy. 2019;54(2):80-7. doi: 10.1177/0018578718823730.

3903. Baker DE, Hollinger MK, Mieure KD. Assessment of a pharmacy technician medication history program. American journal of health-system pharmacy. 2021;78(Supplement_2):S46-S51. doi: 10.1093/ajhp/zxaa312. PubMed PMID: CN-02285414.

3904. Baker DP, Day R, Salas E. Teamwork as an essential component of high-reliability organizations. Health services research. 2006;41(4p2):1576-98.

3905. Baker DP, Gustafson S, Beaubien J, Salas E, Barach P. Medical teamwork and patient safety: the evidence-based relation. AHRQ publication. 2005;5(53):1-64.

3906. Baker DW, Parker RM, Williams MV, Pitkin K, Parikh NS, Coates W, et al. The health care experience of patients with low literacy. Archives of family medicine. 1996;5(6):329.

3907. Baker EB, Wellman NS. Nutrition concerns in discharge planning for older adults: A need for multidisciplinary collaboration. Journal of the American Dietetic Association. 2005;105(4):603-7. doi: 10.1016/j.jada.2005.01.006. PubMed PMID: WOS:000228076600019.

3908. Baker ES, Connor TH. Monitoring occupational exposure to cancer chemotherapy drugs. American journal of health-system pharmacy. 1996;53(22):2713-23.

3909. Baker J, McCune JS, Harvey RD, Bonsignore C, Lindley CM. Granulocyte colony-stimulating factor use in cancer patients. Annals of Pharmacotherapy. 2000;34(7-8):851-7. PubMed PMID: WOS:000088105400005.

3910. Baker J, Patel M, Neogi T, Robinson K, Ogdie A, Scanzello C. A Double-Blind Randomized Trial to Evaluate the Efficacy of Corticosteroid Injections for Osteoarthritis of the Knee Using Mobile Devices. Arthritis & rheumatology. 2020;72(SUPPL 10):3332-4. doi: 10.1002/art.41538. PubMed PMID: CN-02242767.

3911. Baker JF, Patel M, Neogi T, Robinson K, Ogdie A, Scanzello C. A double-blind randomized trial to evaluate the efficacy of corticosteroid injections for osteoarthritis of the knee using mobile devices. Osteoarthritis and cartilage. 2021;29:S222-. doi: 10.1016/j.joca.2021.02.302. PubMed PMID: CN-02274578.

3912. Baker JG. Engaging community mental health stakeholders in pharmacy cost management. Psychiatric Services. 2001;52(5):650-3. doi: 10.1176/appi.ps.52.5.650. PubMed PMID: WOS:000168682900014.

3913. Baker JT, Borris RP, Carté B, Cordell GA, Soejarto DD, Cragg GM, et al. Natural product drug discovery and development: new perspectives on international collaboration. Journal of natural products. 1995;58(9):1325-57.

3914. Baker JW, Forkum W, McNeal J. Utilizing clinical video telehealth to improve access and optimize pharmacists' role in diabetes management. Journal of the American Pharmacists Association. 2019;59(2):S63-S6. doi: 10.1016/j.japh.2019.01.004. PubMed PMID: WOS:000460662000013.

3915. Baker JW, Pierce KL, Ryals CA. INR Goal Attainment and Oral Anticoagulation Knowledge of Patients Enrolled in an Anticoagulation Clinic in a Veterans Affairs Medical Center. Journal of Managed Care Pharmacy. 2011;17(2):133-42. PubMed PMID: WOS:000288307000002.

3916. Baker KR. Why Pharmacists Should Document Their Actions: Successfully defending a particular action is more likely if events, thoughts, or facts behind the decision have been recorded. American Pharmacy. 1991;31(12):38-41. doi: https://doi.org/10.1016/S0160-3450(15)31290-3.

3917. Baker KR. Risk management from a collaborative perspective. Journal of the American Pharmacists

Association: JAPhA. 2003;43(5 Suppl 1):S54-5. PubMed PMID: 14626535.

3918. Baker KR. Insurance for the compounding pharmacy business, part 1: who is covered? International Journal of Pharmaceutical Compounding. 2008;12(5):420-4. PubMed PMID: 105553617. Language: English. Entry Date: 20090410. Revision Date: 20150711. Publication Type: Journal Article.

3919. Baker KR. Insurance for the compounding pharmacy business, part 2: what, when, and where. International Journal of Pharmaceutical Compound. 2008;12(6):514-23. PubMed PMID: 23969929.

3920. Baker KR. Insurance for the Compounding Business, Part 1: Who is Covered? International Journal of Pharmaceutical Compound. 2008;12(5):420-4. PubMed PMID: 23969866.

3921. Baker KR, Baker KRBJ. Insurance for the compounding pharmacy business, part 2: what, when, and where. International Journal of Pharmaceutical Compounding. 2008;12(6):514-23. PubMed PMID: 105585926. Language: English. Entry Date: 20090501. Revision Date: 20151028. Publication Type: journal article. Journal Subset: Biomedical.

3922. Baker KR, Mondt D. Risk Management in Pharmacy: Preventing Liability Claims: Risk managers can identify and analyze the potential for liability claims and implement changes to reduce the likelihood of such problems. American Pharmacy. 1994;34(10):60-72. doi: https://doi.org/10.1016/S0160-3450(15)30285-3.

3923. Baker KR, Renaud C. Insurance for the compounding pharmacy business, part 2: What, when, and where. International Journal of Pharmaceutical Compounding. 2008;12(6):514-23.

3924. Baker L. The Role of Pharmacists in Travel Medicine in South Africa. Pharmacy. 2018;6(3). doi: 10.3390/pharmacy6030068. PubMed PMID: WOS:000448399800014.

3925. Baker M, Bell CM, Xiong W, Etchells E, Rossos PG, Shojania KG, et al. Do Combined Pharmacist and Prescriber Efforts on Medication Reconciliation Reduce Postdischarge Patient Emergency Department Visits and Hospital Readmissions? Journal of Hospital Medicine (Online). 2018;13(3):152-7. PubMed PMID: 29069119.

3926. Baker M, Miranda D, Dixon G, Salsman M, Oza MD. Impact of Postsurgical Opioid Prescription Reduction in General Surgery Patients. Hospital Pharmacy. 2023;58(4):389-91. doi: 10.1177/00185787221150924. PubMed PMID: 164441893.

3927. Baker S. Miconazole oral gel and warfarin [12]. Pharmaceutical Journal. 2008;280(7493):308.

3928. Baker S, Lee YP, Hattingh HL. An evaluation of the role of practice pharmacists in Australia: a mixed methods study. International Journal of Clinical Pharmacy. 2019. doi: 10.1007/s11096-019-00807-5.

3929. Baker S, Vandermorris S, Verhoeff N, Troyer AK. A Cost-Benefit Analysis of a Group Memory Intervention for Healthy Older Adults with Memory Concerns. Canadian Journal on Aging-Revue Canadienne Du Vieillissement. 2022;41(4):531-9. doi: 10.1017/s0714980821000726. PubMed PMID: WOS:000813966100001.

3930. Baker SN, Weant KA. Procedural sedation and analgesia in the emergency department. Journal of Pharmacy Practice. 2011;24(2):189-95. PubMed PMID: 21712213.

3931. Baker T. A case study on rheumatoid arthritis. American Journal of Managed Care. 2003;9(4):S87-S98. PubMed PMID: WOS:000185603500001.

3932. Baker WL, Cios DA, Sander SD, Coleman CI. Meta-analysis to assess the quality of warfarin control

in atrial fibrillation patients in the United States. Journal of Managed Care Pharmacy. 2009;15(3):244-52.

3933. Baker WL, Cios DA, Sander SD, Coleman CI. Meta-analysis to assess the quality of warfarin control in artial fibrillation patients in the United States. Journal of Managed Care Pharmacy. 2009;15(3):244-52. doi: 10.18553/jmcp.2009.15.3.244.

3934. Baker WL, Kluger J, Michael White C, Riche KD, Coleman CI. Does magnesium L-lactate improve quality of life in patients with an implantable cardioverter defibrillator? Pharmacotherapy. 2011;31(10):320e. PubMed PMID: CN-01035333.

3935. Baker-Rush ML, Pabst A, Aitchison R, Anzur T, Paschal N. Fear in Interprofessional Simulation: The role of psychology and behaviorism in student participation and learning. Journal of Interprofessional Education & Practice. 2021;24:100432. doi: https://doi.org/10.1016/j.xjep.2021.100432.

3936. Bakey KH, Nguyen C-TN. Impact of a Pharmacist Intervention in the Emergency Department on the Appropriateness of Direct Oral Anticoagulants Prescribed in Venous Thromboembolism Patients. Journal of Pharmacy Practice. 2022;35(4):599-605. doi: 10.1177/08971900211000704. PubMed PMID: 157872736. Language: English. Entry Date: 20220714. Revision Date: 20220715. Publication Type: Article.

3937. Bakey KH, Nguyen CTN. Impact of a Pharmacist Intervention in the Emergency Department on the Appropriateness of Direct Oral Anticoagulants Prescribed in Venous Thromboembolism Patients. Journal of Pharmacy Practice. 2022;35(4):599-605. doi: 10.1177/08971900211000704. PubMed PMID: WOS:000630790300001.

3938. Bakhireva LN, Bautista A, Cano S, Shrestha S, Bachyrycz AM, Cruz TH. Barriers and facilitators to dispensing of intranasal naloxone by pharmacists. Substance Abuse. 2018;39(3):331-41. doi: 10.1080/08897077.2017.1391924.

3939. Bakhriansyah M, Souverein PC, Klungel OH, De Boer A, Blom MT, Tan HL. Non-steroidal anti-inflammatory drugs and the risk of out-of-hospital cardiac arrest: A case-control study. Europace. 2019;21(1):99-105. doi: 10.1093/europace/euy180.

3940. Bakhsh H, Makki H. Splitting teams at a hospital pharmaceutical care services during COVID-19 pandemic: A tertiary hospital experience in Saudi Arabia. Journal of Microscopy and Ultrastructure. 2020;8(4):152-5. doi: 10.4103/JMAU.JMAU_57_20.

3941. Bakhsh HT, Perona SJ. Medical and Nursing Staff Education Reduces Use of Prophylactic Ondansetron With Opioids in the Emergency Department. Journal of Emergency Nursing. 2019;45(3):273-7. doi: 10.1016/j.jen.2018.07.011.

3942. Bakhshi F, Mitchell R, Nasrabadi AN, Varaei S, Hajimaghsoudi M. Behavioural changes in medication safety: Consequent to an action research intervention. Journal of Nursing Management (John Wiley & Sons, Inc). 2021;29(2):152-64. doi: 10.1111/jonm.13128. PubMed PMID: 149107591. Language: English. Entry Date: 20210311. Revision Date: 20210312. Publication Type: Article.

3943. Bakhshi F, Nasrabadi AN, Mitchell R, Bagherabadi M, Saghafi F, Varaei S. Explanatory factors relating to medication error reporting among emergency medicine clinicians: A qualitative study. Journal of Patient Safety & Risk Management. 2023;28(5):215-21. doi: 10.1177/25160435231188545. PubMed PMID: 173276345.

3944. Bakibinga P, Kabaria C, Kasiira Z, Kibe P, Kyobutungi C, Mbaya N, et al. Primary care doctor and nurse consultations among people who live in slums: a retrospective, cross-sectional survey in four countries. Bmj Open. 2022;12(1). doi: 10.1136/bmjopen-2021-054142. PubMed PMID: WOS:000741038800007.

3945. Bakken BK, Bozymski KM, Foster B, Blaeser SD, Mott DA, Doucette WR, et al. Ambulatory care pharmacy practice: Findings from the 2019 National Pharmacist Workforce Survey. American Journal of Health-System Pharmacy. 2023;80(5):284-95. doi: https://dx.doi.org/10.1093/ajhp/zxac352. PubMed PMID: 2023994670.

3946. Bakken BK, Gaither CA, Doucette WR, Witry MJ, Kreling DH, Schommer JC, et al. An intersectional review of discrimination and harassment experiences in pharmacy: findings from the 2019 National Pharmacist Workforce Survey. Journal of the American Pharmacists Association : JAPhA. 2021;61(5):522-32. doi: 10.1016/j.japh.2021.04.002. PubMed PMID: CN-02276123.

3947. Bakken BK, Winn AN. Clinician burnout during the COVID-19 pandemic before vaccine administration. Journal of the American Pharmacists Association. 2021;61(5):e71-e7. doi: https://doi.org/10.1016/j.japh.2021.04.009.

3948. Bakken K, Larsen E, Lindberg PC, Rygh E, Hjortdahl P. Insufficient communication and information regarding patient medication in the primary healthcare sector. Tidsskrift for den Norske Laegeforening. 2007;127(13-14):1766-9.

3949. Bakker MK, Jentink J, Vroom F, Van Den Berg PB, De Walle HEK, De Jong-Van Den Berg LTW. Drug prescription patterns before, during and after pregnancy for chronic, occasional and pregnancy-related drugs in the Netherlands. BJOG: An International Journal of Obstetrics and Gynaecology. 2006;113(5):559-68. doi: 10.1111/j.1471-0528.2006.00927.x.

3950. Bakker T, Abu-Hanna A, Dongelmans DA, Vermeijden WJ, Bosman RJ, de Lange DW, et al. Clinically relevant potential drug-drug interactions in intensive care patients: A large retrospective observational multicenter study. Journal of Critical Care. 2021;62:124-30. doi: https://doi.org/10.1016/j.jcrc.2020.11.020.

3951. Bakker T, Klopotowska JE, de Keizer NF, van Marum R, van der Sijs H, de Lange DW, et al. Improving medication safety in the Intensive Care by identifying relevant drug-drug interactions - Results of a multicenter Delphi study. Journal of Critical Care. 2020;57:134-40. PubMed PMID: 32145656.

3952. Bakker T, Klopotowska JE, de Keizer NF, van Marum R, van der Sijs H, de Lange DW, et al. Improving medication safety in the Intensive Care by identifying relevant drug-drug interactions - Results of a multicenter Delphi study. Journal of Critical Care. 2020;57:134-40. doi: https://doi.org/10.1016/j.jcrc.2020.02.012.

3953. Bakkum MJ, Donker EM, Spitaleri Timpone P, Hagen CAM, Richir MC, van Agtmael MA, et al. Educational value of international and intercultural differences in prescribing: the international and interprofessional student-run clinic project. European Journal of Clinical Pharmacology. 2023;79(4):571-4. doi: 10.1007/s00228-023-03465-9. PubMed PMID: 162682732.

3954. Bakos-Block CR, Al Rawwad T, Cardenas-Turanzas M, Champagne-Langabeer T. Contact based intervention reduces stigma among pharmacy students. Currents in Pharmacy Teaching and Learning. 2022;14(12):1471-7. doi: 10.1016/j.cptl.2022.10.002.

3955. Bakrin FS, Azizi BHO, Yusuf N, Tajuddin Y, Sinniah J, Balakrishnan S, et al. Perceptions of patient safety competency among graduating pharmacy, nursing, physiotherapy and medical imaging students: A cross-sectional study in Malaysia. Pharmacy Education. 2022;22(1):312-22. doi: 10.46542/pe.2022.221.312322.

3956. Bakrin FS, Azizi BO, Yusuf N, Tajuddin Y, Sinniah J, Balakrishnan S, et al. Perceptions of patient safety competency among graduating pharmacy, nursing, physiotherapy and medical imaging students: A cross-sectional study in Malaysia. Pharmacy Education. 2022;22(1):312-22. doi: 10.46542/

pe.2022.221.312322. PubMed PMID: WOS:000804419600001.

3957. Bakris G, Molitch M, Hewkin A, Kipnes M, Sarafidis P, Fakouhi K, et al. Differences in glucose tolerance between fixed-dose antihypertensive drug combinations in people with metabolic syndrome. Diabetes care. 2006;29(12):2592-7.

3958. Baky V, Moran D, Warwick T, George A, Williams T, McWilliams E, et al. Obtaining a follow-up appointment before discharge protects against readmission for patients with acute coronary syndrome and heart failure: A quality improvement project. International Journal of Cardiology. 2018;257:12-5. doi: https://doi.org/10.1016/j.ijcard.2017.10.036.

3959. Bala I, Hariharan S, Kumar MR. PLGA nanoparticles in drug delivery: the state of the art. Critical Reviews™ in Therapeutic Drug Carrier Systems. 2004;21(5).

3960. Balaji K, Anusha A, Chandana AS, Sri BN, K. N CK. AN OBSERVATIONAL STUDY ON CLINICAL SAFETY AND EFFICACY OF ANTIVIRAL MEDICATIONS USED IN CHRONIC HEPATITIS-B VIRUS INFECTION. Journal of Advanced Scientific Research. 2023;14(7):47-54. doi: 10.55218/JASR.202314708. PubMed PMID: 171890062.

3961. Balak DM, Fallah-Arani S, Venema CM, Neumann HA, Thio HB. Addition of an oral histamine antagonist to reduce adverse events associated with fumaric acid esters in the treatment of psoriasis: a randomized double-blind placebo-controlled trial. British journal of dermatology. 2015;172(3):754-9. doi: 10.1111/bjd.13277. PubMed PMID: CN-01075173.

3962. Balak DMW, Fallah-Arani S, Venema CM, Neumann HAM, Thio HB. Addition of an oral histamine antagonist to reduce adverse events associated with fumaric acid esters in psoriasis treatment: a randomized double-blind placebo-controlled trial. Nederlands tijdschrift voor dermatologie en venereologie. 2014;24(1):47-8. PubMed PMID: CN-01056927.

3963. Balakrishnan A, Hossain J, Jones V, Heartin E, Moratuwagama D. Apixaban in the prevention of thromboembolism in patients with Multiple Myeloma receiving immunomodulatory medications. Real world experience - Data from two centers in North Wales, United Kingdom. British journal of haematology. 2021;193(SUPPL 1):174-5. doi: 10.1111/bjh.17492. PubMed PMID: CN-02274925.

3964. Balakrishnan A, Nair S, Kunhikatta V, Rashid M, Unnikrishnan MK, Jagannatha PS, et al. Effectiveness of blended learning in pharmacy education: an experimental study using clinical research modules. PloS one. 2021;16(9):e0256814. doi: 10.1371/journal.pone.0256814. PubMed PMID: CN-02322443.

3965. Bălan A, Bejan I, Bonciu S, Eni CE, Ruță S. Romanian medical students' attitude towards and perceived knowledge on covid-19 vaccination. Vaccines. 2021;9(8). doi: 10.3390/vaccines9080854.

3966. Balan S, Hassali MA, Mak VSL. Awareness, knowledge and views of off-label prescribing in children: a systematic review. British Journal of Clinical Pharmacology. 2015;80(6):1269-80. doi: 10.1111/bcp.12750. PubMed PMID: WOS:000367470200004.

3967. Balan S, Hussain R, Ong SC, Babar Z-U-D. Drug Safety in Children: Research Studies and Evidence Synthesis. Encyclopedia of Evidence in Pharmaceutical Public Health and Health Services Research in Pharmacy: Springer; 2023. p. 1-14.

3968. Balanag V, Yunus F, Yang P-C, Jorup C. Efficacy and safety of budesonide/formoterol compared with salbutamol in the treatment of acute asthma. Pulmonary pharmacology & therapeutics. 2006;19(2):139-47.

3969. Balarajan Y, Selvaraj S, Subramanian S. Health care and equity in India. The Lancet. 2011;377(9764):505-15.

3970. Balas MC, Scott LD, Rogers AE. The prevalence and nature of errors and near errors reported by hospital staff nurses. Applied Nursing Research. 2004;17(4):224-30.

3971. Balasanova AA, Kim J, Wang H, Hiebert R, Spurgin MJ, Potter J, et al. Interprofessional learner perceptions of a brief educational intervention about opioid prescribing for older adults. Journal of the American Geriatrics Society. 2023;71(6):E19-E22. doi: 10.1111/jgs.18358. PubMed PMID: 164231774.

3972. Balasopoulos T, Charonis A, Athanasakis K, Kyriopoulos J, Pavi E. Why do generic drugs fail to achieve an adequate market share in Greece? Empirical findings and policy suggestions. Health Policy. 2017;121(3):265-72. doi: https://doi.org/10.1016/j.healthpol.2016.12.011.

3973. Balasubramaniam R, Benaragama B, Ranganathan SS. A National Survey of Price and Affordability of Key Essential Medicines for Children in Sri Lanka. Indian Journal of Pediatrics. 2014;81(4):362-7. doi: 10.1007/s12098-013-1024-9. PubMed PMID: WOS:000334173700008.

3974. Balasubramaniam R, Benaragama BVSH, Sri Ranganathan S. A national survey of price and affordability of key essential medicines for children in Sri Lanka. Indian Journal of Pediatrics. 2014;81(4):362-7. doi: 10.1007/s12098-013-1024-9.

3975. Balasubramaniam R, Beneragama BV, Sri Ranganathan S. A national survey of availability of key essential medicines for children in Sri Lanka. The Ceylon medical journal. 2011;56(3):101-7. doi: 10.4038/cmj.v56i3.3597.

3976. Balayssac D, Pereira B, Cuq P, Douris J, Ferrari L, Boutet-Robinet E, et al. Perception of pharmacy students toward opioid-related disorders and roles of community pharmacists: A French nationwide cross-sectional study. Substance Abuse. 2021;42(4):706-15. doi: 10.1080/08897077.2020.1850607.

3977. Balayssac D, Pereira B, Desmard J, Viers H, Barillon C, Osseili A, et al. Satisfaction of Cancer Patients Treated with Oral Anticancer Medications regarding Dispensing by Community Pharmacists: A Cross-Sectional Study. European Journal of Cancer Care. 2023;2023. doi: 10.1155/2023/7746349.

3978. Balayssac D, Pereira B, Virot J, Collin A, Alapini D, Cuny D, et al. Burnout, associated comorbidities and coping strategies in French community pharmacies-BOP study: A nationwide cross-sectional study. Plos One. 2017;12(8). doi: 10.1371/journal.pone.0182956. PubMed PMID: WOS:000407431800037.

3979. Balayssac D, Pereira B, Virot J, Lambert C, Collin A, Alapini D, et al. Work-related stress, associated comorbidities and stress causes in French community pharmacies: a nationwide cross-sectional study. PeerJ. 2017;5:e3973.

3980. Balayssac D, Pereira B, Virot J, Lambert C, Collin A, Alapini D, et al. Work-related stress, associated comorbidities and stress causes in French community pharmacies: A nationwide cross-sectional study. PeerJ. 2017;2017(10). doi: 10.7717/peerj.3973.

3981. Baláž P, Dutková E. Fine milling in applied mechanochemistry. Minerals Engineering. 2009;22(7):681-94. doi: https://doi.org/10.1016/j.mineng.2009.01.014.

3982. Balazova M, Grznarova M, Kuzelova M. The role of point-of-care testing in identification of drug-related problems in a community pharmacy. International Journal of Clinical Pharmacy. 2013;35(6):1276-. PubMed PMID: WOS:000328208800091.

3983. Balážová M, Kuželová M. Determination of plasma lipids in a community pharmacy in relation to drug-related problems identification. Farmaceuticky Obzor. 2012;81(7-8):132-6.

3984. Balazova M, Matejka P, Kuzelova M. Identification of drug-related problems in patients with elevated plasma lipids levels in a community pharmacy. International Journal of Clinical Pharmacy. 2013;35(5):892-. PubMed PMID: WOS:000325622700118.

3985. Balch AH, Wilkes J, Enioutina EY. Use of intravenous immunoglobulin to treat encephalitis in a Pediatric Hospital Network. Clinical pharmacology in drug development. 2019;8(SUPPL 1):39-40. doi: 10.1002/cpdd.724. PubMed PMID: CN-02257668.

3986. Balch C, Ogle JD, Senese JL. The national practice benchmark for oncology: 2015 report for 2014 data. Journal of Oncology Practice. 2016;12(4):e437-e75. doi: 10.1200/JOP.2015.008458.

3987. Balcha BA, Endeshaw M, Mebratie AD. Household satisfaction with a pilot community-based health insurance scheme and associated factors in Addis Ababa. Journal of Public Health Research. 2023;12(2). doi: 10.1177/22799036231163382. PubMed PMID: WOS:000970787800001.

3988. Balchaites A, McCarthy S, Fleming A. Exploring the human factors of medication errors in community pharmacy: a mixed methods study...Health Services Research and Pharmacy Practice Conference, 11-12 April, 2022, University of Bath, UK. International Journal of Pharmacy Practice. 2022;30:i39-i40. doi: 10.1093/ijpp/riac019.055. PubMed PMID: 157486002. Language: English. Entry Date: 20220624. Revision Date: 20220624. Publication Type: Article.

3989. Bald EM, Triplett C, Beranek R, Kennelty K. Evaluation of a Pharmacist-Led Diabetes Collaborative Drug Therapy Management Service. Journal of Pharmacy Practice. 2022;35(6):846-52. PubMed PMID: 33840299.

3990. Baldassarre D, Iacoviello L, Baetta R, Roncaglioni MC, Condorelli G, Remuzzi G, et al. Rationale and design of the CV-PREVITAL study: an Italian multiple cohort randomised controlled trial investigating innovative digital strategies in primary cardiovascular prevention. BMJ Open. 2023;13(7). doi: 10.1136/bmjopen-2023-072040.

3991. Balderson BH, Gray SL, Fujii MM, Nakata KG, Williamson BD, Cook AJ, et al. A health-system-embedded deprescribing intervention targeting patients and providers to prevent falls in older adults (STOP-FALLS trial): study protocol for a pragmatic cluster-randomized controlled trial. Trials. 2023;24(1):1-15. doi: 10.1186/s13063-023-07336-7. PubMed PMID: 163724805.

3992. Baldo P, Giulia F, Francescon S, De Re V, Ferrarin E, Vaccher E. Promotion and optimization of care in HIV infected patients with comorbidities, including cancer. International journal of clinical pharmacy. 2017;39(1):268-. doi: 10.1007/s11096-016-0404-4. PubMed PMID: CN-01333863.

3993. Baldon JP, Correr CJ, Melchiors AC, Rossignoli P, Fernández-Llimós F, Pontarolo R. Community pharmacists' attitudes and knowledge on dispensing drugs to pregnant women. Pharmacy Practice. 2006;4(1):38-43.

3994. Baldonedo-Mosteiro C, Franco-Correia S, Mosteiro-Diaz M-P. Psychological impact of COVID19 on community pharmacists and pharmacy technicians. Exploratory Research in Clinical and Social Pharmacy. 2022;5:100118. doi: https://doi.org/10.1016/j.rcsop.2022.100118.

3995. Baldoni AD, Ayres LR, Martinez EZ, Dewulf NDS, dos Santos V, Pereira LRL. Factors associated with potentially inappropriate medications use by the elderly according to Beers criteria 2003 and 2012. International Journal of Clinical Pharmacy. 2014;36(2):316-24. doi: 10.1007/s11096-013-9880-y. PubMed

PMID: WOS:000334495400020.

3996. Baldoni AdO, Ayres LR, Martinez EZ, Dewulf NdLS, Dos Santos V, Pereira LRL. Factors associated with potentially inappropriate medications use by the elderly according to Beers criteria 2003 and 2012. International Journal of Clinical Pharmacy. 2014;36(2):316-24. doi: 10.1007/s11096-013-9880-y. PubMed PMID: 103818012. Language: English. Entry Date: 20150206. Revision Date: 20211029. Publication Type: journal article.

3997. Baldoni AO, Dewulf NLS, dos Santos V, dos Reis TM, Ayres LR, Pereira LRL. Access barriers to pharmaceutical services among the elderly. Revista de Ciencias Farmaceuticas Basica e Aplicada. 2014;35(4):615-21.

3998. Baldoni S, Amenta F, Ricci G. Telepharmacy services: present status and future perspectives: a review. Medicina. 2019;55(7):327.

3999. Baldridge S, Alfieri T, Shah R. Pharmacist awareness of the dea national prescription drug take back day. Postgraduate Medicine. 2018;130(Supplement 1):81. PubMed PMID: 623799933.

4000. Balducci L, Beghe C. The application of the principles of geriatrics to the management of the older person with cancer. Critical Reviews in Oncology Hematology. 2000;35(3):147-54. doi: 10.1016/s1040-8428(00)00089-5. PubMed PMID: WOS:000088839000002.

4001. Baldwin Z, Jiao BS, Basu A, Roth J, Bender MA, Elsisi Z, et al. Medical and Non-medical Costs of Sickle Cell Disease and Treatments from a US Perspective: A Systematic Review and Landscape Analysis. Pharmacoeconomics-Open. 2022;6(4):469-81. doi: 10.1007/s41669-022-00330-w. PubMed PMID: WOS:000787680900001.

4002. Bale AO. Evaluating the Impact of In-Hospital Clinical Pharmacists' Activities on 30-Day Readmissions Among High Risk Medicare Shared Savings Program (MSSP) Patients [M.S.]. United States -- Texas: University of Houston; 2019.

4003. Balen E, Giordani F, Cano MFF, Zonzini FHT, Klein KA, Vieira MH, et al. Potential drug-drug interactions between psychotropic drugs. Jornal Brasileiro de Psiquiatria. 2017;66(3):172-7. doi: 10.1590/0047-2085000000167.

4004. Balestra R. Coordinated hospital-community organisation model for the prevention, monitoring and treatment of patients with addiction and HCV. Acta Biomedica. 2018;89:42-53. doi: 10.23750/abm.v89i10-S.7969.

4005. Balfour DC, Evans S, Januska J, Lee HY, Lewis SJ, Nolan SR, et al. Health information technology—results from a roundtable discussion. Journal of managed care pharmacy. 2009;15(1 Supp A):10-7.

4006. Balfour Iii DC, Evans S, Januska J, Lee HY, Lewis SJ, Nolan SR, et al. Health information technology - Results from a roundtable discussion. Journal of Managed Care Pharmacy. 2009;15(1 SUPPL.):S10-S7.

4007. Balfour JA, Benfield P. Cefpodoxime proxetil an appraisal of its use in antibacterial cost-containment programmes, as stepdown and abbreviated therapy in respiratory tract infections. PharmacoEconomics. 1996;10(2):164-78. doi: 10.2165/00019053-199610020-00008.

4008. Balfour JA, Benfield P. Cefpodoxime proxetil - An appraisal of its use in antibacterial cost-containment programmes, as stepdown and abbreviated therapy in respiratory tract infections. Pharmacoeconomics. 1996;10(2):164-78. doi: 10.2165/00019053-199610020-00008. PubMed PMID: WOS:A1996VA80200008.

4009. Balfour L, Corace K, Tasca GA, Best-Plummer W, MacPherson PA, Cameron DW. High HIV knowledge relates to low stigma in pharmacists and university health science students in Guyana, South America. International Journal of Infectious Diseases. 2010;14(10):e881-e7. doi: https://doi.org/10.1016/j.ijid.2010.03.021.

4010. Balina K, Romagnoli F, Blumberga D. Chemical Composition and Potential Use of Fucus Vesiculosus from Gulf of Riga. Energy Procedia. 2016;95:43-9. doi: https://doi.org/10.1016/j.egypro.2016.09.010.

4011. Balis AF. Miracle medicine: The impact of sulfa drugs on medicine, the pharmaceutical industry and government regulation in the United States in the 1930s [Ph.D.]. United States -- New York: City University of New York; 2000.

4012. Balisa-Rocha BJ, Guimaraes VG, Mesquita AR, Aguiar PM, Krass I, de Lyra DP. Enhancing health care for type 2 diabetes in Northern Brazil: A pilot study of pharmaceutical care in community pharmacy. African Journal of Pharmacy and Pharmacology. 2012;6(35):2584-91. doi: 10.5897/ajpp12.514. PubMed PMID: WOS:000310810500003.

4013. Balka E, Peddie D, Small SS, Ackerley C, Trimble J, Hohl CM, editors. Barriers to scaling up participatory design interventions in health IT: A case study. ACM International Conference Proceeding Series; 2018.

4014. Balkanska-Mitkova A, Yaneva A, Kondeva-Burdina M, Dimitrova M. Pharmacists‘ attitudes to dispensing food supplements to chronically ill patients: a pilot survey among community pharmacies in Bulgaria. Pharmacia. 2022;69(3):589-93. doi: 10.3897/pharmacia.69.e85471.

4015. Balkanski S, Simeonova J, Gitev I, Getov I. Evaluation of the current status of the value-added pharmacy services and pharmacists' attitude in Bulgaria. Pharmacia. 2019;66(2):79-83. doi: 10.3897/pharmacia.66.e36530. PubMed PMID: WOS:000476945000008.

4016. Balkrishnan R, Arondekar BV, Camacho FT, Shenolikar RA, Horblyuk R, Anderson RT. Comparisons of rosiglitazone versus pioglitazone monotherapy introduction and associated health care utilization in Medicaid-enrolled patients with type 2 diabetes mellitus. Clinical Therapeutics. 2007;29:1306-15. doi: 10.1016/j.clinthera.2007.06.019. PubMed PMID: WOS:000248371200007.

4017. Ball D, Ewen M, Laing R, Beran D. Insulin price components: case studies in six low/middle-income countries. Bmj Global Health. 2019;4(5). doi: 10.1136/bmjgh-2019-001705. PubMed PMID: WOS:000500476500028.

4018. Ball D, Salenga R. Pharmaceutical policy in the Philippines. Pharmaceutical Policy in Countries with Developing Healthcare Systems2017. p. 45-73.

4019. Ball DE, Al-Othman F. Drug information resources at private community pharmacies in Kuwait. Medical Principles and Practice. 2007;16(2):107-9. doi: 10.1159/000098361.

4020. Ball DE, Mazarurwi P. HIV/AIDS knowledge and attitudes amongst pharmacists in Zimbabwe. The Central African journal of medicine. 2003;49(3-4):27-31.

4021. Ball MJ, Lillis J. E-health: transforming the physician/patient relationship. International journal of medical informatics. 2001;61(1):1-10.

4022. Ball MJ, Weaver C, Abbott PA. Enabling technologies promise to revitalize the role of nursing in an era of patient safety. International Journal of Medical Informatics. 2003;69(1):29-38.

4023. Ball P, Quaass M. Removing the noose from rural communities. Australian Journal of Pharmacy. 2007;88(1047):24.

4024. Ball SJ, McCauley JA, Pruitt M, Zhang J, Marsden J, Barth KS, et al. Academic detailing increases prescription drug monitoring program use among primary care practices. Journal of the American Pharmacists Association. 2021;61(4):418-24.e2. doi: https://doi.org/10.1016/j.japh.2021.02.019.

4025. Ball SJ, McCauley JA, Pruitt M, Zhang JW, Marsden J, Barth KS, et al. The use of an integrated opioid and medical marijuana prescription drug monitoring program. Journal of the American Pharmacists Association. 2021;61(4):418-+. doi: 10.1016/j.japh.2021.02.019. PubMed PMID: WOS:000670385400036.

4026. Balladares EH, Heredia AB, Martínez-Martínez F. Action guide for the community pharmacist in inflammatory bowel disease. Ars Pharmaceutica. 2017;58(3):115-26. doi: 10.4321/S2340-98942017000300005.

4027. Ballantyne PJ. Medicines use and access research: Diverse methodologies show a complex picture requiring our attention. Southern Med Review. 2011;4(1):2-3. doi: 10.5655/smr.v4i1.79.

4028. Ballantyne PJ. Assessing pharmacists' impacts in primary health care: Are we asking the right questions? Southern Med Review. 2011;4(1):17-21. doi: 10.5655/smr.v4i1.76.

4029. Ballard KA. Patient safety: A shared responsibility. Online Journal of issues in nursing. 2003;8(3):4.

4030. Ballentine AJ, Kinnaird D, Wilson JP. Prescription errors occur despite computerized prescriber order entry. American Journal of Health-System Pharmacy. 2003;60(7):708-9. PubMed PMID: 12701557.

4031. Ballentine AJ, Lipowski EE. Judging a Book by Its Cover: The Predicament with Canadian Pharmacy Advertisements and American Consumers. Journal of the American Pharmacists Association. 2005;45(6):740-3. doi: https://doi.org/10.1331/154434505774909599.

4032. Ballerie A, Alix L, Bajeux E, Jégo P, Lescoat A, Hue B. The collaboration of clinical pharmacists and physicians for medication safety. The Lancet. 2019;394(10216):2240. doi: https://doi.org/10.1016/S0140-6736(19)32608-X.

4033. Ballesta Méndez M, Van Pesch V, Capron A, Hantson P. P31: Seizures and sustained encephalopathy following an accidental 4-aminopyridine overdose. Toxicologie Analytique et Clinique. 2014;26(2, Supplement):S43-S4. doi: https://doi.org/10.1016/S2352-0078(14)70092-7.

4034. Ballester P, Espadas C, Almenara S, Barrachina J, Muriel J, Ramos E, et al. CYP2D6 Genotype and Pharmacovigilance Impact on Autism Spectrum Disorder: A Naturalistic Study with Extreme Phenotype Analysis. Pharmaceuticals (14248247). 2023;16(7):954. doi: 10.3390/ph16070954. PubMed PMID: 169325070.

4035. Ballesteros A, Saiz H, Rosero ÁS, Portilla A, Montes L, Elorza R. SSRIs and QT interval prolongation management. A review. European Psychiatry. 2017;41:S750. doi: https://doi.org/10.1016/j.eurpsy.2017.01.1393.

4036. Balli ML, Dickey TA, Purvis RS, Warmack TS, Riklon S, McElfish PA. “You Want to Give the Best Care Possible, and You Know When They Leave Your Pharmacy, You Didn’t Give the Best Care Possible Most of the Time”: Pharmacist- and Community Health Worker-Identified Barriers and Facilitators to Medication Adherence in Marshallese Patients. Journal of Racial and Ethnic Health Disparities. 2019;6(4):652-9. doi: 10.1007/s40615-019-00563-2.

4037. Balling L, Erstad BL, Weibel K. Impact of a transition-of-care pharmacist during hospital discharge. Journal of the American Pharmacists Association. 2015;55(4):443-8. doi: https://doi.org/10.1331/JAPhA.2015.14087.

4038. Ballinger A, Peikin SR. Orlistat: its current status as an anti-obesity drug. European journal of pharmacology. 2002;440(2-3):109-17.

4039. Ballou JM, Chapman AR, Roark AM, Huie CH, McKee J, Marciniak MW. Conducting depression screenings in a community pharmacy: A pilot comparison of methods. JACCP Journal of the American College of Clinical Pharmacy. 2019;2(4):366-72. doi: 10.1002/jac5.1156.

4040. Ballough W, Crowe R, Elbert J. Buprenorphine versus methadone in opioid dependant patients. Pharmacotherapy. 2013;33(10):e294-. doi: 10.1002/phar.1356. PubMed PMID: CN-01049670.

4041. Ballreich J, Butz A. PRS17 ECONOMIC COSTS OF PEDIATRIC ASTHMA AMONG URBAN YOUTH. Value in health. 2019;22:S352-. doi: 10.1016/j.jval.2019.04.1721. PubMed PMID: CN-01963962.

4042. Balmer JMT, Chen WF. Corporate heritage tourism brand attractiveness and national identity. Journal of Product and Brand Management. 2016;25(3):223-38. doi: 10.1108/jpbm-08-2015-0959. PubMed PMID: WOS:000379340200002.

4043. Balmes JR. Occupational contribution to the burden of chronic obstructive pulmonary disease. Journal of Occupational and Environmental Medicine. 2005;47(2):154-60. doi: 10.1097/01.jom.0000152923.07801.e1. PubMed PMID: WOS:000227078200007.

4044. Baloch G, Gzara F. Capacity and assortment planning under one-way supplier-driven substitution for pharmacy kiosks with low drug demand. European Journal of Operational Research. 2020;282(1):108-28. doi: https://doi.org/10.1016/j.ejor.2019.09.007.

4045. Balon J, Thomas SA. Comparison of Hospital Admission Medication Lists With Primary Care Physician and Outpatient Pharmacy Lists. Journal of Nursing Scholarship. 2011;43(3):292-300. PubMed PMID: 51543988.

4046. Balquis F, Sohail MF, Hamid H, Ullah W, Khan AH, Shahnaz G. Potential and weak links in the management of tuberculosis by Pakistani private pharmacy staff. Frontiers in Public Health. 2023;11. doi: 10.3389/fpubh.2023.983997.

4047. Balsom C, Kelly D, Legge K, King R, Vaters M, Pittman N, et al. Impact of a pharmacist-administered deprescribing intervention on nursing home residents: a randomized controlled trial. Canadian pharmacists journal. 2018;151(5):S20-. doi: 10.1177/1715163518797716. PubMed PMID: CN-01653540.

4048. Balsom C, Pittman N, King R, Kelly D. Impact of a pharmacist-administered deprescribing intervention on nursing home residents: a randomized controlled trial. International journal of clinical pharmacy. 2020;42(4):1153-67. doi: 10.1007/s11096-020-01073-6. PubMed PMID: CN-02130826.

4049. Bameshki A, Jahanbakhsh S. Abdominal pain after cataract surgery with remifentanil based anesthesia. Iranian journal of pharmaceutical research. 2009;8(1):47-51. PubMed PMID: CN-00754130.

4050. Bamgbade BA, Drph KHF, Barner JC. Impact of a Mental Illness Stigma Awareness Intervention on Pharmacy Student Attitudes and Knowledge. American Journal of Pharmaceutical Education. 2016;80(5). doi: 10.5688/ajpe80580. PubMed PMID: WOS:000379511400009.

4051. Bamgbade BA, McManus DD, Briesacher BA, Lessard D, Mehawej J, Gurwitz JH, et al. Medication

cost-reducing behaviors in older adults with atrial fibrillation: The SAGE-AF study. Journal of the American Pharmacists Association. 2023;63(1):125-34. doi: https://doi.org/10.1016/j.japh.2022.08.030.

4052. Bamgbade BA, McManus DD, Briesacher BA, Lessard D, Mehawej J, Gurwitz JH, et al. Medication cost-reducing behaviors in older adults with atrial fibrillation: The SAGE-AF study. Journal of the American Pharmacists Association. 2023;63(1):125-34. doi: https://doi.org/10.1016/j.japh.2022.08.030.

4053. Bamgboye AO, Hassan IA, Adebisi YA, Farayola RO, Uwizeyimana T. Towards improving community pharmacy-based mental health services in Nigeria. Journal of Pharmaceutical Policy and Practice. 2021;14(1). doi: 10.1186/s40545-021-00316-9.

4054. Bamidele EF, Amaike C, Taiwo AA, Egejuru JC, Wali GO, Ukpai CU, et al. Healthcare-Seeking Behaviours of Undergraduate Students and Their Perception of Health Services in a Nigerian Private University Health Centre. Annals of Health Research. 2023;9(2):108-19. doi: 10.30442/ahr.0902-03-196.

4055. Bamiro OA, Odeniyi MA, Osonuga OA. Brand variations in the physicochemical properties of metronidazole tablets. Nigerian quarterly journal of hospital medicine. 2007;17(1):22-5.

4056. Ban B, Hodgins S, Thapa P, Thapa S, Joshi D, Dhungana A, et al. A national survey of private-sector outpatient care of sick infants and young children in Nepal. Bmc Health Services Research. 2020;20(1). doi: 10.1186/s12913-020-05393-1. PubMed PMID: WOS:000542957500004.

4057. Ban B, Hodgins S, Thapa P, Thapa S, Joshi D, Dhungana A, et al. A national survey of private-sector outpatient care of sick infants and young children in Nepal. BMC Health Services Research. 2020;20(1):545. PubMed PMID: 32546276.

4058. Ban JK, Hao BB, McCarthy L, Guilcher SJT, Cadarette SM. Denosumab utilization among older adults in Ontario: patient characteristics, persistence with therapy, and return to therapy after an extended gap. Osteoporosis International. 2019;30(9):1865-72. doi: 10.1007/s00198-019-05051-1.

4059. Banack HR, Grover S, Kaouche M, Marchand S, Lowensteyn I. The MyHealthCheckup study: Training graduate students to implement cardiovascular risk screening programs in community pharmacies. Canadian Pharmacists Journal. 2012;145(6):268-75. doi: 10.3821/145.6.cpj268.

4060. Banahan BF, Holmes ER. A MODEL TO ESTIMATE INCREASE IN REVENUE FROM IMPLEMENTING MEDICATION ADHERENCE MANAGEMENT SERVICES IN COMMUNITY PHARMACIES. Value in Health. 2010;13(3):A93-A. doi: 10.1016/s1098-3015(10)72442-6. PubMed PMID: WOS:000277121900459.

4061. Banakh I, Lam A, Turek M, Htet T, Vorlander C. Rapid versus standard iron polymaltose infusions: a single centre safety study. Journal of pharmacy practice and research. 2016;(no pagination). doi: 10.1002/jppr.1236. PubMed PMID: CN-01289393.

4062. Banawis M, Mah G, Mohsin R, Pobre J, Tracy D, Song AV, et al. An observational study of the extent of naloxone furnishing in California Central Valley community pharmacies. Journal of the American Pharmacists Association. 2022. doi: 10.1016/j.japh.2022.10.028.

4063. Banawis M, Mah G, Mohsin R, Pobre J, Tracy D, Song AV, et al. An observational study of the extent of naloxone furnishing in California Central Valley community pharmacies. Journal of the American Pharmacists Association. 2023;63(2):566-73. doi: 10.1016/j.japh.2022.10.028.

4064. Bancescu AA, Ionescu TM, Bancescu G, Defta CL. ANTIBIOTIC RESISTANCE PHENOTYPES OF S. AUREUS ISOLATES FROM NOSE AND PHARYNX OF HEALTHY YOUNG ADULTS. Farmacia.

2022;70(1):42-6. doi: 10.31925/farmacia.2022.1.6. PubMed PMID: WOS:000767296200006.

4065. Bancroft T, Lim J, Wang C, Sander SD, Swindle JP. Health Care Resource Utilization, Costs, and Persistence in Patients Newly Diagnosed as Having Nonvalvular Atrial Fibrillation and Newly Treated With Dabigatran versus Warfarin in the United States. Clinical Therapeutics. 2016;38(3):545-56. doi: 10.1016/j.clinthera.2016.01.008. PubMed PMID: WOS:000373248600012.

4066. Bandara K, Bergmeir C, Campbell S, Scott D, Lubman D, Ieee, editors. Towards Accurate Predictions and Causal 'What-if' Analyses for Planning and Policy-making: A Case Study in Emergency Medical Services Demand. International Joint Conference on Neural Networks (IJCNN) held as part of the IEEE World Congress on Computational Intelligence (IEEE WCCI); 2020 Jul 19-24; Electr Network2020.

4067. Bandaranayake WM. Quality control, screening, toxicity, and regulation of herbal drugs. Modern phytomedicine: turning medicinal plants into drugs. 2006:25-57.

4068. Bandhu A, Sarkar S, Karmakar S, Singh OP. Level of Satisfaction Towards Healthcare Services in Patients Attending Psychiatry Outpatient Department of a Tertiary Care Hospital in Eastern India. Indian Journal of Psychological Medicine. 2023;45(6):591-7. doi: 10.1177/02537176231163580. PubMed PMID: WOS:000971173700001.

4069. BANDIERA C. AN INTERPROFESSIONAL INTERVENTION TO SUPPORT ADHERENCE TO COMPLEX AND POLYPHARMACY TREATMENTS IN PATIENTS WITH SOLID CANCER AND PATIENTS WITH DIABETIC KIDNEY DISEASE: Université de Genève; 2023.

4070. Bandiera C, Locatelli I, Courlet P, Cardoso E, Zaman K, Stravodimou A, et al. Adherence to the CDK 4/6 inhibitor palbociclib and omission of dose management supported by pharmacometric modelling as part of the OpTAT study. Cancers. 2023;15(1):316.

4071. Bandurska E, Zaleska J, Zarzeczna-Baran M. POLES IN PHARMACY - SOURCE OF KNOWLEDGE AND PREVALENCE OF OVER THE COUNTER USAGE. Acta Poloniae Pharmaceutica. 2018;75(4):1041-9. doi: 10.32383/appdr/80862. PubMed PMID: WOS:000447231500021.

4072. Banerjee A, O'Hearn T. Drug Revolving Funds: A low-cost sustainable intervention? Tropical Doctor. 2001;31(1):34-5. doi: 10.1177/004947550103100113.

4073. Banerjee AK. Pharmacists are key to enhancing benefit risk for medicines. Archives of Internal Medicine. 2009;169(18):1723; author reply -4. PubMed PMID: 19822832.

4074. Banerjee J, Ghosh R, Paul A, Banik S, Halder S, Sarkar J. An In-Depth Analysis of Covid-19 Medications and Vaccination with A Focus on Side Effects. National Journal of Community Medicine. 2023;14(11):752-68. doi: 10.55489/njcm.141120233243. PubMed PMID: 175928773.

4075. Banerjee S, Bhattacharjee P, Kar A, Mukherjee PK. LC–MS/MS analysis and network pharmacology of Trigonella foenum-graecum–A plant from Ayurveda against hyperlipidemia and hyperglycemia with combination synergy. Phytomedicine. 2019;60:152944.

4076. Banerjee S, Garrison L, Flum D, Arterburn D. Cost and healthcare use implications of bariatric surgery vs lifestyle/medical intervention for type 2 diabetes. Value in health. 2017;20(5):A170-. PubMed PMID: CN-01407784.

4077. Banerjee S, Garrison LP, Flum DR, Arterburn DE. Cost and Health Care Utilization Implications of Bariatric Surgery Versus Intensive Lifestyle and Medical Intervention for Type 2 Diabetes. Obesity (Silver Spring, Md). 2017;25(9):1499-508. doi: 10.1002/oby.21927. PubMed PMID: CN-01446879.

4078. Banerjee S, Hellier J, Romeo R, Dewey M, Knapp M, Ballard C, et al. Study of the use of antidepressants for depression in dementia: the HTA-SADD trial--a multicentre, randomised, double-blind, placebo-controlled trial of the clinical effectiveness and cost-effectiveness of sertraline and mirtazapine. Health technology assessment (Winchester, England). 2013;17(7):1-166. doi: 10.3310/hta17070. PubMed PMID: CN-00863315.

4079. Banerjee S, Hellier J, Romeo R, Dewey M, Knapp M, Ballard C, et al. Study of the use of antidepressants for depression in dementia: The HTA-SADD trial- A multicentre, randomised, double-blind, placebo-controlled trial of the clinical effectiveness and cost-effectiveness of sertraline and mirtazapine. Health Technology Assessment. 2013;17(7):1-43. doi: 10.3310/hta17070.

4080. Banerjee S, Nau S, Hochwald SN, Xie H, Zhang J. Anticancer properties and mechanisms of botanical derivatives. Phytomedicine Plus. 2023;3(1):100396. doi: https://doi.org/10.1016/j.phyplu.2022.100396.

4081. Banerjee S, Nau S, Hochwald SN, Xie H, Zhang J. Anticancer properties and mechanisms of botanical derivatives. Phytomedicine Plus. 2023;3(1):100396. doi: https://doi.org/10.1016/j.phyplu.2022.100396.

4082. Banerji V, Dawe DE, Xue L, Bucher O, Yang L, Johnston JB, et al. Clime: CLL Immune Micro-Environment and Risk of Infections Pre- and Post-Diagnosis: A Population-Based Study. Blood. 2023;142:7396. doi: https://doi.org/10.1182/blood-2023-187573.

4083. Bangen M. An evaluation of drug prescribing in veterinary medicine in Norway [Dr.Sc.]. Norway: Norges Veterinaerhogskole (Norway); 1995.

4084. Banh HL, Cave AJ. A De Novo Pharmacist-Family Physician Collaboration Model in a Family Medicine Clinic in Alberta, Canada. Pharmacy. 2021;9(2). doi: 10.3390/pharmacy9020107. PubMed PMID: WOS:000665604300001.

4085. Banh HL, Chow S, Li S, Letassy N, Cox C, Cave A. Pharmacy students screening for pre-diabetes/ diabetes with a validated questionnaire in community pharmacies during their experiential rotation in Alberta, Canada. SAGE Open Medicine. 2015;3. doi: 10.1177/2050312115585040.

4086. Bank PCD, Swen JJ, Guchelaar HJ. Estimated nationwide impact of implementing a preemptive pharmacogenetic panel approach to guide drug prescribing in primary care in the Netherlands. BMC Medicine. 2019;17(1). doi: 10.1186/s12916-019-1342-5.

4087. Bankes D, Pizzolato K, Finnel S, Awadalla MS, Stein A, Johnson J, et al. Medication-related problems identified by pharmacists in an enhanced medication therapy management model. American Journal of Managed Care. 2021;27(16 Suppl):S292-S9. PubMed PMID: 34529368.

4088. Bankes DL, Amin NS, Bardolia C, Awadalla MS, Knowlton CH, Bain KT. Medication-related problems encountered in the Program of All-Inclusive Care for the Elderly: An observational study. Journal of the American Pharmacists Association. 2020;60(2):319-27. doi: https://doi.org/10.1016/j.japh.2019.10.012.

4089. Bankes DL, Jin H, Williams R, Awadalla MS, Johnson J, Turgeon J, et al. Implementing pharmacists' medication safety recommendations is associated with reduced health care resource utilization and mortality in high-risk older Americans. Journal of the American Pharmacists Association. 2023;63(1):118-24. doi: https://dx.doi.org/10.1016/j.japh.2022.08.022. PubMed PMID: 2020398766.

4090. Bankes DL, Jin H, Williams R, Awadalla MS, Johnson J, Turgeon J, et al. Implementing pharmacists' medication safety recommendations is associated with reduced health care resource utilization and mortality in high-risk older Americans. Journal of the American Pharmacists Association. 2023;63(1):118-24. doi:

https://doi.org/10.1016/j.japh.2022.08.022.

4091. Bankhead R, Boullata J, Brantley S, Corkins M, Guenter P, Krenitsky J, et al. ASPEN enteral nutrition practice recommendations. Journal of Parenteral and Enteral Nutrition. 2009;33(2):122-67.

4092. Banks AM, Peter ME, Holder GM, Jolly JA, Markley BM, Zuckerman SL, et al. Adherence to Disease-Modifying Therapies at a Multiple Sclerosis Clinic: The Role of the Specialty Pharmacist. Journal of Pharmacy Practice. 2020;33(5):605-11. doi: 10.1177/0897190018824821. PubMed PMID: WOS:000584468800006.

4093. Banks J, Shaw A, Weiss MC. Walking a line between health care and sales: The role of the medicines counter assistant. Pharmaceutical Journal. 2005;274(7349):586-9.

4094. Banks J, Shaw A, Weiss MC. The community pharmacy and discursive complexity: A qualitative study of interaction between counter assistants and customers. Health and Social Care in the Community. 2007;15(4):313-21. doi: 10.1111/j.1365-2524.2007.00690.x.

4095. Bannan D, Alshibani M, Alshehri S, Aljabri A, Kutbi H. Assessing factors influencing pharmacy interns career choices in Saudi Arabia. Saudi Pharmaceutical Journal. 2021;29(1):67-72. doi: 10.1016/j.jsps.2020.12.010.

4096. Bannerman JP. Why community pharmacists are in short supply as fellows [12]. Pharmaceutical Journal. 2004;272(7281):17.

4097. Banning J, Vogel L. Stalled progress on reconciliation in health care. CMAJ Canadian Medical Association Journal. 2022;194(11):E424-E5. PubMed PMID: 35314445.

4098. Bannon L, McGaughey J, Clarke M, McAuley DF, Blackwood B. Designing a nurse-delivered delirium bundle: What intensive care unit staff, survivors, and their families think? Australian Critical Care. 2018;31(3):174-9. doi: https://doi.org/10.1016/j.aucc.2018.02.007.

4099. Bano T, Haq N, Nasim A, Saood M, Tahir M, Yasmin R, et al. Evaluation of medication errors in patients with kidney diseases in Quetta, Pakistan. PLoS ONE. 2023;18(8):1-12. doi: 10.1371/journal.pone.0289148. PubMed PMID: 169729198.

4100. Bansal M, Shah M, Reilly B, Willman S, Gill M, Kaufman FR. Impact of Reducing Glycated Hemoglobin on Healthcare Costs Among a Population with Uncontrolled Diabetes. Applied Health Economics and Health Policy. 2018;16(5):675-84. doi: 10.1007/s40258-018-0398-2. PubMed PMID: WOS:000444184100008.

4101. Bansal N, Khawagi WY, Shang N, Chen L-C. Enhancing Patient Safety in Opioid Prescribing: A Systematic Review of Potential Indicators. medRxiv. 2023:2023.12. 07.23299686.

4102. Bansal N, Tai WT, Chen LC. Implementation of an innovative surgical pharmacy service to improve patient outcomes-Twelve-month outcomes of the Enhanced Surgical Medicines Optimization Service. Journal of Clinical Pharmacy & Therapeutics. 2019;44(6):904-11. PubMed PMID: 31497885.

4103. Bao HK, Dubrovskaya Y, Jen SP, Decano A, Ahmed N, Pham VP, et al. Novel Multidisciplinary Approach for Outpatient Antimicrobial Stewardship Using an Emergency Department Follow-Up Program. Journal of Pharmacy Practice. doi: 10.1177/08971900211048311. PubMed PMID: WOS:000705182100001.

4104. Bao L, Wang Y, Shang T, Ren X, Ma R. A Novel Clinical Pharmacy Management System in Improving the Rational Drug Use in Department of General Surgery. Indian Journal of Pharmaceutical Sciences.

2013;75(1):11-5. doi: 10.4103/0250-474x.113531. PubMed PMID: WOS:000321143700003.

4105. Bao LD, Peng R, Wang Y, Ma RL, Ren XH, Meng WB, et al. Significant Reduction of Antibiotic Consumption and Patients' Costs after an Action Plan in China, 2010-2014. Plos One. 2015;10(3). doi: 10.1371/journal.pone.0118868. PubMed PMID: WOS:000351277500052.

4106. Bao Y, Pan Y, Taylor A, Radakrishnan S, Luo F, Pincus HA, et al. Prescription drug monitoring programs are associated with sustained reductions in opioid prescribing by physicians. Health Affairs. 2016;35(6):1045-51.

4107. Bao Y, Wang C, Xu H, Lai Y, Yan Y, Ma Y, et al. Effects of an mHealth Intervention for Pulmonary Tuberculosis Self-management Based on the Integrated Theory of Health Behavior Change: randomized Controlled Trial. JMIR public health and surveillance. 2022;8(7):e34277. doi: 10.2196/34277. PubMed PMID: CN-02426152.

4108. Bao Y, Wen K, Johnson P, Jeng PJ, Meisel ZF, Schackman BR. Assessing the impact of state policies for prescription drug monitoring programs on high-risk opioid prescriptions. Health Affairs. 2018;37(10):1596-604. doi: 10.1377/hlthaff.2018.0512.

4109. Bao YH, Wen K, Johnson P, Jeng PJ, Meisel ZF, Schackman BR. Assessing The Impact Of State Policies For Prescription Drug Monitoring Programs On High-Risk Opioid Prescriptions. Health Affairs. 2018;37(10):1596-604. doi: 10.1377/hlthaff.2018.0512. PubMed PMID: WOS:000463964200009.

4110. Bao Z. Research on the method to estimate the municipal waste quality in Wuhan [M.Eng.]. Peoples Rep. of China: Wuhan University of Technology (People's Republic of China); 2005.

4111. Bao Z, Ji C, Hu J, Luo C, Fang W. Clinical and economic impact of pharmacist interventions on sampled outpatient prescriptions in a Chinese teaching hospital. BMC Health Services Research. 2018;18(1):N.PAG-N.PAG. doi: 10.1186/s12913-018-3306-4. PubMed PMID: 130571452. Language: English. Entry Date: In Process. Revision Date: 20181215. Publication Type: journal article. Journal Subset: Biomedical.

4112. Baptista ARN. Protetores Gastrointestinais em Clínica de Animais Companhia : Da Farmácia Galénica às Atuais Recomendações [Master's]. Portugal: Universidade de Lisboa (Portugal); 2020.

4113. Baptista CJ, Santos JED, Galassi AD. Socioeconomic and demographic variables associated with self-reported domestic violence during "Stay at Home!": cross-sectional study with a sample from a university community. Mundo Da Saude. 2023;47(1). doi: 10.15343/0104-7809.202347e14052022P. PubMed PMID: WOS:001110417400018.

4114. Baptista R, Williams M, Price J. Using xPIRT to Record Pharmacy Interventions: An Observational, Cross-Sectional and Retrospective Study. Healthcare (Switzerland). 2022;10(12). doi: 10.3390/healthcare10122450.

4115. Baptista R, Williams M, Price J. Improving the impact of pharmacy interventions in hospitals. BMJ Open Quality. 2023;12(4). doi: 10.1136/bmjoq-2023-002276.

4116. Baptista T, Novoa D, Hernández R. Substance use among Venezuelan medical and pharmacy students. Drug and Alcohol Dependence. 1994;34(2):121-7. doi: https://doi.org/10.1016/0376-8716(94)90132-5.

4117. Baqir W, Jones K, Horsley W, Barrett S, Fisher D, Copeland R, et al. Reducing unacceptable missed doses: pharmacy assistant-supported medicine administration. International Journal of Pharmacy Practice. 2015;23(5):327-32. doi: 10.1111/ijpp.12172. PubMed PMID: 109539976. Language: English. Entry Date:

20150923. Revision Date: 20181204. Publication Type: Article.

4118. Baqir W, Maguire A. Consumption of prescribed and over-the-counter medicines with prolonged oral clearance used by the elderly in the Northern Region of England, with special regard to generic prescribing, dose form and sugars content. Public Health. 2000;114(5):367-73. doi: 10.1038/sj.ph.1900673.

4119. Baqir W, Paes P, Stoker A, Morris E, McWhir R, Ridley H, et al. Impact of an integrated pharmacy service on hospital admission costs. Clinical Pharmacist. 2018;10(5). doi: 10.1211/CP.2018.20204550.

4120. Bar D, Lavie O, Stein N, Feferkorn I, Shai A. The effect of metabolic comorbidities and commonly used drugs on the prognosis of patients with ovarian cancer. European Journal of Obstetrics & Gynecology and Reproductive Biology. 2016;207:227-31. doi: https://doi.org/10.1016/j.ejogrb.2016.09.005.

4121. Barach P, Small SD. Reporting and preventing medical mishaps: lessons from non-medical near miss reporting systems. Bmj. 2000;320(7237):759-63.

4122. Baraitser P, Pearce V, Holmes J, Horne N, Boynton PM. Chlamydia testing in community pharmacies: Evaluation of a feasibility pilot in south east London. Quality and Safety in Health Care. 2007;16(4):303-7. doi: 10.1136/qshc.2006.020883.

4123. Barak N, Rice H, Kamsler A. Variability of compounded topical 0.2% nifedipine formulations. Journal of Pharmacy Practice and Research. 2019;49(2):130-4. doi: 10.1002/jppr.1470.

4124. Baraka MA, Elnaem MH, Elkalmi R, Sadeq A, Elnour AA, Chacko RJ, et al. Awareness of statin-food interactions using grapefruit as an example: a cross-sectional study in Eastern Province of Saudi Arabia. Journal of Pharmaceutical Health Services Research. 2021;12(4):545-51. doi: 10.1093/jphsr/rmab047. PubMed PMID: WOS:000736131400012.

4125. Baraka MA, Elnaem MH, Elkalmi R, Sadeq A, Elnour AA, Joseph Chacko R, et al. Awareness of statin-food interactions using grapefruit as an example: A cross-sectional study in Eastern Province of Saudi Arabia. Journal of Pharmaceutical Health Services Research. 2021;12(4):545-51. doi: 10.1093/jphsr/rmab047.

4126. Barakat HE, Aziz CN, Abougalambou SSI. Evaluation of the knowledge, practices, and attitudes of community pharmacists towards adverse effects of non-steroidal anti-inflammatory drugs (NSAIDs): a cross-sectional study. Journal of Pharmaceutical Policy and Practice. 2023;16(1):132. doi: https://dx.doi.org/10.1186/s40545-023-00641-1. PubMed PMID: 2026407511.

4127. Barakat M, Al-Qudah R, Akour A, Al-Qudah N, Bashi YDH. Unforeseen uses of oral contraceptive pills: Exploratory study in Jordanian community pharmacies. Plos One. 2020;15(12). doi: 10.1371/journal.pone.0244373. PubMed PMID: WOS:000603071100032.

4128. Barakat M, Al-Qudah R, Akour A, Al-Qudah N, DallalBashi YH. Unforeseen uses of oral contraceptive pills: Exploratory study in Jordanian community pharmacies. PLoS ONE. 2021;15(12 December). doi: 10.1371/journal.pone.0244373.

4129. Barakat M, Mansour NO, Hassan Elnaem M, Thiab S, Abu Farha R, Sallam M, et al. Evaluation of knowledge, experiences, and fear toward prescribing and dispensing corticosteroids among Egyptian healthcare professionals: A cross-sectional study. Saudi Pharmaceutical Journal. 2023;31(10):101777. doi: https://doi.org/10.1016/j.jsps.2023.101777.

4130. Barake S, Tofaha R, Rahme D, Lahoud N. The Health Status of Lebanese Community Pharmacists: prevalence of Poor Lifestyle Behaviors and Chronic Conditions. Saudi pharmaceutical journal.

2021;29(6):497-505. doi: 10.1016/j.jsps.2021.04.005. PubMed PMID: CN-02284643.

4131. Barako TD. Exploring Antenatal Care Experiences Amongst Pastoralist Communities in Kenya : a Focused Ethnographic Study [Ph.D.]. England: The University of Nottingham (United Kingdom); 2021.

4132. Baraniuk C. FATAL SYSTEM ERROR: There are killer bugs going round hospitals -- and not just medical ones, says Chris Baraniuk. New Scientist. 2018;238(3178):34. doi: 10.1016/S0262-4079(18)30885-6. PubMed PMID: 129660240.

4133. Baranowski PJ, Peterson KL, Statz-Paynter JL, Zorek JA. Incidence and cost of medications dispensed despite electronic medical record discontinuation. Journal of the American Pharmacists Association. 2015;55(3):313-9. doi: https://doi.org/10.1331/JAPhA.2015.14154.

4134. Baranwal D, Hetal D. A clinical study of Kashmaryadi Ghrit Uttar Basti in female infertility. International journal of research in ayurveda and pharmacy. 2019;10(1):35-9. PubMed PMID: CN-01929650.

4135. Barat E, Pouplin S, Chenailler C. The pharmacist's role in dealing with pain. Actualites Pharmaceutiques. 2023;62(629):33-7. doi: https://dx.doi.org/10.1016/j.actpha.2023.07.008. PubMed PMID: 2027259207.

4136. Barat E, Vanbergue B, Leguillon R, Chenailler C, Arrii M, Curado J, et al. Tobacco and surgery: A survey of health professionals' practices and knowledge. A role for the community pharmacist. Annales Pharmaceutiques Francaises. 2023;81(6):1082-9. doi: 10.1016/j.pharma.2023.07.005. PubMed PMID: WOS:001102858400001.

4137. Barata J, Maia F, Mascarenhas A. Digital transformation of the mobile connected pharmacy: a first step toward community pharmacy 5.0. Informatics for Health & Social Care. 2022;47(4):347-60. doi: 10.1080/17538157.2021.2005603. PubMed PMID: WOS:000725431500001.

4138. Barata JMAM. Caracterização do Perfil Farmacoterapêutico dos Utentes de Uma Farmácia da Região “Marão e Douro Norte” Com Enquadramento Regional e Nacional [M.Sc.]. Portugal: Universidade Fernando Pessoa (Portugal); 2017.

4139. Barata JPM. Análise dos Efeitos do Tratamento da Hipertensão na Gravidez: Experiência Profissionalizante na Vertente de Farmácia Comunitária e Investigação [Master's]. Portugal: Universidade da Beira Interior (Portugal); 2013.

4140. Barata RD. Conversão de Antibióticos e de Paracetamol da via Intravenosa Para a via Oral: Experiência Profissionalizante na Vertente de Farmácia Comunitária, Hospitalar e Investigação [M.Sc.]. Portugal: Universidade da Beira Interior (Portugal); 2016.

4141. Barati O, Najibi M, Yusefi AR, Dehghan H, Delavari S. Outsourcing in shiraz university of medical sciences; a before and after study. Journal of the Egyptian Public Health Association. 2019;94(1):1-8. doi: 10.1186/s42506-019-0010-0.

4142. Baratt Y, Li H, Patel N, Strobino K, Kronish I, Elkind M, et al. Clinical pharmacy services as part of the Telehealth After Stroke Care in an underserved setting: a feasibility study. Neurology. 2022;98(18 SUPPL). PubMed PMID: CN-02421911.

4143. Baratta F, Allais G, Rolando S, Manzoni GC, Bussone G, Benedetto C, et al. Prevention, education and counselling: the worldwide role of the community pharmacist as an epidemiological sentinel of headaches. Neurological Sciences. 2019;40:S15-S21. doi: 10.1007/s10072-019-03794-7. PubMed PMID: WOS:000467932000004.

4144. Baratta F, Di Lascio G, Tarditi F, Petieau R, Brusa P. Galenic formulations to fight the phenomenon of counterfeiting in developing Countries. Journal of Drug Delivery Science and Technology. 2016;32:313-5. doi: 10.1016/j.jddst.2015.10.006.

4145. Baratta F, Enri LR, Brusa P. Community pharmacists as vaccinators in the Italian SARS-CoV-2 immunization campaign: implications beyond the pandemic. Health Policy. 2023;131. doi: 10.1016/j.healthpol.2023.104798. PubMed PMID: WOS:001029156600001.

4146. Baratta F, Folpini E, Ciccolella M, Brusa P. Patient Care in Community Pharmacies during the COVID-19 (SARS-CoV-2) Pandemic: Effectiveness of Post-Graduate Education and Further Training Courses on Revenues. International Journal of Environmental Research and Public Health. 2023;20(5). doi: 10.3390/ijerph20053774.

4147. Baratta F, Germano A, Di Lascio G, Petieau R, Brusa P. Establishment of galenic laboratories in developing countries to produce high quality medicines: Results of Aid Progress Pharmacist Agreement (A.P.P.A.®) Project. Croatian Medical Journal. 2014;55(6):662-8. doi: 10.3325/cmj.2014.55.662.

4148. Baratta F, Peira E, Maza C, Gallarate M, Brusa P. Cannabis-Based Oral Emulsion for Medical Purposes to Meet the Needs of Patients: Formulation, Quality and Stability. Pharmaceutics. 2022;14(3). doi: 10.3390/pharmaceutics14030513.

4149. Baratta F, Pignata I, Onorati R, Gnavi R, Costa G, Brusa P, et al. Monitoring and screening COPD in community pharmacies: experimentation in Italy. Pharmacy Practice. 2023;21(3). doi: 10.18549/PharmPract.2023.3.2854.

4150. Baratta F, Pignata I, Vicenzi G, Enri LR, Quaglino P, Comandone A, et al. Oncological patient management on the territory: the results of a survey in the north-west of Italy. Pharmacy Practice. 2022;20(4). doi: 10.18549/PharmPract.2022.4.2716.

4151. Barbanel D, Eldridge S, Griffiths C. Can a self-management programme delivered by a community pharmacist improve asthma control? A randomised trial. Thorax. 2003;58(10):851-4. doi: 10.1136/thorax.58.10.851. PubMed PMID: CN-00440546.

4152. Barbarisi I, Bruno G, Diglio A, Elizalde J, Piccolo C. A spatial analysis to evaluate the impact of deregulation policies in the pharmacy sector: Evidence from the case of Navarre. Health Policy. 2019;123(11):1108-15. doi: https://doi.org/10.1016/j.healthpol.2019.08.010.

4153. Barbazza E, Verheij RA, Ramerman L, Klazinga N, Kringos D. Optimising the secondary use of primary care prescribing data to improve quality of care: A qualitative analysis. BMJ Open. 2022;12(7). doi: 10.1136/bmjopen-2022-062349.

4154. Barber C, Gagnon D, Fonda J, Cho K, Hermos J, Miller M. Assessing the impact of prescribing directives on opioid prescribing practices among Veterans Health Administration providers. Pharmacoepidemiology and drug safety. 2017;26(1):40-6. doi: 10.1002/pds.4066. PubMed PMID: CN-02148422.

4155. Barber C, Lacaille D, Fortin PR. Systematic review of validation studies of the use of administrative data to identify serious infections. Arthritis Care and Research. 2013;65(8):1343-57. doi: 10.1002/acr.21959.

4156. Barber C, Mashburn P, Kennelty K, Look K, Stover A, Westrick S. O.27.5 - Exploring racial differences in opioid overdose terminology preferences for rural patients: Presenter(s): Delesha Carpenter, University of North Carolina at Chapel Hill, United States. Patient Education and Counseling. 2023;109:105. doi: https://doi.org/10.1016/j.pec.2022.10.243.

4157. Barber N, Alldred DP, Raynor D, Dickinson R, Garfield S, Jesson B, et al. Care homes' use of medicines study: prevalence, causes and potential harm of medication errors in care homes for older people. BMJ Quality & Safety. 2009;18(5):341-6.

4158. Barber N, Dean B. The incidence of medication errors and ways to reduce them. Clinical Risk. 1998;4(4):103-6.

4159. Barber N, Parsons J, Clifford S, Darracott R, Horne R. Patients' problems with new medication for chronic conditions. BMJ Quality & Safety. 2004;13(3):172-5.

4160. Barber N, Rawlins M, Dean Franklin B. Reducing prescribing error: competence, control, and culture. Quality & Safety in Health Care. 2003;12 Suppl 1:i29-32. PubMed PMID: 14645746.

4161. Barber N, Rawlins M, Franklin BD. Reducing prescribing error: competence, control, and culture. BMJ Quality & Safety. 2003;12(suppl 1):i29-i32.

4162. Barber N, Safdar A, Franklin BD. Can human error theory explain non-adherence? Pharmacy World and Science. 2005;27:300-4.

4163. Barber ND. Care homes' use of medicines study: prevalence, causes and potential harm of medication errors in care homes for older people.

4164. Barber ND, Alldred DP, Raynor DK. Care homes' use of medicines study: prevalence, causes and potential harm of medication errors in care homes for older people.

4165. Barber ND, Alldred DP, Raynor DK, Dickinson R, Garfield S, Jesson B, et al. Care homes' use of medicines study: prevalence, causes and potential harm of medication errors in care homes for older people. Quality & Safety in Health Care. 2009;18(5):341-6. doi: 10.1136/qshc.2009.034231. PubMed PMID: 105235849. Language: English. Entry Date: 20100101. Revision Date: 20150711. Publication Type: Journal Article.

4166. Barbero A, Alfonso Galán T. Responding to symptoms in community pharmacy. Pharmaceutical Care Espana. 2002;4(2):106-17.

4167. Barbero FC, Gimenez MDG, Guerrero AMQ. Potential interactions between phytotherapycs anthracenics laxatives products and other drugs. Detection in community pharmacies of Seville. Farmaceuticos Comunitarios. 2018;10(4):5-13. doi: 10.5672/FC.2173-9218.(2018/Vol10).004.02. PubMed PMID: WOS:000454562500002.

4168. Barbero González A, Álvarez de Toledo Saavedra F, Esteban Fernández J, Pastor-Sánchez R, Gil de Miguel A, Rodríguez Barrios JM, et al. Seguimiento desde la farmacia comunitaria de la vacunación y la quimioprofilaxis de los viajeros internacionales. Estudio VINTAF. Atención Primaria. 2003;32(5):276-81. doi: https://doi.org/10.1016/S0212-6567(03)79275-0.

4169. Barbero González A, Álvarez de Toledo Saavedra F, Esteban Fernández J, Pastor-Sánchez R, Gil de Miguel A, Rodríguez Barrios JM, et al. Management of vaccinations and prophylaxis of international travellers from community pharmacy (VINTAF Study). Atencion Primaria. 2003;32(5):276-81. doi: 10.1157/13051594.

4170. Barbero González JA. Detection of drug-related problems in the community pharmacy: Registered users versus non-registered users. Pharmaceutical Care Espana. 2001;3(3):204-15.

4171. Barbero González JA, Quintas Rodríguez AM, Camacho JE. Smoking cessation from the community

pharmacy. Atencion primaria / Sociedad Española de Medicina de Familia y Comunitaria. 2000;26(10):693-6. doi: 10.1016/S0212-6567(00)78754-3.

4172. Barbero-González A, Pastor-Sánchez R, del Arco-Ortiz de Zárate J, Eyaralar-Riera T, Espejo-Guerrero J. Demanda de medicamentos de prescripción sin receta médica. Atención Primaria. 2006;37(2):78-87. doi: https://doi.org/10.1157/13084485.

4173. Barbero-González A, Pastor-Sánchez R, Del Arco-Ortiz De Zárate J, Eyaralar-Riera T, Espejo-Guerrero J. Demand for dispensing of medicines without medical prescription. Atencion Primaria. 2006;37(2):78-87. doi: 10.1157/13084485.

4174. Barbier E, Chaumais MC, Launay-Vacher G, Rieutord A, Haddad R. Training of the pharmaceutical team to vitamin K antagonists (VKA) consultations: A blended-learning experience in hospital. Journal de Pharmacie Clinique. 2014;33(3):125-32. doi: 10.1684/jpc.2014.0285.

4175. Barbier G, Berthou J, Medjoub M, Limat S, Clairet AL. Mise en place d'un livret thérapeutique gériatrique territorial : concilier la performance achat et la sécurité d'usage. Le Pharmacien Clinicien. 2022;57(3):254-61. doi: https://doi.org/10.1016/j.phacli.2021.11.002.

4176. Barbier L, Vandenplas Y, Simoens S, Declerck P, Vulto AG, Huys I. Knowledge and perception of biosimilars in ambulatory care: a survey among Belgian community pharmacists and physicians. Journal of Pharmaceutical Policy and Practice. 2021;14(1). doi: 10.1186/s40545-021-00330-x.

4177. Barbieri E, Minotti C, Cavagnis S, Giaquinto C, Cappello B, Penazzato M, et al. Paediatric medicine issues and gaps from healthcare workers point of view: survey results and a narrative review from the global accelerator for paediatric formulations project. Frontiers in Pharmacology. 2023;14:1200848. doi: https://dx.doi.org/10.3389/fphar.2023.1200848. PubMed PMID: 2024772728.

4178. Barbieri M, Drummond M, Willke R, Chancellor J, Jolain B, Towse A. Variability of cost-effectiveness estimates for pharmaceuticals in Western Europe: lessons for inferring generalizability. Value in health. 2005;8(1):10-23.

4179. Barbieri MA, Irrera N, Convertino I. Drug Safety and Relevant Issues in the Real-World. Pharmaceuticals (14248247). 2023;16(12):1689. doi: 10.3390/ph16121689. PubMed PMID: 174464594.

4180. Barbosa TFP. Implementação de Alertas de Interações Medicamentosas na Construção de uma Farmácia Online [Master's]. Portugal: Instituto Politecnico do Porto (Portugal); 2014.

4181. Barbour E, Hughes J, Gill D. Improving the completion of the medication prescription and administration record on a paediatric ward. Archives of disease in childhood. 2018;103(2). doi: 10.1136/archdischild-2017-314585.20. PubMed PMID: CN-01619784.

4182. Barbour SY, Braccia D. Efficacy and safety of the granisetron transdermal system for chemotherapy-induced nausea and vomiting in elderly patients. Journal of oncology pharmacy practice. 2014;20(2 SUPPL. 1):11-2. doi: 10.1177/1078155213520288. PubMed PMID: CN-01057082.

4183. Barbour SY, Brufsky A, Braccia D. Comparison of the granisetron transdermal system to oral granisetron for controlling chemotherapy-induced nausea and vomiting (CINV) in multiday chemotherapeutic regimens for breast cancer patients. Journal of oncology pharmacy practice. 2014;20(2 SUPPL. 1):9. doi: 10.1177/1078155213520288. PubMed PMID: CN-01057084.

4184. Barboza-Zanetti MO, Barboza-Zanetti AC, Rodrigues-Abjaude SA, Pinto-Simoes B, Leira-Pereira LR. Clinical pharmacists' contributions to hematopoietic stem cell transplantation: A systematic review. Journal

of Oncology Pharmacy Practice. 2019;25(2):423-33. doi: 10.1177/1078155218782372. PubMed PMID: WOS:000456863400020.

4185. Barbuto AF, Burns MM. Clonidine Compounding Error: Bradycardia and Sedation in a Pediatric Patient. Journal of Emergency Medicine (0736-4679). 2020;59(1):53-5. doi: 10.1016/j.jemermed.2020.04.027. PubMed PMID: 145517860. Language: English. Entry Date: 20210228. Revision Date: 20211013. Publication Type: journal article.

4186. Barcelo Colomer E, Bartomeu JG, Serre Delcor N, Salgado Pineda M, Marti Dillet M, Hidalgo Ortiz M. Externally induced prescriptions, degree of agreement in primary care and… The possibility of change in primary care? Atencion Primaria. 2000;26(4):231-8. doi: 10.1016/S0212-6567(00)78652-5.

4187. Barchitta M, Quattrocchi A, Maugeri A, La Rosa MC, La Mastra C, Sessa L, et al. Antibiotic consumption and resistance during a 3-year period in Sicily, Southern Italy. International Journal of Environmental Research and Public Health. 2019;16(13). doi: 10.3390/ijerph16132253.

4188. Bardage C, Ekedahl A, Ring L. Health care professionals' perspectives on automated multi-dose drug dispensing. Pharmacy Practice (1886-3655). 2014;12(4):1-8. PubMed PMID: 103926611. Language: English. Entry Date: 20141218. Revision Date: 20180305. Publication Type: Journal Article.

4189. Bardage C, Westerlund T, Barzi S, Bernsten C. Non-prescription medicines for pain and fever-A comparison of recommendations and counseling from staff in pharmacy and general sales stores. Health Policy. 2013;110(1):76-83. doi: 10.1016/j.healthpol.2012.12.006. PubMed PMID: WOS:000317372200010.

4190. Bardet C, Buxeraud J, Cook-Moreau J. Dispensing a monoclonal antibody at the community pharmacy. Actualites Pharmaceutiques. 2013;52(530):33-4. doi: 10.1016/j.actpha.2013.09.007.

4191. Bardet JD, Joncourt J, Bellet B, Allenet B. Implementation of medication review in community pharmacy: What is the feasibility? Pilot study in 41 sites of French Alps. Pharmacien Hospitalier et Clinicien. 2021;56(1):28-35. doi: 10.1016/j.phclin.2020.08.006.

4192. Bardet JD, Vo TH, Bedouch P, Allenet B. Physicians and community pharmacists collaboration in primary care: A review of specific models. Research in Social & Administrative Pharmacy. 2015;11(5):602-22. doi: 10.1016/j.sapharm.2014.12.003. PubMed PMID: WOS:000360049000002.

4193. Bardin C, Astier A, Vulto A, Sewell G, Vigneron J, Trittler R, et al. Guidelines for the practical stability studies of anticancer drugs: a European consensus conference. European Journal of Hospital Pharmacy. 2012;19(3):278-85. doi: 10.1136/ejhpharm-2012-000112. PubMed PMID: WOS:000309584800004.

4194. Bardosh K, Waiswa C, Welburn SC. Conflict of interest: use of pyrethroids and amidines against tsetse and ticks in zoonotic sleeping sickness endemic areas of Uganda. Parasites & Vectors. 2013;6. doi: 10.1186/1756-3305-6-204. PubMed PMID: WOS:000321783400001.

4195. Bare A, Ivey M, Kibuule D, Stevenson JG. An analysis of hospital pharmacy practice in Namibia, based on FIP's Basel Statements. International Journal of Pharmacy Practice. 2021;29(4):350-5. doi: 10.1093/ijpp/riab019.

4196. Barenfanger J, Short MA, Groesch AA. Improved antimicrobial interventions have benefits. Journal of clinical microbiology. 2001;39(8):2823-8. PubMed PMID: CN-00549261.

4197. Barenholtz Levy H. Self-administered medication-risk questionnaire in an elderly population. Annals of Pharmacotherapy. 2003;37(7-8):982-7. PubMed PMID: 12841804.

4198. Barenholz YC. Doxil®—The first FDA-approved nano-drug: Lessons learned. Journal of controlled release. 2012;160(2):117-34.

4199. Barenie RE, Leav K, Edward Johnson WT, Wheeler JS. Review and assessment of drug disposal state statutes in the United States, 2022. Journal of the American Pharmacists Association. 2022;62(6):1865-9. doi: https://doi.org/10.1016/j.japh.2022.06.001.

4200. Barennes H, Tran DS, Latthaphasavang V, Preux PM, Odermatt P. Epilepsy in Lao PDR: From research to treatment intervention. Neurology Asia. 2008;13:27-31.

4201. Barf T, Kaptein A. Irreversible protein kinase inhibitors: balancing the benefits and risks. Journal of medicinal chemistry. 2012;55(14):6243-62.

4202. Barger S, Sullivan SD, Bell-Brown A, Bott B, Ciccarella AM, Golenski J, et al. Effective stakeholder engagement: Design and implementation of a clinical trial (SWOG S1415CD) to improve cancer care. BMC Medical Research Methodology. 2019;19(1). doi: 10.1186/s12874-019-0764-2.

4203. Baril C, Gascon V, Brouillette C. Impact of technological innovation on a nursing home performance and on the medication-use process safety. Journal of medical systems. 2014;38:1-12.

4204. Barillas JE, Eichner D, Van Wagoner R, Speiser PW. Iatrogenic Cushing Syndrome in a Child With Congenital Adrenal Hyperplasia: Erroneous Compounding of Hydrocortisone. Journal of Clinical Endocrinology & Metabolism. 2017:N.PAG-N.PAG. doi: 10.1210/jc.2017-01595. PubMed PMID: 125771676. Language: English. Entry Date: 20180724. Revision Date: 20180724. Publication Type: journal article.

4205. Barin-Le Guellec C, Lafay-Chebassier C, Ingrand I, Tournamille JF, Boudet A, Lanoue MC, et al. Toxicities associated with chemotherapy regimens containing a fluoropyrimidine: a real-life evaluation in France. Fundamental & clinical pharmacology. 2021;35(SUPPL 1):76-. doi: 10.1111/fcp.12671. PubMed PMID: CN-02284966.

4206. Barkan S, Breitbart R, Brenner-Zada G, Feldon M, Assa A, Toledano M, et al. A double-blind, randomised, placebo-controlled trial of oral midazolam plus oral ketamine for sedation of children during laceration repair. Emergency medicine journal. 2014;31(8):649-53. doi: 10.1136/emermed-2012-202189. PubMed PMID: CN-00998743.

4207. Barker A, Barlis P, Berlowitz D, Page K, Jackson B, Lim WK. Pharmacist directed home medication reviews in patients with chronic heart failure: a randomised clinical trial. International journal of cardiology. 2012;159(2):139-43. doi: 10.1016/j.ijcard.2011.02.034. PubMed PMID: CN-00972749.

4208. Barker AK, Brown K, Ahsan M, Sengupta S, Safdar N. Social determinants of antibiotic misuse: a qualitative study of community members in Haryana, India. BMC Public Health. 2017;17(1). doi: 10.1186/s12889-017-4261-4.

4209. Barker AK, Brown K, Ahsan M, Sengupta S, Safdar N. What drives inappropriate antibiotic dispensing? A mixed-methods study of pharmacy employee perspectives in Haryana, India. BMJ Open. 2017;7(3). doi: 10.1136/bmjopen-2016-013190.

4210. Barker DC, Henriksen L, Voelker DH, Ali A, Raskind IG, Schleicher NC, et al. Turning over a new leaf: Vape shop closings, openings and transitions in six US Metropolitan statistical areas. Preventive Medicine Reports. 2021;23. doi: 10.1016/j.pmedr.2021.101428. PubMed PMID: WOS:000684860600006.

4211. Barker JR, Boyle TC, Tay L, Bishop A, Morrison B, Murphy A, et al. Barriers to the use of patient

safety information sources by community pharmacies. Research in Social & Administrative Pharmacy. 2019;15(7):895-901. doi: 10.1016/j.sapharm.2019.02.015. PubMed PMID: 136980149. Language: English. Entry Date: In Process. Revision Date: 20200325. Publication Type: journal article.

4212. Barker KN, Flynn EA, Pepper GA, Bates DW, Mikeal RL. Medication errors observed in 36 health care facilities. Archives of Internal Medicine. 2002;162(16):1897-903. PubMed PMID: 12196090.

4213. Barker R, Abrahamsson B, Kruusmägi M. Application and Validation of an Advanced Gastrointestinal In Vitro Model for the Evaluation of Drug Product Performance in Pharmaceutical Development. Journal of Pharmaceutical Sciences. 2014;103(11):3704-12. doi: https://doi.org/10.1002/jps.24177.

4214. Barkondaj B, Mukhopadhyay K, Das S, Chatterjee C, Mukherjee S, Hazra A. Information adequacy of medicine package inserts in India: A critical evaluation. Perspectives in Clinical Research. 2021;12(2):87-92. doi: 10.4103/picr.PICR_177_18.

4215. Barkoudah E, Piazza G, Hecht TEH, Grant P, Deitelzweig S, Fang MC, et al. Extended Venous Thromboembolism Prophylaxis in Medically Ill Patients: an NATF Anticoagulation Action Initiative. American journal of medicine. 2020;133 Suppl 1:1-27. doi: 10.1016/j.amjmed.2019.12.001. PubMed PMID: CN-02182282.

4216. Barlam TF, Cosgrove SE, Abbo LM, MacDougall C, Schuetz AN, Septimus EJ, et al. Implementing an Antibiotic Stewardship Program: Guidelines by the Infectious Diseases Society of America and the Society for Healthcare Epidemiology of America. Clinical Infectious Diseases. 2016;62(10):E51-E77. doi: 10.1093/cid/ciw118. PubMed PMID: WOS:000378432100001.

4217. Barlas S. "Authorized" generics may pose unauthorized problems: Government worries about potential brand-name blocking technique. P and T. 2005;30(8):435.

4218. Barlas S. FDA considers a new paradigm: For over-the-counter medications: More power-but more burdens-for pharmacists and pharmacies. P and T. 2012;37(5):300-2+5.

4219. Barlow BT, Roberts RJ, Newman K, Harrison SK, Sin JH. Economic Evaluation of a Pharmacist-Led 5-Day Therapeutic Hold of IV Levothyroxine at an Academic Medical Center. Hospital Pharmacy. 2022;57(1):20-5. doi: 10.1177/0018578720970457. PubMed PMID: WOS:000626208100001.

4220. Barlow JF, Faris RJ, Wang W, Verbrugge RR, Garavaglia SB, Aubert RE. Impact of specialty pharmacy on treatment costs for rheumatoid arthritis. American Journal of Pharmacy Benefits. 2012;4(6):SP49-SP56.

4221. Barnabas RV, Brown E, Bershteyn A, Miller RS, Wener M, Celum C, et al. Efficacy of hydroxychloroquine for post-exposure prophylaxis to prevent severe acute respiratory syndrome coronavirus 2 (SARS-CoV-2) infection among adults exposed to coronavirus disease (COVID-19): a structured summary of a study protocol for a randomised controlled trial. Trials. 2020;21(1):475. doi: 10.1186/s13063-020-04446-4. PubMed PMID: CN-02139060.

4222. Barnard M. The potential for screening for interpersonal violence in community pharmacies: An exploratory study [Ph.D.]. United States -- Mississippi: The University of Mississippi; 2012.

4223. Barnard M, Bynum LA, Bouldin A. Preparing student pharmacists to care for patients exposed to intimate partner violence. Currents in Pharmacy Teaching and Learning. 2021;13(12):1619-26. doi: https://doi.org/10.1016/j.cptl.2021.09.038.

4224. Barnard M, West-Strum D, Holmes E, Yang Y, Fisher A. The Potential for Screening for Intimate

Partner Violence in Community Pharmacies: An Exploratory Study of Female Consumers' Perspectives. Journal of Interpersonal Violence. 2018;33(6):960-79. doi: 10.1177/0886260515614272.

4225. Barnard M, West-Strum D, Yang Y, Holmes E. Evaluation of a Tool to Measure Pharmacists' Readiness to Manage Intimate Partner Violence. Pharmacy. 2018;6(3). doi: 10.3390/pharmacy6030066. PubMed PMID: WOS:000448399800012.

4226. Barnard M, White A, Bouldin A. Preparing Pharmacists to Care for Patients Exposed to Intimate Partner Violence. Pharmacy. 2020;8(2). doi: 10.3390/pharmacy8020100. PubMed PMID: WOS:000552756500046.

4227. Barner JC, Bennett RW. Pharmaceutical care certificate program: assessment of pharmacists' implementation into practice. Journal of the American Pharmaceutical Association (Washington,DC : 1996). 1999;39(3):362-7. doi: 10.1016/S1086-5802(16)30452-1.

4228. Barnert A-K, Hofmann P, Liekweg A, Kunze A, Scheid C, Hermes-Moll K, et al. Akzeptanz eines onkologischen Addendums zur Anwendung des Bundeseinheitlichen Medikationsplans in der oralen Tumortherapie. Zeitschrift für Evidenz, Fortbildung und Qualität im Gesundheitswesen. 2022;175:29-42. doi: https://doi.org/10.1016/j.zefq.2022.07.007.

4229. Barnert AK, Hofmann P, Liekweg A, Kunze A, Scheid C, Hermes-Moll K, et al. [Acceptance of an oncologic addendum for the application of the German Standardized Medication Plan in oral anticancer drug therapy]. Zeitschrift fur Evidenz Fortbildung und Qualitat im Gesundheitswesen. 2022;175:29-42. PubMed PMID: 36372643.

4230. Barnes B, Hincapie AL, Luder H, Kirby J, Frede S, Heaton PC. Appointment-based models: A comparison of three model designs in a large chain community pharmacy setting. Journal of the American Pharmacists Association. 2018;58(2):156-62.e1. doi: https://doi.org/10.1016/j.japh.2018.01.005.

4231. Barnes B, Luder H, Pleiman M, Frede S. Appointment-based medication synchronization: a community pharmacy model investigating impact on medication use and pharmacy productivity. Journal of the american pharmacists association. 2015;55(2):e200. doi: 10.1331/JAPhA.2015.15515. PubMed PMID: CN-01108016.

4232. Barnes DE, Palmer RM, Kresevic DM, Fortinsky RH, Kowal J, Chren MM, et al. Acute care for elders units produced shorter hospital stays at lower cost while maintaining patients' functional status. Health affairs (Project Hope). 2012;31(6):1227-36. doi: 10.1377/hlthaff.2012.0142. PubMed PMID: CN-00872460.

4233. Barnes E, Zhao J, Giumenta A, Johnson M. The Effect of an Integrated Health System Specialty Pharmacy on HIV Antiretroviral Therapy Adherence, Viral Suppression, and CD4 Count in an Outpatient Infectious Disease Clinic. Journal of Managed Care & Specialty Pharmacy. 2020;26(2):95-+. doi: 10.18553/jmcp.2020.26.2.95. PubMed PMID: WOS:000512370600004.

4234. Barnes GD, Acosta J, Graves C, Puroll E, Kline-Rogers E, Gu XK, et al. Barriers to integrating direct oral anticoagulants into anticoagulation clinic care: A mixed-methods study. Research and Practice in Thrombosis and Haemostasis. 2019;3(1):79-84. doi: 10.1002/rth2.12157. PubMed PMID: WOS:000455653800012.

4235. Barnes GD, Mouland E. Peri-Procedural Management of Oral Anticoagulants in the DOAC Era. Progress in Cardiovascular Diseases. 2018;60(6):600-6. doi: https://doi.org/10.1016/j.pcad.2018.03.002.

4236. Barnes J. An examination of the role of the pharmacist in the safe, effective and appropriate use of complementary medicines [Ph.D.]. England: University of London, University College London (United

Kingdom); 2001.

4237. Barnes J. Quality, efficacy and safety of complementary medicines: fashions, facts and the future. Part I. Regulation and quality. British journal of clinical pharmacology. 2003;55(3):226-33.

4238. Barnes J. Pharmacovigilance of herbal medicines - A UK perspective. Drug Safety. 2003;26(12):829-51. doi: 10.2165/00002018-200326120-00001. PubMed PMID: WOS:000185694800001.

4239. Barnes J, Abbot NC. Professional practices and experiences with complementary medicines: A cross-sectional study involving community pharmacists in England. International Journal of Pharmacy Practice. 2007;15(3):167-75. doi: 10.1211/ijpp.15.3.0003.

4240. Barnes J, Barber N, Wheatley D, Williamson EM. A pilot randomised, open, uncontrolled, clinical study of two dosages of St John's wort (Hypericum perforatum) herb extract (LI-160) as an aid to motivational/behavioural support in smoking cessation. Planta medica. 2006;72(4):378-82. doi: 10.1055/s-2005-916219. PubMed PMID: CN-00563601.

4241. Barnes J, Butler R. Community pharmacists' views on the regulation of complementary medicines and complementary-medicines practitioners: a qualitative study in New Zealand. International Journal of Pharmacy Practice. 2018;26(6):485-93. doi: 10.1111/ijpp.12428.

4242. Barnes J, Butler R. Community Pharmacists' Views and Experiences with ADR Reporting for Complementary Medicines: A Qualitative Study in New Zealand. Drug Safety. 2020;43(11):1157-70. doi: 10.1007/s40264-020-00980-x.

4243. Barnes J, Butler R. Community pharmacists' professional practices for complementary medicines: a qualitative study in New Zealand. International Journal of Clinical Pharmacy. 2020;42(4):1109-17. doi: 10.1007/s11096-020-01093-2.

4244. Barnes J, Sheridan JL, Dong CY, Härmark L, Vohra S, Harrison-Woolrych M. Evaluation of a Web-Based, 'Purchase Event' Intensive Monitoring Method for Pharmacovigilance of Natural Health Products: Lessons and Insights from Development Work in New Zealand. Drug Safety. 2020;43(10):981-98. doi: 10.1007/s40264-020-00963-y.

4245. Barnes JM, Riedlinger JE, McCloskey WW, Montagne M. Barriers to compliance with OBRA'90 regulations in community pharmacies. Annals of Pharmacotherapy. 1996;30(10):1101-5. doi: 10.1177/106002809603001006. PubMed PMID: WOS:A1996VL13300006.

4246. Barnes K. Do no harm: Prescription drug abuse and the paraprofessionalism of pharmacists [M.S.]. United States -- Wisconsin: The University of Wisconsin - Milwaukee; 2013.

4247. Barnes K. Essays on Industrial Organization2023.

4248. Barnes LAJ, Rolfe MI, Barclay L, McCaffery K, Aslani P. Women's reasons for taking complementary medicine products in pregnancy and lactation: Results from a national Australian survey. Complementary Therapies in Clinical Practice. 2022;49:101673. doi: https://doi.org/10.1016/j.ctcp.2022.101673.

4249. Barnes TL. Identifying Factors that Contribute to the Increase of Emergency Room Utilization in Rural Communities of East Texas [D.P.H.]. United States -- Minnesota: Capella University; 2022.

4250. Barnett A, Savic M, Lintzeris N, Bathish R, Arunogiri S, Dunlop AJ, et al. Tracing the affordances of long-acting injectable depot buprenorphine: A qualitative study of patients' experiences in Australia. Drug and Alcohol Dependence. 2021;227:108959. doi: https://doi.org/10.1016/j.drugalcdep.2021.108959.

4251. Barnett A, Subramanian G, Arnoldini S, Hunt B, Stentoft Hoxer C. Evaluation of the long-term cost-effectiveness of treatment switching from sitagliptin to liraglutide in subjects with Type 2 diabetes in the United Kingdom. Diabetic medicine. 2018;35:147-. doi: 10.1111/dme.39_13571. PubMed PMID: CN-01572279.

4252. Barnett BS, Morris NP. Pharmacy Access to Sterile Injection Supplies for People Who Inject Drugs. JAMA Internal Medicine. 2021;181(2):153-4. doi: 10.1001/jamainternmed.2020.6840.

4253. Barnett CW. Need for community pharmacist-provided food-allergy education and auto-injectable epinephrine training. Journal of the American Pharmacists Association. 2005;45(4):479-85. doi: 10.1331/1544345054475432.

4254. Barnett CW, Nykamp D, Ellington AM. Patient-guided counseling in the community pharmacy setting. Journal of the american pharmaceutical association (washington, DC : 1996). 2000;40(6):765-72. doi: 10.1016/s1086-5802(16)31123-8. PubMed PMID: CN-00372714.

4255. Barnett JR, Kearns RA. Shopping around? Consumerism and the use of private accident and medical clinics in Auckland, New Zealand. Environment and Planning A. 1996;28(6):1053-75. doi: 10.1068/a281053. PubMed PMID: WOS:A1996UW50800007.

4256. Barnett MJ, Doroudgar S, Khosraviani V, Ip EJ. Multiple comparisons: To compare or not to compare, that is the question. Research in Social and Administrative Pharmacy. 2022;18(2):2331-4. doi: https://doi.org/10.1016/j.sapharm.2021.07.006.

4257. Barnett MJ, Frank J, Wehring H, Newland B, VonMuenster S, Kumbera P, et al. Analysis of pharmacist-provided medication therapy management (MTM) services in community pharmacies over 7 years. Journal of Managed Care Pharmacy. 2009;15(1):18-31. PubMed PMID: 19125547.

4258. Barnett MJ, Perry PJ, Langstaff JD, Kaboli PJ. Comparison of rates of potentially inappropriate medication use according to the Zhan criteria for VA versus private sector medicare HMOs. Journal of Managed Care Pharmacy. 2006;12(5):362-70. doi: 10.18553/jmcp.2006.12.5.362.

4259. Barnett N, Minshull J, Algebory M, Dave K, Jhingan M, Kalsi D, et al. The Scottish Polypharmacy Guidance app in practice. Prescriber. 2020;31(6):10-3. doi: 10.1002/psb.1847. PubMed PMID: 144259444. Language: English. Entry Date: 20200703. Revision Date: 20210601. Publication Type: Article.

4260. Barnett NL. Opportunities for collaboration between pharmacists and clinical pharmacologists to support medicines optimisation in the UK. British Journal of Clinical Pharmacology. 2019;85(8):1666-9. PubMed PMID: 30986325.

4261. Barnett NL. Guide to undertaking person-centred inpatient (ward) outpatient (clinic) and dispensary-based pharmacy consultations. European Journal of Hospital Pharmacy. 2020;27(5):302-5. doi: 10.1136/ejhpharm-2018-001708.

4262. Barnett NL, Dave K, Athwal D, Parmar P, Kaher S, Ward C. Impact of an integrated medicines management service on preventable medicines-related readmission to hospital: a descriptive study. European Journal of Hospital Pharmacy Science & Practice. 2017;24(6):327-31. PubMed PMID: 31156966.

4263. Barnett NL, Oboh L. When less is more: the challenge of polypharmacy. European Journal of Hospital Pharmacy: Science and Practice. 2014;21(1):63-4.

4264. Barnett SG, Maes ML, Portillo EC, Shah SR. Instrument development to determine student self-efficacy to present a patient to a pharmacy preceptor (4P). Currents in Pharmacy Teaching and Learning.

2023;15(5):455-60. doi: https://doi.org/10.1016/j.cptl.2023.04.019.

4265. Barnett SJ, Frischer JS, Gaskey JA, Ryckman FC, von Allmen D. Pediatric hernia repair: 1-stop shopping. Journal of Pediatric Surgery. 2012;47(1):213-6. doi: 10.1016/j.jpedsurg.2011.10.047. PubMed PMID: WOS:000299351400043.

4266. Barnett T, Hoang H, Stuart J, Crocombe L, Bell E. Utilisation of oral health services provided by non-dental health practitioners in developed countries: a review of the literature. Community Dental Health. 2014;31(4):224-33. doi: 10.1922/CDH_3465Hoang10. PubMed PMID: WOS:000348592900007.

4267. Barney CK, Lambert DK, Alder SC, Scoffield SH, Schmutz N, Christensen RD. Treating feeding intolerance with an enteral solution patterned after human amniotic fluid: a randomized, controlled, masked trial. Journal of perinatology. 2007;27(1):28-31. doi: 10.1038/sj.jp.7211609. PubMed PMID: CN-00577380.

4268. Barney P, Sevin CM, Stollings J, Bose S, Groat D. ASSESSMENT OF POTENTIALLY INAPPROPRIATE DISCHARGE MEDICATIONS IN SURVIVORS OF ACUTE RESPIRATORY FAILURE. Chest. 2023;164(4 Supplement):A1730. doi: https://dx.doi.org/10.1016/j.chest.2023.07.1190. PubMed PMID: 2027228721.

4269. Barnoya J, Jin L, Hudmon KS, Schootman M. Nicotine replacement therapy, tobacco products, and electronic cigarettes in pharmacies in St. Louis, Missouri. Journal of the American Pharmacists Association. 2015;55(4):405-12. doi: 10.1331/JAPhA.2015.14230.

4270. Barnsteiner JH. Medication reconciliation. Patient safety and quality: an evidence-based handbook for nurses. 2008.

4271. Barnsteiner JH, Disch JM, Hall L, Mayer D, Moore SM. Promoting interprofessional education. Nursing outlook. 2007;55(3):144-50.

4272. Baron J, Lombardi CL, Yu H, Przespolewski A, Griffiths EA, Thompson JE, et al. Benefits of a Pharmacist Led Oral Chemotherapy Monitoring Program for Patients with Chronic Myeloid Malignancies: A Patient Reported Outcome (PRO) Study. Blood. 2019;134:3501. doi: https://doi.org/10.1182/blood-2019-131629.

4273. Baron K. COPD intervention investigation: comparing the effect of an outpatient counseling session after discharge to an educational counseling session on admission day 2 on hospitalization rates in patients with COPD. Journal of pharmacy practice Conference: 50th annual assembly of the new york state council of health-system pharmacists, NYSCHP 2011 verona, NY united states Conference start: 20110429 conference end: 20110501 Conference publication: (varpagings). 2011;24(3):354. doi: 10.1177/0897190011411560. PubMed PMID: CN-00834537.

4274. Barone J, Chapet N, Moulis L, Maire A, Villiet M, Jalabert A, et al. Evaluation of factors influencing immunosuppressant adherence among heart transplant patients: FIAHT study. Archives of Cardiovascular Diseases Supplements. 2023;15(1):49. doi: https://doi.org/10.1016/j.acvdsp.2022.10.088.

4275. Barone J, Chapet N, Moulis L, Maire A, Villiet M, Jalabert A, et al. Evaluation of factors influencing immunosuppressant adherence among heart transplant patients: FIAHT study. Archives of Cardiovascular Diseases Supplements. 2023;15(1):49. doi: https://doi.org/10.1016/j.acvdsp.2022.10.088.

4276. Baroy J, Chung D, Frisch R, Apgar D, Slack MK. The impact of pharmacist immunization programs on adult immunization rates: A systematic review and meta-analysis. Journal of the American Pharmacists Association. 2016;56(4):418-26. doi: https://doi.org/10.1016/j.japh.2016.03.006.

4277. Barr CE, Schulman K, Iacuzio D, Bradley JS. Effect of oseltamivir on the risk of pneumonia and use of health care services in children with clinically diagnosed influenza. Current Medical Research and Opinion. 2007;23(3):523-31. doi: 10.1185/030079906x167499. PubMed PMID: WOS:000245011400007.

4278. Barr H, Ross F. Student voices. Journal of Interprofessional Care. 2006;20(3):221-2. doi: 10.1080/13561820600780329.

4279. Barr PJ, McElnay JC, Hughes CM. Connected health care: the future of health care and the role of the pharmacist. Journal of Evaluation in Clinical Practice. 2012;18(1):56-62. doi: 10.1111/j.1365-2753.2010.01522.x. PubMed PMID: WOS:000298844600010.

4280. Barra ME, Culbreth SE, Sylvester KW, Rocchio MA. Utilization of an Integrated Electronic Health Record in the Emergency Department to Increase Prospective Medication Order Review by Pharmacists. Journal of Pharmacy Practice. 2018;31(6):636-41. PubMed PMID: 29017423.

4281. Barra ME, Webb AJ, Roberts RJ, Ross M, Hallisey R, Szumita P, et al. Implementation of a myasthenia gravis drug-disease interaction clinical decision support tool reduces prescribing of high-risk medications. Muscle and Nerve. 2023;67(4):284-90. doi: https://dx.doi.org/10.1002/mus.27790. PubMed PMID: 2021408680.

4282. Barra ME, Zink EK, Bleck TP, Cáceres E, Farrokh S, Foreman B, et al. Common Data Elements for Disorders of Consciousness: Recommendations from the Working Group on Hospital Course, Confounders, and Medications. Neurocritical Care. 2023;39(3):586-92. doi: 10.1007/s12028-023-01803-4.

4283. Barrachina J, Margarit C, Andreu B, Zandonai T, Ballester P, Muriel J, et al. Therapeutic alliance impact on analgesic outcomes in a real-world clinical setting: An observational study. Acta Pharmaceutica. 2022;72(4):529-45. doi: 10.2478/acph-2022-0035. PubMed PMID: WOS:000869334200004.

4284. Barraclough F, Pit S. Online multidisciplinary integrated rural healthcare education programs during the COVID-19 pandemic for students from different universities: experiences and guidelines. Health Education. 2022;122(2):202-16. doi: 10.1108/HE-06-2021-0096.

4285. Barradell LB, Goa KL. Alteplase: A Pharmacoeconomic Evaluation of its Use in the Management of Myocardial Infarction. PharmacoEconomics: PharmacoEconomics. 1995;8(5):428-59. doi: 10.2165/00019053-199508050-00006.

4286. Barratt MJ, Lentonb S, Maddox A, Allen M. 'What if you live on top of a bakery and you like cakes?' - Drug use and harm trajectories before, during and after the emergence of Silk Road. International Journal of Drug Policy. 2016;35:50-7. doi: 10.1016/j.drugpo.2016.04.006. PubMed PMID: WOS:000383302200008.

4287. Barraud D. Nosocomial infectious: The realities of an endless fight. Annales Pharmaceutiques Francaises. 2002;60(2):75-87.

4288. Barre E, Bisseux L, Chiadmi E, Toledano A, Cisternino S, Schlatter J, et al. Drug interactions in an elderly population. Presse Medicale. 2005;34(12):837-41. doi: 10.1016/s0755-4982(05)84059-x. PubMed PMID: WOS:000230595700003.

4289. Barré T, Vorspan F, Fortias M, Veyrier M, Cavagna P, Azuar J, et al. Pratique de l'ATU de cohorte de la naloxone intranasale (Nalscue®) : mise en place dans un CSAPA parisien. Therapies. 2018;73(6):495-500. doi: https://doi.org/10.1016/j.therap.2018.03.003.

4290. Barreirinhas S, Ramos D, Pinheiro R, Oliveira L, Rosa M, Paulino E. Cardiovascular risk factors and its association with overweight and obese patients followed in portuguese community pharmacies. Clinical

Nutrition. 2018;37:S267. doi: https://doi.org/10.1016/j.clnu.2018.06.1939.

4291. Barrense-Dias Y, Stadelmann S, Suris JC, Akre C. From request to dispensation: how adolescent and young adult females experience access to emergency contraception in pharmacies. European Journal of Contraception and Reproductive Health Care. 2022;27(5):403-8. doi: 10.1080/13625187.2022.2093849.

4292. Barrera-Anderson T, Olives T, Larsen K, Pereira A. Health literacy in native Spanish-speaking immigrants in Minneapolis. Travel Medicine and Infectious Disease. 2007;5(6):410. doi: https://doi.org/10.1016/j.tmaid.2007.09.032.

4293. Barret R. 1 - Medicines and Drugs. In: Barret R, editor. Therapeutical Chemistry: Elsevier; 2018. p. 1-20.

4294. Barreto GG, Simões MJS. Factors affecting the accession of private drugstores and pharmacies in Tatuí city (state of São Paulo) into the Notifying Pharmacy Scheme. Revista de Ciencias Farmaceuticas Basica e Aplicada. 2008;29(1):59-68.

4295. Barrett B, Brown R, Mundt M, Dye L, Alt J, Safdar N, et al. Using benefit harm tradeoffs to estimate sufficiently important difference: The case of the common cold. Medical Decision Making. 2005;25(1):47-55. doi: 10.1177/0272989X04273147.

4296. Barrett M, Keating A, Lynch D, Scanlon G, Kigathi M, Corcoran F, et al. Clozapine Patients at the Interface between Primary and Secondary Care. Pharmacy. 2018;6(1). doi: 10.3390/pharmacy6010019. PubMed PMID: WOS:000428562300018.

4297. Barrett R. Evaluation of community pharmacists' readiness to implement the Falsified Medicines Directive (Directive 2011/62/EC): An English cross-sectional survey with geospatial analysis. BMJ Open. 2020;10(1). doi: 10.1136/bmjopen-2019-033405.

4298. Barrett R. A cross-sectional study on substandard and falsified medicines (fake or counterfeit drugs) in UK pharmacies during the COVID-19 pandemic. Expert Opinion on Drug Safety. 2023;22(12):1289-99. doi: 10.1080/14740338.2023.2147922.

4299. Barrett R, Al-Mousawi HA. Development and initial validation of a postal survey evaluation of community pharmacists' opinion regarding falsified (counterfeit) medicines in Hampshire (UK). Journal of Pharmacy and Pharmacognosy Research. 2018;6(4):242-9.

4300. Barrett R, Aldamkhi H. An Evaluation of the Knowledge and Perceptions of Pharmacy Staff and Pre-Registration Students of E-Cigarettes Use: A Systematic Review. Tobacco Use Insights. 2021;14. doi: 10.1177/1179173x211016867. PubMed PMID: WOS:000662986600001.

4301. Barrett R, Costa D. An evaluation of community pharmacist perception of the misuse and abuse of over-the-counter co-codamol in cornwell and devon, UK: A cross-sectional survey. Heroin Addiction and Related Clinical Problems. 2018;20(5):13-7.

4302. Barrett R, Hodgkinson J. Quality evaluation of community pharmacy blood pressure (BP) screening services: An English cross-sectional survey with geospatial analysis. BMJ Open. 2019;9(12). doi: 10.1136/bmjopen-2019-032342.

4303. Barrickman A, Baugh G, Purnell K, Wiener RC, Funk A, Whetsel T, et al. Development and implementation of a virtual interprofessional escape room to reinforce tobacco cessation concepts. Journal of Interprofessional Education & Practice. 2022;27:100511. doi: https://doi.org/10.1016/j.xjep.2022.100511.

4304. Barrickman AL, Adelman M, Garofoli GK, Martello JL, Bardsley C, McCarthy L. Development of coupled patient care experience courses to enhance patient care skills in the ambulatory and acute care settings. Currents in Pharmacy Teaching and Learning. 2020;12(9):1137-44. doi: https://doi.org/10.1016/j.cptl.2020.04.023.

4305. Barrier M, McDaniel C, Lapreze J. Venous Thromboembolism Prophylaxis in Obstetrics. US Pharmacist. 2023;48(9):HS-11. PubMed PMID: 2025928915.

4306. Barris Blundell D. Outcomes in the management of the pharmacotherapy follow-up service in a community pharmacy. Pharmaceutical Care Espana. 2014;16(1):11-21.

4307. Barris Blundell D, Navarro Visa E, Sabio Sánchez B, Sánchez Gómez R, Cueto Jiménez A, Toril Lara C, et al. Treatment adherence of osteoporosis in patients of a community pharmacy. Pharmaceutical Care Espana. 2010;12(2):61-8.

4308. Barris Blundell D, Rodríguez Zarzuelo C, del Mar Salinas Muñoz M, Zarzuelo Pastor C. The demand for oral antibiotics without a medical prescription in a community pharmacy. Pharmaceutical Care Espana. 2001;3(6):412-20.

4309. Barris Blundell D, Rodríguez Zarzuelo C, Martínez-Rey Jiménez A, Sabio Sánchez B, Gutiérrez Álvarez JL, Garrido Jiménez B. Drug-related problems detected in patients who use antiasthmatic medications in a community pharmacy. Pharmaceutical Care Espana. 2004;6(4):202-8.

4310. Barris Blundell D, Rodríguez Zarzuelo C, Sabio Sánchez B, Garrido Jiménez B. Pharmacological interactions detected at a community pharmacy office. Pharmaceutical Care Espana. 2003;5(6):261-7.

4311. Barris Blundell D, Rodriguez Zarzuelo C, Sabio Sanchez B, Gutierrez Alvarez JL, Navarro Visa E, Munoz Valdes O, et al. Screening for osteoporosis among post-menopausal women in community pharmacy. Pharmacy Practice. 2006;4(2):95-101. PubMed PMID: 25247006.

4312. Barris Blunder D, Rodriguez Zarzuelo C, Sabio Sánchez B, Gutiérrez Alvarez JL, Navarro Visa E, Muñoz Valdés Ó, et al. Screening for osteoporosis among post-menopausal women in community pharmacy. Pharmacy Practice. 2006;4(2):95-101.

4313. Barritt ER. Professional Survival: Adjusting to Academia by Degrees. American Pharmacy. 1978;18(6):24-8. doi: https://doi.org/10.1016/S0160-3450(15)32509-5.

4314. Barroga TRM, Morales RG, Benigno CC, Castro SJM, Caniban MM, Cabullo MFB, et al. Antimicrobials Used in Backyard and Commercial Poultry and Swine Farms in the Philippines: A Qualitative Pilot Study. Frontiers in Veterinary Science. 2020;7. doi: 10.3389/fvets.2020.00329. PubMed PMID: WOS:000554025000001.

4315. Barron J, Xie Y, Wu SJ, White J, Singer J, Tulsi B, et al. Treatment of chronic hepatitis C infection with Sofosbuvir-based regimens in a commercially insured patient population. American health and drug benefits. 2016;9(6):327-35. PubMed PMID: CN-01208246.

4316. Barron WM, Reed RL, Forsythe S, Hecht D, Glen J, Murphy B, et al. Information technology. Implementing computerized provider order entry with an existing clinical information system. Joint Commission Journal on Quality & Patient Safety. 2006;32(9):506-16. PubMed PMID: 106235830. Language: English. Entry Date: 20070216. Revision Date: 20150711. Publication Type: Journal Article.

4317. Barron WM, Reed RL, Forsythe S, Hecht D, Glen J, Murphy B, et al. Implementing computerized provider order entry with an existing clinical information system. Joint commission journal on quality and

patient safety. 2006;2006 Sep; 32(9):506. PubMed PMID: CN-00634332.

4318. Barrons R, Pegram A, Borries A. Inhaler device selection: Special considerations in elderly patients with chronic obstructive pulmonary disease. American Journal of Health-System Pharmacy. 2011;68(13):1221-32. doi: 10.2146/ajhp100452. PubMed PMID: 104655164. Language: English. Entry Date: 20110715. Revision Date: 20200708. Publication Type: Journal Article.

4319. Barros AM. Controlo do Apetite: Fármacos Estimulantes e Fármacos Depressores [Master's]. Portugal: Universidade Fernando Pessoa (Portugal); 2011.

4320. Barros DSL, da Silva DLM, Leite SN. Access and use of medicines by elderly individuals with dementia. Brazilian Journal of Pharmaceutical Sciences. 2019;55. doi: 10.1590/s2175-97902019000117539. PubMed PMID: WOS:000488018600001.

4321. Barrow L, Richburg-Hayes L, Rouse CE, Brock T. Paying for performance: The education impacts of a community college scholarship program for low-income adults. Journal of Labor Economics. 2014;32(3):563-99.

4322. Barroyer P, Desport J-C. Prise en charge de la dénutrition du sujet âgé. Actualités Pharmaceutiques. 2021;60(611, Supplement):16-8. doi: https://doi.org/10.1016/j.actpha.2021.11.005.

4323. Barroyer P, Lemoine P. Dispensation de la mélatonine, un conseil indispensable. Actualités Pharmaceutiques. 2022;61(617, Supplement):24-5. doi: https://doi.org/10.1016/j.actpha.2022.03.024.

4324. Barry HE, Bedford LE, McGrattan M, Ryan C, Passmore AP, Robinson AL, et al. Improving medicines management for people with dementia in primary care: a qualitative study of healthcare professionals to develop a theory-informed intervention. BMC Health Services Research. 2020;20(1):120. PubMed PMID: 32059718.

4325. Barry HE, McGrattan M, Ryan C, Passmore AP, Robinson AL, Molloy GJ, et al. ‘I just take them because I know the people that give them to me’: A theory-informed interview study of community-dwelling people with dementia and carers' perspectives of medicines management. International Journal of Geriatric Psychiatry. 2021;36(6):883-91. doi: 10.1002/gps.5488.

4326. Barry HE, Parsons C, Passmore AP, Hughes CM. Community pharmacists and people with dementia: a cross-sectional survey exploring experiences, attitudes, and knowledge of pain and its management. International Journal of Geriatric Psychiatry. 2013;28(10):1077-85. doi: 10.1002/gps.3931. PubMed PMID: WOS:000323844800012.

4327. Barry JG, Colville JA, Donnelly RF. Attitudes of pharmacy students and community pharmacists to numeracy. Pharmacy Education. 2007;7(2):123-31. doi: 10.1080/15602210701264850.

4328. Barry MP, Austin EJ, Bhatraju EP, Glick SN, Stekler JD, Tung EL, et al. Qualitative inquiry into perceptions of HIV pre-exposure prophylaxis among people who inject drugs living with hepatitis C in Seattle, WA, USA. Harm Reduction Journal. 2022;19(1). doi: 10.1186/s12954-022-00706-5.

4329. Barseghyan A, Nazaryan L, Simonyan M. THE IMPACT OF AGE AND EDUCATION ON SELF-MEDICATION OF COMMON COLD AND COUGH-AN ARMENIAN EXPERIENCE. Farmacia. 2023;71(3):617-23. doi: 10.31925/farmacia.2023.3.21. PubMed PMID: WOS:001025500200021.

4330. Barseghyan AB, Nazaryan LG, Simonyan MH. EVALUATION OF PHARMACIST INTERVENTIONS AS A PART OF A MULTIDISCIPLINARY PAIN MANAGEMENT TEAM. New Armenian Medical Journal. 2022;16(3):100-6. doi: 10.56936/18290825-2022.16.3-100.

4331. Bartel SB. Safe practices and financial considerations in using oral chemotherapeutic agents. American Journal of Health-System Pharmacy. 2007;64:S8-40. doi: 10.2146/ajhp070036. PubMed PMID: 106164587. Language: English. Entry Date: 20071005. Revision Date: 20200708. Publication Type: Journal Article.

4332. Bartelme KM, Cieri-Hutcherson NE, Anderson L, Barnes KN, Karaoui LR, Meredith AH. Survey of colleges and schools of pharmacy to determine restrictions for teaching, research, and advocacy related to contraception. Currents in Pharmacy Teaching and Learning. 2023;15(6):551-8. doi: 10.1016/j.cptl.2023.06.002. PubMed PMID: WOS:001044155000001.

4333. Bartels SJ, Clark RE, Peacock WJ, Dums AR, Pratt SI. Medicare and Medicaid Costs for Schizophrenia Patients by Age Cohort Compared with Costs for Depression, Dementia, and Medically Ill Patients. American Journal of Geriatric Psychiatry. 2003;11(6):648-57. doi: 10.1097/00019442-200311000-00009.

4334. Bartels SJ, Horn S, Sharkey P, Levine K. Treatment of depression in older primary care patients in health maintenance organizations. International Journal of Psychiatry in Medicine. 1997;27(3):215-31. doi: 10.2190/vkbr-1ar6-9nbd-0n25. PubMed PMID: WOS:A1997YG57900004.

4335. Bartels-Peculis L, Sharma A, Edwards AM, Sanyal A, Connolly-Strong E, Nelson WW. Treatment Patterns and Health Care Costs of Lupus Nephritis in a United States Payer Population. Open Access Rheumatology-Research and Reviews. 2020;12:117-24. doi: 10.2147/oarrr.S248750. PubMed PMID: WOS:000542413300001.

4336. Bartfield M, Carlan SJ. The safety and efficacy of prolonged outpatient sulindac to prevent the recurrence of preterm labor: a prospective double-blind study. Primary care update for Ob/Gyns. 1998;5(4):178. PubMed PMID: CN-00601170.

4337. Barth AS. Is three a crowd or company?: Behind-the-counter drugs. Food & Drug Law Journal. 2008;63(4):865-89. PubMed PMID: 19601386.

4338. Barth KS, Ball S, Adams RS, Nikitin R, Wooten NR, Qureshi ZP, et al. Development and Feasibility of an Academic Detailing Intervention to Improve Prescription Drug Monitoring Program Use Among Physicians. Journal of Continuing Education in the Health Professions. 2017;37(2):98-105. doi: 10.1097/CEH.0000000000000149. PubMed PMID: 123642170. Language: English. Entry Date: 20170619. Revision Date: 20190712. Publication Type: Article.

4339. Bartholomew CR, Suzuki T, Du Z, Backues SK, Jin M, Lynch-Day MA, et al. Ume6 transcription factor is part of a signaling cascade that regulates autophagy. Proceedings of the National Academy of Sciences. 2012;109(28):11206-10.

4340. Bartholomew RR, Noble BN, Stanislaw JJ, Viehmann M, Herink MC, Furuno JP. Frequency and clinical outcomes of pharmacist-driven switching from warfarin to direct oral anticoagulants in an underserved patient population: A retrospective cohort study. American Journal of Health-System Pharmacy. 2023;80:S103-S10. doi: 10.1093/ajhp/zxac375. PubMed PMID: 170047705.

4341. Bartholomew TS, Andraka-Cristou B, Totaram RK, Harris S, Doblecki-Lewis S, Ostrer L, et al. "We want everything in a one-stop shop": acceptability and feasibility of PrEP and buprenorphine implementation with mobile syringe services for Black people who inject drugs. Harm Reduction Journal. 2022;19(1). doi: 10.1186/s12954-022-00721-6. PubMed PMID: WOS:000893933700003.

4342. Barthwell AG, Barnes MC, Leopold VR, Wichelecki JL. A vital balancing act: Multi-sector approaches to preventing prescription drug abuse in the united states while ensuring adequate patient access to medications: Part 1. Journal of Global Drug Policy and Practice. 2009;3(1).

4343. Bartlett D, Pang N, Massey C, Evans P. Pharmacist consultations: simplifying daily drug regimens and providing education on fall risk for older adults. Consultant Pharmacist. 2015;30(3):141-52. PubMed PMID: 25760664.

4344. Bartlett JD, Jaanus SD. Clinical ocular pharmacology: Elsevier Health Sciences; 2007.

4345. Bartlett JG, Gilbert DN, Spellberg B. Seven ways to preserve the miracle of antibiotics. Clinical Infectious Diseases. 2013;56(10):1445-50.

4346. Bartlett JL, Kinsey JD. Large-group, asynchronous, interprofessional simulation: Identifying roles and improving communication with student pharmacists and student nurses. Currents in Pharmacy Teaching and Learning. 2020;12(6):763-70. doi: https://doi.org/10.1016/j.cptl.2020.01.023.

4347. Bartlett JM, Siola PL. Implementation and first-year results of an antimicrobial stewardship program at a community hospital. American Journal of Health-System Pharmacy. 2014;71(11):943-9. doi: 10.2146/ajhp130602.

4348. Bartlett NL, Sehn LH, Matasar M, Schuster SJ, Assouline S, Giri P, et al. IBCL-458 Mosunetuzumab Monotherapy Demonstrates Durable Efficacy With a Manageable Safety Profile in Patients With Relapsed/Refractory Follicular Lymphoma who Received ≥2 Prior Therapies: Updated Results From a Pivotal Phase II Study. Clinical Lymphoma Myeloma and Leukemia. 2023;23:S453. doi: https://doi.org/10.1016/S2152-2650(23)01361-7.

4349. Bartlett RJ, Hertz D, Callahan P, Ruppar TM. Self-reported nonadherence associated with pharmacy and home medication management inconvenience factors in a US adult population. Patient Preference and Adherence. 2020;14:529-39. doi: 10.2147/PPA.S223408.

4350. Bartlett S, Bullock A, Broad B, Doyle L. Well-rounded pharmacists: a longitudinal evaluation of a multi-sector pre-registration programme. International Journal of Pharmacy Practice. 2022;30(3):268-72. doi: 10.1093/ijpp/riac023. PubMed PMID: WOS:000792389800001.

4351. Bartoli-Abdou JK, Weinman J, McRobbie D, Stebbins M, Brock T, Auyeung V. The transition from hospital to home following acute coronary syndrome: An exploratory qualitative study of patient perceptions and early experiences in two countries. International Journal of Pharmacy Practice. 2021;29(1):61-9. doi: 10.1093/ijpp/riaa009.

4352. Bartolini EZ, Hubbard T. Community pharmacies meet mobile technologies: A whole new world of opportunity. US Pharmacist. 2013;38(8):43-50.

4353. Bartolo NS, Grech V, Serracino-Inglott A, Azzopardi LM. Influenza and COVID-19 vaccine hesitancy in pharmacists and pharmacy students. Malta medical journal. 2022;34(1):58-68. PubMed PMID: CN-02492360.

4354. Barton AL, Moffett BS, Valdes SO, Miyake C, Kim JJ. Efficacy and Safety of High-Dose Propranolol for the Management of Infant Supraventricular Tachyarrhythmias. The Journal of Pediatrics. 2015;166(1):115-8. doi: https://doi.org/10.1016/j.jpeds.2014.08.067.

4355. Barton J. Pharmacist support for people with learning disabilities. Pharmaceutical Journal. 1999;263(7072):826-31.

4356. Barton L, Futtermenger J, Gaddi Y. Simple prescribing errors and allergy documentation in medical hospital admissions in Australia and New Zealand.

4357. Barton L, Futtermenger J, Gaddi Y, Kang A, Rivers J, Spriggs D, et al. Simple prescribing errors and allergy documentation in medical hospital admissions in Australia and New Zealand. Clinical Medicine. 2012;12(2):119-23. doi: 10.7861/clinmedicine.12-2-119. PubMed PMID: 78148804. Language: English. Entry Date: 20120904. Revision Date: 20190102. Publication Type: Article.

4358. Barton L, Futtermenger J, Gaddi Y, Kang A, Rivers J, Spriggs D, et al. Simple prescribing errors and allergy documentation in medical hospital admissions in Australia and New Zealand. Clinical Medicine. 2012;12(2):119.

4359. Bartsch SM, Taitel MS, DePasse JV, Cox SN, Smith-Ray RL, Wedlock P, et al. Epidemiologic and economic impact of pharmacies as vaccination locations during an influenza epidemic. Vaccine. 2018;36(46):7054-63. doi: https://doi.org/10.1016/j.vaccine.2018.09.040.

4360. Baruth JM, Gentry MT, Rummans TA, Miller DM, Burton MC. Polypharmacy in older adults: the role of the multidisciplinary team. Hospital practice (1995) Hospital practice. 2020;48(sup1):56-62. PubMed PMID: 31900000.

4361. Baruwal S, Hall RG, Pass SE, Safri A, Edwards KL. Efficacy and benefits of mock residency interviews. Currents in Pharmacy Teaching and Learning. 2021;13(8):958-63. doi: https://doi.org/10.1016/j.cptl.2021.06.048.

4362. Baryakova TH, Pogostin BH, Langer R, McHugh KJ. Overcoming barriers to patient adherence: the case for developing innovative drug delivery systems. Nature Reviews Drug Discovery. 2023;22(5):387-409.

4363. Basak R, Bentley JP, McCaffrey DJ, Bouldin AS, Banahan BF. The role of perceived impact on relationship quality in pharmacists' willingness to influence indication-based off-label prescribing decisions. Social science & medicine (1982). 2015;132:181-9. doi: 10.1016/j.socscimed.2015.03.028. PubMed PMID: CN-01127492.

4364. Basak S, Prasad G, Arunkumar A, Senthilkumar S. An Attempt To Develop Community Pharmacy Practice: Results Of Two Surveys And Two Workshops Conducted Tamilnadu. Indian journal of pharmaceutical sciences. 2005;67(3):362.

4365. Basak SC, Dondeti S. Evaluating medicines dispensing patterns at private community pharmacies in Tamilnadu, India. Southern Med Review. 2010;3(2):27-31.

4366. Basak SC, Prasad GS, Arunkumar A, Senthilkumar S. An attempt to develop community pharmacy practice: Results of two surveys and two workshops conducted in Tamilnadu. Indian Journal of Pharmaceutical Sciences. 2005;67(3):362-7.

4367. Basak SC, Sathyanarayana D. Evaluating medicines dispensing patterns at private community pharmacies in Tamilnadu, India. Southern Med Review. 2010;3(2):27-31.

4368. Basak SC, Sathyanarayana D. Exploring knowledge and perceptions of generic medicines among drug retailers and community pharmacists. Indian Journal of Pharmaceutical Sciences. 2012;74(6):571-5. doi: 10.4103/0250-474X.110611.

4369. Basaraba JE, Picard M, George-Phillips K, Mysak T. Pharmacists as Care Providers for Stroke Patients: A Systematic Review. Canadian Journal of Neurological Sciences. 2018;45(1):49-55. doi: 10.1017/cjn.2017.233.

4370. Basch CH, Roberts KJ, Zagnit EA, Martinez A. Marketing Strategies Used to Promote Children's Medicine Sold on Internet Sites of Pharmaceutical Stores. Journal of Community Health. 2016;41(6):1212-6.

doi: 10.1007/s10900-016-0205-7.

4371. Basciotta M, Zhou WX, Ngo L, Donnino M, Marcantonio ER, Herzig SJ. Antipsychotics and the Risk of Mortality or Cardiopulmonary Arrest in Hospitalized Adults. Journal of the American Geriatrics Society. 2020;68(3):544-50. doi: 10.1111/jgs.16246. PubMed PMID: WOS:000497149200001.

4372. Basco Jr WT, Garner SS, Ebeling M, Freeland KD, Hulsey TC, Simpson K. Evaluating the Potential Severity of Look-Alike, Sound-Alike Drug Substitution Errors in Children. Academic Pediatrics. 2016;16(2):183-91. doi: 10.1016/j.acap.2015.06.014. PubMed PMID: 113626535. Language: English. Entry Date: 20170920. Revision Date: 20190103. Publication Type: Article.

4373. Basco WT, Garner SS, Ebeling M, Freeland KD, Hulsey TC, Simpson K. Evaluating the Potential Severity of Look-Alike, Sound-Alike Drug Substitution Errors in Children. Academic Pediatrics. 2016;16(2):183-91. PubMed PMID: 609057162.

4374. Bascom CS, Rosenthal MM, Houle SKD. Are pharmacists ready for a greater role in travel health? An evaluation of the knowledge and confidence in providing travel health advice of pharmacists practicing in a community pharmacy chain in Alberta, Canada. Journal of Travel Medicine. 2015;22(2):99-104. doi: 10.1111/jtm.12172.

4375. Baser O, Burkan A, Baser E, Koselerli R, Ertugay E, Altinbas A. Direct medical costs associated with rheumatoid arthritis in Turkey: analysis from National Claims Database. Rheumatology International. 2013;33(10):2577-84. doi: 10.1007/s00296-013-2782-4. PubMed PMID: WOS:000324824500017.

4376. Basetty P, Buffie CG, Lagman J, Tigue CC, Dandade N, Bennett CL. Inadvertent Intrathecal Administration of Vincristine. Blood. 2006;108(11):3327. doi: https://doi.org/10.1182/blood.V108.11.3327.3327.

4377. Basey AJ. Exploring Key Risks in the Medical Admissions Process [Ph.D.]. England: Liverpool John Moores University (United Kingdom); 2014.

4378. Basger BJ, Moles RJ, Chen TF. Application of drug-related problem (DRP) classification systems: a review of the literature. European journal of clinical pharmacology. 2014;70:799-815.

4379. Basger BJ, Moles RJ, Chen TF. Impact of an enhanced pharmacy discharge service on prescribing appropriateness criteria: a randomised controlled trial. International journal of clinical pharmacy. 2015;37(6):1194-205. doi: 10.1007/s11096-015-0186-0. PubMed PMID: CN-01101180.

4380. Basger BJ, Moles RJ, Chen TF. Development of an aggregated system for classifying causes of drug-related problems. Annals of Pharmacotherapy. 2015;49(4):405-18.

4381. Basger BJ, Moles RJ, Chen TF. Uptake of pharmacist recommendations by patients after discharge: Implementation study of a patient-centered medicines review service. BMC geriatrics. 2023;23(1):183.

4382. Bashaar M, Thawani V, Hassali MA, Saleem F. Disposal practices of unused and expired pharmaceuticals among general public in Kabul. BMC public health. 2017;17:1-8.

4383. Bashatah AS, Syed W, Al-rawi MBA. PERCEPTIONS OF PEOPLES TOWARDS HEALTH CARE SEEKING BEHAVIOUR FROM HEALTH CARE PROFESSIONALS - FINDINGS FROM SAUDI COMMUNITY PERSPECTIVE. Farmacia. 2023;71(5):1095-102. doi: 10.31925/farmacia.2023.5.24. PubMed PMID: WOS:001098344200024.

4384. Basher MS, Haque MM, Ullah MS, Nasreen SA, Begum AA, Islam MN, et al. Lifestyle pattern in

selected slums in Mymensingh Municipal area. Mymensingh medical journal : MMJ. 2012;21(2):207-12.

4385. Basheti I. Inpatients with asthma: effect of education on inhaler technique and asthma control test. International journal of pharmacy practice Conference: 19th international social pharmacy workshop United kingdom Conference start: 20160719 Conference end: 20160722. 2016;24:5. doi: 10.1111/ijpp.12278/full. PubMed PMID: CN-01473551.

4386. Basheti I, Armour CL, Reddel HK, Bosnic Anticevich SZ. A pilot study of the effect of three educational interventions on Turbuhaler technique in community pharmacy. Respirology (Carlton, Vic). 2002;7(Suppl):A37; P91. PubMed PMID: CN-00790725.

4387. Basheti I, Armour CL, Reddel HK, Bosnic-Anticevich SZ. A pilot study of the effect of three educational interventions on Turbuhaler® technique in community pharmacy. Respirology (Carlton, Vic). 2002;7(Suppl):A37. PubMed PMID: CN-00430951.

4388. Basheti IA. The effect of using simulation for training pharmacy students on correct device technique. American journal of pharmaceutical education. 2014;78(10):177. doi: 10.5688/ajpe7810177. PubMed PMID: CN-01066956.

4389. Basheti IA, Abu-Gharbieh E. Revealing treatment-related problems for outpatients with alarming health status in the United Arab Emirates. Journal of Pharmaceutical Health Services Research. 2017;8(3):193-9. doi: 10.1111/jphs.12186.

4390. Basheti IA, Al-Qudah RA, Obeidat NM, Bulatova NR. Home medication management review in outpatients with chronic diseases in Jordan: a randomized control trial. International journal of clinical pharmacy. 2016;38(2):404-13. doi: 10.1007/s11096-016-0266-9. PubMed PMID: CN-01264187.

4391. Basheti IA, Armour CL, Bosnic-Anticevich SZ, Reddel HK. Evaluation of a novel educational strategy, including inhaler-based reminder labels, to improve asthma inhaler technique 1503. Patient education and counseling. 2008;72(1):26-33. PubMed PMID: CN-00651865.

4392. Basheti IA, Armour CL, Bosnic-Anticevich SZ, Reddel HK. Evaluation of a novel educational strategy, including inhaler-based reminder labels, to improve asthma inhaler technique. Patient Education and Counseling. 2008;72(1):26-33. doi: 10.1016/j.pec.2008.01.014.

4393. Basheti IA, Armour CL, Reddel HK, Bosnic-Anticevich SZ. Long-term maintenance of pharmacists' inhaler technique demonstration skills. American journal of pharmaceutical education. 2009;73(2):32. doi: 10.5688/aj730232. PubMed PMID: CN-00727802.

4394. Basheti IA, Ayasrah SM, Ahmad M. Identifying treatment related problems and associated factors among hospitalized post-stroke patients through medication management review: A multi-center study. Saudi Pharmaceutical Journal. 2019;27(2):208-19. doi: https://doi.org/10.1016/j.jsps.2018.10.005.

4395. Basheti IA, Bosnic-Anticevich SZ, Armour CL, Reddel HK. Checklists for powder inhaler technique: a review and recommendations. Respiratory care. 2014;59(7):1140-54.

4396. Basheti IA, Mhaidat NM, Al-Azzam S, Alqudah R, Nassar R, Alzubaidi H, et al. Knowledge and perceptions of pharmacists' readiness during coronavirus pandemic: the case of United Arab Emirates. Journal of Pharmaceutical Policy and Practice. 2021;14(1). doi: 10.1186/s40545-021-00382-z. PubMed PMID: WOS:000725513700001.

4397. Basheti IA, Mhaidat NM, Alqudah R, Nassar R, Othman B, Mukattash TL. Primary health care policy and vision for community pharmacy and pharmacists in Jordan. Pharmacy Practice. 2020;18(4). doi:

10.18549/PharmPract.2020.4.2184.

4398. Basheti IA, Nassar R, Barakat M, Alqudah R, Farha RA, Muqatash T, et al. Pharmacists' perceived barriers towards delivering their emergency roles during the COVID-19 pandemic and perceived policymakers' responsibilities. Journal of pharmaceutical policy and practice. 2020;13(1):1-11.

4399. Basheti IA, Obeidat NM, Reddel HK. Effect of novel inhaler technique reminder labels on the retention of inhaler technique skills in asthma: a single-blind randomized controlled trial. NPJ primary care respiratory medicine. 2017;27(1):9. doi: 10.1038/s41533-017-0011-4. PubMed PMID: CN-01402613.

4400. Basheti IA, Qunaibi E, Bosnic-Anticevich SZ, Armour CL, Khater S, Omar M, et al. User error with Diskus and Turbuhaler by asthma patients and pharmacists in Jordan and Australia. Respiratory Care. 2011;56(12):1916-23.

4401. Basheti IA, Qunaibi EA, AbuRuz S, Samara S, Bulatova NR. Home medication reviews in a patient care experience for undergraduate pharmacy students. American Journal of Pharmaceutical Education. 2013;77(8):173. PubMed PMID: 24159214.

4402. Basheti IA, Qunaibi EA, Bulatova NR, Samara S, Aburuz S. Treatment related problems for outpatients with chronic diseases in Jordan: the value of home medication reviews. International Journal of Clinical Pharmacy. 2013;35(1):92-100. doi: 10.1007/s11096-012-9713-4. PubMed PMID: 104239653. Language: English. Entry Date: 20130906. Revision Date: 20211029. Publication Type: journal article.

4403. Basheti IA, Qunaibi EA, Hamadi SA, Abu-Gharbieh E, Saleh S, AbuRuz S, et al. Patient perspectives of the role of the community pharmacist in the Middle East: Jordan, United Arab Emirates and Iraq. Pharmacology & Pharmacy. 2014;5(06):588.

4404. Basheti IA, Reddel HK, Armour CL, Bosnic-Anticevich SZ. Counseling about turbuhaler technique: needs assessment and effective strategies for community pharmacists. Respiratory care. 2005;50(5):617-23. PubMed PMID: CN-01706095.

4405. Basheti IA, Reddel HK, Armour CL, Bosnic-Anticevich SZ. Impact of inhaler technique: improved asthma outcomes with a simple intervention by community pharmacists. Respirology (Carlton, Vic). 2006;11(Suppl 2):Abstract no. 212. PubMed PMID: CN-00592183.

4406. Basheti IA, Reddel HK, Armour CL, Bosnic-Anticevich SZ. Improved asthma outcomes with a simple inhaler technique intervention by community pharmacists. Journal of allergy and clinical immunology. 2007;119(6):1537-8. doi: 10.1016/j.jaci.2007.02.037. PubMed PMID: CN-00587947.

4407. Basheti IA, Rizik M, Bulatova NR. Home medication management review in outpatients with alarming health issues in Jordan: a randomized control trial. Journal of pharmaceutical health services research. 2018;9(2):91-100. doi: 10.1111/jphs.12213. PubMed PMID: CN-01606809.

4408. Basheti IA, Tadros OK, Aburuz S. Value of a Community-Based Medication Management Review Service in Jordan: a Prospective Randomized Controlled Study. Pharmacotherapy. 2016;36(10):1075-86. doi: 10.1002/phar.1833. PubMed PMID: CN-01411182.

4409. Basheti IA, Tadros OK, Aburuz S. Value of a community-based medication management review service in Jordan: a prospective randomized controlled study. Pharmacotherapy: The Journal of Human Pharmacology and Drug Therapy. 2016;36(10):1075-86.

4410. Basheti IA, Tadros OKI, Aburuz S. Value of a Community-Based Medication Management Review Service in Jordan: A Prospective Randomized Controlled Study. Pharmacotherapy. 2016;36(10):1075-86.

doi: 10.1002/phar.1833.

4411. Basheti IA, Tadros OKI, Alnajjar MS, Aburuz S. Assessing patient satisfaction with the Medication Management Review service delivered in Jordan. Journal of pharmaceutical health services research. 2019;10(1):49-55. doi: 10.1111/jphs.12233. PubMed PMID: CN-01612009.

4412. Basheti MM, Gordon C, Bawa Z, Grunstein R, Saini B. Sleep health management in community pharmacy: Where are we and where should we be heading? Research in Social and Administrative Pharmacy. 2021;17(11):1945-56. doi: https://doi.org/10.1016/j.sapharm.2021.02.011.

4413. Bashir A, Galal S, Ramadan A, Wahdan A, El-Khordagui L. Community pharmacists' perceptions, awareness and practices regarding counterfeit medicines: A cross-sectional survey in Alexandria, Egypt. Eastern Mediterranean Health Journal. 2020;26(5):556-64. doi: 10.26719/emhj.19.058.

4414. Bashir A, Galal S, Ramadan A, Wandan A, El-Khordagui L. Community pharmacists' perceptions, awareness and practices regarding counterfeit medicines: a cross-sectional survey in Alexandria, Egypt. Eastern Mediterranean Health Journal. 2020;26(5):556-64. doi: 10.26719/emhj.19.058. PubMed PMID: WOS:000535995800012.

4415. Bashir Q, Thall PF, Sui D, Knape C, Kawedia J, Qazilbash MH. Trial in progress: a prospective phase i/ii trial to jointly optimizethe administration schedule(S) and dose(S) of melphalan forinjection (EVOMELA) as a preparative regimen for autologoushematopoietic stem cell transplantation in newly diagnosedmultiple myeloma. Blood. 2020;136(SUPPL 1):37-8. doi: 10.1182/blood-2020-139853. PubMed PMID: CN-02258929.

4416. Bashrahil KA, Bingouth AS, Baruzaig AS. Antimalarial drugs: Availability and mode of prescribing in Mukalla, Yemen. Eastern Mediterranean Health Journal. 2010;16(2):146-50. doi: 10.26719/2010.16.2.146.

4417. Basil JH, Premakumar CM, Ali AM, Mohd Tahir NA, Seman Z, Hon Voo JY, et al. Nurses' perception of medication administration errors and factors associated with their reporting in the neonatal intensive care unit. International Journal for Quality in Health Care. 2023;35(4):mzad101. doi: https://dx.doi.org/10.1093/intqhc/mzad101. PubMed PMID: 2029626414.

4418. Basil JH, Premakumar CM, Ali AM, Tahir NAM, Seman Z, Voo JYH, et al. Nurses' perception of medication administration errors and factors associated with their reporting in the neonatal intensive care unit. International Journal for Quality in Health Care. 2023;35(4):1-8. doi: 10.1093/intqhc/mzad101. PubMed PMID: 174684250.

4419. Basingab M, Balamesh U, Bafaqih R, Alamoudi O, Hesiki A, Alghefari A, editors. Agent-based modeling for internet of things (Iot) application in a retail pharmacy industry. Proceedings of the International Conference on Industrial Engineering and Operations Management; 2020.

4420. Baskin T. Student Forum Community Pharmacy Robberies: A Serious Problem. Journal of the American Pharmaceutical Association (1961). 1977;17(4):259. doi: https://doi.org/10.1016/S0003-0465(16)33982-9.

4421. Basran JFS, Dal Bello-Haas V, Walker D, MacLeod P, Allen B, D'Eon M, et al. The Longitudinal Elderly Person Shadowing Program: Outcomes From an Interprofessional Senior Partner Mentoring Program. Gerontology and Geriatrics Education. 2012;33(3):302-23. doi: 10.1080/02701960.2012.679369.

4422. Basri DS, DiScala SL, Brooks AT, Vartan CM, Silverman MA, Quellhorst J. Analysis of Inpatient Hospice Pharmacist Interventions Within a Veterans Affairs Medical Center. Journal of Pain & Palliative Care Pharmacotherapy. 2018;32(4):240-7. PubMed PMID: 31290723.

4423. Bass M, Gonzalez LJ, Colip L, Sharon N, Conklin J. Rethinking gender: The nonbinary approach. American Journal of Health-System Pharmacy. 2018;75(22):1821-3. PubMed PMID: 30228164.

4424. Bassett-Clarke D, Krass I, Bajorek B. Ethnic differences of medicines-taking in older adults: a cross cultural study in New Zealand. International Journal of Pharmacy Practice. 2012;20(2):90-8.

4425. Bassili A, Zaki A, Zaher SR, El-Sawy IH, Ahmed MH, Omar M, et al. Quality of care of children with chronic diseases in Alexandria, Egypt: The models of asthma, type I diabetes, epilepsy, and rheumatic heart disease. Pediatrics. 2000;106(1). doi: 10.1542/peds.106.1.e12. PubMed PMID: WOS:000087990400004.

4426. Basso Boccabella R, Dussaulx S, Germain Y, Hermitte-Gandolière A, Rondelot G. Interface entre deux logiciels de prescriptions : états des lieux des difficultés rencontrées et évaluation des risques. Le Pharmacien Hospitalier et Clinicien. 2018;53(2):97-108. doi: https://doi.org/10.1016/j.phclin.2017.12.006.

4427. Bassoum O, Sougou NM, Diongue M, Leye MMM, Mbodji M, Fall D, et al. Assessment of General Public's Knowledge and Opinions towards Antibiotic Use and Bacterial Resistance: A Cross-Sectional Study in an Urban Setting, Rufisque, Senegal. Pharmacy. 2018;6(4). doi: 10.3390/pharmacy6040103. PubMed PMID: WOS:000455628300001.

4428. Bassuk SS, Berkman LF, Wypij D. Depressive symptomatology and incident cognitive decline in an elderly community sample. Archives of General Psychiatry. 1998;55(12):1073-81.

4429. Bastam MM, Jalili M, Pakzad I, Maleki A, Ghafourian S. Pathogenic bacteria in cheese, raw and pasteurised milk. Veterinary Medicine and Science. 2021;7(6):2445-9. doi: 10.1002/vms3.604. PubMed PMID: WOS:000684858900001.

4430. Bastani P, Jooybar M, Zadeh MA, Samadbeik M. Community pharmacy-based survey on pharmacists' knowledge, attitude, and performance regarding dietary supplements: Evidence from south of Iran. National Journal of Physiology, Pharmacy and Pharmacology. 2017;7(4):396-402. doi: 10.5455/njppp.2017.7.1233518122016.

4431. Bastani P, Marzaleh MA, Dehghani M, Falahatzadeh M, Rahmati E, Tahernezhad A. The status of Iranian hospital pharmacies according to age-friendly pharmacies criteria. Journal of Advanced Pharmaceutical Technology and Research. 2017;8(4):120-4. doi: 10.4103/japtr.JAPTR_118_17.

4432. Bastani P, Yusefil A, Dehghanian P, Manesh MR, Zadeh NS, Khademian F. The pharmacists' knowledge, attitude, and practice toward the application of radio-frequency identification in Iranian pharmacies. International Journal of Pharmaceutical Investigation. 2018;8(1):44-9. doi: 10.4103/jphi.JPHI_86_17. PubMed PMID: WOS:000439220700007.

4433. Bastawrous A, Rono HK, Livingstone IA, Weiss HA, Jordan S, Kuper H, et al. Development and validation of a smartphone-based visual acuity test (peek acuity) for clinical practice and community-based fieldwork. JAMA ophthalmology. 2015;133(8):930-7.

4434. Bastian L, Hill A, Wilcock M. A cross-sectional study of hospital pharmacists communicating medication changes at discharge. International Journal of Pharmacy Practice. 2023;31(Supplement 2):ii8. PubMed PMID: 643188349.

4435. Bastos RM. Terapêutica Antifúngica de Onicomicoses: Potencial Bioativo de Três Extratos de Plantas: Experiência Profissionalizante na Vertente de Farmácia Comunitária, Hospitalar e Investigação [M.Sc.]. Portugal: Universidade da Beira Interior (Portugal); 2018.

4436. Basu A, Benson C, Alphs L. Projecting the potential effect of using paliperidone palmitate once-

monthly and once-every-3-months long-acting injections among medicaid beneficiaries with schizophrenia. Journal of managed care and specialty pharmacy. 2018;24(8):759-68a. doi: 10.18553/jmcp.2018.24.8.759. PubMed PMID: CN-01920910.

4437. Basu S, Andrews J, Kishore S, Panjabi R, Stuckler D. Comparative performance of private and public healthcare systems in low-and middle-income countries: a systematic review. PLoS medicine. 2012;9(6):e1001244.

4438. Basu S, Garg S, Sharma N, Meghachandra Singh M. Improving the assessment of medication adherence: Challenges and considerations with a focus on low-resource settings. Tzu Chi Medical Journal. 2019;31(2):73-80. PubMed PMID: 626961628.

4439. Basu S, Garg S, Sharma N, Singh MM. Improving the assessment of medication adherence: Challenges and considerations with a focus on low-resource settings. Tzu Chi Medical Journal. 2019;31(2):73-80. doi: 10.4103/tcmj.tcmj_177_18. PubMed PMID: WOS:000462704300002.

4440. Basu S, Modrek S, Bendavid E. Comparing Decisions for Malaria Testing and Presumptive Treatment: A Net Health Benefit Analysis. Medical Decision Making. 2014;34(8):996-1005. doi: 10.1177/0272989x14533609. PubMed PMID: WOS:000344064400008.

4441. Bataille J, Prot-Labarthe S, Bourdon O, Joret P, Brion F, Hartmann J-F. High-alert medications in a French paediatric university hospital. Journal of Evaluation in Clinical Practice. 2015;21(2):262-70. doi: 10.1111/jep.12302. PubMed PMID: 103778458. Language: English. Entry Date: 20150330. Revision Date: 20200708. Publication Type: Journal Article.

4442. Batarseh Y, Abukhalaf M, Fakhoury R, Alfalah L, Wazaify M. Mental health literacy and stigma towards patients with mental health disorders among pharmacists in the MENA region. Journal of Pharmaceutical Health Services Research. 2022;13(2):143-50. doi: 10.1093/jphsr/rmac017. PubMed PMID: WOS:000835766900001.

4443. Batarseh YS, Rouse MJ, AlEjielat R, Khaleel A, Aluwidi M, Ayyad D, et al. Developing advanced hospital pharmacy practice experiences in a Bachelor of Pharmacy programme in Jordan: A novel training programme and evaluation of student perceptions. Pharmacy Education. 2021;21(1):334-43. doi: 10.46542/pe.2021.211.334343. PubMed PMID: WOS:000684175000004.

4444. Batchelor S, Gemmell L, Kirk C, Mountford C, Thompson N. The effectiveness of buccal Vitamin D replacement in patients requiring home parenteral nutrition. Clinical Nutrition ESPEN. 2021;42:153-7. doi: https://doi.org/10.1016/j.clnesp.2021.01.047.

4445. Bate A, Lindquist M, Edwards IR, Olsson S, Orre R, Lansner A, et al. A Bayesian neural network method for adverse drug reaction signal generation. European journal of clinical pharmacology. 1998;54:315-21.

4446. Bate R, Coticelli P, Tren R, Attaran A. Antimalarial drug quality in the most severely malarious parts of Africa - A six country study. PLoS ONE. 2008;3(5). doi: 10.1371/journal.pone.0002132.

4447. Bate R, Jensen P, Hess K, Mooney L, Milligan J. Substandard and falsified anti-tuberculosis drugs: A preliminary field analysis. International Journal of Tuberculosis and Lung Disease. 2013;17(3):308-11. doi: 10.5588/ijtld.12.0355.

4448. Bate R, Jin GZ, Mathur A. Does price reveal poor-quality drugs? Evidence from 17 countries. Journal of Health Economics. 2011;30(6):1150-63. doi: 10.1016/j.jhealeco.2011.08.006.

4449. Bate R, Tren R, Mooney L, Hess K, Mitra B, Debroy B, et al. Pilot study of essential drug quality in two major cities in India. PLoS ONE [Electronic Resource]. 2009;4(6):e6003. PubMed PMID: 19547757.

4450. Batema MNP. Sierra Leone: An exploratory study of community pharmacy practice and medicines regulation [M.Sc.]. Canada -- Ontario, CA: University of Toronto (Canada); 2005.

4451. Bateman BT, Hernandez-Diaz S, Huybrechts KF, Palmsten K, Mogun H, Ecker JL, et al. Patterns of Outpatient Antihypertensive Medication Use During Pregnancy in a Medicaid Population. Hypertension. 2012;60(4):913-+. doi: 10.1161/hypertensionaha.112.197095. PubMed PMID: WOS:000308812000013.

4452. Bateman C. Drug laws - State to comply... But later. South African Medical Journal. 2004;94(8 I):605-8.

4453. Bateman ME, Hammer R, Byrne A, Ravindran N, Chiurco J, Lasky S, et al. Death Cafés for prevention of burnout in intensive care unit employees: study protocol for a randomized controlled trial (STOPTHEBURN). Trials. 2020;21(1):1019. doi: 10.1186/s13063-020-04929-4. PubMed PMID: CN-02208632.

4454. Bateman MT, McCarthy C, Simmons D, Xie MG, McGuigan K, Wagner ML. Evaluation of patient health questionnaire (PHQ-9) score and hemoglobin A1C (Hba1C) in patients with type 2 diabetes mellitus. Journal of pharmacy practice. 2016;29(3):305-. doi: 10.1177/0897190016645328. PubMed PMID: CN-01362011.

4455. Bateman MT, Nguyen R, Navathe AB, McCarthy C. Pharmacist-Led Review of Empagliflozin and Ertugliflozin Following Formulary Update. American Journal of Managed Care. 2023;29(8):424-8. doi: https://dx.doi.org/10.37765/ajmc.2023.89408. PubMed PMID: 2027153985.

4456. Bateman SD. Reduced skin integrity: Is what we have enough? British Journal of Health Care Management. 2013;19(8):374-82. doi: 10.12968/bjhc.2013.19.8.374.

4457. Bateni B, Shalansky S, Simpson S. Barriers to general practitioner participation in a clinical trial initiated by pharmacists. Canadian pharmacists journal. 2005;138(8):52-60. PubMed PMID: CN-01722203.

4458. Bates C, Laciak R, Southwick A, Bishoff J. Overprescription of postoperative narcotics: a look at postoperative pain medication delivery, consumption and disposal in urological practice. The Journal of urology. 2011;185(2):551-5.

4459. Bates DW. Using information technology to reduce rates of medication errors in hospitals. Bmj. 2000;320(7237):788-91.

4460. Bates DW. A 40-year-old woman who noticed a medication error. Jama. 2001;285(24):3134-40.

4461. Bates DW. Preventing medication errors: a summary. American Journal of Health-System Pharmacy. 2007;64(14_Supplement_9):S3-S9.

4462. Bates DW, Cohen M, Leape LL, Overhage JM, Shabot MM, Sheridan T. Reducing the frequency of errors in medicine using information technology. Journal of the American Medical Informatics Association. 2001;8(4):299-308.

4463. Bates DW, Evans RS, Murff H, Stetson PD, Pizziferri L, Hripcsak G. Detecting adverse events using information technology. Journal of the American Medical Informatics Association. 2003;10(2):115-28.

4464. Bates DW, Kuperman GJ, Wang S, Gandhi T, Kittler A, Volk L, et al. Ten commandments for effective

clinical decision support: making the practice of evidence-based medicine a reality. Journal of the American Medical Informatics Association. 2003;10(6):523-30.

4465. Bates DW, Leape LL, Cullen DJ, Laird N, Petersen LA, Teich JM, et al. Effect of computerized physician order entry and a team intervention on prevention of serious medication errors. JAMA. 1998;280(15):1311-6. PubMed PMID: 9794308.

4466. Bates DW, Leape LL, Cullen DJ, Laird N, Petersen LA, Teich JM, et al. Effect of computerized physician order entry and a team intervention on prevention of serious medication errors. JAMA: Journal of the American Medical Association. 1998;280(15):1311-6. doi: 10.1001/jama.280.15.1311. PubMed PMID: 107203814. Language: English. Entry Date: 19990801. Revision Date: 20200708. Publication Type: journal article.

4467. Bates DW, Teich JM, Lee J, Seger D, Kuperman GJ, Ma'Luf N, et al. The impact of computerized physician order entry on medication error prevention. Journal of the American Medical Informatics Association. 1999;6(4):313-21.

4468. Bates G, Cochrane M, Mackridge AJ. The extent that health professionals suspect and address addiction to medicines in primary care: Findings from a survey in Northwest England. Journal of Addictive Diseases. 2017;36(3):147-50. doi: 10.1080/10550887.2017.1299895.

4469. Bates JS, Buie LW, Amerine LB, Savage SW, Eckel SF, Patel R, et al. Expanding care through a layered learning practice model. American Journal of Health-System Pharmacy. 2016;73(22):1869-75. doi: 10.2146/ajhp150593. PubMed PMID: 119443323. Language: English. Entry Date: 20161130. Revision Date: 20181206. Publication Type: Article.

4470. Bates K, Beddy D, Whirisky C, Murphy M, O'Mahony J, Mealy K. Determining the frequency of prescription errors in an Irish hospital. Irish journal of medical science. 2010;179:183-6.

4471. Bates KA, Stafford RA, Teeter BS, Diemer T, Thomas JL, Curran GM. Pharmacist-provided services and community pharmacist integration into a patient-centered medical home: A qualitative study of primary care clinic staff perceptions. Journal of the American Pharmacists Association. 2019;59(4):S6-S11.e1. doi: 10.1016/j.japh.2019.03.019.

4472. Bath D, Goodman C, Yeung S. Modelling the cost-effectiveness of introducing subsidised malaria rapid diagnostic tests in the private retail sector in sub-Saharan Africa. Bmj Global Health. 2020;5(5). doi: 10.1136/bmjgh-2019-002138. PubMed PMID: WOS:000573885600008.

4473. Bati AH, Mandiracioglu A, Govsa F, Çam O. Health anxiety and cyberchondria among Ege University health science students. Nurse Education Today. 2018;71:169-73. doi: https://doi.org/10.1016/j.nedt.2018.09.029.

4474. Batista AD, Rodrigues DA, Figueiras A, Zapata-Cachafeiro M, Roque F, Herdeiro MT. Antibiotic dispensation without a prescription worldwide: A systematic review. Antibiotics. 2020;9(11):1-49. doi: 10.3390/antibiotics9110786.

4475. Batista EMM. Avaliação da Adesão à Terapêutica Farmacológica com Antineoplásicos Orais [Master's]. Portugal: Universidade da Beira Interior (Portugal); 2012.

4476. Batra P, Aquilino ML, Farris KB. Pharmacy staff perceptions and selfreported behaviors related to providing contraceptive information and counseling. Journal of the American Pharmacists Association. 2015;55(5):481-7. doi: 10.1331/JAPhA.2015.15037.

4477. Batra P, Aquilino ML, Farris KB. Pharmacy staff perceptions and self-reported behaviors related to providing contraceptive information and counseling. Journal of the American Pharmacists Association. 2015;55(5):481-7. doi: 10.1331/JAPhA.2015.15037. PubMed PMID: WOS:000365511000006.

4478. Batra R, Wolbach-Lowes J, Swindells S, Scarsi KK, Podany AT, Sayles H, et al. Impact of an electronic medical record on the incidence of antiretroviral prescription errors and HIV pharmacist reconciliation on error correction among hospitalized HIV-infected patients. Antiviral Therapy. 2015;20(5):555-9. PubMed PMID: 25560773.

4479. Batran RA, Sabri NA, Ali I, Fahmy SF. Cost-Effectiveness of the Pharmacist-Managed Warfarin Therapy vs. Standard Care for Patients With Mechanical Mitral Valve Prostheses: an Egyptian Healthcare Perspective. Frontiers in cardiovascular medicine. 2022;9. doi: 10.3389/fcvm.2022.889197. PubMed PMID: CN-02426180.

4480. Batson S, Herranz A, Rohrbach N, Canobbio M, Mitchell SA, Bonnabry P. Automation of in-hospital pharmacy dispensing: a systematic review. European Journal of Hospital Pharmacy. 2021;28(2):58-64. doi: 10.1136/ejhpharm-2019-002081. PubMed PMID: 148865733. Language: English. Entry Date: 20210225. Revision Date: 20210225. Publication Type: Article.

4481. Batsuli G, Zimowski KL, Carroll R, White MH, Woods GM, Meeks SL, et al. Successful Perioperative Management of Orthotopic Cardiac Transplantation in a Pediatric Patient With Concurrent Congenital von Willebrand Disease and Acquired von Willebrand Syndrome Using Recombinant von Willebrand Factor. Journal of Cardiothoracic and Vascular Anesthesia. 2022;36(3):724-7. doi: https://doi.org/10.1053/j.jvca.2021.01.038.

4482. Batta RA, Kasabri V, Akour A, Hyassat D, Albsoul-Younes A. Impact of clinical pharmacists intervention on management of hyperglycemia in pregnancy in Jordan. International journal of clinical pharmacy. 2017:1-8. doi: 10.1007/s11096-017-0550-3. PubMed PMID: CN-01429512.

4483. Batta RA, Kasabri V, Akour A, Hyassat D, Albsoul-Younes A. Impact of clinical pharmacists intervention on management of hyperglycemia in pregnancy in Jordan. International journal of clinical pharmacy. 2018;40(1):48-55. doi: 10.1007/s11096-017-0550-3. PubMed PMID: CN-01627681.

4484. Battaglia JN, Kieser MA, Bruskiewitz RH, Pitterle ME, Thorpe JM. An online virtual-patient program to teach pharmacists and pharmacy students how to provide diabetes-specific medication therapy management. American Journal of Pharmaceutical Education. 2012;76(7):131. PubMed PMID: 23049103.

4485. Batten M, Kosari S, Koerner J, Naunton M, Cargo M. Evaluation approaches, tools and aspects of implementation used in pharmacist interventions in residential aged care facilities: A scoping review. Research in Social and Administrative Pharmacy. 2022;18(10):3714-23. doi: https://doi.org/10.1016/j.sapharm.2022.05.006.

4486. Batterham PJ, Calear AL, Gulliver A, Farrer LM. Efficacy of a transdiagnostic, video-based online program for reducing depression, anxiety, and suicidal ideation in adults: protocol for a randomised controlled trial. Contemporary clinical trials communications. 2019;14. doi: 10.1016/j.conctc.2019.100341. PubMed PMID: CN-01915723.

4487. Batterham PJ, Gulliver A, Kurz E, Farrer LM, Vis C, Schuurmans J, et al. The Effect of Dissemination Pathways on Uptake and Relative Costs for a Transdiagnostic, Self-guided Internet Intervention for Reducing Depression, Anxiety, and Suicidal Ideation: Comparative Implementation Study. Journal of Medical Internet Research. 2022;24(5). doi: 10.2196/34769. PubMed PMID: WOS:000800610900001.

4488. Battis B, Clifford L, Huq M, Pejoro E, Mambourg S. The impacts of a pharmacist-managed outpatient

clinic and chemotherapy-directed electronic order sets for monitoring oral chemotherapy. Journal of Oncology Pharmacy Practice. 2017;23(8):582-90. doi: 10.1177/1078155216672314. PubMed PMID: 125887554. Language: English. Entry Date: 20171129. Revision Date: 20171129. Publication Type: Article.

4489. Battise D, Harris JB, Haltom W, Waghel R, Wilson J. Impact of removing group SOAP note writing from a problem-based learning course series on documentation performance during experiential rotations. Currents in Pharmacy Teaching and Learning. 2023;15(12):1046-51. doi: https://doi.org/10.1016/j.cptl.2023.10.003.

4490. Battista L, Winovitch KC, Rumney PJ, Davis E, Hagemann C, Wing DA. A case-control comparison of the effectiveness of betamethasone to prevent neonatal morbidity and mortality in preterm twin and singleton pregnancies. American Journal of Perinatology. 2008;25(7):449-53. doi: 10.1055/s-0028-1085070.

4491. Battleman DS, Callahan M, Thaler HT. Rapid antibiotic delivery and appropriate antibiotic selection reduce length of hospital stay of patients with community-acquired pneumonia: link between quality of care and resource utilization. Archives of Internal Medicine. 2002;162(6):682-8.

4492. Battu C, Buxeraud J. Pathologies courantes chez la femme enceinte et conseil officinal. Actualités Pharmaceutiques. 2019;58(591, Supplement):8-11. doi: https://doi.org/10.1016/j.actpha.2019.09.023.

4493. Bauder L, Giangobbe K, Asgary R. Barriers and Gaps in Effective Health Communication at Both Public Health and Healthcare Delivery Levels During Epidemics and Pandemics; Systematic Review. Disaster Medicine and Public Health Preparedness. 2023;17. doi: 10.1017/dmp.2023.61. PubMed PMID: WOS:000990409300001.

4494. Baudouin A, Guillemin M-D, Rioufol C, Ranchon F, Parat S. Pandémie à SARS-COV-2 : implication du pharmacien hospitalier dans la sécurisation de la prise en charge des patients. Annales Pharmaceutiques Françaises. 2023;81(5):900-8. doi: https://doi.org/10.1016/j.pharma.2023.04.005.

4495. Baudouin A, Guillemin MD, Rioufol C, Ranchon F, Parat S. [SARS-COV-2 pandemic: Involvement of the hospital pharmacist in securing patient care]. Annales Pharmaceutiques Francaises. 2023;81(5):900-8. PubMed PMID: 37086966.

4496. Baudouin A, Herledan C, Poletto N, Guillemin M-D, Maison O, Garreau R, et al. Economic impact of clinical pharmaceutical activities in hospital wards: A systematic review. Research in Social and Administrative Pharmacy. 2021;17(3):497-505. doi: https://doi.org/10.1016/j.sapharm.2020.07.016.

4497. Baudouin A, Herledan C, Poletto N, Guillemin MD, Maison O, Garreau R, et al. Economic impact of clinical pharmaceutical activities in hospital wards: A systematic review. Research in Social & Administrative Pharmacy. 2021;17(3):497-505. doi: 10.1016/j.sapharm.2020.07.016. PubMed PMID: WOS:000617989800002.

4498. Bauer AM, Parker MM, Schillinger D, Katon W, Adler N, Adams AS, et al. Associations between antidepressant adherence and shared decision-making, patient-provider trust, and communication among adults with diabetes: diabetes study of Northern California (DISTANCE). Journal of general internal medicine. 2014;29(8):1139-47. doi: 10.1007/s11606-014-2845-6. PubMed PMID: CN-01050816.

4499. Bauer CA, Yee J, Campbell RC. Chapter 48 - The Interdisciplinary Chronic Kidney Disease Clinic. In: Kimmel PL, Rosenberg ME, editors. Chronic Renal Disease. San Diego: Academic Press; 2015. p. 587-97.

4500. Bauer DT, Guerlain S. Improving the usability of intravenous medication labels to support safe medication delivery. International Journal of Industrial Ergonomics. 2011;41(4):394-9. doi: https://doi.org/10.1016/j.ergon.2011.02.005.

4501. Bauer GA, Szeinbach S, Griffith N, Siegel J. Perceptions of quality and value in state and local pharmacy professional organizations. American Journal of Health-System Pharmacy. 2002;59(11):1082-9. doi: 10.1093/ajhp/59.11.1082.

4502. Bauer JB, Chun DS, Karpinski TA. Pharmacist-led program to improve venous thromboembolism prophylaxis in a community hospital. American Journal of Health-System Pharmacy. 2008;65(17):1643-7. PubMed PMID: 18714111.

4503. Bauer KA, Johnson K, Stephenson JJ, Visaria J, Chung H, York W, et al. Rate of preventative vaccine use and vaccine beliefs among a commercially insured population. Vaccine. 2020;38(45):7087-93. doi: https://doi.org/10.1016/j.vaccine.2020.09.001.

4504. Bauer M, Monteith S, Geddes J, Gitlin MJ, Grof P, Whybrow PC, et al. Automation to optimise physician treatment of individual patients: examples in psychiatry. The Lancet Psychiatry. 2019;6(4):338-49.

4505. Bauer NS, Anand V, Carroll AE, Downs SM. Secondhand Smoke Exposure, Parental Depressive Symptoms and Preschool Behavioral Outcomes. Journal of Pediatric Nursing. 2015;30(1):227-35. doi: https://doi.org/10.1016/j.pedn.2014.06.004.

4506. Bauer SR, Kane-Gill SL. Outcome Assessment of Critical Care Pharmacist Services. Hospital Pharmacy. 2016;51(7):507-13. doi: 10.1310/hpj5107-507. PubMed PMID: 117342642. Language: English. Entry Date: 20170818. Revision Date: 20170818. Publication Type: Article.

4507. Bauermeister JA, Golinkoff JM, Horvath KJ, Hightow-Weidman LB, Sullivan PS, Stephenson R. A Multilevel Tailored Web App-Based Intervention for Linking Young Men Who Have Sex With Men to Quality Care (Get Connected): Protocol for a Randomized Controlled Trial. Jmir Research Protocols. 2018;7(8). doi: 10.2196/10444. PubMed PMID: WOS:000443597300020.

4508. Bauermeister JA, Golinkoff JM, Lin WY, Claude KF, Horvath KJ, Dowshen N, et al. Testing the Testers: Are Young Men Who Have Sex With Men Receiving Adequate HIV Testing and Counseling Services? Jaids-Journal of Acquired Immune Deficiency Syndromes. 2019;82:S133-S41. doi: 10.1097/qai.0000000000002173. PubMed PMID: WOS:000518230800012.

4509. Bauermeister JA, Tingler RC, Johnson S, Macagna N, Lucas J, Dominguez-Islas C, et al. Acceptability of a dapivirine gel administered rectally to HIV-1 seronegative adults (MTN-033 study). AIDS education and prevention. 2021;33(5):361-76. doi: 10.1521/AEAP.2021.33.5.361. PubMed PMID: CN-02348292.

4510. Baughman AW, Murphy N, Driscoll L, Moses C, Norstrom J, Balasubramaniam R, et al. Improving medication reconciliation in ava nursing home as part of marquis2. Journal of general internal medicine Conference: 40th annual meeting of the society of general internal medicine, SGIM 2017 United states. 2017;32(2 Supplement 1):S765. PubMed PMID: CN-01362574.

4511. Baughman AW, Murphy N, Driscoll L, Norstrom J, Nelson S, Roehm J, et al. Improving medication reconciliation in a veterans affairs skilled nursing facility as part of MARQUIS2. Journal of General Internal Medicine. 2018;33(2 Supplement 1):804-5. PubMed PMID: 622330258.

4512. Bauguil-Copéret A, Limat S, Clairet AL. Les rétrocessions, une zone à risque dans le parcours des patients. Annales Pharmaceutiques Françaises. 2020;78(6):534-43. doi: https://doi.org/10.1016/j.pharma.2020.07.009.

4513. Bauguil-Coperet A, Limat S, Clairet AL. [Retrocession, a risk area in care pathway]. Annales Pharmaceutiques Francaises. 2020;78(6):534-43. PubMed PMID: 32795457.

4514. Bauld L, Chesterman J, Ferguson J, Judge K. A comparison of the effectiveness of group-based and pharmacy-led smoking cessation treatment in Glasgow. Addiction. 2009;104(2):308-16. doi: 10.1111/j.1360-0443.2008.02446.x.

4515. Baum L, Badejo G, Chaboyer K, Leong C. Pharmacist-led interventions for the management of medication misuse and abuse: A systematic review. Journal of the American Pharmacists Association. 2017;57(3):297-8. doi: 10.1016/j.japh.2017.01.012.

4516. Bauman AN, Pedersen CA, Schommer JC, Griffith NL. Policies on documentation and disciplinary action in hospital pharmacies after a medication error. American Journal of Health-System Pharmacy. 2001;58(12):1120-5. PubMed PMID: 106532107. Language: English. Entry Date: 20051028. Revision Date: 20150711. Publication Type: Journal Article.

4517. Baumann AJ, Cleveland KO, Gelfand MS, Perkins III NB, Covington AD, Hobbs AL. Residual infusion performance evaluation (RIPE): a single-center evaluation of residual volume post-intravenous eravacycline infusion. Pharmacy. 2023;11(2):75.

4518. Baumann-Birkbeck L, Anoopkumar-Dukie S, Khan SA, Cheesman MJ, O'Donoghue M, Grant GD. Can a virtual microbiology simulation be as effective as the traditional Wetlab for pharmacy student education? BMC medical education. 2021;21(1):583. doi: 10.1186/s12909-021-03000-3. PubMed PMID: CN-02347230.

4519. Baumber N. Competing for an independent future. Pharmaceutical Journal. 2005;274(7346):486.

4520. Baumers M, Dominy J. Practical management for the digital age: An introduction for engineers, scientists, and other disciplines2022. 1-439 p.

4521. Baumevieille M. Benzodiazepines and related drugs: assessment of abuse or dependence potential from falsified prescription forms. Therapie. 1999;54(2):291.

4522. Baumevieille M, Haramburu F, Bégaud B. Abuse of prescription medicines in southwestern France. Annals of Pharmacotherapy. 1997;31(7-8):847-50. doi: 10.1177/106002809703100706.

4523. Baumevieille M, Rambaud A, Perri-Plande J, Daveluy A. Pharmaciens d'officine, étudiants en pharmacie et demandes de médicaments à base de codéine : étude observationnelle. Therapies. 2020;75(6):569-77. doi: https://doi.org/10.1016/j.therap.2019.12.006.

4524. Baumevieille M, Rambaud A, Perri-Plande J, Daveluy A. Community pharmacists, students in pharmacy, and requests of codeine-based medicines: Observational study. Therapie. 2020. doi: 10.1016/j.therap.2019.12.006.

4525. Baumgartner AD, Clark CM, LaValley SA, Monte SV, Wahler RG, Singh R. Interventions to deprescribe potentially inappropriate medications in the elderly: Lost in translation? Journal of Clinical Pharmacy & Therapeutics. 2020;45(3):453-61. doi: 10.1111/jcpt.13103. PubMed PMID: 143043046. Language: English. Entry Date: 20200507. Revision Date: 20210601. Publication Type: Article.

4526. Baumgartner PC, Vrijens B, Allemann S, Hersberger KE, Arnet I. Delta T, a Useful Indicator for Pharmacy Dispensing Data to Monitor Medication Adherence. Pharmaceutics. 2022;14(1). doi: 10.3390/pharmaceutics14010103.

4527. Baurès E, Baudet A, Blanchard O, Guegan H, Guillaso M, le Cann P, et al., editors. Indoor air quality in healthcare and care facilities: Chemical pollution and microbiological contamination. 16th Conference of the International Society of Indoor Air Quality and Climate: Creative and Smart Solutions for Better Built

Environments, Indoor Air 2020; 2020.

4528. Bautz A. CommunityCare: An Outpatient Autoimmune Clinic Fostering a Network of Patients and Caregivers through Medical, Educational, and Social Interaction [Master's]. United States -- Washington: University of Washington; 2012.

4529. Bawab N, Moullin J, Jotterand S, Rossier C, Schneider MP, Perraudin C. Building Interprofessional Collaborative Practices Through a Support Program for Patients with Type 2 Diabetes in Primary Care. Journal of Continuing Education in the Health Professions. 2023;43(2):77-86. doi: 10.1097/CEH.0000000000000466.

4530. Bawab N, Moullin JC, Bugnon O, Perraudin C. Implementation Evaluation of an Interprofessional Programme (Siscare) for Supporting Patients with Type 2 Diabetes in a primary care setting. Research in Social and Administrative Pharmacy. 2021;17(11):1968-77. doi: 10.1016/j.sapharm.2021.02.019.

4531. Bawab N, Moullin JC, Perraudin C, Bugnon O. Implementation and Effectiveness of an Interprofessional Support Program for Patients with Type 2 Diabetes in Swiss Primary Care: A Study Protocol. Pharmacy. 2020;8(2). doi: 10.3390/pharmacy8020106. PubMed PMID: WOS:000552756500033.

4532. Bawab N, Schneider MP, Ballabeni P, Locatelli I, Bugnon O, Perraudin C. Effectiveness of an Interprofessional Program (Siscare) for Supporting Patients With Type 2 Diabetes. Diabetes Spectrum. 2023;36(1):41-51. doi: 10.2337/ds21-0084.

4533. Bawazeer G, Sales I, Alsunaidi A, Aljahili S, Aljawadi MH, Almalag HM, et al. Student-Led discharge counseling program for High-Risk medications in a teaching hospital in Saudi Arabia: a pilot study. Saudi pharmaceutical journal. 2021;29(10):1129-36. doi: 10.1016/j.jsps.2021.08.004. PubMed PMID: CN-02305216.

4534. Bawazir SA. Consumer attitudes towards community pharmacy services in Saudi Arabia. International Journal of Pharmacy Practice. 2004;12(2):83-9. doi: 10.1211/0022357023718.

4535. Bawazir SA, Abou-Auda HS, Gubara OA, Al-Khamis KI, Al-Yamani MJ. Public attitude toward drug technical package inserts in Saudi Arabia. Journal of Pharmacy Technology. 2003;19(3):209-18.

4536. Bawazir SA, Al-Yamani MJ, Makeen HA, Al-Rayes SM. Physicians and pharmacists adherence to prescribing guidelines of controlled psychotropic drugs: A study in private sector, Riyadh, Saudi Arabia. Saudi Pharmaceutical Journal. 2005;13(2-3):111-9.

4537. Bax R, Gabbay F, Phillips I. Antibiotic clinical trials - The witley park symposium summary. Clinical microbiology and infection. 2000;6(6):324-. doi: 10.1046/j.1469-0691.2000.00085.x. PubMed PMID: CN-01759836.

4538. Bax R, Gabbay F, Phillips I. Antibiotic clinical trials—the Witley Park Symposium Summary. Clinical Microbiology and Infection. 2000;6(6):324. doi: https://doi.org/10.1046/j.1469-0691.2000.00085.x.

4539. Baxter K, Preston CL. Stockley's drug interactions: Pharmaceutical Press London; 2010.

4540. Baxter KL, Jones RH, Jordan KM, Petz CA. Using pharmacy consults to improve blood pressure control in a resident teaching clinic. Journal of Pharmacy Practice & Research. 2019;49(4):399-400. doi: 10.1002/jppr.1593. PubMed PMID: 138010700. Language: English. Entry Date: 20190814. Revision Date: 20200731. Publication Type: Article.

4541. Bayked EM, Taye GN, Zewdie S, Aklilu T, Kahissay MH, Toleha HN. Pharmacy professionals'

perceptions of their professional duties in the Ethiopian health care system: a mixed methods study. Journal of Pharmaceutical Policy and Practice. 2023;16(1). doi: 10.1186/s40545-023-00656-8. PubMed PMID: WOS:001107710000001.

4542. Bayón García C, Binks R, De Luca E, Dierkes C, Franci A, Gallart E, et al. Prevalence, management and clinical challenges associated with acute faecal incontinence in the ICU and critical care settings: The FIRST™ cross-sectional descriptive survey. Intensive and Critical Care Nursing. 2012;28(4):242-50. doi: https://doi.org/10.1016/j.iccn.2012.01.005.

4543. Bayoud T, Waheedi M, Lemay J, Awad A. Drug therapy problems identification by clinical pharmacists in a private hospital in Kuwait. Annales Pharmaceutiques Françaises. 2018;76(3):210-7. doi: https://doi.org/10.1016/j.pharma.2018.01.002.

4544. Bayoumi I, Howard M, Holbrook AM, Schabort I. Interventions to improve medication reconciliation in primary care. Annals of Pharmacotherapy. 2009;43(10):1667-75.

4545. Baypinar F, Kingma H, Hoeven R, Becker M. Physicians' Compliance with a Clinical Decision Support System Alerting during the Prescribing Process. Journal of Medical Systems. 2017;41(6):1-6. doi: 10.1007/s10916-017-0717-4. PubMed PMID: 123385701. Language: English. Entry Date: 20170606. Revision Date: 20180601. Publication Type: Article.

4546. Bayrak Ş. Using Animated Graphics in Health Communication and Animated Prospectus Application2023.

4547. Bayraktar Ekincioğlu A, Hudson S. Design and evaluation of monitoring programme for methotrexate users in the arthritic population - A study at the primary and the secondary care interface. Turkish Journal of Pharmaceutical Sciences. 2013;10(1):125-36.

4548. Bays H. Statin safety: an overview and assessment of the data—2005. The American journal of cardiology. 2006;97(8):S6-S26.

4549. Baysari MT, Hardie R-A, Lake R, Richardson L, McCullagh C, Gardo A, et al. Longitudinal study of user experiences of a CPOE system in a pediatric hospital. International journal of medical informatics. 2018;109:5-14.

4550. Baysari MT, Lehnbom EC, Li L, Hargreaves A, Day RO, Westbrook JI. The effectiveness of information technology to improve antimicrobial prescribing in hospitals: a systematic review and meta-analysis. International journal of medical informatics. 2016;92:15-34.

4551. Baysari MT, Westbrook JI, Richardson KL, Day RO. The influence of computerized decision support on prescribing during ward-rounds: are the decision-makers targeted? Journal of the American Medical Informatics Association. 2011;18(6):754-9.

4552. Baysari MT, Zheng WY, Li L, Westbrook J, Day RO, Hilmer S, et al. Optimising computerised decision support to transform medication safety and reduce prescriber burden: study protocol for a mixed-methods evaluation of drug-drug interaction alerts. BMJ open. 2019;9(8):e026034. doi: 10.1136/bmjopen-2018-026034. PubMed PMID: CN-02144976.

4553. Bazan N, Shawer A, Abdelaziz A, Farid M. Impact of clinical pharmacy services on Egyptian patients with chronic heart failure in the outpatient setting. European Heart Journal Supplements. 2020;22(A):A9-A. PubMed PMID: WOS:000526981800034.

4554. Bazargan M, Smith J, Movassaghi M, Martins D, Yazdanshenas H, Salehe Mortazavi S, et al.

Polypharmacy among Underserved Older African American Adults. Journal of Aging Research. 2017:1-8. doi: 10.1155/2017/6026358. PubMed PMID: 123181169. Language: English. Entry Date: 20180117. Revision Date: 20190222. Publication Type: Article.

4555. Bazargan M, Yazdanshenas H, Han S, Orum G. Inappropriate Medication Use Among Underserved Elderly African Americans. Journal of Aging & Health. 2016;28(1):118-38. doi: 10.1177/0898264315589571. PubMed PMID: 112723277. Language: English. Entry Date: 20170622. Revision Date: 20190308. Publication Type: Article.

4556. Bazzell B, Kelling S, Diez H, Klein K. Identifying Opportunities for Improvement in Safety and Efficacy of Community Pharmacy Immunization Programs. Journal of Pharmacy Practice. 2019;32(4):428-33. doi: 10.1177/0897190018761410.

4557. Bazzi AR, Bordeu M, Baumgartner K, Sproesser DM, Bositis CM, Krakower DS, et al. Study protocol for an efficacy trial of the “PrEP for Health” intervention to increase HIV PrEP use among people who inject drugs. BMC Public Health. 2023;23(1). doi: 10.1186/s12889-023-15429-w.

4558. Bazzi AR, Shaw LC, Biello KB, Vahey S, Brody JK. Patient and Provider Perspectives on a Novel, Low-Threshold HIV PrEP Program for People Who Inject Drugs Experiencing Homelessness. Journal of General Internal Medicine. doi: 10.1007/s11606-022-07672-5. PubMed PMID: WOS:000802312000003.

4559. Bazzi AR, Shaw LC, Biello KB, Vahey S, Brody JK. Patient and Provider Perspectives on a Novel, Low-Threshold HIV PrEP Program for People Who Inject Drugs Experiencing Homelessness. Journal of General Internal Medicine. 2022. doi: 10.1007/s11606-022-07672-5.

4560. Bazzi AR, Shaw LC, Biello KB, Vahey S, Brody JK. Patient and Provider Perspectives on a Novel, Low-Threshold HIV PrEP Program for People Who Inject Drugs Experiencing Homelessness. Journal of General Internal Medicine. 2023;38(4):913-21. doi: 10.1007/s11606-022-07672-5.

4561. BComp KS. A Simulated Learning Environment for Teaching Medicine Dispensing Skills. American Journal of Pharmaceutical Education. 2016;80(1):1.

4562. Beachy S, Baker AM, Quittner AL, Riekert KA. Treatment adherence barriers identified by CF providers. Pediatric pulmonology Conference: 30th annual north american cystic fibrosis conference United states Conference start: 20161027 Conference end: 20161029. 2016;51:452-3. doi: 10.1002/ppul.23576. PubMed PMID: CN-01212528.

4563. Beahm NP, Smyth DJ, Tsuyuki RT. Outcomes of Urinary Tract Infection Management by Pharmacists (RxOUTMAP): A study of pharmacist prescribing and care in patients with uncomplicated urinary tract infections in the community. Canadian Pharmacists Journal. 2018;151(5):305-14. doi: 10.1177/1715163518781175.

4564. Beahm NP, Smyth DJ, Tsuyuki RT. Antimicrobial utilization and stewardship in patients with uncomplicated urinary tract infections managed by pharmacists in the community: A sub-study of the rxoutmap trial. JAMMI. 2021;6(3):205-12. doi: 10.3138/JAMMI-2020-0047.

4565. Beaird SL. HMG-CoA Reductase Inhibitors: Assessing Differences in Drug Interactions and Safety Profiles. Journal of the American Pharmaceutical Association (1996). 2000;40(5):637-44. doi: https://doi.org/10.1016/S1086-5802(16)31104-4.

4566. Beak SG, Kim DJ. Effects of green tea intake and flexible exercise on oxidative stress and catecholamines. Research journal of pharmacy and technology. 2017;10(11):1994-7. PubMed PMID: CN-01919887.

4567. Beam TA, Klepser DG, Klepser ME, Bright DR, Klepser N, Schuring H, et al. COVID-19 host genetic risk study conducted at community pharmacies: Implications for public health, research and pharmacists' scope of practice. Research in Social and Administrative Pharmacy. 2023;19(10):1360-4. doi: https://doi.org/10.1016/j.sapharm.2023.06.003.

4568. Beaney AM. Preparation of parenteral medicines in clinical areas: how can the risks be managed - a UK perspective?

4569. Beaney AM. Preparation of parenteral medicines in clinical areas: how can the risks be managed -- a UK perspective? Journal of Clinical Nursing (John Wiley & Sons, Inc). 2010;19(11-12):1569-77. doi: 10.1111/j.1365-2702.2010.03195.x. PubMed PMID: 105204279. Language: English. Entry Date: 20100716. Revision Date: 20190412. Publication Type: Journal Article.

4570. Beaney AM. Preparation of parenteral medicines in clinical areas: how can the risks be managed – a UK perspective? Journal of Clinical Nursing (John Wiley & Sons, Inc). 2010;19(11-12):1569-77. doi: 10.1111/j.1365-2702.2010.03195.x.

4571. Beano H, Helms C, Wang L, Kennelly M. Clinical pharmacist-led overactive bladder supplemental management support: prospective pilot study. Neurourology and urodynamics. 2020;39:S208-S9. doi: 10.1002/nau.24307. PubMed PMID: CN-02139104.

4572. Bear KA, Higginson AI, Hickey PW. Disparities exist in the availability of outpatient malaria treatment in Maryland, USA. Journal of Travel Medicine. 2010;17(4):228-32. doi: 10.1111/j.1708-8305.2010.00404.x.

4573. Beard R. Is electronic prescribing the best system for preventing pharmacy dispensing errors.

4574. Beard R, Candlish C. Is electronic prescribing the best system for preventing pharmacy dispensing errors?

4575. Beard R, Candlish C. Is electronic prescribing the best system for preventing pharmacy dispensing errors? British Journal of Healthcare Computing & Information Management. 2007;24(1):15-8. PubMed PMID: 105971322. Language: English. Entry Date: 20080215. Revision Date: 20150820. Publication Type: Journal Article.

4576. Bearden DT, Allen GP. Impact of antimicrobial control programs on patient outcomes - Pharmacy perspective. Disease Management & Health Outcomes. 2003;11(11):723-36. doi: 10.2165/00115677-200311110-00004. PubMed PMID: WOS:000186484700004.

4577. Beardsley J, Chambers JM, Lam TT, Zawahir S, Le H, Nguyen TA, et al. Mapping access to drug outlets in Vietnam: distribution of drug outlets and the sociodemographic characteristics of the communities they serve. The Lancet Regional Health - Western Pacific. 2023;30:100668. doi: https://doi.org/10.1016/j.lanwpc.2022.100668.

4578. Beardsley J, Chambers JM, Lam TT, Zawahir S, Le H, Nguyen TA, et al. Mapping access to drug outlets in Vietnam: distribution of drug outlets and the sociodemographic characteristics of the communities they serve. The Lancet Regional Health - Western Pacific. 2023;30:100668. doi: https://doi.org/10.1016/j.lanwpc.2022.100668.

4579. Beardsley J, Chambers JM, Lam TT, Zawahir S, Le H, Nguyen TA, et al. Mapping access to drug outlets in Vietnam: distribution of drug outlets and the sociodemographic characteristics of the communities serve. Lancet Regional Health-Western Pacific. 2023;30. doi: 10.1016/j.lanwpc.2022.100668. PubMed PMID: WOS:000974232600001.

4580. Beardsley R. Communication skills in pharmacy practice: Lippincott Williams & Wilkins; 2019.

4581. Bearman M, Bessell T. Improving Australian pharmacists' attitudes to internet use in community pharmacy practice. International Journal of Pharmacy Practice. 2005;13(2):117-21. doi: 10.1211/0022357055885.

4582. Bearman M, Bessell T, Gogler J, McPhee W. Educating Australian pharmacists about the use of online information in community pharmacy practice. International Journal of Pharmacy Practice. 2005;13(2):109-15. doi: 10.1211/0022357055876.

4583. Beasley R, Fishwick D, Miles JF, Hendeles L. Preservatives in nebulizer solutions: risks without benefit. Pharmacotherapy. 1998;18(1):130-9. PubMed PMID: CN-01729024.

4584. Beatriz G-C, María José O, Inés J-L, Yolanda HG, Concha Á-D-V, Javier T-S, et al. [Artículo traducido] Errores de medicación en niños que acuden a los servicios de urgencias pediátricas. Farmacia Hospitalaria. 2023;47(4):T141-T7. doi: https://doi.org/10.1016/j.farma.2023.06.001.

4585. Beatriz G-C, María José O, Inés J-L, Yolanda HG, Concha Á-D-V, Javier T-S, et al. Medication errors in children visiting pediatric emergency departments. Farmacia Hospitalaria. 2023;47(4):141-7. doi: https://doi.org/10.1016/j.farma.2023.03.006.

4586. Beatty SJ, McCormick KM, Beale DJ, Bruggeman AM, Rodis JL, Mehta BH, et al. Current trends in outpatient pharmacy services and billing. Journal of the American Pharmacists Association. 2012;52(2):154-60. PubMed PMID: 2010091809.

4587. Beatty SJ, Rodis JL, Bellebaum KL, Mehta BH. Community and ambulatory pharmacy: Evaluation of patient care services and billing patterns before implementation of Medicare Part D. Journal of the American Pharmacists Association. 2006;46(6):707-14. doi: 10.1331/1544-3191.46.6.707.Beatty.

4588. Beatty SJ, Westberg SM, Sharma A. Professional responsibilities reported by pharmacists completing residencies in community-based settings. Journal of the American Pharmacists Association. 2019;59(2):217-+. doi: 10.1016/j.japh.2018.11.004. PubMed PMID: WOS:000460056600012.

4589. Beaubien JM. Effectiveness of recipient restriction programs in Medicaid managed care organizations [Ph.D.]. United States -- Rhode Island: University of Rhode Island; 2005.

4590. Beaucage K, Lachance-Demers H, Ngo TT, Vachon C, Lamarre D, Guévin JF, et al. Telephone follow-up of patients receiving antibiotic prescriptions from community pharmacies. American journal of health-system pharmacy. 2006;63(6):557-63. doi: 10.2146/ajhp050177. PubMed PMID: CN-00563140.

4591. Beaucage K, Lachance-Demers H, Ngo TTT, Vachon C, Lamarre D, Guévin JF, et al. Telephone follow-up of patients receiving antibiotic prescriptions from community pharmacies. American Journal of Health-System Pharmacy. 2006;63(6):557-63. doi: 10.2146/ajhp050177.

4592. Beaucage-Charron J, Rinfret J, Coveney R, Williamson D. Melatonin and Ramelteon for the treatment of delirium: A systematic review and meta-analysis. Journal of Psychosomatic Research. 2023;170:N.PAG-N.PAG. doi: 10.1016/j.jpsychores.2023.111345. PubMed PMID: 164090096.

4593. Beauchemin MP, Lichtenstein MR, Raghunathan R, Doshi SD, Lee S, Law C, et al. Impact of a hospital-based specialty pharmacy in partnership with a care coordination organization on time to delivery and receipt of oral anticancer drugs. JCO Oncology Practice. 2023;19(3):e326-e35.

4594. Beauchesne MF, Nenciu LM, Dinh TH, Tasse M, Fillion A, Labrecque M, et al. Active communication

of a pharmacy discharge plan for patients with respiratory diseases: a pilot study. Journal of pharmacy technology. 2007;23(2):67-74. PubMed PMID: CN-00640827.

4595. Beaudoin FL, Merchant RC, Janicki A, McKaig DM, Babu KM. Preventing iatrogenic overdose: a review of in-emergency department opioid-related adverse drug events and medication errors. Annals of Emergency Medicine. 2015;65(4):423-31. doi: 10.1016/j.annemergmed.2014.11.016. PubMed PMID: 109714374. Language: English. Entry Date: 20150529. Revision Date: 20160507. Publication Type: journal article. Journal Subset: Allied Health.

4596. Beaudoin FL, Merchant RC, Janicki A, McKaig DM, Babu KM. Preventing Iatrogenic Overdose: A Review of In–Emergency Department Opioid-Related Adverse Drug Events and Medication Errors. Annals of Emergency Medicine. 2015;65(4):423-31. doi: https://doi.org/10.1016/j.annemergmed.2014.11.016.

4597. Beaujard M, Buxeraud J, Faure S. Prise en charge officinale des blessures courantes de l'été. Actualités Pharmaceutiques. 2019;58(587, Supplement):8-10. doi: https://doi.org/10.1016/j.actpha.2019.03.026.

4598. Beaujard M, Buxeraud J, Faure S. Treating common summer injuries in the community pharmacy. Actualites Pharmaceutiques. 2019;58(587):8-10. doi: 10.1016/j.actpha.2019.03.026.

4599. Beaujard M, Buxeraud J, Faure S. Pharmacy management of common summer injuries. Actualites Pharmaceutiques. 2019;58(587):8-10. doi: 10.1016/j.actpha.2019.03.026. PubMed PMID: WOS:000469390200009.

4600. Beaulieu S, Bertin E, Bouvrette M, Daigneault AM, El Bouchikhi M, Forget R, et al. Programme ProFil-A training-and-communication network program in nephrology: an interim analysis. Journal of population therapeutics and clinical pharmacology. 2012;19(2):e138-e9. PubMed PMID: CN-01004154.

4601. Beaulieu-Genest L, Chrétien D, Maltais F, Pelletier K, Parent JG, Lacasse Y. Self-administered prescriptions of oral steroids and antibiotics in chronic obstructive pulmonary disease: Are we doing more harm than good? Chronic Respiratory Disease. 2007;4(3):143-7. doi: 10.1177/1479972307079512.

4602. Beaunoyer S, Dupuis S, Dumoulin-Charette A, Marie M, Daigneault AM, Lord A, et al. The impact of profil program on the progression of chronic kidney disease (CKD) and its risk factors. An interim analysis. Journal of population therapeutics and clinical pharmacology. 2013;20(3):e298-e9. PubMed PMID: CN-01006573.

4603. Beaupre LA, Hammal F, Stiegelmar R, Masson E, Finegan B. A community-based pharmacist-led smoking cessation program, before elective total joint replacement surgery, markedly enhances smoking cessation rates. Tobacco Induced Diseases. 2020;18. doi: 10.18332/TID/126405.

4604. Beavers J, Schell RF, VanCleave H, Dillon RC, Simmons A, Chen H, et al. Evaluation of inpatient medication guidance from an artificial intelligence chatbot. American Journal of Health-System Pharmacy. 2023;80(24):1822-9. doi: 10.1093/ajhp/zxad193. PubMed PMID: 174081739.

4605. Bebitoglu BT, Hidiroglu S, Bilir RA, Sarisaltik A, Koc D. Investigation of medication use patterns among pregnant women attending a tertiary referral hospital. Istanbul Journal of Pharmacy. 2022;52(1):90-5. doi: 10.26650/IstanbulJPharm.2022.980889. PubMed PMID: WOS:000793925000013.

4606. Beceren A. SAFETY OF HERBAL DRUGS. Journal of Research in Pharmacy. 2023;27:1-3. doi: 10.29228/jrp.377. PubMed PMID: 173262022.

4607. Becerra-Camargo J, Martinez-Martinez F, Garcia-Jimenez E. A multicentre, double-blind, randomised, controlled, parallel-group study of the effectiveness of a pharmacist-acquired medication history in an

emergency department. BMC health services research. 2013;13:337. doi: 10.1186/1472-6963-13-337. PubMed PMID: CN-01121788.

4608. Becerra-Camargo J, Martínez-Martínez F, García-Jiménez E. The effect on potential adverse drug events of a pharmacist-acquired medication history in an emergency department: a multicentre, double-blind, randomised, controlled, parallel-group study. BMC health services research. 2015;15:337. doi: 10.1186/s12913-015-0990-1. PubMed PMID: CN-01170232.

4609. Becerril Moreno F, Bustamante Munguira E, Garcia Verdejo JA, Bartual Lobato E, Ros Martinez M, Merino de Cos P. [Risk management at an Intensive Care Department: conciliation of medication]. Farmacia Hospitalaria. 2013;37(6):514-20. PubMed PMID: 24256015.

4610. Bech CF, Frederiksen T, Villesen CT, Hojsted J, Nielsen PR, Kjeldsen LJ, et al. Healthcare professionals' agreement on clinical relevance of drug-related problems among elderly patients. International Journal of Clinical Pharmacy. 2018;40(1):119-25. PubMed PMID: 29248987.

4611. Bechara A, Casabé A, De Bonis W, Helien A, Bertolino MV. Recreational Use of Phosphodiesterase Type 5 Inhibitors by Healthy Young Men. The Journal of Sexual Medicine. 2010;7(11):3736-42. doi: https://doi.org/10.1111/j.1743-6109.2010.01965.x.

4612. Bechet C, Pichon R, Giordan A, Bonnabry P. Hospital pharmacists seen through the eyes of physicians: qualitative semi-structured interviews. International Journal of Clinical Pharmacy. 2016;38(6):1483-96. doi: 10.1007/s11096-016-0395-1. PubMed PMID: WOS:000389302300019.

4613. Béchet C, Pichon R, Giordan A, Bonnabry P. A cross-sectional comparison between the perception of physicians and pharmacists concerning the role of the pharmacist in physician training. Annales Pharmaceutiques Françaises. 2018;76(5):408-17. doi: https://doi.org/10.1016/j.pharma.2018.04.002.

4614. Bechman K. The 'oRAcle' Study : Identifying Predictors of Adverse Outcomes in Rheumatoid Arthritis [Ph.D.]. England: University of London, King's College (United Kingdom); 2021.

4615. Beck AF, Bradley CL, Huang B, Simmons JM, Heaton PC, Kahn RS. The Pharmacy-level asthma medication ratio and population health. Pediatrics. 2015;135(6):1009-17. doi: 10.1542/peds.2014-3796.

4616. Beck Dallaghan GL, Hultquist TB, Nickol D, Collier D, Geske J. Attitudes toward interprofessional education improve over time. Journal of Interprofessional Education & Practice. 2018;13:24-6. doi: https://doi.org/10.1016/j.xjep.2018.08.007.

4617. Beck E. The UCSD student-run free clinic project: Transdisciplinary health professional education. Journal of Health Care for the Poor and Underserved. 2005;16(2):207-19. doi: 10.1353/hpu.2005.0026.

4618. Beck S, Bui TT, Davies A, Courtney P, Brown A, Geudens J, et al. An evaluation of the drone delivery of adrenaline auto-injectors for anaphylaxis: Pharmacists' perceptions, acceptance, and concerns. Drones. 2020;4(4):1-21. doi: 10.3390/drones4040066.

4619. Becker C, Bjornson DC, Kuhle JW. Pharmacist Care Plans and Documentation of Follow-up Before the Iowa Pharmaceutical Case Management Program. Journal of the American Pharmacists Association. 2004;44(3):350-7. doi: https://doi.org/10.1331/154434504323063986.

4620. Becker C, Dressman JB, Amidon GL, Junginger HE, Kopp S, Midha KK, et al. Biowaiver Monographs for Immediate Release Solid Oral Dosage Forms: Ethambutol Dihydrochloride**This paper reflects the scientific opinion of the authors and not the policies of regulating agencies.**A project of the International Pharmaceutical Federation FIP, Groupe BCS, http://www.fip.org/bcs. Journal of Pharmaceutical Sciences.

2008;97(4):1350-60. doi: https://doi.org/10.1002/jps.21061.

4621. Becker C, Dressman JB, Junginger HE, Kopp S, Midha KK, Shah VP, et al. Biowaiver monographs for immediate release solid oral dosage forms: Rifampicin. Journal of Pharmaceutical Sciences. 2009;98(7):2252-67. doi: https://doi.org/10.1002/jps.21624.

4622. Becker D, Garcia SG, Ellertson C. Do Mexico City pharmacy workers screen women for health risks when they sell oral contraceptive pills over-the-counter? Contraception. 2004;69(4):295-9. doi: https://doi.org/10.1016/j.contraception.2003.11.014.

4623. Becker K. Efficacy of a behavioral intervention to decrease medication transcription errors among professional nurses: Marquette University; 2009.

4624. Becker MA, Boaz TL, DeMuth A, Andel R. Predictors of emergency commitment for nursing home residents: the role of resident and facility characteristics. International Journal of Geriatric Psychiatry. 2012;27(10):1028-35. doi: 10.1002/gps.2817. PubMed PMID: WOS:000308305500005.

4625. Becker MA, MacDonald P, Hunt B, Jackson RL. Febuxostat (FEB) vs. Allopurinol (ALLO) in treating the hyperuricemia of gout in diabetic patients. Pharmacotherapy. 2011;31(10):381e. PubMed PMID: CN-01035319.

4626. Becker ML, Caspers PW, Kallewaard M, Bruinink RJ, Kylstra NB, Heisterkamp S, et al. Determinants of potential drug–drug interaction associated dispensing in community pharmacies in the Netherlands. Pharmacy world & science. 2007;29:51-7.

4627. Becker ML, Caspers PWJ, Kallewaard M, Bruinink RJ, Kylstra NB, Heisterkamp S, et al. Determinants of potential drug-drug interaction associated dispensing in community pharmacies in the Netherlands. Pharmacy World and Science. 2007;29(2):51-7. doi: 10.1007/s11096-006-9061-3.

4628. Becker ML, Kallewaard M, Caspers PW, Schalekamp T, Stricker BH. Potential determinants of drug-drug interaction associated dispensing in community pharmacies. Drug safety. 2005;28:371-8.

4629. Becker ML, Kallewaard M, Caspers PWJ, Schalekamp T, Stricker BHC. Potential determinants of drug-drug interaction associated dispensing in community pharmacies. Drug Safety. 2005;28(5):371-8. doi: 10.2165/00002018-200528050-00001.

4630. Becker ML, Visser LE, Van Gelder T, Hofman A, Stricker BHC. Increasing exposure to drug-drug interactions between 1992 and 2005 in people aged ≥55 years. Drugs and Aging. 2008;25(2):145-52. doi: 10.2165/00002512-200825020-00006.

4631. Becker ML, Visser LE, van Gelder T, Hofman A, Stricker BHC. Increasing exposure to drug-drug interactions between 1992 and 2005 in people aged >= 55 years. Drugs & Aging. 2008;25(2):145-52. doi: 10.2165/00002512-200825020-00006. PubMed PMID: WOS:000253800100006.

4632. Becker WC, Mattocks KM, Frank JW, Bair MJ, Jankowski RL, Kerns RD, et al. Mixed methods formative evaluation of a collaborative care program to decrease risky opioid prescribing and increase non-pharmacologic approaches to pain management. Addictive Behaviors. 2018;86:138-45. doi: https://doi.org/10.1016/j.addbeh.2018.03.009.

4633. Beckerleg S, Lewando-Hundt G, Eddama M, el Alem A, Shawa R, Abed Y. Purchasing a quick fix from private pharmacies in the Gaza Strip. Social Science & Medicine. 1999;49(11):1489-500. doi: https://doi.org/10.1016/S0277-9536(99)00212-9.

4634. Beckett K, Nyrop K, Pfingst L. Race, drugs, and policing: Understanding disparities in drug delivery arrests. Criminology. 2006;44(1):105-37.

4635. Beckett RD, Crank CW, Wehmeyer A. Effectiveness and feasibility of pharmacist-led admission medication reconciliation for geriatric patients. Journal of pharmacy practice. 2012;25(2):136-41. doi: 10.1177/0897190011422605. PubMed PMID: CN-00971619.

4636. Beckett RD, Ersin OH, Chen YC. Assessment of Student Performance and Perceptions of Learning in a Medication Use Evaluation Project. American Journal of Pharmaceutical Education. 2014;78(8). doi: 10.5688/ajpe788155. PubMed PMID: WOS:000344868000016.

4637. Beckett RD, Gratz MA, Marwitz KK, Hanson KM, Isch J, Robison HD. Development, Validation, and Reliability of a P1 Objective Structured Clinical Examination Assessing the National EPAs. American Journal of Pharmaceutical Education. 2023;87(6):100054. doi: https://doi.org/10.1016/j.ajpe.2023.100054.

4638. Bedenbaugh AV, Bonafede M, Marchlewicz EH, Lee V, Tambiah J. Real-World Health Care Resource Utilization and Costs Among US Patients with Knee Osteoarthritis Compared with Controls. Clinicoeconomics and Outcomes Research. 2021;13:421-35. doi: 10.2147/ceor.S302289. PubMed PMID: WOS:000655300900001.

4639. Bedhomme S, Vaillant-Roussel H, Vorilhon P, Lafarge E, Pereton B, Prunet-Spano C, et al. Pediatric pharmaceutical interventions in self-medication: a descriptive study in community pharmacies. BMC Primary Care. 2023;24(1):232.

4640. Bedia-Tanoh VA, Angora EK, Miezan SAJ, Koné-Bravo EDM, Konaté-Touré A, Bosson-Vanga H, et al. Knowledge and practices of private pharmacy auxiliaries on malaria in Abidjan, Cote d'Ivoire. Malaria Journal. 2023;22(1). doi: 10.1186/s12936-023-04751-8. PubMed PMID: WOS:001092155100001.

4641. Bednall R, Mc Robbie D, Williams D. Identification of patients attending accident and emergency who may be suitable for treatment by a pharmacist. Family Practice. 2003;20(1):54-7. doi: 10.1093/fampra/20.1.54.

4642. Bednarczyk RA, Nadeau JA, Davis CF, McCarthy A, Hussain S, Martiniano R, et al. Privacy in the pharmacy environment: Analysis of observations from inside the pharmacy. Journal of the American Pharmacists Association. 2010;50(3):362-7. doi: 10.1331/JAPhA.2010.09001.

4643. Bedouch P, Allenet B, Grass A, Labarère J, Brudieu E, Bosson J, et al. Drug-related problems in medical wards with a computerized physician order entry system. Journal of Clinical Pharmacy & Therapeutics. 2009;34(2):187-95. doi: 10.1111/j.1365-2710.2008.00990.x. PubMed PMID: 105470502. Language: English. Entry Date: 20090605. Revision Date: 20200708. Publication Type: Journal Article.

4644. Bedouch P, Allenet B, Labarere J, Brudieu E, Chen C, Chevrot D, et al. Diffusion des opinions pharmaceutiques dans le cadre d'une activité de pharmacie clinique en unité de soins. Therapies. 2005;60(5):515-22. doi: https://doi.org/10.2515/therapie:2005015.

4645. Bedouch P, Baudrant M, Detavernier M, Rey C, Brudieu É, Foroni L, et al. La sécurisation du circuit du médicament dans les établissements de santé : données actuelles et expérience du centre hospitalier universitaire de Grenoble. Annales Pharmaceutiques Françaises. 2009;67(1):3-15. doi: https://doi.org/10.1016/j.pharma.2008.10.006.

4646. Bedouch P, Baudrant M, Detavernier M, Rey C, Brudieu E, Foroni L, et al. [Drug supply chain safety in hospitals: current data and experience of the Grenoble university hospital]. Annales Pharmaceutiques Francaises. 2009;67(1):3-15. PubMed PMID: 19152845.

4647. Bedouch P, Charpiat B, Conort O, Rose FX, Escofier L, Juste M, et al. Assessment of clinical pharmacists' interventions in French hospitals: results of a multicenter study. Annals of Pharmacotherapy. 2008;42(7):1095-103. PubMed PMID: 18559955.

4648. Bedouch P, Sylvoz N, Charpiat B, Juste M, Roubille R, Rose FX, et al. Trends in pharmacists' medication order review in French hospitals from 2006 to 2009: analysis of pharmacists' interventions from the Act-IP© website observatory. Journal of Clinical Pharmacy & Therapeutics. 2015;40(1):32-40. PubMed PMID: 25303720.

4649. Bedouch P, Tessier A, Baudrant M, Labarere J, Foroni L, Calop J, et al. Computerized physician order entry system combined with on-ward pharmacist: analysis of pharmacists' interventions. Journal of Evaluation in Clinical Practice. 2012;18(4):911-8. PubMed PMID: 21689216.

4650. Bedrood S, Berdahl J, Sheybani A, Singh IP. Alternatives to Topical Glaucoma Medication for Glaucoma Management. Clinical Ophthalmology. 2023;17:3899-913. doi: 10.2147/OPTH.S439457. PubMed PMID: 174662124.

4651. Beduschi MC, Oesterling JE. Transurethral needle ablation of the prostate: A minimally invasive treatment for symptomatic benign prostatic hyperplasia. Mayo Clinic Proceedings. 1998;73(7):696-701. PubMed PMID: WOS:000074598400015.

4652. Bedwick BW, Garofoli GK, Elswick BM, editors. Assessment of targeted automated messages on herpes zoster immunization numbers in an independent community pharmacy. Journal of the American Pharmacists Association; 2017.

4653. Bedwick BW, Garofoli GK, Elswick BM. Assessment of targeted automated messages on herpes zoster immunization numbers in an independent community pharmacy. Journal of the American Pharmacists Association. 2017;57(3):S293-+. doi: 10.1016/j.japh.2017.02.007. PubMed PMID: WOS:000432472200020.

4654. Bee JS, Goletz TJ, Ragheb JA. The future of protein particle characterization and understanding its potential to diminish the immunogenicity of biopharmaceuticals: A shared perspective. Journal of Pharmaceutical Sciences. 2012;101(10):3580-5. doi: https://doi.org/10.1002/jps.23247.

4655. Bee JS, Randolph TW, Carpenter JF, Bishop SM, Dimitrova MN. Effects of Surfaces and Leachables on the Stability of Biopharmaceuticals. Journal of Pharmaceutical Sciences. 2011;100(10):4158-70. doi: https://doi.org/10.1002/jps.22597.

4656. Beechinor D. Improving Rates of Appropriate Venous Thromboembolism Prophylaxis in a Community Hospital. Blood. 2012;120(21):3159. doi: https://doi.org/10.1182/blood.V120.21.3159.3159.

4657. Beecroft G. Generic drug policy in Australia: a community pharmacy perspective. Australia and New Zealand Health Policy. 2007;4(1).

4658. Beeler PE, Eschmann E, Rosen C, Blaser J. Use of an on-demand drug-drug interaction checker by prescribers and consultants: a retrospective analysis in a Swiss teaching hospital. Drug Safety. 2013;36(6):427-34. doi: 10.1007/s40264-013-0022-1. PubMed PMID: 104171619. Language: English. Entry Date: 20131108. Revision Date: 20211029. Publication Type: journal article.

4659. Beer N, Kaae S, Genina N, Sporrong SK, Alves TL, Hoebert J, et al. Magistral compounding with 3D printing: a promising way to achieve personalized medicine. Therapeutic Innovation & Regulatory Science. 2023;57(1):26-36.

4660. Beer N, Kaae S, Genina N, Sporrong SK, Alves TL, Hoebert J, et al. Magistral Compounding with 3D

Printing: A Promising Way to Achieve Personalized Medicine. Therapeutic Innovation & Regulatory Science. 2023;57(1):26-36.

4661. Beernink JM, Oosterwijk MM, van Boven JFM, Heerspink HJL, Bakker SJL, Navis G, et al. Adherence to Statin Therapy and Attainment of LDL Cholesterol Targets in an Outpatient Population of Type 2 Diabetes Patients: Analysis in the DIAbetes and LifEstyle Cohort Twente (DIALECT). Frontiers in Pharmacology. 2022;13. doi: 10.3389/fphar.2022.888110. PubMed PMID: WOS:000831748900001.

4662. Beers M, Nunemacher C, Holland CR, Rhodes LA, Marciniak MW. Effect of a standardized module for training pharmacy technicians to assist with chronic care management services. Journal of the American Pharmacists Association. 2020;60(3):S80-S3. doi: 10.1016/j.japh.2020.02.015. PubMed PMID: WOS:000542180600014.

4663. Beers MH, Baran RW, Frenia K. Drugs and the elderly, Part 1: The problems facing managed care. American Journal of Managed Care. 2000;6(12):1313-20.

4664. Beers MH, Baran RW, Frenia K. Drugs and the elderly, part 2: Strategies for improving prescribing in a managed care environment. American Journal of Managed Care. 2001;7(1):69-72.

4665. Beex-Oosterhuis MM, de Vogel EM, van der Sijs H, Dieleman HG, van den Bemt PM. Detection and correct handling of prescribing errors in Dutch hospital pharmacies using test patients. International Journal of Clinical Pharmacy. 2013;35(6):1188-202. PubMed PMID: 24062191.

4666. Beex-Oosterhuis MM, de Vogel EM, van der Sijs H, Dieleman HG, van den Bemt PMLA. Detection and correct handling of prescribing errors in Dutch hospital pharmacies using test patients. International Journal of Clinical Pharmacy. 2013;35(6):1188-202. doi: 10.1007/s11096-013-9848-y. PubMed PMID: 104122492. Language: English. Entry Date: 20140912. Revision Date: 20211029. Publication Type: journal article.

4667. Beex-Oosterhuis MM, Heerdink ER, Van Gool AR, van Marum RJ. Predicting Unsuccessful Clozapine Treatment After First Use in Adult Patients With Psychotic Disorders. Journal of Clinical Psychopharmacology. 2018;38(6):604-8. doi: 10.1097/jcp.0000000000000977. PubMed PMID: WOS:000450084600012.

4668. Beex-Oosterhuis MM, Heerdink ERR, Van Gool AR, Van Marum RJ. Predicting unsuccessful clozapine treatment after first use in adult patients with psychotic disorders. Journal of Clinical Psychopharmacology. 2018;38(6):604-8. doi: 10.1097/JCP.0000000000000977.

4669. Beganovic M, Wieczorkiewicz SM. MALDI-TOF alone versus MALDI-TOF combined with real-time antimicrobial stewardship interventions on time to optimal antimicrobial therapy in patients with positive blood cultures. Open forum infectious diseases. 2016;3. doi: 10.1093/ofid/ofw172.1421. PubMed PMID: CN-02080504.

4670. Beger RD, Dunn W, Schmidt MA, Gross SS, Kirwan JA, Cascante M, et al. Metabolomics enables precision medicine:“a white paper, community perspective”. Metabolomics. 2016;12:1-15.

4671. Begg EJ, Barclay ML. Aminoglycosides--50 years on. British journal of clinical pharmacology. 1995;39(6):597.

4672. Beggi B, Asik Z. Evaluation of rational drug use in patients applying to family medicine outpatient clinic. Ankara Medical Journal. 2019;18(2):251-60. doi: 10.17098/AMJ.582021.

4673. Begley K, O’brien K, Packard K, Castillo S, Haddad AR, Johnson K, et al. Impact of interprofessional

telehealth case activities on students' perceptions of their collaborative care abilities. American journal of pharmaceutical education. 2019;83(4).

4674. Begley KJ, Coover KL, Tilleman JA, Haddad AM, Augustine SC. Medication therapy management training using case studies and the MirixaPro platform. American Journal of Pharmaceutical Education. 2011;75(3):49. PubMed PMID: 21655403.

4675. Begley S. The establishment and evaluation of a domiciliary pharmaceutical service [Ph.D.]. England: University of Brighton (United Kingdom); 1995.

4676. Begley S. WHY ALMOST EVERYTHING YOU HEAR ABOUT MEDICINE IS WRONG. Newsweek. 2011;157(5):8. PubMed PMID: 57649287.

4677. Begley S, Livingstone C, Hodges N, Williamson V. Impact of domiciliary pharmacy visits on medication management in an elderly population. International journal of pharmacy practice. 1997;5(3):111-21. PubMed PMID: CN-00196385.

4678. Begum A. SIGNIFICANCE AND CHALLENGES IN MEDICATION THERAPY MANAGEMENT (MTM.). International Journal of Applied Pharmaceutics. 2023;15(1):207. PubMed PMID: 640230533.

4679. Begun Z, Thompson E, Zhan T, Keiffer G, Kasner M, Wilde L. Tumor Lysis Syndrome in Patients with AML Initiated on Venetoclax-Based Treatment without a Dose Ramp-up. Blood. 2023;142:3720. doi: https://doi.org/10.1182/blood-2023-184610.

4680. Behnam B, Semnani V, Saghafi N, Ghorbani R, Shori MD, Choobmasjedi SG. Gabapentin effect on pain associated with heroin withdrawal in Iranian crack: a randomized double-blind clinical trial. Iranian journal of pharmaceutical research. 2012;11(3):979-83. PubMed PMID: CN-00903099.

4681. Behta M, Ross B, Chaudhry R. A comprehensive decision support system for the identification, monitoring and management of patients with multi-drug resistant organisms (MDRO). AMIA Annual Symposium proceedings / AMIA Symposium AMIA Symposium. 2008:1218.

4682. Beiderbeck AB, Holly EA, Sturkenboom M, Coebergh JW, Stricker BHC, Leufkens HGM. No increased risk of non-Hodgkin's lymphoma with steroids, estrogens and psychotropics (Netherlands). Cancer Causes & Control. 2003;14(7):639-44. doi: 10.1023/a:1025698109991. PubMed PMID: WOS:000185261300005.

4683. Beiderbeck AB, Holly EA, Sturkenboom M, Coebergh JWW, Stricker BHC, Leufkens HGM. Prescription medications associated with a decreased risk of non-Hodgkin's lymphoma. American Journal of Epidemiology. 2003;157(6):510-6. doi: 10.1093/aje/kwg004. PubMed PMID: WOS:000181611600005.

4684. Beiderbeck AB, Holly EA, Sturkenboom MCJM, Coebergh JW, Stricker BHC, Leufkens HGM. No increased risk of non-Hodgkin's lymphoma with steroids, estrogens and psyehotropies (Netherlands). Cancer Causes and Control. 2003;14(7):639-44. doi: 10.1023/A:1025698109991.

4685. Beiderbeck AB, Holly EA, Sturkenboom MCJM, Coebergh JWW, Stricker BHC, Leufkens HGM. Prescription medications associated with a decreased risk of non-Hodgkin's lymphoma. American Journal of Epidemiology. 2003;157(6):510-6. doi: 10.1093/aje/kwg004.

4686. Beiderbeck-Noll AB, Sturkenboom M, van der Linden PD, Herings RMC, Hofman A, Coebergh JWW, et al. Verapamil is associated with an increased risk of cancer in the elderly: the Rotterdam study. European Journal of Cancer. 2003;39(1):98-105. doi: 10.1016/s0959-8049(02)00157-0. PubMed PMID: WOS:000181156200015.

4687. Beiderbeck-Noll AB, Sturkenboom MCJM, van der Linden PD, Herings RMC, Hofman A, Coebergh JWW, et al. Verapamil is associated with an increased risk of cancer in the elderly: the Rotterdam study. European Journal of Cancer. 2003;39(1):98-105. doi: https://doi.org/10.1016/S0959-8049(02)00157-0.

4688. Beiers-Jones K, Doyle B, Lanciotti K, Lemon E. Population Health Projects: An Innovative Teaching Strategy for Baccalaureate Nursing Education. Journal of Professional Nursing. 2023;49:26-32. doi: 10.1016/j.profnurs.2023.08.011.

4689. Beigel JH, Voell J, Kumar P, Raviprakash K, Wu H, Jiao J-A, et al. Safety and tolerability of a novel, polyclonal human anti-MERS coronavirus antibody produced from transchromosomic cattle: a phase 1 randomised, double-blind, single-dose-escalation study. The Lancet Infectious Diseases. 2018;18(4):410-8. doi: https://doi.org/10.1016/S1473-3099(18)30002-1.

4690. Beigel JH, Voell J, Kumar P, Raviprakash K, Wu H, Jiao JA, et al. Safety and tolerability of a novel, polyclonal human anti-MERS coronavirus antibody produced from transchromosomic cattle: a phase 1 randomised, double-blind, single-dose-escalation study. Lancet infectious diseases. 2018;18(4):410-8. doi: 10.1016/S1473-3099%2818%2930002-1. PubMed PMID: CN-01603631.

4691. Beijer H, De Blaey C. Hospitalisations caused by adverse drug reactions (ADR): a meta-analysis of observational studies. Pharmacy World and Science. 2002;24(2):46-54.

4692. Beilby J. The general practitioner and the pharmacist: a policy enigma? Medical Journal of Australia. 2021;214(5):210-1. PubMed PMID: 33634476.

4693. Bejerholm U, Allaskog C, Andersson J, Nordstrom L, Roe D. Implementation of the Recovery Guide in inpatient mental health services in Sweden-A process evaluation study. Health Expectations. 2022;25(4):1405-17. doi: 10.1111/hex.13480. PubMed PMID: WOS:000773357300001.

4694. Bekele F, Bereda G, Tamirat L, Geleta BA, Jabessa D. “Childrens are not just “little adults”. The rate of medication related problems and its predictors among patients admitted to pediatric ward of southwestern Ethiopian hospital: A prospective observational study. Annals of Medicine and Surgery. 2021;70:102827. doi: https://doi.org/10.1016/j.amsu.2021.102827.

4695. Bekele F, Fekadu G, Bekele K, Dugassa D, Sori J. Drug-related problems among patients with infectious disease admitted to medical wards of Wollega University Referral Hospital: Prospective observational study. Sage Open Medicine. 2021;9. doi: 10.1177/2050312121989625. PubMed PMID: WOS:000613631300001.

4696. Bekelman JE, Lu H, Pugh S, Baker K, Berg CD, de Gonzalez AB, et al. Pragmatic randomised clinical trial of proton versus photon therapy for patients with non-metastatic breast cancer: the Radiotherapy Comparative Effectiveness (RadComp) Consortium trial protocol. Bmj Open. 2019;9(10). doi: 10.1136/bmjopen-2018-025556. PubMed PMID: WOS:000512882200014.

4697. Bekker C, Van Den Bemt B, Egberts A, Bouvy M, Gardarsdottir H. Patient and medication factors associated with preventable medication waste and possibilities for redispensing. International journal of clinical pharmacy. 2018;40:704-11.

4698. Bekker C, Van Den Bemt B, Egberts TCG, Bouvy M, Gardarsdottir H. Willingness of patients to use unused medication returned to the pharmacy by another patient: A cross-sectional survey. BMJ Open. 2019;9(5). doi: 10.1136/bmjopen-2018-024767.

4699. Bekker CL, Gardarsdottir H, Egberts ACG, Molenaar HA, Bouvy ML, Van Den Bemt BJF, et al. What does it cost to redispense unused medications in the pharmacy? A micro-costing study. BMC Health Services

Research. 2019;19(1). doi: 10.1186/s12913-019-4065-6.

4700. Bekker CL, Melis EJ, Egberts ACG, Bouvy ML, Gardarsdottir H, van den Bemt BJF. Quantity and economic value of unused oral anti-cancer and biological disease-modifying anti-rheumatic drugs among outpatient pharmacy patients who discontinue therapy. Research in Social & Administrative Pharmacy. 2019;15(1):100-5. doi: 10.1016/j.sapharm.2018.03.064. PubMed PMID: WOS:000452731700025.

4701. Bekker CL, van den Bemt BJF, Egberts ACG, Bouvy ML, Gardarsdottir H. Patient and medication factors associated with preventable medication waste and possibilities for redispensing. International Journal of Clinical Pharmacy. 2018;40(3):704-11. doi: 10.1007/s11096-018-0642-8.

4702. Bekker LG, Montaner J, Ramos C, Sherer R, Celletti F, Cutler B, et al. IAPAC guidelines for optimizing the HIV care continuum for adults and adolescents. Journal of the International Association of Providers of AIDS Care. 2015;14:S3-S34. doi: 10.1177/2325957415613442.

4703. Bekkers EJ. Multi-scale vascular geometry construction and hemodynamic quantification from medical image data [Ph.D.]. United States -- California: Stanford University; 2006.

4704. Beks H, Namara KM, Manias E, Dalton A, Tong E, Dooley M. Hospital pharmacists' experiences of participating in a partnered pharmacist medication charting credentialing program: a qualitative study. BMC Health Services Research. 2021;21(1):1-9. doi: 10.1186/s12913-021-06267-w. PubMed PMID: 149371128. Language: English. Entry Date: In Process. Revision Date: 20210923. Publication Type: journal article. Journal Subset: Biomedical.

4705. Bektay MY. Göğüs Hastaliklari Servisinde Klinik Eczacilik Hizmetlerinin Geliştirilmesi ve Değerlendirilmesi [Ph.D.]. Turkey: Marmara Universitesi (Turkey); 2022.

4706. Bektay MY, Sancar M, Okyaltirik F, Durdu B, Izzettin FV. Investigation of drug-related problems in patients hospitalized in chest disease wards: A randomized controlled trial. Frontiers in Pharmacology. 2023;13:1049289.

4707. Belachew EA, Sendekie AK, Wondm SA, Ayele EM, Netere AK. Misunderstanding of dosing regimen instructions among patients with chronic diseases receiving polypharmacy at the University of Gondar comprehensive specialized hospital. Plos One. 2023;18(1). doi: 10.1371/journal.pone.0280204. PubMed PMID: WOS:000946156200001.

4708. Belachew SA, Hall L, Erku DA, Selvey LA. No prescription? No problem: drivers of non-prescribed sale of antibiotics among community drug retail outlets in low and middle income countries: a systematic review of qualitative studies. BMC Public Health. 2021;21(1). doi: 10.1186/s12889-021-11163-3.

4709. Belachew SA, Hall L, Selvey LA. Non-prescription dispensing of antibiotic agents among community drug retail outlets in Sub-Saharan African countries: a systematic review and meta-analysis. Antimicrobial Resistance and Infection Control. 2021;10(1). doi: 10.1186/s13756-020-00880-w.

4710. Belachew SA, Hall L, Selvey LA. Community drug retail outlet staff’s knowledge, attitudes and practices towards non-prescription antibiotics use and antibiotic resistance in the Amhara region, Ethiopia with a focus on non-urban towns. Antimicrobial Resistance and Infection Control. 2022;11(1). doi: 10.1186/s13756-022-01102-1.

4711. Belachew SA, Hall L, Selvey LA. Magnitude of non-prescribed antibiotic dispensing in Ethiopia: a multicentre simulated client study with a focus on non-urban towns. The Journal of antimicrobial chemotherapy. 2022;77(12):3462-5. doi: 10.1093/jac/dkac341.

4712. Belachew SA, Hall L, Selvey LA. “Handing out non-prescribed antibiotics is storing up trouble for the next generation!” Unpacking multistakeholder views of drivers and potential solutions in Ethiopia. BMC Health Services Research. 2023;23(1):830.

4713. Belachew SA, Muluneh NY, Erku DA, Netere AK. A cross sectional study on beliefs and roles of community pharmacy professionals in preventing and managing metabolic syndrome in an Ethiopian setting. Plos One. 2020;15(12). doi: 10.1371/journal.pone.0244211. PubMed PMID: WOS:000603071100037.

4714. Belachew SA, Muluneh NY, Erku DA, Netere AK. A cross sectional study on beliefs and roles of community pharmacy professionals in preventing and managing metabolic syndrome in an Ethiopian setting. PLoS ONE. 2021;15(12 December). doi: 10.1371/journal.pone.0244211.

4715. Belachew SA, Tilahun F, Ketsela T, Ayele AA, Netere AK, Mersha AG, et al. Competence in metered dose inhaler technique among community pharmacy professionals in Gondar town, Northwest Ethiopia: Knowledge and skill gap analysis. PLoS ONE. 2017;12(11). doi: 10.1371/journal.pone.0188360.

4716. Belaiche S, Goulois S, DeBerranger E, Odou P, Balagny S, Decaudin B. Clinical pharmacist and pharmaceutical interventions in HBH unit: a French observational study. Acta Clinica Belgica. 2021;76(4):258-63. doi: 10.1080/17843286.2020.1716148. PubMed PMID: WOS:000509014400001.

4717. Belaiche S, Mercier E, Cuny D, Kambia N, Wierre P, Bertoux É, et al. Implication du pharmacien d’officine dans le parcours de soins de la maladie rénale chronique. Néphrologie & Thérapeutique. 2017;13(2):87-92. doi: https://doi.org/10.1016/j.nephro.2016.06.006.

4718. Belaiche S, Mercier E, Cuny D, Kambia N, Wierre P, Bertoux É, et al. Community pharmacists’ interventions to prevent and screen chronic kidney disease patients. Nephrologie et Therapeutique. 2017;13(2):87-92. doi: 10.1016/j.nephro.2016.06.006.

4719. Belaiche S, Mercier E, Cuny D, Kambia N, Wierre P, Bertoux E, et al. Community pharmacists' interventions to prevent and screen chronic kidney disease patients. Nephrologie & Therapeutique. 2017;13(2):87-92. doi: 10.1016/j.nephro.2016.06.006. PubMed PMID: WOS:000400720700004.

4720. Belaiche S, Mercier E, Cuny D, Kambia N, Wierre P, Bertoux E, et al. Community pharmacists' interventions to prevent and screen chronic kidney disease patients. Journal de Pharmacie Clinique. 2020;39(1):29-36. doi: 10.1016/j.nephro.2016.06.006.

4721. Belaiche S, Romanet T, Allenet B, Calop J, Zaoui P. Identification of drug-related problems in ambulatory chronic kidney disease patients: a 6-month prospective study. Journal of Nephrology. 2012;25(5):782-8. PubMed PMID: 22322820.

4722. Belaiche S, Romanet T, Bell R, Calop J, Allenet B, Zaoui P. Pharmaceutical care in chronic kidney disease: experience at Grenoble University Hospital from 2006 to 2010. Journal of Nephrology. 2012;25(4):558-65. PubMed PMID: 22101677.

4723. Belavy DL, Tagliaferri SD, Buntine P, Saueressig T, Sadler K, Ko C, et al. Clinician education unlikely effective for guideline-adherent medication prescription in low back pain: systematic review and meta-analysis of RCTs. eClinicalMedicine. 2022;43:101193. doi: https://doi.org/10.1016/j.eclinm.2021.101193.

4724. Belcher J, Nielsen S, Campbell G, Bruno R, Hoban B, Larance B, et al. Diversion of prescribed opioids by people living with chronic pain: Results from an Australian community sample. Drug and Alcohol Review. 2014;33(1):27-32. doi: 10.1111/dar.12084.

4725. Belcher RM, Blair A, Chauv S, Hoang Q, Hickman AW, Peng M, et al. Implementation and Impact of

Critical Care Pharmacist Addition to a Telecritical Care Network. Critical Care Explorations. 2023;5(1):E0839. doi: 10.1097/CCE.0000000000000839.

4726. Belcher RM, Blair A, Chauv S, Hoang Q, Hickman AW, Peng M, et al. Implementation and Impact of Critical Care Pharmacist Addition to a Telecritical Care Network. Critical Care Explorations. 2023;5(1):E0839. doi: 10.1097/CCE.0000000000000839.

4727. Belda-Rustarazo S, Cantero-Hinojosa J, Salmeron-García A, González-García L, Cabeza-Barrera J, Galvez J. Medication reconciliation at admission and discharge: an analysis of prevalence and associated risk factors. International Journal of Clinical Practice. 2015;69(11):1268-74. doi: 10.1111/ijcp.12701. PubMed PMID: 110546832. Language: English. Entry Date: 20161223. Revision Date: 20170104. Publication Type: journal article.

4728. Belete TM, Tadesse SA, Atnafu K, Kelemu M, Asrie AB. Patient satisfaction with antiretroviral therapy service provided by pharmacists in Dembia district health institutions, Northwest Ethiopia. AIDS Research and Therapy. 2023;20(1). doi: 10.1186/s12981-023-00533-z.

4729. Beleva Y, Pike JR, Miller S, Xie B, Ames SL, Stacy AW. Share of Advertising Voice at the Point-of-Sale and Its Influence on At-Risk Students' Use of Alternative Tobacco Products. Nicotine & Tobacco Research. 2019;21(7):903-10. doi: 10.1093/ntr/nty152. PubMed PMID: WOS:000493384200008.

4730. Belew S, Suleman S, Wynendaele E, D'Hondt M, Kosgei A, Duchateau L, et al. Quality of anthelminthic medicines available in Jimma Ethiopia. Acta Tropica. 2018;177:157-63. doi: 10.1016/j.actatropica.2017.10.006.

4731. Belew S, Suleman S, Wynendaele E, D’Hondt M, Kosgei A, Duchateau L, et al. Quality of anthelminthic medicines available in Jimma Ethiopia. Acta Tropica. 2018;177:157-63. doi: https://doi.org/10.1016/j.actatropica.2017.10.006.

4732. Belfield KD, Kuyumjian AG, Teran R, Amadi M, Blatt M, Bicking K. Impact of A Collaborative Strategy to Reduce the Inappropriate Use of Acid Suppressive Therapy in Non-Intensive Care Unit Patients. Annals of Pharmacotherapy. 2017;51(7):577-83. doi: 10.1177/1060028017698797. PubMed PMID: 123662235. Language: English. Entry Date: 20170910. Revision Date: 20191029. Publication Type: journal article.

4733. Belfodil F, de Falvelly D, Medjahed S, Belmin J. [Medication review by pharmacist-geriatrician collaboration for elderly inpatients: The absence of drug indication is the main target]. Presse Medicale. 2015;44(9):956-8. PubMed PMID: 26067574.

4734. Belfrage B, Koldestam A, Sjöberg C, Wallerstedt SM. Prevalence of suboptimal drug treatment in patients with and without multidose drug dispensing—a cross-sectional study. European journal of clinical pharmacology. 2014;70:867-72.

4735. Beliard RC, Merrey JW. Evaluation of interventions in clinical pharmacist-led annual medicare wellness visits compared with usual care. Consultant Pharmacist. 2017;32(11):687-99. doi: 10.4140/TCP.n.2017.687.

4736. Belizário SC. Diclofenac: Perfil de Utilização, Efeitos Secundários e Interações Medicamentosas Experiência Profissionalizante Na Vertente de Farmácia Comunitária e Investigação [M.Sc.]. Portugal: Universidade da Beira Interior (Portugal); 2017.

4737. Beljaars D. Geographies of compulsive interactions: bodies, objects, spaces [Ph.D.]. Wales: Cardiff University (United Kingdom); 2018.

4738. Belkina T, Al Warafi A, Eltom EH, Tadjieva N, Kubena A, Vlcek J. Antibiotic use and knowledge in the community of Yemen, Saudi Arabia, and Uzbekistan. Journal of Infection in Developing Countries. 2014;8(4):424-9. doi: 10.3855/jidc.3866. PubMed PMID: WOS:000339927900005.

4739. Belkina T, Al Warafi A, Hussein Eltom E, Tadjieva N, Kubena A, Vlcek J. Antibiotic use and knowledge in the community of Yemen, Saudi Arabia, and Uzbekistan. Journal of Infection in Developing Countries. 2014;8(4):424-9. doi: 10.3855/jidc.3866.

4740. Belkina TV, Khojiev DS, Tillyashaykhov MN, Tigay ZN, Kudenov MU, Tebbens JD, et al. Delay in the diagnosis and treatment of pulmonary tuberculosis in Uzbekistan: A cross-sectional study. BMC Infectious Diseases. 2014;14(1). doi: 10.1186/s12879-014-0624-y.

4741. Bell C, Daniel S. Pharmacy leader's role in hospital emergency preparedness planning. Hospital Pharmacy. 2014;49(4):398-404. doi: 10.1310/hpj4904-398.

4742. Bell C, Moore J, Couldry R, Grauer D. The Use of Individualized Pharmacist Performance Reports to Reduce Pharmacist-Related Medication Order Entry Errors Following Electronic Medical Record Implementation. Hospital Pharmacy. 2012;47(10):771-5. doi: 10.1310/hpj4710-771. PubMed PMID: 82371357. Language: English. Entry Date: 20121019. Revision Date: 20190102. Publication Type: Article.

4743. Bell CA, Duncan GJ, Eang R, Saini B. Stakeholder perceptions of a pharmacy-initiated tuberculosis referral program in Cambodia, 2005-2012. Asia-Pacific Journal of Public Health. 2015;27(2):NP2570-NP7. doi: 10.1177/1010539513500335.

4744. Bell CA, Eang MT, Dareth M, Rothmony E, Duncan GJ, Saini B. Provider perceptions of pharmacy-initiated tuberculosis referral services in Cambodia, 2005-2010. International Journal of Tuberculosis and Lung Disease. 2012;16(8):1086-91. doi: 10.5588/ijtld.11.0669.

4745. Bell CA, Ilomäki J, Pichenda K, Duncan GJ, Saini B. Referral of tuberculosis symptomatic clients from private pharmacies to public sector clinics for diagnosis and treatment in Cambodia. Journal of Evaluation in Clinical Practice. 2015;21(2):285-91. doi: 10.1111/jep.12306.

4746. Bell CF, Priest J, Stott-Miller M, Kan H, Amelio J, Song X, et al. Real-world treatment patterns, healthcare resource utilisation and costs in patients with systemic lupus erythematosus treated with belimumab: a retrospective analysis of claims data in the USA. Lupus Science & Medicine. 2020;7(1). doi: 10.1136/lupus-2019-000357. PubMed PMID: WOS:000527760600003.

4747. Bell CF, Wu B, Huang SP, Rubin B, Averell CM, Chastek B, et al. Healthcare Resource Utilization and Associated Costs in Patients With Systemic Lupus Erythematosus Diagnosed With Lupus Nephritis. Cureus Journal of Medical Science. 2023;15(4). doi: 10.7759/cureus.37839. PubMed PMID: WOS:001067438000032.

4748. Bell CM, Bajcar J, Bierman AS, Li P, Mamdani MM, Urbach DR. Potentially unintended discontinuation of long-term medication use after elective surgical procedures. Archives of internal medicine. 2006;166(22):2525-31.

4749. Bell CM, Brener SS, Gunraj N, Huo C, Bierman AS, Scales DC, et al. Association of ICU or hospital admission with unintentional discontinuation of medications for chronic diseases. Jama. 2011;306(8):840-7.

4750. Bell CM, Rahimi-Darabad P, Orner AI. Discontinuity of chronic medications in patients discharged from the intensive care unit. Journal of general internal medicine. 2006;21(9):937-41.

4751. Bell DS, Cretin S, Marken RS, Landman AB. A conceptual framework for evaluating outpatient

electronic prescribing systems based on their functional capabilities. Journal of the American Medical Informatics Association. 2004;11(1):60-70.

4752. Bell DS, Friedman MA. E-prescribing and the Medicare Modernization Act of 2003. Health Affairs. 2005;24(5):1159-69. doi: 10.1377/hlthaff.24.5.1159. PubMed PMID: 106538730. Language: English. Entry Date: 20051111. Revision Date: 20200708. Publication Type: Journal Article.

4753. Bell HM, McElnay JC, Hughes CM. A self-reported work sampling study in community pharmacy practice. Pharmacy World and Science. 1999;21:210-6.

4754. Bell HM, McElnay JC, Hughes CM. Changing profile in the use of anti-asthma drugs. Pharmacoepidemiology and Drug Safety. 1999;8(6):385-94. PubMed PMID: WOS:000083809500002.

4755. Bell HM, McElnay JC, Hughes CM, Gleadhill I. Primary schoolteachers' knowledge of asthma: the impact of pharmacist intervention. Journal of asthma. 2000;37(7):545-55. doi: 10.3109/02770900009090809. PubMed PMID: CN-00331659.

4756. Bell HT, Granas AG, Enmarker I, Omli R, Steinsbekk A. Nurses' and pharmacists' learning experiences from participating in interprofessional medication reviews for elderly in primary health care - a qualitative study. BMC Family Practice. 2017;18(1):30. PubMed PMID: 28241789.

4757. Bell IR, Lewis DA, Brooks AJ, Schwartz GE, Lewis SE, Caspi O, et al. Individual differences in response to randomly assigned active individualized homeopathic and placebo treatment in fibromyalgia: implications of a double-blinded optional crossover design. Journal of alternative and complementary medicine (New York, NY). 2004;10(2):269-83. doi: 10.1089/107555304323062266. PubMed PMID: CN-00482302.

4758. Bell IR, Lewis Ii DA, Brooks AJ, Schwartz GE, Lewis SE, Caspi O, et al. Individual Differences in Response to Randomly Assigned Active Individualized Homeopathic and Placebo Treatment in Fibromyalgia: Implications of a Double-Blinded Optional Crossover Design. Journal of Alternative and Complementary Medicine. 2004;10(2):269-83. doi: 10.1089/107555304323062266.

4759. Bell J, Dziekan G, Pollack C, Mahachai V. Self-Care in the Twenty First Century: A Vital Role for the Pharmacist. Advances in Therapy. 2016;33(10):1691-703. doi: 10.1007/s12325-016-0395-5.

4760. Bell JA, Galaznik A, Blazer M, Shih HC, Farrelly E, Ogbonnaya A, et al. Economic Burden of Patients Treated for Higher-Risk Myelodysplastic Syndromes (HR-MDS) in Routine Clinical Care in the United States. Pharmacoeconomics-Open. 2019;3(2):237-45. doi: 10.1007/s41669-018-0100-5. PubMed PMID: WOS:000468880700012.

4761. Bell JS, Kirkpatrick CM, Alderman CP. Balancing evidence and social responsibility when targeting clinical pharmacy services: the example of people with intellectual disabilities. International Journal of Clinical Pharmacy. 2015;37(4):549-50. PubMed PMID: 25903939.

4762. Bell JS, Ooi CE, Troeung L, Craik S, Walton R, Martini A. Protocol for a pilot and feasibility study of nurse practitioner-pharmacist telehealth collaboration to simplify complex medication regimens. Research in social & administrative pharmacy : RSAP. 2022;18(9):3687-93. doi: 10.1016/j.sapharm.2022.03.010. PubMed PMID: CN-02430752.

4763. Bell JS, Rosen A, Aslani P, Whitehead P, Chen TF. Developing the role of pharmacists as members of community mental health teams: perspectives of pharmacists and mental health professionals. Research in Social and Administrative Pharmacy. 2007;3(4):392-409.

4764. Bell JS, Väänänen M, Ovaskainen H, Närhi U, Airaksinen MS. Providing patient care in community pharmacies: Practice and research in Finland. Annals of Pharmacotherapy. 2007;41(6):1039-46. doi: 10.1345/aph.1H638.

4765. Bell JS, Whitehead P, Aslani P, McLachlan AJ, Chen TF. Drug-related problems in the community setting: Pharmacists' findings and recommendations for people with mental illnesses. Clinical Drug Investigation. 2006;26(7):415-25. doi: 10.2165/00044011-200626070-00003.

4766. Bell JS, Whitehead P, Aslani P, McLachlan AJ, Chen TF. Drug-related problems in the community setting - Pharmacists' findings and recommendations for people with mental illnesses. Clinical Drug Investigation. 2006;26(7):415-25. doi: 10.2165/00044011-200626070-00003. PubMed PMID: WOS:000239379200003.

4767. Bell K, Hartmann C, Baughman AW. A pharmacist-led pilot using a performance dashboard to improve psychotropic medication use in a skilled nursing facility. BMJ Open Quality. 2020;9(3):08. PubMed PMID: 32816865.

4768. Bell S, Kelly H, Hennessy E, Bermingham M, O'Flynn JR, Sahm LJ. Healthcare professional perspectives on medication challenges in the post-stroke patient. Frontiers in Pharmacology. 2023;14. doi: 10.3389/fphar.2023.1266277. PubMed PMID: WOS:001115895800001.

4769. Bell S, Kelly H, Hennessy E, Bermingham M, O'Flynn JR, Sahm LJ. Healthcare professional perspectives on medication challenges in the post-stroke patient. Frontiers in Pharmacology. 2023;14. doi: 10.3389/fphar.2023.1266277.

4770. Bell S, McLachlan AJ, Aslani P, Whitehead P, Chen TF. Community pharmacy services to optimise the use of medications for mental illness: A systematic review. Australia and New Zealand Health Policy. 2005;2(1). doi: 10.1186/1743-8462-2-29.

4771. Bell S, McLachlan AJ, Chen TF, Whitehead P, Aslani P. Community pharmacy services to optimise the use of medications for mental illness: a systematic review. Australia and New Zealand health policy. 2005;2(1).

4772. Bell SA, Munro-Kramer ML, Eisenberg MC, Williams G, Amarah P, Lori JR. "Ebola kills generations": Qualitative discussions with Liberian healthcare providers. Midwifery. 2017;45:44-9. doi: 10.1016/j.midw.2016.12.005.

4773. Bell SK, Delbanco T, Elmore JG, Fitzgerald PS, Fossa A, Harcourt K, et al. Frequency and types of patient-reported errors in electronic health record ambulatory care notes. JAMA network open. 2020;3(6):e205867-e.

4774. Bell SO, Shankar M, OlaOlorun F, Omoluabi E, Khanna A, Ahmad D, et al. Menstrual regulation: examining the incidence, methods, and sources of care of this understudied health practice in three settings using cross-sectional population-based surveys. BMC Women's Health. 2023;23(1). doi: 10.1186/s12905-023-02216-3.

4775. Bell SP, Schnipper JL, Goggins K, Bian A, Shintani A, Roumie C, et al. Effect of a pharmacist counseling intervention on healthcare utilization after hospital discharge: a randomized controlled trial. Journal of general internal medicine. 2015;30:S55-. PubMed PMID: CN-01716700.

4776. Bell SP, Schnipper JL, Goggins K, Bian A, Shintani A, Roumie CL, et al. Effect of Pharmacist Counseling Intervention on Health Care Utilization Following Hospital Discharge: a Randomized Control Trial. Journal of general internal medicine. 2016;31(5):470-7. doi: 10.1007/s11606-016-3596-3. PubMed

PMID: CN-01153457.

4777. Bell SP, Schnipper JL, Goggins KM, Bian A, Shintani A, Roumie CL. Effect of a pharmacist counseling intervention on healthcare utilization after hospital discharge: a randomized controlled trial. Journal of general internal medicine. 2015;30(Supplement 2):S55. PubMed PMID: CN-01412550.

4778. Bell T, Sprajcer M, Flenady T, Sahay A. Fatigue in nurses and medication administration errors: A scoping review. Journal of Clinical Nursing (John Wiley & Sons, Inc). 2023;32(17/18):5445-60. doi: 10.1111/jocn.16620. PubMed PMID: 170008015.

4779. Bell TJ, Poston SA, Kraft MD, Senagore AJ, Delaney CP, Techner L. Economic analysis of alvimopan in North American Phase III efficacy trials. American journal of health-system pharmacy. 2009;66(15):1362-8. PubMed PMID: CN-00741219.

4780. Bell-Higgs AE, Brosnahan NT, Clarke AM, Dow MS, Haynes SM, Lyons GF, et al. The implementation of the Counterweight programme in scotland, UK. Family Practice. 2012;29(SUPPL. 1):i139-i44. doi: 10.1093/fampra/cmr074.

4781. Bell-Higgs AE, Brosnahan NT, Clarke AM, Dow MSA, Haynes SM, Lyons GF, et al. The implementation of the Counterweight Programme in Scotland, UK. Family Practice. 2012;29:i139-i44. doi: 10.1093/fampra/cmr074. PubMed PMID: WOS:000301298400021.

4782. Bellamy K, Ostini R, Martini N, Kairuz T. Access to medication and pharmacy services for resettled refugees: A systematic review. Australian Journal of Primary Health. 2015;21(3):273-8. doi: 10.1071/PY14121.

4783. Bellamy K, Ostini R, Martini N, Kairuz T. Perspectives of resettled African refugees on accessing medicines and pharmacy services in Queensland, Australia. International Journal of Pharmacy Practice. 2017;25(5):358-64. doi: 10.1111/ijpp.12324.

4784. Bellamy MF, Pellikka PA, Klarich KW, Tajik AJ, Enriquez-Sarano M. Association of cholesterol levels, hydroxymethylglutaryl coenzyme-A reductase inhibitor treatment, and progression of aortic stenosis in the community. Journal of the American College of Cardiology. 2002;40(10):1723-30.

4785. Bellanca CM, Augello E, Cantone AF, Di Mauro R, Attaguile GA, Di Giovanni V, et al. Insight into Risk Factors, Pharmacogenetics/Genomics, and Management of Adverse Drug Reactions in Elderly: A Narrative Review. Pharmaceuticals. 2023;16(11):1542.

4786. Bellanger R, Metz SG, Sausen TR, Oliveira MA. Developing an international industrial advanced pharmacy practice experience in Brazil. Currents in Pharmacy Teaching and Learning. 2021;13(10):1265-9. doi: 10.1016/j.cptl.2021.07.019.

4787. Bellanger RA, Shank TC. Continuing professional development in Texas: Survey of pharmacists' knowledge and attitudes: 2008. Journal of the American Pharmacists Association. 2010;50(3):368-74. doi: 10.1331/JAPhA.2010.09011.

4788. Bellantoni J, Clark E, Wilson J, Pendergast J, Pavon JM, White HK, et al. Implementation of a telehealth videoconference to improve hospital-to-skilled nursing care transitions: Preliminary data. Journal of the American Geriatrics Society. 2022;70(6):1828-37. doi: 10.1111/jgs.17751. PubMed PMID: 157298835. Language: English. Entry Date: 20220617. Revision Date: 20220623. Publication Type: Article.

4789. Bellegarde J, Bernard L, Chennell P, Sautou V. On-call duties in hospital pharmacies: National survey and elaboration of a training program for pharmacy residents. Annales Pharmaceutiques Françaises.

2021;79(2):142-51. doi: https://doi.org/10.1016/j.pharma.2020.09.005.

4790. Bellesoeur A, Gataa I, Jouinot A, El Mershati S, Arrondeau J, Tlemsani C, et al. 1632P_PR - Characterizing the risk of drug-drug interactions in sarcoma treated patients: Role of pharmacist integration. Annals of Oncology. 2018;29:viii753-viii4. doi: https://doi.org/10.1093/annonc/mdy424.083.

4791. Bellesoeur A, Gataa I, Jouinot A, Mershati SE, Piketty AC, Tlemsani C, et al. Prevalence of drug-drug interactions in sarcoma patients: key role of the pharmacist integration for toxicity risk management. Cancer Chemotherapy & Pharmacology. 2021;88(4):741-51. PubMed PMID: 34304283.

4792. Bellesoeur A, Thomas-Schoemann A, Allard M, Smadja D, Vidal M, Alexandre J, et al. Pharmacokinetic variability of anticoagulants in patients with cancer-associated thrombosis: Clinical consequences. Critical Reviews in Oncology/Hematology. 2018;129:102-12. doi: https://doi.org/10.1016/j.critrevonc.2018.06.015.

4793. Bellet F, Joannet B, Marsille F, Mounier G, Guy C, Beyens M-N. Médicaments et grossesse : ce que doit savoir le pharmacien d'officine. Actualités Pharmaceutiques. 2013;52(529):18-25. doi: https://doi.org/10.1016/j.actpha.2013.07.005.

4794. Bellia GF. Investigation of Chemical Differences in Medications Obtained from Diverse Sources Using Novel Spectroscopic and Statistic Approaches [Ph.D.]. England: University of Lincoln (United Kingdom); 2018.

4795. Bellingham C. Is the new contract good or bad news? Pharmaceutical Journal. 2004;273(7324):675.

4796. Bellingham C. Establishing a cross-sector pharmacy service to improve heart failure care. Pharmaceutical Journal. 2004;272(7303):741.

4797. Bellingham C, editor Community pharmacy will be valued. Pharmaceutical Journal; 2004.

4798. Bellingham C. How pharmacists can support self-care. Pharmaceutical Journal. 2005;274(7333):78.

4799. Bellingham C, editor Pharmacy practice research reviewed. Pharmaceutical Journal; 2005.

4800. Bellingham C. Older people to ask about medicines. Pharmaceutical Journal. 2006;277(7425):542-3.

4801. Belliveau PP, Rothman AL, Maday CE. Limiting vancomycin use to combat vancomycin-resistant Enterococcus faecium. American journal of health-system pharmacy. 1996;53(13):1570-5. PubMed PMID: CN-00738820.

4802. Bellman R, Mohebbi S, Nobahar N, Parizher J, Apollonio DE. An observational survey assessing the extent of PrEP and PEP furnishing in San Francisco Bay Area pharmacies. Journal of the American Pharmacists Association. 2022;62(1):370-7.e3. doi: https://doi.org/10.1016/j.japh.2021.08.001.

4803. Bello N. Use of alternative medicine by consumers in health shops in the community and in-patients in secondary healthcare settings in the north east of England [Ph.D.]. England: University of Sunderland (United Kingdom); 2014.

4804. Bello W, Pezzatti J, Berger-Gryllaki M, Rudaz S, Sadeghipour F. Development of a generic approach for monitoring leachable compounds in hospital pharmacy-prepared prefilled plastic packaging by ultrahigh-performance liquid chromatography coupled to high-resolution mass spectrometry with postcolumn infusion. Journal of Pharmaceutical and Biomedical Analysis. 2023;236:115640. doi: https://doi.org/10.1016/j.jpba.2023.115640.

4805. Bellone JM, Barner JC, Lopez DA. Postdischarge interventions by pharmacists and impact on hospital readmission rates. Journal of the American Pharmacists Association. 2012;52(3):358-62.

4806. Bellows BK, Olsen CJ, Voelker J, Wander C. Antihyperlipidemic Medication Treatment Patterns and Statin Adherence Among Patients with ASCVD in a Managed Care Plan After Release of the 2013 ACC/AHA Guideline on the Treatment of Blood Cholesterol. Journal of Managed Care & Specialty Pharmacy. 2016;22(8):892-900. PubMed PMID: WOS:000380579800001.

4807. Bellows BK, Sainski-Nguyen AM, Olsen CJ, Boklage SH, Charland S, Mitchell MP, et al. Identification of Patients with Statin Intolerance in a Managed Care Plan: A Comparison of 2 Claims-Based Algorithms. Journal of Managed Care & Specialty Pharmacy. 2017;23(9):926-34. doi: 10.18553/jmcp.2017.23.9.926. PubMed PMID: WOS:000410718800002.

4808. Belo-Kibabu S, Bottois C, Dumas S, Hubert J, Molto A, Roux C, et al. Implementation, of multidisciplinary consultations for patients with inflammatory arthritis and treated with subcutaneous biologic DMARDs: Assessment at one year and outlook. Annales Pharmaceutiques Francaises. 2023;81(2):370-9. doi: 10.1016/j.pharma.2022.08.011. PubMed PMID: WOS:000990509300001.

4809. Beloeil H, Albaladejo P. Initiatives to broaden safety concerns in anaesthetic practice: The green operating room. Best Practice & Research Clinical Anaesthesiology. 2021;35(1):83-91. doi: https://doi.org/10.1016/j.bpa.2020.07.010.

4810. Beloin-Jubinville B, Joly-Mischlich T, Dufort Rouleau E, Noiseux P, Blais L, Forget A, et al. Does hospitalization influence patients' medication adherence and community pharmacists' interventions? Annals of Pharmacotherapy. 2013;47(9):1143-52. doi: 10.1177/1060028013503123.

4811. Belouin SJ, Averill LA, Henningfield JE, Xenakis SN, Donato I, Grob CS, et al. Policy considerations that support equitable access to responsible, accountable, safe, and ethical uses of psychedelic medicines. Neuropharmacology. 2022;219. doi: 10.1016/j.neuropharm.2022.109214.

4812. Belverud S, Mogilner A, Schulder M. Intrathecal Pumps. Neurotherapeutics. 2008;5(1):114-22. doi: https://doi.org/10.1016/j.nurt.2007.10.070.

4813. Belza AC, Efta J, MacDonald N, Kenney RM, Patel N. Putting a CAP on Discharge Antimicrobial Therapy: Evaluation of a Systematic Transitions of Care Process for Patients with Community Acquired Pneumonia (CAP) and Chronic Obstructive Pulmonary Disease (COPD). Open Forum Infectious Diseases. 2023;10(Supplement 2):S540-S1. doi: https://dx.doi.org/10.1093/ofid/ofad500.1041. PubMed PMID: 643249658.

4814. Bemelmans M, Baert S, Goemaere E, Wilkinson L, Vandendyck M, van Cutsem G, et al. Community-supported models of care for people on HIV treatment in sub-Saharan Africa. Tropical Medicine and International Health. 2014;19(8):968-77. doi: 10.1111/tmi.12332.

4815. Bemt PMVD, Broek SVD, Nunen AKV, Harbers JB, Lenderink AW. Medication reconciliation performed by pharmacy technicians at the time of preoperative screening. Annals of Pharmacotherapy. 2009;43(5):868-74.

4816. Ben Abdelaziz A, Harrabi I, Rahmani S, Gaha R, Ghannem H. Circulation of pharmaceutical information among general practioners in the region of Sousse (Tunisia). Therapie. 2002;57(3):229-35.

4817. Ben Amor F, Kammoun M, Loukil T. Exact Resolution for Healthcare Facility Location Problem: A Case Study of the Specific Pharmacy of Tunisia. Contributions to Management Science2022. p. 129-38.

4818. Ben Jebara M. Essays on Biopharmaceutical Supply Chains [Ph.D.]. United States -- Ohio: The University of Toledo; 2015.

4819. Ben Mabrouk A, Larbi Ammari F, Werdani A, Jemmali N, Chelli J, Mrabet HE, et al. Parental self-medication with antibiotics in a Tunisian pediatric center. Therapies. 2022;77(4):477-85. doi: https://doi.org/10.1016/j.therap.2021.10.007.

4820. Benard R, Chipungahelo MS. Accessibility of women to health information in Tanzania A case study of Morogoro Region. Library Review. 2017;66(6-7):415-29. doi: 10.1108/lr-05-2017-0046. PubMed PMID: WOS:000414973900004.

4821. Benavides S, Huynh D, Morgan J, Briars L. Approach to the pediatric prescription in a community pharmacy. The Journal of Pediatric Pharmacology and Therapeutics. 2011;16(4):298-307.

4822. Benavides S, Kohler LA, Souffrant G. A Clinical Pharmacist's Role in Screening for Metabolic Syndrome in a Rural Pediatric Ambulatory Clinic. Journal of Rural Health. 2011;27(2):184-9. doi: 10.1111/j.1748-0361.2010.00329.x.

4823. Benavides S, Madzhidova S, Hernandez A, Le T, Palma SM, Stephen S. Establishment of Pediatric Medication Therapy Management: A Proposed Model. Pharmacy. 2016;4(1). doi: 10.3390/pharmacy4010005. PubMed PMID: WOS:000374049100005.

4824. Benavides S, Rodriguez JC, Maniscalco-Feichtl M. Pharmacist involvement in improving asthma outcomes in various healthcare settings: 1997 to present. Annals of Pharmacotherapy. 2009;43(1):85-97. doi: 10.1345/aph.1K612.

4825. Benchimol EI, Guttmann A, Mack DR, Nguyen GC, Marshall JK, Gregor JC, et al. Validation of international algorithms to identify adults with inflammatory bowel disease in health administrative data from Ontario, Canada. Journal of Clinical Epidemiology. 2014;67(8):887-96. doi: https://doi.org/10.1016/j.jclinepi.2014.02.019.

4826. Bender BG, Cvietusa P, Goodrich G, Lowe CR, Nuanes H, Shetterly S, et al. A 24-month randomized, controlled trial of an automated speech recognition program to improve adherence in pediatric asthma. Journal of allergy and clinical immunology. 2014;133(2):AB166-. doi: 10.1016/j.jaci.2013.12.600. PubMed PMID: CN-00985861.

4827. Bender BG, Cvietusa PJ, Goodrich GK, Lowe R, Nuanes HA, Rand C, et al. Pragmatic trial of health care technologies to improve adherence to pediatric asthma treatment: a randomized clinical trial. JAMA pediatrics. 2015;169(4):317-23. doi: 10.1001/jamapediatrics.2014.3280. PubMed PMID: CN-01066954.

4828. Bender BG, Hernandez Vecino RA, McGrath K, Jones S. Comparative Analysis of Persistence to Treatment among Patients with Asthma or COPD Receiving AirFluSal Forspiro or Seretide Diskus Salmeterol/Fluticasone Propionate Combination Therapy. The Journal of Allergy and Clinical Immunology: In Practice. 2016;4(5):884-9. doi: https://doi.org/10.1016/j.jaip.2016.07.006.

4829. Bendicksen L, Zuckerman DM, Avorn J, Phillips S, Kesselheim AS. The Regulatory Repercussions of Approving Muscular Dystrophy Medications on the Basis of Limited Evidence. Annals of Internal Medicine. 2023;176(9):1251-6. doi: 10.7326/M23-1073. PubMed PMID: 171988379.

4830. Bendinger GM, Baptist S, Alvarez Tapias C, McGuire E, Rados K, Nixon D, et al. A randomized study comparing MoistHer to topical lidocaine for female breast cancer survivors with dyspareunia. Cancer research. 2017;77(4). doi: 10.1158/1538-7445.SABCS16-OT3-07-04. PubMed PMID: CN-01377796.

4831. Bendtsen P, Damsgaard MT, Tolstrup JS, Ersboll AK, Holstein BE. Adolescent Alcohol Use Reflects Community-Level Alcohol Consumption Irrespective of Parental Drinking. Journal of Adolescent Health. 2013;53(3):368-73. doi: 10.1016/j.jadohealth.2013.04.021. PubMed PMID: WOS:000323602900011.

4832. Benedetto SR, Sloan AS, Duncan BS. Impact of interventions designed to increase market share and prescribing of fexofenadine at HMOs. American journal of health-system pharmacy. 2000;57(19):1778-85. PubMed PMID: CN-00354667.

4833. Benedict AJ. Information flow and knowledge sharing among patients, providers, and community pharmacists in an outpatient prescription setting [Ph.D.]. United States -- Indiana: Purdue University; 2011.

4834. Benefield S. Case study: treatment-resistant schizophrenia. Pharmaceutical Journal. 2022;308(7957). doi: 10.1211/PJ.2021.1.121281.

4835. Bener A, Saleh N, Burgut FT. Prevalence and determinants of fecal incontinence in premenopausal women in an Arabian community. Climacteric. 2008;11(5):429-35. doi: 10.1080/13697130802241519. PubMed PMID: WOS:000259086500012.

4836. Benet LZ, Goyan JE. Bioequivalence and Narrow Therapeutic Index Drugs. Pharmacotherapy: The Journal of Human Pharmacology and Drug Therapy. 1995;15(4):433-40. doi: 10.1002/j.1875-9114.1995.tb04379.x.

4837. Benetoli A, Chen TF, Schaefer M, Chaar B, Aslani P. Do pharmacists use social media for patient care? International journal of clinical pharmacy. 2017;39:364-72.

4838. Bénevent J, Lacroix I. Prise médicamenteuse et grossesse, évaluer la balance bénéfice-risque. Actualités Pharmaceutiques. 2019;58(588):21-7. doi: https://doi.org/10.1016/j.actpha.2019.05.023.

4839. Beney J, Bero L, Bond CM. Expanding the roles of outpatient pharmacists: effects on health services utilisation, costs, and patient outcomes. Cochrane Database of Systematic Reviews. 2000;(2).

4840. Beney J, Bero LA, Bond C. Expanding the roles of outpatient pharmacists: effects on health services utilisation, costs, and patient outcomes. Cochrane database of systematic reviews (Online). 2000;(3):CD000336. PubMed PMID: 33408228.

4841. Beney J, Devine EB, Chow V, Ignoffo RJ, Mitsunaga L, Shahkarami M, et al. Effect of telephone follow-up on the physical well-being dimension of quality of life in patients with cancer. Pharmacotherapy. 2002;22(10):1301-11. doi: 10.1592/phco.22.15.1301.33480. PubMed PMID: CN-00434562.

4842. Beng Yi S, Chan Pei Shan J, Lay Hong G. Medication reconciliation service in tan tock Seng hospital. International Journal of Health Care Quality Assurance. 2013;26(1):31-6.

4843. Bengtsson-Palme J, Gunnarsson L, Larsson DGJ. Can branding and price of pharmaceuticals guide informed choices towards improved pollution control during manufacturing? Journal of Cleaner Production. 2018;171:137-46. doi: https://doi.org/10.1016/j.jclepro.2017.09.247.

4844. Benhamou D, Nacry R, Journois D, Auroy Y, Durand D, Arnoux A, et al. Évaluation de l'impact auprès des professionnels de la mise en œuvre de la seconde vague du plan d'harmonisation de l'étiquetage des injectables. Annales Françaises d'Anesthésie et de Réanimation. 2012;31(1):15-22. doi: https://doi.org/10.1016/j.annfar.2011.09.005.

4845. Benhamou D, Nacry R, Journois D, Auroy Y, Durand D, Arnoux A, et al. [Second wave of the French drug harmonisation programme to prevent medication errors: overall appreciation of healthcare

professionals]. Annales Francaises d Anesthesie et de Reanimation. 2012;31(1):15-22. PubMed PMID: 22154447.

4846. Benin AL, Soe MM, Edwards JR, Bagchi S, Link-Gelles R, Schrag SJ, et al. Ecological Analysis of the Decline in Incidence Rates of COVID-19 Among Nursing Home Residents Associated with Vaccination, United States, December 2020-January 2021. Journal of the American Medical Directors Association. 2021;22(10):2009-15. doi: https://doi.org/10.1016/j.jamda.2021.08.004.

4847. Benin AL, Soe MM, Edwards JR, Bagchi S, Link-Gelles R, Schrag SJ, et al. Ecological Analysis of the Decline in Incidence Rates of COVID-19 Among Nursing Home Residents Associated with Vaccination, United States, December 2020-January 2021. Journal of the American Medical Directors Association. 2021;22(10):2009-15. doi: 10.1016/j.jamda.2021.08.004. PubMed PMID: WOS:000703174500007.

4848. Beninger P. Drug-Drug Interactions: How to Manage the Risk–A Stakeholder Approach. Clinical Therapeutics. 2023;45(2):106-16. doi: https://doi.org/10.1016/j.clinthera.2023.01.009.

4849. Benites-Meza JK, Pinedo-Castillo L, Cabanillas-Lazo M, Herrera-Añazco P, Mougenot B, Benites-Zapata VA. Ethnic disparities in out–of–pocket expense on medicines in Peru: Evidence from a nationwide survey. Public Health in Practice. 2023;6:100442. doi: https://doi.org/10.1016/j.puhip.2023.100442.

4850. Benites-Meza JK, Pinedo-Castillo L, Cabanillas-Lazo M, Herrera-Añazco P, Mougenot B, Benites-Zapata VA. Ethnic disparities in out-of-pocket expense on medicines in Peru: Evidence from a nationwide survey. Public Health in Practice. 2023;6. doi: 10.1016/j.puhip.2023.100442. PubMed PMID: WOS:001134155500001.

4851. Benitez L, Marx KR, Buhlinger KM, Clark SM, Reid JH, Rausch CR, et al. Multicenter Retrospective Evaluation of Treatment of High-Risk Acute Promyelocytic Leukemia (APL): Results from the Hematology Research Outcomes (HERO) Consortium. Blood. 2023;142:4267. doi: https://doi.org/10.1182/blood-2023-189637.

4852. Benitez Y, Forrester L, Hurst C, Turpin D. HospitalReduces Medication Errors Using DMAIC and QFD: QualityProgress; 2007.

4853. Benjamin DM. Reducing medication errors and increasing patient safety: case studies in clinical pharmacology. Journal of Clinical Pharmacology. 2003;43(7):768-83. PubMed PMID: 106721757. Language: English. Entry Date: 20040409. Revision Date: 20150711. Publication Type: Journal Article.

4854. Benjamin EJ, Larson MG, Keyes MJ, Mitchell GF, Vasan RS, Keaney Jr JF, et al. Clinical correlates and heritability of flow-mediated dilation in the community: the Framingham Heart Study. Circulation. 2004;109(5):613-9.

4855. Benjamin H, Motawi A, Smith F. Community pharmacists and primary health care in Alexandria. Journal of Social and Administrative Pharmacy. 1995;12(1):3-11.

4856. Benjamin L, Frush K, Shaw K, Shook JE, Snow SK. Pediatric Medication Safety in the Emergency Department. Pediatrics. 2018;141(3):1-8. doi: 10.1542/peds.2017-4066. PubMed PMID: 128304515. Language: English. Entry Date: 20180308. Revision Date: 20180309. Publication Type: Article.

4857. Benjamin L, Frush K, Shaw K, Shook JE, Snow SK, Wright J, et al. Pediatric medication safety in the emergency department. Pediatrics. 2018;141(3).

4858. Benjamin Sokar-Todd H, Smith F. Management of diarrhoea at community pharmacies in Alexandria. Journal of Social and Administrative Pharmacy. 2003;20(1):32-8.

4859. Benjarattanaporn P, Lindan CP, Mills S, Barclay J, Bennett A, Mugrditchian D, et al. Men with sexually transmitted diseases in Bangkok: Where do they go for treatment and why? AIDS, Supplement. 1997;11(1):S87-S95.

4860. Benkirane RR, R RA, Haimeur C, El Kettani SSS, Azzouzi AA, Alaoui AAM, et al. Incidence of adverse drug events and medication errors in intensive care units: a prospective multicenter study [corrected] [published erratum appears in J PATIENT SAF 2010 Mar;6(1):57]. Journal of Patient Safety. 2009;5(1):16-22. doi: 10.1097/PTS.0b013e3181990d51. PubMed PMID: 109850559. Language: English. Entry Date: 20090515. Revision Date: 20151008. Publication Type: Journal Article.

4861. Benkirane RR, R-Abouqal R, Haimeur CC, Ech SS, Kettani CE, Azzouzi AA, et al. Incidence of adverse drug events and medication errors in intensive care units: a prospective multicenter study. Journal of patient safety. 2009:16-22.

4862. Benmamoun Z, Fethallah W, Bouazza S, Abdo AA, Serrou D, Benchekroun H. A framework for sustainability evaluation and improvement of radiology service. Journal of Cleaner Production. 2023;401:N.PAG-N.PAG. doi: 10.1016/j.jclepro.2023.136796. PubMed PMID: 162920125.

4863. Bennani-Baïti AA, Benbouzid A, Essakalli-Hossyni L. Cervicofacial cellulitis: The impact of non-steroidal anti-inflammatory drugs. A study of 70 cases. European Annals of Otorhinolaryngology, Head and Neck Diseases. 2015;132(4):181-4. doi: https://doi.org/10.1016/j.anorl.2015.06.004.

4864. Benner JS, Kocot SL. Medicare part D: good for patients and an opportunity for pharmacists. Journal of Managed Care Pharmacy. 2009;15(1):66-70. PubMed PMID: 19125552.

4865. Bennett A. Management of Cardiovascular Disorders and the Pharmacist's Role: Heart Failure. In: Babar Z-U-D, editor. Encyclopedia of Pharmacy Practice and Clinical Pharmacy. Oxford: Elsevier; 2019. p. 188-209.

4866. Bennett A, Avancena ALV, Wegbreit J, Cotter C, Roberts K, Gosling R. Engaging the private sector in malaria surveillance: a review of strategies and recommendations for elimination settings. Malaria Journal. 2017;16. doi: 10.1186/s12936-017-1901-1. PubMed PMID: WOS:000403852100001.

4867. Bennett AS, Elliott L. Naloxone's role in the national opioid crisis—past struggles, current efforts, and future opportunities. Translational Research. 2021;234:43-57. doi: https://doi.org/10.1016/j.trsl.2021.03.001.

4868. Bennett AS, Elliott L. Naloxone's role in the national opioid crisis- past struggles, current efforts, and future opportunities. Translational Research. 2021;234:43-57. doi: 10.1016/j.trsl.2021.03.001. PubMed PMID: WOS:000661683600004.

4869. Bennett CL, Nagai S, Bennett AC, Hoque S, Nabhan C, Schoen MW, et al. The First 2 Years of Biosimilar Epoetin for Cancer and Chemotherapy-Induced Anemia in the U.S.: A Review from the Southern Network on Adverse Reactions. Oncologist. 2021;26(8):e1418-e26. doi: 10.1002/onco.13713.

4870. Bennett D, Yin W. The Market for High-Quality Medicine: Retail Chain Entry and Drug Quality in India. Review of Economics and Statistics. 2019;101(1):76-90. doi: 10.1162/rest_a_00758.

4871. Bennett F, Ferner R, Sofat R. Overprescribing and rational therapeutics: Barriers to change and opportunities to improve. British Journal of Clinical Pharmacology. 2021;87(1):34-8. PubMed PMID: 32216108.

4872. Bennett G, Taing MW, Hattingh HL, La Caze A. Pharmacists' perceived responsibility for patient care when there is a risk of misadventure: a qualitative study. International Journal of Pharmacy Practice.

2020;28(6):599-607. doi: 10.1111/ijpp.12580.

4873. Bennett J, Harper-Femson LA, Tone J, Rajmohamed Y. Improving medication administration systems: an evaluation study. Canadian Nurse. 2006;102(8):35-9. PubMed PMID: 17094367.

4874. Bennett M, Goode JVR. Recognition of community-based pharmacist practitioners: Essential health care providers. Journal of the American Pharmacists Association. 2016;56(5):580-3. doi: 10.1016/j.japh.2016.04.566. PubMed PMID: WOS:000389758700018.

4875. Bennett P, Noble S, Johnston S, Jones D, Hunter R. COVID-19 confessions: a qualitative exploration of healthcare workers experiences of working with COVID-19. Bmj Open. 2020;10(12). doi: 10.1136/bmjopen-2020-043949. PubMed PMID: WOS:000600231500035.

4876. Bennett PN, Brown MJ. Clinical Pharmacology E-Book: With STUDENTCONSULT Access: Elsevier health sciences; 2007.

4877. Bennett RW. Components of a Pharmaceutical Care Certificate Program. Journal of the American Pharmaceutical Association (1996). 1998;38(1):76-81. doi: https://doi.org/10.1016/S1086-5802(16)30287-X.

4878. Bennett S. Canadian internet pharmacies and the USA: Technological and regulatory market change. Innovation, Science, Environment: Canadian Policies and Performance, 2007/20082007. p. 118-36.

4879. Bennett S. Companion setting. Journal of Complementary Medicine. 2007;6(1):37-40. PubMed PMID: WOS:000257655600018.

4880. Bennett SA, Ruisinger JF, Prohaska ES, Steele KM, Melton BL. Assessing pet owner and veterinarian perceptions of need for veterinary compounding services in a community pharmacy setting. Pharmacy Practice (Granada). 2018;16(3).

4881. Bennett-Levy J, Singer J, Rotumah D, Bernays S, Edwards D. From Digital Mental Health to Digital Social and Emotional Wellbeing: How Indigenous Community-Based Participatory Research Influenced the Australian Government's Digital Mental Health Agenda. International Journal of Environmental Research and Public Health. 2021;18(18). doi: 10.3390/ijerph18189757. PubMed PMID: WOS:000699793000001.

4882. Bennin F, Rother H-A. “But it's just paracetamol”: Caregivers’ ability to administer over-the-counter painkillers to children with the information provided. Patient Education and Counseling. 2015;98(3):331-7. doi: https://doi.org/10.1016/j.pec.2014.11.025.

4883. Benny Gerard N. Measuring Multimorbidity, Classifying Potentially Inappropriate Medications and Investigating Polypharmacy in Recipients of Medication Reviews and Pharmacy Dispensing Services Conducted by Ontario Community Pharmacists [M.Sc.]. Canada -- Ontario, CA: University of Toronto (Canada); 2021.

4884. Benoit RM, Naslund MJ, Cohen JK. Complications after prostate brachytherapy in the medicare population. Urology. 2000;55(1):91-6. doi: 10.1016/s0090-4295(99)00122-3. PubMed PMID: WOS:000084799500020.

4885. Benoit RM, Naslund MJ, Cohen JK. Complications after radical retropubic prostatectomy in the Medicare population. Urology. 2000;56(1):116-20. doi: 10.1016/s0090-4295(00)00539-2. PubMed PMID: WOS:000087900600033.

4886. Benrimoj SI, Feletto E, Gastelurrutia MA, Martinez Martinez F, Faus MJ. A holistic and integrated approach to implementing cognitive pharmaceutical services. Ars Pharmaceutica. 2010;51(2):69-88.

4887. Benrimoj SI, Frommer MS. Community pharmacy in Australia. Australian Health Review. 2004;28(2):238-46. PubMed PMID: 106540878. Language: English. Entry Date: 20051118. Revision Date: 20150711. Publication Type: Journal Article.

4888. Benrimoj SI, Gilbert A, Quintrell N, De Almeida Neto AC. Non-prescription medicines: A process for standards development and testing in community pharmacy. Pharmacy World and Science. 2007;29(4):386-94. doi: 10.1007/s11096-007-9086-2.

4889. Benrimoj SI, Gilbert AL, De Almeida Neto AC, Kelly F. National implementation of standards of practice for non-prescription medicines in Australia. Pharmacy World and Science. 2009;31(2):230-7. doi: 10.1007/s11096-008-9266-8.

4890. Benrimoj SI, Gilbert AL, Neto ACD, Kelly F. National implementation of standards of practice for non-prescription medicines in Australia. Pharmacy World & Science. 2009;31(2):230-7. doi: 10.1007/s11096-008-9266-8. PubMed PMID: WOS:000264536000015.

4891. Benrimoj SI, Langford JH, Berry G, Collins D, Lauchlan R, Stewart K, et al. Economic impact of increased clinical intervention rates in community pharmacy. A randomised trial of the effect of education and a professional allowance. PharmacoEconomics. 2000;18(5):459-68. doi: 10.2165/00019053-200018050-00005. PubMed PMID: CN-00330135.

4892. Benrimoj SI, Langford JH, Berry G, Collins D, Lauchlan R, Stewart K, et al. Economic impact of increased clinical intervention rates in community pharmacy - A randomised trial of the effect of education and a professional allowance. Pharmacoeconomics. 2000;18(5):459-68. doi: 10.2165/00019053-200018050-00005. PubMed PMID: WOS:000165115600005.

4893. Benrimoj SI, Langford JH, Berry G, Collins D, Lauchlan R, Stewart K, et al. Clinical intervention rates in community pharmacy: a randomised trial of the effect of education and a professional allowance. International journal of pharmacy practice. 2003;11(2):71-80. PubMed PMID: CN-00474112.

4894. Benrimoj SI, Langford JH, Berry G, Collins D, Lauchlan R, Stewart K, et al. Clinical significance of clinical interventions in community pharmacy: a randomised trial of the effect of education and a professional allowance. International journal of pharmacy practice. 2003;11(2):81-7. PubMed PMID: CN-00474113.

4895. Benrimoj SI, Langford JH, Ward PR, Berry G, Collins D, Lauchlan R, et al. Clinical intervention rates in community pharmacy: a randomised trial of the effect of education and a professional allowance. International Journal of Pharmacy Practice. 2003;11(2):71-80.

4896. Benrimoj SI, Peacocke G, Whitehead P, Kopecny E, Ward PR, Emerson L. Cognitive pharmaceutical services in emerging health care systems - New patient medication management and concordance services in community pharmacy. Journal of Social and Administrative Pharmacy. 2003;20(1):2-12.

4897. Benrimoj SI, Roberts AS. Providing patient in community in Australia. Annals of Pharmacotherapy. 2005;39(11):1911-7. doi: 10.1345/aph.1G165.

4898. Benrimoj SI, Roberts AS. Providing patient care in community pharmacies in Australia. Annals of Pharmacotherapy. 2005;39(11):1911-7. doi: 10.1345/aph.1G165. PubMed PMID: WOS:000232858300018.

4899. Benrimoj SI, Werner JB, Raffaele C, Roberts AS. A system for monitoring quality standards in the provision of non-prescription medicines from Australian community pharmacies. Pharmacy World and Science. 2008;30(2):147-53. doi: 10.1007/s11096-007-9162-7.

4900. Benrimoj SI, Werner JB, Raffaele C, Roberts AS, Costa FA. Monitoring quality standards in the provision of non-prescription medicines from Australian Community Pharmacies: Results of a national programme. Quality and Safety in Health Care. 2007;16(5):354-8. doi: 10.1136/qshc.2006.019463.

4901. Benrimoj SI, Werner JB, Raffaele C, Roberts AS, Costa FA. Monitoring quality standards in the provision of non-prescription medicines from Australian Community Pharmacies: Results of a national programme (Quality and Safety in Health Care (2007) 16, (354-358)). Quality and Safety in Health Care. 2007;16(6):461. doi: 10.1136/qshc.2006.019463corr1.

4902. Benrimoj SI, Werner JB, Raffaele C, Roberts AS, Costa FA. Monitoring quality standards in the provision of nonprescription medicines from Australian Community Pharmacies: results of a national programme. Quality & Safety in Health Care. 2007;16(5):354-8. doi: 10.1136/qshc.2006.019463. PubMed PMID: WOS:000249888300007.

4903. Benseler A, McDermott CD. Use of Prophylactic Antibiotics to Prevent Urinary Tract Infection after Urodynamic Testing and Cystoscopy in Women: A Practice Pattern Study. Gynecologic and Obstetric Investigation. 2022;87(2):116-23. doi: 10.1159/000524266. PubMed PMID: WOS:000877413600005.

4904. Bensken WP, Navale SM, Andrew AS, Jobst BC, Sajatovic M, Koroukian SM. Delays and disparities in diagnosis for adults with epilepsy: Findings from U.S. Medicaid data. Epilepsy Research. 2020;166:106406. doi: https://doi.org/10.1016/j.eplepsyres.2020.106406.

4905. Benson AB, Boehmer L, Mi X, Kocherginsky M, Shivakumar L, Trosman JR, et al. Resource and Reimbursement Barriers to Comprehensive Cancer Care Delivery: An Analysis of Association of Community Cancer Centers Survey Data. JCO Oncology Practice. 2023;19(3):E428-E38. doi: 10.1200/OP.22.00417.

4906. Benson B, Justo JA, Winders HR, Kohn J, Al-Hasan M, Bookstaver PB. Effect of pharmacist led weekend antimicrobial stewardship on vancomycin utilization. JACCP journal of the american college of clinical pharmacy. 2020;3(1):235-6. doi: 10.1002/jac5.1204. PubMed PMID: CN-02120600.

4907. Benson H, Lucas C, Kmet W, Benrimoj SI, Williams K. Pharmacists in general practice: a focus on drug-related problems. International Journal of Clinical Pharmacy. 2018;40(3):566-72. doi: 10.1007/s11096-018-0617-9. PubMed PMID: 129929197. Language: English. Entry Date: 20181011. Revision Date: 20190603. Publication Type: journal article.

4908. Benson M, Dewey M, Friesner D. Pharmacist-led urine culture follow-ups in a rural emergency department. Journal of the American Pharmacists Association. 2023;63(4 Supplement):S39-S42.e1. doi: https://dx.doi.org/10.1016/j.japh.2022.08.019. PubMed PMID: 2020220568.

4909. Benson MJ. Sustainability of postabortion care in Peru [Dr.P.H.]. United States -- North Carolina: The University of North Carolina at Chapel Hill; 2003.

4910. Bentley JP. An examination of consumer judgments of the level of inherent burden associated with the medication consumption experience and generated by features of drug therapy [Ph.D.]. United States -- Mississippi: The University of Mississippi; 1998.

4911. Bentley JP, Smith MC, Banahan BF, Frate DA, Parks BR. Quality of life assessment by community pharmacists: an exploratory study. Quality of Life Research. 1998;7(2):175-86. doi: 10.1023/a:1008813711611. PubMed PMID: WOS:000072036900010.

4912. Bentley JP, Smith MC, Banahan Iii BF, Frate DA, Parks BR. Barriers to the assessment of health-related quality of life in community pharmacies: A segmentation analysis. Journal of Research in Pharmaceutical Economics. 2000;10(1):29-50. doi: 10.1300/J063v10n01_04.

4913. Bentley JP, Stroup LJ, Wilkin NE, Bouldin AS. Patient evaluations of pharmacist performance with variations in attire and communication levels. Journal of the American Pharmacists Association : JAPhA. 2005;45(5):600-7. doi: 10.1331/1544345055001337. PubMed PMID: CN-00532030.

4914. Bentley JR, Smith MC, Banahan Iii BF, Frate DA, Parks BR. Quality of life assessment by community pharmacists: An exploratory study. Quality of Life Research. 1998;7(2):175-86. doi: 10.1023/A:1008813711611.

4915. Bentzien J, Bharadwaj R, Thompson DC. Crowdsourcing in pharma: a strategic framework. Drug Discovery Today. 2015;20(7):874-83. doi: 10.1016/j.drudis.2015.01.011.

4916. Benzi G. Is there a rationale for the use of creatine either as nutritional supplementation or drug administration in humans participating in a sport? Pharmacological Research. 2000;41(3):255-64.

4917. Beorlegui Martincorena B, Ortega Eslava A, Aquerreta González I, Aldaz Pastor A, Lacasa Arregui C, Idoate García A, et al. Drug information made available to the population via Internet from a Pharmacy Department. Farmacia Hospitalaria. 2003;27(6):353-9.

4918. Bepari AK, Rabbi G, Shaon HR, Khan SI, Zahid ZI, Dalal K, et al. Factors Driving Antimicrobial Resistance in Rural Bangladesh: A Cross-Sectional Study on Antibiotic Use-Related Knowledge, Attitude, and Practice Among Unqualified Village Medical Practitioners and Pharmacy Shopkeepers. Advances in Therapy. 2023;40(8):3478-94. doi: 10.1007/s12325-023-02547-5.

4919. Bepko Jr RJ, Moore JR, Coleman JR. Implementation of a pharmacy automation system (robotics) to ensure medication safety at Norwalk hospital. Quality Management in Healthcare. 2009;18(2):103-14.

4920. Bepko RJ. Implementation of a pharmacy automation system (Robotics) to ensure medication safety at Norwalk Hospital.

4921. Bepko RJ, Jr., Moore JR, Coleman JR. Implementation of a pharmacy automation system (robotics) to ensure medication safety at Norwalk hospital. Quality Management in Health Care. 2009;18(2):103-14. doi: 10.1097/QMH.0b013e3181a02771. PubMed PMID: 105519159. Language: English. Entry Date: 20090605. Revision Date: 20200708. Publication Type: Journal Article.

4922. Beran M, Asche SE, Bergdall AR, Crabtree B, Green BB, Groen SE, et al. Key components of success in a randomized trial of blood pressure telemonitoring with medication therapy management pharmacists. Journal of the American Pharmacists Association : JAPhA. 2018;58(6):614-21. doi: 10.1016/j.japh.2018.07.001. PubMed PMID: CN-01630261.

4923. Beran M, Asche SE, Bergdall AR, Crabtree B, Green BB, Groen SE, et al. Key components of success in a randomized trial of blood pressure telemonitoring with medication therapy management pharmacists. Journal of the American Pharmacists Association: JAPhA. 2018;58(6):614-21. PubMed PMID: 30077564.

4924. Beran RG. Generic Substitution and Advice to Patients –Potential Legal Medicine Consequences – From A (Australia) to U (United Kingdom or United States of America). Seizure. 2021;91:515-9. doi: https://doi.org/10.1016/j.seizure.2021.03.016.

4925. Bérard G, Marcotte N, Michel M-C, Farand P, Deschenes L, Froment D, et al. Descriptive Analysis of Azacitidine Use in Four Adult University Teaching Hospitals in Quebec, Canada. Blood. 2015;126(23):5244. doi: https://doi.org/10.1182/blood.V126.23.5244.5244.

4926. Berard LD, Pockett SA, Roscoe RS, Siemens RL. A Coloured Pen Needle Education System Improves Insulin Site Rotation Habits: results of a Randomized Study. Diabetes therapy. 2020;11(12):2979-91. doi:

10.1007/s13300-020-00939-2. PubMed PMID: CN-02190728.

4927. Berardi A, Perinelli DR, Merchant HA, Bisharat L, Basheti IA, Bonacucina G, et al. Hand sanitisers amid CoViD-19: A critical review of alcohol-based products on the market and formulation approaches to respond to increasing demand. International Journal of Pharmaceutics. 2020;584:119431. doi: https://doi.org/10.1016/j.ijpharm.2020.119431.

4928. Berardi RR. Safety and Tolerability of Tegaserod in Irritable Bowel Syndrome Management. Journal of the American Pharmacists Association. 2004;44(1):41-51. doi: https://doi.org/10.1331/154434504322713228.

4929. Berassa MS, Worku SG. Availability of Essential Medicines at a University Hospital in Addis Ababa, a cross-sectional study. Ethiopian Journal of Health Development. 2023;37(3). PubMed PMID: WOS:001162775100001.

4930. Berbatis C, Sunderland V, Mills C, Bulsara M. National pharmacy database project. Perth: Curtin University of Technology. 2003.

4931. Berbatis CG. Methadone programs, harm reduction, chronic diseases and hospital pharmacists. Australian Journal of Hospital Pharmacy. 1998;28(1):41-4. doi: 10.1002/jppr199828141.

4932. Berbatis CG, Bruce Sunderland V. Research methods for pharmacy practice development. Translational Research for Primary Healthcare2012. p. 273-86.

4933. Berbatis CG, Sunderland VB. National evaluation of pharmacy services in methadone maintenance treatment. Australian Journal of Hospital Pharmacy. 2001;31(4):309. doi: 10.1002/jppr2001314309.

4934. Berbatis CG, Sunderland VB, Joyce A, Bulsara M, Mills C. Characteristics of Australia's community pharmacies: National Pharmacy Database project. International journal of pharmacy practice. 2007;15(4):265-71.

4935. Berce PC, Bernstein RS, MacKinnon GE, Sorum S, Martin E, MacKinnon KJ, et al. Immunizations at Wisconsin Pharmacies: Results of a statewide vaccine registry analysis and pharmacist survey. Vaccine. 2020;38(28):4448-56. doi: 10.1016/j.vaccine.2020.04.043.

4936. Berdine H, Dougherty T, Ference J, Karpa K, Klootwyk J, Kozminski M, et al. The pharmacists' role in the Patient-Centered Medical Home (PCMH): a white paper created by the Health Policy Committee of the Pennsylvania Pharmacists Association (PPA). Annals of Pharmacotherapy. 2012;46(5):723-50. PubMed PMID: 22535839.

4937. Berdine HJ, O'Neil CK. Development and implementation of a pharmacist-managed university-based wellness center. Journal of the American Pharmacists Association. 2007;47(3):390-7. doi: 10.1331/JAPhA.2007.06077.

4938. Berdine HJ, O'Neil CK. Development and implementation of a pharmacist-managed university-based wellness center. Journal of the American Pharmacists Association. 2007;47(3):390-7. doi: https://doi.org/10.1331/JAPhA.2007.06077.

4939. Berdot S, Boussadi A, Vilfaillot A, Depoisson M, Guihaire C, Durieux P, et al. Integration of a Commercial Barcode-Assisted Medication Dispensing System in a Teaching Hospital. Applied clinical informatics. 2019;10(4):615-24. doi: 10.1055/s-0039-1694749. PubMed PMID: CN-02228660.

4940. Berdot S, Korb-Savoldelli V, Jaccoulet E, Zaugg V, Prognon P, Lê LMM, et al. A centralized

automated-dispensing system in a French teaching hospital: return on investment and quality improvement. International Journal for Quality in Health Care. 2019;31(3):219-24. doi: 10.1093/intqhc/mzy152. PubMed PMID: 135933276. Language: English. Entry Date: 20190818. Revision Date: 20200331. Publication Type: journal article. Journal Subset: Biomedical.

4941. Berdot S, Roudot M, Schramm C, Katsahian S, Durieux P, Sabatier B. Interventions to reduce nurses' medication administration errors in inpatient settings: A systematic review and meta-analysis. International Journal of Nursing Studies. 2016;53:342-50. doi: 10.1016/j.ijnurstu.2015.08.012. PubMed PMID: 111344704. Language: English. Entry Date: 20151218. Revision Date: 20151218. Publication Type: Article.

4942. Berdot S, Sabatier B, Gillaizeau F, Caruba T, Prognon P, Durieux P. Evaluation of drug administration errors in a teaching hospital. BMC Health Services Research. 2012;12(1):60-. doi: 10.1186/1472-6963-12-60. PubMed PMID: 104493398. Language: English. Entry Date: 20140321. Revision Date: 20211028. Publication Type: journal article.

4943. Berenbrok LA, Ciemniecki L, Cremeans AA, Albright R, Mormer E. Pharmacist competencies for over-the-counter hearing aids: A Delphi study. Journal of the American Pharmacists Association. 2021;61(4):e255-e62. doi: 10.1016/j.japh.2021.01.039.

4944. Berenbrok LA, Gabriel N, Coley KC, Hernandez I. Evaluation of Frequency of Encounters With Primary Care Physicians vs Visits to Community Pharmacies Among Medicare Beneficiaries. Jama Network Open. 2020;3(7). doi: 10.1001/jamanetworkopen.2020.9132. PubMed PMID: WOS:000552379200002.

4945. Berenbrok LA, Gessler C, Kirisci L, Herrera-Restrepo O, Coley KC. Impact of pharmacist motivational interviewing on hepatitis B vaccination in adults with diabetes. Journal of the American Pharmacists Association. 2022. doi: 10.1016/j.japh.2022.08.013.

4946. Berenbrok LA, Gessler C, Kirisci L, Herrera-Restrepo O, Coley KC. Impact of pharmacist motivational interviewing on hepatitis B vaccination in adults with diabetes. Journal of the American Pharmacists Association. 2023;63(1):66-73. e1.

4947. Berenbrok LA, Renner HM, McGivney MAS, Coley KC. A conceptual value-based incentivization model of adult immunization for community pharmacists. Journal of the American Pharmacists Association. 2020;60(6):835-42. doi: 10.1016/j.japh.2020.04.023. PubMed PMID: WOS:000585761700077.

4948. Berendt RT, Munson EJ. Detection of Chiral Defects in Crystalline Organic Solids Using Solid-State NMR Spectroscopy. Journal of Pharmaceutical Sciences. 2011;100(5):1879-91. doi: https://doi.org/10.1002/jps.22431.

4949. Berenguer B, La Casa C, de La Matta M, Martin-Calero M. Pharmaceutical care: past, present and future. Current pharmaceutical design. 2004;10(31):3931-46.

4950. Berenguer B, La Casa C, de la Matta MJ, Martín-Calero MJ. Pharmaceutical care: Past, present and future. Current Pharmaceutical Design. 2004;10(31):3931-46. doi: 10.2174/1381612043382521.

4951. Berenguer B, La Casa L, de La Matta MJ, Martin-Calero MJ. Pharmaceutical care: Past, present and future. Current Pharmaceutical Design. 2004;10(31):3931-46. doi: 10.2174/1381612043382521. PubMed PMID: WOS:000225389400009.

4952. Berenholtz SM, Dorman T, Ngo K, Pronovost PJ. Qualitative review of intensive care unit quality indicators. Journal of critical care. 2002;17(1):1-12.

4953. Berenson RA, Hammons T, Gans DN, Zuckerman S, Merrell K, Underwood WS, et al. A house is not

a home: keeping patients at the center of practice redesign. Health Affairs. 2008;27(5):1219-30.

4954. Bereznicki B, Peterson G, Jackson S, Haydn Walters E, Deboos I, Hintz P. Perceived feasibility of a community pharmacy-based asthma intervention: a qualitative follow-up study. Journal of clinical pharmacy and therapeutics. 2011;36(3):348-55. PubMed PMID: CN-00794984.

4955. Bereznicki B, Peterson G, Jackson S, Walters EH, DeBoos I, Hintz P. Perceived feasibility of a community pharmacy-based asthma intervention: a qualitative follow-up study. Journal of Clinical Pharmacy and Therapeutics. 2011;36(3):348-55. doi: 10.1111/j.1365-2710.2010.01187.x. PubMed PMID: WOS:000290313200010.

4956. Bereznicki B, Peterson G, Jackson S, Walters EH, Gee P. The sustainability of a community pharmacy intervention to improve the quality use of asthma medication. Journal of clinical pharmacy and therapeutics. 2011;36(2):144-51. doi: 10.1111/j.1365-2710.2010.01165.x. PubMed PMID: CN-02118581.

4957. Bereznicki BJ, Peterson G, Jackson S, Walters EH, George J, Stewart K, et al. Uptake and effectiveness of a community pharmacy intervention programme to improve asthma management. Journal of clinical pharmacy and therapeutics. 2013;38(3):212-8. doi: 10.1111/jcpt.12017. PubMed PMID: CN-00861150.

4958. Bereznicki BJ, Peterson GM, Jackson SL, Walters EH, Fitzmaurice KD, Gee PR. Data-mining of medication records to improve asthma management. Medical journal of Australia. 2008;189(1):21-5. doi: 10.5694/j.1326-5377.2008.tb01889.x. PubMed PMID: CN-00649517.

4959. Bereznicki BJ, Peterson GM, Jackson SL, Walters H, Fitzmaurice K, Gee P. Pharmacist-initiated general practitioner referral of patients with suboptimal asthma management. Pharmacy world & science : PWS. 2008;30(6):869-75. doi: 10.1007/s11096-008-9242-3. PubMed PMID: CN-00667644.

4960. Bereznicki BJ, Peterson GM, Jackson SL, Walters H, Hardley FL, Gavagna GA, et al. Describing drivers of and barriers to persistence with tiotropium in patients with chronic obstructive pulmonary disease: A mixed-methods approach. International Journal of Pharmacy Practice. 2015;23(2):154-7. doi: 10.1111/ijpp.12129.

4961. Bereznicki LRE, Jackson SL, Peterson GM. Supervised patient self-testing of warfarin therapy using an online system. Journal of Medical Internet Research. 2013;15(7). doi: 10.2196/jmir.2255.

4962. Berg CJ. Vape shop location and marketing in the context of the Food and Drug Administration regulation. Public Health. 2018;165:142-5. doi: 10.1016/j.puhe.2018.09.002. PubMed PMID: WOS:000454153300020.

4963. Berg CJ, Barker DC, Sussman S, Getachew B, Pulvers K, Wagener TL, et al. Vape Shop Owners/Managers' Opinions About FDA Regulation of E-Cigarettes. Nicotine & Tobacco Research. 2021;23(3):535-42. doi: 10.1093/ntr/ntaa138. PubMed PMID: WOS:000637321000016.

4964. Berg CJ, Romm KF, Pannell A, Sridharan P, Sapra T, Rajamahanty A, et al. Cannabis retailer marketing strategies and regulatory compliance: A surveillance study of retailers in 5 US cities. Addictive Behaviors. 2023;143. doi: 10.1016/j.addbeh.2023.107696. PubMed PMID: WOS:000965950900001.

4965. Berg DD, Divakaran S, Stern RM, Warner LN. Fostering meaning in residency to curb the epidemic of resident burnout: recommendations from four chief medical residents. Academic medicine: journal of the Association of American Medical Colleges. 2019;94(11):1675.

4966. Berg J. An evaluation of a self-management program for adults with asthma [Ph.D.]. United States -- Pennsylvania: University of Pittsburgh; 1995.

4967. Berg KM, Arnsten JH. Practical and conceptual challenges in measuring antiretroviral adherence. Journal of acquired immune deficiency syndromes (1999). 2006;43(Suppl 1):S79.

4968. Berge KH, Dillon KR, Sikkink KM, Taylor TK, Lanier WL. Diversion of Drugs Within Health Care Facilities, a Multiple-Victim Crime: Patterns of Diversion, Scope, Consequences, Detection, and Prevention. Mayo Clinic Proceedings. 2012;87(7):674-82. doi: https://doi.org/10.1016/j.mayocp.2012.03.013.

4969. Bergen DC. Reducing the risk of epilepsy: using the pharmacy for more than drug dispensing. Neurology. 2008;71(20):1564-5. PubMed PMID: 18843098.

4970. Bergene EH, Holst L, Rø TB, Steinsbekk A. Considering formulation characteristics when prescribing and dispensing medicinal products for children: a qualitative study among GPs and pharmacists. Family Practice. 2019;36(3):351-6. doi: 10.1093/fampra/cmy086. PubMed PMID: 136673249. Language: English. Entry Date: 20200111. Revision Date: 20200601. Publication Type: journal article.

4971. Bergenstal RM, Wysham C, MacConell L, Malloy J, Walsh B, Yan P, et al. Efficacy and safety of exenatide once weekly versus sitagliptin or pioglitazone as an adjunct to metformin for treatment of type 2 diabetes (DURATION-2): a randomised trial. The Lancet. 2010;376(9739):431-9.

4972. Berger A, Dukes E, Smith M, Hagiwara M, Seifeldin R, Oster G. Use of oral and transdermal opioids among patients with metastatic cancer during the last year of life. Journal of Pain and Symptom Management. 2003;26(2):723-30. doi: 10.1016/s0885-3924(03)00255-0. PubMed PMID: WOS:000184762400008.

4973. Berger A, Dukes EM, Oster G. Clinical characteristics and economic costs of patients with painful neuropathic disorders. The Journal of Pain. 2004;5(3):143-9.

4974. Berger A, Sadosky A, Dukes EM, Edelsberg J, Zlateva G, Oster G. Patterns of Healthcare Utilization and Cost in Patients With Newly Diagnosed Fibromyalgia. American Journal of Managed Care. 2010;16(5):S126-S37. PubMed PMID: WOS:000286877000003.

4975. Berger BA. Patient-centered care: It's about time. American Journal of Pharmaceutical Education. 2009;73(5). doi: 10.5688/aj730591.

4976. Berger BA, Hudmon KS. Readiness for change: implications for patient care. Journal of the American Pharmaceutical Association (Washington,DC : 1996). 1997;NS37(3):321-9. doi: 10.1016/s1086-5802(16)30218-2.

4977. Berger BA, Liang H, Hudmon KS. Evaluation of software-based telephone counseling to enhance medication persistency among patients with multiple sclerosis. Journal of the American Pharmacists Association : JAPhA. 2005;45(4):466-72. doi: 10.1331/1544345054475469. PubMed PMID: CN-01706031.

4978. Berger FA, van der Sijs H, van Gelder T, van den Bemt P. The use of a clinical decision support tool to assess the risk of QT drug-drug interactions in community pharmacies. Therapeutic Advances in Drug Safety. 2021;12. doi: 10.1177/2042098621996098. PubMed PMID: WOS:000623484200001.

4979. Berger FA, van der Sijs H, van Gelder T, van den Bemt PMLA. The use of a clinical decision support tool to assess the risk of QT drug–drug interactions in community pharmacies. Therapeutic Advances in Drug Safety. 2021;12. doi: 10.1177/2042098621996098.

4980. Berger J, Barbalat MJ, Clement VP, Genton B, Bugnon O. Multidisciplinary Collaboration between a Community Pharmacy and a Travel Clinic in a Swiss University Primary Care and Public Health Centre. Pharmacy. 2018;6(4). doi: 10.3390/pharmacy6040126. PubMed PMID: WOS:000455628300024.

4981. Berger J, Bawab N, De Mooij J, Sutter Widmer D, Szilas N, De Vriese C, et al. An open randomized controlled study comparing an online text-based scenario and a serious game by Belgian and Swiss pharmacy students. Currents in pharmacy teaching & learning. 2018;10(3):267-76. doi: 10.1016/j.cptl.2017.11.002. PubMed PMID: CN-01450150.

4982. Berger J, Bourdin A, Pires F, Backes C, Perraudin C, Bugnon O. Improving patient access to hepatitis C antiviral medicines in Switzerland: Understanding the financial risks for community pharmacies. Journal of Evaluation in Clinical Practice. 2019;25(3):476-81. doi: 10.1111/jep.13081.

4983. Berger JE. Solving the obstacles to retail pharmacy as part of the healthcare solution. American Journal of Pharmacy Benefits. 2013;5(5).

4984. Berger JE. The retail pharmacy as a means to help address cost as part of the triple aim. American Journal of Pharmacy Benefits. 2013;5(4).

4985. Berger JE. The role of the pharmacist and retail pharmacy in addressing the quality challenge in healthcare. American Journal of Pharmacy Benefits. 2013;5(3).

4986. Berger JE. The role of the retail pharmacy in the new healthcare environment. American Journal of Pharmacy Benefits. 2013;5(2):52-3.

4987. Berger JS, Ashton V, Laliberté F, Germain G, Bookhart B, Lejeune D, et al. Healthcare Resource Utilization and Costs of Rivaroxaban Versus Warfarin Among Non-valvular Atrial Fibrillation (NVAF) Patients with Diabetes in a US Population. Advances in Therapy. 2023;40(3):1224-41. doi: 10.1007/s12325-022-02422-9. PubMed PMID: WOS:000915838100001.

4988. Berger JS, Laliberte F, Kharat A, Lejeune D, Moore KT, Jung Y, et al. Effectiveness, safety, and healthcare costs associated with rivaroxaban versus warfarin among venous thromboembolism patients with obesity: a real-world study in the United States. Journal of Thrombosis and Thrombolysis. 2022;54(3):438-48. doi: 10.1007/s11239-022-02661-1. PubMed PMID: WOS:000795490000001.

4989. Berger K. Pharmacists tackle opioid crisis with innovative projects two pharmacists discuss projects integrating opioid education and management into community pharmacy settings. Drug Topics. 2021;165(1):20-1.

4990. Berger K, Eickhoff C, Schulz M. Counselling quality in community pharmacies: Implementation of the pseudo customer methodology in Germany. Journal of Clinical Pharmacy and Therapeutics. 2005;30(1):45-57. doi: 10.1111/j.1365-2710.2004.00611.x.

4991. Berger K, Griese N. [Pharmaceutical consultation: the patient is the center]. Pharmazie in Unserer Zeit. 2012;41(4):276-82. PubMed PMID: 22763997.

4992. Berger ML, Mamdani M, Atkins D, Johnson ML. Good research practices for comparative effectiveness research: defining, reporting and interpreting nonrandomized studies of treatment effects using secondary data sources: the ISPOR Good Research Practices for Retrospective Database Analysis Task Force Report—Part I. Value in health. 2009;12(8):1044-52.

4993. Berger RG, Kichak J. Computerized physician order entry: helpful or harmful? Journal of the American Medical Informatics Association. 2004;11(2):100-3.

4994. Berger RL, Hsu JC. Bioequivalence trials, intersection-union tests and equivalence confidence sets. Statistical Science. 1996;11(4):283-319.

4995. Berger S, Hilgarth H, Fischer A, Remane Y, Schmitt J, Knoth H. Scoring tool to identify patients at increased risk for drug-related problems: Results of a point prevalence study at hospital admission. Deutsche Medizinische Wochenschrift. 2023;148(23):E113-E9. doi: https://dx.doi.org/10.1055/a-2161-2655. PubMed PMID: 2028162254.

4996. Bergheim S, Jacobsen CD, Clausen F, Straand J. [Home visits by a pharmacist after discharge from hospital]. Tidsskrift for Den Norske Laegeforening. 2008;128(5):567-9. PubMed PMID: 18311200.

4997. Bergin MJ. Discussion Paper on Achieving the Directions Established in the National Quality and Safety Framework. Pharmacy Guild of Australia. 2001.

4998. Bergk V, Gasse C, Rothenbacher D, Loew M, Brenner H, Haefeli WE. Drug interactions in primary care: Impact of a new algorithm on risk determination. Clinical Pharmacology & Therapeutics. 2004;76(1):85-96. doi: 10.1016/j.clpt.2004.02.009. PubMed PMID: WOS:000222586000009.

4999. Bergkvist A, Midlöv P, Höglund P, Larsson L, Bondesson A, Eriksson T. Improved quality in the hospital discharge summary reduces medication errors -- LIMM: Landskrona Integrated Medicines Management. European Journal of Clinical Pharmacology. 2009;65(10):1037-46. doi: 10.1007/s00228-009-0680-1. PubMed PMID: 105434193. Language: English. Entry Date: 20091204. Revision Date: 20150711. Publication Type: Journal Article.

5000. Bergkvist A, Midlöv P, Höglund P, Larsson L, Bondesson Å, Eriksson T. Improved quality in the hospital discharge summary reduces medication errors—LIMM: Landskrona Integrated Medicines Management. European journal of clinical pharmacology. 2009;65:1037-46.

5001. Bergkvist A, Midlöv P, Höglund P, Larsson L, Eriksson T. A multi-intervention approach on drug therapy can lead to a more appropriate drug use in the elderly. LIMM-Landskrona Integrated Medicines Management. Journal of Evaluation in Clinical Practice. 2009;15(4):660-7. doi: 10.1111/j.1365-2753.2008.01080.x. PubMed PMID: 105396060. Language: English. Entry Date: 20090918. Revision Date: 20200708. Publication Type: Journal Article.

5002. Bergler U, Ailabouni NJ, Pickering JW, Hilmer SN, Mangin D, Nishtala PS, et al. Deprescribing to reduce polypharmacy: study protocol for a randomised controlled trial assessing deprescribing of anticholinergic and sedative drugs in a cohort of frail older people living in the community. Trials. 2021;22(1). doi: 10.1186/s13063-021-05711-w. PubMed PMID: CN-02342091.

5003. Bergman Å, Olsson J, Carlsten A, Waern M, Fastbom J. Evaluation of the quality of drug therapy among elderly patients in nursing homes. Scandinavian Journal of Primary Health Care. 2007;25(1):9-14. doi: 10.1080/02813430600991980. PubMed PMID: 106293794. Language: English. Entry Date: 20070601. Revision Date: 20200708. Publication Type: Journal Article.

5004. Bergman AA, Jaynes HA, Gonzalvo JD, Hudmon KS, Frankel RM, Kobylinski AL, et al. Pharmaceutical role expansion and developments in pharmacist-physician communication. Health communication. 2016;31(2):161-70.

5005. Bergman U. Pharmacoepidemiological perspectives. Journal of Clinical Epidemiology. 1992;45(3):313-7. doi: https://doi.org/10.1016/0895-4356(92)90092-2.

5006. Bergman U, Popa C, Tomson Y, Wettermark B, Einarson T, Åberg H, et al. Drug utilization 90%–a simple method for assessing the quality of drug prescribing. European journal of clinical pharmacology. 1998;54:113-8.

5007. Bergmann JF. [When pharmacist playing doctor...]. Presse Medicale. 2019;48(10):1013-5. PubMed

PMID: 31787170.

5008. Bergmo TS, Sandsdalen V, Manskow US, Småbrekke L, Waaseth M. Internet Use for Obtaining Medicine Information: Cross-sectional Survey. JMIR Formative Research. 2023;7. doi: 10.2196/40466.

5009. Bergsholm YKR, Feiring M, Charnock C, Krogstad T, Holm LB. Positioning of community pharmacists in interactions with general practitioners and patients regarding prescribing and using antibiotics. Journal of Interprofessional Care. 2023;37(6):886-95. doi: 10.1080/13561820.2023.2203698.

5010. Berguillos Moretón L, López González I, Zardain Tamargo E, Comas Vega R. Implantation feasibility of an active dispensation model for compulsory prescription drugs in Asturian pharmacy offices: A pilot study. Pharmaceutical Care Espana. 2003;5(6):247-52.

5011. Berisa Prado S. Analisis of the quality of parenteral nutrition. Nutricion Clinica y Dietetica Hospitalaria. 2016;36(3):59-67. doi: 10.12873/363berisa.

5012. Berk T, Scheinfeld N. Seborrheic dermatitis. P and t. 2010;35(6):348-55. PubMed PMID: CN-01773638.

5013. Berkemeier F, Whaley C, Robinson JC. Increasing Divergence in Drug Prices Between the United States and Germany After Implementation of Comparative Effectiveness Analysis and Collective Price Negotiations. Journal of Managed Care & Specialty Pharmacy. 2019;25(12):1310-+. PubMed PMID: WOS:000500353000003.

5014. Berkhout C, Willefert-Bouche A, Chazard E, Zgorska-Maynard-Moussa S, Favre J, Peremans L, et al. Randomized controlled trial on promoting influenza vaccination in general practice waiting rooms. PloS one. 2018;13(2):e0192155. doi: 10.1371/journal.pone.0192155. PubMed PMID: CN-01453969.

5015. Berkovitz S, Bassett P, Hughes JG. A randomised double-blind comparability study of a placebo for Individualised Western Herbal Medicine. Complementary therapies in medicine. 2013;21(3):195-9. doi: 10.1016/j.ctim.2013.03.006. PubMed PMID: CN-00964033.

5016. Berl T, Quittnat-Pelletier F, Verbalis JG, Schrier RW, Bichet DG, Ouyang J, et al. Oral tolvaptan is safe and effective in chronic hyponatremia. Journal of the American Society of Nephrology. 2010;21(4):705-12.

5017. Berlana D, Almendral MA, Abad MR, Fernández A, Torralba A, Cervera-Peris M, et al. Cost, Time, and Error Assessment During Preparation of Parenteral Nutrition: Multichamber Bags Versus Hospital-Compounded Bags. JPEN Journal of Parenteral & Enteral Nutrition. 2019;43(4):557-65. doi: 10.1002/jpen.1436. PubMed PMID: 136445725. Language: English. Entry Date: 20200216. Revision Date: 20200927. Publication Type: journal article.

5018. Berlin JA, Golub RM. Meta-analysis as evidence: building a better pyramid. Jama. 2014;312(6):603-6.

5019. Berlowitz DR, Pugh MJV. Pharmacoepidemiology in community-dwelling elderly taking antiepileptic drugs. Neurobiology of Epilepsy and Aging. 2007;81:153-63. doi: 10.1016/s0074-7742(06)81009-3. PubMed PMID: WOS:000245980400009.

5020. Berman A, Warde PR, Avalos-Reyes E, Yeon H, Cavers W, Reddy S, et al. Predicting Total Hospitalizations and Emergency Visits in Radiotherapy Patients: A Claims-Based Model. International Journal of Radiation Oncology*Biology*Physics. 2022;114(3, Supplement):e341. doi: https://doi.org/10.1016/j.ijrobp.2022.07.1435.

5021. Berman BP, Nibu Y, Pfeiffer BD, Tomancak P, Celniker SE, Levine M, et al. Exploiting transcription

factor binding site clustering to identify cis-regulatory modules involved in pattern formation in the Drosophila genome. Proceedings of the National Academy of Sciences. 2002;99(2):757-62.

5022. Bermejo Vicedo T, Pérez Menéndez Conde C. Aplicación de las nuevas tecnologías a la farmacia hospitalaria en España. Farmacia Hospitalaria. 2007;31(1):17-22. doi: https://doi.org/10.1016/S1130-6343(07)75706-9.

5023. Bermeo-Ovalle A. Pick Your Poison but Pick It Wisely: choosing a Second-Line Medication for the Management of Status Epilepticus. Epilepsy currents. 2020;20(5):278-81. doi: 10.1177/1535759720949252. PubMed PMID: CN-02176533.

5024. Bermudez EG, Soler NGA, Sanchez FJ, Fernandez VJB. The "Revisa (R)" Project: The Impact of the Medicines Use Review Service in Spain. Time and Costs Report. Farmaceuticos Comunitarios. 2017;9(3):20-4. doi: 10.5672/FC.2173-9218.(2017/Vol9).003.05. PubMed PMID: WOS:000417277900005.

5025. Berna R, Quinlan M, Baumrin E. Author's Reply to Parvathaneni et al. Comment on: "Methotrexate Cutaneous Ulceration: A Systematic Review of Cases". American Journal of Clinical Dermatology. 2023;24(3):491-2. doi: 10.1007/s40257-023-00772-w. PubMed PMID: 163797751.

5026. Bernabe E, Flores M, Martinez F. ANALYSIS OF THE DISPENSATION OF OUTPATIENT ANTIBIOTICS IN A COMMUNITY PHARMACY AT MURCIA, SPAIN. Vitae-Revista De La Facultad De Quimica Farmaceutica. 2013;20(3):203-14. PubMed PMID: WOS:000331413500006.

5027. Bernabé M E, Flores D M, Martínez M F. Analysis of the dispensation of outpatient antibiotics in a community pharmacy at Murcia, Spain. Vitae. 2013;20(3):203-14.

5028. Bernal DD, Stafford L, Bereznicki LR, Castelino RL, Davidson PM, Peterson GM. Home medicines reviews following acute coronary syndrome: study protocol for a randomized controlled trial. Trials. 2012;13:30. doi: 10.1186/1745-6215-13-30. PubMed PMID: CN-00830571.

5029. Bernard GB, Montalvo S, Ivancic S, Eckardt P, Kehn-Yao Poon K, Parmar J, et al. Reprint of Implementation of a pharmacist-led ARVSP in an academic hospital to reduce ART errors. Journal of the American Pharmacists Association. 2022;62(4, Supplement):S47-S52. doi: https://doi.org/10.1016/j.japh.2022.06.003.

5030. Bernard GB, Montalvo S, Ivancic S, Eckardt P, Kehn-Yao Poon K, Parmar J, et al. Implementation of a pharmacist-led ARVSP in an academic hospital to reduce ART errors. Journal of the American Pharmacists Association. 2022;62(1):264-9. doi: https://doi.org/10.1016/j.japh.2021.08.007.

5031. Bernard L, Lecomte B, Pereira B, Proux A, Boyer A, Sautou V. Optimisation de la prévention de la bronchiolite à VRS chez les nouveaux-nés à risque et les prématurés : mesure de l'impact d'une intervention éducative ciblée. Archives de Pédiatrie. 2015;22(2):146-53. doi: https://doi.org/10.1016/j.arcped.2014.11.015.

5032. Bernard L, Lecomte B, Pereira B, Proux A, Boyer A, Sautou V. Impact of a targeted educational intervention on respiratory syncytial virus bronchiolitis prevention in full-term and preterm infants. Archives de Pediatrie. 2015;22(2):146-53. doi: 10.1016/j.arcped.2014.11.015.

5033. Bernard N, Xuereb F, Berdai D, Lahouati M, Perino J, Jenn J, et al. Therapeutic optimization in the elderly: Impact of the clinical pharmacist and synergistic effect of physician-pharmacist collaboration. Pharmacien Clinicien. 2023. doi: https://dx.doi.org/10.1016/j.phacli.2023.11.035. PubMed PMID: 2029207184.

5034. Bernard N, Xuereb F, Berdai D, Lahouati M, Perino J, Jenn J, et al. Optimisation thérapeutique chez la personne âgée : impact du pharmacien clinicien et effet synergique de la collaboration médecin-pharmacien. Le Pharmacien Clinicien. 2023. doi: https://doi.org/10.1016/j.phacli.2023.11.035.

5035. Bernard SA, Wheeler ME, Kolbet KC, Schueler KE, DiCarlo MD, McCord MA, et al. Transplant Pharmacist Intervention During Transplant Center Transfer Among Pediatric Patients. Journal of Heart & Lung Transplantation. 2023;42:S494-S5. doi: 10.1016/j.healun.2023.02.1363. PubMed PMID: 162850249.

5036. Bernard SA, Wheeler ME, Kolbet KC, Schueler KE, DiCarlo MD, McCord MA, et al. (1152) Transplant Pharmacist Intervention During Transplant Center Transfer Among Pediatric Patients. The Journal of Heart and Lung Transplantation. 2023;42(4, Supplement):S494-S5. doi: https://doi.org/10.1016/j.healun.2023.02.1363.

5037. Bernardes Alves AP, Del' Angelo Aredes N, Oliveira Silva G, dos Santos Oliveira F, Monti Fonseca LM, Medeiros Ribeiro L. Nurseped: educational technology for safety in the management of intravenous antibiotics in pediatrics. Revista Latino-Americana de Enfermagem (RLAE). 2023;31:1-12. doi: 10.1590/1518-8345.6886.4068. PubMed PMID: 175510082.

5038. Berndt ER, Cockburn IM, Cocks DL, Epstein AM, Griliches Z, editors. Is price inflation different for the elderly? An empirical analysis of prescription drugs. Conference on Frontiers in Health Policy Research; 1997 Jun 05; Washington, D.C.1998.

5039. Berndt J, Ohmann P, Rahal I, Hoeschen L, Rothstein A. Interdisciplinary and Innovative: A Nursing and Computer Science Collaboration to Create a Barcode Medication Administration System. Nursing Education Perspectives (Wolters Kluwer Health). 2023;44(3):192-3. doi: 10.1097/01.NEP.0000000000000972. PubMed PMID: 163302598.

5040. Berner ES, La Lande TJ. Overview of clinical decision support systems. Clinical decision support systems: Theory and practice. 2007:3-22.

5041. Bernett GB, Feldman S, Martin H, Smith BC, Raineri BD. An Opportunity for Medication Risk Reduction, Healthcare Provider Collaboration, and Improved Patient Care: A Retrospective Analysis of Osteoporosis Management. Journal of the American Medical Directors Association. 2003;4(6):329-36. doi: https://doi.org/10.1016/S1525-8610(04)70393-X.

5042. Bernhardt L. Characterization of Drug-Related Critical Incidents from Multiple Settings in the Critical Incident Reporting System North Rhine-Westphalia [Master's]. Sweden: Uppsala Universitet (Sweden); 2022.

5043. Bernhardt MB, Militano O, Honeyford L, Zupanec S. Blinatumomab use in pediatric ALL: Taking a BiTE out of preparation, administration and toxicity challenges. Journal of Oncology Pharmacy Practice. 2021;27:376-88. doi: 10.1177/1078155220979047. PubMed PMID: 148905763. Language: English. Entry Date: 20210303. Revision Date: 20210304. Publication Type: Article.

5044. Bernieh D. Application of microsampling methods to cardiovascular medication adherence assessment [Ph.D.]. England: De Montfort University (United Kingdom); 2017.

5045. Bernier MC, Desjardins K, Filiatrault J, Sauriol MA, Martineau J, Gilbert E, et al. Implementation and evaluation of a pharmacy-led thromboprophylaxis campaign in a community hospital. Journal of Thrombosis and Thrombolysis. 2011;32(4):431-8. doi: 10.1007/s11239-011-0614-5.

5046. Bernknopf AC, Karpinski JR, McKeever AL, Peak AS, Smith KM, Smith WD, et al. Drug Information: From Education to Practice. Pharmacotherapy. 2009;29(3):331-46. doi: 10.1592/phco.29.3.331. PubMed PMID: WOS:000263898900009.

5047. Bernstein J. Not the Last Word: Primum Non Nocere Is Harmful. Primum Noce Apte May Help. Clinical Orthopaedics & Related Research®. 2023;481(2):211-3. doi: 10.1097/CORR.0000000000002509. PubMed PMID: 161413049.

5048. Bernstein JA, Riedl M, Zacek L, Shapiro RS. Facilitating home-based treatment of hereditary angioedema. Allergy and Asthma Proceedings. 2015;36(2):92-9. doi: 10.2500/aap.2015.36.3820.

5049. Bernstein JA, White AA, Han JK, Lang DM, Elkayam D, Baroody FM. Review of evidence supporting the use of nasal corticosteroid irrigation for chronic rhinosinusitis. Annals of Allergy, Asthma & Immunology. 2023;130(1):46-57. doi: https://doi.org/10.1016/j.anai.2022.09.008.

5050. Bernstein JA, White AA, Han JK, Lang DM, Elkayam D, Baroody FM. Review of evidence supporting the use of nasal corticosteroid irrigation for chronic rhinosinusitis. Annals of Allergy, Asthma & Immunology. 2023;130(1):46-57. doi: https://doi.org/10.1016/j.anai.2022.09.008.

5051. Bernsten C, Andersson K, Gariepy Y, Simoens S. A comparative analysis of remuneration models for pharmaceutical professional services. Health Policy. 2010;95(1):1-9. doi: 10.1016/j.healthpol.2009.11.008.

5052. Bernsten C, Björkman I, Caramona M, Crealey G, Frøkjaer B, Grundberger E, et al. Improving the well-being of elderly patients via community pharmacy-based provision of pharmaceutical care: a multicentre study in seven European countries. Drugs & aging. 2001;18(1):63-77. doi: 10.2165/00002512-200118010-00005. PubMed PMID: CN-00327122.

5053. Bernsten C, Bjorkman I, Caramona M, Crealey G, Frokjaer B, Grundberger E, et al. Improving the well-being of elderly patients via community pharmacy-based provision of pharmaceutical care - A multicentre study in seven European countries. Drugs & Aging. 2001;18(1):63-77. doi: 10.2165/00002512-200118010-00005. PubMed PMID: WOS:000167131800005.

5054. Bero LA, Rennie D. Influences on the quality of published drug studies. International journal of technology assessment in health care. 1996;12(2):209-37.

5055. Beroule B, Grunder O, Barakat O, Aujoulat O. Order Picking Problem in a Warehouse Hospital Pharmacy. IFAC-PapersOnLine. 2017;50(1):5017-22. doi: https://doi.org/10.1016/j.ifacol.2017.08.910.

5056. Beroule B, Grunder O, Barakat O, Aujoulat O, Lustig H. Operating room scheduling including medical devices sterilization: towards a transverse logistic. IFAC-PapersOnLine. 2016;49(12):1146-51. doi: https://doi.org/10.1016/j.ifacol.2016.07.657.

5057. Berquist K, Linnebur SA, Fixen DR. Incorporation of Clinical Pharmacy Into a Geriatric Transitional Care Management Program. Journal of Pharmacy Practice. 2020;33(5):661-5. PubMed PMID: 30791857.

5058. Berringer R, Shibley MC, Cary CC, Pugh CB, Powers PA, Rafi JA. Outcomes of a community pharmacy-based diabetes monitoring program. Journal of the American Pharmaceutical Association (Washington,DC : 1996). 1999;39(6):791-7. doi: 10.1016/s1086-5802(15)30369-7.

5059. Berrut G. Medication reconciliation: a learning process for reduce the risk of medication errors. Geriatrie Et Psychologie Neuropsychiatrie Du Vieillissement. 2017;15(1):4. PubMed PMID: 28266339.

5060. Berry D, Raynor T, Knapp P, Bersellini E. Over the counter medicines and the need for immediate action: a further evaluation of European Commission recommended wordings for communicating risk. Patient Education and Counseling. 2004;53(2):129-34. doi: https://doi.org/10.1016/S0738-3991(03)00111-3.

5061. Berry DC, Raynor D, Knapp P, Bersellini E. Patients' understanding of risk associated with medication

use: impact of European Commission guidelines and other risk scales. Drug Safety. 2003;26:1-11.

5062. Berry J, Hilts KE, Thoma L, Corelli RL, Stump TE, Monahan PO, et al. Patient awareness, perceptions, and attitudes towards pharmacists prescribing tobacco cessation medications. Research in Social and Administrative Pharmacy. 2023;19(12):1531-42. doi: 10.1016/j.sapharm.2023.08.002.

5063. Berry J, Weber SC, Vaidya N, Haataja M, Brangwynne CP. RNA transcription modulates phase transition-driven nuclear body assembly. Proceedings of the National Academy of Sciences. 2015;112(38):E5237-E45.

5064. Berry JD. Collaborative Behaviours and Professional Culture Traits in Real-Time Interprofessional Clinical Simulation [Ph.D.]. England: Keele University (United Kingdom); 2020.

5065. Berry LL, Bendapudi N. Health care: a fertile field for service research. Journal of service research. 2007;10(2):111-22.

5066. Berry S, Harris C, McCormick M. A collaborative model to meet community and institutional needs during a medical emergency: Lessons learned from an influenza vaccine shortage. American Journal of Infection Control. 2005;33(5):e50. doi: https://doi.org/10.1016/j.ajic.2005.04.052.

5067. Berry SD, Goldfeld KS, McConeghy K, Gifford D, Davidson HE, Han L, et al. Evaluating the Findings of the IMPACT-C Randomized Clinical Trial to Improve COVID-19 Vaccine Coverage in Skilled Nursing Facilities. JAMA internal medicine. 2022;182(3):324-31. doi: 10.1001/jamainternmed.2021.8067. PubMed PMID: CN-02373140.

5068. Berry SD, Goldfeld KS, McConeghy K, Gifford D, Davidson HE, Han L, et al. Evaluating the Findings of the IMPACT-C Randomized Clinical Trial to Improve COVID-19 Vaccine Coverage in Skilled Nursing Facilities. JAMA Internal Medicine. 2022;182(3):324-31. doi: 10.1001/jamainternmed.2021.8067.

5069. Berry TM, Murphy JA, Montooth AK. Impact of a community pharmacy experience on medical residents' understanding of the role of a community pharmacist. Journal of the American Pharmacists Association: JAPhA. 2007;47(6):676-7. PubMed PMID: 18032130.

5070. Berry TM, Prosser TR, Wilson K, Castro M. Asthma friendly pharmacies: a model to improve communication and collaboration among pharmacists, patients, and healthcare providers. Journal of Urban Health. 2011;88 Suppl 1:113-25. PubMed PMID: 21337057.

5071. Bert JM. Making your orthopedic office profitable through the addition of ancillary services. Clinics in Sports Medicine. 2002;21(2):245-+. doi: 10.1016/s0278-5919(01)00003-5. PubMed PMID: WOS:000176774700006.

5072. Bertacche V, Pini E, Stradi R, Stratta F. Quantitative determination of amorphous cyclosporine in crystalline cyclosporine samples by Fourier transform infrared spectroscopy. Journal of Pharmaceutical Sciences. 2006;95(1):159-66. doi: https://doi.org/10.1002/jps.20523.

5073. Bertels J, Almoudaris AM, Cortoos P-J, Jacklin A, Franklin BD. Feedback on prescribing errors to junior doctors: exploring views, problems and preferred methods. International Journal of Clinical Pharmacy. 2013;35(3):332-8. doi: 10.1007/s11096-013-9759-y. PubMed PMID: 109856680. Language: English. Entry Date: 20140815. Revision Date: 20211029. Publication Type: journal article.

5074. Bertels J, Almoudaris AM, Cortoos PJ, Jacklin A, Franklin BD. Feedback on prescribing errors to junior doctors: exploring views, problems and preferred methods. International Journal of Clinical Pharmacy. 2013;35(3):332-8. PubMed PMID: 23475495.

5075. Berthe A, Fronteau C, Le Fur E, Morin C, Huon JF, Rouiller-Furic I, et al. Medication reconciliation: a tool to prevent adverse drug events in geriatrics medicine. Geriatrie Et Psychologie Neuropsychiatrie Du Vieillissement. 2017;15(1):19-24. PubMed PMID: 28120773.

5076. Berthe-Aucejo A, Nguyen NPKK, Angoulvant F, Boulkedid R, Bellettre X, Weil T, et al. Interrater reliability of a tool to assess omission of prescription and inappropriate prescriptions in paediatrics. International Journal of Clinical Pharmacy. 2019;41(3):734-40. doi: 10.1007/s11096-019-00819-1. PubMed PMID: 136840337. Language: English. Entry Date: 20190810. Revision Date: 20200601. Publication Type: journal article. Journal Subset: Biomedical.

5077. Berthe-Aucejo A, Nguyen PKH, Angoulvant F, Bellettre X, Albaret P, Weil T, et al. Retrospective study of irrational prescribing in French paediatric hospital: prevalence of inappropriate prescription detected by Pediatrics: Omission of Prescription and Inappropriate prescription (POPI) in the emergency unit and in the ambulatory setting. BMJ Open. 2019;9(3):e019186. PubMed PMID: 30898791.

5078. Berthélémy S. Ménopause : accompagnement à l'officine. Actualités Pharmaceutiques. 2015;54(544):40-4. doi: https://doi.org/10.1016/j.actpha.2014.12.015.

5079. Berthélémy S. Prévention et traitement de la grippe. Actualités Pharmaceutiques. 2015;54(542):36-9. doi: https://doi.org/10.1016/j.actpha.2014.11.007.

5080. Berthelot J-M, Glemarec J, Guillot P, Chiffoleau A, Maugars Y, Rodat O. Informing patients about serious side effects of drugs. A 2001 survey of 341 French rheumatologists. Joint Bone Spine. 2003;70(1):52-7. doi: https://doi.org/10.1016/S1297-319X(02)00011-8.

5081. Berthet S, Charpiat B, Mabrut JY. Erythromycin as a prokinetic agent: Risk factors. Journal of Visceral Surgery. 2010;147(2):e13-e8. doi: https://doi.org/10.1016/j.jviscsurg.2010.06.001.

5082. Berthet S, Charpiat B, Mabrut JY. Facteurs de risque de l'utilisation de l'érythromycine comme agent prokinétique. Journal de Chirurgie Viscérale. 2010;147(2):100-5. doi: https://doi.org/10.1016/j.jchirv.2010.06.001.

5083. Berthod F, Bouchoud L, Grossrieder F, Falaschi L, Senhaji S, Bonnabry P. Learning good manufacturing practices in an escape room: Validation of a new pedagogical tool. Journal of Oncology Pharmacy Practice. 2020;26(4):853-60. PubMed PMID: 31566110.

5084. Bertoldi AD, Barros AJD, Wagner A, Degnan DR, Hallal PC. A descriptive review of the methodologies used in household surveys on medicine utilization. Bmc Health Services Research. 2008;8. doi: 10.1186/1472-6963-8-222. PubMed PMID: WOS:000261240600001.

5085. Bertoldo P, Brignone S, Cignetti F, Bertoldo J. Profile of benzodiazepine consumption in community pharmaci. Revista Cubana de Farmacia. 2017;51(4).

5086. Bertoli AM, Strusberg I, Baravalle M, Betelu Z, Calás D, Morales L, et al. Rate and causes of infliximab discontinuation in patients with rheumatoid arthritis in a private clinical practice. Journal of Clinical Rheumatology. 2008;14(6):313-7. doi: 10.1097/RHU.0b013e31817a7e0e.

5087. Bertoli R, Bissig M, Caronzolo D, Odorico M, Pons M, Bernasconi E. Assessment of potential drug-drug interactions at hospital discharge. Swiss Medical Weekly. 2010;140:w13043. PubMed PMID: 20373176.

5088. Bertolino G, Marras L, Mureddu V, Camboni M, Cadeddu A. Trends of Antimicrobial Consumption in Hospital: Tackling the Hidden Part of the Iceberg with an Electronic Personalised Prescription Software for Antimicrobial Stewardship. Advances in Experimental Medicine and Biology. 2023;1370:113-23. doi: https://

dx.doi.org/10.1007/5584_2022_752. PubMed PMID: 2024247479.

5089. Bertrand F, Nai T, Reiter-Schatz A, Schwarzenbart A, Dory A, Passemard R, et al. Attentat de Strasbourg, 11 décembre 2018 : retour d'expérience du service pharmacie–stérilisation. Le Pharmacien Hospitalier et Clinicien. 2021;56(2):210-6. doi: https://doi.org/10.1016/j.phclin.2021.01.006.

5090. Bertsch DK. A nurse goes shopping for a continuing care retirement community. Geriatric Nursing. 2005;26(4):241-4. doi: 10.1016/j.gerinurse.2005.05.006. PubMed PMID: WOS:000231460700019.

5091. Bertsch NS, Bindler RJ, Wilson PL, Kim AP, Ward B. Medication Therapy Management for Patients Receiving Oral Chemotherapy Agents at a Community Oncology Center: A Pilot Study. Hospital Pharmacy. 2016;51(9):721-9. doi: 10.1310/hpj5109-721. PubMed PMID: 120024778. Language: English. Entry Date: 20161216. Revision Date: 20190103. Publication Type: Article.

5092. Bertsche T, Alexa JM, Eickhoff C, Schulz M. Self-care and self-medication as central components of healthcare in Germany - on the way to evidence-based pharmacy. Exploratory Research in Clinical and Social Pharmacy. 2023;9. doi: 10.1016/j.rcsop.2023.100257. PubMed PMID: WOS:001133314300001.

5093. Bertsche T, Hämmerlein A, Schulz M. German national drug information service: User satisfaction and potential positive patient outcomes. Pharmacy World and Science. 2007;29(3):167-72. doi: 10.1007/s11096-006-9041-7.

5094. Bertsche T, Lindner S, Damm M, Frontini R, Exner C, Himmerich H. An interdisciplinary concept to optimize patient safety - A pilot study. Psychiatrische Praxis. 2015;42(4):216-20. doi: 10.1055/s-0034-1387227. PubMed PMID: CN-01075246.

5095. Bertsche T, Lindner S, Damm M, Frontini R, Exner C, Himmerich H. An interdisciplinary concept to optimize patient safety--a pilot study. Psychiatrische Praxis. 2015;42(4):216-20. doi: 10.1055/s-0034-1387227. PubMed PMID: CN-01254572.

5096. Bertsche T, Maria Alexa J, Eickhoff C, Schulz M. Self-care and self-medication as central components of healthcare in Germany – on the way to evidence-based pharmacy. Exploratory Research in Clinical and Social Pharmacy. 2023;9. doi: 10.1016/j.rcsop.2023.100257.

5097. Bertsche T, Mayer Y, Stahl R, Hoppe-Tichy T, Encke J, Haefeli WE. Prevention of intravenous drug incompatibilities in an intensive care unit. American Journal of Health-System Pharmacy. 2008;65(19):1834-40. doi: 10.2146/ajhp070633. PubMed PMID: 105694404. Language: English. Entry Date: 20081121. Revision Date: 20200708. Publication Type: Journal Article.

5098. Bertsche T, Nachbar M, Fiederling J, Schmitt SPW, Kaltschmidt J, Seidling HM, et al. Assessment of a computerised decision support system for allergic rhino-conjunctivitis counselling in German pharmacy. International Journal of Clinical Pharmacy. 2012;34(1):17-22. doi: 10.1007/s11096-011-9584-0.

5099. Bertz JW, Epstein DH, Preston KL. Combining ecological momentary assessment with objective, ambulatory measures of behavior and physiology in substance-use research. Addictive Behaviors. 2018;83:5-17. doi: 10.1016/j.addbeh.2017.11.027. PubMed PMID: WOS:000435063200002.

5100. Bérubé M, Dupuis S, Leduc S, Roy I, Turcotte V, Côté C, et al. Tapering Opioid Prescription Program for High-Risk Trauma Patients: a Pilot Randomized Controlled Trial. Pain management nursing. 2022;23(2):142-50. doi: 10.1016/j.pmn.2021.08.001. PubMed PMID: CN-02323434.

5101. Berwick DM. Disseminating innovations in health care. Jama. 2003;289(15):1969-75.

5102. Berwick DM, Calkins DR, McCannon CJ, Hackbarth AD. The 100 000 lives campaign: setting a goal and a deadline for improving health care quality. Jama. 2006;295(3):324-7.

5103. Berzanskyte A, Valinteliene R, Haaijer-Ruskamp FM, Gurevicius R, Grigoryan L. Self-medication with antibiotics in Lithuania. International Journal of Occupational Medicine and Environmental Health. 2006;19(4):246-53. doi: 10.2478/v10001-006-0030-9.

5104. Besley N, Browne P, Park M, Pesheva P, Wong K, Hughes-Medlicott NJ, et al. Pharmacists in zoos? A qualitative study investigating the potential for pharmacist involvement in wildlife health care in Aotearoa, New Zealand. Journal of the American Pharmacists Association. 2023;63(3):825-31. doi: https://dx.doi.org/10.1016/j.japh.2023.01.008. PubMed PMID: 2022549126.

5105. Beso A, Franklin BD, Barber N. The frequency and potential causes of dispensing errors in a hospital pharmacy. Pharmacy World and Science. 2005;27:182-90.

5106. Bess DT, Carter J, DeLoach L, White CL. Pharmacy technician-to-pharmacist ratios: a state-driven safety and quality decision. Journal of the American Pharmacists Association: JAPhA. 2014;54(6):648-51. doi: 10.1331/JAPhA.2014.14032. PubMed PMID: 109763063. Language: English. Entry Date: 20150828. Revision Date: 20200708. Publication Type: journal article.

5107. Bessa A, Medina J, Tedesco H, Felipe C. A prospective, randomised, controlled study to assess the effect of pharmaceutical intervention on compliance and knowledge of immunosuppression in kidney transplant recipients. European journal of hospital pharmacy. 2015;22:A109-A10. doi: 10.1136/ejhpharm-2015-000639.264. PubMed PMID: CN-01334319.

5108. Besseling J, Hovingh GK, Huijgen R, Kastelein JJP, Hutten BA. Statins in Familial Hypercholesterolemia: Consequences for Coronary Artery Disease and All-Cause Mortality. Journal of the American College of Cardiology. 2016;68(3):252-60. doi: https://doi.org/10.1016/j.jacc.2016.04.054.

5109. Bessell T, Marriott J, Emmerton L, Nissen L. Improving Australians' access to prescription medicines: Development of pharmacy practice models. Final Report. 2005.

5110. Besson C, Chareyre S, Kirouani N, Jean-Jean S, Bretagnolle C, Henry A, et al. Contribution d'une équipe de pharmacie hospitalière à la prise en charge en réanimation des patients infectés par le SARS-CoV-2. Annales Pharmaceutiques Françaises. 2021;79(4):473-80. doi: https://doi.org/10.1016/j.pharma.2021.01.007.

5111. Besson C, Chareyre S, Kirouani N, Jean-Jean S, Bretagnolle C, Henry A, et al. [Contribution of a hospital pharmacy team to critical care of patients infected with SARS-CoV-2]. Annales Pharmaceutiques Francaises. 2021;79(4):473-80. PubMed PMID: 33516718.

5112. Best JT, Frith K, Anderson F, Rapp CG, Rioux L, Ciccarello C. Implementation of an Evidence-Based Order Set to Impact Initial Antibiotic Time Intervals in Adult Febrile Neutropenia. Oncology Nursing Forum. 2011;38(6):661-8. doi: 10.1188/11.ONF.661-668. PubMed PMID: 104637906. Language: English. Entry Date: 20111103. Revision Date: 20200708. Publication Type: Journal Article.

5113. Bethishou L, Herzik K, Fang N, Abdo C, Tomaszewski DM. The impact of the pharmacist on continuity of care during transitions of care: A systematic review. Journal of the American Pharmacists Association: JAPhA. 2020;60(1):163-77. doi: 10.1016/j.japh.2019.06.020. PubMed PMID: 141595061. Language: English. Entry Date: 20200731. Revision Date: 20211014. Publication Type: journal article.

5114. Beto JA, Listecki RE, Meyer DA, Budhy RJ, Bansal VK. Use of pharmacy computer prescription database to access hypertensive patients for mailed survey research. Annals of Pharmacotherapy.

1996;30(4):351-5. doi: 10.1177/106002809603000405.

5115. Betrán AP, Bergel E, Griffin S, Melo A, Nguyen MH, Carbonell A, et al. Provision of medical supply kits to improve quality of antenatal care in Mozambique: a stepped-wedge cluster randomised trial. The Lancet Global health. 2018;6(1):e57-e65. doi: 10.1016/S2214-109X(17)30421-7. PubMed PMID: CN-01442004.

5116. Betses M, Brennan T. Abusive prescribing of controlled substances--a pharmacy view. New England Journal of Medicine. 2013;369(11):989-91. PubMed PMID: 23964897.

5117. Bett B, MacHila N, Gathura PB, McDermott JJ, Eisler MC. Characterisation of shops selling veterinary medicines in a tsetse-infested area of Kenya. Preventive Veterinary Medicine. 2004;63(1-2):29-38. doi: 10.1016/j.prevetmed.2004.02.004.

5118. Bettenay K. An evaluation of pharmacist and physiotherapist prescribing trials in Queensland: Queensland University of Technology; 2020.

5119. Bettencourt de Sousa NTdVR. Optimizing the Detection of Myocardial Ischemia Using Advanced Cardiac Imaging [Ph.D.]. Portugal: Universidade do Porto (Portugal); 2014.

5120. Betti AH, Gallina SM, Fuchs FD, de Castro MS. Outpatient Satisfaction During Pharmacotherapy Followup. Latin American Journal of Pharmacy. 2011;30(4):702-7. PubMed PMID: WOS:000294752700012.

5121. Bettington E, Spinks J, Kelly F, Wheeler AJ. Returning unwanted medicines to pharmacies: Prescribing to reduce waste. Australian Prescriber. 2018;41(3):78-81. doi: 10.18773/austprescr.2018.015.

5122. Beumont MG, Graziani A, Ubel PA, MacGregor RR. Safety of dapsone as Pneumocystis carinii pneumonia prophylaxis in human immunodeficiency virus-infected patients with allergy to trimethoprim/ sulfamethoxazole. The American Journal of Medicine. 1996;100(6):611-6. doi: https://doi.org/10.1016/S0002-9343(96)00008-3.

5123. Beuscart J-B, Genin M, Dupont C, Verloop D, Duhamel A, Defebvre M-M, et al. Potentially inappropriate medication prescribing is associated with socioeconomic factors: a spatial analysis in the French Nord-Pas-de-Calais Region. Age & Ageing. 2017;46(4):607-13. doi: 10.1093/ageing/afw245. PubMed PMID: 123799954. Language: English. Entry Date: 20170628. Revision Date: 20180702. Publication Type: Article.

5124. Beuscart JB, Dupont C, Defebvre MM, Puisieux F. Potentially inappropriate medications (PIMs) and anticholinergic levels in the elderly: a population based study in a French region. Archives of Gerontology & Geriatrics. 2014;59(3):630-5. PubMed PMID: 25192614.

5125. Beuscart JB, Genin M, Dupont C, Verloop D, Duhamel A, Defebvre MM, et al. Potentially inappropriate medication prescribing is associated with socioeconomic factors: a spatial analysis in the French Nord-Pas-de-Calais Region. Age & Ageing. 2017;46(4):607-13. PubMed PMID: 28064169.

5126. Beuscart JB, Petit S, Gautier S, Wierre P, Balcaen T, Lefebvre JM, et al. Polypharmacy in older patients: identifying the need for support by a community pharmacist. BMC Geriatrics. 2019;19(1):277. PubMed PMID: 31638909.

5127. Beuschel T, Gootee E, Jordan M, Sikkenga T, Klepser DG, Holmquist H, et al. Time and motion study of hepatitis C virus point-of-care testing in community pharmacies. Journal of the American Pharmacists Association. 2023;63(1):435-9. doi: 10.1016/j.japh.2022.11.002. PubMed PMID: WOS:000925685900001.

5128. Bevacqua M, Sforzi I, Bressan S, Barbi E, Sahyoun C. "Procedural sedation and analgesia in Italian pediatric emergency departments: a subgroup analysis in italian hospitals". Italian Journal of Pediatrics. 2023;49(1):1-8. doi: 10.1186/s13052-023-01426-7. PubMed PMID: 161899222.

5129. Beverina I, Aloni A, Gatti F, Varalli L, Brando B. Attitude changes in prescribing intravenous iron supplementation in different settings at a hospital consortium in Italy. Transfusion and Apheresis Science. 2021;60(4):103139. doi: https://doi.org/10.1016/j.transci.2021.103139.

5130. Beverley CA, Bath P, Booth A. Health information needs of visually impaired people: a systematic review of the literature. Health & Social Care in the Community. 2004;12(1):1-24.

5131. Bevilacqua S, Demoré B, Erpelding ML, Boschetti E, May T, May I, et al. Effects of an operational multidisciplinary team on hospital antibiotic use and cost in France: a cluster controlled trial. International journal of clinical pharmacy. 2011;33(3):521-8. doi: 10.1007/s11096-011-9499-9. PubMed PMID: CN-00812241.

5132. Bex SD, Boldt AS, Needham SB, Bolf SM, Walston CM, Ramsey DC, et al. Effectiveness of a hypertension care management program provided by clinical pharmacists for veterans. Pharmacotherapy. 2011;31(1):31-8. doi: 10.1592/phco.31.1.31.

5133. Beyea SC, Hicks RW, Becker SC. Medication errors in the OR—a secondary analysis of Medmarx. AORN journal. 2003;77(1):122-34.

5134. Beyeler N, Liu J, Sieverding M. A Systematic Review of the Role of Proprietary and Patent Medicine Vendors in Healthcare Provision in Nigeria. Plos One. 2015;10(1). doi: 10.1371/journal.pone.0117165. PubMed PMID: WOS:000348732100071.

5135. Beyene K, Aspden T, Sheridan J. Using the Behaviour Change Wheel to explore potential strategies for minimising harms from non-recreational prescription medicine sharing. Research in Social and Administrative Pharmacy. 2019;15(2):130-44. doi: https://doi.org/10.1016/j.sapharm.2018.04.001.

5136. Beyene K, Chan AHY, Bandreddi NST, Tabar RB, Moyle E, Nath S, et al. Patient satisfaction with community pharmacist-led anticoagulation management services and its relationship with patient characteristics in New Zealand. International Journal of Clinical Pharmacy. 2021;43(1):154-64. doi: 10.1007/s11096-020-01124-y. PubMed PMID: WOS:000560266100001.

5137. Beyene K, Chan AHY, Barton E, Yan SY, Singh S, Basani A, et al. Pharmacists views on participating in New Zealand's community pharmacy anticoagulation management service: a mixed-methods study. International Journal of Clinical Pharmacy. 2021;43(1):251-62. doi: 10.1007/s11096-020-01148-4. PubMed PMID: WOS:000568972200002.

5138. Beyene KA, Chan AHY, Aquite OM, Kumar M, Moore S, Park YJ, et al. Postdischarge opioid use and persistent use after general surgery: A retrospective study. Surgery. 2022;172(2):602-11. doi: https://doi.org/10.1016/j.surg.2022.02.017.

5139. Beyene KA, Chan AHY, Aquite OM, Kumar M, Moore S, Park YJ, et al. Postdischarge opioid use and persistent use after general surgery: A retrospective study. Surgery. 2022;172(2):602-11. doi: 10.1016/j.surg.2022.02.017. PubMed PMID: WOS:000863530900019.

5140. Beyk Mirza A, Ashourkhani M, Abdi K, Shlaviri G. Identifying the factors affecting the irrational prescription and use of injectable drugs based on the perception and experience of the stakeholders. Iranian Journal of Health Education and Health Promotion. 2022;10(4):335-49. doi: 10.52547/ijhehp.10.4.335.

5141. Beyleveldt J. Health-Related Knowledge and Behaviour of Primary School Children [M.Com.]. South Africa: University of Johannesburg (South Africa); 2003.

5142. Beyth RJ, Quinn L, Landefeld CS. A multicomponent intervention to prevent major bleeding complications in older patients receiving warfarin: a randomized, controlled trial. Annals of internal medicine. 2000;133(9):687-95.

5143. Bezdicek O, Lukavsky J, Preiss M. Functional Activities Questionnaire, Czech Version - a Validation Study. Ceska a Slovenska Neurologie a Neurochirurgie. 2011;74(1):36-42. PubMed PMID: WOS:000287787700004.

5144. Bezerra HS, Brasileiro Costa AL, Pinto RS, Ernesto de Resende P, Martins de Freitas GR. Economic impact of pharmaceutical services on polymedicated patients: A systematic review. Research in Social and Administrative Pharmacy. 2022;18(9):3492-500. doi: https://doi.org/10.1016/j.sapharm.2022.03.005.

5145. Bezerra S, Guerreiro M, Gomes JJ, Pessoa N, Auad R, Sobrinho JLS. Cure and discontinuation of treatment in a tuberculosis control state programme in Brazil: insights from dispensing data. International journal of clinical pharmacy Conference: 45th ESCP-NSF international symposium on clinical pharmacy: clinical pharmacy tackling inequalities and access to health care Norway Conference start: 20161005 Conference end: 20161007. 2017;39(1):224-5. doi: 10.1007/s11096-016-0404-4. PubMed PMID: CN-01333869.

5146. Bezerra SS, Guerreiro M, Pessoa N, Athayde MP, Auad R, Jose Gomes J, et al. Clinical profile and treatment discontinuation in a tuberculosis control state programme in Brazil: preliminary results from SINAN database. International journal of clinical pharmacy Conference: 45th ESCP-NSF international symposium on clinical pharmacy: clinical pharmacy tackling inequalities and access to health care Norway Conference start: 20161005 Conference end: 20161007. 2017;39(1):222. doi: 10.1007/s11096-016-0404-4. PubMed PMID: CN-01333871.

5147. Bezverhni Z, Grintsova O. Pharmacy Professional Standards Defining Quality of Services. In: Babar Z-U-D, editor. Encyclopedia of Pharmacy Practice and Clinical Pharmacy. Oxford: Elsevier; 2019. p. 174-81.

5148. Bhagat H, Sikka MK, Sukerman ES, Makadia J, Lewis JS, Streifel AC. Evaluation of Opportunities for Oral Antibiotic Therapy in Bone and Joint Infections. Annals of Pharmacotherapy. 2023;57(2):156-62. doi: 10.1177/10600280221101105. PubMed PMID: WOS:000806894800001.

5149. Bhagavathula AS, Obamiro K, Hussain Z, Tesfaye W. Workplace violence against pharmacists: A systematic review and meta-analysis. Journal of the American Pharmacists Association. 2023;63(1):23-31. doi: https://doi.org/10.1016/j.japh.2022.07.012.

5150. Bhagavathula AS, Obamiro K, Hussain Z, Tesfaye W. Workplace violence against pharmacists: A systematic review and meta-analysis. Journal of the American Pharmacists Association. 2023;63(1):23-31. doi: https://doi.org/10.1016/j.japh.2022.07.012.

5151. Bhagwan V, Kirke C, Meehan F, Hickey M, Kinlan E, Grimes T. A quality improvement project addressing peri-operative medication reconciliation and administration in elective surgical patients using five or more medications pre-admission. International journal of pharmacy practice. 2018;26:35-6. doi: 10.1111/ijpp.2018.26.issue-S1. PubMed PMID: CN-01570735.

5152. Bhagwat AM, Ferryman KS, Gibbons JB. Mitigating algorithmic bias in opioid risk-score modeling to ensure equitable access to pain relief. Nature Medicine. 2023;29(4):769-70. doi: https://dx.doi.org/10.1038/s41591-023-02256-0. PubMed PMID: 2022181867.

5153. Bhakta SB, Colavecchia AC, Haines L, Varkey D, Garey KW. A systematic approach to optimize electronic health record medication alerts in a health system. American Journal of Health-System Pharmacy. 2019;76(8):530-6. doi: 10.1093/ajhp/zxz012. PubMed PMID: 135845539. Language: English. Entry Date: 20190415. Revision Date: 20200414. Publication Type: Article.

5154. Bhalla IP, Deegan D, Stefanovics EA, Rosenheck RA. Psychiatric Multimorbidity in a Specialized Program for Severely Mentally Ill Veterans. Psychiatric Quarterly. 2021;92(2):489-99. doi: 10.1007/s11126-020-09826-2. PubMed PMID: WOS:000560623200003.

5155. Bhamra SK, Parmar J, Heinrich M. Impact of the coronavirus pandemic (COVID-19) on the professional practice and personal wellbeing of community pharmacy teams in the UK. International Journal of Pharmacy Practice. 2021;29(6):556-65. doi: 10.1093/ijpp/riab062. PubMed PMID: WOS:000733332600005.

5156. Bhandari A, Lee JH, Ross E, Bomaliyamu A, Lee YJ, Donahue KT. Retrospective chart-review to study differences in pain-score between smoking and nonsmoking patients following SCS implantation. Neuromodulation. 2019;22(3):E248-. doi: 10.1111/ner.12950. PubMed PMID: CN-01936963.

5157. Bhandari DR, Subish P, Mishra P, Alam K. Do pharmacists have a scope in south Asian hospitals? An experience from Nepal. Pharmacologyonline (Newsletter). 2006;3:146-55.

5158. Bhandari R, Orgel E, Rushing T, Malicse K, Evangelista V, Jodele S, et al. Improving the Timeliness of Chemotherapy Administration in the Bone Marrow Transplant Unit. Biology of Blood and Marrow Transplantation. 2020;26(1):150-6. doi: https://doi.org/10.1016/j.bbmt.2019.09.026.

5159. Bharat C, Chidwick K, Gisev N, Farrell M, Ali R, Degenhardt L. Trends in use of medicines for opioid agonist treatment in Australia, 2013-2022. International Journal of Drug Policy. 2024;123. doi: 10.1016/j.drugpo.2023.104255. PubMed PMID: WOS:001132029000001.

5160. Bhardwaja B, Carroll NM, Raebel MA, Chester EA, Korner EJ, Rocho BE, et al. Improving prescribing safety in patients with renal insufficiency in the ambulatory setting: the Drug Renal Alert Pharmacy (DRAP) program. Pharmacotherapy. 2011;31(4):346-56. doi: 10.1592/phco.31.4.346. PubMed PMID: CN-00788606.

5161. Bhargava V, Greg ME, Shields MC. Addition of generic medication vouchers to a pharmacist academic detailing program: effects on the generic dispensing ratio in a physician-hospital organization. Journal of managed care pharmacy. 2010;16(6):384-92. doi: 10.18553/jmcp.2010.16.6.384. PubMed PMID: CN-01089728.

5162. Bharti V, Tiwari RK, Gupta S, Upadhyay R, Singh MK, Singh DK. The spectrum and etiologies of lower urinary tract symptoms in postmenopausal women. Current Urology. 2023;17(3):179-83. doi: 10.1097/cu9.0000000000000196. PubMed PMID: WOS:001021569600007.

5163. Bhaskar S, Pullarkat P, Wan F, Butler S, Mehta-Shah N. BMI Is Not Associated with Toxicity to High Dose Methotrexate: A Single Institution Retrospective Study of 147 Patients. Blood. 2020;136:42. doi: https://doi.org/10.1182/blood-2020-137536.

5164. Bhat S, Coyle DH, Trieu K, Neal B, Mozaffarian D, Marklund M, et al. Healthy Food Prescription Programs and their Impact on Dietary Behavior and Cardiometabolic Risk Factors: A Systematic Review and Meta-Analysis. Advances in Nutrition. 2021;12(5):1944-56. doi: https://doi.org/10.1093/advances/nmab039.

5165. Bhat S, Kansal M, Kondos GT, Groo V. Outcomes of a Pharmacist-Managed Heart Failure Medication Titration Assistance Clinic. Annals of Pharmacotherapy. 2018;52(8):724-32. PubMed PMID: 29463107.

5166. Bhat S, Khamo N, Abdou S, Hanson R, Khiani V, Stubbings J. The Pharmacist's Role in Biologic Management for IBD in a Health System-Integrated Practice Model. American Journal of Pharmacy Benefits. 2015;7(5):215-+. PubMed PMID: WOS:000410458100005.

5167. Bhat S, Kroehl M, Yi WM, Jaeger J, Thompson AM, Lam HM, et al. Factors influencing the acceptance of referrals for clinical pharmacist managed disease states in primary care. Journal of the American Pharmacists Association: JAPhA. 2019;59(3):336-42. PubMed PMID: 30948239.

5168. Bhat YR, Acharya LD, Rao PGM, Sam KG. Survey and Evaluation of Prescription Drugs Dispensed without a Valid Prescription in Three Community Pharmacies of South India. Indian Journal of Pharmaceutical Education and Research. 2008;42(3):256-61. PubMed PMID: WOS:000268896200009.

5169. Bhatt AS, Varshney A, Moscone A, Cunningham J, Jering K, Sinnenberg L, et al. Feasibility of Virtual Optimization of Guideline Directed Medical Therapy in Hospitalized Patients with HFrEF During the Covid-19 Pandemic: The IMPLEMENT-HF Pilot Study. Journal of Cardiac Failure. 2020;26(10, Supplement):S162-S3. doi: https://doi.org/10.1016/j.cardfail.2020.09.467.

5170. Bhatt AS, Varshney AS, Moscone A, Claggett BL, Miao ZM, Chatur S, et al. Virtual Care Team Guided Management of Patients With Heart Failure During Hospitalization. Journal of the American College of Cardiology. 2023;81(17):1680-93. doi: https://doi.org/10.1016/j.jacc.2023.02.029.

5171. Bhatt AS, Varshney AS, Nekoui M, Moscone A, Cunningham JW, Jering KS, et al. Virtual optimization of guideline-directed medical therapy in hospitalized patients with heart failure with reduced ejection fraction: the IMPLEMENT-HF pilot study. European journal of heart failure. 2021;23(7):1191-201. doi: 10.1002/ejhf.2163. PubMed PMID: CN-02266755.

5172. Bhatt LD, Pal L, Dhami SS, Thapa K. Compliance of iron and folic acid supplementation among postpartum urban mothers of kathmandu valley. Journal of Nepal Paediatric Society. 2021;41(2):154-61. doi: 10.3126/JNPS.V41I2.31248.

5173. Bhatt P, Lalani R, Vhora I, Patil S, Amrutiya J, Misra A, et al. Liposomes encapsulating native and cyclodextrin enclosed paclitaxel: Enhanced loading efficiency and its pharmacokinetic evaluation. International journal of pharmaceutics. 2018;536(1):95-107.

5174. Bhatt P, Patel Z. Temporal trends in pharmacology publications by pharmacy institutes: A deeper dig. Indian Journal of Pharmacology. 2016;48(7):S74-S7. doi: 10.4103/0253-7613.193329.

5175. Bhatt S, McCurdy CE, Liew D, Russell DM. Effect of warfarin education on patient knowledge and anticoagulation control: a randomised controlled trial. Journal of pharmacy practice and research. 2018;(no pagination). doi: 10.1002/jppr.1430. PubMed PMID: CN-01667508.

5176. Bhattacharjee P, Emmanuel F, Kioko J, Musyoki H, Kaosa S, Ongaro MK, et al. Mapping virtual platforms to estimate the population size of men who have sex with men (MSM) who use internet to find sexual partners: implications to enhance HIV prevention among MSM in Kenya. Gates open research. 2020;4. doi: 10.12688/gatesopenres.13158.1. PubMed PMID: CN-02205470.

5177. Bhattacharjee S, Knapp S, Malone DC, Lee JK, Patanwala AE, Vadiei N, et al. Pnd23 Healthcare Expenditures Associated with Adherence to Antidepressant Medication Management Measures during Acute and Continuation Phases of Depression Treatment among Older Adults with Dementia and Major Depressive Disorder. Value in Health. 2019;22(Supplement 3):S740. PubMed PMID: 2004267945.

5178. Bhattacharya BS. On Equitably Reducing Stock-Outs for Essential Pharmaceuticals in Low-Resource Countries [Ph.D.]. United States -- New York: State University of New York at Buffalo; 2019.

5179. Bhattacharya D, Aldus CF, Barton G, Bond CM, Boonyaprapa S, Charles IS, et al. The feasibility of determining the effectiveness and cost-effectiveness of Medication Organisation Devices compared to usual care for older people in a community setting: Systematic review, stakeholder focus groups and feasibility RCT. Health Technology Assessment. 2016.

5180. Bhattacharya D, Brown TJ, Clark AB, Dima AL, Easthall C, Taylor N, et al. Validation of the Identification of Medication Adherence Barriers Questionnaire (IMAB-Q); a Behavioural Science-Underpinned Tool for Identifying Non-Adherence and Diagnosing an Individual's Barriers to Adherence. Patient Preference and Adherence. 2023;17:2991-3000. doi: 10.2147/PPA.S427207.

5181. Bhattacharya K. Multiple Myeloma – Treatments, Economic Burden, and End of Life Care [Ph.D.]. United States -- Mississippi: The University of Mississippi; 2020.

5182. Bhattacharya R, Herren K, Poonawalla I, Bunniran S, Bloomfield A, Schwab P. Comparing Medical Utilization and Cost Outcomes in Oral Versus Injectable Immunotherapy Users with Chronic Inflammatory Joint and Skin Diseases. Journal of Managed Care & Specialty Pharmacy. 2020;26(10):1246-56. doi: 10.18553/jmcp.2020.26.10.1246. PubMed PMID: WOS:000576587400006.

5183. Bhattacharyya SK, Else BA. Medical costs of managed care in patients with type 2 diabetes mellitus. Clinical Therapeutics. 1999;21(12):2131-42. doi: https://doi.org/10.1016/S0149-2918(00)87243-4.

5184. Bhatti S, Malik AA, Rafaqat M, Ishaque U, Ayyaz M. Acuphagia as a Cause of Gastric Bezoar Causing Gastric Outlet Obstruction. Jcpsp-Journal of the College of Physicians and Surgeons Pakistan. 2014;24:S190-S2. PubMed PMID: WOS:000351754600015.

5185. Bhatti S, Wake N, Jani Y. Evaluating the effectiveness of digital communication within the National Medication Safety Network for England. European Journal of Hospital Pharmacy. 2022;29(5):275-9. doi: 10.1136/ejhpharm-2020-002517. PubMed PMID: 160078458. Language: English. Entry Date: 20230103. Revision Date: 20230103. Publication Type: Article.

5186. Bhaumik U, Woods ER, Samnaliev M, Sommer SJ, Lockridge R, Penzias RE, et al. Community asthma initiative: cost analyses using claims data from a Medicaid managed care organization. Pediatrics. 2019;144(2). doi: 10.1542/peds.144.2-MeetingAbstract.101. PubMed PMID: CN-02073388.

5187. Bhave PD, Goldman LE, Vittinghoff E, Maselli JH, Auerbach A. Statin Use And Postoperative Atrial Fibrillation After Major Noncardiac Surgery. Heart Rhythm. 2012;9(2):163-9. doi: https://doi.org/10.1016/j.hrthm.2011.09.003.

5188. Bhavsar TR, Kim H-J, Yu Y. Roles and contributions of pharmacists in regulatory affairs at the Centers for Disease Control and Prevention for public health emergency preparedness and response. Journal of the American Pharmacists Association. 2010;50(2):165-8. doi: https://doi.org/10.1331/JAPhA.2010.09220.

5189. Bhavya R, Sarwar F, Shivani P, Jagruthi P. BARCODE MEDICATION ADMINISTRATION. International Journal of Applied Pharmaceutics. 2023;15(1):204. PubMed PMID: 640230512.

5190. Bhayani D, Mehta P, Patel M, Naik H, Nathaniel TN, Khan S. Ground-based selected ionizing space radiation effects on stability of APIs and their formulations. Journal of Pharmaceutical and Biomedical Analysis. 2022;220:115019. doi: https://doi.org/10.1016/j.jpba.2022.115019.

5191. Bheekie A, Syce JA, Weinberg EG. An assessment of asthmatic patients at four Western Cape community pharmacies. South African Medical Journal. 1998;88(3):262-6. PubMed PMID: WOS:000073660700022.

5192. Bheekie A, Syce JA, Weinberg EG. Peak expiratory flow rate and symptom self-monitoring of asthma initiated from community pharmacies. Journal of clinical pharmacy and therapeutics. 2001;26(4):287-96. doi: 10.1046/j.1365-2710.2001.00361.x. PubMed PMID: CN-00442989.

5193. Bhella S, Majhail NS, Betcher J, Costa LJ, Daly A, Dandoy CE, et al. Choosing Wisely BMT: American Society for Blood and Marrow Transplantation and Canadian Blood and Marrow Transplant Group's List of 5 Tests and Treatments to Question in Blood and Marrow Transplantation. Biology of Blood and Marrow Transplantation. 2018;24(5):909-13. doi: https://doi.org/10.1016/j.bbmt.2018.01.017.

5194. bhgyb RBR. Topical Treatment of Phimosis. https://trialsearchwhoint/Trial2aspx?TrialID=RBR-76bhgyb. 2022. PubMed PMID: CN-02475994.

5195. Bhise NS, Ribas J, Manoharan V, Zhang YS, Polini A, Massa S, et al. Organ-on-a-chip platforms for studying drug delivery systems. Journal of Controlled Release. 2014;190:82-93.

5196. Bhoi P, Bessette L, Bell MJ, Tkaczyk C, Nantel F, Maslova K. Adherence and dosing interval of subcutaneous antitumour necrosis factor biologics among patients with inflammatory arthritis: analysis from a Canadian administrative database. Bmj Open. 2017;7(9). doi: 10.1136/bmjopen-2017-015872. PubMed PMID: WOS:000412650700078.

5197. Bhosle M, Sansgiry SS. Computerized physician order entry systems: is the pharmacist's role justified? Journal of the American Medical Informatics Association. 2004;11(2):125-6; author reply 7-8. PubMed PMID: 14662802.

5198. Bhowmick S, Saha AK, Jana S, Patil N, Munshi M. Need for recognition of pharmacists as front liners in COVID-19 pandemic: An Indian perspective. International Journal of Research in Pharmaceutical Sciences. 2020;11(Special Issue 1):1226-9. doi: 10.26452/ijrps.v11iSPL1.3601.

5199. Bhowmik D, Hines DM, Intorcia M, Wade RL. Economic burden of skeletal-related events in patients with multiple myeloma: analysis of US commercial claims database. Journal of Medical Economics. 2018;21(6):622-8. doi: 10.1080/13696998.2018.1457531. PubMed PMID: WOS:000433986300013.

5200. Bhutani T, Peng D, Shin T, Lee H. Common sources of skin care education: a cross-sectional study. International Journal of Dermatology. 2009;48(5):506-12. doi: 10.1111/j.1365-4632.2009.04040.x. PubMed PMID: WOS:000265070700010.

5201. Bhutta TI, Balchin C. Assessing the impact of a regulatory intervention in Pakistan. Social Science & Medicine. 1996;42(8):1195-202. doi: 10.1016/0277-9536(95)00392-4. PubMed PMID: WOS:A1996UF59200011.

5202. Bhuvan KC, Alrasheedy AA, Ibrahim MIM. A case report from Nepalese community pharmacy on levofloxacin induced severe abdominal pain. Saudi Pharmaceutical Journal. 2013;21(3):323-5. doi: https://doi.org/10.1016/j.jsps.2013.04.006.

5203. Bhuvan KC, Alrasheedy AA, Ibrahim MIM. Do community pharmacists in Nepal have a role in adverse drug reaction reporting systems? Australasian Medical Journal. 2013;6(2):100-3. doi: 10.4066/AMJ.2013.1544.

5204. Bhuvan KC, Khan TM, Xuan WY, Alrasheedy AA, Ibrahim MIM, Leggat PA. Travel health-related activities and services provided by community pharmacies in Selangor, Malaysia: A cross-sectional analysis. Travel Medicine and Infectious Disease. 2020;33. doi: 10.1016/j.tmaid.2019.07.019. PubMed PMID: WOS:000517778600032.

5205. Bhuvan KC, Lim D, Low CC, Chew C, Blebil AQ, Dujaili JA, et al. Positioning and Utilization of Information and Communication Technology in Community Pharmacies of Selangor, Malaysia: Cross-Sectional Study. Jmir Medical Informatics. 2020;8(7). doi: 10.2196/17982. PubMed PMID: WOS:000557459300024.

5206. Bi V, Jawa V, Joubert MK, Kaliyaperumal A, Eakin C, Richmond K, et al. Development of a Human Antibody Tolerant Mouse Model to Assess the Immunogenicity Risk Due to Aggregated Biotherapeutics. Journal of Pharmaceutical Sciences. 2013;102(10):3545-55. doi: https://doi.org/10.1002/jps.23663.

5207. Bian S, Tang X, Ye S, Lei W. Alkalescent soda beverage caused the disappearance of gefitinib-induced rashes and decreased efficacy in a non-small-cell lung cancer patient treated with gefitinib: A case report. Respiratory Medicine Case Reports. 2020;31:101228. doi: https://doi.org/10.1016/j.rmcr.2020.101228.

5208. Bianchi M, Clavenna A, Labate L, Bortolotti A, Fortino I, Locatelli GW, et al. Antiasthmatic drug prescription profile in a paediatric Italian population. [Italian]. Medico e Bambino. 2007;26(10):653-9. PubMed PMID: 351152390.

5209. Bianchi M, Clavenna A, Labate L, Bortolotti A, Fortino I, Merlino L, et al. Anti-asthmatic drug prescriptions to an Italian paedriatic population. Pediatric Allergy and Immunology. 2009;20(6):585-91. doi: 10.1111/j.1399-3038.2008.00797.x.

5210. Bianchini ML, Mercuro NJ, Kenney RM, Peters MA, Samuel LP, Swiderek J, et al. Improving care for critically ill patients with community-acquired pneumonia. American Journal of Health-System Pharmacy. 2019;76(12):861-8. doi: 10.1093/ajhp/zxz068.

5211. Bianco A, Licata F, Trovato A, Napolitano F, Pavia M. Antibiotic-dispensing practice in community pharmacies: results of a cross-sectional study in Italy. Antimicrobial Agents and Chemotherapy. 2021;65(6). doi: 10.1128/AAC.02729-20.

5212. Bianco T, Haxby DG, Vanderveen RP, Boyce R, Lee NL, Muilenburg N. Development of ambulatory clerkships in a managed care setting. American Journal of Pharmaceutical Education. 1996;60(1):48-52. PubMed PMID: WOS:A1996UE20500008.

5213. Biardeau X, Elkoushy MA, Aharony S, Elhilali M, Corcos J. Is multichannel urodynamic assessment necessary before considering a surgical treatment of BPH? Pros and cons. World Journal of Urology. 2016;34(4):463-9. doi: 10.1007/s00345-015-1647-8. PubMed PMID: WOS:000372626700001.

5214. Bibi R, Adnan M, Tariq A, Mussarat S, Andaleeb R, Khan AUR. Traditional medicines of the Himalayan region: Anti-dermatophytic potential and mode of action of isolated compounds. Journal of Herbal Medicine. 2023;38:100639. doi: https://doi.org/10.1016/j.hermed.2023.100639.

5215. Bibi R, Adnan M, Tariq A, Mussarat S, Andaleeb R, Khan AUR. Traditional medicines of the Himalayan region: Anti-dermatophytic potential and mode of action of isolated compounds. Journal of Herbal Medicine. 2023:100639. doi: https://doi.org/10.1016/j.hermed.2023.100639.

5216. Bible LJ, Casper KA, Seifert JL, Porter KA. Assessment of self-care and medication adherence in individuals with mental health conditions. Journal of the American Pharmacists Association: JAPhA. 2017;57(3S):S203-S10.e3. PubMed PMID: 28400250.

5217. Bible LJ, Casper KA, Seifert JL, Porter KA, editors. Assessment of self-care and medication adherence in individuals with mental health conditions. Journal of the American Pharmacists Association; 2017.

5218. Bick RL. Low molecular weight heparins in the outpatient management of venous thromboembolism.

Seminars in Thrombosis and Hemostasis. 1999;25:97-9. PubMed PMID: WOS:000083298700015.

5219. Bickel S, Eid S, Eid N. Prescription Fill Adherence in Pediatric Asthma: Are Privately Insured Children at Increased Risk? Pediatric Allergy Immunology and Pulmonology. 2016;29(3):125-9. doi: 10.1089/ped.2016.0674. PubMed PMID: WOS:000384309700004.

5220. Bickerton GR, Paolini GV, Besnard J, Muresan S, Hopkins AL. Quantifying the chemical beauty of drugs. Nature chemistry. 2012;4(2):90-8.

5221. Bicket MC, Brat GA, Hutfless S, Wu CL, Nesbit SA, Alexander GC. Optimizing opioid prescribing and pain treatment for surgery: Review and conceptual framework. American Journal of Health-System Pharmacy. 2019;76(18):1403-12. PubMed PMID: 31505561.

5222. Bicket MC, Fu D, Swarthout MD, White E, Nesbit SA, Monitto CL. Effect of Drug Disposal Kits and Fact Sheets on Elimination of Leftover Prescription Opioids: the DISPOSE Multi-Arm Randomized Controlled Trial. Pain medicine (Malden, Mass). 2021;22(4):961-9. doi: 10.1093/pm/pnaa431. PubMed PMID: CN-02258471.

5223. Bicket MC, Kattail D, Yaster M, Wu CL, Pronovost P. An analysis of errors, discrepancies, and variation in opioid prescriptions for adult outpatients at a teaching hospital. Journal of Opioid Management. 2017;13(1):51-7. PubMed PMID: 615089881.

5224. Bickford CL, Agarwal R, Durand J-B, Urbauer DL, Lenihan DJ. Efficacy and Safety of Ibutilide for Chemical Cardioversion of Atrial Fibrillation and Atrial Flutter in Cancer Patients. The American Journal of the Medical Sciences. 2014;347(4):277-81. doi: https://doi.org/10.1097/MAJ.0b013e31828a7656.

5225. Bickham P, Golembiewski J, Meyer T, Murray CG, Wagner D. ASHP guidelines on perioperative pharmacy services. American Journal of Health-System Pharmacy. 2019;76(12):903-820. doi: 10.1093/ajhp/zxz073. PubMed PMID: 136828651. Language: English. Entry Date: 20190608. Revision Date: 20200615. Publication Type: Article. Journal Subset: Biomedical.

5226. Bickmore TW, Trinh H, Olafsson S, O'Leary TK, Asadi R, Rickles NM, et al. Patient and consumer safety risks when using conversational assistants for medical information: an observational study of Siri, Alexa, and Google Assistant. Journal of medical Internet research. 2018;20(9):e11510.

5227. Bickston SJ, Waters HC, Dabbous O, Tang BX, Rahman MI. Administrative claims analysis of all-cause annual costs of care and resource utilization by age category for ulcerative colitis patients. Journal of Managed Care Pharmacy. 2008;14(4):352-62. doi: 10.18553/jmcp.2008.14.4.352. PubMed PMID: WOS:000256452300001.

5228. Biddle MA, Cleveland KK, O'Connor SK, Hruza H, Foster M, Nguyen E, et al. Assessing Pharmacists' Views and Barriers to Providing and Billing for Pharmacist-Provided Health Care Services. Journal of Pharmacy Technology. 2021;37(4):186-92. doi: 10.1177/87551225211021187. PubMed PMID: WOS:000657998900001.

5229. Biddle MA, Cleveland KK, O’Connor SK, Hruza H, Foster M, Nguyen E, et al. Assessing Pharmacists’ Views and Barriers to Providing and Billing for Pharmacist-Provided Health Care Services. Journal of Pharmacy Technology. 2021;37(4):186-92. doi: 10.1177/87551225211021187.

5230. Bidon D, Lecoeur A, Segui E, Seguette N, Le Mercier F, Bauler S. Suivi des patients traités par AVK : intérêt d’un relais pharmaceutique entre l’hôpital et la ville. Annales Pharmaceutiques Françaises. 2017;75(1):45-53. doi: https://doi.org/10.1016/j.pharma.2016.04.001.

5231. Bidon D, Lecoeur A, Segui E, Seguette N, Le Mercier F, Bauler S. Follow-up of patients treated by VKA: Interest of a pharmaceutical link between the hospital and the retail pharmacies. Annales Pharmaceutiques Francaises. 2017;75(1):45-53. doi: 10.1016/j.pharma.2016.04.001.

5232. Bidulka P, Vestergaard SV, Hlupeni A, Kjærsgaard A, Wong AYS, Langan SM, et al. Adverse outcomes after partner bereavement in people with reduced kidney function: Parallel cohort studies in England and Denmark. PLoS ONE. 2021;16(9 September). doi: 10.1371/journal.pone.0257255.

5233. Bidwell S, Copeland A. A model of multidisciplinary professional development for health professionals in rural Canterbury, New Zealand. Journal of Primary Health Care. 2017;9(4):292-6. doi: 10.1071/HC17049.

5234. Bidwell S, Thompson L. GPs, community pharmacists and shifting professional boundaries. The New Zealand Medical Journal (Online). 2015;128(1414):19.

5235. Bielefeldt K, Dudekula A, Levinthal DJ. The value of care: a meta-analytic approach to frame benefits, harms and costs of care in irritable bowel syndrome. Gastroenterology. 2016;150(4):S734-S5. PubMed PMID: CN-01160719.

5236. Biello KB, Grinsztejn B, Fernandes NM, Edeza A, Kamel L, Salhaney P, et al. Development of a social network- based intervention to overcome multilevel barriers to art adherence among adolescents in brazil. AIDS Education and Prevention. 2019;31(2):111-26.

5237. Bien-Aimé S. The Effects of a Same Gender and Same Race Coaching Intervention on the Resilience of Black Baccalaureate Nursing Students [Ph.D.]. United States -- Pennsylvania: Widener University; 2022.

5238. Bienert A, Meier K, Kokisheva A, Gama Brambila RA, Veraldi M, De Lima Vilaca Marques NCR, et al. Impact of COVID-19 on Mental Health of Oncology Health Care Workers and Interdisciplinary Collaboration: Results of Surveys in 2021 and 2022. European Journal of Oncology Pharmacy. 2023;6(1). doi: 10.1097/OP9.0000000000000044.

5239. Bienvenida A, Kroll C, Ruhland D, Steffenhagen A, Patterson BW, Halfpap J. Implementation and safety evaluation of autoverification for select low-risk, high-volume medications in the emergency department. American Journal of Health-System Pharmacy. 2022;79(23):2150-8. doi: 10.1093/ajhp/zxac241. PubMed PMID: 160376841. Language: English. Entry Date: 20221130. Revision Date: 20221201. Publication Type: Article.

5240. Bierle L, Yoshida C. March is Colorectal Cancer Awareness Month - What Clinicians Need to Know in 2021. Practical Gastroenterology. 2021;45(2):30-7. PubMed PMID: WOS:000635524800003.

5241. Biersteker TE, Schalij MJ, Treskes RW. Impact of Mobile Health Devices for the Detection of Atrial Fibrillation: Systematic Review. Jmir Mhealth and Uhealth. 2021;9(4). doi: 10.2196/26161. PubMed PMID: WOS:000708587700002.

5242. Bierut LJ, Heath AC, Bucholz KK, Dinwiddie SH, Madden PA, Statham DJ, et al. Major depressive disorder in a community-based twin sample: are there different genetic and environmental contributions for men and women? Archives of general psychiatry. 1999;56(6):557-63.

5243. Bierwirth R, Kostev K, Dippel FW, Fuchs S, Kotowa W. Resource utilisation and treatment costs of an intensified conventional therapy (ICT) with insulin glargine, insulin detemir or NPH insulin in patients with type 1 diabetes. Perfusion. 2010;23(3):96-103.

5244. Bieszk N, Grabner M, Wei W, Barron J, Quimbo R, Yan T, et al. Personalized care and the role of

insulin as a vehicle to optimizing treatments in diabetes care. Journal of managed care & specialty pharmacy. 2017;23(11):1160-8. doi: 10.18553/jmcp.2017.23.11.1160. PubMed PMID: CN-01430277.

5245. Bieszk N, Patel R, Heaberlin A, Wlasuk K, Zarowitz B. Detection of medication nonadherence through review of pharmacy claims data. American Journal of Health-System Pharmacy. 2003;60(4):360-6. PubMed PMID: 12625218.

5246. Bieszk N, Reynolds S, Wei W, Davis C, Kamble P, Uribe C. Evaluating the effect of a randomized controlled educational intervention targeting improved glycemic control: "act on threes" paradigm for treatment escalation of type 2 diabetes mellitus (T2DM) in managed care. Journal of managed care and specialty pharmacy Conference: 27th annual meeting and expo of the academy of managed care pharmacy, AMCP 2015 United states. 2015;21(4-a SUPPL.):S22. PubMed PMID: CN-01669757.

5247. Biezen R, Grando D, Mazza D, Brijnath B. Why do we not want to recommend influenza vaccination to young children? A qualitative study of Australian parents and primary care providers. Vaccine. 2018;36(6):859-65. doi: https://doi.org/10.1016/j.vaccine.2017.12.066.

5248. Bifari I, Alkhaldi AA, Almalki RK, Mashrah HT, Asiri OA, Altwairqi AT, et al. Use of traditional eye medicine and self-medication among population of Taif City, Saudi Arabia: a cross sectional study. World Family Medicine. 2020;18(1):43-8. doi: 10.5742/mewfm.2020.93722. PubMed PMID: WOS:000505070900007.

5249. Bifftu BB, Dachew BA, Tiruneh BT, Beshah DT. Medication administration error reporting and associated factors among nurses working at the University of Gondar referral hospital, Northwest Ethiopia, 2015. BMC nursing. 2016;15:1-7.

5250. Biga J, Taboulet F, Lapeyre-Mestre M, Duguet A-M, Sciortino V, Montastruc J-L. La prescription médicamenteuse en dénomination commune : perception des acteurs de santé en Midi-Pyrénées. Therapies. 2005;60(4):401-7. doi: https://doi.org/10.2515/therapie:2005058.

5251. Bigelow S, Schulz H, Dobish R, Chambers CR. Antineoplastic agent workplace contamination study: The Alberta Cancer Board Pharmacy perspective Phase III. Journal of Oncology Pharmacy Practice. 2009;15(3):157-60. doi: 10.1177/1078155208101097.

5252. Biggs MJ, Biggs TC. Independent Prescribing Pharmacists Supporting the Early Discharge of Patients Through Completion of Medical Discharge Summaries. Journal of Pharmacy Practice. 2020;33(2):173-5. doi: 10.1177/0897190018795950. PubMed PMID: 142293313. Language: English. Entry Date: 20200331. Revision Date: 20200331. Publication Type: Article.

5253. Bigot A, Promis A, Irazusta O, Cazal J, Delenclos L, Le Floch Meunier B, et al. Assessment of the medication system in an university hospital: risk mapping with a priori and a posteriori approaches. European journal of hospital pharmacy. 2014;21:A175-. doi: 10.1136/ejhpharm-2013-000436.428. PubMed PMID: CN-01731019.

5254. Bijlsma JWJ, Welsing PMJ, Woodworth TG, Middelink LM, Pethö-Schramm A, Bernasconi C, et al. Early rheumatoid arthritis treated with tocilizumab, methotrexate, or their combination (U-Act-Early): a multicentre, randomised, double-blind, double-dummy, strategy trial. Lancet (london, england). 2016;388(10042):343-55. doi: 10.1016/S0140-6736(16)30363-4. PubMed PMID: CN-01167910.

5255. Bijlsma JWJ, Welsing PMJ, Woodworth TG, Middelink LM, Pethö-Schramm A, Bernasconi C, et al. Early rheumatoid arthritis treated with tocilizumab, methotrexate, or their combination (U-Act-Early): a multicentre, randomised, double-blind, double-dummy, strategy trial. The Lancet. 2016;388(10042):343-55. doi: https://doi.org/10.1016/S0140-6736(16)30363-4.

5256. Bilal AI, Tilahun Z, Gebretekle GB, Ayalneh B, Hailemeskel B, Engidawork E. Current status, challenges and the way forward for clinical pharmacy service in Ethiopian public hospitals. BMC Health Services Research. 2017;17:1-11. doi: 10.1186/s12913-017-2305-1. PubMed PMID: 123166088. Language: English. Entry Date: 20180725. Revision Date: 20190708. Publication Type: journal article.

5257. Bilavsky E, Schwaber MJ, Carmeli Y. How to stem the tide of carbapenemase-producing Enterobacteriaceae?: Proactive versus reactive strategies. Current Opinion in Infectious Diseases. 2010;23(4):327-31. doi: 10.1097/QCO.0b013e32833b3571.

5258. Bild V, Bulea D, Neica S, Pavelescu MD. [Community pharmacy counseling. An important role in reducing and optimizing self-medication]. Revista medico-chirurgicală a Societăţii de Medici şi Naturalişti din Iaşi. 2009;113(2):589-92.

5259. Bilgener E, Bulut S. Evaluation of electronic prescriptions in Turkey: A community pharmacy perspective. Health Policy and Technology. 2021;10(1):52-9. doi: https://doi.org/10.1016/j.hlpt.2020.11.003.

5260. BiLgener E, Ünal A. Determining of satisfaction from pharmacy services corum city sample. Ankara Universitesi Eczacilik Fakultesi Dergisi. 2019;43(3):259-73. doi: 10.33483/jfpau.480903.

5261. Bilkhu P. Management of allergic conjunctivitis and dry eye [Ph.D.]. England: Aston University (United Kingdom); 2014.

5262. Bilkhu P, Wolffsohn JS, Taylor D, Gibson E, Hirani B, Naroo SA. The management of ocular allergy in community pharmacies in the United Kingdom. International Journal of Clinical Pharmacy. 2013;35(2):190-4. doi: 10.1007/s11096-012-9742-z. PubMed PMID: WOS:000318949500006.

5263. Bilkhu PS, Wolffsohn JS, Tang GW, Naroo SA. Management of dry eye in UK pharmacies. Contact Lens and Anterior Eye. 2014;37(5):382-7. doi: 10.1016/j.clae.2014.06.002.

5264. Billeter M. Drug-resistant pneumococci: What can pharmacists do about it? American Journal of Health-System Pharmacy. 2004;61(22):2371. doi: 10.1093/ajhp/61.22.2371.

5265. Billings DL, Walker D, Mainero del Paso G, Clark KA, Dayananda I. Pharmacy worker practices related to use of misoprostol for abortion in one Mexican state. Contraception. 2009;79(6):445-51. doi: https://doi.org/10.1016/j.contraception.2008.12.011.

5266. Billstein-Leber M, Carrillo COLJD, Cassano AT, Moline K, Robertson JJ. ASHP Guidelines on Preventing Medication Errors in Hospitals. American Journal of Health-System Pharmacy. 2018;75(19):1493-517. doi: 10.2146/ajhp170811. PubMed PMID: 132037195. Language: English. Entry Date: 20181003. Revision Date: 20190405. Publication Type: Article.

5267. Billups MW. Modeling, Control and Material Traceability in Continuous Pharmaceutical Manufacturing [M.S.]. United States -- New Jersey: Rutgers The State University of New Jersey, School of Graduate Studies; 2018.

5268. Billups SJ, Delate T, Newlon C, Schwiesow S, Jahnke R, Nadrash A. Outcomes of a pharmacist-managed medication refill program. Journal of the American Pharmacists Association. 2013;53(5):505-12. doi: 10.1331/JAPhA.2013.13008.

5269. Billups SJ, Okano G, Malone D, Carter BL, Valuck R, Barnette DJ, et al. Assessing the structure and process for providing pharmaceutical care in Veterans Affairs medical centers. American Journal of Health-System Pharmacy. 2000;57(1):29-39. doi: 10.1093/ajhp/57.1.29. PubMed PMID: WOS:000084761800008.

5270. Bilodeau V, Huot J, Perreault C, Haraoui LP, Delorme C, Poudrette J, et al. VANCOmycin dose adjustments comparing trough levels to the ratio of the area under de curve to the minimum inhibitory concentration method using a BAYESian approach: a feasibility study. Journal of clinical pharmacy and therapeutics. 2022;47(12):2335-44. doi: 10.1111/jcpt.13818. PubMed PMID: CN-02503894.

5271. Bilora F, Petrobelli F, Leo T, Fioretti M, Boccioletti V. Quality evaluation of health care offered by an Internal Medicine Department. Panminerva Medica. 2001;43(1):57-62.

5272. Biltaji E, Yoo M, Jennings BT, Leiser JP, McAdam-Marx C. Outcomes associated with pharmacist-led diabetes collaborative drug therapy management in a medicaid population. Journal of Pharmaceutical Health Services Research. 2017;8(1):59-62. doi: 10.1111/jphs.12162.

5273. Biltoft J, Finneman L. Clinical and financial effects of smart pump--electronic medical record interoperability at a hospital in a regional health system. American Journal of Health-System Pharmacy. 2018;75(14):1064-8. doi: 10.2146/ajhp161058. PubMed PMID: 130639256. Language: English. Entry Date: 20180714. Revision Date: 20190130. Publication Type: Article. Journal Subset: Biomedical.

5274. Biltoft J, Finneman L. Clinical and financial effects of smart pump-electronic medical record interoperability at a hospital in a regional health system. American Journal of Health-System Pharmacy. 2018;75(14):1064-8. PubMed PMID: 29987060.

5275. Bin Abdulhak AA, Altannir MA, Almansor MA, Almohaya MS, Onazi AS, Marei MA, et al. Non prescribed sale of antibiotics in Riyadh, Saudi Arabia: A Cross Sectional Study. Bmc Public Health. 2011;11. doi: 10.1186/1471-2458-11-538. PubMed PMID: WOS:000293328900001.

5276. Bin Ng D, Espinosa R, Johnson SJ, Walker D, Gooch K. The impact of persistence with mirabegron usage vs switching to onabotulinumtoxinA on healthcare costs and resource utilization in patients with overactive bladder in the United States. Journal of Medical Economics. 2017;20(12):1272-80. doi: 10.1080/13696998.2017.1367300. PubMed PMID: WOS:000418053100009.

5277. Binaso KA, Skelton JB, Amer Pharmacists A. DOTx.MED: Pharmacist-delivered interventions to improve care for patients with diabetes. Journal of the American Pharmacists Association. 2012;52(1):25-33. doi: 10.1331/JAPhA.2012.12501. PubMed PMID: WOS:000299523100005.

5278. Bindler RJ. Inpatient and outpatient pharmacy monitoring of oral antineoplastic medications. Hospital Pharmacy. 2015;50(2):91-2.

5279. Bindoff I, Ling T, Bereznicki L, Westbury J, Chalmers L, Peterson G, et al. A computer simulation of community pharmacy practice for educational use. American journal of pharmaceutical education. 2014;78(9).

5280. Bing-Jonsson PC, Hofoss D, Kirkevold M, Bjørk IT, Foss C. Sufficient competence in community elderly care? Results from a competence measurement of nursing staff. BMC Nursing. 2016;15(1). doi: 10.1186/s12912-016-0124-z.

5281. Bingham J, Silva-Almodovar A, Lee H, Benson C, Michael R, Azurin CM, et al. The role of the pharmacist in mental health: An investigation of the impact of pharmacist-led interventions on psychotropic medication adherence in patients with diabetes. Journal of the American Pharmacists Association: JAPhA. 2020;60(4):e58-e63. PubMed PMID: 32113946.

5282. Bingham JM, Stanislaw J, Warholak T, Scovis N, Axon DR, Turgeon J, et al. Assessment of Glycosylated Hemoglobin Outcomes Following an Enhanced Medication Therapy Management Service via Telehealth. International Journal of Environmental Research & Public Health [Electronic Resource].

2021;18(12):18. PubMed PMID: 34207164.

5283. Binkhorst L, Mathijssen RHJ, Van Herk-Sukel MPP, Bannink M, Jager A, Wiemer EAC, et al. Unjustified prescribing of CYP2D6 inhibiting SSRIs in women treated with tamoxifen. Breast Cancer Research and Treatment. 2013;139(3):923-9. doi: 10.1007/s10549-013-2585-z.

5284. Binkley D, Waller L, Potts L, Bronstein J. Pharmacists as HIV/AIDS information resources: Survey of Alabama pharmacists. AIDS Education and Prevention. 1995;7(5):455-66.

5285. Binkley D, Waller L, Potts L, Bronstein J. PHARMACISTS AS HIV AIDS INFORMATION RESOURCES - SURVEY OF ALABAMA PHARMACISTS. Aids Education and Prevention. 1995;7(5):455-66. PubMed PMID: WOS:A1995RY81500007.

5286. Binns P. The impact of the electronic health record on patient safety: an Alberta perspective. Healthcare Papers. 2004;5(3):47-51. PubMed PMID: 106512632. Language: English. Entry Date: 20050909. Revision Date: 20150711. Publication Type: Journal Article.

5287. Binobaid S, Almeziny M, Fan I-S. Using an integrated information system to reduce interruptions and the number of non-relevant contacts in the inpatient pharmacy at tertiary hospital. Saudi Pharmaceutical Journal. 2017;25(5):760-9. doi: https://doi.org/10.1016/j.jsps.2016.11.005.

5288. Binobaid S, Fan I-S, Almeziny M. Investigation Interoperability Problems in Pharmacy Automation: A Case Study in Saudi Arabia. Procedia Computer Science. 2016;100:329-38. doi: https://doi.org/10.1016/j.procs.2016.09.166.

5289. Binswanger IA, Rinehart D, Mueller SR, Narwaney KJ, Stowell M, Wagner N, et al. Naloxone Co-Dispensing with Opioids: a Cluster Randomized Pragmatic Trial. Journal of general internal medicine. 2022;37(11):2624-33. doi: 10.1007/s11606-021-07356-6. PubMed PMID: CN-02373045.

5290. Biola H, Deyo J, Hayes T, Small L, Chaplin J, Pak-Harvey E, et al. Reaching the Hard-to-Reach: Outcomes of the Severe Hypertension Outreach Intervention. American Journal of Preventive Medicine. 2020;59(5):725-32. doi: https://doi.org/10.1016/j.amepre.2020.05.030.

5291. Biraben A, De Toffol B, Semah F, Rouaud T. Utilisation des médicaments génériques des anti-épileptiques en France : résultats d'une enquête auprès des neurologues et revue de la littérature. Revue Neurologique. 2007;163(4):455-61. doi: https://doi.org/10.1016/S0035-3787(07)90421-X.

5292. Birabwa C, Murison J, Evans V, Obua C, Agaba A, Waako P, et al. The availability of six tracer medicines in private medicine outlets in Uganda. Journal of Pharmaceutical Policy and Practice. 2014;7(1):1-8. doi: 10.1186/s40545-014-0018-y.

5293. Biradar SS, Kapatae R, Reddy S, Raju SA. Assesment of pharmacist mediated patient couensilling on hypertension incompliance with quality of life in South Indian City. International research journal of pharmacy. 2012;3(1):206-9. PubMed PMID: CN-00868860.

5294. Birarra MK, Baye E, Tesfa W, Kifle ZD. Knowledge of cardiovascular disease risk factors, practice, and barriers of community pharmacists on cardiovascular disease prevention in North West Ethiopia. Metabolism Open. 2022;16:100219. doi: https://doi.org/10.1016/j.metop.2022.100219.

5295. Bird K, Socías ME, Ti L. Integrating hepatitis C and addiction care for people who inject drugs in the era of direct-acting antiviral therapy. International Journal of Drug Policy. 2018;59:1-2. doi: 10.1016/j.drugpo.2018.06.002.

5296. Bird R, Ozer-Erdogdu I, Aslan M, Tezcan-Guntekin H. Healthcare Provider Perspectives on Digital and Interprofessional Medication Management in Chronically Ill Older Adults of Turkish Descent in Germany: A Qualitative Structuring Content Analysis. Frontiers in Public Health. 2022;10:838427. PubMed PMID: 35719659.

5297. Bird ST, Flowers N, Zhao Y, McKean S, Izem R, Wernecke M, et al. Healthy User Bias in Comparative Safety Studies for Brand-Name vs. Generic Products: The Example of Warfarin. Clinical Pharmacology and Therapeutics. 2019;106(5):1037-45. doi: 10.1002/cpt.1498.

5298. Bird ST, Flowers N, Zhao YQ, McKean S, Izem R, Wernecke M, et al. Healthy User Bias in Comparative Safety Studies for Brand-Name vs. Generic Products: The Example of Warfarin. Clinical Pharmacology & Therapeutics. 2019;106(5):1037-45. doi: 10.1002/cpt.1498. PubMed PMID: WOS:000474066400001.

5299. Birdsall AD, Kappenman AM, Covey BT, Rim MH. Implementation and impact assessment of integrated electronic prior authorization in an academic health system. Journal of the American Pharmacists Association. 2020;60(4):e93-e9. doi: https://doi.org/10.1016/j.japh.2020.01.012.

5300. Birkhead GS, Klein SJ, Candelas AR, O'Connell DA, Rothman JR, Feldman IS, et al. Integrating multiple programme and policy approaches to hepatitis C prevention and care for injection drug users: A comprehensive approach. International Journal of Drug Policy. 2007;18(5):417-25. doi: 10.1016/j.drugpo.2007.01.013.

5301. Birring SS, Brew J, Kilbourn A, Edwards V, Wilson R, Morice AH. Rococo study: a real-world evaluation of an over-the-counter medicine in acute cough (a multicentre, randomised, controlled study). BMJ open. 2017;7(1):e014112. doi: 10.1136/bmjopen-2016-014112. PubMed PMID: CN-01443814.

5302. Birring SS, Brew J, Kilbourn T, Morice AH. A randomised controlled trial of over the counter medicine CS1002 for acute cough. Thorax. 2016;71:A17-A8. doi: 10.1136/thoraxjnl-2016-209333.35. PubMed PMID: CN-01360148.

5303. Birsan M, Dragan M, Stan CD, Cristofor AC, Palimariciuc M, Stan CI, et al. PATIENT SATISFACTION REGARDING COMPOUNDED PHARMACEUTICAL PRODUCTS AND IMPLICATIONS ON PHARMACEUTICAL PRACTICE MANAGEMENT. Farmacia. 2021;69(4):806-12. doi: 10.31925/farmacia.2021.4.24. PubMed PMID: WOS:000687039600024.

5304. Bîrsan M, Focșa AV, Cristofor AC, Scripcariu SI, Antonoaea P, Vlad RA, et al. Healthcare Service Quality Evaluated Using the Degree of Satisfaction of Patients in Romanian Community Pharmacies. Healthcare (Switzerland). 2023;11(23). doi: 10.3390/healthcare11233038.

5305. Birt L, Dalgarno L, Bond CM, Holland R, Alldred DP, Hughes C, et al. Evaluation of a training programme for Pharmacist Independent Prescribers in a care home medicine management intervention. BMC medical education. 2022;22(1):551. doi: 10.1186/s12909-022-03575-5. PubMed PMID: CN-02426118.

5306. Birt L, Dalgarno L, Poland F, Wright D, Bond C. What happens when pharmacist independent prescribers lead on medicine management in older people's care homes: A qualitative study. BMJ Open. 2023;13(10):e068678. doi: https://dx.doi.org/10.1136/bmjopen-2022-068678. PubMed PMID: 2028559147.

5307. Birt L, Dalgarno L, Wright DJ, Alharthi M, Inch J, Spargo M, et al. Process evaluation for the Care Homes Independent Pharmacist Prescriber Study (CHIPPS). BMC health services research. 2021;21(1):1041. doi: 10.1186/s12913-021-07062-3. PubMed PMID: CN-02330966.

5308. Birt L, Dalgarno L, Wright DJ, Alharthi M, Inch J, Spargo M, et al. Process evaluation for the Care

Homes Independent Pharmacist Prescriber Study (CHIPPS). BMC Health Services Research. 2021;21(1):1041. PubMed PMID: 34600542.

5309. Birt L, Wright DJ, Blacklock J, Bond CM, Hughes CM, Alldred DP, et al. Enhancing deprescribing: A qualitative understanding of the complexities of pharmacist-led deprescribing in care homes. Health & Social Care in the Community. 2022;30(6):e6521-e31. doi: 10.1111/hsc.14099. PubMed PMID: 160813039. Language: English. Entry Date: 20221222. Revision Date: 20221222. Publication Type: Article.

5310. Birt L, Wright DJ, Blacklock J, Bond CM, Hughes CM, Alldred DP, et al. Enhancing deprescribing: A qualitative understanding of the complexities of pharmacist-led deprescribing in care homes. Health & Social Care in the Community. 2022;30(6):e6521-e31. PubMed PMID: 36336895.

5311. Birtcher KK, Bowden C, Ballantyne CM, Huyen M. Strategies for implementing lipid-lowering therapy: pharmacy-based approach. The American Journal of Cardiology. 2000;85(3, Supplement 1):30-5. doi: https://doi.org/10.1016/S0002-9149(99)00936-4.

5312. Biscaldi L. Partnering for Success: How the Good Neighbor Pharmacy Business Coach–Pharmacy Partnership Helped Propel One Independent Pharmacy to Success. Drug Topics. 2022;166(9):38-9.

5313. Bischof E. Mitigating COVID-19 Mortality and Morbidity in China's Aging Population: A Focus on Available Medications and Future Developments. Aging & Disease. 2023;14(6):1967-76. doi: 10.14336/AD.2023.0318. PubMed PMID: 174570611.

5314. Biset N, Lelubre M, Senterre C, Amighi K, Bugnon O, Schneider MP, et al. Assessment of medication adherence and responsible use of isotretinoin and contraception through belgian community pharmacies by using pharmacy refill data. Patient Preference and Adherence. 2018;12:153-61. doi: 10.2147/PPA.S149355.

5315. Bishop A, Zwicker B. Report from the 2018 National Summit on Wicked Problems in Community Pharmacy. Canadian Pharmacists Journal. 2019;152(3):158-9. doi: 10.1177/1715163519840374.

5316. Bishop AC, Boyle TA, Morrison B, Barker JR, Zwicker B, Mahaffey T, et al. Public perceptions of pharmacist expanded scope of practice services in Nova Scotia. Canadian Pharmacists Journal. 2015;148(5):274-83. doi: 10.1177/1715163515596757.

5317. Bishop C, Yacoob Z, Knobloch MJ, Safdar N. Community pharmacy interventions to improve antibiotic stewardship and implications for pharmacy education: A narrative overview. Research in Social and Administrative Pharmacy. 2019;15(6):627-31. doi: https://doi.org/10.1016/j.sapharm.2018.09.017.

5318. Bishop LD, Rosenberg-Yunger ZRS. Pharmacists expanded role in providing care for opioid use disorder during COVID-19: A qualitative study exploring pharmacists' experiences. Drug and Alcohol Dependence. 2022;232:109303. doi: https://doi.org/10.1016/j.drugalcdep.2022.109303.

5319. Bishop LD, Rosenberg-Yunger ZRS, Dattani S. Pharmacists' perceptions of the Canadian opioid regulatory exemptions on patient care and opioid stewardship. Canadian Pharmacists Journal. 2021;154(6):394-403. doi: 10.1177/17151635211034530.

5320. Bisht VK, Uniyal RC. Why primary processing of herbal raw drugs is important. Current Science. 2020;119(10):1699-702. doi: 10.18520/cs/v119/i10/1699-1702. PubMed PMID: WOS:000592585300027.

5321. Bislev LS, Langagergaard Rodbro L, Rolighed L, Sikjaer T, Rejnmark L. Effects of Vitamin D3 Supplementation on Muscle Strength, Mass, and Physical Performance in Women with Vitamin D Insufficiency: a Randomized Placebo-Controlled Trial. Calcified tissue international. 2018;103(5):483-93. doi: 10.1007/s00223-018-0443-z. PubMed PMID: CN-01611508.

5322. Bislew HD, Sorensen TD. Use of focus groups as a tool to enhance a pharmaceutical care practice. Journal of the American Pharmacists Association. 2003;43(3):424-34. doi: 10.1331/154434503321831157.

5323. Bismuth M, Oustric S, Boyer P, Escourrou B, Brillac T, Mesthe P, et al. La pharmacie familiale : enquête auprès d'un échantillon de patients en Midi-Pyrénées. Therapies. 2011;66(2):131-4. doi: https://doi.org/10.2515/therapie/2011008.

5324. Bisognano JD, Bakris G, Nadim MK, Sanchez L, Kroon AA, Schafer J, et al. Baroreflex activation therapy lowers blood pressure in patients with resistant hypertension: results from the double-blind, randomized, placebo-controlled rheos pivotal trial. Journal of the American College of Cardiology. 2011;58(7):765-73.

5325. Bissel P, Anderson C, Blenkinsopp A. Has community pharmacy a role in dealing with health inequalities? Pharmaceutical Journal. 2002;267(7179):880.

5326. Bissell P, Blenkinsopp A, Short D, Mason L. Patients' experiences of a community pharmacy-led medicines management service. Health & Social Care in the Community. 2008;16(4):363-9. doi: 10.1111/j.1365-2524.2007.00749.x. PubMed PMID: WOS:000256970600004.

5327. Bissell P, Savage I, Anderson C. A qualitative study of pharmacists' perspectives on the supply of emergency hormonal contraception via patient group direction in the UK. Contraception. 2006;73(3):265-70. doi: 10.1016/j.contraception.2005.07.017. PubMed PMID: WOS:000235582900010.

5328. Bissell P, Ward PR, Noyce PR. Appropriateness measurement: application to advice-giving in community pharmacies. Social Science & Medicine. 2000;51(3):343-59. doi: https://doi.org/10.1016/S0277-9536(99)00458-X.

5329. Bissell P, Ward PR, Noyce PR. Mapping the contours of risk: Consumer perceptions of non-prescription medicines. Journal of Social and Administrative Pharmacy. 2000;17(3):136-42.

5330. Bissell P, Ward PR, Noyce PR. The dependent consumer: Reflections on accounts of the risks of non-prescription medicines. Health. 2001;5(1):5-30. doi: 10.1177/136345930100500101.

5331. Bissett SM, Sturrock A, Carrozzo M, Lish R, Howe D, Mountain S, et al. Is the early identification and referral of suspected head and neck cancers by community pharmacists feasible? A qualitative interview study exploring the views of patients in North East England. Health Expectations. 2023;26(5):2089-97. doi: 10.1111/hex.13816.

5332. Bisson C, Buxeraud J. Prise en charge de l'halitose. Actualités Pharmaceutiques. 2016;55(558):47-50. doi: https://doi.org/10.1016/j.actpha.2016.06.018.

5333. Bissonnette S, Goeres LM, Lee DSH, editors. Pharmacy density in rural and urban communities in the state of Oregon and the association with hospital readmission rates. Journal of the American Pharmacists Association; 2016.

5334. Bissonnette S, Goeres LM, Lee DSH. Pharmacy density in rural and urban communities in the state of Oregon and the association with hospital readmission rates. Journal of the American Pharmacists Association. 2016;56(5):533-7. doi: 10.1016/j.japh.2016.05.008. PubMed PMID: WOS:000389758700010.

5335. Bissonnier J, Bonnici A, Crotet C, Boudaud-Matjasec V, Drouard S. A community and hospital cooperation approach by Experience Feedback Committee between Hospital and community Pharmacies. Journal de Pharmacie Clinique. 2021;40(1):32-40. doi: 10.1684/jpc.2021.0457.

5336. Biswas A, Sinha N, Ray K, Tripathi SK. A study on drug use and medication management perspectives among elderly and the impact of professional oversight. Journal of Clinical and Diagnostic Research. 2018;12(5):FC01-FC7. doi: 10.7860/JCDR/2018/32786.11518.

5337. Biszewski M, Nitzki-George D, Zhou Y. Comparison of warfarin time in the therapeutic range at a pharmacist-run anticoagulation clinic and the RE-LY trial. American journal of health-system pharmacy. 2015;72(7):557-62. doi: 10.2146/ajhp130800. PubMed PMID: CN-01110104.

5338. Bitan Y, Nunnally ME. Shape Matters: A Neglected Feature of Medication Safety: Why Regulating the Shape of Medication Containers Can Improve Medication Safety. Journal of Medical Systems. 2023;47(1):1-5. doi: 10.1007/s10916-022-01905-0. PubMed PMID: 161820951.

5339. Bitencourt G, Stella Zanotto B, Tortato C, Martinbiancho J, Gonzatti J, Jacoby T, et al. PNS189 A CAPACITY ASSESSMENT FOR A CLINICAL PHARMACY SERVICE OF A PUBLIC-SCHOOL HOSPITAL: A DISCRETE-EVENT SIMULATION CASE STUDY. Value in Health. 2019;22:S317. doi: https://doi.org/10.1016/j.jval.2019.04.1546.

5340. Bitomsky M. Compounding standards. Journal of Complementary Medicine. 2008;7(5):29-+. PubMed PMID: WOS:000258941700021.

5341. Bitonti M, Patel P, Dickinson R, Knapp P, Blalock SJ. The effect of counseling on willingness to use a hypothetical medication and perceptions of medication safety. Research in Social and Administrative Pharmacy. 2018;14(3):295-302. doi: https://doi.org/10.1016/j.sapharm.2017.03.058.

5342. Bitter K, Pehe C, Kruger M, Heuer G, Quinke R, Jaehde U. Pharmacist-led medication reviews for geriatric residents in German long-term care facilities. BMC Geriatrics. 2019;19(1):39. PubMed PMID: 30744564.

5343. Bix LL. Toward a performance standard for typeface legibility: The Lockhart Legibility Instrument [Ph.D.]. United States -- Michigan: Michigan State University; 2001.

5344. Biyase N, Perrie H, Scribante J, Muteba M, Chetty S. Stress ulcer prophylaxis use in critical care units at public hospitals in Johannesburg, South Africa. Southern African Journal of Critical Care. 2021;37(1):16-20. doi: 10.7196/SAJCC.2021.v37i1.439. PubMed PMID: 151704768. Language: English. Entry Date: 20210808. Revision Date: 20210809. Publication Type: Article.

5345. Bizimana T, Hagen N, Gnegel G, Kayumba PC, Heide L. Quality of oxytocin and misoprostol in health facilities of Rwanda. PLoS ONE. 2021;16(1 January). doi: 10.1371/journal.pone.0245054.

5346. Bizimana T, Kagisha V, Nyandwi JB, Nyirimigabo AK, Muganga R, Mukanyangezi MF, et al. Investigation of the Quality of the 12 Most-Used Antibiotics Available in Retail Private Pharmacies in Rwanda. Antibiotics. 2022;11(3). doi: 10.3390/antibiotics11030329.

5347. Bizimana T, Kayumba PC, Heide L. Prices, availability and affordability of medicines in Rwanda. PLoS ONE. 2020;15(8 August). doi: 10.1371/journal.pone.0236411.

5348. Bizovi KE, Beckley BE, McDade MC, Adams AL, Lowe RA, Zechnich AD, et al. The effect of computer-assisted prescription writing on emergency department prescription errors. Academic Emergency Medicine. 2002;9(11):1168-75. PubMed PMID: 106840398. Language: English. Entry Date: 20030613. Revision Date: 20150711. Publication Type: Journal Article.

5349. Bjerkeli PJ, Skoglund I, Holmgren K. Does early identification of high work related stress affect pharmacological treatment of primary care patients? - analysis of Swedish pharmacy dispensing data in a

randomised control study. BMC family practice. 2020;21(1):70. doi: 10.1186/s12875-020-01140-x. PubMed PMID: CN-02120638.

5350. Bjerrum A, Garcia-Sangenis A, Modena D, Cordoba G, Bjerrum L, Chalkidou A, et al. Health alliance for prudent prescribing and yield of antibiotics in a patient-centred perspective (HAPPY PATIENT): a before-and-after intervention and implementation study protocol. BMC Primary Care. 2022;23(1):102. PubMed PMID: 35501712.

5351. Bjerrum A, Garcia-Sangenis A, Modena D, Cordoba G, Bjerrum L, Chalkidou A, et al. Health alliance for prudent prescribing and yield of antibiotics in a patient-centred perspective (HAPPY PATIENT): a before-and-after intervention and implementation study protocol. Bmc Primary Care. 2022;23(1). doi: 10.1186/s12875-022-01710-1. PubMed PMID: WOS:000789811100005.

5352. Bjerrum L, Foged A. Patient information leaflets-helpful guidance or a source of confusion? Pharmacoepidemiology and Drug Safety. 2003;12(1):55-9. doi: 10.1002/pds.795.

5353. Bjerrum OJ, Gautam Y, Honore PH, Vana V. Drug-drug combinations revisited. European Journal of Hospital Pharmacy-Science and Practice. 2014;21(1):8-12. doi: 10.1136/ejhpharm-2013-000390. PubMed PMID: WOS:000337695300004.

5354. Björkman IK, Sanner MA, Bernsten CB. Comparing 4 classification systems for drug-related problems: Processes and functions. Research in Social and Administrative Pharmacy. 2008;4(4):320-31. doi: https://doi.org/10.1016/j.sapharm.2007.10.006.

5355. Björnsdottir I, Granas AG, Bradley A, Norris P. A systematic review of the use of simulated patient methodology in pharmacy practice research from 2006 to 2016. International Journal of Pharmacy Practice. 2020;28(1):13-25. doi: 10.1111/ijpp.12570.

5356. Bjornson DC. Interpretation of drug risk and benefit: individual and population perspectives. Annals of Pharmacotherapy. 2004;38(4):694-9. PubMed PMID: 14990777.

5357. Bjornson L, Bucevska M, Tilley P, Verchere C. Is it safe to re-access sodium bicarbonate bottles for use in minor surgery? Journal of Pediatric Surgery. 2018;53(11):2290-3. doi: https://doi.org/10.1016/j.jpedsurg.2018.04.005.

5358. Bjornson S, Grindeland CJ, Werremeyer AB. Impact of Implementing Screening and Interventions to Target Prevention of Opioid Misuse and Accidental Overdose in the Inpatient Setting. Journal of Pharmacy Practice. doi: 10.1177/08971900221144183. PubMed PMID: WOS:000893612700001.

5359. Björnsson ES, Bergmann OM, Björnsson HK, Kvaran RB, Olafsson S. Incidence, presentation, and outcomes in patients with drug-induced liver injury in the general population of Iceland. Gastroenterology. 2013;144(7):1419-25. e3.

5360. Blachly JS, Ruppert AS, Zhao W, Long S, Flynn J, Flinn I, et al. Immunoglobulin transcript sequence and somatic hypermutation computation from unselected RNA-seq reads in chronic lymphocytic leukemia. Proceedings of the National Academy of Sciences. 2015;112(14):4322-7.

5361. Blachman NL, Blaum CS. Integrating Care Across Disciplines. Clinics in Geriatric Medicine. 2016;32(2):373-83. doi: https://doi.org/10.1016/j.cger.2016.01.010.

5362. Black E, Cartwright A, Bakharaiba S, Al-Mekaty E, Alsahan D. A qualitative study of pharmacists' perceptions of, and recommendations for improvement of antibiotic use in Qatar. International Journal of Clinical Pharmacy. 2014;36(4):787-94. doi: 10.1007/s11096-014-9960-7.

5363. Black E, Murphy AL, Gardner DM. Community pharmacist services for people with mental illnesses: Preferences, satisfaction, and stigma. Psychiatric Services. 2009;60(8):1123-7. doi: 10.1176/ps.2009.60.8.1123.

5364. Black K. Emergency contraception as a sexual health intervention: access and clinical outcomes through pharmacy and community provision [Ph.D.]. England: University of London, London School of Hygiene and Tropical Medicine (United Kingdom); 2008.

5365. Black K, Anderson C, Kubba A, Wellings K. Involving pharmacists in sexual health research: Experience from an emergency contraception study. Journal of Family Planning and Reproductive Health Care. 2009;35(1):41-3. doi: 10.1783/147118909787072478.

5366. Black L, Kulkarni D. Perspectives of Oncology Nursing and Investigational Pharmacy in Oncology Research. Seminars in Oncology Nursing. 2020;36(2). doi: 10.1016/j.soncn.2020.151004. PubMed PMID: WOS:000531639300008.

5367. Black PE, Misson J, Bracegirdle L. The formative developmental evaluation of an online module in health promotion using asynchronous computer-mediated communication. Pharmacy Education. 2003;3(3):195-204. doi: 10.1080/15602210310001623160.

5368. Black RM, Hughes TD, Ma F, Hudzik AA, Shepherd G, Ferreri S, et al. Systematic review of community pharmacist administration of long-acting injectable antipsychotic medications. Journal of the American Pharmacists Association. 2022. doi: https://doi.org/10.1016/j.japh.2022.08.006.

5369. Black RM, Hughes TD, Ma F, Hudzik AA, Shepherd G, Ferreri S, et al. Systematic review of community pharmacist administration of long-acting injectable antipsychotic medications. Journal of the American Pharmacists Association. 2023;63(3):742-50. e3.

5370. Black RM, Hughes TD, Ma F, Hudzik AA, Shepherd G, Ferreri S, et al. Systematic review of community pharmacist administration of long-acting injectable antipsychotic medications. Journal of the American Pharmacists Association. 2023. doi: 10.1016/j.japh.2022.08.006.

5371. Black RM, Ma FY, Hudzik AA, Shepherd G, Ferreri S, Ozawa S. Systematic review of community pharmacist administration of long-acting injectable antipsychotic medications. Journal of the American Pharmacists Association. 2023;63(3):742-+. doi: 10.1016/j.japh.2022.08.006. PubMed PMID: WOS:000998330500001.

5372. Blackburn DF, Evans C, Lamb DA, Taylor JG, Skilton K. Cardiovascular risk reduction strategies in community pharmacy settings need real world angle. Canadian Pharmacists Journal. 2007;140(5):295-7. doi: 10.3821/1913-701X(2007)140[295:CRRSIC]2.0.CO;2.

5373. Blackburn DF, Evans CD, Eurich DT, Mansell KD, Jorgenson DJ, Taylor JG, et al. Community Pharmacists Assisting in Total Cardiovascular Health (CPATCH): A Cluster-Randomized, Controlled Trial Testing a Focused Adherence Strategy Involving Community Pharmacies. Pharmacotherapy. 2016;36(10):1055-64. doi: 10.1002/phar.1831.

5374. Blackburn DF, Evans CD, Eurich DT, Mansell KD, Jorgenson DJ, Taylor JG, et al. Community Pharmacists Assisting in Total Cardiovascular Health (CPATCH): a Cluster-Randomized, Controlled Trial Testing a Focused Adherence Strategy Involving Community Pharmacies. Pharmacotherapy. 2016;36(10):1055-64. doi: 10.1002/phar.1831. PubMed PMID: CN-01411183.

5375. Blackburn DF, Evans CD, Eurich DT, Remillard AJ, Shevchuk YM, Blackburn DF. The Impact of First-Fill Discontinuations on Non-Adherence to Antihypertensive Therapy: An Observational Study.

Circulation. 2011;124(21). PubMed PMID: WOS:000299738704059.

5376. Blackburn DF, Fowler S, Crawley A, Gerwing S, Alcorn J, Mansell H, et al. An Advanced Pharmacy Practice Experience for Community Pharmacies Based on a Clinical Intervention Targeting Patients With Inflammatory Bowel Disease. Journal of Pharmacy Practice. 2021;34(1):51-7. doi: 10.1177/0897190019857410.

5377. Blackburn DF, Tran D, Quiring C. Evaluation of a refill synchronization program in two community pharmacies. Journal of the American Pharmacists Association. 2016;56(6):656-9. doi: https://doi.org/10.1016/j.japh.2016.06.015.

5378. Blackburn DF, Tran D, Quiring C, editors. Evaluation of a refill synchronization program in two community pharmacies. Journal of the American Pharmacists Association; 2016.

5379. Blackburn GL, Hutter MM, Harvey AM, Apovian CM, Boulton HR, Cummings S, et al. Expert panel on weight loss surgery: executive report update. Obesity. 2009;17(5):842-62.

5380. Blackmer A, Phan H, Ziniel S, Wright G, Borgelt L. Evaluation of clinical pharmacy services in United States pediatric ambulatory care settings. JACCP Journal of the American College of Clinical Pharmacy. 2020;3(1):265-6.

5381. Blackmer J, Amoline K, Amancher J, Vogan E, Zimmer H, Borton M, et al. Leveraging advanced preparation of oncology medications: Decreasing turnaround-times in an outpatient infusion center. Journal of Oncology Pharmacy Practice. 2021;27(6):1454-60. doi: 10.1177/1078155220960222. PubMed PMID: WOS:000573245000001.

5382. Blackmer J, Lindahl E, Strahl A, Schadler A, Freeman PR. Regulating gabapentin as a drug of abuse: A survey study of Kentucky community pharmacists. Journal of the American Pharmacists Association. 2019;59(3):379-82. doi: https://doi.org/10.1016/j.japh.2018.12.018.

5383. Blackstone EA, Joseph PF. The economics of biosimilars. American health & drug benefits. 2013;6(8):469.

5384. Blackwell SA. An empirical examination of customer loyalty [Ph.D.]. United States -- Mississippi: The University of Mississippi; 1998.

5385. Blade J, Calleja MÁ, Lahuerta JJ, Poveda JL, De Paz HD, Lizán L. Defining a set of standardised outcome measures for newly diagnosed patients with multiple myeloma using the Delphi consensus method: The IMPORTA project. BMJ Open. 2018;8(2). doi: 10.1136/bmjopen-2017-018850.

5386. Bladh L, Ottosson E, Karlsson J, Klintberg L, Wallerstedt SM. Effects of a clinical pharmacist service on health-related quality of life and prescribing of drugs: a randomised controlled trial. BMJ quality & safety. 2011;20(9):738-46. doi: 10.1136/bmjqs.2009.039693. PubMed PMID: CN-00812842.

5387. Blain L, Rappaport P. Pharmaceutical care implementation in a community teaching hospital. Canadian Journal of Hospital Pharmacy. 1996;49(2):72-9.

5388. Blaine K, Wright J, Pinkham A, O'Neill M, Wilkerson S, Rogers J, et al. Medication Order Errors at Hospital Admission Among Children With Medical Complexity. Journal of Patient Safety. 2022;18(1):e156-e62. doi: 10.1097/PTS.0000000000000719. PubMed PMID: 154310793. Language: English. Entry Date: 20220201. Revision Date: 20220201. Publication Type: Article.

5389. Blair M, Menon A. Community Pharmacy Use by Children across Europe: A Narrative Literature

Review. Pharmacy. 2018;6(2). doi: 10.3390/pharmacy6020051. PubMed PMID: WOS:000436509700027.

5390. Blair R. Medication transformation: pharmacists on the floor. A Midwest healthcare system takes bar code technology to new heights, as part of an enterprise initiative to optimize patient safety. Health Management Technology. 2004;25(10):26, 8, 30 passim. PubMed PMID: 15508685.

5391. Blair SM, Cournoyer RM, Newcomb MR, Owen JW. Novel treatment of gastric outlet obstruction secondary to incarcerated inguinal hernia: percutaneous endoscopic gastrostomy tube. Journal of Surgical Case Reports. 2023;2023(6). doi: 10.1093/jscr/rjad294. PubMed PMID: WOS:001011173900005.

5392. Blais N, Butts CA, Crowther MA, Cox-Kennett N, Martineau J. Long Term Treatment and Prevention Of Recurrent VTE In Cancer Patients: Results Of The Canadian Proact Survey. Blood. 2013;122(21):5581. doi: https://doi.org/10.1182/blood.V122.21.5581.5581.

5393. Blais R, Laurier C, Paré M. Effect of feedback letters to physicians and pharmacists on the appropriate use of medication in the treatment of asthma. Journal of asthma. 2008;45(3):227-31. doi: 10.1080/02770900701883774. PubMed PMID: CN-00638970.

5394. Blake C, Bohle LF, Rotaru C, Zarbailov N, Sava V, Sécula F, et al. Quality of care for non-communicable diseases in the Republic of Moldova: A survey across primary health care facilities and pharmacies. BMC Health Services Research. 2019;19(1). doi: 10.1186/s12913-019-4180-4.

5395. Blake KB, Madhavan SS. Perceived barriers to provision of medication therapy management services (MTMS) and the likelihood of a pharmacist to work in a pharmacy that provides MTMS. Annals of Pharmacotherapy. 2010;44(3):424-31. PubMed PMID: 20179254.

5396. Blake KB, Madhavan SS, Scott V, Elswick BLM. Medication therapy management services in West Virginia: Pharmacists' perceptions of educational and training needs. Research in Social & Administrative Pharmacy. 2009;5(2):182-8. doi: 10.1016/j.sapharm.2008.06.003. PubMed PMID: WOS:000267494100009.

5397. Blake KB, Madhavan SS, Scott VG, Meredith Elswick BL. Medication therapy management services in West Virginia: pharmacists' perceptions of educational and training needs. Research In Social & Administrative Pharmacy. 2009;5(2):182-8. PubMed PMID: 19524865.

5398. Blake SC, McMorris K, Jacobson KL, Gazmararian JA, Kripalani S. A qualitative evaluation of a health literacy intervention to improve medication adherence for underserved pharmacy patients. Journal of health care for the poor and underserved. 2010;21(2):559-67.

5399. Blake SG. The impact of the Louisiana Medicaid Lock-in Program on the process and clinical and economic outcomes of recipient care [Ph.D.]. United States -- Louisiana: Northeast Louisiana University; 1997.

5400. Blake SG, Hunter TS, Kost TM. Ensuring a role in diabetes disease management programs. Journal of Pharmacy Practice. 1999;12(2):155-62. doi: 10.1177/089719009901200208.

5401. Blakely ML, Sherbeny F, Hastings TJ, Boyd L, Adeoye-Olatunde OA. Exploratory analysis of medication adherence and social determinants of health among older adults with diabetes. Exploratory Research in Clinical and Social Pharmacy. 2023;12:100371. doi: https://doi.org/10.1016/j.rcsop.2023.100371.

5402. Blakemore S. It’s in the bag: reducing medication waste speeds up diagnosis and streamlines care.

5403. Blakey SA, Hixson-Wallace JA. Clinical and economic effects of pharmacy services in a geriatric ambulatory clinic. Pharmacotherapy. 2000;20(10):1198-203. doi: 10.1592/phco.20.15.1198.34581. PubMed

PMID: WOS:000089681100007.

5404. Blalock SJ, Casteel C, Roth MT, Ferreri S, Demby KB, Shankar V. Impact of enhanced pharmacologic care on the prevention of falls: a randomized controlled trial. American journal of geriatric pharmacotherapy. 2010;8(5):428-40. doi: 10.1016/j.amjopharm.2010.09.002. PubMed PMID: CN-00787271.

5405. Blalock SJ, Ferreri SP, Renfro CP, Robinson JM, Farley JF, Ray N, et al. Impact of STEADI-Rx: a Community Pharmacy-Based Fall Prevention Intervention. Journal of the American Geriatrics Society. 2020;68(8):1778-86. doi: 10.1111/jgs.16459. PubMed PMID: CN-02248900.

5406. Blalock SJ, Ferreri SP, Renfro CP, Robinson JM, Farley JF, Ray N, et al. Impact of STEADI-Rx: a Community Pharmacy-Based Fall Prevention Intervention. Journal of the American Geriatrics Society. 2020;68(8):1778-86. doi: 10.1111/jgs.16459. PubMed PMID: CN-02418129.

5407. Blalock SJ, Renfro CP, Robinson JM, Farley JF, Busby-Whitehead J, Ferreri SP. Using the Drug Burden Index to identify older adults at highest risk for medication-related falls. BMC Geriatrics. 2020;20(1). doi: 10.1186/s12877-020-01598-5.

5408. Blalock SJ, Roberts AW, Lauffenburger JC, Thompson T, O'Connor SK. The effect of community pharmacy-based interventions on patient health outcomes: A systematic review. Medical Care Research and Review. 2013;70(3):235-66. doi: 10.1177/1077558712459215.

5409. Blalock SJ, Roberts AW, Lauffenburger JC, Thompson T, O’Connor SK. The Effect of community pharmacy–based interventions on patient health outcomes: a systematic review. Medical care research and review. 2013;70(3):235-66.

5410. Blanc A, Guignard B, Desnoyer A, Grosgurin O, Marti C, Samer C, et al. PIM-Check© used by physicians to reduce drug-related problems in internal medicine. Pharmacien hospitalier ET clinicien Conference: resumes des 20e journees franco-suisses de pharmacie hospitaliere Switzerland. 2017;52(1):e17-e8. doi: 10.1016/j.phclin.2017.01.046. PubMed PMID: CN-01376653.

5411. Blanc A, Roy H. Feedback on the 32nd CHU Sainte-Justine Pharmacotherapy Day, 2023. Journal de Pharmacie Clinique. 2023;42(4):191-6. doi: 10.1684/JPC.2023.0532.

5412. Blanc AL, Guignard B, Desnoyer A, Grosgurin O, Marti C, Samer C, et al. Pim-check used by physicians to reduce drug related problems in internal medicine. European journal of hospital pharmacy Conference: 22nd annual congress of the european association of hospital pharmacists, EAHP 2017 France. 2017;24:A12-A3. doi: 10.1136/ejhpharm-2017-000640.28. PubMed PMID: CN-01406490.

5413. Blanc AL, Guignard B, Desnoyer A, Grosgurin O, Marti C, Samer C, et al. Prevention of potentially inappropriate medication in internal medicine patients: A prospective study using the electronic application PIM-Check. Journal of Clinical Pharmacy & Therapeutics. 2018;43(6):860-6. doi: 10.1111/jcpt.12733. PubMed PMID: 132656165. Language: English. Entry Date: 20181031. Revision Date: 20191202. Publication Type: Article.

5414. Blanc AL, Spasojevic S, Leszek A, Théodoloz M, Bonnabry P, Fumeaux T, et al. A comparison of two tools to screen potentially inappropriate medication in internal medicine patients. Journal of Clinical Pharmacy & Therapeutics. 2018;43(2):232-9. doi: 10.1111/jcpt.12638. PubMed PMID: 128258163. Language: English. Entry Date: 20180312. Revision Date: 20190401. Publication Type: Article.

5415. Blanc C, Chevalier D, Berdot S, Sabatier B, Bezie Y. Comparison of the economic impact of two drug storage systems. International journal of clinical pharmacy Conference: 45th ESCP-NSF international symposium on clinical pharmacy: clinical pharmacy tackling inequalities and access to health care Norway

Conference start: 20161005 Conference end: 20161007. 2017;39(1):315. doi: 10.1007/s11096-016-0404-4. PubMed PMID: CN-01333880.

5416. Blanchard CM, Duboski V, Graham J, Webster L, Kern MS, Wright EA, et al. A mixed methods evaluation of the implementation of pharmacy services within a team-based at-home care program. Research In Social & Administrative Pharmacy. 2021;17(11):1978-88. PubMed PMID: 33745855.

5417. Blanchard K, Bungay H, Furedi A, Sanders L. Evaluation of an emergency contraception advance provision service. Contraception. 2003;67(5):343-8. doi: https://doi.org/10.1016/S0010-7824(03)00023-4.

5418. Blanchette CM, Akazawa M, Dalal A, Simoni-Wastila L. Risk of hospitalizations/emergency department visits and treatment costs associated with initial maintenance therapy using fluticasone propionate 500 µg/salmeterol 50 µg compared with ipratropium for chronic obstructive pulmonary disease in older adults. The American Journal of Geriatric Pharmacotherapy. 2008;6(3):138-46. doi: https://doi.org/10.1016/j.amjopharm.2008.08.005.

5419. Blanchette CM, Broder M, Ory C, Chang E, Akazawa M, Dalal AA. Cost and utilization of COPD and asthma among insured adults in the US. Current Medical Research and Opinion. 2009;25(6):1385-92. doi: 10.1185/03007990902875927. PubMed PMID: WOS:000267933600007.

5420. Blanchette CM, Gutierrez B, Ory C, Chang E, Akazawa M. Economic burden in direct costs of concomitant chronic obstructive pulmonary disease and asthma in a Medicare Advantage population. Journal of Managed Care Pharmacy. 2008;14(2):176-85. doi: 10.18553/jmcp.2008.14.2.176.

5421. Blanco G. Driving and Community Mobility: An Online Educational Program for Generalist Occupational Therapy Practitioners2023.

5422. Blanco M, Chen L, Melamed A, Tergas AI, Khoury-Collado F, Hou JY, et al. Cost of care for the initial management of cervical cancer in women with commercial insurance. American Journal of Obstetrics and Gynecology. 2021;224(3). doi: 10.1016/j.ajog.2020.08.039. PubMed PMID: WOS:000621667400010.

5423. Blanco PJ, Holliman RP, Ceballos PL, Farnam JL. Exploring the Impact of Child-Centered Play Therapy on Academic Achievement of At-Risk Kindergarten Students. International journal of play therapy. 2019;28(3):133-43. doi: 10.1037/pla0000086. PubMed PMID: CN-02417287.

5424. Blanco-Aparicio M, Almonacid C, Calvín Lamas M, Delgado J, Gandolfo-Cano M, López-Carrasco V, et al. Telemedicina y asma grave en nuestro entorno: reflexiones sobre la experiencia de los profesionales y propuestas para hacerla realidad. Open Respiratory Archives. 2023;5(3):100239. doi: https://doi.org/10.1016/j.opresp.2023.100239.

5425. Blandford A, Furniss D, Lyons I, Chumbley G, Iacovides I, Wei L, et al. Exploring the Current Landscape of Intravenous Infusion Practices and Errors (ECLIPSE): protocol for a mixed-methods observational study. BMJ open. 2016;6(3):e009777.

5426. Blank C. Million hearts campaign gears up to save lives. Drug Topics. 2011;155(11).

5427. Blank C. NCPA to be loud voice for community pharmacy at state and federal levels. Drug Topics. 2011;155(5).

5428. Blank C. Retail pharmacy project aims to boost vaccination rates. Drug Topics. 2015;159(11). PubMed PMID: 606913110.

5429. Blank C. Retail pharmacy: Know the marketplace to grow the customer base. Drug Topics.

2015;159(7).

5430. Blank C. Genoa MMS: Medication management for its community pharmacies: Two groups are joining forces to enhance medication management. Drug Topics. 2017;161(10).

5431. Blank-Reid CA, Kaplan LJ. System improvements for the use of neuromuscular blocking agents. International Journal of Trauma Nursing. 1998;4(1):9-12. doi: https://doi.org/10.1016/S1075-4210(98)90024-4.

5432. Blaschke TF, Osterberg L, Vrijens B, Urquhart J. Adherence to medications: insights arising from studies on the unreliable link between prescribed and actual drug dosing histories. Annual review of pharmacology and toxicology. 2012;52:275-301.

5433. Blassmann U, Morath B, Fischer A, Knoth H, Hoppe-Tichy T. [Medication safety in hospitals : Integration of clinical pharmacists to reduce drug-related problems in the inpatient setting]. Bundesgesundheitsblatt, Gesundheitsforschung, Gesundheitsschutz. 2018;61(9):1103-10. PubMed PMID: 30022237.

5434. Blaszczyk A, Brandt N, Ashley J, Tuders N, Doles H, Stefanacci RG. Crushed Tablet Administration for Patients with Dysphagia and Enteral Feeding: Challenges and Considerations. Drugs & Aging. 2023;40(10):895-907. doi: 10.1007/s40266-023-01056-y. PubMed PMID: 172040517.

5435. Blauer-Peterson C, Yehoshua A, Carlyle M, Walker V, Gandra S. Randomized Study to Evaluate a Secondary Prevention Program for Women with Osteoporotic Fractures. Journal of bone and mineral research. 2020;35(SUPPL 1):250-. doi: 10.1002/jbmr.4206. PubMed PMID: CN-02229158.

5436. Blaya JA, Fraser HS, Holt B. E-health technologies show promise in developing countries. Health Affairs. 2010;29(2):244-51.

5437. Blazejewski L, Vaidya V, Pinto S, Gaither C. Pharmacists' Perceived Barriers Providing Non-Dispensing Services to Underserved Populations. Journal of Community Health. 2013;38(5):812-22. doi: 10.1007/s10900-013-9682-0. PubMed PMID: WOS:000324118300004.

5438. Blebil A, Dujaili J, Elkalmi R, Tan HL, Tai M, Khan T. Community pharmacist's role in providing oral health-care services: Findings from Malaysia. Journal of Pharmacy and Bioallied Sciences. 2020;12(1):64-71. doi: 10.4103/jpbs.JPBS_152_19.

5439. Blebil A, Dujaili J, Elkalmi R, Tan HLK, Tai MS, Khan TM. Community pharmacist's role in providing oral health-care services: Findings from Malaysia. Journal of Pharmacy and Bioallied Sciences. 2020;12(1):64-71. doi: 10.4103/jpbs.JPBS_152_19. PubMed PMID: WOS:000511465800009.

5440. Blebil A, Dujaili J, Mohammed AH, Cheong CM, Hoo Y. The effect of stress and depression on quality of life of pharmacy students in Malaysia. Pharmacy Education. 2021;21(1):323-33. doi: 10.46542/pe.2021.211.323333.

5441. Blebil AQ, Dujaili JA, Mohammed AH, Loh LL, Chung WX, Selvam T, et al. Exploring the eHealth literacy and mobile health application utilisation amongst Malaysian pharmacy students. Journal of Telemedicine and Telecare. 2023;29(1):58-71. doi: 10.1177/1357633X221077869.

5442. Blebil AQ, Dujaili JA, Mohammed AH, Loh LL, Chung WX, Selvam T, et al. Exploring the eHealth literacy and mobile health application utilisation amongst Malaysian pharmacy students. Journal of Telemedicine and Telecare. 2023;29(1):58-71. doi: 10.1177/1357633X221077869.

5443. Blecker S, Adhikari S, Zhang HC, Dodson JA, Desai SM, Anzisi L, et al. Validation of EHR medication fill data obtained through electronic linkage with pharmacies. Journal of Managed Care & Specialty Pharmacy. 2021;27(10):1482-7. PubMed PMID: WOS:000717085200013.

5444. Bledsoe S, student AVBPD, student RJFPD, student AHPD, Hoebing W, Jokic S. Errors detected in pediatric oral liquid medication doses prepared in an automated workflow management system. American Journal of Health-System Pharmacy. 2018;75(3):127-31. doi: 10.2146/ajhp160902. PubMed PMID: 127873981. Language: English. Entry Date: 20180221. Revision Date: 20190130. Publication Type: Article.

5445. Bledsoe S, Van Buskirk A, Falconer RJ, Hollon A, Hoebing W, Jokic S. Errors detected in pediatric oral liquid medication doses prepared in an automated workflow management system. American Journal of Health-System Pharmacy. 2018;75(3):127-31. PubMed PMID: 29371193.

5446. Blee J, Roux RK, Gautreaux S, Sherer JT, Garey KW. Dispensing inhalers to patients with chronic obstructive pulmonary disease on hospital discharge: effects on prescription filling and readmission. American journal of health-system pharmacy. 2015;72(14):1204-8. doi: 10.2146/ajhp.140621. PubMed PMID: CN-01139202.

5447. Bleich SN. Generating better evidence to engage local food outlets in obesity prevention research. Preventive Medicine. 2013;57(4):265-7. doi: 10.1016/j.ypmed.2013.07.015. PubMed PMID: WOS:000324789100003.

5448. Bleich SN, Chang HY, Lau B, Steele K, Clark JM, Richards T, et al. Impact of Bariatric Surgery on Health Care Utilization and Costs Among Patients With Diabetes. Medical Care. 2012;50(1):58-65. doi: 10.1097/MLR.0b013e3182290349. PubMed PMID: WOS:000298157200008.

5449. Bleijenberg N, Zuithoff NPA, Smith AK, de Wit N, Schuurmans MJ. DISABILITY IN THE INDIVIDUAL ADL, IADL, AND MOBILITY AMONG OLDER ADULTS: A PROSPECTIVE COHORT STUDY. Journal of Nutrition Health & Aging. 2017;21(8):897-903. doi: 10.1007/s12603-017-0891-6. PubMed PMID: WOS:000410824300008.

5450. Bleiker P, Lewis A. Extending the role of community pharmacists: The views of GPs. International Journal of Pharmacy Practice. 1998;6(3):140-4. doi: 10.1111/j.2042-7174.1998.tb00929.x.

5451. Blenkinsopp A, Anderson C, Armstrong M. Systematic review of the effectiveness of community pharmacy-based interventions to reduce risk behaviours and risk factors for coronary heart disease. Journal of Public Health Medicine. 2003;25(2):144-53. doi: 10.1093/pubmed/fdg030.

5452. Blenkinsopp A, Anderson C, Armstrong M. Community pharmacy's contribution to improving the public's health: The case of weight management. International Journal of Pharmacy Practice. 2008;16(3):123-5. doi: 10.1211/ijpp.16.3.0001.

5453. Blenkinsopp A, Anderson C, Armstrong M, Riley J, Tsuyuki RT. Community pharmacy-based interventions can reduce risk behaviour and risk factors for coronary heart disease. Evidence-Based Healthcare. 2004;8(1):21-3. doi: 10.1016/j.ehbc.2003.12.020.

5454. Blenkinsopp A, Bond C. Pharmacists must learn to play their part in multidisciplinary health teams. Pharmaceutical Journal. 2007;279(7470):330.

5455. Blenkinsopp A, Bond C, Celino G, Inch J, Gray N. National evaluation of the new community pharmacy contract. Pharmacy Practice Research Trust. 2007.

5456. Blenkinsopp A, Bond C, Celino G, Inch J, Gray N. Medicines use review: Adoption and spread of a

service innovation. International Journal of Pharmacy Practice. 2008;16(4):271-6. doi: 10.1211/ijpp.16.4.0010.

5457. Blenkinsopp A, Bond C, Raynor DK. Medication reviews. British journal of clinical pharmacology. 2012;74(4):573-80.

5458. Blenkinsopp A, Bond CM. The potential and pitfalls of medicine management: What have we learned so far? Disease Management and Health Outcomes. 2008;16(2):79-86. doi: 10.2165/00115677-200816020-00002.

5459. Blenkinsopp A, Bond CM. The potential and pitfalls of medicine management - What have we learned so far? Disease Management & Health Outcomes. 2008;16(2):79-86. doi: 10.2165/00115677-200816020-00002. PubMed PMID: WOS:000255765700002.

5460. Blenkinsopp A, Bradley C. Over the Counter Drugs: Patients, society, and the increase in self medication. Bmj. 1996;312(7031):629-32.

5461. Blenkinsopp A, Duerden M, Blenkinsopp J. Symptoms in the pharmacy: a guide to the management of common illnesses: John Wiley & Sons; 2022.

5462. Blenkinsopp A, Hassey A. Effectiveness and acceptability of community pharmacy-based interventions in type 2 diabetes: A critical review of intervention design, pharmacist and patient perspectives. International Journal of Pharmacy Practice. 2005;13(4):231-40. doi: 10.1211/ijpp.13.4.0001.

5463. Blenkinsopp A, Holmes J, Mitra G, Pringle M. Joining Up Self-Care: Evaluation of a PCT-wide programme of support for self-care. Primary Health Care Research and Development. 2009;10(2):83-97. doi: 10.1017/S1463423608000984.

5464. Blenkinsopp A, Panton R, Anderson C. Health Promotion for Pharmacists1999. 1-266 p.

5465. Blenkinsopp A, Phelan M, Bourne J, Dakhil N. Extended adherence support by community pharmacists for patients with hypertension: a randomised controlled trial. International journal of pharmacy practice. 2000;8(3):165-75. PubMed PMID: CN-00343240.

5466. Blenkinsopp A, Tann J, Platts A, Allen J. Evaluation of feasibility and acceptability of a community pharmacy health promotion scheme - Views of users and providers. Health Education Journal. 2002;61(1):52-69. doi: 10.1177/001789690206100106.

5467. Blenkinsopp J, Cottrell J, Mann SG. The utility and feasibility of a community pharmacist-supported protocol assessment of eligibility for OTC statin treatment. Pharmaceutical Journal. 2004;273(7322):606-9.

5468. Blenkinsopp J, Gathoga L, O'Connell K, Mukhtar M, Rehman I, Shan N, et al. OTC simvastatin supply - What changes in practice and education do pharmacists want? Pharmaceutical Journal. 2004;273(7311):191-3.

5469. Blennerhassett JD, Cusack BM, Smith CD, Green L, Tribe KL. A novel medicines management pathway. Journal of Pharmacy Practice and Research. 2006;36(3):175-9. doi: 10.1002/j.2055-2335.2006.tb00601.x.

5470. Bleske BE, Dillman NO, Cornelius D, Ward JK, Burson SC, Diez HL, et al. Heart failure assessment at the community pharmacy level: A feasibility pilot study. Journal of the American Pharmacists Association. 2014;54(6):634-41. doi: https://doi.org/10.1331/JAPhA.2014.14039.

5471. Bleske BE, Raley RE, Hunter TS, Anderson JR. Curricular-driven screening for atrial fibrillation at community pharmacies. Journal of the American Pharmacists Association. 2022;62(2):541-5.e1. doi: https://doi.org/10.1016/j.japh.2021.10.017.

5472. Bleske BE, Remington TL, Wells TD, Klein KC, Guthrie SK, Tingen JM, et al. A Randomized Crossover Comparison of Team-based Learning and Lecture Format on Learning Outcomes. American journal of pharmaceutical education. 2016;80(7):120. doi: 10.5688/ajpe807120. PubMed PMID: CN-01335893.

5473. Blessing JA, Bialy SA, Whitney CW, Stonebraker BL, Stehman FB. Gynecologic Oncology Group quality assurance audits: Analysis and initiatives for improvement. Clinical Trials. 2010;7(4):390-9. doi: 10.1177/1740774510372535.

5474. Blette G, Leslie L, Focht J, Snyder M. Evaluation of pharmacy technician education in relation to community pharmacy vaccination rates. Journal of the american pharmacists association. 2015;55(2):e158-e9. doi: 10.1331/JAPhA.2015.15515. PubMed PMID: CN-01108024.

5475. Bliek-Bueno K, Mucherino S, Poblador-Plou B, González-Rubio F, Aza-Pascual-salcedo M, Orlando V, et al. Baseline drug treatments as indicators of increased risk of COVID-19 mortality in Spain and Italy. International Journal of Environmental Research and Public Health. 2021;18(22). doi: 10.3390/ijerph182211786.

5476. Blignaut AJ, Coetzee SK, Klopper HC, Ellis SM. Medication administration errors and related deviations from safe practice: an observational study. Journal of clinical nursing. 2017;26(21-22):3610-23.

5477. Blin A. Voyages et vaccination. Actualités Pharmaceutiques. 2020;59(594):45-9. doi: https://doi.org/10.1016/j.actpha.2020.01.013.

5478. Blin A, Pillon F. Médicaments et grossesse, application à l'exercice officinal. Actualités Pharmaceutiques. 2017;56(571):33-7. doi: https://doi.org/10.1016/j.actpha.2017.09.024.

5479. Blin A, Pillon F. Medication and pregnancy, the role of the community pharmacy. Actualites Pharmaceutiques. 2017;56(571):33-7. doi: 10.1016/j.actpha.2017.09.024.

5480. Blin M, Cherel A, Bougle C, Piriou G. Definition and evaluation of a multidisciplinary patient's pharmacotherapy management method: The pharmaceutical care plan. Annales Pharmaceutiques Francaises. 2023;81(1):13-29. doi: https://dx.doi.org/10.1016/j.pharma.2022.06.006. PubMed PMID: 2020266346.

5481. Blin M, Cherel A, Bouglé C, Piriou G. Définition et évaluation d'une méthodologie interprofessionnelle d'optimisation de la pharmacothérapie des patients : le Plan de Médication Partagé. Annales Pharmaceutiques Françaises. 2023;81(1):13-29. doi: https://doi.org/10.1016/j.pharma.2022.06.006.

5482. Blin M, Cherel A, Bouglé C, Piriou G. Définition et évaluation d'une méthodologie interprofessionnelle d'optimisation de la pharmacothérapie des patients : le Plan de Médication Partagé. Annales Pharmaceutiques Françaises. 2023;81(1):13-29. doi: https://doi.org/10.1016/j.pharma.2022.06.006.

5483. Blin M, Cherel A, Bougle C, Piriou G. [Definition and evaluation of a multidisciplinary patient's pharmacotherapy management method: The pharmaceutical care plan]. Annales Pharmaceutiques Francaises. 2023;81(1):13-29. PubMed PMID: 35724711.

5484. Blind JE, Nowicki DN, McConnell K, Motsney AJ, Kong A. A call to action: Health-system pharmacists must stand up to meet the growing demand for cellular-based therapies. American Journal of Health-System Pharmacy. 2023;80(14):944-7. doi: 10.1093/ajhp/zxad071. PubMed PMID: 164799008.

5485. Blind S, Wong-Go E. Electronic medication data transfer programme: A multiparty collaboration of 24 healthcare professional associations and organisations in The Netherlands. Pharmacy Education. 2023;23(3):94. doi: https://dx.doi.org/10.46542/pe.2023.233.60135. PubMed PMID: 642266193.

5486. Blinova E, Volling J, Koczmara C, Greenall J. Oral methotrexate: preventing inadvertent daily administration. Canadian Journal of Hospital Pharmacy. 2008;61(4).

5487. Bliss E, Codack H, Boothe J. Diabetes care - An evaluation of a community pharmacy based HbA1c testing service. Pharmaceutical Journal. 2001;267(7162):264-6.

5488. Blissenbach HF. Use of Cost-Consequence Models in Managed Care. Pharmacotherapy: The Journal of Human Pharmacology and Drug Therapy. 1995;15(5P2):59S-61S. doi: 10.1002/j.1875-9114.1995.tb04401.x.

5489. Blissenbach HF. USE OF COST-CONSEQUENCE MODELS IN MANAGED CARE. Pharmacotherapy. 1995;15(5):S59-S61. PubMed PMID: WOS:A1995RW19700005.

5490. Blix HS, Viktil KK, Moger TA, Reikvam Å. Characteristics of drug-related problems discussed by hospital pharmacists in multidisciplinary teams. Pharmacy World and Science. 2006;28:152-8.

5491. Blix HS, Viktil KK, Moger TA, Reikvam A. Drugs with narrow therapeutic index as indicators in the risk management of hospitalised patients. Pharmacy practice. 2010;8(1):50.

5492. Blix HS, Viktil KK, Reikvam Å, Moger TA, Hjemaas BJ, Pretsch P, et al. The majority of hospitalised patients have drug-related problems: results from a prospective study in general hospitals. European journal of clinical pharmacology. 2004;60:651-8.

5493. Block G, Jensen C, Dietrich M, Norkus EP, Hudes M, Packer L. Plasma C-reactive protein concentrations in active and passive smokers: influence of antioxidant supplementation. Journal of the American College of Nutrition. 2004;23(2):141-7.

5494. Block H, Annesley A, Lockwood K, Xu L, Cameron ID, Laver K, et al. Frailty in older people: rehabilitation Treatment Research Examining Separate Settings (FORTRESS): protocol for a hybrid type II stepped wedge, cluster, randomised trial. BMC geriatrics. 2022;22(1):527. doi: 10.1186/s12877-022-03178-1. PubMed PMID: CN-02421150.

5495. Block H, Annesley A, Lockwood K, Xu L, Cameron ID, Laver K, et al. Frailty in older people: Rehabilitation Treatment Research Examining Separate Settings (FORTRESS): protocol for a hybrid type II stepped wedge, cluster, randomised trial. BMC Geriatrics. 2022;22(1). doi: 10.1186/s12877-022-03178-1.

5496. Block H, Annesley A, Lockwood K, Xu LD, Cameron ID, Laver K, et al. Frailty in older people: Rehabilitation Treatment Research Examining Separate Settings (FORTRESS): protocol for a hybrid type II stepped wedge, cluster, randomised trial. Bmc Geriatrics. 2022;22(1). doi: 10.1186/s12877-022-03178-1. PubMed PMID: WOS:000817288900003.

5497. Bloland PB, Organization WH. Drug resistance in malaria. World Health Organization, 2001.

5498. Blom ATG, Hugtenburg JG, Post CP. Second drug delivery contacts: Useful for the patient? A study in the Netherlands. Farmaceutisch Tijdschrift voor Belgie. 2006;83(1):13-7.

5499. Blom ATG, Hugtenburg JG, Post CP. Second drug delivery contact can benefit patients. Pharmaceutisch Weekblad. 2007;142(47):156-9.

5500. Blom L. Does pharmaceutical care for repeated deliveries of cardiovascular medication comply with

the guidelines? Pharmaceutisch Weekblad. 2018;153(50):17-21.

5501. Blom L, Bakx JCH, Van Burg A, Pronk M. Optimism with 'counter coaches'. Implementation of patient information. Pharmaceutisch Weekblad. 2002;137(17):615-8.

5502. Blom L, Krass I. Introduction: The role of pharmacy in patient education and counseling. Patient Education and Counseling. 2011;83(3):285-7. doi: 10.1016/j.pec.2011.05.021.

5503. Blom L, Winters N, Mantet-Teeuwisse A, Bouvy M. UPPER policy for training pharmacists in community pharmacy: Internship in pharmacy requires quality of educator. Pharmaceutisch Weekblad. 2016;151(50):10-3.

5504. Blom MT, Van Hoeijen DA, Bardai A, Berdowski J, Souverein PC, De Bruin ML, et al. Genetic, clinical and pharmacological determinants of out-of-hospital cardiac arrest: Rationale and outline of the AmsteRdam Resuscitation Studies (ARREST) registry. Open Heart. 2014;1(1). doi: 10.1136/openhrt-2014-000112.

5505. Blondal AB, Almarsdottir AB, Jonsson JS, Gizurarson S. [Pharmaceutical Care at the primary care clinic in Garabaer - number and type of drug therapy problems identified among elderly clients]. Laeknabladid. 2017;103(11):481-6. PubMed PMID: 29083310.

5506. Blöndal AB, Almarsdóttir AB, Jónsson JS, Gizurarson S. Pharmaceutical Care at the primary care clinic in Garoabær - number and type of drug therapy problems identified among elderly clients. Laeknabladid. 2017;103(11):481-6. doi: 10.17992/lbl.2017.11.159.

5507. Blondal AB, Jonsson JS, Sporrong SK, Almarsdottir AB. General practitioners' perceptions of the current status and pharmacists' contribution to primary care in Iceland. International Journal of Clinical Pharmacy. 2017;39(4):945-52. doi: 10.1007/s11096-017-0478-7.

5508. Blonde L, Umpierrez GE, Reddy SS, McGill JB, Berga SL, Bush M, et al. American Association of Clinical Endocrinology Clinical Practice Guideline: Developing a Diabetes Mellitus Comprehensive Care Plan—2022 Update. Endocrine Practice. 2022;28(10):923-1049. doi: https://doi.org/10.1016/j.eprac.2022.08.002.

5509. Blondeau JM, Tillotson GS. Formula to help select rational antimicrobial therapy (FRAT): its application to community- and hospital-acquired urinary tract infections. International Journal of Antimicrobial Agents. 1999;12(2):145-50. doi: 10.1016/s0924-8579(98)00107-1. PubMed PMID: WOS:000081241900010.

5510. Blondon M. The association of smoking with venous thrombosis in women and its modification by exogenous hormones [M.S.]. United States -- Washington: University of Washington; 2012.

5511. Bloodworth LS, Malinowski SS, Lirette ST, Ross LA. Pharmacist linkage in care transitions: From academic medical center to community. Journal of the American Pharmacists Association. 2019;59(6):896-904. PubMed PMID: 2003272749.

5512. Bloom BS. Continuation of initial antihypertensive medication after 1 year of therapy. Clinical therapeutics. 1998;20(4):671-81.

5513. Bloom BS, Iannacone RC. Internet availability of prescription pharmaceuticals to the public. Annals of Internal Medicine. 1999;131(11):830-3. doi: 10.7326/0003-4819-131-11-199912070-00005.

5514. Bloom BS, Iannacone RC. Changing availability and cost of Internet physician consultations and

prescription medications. Medical Informatics and the Internet in Medicine. 2006;31(4):247-53. doi: 10.1080/14639230600551405.

5515. Bloom MA, Barghout V, Kahler KH, Bentkover J, Kurth H, Gralnek IM, et al. Budget impact of tegaserod on a managed care organization formulary. American Journal of Managed Care. 2005;11(1):S27-S34. PubMed PMID: WOS:000228528000004.

5516. Bloom MZ. Compounding in today's practice: Concern about pharmacists' right to compound has heated up since compounding problems occurred in Pittsburgh, prompting increased interest by FDA. American Pharmacy. 1991;31(10):31-7. doi: https://doi.org/10.1016/S0160-3450(15)31332-5.

5517. Bloom S, Stollings J, Kirkpatrick O, Wang L, Byrne D, Sevin C, et al. Randomized trial of an intensive care unit recovery program for survivors of critical illness. Critical care medicine. 2019;47(1). PubMed PMID: CN-01995871.

5518. Bloom SL, Stollings JL, Kirkpatrick O, Wang L, Byrne DW, Sevin CM, et al. Randomized Clinical Trial of an ICU Recovery Pilot Program for Survivors of Critical Illness. Critical care medicine. 2019;47(10):1337-45. doi: 10.1097/CCM.0000000000003909. PubMed PMID: CN-01977032.

5519. Bloomfield K, Wu Z, Broad JB, Tatton A, Calvert C, Hikaka J, et al. Learning from a multidisciplinary randomized controlled intervention in retirement village residents. Journal of the American Geriatrics Society. 2021. doi: 10.1111/jgs.17533. PubMed PMID: CN-02343315.

5520. Bloomfield K, Wu Z, Broad JB, Tatton A, Calvert C, Hikaka J, et al. Learning from a multidisciplinary randomized controlled intervention in retirement village residents. Journal of the American Geriatrics Society. 2022;70(3):743-53. doi: 10.1111/jgs.17533. PubMed PMID: CN-02398108.

5521. Blue H, Hawthorne AN, Melgaard K, Dahly A, Lunos S, Palombi L. Pharmacist involvement in combating the opioid crisis: A mixed-methods approach revealing conflicting perceptions. JACCP Journal of the American College of Clinical Pharmacy. 2020;3(1):21-9. doi: 10.1002/jac5.1124.

5522. Blum E, Thavarajah K, Martirosov AL. Optimization of patient-specific inhaler regimens with the use of the aerosol inhalation monitor. Journal of the American Pharmacists Association. 2019;59(2):252-7. doi: https://doi.org/10.1016/j.japh.2018.10.012.

5523. Blum JM, Morris PE, Martin GS, Gong MN, Bhagwanjee S, Cairns CB, et al. United States Critical Illness and Injury Trials Group. Chest. 2013;143(3):808-13. doi: 10.1378/chest.12-2287. PubMed PMID: WOS:000317871300036.

5524. Blum M, Peck V, Seltzer T, Goldberg-Berman J. Alert: 6-mercaptopurine may be erroneously dispensed instead of propylthiouracil. Thyroid. 2005;15(11):1315. PubMed PMID: 16356101.

5525. Blum MR, Sallevelt B, Spinewine A, O'Mahony D, Moutzouri E, Feller M, et al. Optimizing Therapy to Prevent Avoidable Hospital Admissions in Multimorbid Older Adults (OPERAM): cluster randomised controlled trial. BMJ (Clinical research ed). 2021;374:n1585. doi: 10.1136/bmj.n1585. PubMed PMID: CN-02292192.

5526. Blumenthal WJ, Springer KW, Stephen Jones T, Sterk CE. Pharmacy Student Knowledge, Attitudes, and Beliefs About Selling Syringes to Injection Drug Users. Journal of the American Pharmaceutical Association (1996). 2002;42(6, Supplement 2):S34-S9. doi: https://doi.org/10.1331/1086-5802.42.0.S34.Blumenthal.

5527. Bluml BM. Definition of Medication Therapy Management: Development of Professionwide

Consensus. Journal of the American Pharmacists Association. 2005;45(5):566-72. doi: https://doi.org/10.1331/1544345055001274.

5528. Bluml BM, Brock KA, Hamstra S, Tonrey L. Evaluation of the Impact of an Innovative Immunization Practice Model Designed to Improve Population Health: Results of the Project IMPACT Immunizations Pilot. Population Health Management. 2018;21(1):55-62. doi: 10.1089/pop.2017.0049. PubMed PMID: WOS:000423510400010.

5529. Bluml BM, McKenney JM, Cziraky MJ. Pharmaceuticalcare services and results in project ImPACT: Hyperlipidemia. Journal of the American Pharmaceutical Association (Washington, DC : 1996). 2000;40(2):157-65. doi: 10.1016/S1086-5802(16)31059-2.

5530. Bluml BM, Watson LL, Skelton JB, Manolakis PG, Brock KA. Improving outcomes for diverse populations disproportionately affected by diabetes: Final results of Project IMPACT: Diabetes. Journal of the American Pharmacists Association. 2014;54(5):477-85. doi: https://doi.org/10.1331/JAPhA.2014.13240.

5531. Blundell DB. Implementation of the EFQM model in the management of healthcare services in a community pharmacy. Pharmaceutical Care Espana. 2012;14(2):50-60.

5532. Blundell DB. Medication review according to STOPP-START criteria in older patients from the dose administration aids service of a community pharmacy. Farmaceuticos Comunitarios. 2015;7(2):31-6. doi: 10.5672/FC.21739218.(2015/Vol7).002.05. PubMed PMID: WOS:000417267300005.

5533. Blundell DB. Analysis of the effects of a robotic dispensing system implementation both, on the healthcare activities' results and on the internal processes of a community pharmacy. Pharmaceutical Care Espana. 2015;17(1):261-71. PubMed PMID: WOS:000420537100002.

5534. Blundell DB, Sanchez BS, Gomez RS, Pacheco GB, Ariza MIC, Visa EN. Results of pharmacovigilance in a community pharmacy. Farmaceuticos Comunitarios. 2020;12(4):30-6. doi: 10.33620/FC.2173-9218.(2020/Vol12).004.04. PubMed PMID: WOS:000581116600004.

5535. Blundell DB, Zarzuelo CR, Sánchez BS, Jiménez BG, Jiménez AMR, Álvarez JLG. Are patients correctly using inhalants from a community pharmacy? Pharmaceutical Care Espana. 2004;6(1):25-31.

5536. Blyler CA, Ebinger J, Rashid M, Moy NP, Cheng S, Albert CM, et al. Improving efficiency of the barbershop model of hypertension care for black men with virtual visits. Journal of the American Heart Association. 2021;10(13). doi: 10.1161/JAHA.120.020796.

5537. Blyler CA, Rader F. Sustainability of blood pressure reduction in black barbershops. Current opinion in cardiology. 2019;34(6):693-9. doi: 10.1097/HCO.0000000000000674. PubMed PMID: CN-01988288.

5538. Bø K-E, Halvorsen KH, Risør T, Lehnbom EC. 'Illuminating determinants of implementation of non-dispensing pharmacist services in home care: a qualitative interview study'. Scandinavian Journal of Primary Health Care. 2023;41(1):43-51. doi: 10.1080/02813432.2023.2164840. PubMed PMID: 163051179.

5539. Boakye-Agyeman F. Quantifying the Quality of Antimalarial Drugs in Ghana [Ph.D.]. United States -- Minnesota: Walden University; 2017.

5540. Board AR, Guy G, Jones CM, Hoots B. Trends in stimulant dispensing by age, sex, state of residence, and prescriber specialty — United States, 2014–2019. Drug and Alcohol Dependence. 2020;217:108297. doi: https://doi.org/10.1016/j.drugalcdep.2020.108297.

5541. Boardman H, Lewis M, Trinder P, Rajaratnam G, Croft P. Use of community pharmacies: A

population-based survey. Journal of Public Health. 2005;27(3):254-62. doi: 10.1093/pubmed/fdi032.

5542. Boardman HF, Avery AJ. Effectiveness of a community pharmacy weight management programme. International Journal of Clinical Pharmacy. 2014;36(4):800-6. doi: 10.1007/s11096-014-9964-3.

5543. Boardman HF, Gray NJ, Symonds BS. Interactions between parents/carers of pre-school children and pharmacy staff when buying non-prescription medicines. International Journal of Clinical Pharmacy. 2011;33(5):832-41. doi: 10.1007/s11096-011-9546-6.

5544. Boateng JS, Matthews KH, Stevens HN, Eccleston GM. Wound healing dressings and drug delivery systems: a review. Journal of pharmaceutical sciences. 2008;97(8):2892-923.

5545. Boateng R, Renner L, Petricca K, Gupta S, Denburg A. Health system determinants of access to essential medicines for children with cancer in Ghana. BMJ Global Health. 2020;5(9). doi: 10.1136/bmjgh-2020-002906.

5546. Boatwright DE. Legal aspects of expanded prescribing authority for pharmacists. American Journal of Health-System Pharmacy. 1998;55(6):585-94. PubMed PMID: 9544336.

5547. Bobade RA, Helmers RA, Jaeger TM, Odell LJ, Haas DA, Kaplan RS. Time-driven activity-based cost analysis for outpatient anticoagulation therapy: direct costs in a primary care setting with optimal performance. Journal of Medical Economics. 2019;22(5):471-7. doi: 10.1080/13696998.2019.1582058. PubMed PMID: WOS:000464563900008.

5548. Bobbins AC, Burton S, Fogarty TL. Different models of pharmaceutics services and care in primary healthcare clinics in the Eastern Cape, South Africa: Challenges and opportunities for pharmacy practice. African Journal of Primary Health Care & Family Medicine. 2020;12(1). doi: 10.4102/phcfm.v12i1.2323. PubMed PMID: WOS:000562015800001.

5549. Bobo KS, Cober MP, Eiland LS, Heigham M, King M, Johnson PN, et al. Key articles and guidelines for the pediatric clinical pharmacist from 2019 and 2020. American Journal of Health-System Pharmacy. 2022;79(5):364-84. PubMed PMID: 34864839.

5550. Bobo WV, Davis RL, Toh S, Li DK, Andrade SE, Cheetham TC, et al. Trends in the Use of Antiepileptic Drugs among Pregnant Women in the US, 2001-2007: A Medication Exposure in Pregnancy Risk Evaluation Program Study. Paediatric and Perinatal Epidemiology. 2012;26(6):578-88. doi: 10.1111/ppe.12004. PubMed PMID: WOS:000310031000011.

5551. Bobo WV, Epstein RA, Hayes RM, Shelton RC, Hartert TV, Mitchel E, et al. The effect of regulatory advisories on maternal antidepressant prescribing, 1995-2007: an interrupted time series study of 228,876 pregnancies. Archives of Womens Mental Health. 2014;17(1):17-26. doi: 10.1007/s00737-013-0383-6. PubMed PMID: WOS:000330772900003.

5552. Bobo WV, Miller SC, Martin BD. The abuse liability of dextromethorphan among adolescents: A review. Journal of Child & Adolescent Substance Abuse. 2005;14(4):55-75. doi: 10.1300/J029v14n04_04. PubMed PMID: WOS:000231322400004.

5553. Boboia A, Florea LS, Turcu-Stiolica A, Tăerel AE, Rais C, Revnic C, et al. Decision analysis of antibiotic use. Farmacia. 2020;68(4):757-65. doi: 10.31925/farmacia.2020.4.24.

5554. Boboia A, Grigorescu MR, Turcu-Ştiolică A. Marketing research on the angiotensin-converting enzyme inhibitors antihypertensive medicines. Clujul Medical. 2017;90(1):86-92. doi: 10.15386/cjmed-665.

5555. Boboia A, Oros GC, Polinicencu C, Mirel S. Researches concerning the profitability of the community pharmacy within the context of world economic and financial crisis. Farmacia. 2014;62(5):1025-36.

5556. Boboia A, Turcu-Stiolica A, Groza MR, Stoica CI, Florea A, Revnic C, et al. THE ROLE OF THE PHARMACISTS AND THE PHARMACEUTICAL COMPANIES IN THE ECOSYSTEM OF THE PUBLIC HEALTH CAMPAIGNS - A CASE STUDY IN INFORMATION CAMPAIGNS ON DIABETES MELLITUS. Acta Poloniae Pharmaceutica. 2020;77(6):951-62. doi: 10.32383/appdr/130067. PubMed PMID: WOS:000608019200014.

5557. Bocanegra AMR. Importance of the reconciliation of the treatment in the community pharmacy of a chronic, polymedicated and anticoagulated patient. Pharmaceutical Care Espana. 2018;20(5):389-96. PubMed PMID: WOS:000447232000005.

5558. Boccardi V, Baroni M, Paolacci L, Ercolani S, Longo A, Giordano M, et al. Anticholinergic burden and functional status in older people with cognitive impairment: Results from the ReGAl project. Journal of Nutrition Health & Aging. 2017;21(4):389-96. doi: 10.1007/s12603-016-0787-x. PubMed PMID: WOS:000399010700005.

5559. Boccuzzi SJ, Martin J, Stephenson J, Kreilick C, Fernandes J, Beaulieu J, et al. Retrospective study of total healthcare costs associated with chronic nonvalvular atrial fibrillation and the occurrence of a first transient ischemic attack, stroke or major bleed. Current Medical Research and Opinion. 2009;25(12):2853-64. doi: 10.1185/03007990903196422. PubMed PMID: WOS:000273417800005.

5560. Bochniarz M, Inglot-Brzek E, Lewandowska A, Podgorska J. Directions of Changes in the Profession of Hospital Pharmacist in Poland. International Journal of Environmental Research and Public Health. 2022;19(21). doi: 10.3390/ijerph192114522. PubMed PMID: WOS:000883918100001.

5561. Bock BC, Hudmon KS, Christian J, Graham AL, Bock FR. A tailored intervention to support pharmacy-based counseling for smoking cessation. Nicotine & tobacco research. 2010;12(3):217-25. doi: 10.1093/ntr/ntp197. PubMed PMID: CN-00743564.

5562. Bocker W, Doobare IU, Khachatryan A, Dornstauder E, Bartsch R, Worth G, et al. Fractures in untreated patients with osteoporosis in Germany: an InGef healthcare insurance database analysis. Osteoporosis International. 2022;33(1):77-86. doi: 10.1007/s00198-021-06051-w. PubMed PMID: WOS:000673875600003.

5563. Bocknek LS, Kim TC, Spaar PA, Russell JL, Busog D-NC, Howe JL, et al. Duplicate Medication Order Errors: Safety Gaps and Recommendations for Improvement. Patient Safety (2689-0143). 2022;4(3):39-47. doi: 10.33940/data/2022.9.6. PubMed PMID: 159321879. Language: English. Entry Date: 20221013. Revision Date: 20221013. Publication Type: Article.

5564. Bockting CLH, Klein NS, Elgersma HJ, van Rijsbergen GD, Slofstra C, Ormel J, et al. Effectiveness of preventive cognitive therapy while tapering antidepressants versus maintenance antidepressant treatment versus their combination in prevention of depressive relapse or recurrence (DRD study): a three-group, multicentre, randomised controlled trial. The lancet Psychiatry. 2018;5(5):401-10. doi: 10.1016/S2215-0366(18)30100-7. PubMed PMID: CN-01955321.

5565. Bocos Baelo A, Garcia-Castineira C, Vilarino A, Martin A, Arias A, Codina-Jimenez C, et al. A CROSS-SECTIONAL STUDY ON THE POTENTIALLY INAPPROPRIATE PRESCRIBED AND CONTRAINDICATED HIGH-RISK MEDICATION IN HOSPITALISED CHRONIC COMPLEX PATIENTS. European Journal of Hospital Pharmacy. 2023;30(Supplement 1):A133-A4. doi: https://dx.doi.org/10.1136/ejhpharm-2023-eahp.278. PubMed PMID: 640966199.

5566. Bocsan CI, Bujor AI, Miron N, Vesa SC, Deleanu D, Buzoianu AD. In vivo anti-inflammatory effect of H1 antihistamines in allergic rhinitis: a randomized clinical trial. Balkan medical journal. 2015;32(4):352-8. doi: 10.5152/balkanmedj.2015.15884. PubMed PMID: CN-01736009.

5567. Boczek C, Vigier C, Jacob C, Dumas S. Drug shortages in retail pharmacies: which economic impact, management difficulties and challenges for hospital pharmacies? International Journal of Clinical Pharmacy. 2013;35(6):1263-4. PubMed PMID: WOS:000328208800060.

5568. Bodaghi B, Korobelnik JF, Cochereau I, Hajjar J, Goebel F, Dumarcet N. Injections intravitréennes : mise au point de l'AFSSAPS. Journal Français d'Ophtalmologie. 2012;35(1):69-71. doi: https://doi.org/10.1016/j.jfo.2011.11.005.

5569. Boddapati V, Danford NC, Lopez CD, Levine WN, Lehman RA, Lenke LG. Recent Trends in Private Equity Acquisition of Orthopaedic Practices in the United States. Journal of the American Academy of Orthopaedic Surgeons. 2022;30(8):E664-E72. doi: 10.5435/jaaos-d-21-00783. PubMed PMID: WOS:000781011600010.

5570. Bode G, Clausing P, Gervais F, Loegsted J, Luft J, Nogues V, et al. The utility of the minipig as an animal model in regulatory toxicology. Journal of pharmacological and toxicological methods. 2010;62(3):196-220.

5571. Bodega-Azuara J, Medall MDB, Edo-Penarrocha J, Pupla-Bartoll A, Piqueres RF, Torres-Gorriz MC, et al. Beta-lactam allergy in patients: an antibiotic stewardship challenge. European Journal of Hospital Pharmacy. doi: 10.1136/ejhpharm-2022-003304. PubMed PMID: WOS:000902594900001.

5572. Bodein I, Forestier M, Le Borgne C, Lefebvre JM, Pinçon C, Garat A, et al. Evaluation of simulation-based training program intended to improve interprofessional communication skills of community pharmacy and general medicine students. Annales Pharmaceutiques Francaises. 2022. doi: 10.1016/j.pharma.2022.06.008.

5573. Bodein I, Forestier M, Le Borgne C, Lefebvre JM, Pinçon C, Garat A, et al. Evaluation of simulation-based training program intended to improve interprofessional communication skills of community pharmacy and general medicine students. Annales Pharmaceutiques Francaises. 2023;81(2):354-65. doi: 10.1016/j.pharma.2022.06.008.

5574. Bodek A, Pommée M, Berger A, Giraki M, Müller BS, Schütze D. Blackbox error management: how do practices deal with critical incidents in everyday practice? A qualitative interview study. BMC Primary Care. 2023;24(1):1-9. doi: 10.1186/s12875-023-02206-2. PubMed PMID: 173926447.

5575. Bodenheimer T, Chen E, Bennett HD. Confronting the growing burden of chronic disease: can the US health care workforce do the job? Health affairs. 2009;28(1):64-74.

5576. Bodenheimer T, Grumbach K. Electronic technology: a spark to revitalize primary care? Jama. 2003;290(2):259-64.

5577. Bodenheimer T, Laing BY. The teamlet model of primary care. The Annals of Family Medicine. 2007;5(5):457-61.

5578. Bodenheimer TS, Smith MD. Primary care: proposed solutions to the physician shortage without training more physicians. Health Affairs. 2013;32(11):1881-6.

5579. Bodhani A, West D, Li C, Ounpraseuth S, Pace A. Psychometric assessment of an instrument to measure patient satisfaction with medication therapy management services. Value in Health. 2009;12(3):A30.

PubMed PMID: 70015286.

5580. Bodle J. Drug and alcohol misuse in pregnancy. Antenatal Disorders for the MRCOG and Beyond2016. p. 169-88.

5581. Boehler M, Schechtman B, Rivero R, Jacob B-A, Sherer R, Wagner C, et al. Developing the HIV Workforce: The MATEC Clinician Scholars Program. Journal of the Association of Nurses in AIDS Care. 2016;27(3):246-60. doi: https://doi.org/10.1016/j.jana.2015.06.006.

5582. Boehm LM, Danesh V, Eaton TL, McPeake J, Pena MA, Bonnet KR, et al. Multidisciplinary ICU Recovery Clinic Visits: A Qualitative Analysis of Patient-Provider Dialogues. Chest. 2022. doi: https://doi.org/10.1016/j.chest.2022.10.001.

5583. Boehm LM, Danesh V, Eaton TL, McPeake J, Pena MA, Bonnet KR, et al. Multidisciplinary ICU Recovery Clinic Visits: A Qualitative Analysis of Patient-Provider Dialogues. Chest. 2023;163(4):843-54. doi: https://doi.org/10.1016/j.chest.2022.10.001.

5584. Boehme S, Wohlt P, Valentine J, Ensign R. Sustained barcode medication administration rates less than 2 percent in a large healthcare system. Journal of Patient Safety & Risk Management. 2022;27(6):268-74. doi: 10.1177/25160435221137145. PubMed PMID: 160562850. Language: English. Entry Date: 20221215. Revision Date: 20221220. Publication Type: Article.

5585. Boeni F, Hersberger KE, Arnet I. Multidrug punch cards in primary care: a mixed methods study on patients' preferences and impact on adherence. Frontiers in Pharmacology. 2014;5:220. PubMed PMID: 25324777.

5586. Boer CG, Szilagyi I, Nguyen NL, Neogi T, Ikram MA, Uitterlinden AG, et al. Vitamin K antagonist usage is associated with increased incidence and progression of osteoarthritis. Osteoarthritis and cartilage. 2020;28:S405-. doi: 10.1016/j.joca.2020.02.633. PubMed PMID: CN-02096793.

5587. Boermans IM, Dijken CWA. The Dutch Occupational Health and Safety Act and drug compounding in community pharmacies. Part 2. Drug preparation and exposure. Pharmaceutisch Weekblad. 2000;135(42):1566-7.

5588. Boermeester MA. Effect of a ward-based pharmacy team on preventable adverse drug events in surgical patients (SUREPILL study). British journal of surgery. 2015;102(10):1204-12. doi: 10.1002/bjs.9876. PubMed PMID: CN-02146837.

5589. Boersma C, Postma MJ, Visser ST, Atthobari J, De Jong PE, De Jong-Van Den Berg LTW, et al. Baseline albuminuria predicts the efficacy of blood pressure-lowering drugs in preventing cardiovascular events. British Journal of Clinical Pharmacology. 2008;65(5):723-32. doi: 10.1111/j.1365-2125.2008.03097.x.

5590. Boersma C, Postma MJ, Visser ST, Atthobari J, de Jong PE, den Berg L, et al. Baseline albuminuria predicts the efficacy of blood pressure-lowering drugs in preventing cardiovascular events. British Journal of Clinical Pharmacology. 2008;65(5):723-32. doi: 10.1111/j.1365-2125.2008.03097.x. PubMed PMID: WOS:000254930800014.

5591. Boersma HH, Van Hulst W. Individualized care by the community pharmacy. Introduction and protocolling of compliance pack distribution. Pharmaceutisch Weekblad. 1996;131(41):1169-73.

5592. Boesen KP, Perera PN, Guy MC, Sweaney AM. Evaluation of prescriber responses to pharmacist recommendations communicated by fax in a medication therapy management program (MTMP). Journal of

Managed Care Pharmacy. 2011;17(5):345-54.

5593. Boeuf O, Lapeyre-Mestre M. Survey of forged prescriptions to investigate risk of psychoactive medications abuse in France: Results of OSIAP survey. Drug Safety. 2007;30(3):265-76. doi: 10.2165/00002018-200730030-00007.

5594. Boeuf O, Lapeyre-Mestre M, Ceip. Survey of forged prescriptions to investigate risk of psychoactive medications abuse in France - Results of OSIAP survey. Drug Safety. 2007;30(3):265-76. doi: 10.2165/00002018-200730030-00007. PubMed PMID: WOS:000245504300008.

5595. Bofí Martínez P, García Jiménez E, Martínez Martínez F. Comparison of health education and drug therapy monitoring interventions in patients with cardiovascular risk factors attending a community pharmacy (FISFTES-PM Study). Atencion primaria / Sociedad Espanola de Medicina de Familia y Comunitaria. 2015;47(3):141-8. doi: 10.1016/j.aprim.2014.04.012. PubMed PMID: CN-01254269.

5596. Bogaert P, Bochenek T, Prokop A, Pilc A. A qualitative approach to a better understanding of the problems underlying drug shortages, as viewed from Belgian, French and the European Union's perspectives. PloS one. 2015;10(5):e0125691.

5597. Bogaerts C, Schoenmaekers N, Haems M, Storme M, De Loof H. A quality improvement study of the implementation and initial results of a pragmatic clinical decision support system in the community pharmacy setting. International Journal of Clinical Pharmacy. 2024;46(1):141-9. doi: 10.1007/s11096-023-01648-z. PubMed PMID: WOS:001104829800001.

5598. Bogale AA, Amhare AF, Chang J, Bogale HA, Betaw ST, Gebrehiwot NT, et al. Knowledge, attitude, and practice of self-medication with antibiotics among community residents in Addis Ababa, Ethiopia. Expert Review of Anti-Infective Therapy. 2019;17(6):459-66. doi: 10.1080/14787210.2019.1620105. PubMed PMID: WOS:000469721800001.

5599. Bogden PE, Abbott RD, Williamson P, Onopa JK, Koontz LM. Comparing standard care with a physician and pharmacist team approach for uncontrolled hypertension. Journal of general internal medicine. 1998;13(11):740-5. doi: 10.1046/j.1525-1497.1998.00225.x. PubMed PMID: CN-00157273.

5600. Bogden PE, Koontz LM, Williamson P, Abbott RD. The physician and pharmacist team. An effective approach to cholesterol reduction. Journal of general internal medicine. 1997;12(3):158-64. doi: 10.1007/s11606-006-5023-7. PubMed PMID: CN-00138530.

5601. Boggs JM, Simon GE, Beck A, Rossom RC, Lynch FL, Lu CY, et al. Are People Who Die by Intentional Medication Poisoning Dispensed Those Medications in the Year Prior to Death? Archives of Suicide Research. 2023;27(3):1083-90. doi: 10.1080/13811118.2022.2072253. PubMed PMID: 166103917.

5602. Bogle KE, Smith BH. Illicit methylphenidate use: a review of prevalence, availability, pharmacology, and consequences. Current drug abuse reviews. 2009;2(2):157-76.

5603. Bogner MS. Human error in medicine: CRC Press; 2018.

5604. Bograd H, Ritzwoller DP, Calonge N, Shields K, Hanrahan M. Extending health maintenance organization insurance to the uninsured - A controlled measure of health care utilization. Jama-Journal of the American Medical Association. 1997;277(13):1067-72. doi: 10.1001/jama.277.13.1067. PubMed PMID: WOS:A1997WQ08900033.

5605. Bohand X, Aupee O, Le Garlantezec P, Mullot H, Lefeuvre L, Simon L. Medication dispensing errors in a French military hospital pharmacy. Pharmacy World & Science. 2009;31(4):432-8. PubMed PMID:

19306071.

5606. Bohand X, Simon L, Perrier E, Mullot H, Lefeuvre L, Plotton C. Frequency, Types, and Potential Clinical Significance of Medication-Dispensing Errors. Clinics. 2009;64(1):11-6. doi: https://doi.org/10.1590/S1807-59322009000100003.

5607. Bohl AA, Phelan EA, Fishman PA, Harris JR. How are the costs of care for medical falls distributed? The costs of medical falls by component of cost, timing, and injury severity. Gerontologist. 2012;52(5):664-75. doi: 10.1093/geront/gnr151.

5608. Bohl CJ, Parks A. A Mnemonic for Pharmacists to Ensure Optimal Monitoring and Safety of Total Parenteral Nutrition: I AM FULL. Annals of Pharmacotherapy. 2017;51(7):603-13. PubMed PMID: 28622737.

5609. Bohlen KN, Kittelsrud JM, Nelson ME, Weisser LK, Matthiesen NJ, Fieldsend JA, et al. Clinical utility of pharmacogenetics in a psychiatric and primary care population. Pharmacogenomics journal. 2022. doi: 10.1038/s41397-022-00292-6. PubMed PMID: CN-02493263.

5610. Bohn BC, Neuner EA, Athans V, Wesolowski J, Fraser TG, Gordon SM. Applying antimicrobial consumption metrics to characterize inpatient stewardship opportunities. Open forum infectious diseases. 2019;6:S709-. doi: 10.1093/ofid/ofz360.1778. PubMed PMID: CN-02075056.

5611. Bohnert AS, Guy Jr GP, Losby JL. Opioid prescribing in the United States before and after the Centers for Disease Control and Prevention's 2016 opioid guideline. Annals of internal medicine. 2018;169(6):367-75.

5612. Bohomol E, Ramos LH, D'Innocenzo M. Medication errors in an intensive care unit. Journal of Advanced Nursing (Wiley-Blackwell). 2009;65(6):1259-67. doi: 10.1111/j.1365-2648.2009.04979.x. PubMed PMID: 38015032. Language: English. Entry Date: 20090619. Revision Date: 20200708. Publication Type: Article. Journal Subset: Core Nursing.

5613. Boiano JM, Steege AL, Sweeney MH. Adherence to Safe Handling Guidelines by Health Care Workers Who Administer Antineoplastic Drugs. Journal of Occupational and Environmental Hygiene. 2014;11(11):728-40. doi: 10.1080/15459624.2014.916809. PubMed PMID: WOS:000342283700009.

5614. Boinet T, David CL. Alcoolodépendance chez l'adulte. Actualités Pharmaceutiques. 2020;59(595):13-7. doi: https://doi.org/10.1016/j.actpha.2020.03.006.

5615. Boinet T, Leroy-David C. Hypertension artérielle essentielle chez l'adulte. Actualités Pharmaceutiques. 2020;59(601):13-7. doi: https://doi.org/10.1016/j.actpha.2020.10.016.

5616. Boire G, Cabana F, Beaulieu M, Mikhail S, Lambert D. Who is not treated following the implementation of optimus, a successful initiative to treat osteoporosis following a fragility fracture. Journal of bone and mineral research. 2010;25:S340. doi: 10.1002/jbmr.5650251304. PubMed PMID: CN-01060883.

5617. Boisen L. Going viral: a critical, post-structural exploration of feminist culture jamming as cultural therapeutic [Ph.D.]. United States -- Pennsylvania: Duquesne University; 2015.

5618. Boiteux-Jurain M, Bichard D, Chabanon P, Berthou J, Medjoub M, Cornette C, et al. Mise sous assurance qualité de l'analyse pharmaceutique : évaluation des pratiques. Le Pharmacien Hospitalier et Clinicien. 2017;52(3):260-8. doi: https://doi.org/10.1016/j.phclin.2017.02.002.

5619. Boiteux-Jurain M, Limousin-Dubois L, Berthou-Contreras J, Bichard D, Limat S, Clairet AL. Analyse

de huit ans d'interventions pharmaceutiques: description et identification des situations à risque d'iatrogénie. Le Pharmacien Clinicien. 2022;57(4):317-27. doi: https://doi.org/10.1016/j.phacli.2022.04.009.

5620. Bojanic L, Markovic-Pekovic V, Skrbic R, Stojakovic N, Dermanovic M, Bojanic J, et al. Recent Initiatives in the Republic of Srpska to Enhance Appropriate Use of Antibiotics in Ambulatory Care; Their Influence and Implications. Frontiers in Pharmacology. 2018;9. doi: 10.3389/fphar.2018.00442. PubMed PMID: WOS:000433329100001.

5621. Bojar I, Sarecka-Hujar B, Owoc J, Pawelczak-Barszczowska A, Raczkiewicz D. Self-assessment of Polish pharmacy staff's readiness to promote health. International Journal of Clinical Pharmacy. 2020;42(5):1354-63. doi: 10.1007/s11096-020-01099-w. PubMed PMID: WOS:000557347200001.

5622. Bojilova-Dor L, Pinkney K, Cauff B, Kramer D, Schaefer AM, Ballestas C, et al. Successful Outpatient Administration of Blinatumomab Infusion in Pediatric Patients with Acute Lymphoblastic Leukemia. Blood. 2021;138:4028. doi: https://doi.org/10.1182/blood-2021-148865.

5623. Bojke C, Philips Z, Sculpher M, Campion P, Chrystyn H, Coulton S, et al. Cost-effectiveness of shared pharmaceutical care for older patients: RESPECT trial findings. British Journal of General Practice. 2010;60(570). doi: 10.3399/bjgp09X482312. PubMed PMID: WOS:000274611100007.

5624. Bojuwoye AO, Suleman F, Perumal-Pillay VA. Polypharmacy and the occurrence of potential drug-drug interactions among geriatric patients at the outpatient pharmacy department of a regional hospital in Durban, South Africa. Journal of Pharmaceutical Policy and Practice. 2022;15(1). doi: 10.1186/s40545-021-00401-z. PubMed PMID: WOS:000738583300001.

5625. Bokam YK, Guntupalli C, Gudhanti S, Manne R, Alavala RR, Alla NR. Importance of pharmacists as a front line warrior in improving medication compliance in Covid 19 patients. Indian journal of pharmaceutical sciences. 2021;83(2):398-401. doi: 10.36468/pharmaceutical-sciences.788. PubMed PMID: CN-02275022.

5626. Boland B, Dalleur O, Losseau C, Spinewine A. Reduction of inappropriate medications in frail older inpatients through recommendations by a geriatrician within the internal liaison team. European geriatric medicine. 2012;3:S120-. doi: 10.1016/j.eurger.2012.07.289. PubMed PMID: CN-01004624.

5627. Boland B, Marien S, Sibille FX, Locke S, Mouzon A, Spinewine A, et al. Clinical appraisal of STOPP/START version 3 criteria. European Geriatric Medicine. 2023;14(5):1149-50. doi: https://dx.doi.org/10.1007/s41999-023-00839-1. PubMed PMID: 2024649355.

5628. Bolas H, Brookes K, Scott M, McElnay J. Evaluation of a hospital-based community liaison pharmacy service in Northern Ireland. Pharmacy world & science : PWS. 2004;26(2):114-20. doi: 10.1023/b:phar.0000018601.11248.89. PubMed PMID: CN-00468980.

5629. Bolduc C, McCall K, 3rd, Stickney K, Gelinas A, Levesque E. Applicability of a new specialty pharmacy-reported measure describing completion of therapy for hepatitis C. Journal of Managed Care & Specialty Pharmacy. 2021;27(2):263-7. PubMed PMID: 33506724.

5630. Boleda Relats X, editor Roundtable: Prevention and detection of health problems in community pharmacy. "Screening of streptococcal infection in community pharmacy: Streptotest". Ars Pharmaceutica; 2014.

5631. Bolesta S, Berger A, Black E, Greskovic GA, Davis TW. Disease and medication burden of medically complex adolescents seen in an interdisciplinary transitional care clinic. Journal of Pediatric Rehabilitation Medicine. 2021;14(4):605-12. PubMed PMID: 34864698.

5632. Bolger AP, Bartlett FR, Penston HS, O'Leary J, Pollock N, Kaprielian R, et al. Intravenous iron alone for the treatment of anemia in patients with chronic heart failure. Journal of the American College of Cardiology. 2006;48(6):1225-7.

5633. Bolger GT. From B.Sc. to Ph.D., my shuffle off to Buffalo. Biochemical Pharmacology. 2015;98(2):283-91. doi: https://doi.org/10.1016/j.bcp.2015.06.019.

5634. Bolin JN, Ory MG, Wilson AD, Salge L. Diabetes Education Kiosks in a Latino Community. The Diabetes Educator. 2013;39(2):204-12. doi: 10.1177/0145721713476346.

5635. Boling B, McKibben M, Hingl J, Worth P, Jacobs BR. Effectiveness of computerized provider order entry with dose range checking on prescribing forms. Journal of Patient Safety. 2005;1(4):190-4. doi: 10.1097/01.jps.0000215339.03807.fd. PubMed PMID: 109848981. Corporate Author: Clinical Informatics Outcomes Research Group. Language: English. Entry Date: 20080425. Revision Date: 20210407. Publication Type: Journal Article.

5636. Boling PA. Care transitions and home health care. Clinics in geriatric medicine. 2009;25(1):135-48.

5637. Bolivar D, Hartung D, Silcox J, Bratberg J, Boggis J, Rabin M, et al. Examining the effects of COVID-19 on pharmacy dispensing of naloxone and syringes sales across Massachusetts and New Hampshire. Journal of the American Pharmacists Association. 2023;63(1):330-5.e1. doi: https://doi.org/10.1016/j.japh.2022.10.012.

5638. Bolivar D, Hartung D, Silcox J, Bratberg J, Boggis J, Rabin M, et al. Examining the effects of COVID-19 on pharmacy dispensing of naloxone and syringes sales across Massachusetts and New Hampshire. Journal of the American Pharmacists Association. 2023;63(1):330-5.e1. doi: https://doi.org/10.1016/j.japh.2022.10.012.

5639. Bollella G, Angley MT, Pink JA, Caird CJ, Goldsworthy SJ. Optimal level of liaison pharmacist intervention to facilitate a post-discharge home medicines review. Journal of pharmacy practice and research. 2008;38(2):107-10. PubMed PMID: CN-00708209.

5640. Bollen A, Harrison R, Aslani P, van Haastregt JC. Factors influencing interprofessional collaboration between community pharmacists and general practitioners—A systematic review. Health & social care in the community. 2019;27(4):e189-e212.

5641. Bollmeier SG, Seaton TL, Prosser TR, Chou Y-T, Reckenberg K, Hahn B, et al. Assessment of symptom burden and adherence to respiratory medications in individuals self-reporting a diagnosis of COPD within a community pharmacy setting. Journal of the American Pharmacists Association. 2019;59(4):479-88.e1. doi: https://doi.org/10.1016/j.japh.2019.04.017.

5642. Bollmeier SG, Seaton TL, Prosser TR, Chou YT, Reckenberg K, Hahn B, et al. Assessment of symptom burden and adherence to respiratory medications in individuals self-reporting a diagnosis of COPD within a community pharmacy setting. Journal of the American Pharmacists Association. 2019;59(4):479-88.e1. doi: 10.1016/j.japh.2019.04.017.

5643. Bolot C, Bourrel F, Biechlin-Chassel ML, Desruet MD, Lao S. Les essais cliniques en médecine nucléaire. Médecine Nucléaire. 2010;34(11):612-9. doi: https://doi.org/10.1016/j.mednuc.2010.06.014.

5644. Bolot C, Mendjel-Herda M, Saibi-Hajji S, Grandeau E, Blondeel-Gomes S. Utilisation des médicaments radiopharmaceutiques en pédiatrie : spécificités et recommandations de la SoFRa (Société française de radiopharmacie). Annales Pharmaceutiques Françaises. 2021;79(3):230-43. doi: https://doi.org/10.1016/j.pharma.2020.10.013.

5645. Bolouri F, Tabrizi NM, Tanha FD, Niroomand N, Azmoodeh A, Emami S, et al. Effectiveness of fluconazole for suppressive maintenance therapy in patients with RVVC: a randomized placebo-controlled study. Iranian journal of pharmaceutical research. 2009;8(4):307-13. PubMed PMID: CN-00802865.

5646. Bolster D, Manias E. Person-centred interactions between nurses and patients during medication activities in an acute hospital setting: Qualitative observation and interview study. International journal of nursing studies. 2010;47(2):154-65.

5647. Bolt J, Park E, Wong K, Lane M, Gorman SK. Retrospective cross-sectional analysis of potentially inappropriate medication use in ambulatory seniors with geriatric syndromes. Drugs & Therapy Perspectives. 2022;38(3):156-63. doi: 10.1007/s40267-022-00904-y. PubMed PMID: 155957109. Language: English. Entry Date: 20220420. Revision Date: 20220420. Publication Type: Article.

5648. Bombah AE, Tene LN, Essomba NC, Same-Ekobo A. Management of presumptive malaria in pharmacies in Cameroon: case of 30 private pharmacies in Yaounde. Transactions of the Royal Society of Tropical Medicine and Hygiene. 2019;113:S172-. doi: 10.1093/trstmh/trz090. PubMed PMID: CN-02074914.

5649. Bonadiman RL, Santanna AF, Brasil GA, de Lima EM, Lenz D, Endringer DC, et al. Satisfaction levels of users and verification of the knowledge of pharmacists in public pharmacies in the State of Espirito Santo, Brazil. Ciencia & Saude Coletiva. 2018;23(2):627-38. doi: 10.1590/1413-81232018232.03462016. PubMed PMID: WOS:000424384800028.

5650. Bonafede MM, Suaya JA, Wilson KL, Mannino DM, Polsky D. Incidence and cost of CAP in a large working-age population. American Journal of Managed Care. 2012;18(7):380-7.

5651. Bonam SP. Preparation and Evaluation of Pluronic Lecithin Organogel Containing Ricinoleic acid for Transdermal Drug Delivery [M.S.P.S.]. United States -- Ohio: The University of Toledo; 2013.

5652. Bonar S, Schneider HL, Cook K, Samala RV, Poole MFF. Olanzapine for Non-Chemotherapy Related Nausea and Emesis in Patients with a Palliative Care Consult. Journal of Pain & Symptom Management. 2023;66(4):e455-e9. doi: 10.1016/j.jpainsymman.2023.06.019. PubMed PMID: 172024447.

5653. Bond C. The MEDMAN study: A randomized controlled trial of community pharmacy-led medicines management for patients with coronary heart disease. Family Practice. 2007;24(2):189-200. doi: 10.1093/fampra/cml075.

5654. Bond C. What will this decade bring? International Journal of Pharmacy Practice. 2011;19(1):1-2. PubMed PMID: 21235652.

5655. Bond C. Pharmacy: Finally part of the team? British Journal of General Practice. 2015;65(639):e638-e9. doi: 10.3399/bjgp15X686797.

5656. Bond C, Blenkinsopp A, Inch J, Celino G, Gray N. The effect of the new community pharmacy contract on the community pharmacy workforce. London: Pharmacy Practice Research Trust. 2008;8:9.

5657. Bond C, Hannaford P. Issues Related to Monitoring the Safety of Over-The-Counter (OTC) Medicines. Drug Safety. 2003;26(15):1065-74. doi: 10.2165/00002018-200326150-00001.

5658. Bond C, Maskrey V, Alldred D, Blyth A, Daffu-O'Reilly A, Inch J, et al. Care Homes Independent Pharmacist Prescribing Study (CHIPPS): experiences from a non-randomised feasibility study. International journal of pharmacy practice. 2017;25:5-6. doi: 10.1111/ijpp.12416. PubMed PMID: CN-01451048.

5659. Bond C, Matheson C, Williams S, Williams P, Donnan P. Repeat prescribing: a role for community

pharmacists in controlling and monitoring repeat prescriptions. British journal of general practice. 2000;50(453):271-5. PubMed PMID: CN-00298305.

5660. Bond C, Morgenstern J, Heitz C, Milne WK. Hot off the Press: SGEM #226. I Want a New Drug-One That Doesn't Cause an Adverse Drug Event. Academic Emergency Medicine. 2019;26(4):447-50. PubMed PMID: 30207622.

5661. Bond C, Morgenstern J, Heitz C, Milne WK, Choo EK. Hot off the Press: SGEM #226. I Want a New Drug—One That Doesn't Cause an Adverse Drug Event. Academic Emergency Medicine. 2019;26(4):447-50. doi: 10.1111/acem.13566. PubMed PMID: 135845359. Language: English. Entry Date: 20190415. Revision Date: 20200331. Publication Type: Article.

5662. Bond C, Raehl CL. Clinical pharmacy services, pharmacy staffing, and hospital mortality rates. Pharmacotherapy: The Journal of Human Pharmacology and Drug Therapy. 2007;27(4):481-93.

5663. Bond C, Raehl CL. 2006 national clinical pharmacy services survey: clinical pharmacy services, collaborative drug management, medication errors, and pharmacy technology. Pharmacotherapy: The Journal of Human Pharmacology and Drug Therapy. 2008;28(1):1-13.

5664. Bond C, Raehl CL, Franke T. Medication errors in United States hospitals. Pharmacotherapy: The Journal of Human Pharmacology and Drug Therapy. 2001;21(9):1023-36.

5665. Bond C, Raehl CL, Franke T. Clinical pharmacy services, hospital pharmacy staffing, and medication errors in United States hospitals. Pharmacotherapy: The Journal of Human Pharmacology and Drug Therapy. 2002;22(2):134-47.

5666. Bond C, Raehl CL, Patry R. The feasibility of implementing an evidence-based core set of clinical pharmacy services in 2020: manpower, marketplace factors, and pharmacy leadership. Pharmacotherapy: The Journal of Human Pharmacology and Drug Therapy. 2004;24(4):441-52.

5667. Bond CA, Raehl CL, Franke T. Medication errors in United States hospitals. Pharmacotherapy:The Journal of Human Pharmacology & Drug Therapy. 2001;21(9):1023-36. PubMed PMID: 11560192.

5668. Bond CA, Raehl CL, Patry R. The feasibility of implementing an evidence-based core set of clinical pharmacy services in 2020: Manpower, marketplace factors, and pharmacy leadership. Pharmacotherapy. 2004;24(4):441-52. doi: 10.1592/phco.24.5.441.33354. PubMed PMID: WOS:000220624600003.

5669. Bond CM. Adherence: The Holy Grail? BMJ Quality and Safety. 2016;25(10):736-8. doi: 10.1136/bmjqs-2015-004886.

5670. Bond CM, Fish A, Porteous TH, Reid JP, Scott A, Antonazzo E. A randomised controlled trial of the effects of note-based medication review by community pharmacists on prescribing of cardiovascular drugs in general practice. International journal of pharmacy practice. 2007;15(1):39-46. doi: 10.1211/ijpp.15.1.0007. PubMed PMID: CN-00642268.

5671. Bond CM, Grimshaw JM. Multi-disciplinary guideline development: a case study from community pharmacy. Health bulletin. 1995;53(1):26-33.

5672. Bond CM, Holland R, Alldred DP, Arthur A, Barton G, Birt L, et al. Protocol for the process evaluation of a cluster randomised controlled trial to determine the effectiveness and cost-effectiveness of independent pharmacist prescribing in care home: the CHIPPS study. Trials. 2020;21(1):439. doi: 10.1186/s13063-020-04264-8. PubMed PMID: CN-02130911.

5673. Bond CM, Holland R, Alldred DP, Arthur A, Barton G, Birt L, et al. Protocol for the process evaluation of a cluster randomised controlled trial to determine the effectiveness and cost-effectiveness of independent pharmacist prescribing in care home: the CHIPPS study. Trials. 2020;21(1):1-10. doi: 10.1186/s13063-020-04264-8. PubMed PMID: 143492178. Corporate Author: CHIPPS Team. Language: English. Entry Date: In Process. Revision Date: 20210223. Publication Type: journal article. Journal Subset: Biomedical.

5674. Bond CM, Holland R, Alldred DP, Arthur A, Barton G, Birt L, et al. Protocol for the process evaluation of a cluster randomised controlled trial to determine the effectiveness and cost-effectiveness of independent pharmacist prescribing in care home: the CHIPPS study. Trials [Electronic Resource]. 2020;21(1):439. PubMed PMID: 32471514.

5675. Bond CM, Holland R, Alldred DP, Arthur A, Barton G, Blyth A, et al. Protocol for a cluster randomised controlled trial to determine the effectiveness and cost-effectiveness of independent pharmacist prescribing in care homes: the CHIPPS study. Trials. 2020;21(1):103. doi: 10.1186/s13063-019-3827-0. PubMed PMID: CN-02087939.

5676. Bond CM, Holland R, Alldred DP, Arthur A, Barton G, Blyth A, et al. Protocol for a cluster randomised controlled trial to determine the effectiveness and cost-effectiveness of independent pharmacist prescribing in care homes: the CHIPPS study. Trials. 2020;21(1):1-11. doi: 10.1186/s13063-019-3827-0. PubMed PMID: 141317918. Corporate Author: CHIPPS Team. Language: English. Entry Date: In Process. Revision Date: 20210111. Publication Type: journal article.

5677. Bond CM, Holland R, Alldred DP, Arthur A, Barton G, Blyth A, et al. Protocol for a cluster randomised controlled trial to determine the effectiveness and cost-effectiveness of independent pharmacist prescribing in care homes: the CHIPPS study. Trials [Electronic Resource]. 2020;21(1):103. PubMed PMID: 31964398.

5678. Bond CM, Watson MC. The development of evidence-based guidelines for over-the-counter treatment of vulvovaginal candidiasis. Pharmacy World and Science. 2003;25(4):177-81. doi: 10.1023/A:1024842712675.

5679. Bond CM, Watson MC, Grampian Evidence Based C. The development of evidence-based guidelines for over-the-counter treatment of vulvovaginal candidiasis. Pharmacy World & Science. 2003;25(4):177-81. doi: 10.1023/a:1024842712675. PubMed PMID: WOS:000184279000011.

5680. Bond KT, Jones K, Ompad DC, Vlahov D. Resources and Interest Among Faith Based Organizations for Influenza Vaccination Programs. Journal of Immigrant and Minority Health. 2013;15(4):758-63. doi: 10.1007/s10903-012-9645-z. PubMed PMID: WOS:000321241100013.

5681. Bond L, Smith E, Broom M, Kearns R. Pre-drawn diamorphine for obstetric anaesthesia: mitigating drug supply shortages and reducing spinal preparation time during the COVID-19 pandemic. Anaesthesia. 2021;76(SUPPL 2):22-. doi: 10.1111/anae.15338. PubMed PMID: CN-02245458.

5682. Bonderski V, Morrow DG, Chin J, Murray MD. Pharmacy-Based Approach to Improving Heart Failure Medication Use by Older Adults with Limited Health Literacy: learning from Interdisciplinary Experience. Drugs & aging. 2018;35(11):951-7. doi: 10.1007/s40266-018-0586-7. PubMed PMID: CN-01644298.

5683. Bondesson A, Eriksson T, Kragh A, Holmdahl L, Midlov P, Hoglund P. In-hospital medication reviews reduce unidentified drug-related problems. European Journal of Clinical Pharmacology. 2013;69(3):647-55. PubMed PMID: 22955893.

5684. Bondesson A, Holmdahl L, Midlov P, Hoglund P, Andersson E, Eriksson T. Acceptance and

importance of clinical pharmacists' LIMM-based recommendations. International Journal of Clinical Pharmacy. 2012;34(2):272-6. PubMed PMID: 22252773.

5685. Bondesson A, Holmdahl L, Midlöv P, Höglund P, Andersson E, Eriksson T, et al. Acceptance and importance of clinical pharmacists' LIMM-based recommendations. International Journal of Clinical Pharmacy. 2012;34(2):272-6. doi: 10.1007/s11096-012-9609-3. PubMed PMID: 104547131. Language: English. Entry Date: 20120810. Revision Date: 20211028. Publication Type: journal article.

5686. Bone K, Mills S. Principles and practice of phytotherapy: modern herbal medicine: Elsevier Health Sciences; 2012.

5687. Bonekamp NA, Peter B, Hillen HS, Felser A, Bergbrede T, Choidas A, et al. Small-molecule inhibitors of human mitochondrial DNA transcription. Nature. 2020;588(7839):712-6.

5688. Bonella GF, Alves LdS, Souza ARNDd, Silva CHMd. Prescribing errors in a Brazilian teaching hospital: Causes and underlying factors from the perspective of junior doctors. PLoS ONE. 2023;18(4):1-19. doi: 10.1371/journal.pone.0284071. PubMed PMID: 162917766.

5689. Bonella GF, Da SAL, De Souza AND, Da Silva CHM. Prescribing errors in a Brazilian teaching hospital: Causes and underlying factors from the perspective of junior doctors. PLoS ONE. 2023;18(4 April):e0284071. doi: https://dx.doi.org/10.1371/journal.pone.0284071. PubMed PMID: 2024105449.

5690. Bonetti AF, Bagatim BQ, Bottacin WE, Mendes AM, Rotta I, Reis RC, et al. Pharmacotherapy problems in cardiology patients 30 days post discharge from a tertiary hospital in Brazil: a randomized controlled trial. Clinics (Sao Paulo, Brazil). 2019;74:e1091. doi: 10.6061/clinics/2019/e1091. PubMed PMID: CN-02050015.

5691. Bonetti AF, Bagatim BQ, Mendes AM, Rotta I, Reis RC, Fávero MLD, et al. Impact of discharge medication counseling in the cardiology unit of a tertiary hospital in Brazil: a randomized controlled trial. Clinics (Sao Paulo, Brazil). 2018;73:e325. doi: 10.6061/clinics/2018/e325. PubMed PMID: CN-01642829.

5692. Bonetti AF, Reis WC, Lombardi NF, Mendes AM, Netto HP, Rotta I, et al. Pharmacist-led discharge medication counselling: A scoping review. Journal of Evaluation in Clinical Practice. 2018;24(3):570-9. PubMed PMID: 29691955.

5693. Bonetti AF, Reis WC, Mendes AM, Rotta I, Tonin FS, Fernandez-Llimos F, et al. Impact of Pharmacist-led Discharge Counseling on Hospital Readmission and Emergency Department Visits: A Systematic Review and Meta-analysis. Journal of Hospital Medicine. 2020;15(1):52-9. doi: 10.12788/jhm.3182. PubMed PMID: 141038251. Language: English. Entry Date: 20200715. Revision Date: 20211102. Publication Type: journal article.

5694. Bonetti AF, Tonin FS, Della Rocca AM, Lucchetta RC, Fernandez-Llimos F, Pontarolo R. Methodological quality and risk of bias of meta-analyses of pharmacy services: A systematic review. Research in Social and Administrative Pharmacy. 2022;18(3):2403-9. doi: https://doi.org/10.1016/j.sapharm.2020.12.011.

5695. Bonetti AF, Tonin FS, Lucchetta RC, Pontarolo R, Fernandez-Llimos F. Methodological standards for conducting and reporting meta-analyses: Ensuring the replicability of meta-analyses of pharmacist-led medication review. Research in Social and Administrative Pharmacy. 2022;18(2):2259-68. doi: https://doi.org/10.1016/j.sapharm.2021.06.002.

5696. Bonfiglio MF, Lewis JD, Nesbit SA, Krinsky DL. A Contemporary Perspective on Pharmacy's Traditional Strengths. Journal of the American Pharmaceutical Association (1996). 1997;37(6):700-4. doi:

https://doi.org/10.1016/S1086-5802(16)30263-7.

5697. Bongard A, Strub B, Berger J, Gouveia A. [Optimizing the therapeutic adherence in ambulatory care The link between patients, home care nurses, pharmacists and physicans]. Revue Medicale Suisse. 2022;18(803):2090-5. PubMed PMID: 36350019.

5698. Bonham CA, Patterson KC, Strek ME. Asthma Outcomes and Management During Pregnancy. Chest. 2018;153(2):515-27. doi: https://doi.org/10.1016/j.chest.2017.08.029.

5699. Bonk N, Milsap A, Goplen A, McElray K, Rabago D. Reducing Discharge Delay Through Resident-Pharmacist Colocation: A Pilot Study. Family Medicine. 2020;52(9):665-7. doi: 10.22454/FamMed.2020.708034. PubMed PMID: 146291839. Language: English. Entry Date: 20210619. Revision Date: 20211118. Publication Type: journal article. Journal Subset: Biomedical.

5700. Bonnabry P, Cingria L, Ackermann M, Sadeghipour F, Bigler L, Mach N. Use of a prospective risk analysis method to improve the safety of the cancer chemotherapy process. International Journal for Quality in Health Care. 2006;18(1):9-16. doi: intqhc/mzi082. PubMed PMID: 104743180. Language: English. Entry Date: 20110610. Revision Date: 20161117. Publication Type: journal article.

5701. Bonnan D, Amouroux F, Aulois-Griot M. Clinical medication review in French community pharmacy: Interest of a new pharmaceutical service for detection of drug related problems. Annales Pharmaceutiques Francaises. 2021;79(5):597-603. doi: 10.1016/j.pharma.2021.02.009.

5702. Bonnay M, Soeiro T, Megard R, Micallef J, Rolland B, Chappuy M. Usages et bon usage des benzodiazépines anxiolytiques et hypnotiques. Annales Médico-psychologiques, revue psychiatrique. 2021;179(8):748-56. doi: https://doi.org/10.1016/j.amp.2021.08.009.

5703. Bonneau A, Labadens I, Monchaud C. Accompagnement postgreffe à l'officine. Actualités Pharmaceutiques. 2021;60(605):36-40. doi: https://doi.org/10.1016/j.actpha.2021.02.009.

5704. Bonnefont-Rousselot D, Delpech M, Chatron P, Gueant JL, Le Bouc Y, Maquart FX, et al. Rapport 22-12. La biologie médicale en France : évolutions et enjeux. Bulletin de l'Académie Nationale de Médecine. 2023;207(1):3-15. doi: https://doi.org/10.1016/j.banm.2022.10.008.

5705. Bonnefont-Rousselot D, Delpech M, Chatron P, Gueant JL, Le Bouc Y, Maquart FX, et al. Laboratory medicine in France: Developments and challenges. Bulletin de l'Academie Nationale de Medecine. 2023;207(1):3-15. doi: 10.1016/j.banm.2022.10.008.

5706. Bonnefont-Rousselot D, Delpech M, Chatron P, Gueant JL, Le Bouc Y, Maquart FX, et al. Laboratory medicine in France: Developments and challenges. Bulletin de l'Academie Nationale de Medecine. 2023;207(1):3-15. doi: 10.1016/j.banm.2022.10.008.

5707. Bonnema CE, Van Woerden I, Steinberg JR, Nguyen E, Oliphant CM, Cleveland KW, et al. Understanding covid-19 vaccine hesitancy among students in health professions a cross-sectional analysis. Journal of Allied Health. 2021;50(4):314-20.

5708. Bonnemain B. Pierre Pomet (1658-1699) and his Histoire des drogues (History of drugs) (1694 & 1735). Revue d'histoire de la pharmacie. 2016;64(391):433-42. doi: 10.3406/pharm.2016.23180.

5709. Bonnemain B, Ricordel I. A brief history of toxicology in France during the last two centuries (1789–1989). Toxicology Reports. 2022;9:505-12. doi: https://doi.org/10.1016/j.toxrep.2022.03.005.

5710. Bonnerup DK, Lisby M, Saedder EA, Brock B, Truelshoj T, Sorensen CA, et al. Effects of stratified

medication review in high-risk patients at admission to hospital: a randomised controlled trial. Therapeutic advances in drug safety. 2020;11. doi: 10.1177/2042098620957142. PubMed PMID: CN-02178542.

5711. Bonnesen K, Pedersen L, Ehrenstein V, Sørensen HT, Lash TL, Schmidt M. Impact of hemoglobin A1c level on the association between non-steroidal anti-inflammatory drug use and cardiovascular events in patients with type 2 diabetes: A population-based cohort study. Pharmacoepidemiology and Drug Safety. 2023;32(11):1233-43. doi: 10.1002/pds.5652.

5712. Bonnet N. Intérêt d'un programme d'échange de seringues en pharmacie d'officine pour les usagers de drogues par voie intraveineuse. La Presse Médicale. 2006;35(12, Part 1):1811-8. doi: https://doi.org/10.1016/S0755-4982(06)74906-5.

5713. Bonnet N. Pharmacien et addictions, un engagement évident. Actualités Pharmaceutiques. 2022;61(615):33-7. doi: https://doi.org/10.1016/j.actpha.2022.02.008.

5714. Bonnet N, Beauverie P, Gaudoneix-Taïeb M, Poisson N, Imbert E, Fournier G. Changes in pharmacists practices (1996 - 2000) related to harm reduction policy (condoms, injecting equipment, methylmorphine) and maintenance therapy delivery (buprenorphine, methadone). Annales de Medecine Interne. 2001;152(7 SUPPL.):2S15-2S20.

5715. Bono JD, Crawford SY. Impact of Medicare Part D on independent and chain community pharmacies in rural Illinois-A qualitative study. Research in Social & Administrative Pharmacy. 2010;6(2):110-20. doi: 10.1016/j.sapharm.2009.11.007. PubMed PMID: WOS:000278771400004.

5716. Bonomi AE, Anderson ML, Rivara FP, Thompson RS. Health Care Utilization and Costs Associated with Physical and Nonphysical-Only Intimate Partner Violence. Health Services Research. 2009;44(3):1052-67. doi: 10.1111/j.1475-6773.2009.00955.x. PubMed PMID: WOS:000266111600016.

5717. Bonuck K, Drucker E, Tuchman E, Hartel D. Initiating Office Based Prescribing of Methadone: Experience of Primary Care Providers in New York City. Journal of Maintenance in the Addictions. 2003;2(3):19-34. doi: 10.1300/J126v02n03_03.

5718. Bony H, Lloyd RA, Hotham ED, Corre LJ, Corlis ME, Loffler HA, et al. Differences in the prescribing of potentially inappropriate medicines in older Australians: comparison of community dwelling and residential aged care residents. Scientific Reports. 2020;10(1):10170. PubMed PMID: 32576947.

5719. Boockvar KS, Blum S, Kugler A, Livote E, Mergenhagen KA, Nebeker JR, et al. Effect of admission medication reconciliation on adverse drug events from admission medication changes. Archives of internal medicine. 2011;171(9):860-1.

5720. Boockvar KS, Carlson LaCorte H, Giambanco V, Fridman B, Siu A. Medication reconciliation for reducing drug-discrepancy adverse events. The American Journal of Geriatric Pharmacotherapy. 2006;4(3):236-43. doi: https://doi.org/10.1016/j.amjopharm.2006.09.003.

5721. Boockvar KS, Ho W, Pruskowski J, DiPalo KE, Wong JJ, Patel J, et al. Effect of health information exchange on recognition of medication discrepancies is interrupted when data charges are introduced: results of a cluster-randomized controlled trial. Journal of the American Medical Informatics Association : JAMIA. 2017;24(6):1095-101. doi: 10.1093/jamia/ocx044. PubMed PMID: CN-01429589.

5722. Boockvar KS, Livote EE, Goldstein N, Nebeker JR, Siu A, Fried T. Electronic health records and adverse drug events after patient transfer. Quality & Safety in Health Care. 2010;19(5):e16-e. doi: 10.1136/qshc.2009.033050. PubMed PMID: 105014210. Language: English. Entry Date: 20101210. Revision Date: 20200708. Publication Type: Journal Article.

5723. Boockvar KS, Santos SL, Kushniruk A, Johnson C, Nebeker JR. Medication reconciliation: barriers and facilitators from the perspectives of resident physicians and pharmacists. Journal of Hospital Medicine (Online). 2011;6(6):329-37. PubMed PMID: 21834114.

5724. Boog K, Chen ZE, Cameron S. Sexual and reproductive healthcare providers' opinions on expansion of pharmacy-led provision of contraception. BMJ Sexual and Reproductive Health. 2019;45(3):183-9. doi: 10.1136/bmjsrh-2018-200252.

5725. Booij AD, de Boer WO, Kokenberg ME, Tromp TF. Interventions in seamless care. Pharmacy World & Science. 2003;25(2):41-2. PubMed PMID: 12774562.

5726. Booij AD, De Boer WO, Kokenberg MEAP, Tromp FJ, editors. Interventions in seamless care. Pharmacy World and Science; 2003.

5727. Bookman MA, Kloth DD, Kover PE, Smolinski S, Ozols RF. Short-course intravenous prophylaxis for paclitaxel-related hypersensitivity reactions. Annals of Oncology. 1997;8(6):611-4. doi: 10.1023/a:1008207025430. PubMed PMID: WOS:A1997XN79200025.

5728. Bookser M, Drennen C, Leonard E, McConaha J. Pharmacist-led medication intervention for patients with asthma and COPD within a primary care setting. Journal of the american pharmacists association. 2018;58(3):e24-. doi: 10.1016/j.japh.2018.04.004. PubMed PMID: CN-01612172.

5729. Boom FA, Brun PPHL, Bühringer S, Touw DJ. Microbiological monitoring during aseptic handling: Methods, limits and interpretation of results. European Journal of Pharmaceutical Sciences. 2020;155:105540. doi: https://doi.org/10.1016/j.ejps.2020.105540.

5730. Boom FA, Le Brun PPH, Bühringer S, Sirks M, Touw DJ. Microbiological controls of aseptic handling in Dutch hospital pharmacies: Results, limits, and methods for assessing. European Journal of Pharmaceutical Sciences. 2022;175:106228. doi: https://doi.org/10.1016/j.ejps.2022.106228.

5731. Boon MR, Hanssen MJW, Brans B, Hülsman CJM, Hoeks J, Nahon KJ, et al. Effect of L-arginine on energy metabolism, skeletal muscle and brown adipose tissue in South Asian and Europid prediabetic men: a randomised double-blinded crossover study. Diabetologia. 2019;62(1):112-22. doi: 10.1007/s00125-018-4752-6. PubMed PMID: CN-01943982.

5732. Boone CG, Bishop AC, Ivers N, Desveaux L, Tadrous M. Audit and feedback in community pharmacy: An untapped strategy of quality improvement. Canadian Pharmacists Journal. 2022;155(2):79-81. doi: 10.1177/17151635211056528.

5733. Boone JM, Kues JR. Interprofessional Clinical Education. In: Babar Z-U-D, editor. Encyclopedia of Pharmacy Practice and Clinical Pharmacy. Oxford: Elsevier; 2019. p. 119-33.

5734. Boonstra E, Lindbaek M, Ngome E, Tshukudu K, Fugelli P. Labelling and patient knowledge of dispensed drugs as quality indicators in primary care in Botswana. Quality & safety in health care. 2003;12(3):168-75. doi: 10.1136/qhc.12.3.168. PubMed PMID: CN-00438156.

5735. Boonyaprapa S. Self-care in pregnancy and breastfeeding: views of women and community pharmacists in thailand [Ph.D.]. England: The University of Nottingham (United Kingdom); 2010.

5736. Boorman RS, D'Arcy J. Questions have arisen in my mind (multiple letters) [1]. Pharmaceutical Journal. 2005;274(7341):334.

5737. Booth C. Setting up a pharmacist-led chemotherapy clinic: A practical guide. European Journal of

Oncology Pharmacy. 2012;5(3-4):28-9.

5738. Booth JL, Mullen AB, Thomson DA, Johnstone C, Galbraith SJ, Bryson SM, et al. Antibiotic treatment of urinary tract infection by community pharmacists: a cross-sectional study. British Journal of General Practice. 2013;63(609):E244-E9. doi: 10.3399/bjgp13X665206. PubMed PMID: WOS:000325522800002.

5739. Booth JN, Colantonio LD, Rosenson RS, Safford MM, Chen LG, Kilgore ML, et al. Healthcare Utilization and Statin Re-Initiation Among Medicare Beneficiaries With a History of Myocardial Infarction. Journal of the American Heart Association. 2018;7(10). doi: 10.1161/jaha.117.008462. PubMed PMID: WOS:000432333300025.

5740. Booth JP, Kennerly-Shah JM, Hartman AD. Description of the role of pharmacist independent double checks during cognitive order verification of outpatient parenteral anti-cancer therapy. Journal of Oncology Pharmacy Practice. 2022;28(2):381-6. doi: 10.1177/1078155221994319. PubMed PMID: 155142159. Language: English. Entry Date: 20220224. Revision Date: 20220224. Publication Type: Article.

5741. Booth S, Parsons R, Sunderland B, Sim TF. Managing migraine with over-the-counter provision of triptans: the perspectives and readiness of Western Australian community pharmacists. Peerj. 2019;7. doi: 10.7717/peerj.8134. PubMed PMID: WOS:000502758700002.

5742. Boothby LA, Doering PL, Jaco VT. When prescription drugs go over-the-counter: Where are the boundaries? Journal of Pharmacy Technology. 2004;20(4):215-20. doi: 10.1177/875512250402000402.

5743. Boothby LA, Wang LJ, Mayhew S, Chestnutt L. Academic detailing of meperidine at a teaching hospital. Hospital Pharmacy. 2003;38(1):30-5. doi: 10.1177/001857870303800111.

5744. Bootman JL. To err is human. Archives of Internal Medicine. 2000;160(21):3189. PubMed PMID: 11088077.

5745. Bootman JL, Boyle CJ, Chase P, Dipiro JT, Piascik P, Maine LL. Vital directions for pharmacy education and practice: report of the 2017-18 argus commission. American Journal of Pharmaceutical Education. 2018;82(7):874-84. doi: 10.5688/AJPE7161.

5746. Boqué C, Abad MR, Agustín MJ, García-Goñi M, Moreno C, Gabás-Rivera C, et al. Treatment decision-making in chronic lymphocytic leukaemia: Key factors for healthcare professionals. PRELIC study. Journal of Geriatric Oncology. 2020;11(1):24-30. doi: https://doi.org/10.1016/j.jgo.2019.03.010.

5747. Bora G, Akgül Ö, Kıymaz EN. Investigation of antibiotic resistance genes of beta-hemolytic and non-hemolytic streptococci with molecular methods. Eastern Journal of Medicine. 2019;24(1):47-52. doi: 10.5505/ejm.2019.68542.

5748. Borah BJ, Burns ME, Shah ND. ASSESSING THE IMPACT OF HIGH DEDUCTIBLE HEALTH PLANS ON HEALTH-CARE UTILIZATION AND COST: A CHANGES-IN-CHANGES APPROACH. Health Economics. 2011;20(9):1025-42. doi: 10.1002/hec.1757. PubMed PMID: WOS:000294180500003.

5749. Borah BJ, Naessens JM, Glasgow AE, Bauer BA, Chon TY. Cost-effectiveness of acupuncture in an employee population: a retrospective analysis. Complementary therapies in medicine. 2017;31:14-9. doi: 10.1016/j.ctim.2017.01.002. PubMed PMID: CN-01327757.

5750. Boralkar A, Bobhate P, Khairnar A. Prospective cross-sectional study in evaluation of prescribing pattern of doctors for oncology treatment. Research Journal of Pharmacy and Technology. 2011;4(4):634-7.

5751. Bord S, Dernat S, Ouillon L, René-Martellet M, Vourc'h G, Lesens O, et al. Tick ecology and Lyme

borreliosis prevention: a regional survey of pharmacists' knowledge in Auvergne-Rhône-Alpes, France. Ticks and Tick-borne Diseases. 2022;13(3):101932. doi: https://doi.org/10.1016/j.ttbdis.2022.101932.

5752. Bord S, Dernat S, Ouillon L, René-Martellet M, Vourc'h G, Lesens O, et al. Tick ecology and Lyme borreliosis prevention: A regional survey of pharmacists' knowledge in Auvergne-Rhône-Alpes, France: Ticks and tick-born Disease (TTBD). Ticks and Tick-borne Diseases. 2022;13(3). doi: 10.1016/j.ttbdis.2022.101932.

5753. Borda LA, Någård M, Boulton DW, Venkataramanan R, Coppola P. A systematic review of pregnancy-related clinical intervention of drug regimens due to pharmacokinetic reasons. Frontiers in Medicine. 2023;10:1241456.

5754. Borde JP, Litterst S, Ruhnke M, Feik R, Hübner J, deWith K, et al. Implementing an intensified antibiotic stewardship programme targeting cephalosporin and fluoroquinolone use in a 200-bed community hospital in Germany. Infection. 2015;43(1):45-50. doi: 10.1007/s15010-014-0693-2.

5755. Borde JP, Nussbaum S, Hauser S, Hehn P, Hübner J, Sitaru G, et al. Implementing an intensified antibiotic stewardship programme targeting daptomycin use in orthopaedic surgery: a cost–benefit analysis from the hospital perspective. Infection. 2016;44(3):301-7. doi: 10.1007/s15010-015-0854-y.

5756. Borde JP, Nussbaum S, Hauser S, Hehn P, Hubner J, Sitaru G, et al. Implementing an intensified antibiotic stewardship programme targeting daptomycin use in orthopaedic surgery: a cost-benefit analysis from the hospital perspective. Infection. 2016;44(3):301-7. doi: 10.1007/s15010-015-0854-y. PubMed PMID: WOS:000377598400005.

5757. Borders-Hemphill V, Chan IZ, Brown K, Rider B, Taylor K, Chai G. Use of Proprietary Names by Prescribers for Generic Products. Therapeutic Innovation and Regulatory Science. 2018;52(2):256-60. doi: 10.1177/2168479017723399.

5758. Borek AJ, Wanat M, Sallis A, Ashiru-Oredope D, Atkins L, Beech E, et al. How can national antimicrobial stewardship interventions in primary care be improved? A stakeholder consultation. Antibiotics. 2019;8(4). doi: 10.3390/antibiotics8040207.

5759. Borenstein JE, Graber G, Saltiel E, Wallace J, Ryu S, Archi J, et al. Physician-pharmacist comanagement of hypertension: a randomized, comparative trial. Pharmacotherapy. 2003;23(2):209-16. doi: 10.1592/phco.23.2.209.32096. PubMed PMID: CN-00435402.

5760. Borer JS, Kansal AR, Dorman ED, Krotneva S, Zheng Y, Patel HK, et al. Budget impact of adding ivabradine to standard of care in patients with chronic systolic heart failure in the United States. Journal of Managed Care and Specialty Pharmacy. 2016;22(9):1064-71. doi: 10.18553/jmcp.2016.22.9.1064.

5761. Borg JJ, Aislaitner G, Pirozynski M, Mifsud S. Strengthening and rationalizing pharmacovigilance in the EU: where is Europe heading to? A review of the new EU legislation on pharmacovigilance. Drug Safety. 2011;34(3):187-97. PubMed PMID: 21332243.

5762. Borg MA, Scicluna EA. Over-the-counter acquisition of antibiotics in the Maltese general population. International journal of antimicrobial agents. 2002;20(4):253-7.

5763. Borg ML, Gimona A, Renoux A, Douin MJ, Djebbar F, Panis-Rouzier R. Pharmacokinetics of estradiol and estrone following repeated administration of Menorest®, a new estrogen transdermal delivery system, in menopausal women. Clinical drug investigation. 1995;10(2):96-103. PubMed PMID: CN-00169098.

5764. Borgelt LM, Franson KL, Nussbaum AM, Wang GS. The P harmacologic and C linical E ffects of M

edical C annabis. Pharmacotherapy: The Journal of Human Pharmacology and Drug Therapy. 2013;33(2):195-209.

5765. Borgelt-Hansen L. Oral Contraceptives: An Update on Health Benefits and Risks. Journal of the American Pharmaceutical Association (1996). 2001;41(6):875-86. doi: https://doi.org/10.1016/S1086-5802(16)31329-8.

5766. Borges A, Guidoni CM, Ferreira LD, Freitas OD, Pereira LRL. The pharmaceutical care of patients with type 2 diabetes mellitus. Pharmacy world and science. 2010;32(6):730-6. doi: 10.1007/s11096-010-9428-3. PubMed PMID: CN-00892499.

5767. Borges FSG. Caracterização da Complexidade da Terapêutica Geriátrica [Master's]. Portugal: Universidade da Beira Interior (Portugal); 2011.

5768. Borges M, Gouveia M, Fiorentino F, Jesus G, Cary M, Guerreiro JP, et al. Costs and consequences of the Portuguese needle-exchange program in community pharmacies. Canadian Pharmacists Journal. 2020;153(3):170-8. doi: 10.1177/1715163520915744.

5769. Borges RMR. Estudos de Estabilidade e Aceitabilidade de Gomas Orais de Prednisolona: Experiência Profissionalizante na Vertente de Farmácia Hospitalar e Investigação [M.Sc.]. Portugal: Universidade da Beira Interior (Portugal); 2019.

5770. Borget I, Laville I, Paci A, Michiels S, Mercier L, Desmaris R-P, et al. Application of an acceptance sampling plan for post-production quality control of chemotherapeutic batches in an hospital pharmacy. European Journal of Pharmaceutics and Biopharmaceutics. 2006;64(1):92-8. doi: https://doi.org/10.1016/j.ejpb.2006.04.002.

5771. Borgh M, Van Eijk M, Giesen A, Van Kessel J. Test, compare, choose. Data systems for feedback on prescriptions. Pharmaceutisch Weekblad. 2003;138(26):910-5.

5772. Borgonje PE, Dunnewind T, Bosma L, Tokromo-Evegaars M, Meulenhoff PCW, Breukels O. Testing of parenteral drug products for visible particles: Comparison of the Ph. Eur. method with an alternative method using polarised light. European Journal of Hospital Pharmacy. 2023:ejhpharm-2022-003633. doi: https://dx.doi.org/10.1136/ejhpharm-2022-003633. PubMed PMID: 2023870919.

5773. Borgsteede SD, Rhodius CA, De Smet P, Pasman HRW, Onwuteaka-Philipsen BD, Rurup ML. The use of opioids at the end of life: knowledge level of pharmacists and cooperation with physicians. European Journal of Clinical Pharmacology. 2011;67(1):79-89. doi: 10.1007/s00228-010-0901-7. PubMed PMID: WOS:000286881600011.

5774. Borja Lopetegi A. Measuring and modeling performance in the NHS pharmacy workforce [Ph.D.]. England: University of London, School of Pharmacy (United Kingdom); 2007.

5775. Borja VA, Galbraith K. Medication-related issues associated with the documentation and administration of long-acting injectable antipsychotics. International Journal of Clinical Pharmacy. 2019;41(3):623-9. doi: 10.1007/s11096-019-00814-6. PubMed PMID: 136840333. Language: English. Entry Date: 20190810. Revision Date: 20200601. Publication Type: journal article.

5776. Borja-Hart N, Douglas SM, Marino J, Garcia A, Anderson-Worts P. Preparation for a Medical Mission to Jamaica - Pharmacy's Perspective. West Indian Medical Journal. 2011;60(2):199-202. PubMed PMID: WOS:000295239700018.

5777. Borja-Hart N, Douglas SM, Marino J, Garcia A, Anderson-Worts P. Preparation for a medical mission

to Jamaica - Pharmacy's perspective. West Indian Medical Journal. 2012;60(2):199-202.

5778. Borja-Hart NL, Leachman BG. Drug Information Resources Used by Chain Community Pharmacists in Tennessee. Journal of Pharmacy Technology. 2016;32(5):185-90. doi: 10.1177/8755122516653611. PubMed PMID: WOS:000382944200002.

5779. Borja-Hart NL, Maniscalco-Feichtl M. Development of a comprehensive community pharmacy patient intake form compliant with OBRA'90 requirements. Journal of Pharmacy Practice. 2011;24(5):480-4. PubMed PMID: 21844217.

5780. Bork K, Horstkorte R, Weidemann W. Increasing the sialylation of therapeutic glycoproteins: The potential of the sialic acid biosynthetic pathway. Journal of Pharmaceutical Sciences. 2009;98(10):3499-508. doi: https://doi.org/10.1002/jps.21684.

5781. Borkar DS, Tham VM, Shen E, Parker JV, Uchida A, Vinoya AC, et al. Association Between Statin Use and Uveitis: Results From the Pacific Ocular Inflammation Study. American Journal of Ophthalmology. 2015;159(4):707-13.e2. doi: https://doi.org/10.1016/j.ajo.2015.01.009.

5782. Born H, Persky M, Kraus DH, Peng R, Amin MR, Branski RC. Electronic Cigarettes: A Primer for Clinicians. Otolaryngology-Head and Neck Surgery. 2015;153(1):5-14. doi: 10.1177/0194599815585752. PubMed PMID: WOS:000357297000002.

5783. Born M, Carlson M, Ferkul K. Impact of an inpatient medication therapy management referral program in an integrated care delivery system. Journal of the American Pharmacists Association. 2014;54(2):e141. PubMed PMID: 71563245.

5784. Borne RT, O'Donnell C, Turakhia MP, Varosy PD, Jackevicius CA, Marzec LN, et al. Adherence and outcomes to direct oral anticoagulants among patients with atrial fibrillation: findings from the veterans health administration. Bmc Cardiovascular Disorders. 2017;17. doi: 10.1186/s12872-017-0671-6. PubMed PMID: WOS:000409138600002.

5785. Bornstein BH, Marcus D, Cassidy W. Choosing a doctor: an exploratory study of factors influencing patients' choice of a primary care doctor. Journal of Evaluation in Clinical Practice. 2000;6(3):255-62. doi: 10.1046/j.1365-2753.2000.00256.x. PubMed PMID: WOS:000090133300004.

5786. Bornstein C. CSHP 2015 - Can you hit the target? Canadian Journal of Hospital Pharmacy. 2007;60(4):283-4.

5787. Borralho J, Gregório J. “Each one has their own role”: Exploratory study on consumers’ perceptions about nutritionists services provided in community pharmacies. Research in Social and Administrative Pharmacy. 2021;17(2):475-9. doi: https://doi.org/10.1016/j.sapharm.2020.04.008.

5788. Borras-Blasco J, Herráiz ER, Sánchez PL, Romero-Jimenez R, Navarro-Ruiz A, Sola NR. Consensus of Spanish Society of Hospital Pharmacy on optimal medication therapy management of atopic dermatitis. Farmacia Hospitalaria. 2023;47(4):148-54. doi: https://doi.org/10.1016/j.farma.2023.03.007.

5789. Borras-Blasco J, Ramírez Herráiz E, López Sánchez P, Romero-Jimenez R, Navarro-Ruiz A, Rudi Sola N. [Artículo traducido] Consenso de la Sociedad Española de Farmacia Hospitalaria sobre el manejo fármacoterapéutico óptimo de la dermatitis atópica. Farmacia Hospitalaria. 2023;47(4):T148-T54. doi: https://doi.org/10.1016/j.farma.2023.05.007.

5790. Borrego ME, Rhyne R, Clark Hansbarger L, Geller Z, Edwards P, Griffin B, et al. Pharmacy student participation in rural interdisciplinary education using problem based learning (PBL) case tutorials. American

Journal of Pharmaceutical Education. 2000;64(4):355-63.

5791. Borrego ME, Rhyne R, Hansbarger LC, Geller Z, Edwards P, Griffin B, et al. Pharmacy student participation in rural interdisciplinary education using problem based learning (PBL) case tutorials. American Journal of Pharmaceutical Education. 2000;64(4):355-63. PubMed PMID: WOS:000166349800003.

5792. Borrell JR, Cassó C. Pharmacies. Encyclopedia of Health Economics2014. p. 49-55.

5793. Borrelli EP. Prescription Controlled Substance Utilization: Evaluating Benzodiazepine Dose Intensity, Development and Application of a Measurement Framework, and Use of Tramadol Among Patients with Higher Risk [Ph.D.]. United States -- Rhode Island: University of Rhode Island; 2020.

5794. Borro M, Salerno G, Gentile G, Simmaco M. Opinion paper on the systematic application of integrated bioinformatic tools to actuate routine precision medicine in poly-treated patients. Clinical Chemistry & Laboratory Medicine. 2023;61(4):662-5. doi: 10.1515/cclm-2022-1293. PubMed PMID: 162060387.

5795. Borsch J. ARMIN'S legacy. Deutsche Apotheker Zeitung. 2023;2023(17). PubMed PMID: 2029032740.

5796. Borschuk AP, Everhart R, Eakin MN, Riekert KA. Impact of disclosure to colleagues, friends and romantic partners on older adolescent and adult psychosocial outcome. Pediatric pulmonology. 2015;50:190-1. doi: 10.1002/ppul.23296. PubMed PMID: CN-01126911.

5797. Borschuk AP, Everhart RS, Eakin MN, Rand-Giovannetti D, Borrelli B, Riekert KA. Disease disclosure in individuals with cystic fibrosis: association with psychosocial and health outcomes. Journal of cystic fibrosis. 2016;15(5):696-702. doi: 10.1016/j.jcf.2016.02.011. PubMed PMID: CN-01305603.

5798. Borson S, Scanlan JM, Lessig M, DeMers S. Comorbidity in Aging and Dementia: Scales Differ, and the Difference Matters. American Journal of Geriatric Psychiatry. 2010;18(11):999-1006. doi: 10.1097/JGP.0b013e3181d695af. PubMed PMID: WOS:000283326600006.

5799. Borthwick M, Barton G, Bourne RS, McKenzie C. Critical care pharmacy workforce: UK deployment and characteristics in 2015. International Journal of Pharmacy Practice. 2018;26(4):325-33. PubMed PMID: 29024199.

5800. Bortolussi-Courval E, Podymow T, Trinh E, Moryousef J, Hanula R, Huon JF, et al. Electronic Decision Support for Deprescribing in Patients on Hemodialysis: Clinical Research Protocol for a Prospective, Controlled, Quality Improvement Study. Canadian Journal of Kidney Health and Disease. 2023;10. doi: [https://dx.doi.org/10.1177/20543581231165712.](https://dx.doi.org/10.1177/20543581231165712) PubMed PMID: 2024009796.

5801. Borulkar RP, Dhande PP. Medication Reconciliation: A beneficial tool in patient safety.

5802. Borup C, Vinter-Jensen L, Jørgensen SPG, Wildt S, Graff J, Gregersen T, et al. Efficacy and safety of colesevelam for the treatment of bile acid diarrhoea: a double-blind, randomised, placebo-controlled, phase 4 clinical trial. The Lancet Gastroenterology & Hepatology. 2023;8(4):321-31. doi: [https://doi.org/10.1016/S2468-1253(22)00401-0.](https://doi.org/10.1016/S2468-1253(22)00401-0)

5803. Borycki EM, Farghali A, Macdonald S. Pharmacist's perception of the impact of electronic prescribing on medication errors and productivity in community pharmacies. Knowledge Management & E-Learning-an International Journal. 2021;13(4):536-58. doi: 10.34105/j.kmel.2021.13.029. PubMed PMID: WOS:000770291900011.

5804. Borycki EM, Kushniruk AW. Where do technology-induced errors come from? Towards a model for

conceptualizing and diagnosing errors caused by technology. Human, social, and organizational aspects of health information systems: IGI global; 2008. p. 148-66.

5805. Borycki EM, Kushniruk AW. Chapter 2 - Patient Safety and Health Information Technology: Platforms That Led to the Emergence of a Two-Sided Market. In: Vimarlund V, editor. E-Health Two-Sided Markets: Academic Press; 2017. p. 19-31.

5806. Borzutzky C, Rocha JN, Johnson EM, Jin E, Nielsen DA, Iverson E. 89. LARC-ABACUS: Long-Acting Reversible Contraception Attitudes and Beliefs Among Youth with Chronic Underlying Conditions Study. Journal of Pediatric & Adolescent Gynecology. 2023;36(2):212-. doi: 10.1016/j.jpag.2023.01.232. PubMed PMID: 162391465.

5807. Bos E. Outpatient pharmacist calls with patient pharmacist about medication changes: Permanent contact moment about baxter at discharge from the hospital. [Dutch]. Pharmaceutisch Weekblad. 2018;153(23):10-1. PubMed PMID: 622891547.

5808. Bos E, Jansen F. Hospital and primary care are jointly responsible for medication transfer. South Kennemerland and Haarlemmermeer: Pharmacist manages medication file. Pharmaceutisch Weekblad. 2023;158(14):10-3. PubMed PMID: 2027725817.

5809. Bos J, Van Den Bemt PM, Kievit W, Pot JL, Nagtegaal JE, Wieringa A, et al. A multifaceted intervention to reduce drug-related complicationsinsurgical patients (the P-review study). Clinical pharmacology and therapeutics Conference: 118th annual meeting of the american society for clinical pharmacology and therapeutics, ASCPT 2017 United states Conference start: 20170315 Conference end: 20170317. 2017;101:S23. doi: 10.1002/cpt.570. PubMed PMID: CN-01303476.

5810. Bos JM, van den Bemt P, Kievit W, Pot JLW, Nagtegaal JE, Wieringa A, et al. A multifaceted intervention to reduce drug-related complications in surgical patients. British journal of clinical pharmacology. 2016;(no pagination). doi: 10.1111/bcp.13141. PubMed PMID: CN-01291303.

5811. Bos JM, van den Bemt PM, Kievit W, Pot JL, Nagtegaal JE, Wieringa A, et al. A multifaceted intervention to reduce drug-related complications in surgical patients. British Journal of Clinical Pharmacology. 2017;83(3):664-77. PubMed PMID: 27670813.

5812. Bosch-Lenders D, Jansen J, Stoffers H, Winkens B, Aretz K, Twellaar M, et al. The effect of a comprehensive, interdisciplinary medication review on quality of life and medication use in community dwelling older people with polypharmacy. Journal of clinical medicine. 2021;10(4):1-13. doi: 10.3390/jcm10040600. PubMed PMID: CN-02245474.

5813. Boscheck R. Pharmacy Benefit Managers: Fixing Healthcare Market Failures or Straining Regulatory Logics!? World Competition. 2017;40(3):459-69. PubMed PMID: WOS:000426158700005.

5814. Bose R. Knowledge management-enabled health care management systems: capabilities, infrastructure, and decision-support. Expert systems with Applications. 2003;24(1):59-71.

5815. Bosh TK. Prioritization of hospital patients for pharmacist intervention using cluster analysis [Ph.D.]. United States -- Ohio: The Ohio State University; 1996.

5816. Boshart T, Rottar C, Grin S, Bunston G. DERS - What is it Good For? Vascular Access. 2016;10(2):8-. PubMed PMID: 126303240. Language: English. Entry Date: 20171219. Revision Date: 20180309. Publication Type: Article.

5817. Bosko A. General Electric PETtrace cyclotron as a neutron source for boron neutron capture therapy

[Ph.D.]. United States -- Texas: Texas A&M University; 2005.

5818. Boskovic DH, Liang ST, Parab P, Wiggins E, Liberman JN. Real-World Evidence of Aripiprazole Tablets with Sensor: Treatment Patterns and Impacts on Psychiatric Healthcare Resource Utilization. Clinicoeconomics and Outcomes Research. 2023;15:487-98. doi: 10.2147/ceor.S402357. PubMed PMID: WOS:001020718200001.

5819. Boskovic J, Leppee M, Culig J, Fuckar S, Mandic-Zovko N, Jakovljevic M, et al. COMPARISON OF TWO DIFFERENT METHODS (PATIENT QUESTIONNAIRE AND MEDICATION POSSESSION RATIO - MPR) FOR MEASURING THE CHRONIC PATIENT'S BEHAVIOR. Psychiatria Danubina. 2014;26:498-508. PubMed PMID: WOS:000209748400014.

5820. Boskovic J, Leppée M, Culig J, Fuckar S, Mandic-Zovko N, Ratz A, et al. Comparison of two different methods (patient questionnaire and medication possession ratio - MPR) for measuring the chronic patient's behavior. Psychiatria Danubina. 2014;26:498-508.

5821. Boskovic J, Mestrovic A, Leppée M, Bago M, Sostar Z, Naletilic D. Pharmacist competences and impact of pharmacist intervention on medication adherence: An observational study. Psychiatria Danubina. 2016;28(4):420-7.

5822. Bosma BE, Meuwese E, Siok Swan T, van Bommel J, Jan Melief PHG, Hunfeld NGM, et al. The effect of the TIM program (Transfer ICU Medication reconciliation) on medication transfer errors in two Dutch intensive care units: design of a prospective 8-month observational study with a before and after period. BMC Health Services Research. 2017;17:1-10. doi: 10.1186/s12913-017-2065-y. PubMed PMID: 121220293. Language: English. Entry Date: 20180728. Revision Date: 20190711. Publication Type: journal article.

5823. Bosma BE, Meuwese E, Tan SS, van Bommel J, Melief PH, Hunfeld NG, et al. The effect of the TIM program (Transfer ICU Medication reconciliation) on medication transfer errors in two Dutch intensive care units: design of a prospective 8-month observational study with a before and after period. BMC Health Services Research. 2017;17(1):124. PubMed PMID: 28183302.

5824. Bosma BE, van den Bemt P, Melief P, van Bommel J, Tan SS, Hunfeld NGM. Pharmacist interventions during patient rounds in two intensive care units: Clinical and financial impact. Netherlands Journal of Medicine. 2018;76(3):115-24. PubMed PMID: 29667584.

5825. Bosma L, Jansman FG, Franken AM, Harting JW, Van den Bemt PM. Evaluation of pharmacist clinical interventions in a Dutch hospital setting. Pharmacy World & Science. 2008;30(1):31-8. PubMed PMID: 105673252. Language: English. Entry Date: 20081024. Revision Date: 20171129. Publication Type: Journal Article.

5826. Bosma L, Jansman FGA, Franken AM, Harting JW, Van den Bemt P. Evaluation of pharmacist clinical interventions in a Dutch hospital setting. Pharmacy World & Science. 2008;30(1):31-8. doi: 10.1007/s11096-007-9136-9. PubMed PMID: WOS:000251147100006.

5827. Bosma LB, Hunfeld NG, Quax RA, Meuwese E, Melief PH, van Bommel J, et al. The effect of a medication reconciliation program in two intensive care units in the Netherlands: a prospective intervention study with a before and after design. Annals of intensive care. 2018;8:1-13.

5828. Bosmans JE, Brook OH, van Hout HP, de Bruijne MC, Nieuwenhuyse H, Bouter LM, et al. Cost effectiveness of a pharmacy-based coaching programme to improve adherence to antidepressants. PharmacoEconomics. 2007;25(1):25-37. doi: 10.2165/00019053-200725010-00004. PubMed PMID: CN-00577458.

5829. Bosmans JE, Brook OH, van Hout HPJ, de Bruijne MC, Nieuwenhuyse H, Bouter LM, et al. Cost effectiveness of a pharmacy-based coaching programme to improve adherence to antidepressants. Pharmacoeconomics. 2007;25(1):25-37. doi: 10.2165/00019053-200725010-00004. PubMed PMID: WOS:000243666800004.

5830. Bosmans JE, Van Der Laan DM, Yang Y, Elders PJM, Boons C, Nijpels G, et al. The cost-effectiveness of an intervention program to enhance adherence to antihypertensive medication in comparison with usual care in community pharmacies. Frontiers in pharmacology. 2019;10(MAR). doi: 10.3389/fphar.2019.00210. PubMed PMID: CN-01956742.

5831. Bosmans JE, Van Der Laan DM, Yang Y, Elders PJM, Boons CCLM, Nijpels G, et al. The cost-effectiveness of an intervention program to enhance adherence to antihypertensive medication in comparison with usual care in community pharmacies. Frontiers in Pharmacology. 2019;10(MAR). doi: 10.3389/fphar.2019.00210.

5832. Bosmans JE, van der Laan DM, Yang YH, Elders PJM, Boons C, Nijpels G, et al. The Cost-Effectiveness of an Intervention Program to Enhance Adherence to Antihypertensive Medication in Comparison With Usual Care in Community Pharmacies. Frontiers in Pharmacology. 2019;10. doi: 10.3389/fphar.2019.00210. PubMed PMID: WOS:000460779200001.

5833. Bosnic-Anticevich S, Bakerly ND, Chrystyn H, Hew M, van der Palen J. Advancing Digital Solutions to Overcome Longstanding Barriers in Asthma and COPD Management. Patient Preference & Adherence. 2023;17:259-72. doi: 10.2147/PPA.S385857. PubMed PMID: 161723344.

5834. Bosnic-Anticevich S, Costa E, Menditto E, Lourenço O, Novellino E, Bialek S, et al. ARIA pharmacy 2018 “Allergic rhinitis care pathways for community pharmacy”: AIRWAYS ICPs initiative (European Innovation Partnership on Active and Healthy Ageing, DG CONNECT and DG Santé) POLLAR (Impact of Air POLLution on Asthma and Rhinitis)GARD Demonstration project. Allergy: European Journal of Allergy and Clinical Immunology. 2019;74(7):1219-36. doi: 10.1111/all.13701.

5835. Bosnic-Anticevich S, Costa E, Menditto E, Lourenco O, Novellino E, Bialek S, et al. ARIA pharmacy 2018 "Allergic rhinitis care pathways for community pharmacy" AIRWAYS ICPs initiative (European Innovation Partnership on Active and Healthy Ageing, DG CONNECT and DG Sante) \nPOLLAR (Impact of Air POLLution on Asthma and Rhinitis)\nGARD Demonstration project. Allergy. 2019;74(7):1219-36. doi: 10.1111/all.13701. PubMed PMID: WOS:000477968800001.

5836. Bosnic-Anticevich S, Williamson M, Cvetkovski B, Mavritsakis S, Travsers-Mason P, Mendrela G, et al. Comparing three different forms of interprofessional education on health professional inhaler technique and maintenance of correct technique. European respiratory journal. 2013;42. PubMed PMID: CN-01099978.

5837. Bosnic-Anticevich SZ. Inhaler device handling: have we really started to address the problem? European Respiratory Journal. 2017;49(2). doi: 10.1183/13993003.00120-2017. PubMed PMID: WOS:000397858100050.

5838. Bosnic-Anticevich SZ. Continued Innovation in Respiratory Care: The Importance of Inhaler Devices. Tuberculosis and Respiratory Diseases. 2018;81(2):91-8. doi: 10.4046/trd.2017.0119. PubMed PMID: WOS:000429232900001.

5839. Bosnic-Anticevich SZ, Armour CL, SainiB, Krass I, Wettenhall J, Emmerton L, et al. Improving asthma outcomes through community pharmacy intervention. European respiratory journal. 2006;28(Suppl 50):328s 1978. PubMed PMID: CN-00623482.

5840. Bosnic-Anticevich SZ, Sinha H, So S, Reddel HK. Metered-dose inhaler technique: the effect of two

educational interventions delivered in community pharmacy over time. Journal of asthma. 2010;47(3):251-6. doi: 10.3109/02770900903580843. PubMed PMID: CN-00743219.

5841. Bosnic-Anticevich SZ, So S, Armour CL, Reddel HK. Apilot study of the effect of two educational interventions on metered-dose inhaler technique in community pharmacy. American journal of respiratory and critical care medicine. 2002;165(Suppl 8):A112. PubMed PMID: CN-00382376.

5842. Bosnic-Anticevich SZ, So S, Sinha H, Armour CL, Reddel HK. MDI technique: effect of education interventions delivered in community pharmacy over time. American thoracic society 99th international conference. 2003:D035 Poster C65. PubMed PMID: CN-00444496.

5843. Bosnic-Anticevich SZ, Stuart M, Mackson J, Cvetkovski B, Sainsbury E, Armour C, et al. Development and evaluation of an innovative model of inter-professional education focused on asthma medication use. BMC medical education. 2014;14:72. doi: 10.1186/1472-6920-14-72. PubMed PMID: CN-01073334.

5844. Bossaert S, Corvaisier S, Livrozet A, Auxenfans C, Billard M, Martin P, et al. [Clinical trials and outpatient dispensation: the pharmaceutical counseling place]. Journal de Pharmacie de Belgique. 2003;58(2):44-50. PubMed PMID: 12879719.

5845. Bosse N, Machado M, Mistry A. Efficacy of an over-the-counter intervention follow-up program in community pharmacies. Journal of the American Pharmacists Association. 2012;52(4):535-40. doi: https://doi.org/10.1331/JAPhA.2012.10093.

5846. Bosson L, Veyer F, Bardet JD, Vermorel C, Foote A, Allenet B, et al. Evidence-Based Pharmaceutical Care in Community Pharmacies: A Survey of 595 French Pharmacists. Pharmacy. 2023;11(5). doi: 10.3390/pharmacy11050161. PubMed PMID: WOS:001119457600001.

5847. Bostock CV, Soiza RL, Mangoni AA. Association between prescribing of antimuscarinic drugs and antimuscarinic adverse effects in older people. Expert Review of Clinical Pharmacology. 2010;3(4):441-52.

5848. Bostwick JR. Optimizing care for patients with depression in the community pharmacy setting. US Pharmacist. 2008;33(11).

5849. Boswell D, Conradie S, Keshave B, Khan T, Mngadi S, Tebeile L, et al. Medicine Runners: A community-initiated solution to chronic medicine access in underserved communities. SA Pharmaceutical Journal. 2018;85(4):64-7.

5850. Bosworth HB, Granger BB, Mendys P, Brindis R, Burkholder R, Czajkowski SM, et al. Medication adherence: a call for action. American heart journal. 2011;162(3):412-24.

5851. Bosworth HB, Granger BB, Mendys P, Brindis R, Burkholder R, Czajkowski SM, et al. Medication adherence: A call for action. American Heart Journal. 2011;162(3):412-24. doi: 10.1016/j.ahj.2011.06.007.

5852. Bosworth HB, Olsen MK, McCant F, Stechuchak KM, Danus S, Crowley MJ, et al. Telemedicine cardiovascular risk reduction in veterans: the CITIES trial. American heart journal. 2018;199:122-9. doi: 10.1016/j.ahj.2018.02.002. PubMed PMID: CN-01613880.

5853. Botelho MSL. Screening da Atividade Antimicrobiana de Extratos de Plantas: Experiência Profissionalizante na Vertente de Investigação, Farmácia Comunitária e Hospitalar [M.Sc.]. Portugal: Universidade da Beira Interior (Portugal); 2015.

5854. Botelho SF, Neiva Pantuzza LL, Marinho CP, Moreira Reis AM. Prognostic prediction models and

clinical tools based on consensus to support patient prioritization for clinical pharmacy services in hospitals: A scoping review. Research in Social and Administrative Pharmacy. 2021;17(4):653-63. doi: https://doi.org/10.1016/j.sapharm.2020.08.002.

5855. Botermann L, Krueger K, Eickhoff C, Kloft C, Schulz M. Patients' handling of a standardized medication plan: a pilot study and method development. Patient preference & adherence. 2016;10:621-30. PubMed PMID: 27175062.

5856. Botha F-M. The influence of the rural survivalist culture on corporate image [Ph.D.]. South Africa: University of Pretoria (South Africa); 2007.

5857. Botha MJ. In Silico and in Vitro Investigation into the Next Generation of New Psychoactive Substances [Ph.D.]. England: University of Hertfordshire (United Kingdom); 2019.

5858. Boto PA. Risk adjustment in a European primary care context: Assessing the properties of Adjusted Clinical Groups (ACG) for predictive modeling, for special populations and using ATC pharmacy codes [Ph.D.]. United States -- Maryland: The Johns Hopkins University; 2011.

5859. Botomino A, Bruppacher R, Krähenbühl S, Hersberger KE. Change of body weight and lifestyle of persons at risk for diabetes after screening and counselling in pharmacies. Pharmacy World and Science. 2008;30(3):222-6. doi: 10.1007/s11096-007-9174-3.

5860. Bots SH, Siegersma KR, Onland-Moret NC, Asselbergs FW, Somsen GA, Tulevski, II, et al. Routine clinical care data from thirteen cardiac outpatient clinics: design of the Cardiology Centers of the Netherlands (CCN) database. Bmc Cardiovascular Disorders. 2021;21(1). doi: 10.1186/s12872-021-02020-7. PubMed PMID: WOS:000662961700001.

5861. Bou-Saba A, Kassak KM, Salameh P. Adherence to Good Pharmacy Practices by Community Pharmacies in Lebanon and Opportunities for Quality Improvement: It is Time to Act. Integrated Pharmacy Research and Practice. 2023:25-35.

5862. Bou-Saba AW, Kassak KM, Salameh PR. The current trends and challenges towards good community pharmacy practice and the way forward. Exploratory Research in Clinical and Social Pharmacy. 2022;6:100152. doi: https://doi.org/10.1016/j.rcsop.2022.100152.

5863. Bou-Saba AW, Kassak KM, Salameh PR. Public views of community pharmacy services during the COVID-19 pandemic: a national survey. Journal of Pharmaceutical Policy and Practice. 2022;15(1). doi: 10.1186/s40545-022-00474-4.

5864. Boua ATK, Gbaka RK, Doffou E, Danho PA. Analyse des divergences non intentionnelles au cours de la conciliation médicamenteuse dans un service de pneumo-phtisiologie à Abidjan (Côte d'Ivoire). Journal de Pharmacie Clinique. 2023;42(3):119-24. doi: 10.1684/jpc.2023.0525. PubMed PMID: 173049319.

5865. Bouami H, Millot P. Healthcare Delivery System Security: the orchestration of automated and organizational solutions. IFAC-PapersOnLine. 2019;52(19):127-32. doi: https://doi.org/10.1016/j.ifacol.2019.12.123.

5866. Bouças E, Martins TR, Futuro DO, de Castilho SR. Accreditation in the field of hospital pharmacy services: A qualitative approach of its effects. Physis. 2018;28(3). doi: 10.1590/S0103-73312018280317.

5867. Bouchahda M, Macarulla T, Spano JP, Bachet JB, Lledo G, Andre T, et al. Cetuximab efficacy and safety in a retrospective cohort of elderly patients with heavily pretreated metastatic colorectal cancer. Critical Reviews in Oncology/Hematology. 2008;67(3):255-62. doi: https://doi.org/10.1016/

j.critrevonc.2008.02.003.

5868. Bouchand F, Leplay C, Guimaraes R, Fontenay S, Fellous L, Dinh A, et al. Impact of a medication reconciliation care bundle at hospital discharge on continuity of care: a randomised controlled trial. International journal of clinical practice. 2021;75(8):e14282. doi: 10.1111/ijcp.14282. PubMed PMID: CN-02275865.

5869. Bouchand F, Thomas A, Zerhouni L, Dauphin A, Conort O. Interventions pharmaceutiques avant et après informatisation de la prescription dans un service de médecine interne. La Presse Médicale. 2007;36(3, Part 1):410-8. doi: https://doi.org/10.1016/j.lpm.2006.07.011.

5870. Bouchand F, Thomas A, Zerhouni L, Dauphin A, Conort O. [Pharmacists' interventions before and after prescription computerization in an internal medicine department]. Presse Medicale. 2007;36(3 Pt 1):410-8. PubMed PMID: 17321362.

5871. Bouchard AP. Development of New Centrifugal Microfluidic Functions [Ph.D.]. Canada -- Quebec, CA: McGill University (Canada); 2016.

5872. Boucher HW, Wilcox M, Talbot GH, Puttagunta S, Das AF, Dunne MW. Once-weekly dalbavancin versus daily conventional therapy for skin infection. New England Journal of Medicine. 2014;370(23):2169-79.

5873. Boucher MC, Desroches G, Garcia-Salinas R, Kherani A, Maberley D, Olivier S, et al. Teleophthalmology screening for diabetic retinopathy through mobile imaging units within Canada. Canadian Journal of Ophthalmology. 2008;43(6):658-68. doi: 10.3129/I08-120.

5874. Bouchet-Benezech B, Champanet B, Rouzaud P. Smoking cessation at the pharmacy: feasibility and benefits based on a French observational study with six-month follow-up. Substance Abuse and Rehabilitation. 2018;9:31-42. doi: 10.2147/sar.S152186. PubMed PMID: WOS:000439632800001.

5875. Boudoukha AH, Bonnaud-Antignac A, Acier D, Chaillou A. Anxiété et stress : impact de la préparation du concours de pharmacie. Journal de Thérapie Comportementale et Cognitive. 2011;21(3):90-6. doi: https://doi.org/10.1016/j.jtcc.2011.07.010.

5876. Boudreau D, Von Korff M, Rutter CM, Saunders K, Ray GT, Sullivan MD, et al. Trends in long-term opioid therapy for chronic non-cancer pain. Pharmacoepidemiology and drug safety. 2009;18(12):1166-75.

5877. Boudreau DM. The association between HMG -CoA inhibitor use and breast cancer risk, and, A validation study of patient interview data and pharmacy records for antihypertensive, statin, and antidepressant medication use [Ph.D.]. United States -- Washington: University of Washington; 2002.

5878. Boudreau DM, Capoccia KL, Sullivan SD, Blough DK, Ellsworth AJ, Clark DL, et al. Collaborative care model to improve outcomes in major depression. Annals of pharmacotherapy. 2002;36(4):585-91. doi: 10.1345/aph.1A259. PubMed PMID: CN-00386115.

5879. Boughen M, Sutton J, Fenn T, Wright D. Defining the Role of the Pharmacy Technician and Identifying Their Future Role in Medicines Optimisation. Pharmacy. 2017;5(3). doi: 10.3390/pharmacy5030040. PubMed PMID: WOS:000411985700007.

5880. Bougouma EC, Soulama I, James ER, Abebe Y, Ouedraogo A, Tiono A, et al. Experience in managing sanaria®PFSPZ vaccine: a randomized, double-blind, placebo controlled phase 1 clinical trial to evaluate the safety and immunogenicity of PFSPZ vaccine in malariaexperienced adults in Burkina Faso. American journal of tropical medicine and hygiene. 2018;99(4):360-. PubMed PMID: CN-01934431.

5881. Bouhaddou O, Warnekar P, Parrish F, Do N, Mandel J, Kilbourne J, et al. Exchange of Computable Patient Data between the Department of Veterans Affairs (VA) and the Department of Defense (DoD): Terminology Mediation Strategy. Journal of the American Medical Informatics Association. 2008;15(2):174-83. doi: https://doi.org/10.1197/jamia.M2498.

5882. Boukhatem BS, Belhadj A-E. In silico anti-inflammatory activity of lavender (Lavandula officinalis) essential oil bioactive compounds: Molecular docking analysis of COX-1 and COX-2, and ADMET prediction. AIMS Allergy & Immunology. 2023;7(2):1-22. doi: 10.3934/Allergy.2023009. PubMed PMID: 167382467.

5883. Boukouvalas EA, El-Den S, Chen TF, Moles R, Saini B, Bell A, et al. Confidence and attitudes of pharmacy students towards suicidal crises: patient simulation using people with a lived experience. Social Psychiatry and Psychiatric Epidemiology. 2018;53(11):1185-95. doi: 10.1007/s00127-018-1582-2. PubMed PMID: WOS:000448474000004.

5884. Boullata JI, Guenter P, Mirtallo JM. A parenteral nutrition use survey with gap analysis. JPEN Journal of Parenteral & Enteral Nutrition. 2013;37(2):212-22. doi: 10.1177/0148607112464781. PubMed PMID: 104312613. Language: English. Entry Date: 20130809. Revision Date: 20200708. Publication Type: Journal Article.

5885. Boullata JI, McDonnell PJ. Preventing medication errors in the intensive care unit. JAMA. 2000;283(10):1287-8; author reply 8-9. PubMed PMID: 10714720.

5886. Boulos MNK, Haywood G. Opportunistic atrial fibrillation screening and detection in "self-service health check-up stations": a brief overview of current technology potential and possibilities. Mhealth. 2021;7(1). doi: 10.21037/mhealth-19-204. PubMed PMID: WOS:000850463900003.

5887. Bound JP. Evaluation of pathways in the development of the environmental risk assessment of pharmaceuticals [Ph.D.]. England: Imperial College London (United Kingdom); 2006.

5888. Bound JP, Voulvoulis N. Household disposal of pharmaceuticals as a pathway for aquatic contamination in the United Kingdom. Environmental health perspectives. 2005;113(12):1705-11.

5889. Bounthavong M. Program Evaluation of Academic Detailing on Naloxone Prescriptions Prescribed at the U.S. Department of Veterans Affairs [Ph.D.]. United States -- Washington: University of Washington; 2018.

5890. Bounthavong M, Shayegani R, Manning JM, Marin J, Spoutz P, Hoffman JD, et al. Comparison of virtual to in-person academic detailing on naloxone prescribing rates at three U.S. Veterans Health Administration regional networks. International Journal of Medical Informatics. 2022;161:104712. doi: https://doi.org/10.1016/j.ijmedinf.2022.104712.

5891. Bounthavong M, Suh K, Christopher MLD, Veenstra DL, Basu A, Devine EB. Providers' perceptions on barriers and facilitators to prescribing naloxone for patients at risk for opioid overdose after implementation of a national academic detailing program: A qualitative assessment. Research in Social and Administrative Pharmacy. 2020;16(8):1033-40. doi: https://doi.org/10.1016/j.sapharm.2019.10.015.

5892. Boura F, Al-Tabakha M, Hassan N, Darwich M. Critical appraisal of simulated patient methodology to assess the practice of community pharmacist in the Middle East and North Africa region: A systematic review. Pharmacy Practice. 2022;20(3). doi: 10.18549/PharmPract.2022.3.2701.

5893. Boura F, Al-Tabakha MM, Hassan N, Darwich M. Community pharmacists’ response to complaints of gastroesophageal reflux: A simulated patient study in the Northern United Arab Emirates. PLoS ONE.

2023;18(1 January). doi: 10.1371/journal.pone.0279922.

5894. Boura F, Al-Tabakha MM, Hassan N, Darwich M. Community pharmacists’ response to complaints of gastroesophageal reflux: A simulated patient study in the Northern United Arab Emirates. PLoS ONE. 2023;18(1 January). doi: 10.1371/journal.pone.0279922.

5895. Bouras A, Masmoudi M, Saadani NE, Bahroun Z, Abdeljaouad MA. Multi-stage appointment scheduling for outpatient chemotherapy unit: a case study. Rairo-Operations Research. 2021;55(2):589-610. doi: 10.1051/ro/2021025. PubMed PMID: WOS:000638300300002.

5896. Bouras A, Masmoudi M, Saadani NE, Bahroun Z, Ieee, editors. A three-stage Appointment Scheduling for an Outpatient Chemotherapy Unit using integer programming. 4th International Conference on Control, Decision and Information Technologies (CoDIT); 2017 Apr 05-07; Barcelona, SPAIN2017.

5897. Bouras A, Masmoudi M, Saadani NEH, Bahroun Z, Abdeljaouad MA. Multi-stage appointment scheduling for outpatient chemotherapy unit: A case study. RAIRO - Operations Research. 2021;55(2):589-610. doi: 10.1051/ro/2021025.

5898. Bourbonnais FF, Caswell W. Teaching successful medication administration today: More than just knowing your ‘rights’. Nurse Education in Practice. 2014;14(4):391-5.

5899. Bourcier E, Mille F, Brunie V, Korb-Savoldelli V, Lafortune C, Buyse M, et al. Quality of prescribing in community-dwelling elderly patients in France: an observational study in community pharmacies. International Journal of Clinical Pharmacy. 2017;39(6):1220-7. doi: 10.1007/s11096-017-0531-6. PubMed PMID: 126307424. Language: English. Entry Date: 20180706. Revision Date: 20181203. Publication Type: journal article.

5900. Bourdet SV, Kelley M, Rublein J, Williams DM. Effect of a pharmacist-managed program of pneumococcal and influenza immunization on vaccination rates among adult inpatients. American journal of health-system pharmacy. 2003;60(17):1767-71. PubMed PMID: CN-00508450.

5901. Bourdin A, Schluep M, Bugnon O, Berger J. Promoting transitions of care, safety, and medication adherence for patients taking fingolimod in community pharmacies. American Journal of Health-System Pharmacy. 2019;76(15):1150-7. PubMed PMID: 31201774.

5902. Bourgeois AL, Thole A, Salamon T, Calvet P, Cantagrel A, Civade E, et al. Prescription of rituximab in Rheumatology: audit of practices. International journal of clinical pharmacy. 2013;35(6):1294-5. doi: 10.1007/s11096-013-9886-5. PubMed PMID: CN-01063451.

5903. Bourgeois HC, Proteau RC, Vielma CV, Hartung DM, Irwin AN. Evaluation of an interdisciplinary controlled substance review committee on opioid prescribing in a community health center. Pain Medicine (United States). 2020;21(9):1840-6. doi: 10.1093/PM/PNAA075.

5904. Bourgeois K, Eggleston W, Schwier N, Adams J. Characterizing colchicine toxicity due to medication errors reported to two regional poison centers over the past decade. Clinical Toxicology. 2023;61(Supplement 2):107. doi: https://dx.doi.org/10.1080/15563650.2023.2233835. PubMed PMID: 642223805.

5905. Bourget S, Allenet B, Bedouch P, Bosson LJ, Calop J. [Physicians' expectations towards pharmacy services on ward]. Journal de Pharmacie de Belgique. 2007;62(4):130-4. PubMed PMID: 18269141.

5906. Bourhis M, Tortolano L, Lalioui S, Chardonnet F, Dufour C, Gaudin A, et al. Évaluation et optimisation des pratiques de perfusion en oncologie pédiatrique. Le Pharmacien Hospitalier et Clinicien. 2017;52(4):355-60. doi: https://doi.org/10.1016/j.phclin.2017.05.003.

5907. Bourne L. Approaches to Accountability in the Medical Laboratory Sector in Ontario [M.H.S.]. Canada -- Ontario, CA: University of Ontario Institute of Technology (Canada); 2013.

5908. Bourne RS, Choo CL. Pharmacist proactive medication recommendations using electronic documentation in a UK general critical care unit. International Journal of Clinical Pharmacy. 2012;34(2):351-7. PubMed PMID: 22354852.

5909. Bourne RS, Choo CL, Dorward BJ. Proactive clinical pharmacist interventions in critical care: effect of unit speciality and other factors. International Journal of Pharmacy Practice. 2014;22(2):146-54. PubMed PMID: 23763333.

5910. Bourne RS, Dorward BJ, Bourne RS, Dorward BJ. Clinical pharmacist interventions on a UK neurosurgical critical care unit: a 2-week service evaluation. International Journal of Clinical Pharmacy. 2011;33(5):755-8. doi: 10.1007/s11096-011-9538-6. PubMed PMID: 104587013. Language: English. Entry Date: 20121019. Revision Date: 20211028. Publication Type: journal article.

5911. Bourne RS, Jeffries M, Phipps DL, Jennings JK, Boxall E, Wilson F, et al. Understanding medication safety involving patient transfer from intensive care to hospital ward: a qualitative sociotechnical factor study. BMJ Open. 2023;13(5):e066757. doi: https://dx.doi.org/10.1136/bmjopen-2022-066757. PubMed PMID: 2024351838.

5912. Bourne RS, Jennings JK, Panagioti M, Hodkinson A, Sutton A, Ashcroft DM. Medication-related interventions to improve medication safety and patient outcomes on transition from adult intensive care settings: a systematic review and meta-analysis. BMJ Quality & Safety. 2022;31(8):609-22. doi: 10.1136/bmjqs-2021-013760. PubMed PMID: 158584955. Language: English. Entry Date: 20220902. Revision Date: 20220902. Publication Type: Article.

5913. Bourne RS, Shulman R, Jennings JK. Reducing medication errors in critical care patients: pharmacist key resources and relationship with medicines optimisation. International Journal of Pharmacy Practice. 2018;26(6):534-40. doi: 10.1111/ijpp.12430. PubMed PMID: 133235613. Language: English. Entry Date: 20181130. Revision Date: 20191202. Publication Type: Article.

5914. Bourne RS, Shulman R, Tomlin M, Borthwick M, Berry W, Mills GH. Reliability of clinical impact grading by healthcare professionals of common prescribing error and optimisation cases in critical care patients. International Journal for Quality in Health Care. 2017;29(2):250-5. PubMed PMID: 28453820.

5915. Bourne RS, Shulman ROB, Tomlin M, Borthwick M, Berry W, Mills GH. Reliability of clinical impact grading by healthcare professionals of common prescribing error and optimisation cases in critical care patients. International Journal for Quality in Health Care. 2017;29(2):250-5. doi: 10.1093/intqhc/mzx003. PubMed PMID: 122906739. Language: English. Entry Date: 20180723. Revision Date: 20220318. Publication Type: journal article.

5916. Bourneau-Martin D, Babin M, Grandvuillemin A, Mullet C, Salvo F, Singier A, et al. Adverse drug reaction related to drug shortage: A retrospective study on the French National Pharmacovigilance Database. British Journal of Clinical Pharmacology. 2023;89(3):1080-8. doi: 10.1111/bcp.15550. PubMed PMID: 161873335.

5917. Bourque G, Leong B, Vega VB, Chen X, Lee YL, Srinivasan KG, et al. Evolution of the mammalian transcription factor binding repertoire via transposable elements. Genome research. 2008;18(11):1752-62.

5918. Bousman CA, Maruf AA, Marques DF, Brown LC, Müller DJ. The emergence, implementation, and future growth of pharmacogenomics in psychiatry: a narrative review. Psychological Medicine. 2023;53(16):7983-93. doi: 10.1017/S0033291723002817. PubMed PMID: 174548499.

5919. Bouso ED. Characterization of legal highs and their pyrolysis products [M.S.]. United States -- Alabama: The University of Alabama at Birmingham; 2013.

5920. Bousquet J, Bourret R, Camuzat T, Augé P, Bringer J, Noguès M, et al. MACVIA-LR (Fighting Chronic Diseases for Active and Healthy Ageing in Languedoc-Roussillon): A Success Story of the European Innovation Partnership on Active and Healthy Ageing. The Journal of frailty & aging. 2016;5(4):233-41.

5921. Boussadi A, Caruba T, Karras A, Berdot S, Degoulet P, Durieux P, et al. Validity of a clinical decision rule-based alert system for drug dose adjustment in patients with renal failure intended to improve pharmacists' analysis of medication orders in hospitals. International Journal of Medical Informatics. 2013;82(10):964-72. doi: https://doi.org/10.1016/j.ijmedinf.2013.06.006.

5922. Boutin K, Nevers W, Gorman SK, Slavik RS, Martinusen DJ, Lo C. Development of intervention-related quality indicators for renal clinical pharmacists using a modified Delphi approach. International Journal of Pharmacy Practice. 2019;27(5):436-42. PubMed PMID: 30209834.

5923. Boutin M. Among other flaws, hypothetical migraine study lacks independent evaluation and patient engagement. Health Affairs. 2012;31(10):2231-5. doi: 10.1377/hlthaff.2012.0764.

5924. Boutron I, Poiraudeau S, Ravaud JF, Baron G, Revel M, Nizard R, et al. Disability in adults with hip and knee arthroplasty: a French national community based survey. Annals of the Rheumatic Diseases. 2003;62(8):748-54. doi: 10.1136/ard.62.8.748. PubMed PMID: WOS:000184154000011.

5925. Bouvy JC, De Bruin ML, Koopmanschap MA. Epidemiology of adverse drug reactions in Europe: a review of recent observational studies. Drug safety. 2015;38:437-53.

5926. Bouvy ML. What is keeping us busy in the pharmacy? International Journal of Pharmacy Practice. 2012;20(4):207-8. doi: 10.1111/j.2042-7174.2012.00229.x.

5927. Bouvy ML, Buurma H, Egberts ACG. Determinants for successful smoking cessation with bupropion in daily practice. Pharmacy World & Science. 2003;25(5):207-11. doi: 10.1023/a:1025892031198. PubMed PMID: WOS:000185515900006.

5928. Bouvy ML, Buurma H, Egberts TCG. Laxative prescribing in relation to opioid use and the influence of pharmacy-based intervention. Journal of Clinical Pharmacy and Therapeutics. 2002;27(2):107-10. doi: 10.1046/j.1365-2710.2002.00385.x.

5929. Bouvy ML, Heerdink ER, Leufkens HG, Hoes AW. Predicting mortality in patients with heart failure: a pragmatic approach. Heart (British Cardiac Society). 2003;89(6):605-9. doi: 10.1136/heart.89.6.605. PubMed PMID: CN-00437456.

5930. Bouvy ML, Heerdink ER, Urquhart J, Grobbee DE, Hoe AW, Leufkens HGM. Effect of a pharmacist-led intervention on diuretic compliance in heart failure patients: a randomized controlled study. Journal of Cardiac Failure. 2003;9(5):404-11. doi: https://doi.org/10.1054/S1071-9164(03)00130-1.

5931. Bouvy ML, Heerdink ER, Urquhart J, Grobbee DE, Hoes AW, Leufkens HG. Effect of a pharmacist-led intervention on diuretic compliance in heart failure patients: a randomized controlled study. Journal of cardiac failure. 2003;9(5):404-11. doi: 10.1054/s1071-9164(03)00130-1. PubMed PMID: CN-00471819.

5932. Bouvy ML, Heerdink ER, Urquhart J, Grobbee DE, Hoes AW, Leufkens HGM. Guidance improves compliance. Pharmacist stimulates proper use of loop diuretics in patient with heart failure. Pharmaceutisch weekblad. 2003;138(41):1432-9. PubMed PMID: CN-00474609.

5933. Bouvy ML, Stuurman-Bieze AGG, Vervloet E, Van Wisse MW, Heerdink ER. Unequal treatment of men en women: Drugs therapy of angira pectoris. Pharmaceutisch Weekblad. 2000;135(21):746-51.

5934. Bouwer A. Nausea and vomiting: An approach for the pharmacist. SA Pharmaceutical Journal. 2011;78(7):28-32.

5935. Bouwman-Boer Y, Fenton-May V, Le Brun P. Practical pharmaceutics. Control Strategy Critical Quality Attributes, Process Parameters and Sources of Variability (Switzerland: Springer International Publishing. 2015:10.1007.

5936. Bouwmeester C. The PACE program: home-based long-term care. Consultant Pharmacist. 2012;27(1):24-30. PubMed PMID: 22231995.

5937. Bouwmeester C, Kraft J, Bungay KM. Optimizing inhaler use by pharmacist-provided education to community-dwelling elderly. Respiratory Medicine. 2015;109(10):1363-8. doi: 10.1016/j.rmed.2015.07.013.

5938. Bouwmeester MC, Laberge N, Bussières JF, Lebel D, Bailey B, Harel F. Program to remove incorrect allergy documentation in pediatrics medical records. American Journal of Health-System Pharmacy. 2001;58(18):1722-7. doi: 10.1093/ajhp/58.18.1722.

5939. Bouzeid M, Clarenne J, Mongaret C, Pluchart H, Chanoine S, Vermorel C, et al. Using national data to describe characteristics and determine acceptance factors of pharmacists' interventions: a six-year longitudinal study. International Journal of Clinical Pharmacy. 2023;45(2):430-41. PubMed PMID: 36566276.

5940. Bowden A, Mullin S, Tak C, Tyler LS, Nickman NA, Moorman K. Effect of a central call center on employee perceptions of safety culture within community pharmacies in an academic health system. American Journal of Health-System Pharmacy. 2019;76(6):360-5. PubMed PMID: 31361840.

5941. Bowden JA, Heckert A, Ulmer CZ, Jones CM, Koelmel JP, Abdullah L, et al. Harmonizing lipidomics: NIST interlaboratory comparison exercise for lipidomics using SRM 1950–Metabolites in Frozen Human Plasma [S]. Journal of lipid research. 2017;58(12):2275-88.

5942. Bowden K. Managing up to maximize Medicare reimbursement for outpatient care. American Journal of Health-System Pharmacy. 2001;58(19):S14-S6. doi: 10.1093/ajhp/58.suppl_1.S14. PubMed PMID: WOS:000171412200005.

5943. Bowden VR, Greenberg CS. Pediatric nursing procedures: Lippincott Williams & Wilkins; 2012.

5944. Bowdle TA, Jelacic S, Webster CS, Merry AF. Take action now to prevent medication errors: lessons from a fatal error involving an automated dispensing cabinet. BJA: The British Journal of Anaesthesia. 2023;130(1):14-6. doi: 10.1016/j.bja.2022.09.017. PubMed PMID: 161060115.

5945. Bowdring MA, Leas EC, Vishwakarma M, Schleicher NC, Prochaska JJ, Henriksen L. Kratom availability in California vape shops. Preventive Medicine Reports. 2023;35. doi: 10.1016/j.pmedr.2023.102380. PubMed PMID: WOS:001072300100001.

5946. Bowen A, Agboatwalla M, Pitz A, Brum J, Plikaytis BD. Bismuth subsalicylate reduces antimicrobial use among adult diarrhea patients in pakistan: a randomized, placebo-controlled, triple-masked clinical trial. American journal of tropical medicine and hygiene. 2016;95(5):376-7. doi: 10.4269/ajtmh.abstract2016. PubMed PMID: CN-01429418.

5947. Bower D, Hachborn F, Huffam P. Clostridium difficile outbreak: A small group of pharmacists makes a

big impact. Canadian Journal of Hospital Pharmacy. 2009;62(2):142-7.

5948. Bowers BL, Heffern C, Billings S. Implementation of a medication therapy management program in a primary care clinic. Journal of the American Pharmacists Association: JAPhA. 2019;59(3):383-9. PubMed PMID: 30954401.

5949. Bowers J, Lyons B, Mould G, Symonds T, editors. Modelling outpatient capacity for a diagnosis and treatment centre. Health Care Management Science; 2005.

5950. Bowers R, Tunney R, Kelly K, Mills B, Trotta K, Wheeless CN, et al. Impact of Standardized Simulated Patients on First-Year Pharmacy Students' Knowledge Retention of Insulin Injection Technique and Counseling Skills. American journal of pharmaceutical education. 2017;81(6):113. doi: 10.5688/ajpe816113. PubMed PMID: CN-01424215.

5951. Bowis J. Who's mind is it anyway? Epidemiologia E Psichiatria Sociale. 2005;14(1):39-43. doi: 10.1017/s1121189x00001913. PubMed PMID: WOS:000232301000007.

5952. Bowker SL, Mitchell CG, Majumdar SR, Toth EL, Johnson JA. Lack of insurance coverage for testing supplies is associated with poorer glycemic control in patients with type 2 diabetes. Canadian Medical Association Journal. 2004;171(1):39-43. doi: 10.1503/cmaj.1031830. PubMed PMID: WOS:000222543500031.

5953. Bowles EJA, Buist DSM, Chubak J, Yu O, Johnson J, Chestnut J, et al. Endocrine therapy initiation from 2001 to 2008 varies by age at breast cancer diagnosis and tumor size. Journal of Oncology Practice. 2012;8(2):113-20. doi: 10.1200/JOP.2011.000417.

5954. Bowman C. Consumer purchasing of medicines in Malta : an insight to how the Maltese population access medicines [Dr.Health]. England: University of Bath (United Kingdom); 2018.

5955. Bowman C, Family H, Agius-Muscat H, Cordina M, Sutton J. Consumer internet purchasing of medicines using a population sample: A mixed methodology approach. Research in Social and Administrative Pharmacy. 2020;16(6):819-27. doi: https://doi.org/10.1016/j.sapharm.2019.09.056.

5956. Bowman C, Holloway M, Scott L, Russell C, Lott S, Amin R. Impact of Pharmacist Involvement on the Utility of a Gram-Negative Blood Culture Identification Panel on Antimicrobial Usage. Journal of Pharmacy Technology. 2021;37(6):304-9. doi: 10.1177/87551225211046627.

5957. Bowman C, McKenna J, Schneider P, Barnes B. Comparison of Medication History Accuracy Between Nurses and Pharmacy Personnel. Journal of Pharmacy Practice. 2019;32(1):62-7. PubMed PMID: 29108459.

5958. Bowman JS. Keep drug misusers out of community pharmacies [12]. Pharmaceutical Journal. 2004;272(7286):184.

5959. Bowman M, Acharya S, editors. Risk assessment of pharmacies & electronic prescriptions. Proceedings of the 2019 IEEE/ACM International Conference on Advances in Social Networks Analysis and Mining, ASONAM 2019; 2019.

5960. Bowman S, Engelman A, Koziol J, Mahoney L, Maxwell C, McKenzie M. The Rhode Island community responds to opioid overdose deaths. Rhode Island medical journal (2013). 2014;97(10):34-7.

5961. Bowman S, Weeks P, Chow E, Huang A, Nathan S, Radovancevic R, et al. Implementation of pharmacist-managed anticoagulation in patients with continuous flow left ventricular assist devices. JACCP journal of the american college of clinical pharmacy. 2019;2(6):623-7. doi: 10.1002/jac5.1081. PubMed

PMID: CN-01966708.

5962. Box MJ, Sullivan EL, Ortwine KN, Parmenter MA, Quigley MM, Aguilar-Higgins LM, et al. Outcomes of rapid identification for gram-positive bacteremia in combination with antibiotic stewardship at a community-based hospital system. Pharmacotherapy. 2015;35(3):269-76. doi: 10.1002/phar.1557.

5963. Boyatzis M, Batty KT. Domiciliary medication reviews by fourth year pharmacy students in Western Australia. International Journal of Pharmacy Practice. 2004;12(2):73-81. doi: 10.1211/0022357023574.

5964. Boyce MR. Use and Limitations of Malaria Rapid Diagnostic Testing in Sub-Saharan Africa: a Systematic Review & Longitudinal Study in Western Kenya [M.S.]. United States -- North Carolina: Duke University; 2017.

5965. Boyce MR, O'Meara WP. Use of malaria RDTs in various health contexts across sub-Saharan Africa: a systematic review. Bmc Public Health. 2017;17. doi: 10.1186/s12889-017-4398-1. PubMed PMID: WOS:000401867000009.

5966. Boyd A, Walroth T, Meadows K, Spera L, Hartman B. The Impact of a Clinical Pharmacist on Medication Management in an Adult Burn Clinic. Journal of Burn Care and Research. 2023;44(Supplement 2):S96. doi: https://dx.doi.org/10.1093/jbcr/irad045.129. PubMed PMID: 641566611.

5967. Boyd AN, Kadi BA, Rothenberger MA, Hartman BC, Spera LJ, Meadows KJ, et al. Evaluation of the Role of a Clinical Pharmacist on Medication Management in an Adult Burn Clinic via a Collaborative Drug Therapy Management Protocol. Journal of burn care & research : official publication of the American Burn Association. 2023;44(5):1023-30. doi: https://dx.doi.org/10.1093/jbcr/irad087. PubMed PMID: 641543725.

5968. Boyd BJ. The effects of community-based pharmaceutical care services on asthma-related health care expenditures, utilization, and quality of life [Ph.D.]. United States -- Texas: The University of Texas at Austin; 1997.

5969. Boyd M, Waring J, Barber N, Mehta R, Chuter A, Avery AJ, et al. Protocol for the New Medicine Service Study: a randomized controlled trial and economic evaluation with qualitative appraisal comparing the effectiveness and cost effectiveness of the New Medicine Service in community pharmacies in England. Trials. 2013;14(1):1-15.

5970. Boyd M, Waring J, Barber N, Mehta R, Chuter A, Avery AJ, et al. Protocol for the New Medicine Service Study: A randomized controlled trial and economic evaluation with qualitative appraisal comparing the effectiveness and cost effectiveness of the New Medicine Service in community pharmacies in England. Trials. 2013;14(1). doi: 10.1186/1745-6215-14-411.

5971. Boyd M, Waring J, Barber N, Mehta R, Chuter A, Avery AJ, et al. Protocol for the New Medicine Service Study: a randomized controlled trial and economic evaluation with qualitative appraisal comparing the effectiveness and cost effectiveness of the New Medicine Service in community pharmacies in England. Trials. 2013;14:411. doi: 10.1186/1745-6215-14-411. PubMed PMID: CN-00883223.

5972. Boyd MJ, Elliott RA, Barber N, Mehta R, Waring J, Chuter A, et al. The impact of the New Medicines Service (NMS) in England on patients adherence to their medicines. International journal of pharmacy practice. 2014;22:66. doi: 10.1111/ijpp.12146. PubMed PMID: CN-01057098.

5973. Boyd ST, Boyd LC, Zillich AJ. Medication therapy management survey of the prescription drug plans. Journal of the American Pharmacists Association. 2006;46(6):692-9. doi: 10.1331/1544-3191.46.6.692.Boyd.

5974. Boyd T, Torrey K, Giuliano M, Youssef R. A federally qualified health center pharmacist embedded

medication reconciliation process improves patient safety at a critical access hospital in new hampshire. Research in Social and Administrative Pharmacy. 2018;14(5):e12. doi: https://doi.org/10.1016/j.sapharm.2018.03.047.

5975. Boyden KJD. Generation Rx: A study of the changeover to the entry-level PharmD degree in pharmacy education [Ed.D.]. United States -- Pennsylvania: University of Pennsylvania; 2006.

5976. Boyé F, Sallerin B, Ah Kang F, Arnaud A, Kantambadouno JB, Amar J, et al. Place du pharmacien clinicien dans la prise en charge thérapeutique du patient hypertendu. Annales de Cardiologie et d'Angéiologie. 2015;64(3):216-21. doi: https://doi.org/10.1016/j.ancard.2015.04.003.

5977. Boyé F, Sallerin B, Amar J, Chamontin B, Bouhanick B. Conciliation médicamenteuse à la sortie d’hospitalisation du patient hypertendu : analyse de l’ordonnance de sortie. Annales de Cardiologie et d'Angéiologie. 2016;65(3):229-35. doi: https://doi.org/10.1016/j.ancard.2016.04.007.

5978. Boye F, Sallerin B, Amar J, Chamontin B, Bouhanick B. [Medication reconciliation at hospital discharge focusing on patient with hypertension: Analysis of the output prescription]. Annales de Cardiologie et d Angeiologie. 2016;65(3):229-35. PubMed PMID: 27180565.

5979. Boyer EW. Management of opioid analgesic overdose. New England Journal of Medicine. 2012;367(2):146-55.

5980. Boyer EW, Babu KM, Macalino GE, Compton W. Self-treatment of opioid withdrawal with a dietary supplement, Kratom. American Journal on Addictions. 2007;16(5). doi: 10.1080/10550490701525368. PubMed PMID: WOS:000250333900004.

5981. Boyina SY, Stokes T, Renall A, Braund R. Clinical pharmacist facilitators in primary care: A descriptive study of their roles and services provided in general practices of southern New Zealand. Journal of Primary Health Care. 2020;12(1):88-95. doi: 10.1071/HC19073.

5982. Boyko JA, Blackburn DF. Medication adherence does not appear to be a clinical priority among community pharmacists. Canadian pharmacists journal. 2011;144(5):216-9. doi: 10.3821/1913-701X-144.5.216. PubMed PMID: CN-01720500.

5983. Boyko Jr WL, Yurkowski PJ, Ivey MF, Armitstead JA, Roberts BL. Pharmacist influence on economic and morbidity outcomes in a tertiary care teaching hospital. American journal of health-system pharmacy. 1997;54(14):1591-5. PubMed PMID: CN-00392644.

5984. Boylan JJ, Gradzka MI. Long-term results of endoscopic balloon dilatation for gastric outlet obstruction. Digestive Diseases and Sciences. 1999;44(9):1883-6. doi: 10.1023/a:1018807125952. PubMed PMID: WOS:000082681000025.

5985. Boylan PM, Santibañez M. Upper Gastrointestinal Bleeding in a Male Patient Taking Megestrol Acetate and Rivaroxaban: A Case Report. The Senior care pharmacist. 2019;34(8):501-9.

5986. Boylan PM, Santibanez M, Lounsbury N, Eltaki SM. Outcomes of a college-led community-based influenza vaccine program for underserved New York City communities. Journal of the American Pharmacists Association. 2021;61(4):E324-E31. doi: 10.1016/j.japh.2021.03.003. PubMed PMID: WOS:000670385400026.

5987. Boyle A, Marra F, Fox R, Morris J, Fleming C, Reilly E, et al. Partial directly observed therapy with ombitasvir/paritaprevir based regimens allows for successful treatment of patients on daily supervised methadone. Journal of hepatology Conference: international liver congress 2017 - 52nd annual meeting of the

european association for the study of the liver Netherlands. 2017;66(1 Supplement 1):S282. PubMed PMID: CN-01469285.

5988. Boyle CM, Maxwell DJ, Meguid SE, Ouaida D, Krass I. Missed doses: an evaluation in two drug distribution systems. Aust j hosp pharm. 1998;28(6):413-6. PubMed PMID: CN-00278995.

5989. Boyle JG, Logan PJ, Jones GC, Small M, Sattar N, Connell JM, et al. AMP-activated protein kinase is activated in adipose tissue of individuals with type 2 diabetes treated with metformin: a randomised glycaemia-controlled crossover study. Diabetologia. 2011;54(7):1799-809. doi: 10.1007/s00125-011-2126-4. PubMed PMID: CN-00800957.

5990. Boyle TA, Bishop A, Morrison B, Murphy A, Barker J, Ashcroft DM, et al. Pharmacist work stress and learning from quality related events. Research in Social & Administrative Pharmacy. 2016;12(5):772-83. doi: 10.1016/j.sapharm.2015.10.003. PubMed PMID: 117582929. Language: English. Entry Date: 20170724. Revision Date: 20191011. Publication Type: journal article.

5991. Boyle TA, Bishop AC, Duggan K, Reid C, Mahaffey T, Mackinnon NJ, et al. Keeping the "continuous" in continuous quality improvement: exploring perceived outcomes of CQI program use in community pharmacy. Research in Social & Administrative Pharmacy. 2014;10(1):45-57. doi: 10.1016/j.sapharm.2013.01.006. PubMed PMID: 103997377. Language: English. Entry Date: 20140912. Revision Date: 20170405. Publication Type: journal article.

5992. Boyle TA, Bishop AC, Hillier C, Mahaffey T, MacKinnon NJ, Zwicker B. Regulatory authority approaches to deploying quality improvement standards to community pharmacies: insights from the SafetyNET-RX program. Journal of Pharmacy Practice. 2014;27(2):138-49. PubMed PMID: 24150487.

5993. Boyle TA, Bishop AC, Mahaffey T, Mackinnon NJ, Ashcroft DM, Zwicker B, et al. Reflections on the role of the pharmacy regulatory authority in enhancing quality related event reporting in community pharmacies. Research in Social & Administrative Pharmacy. 2014;10(2):387-97. doi: 10.1016/j.sapharm.2013.06.002. PubMed PMID: 104042822. Language: English. Entry Date: 20141121. Revision Date: 20211029. Publication Type: journal article.

5994. Boyle TA, Bishop AC, Overmars C, MacMaster K, Mahaffey T, Zwicker B, et al. Uptake of Quality-Related Event Standards of Practice by Community Pharmacies. Journal of Pharmacy Practice. 2015;28(5):442-9. doi: 10.1177/0897190014522066. PubMed PMID: 110001626. Language: English. Entry Date: 20180117. Revision Date: 20180118. Publication Type: Article.

5995. Boyle TA, Ho C, Mackinnon NJ, Mahaffey T, Taylor JM. Safety implications of standardized continuous quality improvement programs in community pharmacy. Journal of Pharmacy Practice. 2013;26(3):228-36. PubMed PMID: 22842505.

5996. Boyle TA, MacKinnon NJ, Mahaffey T, Duggan K, Dow N. Challenges of standardized continuous quality improvement programs in community pharmacies: the case of SafetyNET-Rx. Research in Social and Administrative Pharmacy. 2012;8(6):499-508.

5997. Boyle TA, Mackinnon NJ, Mahaffey T, Duggan K, Dow N, Boyle TA, et al. Challenges of standardized continuous quality improvement programs in community pharmacies: the case of SafetyNET-Rx. Research in Social & Administrative Pharmacy. 2012;8(6):499-508. doi: 10.1016/j.sapharm.2012.01.005. PubMed PMID: 104384402. Language: English. Entry Date: 20130510. Revision Date: 20200708. Publication Type: journal article.

5998. Boyle TA, Mahaffey T, MacKinnon NJ, Deal H, Hallstrom LK, Morgan H. Determinants of medication incident reporting, recovery, and learning in community pharmacies: a conceptual model. Research in Social

and Administrative Pharmacy. 2011;7(1):93-107.

5999. Boyle TA, Scobie AC, MacKinnon NJ, Mahaffey T. Implications of process characteristics on quality-related event reporting in community pharmacy. Research in Social and Administrative Pharmacy. 2012;8(1):76-86. doi: https://doi.org/10.1016/j.sapharm.2010.11.004.

6000. Boyle TA, Scobie AC, MacKinnon NJ, Mahaffey T. Quality-related event learning in community pharmacies: Manual versus computerized reporting processes. Journal of the American Pharmacists Association. 2012;52(4):498-506. doi: https://doi.org/10.1331/JAPhA.2012.11004.

6001. Boyle TC, Coffey J, Palmer T. Men's Health Initiative Risk Assessment Study: Effect of Community Pharmacy–Based Screening. Journal of the American Pharmacists Association. 2004;44(5):569-77. doi: https://doi.org/10.1331/1544-3191.44.5.569.Boyle.

6002. Boyle TC, Coffey J, Palmer T. Men's health initiative risk assessment study: Effect of community pharmacy-based screening. Journal of the American Pharmacists Association. 2004;44(5):569-77. doi: 10.1331/1544-3191.44.5.569.Boyle.

6003. Boyles T, Freedman A. Post-exposure prophylaxis. Medicine. 2005;33(6):32-3. doi: https://doi.org/10.1383/medc.33.6.32.66011.

6004. Boyter AC, Ford NH, Zlotos L. Audit of budesonide/formoterol prescribing for asthma in community pharmacy in the UK. Respiratory Medicine. 2011;105(6):864-8. doi: 10.1016/j.rmed.2010.11.021. PubMed PMID: WOS:000291136800007.

6005. Boytsov N, Stockl K, Chen C-C, Wang F, Wang X, Cao Y, et al. Real-World Assessment of Prior Treatment Patterns in Patients Receiving Belantamab Mafodotin Using a Longitudinal Pharmacy and Medical Open Source Claims Database. Blood. 2021;138:4973. doi: https://doi.org/10.1182/blood-2021-152242.

6006. Boytsov N, Stockl KM, Mackay C, Wang PF, Sun K, Chen C-C, et al. MM-404 Real-World Belantamab Mafodotin Use: A US Retrospective Longitudinal Pharmacy and Medical Open-Source Claims Database Assessment. Clinical Lymphoma Myeloma and Leukemia. 2022;22:S422. doi: https://doi.org/10.1016/S2152-2650(22)01622-6.

6007. Boytsov NN, Bhattacharya R, Saverno K, Dixon L, Abbott PL, Zhang X, et al. Health Care Effect of Disease-Modifying Antirheumatic Drug Use on Patients with Rheumatoid Arthritis. Journal of Managed Care & Specialty Pharmacy. 2019;25(8):879-87. doi: 10.18553/jmcp.2019.25.8.879. PubMed PMID: WOS:000477748000003.

6008. Bozio CH, Isenhour C, McNamara LA. Characteristics of and meningococcal disease prevention strategies for commercially insured persons receiving eculizumab in the United States. Plos One. 2020;15(11). doi: 10.1371/journal.pone.0241989. PubMed PMID: WOS:000593948000055.

6009. Bozorgi A, Fahimnia B. Transforming the vaccine supply chain in Australia: Opportunities and challenges. Vaccine. 2021;39(41):6157-65. doi: https://doi.org/10.1016/j.vaccine.2021.08.033.

6010. Bozorgi A, Fahimnia B. Micro array patch (MAP) for the delivery of thermostable vaccines in Australia: A cost/benefit analysis. Vaccine. 2021;39(41):6166-73. doi: https://doi.org/10.1016/j.vaccine.2021.08.016.

6011. Bozovich M, Rubino CM, Edmunds J. Effect of a clinical pharmacist-managed lipid clinic on achieving National Cholesterol Education Program low-density lipoprotein goals. Pharmacotherapy.

2000;20(11):1375-83. PubMed PMID: CN-00549269.

6012. Braam KI, van Dijk-Lokkart EM, van Dongen JM, van Litsenburg RRL, Takken T, Huisman J, et al. Cost-effectiveness of a combined physical exercise and psychosocial training intervention for children with cancer: results from the quality of life in motion study. European journal of cancer care. 2017;26(6). doi: 10.1111/ecc.12586. PubMed PMID: CN-01657098.

6013. Brabcová I, Hajduchová H, Tóthová V, Chloubová I, Červený M, Prokešová R, et al. Reasons for medication administration errors, barriers to reporting them and the number of reported medication administration errors from the perspective of nurses: A cross-sectional survey. Nurse Education in Practice. 2023;70:103642. doi: https://doi.org/10.1016/j.nepr.2023.103642.

6014. Brabcova I, Tothova V, Hajduchova H, Chloubova I, Cerveny M, Prokesova R, et al. Evaluation of medication errors in the hospital environment. Vnitrni Lekarstvi. 2022;68(E-6):3-9. PubMed PMID: 36316205.

6015. Brabin L, Thomas G, Hopkins M, O'Brien K, Roberts SA. Delivery of chlamydia screening to young women requesting emergency hormonal contraception at pharmacies in Manchester, UK: A prospective study. BMC Women's Health. 2009;9. doi: 10.1186/1472-6874-9-7.

6016. Braccia D, Gilmore J. Effects of body mass index on the efficacy and safety of the granisetron transdermal system for chemotherapy-induced nausea and vomiting. Journal of oncology pharmacy practice. 2014;20(2 SUPPL. 1):11. doi: 10.1177/1078155213520288. PubMed PMID: CN-01057083.

6017. Bracegirdle L, Chapman S. Programmable Patients: Simulation of Consultation Skills in a Virtual Environment. Bio Algorithms Med Syst. 2010;6(11):111-5.

6018. Bracey G. The contribution of a pharmacy admissions service to patient care.

6019. Bracey G, Miller G, Franklin BD, Jacklin A, Gaskin G. The contribution of a pharmacy admissions service to patient care. Clinical Medicine. 2008;8(1):53-7. PubMed PMID: 18335670.

6020. Brach C, Keller D, Hernandez LM, Baur C, Parker R, Dreyer B, et al. Ten attributes of health literate health care organizations. NAM perspectives. 2012.

6021. Brackbill ML, Rahman A, Sandy J, Stam D, Harralson AF. Adjunctive sitagliptin therapy in postoperative cardiac surgery patients: a pilot study. Pharmacotherapy. 2011;31(10):399e. PubMed PMID: CN-01035318.

6022. Brackett A, Butler M, Chapman L. Using motivational interviewing in the community pharmacy to increase adult immunization readiness: A pilot evaluation. Journal of the American Pharmacists Association. 2015;55(2):182-6. doi: 10.1331/JAPhA.2015.14120.

6023. Brackett PD, Byrd DC, Duke LJ, Fetterman JW, Unterwagner WL, Staton AG, et al. Barriers to expanding advanced pharmacy practice experience site availability in an experiential education consortium. American Journal of Pharmaceutical Education. 2009;73(5). doi: 10.5688/aj730582.

6024. Brackley K, Aspden T, Petersen L. Visual Thinking Strategies-using art to support the development of critical thinking in pharmacy technicians. Pharmacy education. 2018;18(1):14-. PubMed PMID: CN-02130197.

6025. Bradbury F. How important is the role of the physician in the correct use of a drug? An observational cohort study in general practice. International Journal of Clinical Practice. 2004;58:27-32.

6026. Bradford DM. The house that Sam built: The Stripling family and Stripling's Drug Store [M.A.]. United States -- Texas: Stephen F. Austin State University; 2000.

6027. Bradley B, Deines S. Targeting transitions of care-Role of the pharmacist in a federally qualified health center. Mental Health Clinician. 2014;4(6):283-6. doi: 10.9740/mhc.n207349.

6028. Bradley CL, Luder HR, Beck AF, Bowen R, Heaton PC, Kahn RS, et al. Pediatric asthma medication therapy management through community pharmacy and primary care collaboration. Journal of the American Pharmacists Association. 2016;56(4):455-60. doi: https://doi.org/10.1016/j.japh.2016.03.007.

6029. Bradley CL, Luder HR, Beck AF, Bowen R, Heaton PC, Kahn RS, et al., editors. Pediatric asthma medication therapy management through community pharmacy and primary care collaboration. Journal of the American Pharmacists Association; 2016.

6030. Bradley CP, Bond C. Increasing the number of drugs available over the counter: Arguments for and against. British Journal of General Practice. 1995;45(399):553-6.

6031. Bradley CP, Bond C. INCREASING THE NUMBER OF DRUGS AVAILABLE OVER-THE-COUNTER - ARGUMENTS FOR AND AGAINST. British Journal of General Practice. 1995;45(399):553-6. PubMed PMID: WOS:A1995RY44300011.

6032. Bradley CP, Taylor RJ, Blenkinsopp A. Primary care–opportunities and threats: Developing prescribing in primary care. BMJ. 1997;314(7082):744. doi: 10.1136/bmj.314.7082.744.

6033. Bradley CT. An exploratoration of the role of community pharmacists in health promotion in Ireland [Ph.D.]. Ireland: Trinity College Dublin (University of Dublin) (Ireland); 2009.

6034. Bradley E, Hynam B, Nolan P. Nurse prescribing: reflections on safety in practice. Social science & medicine. 2007;65(3):599-609.

6035. Bradley F, Ashcroft DM, Crossley N. Negotiating inter-professional interaction: playing the general practitioner-pharmacist game. Sociology of Health and Illness. 2018;40(3):426-44. doi: 10.1111/1467-9566.12656.

6036. Bradley F, Ashcroft DM, Noyce PR. Integration and differentiation: a conceptual model of general practitioner and community pharmacist collaboration. Research in social and administrative pharmacy. 2012;8(1):36-46.

6037. Bradley F, Elvey R, Ashcroft D, Noyce P. Commissioning services and the new community pharmacy contract: (3) uptake of enhanced services. Pharmaceutical Journal. 2006;277(7414):224-6.

6038. Bradley F, Elvey R, Ashcroft DM, Noyce P. Influence of prescription charges on repeat prescribing in primary care. Journal of Clinical Pharmacy and Therapeutics. 2007;32(3):269-75. doi: 10.1111/j.1365-2710.2007.00821.x. PubMed PMID: WOS:000246802100008.

6039. Bradley F, Hammond M, Braund R. Stress, burnout, and the need for support: a survey of early career pharmacists. International Journal of Pharmacy Practice. 2024;32(1):91-6. doi: 10.1093/ijpp/riad079. PubMed PMID: WOS:001107329500001.

6040. Bradley F, Schafheutle EI, Willis SC, Noyce PR. Changes to supervision in community pharmacy: pharmacist and pharmacy support staff views. Health & social care in the community. 2013;21(6):644-54.

6041. Bradley F, Steven A, Ashcroft DM. The role of hidden curriculum in teaching pharmacy students about

patient safety. American journal of pharmaceutical education. 2011;75(7).

6042. Bradley F, Wagner AC, Elvey R, Noyce PR, Ashcroft DM. Determinants of the uptake of medicines use reviews (MURs) by community pharmacies in England: a multi-method study. Health policy. 2008;88(2-3):258-68.

6043. Bradley F, Willis SC, Noyce PR, Schafheutle EI. Restructuring supervision and reconfiguration of skill mix in community pharmacy: Classification of perceived safety and risk. Research in Social and Administrative Pharmacy. 2016;12(5):733-46.

6044. Bradley H, Lehmann U, Butler N. Emerging roles and competencies of district and sub-district pharmacists: A case study from Cape Town. Human Resources for Health. 2015;13(1). doi: 10.1186/s12960-015-0081-8.

6045. Bradley KD, Schumacher C, Borchert JS, Kliethermes MA, Anderson DK. Validity and reliability pilot study of a tool for assessing ambulatory care pharmacist practice. American Journal of Health-System Pharmacy. 2018;75(23):1890-901. doi: 10.2146/ajhp170678. PubMed PMID: WOS:000456291700015.

6046. Bradley KJ, Wros P, Bookman N, Mathews LR, Voss H, Ostrogorsky TL, et al. The Interprofessional Care Access Network (I-CAN): achieving client health outcomes by addressing social determinants in the community. Journal of Interprofessional Care. 2018. doi: 10.1080/13561820.2018.1560246.

6047. Bradley KJ, Wros P, Bookman N, Mathews LR, Voss H, Ostrogorsky TL, et al. The Interprofessional Care Access Network (I-CAN): achieving client health outcomes by addressing social determinants in the community. Journal of Interprofessional Care. 2023;37(sup1):S45-S52. doi: 10.1080/13561820.2018.1560246.

6048. Bradley LR. The effectiveness of patient medication information leaflets for African-American hypertensive patients. 1999. PubMed PMID: CN-00766605.

6049. Bradley NA, Power A, Hesselgreaves H, McMillan F, Bowie P. Safer pharmacy practice: a preliminary study of significant event analysis and peer feedback. International Journal of Pharmacy Practice. 2009;17(5):283-91. PubMed PMID: 20214270.

6050. Bradley PJ, Ferlito A, Lowe J, Devaney KO, Wei WI, Rinaldo A. A head and neck cancer patient dies! Why perform an autopsy: for the relatives, for the clinicians or for the pathologists? Acta Oto-Laryngologica. 2003;123(3):348-54. doi: 10.1080/0036554021000001349. PubMed PMID: WOS:000182665700003.

6051. Bradley R. ROBO-NURSE. Popular Science. 2011;278(6):46. PubMed PMID: 62799791.

6052. Bradley SEK, Rosapep L, Shiras T. Where do caregivers take their sick children for care? an analysis of care seeking and equity in 24 usaid priority countries. Global Health Science and Practice. 2020;8(3):518-33. doi: 10.9745/GHSP-D-20-00115.

6053. Bradley SM, Hess GP, Stewart P, Armstrong EJ, Farmer SA, Wasfy JH, et al. Implications of the PEGASUS-TIMI 54 trial for US clinical practice. Open heart. 2017;4(1). doi: 10.1136/openhrt-2016-000580. PubMed PMID: CN-01373470.

6054. Bradley TJ, Round AP, Ramsden M. The success of an evidence-based rational prescribing intervention: a retrospective study. Pharmaceutical journal. 2000;265(7103):26-9. PubMed PMID: CN-00343242.

6055. Bradley-Baker LR, Mullins CD, Baquet CR. Pharmacists' assessment of facets of health literacy in

pharmacy practice settings. Journal of Pharmacy Technology. 2011;27(2):55-62. doi: 10.1177/875512251102700202.

6056. Bradshaw M, Tomany-Korman S, Flores G. Language barriers to prescriptions for patients with limited english proficiency: A survey of pharmacies. Pediatrics. 2007;120(2):e225-e35. doi: 10.1542/peds.2006-3151.

6057. Bradshaw P, Jarvis A. Reissuing returned medicines to developing countries [8]. Pharmaceutical Journal. 2004;273(7320):514.

6058. Bradshaw SJ, Doucette WR. Community pharmacists as patient advocates: physician attitudes. Journal of the American Pharmaceutical Association (Washington,DC : 1996). 1998;38(5):598-602. doi: 10.1016/S1086-5802(16)30373-4.

6059. BRADY AM, MALONE AM, Fleming S. A literature review of the individual and systems factors that contribute to medication errors in nursing practice. Journal of nursing management. 2009;17(6):679-97.

6060. Brady BL, Tkacz J, Meyer R, Bolge SC, Ruetsch C. Assessment of Rheumatoid Arthritis Quality Process Measures and Associated Costs. Population Health Management. 2017;20(1):31-40. doi: 10.1089/pop.2015.0133. PubMed PMID: WOS:000392877800006.

6061. Brady D, Franklin BD. An evaluation of the contribution of the medical admissions pharmacist at a London teaching hospital. International Journal of Pharmacy Practice. 2004;12(1):1-6.

6062. Braga GK, Fonseca MJV. From record keeping to key performance indicators: managing quality in compounding pharmacies. International Journal of Pharmaceutical Compounding. 2010;14(2):136-9. PubMed PMID: 105158775. Language: English. Entry Date: 20100514. Revision Date: 20150711. Publication Type: Journal Article.

6063. Braga SFF, Clark KJ, Shirer AE. An overview of generalized anxiety disorder for the community pharmacist. US Pharmacist. 2019;44(11):23-8.

6064. Bragazzi NL, Mansour M, Bonsignore A, Ciliberti R. The Role of Hospital and Community Pharmacists in the Management of COVID-19: Towards an Expanded Definition of the Roles, Responsibilities, and Duties of the Pharmacist. Pharmacy. 2020;8(3). doi: 10.3390/pharmacy8030140. PubMed PMID: WOS:000579609500001.

6065. Brahams D. Spring in London with Covid-19: a personal view. The Medico-legal journal. 2020;88(2):57-64. doi: 10.1177/0025817220923692.

6066. Brahm N, Kelly-Rehm M, Farmer KC. Collaboration: What can health-care organizations learn about pharmacist retention from Magnet status hospitals? Research in Social and Administrative Pharmacy. 2009;5(4):382-9. doi: 10.1016/j.sapharm.2008.11.001.

6067. Brahm NC, Davis T. Psychiatric pharmacy delivery via an online course: psychoactive substances. Currents in Pharmacy Teaching and Learning. 2010;2(4):204-10. doi: https://doi.org/10.1016/j.cptl.2010.07.007.

6068. Brahma DK, Wahlang JB, Marak MD, Sangma MC. Adverse drug reactions in the elderly. Journal of pharmacology and pharmacotherapeutics. 2013;4(2):91-4.

6069. Brahmbhatt A. Cybersecurity in Community Pharmacies [M.S.]. United States -- New York: Long Island University, The Brooklyn Center; 2019.

6070. Brailer DJ. Interoperability: The Key To The Future Health Care System: Interoperability will bind together a wide network of real-time, life-critical data that not only transform but become health care. Health affairs. 2005;24(Suppl1):W5-19-W5-21.

6071. Brailey A, Valentine MJ, Sackin P. NHS Education for Scotland (NES) and its multiprofessional role. Education for Primary Care. 2008;19(4):436-40.

6072. Braillon A, Nguyen-Khac E, Merlin J, Dubois G, Gondry J, Capron D. HBsAg screening during pregnancy in the French province Picardy. Gynecologie Obstetrique et Fertilite. 2010;38(1):13-7. doi: 10.1016/j.gyobfe.2009.11.002.

6073. Brailsford M. The Effect of Value-Based Insurance Design on Diabetes Medication Adherence, Glycemic Control, and Pharmacy Cost in Patients with Diabetes in an Employer-Insured Population [Ph.D.]. United States -- Utah: The University of Utah; 2020.

6074. Braithwaite E, Todd OM, Atkin A, Hulatt R, Tadrous R, Alldred DP, et al. Interventions for reducing anticholinergic medication burden in older adults—a systematic review and meta-analysis. Age & Ageing. 2023;52(9):1-9. doi: 10.1093/ageing/afad176. PubMed PMID: 172443445.

6075. Braithwaite J, Herkes J, Ludlow K, Testa L, Lamprell G. Association between organisational and workplace cultures, and patient outcomes: systematic review. BMJ open. 2017;7(11):e017708.

6076. Braithwaite K, Hogarth R, Mackintosh CM, Lewis A. Skills, knowledge and experience lost in York Place (multiple letters) [8]. Pharmaceutical Journal. 2003;271(7275):678.

6077. Brajkovic A, Bicanic LA, Orehovacki H, Prkacin I, de Oliveira DR, Mucalo I. Prescribers' approval rate of pharmacist-initiated interventions to optimise patients' clinical status of hypertension in the ambulatory care setting. Acta Pharmaceutica. 2023;73(4):723-34. doi: https://dx.doi.org/10.2478/acph-2023-0047. PubMed PMID: 2029533204.

6078. Brajkovic A, Bosnar L, Nascimento M, Prkacin I, Balenovic A, Ramalho de Oliveira D, et al. Healthcare Utilisation and Clinical Outcomes in Older Cardiovascular Patients Receiving Comprehensive Medication Management Services: A Nonrandomised Clinical Study. International Journal of Environmental Research & Public Health [Electronic Resource]. 2022;19(5):27. PubMed PMID: 35270472.

6079. Bramble JD, Abbott AA, Fuji KT, Paschal KA, Siracuse MV, Galt K. Patient safety perspectives of providers and nurses: The experience of a rural ambulatory care practice using an EHR with E-prescribing. The Journal of Rural Health. 2013;29(4):383-91.

6080. Bramness JG, Skurtveit S, Mørland J, Engeland A. The risk of traffic accidents after prescriptions of carisoprodol. Accident Analysis & Prevention. 2007;39(5):1050-5. doi: https://doi.org/10.1016/j.aap.2007.02.002.

6081. Branch-Elliman W, Pizer SD, Dasinger EA, Gold HS, Abdulkerim H, Rosen AK, et al. Facility type and surgical specialty are associated with suboptimal surgical antimicrobial prophylaxis practice patterns: a multi-center, retrospective cohort study. Antimicrobial Resistance and Infection Control. 2019;8. doi: 10.1186/s13756-019-0503-9. PubMed PMID: WOS:000460877200003.

6082. Branch-Elliman W, Strymish J, Itani KMF, Gupta K. Using clinical variables to guide surgical site infection detection: A novel surveillance strategy. American Journal of Infection Control. 2014;42(12):1291-5. doi: https://doi.org/10.1016/j.ajic.2014.08.013.

6083. Brand R, ברנד ר. Barriers to Adherence with Oral Anti-Hyperglycemic Medications and Their

Correlation with Adherence Rate: a Cross Sectional Study in Three Sub-Populations in Israel [Master's]. Israel: University of Haifa (Israel); 2018.

6084. Branda ME, LeBlanc A, Shah ND, Tiedje K, Ruud K, Van Houten H, et al. Shared decision making for patients with type 2 diabetes: a randomized trial in primary care. BMC health services research. 2013;13:301. doi: 10.1186/1472-6963-13-301. PubMed PMID: CN-00913692.

6085. Brandt A. Advancements of 3d Bioprinting: a Market Development Study. 2023. PubMed PMID: PQDT:84652726.

6086. Brandt A, Iwarsson S, Stahle A. Older people's use of powered wheelchairs for activity and participation. Journal of Rehabilitation Medicine. 2004;36(2):70-7. doi: 10.1080/16501970310017432. PubMed PMID: WOS:000220146600003.

6087. Brandt J. A roadmap for psychedelic pharmacy in Canada: A proposed policy and operations approach for controlled access to select psychedelics for treatment of mental illness. Drug Science, Policy and Law. 2023;9:20503245231170340.

6088. Brandt J, Lê M-L, Jantscher S, Bricelj A, Louizos C, Ng S, et al. Medication review service implementation in community pharmacy settings: Scoping review with focus on implementation studies. Research in Social and Administrative Pharmacy. 2020;16(7):875-85. doi: https://doi.org/10.1016/j.sapharm.2019.10.005.

6089. Brandt J, Lê ML, Jantscher S, Bricelj A, Louizos C, Ng S, et al. Medication review service implementation in community pharmacy settings: Scoping review with focus on implementation studies. Research in Social and Administrative Pharmacy. 2020;16(7):875-85. doi: 10.1016/j.sapharm.2019.10.005.

6090. Brandt N. Controlling the Use of Antibiotics: Are You Ready? Consultant Pharmacist. 2017;32 Suppl A(5):1-2. PubMed PMID: 28655369.

6091. Brandt NJ, Worz C, Clackum SF. Expanding Opportunities in the Postacute Long-Term Care Setting: Bringing Medication Safety to the Next Level. Pharmacotherapy:The Journal of Human Pharmacology & Drug Therapy. 2018;38(8):862-6. PubMed PMID: 29888800.

6092. Brandys J, Panas M, Skowron A, Urbaczka A. Assessment of the effectiveness of pharmacists' activities in treatment of tobacco-addicted patients in the context of pharmacists' counselling in Cracow's pharmacies. Przegląd lekarski. 2009;66(10):836-40.

6093. Branham AR, Katz AJ, Moose JS, Ferreri SP, Farley JF, Marciniak MW. Retrospective analysis of estimated cost avoidance following pharmacist-provided medication therapy management services. Journal of Pharmacy Practice. 2013;26(4):420-7. PubMed PMID: 23178415.

6094. Branquinho M, Shakeel N, Horsch A, Fonseca A. Frontline health professionals' perinatal depression literacy: A systematic review. Midwifery. 2022;111:103365. doi: https://doi.org/10.1016/j.midw.2022.103365.

6095. Brantley AF, Rossi DM, Barnes-Warren S, Francisco JC, Schatten I, Dave V. Bridging gaps in care: Implementation of a pharmacist-led transitions-of-care program. American Journal of Health-System Pharmacy. 2018;75(5 Supplement 1):S1-S5. PubMed PMID: 29472274.

6096. Brasić JR, Fogelman D. CLINICIAN SAFETY. Psychiatric Clinics of North America. 1999;22(4):923-40. doi: https://doi.org/10.1016/S0193-953X(05)70134-9.

6097. Brata C, Fisher C, Marjadi B, Schneider CR, Clifford RM. Factors influencing the current practice of self-medication consultations in Eastern Indonesian community pharmacies: a qualitative study. Bmc Health Services Research. 2016;16. doi: 10.1186/s12913-016-1425-3. PubMed PMID: WOS:000376572300001.

6098. Brata C, Gudka S, Schneider CR, Clifford RM. A review of the provision of appropriate advice by pharmacy staff for self-medication in developing countries. Research in Social and Administrative Pharmacy. 2015;11(2):136-53. doi: 10.1016/j.sapharm.2014.07.003.

6099. Brata C, Gudka S, Schneider CR, Everett A, Fisher C, Clifford RM. A review of the information-gathering process for the provision of medicines for self-medication via community pharmacies in developing countries. Research in Social & Administrative Pharmacy. 2013;9(4):370-83. doi: 10.1016/j.sapharm.2012.08.001. PubMed PMID: WOS:000322628000002.

6100. Brata C, Halim SV, Setiawan E, Presley B, Wibowo YI, Schneider CR. The competency of Indonesian pharmacy students in handling a self-medication request for a cough: A simulated patient study. Pharmacy Practice. 2021;19(2). doi: 10.18549/PharmPract.2021.2.2269.

6101. Brata C, Marjadi B, Schneider CR, Murray K, Clifford RM. Information-gathering for self-medication via Eastern Indonesian community pharmacies: a cross-sectional study. Bmc Health Services Research. 2015;15. doi: 10.1186/s12913-014-0670-6. PubMed PMID: WOS:000348817600001.

6102. Brata C, Schneider CR, Marjadi B, Clifford RM. The provision of advice by pharmacy staff in eastern Indonesian community pharmacies. Pharmacy Practice-Granada. 2019;17(2). doi: 10.18549/PharmPract.2019.2.1452. PubMed PMID: WOS:000508118700010.

6103. Bratberg JP. Opioids, naloxone, and beyond: The intersection of medication safety, public health, and pharmacy. Journal of the American Pharmacists Association. 2017;57(2, Supplement):S5-S7. doi: https://doi.org/10.1016/j.japh.2017.02.006.

6104. Bratberg JP, Simmons A, Arya V, Bhatia A, Vakharia SP. Support, don't punish: Drug decriminalization is harm reduction. Journal of the American Pharmacists Association. 2023;63(1):224-9. doi: https://doi.org/10.1016/j.japh.2022.12.017.

6105. Bratberg JP, Simmons A, Arya V, Bhatia A, Vakharia SP. Support, don't punish: Drug decriminalization is harm reduction. Journal of the American Pharmacists Association. 2023;63(1):224-9. doi: https://doi.org/10.1016/j.japh.2022.12.017.

6106. Brateanu A, Shah G, Luxenburg J, Reali-Sorrell M, Lovelace R, Pfoh E, et al. Impact on patients compliance with medication using pre-packed blisters for long term medical therapy (i-comply study). Journal of general internal medicine. 2017;32(2):S220-. PubMed PMID: CN-01362598.

6107. Bratkowska K, Religioni U, Krysinski J, Merks P. Quality of pharmaceutical services in independent pharmacies and pharmacy chains in poland from the patient perspective. Patient preference and adherence. 2020;14:2459-67. doi: 10.2147/PPA.S284014. PubMed PMID: CN-02213572.

6108. Brauche CL. Exposure of Pharmacy Students to a Public Health Problem—Mental Retardation*. Journal of the American Pharmaceutical Association (1961). 1972;12(9):462-3. doi: https://doi.org/10.1016/S0003-0465(16)32341-2.

6109. Braun BI, Darcy L, Divi C, Robertson J, Fishbeck J. Hospital bioterrorism preparedness linkages with the community: Improvements over time. American Journal of Infection Control. 2004;32(6):317-26. doi: https://doi.org/10.1016/j.ajic.2004.01.003.

6110. Braun CA, van Bommel-Wegmann S, Simons S, Diederichs L, Wulfing M, Schummer B, et al. Initiation of a drug-drug interaction register for community pharmacies. International Journal of Clinical Pharmacy. 2013;35(3):498-. PubMed PMID: WOS:000318950900058.

6111. Braun CA, Woltersdorf R, Simons S, Klahn D, Jaehde U. Drug-drug interaction management in German community pharmacies. International Journal of Clinical Pharmacy. 2016;38(2):495-6. PubMed PMID: WOS:000374326900103.

6112. Braun L, Cohen M. Herbs and natural supplements, volume 2: An evidence-based guide: Elsevier Health Sciences; 2015.

6113. Braun LA, Cohen M. Use of Complementary Medicines by Cardiac Surgery Patients; Undisclosed and Undetected. Heart, Lung and Circulation. 2011;20(5):305-11. doi: https://doi.org/10.1016/j.hlc.2011.01.013.

6114. Braun LA, Spitzer O, Tiralongo E, Wilkinson JM, Bailey M, Poole S, et al. The prevalence and experience of Australian naturopaths and Western herbalists working within community pharmacies. Bmc Complementary and Alternative Medicine. 2011;11. doi: 10.1186/1472-6882-11-41. PubMed PMID: WOS:000292289300001.

6115. Braun LA, Tiralongo E, Wilkinson JM, Spitzer O, Bailey M, Poole S, et al. Perceptions, use and attitudes of pharmacy customers on complementary medicines and pharmacy practice. BMC Complementary and Alternative Medicine. 2010;10. doi: 10.1186/1472-6882-10-38.

6116. Braun M, Reinhardt H, Rosner A, Schilli P, Hug MJ, Engelhardt M. Chemotherapy (CTx) surveillance at the Comprehensive Cancer Center Freiburg (CCCF) - transferrable patient safety. Oncology Research and Treatment. 2023;46(Supplement 5):125. doi: https://dx.doi.org/10.1159/000533576. PubMed PMID: 642906887.

6117. Braun R, Sheoran B, Idelson L, Lykens J, Klausner J. PrEPTECH - Integration of an on- and off-line holistic PrEP solution for youth. Journal of the International AIDS Society. 2020;23(SUPPL 4). doi: 10.1002/jia2.25547. PubMed PMID: CN-02242893.

6118. Braund R. The Rationale for Robust Pharmacovigilance in Monitoring the Safety of Traditional Medicines and Natural Health Products: Joanne Barnes, editor. Adis: Hardcover ISBN 978-3-031-07273-4, eBook ISBN 978-3-031-07275-8, 2022. Drug Safety. 2023;46(4):417-8. doi: 10.1007/s40264-023-01282-8. PubMed PMID: 163166125.

6119. Braund R, Bannerman SL, Lim C, Moon J, Nicholls J, Straight P, et al. Recommendations of community pharmacists for the treatment of sprains and strains. International Journal of Pharmacy Practice. 2006;14(4):271-6. doi: 10.1211/ijpp.14.4.0007.

6120. Braund R, Chesney KM, Keast EP, Ng LJ, Qi S, Samaranayaka S, et al. Are all pharmacy staff interested in potential future roles? International Journal of Pharmacy Practice. 2012;20(6):417-21. doi: 10.1111/j.2042-7174.2012.00216.x.

6121. Braund R, Coulter CV, Bodington AJ, Giles LM, Greig A-M, Heaslip LJ, et al. Drug related problems identified by community pharmacists on hospital discharge prescriptions in New Zealand. International Journal of Clinical Pharmacy. 2014;36(3):498-502. doi: 10.1007/s11096-014-9935-8. PubMed PMID: 103823116. Language: English. Entry Date: 20150206. Revision Date: 20211030. Publication Type: journal article.

6122. Braund R, Coulter CV, Bodington AJ, Giles LM, Greig AM, Heaslip LJ, et al. Drug related problems identified by community pharmacists on hospital discharge prescriptions in New Zealand. International

Journal of Clinical Pharmacy. 2014;36(3):498-502. PubMed PMID: 24700340.

6123. Braund R, Furlan HM, George K, Havell MM, Murphy JL, West MK. Interventions performed by New Zealand community pharmacists while dispensing prescription medications. Pharmacy world & science. 2010;32:22-5.

6124. Braund R, Furlan HM, George K, Havell MMA, Murphy JL, West MK. Interventions performed by New Zealand community pharmacists while dispensing prescription medications. Pharmacy World and Science. 2010;32(1):22-5. doi: 10.1007/s11096-009-9343-7.

6125. Brauner MS, Accursi ML, Cantillo SL, Baxter MV, Burant CJ. Project EVADE: Evaluating the effects of a pharmacist-run transitions of care clinic on hospital readmissions. Journal of the American Pharmacists Association: JAPhA. 2020;60(3):503-8. PubMed PMID: 31866388.

6126. Braungart C, Watson A, Rubin R. The effects of interprofessional collaboration on nurse managed warfarin program. Journal of Interprofessional Education & Practice. 2018;13:56-8. doi: https://doi.org/10.1016/j.xjep.2018.09.003.

6127. Bravata DM, Coffing JM, Kansagara D, Myers J, Murphy L, Homoya BJ, et al. Association Between Antithrombotic Medication Use After Bioprosthetic Aortic Valve Replacement and Outcomes in the Veterans Health Administration System. Jama Surgery. 2019;154(2). doi: 10.1001/jamasurg.2018.4679. PubMed PMID: WOS:000459484600001.

6128. Bray P, Cummings DM, Morrissey S, Thompson D, Holbert D, Wilson K, et al. Improved outcomes in diabetes care for rural African Americans. Annals of family medicine. 2013;11(2):145-50. doi: 10.1370/afm.1470. PubMed PMID: CN-00910489.

6129. Bray P, Thompson D, Wynn JD, Cummings DM, Whetstone L. Confronting disparities in diabetes care: the clinical effectiveness of redesigning care management for minority patients in rural primary care practices. Journal of rural health. 2005;21(4):317-21. PubMed PMID: CN-00597866.

6130. Brazeale HS, Fuentes A, Adeola M. Analysis of Direct Oral Anticoagulant Therapy With Concomitant Use of Interacting Antiretroviral Agents. Journal of Pharmacy Practice. doi: 10.1177/08971900211034258. PubMed PMID: WOS:000684880900001.

6131. Brazeale HS, Fuentes A, Adeola M. Analysis of Direct Oral Anticoagulant Therapy With Concomitant Use of Interacting Antiretroviral Agents. Journal of Pharmacy Practice. 2023;36(2):286-94. doi: 10.1177/08971900211034258. PubMed PMID: 162839365.

6132. Brazinha I, Fernandez-Llimos F. Barriers to the implementation of advanced clinical pharmacy services at Portuguese hospitals. International Journal of Clinical Pharmacy. 2014;36(5):1031-8. doi: 10.1007/s11096-014-9991-0. PubMed PMID: WOS:000343647000023.

6133. Brearly TW, Goodman CS, Haynes C, McDermott K, Rowland JA. Improvement of postinpatient psychiatric follow-up for veterans using telehealth. American Journal of Health-System Pharmacy. 2020;77(4):288-94. doi: 10.1093/ajhp/zxz314. PubMed PMID: WOS:000565889600009.

6134. Breatnach CV, Howlett MM, Cleary BJ. Defining electronic-prescribing and infusion-related medication errors in paediatric intensive care - a Delphi study. BMC Medical Informatics & Decision Making. 2018;18(5):1-10. doi: 10.1186/s12911-018-0713-8. PubMed PMID: 133437658. Language: English. Entry Date: In Process. Revision Date: 20190809. Publication Type: journal article.

6135. Breault RR, Whissell JG, Hughes CA, Schindel TJ. Development and implementation of the

compensation plan for pharmacy services in Alberta, Canada. Journal of the American Pharmacists Association. 2017;57(4):532-41. doi: 10.1016/j.japh.2017.05.004. PubMed PMID: WOS:000408226300022.

6136. Breaux S, Desrosiers FAD, Neira M, Sinha S, Nislow C. Pharmacogenomics at the point of care: A community pharmacy project in British Columbia. Journal of Personalized Medicine. 2021;11(1):1-18. doi: 10.3390/jpm11010011.

6137. Brecher DB. Intervention with the STOPP/START criteria in elderly residents of a chronic geriatric facility: a randomized clinical trial. Journal of pain and symptom management. 2015;49(5):967-8. doi: 10.1016/j.jpainsymman.2015.04.002. PubMed PMID: CN-01074286.

6138. Breeman D. What the crystal ball holds: Experts give their take. Drug Topics. 2007;151(6).

6139. Breen L. A preliminary examination of risk in the pharmaceutical supply chain (PSC) in the national health service (NHS). 2008.

6140. Breen L, Benn J, Marques I, Peat G, Fylan B, Silcock J, et al. Developing a person-centred deprescribing process in primary care for older people living with frailty. International Journal of Pharmacy Practice. 2023;31(Supplement 1):i27. doi: https://dx.doi.org/10.1093/riad021.031. PubMed PMID: 641262492.

6141. Bregnhøj L, Thirstrup S, Kristensen MB, Bjerrum L, Sonne J. Combined intervention programme reduces inappropriate prescribing in elderly patients exposed to polypharmacy in primary care. European Journal of Clinical Pharmacology. 2009;65(2):199-207. doi: 10.1007/s00228-008-0558-7. PubMed PMID: 105638239. Language: English. Entry Date: 20090515. Revision Date: 20200708. Publication Type: Journal Article.

6142. Breiholz S, Griese-Mammen N, Krueger K, Schaefer M, Schulz M. Acceptance of a medication refill reminder service in German community pharmacy practice. Pharmazie. 2019;74(3):186-90. doi: 10.1691/ph.2019.8669.

6143. Breimer DD. Future training needs in the pharmaceutical sciences: Academia - Industry. European Journal of Pharmaceutical Sciences. 2001;12(4):347-52. doi: 10.1016/S0928-0987(01)00099-9.

6144. Breimer DD. Future training needs in the pharmaceutical sciences: Academia Industry. European Journal of Pharmaceutical Sciences. 2001;12(4):347-52. doi: 10.1016/s0928-0987(01)00099-9. PubMed PMID: WOS:000167531700001.

6145. Breitner JCS, Haneuse S, Walker R, Dublin S, Crane PK, Gray SL, et al. Risk of dementia and AD with prior exposure to NSAIDs in an elderly community-based cohort. Neurology. 2009;72(22):1899-905. doi: 10.1212/WNL.0b013e3181a18691. PubMed PMID: WOS:000266524600005.

6146. Breitner JCS, Haneuse SJPA, Walker R, Dublin S, Crane PK, Gray SL, et al. Risk of dementia and AD with prior exposure to NSAIDs in an elderly community-based cohort. Neurology. 2009;72(22):1899-905. doi: 10.1212/WNL.0b013e3181a18691.

6147. Breland BD. Continuous quality improvement using intelligent infusion pump data analysis. American Journal of Health-System Pharmacy. 2010;67(17):1446-55. doi: 10.2146/ajhp090588.

6148. Breland JY, Chee CP, Zulman DM. Racial Differences in Chronic Conditions and Sociodemographic Characteristics Among High-Utilizing Veterans. Journal of Racial and Ethnic Health Disparities. 2015;2(2):167-75. doi: 10.1007/s40615-014-0060-0. PubMed PMID: WOS:000218510200003.

6149. Brem H, Maggi J, Nierman D, Rolnitzky L, Bell D, Rennert R, et al. High cost of stage IV pressure ulcers. The American Journal of Surgery. 2010;200(4):473-7.

6150. Bremberg ER, Hising C, Nylen U, Ehrsson H, Eksborg S. An evaluation of pharmacist contribution to an oncology ward in a Swedish hospital. Journal of Oncology Pharmacy Practice. 2006;12(2):75-81. PubMed PMID: 16984745.

6151. Bremer S, Henjum S, Sæther EM, Hovland R. Drug-related problems and satisfaction among patients receiving pharmacist-led consultations at the initiation of cardiovascular drugs. Research in Social and Administrative Pharmacy. 2022;18(11):3939-47. doi: https://doi.org/10.1016/j.sapharm.2022.06.005.

6152. Breniaux M, Charpiat B. Analyse des pratiques de prescription de l'héparine sodique au pousse-seringue électrique. Annales Pharmaceutiques Françaises. 2022;80(6):943-9. doi: https://doi.org/10.1016/j.pharma.2022.02.009.

6153. Breniaux M, Charpiat B. [Analysis of heparin sodium prescribing practices with an electric syringe pump]. Annales Pharmaceutiques Francaises. 2022;80(6):943-9. PubMed PMID: 35248541.

6154. Breniaux M, Charpiat B. Choix d'un protocole d'administration d'héparine sodique au pousse-seringue électrique par des infirmières et des prescripteurs. Annales de Cardiologie et d'Angéiologie. 2023;72(4):N.PAG-N.PAG. doi: 10.1016/j.ancard.2023.101645. PubMed PMID: 172778334.

6155. Brennan AT, Dollear JT, Hu M, Matlin SO, Shrank HW, Choudhry KN, et al. An integrated pharmacy-based program improved medication prescription and adherence rates in diabetes patients. Health Affairs. 2012;31(1):120-9. doi: 10.1377/hlthaff.2011.0931.

6156. Brennan R, Wazaify M, Shawabkeh H, Boardley I, McVeigh J, Van Hout MC. A Scoping Review of Non-Medical and Extra-Medical Use of Non-Steroidal Anti-Inflammatory Drugs (NSAIDs). Drug Safety. 2021;44(9):917-28. doi: 10.1007/s40264-021-01085-9. PubMed PMID: WOS:000679650100001.

6157. Brennan TA, Dollear TJ, Hu M, Matlin OS, Shrank WH, Choudhry NK, et al. An Integrated Pharmacy-Based Program Improved Medication Prescription And Adherence Rates In Diabetes Patients. Health Affairs. 2012;31(1):120-9. doi: 10.1377/hlthaff.2011.0931. PubMed PMID: WOS:000299309000016.

6158. Brennan-Bourdon LM, Vázquez-Alvarez AO, Gallegos-Llamas J, Koninckx-Cañada M, Marco-Garbayo JL, Huerta-Olvera SG. A study of medication errors during the prescription stage in the pediatric critical care services of a secondary-tertiary level public hospital. BMC Pediatrics. 2020;20(1):1-8. doi: 10.1186/s12887-020-02442-w. PubMed PMID: 147445184. Language: English. Entry Date: 20210711. Revision Date: 20210923. Publication Type: journal article.

6159. Brennan-Tovey K, Aquino MRJ, Flanagan S, Kaner E, Wearn A, Bigirumurame T, et al. Implementation of the NHS-funded tobacco dependence services in England: a qualitative study to understand the contexts of implementation. The Lancet. 2022;400:S24. doi: https://doi.org/10.1016/S0140-6736(22)02234-6.

6160. Brenneman R, Shirk MB, Liscynesky C, Tran N, Rozycki E. Pharmacist involvement in blood culture follow up for patients discharged from the emergency department. The American Journal of Emergency Medicine. 2023;72:101-6. doi: https://doi.org/10.1016/j.ajem.2023.07.019.

6161. Brenner AT, Waters AR, Wangen M, Rohweder C, Odebunmi O, Marciniak MW, et al. Patient preferences for the design of a pharmacy-based colorectal cancer screening program. Cancer Causes and Control. 2023;34:99-112. doi: 10.1007/s10552-023-01687-x.

6162. Breslau J, Dana B, Pincus H, Horvitz-Lennon M, Matthews L. Empirically identified networks of healthcare providers for adults with mental illness. BMC Health Services Research. 2021;21(1). doi: 10.1186/s12913-021-06798-2.

6163. Breslau N, Kessler RC, Chilcoat HD, Schultz LR, Davis GC, Andreski P. Trauma and posttraumatic stress disorder in the community: the 1996 Detroit Area Survey of Trauma. Archives of general psychiatry. 1998;55(7):626-32.

6164. Breslau N, Reboussin BA, Anthony JC, Storr CL. The structure of posttraumatic stress disorder: latent class analysis in 2 community samples. Archives of General Psychiatry. 2005;62(12):1343-51.

6165. Breslau N, Schultz LR, Johnson EO, Peterson EL, Davis GC. Smoking and the risk of suicidal behavior: a prospective study of a community sample. Archives of general psychiatry. 2005;62(3):328-34.

6166. Breslin S, O'Driscoll L. Three-dimensional cell culture: the missing link in drug discovery. Drug discovery today. 2013;18(5-6):240-9.

6167. Bressler CJ, Letson MM, Kline D, McCarthy T, Davis J, Leonard JC. CHARACTERISTICS OF NEIGHBORHOODS WHERE EMERGENCY MEDICAL SERVICES ENCOUNTER CHILDREN AT RISK FOR MALTREATMENT. Prehospital Emergency Care. 2019;23(5):672-82. doi: 10.1080/10903127.2019.1573940. PubMed PMID: WOS:000509907800009.

6168. Bressler T, Persico L. Interprofessional education: Partnerships in the educational proc. Nurse Education in Practice. 2016;16(1):144-7. doi: https://doi.org/10.1016/j.nepr.2015.07.004.

6169. Breton M, Bourbeau K, Cusson JR, Grégoire J, Guévremont C, Labranche S, et al. The Drug Utilization Review Network of Quebec. Healthcare management forum / Canadian College of Health Service Executives = Forum gestion des soins de santé / Collège canadien des directeurs de services de santé. 1996;9(2):44-7. doi: 10.1016/S0840-4704(10)60852-6.

6170. Breton M, Ferreira E, Letarte N, Bussières JF. Mieux s'approprier les données relatives aux rôles et retombées de l'activité pharmaceutique : étude quantitative et qualitative auprès de quatre groupes d'experts. Annales Pharmaceutiques Françaises. 2017;75(2):152-62. doi: https://doi.org/10.1016/j.pharma.2016.09.003.

6171. Breton M, Ferreira E, Letarte N, Bussieres JF. [Data regarding the roles and impact of pharmaceutical activities: Quantitative and qualitative study with four groups of experts]. Annales Pharmaceutiques Francaises. 2017;75(2):152-62. PubMed PMID: 27855948.

6172. Brett V, Sikes C, Xiang J, Oh C, Biondi D. Post hoc analysis of pooled safety data from eleven phase 3 clinical trials to identify potential pharmacodynamic drug interactions between tapentadol and SSRIs/SNRIs. Journal of pharmacy practice. 2012;25(2):265. doi: 10.1177/0897190012441353. PubMed PMID: CN-01032853.

6173. Brettle RP, Wilson A, Povey S, Morris S, Morgan R, Leen CLS, et al. Combination therapy for HIV: the effect on inpatient activity, morbidity and mortality of a cohort of patients. International Journal of Std & Aids. 1998;9(2):80-7. doi: 10.1258/0956462981921729. PubMed PMID: WOS:000071971400004.

6174. Breuker C, Abraham O, Di Trapanie L, Mura T, Macioce V, Boegner C, et al. Patients with diabetes are at high risk of serious medication errors at hospital: Interest of clinical pharmacist intervention to improve healthcare. European Journal of Internal Medicine. 2017;38:38-45.

6175. Breuker C, Abraham O, di Trapanie L, Mura T, Macioce V, Boegner C, et al. Patients with diabetes are at high risk of serious medication errors at hospital: Interest of clinical pharmacist intervention to improve

healthcare. European Journal of Internal Medicine. 2017;38:38-45. doi: https://doi.org/10.1016/j.ejim.2016.12.003.

6176. Breuker C, Faucanie M, Laureau M, Perier D, Pinzani V, Marin G, et al. Impact of a medico-pharmaceutical follow-up and an optimized communication between hospital and community on the readmission to the emergency department for an adverse drug event: URGEIM, study protocol for a randomized controlled trial. Trials. 2021;22(1):521. doi: 10.1186/s13063-021-05501-4. PubMed PMID: CN-02301141.

6177. Breuker C, Macioce V, Mura T, Audurier Y, Boegner C, Jalabert A, et al. Medication errors at hospital admission and discharge in Type 1 and 2 diabetes. Diabetic Medicine. 2017;34(12):1742-6. doi: 10.1111/dme.13531. PubMed PMID: 126244815. Language: English. Entry Date: 20171120. Revision Date: 20181203. Publication Type: Article.

6178. Breuker C, Macioce V, Mura T, Castet-Nicolas A, Audurier Y, Boegner C, et al. Medication errors at hospital admission and discharge: risk factors and impact of medication reconciliation process to improve healthcare. Journal of Patient Safety. 2021;17(7):e645-e52.

6179. Breuker C, Teasdale J, Mallet L, Ricard G, Turcotte J-P, Gosselin S, et al. Communication between hospitals, Family Medicine Groups and community pharmacists during transitions of care interventions. Research in Social and Administrative Pharmacy. 2022;18(8):3290-6. doi: https://doi.org/10.1016/j.sapharm.2021.09.006.

6180. Breuker C, Teasdale J, Mallet L, Ricard G, Turcotte JP, Gosselin S, et al. Communication between hospitals, Family Medicine Groups and community pharmacists during transitions of care interventions. Research in Social and Administrative Pharmacy. 2022;18(8):3290-6. doi: 10.1016/j.sapharm.2021.09.006.

6181. Brewer A, Hanna C, Eckmann L, Schadler A, Divine H. Patient awareness, willingness, and barriers to point-of-care hepatitis C screening in community pharmacy. Journal of the American Pharmacists Association. 2018;58(4, Supplement):S69-S72.e1. doi: https://doi.org/10.1016/j.japh.2018.04.031.

6182. Brewer R, Daunis C, Ebaady S, Wilton L, Chrestman S, Mukherjee S, et al. Implementation of a Socio-structural Demonstration Project to Improve HIV Outcomes Among Young Black Men in the Deep South. Journal of Racial and Ethnic Health Disparities. 2019;6(4):775-89. doi: 10.1007/s40615-019-00576-x. PubMed PMID: WOS:000477609500014.

6183. Brewster JM, Ashley MJ, Laurier C, Dioso R, Charles Victor J, Ferrence R, et al. On the front line of smoking cessation: Pharmacists' practices and self-perception. Canadian Pharmaceutical Journal. 2005;138(3):32-8. doi: 10.1177/171516350513800308.

6184. Brewster S. The Role of Community Pharmacy in Supporting People with Diabetes Who Have a History of Repeated Non-Attendance at Healthcare Appointments. 2023. PubMed PMID: PQDT:85383188.

6185. Brewster S, Holt R, Portlock J, Price H. The role of community pharmacists and their position in the delivery of diabetes care: an update for medical professionals. Postgraduate Medical Journal. 2020;96(1138):473-9. PubMed PMID: 32217748.

6186. Brey EM, Campanile MF, Lederman NG, editors. Evaluation of a nine year summer undergraduate research program in biomedical engineering. ASEE Annual Conference and Exposition, Conference Proceedings; 2015.

6187. Brey Z, Mash R, Goliath C, Roman D. Home delivery of medication during Coronavirus disease 2019, Cape Town, South Africa: Short report. African Journal of Primary Health Care and Family Medicine.

2020;12(1):1-4. doi: 10.4102/PHCFM.V12I1.2449.

6188. Brguljan PM. The regulation of clinical chemistry in Slovenia. Biochemia Medica. 2011;21(1):7-11.

6189. Brhlikova P, Deivanayagam TA, Babar Z-U-D, Osorio-de-Castro CGS, Caetano R, Pollock AM. Essential medicines concept and health technology assessment approaches to prioritising medicines: selection versus incorporation. Journal of Pharmaceutical Policy & Practice. 2023;16(1):1-6. doi: 10.1186/s40545-023-00595-4. PubMed PMID: 164900199.

6190. Bria CRM. Development of asymmetrical flow field-flow fractionation for the characterization of proteins, protein aggregation, and nanoparticles [Ph.D.]. United States -- Colorado: Colorado School of Mines; 2016.

6191. Brice BF. Understanding customer expectations of community pharmacy services [Ph.D.]. United States -- Florida: University of Florida; 2000.

6192. Briceland LL, Lesar TS, Fausel CA, Stein DS. Antimicrobial prescribing errors averted by pharmacists: why do errors occur? Journal of Infectious Disease Pharmacotherapy. 1999;3(4):1-17. PubMed PMID: 107001883. Language: English. Entry Date: 20010223. Revision Date: 20150711. Publication Type: Journal Article.

6193. Briceland LL, Lomaestro BM, Pasquarella MV, Preston SL. Pharmacy intervention program to minimize ciprofloxacin-cation drug interactions. Hospital pharmacy. 1995;30(12):1099-100+103-104. PubMed PMID: CN-00343244.

6194. Bridgeman MB, Rusay M, Afran J, Yeh DS, Sturgill MG. Impact of an interprofessional medication error workshop on healthcare student perceptions. Currents in Pharmacy Teaching and Learning. 2018;10(7):975-81. doi: https://doi.org/10.1016/j.cptl.2018.04.013.

6195. Bridgeman MB, Wilken LA. Essential Role of Pharmacists in Asthma Care and Management. Journal of Pharmacy Practice. 2021;34(1):149-62. doi: 10.1177/0897190020927274. PubMed PMID: WOS:000538108700001.

6196. Bridgeman PJ, Rynn KO. Medication reconciliation in the emergency department. American Journal of Health-System Pharmacy. 2008;65(24):2325-6. PubMed PMID: 19052273.

6197. Bridges D, Davidson RA, Soule Odegard P, Maki IV, Tomkowiak J. Interprofessional collaboration: three best practice models of interprofessional education. Medical education online. 2011;16(1):6035.

6198. Bridges SM, Chan LK, Chen JY, Tsang JPY, Ganotice FA. Learning environments for interprofessional education: A micro-ethnography of sociomaterial assemblages in team-based learning. Nurse Education Today. 2020;94:104569. doi: https://doi.org/10.1016/j.nedt.2020.104569.

6199. Bridy-Pappas AE, Margolis MB, Center KJ, Isaacman DJ. Streptococcus pneumoniae: Description of the pathogen, disease epidemiology, treatment, and prevention. Pharmacotherapy. 2005;25(9):1193-212. doi: 10.1592/phco.2005.25.9.1193.

6200. Brieger WR, Osamor PE, Salami KK, Oladepo O, Otusanya SA. Interactions between patent medicine vendors and customers in urban and rural Nigeria. Health Policy and Planning. 2004;19(3):177-82. doi: 10.1093/heapol/czh021. PubMed PMID: WOS:000220896400005.

6201. Briel M, Langewitz W, Tschudi P, Young J, Hugenschmidt C, Bucher HC. Communication training and antibiotic use in acute respiratory tract infections. A cluster randomised controlled trial in general practice.

Swiss medical weekly. 2006;136(15-16):241-7. doi: 10.4414/smw.2006.11342. PubMed PMID: CN-00556428.

6202. Brien JA. Clinical governance. Journal of Pharmacy Practice and Research. 2006;36(3):174. doi: 10.1002/j.2055-2335.2006.tb00600.x.

6203. Briesacher B, Corey R. Patient satisfaction with pharmaceutical services at independent and chain pharmacies. American Journal of Health-System Pharmacy. 1997;54(5):531-6. doi: 10.1093/ajhp/54.5.531.

6204. Briesacher B, Limcangco R, Simoni-Wastila L, Doshi J, Gurwitz J. Evaluation of nationally mandated drug use reviews to improve patient safety in nursing homes: a natural experiment. Journal of the American Geriatrics Society. 2005;53(6):991-6. doi: 10.1111/j.1532-5415.2005.53314.x. PubMed PMID: 106520498. Language: English. Entry Date: 20050930. Revision Date: 20200708. Publication Type: Journal Article.

6205. Briesacher B, Limcangco R, Simoni-Wastila L, Doshi J, Gurwitz JH. Evaluation of nationally mandated drug use reviews to improve patient safety in nursing homes: a natural experiment. Journal of the American Geriatrics Society. 2005;53(6):991-6. PubMed PMID: CN-00597792.

6206. Briesacher BA, Andrade SE, Yood RA, Kahler KH. Consequences of poor compliance with bisphosphonates. Bone. 2007;41(5):882-7. doi: https://doi.org/10.1016/j.bone.2007.07.009.

6207. Briggs AL. Pharmacists' increasing involvement in hepatitis C management and prevention. Journal of the American Pharmacists Association. 2018;58(1):5-6. doi: 10.1016/j.japh.2017.12.008.

6208. Briggs AL, Jackson TR, Bruce S, Shapiro NL. The development and performance validation of a tool to assess patient anticoagulation knowledge⋆. Research in Social and Administrative Pharmacy. 2005;1(1):40-59. doi: https://doi.org/10.1016/j.sapharm.2004.12.002.

6209. Briggs C, Guthrie D, Hyde K, Mackie I, Parker N, Popek M, et al. Guidelines for point-of-care testing: haematology. British journal of haematology. 2008;142(6):904-15.

6210. Briggs C, Guthrie D, Hyde K, Mackie I, Parker N, Popek M, et al. Guidelines for point-of-care testing: Haematology. British Journal of Haematology. 2008;142(6):904-15. doi: 10.1111/j.1365-2141.2008.07274.x.

6211. Briggs CH. Problems of the Manufacturing Pharmacist Directly Resulting From War Conditions**Read before Scientific Section A. Ph. A., Chicago meeting, 1918. The Journal of the American Pharmaceutical Association (1912). 1918;7(12):1047-9. doi: https://doi.org/10.1002/jps.3080071210.

6212. Briggs MA, Kalolella A, Bruxvoort K, Wiegand R, Lopez G, Festo C, et al. Prevalence of Malaria Parasitemia and Purchase of Artemisinin-Based Combination Therapies (ACTs) among Drug Shop Clients in Two Regions in Tanzania with ACT Subsidies. Plos One. 2014;9(4). doi: 10.1371/journal.pone.0094074. PubMed PMID: WOS:000336970400018.

6213. Briggs S, Pearce R, Dilworth S, Higgins I, Hullick C, Attia J. Clinical pharmacist review: a randomised controlled trial. Emergency medicine Australasia. 2015;27(5):419-26. doi: 10.1111/1742-6723.12451. PubMed PMID: CN-01786740.

6214. Bright D, Worley M, Porter BL. Patient perceptions of pharmacogenomic testing in the community pharmacy setting. Research in Social and Administrative Pharmacy. 2021;17(4):744-9. doi: https://doi.org/10.1016/j.sapharm.2020.06.022.

6215. Bright DR, DiPietro Mager NA. Preconception care and contraception services: Opportunities for community pharmacists. JACCP Journal of the American College of Clinical Pharmacy. 2019;2(4):414-22.

doi: 10.1002/jac5.1152.

6216. Bright DR, Greco AJ, Langerveld A, Kisor DF. Clinical implementation of personalized medicine in community pharmacies: Considerations for clinicians. Personalized Medicine. 2017;14(6):471-5. doi: 10.2217/pme-2017-0050.

6217. Bright DR, Kisor DF, Smith A, Conaway M, Yu M. Implementation of a pharmacogenetic management service for postmyocardial infarction care in a community pharmacy. Personalized Medicine. 2015;12(4):319-25. doi: 10.2217/PME.15.7.

6218. Bright DR, Klepser ME, Murry L, Klepser DG. Pharmacist-Provided Pharmacogenetic Point-of-Care Testing Consultation Service: A Time and Motion Study. Journal of Pharmacy Technology. 2018;34(4):139-43. doi: 10.1177/8755122518756651. PubMed PMID: WOS:000438590000001.

6219. Bright E, Pearcy R, Abrams P. Automatic evaluation of ultrasonography-estimated bladder weight and bladder wall thickness in community-dwelling men with presumably normal bladder function. Bju International. 2012;109(7):1044-9. doi: 10.1111/j.1464-410X.2011.10507.x. PubMed PMID: WOS:000301437800017.

6220. Bright KL. The traveling tonic: Tradition, commodity, and the body in Unani (Greco-Arab) medicine in India [Ph.D.]. United States -- California: University of California, Santa Cruz; 1998.

6221. Brijnath B. Pills, pluralism, risk and citizenship: Theorising e-pharmacies. BioSocieties. 2012;7(3):294-307. doi: 10.1057/biosoc.2012.11.

6222. Brijnath B, Antoniades J, Adams J. Investigating Patient Perspectives on Medical Returns and Buying Medicines Online in Two Communities in Melbourne, Australia: Results from a Qualitative Study. Patient. 2015;8(2):229-38. doi: 10.1007/s40271-014-0082-z.

6223. Brill JL. A Company's Response to Culture Change in a Time of Reorganization [D.B.A.]. United States -- Minnesota: Walden University; 2013.

6224. Brindal E, Hendrie GA, Taylor P, Freyne J, Noakes M. Cohort Analysis of a 24-Week Randomized Controlled Trial to Assess the Efficacy of a Novel, Partial Meal Replacement Program Targeting Weight Loss and Risk Factor Reduction in Overweight/Obese Adults. Nutrients. 2016;8(5). doi: 10.3390/nu8050265. PubMed PMID: CN-01263877.

6225. Brinker A, Pamer C, Beitz J. Changes in Ciprofloxacin Utilization as Shown in a Large Pharmacy Claims Database: Effects of Proximity to Criminal Anthrax Exposure in October 2001. Journal of the American Pharmacists Association. 2003;43(3):375-8. doi: https://doi.org/10.1331/154434503321831085.

6226. Brinkman DJ, Tichelaar J, Schutte T, Benemei S, Böttiger Y, Chamontin B, et al. Essential competencies in prescribing: a first European cross-sectional study among 895 final-year medical students. Clinical Pharmacology & Therapeutics. 2017;101(2):281-9.

6227. Brion CMP. Interaction between Valsartan and Repaglinide mediated for hepatic transporters OATPs (Organic Anion Transporting Polypeptides): report of a case. Pharmaceutical Care Espana. 2019;21(3):179-85. PubMed PMID: WOS:000471794500003.

6228. Brion F, Prot-Labarthe S, Bourdon O, Masseron S, Awaïda L, Fontan J-E. Accidents médicamenteux en pédiatrie. Comment les limiter et les prévenir ? Bulletin de l'Académie Nationale de Médecine. 2005;189(8):1711-9. doi: https://doi.org/10.1016/S0001-4079(19)33428-4.

6229. Brion F, Prot-Labarthe S, Bourdon O, Masseron S, Awaida L, Fontan JE. [Pediatric medication errors and their prevention]. Bulletin de l Academie Nationale de Medecine. 2005;189(8):1711-9; discussion 9. PubMed PMID: 16737096.

6230. Briquet L. Comprendre les mécanismes de la violence pour recueillir la parole des victimes au comptoir. Actualités Pharmaceutiques. 2020;59(601):20-3. doi: https://doi.org/10.1016/j.actpha.2020.10.018.

6231. Briskin EA, Lusasi A, Lam F, Silumbe R, Mganga M, Rutizibwa R, et al. IMPACT OF TRAINING AND SUPPORTIVE INTERVENTIONS ON CASE MANAGEMENT AND REPORTING IN PRIVATE DRUG SHOPS IN TANZANIA. American Journal of Tropical Medicine and Hygiene. 2019;101:615-6. PubMed PMID: WOS:000507364505115.

6232. Brisseau L, Chiveri A, Lebel D, Bussières J-F. A pilot study of bar codes in a Canadian Hospital. Canadian Journal of Hospital Pharmacy. 2011;64(4):257-61. PubMed PMID: 108167666. Language: English. Entry Date: 20120420. Revision Date: 20150712. Publication Type: Journal Article.

6233. Britain RPSoG. Medicines, ethics and practice 39: the professional guide for pharmacists: Pharmaceutical Press; 2015.

6234. Brito AM, Simoes AM, Alcobia A, Alves da Costa F. Optimising patient safety using pharmaceutical intervention in domiciliary hospitalization. International Journal of Clinical Pharmacy. 2017;39(5):980-4. PubMed PMID: 28840436.

6235. Brits H, Botha A, Niksch L, Terblanché R, Venter K, Joubert G. Illegible handwriting and other prescription errors on prescriptions at National District Hospital, Bloemfontein. South African Family Practice. 2017;59(1):52-5. doi: 10.1080/20786190.2016.1254932. PubMed PMID: 121890722. Language: English. Entry Date: 20170322. Revision Date: 20190603. Publication Type: Article.

6236. Brits H, Botha A, Niksch L, Venter K, Terblanché R, Joubert G. Illegible handwriting and other prescription errors on prescriptions at National District Hospital, Bloemfontein. Professional Nursing Today. 2017;21(2):53-6.

6237. Britt NS, Khader K, He T, Willson TM, Effiong A, Timbrook TT, et al. Examining the clinical impact of rapid multiplex polymerase chain reaction-based diagnostic testing for bloodstream infections in a national cohort of the Veterans Health Administration. Pharmacotherapy: The Journal of Human Pharmacology and Drug Therapy. 2023;43(1):24-34.

6238. Britt RB, Hashem MG, Bryan WE, Kothapalli R, Brown JN. Economic Outcomes Associated with a Pharmacist-Adjudicated Formulary Consult Service in a Veterans Affairs Medical Center. Journal of Managed Care & Specialty Pharmacy. 2016;22(9):1051-6. doi: 10.18553/jmcp.2016.22.9.1051. PubMed PMID: WOS:000382901700006.

6239. Brittain K, Shirley DW, DeClue A, Kolo SJ, Worrall C. Longitudinal assessment of pharmacy student well-being using the well-being index and 5 gears assessment. American Journal of Pharmaceutical Education. 2023;87(7):100086. doi: https://doi.org/10.1016/j.ajpe.2023.100086.

6240. Brittain KL, Kuhn CH. Use of community pharmacy-provided diabetes services to aid physicians in the National Committee for Quality Assurance recognition program. Journal of the American Pharmacists Association: JAPhA. 2009;49(2):209-11. PubMed PMID: 19289347.

6241. Britten N, Brant S, Cairns A, Hall WW, Jones I, Salisbury C, et al. Continued prescribing of inappropriate drugs in general practice. Journal of Clinical Pharmacy & Therapeutics. 1995;20(4):199-205.

PubMed PMID: 8557783.

6242. Brixner D, Mittal M, Rubin DT, Mease P, Liu HH, Davis M, et al. Participation in an innovative patient support program reduces prescription abandonment for adalimumab-treated patients in a commercial population. Patient Preference and Adherence. 2019;13:1545-56. doi: 10.2147/ppa.S215037. PubMed PMID: WOS:000487423900001.

6243. Brixner D, Mittal M, Rubin DT, Mease P, Liu HH, Davis M, et al. Participation in an innovative patient support program reduces prescription abandonment for adalimumab-treated patients in a commercial population. Patient Preference and Adherence. 2019;13:1545-56. doi: 10.2147/PPA.S215037.

6244. Broach V, Montovano M, Drill E, Leitao MM, Abu-Rustum NR, Gardner GJ, et al. Cost-effectiveness analysis of conventional laparoscopic versus robotically assisted laparoscopic adnexal surgery for benign indications performed by gynecologic oncologists. Gynecologic oncology. 2015;137:47. doi: 10.1016/j.ygyno.2015.01.111. PubMed PMID: CN-01080432.

6245. Broad B. An exploratory study of pharmacy graduate preparedness for preregistration training [Ph.D.]. Wales: Cardiff University (United Kingdom); 2018.

6246. Broad J, Mason K, Guyton M, Lettner B, Matelski J, Powis J. Peer outreach point-of-care testing as a bridge to hepatitis C care for people who inject drugs in Toronto, Canada. International Journal of Drug Policy. 2020;80. doi: 10.1016/j.drugpo.2020.102755. PubMed PMID: WOS:000543450100017.

6247. Broccolo BM, Petersen BW. Final HIPAA privacy rules: "How do we get started?". Journal of Health Care Finance. 2001;27(4):7-23.

6248. Brochot C, Marchand S, Couet W, Gelman A, Bois FY. Extension of the Isobolographic Approach to Interactions Studies Between More than Two Drugs: Illustration with the Convulsant Interaction between Pefloxacin, Norfloxacin, and Theophylline in Rats. Journal of Pharmaceutical Sciences. 2004;93(3):553-62. doi: https://doi.org/10.1002/jps.10574.

6249. Brock KA, Casper KA, Green TR, Pedersen CA. Documentation of patient care services in a community pharmacy setting. Journal of the American Pharmacists Association. 2006;46(3):378-84. doi: 10.1331/154434506777069642.

6250. Brock KA, Doucette WR. Collaborative working relationships between pharmacists and physicians: An exploratory study. Journal of the American Pharmacists Association. 2004;44(3):358-65. doi: 10.1331/154434504323063995.

6251. Brock TP, Franklin BD. Differences in pharmacy terminology and practice between the United Kingdom and the United States. American Journal of Health-System Pharmacy. 2007;64(14):1541-6.

6252. Brock-Utne JG, Macario A, Dillingham MF, Fanton GS. Postoperative epidural injection of saline can shorten postanesthesia care unit time for knee arthroscopy patients. Regional anesthesia and pain medicine. 1998;23(3):247-51. PubMed PMID: CN-00685030.

6253. Brodaty H, Aerts L, Harrison F, Jessop T, Cations M, Chenoweth L, et al. Antipsychotic Deprescription for Older Adults in Long-term Care: The HALT Study. Journal of the American Medical Directors Association. 2018;19(7):592-600.e7. doi: https://doi.org/10.1016/j.jamda.2018.05.002.

6254. Brodie MJ, Elder AT, Kwan P. Epilepsy in later life. The Lancet Neurology. 2009;8(11):1019-30.

6255. Broedel-Zaugg K, Shaffer V, Mawer M, Sullivan DL. Frequency and severity of sexual harassment in

pharmacy practice in Ohio. Journal of the American Pharmaceutical Association (Washington,DC : 1996). 1999;39(5):677-82. doi: 10.1016/S1086-5802(15)30352-1.

6256. Brogan AP, Hogue SL, Vekaria RM, Reynolds I, Coukell A. Understanding payer perspectives on value in the use of pharmaceuticals in the United States. Journal of Managed Care and Specialty Pharmacy. 2019;25(12):1319-27. doi: 10.18553/jmcp.2019.25.12.1319.

6257. Brokaar EJ, Visser LE, van den Bos F, Portielje JEA. Medication optimization in older adults with advanced cancer and a limited life expectancy: A prospective observational study. Journal of Geriatric Oncology. 2023;14(8):101606. doi: https://doi.org/10.1016/j.jgo.2023.101606.

6258. Broman P, Tokolahi E, Wilson OWA, Haggie M, Andersen P, Brownie S. Patient Outcomes from Student-Run Health Services: An Integrative Review. Journal of Multidisciplinary Healthcare. 2022;15:641-65. doi: 10.2147/jmdh.S348411. PubMed PMID: WOS:000779842500006.

6259. Bronger J, editor Pharmacy's value towards prevention. Australian Journal of Pharmacy; 2003.

6260. Bronkhorst E, Schellack N, Gous AGS. Contextualising the Perceptions of Pharmacists Practicing Clinical Pharmacy in South Africa—Do We Practice what We Preach? Frontiers in Pharmacology. 2021;12. doi: 10.3389/fphar.2021.734654.

6261. Bronkhorst E, Schellack N, Gous AGS. Contextualising the Perceptions of Pharmacists Practicing Clinical Pharmacy in South Africa-Do We Practice what We Preach? Frontiers in Pharmacology. 2021;12. doi: 10.3389/fphar.2021.734654. PubMed PMID: WOS:000730997900001.

6262. Bronsoler Nurko A. Essays on Healthcare Delivery innovation: The role ofInformation and Communication Technology [Ph.D.]. United States -- Massachusetts: Massachusetts Institute of Technology; 2022.

6263. Brook O, van Hout H, Nieuwenhuyse H, Heerdink E. Impact of coaching by community pharmacists on drug attitude of depressive primary care patients and acceptability to patients; a randomized controlled trial. European neuropsychopharmacology. 2003;13(1):1-9. doi: 10.1016/s0924-977x(02)00074-3. PubMed PMID: CN-00412213.

6264. Brook OH, van Hout H, Stalman W, Nieuwenhuyse H, Bakker B, Heerdink E, et al. A pharmacy-based coaching program to improve adherence to antidepressant treatment among primary care patients. Psychiatric services (Washington, DC). 2005;56(4):487-9. doi: 10.1176/appi.ps.56.4.487. PubMed PMID: CN-00521350.

6265. Brook OH, Van Hout HP, Nieuwenhuysea H, De Haan M. Effects of coaching by community pharmacists on psychological symptoms of antidepressant users; a randomised controlled trial. European neuropsychopharmacology. 2003;13(5):347-54. doi: 10.1016/s0924-977x(03)00031-2. PubMed PMID: CN-00450393.

6266. Brook OH, van Hout HP, Stalman WA, de Haan M. Nontricyclic antidepressants: predictors of nonadherence. Journal of clinical psychopharmacology. 2006;26(6):643-7. doi: 10.1097/01.jcp.0000246217.34024.53. PubMed PMID: CN-00573762.

6267. Brook OH, Van Hout HPJ, Stalman WAB, De Haan M. Nontricyclic antidepressants: Predictors of nonadherence. Journal of Clinical Psychopharmacology. 2006;26(6):643-7.

6268. Brook OH, van Hout HPJ, Stalman WAB, de Haan M. Nontricyclic antidepressants - Predictors of nonadherence. Journal of Clinical Psychopharmacology. 2006;26(6):643-7. doi: 10.1097/01.jcp.0000246217.34024.53. PubMed PMID: WOS:000242256500015.

6269. Brooke BS, Griffin CL, Glotzbach JP, Horns JJ, Patel S, Kraiss LW. Predictors of Adherence to Anti-Impulse Therapy Among Patients Treated for Acute Type-B Aortic Dissections. Annals of vascular surgery. 2021;02. PubMed PMID: 634975635.

6270. Brooker C. Frequency of near-miss dispensing errors revealed. Pharmacy News. 2011:9-. PubMed PMID: 67518068. Language: English. Entry Date: 20111207. Revision Date: 20111208. Publication Type: Article.

6271. Brooker C. Pay attention when dispensing: UK study. Pharmacy News. 2011:6-. PubMed PMID: 58559406. Language: English. Entry Date: 20110303. Revision Date: 20110304. Publication Type: Article.

6272. Brooker C. Profile. Innovator both here & abroad. Pharmacy News. 2011:13-. PubMed PMID: 59675940. Language: English. Entry Date: 20110408. Revision Date: 20110412. Publication Type: Article.

6273. Brooker C. What's in a NAME? Australian Journal of Pharmacy. 2018;99(1173):28-31.

6274. Brooker C. Gord. Australian Journal of Pharmacy. 2018;99(1170):32-4.

6275. Brookes K, Scott M, McConnell J. The benefits of a hospital based community services liaison pharmacist. Pharmacy World and Science. 2000;22:33-8.

6276. Brookes K, Scott MG, McConnell JB. The benefits of a hospital based community services liaison pharmacist. Pharmacy World and Science. 2000;22(2):33-8. doi: 10.1023/A:1008713304892.

6277. Brookhart MA, Avorn J, Polinksi JM, Brown TV, Mogun H, Solomon DH. The medical license number accurately identifies the prescribing physician in a large pharmacy claims database. Medical Care. 2007;45(9):907-10. doi: 10.1097/MLR.0b013e3180616c67. PubMed PMID: WOS:000249415800014.

6278. Brookhart MA, Wang P, Solomon DH, Schneeweiss S. Evaluating short-term drug effects using a physician-specific prescribing preference as an instrumental variable. Epidemiology (Cambridge, Mass). 2006;17(3):268.

6279. Brookhyser J, Kinzner C, Pahre S. A case study of two successful pregnancies in a patient with end-stage renal disease. Journal of Renal Nutrition. 1996;6(1):26-33. doi: https://doi.org/10.1016/S1051-2276(96)90105-3.

6280. Brooks M. No link between PPIs and dementia in new study. Internal Medicine News. 2023:138-. PubMed PMID: 167354467.

6281. Brooksbank JA, Faulkenberg KD, Tang WW, Martyn T. Novel strategies to improve prescription of guideline-directed medical therapy in heart failure. Current Treatment Options in Cardiovascular Medicine. 2023;25(5):93-110.

6282. Broom A, Broom J, Kirby E, Scambler G. The path of least resistance? Jurisdictions, responsibility and professional asymmetries in pharmacists' accounts of antibiotic decisions in hospitals. Social Science & Medicine. 2015;146:95-103. doi: https://doi.org/10.1016/j.socscimed.2015.10.037.

6283. Broom J, Broom A, Kenny K, Chittem M. Antimicrobial overuse in India: A symptom of broader societal issues including resource limitations and financial pressures. Global Public Health. 2021;16(7):1079-87. doi: 10.1080/17441692.2020.1839930. PubMed PMID: 150797724. Language: English. Entry Date: 20210617. Revision Date: 20220926. Publication Type: Article.

6284. Brophy A, Bente J, Sobolewski K, DeStefano D, Costello J. Impact of Expansion of PGY1 Pharmacy

Residency Program From 2 to 4 Residents. Journal of Pharmacy Practice. 2020. doi: 10.1177/0897190020912934.

6285. Brophy A, Bente J, Sobolewski K, DeStefano D, Costello J. Impact of Expansion of PGY1 Pharmacy Residency Program From 2 to 4 Residents. Journal of Pharmacy Practice. 2021;34(5):761-5. doi: 10.1177/0897190020912934. PubMed PMID: WOS:000523009900001.

6286. Brophy L, Williams A, Berman EJ, Keleti D, Michael KE, Shepherd M, et al. Collaborative DTM reduces hospitalization and healthcare costs in patients with diabetes treated with polypharmacy. American Journal of Managed Care. 2014;20(3):e72-81. PubMed PMID: 24773329.

6287. Brosius WA. Consequences of misdiagnosing allergic rhinitis. American Journal for Nurse Practitioners. 2005;9(4):35-44. PubMed PMID: 106477130. Language: English. Entry Date: 20050708. Revision Date: 20150818. Publication Type: Journal Article.

6288. Brothers EB, Hay-Mccutcheon MJ, Hughes PJ, Friend ML. Audiology, Medicine, and Pharmacy Interprofessional Preliminary Interviews and Discussions: Improving Hearing Health Care in Rural Alabama. American Journal of Audiology. 2022;31(3):656-68. doi: 10.1044/2022_AJA-21-00272.

6289. Brott T, Broderick J, Kothari R, Odonoghue M, Barsan W, Tomsick T, et al. A systems approach to immediate evaluation and management of hyperacute stroke - Experience at eight centers and implications for community practice and patient care. Stroke. 1997;28(8):1530-40. doi: 10.1161/01.Str.28.8.1530. PubMed PMID: WOS:A1997XQ01300002.

6290. Brouker ME, Gallagher K, Larrat EP, Dufresne RL. Patient compliance and blood pressure control on a nuclear-powered aircraft carrier: impact of a pharmacy officer. Military medicine. 2000;165(2):106-10. PubMed PMID: CN-00276012.

6291. Broulette J, Yu H, Pyenson B, Iwasaki K, Sato R. The incidence rate and economic burden of community-acquired pneumonia in a working-age population. American Health and Drug Benefits. 2013;6(8):494-503.

6292. Broussard DL. Public health in pharmacy: Improving vitamin D status in the U.S. population. Journal of the American Pharmacists Association. 2013;53(2):206-9. doi: https://doi.org/10.1331/JAPhA.2013.12106.

6293. Broussard K. P010Healthcare at the margins: The role of healthcare providers in self-managed abortion near the us-mexico border. Contraception. 2022;116:76. doi: https://doi.org/10.1016/j.contraception.2022.09.035.

6294. Brouwer J, Risselada AJ, de Wit M, Lubberts J, Westerhuis H, Doornbos B, et al. Lithium surveillance by community pharmacists and physicians in ambulatory patients: a retrospective cohort study. International Journal of Clinical Pharmacy. 2022;44(4):975-84. doi: 10.1007/s11096-022-01420-9. PubMed PMID: WOS:000824250200001.

6295. Brouwer JMJL, Risselada AJ, de Wit M, Lubberts J, Westerhuis H, Doornbos B, et al. Lithium surveillance by community pharmacists and physicians in ambulatory patients: a retrospective cohort study. International Journal of Clinical Pharmacy. 2022;44(4):975-84. doi: 10.1007/s11096-022-01420-9.

6296. Brown AF, Gross AG, Gutierrez PR, Jiang L, Shapiro MF, Mangione CM. Income-related differences in the use of evidence-based therapies in older persons with diabetes mellitus in for-profit managed care. Journal of the American Geriatrics Society. 2003;51(5):665-70. doi: 10.1034/j.1600-0579.2003.00211.x.

6297. Brown AF, Gross AG, Gutierrez PR, Jiang LH, Shapiro MF, Mangione CM. Income-related differences

in the use of evidence-based therapies in older persons with diabetes mellitus in for-profit managed care. Journal of the American Geriatrics Society. 2003;51(5):665-70. doi: 10.1034/j.1600-0579.2003.00211.x. PubMed PMID: WOS:000182577900011.

6298. Brown AN, Ward-Panckhurst L, Cooper G. Factors affecting learning and teaching for medicines supply management training in Pacific Island Countries--a realist review. Rural and remote health. 2013;13(2):2327.

6299. Brown CA, Bailey JH, Lee J, Garrett PK, Rudman WJ. The pharmacist-physician relationship in the detection of ambulatory medication errors. American Journal of the Medical Sciences. 2006;331(1):22-4. PubMed PMID: 16415659.

6300. Brown CA, Bailey JH, Rudman WJ, Lee J, Garrett PK. The Pharmacist-Physician Relationship in the Detection of Ambulatory Medication Errors. The American Journal of the Medical Sciences. 2006;331(1):22-4. doi: https://doi.org/10.1097/00000441-200601000-00007.

6301. Brown CL, Mulcaster HL, Triffitt KL, Sittig DF, Ash JS, Reygate K, et al. A systematic review of the types and causes of prescribing errors generated from using computerized provider order entry systems in primary and secondary care. Journal of the American Medical Informatics Association. 2017;24(2):432-40.

6302. Brown CM. Use of Alternative Therapies and Their Impact on Compliance: Perceptions of Community Pharmacists in Texas. Journal of the American Pharmaceutical Association (1996). 1998;38(5):603-8. doi: https://doi.org/10.1016/S1086-5802(16)30374-6.

6303. Brown CM, Barner JC, Shah S. Community Pharmacists' Actions When Patients Use Complementary and Alternative Therapies with Medications. Journal of the American Pharmacists Association. 2005;45(1):41-7. doi: https://doi.org/10.1331/1544345052843011.

6304. Brown CM, Cantu R, Corbell Z, Roberts K. Attitudes and interests of pharmacists regarding independent pharmacy ownership. Journal of the American Pharmacists Association. 2007;47(2):174-80. doi: 10.1331/D525-0970-21X5-8UU7.

6305. Brown CM, Green AL. Expectations of patients with diabetes for pharmaceutical care activities in community pharmacy settings. Journal of Social and Administrative Pharmacy. 2000;17(4):219-23.

6306. Brown CM, Pena A, Resendiz K. Pharmacists' actions when patients use complementary and alternative medicine with medications: A look at Texas–Mexico border cities. Journal of the American Pharmacists Association. 2011;51(5):619-22. doi: https://doi.org/10.1331/JAPhA.2011.10021.

6307. Brown CM, Pena A, Resendiz K. Pharmacists' actions when patients use complementary and alternative medicine with medications: A look at Texas-Mexico border cities. Journal of the American Pharmacists Association. 2011;51(5):619-22. doi: 10.1331/JAPhA.2011.10021.

6308. Brown D. The paradox of pharmacy: A profession's house divided. Journal of the American Pharmacists Association: JAPhA. 2012;52(6):e139-43. PubMed PMID: 23143300.

6309. Brown D, Portlock J, Rutter P. Review of services provided by pharmacies that promote healthy living. International Journal of Clinical Pharmacy. 2012;34(3):399-409. doi: 10.1007/s11096-012-9634-2.

6310. Brown D, Portlock J, Rutter P, Nazar Z. From community pharmacy to healthy living pharmacy: Positive early experiences from Portsmouth, England. Research in Social & Administrative Pharmacy. 2014;10(1):72-87. doi: 10.1016/j.sapharm.2013.04.014. PubMed PMID: WOS:000328478600005.

6311. Brown D, Sautreau A, Soobiah T, Ali M, Ahmed M, Hussain S. Course-related extra curricular activities of M. Pharm undergraduate students at the University of Portsmouth. Pharmacy Education. 2005;5(2):143-56. doi: 10.1080/15602210500193318.

6312. Brown EE, Kumar S, Rajji TK, Pollock BG, Mulsant BH. Anticipating and mitigating the impact of the COVID-19 pandemic on Alzheimer's disease and related dementias. The American Journal of Geriatric Psychiatry. 2020;28(7):712-21.

6313. Brown EL, Raue PJ, Mlodzianowski AE, Meyers BS, Greenberg RL, Bruce ML. Transition to home care: Quality of mental health, pharmacy, and medical history information. International Journal of Psychiatry in Medicine. 2006;36(3):339-49. doi: 10.2190/6N5P-5CXH-L750-A8HV.

6314. Brown GD, Hunter WG, Hesson A, Davis JK, Kirby C, Barnett JA, et al., editors. Discussing out-of-pocket expenses during clinical appointments: An observational study of patient-psychiatrist interactions. Psychiatric Services; 2017.

6315. Brown GD, Hunter WG, Hesson A, Davis K, Kirby C, Barnett JA, et al. Discussing Out-of-Pocket Expenses During Clinical Appointments: An Observational Study of Patient-Psychiatrist Interactions. Psychiatric Services. 2017;68(6):610-7. doi: 10.1176/appi.ps.201600275. PubMed PMID: WOS:000402490700014.

6316. Brown GFC. Factors that facilitate or inhibit interdisciplinary collaboration within a professional bureaucracy [Ed.D.]. United States -- Arkansas: University of Arkansas; 1995.

6317. Brown JA, Roufogalis BD, Williamson M. Complementary medicines: Hospital pharmacists' attitude, knowledge and information seeking behaviour. Journal of Pharmacy Practice and Research. 2009;39(4):281-5. doi: 10.1002/j.2055-2335.2009.tb00475.x.

6318. Brown JB, Delea TE, Nichols GA, Edelsberg J, Elmer PJ, Oster G. Use of oral Antithrombotic agents among health maintenance organization members with atherosclerotic cardiovascular disease. Archives of Internal Medicine. 2002;162(2):193-9. doi: 10.1001/archinte.162.2.193. PubMed PMID: WOS:000173453100009.

6319. Brown JD, Delcher PC, Bae J, Roussos-Ross D, Goodin AJ. OB/GYN perceptions of prescription drug monitoring programs as a primary prevention tool for neonatal abstinence syndrome. Research in Social and Administrative Pharmacy. 2020;16(12):1789-91. doi: https://doi.org/10.1016/j.sapharm.2020.03.010.

6320. Brown JD, Vouri SM, Manini TM. Survey-reported medication changes among older adults during the SARS-CoV-2 (COVID-19) pandemic. Research in Social and Administrative Pharmacy. 2021;17(8):1478-82. doi: https://doi.org/10.1016/j.sapharm.2020.11.005.

6321. Brown JH, Torres HP, Maddi RD, Williams JL, Dibaj SS, Liu D, et al. Cancer Patients' Perceived Difficulties Filling Opioid Prescriptions After Receiving Outpatient Supportive Care. Journal of Pain and Symptom Management. 2020;60(5):915-22. doi: 10.1016/j.jpainsymman.2020.06.015. PubMed PMID: WOS:000579391100012.

6322. Brown JL, Cochran G, Bryan MA, Charron E, Winhusen TJ. Associations between elevated depressive symptoms and substance use, prescription opioid misuse, overdose history, pain, and general health among community pharmacy patients prescribed opioids. Substance Abuse. 2022;43(1):1110-5. doi: 10.1080/08897077.2022.2060450.

6323. Brown JN, Barnes CL, Beasley B, Cisneros R, Pound M, Herring C. Effect of pharmacists on medication errors in an emergency department. American Journal of Health-System Pharmacy.

2008;65(4):330-3. doi: 10.2146/ajhp070391. PubMed PMID: 105860074. Language: English. Entry Date: 20080314. Revision Date: 20200708. Publication Type: Journal Article.

6324. Brown JN, Britnell SR, Stivers AP, Cruz JL. Medication Safety in Clinical Trials: Role of the Pharmacist in Optimizing Practice, Collaboration, and Education to Reduce Errors. Yale Journal of Biology & Medicine. 2017;90(1):125-33. PubMed PMID: 28356900.

6325. Brown JR, Hirsch JA, Judd SE, Hurvitz PM, Howard VJ, Safford M, et al. The Association of Neighborhood Medical Facilities with Aging in Place and Risk of Incident Myocardial Infarction. Journal of Aging and Health. 2021;33(3-4):227-36. doi: 10.1177/0898264320975228.

6326. Brown JT, McGonagle E, Seifert R, Speedie M, Jacobson PA. Addressing disparities in pharmacogenomics through rural and underserved workforce education. Frontiers in Genetics. 2023;13. doi: 10.3389/fgene.2022.1082985. PubMed PMID: WOS:000920748400001.

6327. Brown JVE, Walton N, Meader N, Todd A, Webster LAD, Steele R, et al. Pharmacy-based management for depression in adults. Cochrane Database of Systematic Reviews. 2019;2019(12). doi: 10.1002/14651858.CD013299.pub2.

6328. Brown JVE, Walton N, Meader N, Todd A, Webster LAD, Steele R, et al. Pharmacy-based management for depression in adults (Review). Cochrane Database of Systematic Reviews. 2019;(12). doi: 10.1002/14651858.CD013299.pub2. PubMed PMID: WOS:000505243400010.

6329. Brown JVE, Walton N, Meader N, Todd A, Webster LAD, Steele R, et al. Pharmacy-based management for depression in adults. Cochrane Database of Systematic Reviews. 2019;(12). doi: 10.1002/14651858.CD013299.pub2. PubMed PMID: CD013299.

6330. Brown K, Cunningham D, Allan R, Lee K. GP trainee prescribing assessment – a qualitative evaluation of the involvement of pharmacists. Education for Primary Care. 2022;33(4):221-8. doi: 10.1080/14739879.2021.2021380. PubMed PMID: 158065726. Language: English. Entry Date: 20220728. Revision Date: 20220729. Publication Type: Article.

6331. Brown KA, Khanafer N, Daneman N, Fisman DN. Meta-analysis of antibiotics and the risk of community-associated Clostridium difficile infection. Antimicrobial agents and chemotherapy. 2013;57(5):2326-32.

6332. Brown KE, Hertig JB. Determining Current Insulin Pen Use Practices and Errors in the Inpatient Setting. The Joint Commission Journal on Quality and Patient Safety. 2016;42(12):568-AP7. doi: https://doi.org/10.1016/S1553-7250(16)30109-X.

6333. Brown KN, Bergsbaken J, Reichard JS. Medication safety pharmacy technician in a large, tertiary care, community hospital. American Journal of Health-System Pharmacy. 2016;73(4):188-91. doi: 10.2146/ajhp150098. PubMed PMID: 113282239. Language: English. Entry Date: 20160226. Revision Date: 20160302. Publication Type: Article.

6334. Brown LG, Prather C. A Diabetes Screening and Educational Event in Rural Alabama. American Journal of Nursing. 2020;120(4):61-3. doi: 10.1097/01.NAJ.0000660060.90674.5e.

6335. Brown LM. Measurement of pharmacy quality metrics at the pharmacy level should be our goal. Journal of the American Pharmacists Association: JAPhA. 2009;49(2):153. PubMed PMID: 19289340.

6336. Brown LM, Rashrash ME, Schommer JC. The certainty in consumers' willingness to accept pharmacist-provided medication therapy management services. Journal of the American Pharmacists

Association. 2017;57(2):211-6. doi: https://doi.org/10.1016/j.japh.2017.02.012.

6337. Brown M, Fleming L, Sewell MJ, Ross LA. Evaluation of the impact of a residency in an independent pharmacy in rural Mississippi. Journal of the American Pharmacists Association. 2017;57(3):S243-S6. doi: 10.1016/j.japh.2017.03.004. PubMed PMID: WOS:000432472200010.

6338. Brown M, Frost R, Ko Y, Woosley R. Diagramming patients' views of root causes of adverse drug events in ambulatory care: an online tool for planning education and research. Patient Education & Counseling. 2006;62(3):302-15. doi: 10.1016/j.pec.2006.02.007. PubMed PMID: 106215608. Language: English. Entry Date: 20070119. Revision Date: 20200624. Publication Type: Journal Article.

6339. Brown M, Tran C, Dadiomov D. Lowering barriers to naloxone access through a student-led harm reduction program. Journal of the American Pharmacists Association. 2022. doi: 10.1016/j.japh.2022.10.030.

6340. Brown M, Tran C, Dadiomov D. Lowering barriers to naloxone access through a student-led harm reduction program. Journal of the American Pharmacists Association. 2023;63(1):349-55. doi: https://doi.org/10.1016/j.japh.2022.10.030.

6341. Brown M, Tran C, Dadiomov D. Lowering barriers to naloxone access through a student-led harm reduction program. Journal of the American Pharmacists Association. 2023;63(1):349-55. doi: https://doi.org/10.1016/j.japh.2022.10.030.

6342. Brown ME. A sociological perspective of pharmacy: (2) To take or not to take. Pharmaceutical Journal. 2001;267(7170):562-3.

6343. Brown ME. Violent Encounters: Mediatization, Socio-Medical Legitimation, TBI, and CTE-Lived Experiences of NFL Players, Military Veterans, and Their Caretakers [M.S.]. United States -- Massachusetts: Boston University; 2019.

6344. Brown ME, Bellaby P. Community pharmacy as a performance: A participant observer's account of a day in the life of a locum. International Journal of Pharmacy Practice. 2002;10(3):201-12. doi: 10.1111/j.2042-7174.2002.tb00609.x.

6345. Brown ME, Hoodless J. Prolonged use of nitrous oxide and oxygen mixture [4]. Pharmaceutical Journal. 2005;275(7361):163.

6346. Brown MJ, Sharma P, Bennett PN. Clinical pharmacology: Elsevier Health Sciences; 2012.

6347. Brown MT, Bussell J, Dutta S, Davis K, Strong S, Mathew S. Medication adherence: truth and consequences. The American journal of the medical sciences. 2016;351(4):387-99.

6348. Brown MT, Bussell JK, editors. Medication adherence: WHO cares? Mayo clinic proceedings; 2011: Elsevier.

6349. Brown NJ, Griffin MR, Ray WA, Meredith S, Beers MH, Marren J, et al. A model for improving medication use in home health care patients. Journal of the american pharmaceutical association (washington, DC : 1996). 1998;38(6):696-702. doi: 10.1016/s1086-5802(16)30390-4. PubMed PMID: CN-01720320.

6350. Brown NJ, Griffin MR, Ray WA, Meredith S, Beers MH, Marren J, et al. A Model for Improving Medication Use in Home Health Care Patients. Journal of the American Pharmaceutical Association (1996). 1998;38(6):696-702. doi: https://doi.org/10.1016/S1086-5802(16)30390-4.

6351. Brown P. P-1073 - An examination of current clinical performance with regards to safe lithium

prescribing and monitoring in our inpatient unit. European Psychiatry. 2012;27:1. doi: https://doi.org/10.1016/S0924-9338(12)75240-9.

6352. Brown P, Bluml BM, Kritzler R, Petryna M, Polley CM, Tietze KJ, et al. White paper on expanding the role of pharmacists in chronic obstructive pulmonary disease. Journal of the American Pharmacists Association. 2011;51(2):203-11. doi: 10.1331/JAPhA.2011.11513. PubMed PMID: WOS:000297752100013.

6353. Brown PJ. What should clinical training be about? Pharmaceutical Journal. 2008;281(7518):265.

6354. Brown RT, Borden KA, Wynne ME, Spunt AL, Clingerman SR. Compliance With Pharmacological and Cognitive Treatments for Attention Deficit Disorder. Journal of the American Academy of Child & Adolescent Psychiatry. 1987;26(4):521-6. doi: https://doi.org/10.1097/00004583-198707000-00010.

6355. Brown S, Henderson E, Sullivan C. The feasibility and acceptability of the provision of alcohol screening and brief advice in pharmacies for women accessing emergency contraception: An evaluation study. BMC Public Health. 2014;14(1). doi: 10.1186/1471-2458-14-1139.

6356. Brown SH, Lincoln MJ, Groen PJ, Kolodner RM. VistA—US department of veterans affairs national-scale HIS. International journal of medical informatics. 2003;69(2-3):135-56.

6357. Brown TJ. Let in the sunshine. Modern Steel Construction. 2006;46(9):24-6.

6358. Brown TJ, Todd A, O'Malley C, Moore HJ, Husband AK, Bambra C, et al. Community pharmacy-delivered interventions for public health priorities: a systematic review of interventions for alcohol reduction, smoking cessation and weight management, including meta-analysis for smoking cessation. BMJ Open. 2016;6(2):e009828. doi: 10.1136/bmjopen-2015-009828.

6359. Brown TJ, Todd A, O'Malley CL, Moore HJ, Husband AK, Bambra C, et al. Community pharmacy interventions for public health priorities: a systematic review of community pharmacy-delivered smoking, alcohol and weight management interventions. Public Health Research. 2016.

6360. Brown TL. Access to Psychotropic Medication and Choice of Method in a Suicide Attempt [Ph.D.]. United States -- Colorado: University of Colorado Denver, Anschutz Medical Campus; 2017.

6361. Brown TL, Milavetz G, Gaffney G, Spurgin A. Evaluating drugged driving: effects of exemplar pain and anxiety medications. Traffic injury prevention. 2018;19(sup1):S97-S103. doi: 10.1080/15389588.2017.1378814. PubMed PMID: CN-01703764.

6362. Brown W, Scott D, Friesner D, Schmitz T. Impact of telepharmacy services as a way to increase access to asthma care. Journal of Asthma. 2017;54(9):961-7. doi: 10.1080/02770903.2017.1281292.

6363. Brown WI, Cernusca D, Roehrich L. Evaluation of a Hybrid Training Module for Community Pharmacy Staff Providing Hypertension Medication Therapy Management. Journal of Pharmacy Practice. 2018;31(2):183-9. PubMed PMID: 28460560.

6364. Brown-Luthango M. Collective (in) efficacy, substance abuse and violence in "Freedom Park," Cape Town. Journal of Housing and the Built Environment. 2016;31(1):123-40. doi: 10.1007/s10901-015-9448-3. PubMed PMID: WOS:000372123200008.

6365. Browne C, Kingston C, Keane C. Falls prevention focused medication review by a pharmacist in an acute hospital: implications for future practice. International Journal of Clinical Pharmacy. 2014;36(5):969-75. PubMed PMID: 25108410.

6366. Browne GJ, Trieu L, Van Asperen P. Randomized, double-blind, placebo-controlled trial of intravenous salbutamol and nebulized ipratropium bromide in early management of severe acute asthma in children presenting to an emergency department. Critical care medicine. 2002;30(2):448-53. doi: 10.1097/00003246-200202000-00030. PubMed PMID: CN-00378670.

6367. Browne S, Kelly L, Geraghty AA, Reynolds CME, McBean L, McCallum K, et al. Healthcare professionals' perceptions of malnutrition management and oral nutritional supplement prescribing in the community: A qualitative study. Clinical Nutrition ESPEN. 2021;44:415-23. doi: https://doi.org/10.1016/j.clnesp.2021.04.024.

6368. Brownlee S. Overtreated: Why too much medicine is making us sicker and poorer: Bloomsbury Publishing USA; 2010.

6369. Brownlie K, Schneider C, Culliford R, Fox C, Boukouvalas A, Willan C, et al. Medication reconciliation by a pharmacy technician in a mental health assessment unit. International journal of clinical pharmacy. 2014;36:303-9.

6370. Brownsmith R. Evaluation of the Portsmouth medicines advice at home service. Pharmaceutical Journal. 2019;303(7931). doi: 10.1211/PJ.2019.20207348.

6371. Bruce JC, Cagle W. Medication error reduction in the children's hospital of Georgia. American Journal of the Medical Sciences. 2023;365(Supplement 1):S359. doi: https://dx.doi.org/10.1016/S0002-9629%2823%2900663-8. PubMed PMID: 2022348914.

6372. Bruce N, Pope D, Arana B, Shiels C, Romero C, Klein R, et al. Determinants of care seeking for children with pneumonia and diarrhea in guatemala: Implications for intervention strategies. American Journal of Public Health. 2014;104(4):647-57. doi: 10.2105/AJPH.2013.301658.

6373. Bruce R, Murdoch W, Kable A, Palazzi K, Hullick C, Pond D, et al. Evaluation of Carer Strain and Carer Coping with Medications for People with Dementia after Discharge: Results from the SMS Dementia Study. Healthcare. 2020;8(3):31. PubMed PMID: 32751986.

6374. Bruckdorfer T, Marder O, Albericio F. From production of peptides in milligram amounts for research to multi-tons quantities for drugs of the future. Current pharmaceutical biotechnology. 2004;5(1):29-43.

6375. Brudnak MA. Weight-loss drugs and supplements: are there safer alternatives? Medical Hypotheses. 2002;58(1):28-33. doi: https://doi.org/10.1054/mehy.2001.1444.

6376. Brueckl MN, Carey GB, Tofade T. Creating Global Experiences With Local Impact for Pharmacy and Medical Students. Annals of Global Health. 2016;82(6):1043-7. doi: 10.1016/j.aogh.2016.11.002.

6377. Bruene D, Bauer M. Improving Pain Management through Creative Partnerships. Pain Management Nursing. 2023;24(2):241. doi: https://doi.org/10.1016/j.pmn.2023.02.029.

6378. Bruggmann C, Adjedj J, Sardy S, Muller O, Voirol P, Sadeghipour F. Effects of the Interactive Web-Based Video "Mon Coeur, Mon BASIC" on Drug Adherence of Patients With Myocardial Infarction: randomized Controlled Trial. Journal of medical Internet research. 2021;23(8):e21938. doi: 10.2196/21938. PubMed PMID: CN-02324220.

6379. Bruhn H, Bond CM, Elliott AM, Hannaford PC, Lee AJ, McNamee P. Pharmacist-led management of chronic pain in primary care: results from a randomised controlled exploratory trial. BMJ open. 2013;3:2013. PubMed PMID: CN-01412626.

6380. Bruhn H, Bond CM, Elliott AM, Hannaford PC, Lee AJ, McNamee P, et al. Pharmacist-led management of chronic pain in primary care: results from a randomised controlled exploratory trial. BMJ open. 2013;3(4). doi: 10.1136/bmjopen-2012-002361. PubMed PMID: CN-00906909.

6381. Bruhn H, Watson M, Blyth A, Bond CM. Pharmacist-led management of chronic pain in primary care: patient expectations, attitudes and concerns. International journal of pharmacy practice. 2010;18:23-4. doi: 10.1111/j.2042-7174.2010.00023.x. PubMed PMID: CN-01772945.

6382. Brühwiler L, Gresch SJ, Schwappach DLB. Implementation status of safety measures to prevent errors with non-oncologic methotrexate: surveys in community and hospital pharmacies. International Journal of Clinical Pharmacy. 2023;45(3):739-47. doi: 10.1007/s11096-023-01567-z. PubMed PMID: WOS:000960615500002.

6383. Brühwiler LD, Beeler PE, Böni F, Giger R, Wiedemeier PG, Hersberger KE, et al. A RCT evaluating a pragmatic in-hospital service to increase the quality of discharge prescriptions. International journal for quality in health care : journal of the international society for quality in health care. 2019;31(8):G74-G80. doi: 10.1093/intqhc/mzz043. PubMed PMID: CN-02002969.

6384. Bruhwiler LD, Beeler PE, Boni F, Wiedemeier PG, Hersberger KE, Lutters M. A pragmatic in-hospital service to reduce workload of community pharmacists after discharge: a randomised controlled trial. International journal of clinical pharmacy. 2018;40(1):207-. doi: 10.1007/s11096-017-0565-9. PubMed PMID: CN-01468266.

6385. Bruhwiler LD, Gresch SJ, Schwappach DLB. Implementation status of safety measures to prevent errors with non-oncologic methotrexate: surveys in community and hospital pharmacies. International Journal of Clinical Pharmacy. 2023;45(3):739-47. doi: https://dx.doi.org/10.1007/s11096-023-01567-z. PubMed PMID: 2022342894.

6386. Brühwiler LD, Hersberger KE, Lutters M. Hospital discharge: What are the problems, information needs and objectives of community pharmacists? A mixed method approach. Pharmacy Practice. 2017;15(3). doi: 10.18549/PharmPract.2017.03.1046.

6387. Brühwiler LD, Schwappach DLB. Safe vincristine use in Switzerland: Still a long way to go? Journal of Oncology Pharmacy Practice. 2020;26(1):51-9. doi: 10.1177/1078155219835598. PubMed PMID: 139959772. Language: English. Entry Date: 20191207. Revision Date: 20200308. Publication Type: Article.

6388. Bruins AP. Atmospheric-pressure-ionization mass spectrometry: II. Applications in pharmacy, biochemistry and general chemistry. TrAC Trends in Analytical Chemistry. 1994;13(2):81-90. doi: https://doi.org/10.1016/0165-9936(94)85069-0.

6389. Brummel A, Carlson AM. Comprehensive Medication Management and Medication Adherence for Chronic Conditions. Journal of Managed Care & Specialty Pharmacy. 2016;22(1):56-62. doi: 10.18553/jmcp.2016.22.1.56. PubMed PMID: WOS:000376801900007.

6390. Brummel A, Lustig A, Westrich K, Evans MA, Plank GS, Penso J, et al. Best practices: improving patient outcomes and costs in an ACO through comprehensive medication therapy management. Journal of Managed Care & Specialty Pharmacy. 2014;20(12):1152-8. PubMed PMID: 25597053.

6391. Brummel AR, Soliman AM, Carlson AM, de Oliveira DR. Optimal diabetes care outcomes following face-to-face medication therapy management services. Population Health Management. 2013;16(1):28-34. PubMed PMID: 23113628.

6392. Brummett CM, England C, Evans-Shields J, Kong AM, Lew CR, Henriques C, et al. Health Care

Burden Associated with Outpatient Opioid Use Following Inpatient or Outpatient Surgery. Journal of Managed Care & Specialty Pharmacy. 2019;25(9):973-+. doi: 10.18553/jmcp.2019.19055. PubMed PMID: WOS:000483684200003.

6393. Brummett CM, Evans-Shields J, England C, Kong AM, Lew CR, Henriques C, et al. Increased health care costs associated with new persistent opioid use after major surgery in opioid-naive patients. Journal of Managed Care & Specialty Pharmacy. 2021;27(6):760-71. doi: 10.18553/jmcp.2021.20507. PubMed PMID: WOS:000657629000008.

6394. Brummond PW, Chen DF, Churchill WW, Clark JS, Dillon KR, Dumitru D, et al. ASHP guidelines on preventing diversion of controlled substances. American Journal of Health-System Pharmacy. 2017;74(5):325-48.

6395. Brun M, Zerguit S, Moutel E, Le Falher B, Lemann F, Plassart F, et al. Écrasement des comprimés et ouverture de gélules : évaluation des pratiques professionnelles et des connaissances des soignants au sein d'un centre hospitalier général. Le Pharmacien Hospitalier et Clinicien. 2020;55(3):228-34. doi: https://doi.org/10.1016/j.phclin.2020.01.007.

6396. Bruneau J, Daniel M, Kestens Y, Zang G, Généreux M. Associations between HIV-related injection behaviour and distance to and patterns of utilisation of syringe-supply programmes. Journal of Epidemiology and Community Health. 2008;62(9):804-10. doi: 10.1136/jech.2007.064154.

6397. Brunelli L, Arnoldo L, Mazzilis G, d'Angelo M, Colautti L, Cojutti PG, et al. The knowledge and attitudes of pharmacists related to the use of dietary supplements: An observational study in northeastern Italy. Preventive Medicine Reports. 2022;30:101986. doi: https://doi.org/10.1016/j.pmedr.2022.101986.

6398. Brunet M, Van Gelder T, Åsberg A, Haufroid V, Hesselink DA, Langman L, et al. Therapeutic drug monitoring of tacrolimus-personalized therapy: second consensus report. Therapeutic drug monitoring. 2019;41(3):261-307.

6399. Brunette MF, Cotes RO, De Nesnera A, McHugo G, Dzebisashvili N, Xie H, et al. Use of academic detailing with audit and feedback to improve antipsychotic pharmacotherapy. Psychiatric Services. 2018;69(9):1021-8. doi: 10.1176/appi.ps.201700536.

6400. Brunette MF, Cotes RO, de Nesnera A, McHugo G, Dzebisashvili N, Xie HY, et al. Use of Academic Detailing With Audit and Feedback to Improve Antipsychotic Pharmacotherapy. Psychiatric Services. 2018;69(9):1021-8. doi: 10.1176/appi.ps.201700536. PubMed PMID: WOS:000443271000015.

6401. Brunette MF, Dzebisashvili N, Xie H, Akerman S, Ferron JC, Bartels S. Expanding Cessation Pharmacotherapy Via Videoconference Educational Outreach to Prescribers. Nicotine & tobacco research. 2015;17(8):960-7. doi: 10.1093/ntr/ntv006. PubMed PMID: CN-01107209.

6402. Brunette MF, Dzebisashvili N, Xie H, Akerman S, Pratt S, Ferron JC, et al. Using technology to improve access, quality and outcomes: tobacco treatment in mental health settings. Journal of mental health policy and economics. 2015;18:S4-S5. PubMed PMID: CN-01142726.

6403. Brunetti ND, Molinari G, Acquistapace F, Zimotti T, Parati G, Indolfi C, et al. 2019 Italian Society of Cardiology Census on telemedicine in cardiovascular disease: A report from the working group on telecardiology and informatics. Open Heart. 2020;7(1). doi: 10.1136/openhrt-2019-001157.

6404. Brunetti O. the career of the Florentine stonemason Francesco Cerroti (1680-1768), an important figure within the artistic culture of the Settecento in Florence and Rome. Mitteilungen Des Kunsthistorischen Institutes in Florenz. 2020;62(2-3):237-65. PubMed PMID: WOS:000649404000005.

6405. Brungger B, Blozik E. Hospital readmission risk prediction based on claims data available at admission: a pilot study in Switzerland. Bmj Open. 2019;9(6). doi: 10.1136/bmjopen-2018-028409. PubMed PMID: WOS:000562117100168.

6406. Brunk D. FDA adds safety-related information to its dermal filler webpage. Internal Medicine News. 2023:1-. PubMed PMID: 169821180.

6407. Bruno B, Capelle L, Denis V, Duval O, Selmouni S, Villate A, et al. Interactions médicamenteuses et incompatibilités physico-chimiques en phase aiguë post-allogreffe : quelle influence des médicaments de support ? Recommandations de la Société francophone de greffe de moelle et de thérapie cellulaire (SFGM-TC). Bulletin du Cancer. 2022. doi: https://doi.org/10.1016/j.bulcan.2022.02.004.

6408. Bruno B, Capelle L, Denis V, Duval O, Selmouni S, Villate A, et al. Interactions médicamenteuses et incompatibilités physico-chimiques en phase aiguë post-allogreffe : quelle influence des médicaments de support ? Recommandations de la SFGM-TC. Bulletin du Cancer. 2023;110(2, Supplement):S88-S96. doi: https://doi.org/10.1016/j.bulcan.2022.02.004.

6409. Bruno BA, Guirguis K, Rofaiel D, Yu CH. Is Sociodemographic Status Associated with Empathic Communication and Decision Quality in Diabetes Care? Journal of general internal medicine. 2022;37(12):3013-9. doi: 10.1007/s11606-021-07230-5. PubMed PMID: CN-02463436.

6410. Bruno C, Saberi P. Pharmacists as providers of HIV pre-exposure prophylaxis. International Journal of Clinical Pharmacy. 2012;34(6):803-6. doi: 10.1007/s11096-012-9709-0.

6411. Bruno CB, Ip E, Shah B, Linn WD. A mnemonic for pharmacy students to use in pharmacotherapy assessment. American journal of pharmaceutical education. 2012;76(1):16. doi: 10.5688/ajpe76116. PubMed PMID: CN-00860063.

6412. Bruppacher R. Drug administration research — The contribution of pharmacoepidemiology to pharmaceutical monitoring. Pharmaceutica Acta Helvetiae. 1996;71(5):359-61. doi: https://doi.org/10.1016/S0031-6865(96)00033-7.

6413. Bruppacher R, editor Drug administration research - The contribution of pharmacoepidemiology to pharmaceutical monitoring. Pharmaceutica Acta Helvetiae; 1996.

6414. Brusa P, Allais G, Bussone G, Rolando S, Giaccone M, Aguggia M, et al. Migraine attacks in the pharmacy: a survey in Piedmont, Italy. Neurological Sciences. 2014;35:A5-A9. doi: 10.1007/s10072-014-1733-5. PubMed PMID: WOS:000336744400002.

6415. Brusa P, Allais G, Rolando S, Baratta F, Giaccone M, Bussone G, et al. Migraine attacks in the pharmacy: a gender subanalysis on treatment preferences. Neurological Sciences. 2015;36:S93-S5. doi: 10.1007/s10072-015-2156-7. PubMed PMID: WOS:000367834700021.

6416. Brusa P, Parente M, Allais G, Rolando S, Costa G, Gnavi R, et al. Community pharmacies as epidemiological sentinels of headache: first experience in Italy. Neurological Sciences. 2017;38:15-20. doi: 10.1007/s10072-017-2908-7.

6417. Brusciano L, Limongelli P, del Genio G, Di Stazio C, Rossetti G, Sansone S, et al. Short-term outcomes after rehabilitation treatment in patients selected by a novel rehabilitation score system (Brusciano score) with or without previous stapled transanal rectal resection (STARR) for rectal outlet obstruction. International Journal of Colorectal Disease. 2013;28(6):783-93. doi: 10.1007/s00384-012-1565-9. PubMed PMID: WOS:000319431600005.

6418. Brushwood DB. Pharmacy department not liable for use of contraindicated drug in surgical suite. American Journal of Health-System Pharmacy. 2012;69(4):349-51. PubMed PMID: 22302260.

6419. Brushwood DB, Kimberlin CA. RESEARCH: Media Coverage of Controlled Substance Diversion Through Theft or Loss. Journal of the American Pharmacists Association. 2004;44(4):439-44. doi: https://doi.org/10.1331/1544345041475779.

6420. Brushwood DB, Mullan K. Corporate pharmacy's responsibility for a dispensing error. American Journal of Health-System Pharmacy. 1996;53(6):668-70. PubMed PMID: 8800974.

6421. Brusig E, Davies W. PSTAC survey in 2006 was instrumental in obtaining permanent (category I) CPT codes for MTM services performed by pharmacists. Journal of Managed Care Pharmacy. 2008;14(1):86-7. PubMed PMID: 18240887.

6422. Bruthans J. The past and current state of the Czech outpatient electronic prescription (eRecept). International Journal of Medical Informatics. 2019;123:49-53. doi: 10.1016/j.ijmedinf.2019.01.003. PubMed PMID: 134153331. Language: English. Entry Date: 20190815. Revision Date: 20210513. Publication Type: journal article. Journal Subset: Biomedical.

6423. Bruxvoort K. Evaluation of patient adherence to artemether-lumefantrine obtained from public and private drug outlets in tanzania [Ph.D.]. England: University of London, London School of Hygiene and Tropical Medicine (United Kingdom); 2015.

6424. Bruxvoort K, Festo C, Kalolella A, Cairns M, Lyaruu P, Kenani M, et al. Cluster Randomized Trial of Text Message Reminders to Retail Staff in Tanzanian Drug Shops Dispensing Artemether-Lumefantrine: Effect on Dispenser Knowledge and Patient Adherence. American Journal of Tropical Medicine and Hygiene. 2014;91(4):844-53. doi: 10.4269/ajtmh.14-0126. PubMed PMID: WOS:000342957600034.

6425. Bruzelius E, Cerdá M, Davis CS, Jent V, Wheeler-Martin K, Mauro CM, et al. Naloxone expansion is not associated with increases in adolescent heroin use and injection drug use: Evidence from 44 US states. International Journal of Drug Policy. 2023;114:103980. doi: https://doi.org/10.1016/j.drugpo.2023.103980.

6426. Bryan AR, Liu Y, Kuehl PG. Advocating zoster vaccination in a community pharmacy through use of personal selling. Journal of the American Pharmacists Association. 2013;53(1):70-7. doi: 10.1331/JAPhA.2013.11097.

6427. Bryan AR, Liu YF, Kuehl PG. Advocating zoster vaccination in a community pharmacy through use of personal selling. Journal of the American Pharmacists Association. 2013;53(1):70-7. doi: 10.1331/JAPhA.2013.11097. PubMed PMID: WOS:000315937700015.

6428. Bryan MA, Charron E, Adeoye-Olatunde O, Brown J, Ghitza U, Winhusen TJ, et al. Concomitant Cannabis Misuse and Associations with Depression, Pain and Substance Misuse among Patients Prescribed Opioids. Pharmacy. 2021;9(3). doi: 10.3390/pharmacy9030134. PubMed PMID: WOS:000701648400001.

6429. Bryan NP, Byrne L, Hastie KJ, Anderson JB, Moore KTH, Chapple CR. A pilot study for a randomized controlled trial comparing the efficacy, safety and cost-effectiveness of surgical treatments of the prostate. Bju International. 1999;83(3):249-53. PubMed PMID: WOS:000079776400008.

6430. Bryant A. Parenting Blog Coverage of the Autism-Vaccine Controversy [M.A.]. United States -- New York: Syracuse University; 2012.

6431. Bryant AD. Impact of FDA Advisories on Smoking Cessation for Veterans with Chronic Obstructive Pulmonary Disease [Master's]. United States -- Washington: University of Washington; 2020.

6432. Bryant J. A study of young people who inject drugs: an opportunity to decrease high risk injecting by improving knowledge about hepatitis C prevention. Vulnerable Children and Youth Studies. 2014;9(2):104-13. doi: 10.1080/17450128.2013.855348.

6433. Bryant J, Brener L, Hull P, Treloar C. Needle sharing in regular sexual relationships: An examination of serodiscordance, drug using practices, and the gendered character of injecting. Drug and Alcohol Dependence. 2010;107(2-3):182-7. doi: 10.1016/j.drugalcdep.2009.10.007. PubMed PMID: WOS:000275732700009.

6434. Bryant J, Hopwood M. Secondary exchange of sterile injecting equipment in a high distribution environment: A mixed method analysis in south east Sydney, Australia. International Journal of Drug Policy. 2009;20(4):324-8. doi: https://doi.org/10.1016/j.drugpo.2008.06.006.

6435. Bryant J, Prohmmo A. Use of drug sellers by old people in northeast Thailand. Asia-Pacific journal of public health / Asia-Pacific Academic Consortium for Public Health. 2001;13(2):91-5. doi: 10.1177/101053950101300206.

6436. Bryant J, Treloar C. Risk practices and other characteristics of injecting drug users who obtain injecting equipment from pharmacies and personal networks. International Journal of Drug Policy. 2006;17(5):418-24. doi: https://doi.org/10.1016/j.drugpo.2006.07.004.

6437. Bryant L, Bang C, Chew C, Baik SH, Wiseman D. Adequacy of inhaler technique used by people with asthma or chronic obstructive pulmonary disease. Journal of primary health care. 2013;5(3):191-8.

6438. Bryant L, Coster G, McCormick R. General practitioner perceptions of clinical medication reviews undertaken by community pharmacists. Journal of Primary Health Care. 2010;2(3):225-33. doi: 10.1071/hc10225.

6439. Bryant L, Maney J, Martini N. Changing perspectives of the role of community pharmacists: 1998 - 2012. Journal of Primary Health Care. 2017;9(1):34-46. doi: 10.1071/hc16032. PubMed PMID: WOS:000404613300008.

6440. Bryant LJ, Coster G, Gamble GD, McCormick RN. The General Practitioner-Pharmacist Collaboration (GPPC) study: a randomised controlled trial of clinical medication reviews in community pharmacy. International journal of pharmacy practice. 2011;19(2):94-105. doi: 10.1111/j.2042-7174.2010.00079.x. PubMed PMID: CN-00778595.

6441. Bryant LJM, Coster G, Gamble GD, McCormick RN. General practitioners' and pharmacists' perceptions of the role of community pharmacists in delivering clinical services. Research in Social & Administrative Pharmacy. 2009;5(4):347-62. doi: 10.1016/j.sapharm.2009.01.002. PubMed PMID: WOS:000208739000007.

6442. Bryant LJM, Coster G, Gamble GD, McCormick RN. The General Practitioner-Pharmacist Collaboration (GPPC) study: a randomised controlled trial of clinical medication reviews in community pharmacy. International journal of pharmacy practice. 2011;19(2):94-105. doi: 10.1111/j.2042-7174.2010.00079.x. PubMed PMID: CN-02128455.

6443. Bryant PA. Antimicrobial stewardship resources and activities for children in tertiary hospitals in Australasia: a comprehensive survey. Medical Journal of Australia. 2015;202(3):134-8. doi: 10.5694/mja13.00143. PubMed PMID: 109776418. Corporate Author: Australasian Stewardship of Antimicrobials in Paediatrics group. Language: English. Entry Date: 20150923. Revision Date: 20200708. Publication Type: journal article.

6444. Bryant PA, Australasian Stewardship of Antimicrobials in Paediatrics g. Antimicrobial stewardship resources and activities for children in tertiary hospitals in Australasia: a comprehensive survey. Medical Journal of Australia. 2015;202(3):134-8. PubMed PMID: 25669475.

6445. Bryant R, Chaar B, Schneider C. Differing clinical pharmacy service models: Quantitative and qualitative analysis of nurse perceptions of support from pharmacists. International Journal of Nursing Studies. 2018;86:90-8. doi: https://doi.org/10.1016/j.ijnurstu.2018.04.003.

6446. Bryant-Comstock L, Stender M, Devercelli G. Health care utilization and costs among privately insured patients with bipolar I disorder. Bipolar Disorders. 2002;4(6):398-405. doi: 10.1034/j.1399-5618.2002.01148.x.

6447. Bryant-Stephens T, Williams Y, Minto N, Apter AJ, Localio R, Shults J, et al. Adaptation of school based asthma therapy in a large urban cohort. American journal of respiratory and critical care medicine. 2020;201(1). PubMed PMID: CN-02139325.

6448. Bryant-Waugh R, Turner H, East P. Over-the-counter laxatives and eating disorders - a survey of pharmacists' and other retailers' views and practice. Pharmaceutical Journal. 2005;275(7358):87-91. PubMed PMID: WOS:000231461600045.

6449. Bryant-Wimp J, Pannell D. Algorithmic evidence-based phenotype screening and collaborative approach to attacking symptoms of nonadherence in patients with bleeding disorders. Haemophilia. 2016;22:5-. doi: 10.1111/hae.12980. PubMed PMID: CN-01409421.

6450. Bryce J. What's new with medicines? Australian Nursing Journal. 2012;19(8):21-. PubMed PMID: 72365154. Language: English. Entry Date: 20120314. Revision Date: 20120606. Publication Type: Book Review.

6451. Brydges S, Rennick-Egglestone S, Anderson C. Men's views of antidepressant treatment for depression, and their implications for community pharmacy practice. Research in Social and Administrative Pharmacy. 2020;16(8):1041-9. doi: 10.1016/j.sapharm.2019.10.016.

6452. Bryk A, Koontz S, Mayor J, Betcher J, Tombleson R, Bookout R, et al. Characterization of collaborative practice agreements held by hematopoietic stem cell transplant pharmacists. Journal of Oncology Pharmacy Practice. 2019;25(3):558-66. PubMed PMID: 29207938.

6453. Bryndová L, Hroboň P, Tulejová H. The 2018 risk-adjustment reform in the Czech Republic: Introducing Pharmacy-based Cost Groups and strengthening reinsurance. Health Policy. 2019;123(8):700-5. doi: https://doi.org/10.1016/j.healthpol.2019.05.017.

6454. Bryony D. Can simulation be used to reduce errors in health care delivery?: The hospital drug distribution system.

6455. Bryson CL, Au DH, Sun H, Williams EC, Kivlahan DR, Bradley KA. Alcohol screening scores and medication nonadherence. Annals of internal medicine. 2008;149(11):795-804. doi: 10.7326/0003-4819-149-11-200812020-00004. PubMed PMID: CN-00667379.

6456. Buabeng KO, Duwiejua M, Dodoo AN, Matowe LK, Enlund H. Self-reported use of anti-malarial drugs and health facility management of malaria in Ghana. Malaria Journal. 2007;6. doi: 10.1186/1475-2875-6-85.

6457. Buabeng KO, Matowe LK, Smith F, Duwiejua M, Enlund H. Knowledge of medicine outlets' staff and their practices for prevention and management of malaria in Ghana. Pharmacy World and Science.

2010;32(4):424-31. doi: 10.1007/s11096-010-9397-6.

6458. Buatois EM, Akunna AA, Bailey T, Coomer TN, Putnam WC, Hall RG, et al. Using the HyFlex model to deliver a capstone seminar course. Currents in Pharmacy Teaching and Learning. 2022;14(9):1109-15. doi: https://doi.org/10.1016/j.cptl.2022.07.037.

6459. Bubalo J, Clark RK, Jiing SS, Johnson NB, Miller KA, Clemens-Shipman CJ, et al. Medication adherence: Pharmacist perspective. Journal of the American Pharmacists Association. 2010;50(3):394-406. doi: 10.1331/JAPhA.2010.08180. PubMed PMID: WOS:000277802800013.

6460. Bubalo J, Warden BA, Wiegel JJ, Nishida T, Handel E, Svoboda LM, et al. Does applying technology throughout the medication use process improve patient safety with antineoplastics? Journal of Oncology Pharmacy Practice. 2014;20(6):445-60.

6461. Bubp JL, Park MA, Kapusnik-Uner J, Dang T, Matuszewski K, Ly D, et al. Successful deployment of drug-disease interaction clinical decision support across multiple Kaiser Permanente regions. Journal of the American Medical Informatics Association. 2019;26(10):905-10. doi: 10.1093/jamia/ocz020. PubMed PMID: 138837239. Language: English. Entry Date: 20210203. Revision Date: 20220124. Publication Type: journal article.

6462. Buccellato E, Biagi C, Melis M, Lategana R, Motola D, Vaccheri A. Use of antibacterial agents in Italian hospitals: a 2004 to 2011 drug utilization survey in the Emilia-Romagna region. Expert Review of Anti-Infective Therapy. 2014;12(3):383-92. doi: 10.1586/14787210.2014.884459. PubMed PMID: WOS:000335324300012.

6463. Bucci C, Jackevicius C, McFarlane K, Liu P. Pharmacist's contribution in a heart function clinic: patient perception and medication appropriateness. Canadian journal of cardiology. 2003;19(4):391-6. PubMed PMID: CN-00436789.

6464. Bucci C, Jackevicius C, McFarlene K, Liu P. Pharmacist's contribution in a heart function clinic: Patient perception and medication appropriateness. Canadian Journal of Cardiology. 2003;19(4):391-6.

6465. Buccino P, Savio E, Porcal W. Fully-automated radiosynthesis of the amyloid tracer [11C] PiB via direct [11C]CO2 fixation-reduction. EJNMMI Radiopharmacy and Chemistry. 2019;4(1). doi: 10.1186/s41181-019-0065-4.

6466. Bucek RA, Schnurer G, Ahmadi A, Maca TH, Meissl G, Minar E. A severe case of vascular thoracic outlet syndrome. Wiener Klinische Wochenschrift. 2000;112(22):973-7. PubMed PMID: WOS:000165860500005.

6467. Buchanan D, Shaw S, Teng W, Hiser P, Singer M. Neighborhood Differences in Patterns of Syringe Access, Use, and Discard among Injection Drug Users: Implications for HIV Outreach and Prevention Education. Journal of Urban Health. 2003;80(3):438-54. doi: 10.1093/jurban/jtg050.

6468. Buchanan EC. Computer simulation as a basis for pharmacy reengineering. Nursing Administration Quarterly. 2003;27(1):33-40. doi: 10.1097/00006216-200301000-00008. PubMed PMID: 106821455. Language: English. Entry Date: 20050425. Revision Date: 20200708. Publication Type: Journal Article.

6469. Buchanan J, Roope LSJ, Morrell L, Pouwels KB, Robotham JV, Abel L, et al. Preferences for Medical Consultations from Online Providers: Evidence from a Discrete Choice Experiment in the United Kingdom. Applied Health Economics and Health Policy. 2021;19(4):521-35. doi: 10.1007/s40258-021-00642-8. PubMed PMID: WOS:000625905100001.

6470. Buchanan R, Cooper K, Grellier L, Khakoo SI, Parkes J. The testing of people with any risk factor for hepatitis C in community pharmacies is cost-effective. Journal of Viral Hepatitis. 2020;27(1):36-44. doi: 10.1111/jvh.13207.

6471. Buchanan R, Hassan-Hicks P, Noble K, Grellier L, Parkes J, Khakoo SI. Integrating community pharmacy testing for hepatitis c with specialist care. Clinical Pharmacist. 2016;8(8). doi: 10.1211/CP.2016.20201549.

6472. Buchbinder R, Forbes A, Kobben F, Boyd I, Snow RM, McNeil JJ. Clinical features of tiaprofenic acid (surgam) associated cystitis and a study of risk factors for its development. Journal of Clinical Epidemiology. 2000;53(10):1013-9. doi: https://doi.org/10.1016/S0895-4356(00)00192-X.

6473. Buchegger S, Iglseder B, Alzner R, Kogler M, Rose O, Kutschar P, et al. Patient perspectives on, and effects of, medication management in geriatric fallers (the EMMA study): protocol for a mixed-methods pre-post study. BMJ open. 2023;13(2):e066666.

6474. Bucheit JD, Helsing H, Nadpara P, Virani SS, Dixon DL. Clinical pharmacist understanding of the 2013 American College of Cardiology/American Heart Association cholesterol guideline. Journal of Clinical Lipidology. 2018;12(2):367-74.e3. doi: https://doi.org/10.1016/j.jacl.2017.11.010.

6475. Buchheit J, Reddy P, Connors JM. Idarucizumab (Praxbind) Formulary Review. Critical pathways in cardiology 15 (3) (pp 77-81), 2016 Date of publication: 01 sep 2016. 2016. doi: 10.1097/HPC.0000000000000076. PubMed PMID: CN-01195084.

6476. Buchholz U, Jahn HJ, Brodhun B, Lehfeld AS, Lewandowsky MM, Reber F, et al. Source attribution of community-acquired cases of Legionnaires' disease-results from the German LeTriWa study; Berlin, 2016-2019. Plos One. 2020;15(11). doi: 10.1371/journal.pone.0241724. PubMed PMID: WOS:000593887000040.

6477. Buchinger SM. [One in the morning, none at noon, one in the evening]. Pflege Zeitschrift. 2012;65(10):604-6. PubMed PMID: 23098029.

6478. Buchmueller TC, Carey C. The effect of prescription drug monitoring programs on opioid utilization in Medicare. American Economic Journal: Economic Policy. 2018;10(1):77-112.

6479. Buchner DL, Awor P. A protocol for engaging unlicensed private drug shops in Rural Eastern Uganda for Integrated Community Case Management (ICCM) of malaria, pneumonia and diarrhoea in children under 5 years of age. BMJ Open. 2015;5(10). doi: 10.1136/bmjopen-2015-009133.

6480. Buchner DL, Kitutu FE, Cross DE, Nakamoga E, Awor P. A cross-sectional study to identify the distribution and characteristics of licensed and unlicensed private drug shops in rural Eastern Uganda to inform an iCCM intervention to improve health outcomes for children under five years. PLoS ONE. 2019;14(1). doi: 10.1371/journal.pone.0209641.

6481. Buchner DM, Cress ME, De Lateur BJ, Esselman PC, Margherita AJ, Price R, et al. The effect of strength and endurance training on gait, balance, fall risk, and health services use in community-living older adults. The Journals of Gerontology Series A: Biological Sciences and Medical Sciences. 1997;52(4):M218-M24.

6482. Buchs AE, Silverman BG. Incidence of malignancies in patients with diabetes mellitus and correlation with treatment modalities in a large Israeli health maintenance organization: a historical cohort study. Metabolism. 2011;60(10):1379-85. doi: https://doi.org/10.1016/j.metabol.2011.05.002.

6483. Buck J, Kaboli P, Gage BF, Cram P, Vaughan Sarrazin MS. Trends in antithrombotic therapy for atrial fibrillation: Data from the Veterans Health Administration Health System. American Heart Journal. 2016;179:186-91. doi: https://doi.org/10.1016/j.ahj.2016.03.029.

6484. Buckberg GD. Cardioplegia Solutions—Unproven Herbal Approach Versus Tested Scientific Study. Seminars in Thoracic and Cardiovascular Surgery. 2001;13(1):52-5. doi: https://doi.org/10.1053/stcs.2001.22737.

6485. Buckel WR, Hersh AL, Pavia AT, Jones PS, Owen-Smith AA, Stenehjem E. Antimicrobial stewardship knowledge, attitudes, and practices among health care professionals at small community hospitals. Hospital Pharmacy. 2016;51(2):149-57. doi: 10.1310/hpj5102-149.

6486. Buckingham P, Amos N, Hussainy SY, Mazza D. Scoping review of pharmacy-based initiatives for preventing unintended pregnancy: Protocol. BMJ Open. 2020;10(1). doi: 10.1136/bmjopen-2019-033002.

6487. Buckingham PLM, Hussainy S, Soon J, Norman WV, Bateson D, Mazza D. Improving access to quality contraceptive counselling in community pharmacy: examining the knowledge, attitudes and practices of community pharmacists in Australia. BMJ Sexual and Reproductive Health. 2022. doi: 10.1136/bmjsrh-2022-201623.

6488. Buckingham PLM, Hussainy S, Soon J, Norman WV, Bateson D, Mazza D. Improving access to quality contraceptive counselling in community pharmacy: Examining the knowledge, attitudes and practices of community pharmacists in Australia. BMJ Sexual and Reproductive Health. 2023;49(2):87-96. doi: 10.1136/bmjsrh-2022-201623.

6489. Buckle P, Clarkson P, Coleman R, Bound J, Ward J, Brown J. Systems mapping workshops and their role in understanding medication errors in healthcare. Applied ergonomics. 2010;41(5):645-56.

6490. Buckle P, Clarkson P, Coleman R, Ward J, Anderson J. Patient safety, systems design and ergonomics. Applied ergonomics. 2006;37(4):491-500.

6491. Buckley K, Karr S, Nisly SA, Kelley K. Evaluation of a mock interview session on residency interview skills. Currents in pharmacy teaching & learning. 2018;10(4):511-6. doi: 10.1016/j.cptl.2017.12.021. PubMed PMID: CN-01913572.

6492. Buckley L, Dixon D, Wohlford G, Wijesinghe D, Baker W, Van Tassell B. Intensive blood pressure control in patients with and without type 2 diabetes mellitus: an analysis of SPRINT and ACCORD BP. Pharmacotherapy. 2017;37(12):e143-e4. doi: 10.1002/phar.2052. PubMed PMID: CN-01449756.

6493. Buckley MS, Acquisto NM, Adams C, Brandt K, Ammar MA, Deshpande R, et al. Critical care pharmacy practice advancement recommendations on direct patient care activities: An opinion of the American College of Clinical Pharmacy Critical Care Practice and Research Network. JACCP Journal of the American College of Clinical Pharmacy. 2023;6(8):925-33. doi: https://dx.doi.org/10.1002/jac5.1816. PubMed PMID: 2023335814.

6494. Buckley MS, Agarwal SK, Lansburg JM, Kopp BJ, Erstad BL. Clinical Pharmacist-Led Impact on Inappropriate Albumin Utilization and Associated Costs in General Ward Patients. Annals of Pharmacotherapy. 2021;55(1):44-51. PubMed PMID: 32578446.

6495. Buckley MS, Erstad BL, Kopp BJ, Theodorou AA, Priestley G. Direct observation approach for detecting medication errors and adverse drug events in a pediatric intensive care unit. Pediatric Critical Care Medicine. 2007;8(2):145-52.

6496. Buckley MS, Harinstein LM, Clark KB, Smithburger PL, Eckhardt DJ, Alexander E, et al. Impact of a clinical pharmacy admission medication reconciliation program on medication errors in “high-risk” patients. Annals of Pharmacotherapy. 2013;47(12):1599-610.

6497. Buckley MS, Kane-Gill SL, Patel SA. Clinical and Economic Evaluation of an Evidence-Based Institutional Epoetin-Utilization Management Program. Clinical Therapeutics. 2013;35(3):294-302. doi: https://doi.org/10.1016/j.clinthera.2013.02.002.

6498. Buckley MS, Knutson KD, Agarwal SK, Lansburg JM, Wicks LM, Saggar RC, et al. Clinical Pharmacist-Led Impact on Inappropriate Albumin Use and Costs in the Critically Ill. Annals of Pharmacotherapy. 2020;54(2):105-12. doi: 10.1177/1060028019877471. PubMed PMID: 140824138. Language: English. Entry Date: 20200628. Revision Date: 20211108. Publication Type: journal article.

6499. Buckley MS, Park AS, Anderson CS, Barletta JF, Bikin DS, Gerkin RD, et al. Impact of a Clinical Pharmacist Stress Ulcer Prophylaxis Management Program on Inappropriate Use in Hospitalized Patients. The American Journal of Medicine. 2015;128(8):905-13. doi: https://doi.org/10.1016/j.amjmed.2015.02.014.

6500. Buckner TW, Hagan KA, Orvis E, Yang H, Sawyer EK, Li N. Healthcare resource utilization and cost burden of hemophilia B in the US. Research and practice in thrombosis and haemostasis. 2020;4(SUPPL 1):422-. doi: 10.1002/rth2.12393. PubMed PMID: CN-02213065.

6501. Bucsa C, Farcas A, Udrea M, Bojita M, Mogosan C, Leucuta D. Concordance of pharmacist versus patient responses regarding counselling in community pharmacy. Journal of Evaluation in Clinical Practice. 2022;28(4):558-65. doi: 10.1111/jep.13635.

6502. Bucsa CD, Cazacu I, Farcas AM. THE PREVALENCE OF POTENTIAL DRUG-DRUG INTERACTIONS IN THE THERAPY OF ROMANIAN COMMUNITY PHARMACY'S PATIENTS. Farmacia. 2012;60(4):510-6. PubMed PMID: WOS:000307493600006.

6503. Bucsa CD, Cazacu I, Farcas AM, Bojiţă M. The prevalence of potential drug-drug interactions in the therapy of Romanian community pharmacy's patients. Farmacia. 2012;60(4):510-6.

6504. Buczek L, Azar F, Bauzon J, Batra K, Murphy C, Wahi-Gururaj S. The Data Error Criteria (DEC) for retrospective studies: development and preliminary application. Journal of Investigative Medicine. 2023;71(4):448-54.

6505. Buczek L, Azar F, Bauzon J, Batra K, Murphy C, Wahi-Gururaj S. The Data Error Criteria (DEC) for retrospective studies: development and preliminary application. Journal of Investigative Medicine. 2023:10815589231151437.

6506. Buda V, Prelipcean A, Cristescu C, Roja A, Dalleur O, Andor M, et al. Prescription Habits Related to Chronic Pathologies of Elderly People in Primary Care in the Western Part of Romania: Current Practices, International Recommendations, and Future Perspectives Regarding the Overuse and Misuse of Medicines. International Journal of Environmental Research & Public Health [Electronic Resource]. 2021;18(13):01. PubMed PMID: 34280980.

6507. Budanov V, Aseeva I. Manipulative marketing technologies in new digital reality. Economic Annals-XXI. 2019;180(11-12):58-68. doi: 10.21003/EA.V180-07.

6508. Budd JM, Sievert M, Schultz TR. Phenomena of retraction: reasons for retraction and citations to the publications. Jama. 1998;280(3):296-7.

6509. Budi M, Focht J, Lake L, Fox G, Snyder M. The impact of a pharmacist-centered education module on

medicare part D plan performance measures. Journal of the american pharmacists association Conference: apha2016 Baltimore, MD united states Conference start: 20160304 Conference end: 20160307 Conference publication: (varpagings). 2016;56(3):e126-e7. doi: 10.1016/j.japh.2016.03.014. PubMed PMID: CN-01267566.

6510. Budiman T, Snodgrass K, Komatsu Chang A. Evaluation of Pharmacist Medication Education and Post-discharge Follow-up in Reducing Readmissions in Patients With ST-Segment Elevation Myocardial Infarction (STEMI). Annals of Pharmacotherapy. 2016;50(2):118-24. PubMed PMID: 26681443.

6511. Budnitz DS, Shehab N, Kegler SR, Richards CL. Medication use leading to emergency department visits for adverse drug events in older adults. Annals of internal medicine. 2007;147(11):755-65.

6512. Buehler SS, Madison B, Snyder SR, Derzon JH, Cornish NE, Saubolle MA, et al. Effectiveness of practices to increase timeliness of providing targeted therapy for inpatients with bloodstream infections: A laboratory medicine best practices systematic review and meta-analysis. Clinical Microbiology Reviews. 2015;29(1):59-103. doi: 10.1128/CMR.00053-14.

6513. Buehler SS, Madison B, Snyder SR, Derzon JH, Cornish NE, Saubolle MA, et al. Effectiveness of Practices To Increase Timeliness of Providing Targeted Therapy for Inpatients with Bloodstream Infections: a Laboratory Medicine Best Practices Systematic Review and Meta-analysis. Clinical Microbiology Reviews. 2016;29(1):59-103. doi: 10.1128/cmr.00053-14. PubMed PMID: WOS:000372251700003.

6514. Bueno FJP, del Pozo RBM, Jerez MAH. Study of the perception of the professionals involved in the process of administering medications to the elderly who live in residences from a community pharmacy. Farmaceuticos Comunitarios. 2019;11(4):49-55. doi: 10.5672/FC.2173-9218.(2019/Vol11).004.05. PubMed PMID: WOS:000504905600005.

6515. Buerhaus PI. Is hospital patient care becoming safer? A conversation with lucian leape. Health Affairs. 2007;26(6):w687-96. doi: 10.1377/hlthaff.26.6.w687. PubMed PMID: 105827703. Language: English. Entry Date: 20080307. Revision Date: 20200623. Publication Type: Journal Article.

6516. Buetow S, Henshaw J, Bryant L, O'Sullivan D. Medication timing errors for Parkinson's disease: perspectives held by caregivers and people with Parkinson's in new zealand. Parkinson’s Disease. 2010;2010.

6517. Buetow S, Kiata L, Liew T, Kenealy T, Dovey S, Elwyn G. Approaches to reducing the most important patient errors in primary health-care: patient and professional perspectives. Health & Social Care in the Community. 2010;18(3):296-303. PubMed PMID: 20141539.

6518. Bufarini C, Leoni S, Guglielmi S, Caimmi S, Bartolini G, Ciaffi V, et al. Centralised non-hazardous intravenous compounding: from theory to clinical practice. European journal of hospital pharmacy Conference: 22nd annual congress of the european association of hospital pharmacists, EAHP 2017 France. 2017;24:A219-A20. doi: 10.1136/ejhpharm-2017-000640.488. PubMed PMID: CN-01406482.

6519. Buff SM, Jenkins K, Kern D, Worrall C, Howell D, Martin K, et al. Interprofessional service-learning in a community setting: findings from a pilot study. Journal of Interprofessional Care. 2015;29(2):159-61. doi: 10.3109/13561820.2014.934956.

6520. Buggisch P, Petersen J, Wursthorn K, Bec M, Schwarzbard J. Effectiveness, safety and cost per sustained virological response in Hepatitis C patients treated with Sofosbuvir based therapies in clinical practice in Germany. Zeitschrift fur gastroenterologie Conference: viszeralmedizin 2015, 70 Jahrestagung der deutschen gesellschaft fur gastroenterologie, verdauungs- und stoffwechselkrankheiten mit sektion endoskopie - 9 Herbsttagung der deutschen gesellschaft fur allgemein- und viszeralchirurgie Germany. 2015;53(8) (no pagination). doi: 10.1055/s-0035-1559165. PubMed PMID: CN-01461979.

6521. Bugnon O, Hugentobler-Hampaï D, Berger J, Schneider MP. New roles for Community pharmacists in modern health care systems: A challenge for Pharmacy education and research. Chimia. 2012;66(5):304-7. doi: 10.2533/chimia.2012.304.

6522. Buhelaiga SMT, Abuhaleegah KS, Al Eisa EMA, Alkhars KM, Alsadah SAM, Buhligha LA, et al. SELF-MEDICATION AND ITS DETERMINANTS IN DIFFERENT AREAS OF SAUDI ARABIA: A CROSS SECTIONAL COMMUNITY-BASED STUDY. Indo American Journal of Pharmaceutical Sciences. 2019;6(1):1885-91. doi: 10.5281/zenodo.2548749. PubMed PMID: WOS:000457554400003.

6523. Buhl A, Augustine J, Taylor AM, Martin R, Warholak TL. Positive Medication Changes Resulting from Comprehensive and Noncomprehensive Medication Reviews in a Medicare Part D Population. Journal of Managed Care & Specialty Pharmacy. 2017;23(3):388-94. PubMed PMID: 28230451.

6524. Buhl C, Olsen NL, Nørgaard LS, Thomsen LA, Jacobsen R. Community Pharmacy Staff's Knowledge, Educational Needs, and Barriers Related to Counseling Cancer Patients and Cancer Survivors in Denmark. International Journal of Environmental Research and Public Health. 2023;20(3). doi: 10.3390/ijerph20032287.

6525. Buhrich N, Butchart A, Johnston S, Lauchlan R. Delivery of medication to psychiatric patients in community health services in New South Wales. Australian and New Zealand Journal of Psychiatry. 1996;30(4):523-30. doi: 10.3109/00048679609065027. PubMed PMID: WOS:A1996VF92400016.

6526. Bui QTH, Nguyen ATD. Effectiveness of education intervention carried out by clinical pharmacist on quality of life of patients with COPD: a randomized controlled trial. Pharmaceutical sciences asia. 2020;47(3):238-45. doi: 10.29090/psa.2020.03.019.0021. PubMed PMID: CN-02160544.

6527. Bui QTH, Pham KX, Tran TH, Le LTT, Nguyen HN. Impact of a pharmacist-led educational intervention on quality of life among patients with asthma. Asian journal of pharmaceutical and clinical research. 2019;12(6):307-12. doi: 10.22159/ajpcr.2019.v12i6.33705. PubMed PMID: CN-01976878.

6528. Bui TNT, Hotham E, Kelly F, Suppiah V. Feasibility of a pharmacist-led physical health monitoring for patients on antipsychotic medications: Protocol for a longitudinal study. BMJ Open. 2022;12(6). doi: 10.1136/bmjopen-2021-059573.

6529. Buicu CF, Moldovan I, Cotoi CT, Chiotoroiu AL, Moisi RE, Rafila A. VACCINATION IN COMMUNITY PHARMACIES - A GLOBAL REVIEW AND THE ROMANIAN OPPORTUNITY. Farmacia. 2018;66(2):230-6. PubMed PMID: WOS:000431275100006.

6530. Buising KL, Thursky KA, Black JF, MacGregor L, Street AC, Kennedy MP, et al. Improving antibiotic prescribing for adults with community acquired pneumonia: Does a computerised decision support system achieve more than academic detailing alone?–a time series analysis. BMC Medical Informatics and Decision Making. 2008;8:1-10.

6531. Buisson J. Protocol medicines supply: An end to supervision in community pharmacy? Pharmaceutical Journal. 2002;269(7218):480.

6532. Buitron de la Vega P, Ashe EM, Xuan Z, Gast V, Saint-Phard T, Brody-Fialkin J, et al. A Pharmacy Liaison-Patient Navigation Intervention to Reduce Inpatient and Emergency Department Utilization Among Primary Care Patients in a Medicaid Accountable Care Organization: A Nonrandomized Controlled Trial. JAMA network open. 2023;6(1):e2250004. doi: 10.1001/jamanetworkopen.2022.50004.

6533. Buitron de la Vega P, Ashe EM, Xuan Z, Gast V, Saint-Phard T, Brody-Fialkin J, et al. A Pharmacy Liaison-Patient Navigation Intervention to Reduce Inpatient and Emergency Department Utilization Among

Primary Care Patients in a Medicaid Accountable Care Organization: A Nonrandomized Controlled Trial. JAMA network open. 2023;6(1):e2250004. doi: 10.1001/jamanetworkopen.2022.50004.

6534. Buja A, Bardin A, Grotto G, Elvini S, Gallina P, Zumerle G, et al. How different combinations of comorbidities affect healthcare use by elderly patients with obstructive lung disease. Npj Primary Care Respiratory Medicine. 2021;31(1). doi: 10.1038/s41533-021-00242-y. PubMed PMID: WOS:000658731500002.

6535. Buja A, Elvini S, Caberlotto R, Pinato C, Mafrici SF, Grotto G, et al. Healthcare Service Usage and Costs for Elderly Patients with Obstructive Lung Disease. International Journal of Chronic Obstructive Pulmonary Disease. 2020;15:3357-66. doi: 10.2147/copd.S275687. PubMed PMID: WOS:000602550700001.

6536. Bujanda IL, Loureiro-Amigo J, Bastons FC, Guerra IE, Sanchez JA, Murgadella-Sancho A, et al. Treatment of COVID-19 pneumonia with glucocorticoids (CORTIVID): a structured summary of a study protocol for a randomised controlled trial. Trials. 2021;22(1). doi: 10.1186/s13063-020-04999-4. PubMed PMID: WOS:000609508300003.

6537. Bujold E, Huff J, Staton EW, Pace WD. Improving use of narcotics for nonmalignant chronic pain: A lesson from Community Care of North Carolina. Journal of Opioid Management. 2012;8(6):363-7. doi: 10.5055/jom.2012.0136.

6538. Bukhari N, Rasheed H, Nayyer B, Babar ZUD. Pharmacists at the frontline beating the COVID-19 pandemic. Journal of Pharmaceutical Policy and Practice. 2020;13(1). doi: 10.1186/s40545-020-00210-w.

6539. Bukhsh A, Khan TM, Phisalprapa P, Duangjai A, Saokaew S, Nawaz MS, et al. Impact of Pharmacist-Led Diabetes Self-Care Education on Patients With Type 2 Diabetes in Pakistan: a Randomized Controlled Trial. Frontiers in pharmacology. 2022;13. doi: 10.3389/fphar.2022.754999. PubMed PMID: CN-02375284.

6540. Bukhsh A, Nawaz MS, Ahmed HS, Khan TM. A randomized controlled study to evaluate the effect of pharmacist-led educational intervention on glycemic control, self-care activities and disease knowledge among type 2 diabetes patients: a consort compliant study protocol. Medicine. 2018;97(12):e9847. doi: 10.1097/MD.0000000000009847. PubMed PMID: CN-01599566.

6541. Bukic J, Kuzmanic B, Rusic D, Portolan M, Mihanovic A, Perisin AS, et al. Community pharmacists' use, perception and knowledge on dietary supplements: a cross sectional study. Pharmacy Practice-Granada. 2021;19(1). doi: 10.18549/PharmPract.2021.1.2251. PubMed PMID: WOS:000663380400011.

6542. Bukic J, Kuzmanic B, Rusic D, Portolan M, Mihanovic A, Seselja Perisin A, et al. Community pharmacists’ use, perception and knowledge on dietary supplements: A cross sectional study. Pharmacy Practice. 2021;19(1). doi: 10.18549/PHARMPRACT.2021.1.2251.

6543. Bukonda NKZ, Disashi TG, Mukonu KA, Facilitators N. Assessing the State of Nosocomial HIV Infection Control (NHIC) in Health Zones in the Eastern Kasai Province (Democratic Republic of the Congo). American Journal of Infection Control. 2006;34(5):E89. doi: https://doi.org/10.1016/j.ajic.2006.05.235.

6544. Bulaj G, Clark J, Ebrahimi M, Bald E. From Precision Metapharmacology to Patient Empowerment: Delivery of Self-Care Practices for Epilepsy, Pain, Depression and Cancer Using Digital Health Technologies. Frontiers in Pharmacology. 2021;12. doi: 10.3389/fphar.2021.612602.

6545. Buljan J. Vital statistic changes in Velika Kopanica municipality in the 1952-2013 period as basis for primary health care planing. Medica Jadertina. 2016;45(3-4):105-14.

6546. Bullen K, Ainsley K, Davison K. Evaluating the numeracy skills of pharmacy students in multiple choice questions and free-text answer assessments and their perception of numeracy in clinical practice. Currents in Pharmacy Teaching and Learning. 2020;12(6):648-55. doi: https://doi.org/10.1016/j.cptl.2020.01.028.

6547. Bullen K, Davison K, Hardisty J. Community pharmacy as an effective teaching and learning environment: Student perspectives from a UK MPharm programme. Currents in Pharmacy Teaching and Learning. 2019;11(9):902-8. doi: 10.1016/j.cptl.2019.05.007.

6548. Buller DB, Woodall WG, Saltz R, Starling R. Pseudo-Underage Assessment of Compliance With Identification Regulations at Retail Marijuana Outlets in Colorado. Journal of Studies on Alcohol and Drugs. 2016;77(6):868-72. doi: 10.15288/jsad.2016.77.868. PubMed PMID: WOS:000386986600003.

6549. Bulloch M, Shunnarah A, Gwaltney A, Hendricks K. 268: EVALUATION OF CLINICAL TRIALS ENROLLMENT OF GERIATRIC PATIENTS FOR THE PREVENTION OF STRESS ULCERS. Critical Care Medicine. 2023;51:119-. doi: 10.1097/01.ccm.0000906808.50995.ae. PubMed PMID: 160832736.

6550. Bulloch MN, Olin JL. Instruments for evaluating medication use and prescribing in older adults. Journal of the American Pharmacists Association: JAPhA. 2014;54(5):530-7. PubMed PMID: 25216883.

6551. Bullock A, Rowan CG, Oestreicher N, Yeganegi H, Chiorean EG. Real-World Assessment of Health Care Costs for Patients with Metastatic Pancreatic Cancer Following Initiation of First-Line Chemotherapy. Journal of managed care & specialty pharmacy. 2020;26(7):872-8. doi: 10.18553/jmcp.2020.26.7.872. PubMed PMID: CN-02252851.

6552. Bullock A, Rowan CG, Oestreicher N, Yeganegi H, Chiorean EG. Real-World Assessment of Health Care Costs for Patients with Metastatic Pancreatic Cancer Following Initiation First-Line Chemotherapy. Journal of Managed Care & Specialty Pharmacy. 2020;26(7):872-8. PubMed PMID: WOS:000544082000010.

6553. Bullock B, Donovan P, Mitchell C, Whitty JA, Coombes I. The impact of a pharmacist on post-take ward round prescribing and medication appropriateness. International Journal of Clinical Pharmacy. 2019;41(1):65-73. doi: 10.1007/s11096-018-0775-9. PubMed PMID: 134695122. Language: English. Entry Date: 20190607. Revision Date: 20200203. Publication Type: journal article.

6554. Bullock H, Steele S, Kurata N, Tschann M, Elia J, Kaneshiro B, et al. Pharmacy access to ulipristal acetate in Hawaii: Is a prescription enough? Contraception. 2016;93(5):452-4. doi: 10.1016/j.contraception.2015.12.003.

6555. Bullock KC. Development, implementation, and evaluation of a service-learning series for pharmacy students using a public health tool. Currents in Pharmacy Teaching and Learning. 2017;9(5):828-34. doi: https://doi.org/10.1016/j.cptl.2017.05.015.

6556. Bulow C, Faerch KU, Armandi H, Jensen BN, Sonne J, Christensen HR, et al. Important Aspects of Pharmacist-led Medication Reviews in an Acute Medical Ward. Basic & Clinical Pharmacology & Toxicology. 2018;122(2):253-61. PubMed PMID: 28871627.

6557. Bulow C, Flagstad Bech C, Ullitz Faerch K, Traerup Andersen J, Byg Armandi H, Treldal C. Discrepancies Between the Medication List in Electronic Prescribing Systems and Patients' Actual Use of Medicines. The Senior Care Pharmacist. 2019;34(5):317-24. PubMed PMID: 31054590.

6558. Bulska E. Quality assurance and quality control of analytical results. Analytical Measurements in Aquatic Environments2009. p. 389-98.

6559. Bultman DC. Consumer perspectives of provider communication styles and antidepressants: A study of beliefs and outcomes [Ph.D.]. United States -- Wisconsin: The University of Wisconsin - Madison; 1998.

6560. Bultman DC, Svarstad BL. Effects of Pharmacist Monitoring on Patient Satisfaction With Antidepressant Medication Therapy. Journal of the American Pharmaceutical Association (1996). 2002;42(1):36-43. doi: https://doi.org/10.1331/108658002763538053.

6561. Bulut S, Bilgener E. Evaluation of turkish pharmaceutical track and trace system (ITS): perspective of community pharmacists. Brazilian Journal of Pharmaceutical Sciences. 2022;58. doi: 10.1590/s2175-97902022e20407.

6562. Bunchman TE, Maxvold NJ, Brophy PD. Pediatric convective hemofiltration: Normocarb replacement fluid and citrate anticoagulation. American Journal of Kidney Diseases. 2003;42(6):1248-52. doi: https://doi.org/10.1053/j.ajkd.2003.08.026.

6563. Bungard TJ, Ritchie B, Garg S, Tsuyuki RT. Sustained impact of anticoagulant control achieved in an anticoagulation management service after transfer of management to the primary care physician. Pharmacotherapy. 2012;32(2):112-9. doi: 10.1002/PHAR.1011. PubMed PMID: CN-00970329.

6564. Bungau S, Tit DM, Fodor K, Cioca G, Agop M, Iovan C, et al. Aspects Regarding the Pharmaceutical Waste Management in Romania. Sustainability. 2018;10(8). doi: 10.3390/su10082788. PubMed PMID: WOS:000446767700204.

6565. Bungay KM, Adler DA, Rogers WH, McCoy C, Kaszuba M, Supran S, et al. Description of a clinical pharmacist intervention administered to primary care patients with depression. General hospital psychiatry. 2004;26(3):210-8. doi: 10.1016/j.genhosppsych.2004.01.004. PubMed PMID: CN-00481966.

6566. Bunge P. A model for internal medicine physicians in a small rural hospital. Rural and Remote Health. 2018;18(3). doi: 10.22605/RRH4419.

6567. Bunniran S. Patient Service Experiences in Community Pharmacy: An Examination of Health Criticality, Service Failure Incidents, and Service Recovery Efforts and Their Influence on Patronage Outcomes [Ph.D.]. United States -- Mississippi: The University of Mississippi; 2010.

6568. Bunniran S, McCaffrey DJ, Bentley JP, Bouldin AS. Pharmaceutical product withdrawal: attributions of blame and its impact on trust. Research in social & administrative pharmacy : RSAP. 2009;5(3):262-73. doi: 10.1016/j.sapharm.2008.10.002. PubMed PMID: CN-00722397.

6569. Bunting BA, Cranor CW. The Asheville Project: Long-Term Clinical, Humanistic, and Economic Outcomes of a Community-Based Medication Therapy Management Program for Asthma. Journal of the American Pharmacists Association. 2006;46(2):133-47. doi: https://doi.org/10.1331/154434506776180658.

6570. Bunting BA, Smith BH, Sutherland SE. The Asheville Project: Clinical and economic outcomes of a community-based long-term medication therapy management program for hypertension and dyslipidemia. Journal of the American Pharmacists Association. 2008;48(1):23-31. doi: https://doi.org/10.1331/JAPhA.2008.07140.

6571. Bunting SR, Feinstein BA, Bertram C, Hazra A, Sheth NK, Garber SS. Effects of knowledge and implicit biases on pharmacy students' decision-making regarding pre-exposure prophylaxis for HIV prevention: A vignette-based experimental study. Currents in Pharmacy Teaching and Learning. 2023;15(2):139-48. doi: https://doi.org/10.1016/j.cptl.2023.02.019.

6572. Bunting SR, Saqueton R, Batteson TJ. A Guide for Designing Student-Led, Interprofessional

Community Education Initiatives About HIV Risk and Pre-Exposure Prophylaxis. MedEdPORTAL : the journal of teaching and learning resources. 2019;15:10818. doi: 10.15766/mep_2374-8265.10818.

6573. Buono JL, Mathur K, Averitt AJ, Andrae DA. Economic Burden of Irritable Bowel Syndrome with Diarrhea: Retrospective Analysis of a US Commercially Insured Population. Journal of Managed Care & Specialty Pharmacy. 2017;23(4):435-42. PubMed PMID: WOS:000399062100004.

6574. Buono JL, Mathur K, Averitt AJ, Andrae DA. Economic burden of inadequate symptom control among US commercially insured patients with irritable bowel syndrome with diarrhea. Journal of Medical Economics. 2017;20(4):353-62. doi: 10.1080/13696998.2016.1269016. PubMed PMID: WOS:000396843200006.

6575. Burapat C, Kittikraisak W, Cain KP, Tasaneeyapan T, Nateniyom S, Akksilp S, et al. HEALTH-SEEKING BEHAVIOR AMONG HIV-INFECTED PATIENTS TREATED FOR TB IN THAILAND. Southeast Asian Journal of Tropical Medicine and Public Health. 2009;40(6):1335-46. PubMed PMID: WOS:000272578200024.

6576. Burcham L. Internet Pharmacy: Trust or Mistrust? [M.S.]. United States -- Minnesota: The College of St. Scholastica; 2012.

6577. Burchett HED, Leurent B, Baiden F, Baltzell K, Bjorkman A, Bruxvoort K, et al. Improving prescribing practices with rapid diagnostic tests (RDTs): synthesis of 10 studies to explore reasons for variation in malaria RDT uptake and adherence. Bmj Open. 2017;7(3). doi: 10.1136/bmjopen-2016-012973. PubMed PMID: WOS:000398959400036.

6578. Burden AM. Lost in translation: Exposure misclassification when relying on days supply in pharmacy claims data [Ph.D.]. Canada -- Ontario, CA: University of Toronto (Canada); 2014.

6579. Burden AM, Huang A, Tadrous M, Cadarette SM. Variation in the days supply field for osteoporosis medications in Ontario. Archives of Osteoporosis. 2013;8(1-2). doi: 10.1007/s11657-013-0128-1.

6580. Burden AM, Huang AJ, Tadrous M, Cadarette SM. Variation in the days supply field for osteoporosis medications in Ontario. Archives of Osteoporosis. 2013;8(1-2). doi: 10.1007/s11657-013-0128-1. PubMed PMID: WOS:000209644900015.

6581. Burden AM, Paterson JM, Gruneir A, Cadarette SM. Adherence to osteoporosis pharmacotherapy is underestimated using days supply values in electronic pharmacy claims data. Pharmacoepidemiology and Drug Safety. 2015;24(1):67-74. doi: 10.1002/pds.3718. PubMed PMID: WOS:000347710100010.

6582. Burden N, Sewell F, Andersen ME, Boobis A, Chipman JK, Cronin MT, et al. Adverse outcome pathways can drive non-animal approaches for safety assessment. Journal of Applied Toxicology. 2015;35(9):971-5.

6583. Burdon M. Reconsider community pharmacy proposals. Pharmaceutical Journal. 2016;296(7890):359.

6584. Buregyeya E, Rutebemberwa E, LaRussa P, Lal S, Clarke SE, Hansen KS, et al. Comparison of the capacity between public and private health facilities to manage under-five children with febrile illnesses in Uganda. Malaria Journal. 2017;16. doi: 10.1186/s12936-017-1842-8. PubMed PMID: WOS:000402208300004.

6585. Buregyeya E, Rutebemberwa E, LaRussa P, Mbonye A. Strengthening referral of sick children from the private health sector and its impact on referral uptake in Uganda: a cluster randomized controlled trial protocol. Bmc Health Services Research. 2016;16. doi: 10.1186/s12913-016-1885-5. PubMed PMID:

WOS:000387540000001.

6586. Burford O, Jiwa M, Carter O, Parsons R, Hendrie D. Internet-based photoaging within Australian pharmacies to promote smoking cessation: randomized controlled trial. Journal of medical Internet research. 2013;15(3):e64. doi: 10.2196/jmir.2337. PubMed PMID: CN-00874631.

6587. Burford O, Kindarji S, Parsons R, Falcoff H. Using visual demonstrations in young adults to promote smoking cessation: preliminary findings from a French pilot study. Research in social & administrative pharmacy : RSAP. 2018;14(4):398-400. doi: 10.1016/j.sapharm.2017.04.050. PubMed PMID: CN-01656409.

6588. Burge R, Sato M, Sugihara T. Real-world clinical and economic outcomes for daily teriparatide patients in Japan. Journal of Bone and Mineral Metabolism. 2016;34(6):692-702. doi: 10.1007/s00774-015-0720-0. PubMed PMID: WOS:000387272300011.

6589. Burger CD, Ozbay AB, Lazarus HM, Riehle E, Montejano LB, Lenhart G, et al. Treatment Patterns and Associated Health Care Costs Before and After Treatment Initiation Among Pulmonary Arterial Hypertension Patients in the United States. Journal of Managed Care & Specialty Pharmacy. 2018;24(8):834-42. doi: 10.18553/jmcp.2018.17391. PubMed PMID: WOS:000440131000011.

6590. Burger D, Oosterhof P, Grintjes K, Marneef-Pietersma M, d'Ancona F, Zhu X, et al. Potential drug–drug interactions in males living with HIV who use drugs to treat lower urinary tract symptoms. HIV Medicine. 2023;24(10):1083-7. doi: 10.1111/hiv.13519. PubMed PMID: 172804413.

6591. Burger GS, Jorgenson JA, Stevenson JG. Building a business case for an outpatient pharmacy. Healthcare financial management : journal of the Healthcare Financial Management Association. 2015;69(6):76-81.

6592. Burger NE, Kopf D, Spreng CP, Yoong J, Sood N. Healthy firms: Constraints to growth among private health sector facilities in Ghana and Kenya. PLoS ONE. 2012;7(2). doi: 10.1371/journal.pone.0027885.

6593. Burgess C, Wright AJ, Forster AS, Dodhia H, Miller J, Fuller F, et al. Influences on individuals' decisions to take up the offer of a health check: a qualitative study. Health Expectations. 2015;18(6):2437-48.

6594. Burgess DJ, Ding Y, Hargreaves M, Van Ryn M, Phelan S. The association between perceived discrimination and underutilization of needed medical and mental health care in a multi-ethnic community sample. Journal of Health Care for the Poor and Underserved. 2008;19(3):894-911.

6595. Burgess LH, Cohen MR, Denham CR. A new leadership role for pharmacists: a prescription for change. Journal of Patient Safety. 2010;6(1):31-7. doi: 10.1097/PTS.0b013e3181d108cb. PubMed PMID: 109852525. Language: English. Entry Date: 20100430. Revision Date: 20151008. Publication Type: Journal Article. Journal Subset: Health Services Administration.

6596. Burgess LH, Cohen MR, Denham CR. A New Leadership Role for Pharmacists. Journal of patient safety. 2010;6(1):31-7.

6597. Burgess LH, Cooper MK, Wiggins EH, Miller KM, Murray E, Harris S, et al. Utilizing Pharmacists to Optimize Medication Management Strategies During the COVID-19 Pandemic. Journal of Pharmacy Practice. 2022;35(2):184-9. PubMed PMID: 33016180.

6598. Burghardt KJ, Bowman MR, Hibino M, Opong-Owusu BK, Pokora TD, Reeves K, et al. Using educational games to promote the seeking of a pharmacist and to teach key medication use messages: Results from an inner city health party. Research in Social and Administrative Pharmacy. 2013;9(5):542-52. doi: 10.1016/j.sapharm.2013.01.001.

6599. Burghle A, Abrahamsen B, Lundby C, Rossing C, Hansen RN, Nørgaard LS, et al. Customers' information seeking behavior prior to community pharmacy visits: A community pharmacy survey. Research in Social and Administrative Pharmacy. 2020;16(10):1442-6. doi: 10.1016/j.sapharm.2019.12.021.

6600. Burghle A, Pottegard A, Rasmussen M, Bruun B, Hosbjerg JR, Lundby C. Use of analgesics in Denmark: A national survey. Basic & Clinical Pharmacology & Toxicology. 2023;132(4):321-7. doi: 10.1111/bcpt.13837. PubMed PMID: WOS:000919120000001.

6601. Burghle A, Pottegård A, Rasmussen M, Bruun B, Roost Hosbjerg J, Lundby C. Use of analgesics in Denmark: A national survey. Basic and Clinical Pharmacology and Toxicology. 2023;132(4):321-7. doi: 10.1111/bcpt.13837.

6602. Burghle A, Pottegård A, Rasmussen M, Bruun B, Roost Hosbjerg J, Lundby C. Use of analgesics in Denmark: A national survey. Basic and Clinical Pharmacology and Toxicology. 2023. doi: 10.1111/bcpt.13837.

6603. Burgin A, O'Rourke R, Tully MP. Learning to work with electronic patient records and prescription charts: experiences and perceptions of hospital pharmacists. Research in Social and Administrative Pharmacy. 2014;10(5):741-55. doi: https://doi.org/10.1016/j.sapharm.2013.11.005.

6604. Burgio KL, Goode PS, Johnson TM, Hammontree L, Ouslander JG, Markland AD, et al. Behavioral Versus Drug Treatment for Overactive Bladder in Men: The Male Overactive Bladder Treatment in Veterans (MOTIVE) Trial. Journal of the American Geriatrics Society. 2011;59(12):2209-16. doi: 10.1111/j.1532-5415.2011.03724.x. PubMed PMID: WOS:000298600300003.

6605. Burguet Lago N, Troche Concepción Y. PERFIL DE SEGURIDAD DEL USO DE UN ANTICOAGULANTE EN PACIENTES GERIÁTRICOS MAYORES DE 65 AÑOS. SAFETY PROFILE OF THE USE OF AN ANTICOAGULANT IN GERIATRIC PATIENTS OLDER THAN 65 YEARS. 2023;54:112-21. PubMed PMID: 176402003.

6606. Buring SM, Bhushan A, Brazeau G, Conway S, Hansen L, Westberg S. Keys to successful implementation of interprofessional education: learning location, faculty development, and curricular themes. American Journal of Pharmaceutical Education. 2009;73(4).

6607. Buring SM, Bhushan A, Broeseker A, Conway S, Duncan-Hewitt W, Hansen L, et al. Interprofessional education: definitions, student competencies, and guidelines for implementation. American journal of pharmaceutical education. 2009;73(4).

6608. Burk M, Moore V, Glassman P, Good CB, Emmendorder T, Leadholm TC, et al. Medication-use evaluation with a Web application. American Journal of Health-System Pharmacy. 2013;70(24):2226-34. doi: 10.2146/ajhp130252. PubMed PMID: 104128323. Language: English. Entry Date: 20131219. Revision Date: 20200708. Publication Type: Journal Article.

6609. Burke A, Maundu J, Clark B, Wilkinson G, Saini B, Moles R. Status of Aboriginal and Torres Strait Islander people's cultural safety/competence education in Australian pharmacy schools. Research in Social and Administrative Pharmacy. 2023;19(7):12. doi: https://doi.org/10.1016/j.sapharm.2023.04.034.

6610. Burke A, O'Driscoll M, Crowley EK, Dhubhlaing CN. Implementation and evaluation of STOPP/START criteria to address polypharmacy in older adults in an inpatient psychiatric setting. Exploratory Research in Clinical and Social Pharmacy. 2023;9:100245. doi: https://dx.doi.org/10.1016/j.rcsop.2023.100245. PubMed PMID: 2023603217.

6611. Burke J, Kovacs B, Barton L, Sander S. Health Care Utilization and Costs in Type 2 Diabetes Mellitus

and Their Association with Renal Impairment. Postgraduate Medicine. 2012;124(2):77-91. doi: 10.3810/pgm.2012.03.2539. PubMed PMID: WOS:000303420500008.

6612. Burke MJ. Drug free: How North Memorial tackled its pharmaceutical waste problem. Health facilities management. 2008;21(10):67-8,70.

6613. Burke R. PHARMACY PRACTICE. Journal of Pharmacy Practice & Research. 2010;40(3):243-4. PubMed PMID: 54400824. Language: English. Entry Date: 20101210. Revision Date: 20101213. Publication Type: Abstract.

6614. Burke R. PHARMACY PRACTICE. Journal of Pharmacy Practice & Research. 2011;41(1):71-2. PubMed PMID: 61017152. Language: English. Entry Date: 20110610. Revision Date: 20110617. Publication Type: Abstract.

6615. Burke R. PHARMACY PRACTICE. Journal of Pharmacy Practice & Research. 2012;42(2):164-5. PubMed PMID: 77909296. Language: English. Entry Date: 20120831. Revision Date: 20190308. Publication Type: Abstract.

6616. Burke R. PHARMACY PRACTICE. Journal of Pharmacy Practice & Research. 2013;43(2):158-9. PubMed PMID: 89294504. Language: English. Entry Date: 20130731. Revision Date: 20170203. Publication Type: Abstract.

6617. Burkhard A, Toliver J, Rascati K. Association between multiple sclerosis disease severity and adherence to disease-modifying therapies. Journal of Managed Care & Specialty Pharmacy. 2021;27(7):915-23. doi: 10.18553/jmcp.2021.27.7.915. PubMed PMID: WOS:000668138500011.

6618. Burkhardt C, Crowl A, Ramirez M, Long B, Shrader S. A reflective assignment assessing pharmacy students' interprofessional collaborative practice exposure during introductory pharmacy practice experiences. American Journal of Pharmaceutical Education. 2019;83(6):1300-9.

6619. Burkiewicz JS, Hassenplug KL. Educational intervention to reduce frequency of use of restricted abbreviations in a community health center. Journal of Pharmacy Technology. 2006;22(6):332-5. PubMed PMID: 46025684.

6620. Burkiewicz JS, Sweeney BL. Medication reviews in senior community housing centers. Consultant Pharmacist. 2006;21(9):715-8. doi: 10.4140/TCP.n.2006.715.

6621. Burman M, Copas A, Zenner D, Hickson V, Gosce L, Trathen D, et al. Protocol for a cluster randomised control trial evaluating the efficacy and safety of treatment for latent tuberculosis infection in recent migrants within primary care: the CATAPuLT trial. BMC public health. 2019;19(1):1598. doi: 10.1186/s12889-019-7983-7. PubMed PMID: CN-02051487.

6622. Burman M, Zenner D, Copas A, Gosce L, White P, Hickson V, et al. Is the treatment of latent tuberculosis infection amongst recent migrants safe and effective in primary care? American journal of respiratory and critical care medicine. 2021;203(9). doi: 10.1164/ajrccm-conference.2021.203.1_MeetingAbstracts.A1191. PubMed PMID: CN-02286298.

6623. Burnett A, Rudd KM, Triller D. Advancing anticoagulation stewardship: A call to action for stewardship from the US-based anticoagulation forum. Thrombosis Update. 2022;9:100125. doi: https://doi.org/10.1016/j.tru.2022.100125.

6624. Burnett KM. The value of hospital based pharmaceutical audit (BL) [Ph.D.]. Northern Ireland: Queen's University Belfast (United Kingdom); 2000.

6625. Burnette CE, Roh S, Liddell J, Lee YS. American Indian women cancer survivors' coping with depressive symptoms. Journal of Psychosocial Oncology. 2019;37(4):494-508. doi: 10.1080/07347332.2018.1525467. PubMed PMID: WOS:000468616000006.

6626. Burningham Z, Jackson GL, Kelleher J, Stevens M, Morris I, Cohen J, et al. The Enhancing Quality of Prescribing Practices for Older Veterans Discharged From the Emergency Department (EQUIPPED) Potentially Inappropriate Medication Dashboard: a Suitable Alternative to the In-person Academic Detailing and Standardized Feedback Reports of Traditional EQUIPPED? Clinical therapeutics. 2020;42(4):573-82. doi: 10.1016/j.clinthera.2020.02.013. PubMed PMID: CN-02096919.

6627. Burningham Z, Wei C, Sauer BC, Richter Lagha R, Hansen J, Huynh T, et al. VA Geriatric Scholars Program's Impact on Prescribing Potentially Inappropriate Medications. American Journal of Managed Care. 2019;25(9):425-30. PubMed PMID: 138669907. Language: English. Entry Date: 20190920. Revision Date: 20190921. Publication Type: Article.

6628. Burns A. Medication errors: Why they are important and what you can do: Evaluation of dispensing process vital for all pharmacies. Journal of the American Pharmaceutical Association (1996). 2001;41(2, Supplement):14-22. doi: https://doi.org/10.1016/S1086-5802(16)31358-4.

6629. Burns A. Medication therapy management services: A critical review. Journal of the American Pharmacists Association. 2005;45(5):580-7. PubMed PMID: 46037269.

6630. Burns A. Medication therapy management in community pharmacy practice: Core elements of an MTM service (Version 1.0). Journal of the American Pharmacists Association. 2005;45(5):573-9. doi: 10.1331/1544345055001256.

6631. Burns A. Medication therapy management in pharmacy practice: core elements of an MTM service model (version 2.0). Journal of the American Pharmacists Association. 2008;48(3):341-53.

6632. Burns A, Goodlet KJ, Chapman A, Roberts EP. A case report of self-medication with over-the-counter fish antibiotic: Implications for pharmacists. Journal of the American Pharmacists Association. 2020;60(4):e121-e3. doi: https://doi.org/10.1016/j.japh.2019.12.020.

6633. Burns A, Lewin G. Medication therapy management services: A critical review. Journal of the American Pharmacists Association. 2005;45(5):580-7. PubMed PMID: WOS:000203201900009.

6634. Burns C. National pharmacy boards meeting: 3 February 2021. Pharmaceutical Journal. 2021;306(7947). doi: 10.1211/PJ.2021.1.49320.

6635. Burns C. Pharmacists should have read-write access to health records, standards body says. Pharmaceutical Journal. 2022;309(7966). doi: 10.1211/PJ.2022.1.161155.

6636. Burns C. Repeat prescribing intervals in Wales to increase from 28 to 56 days. Pharmaceutical Journal. 2022;308(7958). doi: 10.1211/PJ.2022.1.130337.

6637. Burns C. NHS to fund nearly 330 independent prescriber trainingplaces for community pharmacists. Pharmaceutical Journal. 2022;308(7957). doi: 10.1211/PJ.2022.1.126006.

6638. Burns C. Community pharmacies to supply antiviral treatmentsto high-risk COVID-19 patients. Pharmaceutical Journal. 2022;308(7957). doi: 10.1211/PJ.2022.1.123730.

6639. Burns CM, Endres K, Derrick C, Cooper A, Fabel P, Okeke NL, et al. A survey of South Carolina pharmacists' readiness to prescribe human immunodeficiency virus pre-exposure prophylaxis. Journal of the

American College of Clinical Pharmacy. 2023;6(4):329-38. doi: 10.1002/jac5.1773. PubMed PMID: WOS:000947739400001.

6640. Burns CV, Lucas K, Faraj M, Millis S, Garwood C, Marawar R. Impact of ambulatory pharmacist on hospital visits in older adults with epilepsy: A case-control study. Epilepsy & Behavior. 2023;140:109109. doi: https://doi.org/10.1016/j.yebeh.2023.109109.

6641. Burns D, Kula J, Marshall S, Ashworth E, Ornelas M. Best Practice Approach to Successful Conversion of Fosaprepitant to Aprepitant IV in a Large Multisite Community Oncology Infusion Center: A Retrospective Analysis. Advances in Therapy. 2020;37(7):3265-77. doi: 10.1007/s12325-020-01377-z. PubMed PMID: WOS:000534997900002.

6642. Burns D, Lal R, Donnell CM. Paediatric harmful adverse drug events (PHADE). Paediatrics & Child Health (1205-7088). 2023;28(5):299-304. doi: 10.1093/pch/pxac132. PubMed PMID: 169793451.

6643. Burns J, Ouvrier RA, Yiu EM, Joseph PD, Kornberg AJ, Fahey MC, et al. Ascorbic acid for Charcot-Marie-Tooth disease type 1A in children: a randomised, double-blind, placebo-controlled, safety and efficacy trial. The lancet Neurology. 2009;8(6):537-44. doi: 10.1016/S1474-4422(09)70108-5. PubMed PMID: CN-00703105.

6644. Burns J, Ouvrier RA, Yiu EM, Joseph PD, Kornberg AJ, Fahey MC, et al. Ascorbic acid for Charcot–Marie–Tooth disease type 1A in children: a randomised, double-blind, placebo-controlled, safety and efficacy trial. The Lancet Neurology. 2009;8(6):537-44. doi: https://doi.org/10.1016/S1474-4422(09)70108-5.

6645. Burns K, Turnbull F, Patel A, Peiris D. Opinions of community pharmacists on the value of a cardiovascular polypill as a means of improving medication compliance. International Journal of Pharmacy Practice. 2012;20(3):155-63. doi: 10.1111/j.2042-7174.2011.00175.x.

6646. Burns KE, Chant C, Smith O, Cuthbertson B, Fowler R, Cook DJ, et al. A Canadian Critical Care Trials Group project in collaboration with the international forum for acute care trialists - Collaborative H1N1 Adjuvant Treatment pilot trial (CHAT): study protocol and design of a randomized controlled trial. Trials. 2011;12:70. doi: 10.1186/1745-6215-12-70. PubMed PMID: CN-00786093.

6647. Burns KW, Johnson KM, Pham SN, Egwuatu NE, Dumkow LE. Implementing outpatient antimicrobial stewardship in a primary care office through ambulatory care pharmacist-led audit and feedback. Journal of the American Pharmacists Association: JAPhA. 2020;60(6):e246-e51. doi: 10.1016/j.japh.2020.08.003. PubMed PMID: 146713004. Language: English. Entry Date: 20210726. Revision Date: 20211014. Publication Type: journal article.

6648. Burns N. Evaluation of warfarin dosing by pharmacists for elderly medical in-patients. Pharmacy World & Science. 2004;26(4):232-7. PubMed PMID: 15446781.

6649. Burns N, Alkaisy Z, Sharp E. Doctors attitudes towards medication errors at 2002 & 2015. International Journal of Health Care Quality Assurance (09526862). 2018;31(6):451-63. doi: 10.1108/IJHCQA-04-2016-0038. PubMed PMID: 130417695. Language: English. Entry Date: 20181215. Revision Date: 20190308. Publication Type: journal article.

6650. Burnside TPT, Scott NJ, Smith MG. Implementation of technician-driven medication therapy management program in community pharmacies. Journal of the American Pharmacists Association: JAPhA. 2019;59(4S):S156-S60.e2. PubMed PMID: 31326039.

6651. Burón A, Grau J, Andreu M, Augé JM, Guayta-Escolies R, Barau M, et al. Colorectal Cancer Early Screening Program of Barcelona, Spain: Indicators of the first round of a program with participation of

community pharmacies. Medicina Clínica (English Edition). 2015;145(4):141-6. doi: https://doi.org/10.1016/j.medcle.2016.01.019.

6652. Burón A, Grau J, Andreu M, Augé JM, Guayta-Escolies R, Barau M, et al. Programa de Detección Precoz de Cáncer de Colon y Recto de Barcelona: indicadores de la primera ronda de un programa con participación de la farmacia comunitaria. Medicina Clínica. 2015;145(4):141-6. doi: https://doi.org/10.1016/j.medcli.2014.05.027.

6653. Burón A, Posso M, Sivilla J, Grau J, Guayta R, Castells X, et al. Analysis of participant satisfaction in the Barcelona colorectal cancer screening programme: Positive evaluation of the community pharmacy. Gastroenterologia y Hepatologia. 2017;40(4):265-75. doi: 10.1016/j.gastrohep.2016.04.015.

6654. Buron A, Posso M, Sivilla J, Grau J, Guayta R, Castells X, et al. Analysis of participant satisfaction in the Barcelona colorectal cancer screening programme: Positive evaluation of the community pharmacy. Gastroenterologia Y Hepatologia. 2017;40(4):265-75. doi: 10.1016/j.gastrohep.2016.04.015. PubMed PMID: WOS:000399966900001.

6655. Burrell A, Ethun L, Fawcett JA, Rickard-Aasen S, Williams K, Kearney SM, et al. The pharmacist's role in overdose: Using mapping technologies to analyze naloxone and pharmacy distribution. Journal of the American Pharmacists Association. 2017;57(2):S73-S7.e1. doi: 10.1016/j.japh.2016.11.006.

6656. Burri S, Scheidegger R, editors. The EU falsified medicines directive a concept for drug decommissioning in hospitals. Studies in Health Technology and Informatics; 2019.

6657. Burrill PD. No benefit in primary prevention trials [7]. Pharmaceutical Journal. 2004;272(7301):670.

6658. Burris S, Welsh J, Ng M, Li M, Ditzler A. State syringe and drug possession laws potentially influencing safe syringe disposal by injection drug users. Journal of the American Pharmaceutical Association (Washington,DC : 1996). 2002;42(6 Suppl 2):S94-8. doi: 10.1331/1086-5802.42.0.s94.burris.

6659. Burroughs VJ. Racial and ethnic inclusiveness in clinical trials. Ethics and the Pharmaceutical Industry2005. p. 80-96.

6660. Burrows J, Dall'Alba G, La Caze A. We are all patient-centred now, aren't we? Lessons from new pharmacy graduates. Currents in Pharmacy Teaching and Learning. 2020;12(5):508-16. doi: https://doi.org/10.1016/j.cptl.2020.01.017.

6661. Burruss RA, Carroll NV, Schraa C, Burton B. Outsourcing inpatient i.v. compounding: expense and medication error implications. Pharmacy practice management quarterly. 1996;16(3):52-9.

6662. Bursey F, Crowley M, Janes C, Turner CJ. Cost analysis of a provincial drug program to guide the treatment of upper gastrointestinal disorders. CMAJ : Canadian Medical Association journal. 2000;162(6):817-23. PubMed PMID: CN-00343245.

6663. Burson RC, Buttenheim AM, Armstrong A, Feemster KA. Community pharmacies as sites of adult vaccination: A systematic review. Human Vaccines & Immunotherapeutics. 2016;12(12):3146-59. doi: 10.1080/21645515.2016.1215393. PubMed PMID: WOS:000392826800027.

6664. Burstein DS, Griffis H, Zhang X, Cantor RS, Dai D, Shamszad P, et al. Resource utilization in children with paracorporeal continuous-flow ventricular assist devices. The Journal of Heart and Lung Transplantation. 2021;40(6):478-87. doi: https://doi.org/10.1016/j.healun.2021.02.011.

6665. Bursua A, Hartke PL, Larson CM, Gorbach C, Blanton L, Lukawsk B, et al. Benchmarking and

medication error rates. Oxford University Press / USA; 2016. p. 744-6.

6666. Burton K, editor A cure for poor information2012/07//: Australian Doctor Group.

6667. Burton K. DAA packs inaccurate: study. Pharmacy News. 2013:10-. PubMed PMID: 87089330. Language: English. Entry Date: 20130501. Revision Date: 20190409. Publication Type: Article.

6668. Burton LC, Anderson GF, Kues IW. Using electronic health records to help coordinate care. The Milbank Quarterly. 2004;82(3):457-81.

6669. Burton LM, McKay AB. Involvement of Maryland pharmacists in AIDS education. Patient Education and Counseling. 1990;15(3):239-47. doi: https://doi.org/10.1016/0738-3991(90)90099-7.

6670. Burton SA, Amir N, Asbury A, Lange A, Hardinger KL. Treatment of antibody-mediated rejection in renal transplant patients: A clinical practice survey. Clinical Transplantation. 2015;29(2):118-23. doi: 10.1111/ctr.12491.

6671. Burton T. Introducing Self Administration of Bortezomib (Velcade) to Myeloma Patients. Clinical lymphoma, myeloma & leukemia. 2019;19(10):e349-e50. doi: 10.1016/j.clml.2019.09.577. PubMed PMID: CN-01995989.

6672. Burtscher D, Van den Bergh R, Nasim M, Mahama G, Au S, Williams A, et al. 'They eat it like sweets': A mixed methods study of antibiotic perceptions and their use among patients, prescribers and pharmacists in a district hospital in Kabul, Afghanistan. PLoS ONE [Electronic Resource]. 2021;16(11):e0260096. PubMed PMID: 34797865.

6673. Burugupally K, Senthil S, Parameshwarappa DC, Ali H, Balakrishnan D, Garudadri C. Outcomes of lensectomy with glued intraocular lens and factors associated with failure in eyes with spherophakia and glaucoma. Indian Journal of Ophthalmology. 2023;71(6):2487-92. doi: 10.4103/ijo.IJO_1866_22. PubMed PMID: 171862237.

6674. Busa G, Burlina A, Damuzzo V, Chiumente M, Palozzo AC. Comorbidity, Polytherapy, and Drug Interactions in a Neurological Context: An Example of a Multidisciplinary Approach to Promote the Rational Use of Drugs. Journal of Pharmacy Practice. 2018;31(1):58-65. doi: 10.1177/0897190017699138. PubMed PMID: 127444447. Language: English. Entry Date: 20180203. Revision Date: 20190715. Publication Type: Article.

6675. Buschke S, Ring A, Friedrich C, Metzmann K, Meinicke T. Linagliptin fixed-dose combination with metformin is bioequivalent to free-pill combination therapy. Pharmacotherapy. 2012;32(10):e238. doi: 10.1002/j.1875-9114.2012.01219. PubMed PMID: CN-01024851.

6676. Busco S, Tavilla A, Gigli A, Lopez T, Pierannunzio D, Mallone S, et al. A direct method for the identification of patterns of care using administrative databases: the case of breast cancer. European Journal of Health Economics. 2021;22(9):1477-85. doi: 10.1007/s10198-021-01327-8. PubMed PMID: WOS:000678425800001.

6677. Bush AA, Amechi M, Persky A. An exploration of pharmacy education researchers' perceptions and experiences conducting qualitative research. American Journal of Pharmaceutical Education. 2020;84(3).

6678. Bush J. Response to: Supporting adherence for people starting a new medication for a long-term condition through community pharmacies: A pragmatic randomised controlled trial of the New Medicine Service' by Elliott et al. BMJ Quality and Safety. 2017;26(4):e10. doi: 10.1136/bmjqs-2016-005465.

6679. Bush J, Langley CA, Wilson KA. The corporatization of community pharmacy: implications for service provision, the public health function, and pharmacy's claims to professional status in the United Kingdom. Research in social and administrative pharmacy. 2009;5(4):305-18.

6680. Bushardt RL, Massey EB, Simpson TW, Ariail JC, Simpson KN. Polypharmacy: Misleading, but manageable. Clinical Interventions in Aging. 2008;3(2):383-9. doi: 10.2147/cia.S2468. PubMed PMID: WOS:000208332400017.

6681. Bushell MJA, Ball PA. Managing vaccine-associated anaphylaxis in the pharmacy. Journal of Pharmacy Practice and Research. 2015;45(1):24-30. doi: 10.1002/jppr.1053.

6682. Bushett NJ, Dickson-Swift VA, Willis JA, Wood P. Rural Australian community pharmacists' views on complementary and alternative medicine: A pilot study. BMC Complementary and Alternative Medicine. 2011;11. doi: 10.1186/1472-6882-11-103.

6683. Bushra R, Ahmad M, Seidi F, Qurtulen, Song J, Jin Y, et al. Polysaccharide-based nanoassemblies: From synthesis methodologies and industrial applications to future prospects. Advances in Colloid and Interface Science. 2023;318:102953. doi: https://doi.org/10.1016/j.cis.2023.102953.

6684. Büsing C, Gersing T, Koster AMCA. Planning out-of-hours services for pharmacies. Operations Research for Health Care. 2020;27:100277. doi: https://doi.org/10.1016/j.orhc.2020.100277.

6685. Buss VH, Deeks LS, Shield A, Kosari S, Naunton M. Analytical quality and effectiveness of point-of-care testing in community pharmacies: A systematic literature review. Research in Social and Administrative Pharmacy. 2019;15(5):483-95. doi: 10.1016/j.sapharm.2018.07.013.

6686. Buss VH, Shield A, Kosari S, Naunton M. Quality Use of the Pathology Data in Home Medicines Reviews: A Retrospective Evaluation. Annals of Pharmacotherapy. 2018;52(10):992-9. PubMed PMID: 29770700.

6687. Buss VH, Shield A, Kosari S, Naunton M. The impact of clinical services provided by community pharmacies on the Australian healthcare system: A review of the literature. Journal of Pharmaceutical Policy and Practice. 2018;11(1). doi: 10.1186/s40545-018-0149-7.

6688. Buss VH, Shield A, Kosari S, Peterson GM, Naunton M. Exploring pharmacists' views on an in-pharmacy medication review program in Australia using a mixed-methods design. International Journal of Clinical Pharmacy. 2020;42(6):1385-95. PubMed PMID: 32803556.

6689. Bussard BE, McAlearney AS, Pedersen CA, Schneider PJ. Adoption of technology to improve medication safety: perspectives of pharmacy directors. Journal of Patient Safety. 2006;2(4):217-24. doi: 10.1097/01.jps.0000236914.48955.99. PubMed PMID: 109848971. Language: English. Entry Date: 20080418. Revision Date: 20210407. Publication Type: Journal Article.

6690. Busse SR. En busca de otros caminos: Transcending medical boundaries in Cienfuegos, Cuba [M.A.]. Canada -- Ontario, CA: University of Guelph (Canada); 2001.

6691. Bussicott JA. The Use and Perceived Effectiveness of Complementary Medicines for Weight Loss [M.Tech.]. South Africa: University of Johannesburg (South Africa); 2018.

6692. Bussières JF, St-Arnaud C, Schunck C, Lamarre D, Jouberton F. The role of the pharmacist in humanitarian aid in Bosnia-Herzegovina: The experience of Pharmaciens Sans Frontieres. Annals of Pharmacotherapy. 2000;34(1):112-8. doi: 10.1345/aph.19157.

6693. Bussieres JF, St-Arnaud C, Schunck C, Lamarre D, Jouberton F. The rose of the pharamacist in humanitarian aid in Bosnia-Herzegovina: The experience of Pharmaciens Sans Frontieres. Annals of Pharmacotherapy. 2000;34(1):112-8. PubMed PMID: WOS:000084836900016.

6694. Bussing R, Koro-Ljungberg M, Gurnani T, Garvan CW, Mason D, Noguchi K, et al. Willingness to Use ADHD Self-Management: Mixed Methods Study of Perceptions by Adolescents and Parents. Journal of Child and Family Studies. 2016;25(2):562-73. doi: 10.1007/s10826-015-0241-4. PubMed PMID: WOS:000373576500018.

6695. Bustamante ND, Golden CD, Randrianasolo JF, Parmar P. A qualitative evaluation of health care in the Maroantsetra region of Madagascar. International Health. 2019;11(3):185-92. doi: 10.1093/inthealth/ihy070.

6696. Busto UE, Sproule BA, Knight K, Herrmann N. Use of prescription and nonprescription hypnotics in a Canadian elderly population. Canadian Journal of Clinical Pharmacology. 2001;8(4):213-21.

6697. Bustreo F, Harding A, Axelsson H. Can developing countries achieve adequate improvements in child health outcomes without engaging the private sector? Bulletin of the World Health Organization. 2003;81(12):886-95.

6698. Busuttil CA, Wirth F, Azzopardi LM. Establishing a community pharmacist-led vitamin D point-of-care testing service. Journal of the American College of Clinical Pharmacy. 2023;6(12):1330-5. doi: 10.1002/jac5.1867. PubMed PMID: WOS:001082451000001.

6699. Busza J, Douthwaite M, Bani R, Scutelniciuc O, Preda M, Simic D. Injecting behaviour and service use among young injectors in Albania, Moldova, Romania and Serbia. International Journal of Drug Policy. 2013;24(5):423-31. doi: https://doi.org/10.1016/j.drugpo.2013.03.004.

6700. Butkievich LE, Stacy ZA, Daly MW, Huey WY, Taylor CT. Impact of a student-supported pharmacy assessment program on venous thromboembolism prophylaxis rates in hospitalized patients. American Journal of Pharmaceutical Education. 2010;74(6):1-6. doi: 10.5688/aj7406105.

6701. Butler A, Dehner M, Gates RJ, Shane P, Chu M, DeMartini L, et al. Comprehensive Medication Management programs: 2015 status in Southern California. Research in Social and Administrative Pharmacy. 2017;13(1):63-87. doi: https://doi.org/10.1016/j.sapharm.2016.02.003.

6702. Butler AM, Brown DS, Durkin MJ, Sahrmann JM, Nickel KB, O'Neil CA, et al. Association of Inappropriate Outpatient Pediatric Antibiotic Prescriptions With Adverse Drug Events and Health Care Expenditures. JAMA Network Open. 2022;5(5):e2214153-e. doi: 10.1001/jamanetworkopen.2022.14153. PubMed PMID: 157127547. Language: English. Entry Date: 20220606. Revision Date: 20220606. Publication Type: Article.

6703. Butler AM, Brown DS, Newland JG, Nickel KB, Sahrmann JM, O'Neil CA, et al. Comparative safety and attributable healthcare expenditures following inappropriate versus appropriate outpatient antibiotic prescriptions among adults with upper respiratory infections. Clinical Infectious Diseases. 2023;76(6):986-95.

6704. Butler CC, Yu L-M, Dorward J, Gbinigie O, Hayward G, Saville BR, et al. Doxycycline for community treatment of suspected COVID-19 in people at high risk of adverse outcomes in the UK (PRINCIPLE): a randomised, controlled, open-label, adaptive platform trial. The Lancet Respiratory Medicine. 2021;9(9):1010-20.

6705. Butler CD. Medication therapy management for adverse drug event preventive care. Journal of the American Pharmacists Association: JAPhA. 2010;50(4):449. PubMed PMID: 20621858.

6706. Butler DA, Moolick K, McCray D, Gifford M. Compatibility of Omadacycline With Select Parenteral Products in Simulated Y-site Administration. Clinical Therapeutics. 2023;45(11):1137-41. doi: 10.1016/j.clinthera.2023.09.004. PubMed PMID: 174301709.

6707. Butler E, Collins C, McCarthy S. Extending pharmacy services to the point of discharge. Irish Medical Journal. 2021;114(1):1-9.

6708. Butler J, Yang M, Manzi MA, Hess GP, Patel MJ, Rhodes T, et al. Clinical Course of Patients With Worsening Heart Failure With Reduced Ejection Fraction. Journal of the American College of Cardiology. 2019;73(8):935-44. doi: https://doi.org/10.1016/j.jacc.2018.11.049.

6709. Butler KM, Huntington-Moskos L, Rayens MK, Wiggins AT, Hahn EJ. Perceived Synergistic Risk for Lung Cancer After Environmental Report-Back Study on Home Exposure to Tobacco Smoke and Radon. American journal of health promotion. 2019;33(4):597-600. doi: 10.1177/0890117118793886. PubMed PMID: CN-02010529.

6710. Butler KM, Rayens MK, Wiggins AT, Rademacher KB, Hahn EJ. Association of Smoking in the Home With Lung Cancer Worry, Perceived Risk, and Synergistic Risk. Oncology Nursing Forum. 2017;44(2):E55-E63. doi: 10.1188/17.Onf.E55-e63. PubMed PMID: WOS:000401160800002.

6711. Butler LM, Brown BT, Gupchup GV. Growing our own pharmacists: Pharmacy high school minority summer camp. Currents in Pharmacy Teaching and Learning. 2013;5(3):208-12. doi: 10.1016/j.cptl.2012.12.005.

6712. Butt F, Ream E. Implementing oral chemotherapy services in community pharmacies: A qualitative study of chemotherapy nurses' and pharmacists' views. International Journal of Pharmacy Practice. 2016;24(3):149-59. doi: 10.1111/ijpp.12237.

6713. Butt K, Nakhla N. Creating Standardized Tools for the Pharmacist-Led Assessment and Pharmacologic Management of Adult Canadians Wishing to Quit Smoking: A Consensus-Based Approach. Pharmacy. 2021;9(2). doi: 10.3390/pharmacy9020080. PubMed PMID: WOS:000665319200001.

6714. Butt M, Ali AM, Bakry MM. Health-related quality of life in poorly controlled type 2 diabetes patients- association of patients’ characteristics with EQ-5D domains, mean EQ-5D scores, and visaul analog scale score. Asian journal of pharmaceutical and clinical research. 2018;11(1):93-8. doi: 10.22159/ajpcr.2018.v11i1.19120. PubMed PMID: CN-01451608.

6715. Butt M, Mhd Ali A, Bakry MM, Mustafa N. Impact of a pharmacist led diabetes mellitus intervention on HbA1c, medication adherence and quality of life: a randomised controlled study. Saudi pharmaceutical journal. 2016;24(1):40-8. doi: 10.1016/j.jsps.2015.02.023. PubMed PMID: CN-01177833.

6716. Butt N, John J, Ettinger A, Gaur S, Weller A, Zonn S, et al. Pyrimethamine-induced seizure caused by compounding error. Clinical Toxicology. 2014;52(7):801. PubMed PMID: 71567312.

6717. Butt SZ, Ahmad M, Saeed H, Saleem Z, Javaid Z. Post-surgical antibiotic prophylaxis: impact of pharmacist's educational intervention on appropriate use of antibiotics. Journal of infection and public health. 2019;12(6):854-60. doi: 10.1016/j.jiph.2019.05.015. PubMed PMID: CN-02092544.

6718. Butt ZA, Gilani AH, Nanan D, Sheikh AL, White F. Quality of pharmacies in Pakistan: A cross-sectional survey. International Journal for Quality in Health Care. 2005;17(4):307-13. doi: 10.1093/intqhc/mzi049.

6719. Buttenheim AM, Fiks AG, Burson RC, Wang E, Coffin SE, Metlay JP, et al. A behavioral economics

intervention to increase pertussis vaccination among infant caregivers: a randomized feasibility trial. Vaccine. 2016;34(6):839-45. doi: 10.1016/j.vaccine.2015.11.068. PubMed PMID: CN-01138012.

6720. Butterworth J, Sansom A, Sims L, Healey M, Kingsland E, Campbell J. Pharmacists' perceptions of their emerging general practice roles in UK primary care: a qualitative interview study. British journal of general practice. 2017;67(662):e650-e8.

6721. Butts AR, Ellis CS, Travers EA, Reynolds JW, Rogers LE, Otto DE, et al. Implementation of a medication delivery service for ambulatory oncology infusion patients. Journal of Oncology Pharmacy Practice. 2023. doi: 10.1177/10781552231185527.

6722. Butz A, Kub J, Donithan M, James NT, Thompson RE, Bellin M, et al. Influence of caregiver and provider communication on symptom days and medication use for inner-city children with asthma. Journal of asthma. 2010;47(4):478-85. doi: 10.3109/02770901003692793. PubMed PMID: CN-00749212.

6723. Butz A, Morphew T, Lewis-Land C, Kub J, Bellin M, Ogborn J, et al. Factors associated with poor controller medication use in children with high asthma emergency department use. Annals of allergy, asthma and immunology (no pagination), 2017. 2017;Date of Publication: November 22. doi: 10.1016/ j.anai.2017.01.007. PubMed PMID: CN-01472048.

6724. Butz AM, Bollinger ME, Ogborn J, Morphew T, Mudd SS, Kub JE, et al. Children with poorly controlled asthma: randomized controlled trial of a home-based environmental control intervention. Pediatric pulmonology. 2019;54(3):245-56. doi: 10.1002/ppul.24239. PubMed PMID: CN-01707517.

6725. Butz AM, Halterman J, Bellin M, Kub J, Tsoukleris M, Frick KD, et al. Improving preventive care in high risk children with asthma: lessons learned. Journal of asthma. 2014;51(5):498-507. doi: 10.3109/02770903.2014.892608. PubMed PMID: CN-01000168.

6726. Butz AM, Ogborn J, Mudd S, Ballreich J, Tsoukleris M, Kub J, et al. Factors associated with high short-acting beta2-agonist use in urban children with asthma. Annals of allergy, asthma and immunology. 2015;114(5 // (NCATS) *National Institutes of Health* // R01 NR013486 (NIH) *National Institutes of Health* // (NIH) *National Institutes of Health* // (NINR) *National Institutes of Health*):385-92. doi: 10.1016/j.anai.2015.03.002. PubMed PMID: CN-01075266.

6727. Butz AM, Ogborn J, Mudd S, Ballreich J, Tsoukleris M, Kub J, et al. Factors associated with high short-acting β2-agonist use in urban children with asthma. Annals of allergy, asthma & immunology. 2015;114(5):385-92. doi: 10.1016/j.anai.2015.03.002. PubMed PMID: CN-01072951.

6728. Butz AM, Tsoukleris M, Donithan M, Hsu VD, Mudd K, Zuckerman IH, et al. Patterns of inhaled antiinflammatory medication use in young underserved children with asthma. Pediatrics. 2006;118(6):2504-13. doi: 10.1542/peds.2006-1630. PubMed PMID: CN-01774350.

6729. Buurma H, Bouvy M, De Smet P, Floor-Schreudering A, Leufkens H, Egberts A. Prevalence and determinants of pharmacy shopping behaviour. Journal of clinical pharmacy and therapeutics. 2008;33(1):17-23.

6730. Buurma H, De Smet P, Egberts A. Clinical risk management in community pharmacy. Huisarts en Wetenschap. 2007;50(12):623-7.

6731. Buurma H, De Smet P, Egberts ACG. Clinical risk management in Dutch community pharmacies - The case of drug-drug interactions. Drug Safety. 2006;29(8):723-32. doi: 10.2165/00002018-200629080-00009. PubMed PMID: WOS:000240114600009.

6732. Buurma H, De Smet P, Kruijtbosch M, Egberts AC. The occurrence and handling of drug-drug interactions in Dutch community pharmacies. Pharmacy World & Science. 2007;29(5):438-. PubMed PMID: WOS:000249408200003.

6733. Buurma H, De Smet P, Kruijtbosch M, Egberts ACG. Disease and intolerability documentation in electronic patient records. Annals of Pharmacotherapy. 2005;39(10):1640-6. doi: 10.1345/aph.1G071. PubMed PMID: WOS:000232209500008.

6734. Buurma H, De Smet P, Leufkens HGM, Egberts ACG. Evaluation of the clinical value of pharmacists' modifications of prescription errors. British Journal of Clinical Pharmacology. 2004;58(5):503-11. doi: 10.1111/j.1365-2125.2004.02181.x. PubMed PMID: WOS:000224865100008.

6735. Buurma H, de Smet P, van den Hoff OP, Egberts ACG. Nature, frequency and determinants of prescription modifications in Dutch community pharmacies. British Journal of Clinical Pharmacology. 2001;52(1):85-91. doi: 10.1046/j.0306-5251.2001.01406.x. PubMed PMID: WOS:000169719500011.

6736. Buurma H, de Smet P, van den Hoff OP, Sysling H, Storimans M, Egberts ACG. Frequency, nature and determinants of pharmacy compounded medicines in Dutch community pharmacies. Pharmacy World & Science. 2003;25(6):280-7. doi: 10.1023/B:PHAR.0000006521.41736.db. PubMed PMID: WOS:000186972800008.

6737. Buurma H, De Smet PA, Egberts AC. Clinical risk management in Dutch community pharmacies: the case of drug-drug interactions. Drug safety. 2006;29:723-32.

6738. Buurma H, De Smet PA, Kruijtbosch M, Egberts ACG. Disease and intolerability documentation in electronic patient records. Annals of Pharmacotherapy. 2005;39(10):1640-6. doi: 10.1345/aph.1G071.

6739. Buurma H, de Smet PA, van den Hoff OP, Egberts AC. Nature, frequency and determinants of prescription modifications in Dutch community pharmacies. British journal of clinical pharmacology. 2001;52(1):85-91. doi: 10.1046/j.0306-5251.2001.01406.x. PubMed PMID: CN-00349453.

6740. Buurma H, De Smet PAGM, Egberts ACG. Clinical risk management in Dutch community pharmacies: The case of drug-drug interactions. Drug Safety. 2006;29(8):723-32. doi: 10.2165/00002018-200629080-00009.

6741. Buurma H, De Smet PAGM, Leufkens HGM, Egberts ACG. Evaluation of the clinical value of pharmacists' modifications of prescription errors. British Journal of Clinical Pharmacology. 2004;58(5):503-11. doi: 10.1111/j.1365-2125.2004.02181.x.

6742. Buurma H, De Smet PAGM, Van Den Hoff OP, Egberts ACG. Nature, frequency and determinants of prescription modifications in Dutch community pharmacies. British Journal of Clinical Pharmacology. 2001;52(1):85-91. doi: 10.1046/j.0306-5251.2001.01406.x.

6743. Buurma H, Schalekamp T, Egberts AC, De Smet P. Compliance with national guidelines for the management of drug-drug interactions in dutch community pharmacies. Annals of Pharmacotherapy. 2007;41(12):2024-31. doi: 10.1345/aph.1K240. PubMed PMID: WOS:000251280500013.

6744. Buurma H, Schalekamp T, Egberts ACG, De Smet PAGM. Compliance with national guidelines for the management of drug-drug interactions in dutch community pharmacies. Annals of Pharmacotherapy. 2007;41(12):2024-31. doi: 10.1345/aph.1K240.

6745. Buurma H, Schalekamp T, Egberts TCG, De Smet PAG. Adherence to a guideline for the management of drug-drug interactions in Dutch community pharmacies. Pharmacy World & Science. 2008;30(5):665-.

PubMed PMID: WOS:000259443000082.

6746. Buus K, Harbigg P, Olesen C, Damsgaard EM. The effect of pharmaceutical care on three-year mortality a follow-up study. European geriatric medicine. 2013;4:S182-. doi: 10.1016/j.eurger.2013.07.610. PubMed PMID: CN-01006740.

6747. Buxeraud J. Implants dentaires : suivi et hygiène sont indispensables. Actualités Pharmaceutiques. 2014;53(537):45-7. doi: https://doi.org/10.1016/j.actpha.2014.03.018.

6748. Buxeraud J. L'hypersensibilité dentinaire. Actualités Pharmaceutiques. 2017;56(571):51-2. doi: https://doi.org/10.1016/j.actpha.2017.09.033.

6749. Buxeraud J. Prévention de la carie dentaire. Actualités Pharmaceutiques. 2017;56(568):51-4. doi: https://doi.org/10.1016/j.actpha.2017.05.020.

6750. Buxeraud J, Faure S. Recommandations officinales essentielles et formalités pour voyager à l'étranger. Actualités Pharmaceutiques. 2019;58(587, Supplement):15-7. doi: https://doi.org/10.1016/j.actpha.2019.03.028.

6751. Buxeraud J, Faure S, Picard N. Détection et analyse des interactions médicamenteuses à l'officine : une méthode simple et rapide. Actualités Pharmaceutiques. 2016;55(554, Supplement):1-4. doi: https://doi.org/10.1016/j.actpha.2015.12.007.

6752. Buxeraud J, Faure S, Picard N. Detection and analysis of drug interactions in the pharmacy: A fast and simple method. Actualites Pharmaceutiques. 2016;55(554):1-4. doi: 10.1016/j.actpha.2015.12.007.

6753. Buxeraud J, Huck O. Traitement ambulatoire de l'hypersensibilité dentinaire. Actualités Pharmaceutiques. 2016;55(556):47-50. doi: https://doi.org/10.1016/j.actpha.2016.03.011.

6754. Buxeraud J, Vuillet-A-Ciles H. Les hormones de croissance à l'officine. Actualités Pharmaceutiques. 2014;53(532):48-52. doi: https://doi.org/10.1016/j.actpha.2013.10.034.

6755. Buxton JA, Kuo ME, Ramji S, Yu A, Krajden M. Methadone use in relation to hepatitis C virus testing in British Columbia. Canadian Journal of Public Health. 2010;101(6):491-4. doi: 10.1007/bf03403970.

6756. Buxton JA, Preston EC, Mak S, Harvard S, Barley J. More than just needles: An evidence-informed approach to enhancing harm reduction supply distribution in British Columbia. Harm Reduction Journal. 2008;5. doi: 10.1186/1477-7517-5-37.

6757. Buxton JA, Preston EC, Mak S, Harvard S, Barley J, Strategies BCHR, et al. More than just needles: An evidence-informed approach to enhancing harm reduction supply distribution in British Columbia. Harm Reduction Journal. 2008;5. doi: 10.1186/1477-7517-5-37. PubMed PMID: WOS:000207448600037.

6758. Buyse M, George SL, Evans S, Geller NL, Ranstam J, Scherrer B, et al. The role of biostatistics in the prevention, detection and treatment of fraud in clinical trials. Statistics in medicine. 1999;18(24):3435-51.

6759. Buzancic I, Balic P, Pejakovic TI, Markulin L, Hadziabdic MO. Can community pharmacists be elderly patients' bridge to deprescribing? A cross-sectional study. International Journal of Clinical Pharmacy. 2023;45(2):515-6. doi: https://dx.doi.org/10.1007/s11096-023-01576-y. PubMed PMID: 641388242.

6760. Buzancic I, Dragovic P, Pejakovic TI, Markulin L, Ortner-Hadziabdic M. Exploring Patients' Attitudes Toward Deprescribing and Their Perception of Pharmacist Involvement in a European Country: A Cross-Sectional Study. Patient Preference and Adherence. 2021;15:2197-208. doi: 10.2147/ppa.S323846. PubMed

PMID: WOS:000713606900001.

6761. Bužančić I, Ortner Hadžiabdić M. Deprescribing in a multimorbid older adult: A case vignette study among community pharmacists and primary care physicians. Basic & Clinical Pharmacology & Toxicology. 2023;133(6):729-40.

6762. Buzancic I, Pejakovic TI, Hadziabdic MO. A Need for Benzodiazepine Deprescribing in the COVID-19 Pandemic: A Cohort Study. Pharmacy. 2022;10(5). doi: 10.3390/pharmacy10050120. PubMed PMID: WOS:000875233300001.

6763. Buzas B, Smith J, Gilbert GE, Moureau N. Home infusion pharmacy quality improvement for central venous access devices using anti-reflux needleless connectors to reduce occlusions, emergency room visits, and alteplase costs. American Journal of Health-System Pharmacy. 2022;79(13):1079-85. doi: 10.1093/ajhp/zxac083. PubMed PMID: 157640918. Language: English. Entry Date: 20220706. Revision Date: 20220706. Publication Type: Article.

6764. Buzási A. Comparative assessment of heatwave vulnerability factors for the districts of Budapest, Hungary. Urban Climate. 2022;42:101127. doi: https://doi.org/10.1016/j.uclim.2022.101127.

6765. bw2wt RBR. The role of the pharmacist in the team care of hospitalized patients with Chronic Brochitis or Emphysema. https://trialsearchwhoint/Trial2aspx?TrialID=RBR-5bw2wt. 2012. PubMed PMID: CN-01846781.

6766. Bychkova V, Shephard A, Woolfson J, Burova N, Radkova E. Rapid, clinically meaningful relief from sore throat symptoms with flurbiprofen 8.75 mg delivered as a lozenge or spray. International journal of clinical pharmacy. 2017;39(1):324-. doi: 10.1007/s11096-016-0404-4. PubMed PMID: CN-01333872.

6767. Byerley EM, Dykhuizen SN, Haak JR, Grindeland CJ, Muzzy Williamson JD. Medication Management in Lactation—A Pharmacist Education Pilot Project: Part one survey. Journal of the American Pharmacists Association. 2022;62(4):1389-93.e2. doi: https://doi.org/10.1016/j.japh.2022.03.003.

6768. Byerley EM, Dykhuizen SN, Haak JR, Grindeland CJ, Muzzy Williamson JD. Medication Management in Lactation-A Pharmacist Education Pilot Project: Part one survey. Journal of the American Pharmacists Association: JAPhA. 2022;62(4):1389-93.e2. PubMed PMID: 35339393.

6769. Bylsma T, Grossman K, Vindenes T. 1565. Antiretroviral Stewardship: Protocol Development and Implementation at an Academic Medical Center. Open Forum Infectious Diseases. 2023;10(Supplement 2):S723. doi: https://dx.doi.org/10.1093/ofid/ofad500.1400. PubMed PMID: 643249783.

6770. Bynum A, Hopkins D, Thomas A, Copeland N, Irwin C. The effect of telepharmacy counseling on metered-dose inhaler technique among adolescents with asthma in rural Arkansas. Telemedicine journal and e-health. 2001;7(3):207-17. doi: 10.1089/153056201316970902. PubMed PMID: CN-00356483.

6771. Byrd JB, Maddox TM, O'Donnell CI, Grunwald GK, Bhatt DL, Tsai TT, et al. Clopidogrel prescription filling delays and cardiovascular outcomes in a pharmacy system integrating inpatient and outpatient care: Insights from the Veterans Affairs CART Program. American Heart Journal. 2014;168(3):340-5. doi: https://doi.org/10.1016/j.ahj.2014.05.012.

6772. Byrd JE. Impact of medication stressors on emotional health of informal caregivers of demented patients [Ph.D.]. United States -- North Carolina: The University of North Carolina at Chapel Hill; 2007.

6773. Byrd KK, Hardnett F, Clay PG, Delpino A, Hazen R, Shankle MD, et al. Retention in HIV Care among Participants in the Patient-Centered HIV Care Model: A Collaboration between Community-Based

Pharmacists and Primary Medical Providers. AIDS Patient Care and STDs. 2019;33(2):58-66. doi: 10.1089/apc.2018.0216.

6774. Byrn SR, Zografi G, Chen X. Accelerating Proof of Concept for Small Molecule Drugs Using Solid-State Chemistry. Journal of Pharmaceutical Sciences. 2010;99(9):3665-75. doi: https://doi.org/10.1002/jps.22215.

6775. Byrne A. Natural health products (NHPs) and Canadian pharmacy students: Core competencies [M.Sc.]. Canada -- Ontario, CA: University of Toronto (Canada); 2009.

6776. Byrne A, Byrne S, Dalton K. A pharmacist's unique opportunity within a multidisciplinary team to reduce drug-related problems for older adults in an intermediate care setting. Research in Social and Administrative Pharmacy. 2022;18(4):2625-33. doi: https://doi.org/10.1016/j.sapharm.2021.05.003.

6777. Byrne A, Ludin NHH, Byrne S, Purcell R. 108 A Prospective Audit and Review of Proton Pump Inhibitors (PPIs) in Older Adults in a Reablement Unit...67th Annual & Scientific Meeting of the Irish Gerontological Society, Innovation, Advances and Excellence in Ageing, 26–28 September 2019, Cork, Ireland. Age & Ageing. 2019;48:iii17-iii65. doi: 10.1093/ageing/afz103.63. PubMed PMID: 138865049. Language: English. Entry Date: 20191003. Revision Date: 20200901. Publication Type: Article.

6778. Byrne AE, Redmayne GM, Lam T, Tran J, Chan DK. Implementation and evaluation of a pharmacist-led oral anticancer medication management clinic. Journal of Pharmacy Practice & Research. 2018;48(6):509-15. doi: 10.1002/jppr.1429. PubMed PMID: 133581481. Language: English. Entry Date: 20181224. Revision Date: 20191202. Publication Type: Article.

6779. Byrne B, Diaz-Manera J, Goker-Alpan O, Mozaffar T, Wasfi Y, Das SS, et al. P372 Safety of home administration of cipaglucosidase alfa + miglustat in late-onset Pompe disease: results from multiple clinical trials. Neuromuscular Disorders. 2023;33:S148-S. doi: 10.1016/j.nmd.2023.07.336. PubMed PMID: 172304784.

6780. Byrne C, Radley A, Inglis SK, Beer LJZ, Palmer N, Pham MD, et al. Reaching mEthadone users Attending Community pHarmacies with HCV: an international cluster randomised controlled trial protocol (REACH HCV). BMJ open. 2020;10(8):e036501. doi: 10.1136/bmjopen-2019-036501. PubMed PMID: CN-02177151.

6781. Byrne CJ, Beer L, Inglis SK, Robinson E, Radley A, Goldberg DJ, et al. Real-world outcomes of rapid regional hepatitis C virus treatment scale-up among people who inject drugs in Tayside, Scotland. Alimentary Pharmacology and Therapeutics. 2022;55(5):568-79. doi: 10.1111/apt.16728.

6782. Byrne CJ, Cahir C, Curran C, Bennett K. High-risk prescribing in an Irish primary care population: trends and variation. British Journal of Clinical Pharmacology. 2017;83(12):2821-30. doi: 10.1111/bcp.13373.

6783. Byrne CJ, Radley A, Inglis SK, Beer L, Palmer N, Duc Pham M, et al. Reaching people receiving opioid agonist therapy at community pharmacies with hepatitis C virus: an international randomised controlled trial. Alimentary pharmacology & therapeutics. 2022;55(12):1512-23. doi: 10.1111/apt.16953. PubMed PMID: CN-02400432.

6784. Byrne CJ, Radley A, Inglis SK, Beer L, Palmer N, Duc Pham M, et al. Reaching people receiving opioid agonist therapy at community pharmacies with hepatitis C virus: an international randomised controlled trial. Alimentary Pharmacology and Therapeutics. 2022;55(12):1512-23. doi: 10.1111/apt.16953.

6785. Byrne CJ, Radley A, Inglis SK, Beer L, Palmer N, Pham MD, et al. Reaching people receiving opioid

agonist therapy at community pharmacies with hepatitis C virus: An international randomised controlled trial. Alimentary Pharmacology & Therapeutics. 2022;55(12):1512-23. doi: 10.1111/apt.16953. PubMed PMID: WOS:000792939500001.

6786. Byrne CJ, Walsh C, Cahir C, Bennett K. Impact of drug burden index on adverse health outcomes in Irish community-dwelling older people: A cohort study. BMC Geriatrics. 2019;19(1). doi: 10.1186/s12877-019-1138-7.

6787. Byrne CJ, Walsh C, Cahir C, Ryan C, Williams DJ, Bennett K. Anticholinergic and sedative drug burden in community-dwelling older people: a national database study. Bmj Open. 2018;8(7). doi: 10.1136/bmjopen-2018-022500. PubMed PMID: WOS:000446181900214.

6788. Byrne GA, Wood PJ, Spark MJ. Non-prescription supply of combination analgesics containing codeine in community pharmacy: A simulated patient study. Research in Social and Administrative Pharmacy. 2018;14(1):96-105. doi: https://doi.org/10.1016/j.sapharm.2017.01.005.

6789. Byrne ME, Omoluabi E, OlaOlorun FM, Moreau C, Bell SO. Determinants of women’s preferred and actual abortion provision locations in Nigeria. Reproductive Health. 2021;18(1). doi: 10.1186/s12978-021-01290-w.

6790. Byrne S, Grimes T, Jago-Byrne M-C, Galvin M, Byrne SM, Grimes TC, et al. Impact of team-versus ward-aligned clinical pharmacy on unintentional medication discrepancies at admission. International Journal of Clinical Pharmacy. 2017;39(1):148-55. doi: 10.1007/s11096-016-0412-4. PubMed PMID: 121185709. Language: English. Entry Date: 20180720. Revision Date: 20180728. Publication Type: journal article.

6791. Byrne S, Hansen CR, Sahm LJ, O'Mahony D, Kearney PM. Qualitative analysis of community pharmacists' opinions on their involvement in reducing potentially inappropriate prescribing. European Journal of Clinical Pharmacology. 2019;75(2):265-74. doi: 10.1007/s00228-018-2578-2. PubMed PMID: 134311059. Language: English. Entry Date: 20190130. Revision Date: 20200203. Publication Type: Article.

6792. Byrne SM, Grimes TC, Jago-Byrne MC, Galvin M. Impact of team-versus ward-aligned clinical pharmacy on unintentional medication discrepancies at admission. International Journal of Clinical Pharmacy. 2017;39(1):148-55. PubMed PMID: 28004239.

6793. Byrt S. Social, medical and geographical aspects of the provision of community pharmacy services in rural west wales [Ph.D.]. Wales: University of Wales Trinity Saint David (United Kingdom); 1998.

6794. Byshenko VV, Knysh OI, Zadirachenko LN, Egorova AO, Rodina YS. The current state of the veterinary drugs market in the Tyumen Region. Farmakoekonomika. 2022;15(2):267-83. doi: 10.17749/2070-4909/farmakoekonomika.2022.133.

6795. Byström J. Telepharmacy- a Potential Method of Drug Dispensing in Sweden? an Evaluation of a Pilot Project in a Swedish Pharmacy [Master's]. Sweden: Uppsala Universitet (Sweden); 2021.

6796. Bzowyckyj AS, Crabtree B, Franson K, Klepser D, Okere AN, Poirier T, et al. Report of the 2019-2020 aacp academic affairs committee: Developing the pharmacist workforce for society’s medication use needs in 2030. American Journal of Pharmaceutical Education. 2020;84(10):1429-39.

6797. c7n8g RBR. The Effects of Audiovisual Neurostimulation in women with Fibromyalgia. https://trialsearchwhoint/Trial2aspx?TrialID=RBR-67c7n8g. 2021. PubMed PMID: CN-02352186.

6798. c53f3m RBR. Implementation of clinical pharmacy services into Brazilian healthcare system. https://trialsearchwhoint/Trial2aspx?TrialID=RBR-4c53f3m. 2022. PubMed PMID: CN-02457309.

6799. Caamaño F, Ruano A, Figueiras A, Gestal-Otero JJ. Data collection methods for analyzing the quality of the dispensing in pharmacies. Pharmacy World and Science. 2002;24(6):217-23. doi: 10.1023/A:1021808923928.

6800. Caamaño F, Ruano A, Figueiras A, Gestal-Otero J. Data collection methods for analyzing the quality of the dispensing in pharmacies. Pharmacy World and Science. 2002;24:217-23.

6801. Caamaño F, Tomé-Otero M, Takkouche B, Gestal-Otero JJ. Influence of pharmacists' opinions on their dispensing medicines without requirement of a doctor's prescription. Gaceta sanitaria. 2005;19(1):9-14.

6802. Caballer Tarazona V, Guadalajara Olmeda N, Vivas Consuelo D, Clemente Collado A. Impact of Morbidity on Health Care Costs of a Department of Health through Clinical Risk Groups. Valencian Community, Spain. Revista espanola de salud publica. 2016;90:e1-e15.

6803. Caballero J, Jacobs RJ, Ownby RL. Development of a computerized intervention to improve health literacy in older Hispanics with type 2 diabetes using a pharmacist supervised comprehensive medication management. PLoS ONE [Electronic Resource]. 2022;17(2):e0263264. PubMed PMID: 35139107.

6804. Caballero-Romero Á, Fernández S, Morillo AB, Zaragoza-Rascón M, Jaramillo-Pérez C, Del Pozo-Rubio R. Healthcare failure mode and effects analysis and cost-minimization analysis of three pharmaceutical services. Farmacia Hospitalaria. 2021;45(2):66-72. doi: 10.7399/fh.11532.

6805. Caballero-Romero A, Fernandez S, Morillo AB, Zaragoza-Rascon M, Jaramillo-Perez C, Del Pozo-Rubio R. Healthcare failure mode and effects analysis and cost-minimization analysis of three pharmaceutical services. Farmacia Hospitalaria. 2021;45(2):66-72. doi: 10.7399/fh.11532. PubMed PMID: WOS:000628914800005.

6806. Cabana F, Beaulieu M, Lambert D, Cote PY, Vaillancourt M, Beaulieu MC, et al. Incidence of subsequent fractures according to age and site of initial non-vertebral fractures: the first 3 years of the optimus initiative. Journal of bone and mineral research. 2011;26. PubMed PMID: CN-01057318.

6807. Cabanas BN. Increasing the Utilization of Psychologists in Healthcare through Interprofessional Education [Psy.D.]. United States -- New Jersey: Rutgers The State University of New Jersey, Graduate School of Applied and Professional Psychology; 2022.

6808. Cabanillas M, Chimenti EM, Candela CG, Kohen VL, Dassen C, Lajo T. USEFULNESS OF MEAL REPLACEMENT: ANALYSIS OF THE PRINCIPAL MEAL REPLACEMENT PRODUCTS COMMERCIALISED IN SPAIN. Nutricion Hospitalaria. 2009;24(5):535-42. doi: 10.3305/nh.2009.24.5.4464. PubMed PMID: WOS:000270524300004.

6809. Cabas P, Di Bella S, Giuffrè M, Rizzo M, Trombetta C, Luzzati R, et al. Community pharmacists' exposure to COVID-19. Research in Social and Administrative Pharmacy. 2021;17(1):1882-7. doi: https://doi.org/10.1016/j.sapharm.2020.05.020.

6810. Cabello-Muriel A, Gascón-Cánovas JJ, Urbieta-Sanz E, Iniesta-Navalón C. Effectiveness of pharmacist intervention in patients with chronic kidney disease. International journal of clinical pharmacy. 2014;36(5):896-903. doi: 10.1007/s11096-014-0001-3. PubMed PMID: CN-01050576.

6811. Cabezas MD. Current trend in pharmaceutical law in EU. Pharmaceuticals Policy and Law. 2012;14(2-4):195-207. doi: 10.3233/PPL-120354.

6812. Cabilan C, Hughes JA, Shannon C. The use of a contextual, modal and psychological classification of medication errors in the emergency department: a retrospective descriptive study. Journal of clinical nursing.

2017;26(23-24):4335-43.

6813. Cabilan CJ, Boyde M, Currey E. The effectiveness of pharmacist- led discharge medication counselling in the emergency department (ExPLAIN): A pilot quasi-experimental study. Patient Education and Counseling. 2019;102(6):1157-63. doi: https://doi.org/10.1016/j.pec.2019.01.020.

6814. Cabral AC, Lavrador M, Fernandez-Llimos F, Figueiredo IV, Castel-Branco M. Implementation of the medication use review service in a community pharmacy: An open door to other pharmaceutical services. Pharmacy Education. 2023;23(5):15-6. doi: https://dx.doi.org/10.46542/pe.2023.235.138. PubMed PMID: 642369396.

6815. Cabral AC, Moura-Ramos M, Castel-Branco M, Caramona M, Fernandez-Llimos F, Figueiredo IV. Influence of the mode of administration on the results of medication adherence questionnaires. Journal of Evaluation in Clinical Practice. 2017;23(6):1252-7. doi: 10.1111/jep.12773. PubMed PMID: WOS:000418713100016.

6816. Cabral AC, Moura-Ramos M, Castel-Branco M, Fernandez-Llimos F, Figueiredo IV. Cross-cultural adaptation and validation of a European Portuguese version of the 8-item Morisky medication adherence scale. Revista Portuguesa de Cardiologia. 2018;37(4):297-303. doi: 10.1016/j.repc.2017.09.017.

6817. Cabral ACG. Evaluation of Risk Factors Associated With Uncontrolled Blood Pressure of Hypertensive Patients Under Pharmacological Antihypertensive Treatment [Ph.D.]. Portugal: Universidade de Coimbra (Portugal); 2016.

6818. Cabral LFQ. Farmacocinética de Proteínas Terapêuticas em Doenças Neurológicas: Experiência Profissionalizante na Vertente de Farmácia Comunitária e Farmácia Hospitalar [Master's]. Portugal: Universidade da Beira Interior (Portugal); 2013.

6819. Cabral S, Jara J, Goyret A, Castro M, Fabbiani S, Garafoni F, et al. Potentially inappropriate medication use in older adults assisted as outpatients in ASSE's Metropolitan RAP (Primary Health Care) during 2019. Revista Medica Del Uruguay. 2023;39(1). doi: 10.29193/rmu.39.1.3. PubMed PMID: WOS:000946470200005.

6820. Cabrera AG, Sanz-Lorente M, Sanz-Valero J, López-Pintor E. Compliance and adherence to enteral nutrition treatment in adults: A systematic review. Nutrients. 2019;11(11). doi: 10.3390/nu11112627.

6821. Cabrera-Aguas M, Robaei D, McCluskey P, Watson S. Clinical translation of recommendations from randomized trials for management of herpes simplex virus keratitis. Clinical & experimental ophthalmology. 2018;46(9):1008-16. doi: 10.1111/ceo.13319. PubMed PMID: CN-01612013.

6822. Cabri A, Barsegyan N, Postelnick M, Schulz L, Nguyen V, Szwak J, et al. Pharmacist intervention on prescribing errors: Use of a standardized approach in the inpatient setting. American Journal of Health-System Pharmacy. 2021;78(23):2151-8. doi: 10.1093/ajhp/zxab278. PubMed PMID: 153798213. Language: English. Entry Date: 20211201. Revision Date: 20221201. Publication Type: Article.

6823. Cabri AE, King A, Morrow L, Malesker MA. Pharmacists can help prevent lipoid pneumonia: Two case reports. Journal of the American Pharmacists Association. 2017;57(5):616-8. doi: https://doi.org/10.1016/j.japh.2017.05.012.

6824. Caburnay CA, Babb P, Kaphingst KA, Roberts J, Rath S. Characterlistics of Genetics-Related News Content in Black Weekly Newspapers. Public Health Genomics. 2014;17(1):7-15. doi: 10.1159/000354867. PubMed PMID: WOS:000331026700002.

6825. Cáceres Guido P, Riva N, Calle G, Dell'Orso M, Gatto M, Sberna N, et al. Medicinal cannabis in Latin America: History, current state of regulation, and the role of the pharmacist in a new clinical experience with cannabidiol oil. Journal of the American Pharmacists Association. 2020;60(1):212-5. doi: https://doi.org/10.1016/j.japh.2019.09.012.

6826. Caceres R, Guillen J, Gonzalez-Inchausti C, Sobrino MG. Invasive meningococcal disease burden and prophylaxis: update on serogroup B vaccination. Farmaceuticos Comunitarios. 2021;13(4):28-42. doi: 10.33620/FC.2173-9218.(2021/Vol13).004.05. PubMed PMID: WOS:000730750300005.

6827. Cada DJ, Baker DE, Levien T. Meloxicam. Hospital pharmacy. 2000;35(8):852-65. PubMed PMID: CN-01737000.

6828. Cada DJ, Levien TL, Baker DE. Sugammadex. Hospital pharmacy. 2016;51(7):585-96. doi: 10.1310/hpj5107-585. PubMed PMID: CN-01210459.

6829. Cada DJ, Mbogu U, Bindler RJ, Baker DE. Uridine triacetate. Hospital pharmacy 51 (6) (pp 484-488), 2016 Date of publication: june 2016. 2016. doi: 10.1310/hpj5106-484. PubMed PMID: CN-01194961.

6830. Cadarette SM, Jaglal SB, Raman-Wilms L, Beaton DE, Paterson JM. Osteoporosis quality indicators using healthcare utilization data. Osteoporosis International. 2011;22(5):1335-42. doi: 10.1007/s00198-010-1329-8.

6831. Cadarette SM, Levesque L, Mamdani MM, Perreault S, Juurlink DN, Paterson JM, et al. Beware of policy-induced selection bias in drug effect studies: example in the comparative effectiveness of oral bisphosphonates. Journal of population therapeutics and clinical pharmacology. 2012;19(2):e116. PubMed PMID: CN-01030029.

6832. Cadarette SM, Wong L. An introduction to health care administrative data. The Canadian journal of hospital pharmacy. 2015;68(3):232.

6833. Cadavid D, Balcer L, Galetta S, Aktas O, Ziemssen T, Vanopdenbosch L, et al. Safety and efficacy of opicinumab in acute optic neuritis (RENEW): a randomised, placebo-controlled, phase 2 trial. The lancet Neurology. 2017;16(3):189-99. doi: 10.1016/S1474-4422(16)30377-5. PubMed PMID: CN-01328045.

6834. Cadavid D, Balcer L, Galetta S, Aktas O, Ziemssen T, Vanopdenbosch L, et al. Safety and efficacy of opicinumab in acute optic neuritis (RENEW): a randomised, placebo-controlled, phase 2 trial. The Lancet Neurology. 2017;16(3):189-99. doi: https://doi.org/10.1016/S1474-4422(16)30377-5.

6835. Cadilhac DA, Kilkenny MF, Johnson R, Wilkinson B, Amatya B, Lalor E. The Know Your Numbers (KYN) program 2008 to 2010: Impact on knowledge and health promotion behavior among participants. International Journal of Stroke. 2015;10(1):110-6. doi: 10.1111/ijs.12018.

6836. Cadman B, Wright D, Bale A, Barton G, Desborough J, Hammad EA, et al. Pharmacist provided medicines reconciliation within 24 hours of admission and on discharge: a randomised controlled pilot study. BMJ open. 2017;7(3):e013647. doi: 10.1136/bmjopen-2016-013647. PubMed PMID: CN-01443587.

6837. Cadman B, Wright D, Bale A, Barton G, Desborough J, Hammad EA, et al. Pharmacist provided medicines reconciliation within 24hours of admission and on discharge: a randomised controlled pilot study. BMJ open. 2017;7(3) (no pagination). doi: 10.1136/bmjopen-2016-013647. PubMed PMID: CN-01336936.

6838. Cadogan C, Ryder S. An audit of prescribing practices for benzodiazepines and Z-Drugs. Irish Medical Journal. 2015;108(3).

6839. Cadogan CA, Hughes CM. On the frontline against COVID-19: Community pharmacists' contribution during a public health crisis. Research in Social and Administrative Pharmacy. 2021;17(1):2032-5.

6840. Cadogan CA, Ryan C, Francis JJ, Gormley GJ, Passmore P, Kerse N, et al. Improving appropriate polypharmacy for older people in primary care: selecting components of an evidence-based intervention to target prescribing and dispensing. Implementation Science. 2015;10(1):1-14.

6841. Cadogan CA, Ryan C, Hughes C. Making the case for change: What researchers need to consider when designing behavior change interventions aimed at improving medication dispensing. Research In Social & Administrative Pharmacy. 2016;12(1):149-53. PubMed PMID: 25936272.

6842. Cadogan CA, Ryder SA. Community pharmacists' role in benzodiazepine supply in Ireland: a questionnaire of pharmacists' views and experiences. International Journal of Pharmacy Practice. 2015;23(6):415-22. PubMed PMID: 25756294.

6843. Cadwell C, Blom J, Kuehl P. Evaluation of a heart failure service provided by community pharmacists to improve quality of life and reduce utilization of health services. Journal of the American Pharmacists Association. 2013;53(2):e78-e9. PubMed PMID: 71322588.

6844. Cady RK, Goldstein J, Silberstein S, Juhász M, Ramsey K, Rodgers A, et al. Expanding access to triptans: assessment of clinical outcome. Headache. 2009;49(10):1402-13. doi: 10.1111/j.1526-4610.2009.01532.x. PubMed PMID: CN-00786646.

6845. Caetano ML, Pawasauskas J. A retrospective analysis of the impact of disabling item review on item performance on computerized fixed-item tests in a doctor of pharmacy program. Currents in Pharmacy Teaching and Learning. 2020;12(5):539-43. doi: https://doi.org/10.1016/j.cptl.2020.01.018.

6846. Caetano S, Reis I, Galego C, Conceicao L. Community Pharmacy Intervention in the Evaluation and Prevention of Hypertension and in the Identification of Hypertensive Patients. International Journal of Clinical Pharmacy. 2021;43(3):786-. PubMed PMID: WOS:000663530400083.

6847. Caffiero N, Delate T, Ehizuelen MD, Vogel K. Effectiveness of a Clinical Pharmacist Medication Therapy Management Program in Discontinuation of Drugs to Avoid in the Elderly. Journal of Managed Care & Specialty Pharmacy. 2017;23(5):525-31. PubMed PMID: 28448783.

6848. Caffiero NA, Nickman NA, Drews FA, King JB, Moorman K, Tyler LS. Reduction of phone interruptions post implementation of a central call center in community pharmacies of an academic health system. American Journal of Health-System Pharmacy. 2021;78(2):113-21. doi: 10.1093/ajhp/zxaa363.

6849. Caffrey AR, DeAngelis JM, Ward KE, Orr KK, Morrill HJ, Gosciminski M, et al. A pharmacist-driven academic detailing program to increase adult pneumococcal vaccination. Journal of the American Pharmacists Association. 2018;58(3):303-10. doi: https://doi.org/10.1016/j.japh.2017.08.010.

6850. Caffrey AR, DeAngelis JM, Ward KE, Orr KK, Morrill HJ, Gosciminski M, et al. A pharmacist-driven academic detailing program to increase adult pneumococcal vaccination. Journal of the American Pharmacists Association. 2018;58(3):303-10. doi: 10.1016/j.japh.2017.08.010. PubMed PMID: WOS:000432472900015.

6851. Caglar S, Henneman PL, Blank FS, Smithline HA, Henneman EA. Emergency Department Medication Lists Are Not Accurate. The Journal of Emergency Medicine. 2011;40(6):613-6. doi: https://doi.org/10.1016/j.jemermed.2008.02.060.

6852. Cagliano AC, Grimaldi S, Rafele C. A systemic methodology for risk management in healthcare sector.

Safety Science. 2011;49(5):695-708. doi: https://doi.org/10.1016/j.ssci.2011.01.006.

6853. Cahen VC, Li Y, Elgarten CW, DiNofia AM, Wilkes JJ, Winestone LE, et al. Using Administrative Data to Identify Relapse and Hematopoietic Stem Cell Transplantation (HSCT) in Children with Acute Lymphoblastic Leukemia (ALL): Validation at Two Centers and Incidence Estimation in a National Cohort. Blood. 2018;132:624. doi: https://doi.org/10.1182/blood-2018-99-114228.

6854. Cahill JA. Responsibilities of physicians and pharmacists in preventing drug interactions. JAMA. 2002;287(5):586-7. PubMed PMID: 11829691.

6855. Cahir C, Bennett K, Teljeur C, Fahey T. Potentially inappropriate prescribing and adverse health outcomes in community dwelling older patients. British journal of clinical pharmacology. 2014;77(1):201-10.

6856. Cahir C, Fahey T, Tilson L, Teljeur C, Bennett K. Proton pump inhibitors: potential cost reductions by applying prescribing guidelines. Bmc Health Services Research. 2012;12. doi: 10.1186/1472-6963-12-408. PubMed PMID: WOS:000312771000002.

6857. Cahyaningsih I, Lambert M, Ochi T, Li F, Li X, Denig P, et al. Community pharmacist-led interventions for patients with type 2 diabetes in low-income and middle-income countries: A scoping review. Research in social & administrative pharmacy : RSAP. 2023;19(8):1117-30. doi: https://dx.doi.org/10.1016/j.sapharm.2023.04.124. PubMed PMID: 641488567.

6858. Cahyaningsih I, Lambert M, Ochi T, Li F, Li X, Denig P, et al. Community pharmacist-led interventions in patients with type 2 diabetes in low-income and middle-income countries: A scoping review. Research in Social and Administrative Pharmacy. 2023.

6859. Cahyaningsih I, Lambert M, Ochi T, Li F, Li XY, Denig P, et al. Community pharmacist-led interventions for patients with type 2 diabetes in low-income and middle-income countries: A scoping review. Research in Social & Administrative Pharmacy. 2023;19(8):1117-30. doi: 10.1016/j.sapharm.2023.04.124. PubMed PMID: WOS:001051300100001.

6860. Cai BL, Said A, Li X, Li F, Arcona S. Healthcare costs and resource utilization in patients with severe aplastic anemia in the US. Journal of Medical Economics. 2019;22(10):1055-62. doi: 10.1080/13696998.2019.1643354. PubMed PMID: WOS:000479081500001.

6861. Cai H. Care-seeking Patterns among Rural Communities in the Republic of Congo [M.P.H.]. United States -- Connecticut: Yale University; 2016.

6862. Cai M, Wang ZL, Gu HT, Dong H, Zhang X, Cui NX, et al. Occurrence and temporal variation of antibiotics and antibiotic resistance genes in hospital inpatient department wastewater: Impacts of daily schedule of inpatients and wastewater treatment process. Chemosphere. 2022;292. doi: 10.1016/j.chemosphere.2021.133405. PubMed PMID: WOS:000758272600003.

6863. Cai M, Zhou L, Gao D, Mei D, Zhang B, Zuo W, et al. A national survey of individualized pharmaceutical care practice in Chinese hospitals in 2019. Frontiers in Pharmacology. 2023;14. doi: 10.3389/fphar.2023.1022134.

6864. Caiaffa WT, Bastos FI, Proietti FA, Reis ÂCM, Mingoti SA, Gandolfi D, et al. Practices surrounding syringe acquisition and disposal: effects of Syringe Exchange Programmes from different Brazilian regions—the AjUDE-Brasil II Project. International Journal of Drug Policy. 2003;14(5):365-71. doi: https://doi.org/10.1016/S0955-3959(03)00136-1.

6865. Caiaffa WT, Bastos FI, Proietti FA, Reis ÂCM, Mingoti SA, Gandolfi D, et al. Practices surrounding

syringe acquisition and disposal: Effects of Syringe Exchange Programmes from different Brazilian regions - The AjUDE-Brasil II Project. International Journal of Drug Policy. 2003;14(5-6):365-71. doi: 10.1016/S0955-3959(03)00136-1.

6866. Caillet C, Sichanh C, Assemat G, Malet-Martino M, Sommet A, Bagheri H, et al. Role of Medicines of Unknown Identity in Adverse Drug Reaction-Related Hospitalizations in Developing Countries: Evidence from a Cross-Sectional Study in a Teaching Hospital in the Lao People's Democratic Republic. Drug Safety. 2017;40(9):809-21. PubMed PMID: 28528487.

6867. Cain J. Online social networking issues within academia and pharmacy education. American journal of pharmaceutical education. 2008;72(1).

6868. Cain J. We should pay more attention to student curiosity. Currents in Pharmacy Teaching and Learning. 2019;11(7):651-4. doi: https://doi.org/10.1016/j.cptl.2019.03.001.

6869. Cain J. Exploratory implementation of a blended format escape room in a large enrollment pharmacy management class. Currents in Pharmacy Teaching and Learning. 2019;11(1):44-50. doi: https://doi.org/10.1016/j.cptl.2018.09.010.

6870. Cain J, Stowe CD, Ali D, Romanelli F. How Faculty Recognized for Teaching Excellence Interpret and Respond to Student Ratings of Teaching. American journal of pharmaceutical education. 2019;83(4):6680. doi: 10.5688/ajpe6680. PubMed PMID: CN-01960208.

6871. Cain WT, Cable G, Oppenheimer JJ. The ability of the community pharmacist to learn the proper actuation techniques of inhaler devices. Journal of Allergy and Clinical Immunology. 2001;108(6):918-20.

6872. Cairns C, editor Equipping pharmacy undergraduates to deal with life on the jubilee line ! Pharmaceutical Journal; 2008.

6873. Cairns C, Eveleigh M. Involving community pharmacists in asthma care: Lessons from a pilot study. Pharmaceutical Journal. 2000;265(7106):136-40.

6874. Cairns C, Eveleigh M. Community pharmacists' contribution to managing patients with asthma. Asthma Journal. 2000;5(2):80-3.

6875. Cairns C, Marlow H, McCoig A, Hargreaves D. The Croydon community pharmacy accident survey. Pharmaceutical Journal. 1996;257(6920):817-9.

6876. Cajigal S, Wells KE, Peterson EL, Ahmedani BK, Yang JJ, Kumar R, et al. Predictive Properties of the Asthma Control Test and Its Component Questions for Severe Asthma Exacerbations. The Journal of Allergy and Clinical Immunology: In Practice. 2017;5(1):121-7.e2. doi: https://doi.org/10.1016/j.jaip.2016.06.025.

6877. Çakioglu B, Arikan MG, Tas T, Bilir B. The role of primary nocturnal enuresis in the aetiology of overactive bladder syndrome. Central European Journal of Urology. 2023;76(3):207-11. doi: 10.5173/ceju.2023.56. PubMed PMID: WOS:001152127000004.

6878. Calabrese CM, Kirchner E, Husni EM, Moss BP, Fernandez AP, Jin YX, et al. Breakthrough SARS-CoV-2 Infections in Patients With Immune-Mediated Disease Undergoing B Cell-Depleting Therapy: A Retrospective Cohort Analysis. Arthritis & Rheumatology. doi: 10.1002/art.42287. PubMed PMID: WOS:000876319000001.

6879. Calabrese D, Minkoff NB, Rawlings K. Pharmacosynchrony: Road map to transformation in pharmacy benefit management. American Journal of Pharmacy Benefits. 2014;6(2):76-80.

6880. Calabretto H. Emergency contraception - Knowledge and attitudes in a group of Australian university students. Australian and New Zealand Journal of Public Health. 2009;33(3):234-9. doi: 10.1111/j.1753-6405.2009.00381.x.

6881. Calabretto JP, Warren J. Initial assessment of an Internet-based medication record. Journal of Pharmacy Practice and Research. 2005;35(2):99-102. doi: 10.1002/j.2055-2335.2005.tb00315.x.

6882. Calabró P, Elizondo C, Mozeluk N, Permanozaya G, Terrasaardini SA, Blugerman G, et al. Frequency of medication consumption in patients over 65 years of age in an HMO from Buenos Aires, Argentina...21st International Conference on Integrated Care (Virtual), May 1-31, 2021. International Journal of Integrated Care (IJIC). 2022;22:1-2. doi: 10.5334/ijic.ICIC21149. PubMed PMID: 161074054. Language: English. Entry Date: 20230116. Revision Date: 20230116. Publication Type: Article.

6883. Calado SB. Avaliação do Controlo da Asma e Rinite Alérgica em Contexto de Farmácia Comunitária: Aplicação do Carat 10: Experiência Profissionalizante na Vertente de Farmácia Comunitária e Investigação [Master's]. Portugal: Universidade da Beira Interior (Portugal); 2012.

6884. Calamusa A, Di Marzio A, Cristofani R, Arrighetti P, Santaniello V, Alfani S, et al. Factors that influence Italian consumers' understanding of over-the-counter medicines and risk perception. Patient Education and Counseling. 2012;87(3):395-401. doi: https://doi.org/10.1016/j.pec.2011.10.003.

6885. Calarge CA, Murry DJ, Ziegler EE, Arnold LE. Serum Ferritin, Weight Gain, Disruptive Behavior, and Extrapyramidal Symptoms in Risperidone-Treated Youth. Journal of child and adolescent psychopharmacology. 2016;26(5):471-7. doi: 10.1089/cap.2015.0194. PubMed PMID: CN-01445032.

6886. Calarge CA, Nicol G, Schlechte JA, Burns TL. Cardiometabolic outcomes in children and adolescents following discontinuation of long-term risperidone treatment. Journal of child and adolescent psychopharmacology. 2014;24(3):120-9. doi: 10.1089/cap.2013.0126. PubMed PMID: CN-01037926.

6887. Ćalasan J, Mijatović V, Horvat O, Varga J, Sabo A, Stilinović N. The outpatient utilization of non-steroidal anti-inflammatory drugs in South Bac?ka District, Serbia. International Journal of Clinical Pharmacy. 2011;33(2):246-51. doi: 10.1007/s11096-011-9487-0.

6888. Calbo E, Álvarez-Rocha L, Gudiol F, Pasquau J. A review of the factors influencing antimicrobial prescribing. Enfermedades infecciosas y microbiologia clinica. 2013;31:12-5.

6889. Caldas L, Goode J, Fountain K, Harrison A, Smith J. Comparison of delivery models of medication therapy management services in a supermarket chain pharmacy. Journal of the american pharmacists association. 2012;52(2):239. doi: 10.1331/JAPhA.2012.12510. PubMed PMID: CN-01063299.

6890. Caldas LM, Matulewicz AT, Koenig RA, Wei X, Hindle M, Donohoe KL. Team teaching with pharmacy practice and pharmaceutics faculty in a nonsterile compounding laboratory course to increase student problem-solving skills. Currents in Pharmacy Teaching and Learning. 2020;12(3):320-5. doi: https://doi.org/10.1016/j.cptl.2019.12.017.

6891. Calderbank S, Uncles DR, Burns N, Kariyawasam HKCD, Allan GDL. Sequential drug verification errors resulting in wrong drug administration during caesarean section. International Journal of Obstetric Anesthesia. 2011;20(1):73-6. doi: https://doi.org/10.1016/j.ijoa.2010.07.007.

6892. Calderon Y, Cowan E, Rhee JY, Brusalis C, Leider J. Counselor-based rapid HIV testing in community pharmacies. AIDS Patient Care and STDs. 2013;27(8):467-73. doi: 10.1089/apc.2013.0076.

6893. Calderon Y, Cowan E, Schramm C, Stern S, Brusalis C, Iscoe M, et al. HCV and HBV testing

acceptability and knowledge among urban emergency department patients and pharmacy clients. Preventive Medicine. 2014;61:29-33. doi: https://doi.org/10.1016/j.ypmed.2013.12.026.

6894. Calderon-Anyosa R, Raza Garcia M, Carcamo Cavagnaro C, Garcia P. Effectiveness of Kuska Tele-Wasi, a home blood-pressure telemonitoring system for hypertension control in Lima, Peru: a randomised controlled trial. The lancet global health. 2019;7:S9-. doi: 10.1016/S2214-109X(19)30094-4. PubMed PMID: CN-01915881.

6895. Caldwell CC. Exploration and Expansion of Novel Therapeutic Strategies Targeting Brain Metabolism in Neurodegenerative Diseases [Ph.D.]. United States -- California: University of Southern California; 2018.

6896. Caldwell PH, Murphy SB, Butow PN, Craig JC. Clinical trials in children. The Lancet. 2004;364(9436):803-11.

6897. Caldwell SM, Raitt JR. Oral medication administration: Implications caused by capsule splitting. Journal of the American Pharmacists Association. 2010;50(4):532-3. doi: https://doi.org/10.1331/JAPhA.2010.08079.

6898. Calero MJM, Martin M, Isorna FM, Murillo RL, Amado AL, Lama IV. Patient characterization as a useful tool for offering personalized professional services in the community pharmacy. Farmaceuticos Comunitarios. 2015;7(4):5-17. doi: 10.5672/FC.2173-9218.(2015/Vol7).004.02. PubMed PMID: WOS:000417268800002.

6899. Calfee JE. Public policy issues in direct-to-consumer advertising of prescription drugs. Journal of Public Policy & Marketing. 2002;21(2):174-93.

6900. Calgan Z, Aslan D, Yegenoglu S. Community pharmacists’ burnout levels and related factors: an example from Turkey. International journal of clinical pharmacy. 2011;33:92-100.

6901. Çalgan Z, Tahir E, Yeğenoğlu S, Bilir N. Community pharmacists' attitude, habits and actual activities regarding smoking and health promotion in the 1. Region of Ankara chamber of pharmacists. Turkish Journal of Pharmaceutical Sciences. 2007;4(3):125-38.

6902. Caligiuri SPB, Austria JA, Pierce GN. Alarming Prevalence of Emergency Hypertension Levels in the General Public Identified by a Hypertension Awareness Campaign. American Journal of Hypertension. 2017;30(3):236-9. doi: 10.1093/ajh/hpw136. PubMed PMID: WOS:000397027400008.

6903. Çalikuşu M, Güneş G, Özçelikay G. The effects of covid-19 pandemic on community pharmacy services. Ankara Universitesi Eczacilik Fakultesi Dergisi. 2021;45(2):194-211. doi: 10.33483/jfpau.831105.

6904. Çalikuşu M, Güneş G, ÖzçeliKay G. THE EFFECTS OF COVID-19 PANDEMIC ON COMMUNITY PHARMACY SERVICES. Ankara Universitesi Eczacilik Fakultesi Dergisi. 2022;46(2):194-211. doi: 10.33483/jfpau.831105q.

6905. Calip GS, Lee W-J, Lee TA, Schumock GT, Chiu BCH. Tumor Necrosis Factor-Alpha Inhibitor Medications for Inflammatory Conditions and Incidence of Multiple Myeloma. Blood. 2015;126(23):2954. doi: https://doi.org/10.1182/blood.V126.23.2954.2954.

6906. Calip GS, Zhou J, Sweiss K, Patel P, Asfaw AA, Lee T, et al. Venous Thromboembolic Prophylaxis Following Treatment Initiation for Multiple Myeloma. Blood. 2018;132:4693. doi: https://doi.org/10.1182/blood-2018-99-116867.

6907. Caliph S, Lim AS, Karunaratne N. A comparison of the academic performance of graduate entry and

undergraduate entry pharmacy students at the course exit level. Currents in Pharmacy Teaching and Learning. 2022;14(7):881-6. doi: https://doi.org/10.1016/j.cptl.2022.06.019.

6908. Caliph SM, Trevaskis NL, Charman WN, Porter CJH. Intravenous Dosing Conditions May Affect Systemic Clearance for Highly Lipophilic Drugs: Implications for Lymphatic Transport and Absolute Bioavailability Studies. Journal of Pharmaceutical Sciences. 2012;101(9):3540-6. doi: https://doi.org/10.1002/jps.23211.

6909. Calis KA, Hutchison LC, Elliott ME, Ives TJ, Zillich AJ, Poirier T, et al. Healthy People 2010: Challenges, opportunities, and a call to action for America's pharmacists. Pharmacotherapy. 2004;24(9):1241-94. PubMed PMID: WOS:000223597200019.

6910. Callahan LF, Hawk V, Rudd R, Hackney B, Bhandari S, Prizer LP, et al. Adaptation of the health literacy universal precautions toolkit for rheumatology and cardiology – Applications for pharmacy professionals to improve self-management and outcomes in patients with chronic disease. Research in Social and Administrative Pharmacy. 2013;9(5):597-608. doi: https://doi.org/10.1016/j.sapharm.2013.04.016.

6911. Callahan LF, Hawk V, Rudd R, Hackney B, Bhandari S, Prizer LP, et al. Adaptation of the health literacy universal precautions toolkit for rheumatology and cardiology - Applications for pharmacy professionals to improve self-management and outcomes in patients with chronic disease. Research in Social and Administrative Pharmacy. 2013;9(5):597-608. doi: 10.1016/j.sapharm.2013.04.016.

6912. Callahan RJ. The role of commerical nuclear pharmacy in the future practice of nuclear medicine. Seminars in Nuclear Medicine. 1996;26(2):85-90. doi: https://doi.org/10.1016/S0001-2998(96)80029-8.

6913. Callanquin J, Labrude P, Rouyer A. Thoughts and proposals for quality assurance in orthopedics in the community pharmacy. Annales Pharmaceutiques Francaises. 2007;65(3):183-8. doi: 10.1016/S0003-4509(07)90034-6.

6914. Calleja L, Glass BD, Cairns A, Taylor S. Pharmacist-Led Interventions for Medication Adherence in Patients with Chronic Kidney Disease: A Scoping Review. Pharmacy. 2023;11(6):185. doi: https://dx.doi.org/10.3390/pharmacy11060185. PubMed PMID: 2027308505.

6915. Callender SP, Mathews JA, Kobernyk K, Wettig SD. Microemulsion utility in pharmaceuticals: Implications for multi-drug delivery. International journal of pharmaceutics. 2017;526(1-2):425-42.

6916. Calles DF, Hervas EG, Alambiaga-Caravaca AM, Ruiz MA, Ferrandiz EC, Ripoll JB, et al. Encourage reading in older people to protect them from community pharmacie from cognitive dysfunction. Farmaceuticos Comunitarios. 2019;11(1):14-8. doi: 10.33620/FC.2173-9218.(2019/Vol11).001.03. PubMed PMID: WOS:000462800600003.

6917. Calloway S, Akilo H, Bierman K. Impact of a clinical decision support system on pharmacy clinical interventions, documentation efforts, and costs. Hospital Pharmacy. 2013;48(9):744-52. doi: 10.1310/hpj4809-744.

6918. Callréus T. Use of desmopressin in elderly patients in Denmark. Pharmacoepidemiology and Drug Safety. 2007;16(9):995-8. doi: 10.1002/pds.1394.

6919. Calogero S, Caley CF. Supporting patients with mental illness: Deconstructing barriers to community pharmacist access. Journal of the American Pharmacists Association. 2017;57(2):248-55.

6920. Calomo JM. Teaching management in a community pharmacy. American Journal of Pharmaceutical Education. 2006;70(2).

6921. Calonge N. Policy Pitfalls and Challenges in Cannabis Regulation: Lessons from Colorado. International Journal of Mental Health and Addiction. 2018;16(4):783-90. doi: 10.1007/s11469-018-9923-7. PubMed PMID: WOS:000441010800002.

6922. Calop J, Bedouch P, Allenet B. [From treatment history to patient follow-up: the role of the pharmacist]. Bulletin de l Academie Nationale de Medecine. 2013;197(8):1531-43; discussion 43-4. PubMed PMID: 26021176.

6923. Calop N, Allenet B, Calop J, Figari G. The effect of continuous education on the professional practice of French community pharmacists. Pharmacy Education. 2002;2(4):185-90. doi: 10.1080/1560221000050159.

6924. Calva J. Antibiotic use in a periurban community in Mexico: A household and drugstore survey. Social Science & Medicine. 1996;42(8):1121-8. doi: https://doi.org/10.1016/0277-9536(95)00385-1.

6925. Calva J, editor Antibiotic use in a periurban community in Mexico: A household and drugstore survey. Social Science and Medicine; 1996.

6926. Calvert SB, Kramer JM, Anstrom KJ, Kaltenbach LA, Stafford JA, Allen LaPointe NM. Patient-focused intervention to improve long-term adherence to evidence-based medications: a randomized trial. American heart journal. 2012;163(4):657-65.e1. doi: 10.1016/j.ahj.2012.01.019. PubMed PMID: CN-00880528.

6927. Calvert SB, Kramer JM, Anstrom KJ, Kaltenbach LA, Stafford JA, LaPointe NMA. Patient-focused intervention to improve long-term adherence to evidence-based medications: A randomized trial. American Heart Journal. 2012;163(4):657-+. doi: 10.1016/j.ahj.2012.01.019. PubMed PMID: WOS:000303106800027.

6928. Calvo E, Formiga F, Andreu-Periz L, Ariza-Sole A, Comin-Colet J. Components of geriatric assessment and therapeutic adherence in elderly patients with acute myocardial infarction. Revista espanola de geriatria y gerontologia. 2021. doi: 10.1016/j.regg.2021.06.002. PubMed PMID: CN-02305108.

6929. Calvo E, Izquierdo S, Castillo R, César E, Domene G, Gómez AB, et al. Can an individualized adherence education program delivered by nurses improve therapeutic adherence in elderly people with acute myocardial infarction?: a randomized controlled study. International journal of nursing studies. 2021;120:103975. doi: 10.1016/j.ijnurstu.2021.103975. PubMed PMID: CN-02285687.

6930. Calvo E, Izquierdo S, Castillo R, César E, Domene G, Gómez AB, et al. Can an individualized adherence education program delivered by nurses improve therapeutic adherence in elderly people with acute myocardial infarction?: A randomized controlled study. International Journal of Nursing Studies. 2021;120:103975. doi: https://doi.org/10.1016/j.ijnurstu.2021.103975.

6931. Calvo Hernáez B, Gastelurrutia Garralda MÁ, Urionagüena de la Iglesia A, Isla Ruiz A, del Pozo Rodríguez A, Solinís Aspiazu MÁ. Oferta de servicios de atención farmacéutica: clave para un nuevo modelo de servicios de salud. Atención Primaria. 2022;54(1):102198. doi: https://doi.org/10.1016/j.aprim.2021.102198.

6932. Calvo Hernáez B, Gastelurrutia Garralda MÁ, Urionagüena de la Iglesia A, Isla Ruiz A, del Pozo Rodríguez A, Solinís Aspiazu MÁ. Supply of pharmaceutical care services: The key to a new model of health services. Atencion Primaria. 2022;54(1). doi: 10.1016/j.aprim.2021.102198.

6933. Calvo-Cidoncha E, Camacho-Hernando C, Feu F, Pastor-Duran X, Codina-Jané C, Lozano-Rubí R. OntoPharma: ontology based clinical decision support system to reduce medication prescribing errors. BMC Medical Informatics & Decision Making. 2022;22(1):1-12. doi: 10.1186/s12911-022-01979-3. PubMed

PMID: 158998260. Language: English. Entry Date: In Process. Revision Date: 20221003. Publication Type: journal article. Journal Subset: Biomedical.

6934. Cam H, Kempen T. Intervention fidelity within a randomised controlled trial on comprehensive medication reviews in hospitalised patients. International journal of clinical pharmacy. 2018;40(1):251-. doi: 10.1007/s11096-017-0565-9. PubMed PMID: CN-01468259.

6935. Cama E, Beadman M, Beadman K, Hopwood M, Treloar C. Health workers' perspectives of hepatitis B-related stigma among Aboriginal and Torres Strait Islander people in New South Wales, Australia. Harm Reduction Journal. 2023;20(1). doi: 10.1186/s12954-023-00844-4. PubMed PMID: WOS:001054748400002.

6936. Cama LP, Brunsoms MTF, Durán SC, Roig AM. Coordinated project for the management of arterial hypertension in the community. Pharmaceutical Care Espana. 2004;6(1):9-15.

6937. Câmara EBF. A Relação dos Técnicos de Farmácia com o Sistema Pyxis na Gestão Hospitalar do Medicamento [Master's]. Portugal: Instituto Politecnico do Porto (Portugal); 2018.

6938. Cámara R, Cosín A, Dago A, Gutiérrez P, Salar L, Solá N. D-VALUE program, analyzing dispensing records. Benzodiazepines. Pharmaceutical Care Espana. 2013;15(4):140-6.

6939. Camargo Rubio RD. Bioética prescripción antibiótica y resistencia bacteriana. Acta Colombiana de Cuidado Intensivo. 2023;23(4):363-9. doi: https://doi.org/10.1016/j.acci.2023.04.010.

6940. Cambridge L. Health Care Reform and Asthma Care Disparities in Minority Populations. Journal of Asthma and Allergy Educators. 2011;2(1):7-10. doi: 10.1177/2150129710380043.

6941. Cambruzzi M, Knowles T, Macfarlane P. Accuracy of drawing up liquid medications by veterinary anaesthetists and nurses. Veterinary Anaesthesia & Analgesia. 2023;50(6):502-6. doi: 10.1016/j.vaa.2023.09.002. PubMed PMID: 173456378.

6942. Camden M, Smith R, Turturici M, Arnold L, Kelley-Baker T. 2325 - Countermeasures to Prescription and over-the-Counter Drug-Impaired Driving: Pharmacy & Medical and Education & Advertising. Journal of Transport & Health. 2018;9:S43. doi: https://doi.org/10.1016/j.jth.2018.05.026.

6943. Camean Fernandez M, Guerrero Aznar MD, Cobos Garcia FJ, Santos Ramos B, Bautista Paloma J, Del Prado Llergo JR, et al. Use of antimicrobials in the hospitals of the Andalusian Health Service in 1994. Farmacia Hospitalaria. 1996;20(6):365-73.

6944. Cameli D, Francis M, Francois VE, Medder NR, Von Eden L, Truglio-Londrigan M. The effectiveness of medication reconciliation strategies to reduce medication errors in community dwelling older adults: a systematic review. JBI Database of Systematic Reviews & Implementation Reports. 2013;11(7):1-57. doi: 10.11124/jbisrir-2013-463. PubMed PMID: 113609674. Language: English. Entry Date: 20180117. Revision Date: 20180118. Publication Type: Article.

6945. Cameron A, McPhail SM, Hudson K, Fleming J, Lethlean J, Finch E. A pre–post intervention study investigating the confidence and knowledge of health professionals communicating with people with aphasia in a metropolitan hospital. Aphasiology. 2017;31(3):359-74. doi: 10.1080/02687038.2016.1225277.

6946. Cameron A, McPhail SM, Hudson K, Fleming J, Lethlean J, Finch E. A pre-post intervention study investigating the confidence and knowledge of health professionals communicating with people with aphasia in a metropolitan hospital. Aphasiology. 2017;31(3):359-74. doi: 10.1080/02687038.2016.1225277. PubMed PMID: WOS:000392850200005.

6947. Cameron CR, Ewachiw B, Chasler JE, Dalpoas SE, Vázquez J, Burdalski C. Assisted versus referred pharmacy smoking interventions for patients with thoracic malignancies or pulmonary nodules. Drug and Alcohol Dependence. 2022;236:109465. doi: https://doi.org/10.1016/j.drugalcdep.2022.109465.

6948. Cameron G, Chandra RN, Ivey MF, Khatri CS, Nemire RE, Quinn CJ, et al. ASHP statement on the pharmacist's role in public health. American Journal of Health-System Pharmacy. 2022;79(5):388-99.

6949. Cameron IM, Matheson CI, Bond CM, McNamee P, Lawrie T, Robinson A, et al. Pilot randomised controlled trial of community pharmacy administration of buprenorphine versus methadone. International journal of pharmacy practice. 2006;14(4):243-8. doi: 10.1211/ijpp.14.4.0003. PubMed PMID: CN-00612682.

6950. Cameron J, Gilchrist G, Roberts K. Needle exchange services: A profile of service users in community pharmacies and other settings. International Journal of Pharmacy Practice. 2004;12(4):211-5. doi: 10.1211/0022357044706.

6951. Cameron KA. Comment on medication management models and other pharmacist interventions: Implications for policy and practice. Home Health Care Services Quarterly. 2005;24(1-2):73-85. doi: 10.1300/J027v24n01_06.

6952. Cameron KA. Healthy aging: Programs that make a difference-Part 1 - First of a two-part series. Consultant Pharmacist. 2012;27(4):239-53. doi: 10.4140/TCP.n.2012.239.

6953. Cameron KA, Pinkowitz J. Connecting with assisted living consumers. Consultant Pharmacist. 2009;24(1):16-8, 21-8. PubMed PMID: 19275457.

6954. Cameron LK, Lumlertgul N, Bear DE, Cooney E, McKenzie C, Ostermann M. Micronutrient use in critical care: Survey of clinical practice. Clinical Nutrition ESPEN. 2023;55:260-6. doi: https://doi.org/10.1016/j.clnesp.2023.03.023.

6955. Cameron S. The effectiveness of bridging from emergency to regular contraception. https://wwwisrctncom/ISRCTN70616901. 2017. doi: 10.1186/ISRCTN70616901. PubMed PMID: CN-02255031.

6956. Cameron ST, Baraitser P, Glasier A, McDaid L, Norrie J, Radley A, et al. Pragmatic cluster randomised cohort cross-over trial to determine the effectiveness of bridging from emergency to regular contraception: the Bridge-It study protocol. BMJ open. 2019;9(10):e029978. doi: 10.1136/bmjopen-2019-029978. PubMed PMID: CN-02004269.

6957. Cameron ST, Glasier A, McDaid L, Radley A, Baraitser P, Stephenson J, et al. Use of effective contraception following provision of the progestogen-only pill for women presenting to community pharmacies for emergency contraception (Bridge-It): a pragmatic cluster-randomised crossover trial. Lancet (london, england). 2020;396(10262):1585-94. doi: 10.1016/S0140-6736(20)31785-2. PubMed PMID: CN-02199996.

6958. Cameron ST, Glasier A, McDaid L, Radley A, Baraitser P, Stephenson J, et al. Use of effective contraception following provision of the progestogen-only pill for women presenting to community pharmacies for emergency contraception (Bridge-It): a pragmatic cluster-randomised crossover trial. The Lancet. 2020;396(10262):1585-94. doi: https://doi.org/10.1016/S0140-6736(20)31785-2.

6959. Cameron ST, Glasier A, McDaid L, Radley A, Patterson S, Baraitser P, et al. Provision of the progestogen-only pill by community pharmacies as bridging contraception for women receiving emergency contraception: the Bridge-it RCT. Health technology assessment (Winchester, England). 2021;25(27):1-92. doi: 10.3310/hta25270. PubMed PMID: CN-02272128.

6960. Cameron ST, Glasier A, Muir A, Scott G, Johnstone A, Quarrell H, et al. Expedited partner therapy for Chlamydia trachomatis at the community pharmacy. Bjog-an International Journal of Obstetrics and Gynaecology. 2010;117(9):1074-9. doi: 10.1111/j.1471-0528.2010.02573.x. PubMed PMID: WOS:000279532500006.

6961. Cameron ST, Glasier A, Muir A, Scott G, Johnstone A, Quarrell H, et al. Expedited Partner Therapy for Chlamydia trachomatis at the Community Pharmacy. Obstetrical & Gynecological Survey. 2011;66(4):209-10. doi: 10.1097/OGX.0b013e318225cd17. PubMed PMID: WOS:000292735400013.

6962. Cameron ST, Melvin L, Glasier A, Scott G, Johnstone A, Young H. Willingness of gynaecologists, doctors in family planning, GPs, practice nurses and pharmacists to adopt novel interventions for treating sexual partners of women with chlamydia. BJOG: An International Journal of Obstetrics and Gynaecology. 2007;114(12):1516-21. doi: 10.1111/j.1471-0528.2007.01506.x.

6963. Camilleri M, Staiano A. Insights on Obesity in Children and Adults: Individualizing Management. Trends in Endocrinology and Metabolism. 2019;30(10):724-34. doi: 10.1016/j.tem.2019.07.016. PubMed PMID: WOS:000489015300007.

6964. Caminati M, Cegolon L, Bacchini M, Segala N, Dama A, Bovo C, et al. The potential role of local pharmacies to assess asthma control: an Italian cross-sectional study. BMC Public Health. 2021;21(1). doi: 10.1186/s12889-020-10080-1.

6965. Caminati M, Senna G, Segala N, Bacchini M, Stefanizzi G, Bovo C, et al. Evaluation of asthma control in the pharmacy: An italian cross-sectional study. European Annals of Allergy and Clinical Immunology. 2017;49(5):225-30. doi: 10.23822/EurAnnACI.1764-1489.03.

6966. Camiré E, Moyen E, Stelfox HT. Medication errors in critical care: risk factors, prevention and disclosure. Cmaj. 2009;180(9):936-43.

6967. Cammy R, Worster B, Csik V, Garber G. Promoting Value of Non-Billable Interventions in Supportive Oncology Care. Journal of Pain and Symptom Management. 2022;63(5):851-2. doi: https://doi.org/10.1016/j.jpainsymman.2022.02.030.

6968. Campagna EJ, Muser E, Parks J, Morrato EH. Methodological Considerations in Estimating Adherence and Persistence for a Long-Acting Injectable Medication. Journal of Managed Care & Specialty Pharmacy. 2014;20(7):756-+. doi: 10.18553/jmcp.2014.20.7.756. PubMed PMID: WOS:000341403500011.

6969. Campani V, Pagnozzi E, Mataro I, Mayol L, Perna A, D'Urso F, et al. Chitosan Gel to Treat Pressure Ulcers: A Clinical Pilot Study. Pharmaceutics. 2018;10(1). doi: 10.3390/pharmaceutics10010015. PubMed PMID: WOS:000428561900014.

6970. Campbell A, Cong Z, Agodoa I, Song X, Martinez DJ, Black D, et al. The Economic Burden of End-Organ Damage Among Medicaid Patients with Sickle Cell Disease in the United States: A Population-Based Longitudinal Claims Study. Journal of Managed Care & Specialty Pharmacy. 2020;26(9):1121-+. doi: 10.18553/jmcp.2020.20009. PubMed PMID: WOS:000564151000009.

6971. Campbell C, Braund R, Morris C. Beyond the four walls: An exploratory survey of location, employment and roles of pharmacists in primary health care. Journal of Primary Health Care. 2017;9(4):297-310. doi: 10.1071/HC17022.

6972. Campbell C, Morris C, Braund R. Medicines information in New Zealand: Current services and future potential. New Zealand Medical Journal. 2016;129(1447):21-8.

6973. Campbell C, Morris C, McBain L. Electronic transmission of prescriptions in primary care: Transformation, timing and teamwork. Journal of Primary Health Care. 2021;13(4):340-50. doi: 10.1071/HC21050.

6974. Campbell CA, Hahn RA, Elder R, Brewer R, Chattopadhyay S, Fielding P, et al. The Effectiveness of Limiting Alcohol Outlet Density As a Means of Reducing Excessive Alcohol Consumption and Alcohol-Related Harms. American Journal of Preventive Medicine. 2009;37(6):556-69. doi: 10.1016/j.amepre.2009.09.028. PubMed PMID: WOS:000272769700015.

6975. Campbell DB, Sutcliffe JS, Ebert PJ, Militerni R, Bravaccio C, Trillo S, et al. A genetic variant that disrupts MET transcription is associated with autism. Proceedings of the National Academy of Sciences. 2006;103(45):16834-9.

6976. Campbell DJT, Saunders-Smith T, Manns BJ, Tonelli M, Ivers N, Hemmelgarn BR, et al. Exploring patient and pharmacist perspectives on complex interventions for cardiovascular prevention: a qualitative descriptive process evaluation. Health expectations. 2020;23(6):1485-501. doi: 10.1111/hex.13133. PubMed PMID: CN-02207454.

6977. Campbell E, Houston K, Lang E, Hohl CM, Romonko-Slack L, Silvius J, et al. A multicenter comparison of three medication screening tools to identify patients at high risk of adverse drug events. Canadian journal of emergency medicine Conference: 2015 CAEP/ACMU Canada. 2015;17:S55-S6. doi: 10.1017/cem.2015.50. PubMed PMID: CN-01375698.

6978. Campbell EG, Regan S, Gruen RL, Ferris TG, Rao SR, Cleary PD, et al. Professionalism in medicine: results of a national survey of physicians. Annals of Internal Medicine. 2007;147(11):795-802.

6979. Campbell EM, Sittig DF, Ash JS, Guappone KP, Dykstra RH. Types of unintended consequences related to computerized provider order entry. Journal of the American Medical Informatics Association. 2006;13(5):547-56.

6980. Campbell F, Karnon J, Czoski-Murray C, Jones R. A systematic review of the effectiveness and cost-effectiveness of interventions aimed at preventing medication error (medicines reconciliation) at hospital admission. Report for the National Institute for Health and Clinical Excellence as part of the Patient Safety Pilot. 2007.

6981. Campbell G, Hall WD, Peacock A, Lintzeris N, Bruno R, Larance B, et al. Effect of cannabis use in people with chronic non-cancer pain prescribed opioids: findings from a 4-year prospective cohort study. The Lancet Public Health. 2018;3(7):e341-e50. doi: 10.1016/S2468-2667(18)30110-5.

6982. Campbell G, Razouk R, Auyeung V, Webb J, Ismail TF. Evaluation of the impact of the addition of a heart failure prescribing pharmacist to consultant-led heart failure ward round at a tertiary hospital. European Journal of Hospital Pharmacy Science & Practice. 2023;30(e1):e14-e8. PubMed PMID: 34315773.

6983. Campbell GM, Facchinetti NJ. Using process control charts to monitor dispensing and checking errors. American Journal of Health-System Pharmacy. 1998;55(9):946-52. PubMed PMID: 9588253.

6984. Campbell H, Murata A, Conner T. Va rapidly reduces adverse clinical events following randomized clinical trial showing harm. Health services research. 2020;55(SUPPL 1):83-4. doi: 10.1111/1475-6773.13446. PubMed PMID: CN-02242944.

6985. Campbell H, Paterson P, Letley L, Saliba V, Mounier-Jack S, Yarwood J. Vaccination, information and parental confidence in the digital age in England. Vaccine: X. 2023;14:100345. doi: https://doi.org/10.1016/j.jvacx.2023.100345.

6986. Campbell HE. Clinical monograph for drug formulary review: erectile dysfunction agents. Journal of managed care pharmacy. 2005;11(2):151-71. doi: 10.18553/jmcp.2005.11.2.151. PubMed PMID: CN-01770214.

6987. Campbell HM, Khan N, Cone C, Raisch DW. Relationship between diet, exercise habits, and health status among patients with diabetes. Research in Social and Administrative Pharmacy. 2011;7(2):151-61. doi: https://doi.org/10.1016/j.sapharm.2010.03.002.

6988. Campbell JD, Allen-Ramey F, Sajjan SG, Maiese EM, Sullivan SD. Increasing Pharmaceutical Copayments: Impact on Asthma Medication Utilization and Outcomes. American Journal of Managed Care. 2011;17(10):703-10. PubMed PMID: WOS:000296283700010.

6989. Campbell M. Community-Based Therapeutic Drug Monitoring: Useful Development or Unnecessary Distraction? Clinical Pharmacokinetics. 1995;28(4):271-4. doi: 10.2165/00003088-199528040-00001.

6990. Campbell MJ, Wells E, Tietz D, Balmat R, Wesolek J, Mace SE, et al. Direct patient care activities and interventions of emergency medicine pharmacists. International Journal of Clinical Pharmacy. 2019;41(3):667-71. doi: 10.1007/s11096-019-00817-3. PubMed PMID: 136840335. Language: English. Entry Date: 20190810. Revision Date: 20200601. Publication Type: journal article. Journal Subset: Biomedical.

6991. Campbell N, Clark JP, Stecher VJ, Goldstein I. Internet-Ordered Viagra (Sildenafil Citrate) Is Rarely Genuine. The Journal of Sexual Medicine. 2012;9(11):2943-51. doi: https://doi.org/10.1111/j.1743-6109.2012.02877.x.

6992. Campbell NL, Holden R, Gao S, Unverzagt F, Boustani M. R2D2: a cluster-randomized trial of deprescribing to support brain health. Epidemiology (Cambridge, Mass). 2022;70(SUPPL 1):S193-. doi: 10.1111/jgs.17755. PubMed PMID: CN-02401882.

6993. Campbell NL, Khan BA, Farber M, Campbell T, Perkins AJ, Hui SL, et al. Improving delirium care in the intensive care unit: the design of a pragmatic study. Trials. 2011;12:139. doi: 10.1186/1745-6215-12-139. PubMed PMID: CN-00799515.

6994. Campbell NL, Lane KA, Gao S, Boustani MA, Unverzagt F. Anticholinergics Influence Transition from Normal Cognition to Mild Cognitive Impairment in Older Adults in Primary Care. Pharmacotherapy. 2018;38(5):511-9. doi: 10.1002/phar.2106.

6995. Campbell NL, Perkins AJ, Bradt P, Perk S, Wielage RC, Boustani MA, et al. Association of Anticholinergic Burden with Cognitive Impairment and Health Care Utilization Among a Diverse Ambulatory Older Adult Population. Pharmacotherapy. 2016;36(11):1123-31. doi: 10.1002/phar.1843.

6996. Campbell NL, Perkins AJ, Khan BA, Gao S, Farber MO, Khan S, et al. Deprescribing in the Pharmacologic Management of Delirium: a Randomized Trial in the Intensive Care Unit. Journal of the American Geriatrics Society. 2019;67(4):695-702. doi: 10.1111/jgs.15751. PubMed PMID: CN-01937252.

6997. Campbell P, Baker WL, Bendel SD, White WB. Intravenous hydralazine for blood pressure management in the hospitalized patient: its use is often unjustified. Journal of the American Society of Hypertension. 2011;5(6):473-7. doi: https://doi.org/10.1016/j.jash.2011.07.002.

6998. Campbell P, Chinthammit C, Warner M, Boesen K, Warholak T, Barton K. USE PATTERNS OF AN ELECTRONIC MEDICATION THERAPY MANAGEMENT (MTM) PLATFORM IN A NATIONWIDE MASS MERCHANT RETAIL PHARMACY SETTING. Research in Social & Administrative Pharmacy. 2018;14(5):E10-E1. doi: 10.1016/j.sapharm.2018.03.043. PubMed PMID: WOS:000432167600037.

6999. Campbell PJ. Adherence: An Important Metric to Assess Pharmacist Value in Quality Care. Journal of Managed Care & Specialty Pharmacy. 2019;25(10):1044-5. PubMed PMID: 31556826.

7000. Campbell PJ, Axon DR, Taylor AM, Pickering M, Black H, Warholak T, et al. Associations of Renin-Angiotensin System Antagonist Medication Adherence and Economic Outcomes Among Commercially Insured US Adults: A Retrospective Cohort Study. Journal of the American Heart Association. 2020;9(17). doi: 10.1161/jaha.119.016094. PubMed PMID: WOS:000566953100004.

7001. Campbell PJ, Axon DR, Taylor AM, Smith K, Pickering M, Black H, et al. Hypertension, cholesterol and diabetes medication adherence, health care utilization and expenditure in a Medicare Supplemental sample. Medicine. 2021;100(35). doi: 10.1097/md.0000000000027143. PubMed PMID: WOS:000697244600007.

7002. Campbell PJ, Patel M, Martin JR, Hincapie AL, Axon DR, Warholak TL, et al. Systematic review and meta-analysis of community pharmacy error rates in the USA: 1993-2015. BMJ Open Quality. 2018;7(4):e000193. PubMed PMID: 30306141.

7003. Campbell RK, editor Unraveling the mysteries of syndrome X. As the number of Americans with insulin resistance grows, pharmacists can help patients prevent the onset of type 2 diabetes and cardiovascular disease. Journal of the American Pharmacists Association : JAPhA; 2003.

7004. Campino A, Arranz C, Unceta M, Rueda M, Sordo B, Pascual P, et al. Medicine preparation errors in ten Spanish neonatal intensive care units. European Journal of Pediatrics. 2016;175(2):203-10. doi: 10.1007/s00431-015-2615-4. PubMed PMID: 112454222. Language: English. Entry Date: 20161120. Revision Date: 20180713. Publication Type: journal article.

7005. Campino A, Lopez-Herrera MC, Lopez-de-Heredia I, Valls-i-Soler A. Educational strategy to reduce medication errors in a neonatal intensive care unit. Acta Paediatrica. 2009;98(5):782-5.

7006. Campino A, Santesteban E, Pascual P, Sordo B, Arranz C, Unceta M, et al. Strategies implementation to reduce medicine preparation error rate in neonatal intensive care units. European Journal of Pediatrics. 2016;175(6):755-65. doi: 10.1007/s00431-015-2679-1. PubMed PMID: 115376775. Language: English. Entry Date: 20170710. Revision Date: 20180526. Publication Type: journal article.

7007. Campino Villegas A, López Herrera MC, Caballero MI, García Franco M, López de Heredia y Goya I, Valls i Soler A. ¿Tienen los recién nacidos las mismas oportunidades farmacoterapéuticas que los adultos? Anales de Pediatría. 2006;64(5):428-32. doi: https://doi.org/10.1157/13087869.

7008. Campins L, Serra-Prat M, Gózalo I, López D, Palomera E, Agustí C, et al. Randomized controlled trial of an intervention to improve drug appropriateness in community-dwelling polymedicated elderly people. Family Practice. 2017;34(1):36-42. doi: 10.1093/fampra/cmw073. PubMed PMID: 121010696. Corporate Author: REMEI Group. Language: English. Entry Date: 20180121. Revision Date: 20190712. Publication Type: journal article.

7009. Campins L, Serra-Prat M, Gózalo I, López D, Palomera E, Agustí C, et al. Randomized controlled trial of an intervention to improve drug appropriateness in communitydwelling polymedicated elderly people. Family Practice. 2017;34(1):36-42. doi: 10.1093/fampra/cmw073.

7010. Campins L, Serra-Prat M, Palomera E, Bolibar I, Martínez M, Gallo P. Reduction of pharmaceutical expenditure by a drug appropriateness intervention in polymedicated elderly subjects in Catalonia (Spain). Gaceta sanitaria. 2019;33(2):106-11. doi: 10.1016/j.gaceta.2017.09.002. PubMed PMID: CN-01657585.

7011. Campins L, Serra-Prat M, Palomera E, Bolibar I, Martinez MA, Gallo P. Reduction of pharmaceutical

expenditure by a drug appropriateness intervention in polymedicated elderly subjects in Catalonia (Spain). Gaceta Sanitaria. 2019;33(2):106-11. doi: 10.1016/j.gaceta.2017.09.002. PubMed PMID: WOS:000459947800003.

7012. Campling N, Birtwistle J, Richardson A, Bennett IM, Meads D, Santer M, et al. Access to palliative care medicines in the community: An evaluation of practice and costs using case studies of service models in England. International Journal of Nursing Studies. 2022;132. doi: 10.1016/j.ijnurstu.2022.104275. PubMed PMID: WOS:000811849400002.

7013. Campmans Z, van Rhijn A, Dull RM, Santen-Reestman J, Taxis K, Borgsteede SD. Preventing dispensing errors by alerting for drug confusions in the pharmacy information system-A survey of users. PLoS ONE [Electronic Resource]. 2018;13(5):e0197469. PubMed PMID: 29813099.

7014. Campmans Z, Van Rhijn A, Dull RM, Santen-Reestman J, Taxis K, Borgsteede SD. Preventing dispensing errors by alerting for drug confusions in the pharmacy information system—A survey of users. PLoS ONE. 2018;13(5). doi: 10.1371/journal.pone.0197469.

7015. Campo Angora M, Alonso Pérez L, Herreros De Tejada A. Continuity of pharmacotherapy follow-up in polymedicated patients after hospital discharge. Pharmaceutical Care Espana. 2010;12(1):21-8.

7016. Campos EARd, Paula ICd, Pagani RN, Guarnieri P. Reverse logistics for the end-of-life and end-of-use products in the pharmaceutical industry: a systematic literature review. Supply Chain Management: An International Journal. 2017;22(4):375-92.

7017. Campos Gomez S, Morales Perez M, Campos Gomez KA, Toledo Vigueras E, Garcia Garces M, Sanchez Rodriguez I. 1327 Evaluation of pharmacist interventions in reducing medication errors in older (<60 years) cancer patient in an Outpatient Oncology Unit: Mexican Experience. European Journal of Cancer. 2015;51:S196. doi: https://doi.org/10.1016/S0959-8049(16)30570-6.

7018. Campos JADB, Campos LA, Bueno JL, Martins BG. Emotions and mood swings of pharmacy students in the context of the coronavirus disease of 2019 pandemic. Currents in Pharmacy Teaching and Learning. 2021;13(6):635-42. doi: https://doi.org/10.1016/j.cptl.2021.01.034.

7019. Campos MRR, Rodriguez AC. Integrated approach to a complex chronic case through an interdisciplinary team in primary care. Enfermeria Clinica. 2016;26(1):38-44. doi: 10.1016/j.enfcli.2015.11.007. PubMed PMID: WOS:000389364700007.

7020. Campos PD, do Nascimento ACL, de Santana PN, da Costa MF. MAKING UP FOR LOST TIME: ANALYSIS OF REVENGE BUYING INTENTION IN THE POST-PANDEMIC SCENARIO. Revista Brasileira De Marketing. 2023;22(3). doi: 10.5585/remark.v22i3.21129. PubMed PMID: WOS:001157268000009.

7021. Campos TF, de Melo LP, Dantas A, de Oliveira DC, Oliveira R, Cordovil R, et al. Functional activities habits in chronic stroke patients: A perspective based on ICF framework. Neurorehabilitation. 2019;45(1):79-85. doi: 10.3233/nre-192754. PubMed PMID: WOS:000488081200009.

7022. Campsen J, Call T, Rosales A, Allen C, Presson A, Kim R. Prospective, double blind, randomized trial comparing the use of ketorolac and lyrica versus soc+placebo during live donor nephrectomy for transplant. American journal of transplantation. 2018;18:332-. doi: 10.1111/ajt.14917. PubMed PMID: CN-01605134.

7023. Camus D, Dan Glauser ES, Gholamrezaee M, Gasser J, Moulin V. Factors associated with repetitive violent behavior of psychiatric inpatients. Psychiatry Research. 2021;296:113643. doi: https://doi.org/10.1016/j.psychres.2020.113643.

7024. Canadeo A, Fournogerakis M, Zook F. A Multi-disciplinary Approach to Managing Chronic Myelogenous Leukemia Patients on Oral Anticancer Therapy at a Large Academic Medical Center. Current Hematologic Malignancy Reports. 2021;16(6):509-16. doi: 10.1007/s11899-021-00659-9.

7025. Canakis A, Reich J, Shankar D, Jones EA, Weinberg J, Noronha A, et al. INCREASING VACCINATION RATES WITH A PATIENT PORTAL (MYCHART-EPIC) IN PATIENTS WITH INFLAMMATORY BOWEL DISEASE: RESULTS OF A RANDOMIZED CLINICAL TRIAL. Gastroenterology. 2019;156(6):S-29-. doi: 10.1016/S0016-5085(19)36845-3. PubMed PMID: CN-01962825.

7026. Canan C, Alexander GC, Moore R, Murimi I, Chander G, Lau B. Medicaid trends in prescription opioid and non-opioid use by HIV status. Drug and Alcohol Dependence. 2019;197:141-8. doi: https://doi.org/10.1016/j.drugalcdep.2018.11.034.

7027. Cancrinus-Matthijsse AM, Lindenberg SM, Bakker A, Groenewegen PP. The quality of the professional practice of community pharmacists: What can still be improved in Europe? Pharmacy World and Science. 1996;18(6):217-28. doi: 10.1007/BF00735963.

7028. CancrinusMatthijsse AM, Lindenberg SM, Bakker A, Groenewegen PP. The quality of the professional practice of community pharmacists: What can still be improved in Europe? Pharmacy World & Science. 1996;18(6):217-28. doi: 10.1007/bf00735963. PubMed PMID: WOS:A1996WE94600005.

7029. Candelario D, Cunningham K, Solano LA, Pabst A, Srivastava S. Description of a transitions of care and telemedicine simulation lab activity. Currents in Pharmacy Teaching and Learning. 2019;11(11):1184-9. doi: https://doi.org/10.1016/j.cptl.2019.07.017.

7030. Candelario DM, Carson MP. The effect of a centralized transition care discharge unit on reducing hospital readmissions. Journal of General Internal Medicine. 2016;1):S912. PubMed PMID: 72289676.

7031. Candia-de la Rosa RF, Perez-Rodriguez A, Candia-Garcia R, Palacios-Solis JM. Endoscopic transaxillary first rib resection for thoracic outlet syndrome: a safe surgical option. Cirugia Y Cirujanos. 2010;78(1):51-6. PubMed PMID: WOS:000275034900007.

7032. Candon M, Xue L, Shen S, Cole ES, Donohue J, Rothbard A. The impact of opioid prescribing report cards in Medicaid. Journal of Managed Care and Specialty Pharmacy. 2022;28(8):862-70. doi: 10.18553/jmcp.2022.28.8.862.

7033. Cangini A, Fortinguerra F, Di Filippo A, Pierantozzi A, Da Cas R, Villa F, et al. Monitoring the community use of antibiotics in Italy within the National Action Plan on antimicrobial resistance. British Journal of Clinical Pharmacology. 2021;87(3):1033-42. doi: 10.1111/bcp.14461.

7034. Cani C, Lopes L, Queiroz M, Nery M. The impact of educational interventions on patients with type 2 diabetes attending a tertiary care center. Diabetes. 2012;61:A167. doi: 10.2337/db12-656-835. PubMed PMID: CN-01005075.

7035. Cani CG, Lopes Lda S, Queiroz M, Nery M. Improvement in medication adherence and self-management of diabetes with a clinical pharmacy program: a randomized controlled trial in patients with type 2 diabetes undergoing insulin therapy at a teaching hospital. Clinics (Sao Paulo, Brazil). 2015;70(2):102-6. doi: 10.6061/clinics/2015(02)06. PubMed PMID: CN-01110102.

7036. Cani CG, Lopes LDG, Queiroz M, Nery M. Improvement in medication adherence and self-management of diabetes with a clinical pharmacy program: a randomized controlled trial in patients with type 2 diabetes undergoing insulin therapy at a teaching hospital. Clinics. 2015;70(2):102-6. doi: 10.6061/clinics/2015(02)06. PubMed PMID: WOS:000351602700006.

7037. Canning G. Today's Pharmacist: assembling the jigsaw. Pharmacy News. 2011:14-8. PubMed PMID: 59676109. Language: English. Entry Date: 20110408. Revision Date: 20110412. Publication Type: Article.

7038. Canning ML, Munns A, Tai B. Accuracy of best possible medication history documentation by pharmacists at an Australian tertiary referral metropolitan hospital. European journal of hospital pharmacy. 2018;25(e1):E52-E8. doi: 10.1136/ejhpharm-2016-001177. PubMed PMID: CN-01988571.

7039. Cannon AJ, Bargiota A, Billings L, Hunt B, Leiter LA, Malkin S, et al. Evaluation of the Short-Term Cost-Effectiveness of IDegLira Versus Basal Insulin and Basal-Bolus Therapy in Patients with Type 2 Diabetes Based on Attainment of Clinically Relevant Treatment Targets. Journal of managed care & specialty pharmacy. 2020;26(2):143-53. doi: 10.18553/jmcp.2019.19035. PubMed PMID: CN-02076760.

7040. Cannon EC, Zadvorny EB, Sutton SD, Stadler SL, Ruppe LK, Kurz D, et al. Value of Pharmacy Students Performing Population Management Activity Interventions as an Advanced Pharmacy Practice Experience. American Journal of Pharmaceutical Education. 2019;83(5):6759. PubMed PMID: 31333253.

7041. Cannon-Breland ML. An Exploratory Study of Pharmacist Self-Reported Antidepressant Medication Counseling Behaviors [Ph.D.]. United States -- Alabama: Auburn University; 2011.

7042. Cannon-Breland ML, Westrick SC, Kavookjian J, Berger BA, Shannon DM, Lorenz RA. Pharmacist self-reported antidepressant medication counseling. Journal of the American Pharmacists Association. 2013;53(4):390-9. doi: 10.1331/JAPhA.2013.12112.

7043. Cannuscio CC, Hillier A, Karpyn A, Glanz K. The social dynamics of healthy food shopping and store choice in an urban environment. Social Science & Medicine. 2014;122:13-20. doi: 10.1016/j.socscimed.2014.10.005. PubMed PMID: WOS:000345820600002.

7044. Cannuscio CC, Tappe K, Hillier A, Buttenheim A, Karpyn A, Glanz K. Urban Food Environments and Residents' Shopping Behaviors. American Journal of Preventive Medicine. 2013;45(5):606-14. doi: 10.1016/j.amepre.2013.06.021. PubMed PMID: WOS:000325910800010.

7045. Cantarero-Arévalo L, Werremeyer A. Community involvement and awareness raising for better development, access and use of medicines: The transformative potential of photovoice. Research in Social and Administrative Pharmacy. 2021;17(12):2062-9. doi: 10.1016/j.sapharm.2021.05.017.

7046. Canter DL, Atkins MD, McNeal CJ, Bush RL. Risk Factor Treatment in Veteran Women at Risk for Cardiovascular Disease1. Journal of Surgical Research. 2009;157(2):175-80. doi: https://doi.org/10.1016/j.jss.2008.07.014.

7047. Cantin A, Lahaie A, Odobasic B, Tremblay MP, Wazzan D, Caron S, et al. Evaluation of a continuous quality improvement program in anticoagulant therapy: Feasibility, satisfaction and perception. Canadian Pharmacists Journal. 2016;149(6):352-61. doi: 10.1177/1715163516671334.

7048. Cantrell CR, Martin BC. PMC11 DEVELOPMENT AND VALIDATION OF A CLAIMS-BASED RISK ASSESSMENT MODEL TO PREDICT PHARMACY EXPENDITURES IN A U.S. MEDICAID POPULATION. Value in Health. 2005;8(6):A68. doi: https://doi.org/10.1016/S1098-3015(10)67348-2.

7049. Cantrell CR, Martin BC. PHP39 DEVELOPMENT AND VALIDATION OF A CLAIMS-BASED RISK ASSESSMENT MODEL TO PREDICT PHARMACY EXPENDITURES IN A COMMERCIAL POPULATION. Value in Health. 2005;8(3):294. doi: https://doi.org/10.1016/S1098-3015(10)62750-7.

7050. Cantrill J. The PINCER trial: a cluster randomised trial comparing the effectiveness of a pharmacist-led IT-based intervention with simple feedback in reducing rates of clinically important errors in medicines

management in general practices. International journal of pharmacy practice. 2010;18(Suppl S2):5. PubMed PMID: CN-01215936.

7051. Cantudo Cuenca MR, Arenas Jimenez M, Salmeron Cobos AY, Montero Vilchez C, Archilla Amat MI. TELEPHARMACY AND HOME DELIVERY PROGRAMME FOR OUTPATIENTS THROUGH THE COMMUNITY PHARMACY. European Journal of Hospital Pharmacy. 2023;30(Supplement 1):A11. doi: https://dx.doi.org/10.1136/ejhpharm-2023-eahp.25. PubMed PMID: 640966111.

7052. Cantudo-Cuenca MD, Gutierrez-Pizarraya A, Garcia-Lloret P, Gabella-Bazarot E, Morillo-Verdugo R. Prevalence of compliance with PIMDINAC criteria among elderly people living with HIV and in non-infected outpatients with other chronic diseases. European Journal of Hospital Pharmacy. doi: 10.1136/ejhpharm-2022-003454. PubMed PMID: WOS:000907266300001.

7053. Cantudo-Cuenca MR, Jimenez-Morales A, la Plata JEM. Pharmacist-driven antimicrobial stewardship program in a long-term care facility by assessment of appropriateness. European Geriatric Medicine. 2022;13(6):1357-64. PubMed PMID: 36374428.

7054. Cao BY, Chow C, Elliott P, MacPherson RD, Crane J, Bajorek BV. Implementing a Pharmacist Charting Service in the Pre-Admission Clinic. Journal of Pharmacy Practice & Research. 2011;41(2):102-7. doi: 10.1002/j.2055-2335.2011.tb00674.x. PubMed PMID: 62806467. Language: English. Entry Date: 20110720. Revision Date: 20191101. Publication Type: Article.

7055. Cao J, Zhang W, Zhao S, Cai H, Chen B, Ma P. Effect of pharmacist intervention on standardized medication in patients with chronic heart failure. Latin american journal of pharmacy. 2018;37(9):1703-8. PubMed PMID: CN-01922691.

7056. Cao P, Li Y, An B, Ye L, Xu Z. Efficacy and safety of repetitive transcranial magnetic stimulation combined with antidepressants in children and adolescents with depression: A systematic review and meta-analysis. Journal of Affective Disorders. 2023;336:25-34. doi: 10.1016/j.jad.2023.05.051. PubMed PMID: 164110472.

7057. Cao QX. Methods for Analyzing High Dimensional Data with Applications to the Wearable and Microbiome Data Analysis [Ph.D.]. United States -- Montana: University of Montana; 2019.

7058. Cao VFS, Cowley E, Koshman SL, MacGillivray J, Sidsworth M, Turgeon RD. Pharmacist-led optimization of heart failure medications: A systematic review. Journal of the American College of Clinical Pharmacy. 2021;4(7):862-70. doi: 10.1002/jac5.1450. PubMed PMID: WOS:000648976000001.

7059. Cao Y. Medication quota management and dose monitoring system of hangzhou city [Master]. Peoples Rep. of China: Dalian University of Technology (People's Republic of China); 2009.

7060. Cao Y, Hong MH, Chen MD, Xu R, Sun J, Li S, et al. Thoughts and suggestions arising from the review of the clinical research "funding contract" initiated by investigator. Chinese Journal of New Drugs. 2020;29(17):2000-8.

7061. Capelão MAG. Risks Associated With Therapy Medication for Anxiety: Analysis of Adverse Reactions Associated With Benzodiazepines Reported to the System National Pharmacovigilance Professional Experience in the Field of Community Pharmacy, Hospital Pharmacy and In the Field of Community Pharmacy, Hospital Pharmacy and Investigation2023.

7062. Capelle H, Baldin C, Caunes P, Pons I, Meguerditchian C, Argenson J-N, et al. Validation d'une version française de l'outil AT-HARM10 pour la détection des hospitalisations liées au médicament. Therapies. 2023. doi: https://doi.org/10.1016/j.therap.2023.10.015.

7063. Capelle H, Maffre I, Chanoine S, Goyer I, Rasamison R, Berthe-Aucejo A, et al. Creation of an off-label drug prescription tool in pediatrics: The ANTISECHE project. Pharmacien Clinicien. 2023. doi: https://dx.doi.org/10.1016/j.phacli.2023.11.001. PubMed PMID: 2028951882.

7064. Capelle H, Maffre I, Chanoine S, Goyer I, Rasamison R, Berthe-Aucejo A, et al. Création d'un outil d'aide à la prescription médicamenteuse hors AMM en pédiatrie : le projet ANTISECHE. Le Pharmacien Clinicien. 2023. doi: https://doi.org/10.1016/j.phacli.2023.11.001.

7065. Capiau A, Mehuys E, Van Tongelen I, Christiaens T, De Sutter A, Steurbaut S, et al. Community pharmacy-based study of adherence to non-vitamin K antagonist oral anticoagulants. Heart. 2020. doi: 10.1136/heartjnl-2020-316781.

7066. Capiau A, Mehuys E, Van Tongelen I, Christiaens T, De Sutter A, Tommelein E, et al. Detection and management of clinically relevant drug-drug interactions with direct oral anticoagulants in ambulatory care: a prospective interventional study in community pharmacies. European Heart Journal. 2023;44. PubMed PMID: WOS:001115619404492.

7067. Čapková T, Pekárek T, Hanulíková B, Matějka P. Application of reverse engineering in the field of pharmaceutical tablets using Raman mapping and chemometrics. Journal of Pharmaceutical and Biomedical Analysis. 2022;209:114496. doi: https://doi.org/10.1016/j.jpba.2021.114496.

7068. Caplan GA, Brown A, Croker WD, Doolan J. Risk of admission within 4 weeks of discharge of elderly patients from the emergency department - the DEED study. Age and Ageing. 1998;27(6):697-702. doi: 10.1093/ageing/27.6.697. PubMed PMID: WOS:000077415700019.

7069. Caplan PJ, Cohen EH. Rules for new drug development need a serious overhaul. New Scientist. 2007;195(2616):19. doi: 10.1016/S0262-4079(07)62008-9. PubMed PMID: 26299234.

7070. Caplinger C, Crane K, Wilkin M, Bohan J, Remington R, Madaras-Kelly K. Evaluation of a protocol to optimize duration of pneumonia therapy at hospital discharge. American Journal of Health-System Pharmacy. 2016;73(24):2043-54. doi: 10.2146/ajhp160011.

7071. Capó KM, Rutledge DR. Applying managed care performance measures in community pharmacy-based outcomes research. Journal of the American Pharmaceutical Association (Washington,DC : 1996). 1999;39(3):388-94;quiz423. doi: 10.1016/s1086-5802(16)30435-1.

7072. Capoccia KL, Boudreau DM, Blough DK, Ellsworth AJ, Clark DR, Stevens NG, et al. Randomized trial of pharmacist interventions to improve depression care and outcomes in primary care. American journal of health-system pharmacy. 2004;61(4):364-72. doi: 10.1093/ajhp/61.4.364. PubMed PMID: CN-00472197.

7073. Cappelletty D, Jacobs D. Evaluating the impact of a pharmacist's absence from an antimicrobial stewardship team. American Journal of Health-System Pharmacy. 2013;70(12):1065-9. doi: 10.2146/ajhp120482. PubMed PMID: 104190314. Language: English. Entry Date: 20130805. Revision Date: 20200708. Publication Type: Journal Article.

7074. Cappello E, Valdiserra G, Bonaso M, Ferraro S, Convertino I, Tuccori M. Comment on: "Deliberate Self-Poisoning: Real-Time Characterisation of Suicidal Habits and Toxidromes in the Food and Drug Administration Adverse Event Reporting System". Drug Safety. 2023;46(9):917-8. doi: 10.1007/s40264-023-01330-3. PubMed PMID: 170040720.

7075. Cappuzzo KA, Krogsund RR. Postherpetic neuralgia in an elderly patient. Consultant Pharmacist. 2007;22(7):593-8. doi: 10.4140/TCP.n.2007.593.

7076. Capriotti K, Capriotti J, Pelletier J, Stewart K. Chemotherapy-associated paronychia treated with 2% povidone–iodine: A series of cases. Cancer Management and Research. 2017;9:225-8. doi: 10.2147/CMAR.S139301.

7077. Capriotti K, Capriotti J, Pelletier J, Stewart K. Chemotherapy-associated paronychia treated with 2% povidone-iodine: a series of cases. Cancer Management and Research. 2017;9:225-8. doi: 10.2147/cmar.S139301. PubMed PMID: WOS:000404216700001.

7078. Capstick TGD, Azeez NF, Deakin G, Goddard A, Goddard D, Clifton IJ. Ward based inhaler technique service reduces exacerbations of asthma and COPD. Respiratory Medicine. 2021;187:106583. doi: https://doi.org/10.1016/j.rmed.2021.106583.

7079. Capurso KA, Powers MF. Barriers to implementing a phamacist-run immunization service, as perceived by pharmacists, in a community pharmacy chain. Journal of Pharmacy Technology. 2006;22(2):91-4. doi: 10.1177/875512250602200204.

7080. Car J, Tan WS, Huang Z, Sloot P, Franklin BD. eHealth in the future of medications management: personalisation, monitoring and adherence. BMC medicine. 2017;15(1):1-9.

7081. Caraballo PJ, Hodge LS, Bielinski SJ, Stewart AK, Farrugia G, Schultz CG, et al. Multidisciplinary model to implement pharmacogenomics at the point of care. Genetics in Medicine. 2017;19(4):421-9.

7082. Caracuel F, Banos U, Herrera MD, Ramirez G, Munoz N. Influence of pharmaceutical care on the delayed emesis associated with chemotherapy. International journal of clinical pharmacy. 2014;36(2):287-90. doi: 10.1007/s11096-014-9915-z. PubMed PMID: CN-00988131.

7083. Caramlau I, Gallagher J, Hannon B, O'Connor L, Mannion T, Kennedy E, et al. Comprehensive heart failure self-management programme: evaluation of a pilot group intervention. European journal of heart failure. 2018;20:84-5. doi: 10.1002/ejhf.1197. PubMed PMID: CN-01613207.

7084. Carasso BS, Lagarde M, Tesfaye A, Palmer N. Availability of essential medicines in Ethiopia: An efficiency-equity trade-off? Tropical Medicine and International Health. 2009;14(11):1394-400. doi: 10.1111/j.1365-3156.2009.02383.x.

7085. Carayon P. Handbook of human factors and ergonomics in health care and patient safety: CRC press; 2006.

7086. Carayon P, Hundt AS, Karsh B, Gurses AP, Alvarado C, Smith M, et al. Work system design for patient safety: the SEIPS model. BMJ Quality & Safety. 2006;15(suppl 1):i50-i8.

7087. Carayon P, Wetterneck TB, Cartmill R, Blosky MA, Brown R, Hoonakker P, et al. Medication safety in two intensive care units of a community teaching hospital after electronic health record implementation: sociotechnical and human factors engineering considerations. Journal of patient safety. 2021;17(5):e429-e39.

7088. Carayon P, Wetterneck TB, Cartmill R, Blosky MA, Brown R, Kim R, et al. Characterising the complexity of medication safety using a human factors approach: an observational study in two intensive care units. BMJ quality & safety. 2014;23(1):56-65.

7089. Carayon P, Wetterneck TB, Hundt AS, Ozkaynak M, DeSilvey J, Ludwig B, et al. Evaluation of nurse interaction with bar code medication administration technology in the work environment. Journal of Patient Safety. 2007:34-42.

7090. Carayon P, Wetterneck TB, Rivera-Rodriguez AJ, Hundt AS, Hoonakker P, Holden R, et al. Human

factors systems approach to healthcare quality and patient safety. Applied ergonomics. 2014;45(1):14-25.

7091. Carballada ÁM, Lois-González RC. Liberalization and governance in the geographical distribution of pharmacies in Spain. International Journal of Environmental Research and Public Health. 2022;19(1). doi: 10.3390/ijerph19010034.

7092. Carbonell-Abella C, Pages-Castella A, Javaid MK, Nogues X, Farmer AJ, Cooper C, et al. Early (1-year) Discontinuation of Different Anti-osteoporosis Medications Compared: A Population-Based Cohort Study. Calcified Tissue International. 2015;97(6):535-41. doi: 10.1007/s00223-015-0040-3. PubMed PMID: WOS:000363953900001.

7093. Carcelero E, Tuset M, Martin M, De Lazzari E, Codina C, Miro J, et al. Evaluation of antiretroviral-related errors and interventions by the clinical pharmacist in hospitalized HIV-infected patients. HIV Medicine. 2011;12(8):494-9. PubMed PMID: 21395966.

7094. Carcelero E, Tuset M, Martin M, De Lazzari E, Codina C, Miro J, et al. Evaluation of antiretroviral-related errors and interventions by the clinical pharmacist in hospitalized HIV-infected patients. Hiv Medicine. 2011;12(8):494-9. doi: 10.1111/j.1468-1293.2011.00915.x. PubMed PMID: WOS:000293749400006.

7095. Cárdenas Valladolid J, María Mena Mateo J, Asunción Cañada Dorado M, Rodríguez Morales D, Sánchez Perruca L. Introduction and improvement of a care program for elderly on multiple medication in a primary care area. Revista de Calidad Asistencial. 2009;24(1):24-31. doi: 10.1016/S1134-282X(09)70072-7.

7096. Cardinale S, Saraon T, Lodoe N, Alshehry A, Raffoul M, Caspers C, et al. Clinical Pharmacist Led Medication Reconciliation Program in an Emergency Department Observation Unit. Journal of Pharmacy Practice. 2023;36(5):1156-63. doi: 10.1177/08971900221091174. PubMed PMID: 172290279.

7097. Cardinale V. A look at retail, hospital R.Ph.s' influence in four areas. Drug Topics. 2007;151(15).

7098. Cardo Prats E, Corella Piquer MD, García Cebrián F, Mañes Vinuesa J. Pharmaceutical care in hiperlipemic drug patients. Pharmaceutical Care Espana. 2001;3(2):95-104.

7099. Cardona JAM, Erira DAH, Nati-Castillo HA, Sánchez-Vallejo J, Izquierdo-Condoy JS. Characteristics of Inhaler Technique in COPD Patients in a Specialized Center in Armenia, Colombia: A Cross-Sectional Study. Patient Preference & Adherence. 2023;17:2025-38. doi: 10.2147/PPA.S417375. PubMed PMID: 172341938.

7100. Cardona M, Wirth F, Serracino-Inglott A, Xuereb RG, Azzopardi LM. Impact of rivaroxaban in cardiovascular disease. International journal of clinical pharmacy Conference: 46th ESCP symposium on clinical pharmacy Germany. 2018;40(1):226-7. doi: 10.1007/s11096-017-0565-9. PubMed PMID: CN-01468270.

7101. Cardona Pera D. XIII Lesson Jesús Culebras. Hospital pharmacists and nutritional support. From the beginning to the present day (1976-2018). Nutricion Hospitalaria. 2023;40(1):200-12. doi: 10.20960/nh.04574.

7102. Cardone KE, Bacchus S, Assimon MM, Pai AB, Manley HJ. Medication-related Problems in CKD. Advances in Chronic Kidney Disease. 2010;17(5):404-12. doi: https://doi.org/10.1053/j.ackd.2010.06.004.

7103. Cardone KE, Maxson R, Cho KH, Davis JM, El Nekidy WS, Kane-Gill SL, et al. Pharmacy Practice Standards for Outpatient Nephrology Settings. Kidney Medicine. 2022;4(8). doi: 10.1016/j.xkme.2022.100509. PubMed PMID: WOS:000844303300001.

7104. Cardosi L, Hohmeier KC, Fisher C, Wasson M. Patient Satisfaction With a Comprehensive Medication Review Provided by a Community Pharmacist. Journal of Pharmacy Technology. 2018;34(2):48-53. doi: 10.1177/8755122517752158.

7105. Cardoso ACC. Agonistas ß2 e Doping: O Aumento da Performance Física em Atletas: Experiência Profissionalizante na Vertente de Investigação, Farmácia Hospitalar e Farmácia Comunitária [M.Sc.]. Portugal: Universidade da Beira Interior (Portugal); 2019.

7106. Cardoso AF. Aplicabilidade de Métodos Quantitativos de Previsão da Procura ao Consumo de Benzodiazepinas de uma Farmácia Comunitária: Experiência Profissionalizante na Vertente de Farmácia Hospitalar, Comunitária e Investigação [Master's]. Portugal: Universidade da Beira Interior (Portugal); 2013.

7107. Cardoso CL. Seguimento Farmacoterapêutico num Grupo de Doentes Crónicos: A Importância da Adesão: Experiência Profissionalizante na Vertente de Farmácia Hospitalar, Comunitária e Investigação [Master's]. Portugal: Universidade da Beira Interior (Portugal); 2013.

7108. Cardoso RG. Avaliação do Uso de Medicamentos Potencialmente Inapropriados em Idosos Institucionalizados em Lares: Aplicação dos Critérios de Beers: Experiência Profissionalizante na Vertente de Farmácia Comunitária [Master's]. Portugal: Universidade da Beira Interior (Portugal); 2013.

7109. Cardozo T. Pharmacy Manager System Implementation Strategies to Mitigate the Cost of Prescription Errors2023.

7110. Cardwell K. Potentially inappropriate prescribing in older people: outcomes and assessments [Ph.D.]. Northern Ireland: Queen's University Belfast (United Kingdom); 2017.

7111. Cardwell K, Hughes CM, Ryan C. Community pharmacists' views of using a screening tool to structure medicines use reviews for older people: findings from qualitative interviews. International Journal of Clinical Pharmacy. 2018;40(5):1086-95. doi: 10.1007/s11096-018-0659-z. PubMed PMID: WOS:000448698900022.

7112. Cardwell K, Kerse N, Hughes CM, Teh R, Moyes SA, Menzies O, et al. Does potentially inappropriate prescribing predict an increased risk of admission to hospital and mortality? A longitudinal study of the 'oldest old'. BMC Geriatrics. 2020;20(1):1-9. doi: 10.1186/s12877-020-1432-4. PubMed PMID: 141431906. Language: English. Entry Date: 20200715. Revision Date: 20211019. Publication Type: journal article.

7113. Cardwell K, Smith SM, Clyne B, McCullagh L, Wallace E, Kirke C, et al. Evaluation of the General Practice Pharmacist (GPP) intervention to optimise prescribing in Irish primary care: a non-randomised pilot study. BMJ open. 2020;10(6):e035087. doi: 10.1136/bmjopen-2019-035087. PubMed PMID: CN-02139406.

7114. Carey EC, Walter LC, Lindquist K, Covinsky KE. Development and validation of a functional morbidity index to predict mortality in community-dwelling elders. Journal of General Internal Medicine. 2004;19(10):1027-U31. doi: 10.1111/j.1525-1497.2004.40016.x. PubMed PMID: WOS:000224347100006.

7115. Carey HB. Educational Problems in Pharmacy**Abstracts from an address before the Section on Education and Legislation, A. Ph. A., Portland meeting, 1935. The Journal of the American Pharmaceutical Association (1912). 1937;26(4):335-40. doi: https://doi.org/10.1002/jps.3080260416.

7116. Carey RG, Teeters JL. CQI Case Study: Reducing Medication Errors. The Joint Commission Journal on Quality Improvement. 1995;21(5):232-7. doi: https://doi.org/10.1016/S1070-3241(16)30144-4.

7117. Carfagno ML, Schechter LN. Regional anesthesia and acute pain management: A pharmacist's perspective. Techniques in Regional Anesthesia and Pain Management. 2002;6(2):77-86. doi: https://doi.org/10.1053/trap.2002.122933.

7118. Caria JJMA. Atividade anti-Candida do óleo essencial de Helichrysum italicum: Experiência profissionalizante na vertente de farmácia hospitalar, farmácia comunitária e investigação [Master's]. Portugal: Universidade da Beira Interior (Portugal); 2012.

7119. Carico R, Hussain N, Sheppard J, Thomas CB, Fenerty J. Syringe services programs and real-world research: An overview for pharmacists. Journal of the American Pharmacists Association. 2020;60(5):e29-e33. doi: 10.1016/j.japh.2020.02.029.

7120. Caricol FC. Omeprazole safety: is the duration of the treatments suitable? Farmaceuticos Comunitarios. 2015;7(1):5-9. doi: 10.5672/FC.2173-9218.(2015/Vol7).001.02. PubMed PMID: WOS:000417265600002.

7121. Carino SR, Sperry DC, Hawley M. Relative Bioavailability of Three Different Solid Forms of PNU-141659 as Determined with the Artificial Stomach-Duodenum Model. Journal of Pharmaceutical Sciences. 2010;99(9):3923-30. doi: https://doi.org/10.1002/jps.22236.

7122. Cariveau D, Fay AE, Baker D, Fagan EB, Wilson CG. Evaluation of a pharmacist-led naloxone coprescribing program in primary care. Journal of the American Pharmacists Association. 2019;59(6):867-71. doi: https://doi.org/10.1016/j.japh.2019.07.012.

7123. Carlan SJ, O'Brien WF, Jones MH, O'Leary TD, Roth L. Outpatient oral sulindac to prevent recurrence of preterm labor. Obstetrics and gynecology. 1995;85(5 Pt 1):769-74. doi: 10.1016/0029-7844(95)00016-k. PubMed PMID: CN-00113251.

7124. Carlan SJ, Obrien WF, Jones MH, Oleary TD, Roth L. OUTPATIENT ORAL SULINDAC TO PREVENT RECURRENCE OF PRETERM LABOR. Obstetrics and Gynecology. 1995;85(5):769-74. doi: 10.1016/0029-7844(95)00016-k. PubMed PMID: WOS:A1995QU28900027.

7125. Carle PDA, Uema SAN, Perez SRM, Pallares MM, Uthurry NHS, Dader MJF. Validation of a questionnaire on patient satisfaction with the dispensing service in community pharmacies. Atencion Primaria. 2007;39(11):591-6. PubMed PMID: WOS:000254291300003.

7126. Carli D, Guignard B, Bonnabry P, Christian L. Can clinical pharmacist interventions be substituted by a clinical decision support system in the electronic patient record? Le Pharmacien Hospitalier et Clinicien. 2015;50(3):314. doi: https://doi.org/10.1016/j.phclin.2015.07.006.

7127. Carlson AM, Morris LS. Coprescription of Terfenadine and Erythromycin or Ketaconazole: An Assessment of Potential Harm: Pharmacists can be key in changing prescribing behavior to avoid drug-drug interactions. Journal of the American Pharmaceutical Association (1996). 1996;36(4):263-9. doi: https://doi.org/10.1016/S1086-5802(16)30050-X.

7128. Carlson AM, Stockwell Morris L. Coprescription of terfenadine and erythromycin or ketoconazole: An assessment of potential harm. Journal of the American Pharmaceutical Association. 1996;36(4):263-9. doi: 10.1016/S1086-5802(16)30050-X.

7129. Carlson JD, Neumiller JJ, Swain LDW, Mark J, McLeod P, Hirschauer J. Postoperative delirium in Parkinson’s disease patients following deep brain stimulation surgery. Journal of Clinical Neuroscience. 2014;21(7):1192-5. doi: https://doi.org/10.1016/j.jocn.2013.12.007.

7130. Carlson KF, Gilbert TA, Morasco BJ, Wright D, Van Otterloo J, Herrndorf A, et al. Linkage of VA and State Prescription Drug Monitoring Program Data to Examine Concurrent Opioid and Sedative-Hypnotic Prescriptions among Veterans. Health Services Research. 2018;53:5285-308. doi: 10.1111/1475-6773.13025. PubMed PMID: WOS:000450256200009.

7131. Carlson KM, Schroth C, Lammie D, Leehey D, Koppes T, Polzin L, et al. Ptxrx study: how pragmatic trial design can inoculate against risks to study integrity in a global pandemic. Clinical trials (London, England). 2021;18(SUPPL 5):98-. doi: 10.1177/17407745211043721. PubMed PMID: CN-02371186.

7132. Carlson SE, Gajewski BJ, Alhayek S, Colombo J, Kerling EH, Gustafson KM. Dose-response relationship between docosahexaenoic acid (DHA) intake and lower rates of early preterm birth, low birth weight and very low birth weight. Prostaglandins, leukotrienes, and essential fatty acids. 2018;138:1-5. doi: 10.1016/j.plefa.2018.09.002. PubMed PMID: CN-01668332.

7133. Carlton B, Donald Weston W. Changing health professions education in West Virginia. Academic Medicine. 1997;72(2):110-5. doi: 10.1097/00001888-199702000-00011.

7134. Carlton B, Weston WD. Changing health professions education in West Virginia. Academic Medicine. 1997;72(2):110-5. doi: 10.1097/00001888-199702000-00011. PubMed PMID: WOS:A1997WM33300013.

7135. Carmen B, Angeles M, Ana M, María AJ. Efficacy and safety of naltrexone and acamprosate in the treatment of alcohol dependence: a systematic review. Addiction. 2004;99(7):811-28.

7136. Carmen SG, Juan ARR, Ana SD, Lucia AP, Ana FV, Angel CHM. ROLE OF THE COMMUNITY PHARMACIST IN THE MANAGEMENT OF ARTERIAL HYPERTENSION. Atencion Farmaceutica. 2012;14(2):108-15. PubMed PMID: WOS:000305503400005.

7137. Carmenates J, Keith MR. Impact of automation on pharmacist interventions and medication errors in a correctional health care system. American Journal of Health-System Pharmacy. 2001;58(9):779-83. PubMed PMID: 11351917.

7138. Carmichael JM, Alvarez A, Chaput R, DiMaggio J, Magallon H, Mambourg S. Establishment and outcomes of a model primary care pharmacy service system. American journal of health-system pharmacy. 2004;61(5):472-82.

7139. Carmichael JM, Alvarez A, Chaput R, Jennifer Dimaggio R, Magallon H, Mambourg S. Establishment and outcomes of a model primary care pharmacy service system. American Journal of Health-System Pharmacy. 2004;61(5):472-82. PubMed PMID: 106081297. Language: English. Entry Date: 20070101. Revision Date: 20150711. Publication Type: Journal Article. Journal Subset: Biomedical.

7140. Carmichael JM, Cichowlas JA. The changing role of pharmacy practice--a clinical perspective. Specialty Law Digest. 2002;Health Care Law.(280):9-20. PubMed PMID: 12235758.

7141. Carmichael JM, Meier J, Robinson A, Taylor J, Higgins DT, Patel S. Leveraging electronic medical record data for population health management in the Veterans Health Administration: Successes and lessons learned. American Journal of Health-System Pharmacy. 2017;74(18):1447-59. PubMed PMID: 28887346.

7142. Carmona Araújo A, Fernandes E, Franco Ruivo I, Do Céu Machado M, Faria Vaz A, Furtado C. Prevalence of Outpatient Use of Medicines by the Elderly Population in Portugal: A Cross-Sectional Study. Acta Medica Portuguesa. 2023;36(12):792-801. doi: 10.20344/amp.19254.

7143. Carmosino E, Ruisinger JF, Kinsey JD, Melton BL. Vaccination approval literacy and its effects on intention to receive future COVID-19 immunization. Journal of the American Pharmacists Association. 2022;62(4):1374-8.e2. doi: https://doi.org/10.1016/j.japh.2022.01.001.

7144. Carnahan BJ, Maghsoodloo S, Flynn EA, Barker KN. Geometric probability distribution for modeling of error risk during prescription dispensing. American Journal of Health-System Pharmacy. 2006;63(11):1056-61. doi: 10.2146/ajhp040146. PubMed PMID: 106368870. Language: English. Entry Date:

20061201. Revision Date: 20200708. Publication Type: Journal Article.

7145. Carnahan W, Cooper TY. Neoral-to-gengraf conversion in renal transplant recipients. Transplantation Proceedings. 2003;35(4):1308-13. doi: 10.1016/S0041-1345(03)00423-8.

7146. Carneiro ARC. As Plantas Medicinais no Tratamento da Gota: Experiência Profissionalizante na Vertente de Farmácia Comunitária, Hospitalar e Investigação [M.Sc.]. Portugal: Universidade da Beira Interior (Portugal); 2016.

7147. Carnelio S, Khan SA, Rodrigues G. Pharmacovigilance in clinical dentistry: overlooked or axiomatic? General Dentistry. 2011;59(1):24-8; quiz 9-30, 80. PubMed PMID: 21613036.

7148. Carnes P, Koons K. Leading the next renovation: Redesigning a pharmacy structural workspace. Journal of the American Pharmacists Association. 2010;50(3):416-8. doi: 10.1331/JAPhA.2010.09090. PubMed PMID: WOS:000277802800016.

7149. Carney AS, Price DB, Smith PK, Harvey R, Kritikos V, Bosnic-Anticevich SZ, et al. Seasonal patterns of oral antihistamine and intranasal corticosteroid purchases from Australian community pharmacies: a retrospective observational study. Pragmatic and Observational Research. 2017;8:157-65. doi: 10.2147/por.S134266. PubMed PMID: WOS:000409016800001.

7150. Carney G, Kim JD, O'Sullivan C, Thompson W, Bassett K, Levin J, et al. Treatment pattern trends of medications for type 2 diabetes in British Columbia, Canada. BMJ Open Diabetes Research and Care. 2022;10(6). doi: 10.1136/bmjdrc-2022-002995.

7151. Carney PA, Bumatay S, Kuo GM, Darden PM, Hamilton A, Fagnan LJ, et al. The interface between US primary care clinics and pharmacies for HPV vaccination delivery: A scoping literature review. Preventive Medicine Reports. 2022;28. doi: 10.1016/j.pmedr.2022.101893. PubMed PMID: WOS:000835262200012.

7152. Carney T, Wells J, Bergin M, Dada S, Foley M, McGuiness P, et al. A Comparative Exploration of Community Pharmacists' Views on the Nature and Management of Over-the-Counter (OTC) and Prescription Codeine Misuse in Three Regulatory Regimes: Ireland, South Africa and the United Kingdom. International Journal of Mental Health and Addiction. 2016;14(4):351-69. doi: 10.1007/s11469-016-9640-z.

7153. Carney T, Wells J, Parry CDH, McGuinness P, Harris R, Van Hout MC. A comparative analysis of pharmacists' perspectives on codeine use and misuse - a three country survey. Substance Abuse Treatment Prevention and Policy. 2018;13. doi: 10.1186/s13011-018-0149-2. PubMed PMID: WOS:000428569800001.

7154. Caro JJ, Lee K. Pharmacoeconomic evaluation of a pharmacist-managed hypertension clinic. Current hypertension reports. 2002;4(6):418. PubMed PMID: CN-01714617.

7155. Caro JJ, Speckman JL, Salas M, Raggio G, Jackson JD. Effect of initial drug choice on persistence with antihypertensive therapy: the importance of actual practice data. Cmaj. 1999;160(1):41-6.

7156. Caroff DA, Bittermann T, Leonard CE, Gibson GA, Myers JS. A Medical Resident–Pharmacist Collaboration Improves the Rate of Medication Reconciliation Verification at Discharge. The Joint Commission Journal on Quality and Patient Safety. 2015;41(10):457-61. doi: https://doi.org/10.1016/S1553-7250(15)41059-1.

7157. Caroff DA, Bittermann T, Leonard CE, Gibson GA, Myers JS. A Medical Resident-Pharmacist Collaboration Improves the Rate of Medication Reconciliation Verification at Discharge. Joint Commission Journal on Quality & Patient Safety. 2015;41(10):457-61. PubMed PMID: 26404074.

7158. Caron J, Barker M, Schimmoeller N. Remote Provision and Self-Administration of Misoprostol as Cervical Preparation for Hospital-Based Uterine Evacuation Between 14-18 Weeks. Contraception. 2023;121:110016. doi: https://doi.org/10.1016/j.contraception.2023.110016.

7159. Caron MF, Dore DD, Min B, Kluger J, Boguk I, White CM. Electrocardiographic and blood pressure effects of the ephedra-containing TrimSpa thermogenic herbal compound in healthy volunteers. Pharmacotherapy. 2006;26(9):1241-6. doi: 10.1592/phco.26.9.1241. PubMed PMID: CN-00571950.

7160. Carow F, Rieger K, Walter-Sack I, Meyer MR, Peters FT, Maurer HH, et al. Objective assessment of nonadherence and unknown co-medication in hospitalized patients. European Journal of Clinical Pharmacology. 2012;68(8):1191-9. PubMed PMID: 22354152.

7161. Carpay JA, Aalbers K, Graveland GA, Engelsman M. Retention of new AEDs in institutionalized intellectually disabled patients with epilepsy. Seizure. 2009;18(2):119-23. doi: https://doi.org/10.1016/j.seizure.2008.07.007.

7162. Carpay JA, Aldenkamp AP, van Donselaar CA. Complaints associated with the use of antiepileptic drugs: Results from a community-based study. Seizure. 2005;14(3):198-206. doi: 10.1016/j.seizure.2005.01.008.

7163. Carpenter CF, Chambers HF. Daptomycin: another novel agent for treating infections due to drug-resistant gram-positive pathogens. Clinical infectious diseases. 2004;38(7):994-1000.

7164. Carpenter CR, Hammouda N, Linton E, Doering M, Obuabunwa U, Ko KJ, et al. Geriatric emergency care applied research network (GEAR) delirium research prioritization. Journal of the American Geriatrics Society. 2020;68(SUPPL 1):S237-. doi: 10.1111/jgs.16431. PubMed PMID: CN-02229260.

7165. Carpenter D, Geryk L, Blalock S, Lee C, Weaver M, Reuland D, et al. Using videos to improve children's inhaler technique: a randomized controlled trial. Journal of the american pharmacists association. 2014;54(2):e99-e100. doi: 10.1331/JAPhA.2014.14511. PubMed PMID: CN-00999989.

7166. Carpenter DM, Abraham O, Alexander DS, Horowitz K. Counseling of children and adolescents in community pharmacies: results from a 14-day observational study. Journal of the American Pharmacists Association. 2016;56(3):266-9. e1.

7167. Carpenter DM, Abraham O, Alexander DS, Horowitz K, editors. Counseling of children and adolescents in community pharmacies: Results from a 14-day observational study. Journal of the American Pharmacists Association; 2016.

7168. Carpenter DM, Blalock SJ, DeVellis RF. Patients with rare diseases using pharmacists for medication information. Journal of the American Pharmacists Association. 2012;52(6):e175-e82. doi: 10.1331/JAPhA.2012.12047.

7169. Carpenter DM, Lavigne JE, Colmenares EW, Falbo K, Mosley SL. Community pharmacy staff interactions with patients who have risk factors or warning signs of suicide. Research in Social and Administrative Pharmacy. 2020;16(3):349-59. doi: https://doi.org/10.1016/j.sapharm.2019.05.024.

7170. Carpenter DM, Lavigne JE, Roberts CA, Zacher J, Colmenares EW. A review of suicide prevention programs and training policies for pharmacists. Journal of the American Pharmacists Association. 2018;58(5):522-9. doi: https://doi.org/10.1016/j.japh.2018.05.004.

7171. Carpenter DM, Roberts CA, Farley JF. Measuring patient perspectives on community pharmacy quality: Psychometric evaluation of a brief survey. Journal of Managed Care and Specialty Pharmacy.

2021;27(1):105-11. doi: 10.18553/JMCP.2021.27.1.105.

7172. Carpenter DM, Roberts CA, Lavigne JE, Cross WF, editors. Gatekeeper training needs of community pharmacy staff. Suicide and Life-Threatening Behavior; 2021.

7173. Carpenter DM, Roberts CA, Lavigne JE, Cross WF. Gatekeeper training needs of community pharmacy staff. Suicide and Life-Threatening Behavior. 2021;51(2):220-8. doi: 10.1111/sltb.12697. PubMed PMID: WOS:000641276600006.

7174. Carpenter DM, Roberts CA, Westrick SC, Ferreri SP, Kennelty KA, Look KA, et al. A content review of online naloxone Continuing Education courses for pharmacists in states with standing orders. Research in Social & Administrative Pharmacy. 2018;14(10):968-78. doi: 10.1016/j.sapharm.2017.11.011. PubMed PMID: WOS:000444008000013.

7175. Carpenter DM, Stover AN, Harris SC, Anksorus H, Lavigne JE. Impact of a Brief Suicide Prevention Training with an Interactive Video Case Assessment on Student Pharmacist Outcomes. American Journal of Pharmaceutical Education. 2023;87(7):100093. doi: https://doi.org/10.1016/j.ajpe.2023.100093.

7176. Carpes MJ, Cerda B, Ballester P, Zafrilla P. Pacient with chronic obstructive pulmonary disease adherence and satisfaction in community pharmacy. Pharmaceutical Care Espana. 2022;24(5):13-26. PubMed PMID: WOS:000879069900003.

7177. Carpini JA, Sharma A, Kubicki Evans M, Jumani S, Boyne E, Clifford R, et al. Pharmacists and Mental Health First Aid training: A comparative analysis of confidence, mental health assistance behaviours and perceived barriers. Early Intervention in Psychiatry. 2023;17(7):670-80. doi: 10.1111/eip.13361.

7178. Carpino F. Quadrupole magnetic field-flow fractionation: A novel technique for the characterization of magnetic particles [Ph.D.]. United States -- Ohio: Cleveland State University; 2008.

7179. Carr G, Hoover R, Smith R, Bagby C. Stereotypes and team dynamics. Journal of the american pharmacists association. 2018;58(3):e140-. doi: 10.1016/j.japh.2018.04.004. PubMed PMID: CN-01612170.

7180. Carr S. Examining health professional students' attitudes on interprofessional education [Ph.D.]. United States -- Rhode Island: University of Rhode Island; 2015.

7181. Carr W, Kelley AL, Toolin CF, Weber NS. Association of MOS-Based Blast Exposure With Medical Outcomes. Frontiers in Neurology. 2020;11. doi: 10.3389/fneur.2020.00619. PubMed PMID: WOS:000561662200001.

7182. Carr-Locke DL. Role of endoscopic stenting in the duodenum. Annals of Oncology. 1999;10:261-4. doi: 10.1023/a:1008372600666. PubMed PMID: WOS:000081799100065.

7183. Carr-Lopez SM, Strohecker L, Miyahara R, Mai Y, Shek A. The addition of women's health initiatives to an established remote population health introductory pharmacy practice experience. Journal of the American Pharmacists Association. 2022;62(1):357-62. doi: https://doi.org/10.1016/j.japh.2021.09.005.

7184. Carr-Lopez SM, Woelfel JA, Shioji M, Nicolas M, Hoang D, Rodriguez J, et al. Health care Passport: A population-based introductory pharmacy practice model for Medicare beneficiaries. Currents in Pharmacy Teaching and Learning. 2014;6(6):891-9. doi: https://doi.org/10.1016/j.cptl.2014.08.003.

7185. Carranza V, Nastoupil LJ, Taylor BC, Weaver R. Impact of Live Education on Knowledge and Competence in Clinicians Who Specialize in Hematologic Malignancies: An Interprofessional Initiative into Community Practice. Blood. 2020;136:16-7. doi: https://doi.org/10.1182/blood-2020-139692.

7186. Carrera M, Villas-Boas S. Generic aversion and observational learning in the over-the-counter drug market. American Economic Journal: Applied Economics. 2023;15(3):380-410.

7187. Carreras M, Serra D. On optimal location with threshold requirements. Socio-Economic Planning Sciences. 1999;33(2):91-103. doi: https://doi.org/10.1016/S0038-0121(98)00005-6.

7188. Carrez L, Bouchoud L, Fleury S, Combescure C, Falaschi L, Sadeghipour F, et al. Work overload is related to increased risk of error during chemotherapy preparation. Journal of Oncology Pharmacy Practice. 2019;25(6):1456-66. doi: 10.1177/1078155219845432. PubMed PMID: 137592603. Language: English. Entry Date: 20190724. Revision Date: 20200113. Publication Type: Article.

7189. Carrido DI, Macaldo ACL, Lorenzo CAA, Loquias MM. Evaluation of community pharmacy internship programme in the Philippines. Pharmacy Education. 2016;16(1):103-8.

7190. Carrier J. Community-based multidisciplinary screening and intervention by pharmacists and nurses reduced BP in diabetes. Evidence-based nursing. 2009;12(3):77. doi: 10.1136/ebn.12.3.77. PubMed PMID: CN-00746438.

7191. Carrigan A, Roberts N, Han J, John R, Khan U, Sultani A, et al. The Digital Hospital: A Scoping Review of How Technology Is Transforming Cardiopulmonary Care. Heart, Lung & Circulation. 2023;32(9):1057-68. doi: 10.1016/j.hlc.2023.06.725. PubMed PMID: 172775859.

7192. Carrilho JdRF. Adequação do Método Dáder no Seguimento Farmacoterapêutico em Doentes Hipertensos Institucionalizados num Lar de Idosos: Experiência Profissionalizante na Vertente de Farmácia Comunitária e Investigação [Master's]. Portugal: Universidade da Beira Interior (Portugal); 2013.

7193. Carrillo GLB, Gomez SAS, Calva LEG, Solis DAS. Prescription of gastric protectors in rheumatological patients. Analysis from a gastroenterology outpatient clinic. Revista Cubana De Reumatologia. 2022;24(2). PubMed PMID: WOS:000805840400003.

7194. Carrillo M, Sias J, Navarrete JP, Aboud S, Valenzuela E. EXPANSION of diabetes education in a United States–Mexico border community (Expanding Services for Patients to Acquire New Skills, Set Goals, and Improve Overall Knowledge). Journal of the American Pharmacists Association. 2018;58(1):30-5. doi: 10.1016/j.japh.2017.08.008.

7195. Carrillo M, Sias J, Navarrete JP, Aboud S, Valenzuela E. EXPANSION of diabetes education in a United States-Mexico border community (Expanding Services for Patients to Acquire New Skills, Set Goals, and Improve Overall Knowledge). Journal of the American Pharmacists Association. 2018;58(1):30-5. doi: 10.1016/j.japh.2017.08.008. PubMed PMID: WOS:000418895800008.

7196. Carrington C, Stone L, Koczwara B, Searle C, Siderov J, Stevenson B, et al. The Clinical Oncological Society of Australia (COSA) guidelines for the safe prescribing, dispensing and administration of cancer chemotherapy. Asia-Pacific Journal of Clinical Oncology. 2010;6(3):220-37.

7197. Carrión JA, Gonzalez-Colominas E, García-Retortillo M, Cañete N, Cirera I, Coll S, et al. A multidisciplinary support programme increases the efficiency of pegylated interferon alfa-2a and ribavirin in hepatitis C. Journal of hepatology. 2013;59(5):926-33. doi: 10.1016/j.jhep.2013.06.019. PubMed PMID: CN-01122783.

7198. Carrion Madronal IM, Martin Alcalde E, Bartolome Martin I, Febres Panez DF, Colato Lopez CA, Rodriguez-Minon Otero MI. Appropriateness of medication prescribing in nursing homes: A collaboration project between pharmacy and geriatric departments. Revista Espanola de Geriatria y Gerontologia. 2023;58(2):68-74. doi: https://dx.doi.org/10.1016/j.regg.2023.01.008. PubMed PMID: 2022877040.

7199. Carris NW, Cheng F, Kelly WN. The changing cost to prevent diabetes: a retrospective analysis of the Diabetes Prevention Program. Journal of the american pharmacists association. 2017;57(6):717-22. doi: 10.1016/j.japh.2017.05.015. PubMed PMID: CN-02363010.

7200. Carroll A, Kapilashrami A. Barriers to uptake of reproductive information and contraceptives in rural Tanzania: An intersectionality informed qualitative enquiry. BMJ Open. 2020;10(10). doi: 10.1136/bmjopen-2019-036600.

7201. Carroll B, Irwin DE. Health Care Resource Utilization and Costs for Patients with Tardive Dyskinesia. Journal of Managed Care & Specialty Pharmacy. 2019;25(7):810-+. doi: 10.18553/jmcp.2019.25.7.810. PubMed PMID: WOS:000472567600010.

7202. Carroll CA. Applications of economic theory to outpatient prescription markets [Ph.D.]. United States -- Kansas: University of Kansas; 1996.

7203. Carroll CA, Fairman KA, Lage MJ. Updated cost-of-care estimates for commercially insured patients with multiple sclerosis: retrospective observational analysis of medical and pharmacy claims data. Bmc Health Services Research. 2014;14. doi: 10.1186/1472-6963-14-286. PubMed PMID: WOS:000340063700001.

7204. Carroll CA, Oleen-Burkey MA. Assessing variations in transitions in employment in relapsingremitting multiple sclerosis patients treated with either laquinimod, interferon beta 1-a or placebo: expoloratory evidence from the United States substudy of bravo. Value in health. 2012;15(4):A149. doi: 10.1016/j.jval.2012.03.803. PubMed PMID: CN-01033204.

7205. Carroll DG, Noble SL. Hormone replacement therapy: current concerns and considerations. American journal of managed care. 2002;8(7):663-75; quiz 76-8. PubMed PMID: CN-01784315.

7206. Carroll J, Edmondson AC. Leading organisational learning in health care. BMJ Quality & Safety. 2002;11(1):51-6.

7207. Carroll J, Quijada M. Redirecting traditional professional values to support safety: changing organisational culture in health care. BMJ Quality & Safety. 2004;13(suppl 2):ii16-ii21.

7208. Carroll JC, Jiang C, Mook H, Somma McGivney MA, Coley KC. Patients' opinions on participation in patient-centered outcomes research in community pharmacies: A qualitative study. Journal of the American Pharmacists Association. 2023;63(1):182-7. doi: 10.1016/j.japh.2022.09.001.

7209. Carroll JC, Jiang C, Mook H, Somma McGivney MA, Coley KC. Patients' opinions on participation in patient-centered outcomes research in community pharmacies: A qualitative study. Journal of the American Pharmacists Association. 2023;63(1):182-7. doi: 10.1016/j.japh.2022.09.001.

7210. Carroll JC, Jiang CY, Mook H, McGivney MAS, Coley KC. Patients' opinions on participation in patient-centered outcomes research in community pharmacies: A qualitative study. Journal of the American Pharmacists Association. 2023;63(1):182-7. doi: 10.1016/j.japh.2022.09.001. PubMed PMID: WOS:000924063700001.

7211. Carroll JC, Renner HM, McGivney MS, Grapsy J, McCormick K, Cooney SS, et al. Using prescription dispensing data infographics to facilitate collaborative pharmacist-prescriber discussions on mutual patients. Journal of the American Pharmacists Association. 2019;59(2):232-7.e1. doi: https://doi.org/10.1016/j.japh.2018.11.014.

7212. Carroll NV. How effectively do managed care organizations influence prescribing and dispensing

decisions? American Journal of Managed Care. 2002;8(12):1041-54. PubMed PMID: WOS:000179873100002.

7213. Carroll NV. Do community pharmacists influence prescribing? Journal of the American Pharmacists Association. 2003;43(5):612-21.

7214. Carroll NV. Financial management for pharmacists: A decision-making approach: Lippincott Williams & Wilkins; 2007.

7215. Carroll NV. Estimating the impact of Medicare Part D on the profitability of independent community pharmacies. Journal of Managed Care Pharmacy. 2008;14(8):768-79.

7216. Carroll NV, Brusilovsky I, York B, Oscar R. Comparison of costs of community and mail service pharmacy. Journal of the American Pharmacists Association. 2005;45(3):336-43. doi: 10.1331/1544345054003796.

7217. Carroll NV, Miederhoff PA, Waters LW. Profitability, third-party reimbursement, and access to community pharmacies. Clinical Therapeutics. 1996;18(4):703-15. doi: https://doi.org/10.1016/S0149-2918(96)80221-9.

7218. Carroll NV, Rupp MT, Holdford DA. Analysis of costs to dispense prescriptions in independently owned, closed-door long-term care pharmacies. Journal of Managed Care Pharmacy. 2014;20(3):291-300. doi: 10.18553/jmcp.2014.20.3.291.

7219. Carroll NV, Rupp MT, Holdford DA. Analysis of Costs to Dispense Prescriptions in Independently Owned, Closed-Door Long-Term are Pharmacies. Journal of Managed Care & Specialty Pharmacy. 2014;20(3):291-300. doi: 10.18553/jmcp.2014.20.3.291. PubMed PMID: WOS:000341403100006.

7220. Carroll NV, Slattum PW, Cox FM. The cost of falls among the community-dwelling elderly. Journal of Managed Care Pharmacy. 2005;11(4):307-16.

7221. Carroll P. Medication errors: the bigger picture. RN. 2003;66(1):52-8. PubMed PMID: 106812133. Language: English. Entry Date: 20030307. Revision Date: 20150820. Publication Type: Journal Article.

7222. Carroll PR, Chen Y, Vicheth P, Webber P, Hanrahan JR. Evaluation of a vaccination training program for pharmacy graduands in Australia. Currents in Pharmacy Teaching and Learning. 2020;12(7):850-7. doi: 10.1016/j.cptl.2020.02.016.

7223. Carroll PR, Chen YH, Vicheth P, Webber P, Hanrahan JR. Evaluation of a vaccination training program for pharmacy graduands in Australia. Currents in Pharmacy Teaching and Learning. 2020;12(7):850-7. doi: 10.1016/j.cptl.2020.02.016. PubMed PMID: WOS:000541136400010.

7224. Carroll TB, Fisco AJH, Auchus RJ, Kennedy L, Findling JW. Paradoxical Results after Inadvertent Use of Cosyntropin [Adrenocorticotropin Hormone (1-24)] Rather than Acthrel (Ovine Corticotropin Releasing Hormone) during Inferior Petrosal Sinus Sampling. Endocrine Practice. 2014;20(7):646-9. doi: https://doi.org/10.4158/EP13348.OR.

7225. Carson DS, Bucci KK. Infertility in Women: An Update. Journal of the American Pharmaceutical Association (1996). 1998;38(4):480-8. doi: https://doi.org/10.1016/S1086-5802(16)30349-7.

7226. Carson-Chahhoud KV, Livingstone-Banks J, Sharrad KJ, Kopsaftis Z, Brinn MP, To-A-nan R, et al. Community pharmacy personnel interventions for smoking cessation. Cochrane Database of Systematic Reviews. 2019;2019(10). doi: 10.1002/14651858.CD003698.pub3.

7227. Carson-Stevens A, Hingston CD, Wise MP. Minimising drug errors in critically ill patients. Critical Care (London, England). 2011;15(1):401; author reply PubMed PMID: 21235830.

7228. Carson-Chahhoud KV, Livingstone-Banks J, Sharrad KJ, Kopsaftis Z, Brinn MP, To-A-Nan R, et al. Community pharmacy personnel interventions for smoking cessation. Cochrane Database of Systematic Reviews. 2019;(10). doi: 10.1002/14651858.CD003698.pub3. PubMed PMID: CD003698.

7229. Carss KJ, Deaton AM, Del Rio-Espinola A, Diogo D, Fielden M, Kulkarni DA, et al. Using human genetics to improve safety assessment of therapeutics. Nature Reviews Drug Discovery. 2023;22(2):145-62.

7230. Carswell JM, Gordon JH, Popovsky E, Hale A, Brown RS. Generic and brand-name L-thyroxine are not bioequivalent for children with severe congenital hypothyroidism. Journal of clinical endocrinology and metabolism. 2013;98(2):610-7. doi: 10.1210/jc.2012-3125. PubMed PMID: CN-00853619.

7231. Carswell K, Vincent R, Hopkins D, Rubino F, Patel A. Multicentre double-blind randomised-controlled trial of the effect of lipid-lowering treatment upon cardiovascular risk following obesity surgery in type 2 diabetes mellitus dragons' den meets shark tank (proposals for randomized controlled trials). Obesity surgery Conference: 22nd world congress of the international federation for the surgery of obesity and metabolic disorders, IFSO 2017 United kingdom. 2017;27(1 Supplement 1):481-2. doi: 10.1007/s11695-017-2774-7. PubMed PMID: CN-01462448.

7232. Carswell O, Wilton LR, Nicholls K, Thomas V, Clark SR. A 12-month audit of clozapine associated myocarditis in a South Australian Local Health Network: The importance of screening and personalised titration. Schizophrenia Research. 2023. doi: https://doi.org/10.1016/j.schres.2023.09.019.

7233. Carter BL. Implementing the new guidelines for hypertension: JNC 7, ADA, WHO-ISH. Journal of managed care pharmacy : JMCP. 2004;10(5 Suppl A):S18-25.

7234. Carter BL. Improving blood pressure control with physician/pharmacist collaboration. Vnitrni lekarstvi. 2009;55(4):389-94. PubMed PMID: CN-00702272.

7235. Carter BL, Ardery G, Dawson JD, James PA, Bergus GR, Doucette WR, et al. Physician and pharmacist collaboration to improve blood pressure control. Archives of internal medicine. 2009;169(21):1996-2002. doi: 10.1001/archinternmed.2009.358. PubMed PMID: CN-00732362.

7236. Carter BL, Barnette DJ, Chrischilles E, Mazzotti GJ, Asali ZJ. Evaluation of hypertensive patients after care provided by community pharmacists in a rural setting. Pharmacotherapy. 1997;17(6):1274-85. PubMed PMID: CN-00145942.

7237. Carter BL, Bergus GR, Dawson JD, Farris KB, Doucette WR, Chrischilles EA, et al. A cluster randomized trial to evaluate physician/pharmacist collaboration to improve blood pressure control. Journal of clinical hypertension (Greenwich, Conn). 2008;10(4):260-71. doi: 10.1111/j.1751-7176.2008.07434.x. PubMed PMID: CN-00638839.

7238. Carter BL, Chrischilles EA, Scholz D, Hayase N, Bell N. Extent of Services Provided by Pharmacists in the Iowa Medicaid Pharmaceutical Case Management Program. Journal of the American Pharmaceutical Association (1996). 2003;43(1):24-33. doi: https://doi.org/10.1331/10865800360467015.

7239. Carter BL, Clarke W, Ardery G, Weber CA, James PA, Vander Weg M, et al. A cluster-randomized effectiveness trial of a physician-pharmacist collaborative model to improve blood pressure control. Circulation Cardiovascular quality and outcomes. 2010;3(4):418-23. doi: 10.1161/CIRCOUTCOMES.109.908038. PubMed PMID: CN-00768720.

7240. Carter BL, Coffey CS, Ardery G, Ecklund D, James PA, Egan BM, et al. Implementation of a physician\pharmacist model to improve blood pressure control in a diverse population. Journal of the american society of hypertension. 2014;8(4):e126-. doi: 10.1016/j.jash.2014.03.288. PubMed PMID: CN-00993803.

7241. Carter BL, Coffey CS, Ardery G, Uribe L, Ecklund D, James P, et al. Cluster-randomized trial of a physician/pharmacist collaborative model to improve blood pressure control. Circulation Cardiovascular quality and outcomes. 2015;8(3):235-43. doi: 10.1161/CIRCOUTCOMES.114.001283. PubMed PMID: CN-01074500.

7242. Carter BL, Coffey CS, Chrischilles EA, Ardery G, Ecklund D, Gryzlak B, et al. A Cluster-Randomized Trial of a Centralized Clinical Pharmacy Cardiovascular Risk Service to Improve Guideline Adherence. Pharmacotherapy. 2015;35(7):653-62. doi: 10.1002/phar.1603. PubMed PMID: CN-01085478.

7243. Carter BL, Doucette WR, Franciscus CL, Ardery G, Kluesner KM, Chrischilles EA. Deterioration of blood pressure control after discontinuation of a physician-pharmacist collaborative intervention. Pharmacotherapy. 2010;30(3):228-35. doi: 10.1592/phco.30.3.228. PubMed PMID: CN-00750659.

7244. Carter BL, Elliott WJ. The role of pharmacists in the detection, management, and control of hypertension: a national call to action. Wiley Online Library; 2000. p. 119-22.

7245. Carter BL, Farris KB, Abramowitz PW, Weetman DB, Kaboli PJ, Dawson JD, et al. The Iowa Continuity of Care study: Background and methods. American Journal of Health-System Pharmacy. 2008;65(17):1631-42. doi: 10.2146/ajhp070600. PubMed PMID: WOS:000258733500012.

7246. Carter BL, Farris KB, Abramowitz PW, Weetman DB, Kaboli PJ, Dawson JD, et al. The Iowa Continuity of Care study: background and methods. American journal of health-system pharmacy. 2008;65(17):1631-42. doi: 10.2146/ajhp070600. PubMed PMID: CN-00650605.

7247. Carter BL, Foppe Van Mil JW. Comparative effectiveness research: Evaluating pharmacist interventions and strategies to improve medication adherence. American Journal of Hypertension. 2010;23(9):949-55. doi: 10.1038/ajh.2010.136.

7248. Carter BL, Helling DK. Ambulatory care pharmacy services: has the agenda changed? Annals of Pharmacotherapy. 2000;34(6):772-87.

7249. Carter BL, Levy B, Gryzlak B, Xu Y, Chrischilles E, Dawson J, et al. Cluster-Randomized Trial to Evaluate a Centralized Clinical Pharmacy Service in Private Family Medicine Offices. Circulation Cardiovascular quality and outcomes. 2018;11(6):e004188. doi: 10.1161/CIRCOUTCOMES.117.004188. PubMed PMID: CN-01652185.

7250. Carter BL, Levy B, Gryzlak B, Xu YH, Chrischilles E, Dawson J, et al. Cluster-Randomized Trial to Evaluate a Centralized Clinical Pharmacy Service in Private Family Medicine Offices. Circulation-Cardiovascular Quality and Outcomes. 2018;11(6). doi: 10.1161/circoutcomes.117.004188. PubMed PMID: WOS:000435564400003.

7251. Carter BL, Levy BT, Gryzlak B, Chrischilles EA, Vander Weg MW, Christensen AJ, et al. A centralized cardiovascular risk service to improve guideline adherence in private primary care offices. Contemporary clinical trials. 2015;43:25-32. doi: 10.1016/j.cct.2015.04.014. PubMed PMID: CN-01074939.

7252. Carter BL, Levy BT, Gryzlak B, Chrischilles EA, Vander Weg MW, Christensen AJ, et al. A centralized cardiovascular risk service to improve guideline adherence in private primary care offices. Contemporary Clinical Trials. 2015;43:25-32. doi: https://doi.org/10.1016/j.cct.2015.04.014.

7253. Carter BL, Lund BC, Hayase N, Chrischilles E. The extent of potential antihypertensive drug interactions in a Medicaid population. American Journal of Hypertension. 2002;15(11):953-7. doi: https://doi.org/10.1016/S0895-7061(02)03026-1.

7254. Carter BL, Lund BC, Hayase N, Chrischilles E. A longitudinal analysis of antihypertensive drug interactions in a Medicaid population. American Journal of Hypertension. 2004;17(5):421-7. doi: https://doi.org/10.1016/j.amjhyper.2004.01.007.

7255. Carter BL, Malone DC, Billups SJ, Valuck RJ, Barnette DJ, Sintek CD, et al. Interpreting the findings of the IMPROVE study. American journal of health-system pharmacy. 2001;58(14):1330-7. doi: 10.1093/ajhp/58.14.1330. PubMed PMID: CN-00456198.

7256. Carter BL, Rogers M, Daly J, Zheng S, James PA. The potency of team-based care interventions for hypertension: a meta-analysis. Archives of internal medicine. 2009;169(19):1748-55.

7257. Carter BL, Rogers M, Daly J, Zheng SM, James PA. The Potency of Team-Based Care Interventions for Hypertension A Meta-analysis. Archives of Internal Medicine. 2009;169(19):1748-+. doi: 10.1001/archinternmed.2009.316. PubMed PMID: WOS:000271163800005.

7258. Carter BL, van Mil JWF. Comparative Effectiveness Research: Evaluating Pharmacist Interventions and Strategies to Improve Medication Adherence. American Journal of Hypertension. 2010;23(9):949-55. doi: 10.1038/ajh.2010.136. PubMed PMID: WOS:000281520900012.

7259. Carter BL, Vander Weg MW, Parker CP, Goedken CC, Richardson KK, Rosenthal GE. Sustained Blood Pressure Control Following Discontinuation of a Pharmacist Intervention for Veterans. Journal of clinical hypertension. 2015;17(9):701-8. doi: 10.1111/jch.12577. PubMed PMID: CN-01097594.

7260. Carter BL, Zillich AJ, Elliott WJ. How pharmacists can assist physicians with controlling blood pressure. The Journal of Clinical Hypertension. 2003;5(1):31-7.

7261. Carter G, Yu Z, Aryana Bryan M, Brown JL, Winhusen T, Cochran G. Validation of the tobacco, alcohol, prescription medication, and other substance use (TAPS) tool with the WHO alcohol, smoking, and substance Involvement screening test (ASSIST). Addictive Behaviors. 2022;126:107178. doi: https://doi.org/10.1016/j.addbeh.2021.107178.

7262. Carter G, Yu ZJ, Bryan MA, Brown JL, Winhusen T, Cochran G. Validation of the tobacco, alcohol, prescription medication, and other substance use (TAPS) tool with the WHO alcohol, smoking, and substance Involvement screening test (ASSIST). Addictive Behaviors. 2022;126. doi: 10.1016/j.addbeh.2021.107178. PubMed PMID: WOS:000822561500009.

7263. Carter GT, Duong V, Ho S, Ngo KC, Greer CL, Weeks DL. Side Effects of Commonly Prescribed Analgesic Medications. Physical Medicine and Rehabilitation Clinics of North America. 2014;25(2):457-70. doi: https://doi.org/10.1016/j.pmr.2014.01.007.

7264. Carter JA, Huang XY, Jamil K, Corman S, Ektare V, Niewoehner J. Cost-effectiveness of terlipressin for hepatorenal syndrome: the United States hospital perspective. Journal of Medical Economics. 2023;26(1):1342-8. doi: 10.1080/13696998.2023.2260693. PubMed PMID: WOS:001101495500001.

7265. Carter KL, Bonanni S. Role of the pharmacist in refugee health. American Journal of Health-System Pharmacy. 2019;76(6):403-6. PubMed PMID: 31361836.

7266. Carter L, Bailey Wheeler J, Hart T, Chrishon K, Anderson D. Assessing patients' awareness of the benefits of medication therapy management in a predominantly african american community. Journal of the

American Pharmacists Association. 2012;52(2):238-9. PubMed PMID: 71322910.

7267. Carter M. Prevention of Glucocorticoid-Induced Osteoporosis: Clinical audit to evaluate the implementation of National Osteoporosis Guideline Group 2017 guidelines in a primary care setting. Journal of Clinical Densitometry. 2019;22(1):25-30. doi: https://doi.org/10.1016/j.jocd.2018.03.009.

7268. Carter M, Abutheraa N, Ivers N, Grimshaw J, Chapman S, Rogers P, et al. Audit and feedback interventions involving pharmacists to influence prescribing behaviour in general practice: a systematic review and meta-analysis. Family Practice. 2023;40(5-6):615-28.

7269. Carter M, Abutheraa N, Ivers N, Grimshaw J, Chapman S, Rogers P, et al. Audit and feedback interventions involving pharmacists to influence prescribing behaviour in general practice: a systematic review and meta-analysis. Family Practice. 2023:cmac150.

7270. Carter MC, Maric I, Brittain EH, Bai Y, Lumbard K, Bolan H, et al. A randomized double-blind, placebo-controlled study of omalizumab for idiopathic anaphylaxis. Journal of allergy and clinical immunology. 2021;147(3):1004-10.e2. doi: 10.1016/j.jaci.2020.11.005. PubMed PMID: CN-02201290.

7271. Carter MK, Allin DM, Scott LA, Grauer D. Pharmacist-acquired medication histories in a university hospital emergency department. American Journal of Health-System Pharmacy. 2006;63(24):2500-3.

7272. Carter RMN, van Rensburg BJJ, Joubert G. Control of rheumatoid arthritis with conventional disease-modifying antirheumatic drugs in a tertiary hospital in central South Africa. Samj South African Medical Journal. 2020;110(1):27-31. doi: 10.7196/SAMJ.2020.v110i1.14099. PubMed PMID: WOS:000503169600008.

7273. Carter S. Medicines management: Opportunities for pharmacists in new GMS contract. Pharmaceutical Journal. 2004;272(7291):350-1.

7274. Carter S, Ng R, El-Den S, Schneider C. Low perceived service quality in community pharmacy is associated with poor medication adherence. Patient Education and Counseling. 2021;104(2):387-94. doi: https://doi.org/10.1016/j.pec.2020.07.009.

7275. Carter SR, Ahmed AM, Schneider CR. The role of perceived service quality and price competitiveness on consumer patronage of and intentions towards community pharmacies. Research in Social and Administrative Pharmacy. 2023;19(5):717-27. doi: 10.1016/j.sapharm.2023.02.002.

7276. Carter SR, Moles R, White L, Chen TF. Patients' willingness to use a pharmacist-provided medication management service: The influence of outcome expectancies and communication efficacy. Research in Social and Administrative Pharmacy. 2012;8(6):487-98. doi: https://doi.org/10.1016/j.sapharm.2012.01.002.

7277. Carter SR, Moles R, White L, Chen TF. Exploring patients' motivation to participate in Australia's Home Medicines Review program. International Journal of Clinical Pharmacy. 2012;34(4):658-66. PubMed PMID: 22674179.

7278. Carter SR, Moles R, White L, Chen TF. The impact of patients' perceptions of the listening skills of the pharmacist on their willingness to re-use Home Medicines Reviews: a structural equation model. Research In Social & Administrative Pharmacy. 2015;11(2):163-75. PubMed PMID: 25681299.

7279. Carter SR, Moles R, White L, Chen TF. The willingness of informal caregivers to assist their care-recipient to use Home Medicines Review. Health Expectations. 2016;19(3):527-42. doi: 10.1111/hex.12092.

7280. Carter SR, Moles RJ, White L, Chen TF. Consumers' willingness to use a medication management

service: The effect of medication-related worry and the social influence of the general practitioner. Research in Social and Administrative Pharmacy. 2013;9(4):431-45. doi: https://doi.org/10.1016/j.sapharm.2012.07.001.

7281. Carter SR, Ng R, El-Den S, Schneider CR. A patient-reported experience measure for community pharmacy including development of a short-form: The perceived service quality scale. Research in Social and Administrative Pharmacy. 2022;18(8):3369-78. doi: 10.1016/j.sapharm.2021.11.011.

7282. Carthy P, Harvey I, Brawn R, Watkins C. A study of factors associated with cost and variation in prescribing among GPs. Family practice. 2000;17(1):36-41.

7283. Cartier LJ, Robin A, St-Caeur S, Lagace M, Canales DD, Douville P. Impact of the 2016 Canadian Lipid Guidelines on Daily Practice at a Community Hospital. Canadian Journal of Diabetes. 2022;46(1):75-80. doi: 10.1016/j.jcjd.2021.06.002. PubMed PMID: WOS:000754294900012.

7284. Cartier LJ, Robin A, St-Cœur S, Lagacé M, Canales DD, Douville P. Impact of the 2016 Canadian Lipid Guidelines on Daily Practice at a Community Hospital. Canadian Journal of Diabetes. 2022;46(1):75-80. doi: 10.1016/j.jcjd.2021.06.002.

7285. Cartmill RS, Yang DY, Fernandes-Taylor S, Kohler JE. National variation in opioid prescribing after pediatric umbilical hernia repair. Surgery. 2019;165(4):838-42. doi: 10.1016/j.surg.2018.10.029. PubMed PMID: WOS:000462692700024.

7286. Cartwright AF, Velarde M, Beksinska M, Smit J, Kasaro M, Tang JH, et al. Perspectives on sexual and reproductive health self-care among women, healthcare providers, and other key informants: a mixed-methods study in South Africa and Zambia. Reproductive Health. 2023;20(1). doi: 10.1186/s12978-023-01596-x. PubMed PMID: WOS:000978531100001.

7287. Caruba T, Boussadi A, Lenain E, Korb-Savoldelli V, Gillaizeau F, Durieux P, et al. A simulation study of the interception of prescribing errors by clinical pharmacists in an acute hospital setting. Journal of Evaluation in Clinical Practice. 2015;21(4):673-80. doi: 10.1111/jep.12363. PubMed PMID: 109821011. Language: English. Entry Date: 20150731. Revision Date: 20200708. Publication Type: Journal Article.

7288. Caruba T, Colombet I, Gillaizeau F, Bruni V, Korb V, Prognon P, et al. Chronology of prescribing error during the hospital stay and prediction of pharmacist's alerts overriding: a prospective analysis. BMC Health Services Research. 2010;10:13. PubMed PMID: 20067620.

7289. Caruba T, Colombet I, Gillaizeau F, Bruni V, Korb V, Prognon P, et al. Chronology of prescribing error during the hospital stay and prediction of pharmacist's alerts overriding: a prospective analysis. BMC Health Services Research. 2010;10:13-. doi: 10.1186/1472-6963-10-13. PubMed PMID: 105121414. Language: English. Entry Date: 20120511. Revision Date: 20211027. Publication Type: journal article.

7290. Caruso MC, Gittelman MA, Widecan ML, Luria JW. Pediatric emergency department discharge prescriptions requiring pharmacy clarification. Pediatric Emergency Care. 2015;31(6):403-8. PubMed PMID: 604861452.

7291. Carusone SC, Loeb M, Lohfeld L. A clinical pathway for treating pneumonia in the nursing home: part I: the nursing perspective. Journal of the American Medical Directors Association. 2006;7(5):271-8.

7292. Carvajal A, Sáinz M, Velasco V, García Ortega P, Treceño C, Martín Arias LH, et al. Emergency contraceptive pill safety profile. Comparison of the results of a follow-up study to those coming from spontaneous reporting. Pharmacoepidemiology and Drug Safety. 2015;24(1):93-7. doi: 10.1002/pds.3725.

7293. Carvajal A, Sainz M, Velasco V, Ortega PG, Treceno C, Arias LHM, et al. Emergency contraceptive pill safety profile. Comparison of the results of a follow-up study to those coming from spontaneous reporting. Pharmacoepidemiology and Drug Safety. 2015;24(1):93-7. doi: 10.1002/pds.3725. PubMed PMID: WOS:000347710100013.

7294. Carvajal FA, Martinez MG, Santamaria EC, Hernandez BC, Orbis IC, Casanova NM, et al. Adaptation of oral medication in people institutionalized in nursing homes for whom medication is crushed: the ADECUA Study. Farmacia hospitalaria. 2016;40(6):514-28. doi: 10.7399/fh.2016.40.6.10467. PubMed PMID: CN-01245627.

7295. Carvajal MJ, Popovici I. Gender, age, and pharmacists' job satisfaction. Pharmacy Practice (Granada). 2018;16(4).

7296. Carvalhana V, Flak E. Role of the pharmacist on a multidisciplinary psychiatry team: Impact on medication adherence in a community setting. Journal of Pharmacy Technology. 2009;25(3):155-8. doi: 10.1177/875512250902500302.

7297. Carvalho AP, Silva AB, Lebani BR, Pinto ER, Felipe MR, Skaf M, et al. Clinical and Urodynamic results of the Argus T® sling in moderate and severe male stress urinary incontinence after radical prostatectomy-a 5 year prospective study. International Braz J Urol. 2023;49(4):452-61. doi: 10.1590/s1677-5538.Ibju.2023.0003. PubMed PMID: WOS:001014841300007.

7298. Carvalho De Camargo MFF, Santos FVDEB, Seber A, Rabello Chiattone R, Bigni R, Maiolino A, et al. Implementation of Clinical Pathways for CAR-T Cell Therapy in a Brazilian Private Healthcare Organization. Blood. 2023;142:7267. doi: https://doi.org/10.1182/blood-2023-185011.

7299. Carvalho ELF. Métodos de Avaliação da Adesão à Terapêutica Medicamentosa: Experiência Profissionalizante na Vertente de Farmácia Comunitária, Hospitalar e Investigação [M.Sc.]. Portugal: Universidade da Beira Interior (Portugal); 2015.

7300. Carvalho IV, de Sousa VM, Visacri MB, Quintanilha JCF, de Souza CM, Ambrósio RFL, et al. Adverse Drug Event-Related Admissions to a Pediatric Emergency Unit. Pediatric Emergency Care. 2021;37(4):e152-e8. doi: 10.1097/PEC.0000000000001582. PubMed PMID: 149761997. Language: English. Entry Date: 20211118. Revision Date: 20211215. Publication Type: journal article.

7301. Carvalho JM. Caracterização do Perfil de Consumo de Benzodiazepinas na Cova da Beira Experiência Profissionalizante na Vertente de Farmácia Comunitária e Investigação [Master's]. Portugal: Universidade da Beira Interior (Portugal); 2012.

7302. Carvalho M, Almeida IF. The Role of Pharmaceutical Compounding in Promoting Medication Adherence. Pharmaceuticals. 2022;15(9). doi: 10.3390/ph15091091. PubMed PMID: WOS:000856629300001.

7303. Carvalho MSDd. New Heterocyclic Bioactive Fluorescent Compounds: Spectroscopic Studies of Dna Interactions and Encapsulation in Nanoliposomes [Ph.D.]. Portugal: Universidade do Minho (Portugal); 2013.

7304. Carvalho S, Nagavi BG. Impact of community pharmacy based patient education on the quality of life of hypertensive patients. Indian Journal of Pharmaceutical Education and Research. 2007;41(2):164-9. PubMed PMID: WOS:000259222200016.

7305. Carvalho SG, Silvestre ALP, Martins dos Santos A, Fonseca-Santos B, Rodrigues WD, Palmira Daflon Gremião M, et al. Polymeric-based drug delivery systems for veterinary use: State of the art. International

Journal of Pharmaceutics. 2021;604:120756. doi: https://doi.org/10.1016/j.ijpharm.2021.120756.

7306. Carvalho SMM. Avaliação dos Comportamentos de Risco em Viajantes com Destino a Angolal: Experiência Profissionalizante na Vertente de Farmácia Comunitária e Investigação [M.Sc.]. Portugal: Universidade da Beira Interior (Portugal); 2015.

7307. Carvalho VM, Carmo JD, dos Santos LMG, de Almeida FG, Rocha ED, Vieira ACD, et al. Pharmaceutical Evaluation of Medical <i>Cannabis</i> Extracts Prepared by Artisanal and Laboratory Techniques. Revista Brasileira De Farmacognosia-Brazilian Journal of Pharmacognosy. 2023;33(4):724-35. doi: 10.1007/s43450-023-00412-8. PubMed PMID: WOS:001000491700001.

7308. Carvalho VM, dos Santos Carmo J, dos Santos LMG, de Almeida FG, Rocha ED, de Macêdo Vieira AC, et al. Pharmaceutical Evaluation of Medical Cannabis Extracts Prepared by Artisanal and Laboratory Techniques. Revista Brasileira de Farmacognosia. 2023;33(4):724-35. doi: 10.1007/s43450-023-00412-8.

7309. Carver H, Falzon D, Masterton W, Wallace B, Aston EV, Measham F, et al. 'I t's not going to be a one size fits all': a qualitative exploration of the potential utility of three drug checking service models in Scotland. Harm Reduction Journal. 2023;20(1):94.

7310. Carvunis A-R, Rolland T, Wapinski I, Calderwood MA, Yildirim MA, Simonis N, et al. Proto-genes and de novo gene birth. Nature. 2012;487(7407):370-4.

7311. Casale TB, Blaiss MS, Gelfand E, Gilmore T, Harvey PD, Hindmarch I, et al. First do no harm: Managing antihistamine impairment in patients with allergic rhinitis. Journal of Allergy and Clinical Immunology. 2003;111(5):S835-S42. doi: https://doi.org/10.1067/mai.2003.1550.

7312. Casares Li R, Hussein T, Bancsi A, Grindrod K, Burns C. Reason for Use: An Opportunity to Improve Patient Safety. Studies in Health Technology & Informatics. 2019;257:47-52. doi: 10.3233/978-1-61499-951-5-47. PubMed PMID: 134901571. Language: English. Entry Date: 20190227. Revision Date: 20201217. Publication Type: Article.

7313. Casas-Muñoz A, Carranza-Neira J, Intebi I, Lidchi V, Eisenstein E, Greenbaum J. Addressing Child Sexual Abuse: A Call to Action for Providers in Latin America. Revista Panamericana de Salud Publica/Pan American Journal of Public Health. 2023;47. doi: 10.26633/RPSP.2023.54.

7314. Casaurang P. Requests for assistance in pharmacies open to the public. Annales Pharmaceutiques Francaises. 1998;56(4):187-90.

7315. Cascone AE, Sullivan J, Ackerbauer K, Patel S, Lindale DK, Tatro H, et al. Pharmacist-Initiated De-Prescribing Efforts Reduce Inappropriate Continuation of Acid-Suppression Therapy Initiated in the ICU. American Journal of Medicine. 2023;136(2):186-92. PubMed PMID: 36170933.

7316. Cascone AE, Sullivan J, Ackerbauer K, Patel S, Lindale DK, Tatro H, et al. Pharmacist-Initiated De-Prescribing Efforts Reduce Inappropriate Continuation of Acid-Suppression Therapy Initiated in the ICU. The American Journal of Medicine. 2023;136(2):186-92. doi: https://doi.org/10.1016/j.amjmed.2022.09.003.

7317. Case LL, Paparella S. Safety Benefits of a Clinical Pharmacist in the Emergency Department. Journal of Emergency Nursing. 2007;33(6):564-6. doi: https://doi.org/10.1016/j.jen.2007.06.008.

7318. Casebeer L, Huber C, Bennett N, Shillman R, Abdolrasulnia M, Salinas GD, et al. Improving the physician-patient cardiovascular risk dialogue to improve statin adherence. BMC family practice. 2009;10:48. doi: 10.1186/1471-2296-10-48. PubMed PMID: CN-00705497.

7319. Casey MM, Klingner J, Moscovice I. Pharmacy services in rural areas: Is the problem geographic access or financial access? Journal of Rural Health. 2002;18(3):467-77. doi: 10.1111/j.1748-0361.2002.tb00911.x.

7320. Casey MM, Moscovice I. Quality improvement strategies and best practices in critical access hospitals. The Journal of Rural Health. 2004;20(4):327-34.

7321. Casey MM, Sorensen TD, Elias W, Knudson A, Gregg W. Current practices and state regulations regarding telepharmacy in rural hospitals. American Journal of Health-System Pharmacy. 2010;67(13):1085-92. doi: 10.2146/ajhp090531. PubMed PMID: 105035780. Language: English. Entry Date: 20100702. Revision Date: 20200708. Publication Type: Journal Article.

7322. Casey MM, Wakefield M, Coburn AF, Moscovice IS, Loux S. Prioritizing patient safety interventions in small and rural hospitals. Joint Commission Journal on Quality & Patient Safety. 2006;32(12):693-702. PubMed PMID: 106132130. Language: English. Entry Date: 20070810. Revision Date: 20150711. Publication Type: Journal Article.

7323. Casey T, Johnson C, Love D. Adult attention deficit hyperactivity disorder clinic: An interprofessional collaboration. Journal of the American Pharmacists Association: JAPhA. 2020;60(5S):S113-S7. PubMed PMID: 32616446.

7324. Cashen MS. Literacy and adherence to antiretroviral drug therapy in HIV -positive men and women [Ph.D.]. United States -- California: University of California, San Francisco; 2004.

7325. Cashman H, Hamad N, Moore J, Milliken S, Lavee O, Withers B, et al. An Electronic Health Record Facilitates a Safer and More Efficient Rural Outreach Haematology/Oncology Service. Blood. 2019;134:4709. doi: https://doi.org/10.1182/blood-2019-127796.

7326. Caso-González A, Núñez-Rodríguez J, Nebot-Villacampa M-J, González-Pérez Y, Marín-Gorricho R, Leralta-González C, et al. Clinical experience with orphan drugs for rare metabolic diseases. Anales de Pediatría (English Edition). 2022;96(1):8-16. doi: https://doi.org/10.1016/j.anpede.2020.09.014.

7327. Caso-González A, Núñez-Rodríguez J, Nebot-Villacampa M-J, González-Pérez Y, Marín-Gorricho R, Leralta-González C, et al. Experiencia clínica con medicamentos huérfanos para enfermedades raras metabólicas. Anales de Pediatría. 2022;96(1):8-16. doi: https://doi.org/10.1016/j.anpedi.2020.09.019.

7328. Casper AS, Beachy S, Quittner A, Riekert KA. Cystic fibrosis care teams' perceptions of a systematic process to identity barriers affecting treatment adherence. American journal of respiratory and critical care medicine Conference: american thoracic society international conference, ATS 2017 United states. 2017;195(no pagination). doi: 10.1164/ajrccmconference.2017.C37. PubMed PMID: CN-01409184.

7329. Casper EA, El Wakeel LM, Saleh MA, El-Hamamsy MH. Management of pharmacotherapy-related problems in acute coronary syndrome: role of clinical pharmacist in cardiac rehabilitation unit. Basic & clinical pharmacology & toxicology. 2019;125(1):44-53. doi: 10.1111/bcpt.13210. PubMed PMID: CN-01937304.

7330. Caspi A, Rozenfeld V, Kleyman J. Prevention of medication errors in the hospital setting: the role of pharmacy students. P&T: A Peer-Reviewed Journal for Managed Care & Formulary Management. 2005;30(3):183-6. PubMed PMID: 106460610. Language: English. Entry Date: 20060623. Revision Date: 20150711. Publication Type: Journal Article.

7331. Cass Y, Setton I. 25 years of safe handling of cytotoxics (antineoplastics) in Israel. Journal of Oncology Pharmacy Practice. 2006;12(2):83-90. PubMed PMID: 16984746.

7332. Cassam J, Shah C, Lewis P, Al-Tahan S, Pickard K. Development of a community nursing drug chart. Nursing Management. 2014;21(2):22-5. doi: 10.7748/nm2014.04.21.2.22.e1215.

7333. Cassano AT, Cassano AT. Parenteral nutrition training for the pharmacy team: focus on pharmacy technicians. JPEN Journal of Parenteral & Enteral Nutrition. 2012;36(2):48S-50S. doi: 10.1177/0148607111434774. PubMed PMID: 104533068. Language: English. Entry Date: 20120817. Revision Date: 20170411. Publication Type: journal article. Supplement Title: Mar2012 Supplement. Commentary: Gumpper KF, Gumpper Karl F. Drug shortages and training. (JPEN J PARENTER ENTERAL NUTR) Mar2012 Supplement.

7334. Cassar Flores A, Marshall S, Cordina M. Use of the Delphi technique to determine safety features to be included in a neonatal and paediatric prescription chart. International Journal of Clinical Pharmacy. 2014;36(6):1179-89. doi: 10.1007/s11096-014-0014-y. PubMed PMID: 109763867. Language: English. Entry Date: 20150605. Revision Date: 20211030. Publication Type: journal article.

7335. Cassar SJ, Shoemake C, Azzopardi LM. Creation and Evaluation of a 2D/3D Molecular Database for Drugs Used to Target the Respiratory System. Thorax. 2013;68(Suppl 3):A110 [P77] doi: 10.1136/thoraxjnl-2013-204457.227. PubMed PMID: CN-00989834.

7336. Cassarino M, Robinson K, Quinn R, Naddy B, O'Regan A, Ryan D, et al. Effectiveness of early assessment and intervention by interdisciplinary teams including health and social care professionals in the emergency department: protocol for a systematic review. BMJ Open. 2018;8(7):e023464. PubMed PMID: 30012796.

7337. Cassell D. Pharmacists of the year - Part 1: Meet some outstanding R.Ph.s who are making a difference in community pharmacy. Drug Topics. 2000;144(15):22-8.

7338. Casset A, Meunier-Spitz M, Rebotier P, Lefevre H, Barth C, Heitz C, et al. Asthma management and inhalation techniques among community pharmacists in 2009: a comparison with the 1999 survey. Journal of Asthma. 2014;51(9):964-73. doi: 10.3109/02770903.2014.936446. PubMed PMID: WOS:000344080200011.

7339. Cassiani SH. [Medication errors: prevention strategies]. Revista Brasileira de Enfermagem. 2000;53(3):424-30. PubMed PMID: 12138424.

7340. Cassiani SHB. Errors in medication: preventive strategies. Revista Brasileira de Enfermagem. 2000;53(3):424-30. PubMed PMID: 107049360. Language: Portuguese. Entry Date: 20010907. Revision Date: 20150820. Publication Type: Journal Article. Journal Subset: Double Blind Peer Reviewed.

7341. Cassidy A. Differentiated Service Delivery for Males, Youth, and Stable Patients in a Large HIV Treatment Program in South Africa [Ph.D.]. United States -- Massachusetts: Boston University; 2021.

7342. Cassidy CE, Boulos L, McConnell E, Barber B, Delahunty-Pike A, Bishop A, et al. E-prescribing and medication safety in community settings: A rapid scoping review. Exploratory Research in Clinical and Social Pharmacy. 2023;12:100365. doi: https://dx.doi.org/10.1016/j.rcsop.2023.100365. PubMed PMID: 2028586530.

7343. Cassidy S, Mawdsley A, Langran C, Hughes L, Willis SC. A Large-scale Multicenter Study of Academic Resilience and Well-being in Pharmacy Education. American Journal of Pharmaceutical Education. 2023;87(2):ajpe8998. doi: https://doi.org/10.5688/ajpe8998.

7344. Cassie H, Duncan EM, Gibb EA, Power A, Young L, Newlands R, et al. Qualitative study exploring the key determinants of information gathering to inform the management of over-the-counter (OTC) consultations in community pharmacies. BMJ Open. 2019;9(8). doi: 10.1136/bmjopen-2019-029937.

7345. Cassim L, Dludlu D. Impact of a performance management system in a South African retail pharmacy on the provision of pharmaceutical care to patients. SA Pharmaceutical Journal. 2012;79(4):51-8.

7346. Cassinelli EH, McKinley MC, Kent L, Eastwood K-A, Schoenaker D, McGowan L. Preconception health and care policies and guidelines in the UK and Ireland: a scoping review. The Lancet. 2022;400:S61. doi: https://doi.org/10.1016/S0140-6736(22)02271-1.

7347. Cassinelli EH, McKinley MC, Kent L, Eastwood KA, Schoenaker D, McGowan L. Preconception health and care policies and guidelines in the UK and Ireland: a scoping review. Lancet (London, England). 2022;400:S61. doi: 10.1016/S0140-6736(22)02271-1.

7348. Castaldo S, Grosso M, Mallarini E, Rindone M. The missing path to gain customers loyalty in pharmacy retail: The role of the store in developing satisfaction and trust. Research in Social & Administrative Pharmacy. 2016;12(5):699-712. doi: 10.1016/j.sapharm.2015.10.001. PubMed PMID: WOS:000382271000003.

7349. Castaneda C, Nalley K, Mannion C, Bhattacharyya P, Blake P, Pecora A, et al. Clinical decision support systems for improving diagnostic accuracy and achieving precision medicine. Journal of clinical bioinformatics. 2015;5(1):1-16.

7350. Castaneda S, Melendez-Lopez S, Garcia E, Cruz H, Sanchez-Palacio J, De la Cruz H. The Role of the Pharmacist in the Treatment of Patients with Infantile Hemangioma Using Propranolol. Advances in Therapy. 2016;33(10):1831-9. doi: 10.1007/s12325-016-0391-9. PubMed PMID: 118672674. Language: English. Entry Date: 20170903. Revision Date: 20180926. Publication Type: journal article. Journal Subset: Biomedical.

7351. Castaneda S, Melendez-Lopez S, Garcia E, De la Cruz H, Sanchez-Palacio J. The Role of the Pharmacist in the Treatment of Patients with Infantile Hemangioma Using Propranolol. Advances in Therapy. 2016;33(10):1831-9. PubMed PMID: 27461120.

7352. Casteel C, Blalock SJ, Ferreri S, Roth MT, Demby KB. Implementation of a community pharmacy-based falls prevention program. American journal of geriatric pharmacotherapy. 2011;9(5):310-9.e2. doi: 10.1016/j.amjopharm.2011.08.002. PubMed PMID: CN-00895106.

7353. Casteel C, Blalock SJ, Ferreri S, Roth MT, Demby KB. Implementation of a Community Pharmacy–Based Falls Prevention Program. The American Journal of Geriatric Pharmacotherapy. 2011;9(5):310-9.e2. doi: https://doi.org/10.1016/j.amjopharm.2011.08.002.

7354. Castejón AM, Calderón JL, Perez A, Millar C, McLaughlin-Middlekauf J, Sangasubana N, et al. A community - Based pilot study of a diabetes pharmacist intervention in latinos: Impact on weight and hemoglobin A1c. Journal of Health Care for the Poor and Underserved. 2013;24(4 SUPPL.):48-60. doi: 10.1353/hpu.2014.0003.

7355. Castejón AM, Calderón JL, Perez A, Millar C, McLaughlin-Middlekauff J, Sangasubana N, et al. A community-based pilot study of a diabetes pharmacist intervention in Latinos: impact on weight and hemoglobin A1c. Journal of health care for the poor and underserved. 2013;24(4 Suppl):48-60. doi: 10.1353/hpu.2014.0003. PubMed PMID: CN-01001953.

7356. Castel C, Blanchardière A, Michon J, Cattoir V, Saint-Lorant G, de La Blanchardière A. Study on daptomycin use and implementation of an antimicrobial stewardship program. International Journal of Clinical Pharmacy. 2016;38(2):421-8. doi: 10.1007/s11096-016-0271-z. PubMed PMID: 114513566. Language: English. Entry Date: 20170113. Revision Date: 20191029. Publication Type: journal article.

7357. Castel H, Bonneh DY, Sherf M, Liel Y. Awareness of osteoporosis and compliance with management guidelines in patients with newly diagnosed low-impact fractures. Osteoporosis International. 2001;12(7):559-64. doi: 10.1007/s001980170077.

7358. Castelino R, Toh LS. Management of Renal Disorders and the Pharmacist's Role: Acute Kidney Injury. In: Babar Z-U-D, editor. Encyclopedia of Pharmacy Practice and Clinical Pharmacy. Oxford: Elsevier; 2019. p. 408-21.

7359. Castelino R, Toh LS. Management of Renal Disorders and the Pharmacist's Role: Chronic Kidney Disease. In: Babar Z-U-D, editor. Encyclopedia of Pharmacy Practice and Clinical Pharmacy. Oxford: Elsevier; 2019. p. 422-36.

7360. Castelino RL, Bajorek BV, Chen TF. Targeting suboptimal prescribing in the elderly: a review of the impact of pharmacy services. Annals of Pharmacotherapy. 2009;43(6):1096-106.

7361. Castelino RL, Bajorek BV, Chen TF. Retrospective evaluation of home medicines review by pharmacists in older australian patients using the medication appropriateness index. Annals of Pharmacotherapy. 2010;44(12):1922-9. doi: 10.1345/aph.1P373.

7362. Castelino RL, Bajorek BV, Chen TF. Are interventions recommended by pharmacists during Home Medicines Review evidence-based? Journal of Evaluation in Clinical Practice. 2011;17(1):104-10. PubMed PMID: 20722881.

7363. Castelino RL, Hilmer SN, Bajorek BV, Nishtala P, Chen TF. Drug Burden Index and potentially inappropriate medications in community-dwelling older people: the impact of Home Medicines Review. Drugs & Aging. 2010;27(2):135-48. doi: 10.2165/11531560-000000000-00000. PubMed PMID: 105120719. Language: English. Entry Date: 20100409. Revision Date: 20200708. Publication Type: Journal Article.

7364. Castelino RL, Sathvik BS, Parthasarathi G, Gurudev KC, Shetty MS, Narahari MG. Prevalence of medication-related problems among patients with renal compromise in an Indian hospital. Journal of Clinical Pharmacy and Therapeutics. 2011;36(4):481-7. doi: 10.1111/j.1365-2710.2011.01266.x. PubMed PMID: WOS:000292464300004.

7365. Castellana NE, Payne SH, Shen Z, Stanke M, Bafna V, Briggs SP. Discovery and revision of Arabidopsis genes by proteogenomics. Proceedings of the national academy of sciences. 2008;105(52):21034-8.

7366. Castellano JM, Sanz G, Peñalvo JL, Bansilal S, Fernández-Ortiz A, Alvarez L, et al. A polypill strategy to improve adherence: results from the FOCUS project. Journal of the American College of Cardiology. 2014;64(20):2071-82.

7367. Castelli G, Bacci JL, Dombrowski SK, Osborne M, Difilippo A, Klatt PM, et al. Pharmacist-Delivered Comprehensive Medication Management Within Family Medicine Practices An Evaluation of the SCRIPT Project. Family Medicine. 2018;50(8):605-12. PubMed PMID: 30215820.

7368. Castelli M, Saint C, Crannage AJ, Stacy ZA, Pitlick JM. Effect of a rivaroxaban patient assistance kit (R-PAK) for patients discharged with rivaroxaban: a randomized controlled trial. Pharmacotherapy. 2016;36(12):e253-. doi: 10.1002/phar.1877. PubMed PMID: CN-01362134.

7369. Castelli MR, Saint CA, Nuziale BT, Stern GM, Stacy ZA, Crannage AJ, et al. Effect of a rivaroxaban patient assistance kit (r-pak) for patients discharged with rivaroxaban: a randomized controlled trial. Hospital pharmacy. 2017;52(7):496-501. doi: 10.1177/0018578717721105. PubMed PMID: CN-01432239.

7370. Castello FM, Castello SM, Jornet JP, Manglano MLF, Insa MDI. POTENTIALLY INAPPROPRIATE MEDICATION IN OLDER PATIENTS WHO ATTEND TO A COMMUNITY PHARMACY. Basic & Clinical Pharmacology & Toxicology. 2015;117:39-. PubMed PMID: WOS:000365717900123.

7371. Castello FM, Castello SM, Moncho MJR, Mut AS, Ballester PM. Collaboration of a health and social services center and community pharmacies in reviewing medications and processing personalized dosing systems. Farmaceuticos Comunitarios. 2018;10(2):15-20. doi: 10.5672/FC.2173-9218.(2018/Vol10).002.03. PubMed PMID: WOS:000437662000003.

7372. Castello S, Darker C, Vance J, Buckley E, Reynolds C, Buggy A, et al. THE WE CAN QUIT2 SMOKING CESSATION TRIAL KNOWLEDGE EXCHANGE AND DISSEMINATION: PRIORITIES FROM A COMMUNITY PERSPECTIVE, AND RECOMMENDATIONS FOR FUTURE RESEARCH AND POLICY. Journal of epidemiology and community health. 2021;75:A62-A3. doi: 10.1136/jech-2021-SSMabstracts.133. PubMed PMID: CN-02462855.

7373. Castelo M, Li J, Taddio A, Lepore N, Lim R, Reider M, et al. A randomized controlled trial of 2% lidocaine gel compared to current standard of care in infants undergoing urinary catheterization. Annals of emergency medicine. 2014;64(4):S105-. doi: 10.1016/j.annemergmed.2014.07.324. PubMed PMID: CN-01023425.

7374. Casten R, Rovner B, Chang AM, Hollander JE, Kelley M, Leiby B, et al. A randomized clinical trial of a collaborative home-based diabetes intervention to reduce emergency department visits and hospitalizations in black individuals with diabetes. Contemporary clinical trials. 2020;95:106069. doi: 10.1016/j.cct.2020.106069. PubMed PMID: CN-02139435.

7375. Castiglia M, Smego RA. The Global Problem of Antimicrobial Resistance: Pharmacists can help counter this public health threat by becoming involved in the appropriate selection and use of antimicrobial agents. Journal of the American Pharmaceutical Association (1996). 1997;37(4):383-7. doi: https://doi.org/10.1016/S1086-5802(16)30236-4.

7376. Castillo A, Brahim A, Hardwick M, MacKrell M, Fernandez H, Oquendo A. Transformative Leadership: A Model for Building a Comprehensive Cellular Therapy Program Partnership. Biology of Blood and Marrow Transplantation. 2020;26(3, Supplement):S367. doi: https://doi.org/10.1016/j.bbmt.2019.12.332.

7377. Castillo AM, Natkowski J, Rubal-Peace G. Assessing adherence to current national guidelines for appropriate albumin use at an academic medical center. Pharmacy Practice (1886-3655). 2018;16(2):1-4. doi: 10.18549/PharmPract.2018.02.1190. PubMed PMID: 131163473. Language: English. Entry Date: 20180821. Revision Date: 20180824. Publication Type: Article.

7378. Castillo D, Burgos F, Guayta R, Giner J, Lozano P, Estrada M, et al. Airflow obstruction case finding in community-pharmacies: A novel strategy to reduce COPD underdiagnosis. Respiratory Medicine. 2015;109(4):475-82. doi: https://doi.org/10.1016/j.rmed.2015.02.009.

7379. Castillo D, Guayta R, Giner J, Burgos F, Capdevila C, Soriano JB, et al. COPD case finding by spirometry in high-risk customers of urban community pharmacies: A pilot study. Respiratory Medicine. 2009;103(6):839-45. doi: https://doi.org/10.1016/j.rmed.2008.12.022.

7380. Castillo J, Campbell MJ, Sokn E, Spinner M, Lam SW, Meldon S, et al. Discharge prescription optimization by emergency medicine pharmacists in an academic emergency department in the United States. International Journal of Clinical Pharmacy. 2021;43(3):673-80. PubMed PMID: 33124677.

7381. Castillo S, Begley K, Malesker M, Steinshouer C. Sodium oxybate: A primer for pharmacists in the treatment off narcolepsy. Consultant Pharmacist. 2014;29(8):547-54. doi: 10.4140/TCP.n.2014.547.

7382. Castillo-Carniglia A, González-Santa Cruz A, Cerdá M, Delcher C, Shev AB, Wintemute GJ, et al. Changes in opioid prescribing after implementation of mandatory registration and proactive reports within California's prescription drug monitoring program. Drug and Alcohol Dependence. 2021;218:108405. doi: https://doi.org/10.1016/j.drugalcdep.2020.108405.

7383. Castillo-García ML, Martínez-Raga J, López-Castellano AC, Castillo-García E. Health education in the community pharmacy: Controlled study in the province of Castellon. Ars Pharmaceutica. 2011;52(4):5-11.

7384. Castle L, Franzblau-Isaac E, Paulsen J. Using six sigma to reduce medication errors in a home-delivery pharmacy service. Joint Commission Journal on Quality & Patient Safety. 2005;31(6):319-24. PubMed PMID: 106532392. Language: English. Entry Date: 20051028. Revision Date: 20150711. Publication Type: Journal Article.

7385. Castle NG, Ferguson JC. What is nursing home quality and how is it measured? The Gerontologist. 2010;50(4):426-42.

7386. Castora-Binkley M, Hines L, Vaffis S, Dhatt H, Anderson E, Le D, et al. It is time for a new comprehensive medication review quality measure. Journal of Managed Care & Specialty Pharmacy. 2023;29(6):680-4. PubMed PMID: 37276042.

7387. Castrejon I, Cano Garcia L, Cuadrado Lozano MJ, Borras J, Galindo-Izquierdo M, Salman Monte TC, et al. PATIENT-REPORTED OUTCOMES MEASURES FOR SYSTEMIC LUPUS ERYTHEMATOSUS: AN EXPERT DELPHI CONSENSUS TO GUIDE IMPLEMENTATION IN ROUTINE CARE. Annals of the Rheumatic Diseases. 2023;82(Supplement 1):251. doi: https://dx.doi.org/10.1136/annrheumdis-2023-eular.1574. PubMed PMID: 642365560.

7388. Castrillon CC, Faus MJ, Gastclurrutia MA. Patients' Perceptions over a Generalized Medicine Management Service, in a Spanish Community Pharmacy. Pharmacy World & Science. 2009;31(4):499-. PubMed PMID: WOS:000267686100026.

7389. Castrillón CC, Faus MJ, Santos P, Calvo B, García-Delgado P, Gastelurrutia MA. Patients' perception of the pharmacotherapeutic follow-up service in a community pharmacy. Pharmaceutical Care Espana. 2010;12(1):4-11.

7390. Castro C, Pereira T, Sá JC, Santos G. Logistics reorganization and management of the ambulatory pharmacy of a local health unit in Portugal. Evaluation and Program Planning. 2020;80:101801. doi: https://doi.org/10.1016/j.evalprogplan.2020.101801.

7391. Castro Espinosa J, Jiménez Urrego ÁM, Botero Carvajal A. Factors related to technical management and adverse drug event reporting in independent retail pharmacies in Cali, Colombia. Heliyon. 2022;8(3):e09016. doi: https://doi.org/10.1016/j.heliyon.2022.e09016.

7392. Castro JGZ. Dietary Supplements with Green Tea Marketed in Portugal: Information on Website, Labels and Antioxidant Activity [Master's]. Portugal: Instituto Politecnico do Porto (Portugal); 2018.

7393. Castro PF, Larrain G, Baeza R, Corbalán R, Nazzal C, Greig DP, et al. Effects of glucose-insulin-potassium solution on myocardial salvage and left ventricular function after primary angioplasty. Critical care medicine. 2003;31(8):2152-5. doi: 10.1097/01.CCM.0000079604.46997.7B. PubMed PMID: CN-00440412.

7394. Castro R, Senecat J, de Chalendar M, Vajda I, Dan D, Boncz B, et al. Bridging the Gap between Health and Social Care for Rare Diseases: Key Issues and Innovative Solutions. In: DeLaPaz MP, Taruscio D, Groft SC, editors. Rare Diseases Epidemiology: Update and Overview, 2nd Edition. Advances in Experimental Medicine and Biology. 10312017. p. 605-27.

7395. Castro-Balado A, Varela-Rey I, Bandín-Vilar EJ, Busto-Iglesias M, García-Quintanilla L, Mondelo-García C, et al. Clinical research in hospital pharmacy during the fight against COVID-19. Farmacia hospitalaria : organo oficial de expresion cientifica de la Sociedad Espanola de Farmacia Hospitalaria. 2020;44(7):66-70. doi: 10.7399/fh.11494.

7396. Castro-Gómez A. Labeling of medications in anesthesia: colors and letters that save lives. Etiquetado de medicamentos en anestesia: colores y letras que salvan vidas. 2023;51(2):1-6. doi: 10.5554/22562087.e1065. PubMed PMID: 163777528.

7397. Castronovo A, Gervais F, Mongaret C, Slimano F. Pharmacists' interventions on prescription problems in one French community pharmacy: A prospective pilot study. Annales Pharmaceutiques Françaises. 2018;76(4):299-305. doi: https://doi.org/10.1016/j.pharma.2018.02.002.

7398. Casula M, Menditto E, Galimberti F, Russo V, Olmastroni E, Scotti L, et al. A pragmatic controlled trial to improve the appropriate prescription of drugs in adult outpatients: design and rationale of the EDU.RE.DRUG study. Primary Health Care Research & Development. 2020;21:1-7. doi: 10.1017/S1463423620000249. PubMed PMID: 144805708. Language: English. Entry Date: 20200811. Revision Date: 20220513. Publication Type: Article.

7399. Casula M, Menditto E, Galimberti F, Russo V, Olmastroni E, Scotti L, et al. A pragmatic controlled trial to improve the appropriate prescription of drugs in adult outpatients: design and rationale of the EDU.RE.DRUG study. Primary Health Care Research and Development. 2020;21. doi: 10.1017/s1463423620000249. PubMed PMID: WOS:000546581200001.

7400. Catala MTC, Royo LM, Molinera VG, Roy RS, Tur JP. CRIDECO Project. Screening for cognitive impairment in community pharmacy based on subjective memory complaint. Farmaceuticos Comunitarios. 2018;10(4):20-6. doi: 10.5672/FC.2173-9218.(2018/Vol10).004.04. PubMed PMID: WOS:000454562500004.

7401. Catalan A. The management of medication from the pharmacy does not improve drug-use in patients with coronary disease. FMC formacion medica continuada en atencion primaria. 2007;14(9):602-. PubMed PMID: CN-01744444.

7402. Cataldo RRV, Manaças LAR, Figueira PHM, Ferraz CVG, Calil-Elias S. Use of therapeutic outcomes monitoring method for performing of pharmaceutical care in oncology patients. Journal of Oncology Pharmacy Practice. 2022;28(4):884-91. doi: 10.1177/10781552211005072. PubMed PMID: 156580203. Language: English. Entry Date: 20220516. Revision Date: 20220517. Publication Type: Article.

7403. Cateau D, Ballabeni P, Bugnon O, Niquille A. Opportunities and limits to deprescribing in nursing homes (OLD-NH). Pharmacy practice. 2018;16. doi: 10.18549/PharmPract.2018.s1.1338. PubMed PMID: CN-02138134.

7404. Cateau D, Ballabeni P, Mena S, Bugnon O, Niquille A. Deprescribing in nursing homes: Protocol for nested, randomised controlled hybrid trials of deprescribing interventions. Research in Social and Administrative Pharmacy. 2021;17(4):786-94. doi: https://doi.org/10.1016/j.sapharm.2020.05.026.

7405. Cateau D, Ballabeni P, Niquille A. Effects of an interprofessional Quality Circle-Deprescribing Module (QC-DeMo) in Swiss nursing homes: a randomised controlled trial. BMC Geriatrics. 2021;21(1):1-11. doi: 10.1186/s12877-021-02220-y. PubMed PMID: 150197970. Language: English. Entry Date: In Process. Revision Date: 20211103. Publication Type: journal article.

7406. Cateau D, Ballabeni P, Niquille A. Effects of an interprofessional deprescribing intervention in Swiss nursing homes: the Individual Deprescribing Intervention (IDeI) randomised controlled trial. BMC geriatrics.

2021;21(1):655. doi: 10.1186/s12877-021-02465-7. PubMed PMID: CN-02356182.

7407. Cateau D, Bugnon O, Niquille A. Evolution of potentially inappropriate medication use in nursing homes: Retrospective analysis of drug consumption data. Research in Social & Administrative Pharmacy. 2021;17(4):701-6. doi: 10.1016/j.sapharm.2020.05.032. PubMed PMID: 149243051. Language: English. Entry Date: 20211111. Revision Date: 20211231. Publication Type: journal article.

7408. Cater SW, Luzum M, Serra AE, Arasaratnam MH, Travers D, Martin IBK, et al. A Prospective Cohort Study of Medication Reconciliation Using Pharmacy Technicians in the Emergency Department to Reduce Medication Errors among Admitted Patients. The Journal of Emergency Medicine. 2015;48(2):230-8. doi: https://doi.org/10.1016/j.jemermed.2014.09.065.

7409. Cates ME, Hogue MD. Experience with a drug screening program at a school of pharmacy. Journal of American College Health. 2012;60(6):476-80. doi: 10.1080/07448481.2012.688780.

7410. Cates ME, Hogue MD, Woolley TW. Effects of actual participation in drug testing on the attitudes of first-year pharmacy students toward a mandatory drug testing program. Currents in Pharmacy Teaching and Learning. 2015;7(2):224-30. doi: https://doi.org/10.1016/j.cptl.2014.11.006.

7411. Catic T, Begovic B. Outpatient antihypertensive drug utilization in Canton Sarajevo during five years period (2004-2008) and adherence to treatment guidelines assessment. Bosnian Journal of Basic Medical Sciences. 2011;11(2):97-102. PubMed PMID: WOS:000292012500007.

7412. Catic T, Stimac D, Zivkovic K, Zelic A. Outpatient utilization of psychopharmaceuticals: comparison between the cities of Zagreb and Sarajevo (2006-2009). Medicinski Glasnik. 2012;9(2):370-5. PubMed PMID: WOS:000307479600033.

7413. Catley A, Leyland T, Mariner JC, Akabwai DMO, Admassu B, Asfaw W, et al. Para-veterinary professionals and the development of quality, self-sustaining community-based services. OIE Revue Scientifique et Technique. 2004;23(1):225-52. doi: 10.20506/rst.23.1.1476.

7414. Caton J, Juillet Y. Rapport 23-15. Rapport inter-académique. La mise en application du règlement européen 2017/745/EU sur les dispositifs médicaux. Bulletin de l'Académie Nationale de Médecine. 2023;207(8):1007-13. doi: https://doi.org/10.1016/j.banm.2023.07.004.

7415. Cattaneo D, Lamers I, Bertoni R, Feys P, Jonsdottir J. Participation Restriction in People With Multiple Sclerosis: Prevalence and Correlations With Cognitive, Walking, Balance, and Upper Limb Impairments. Archives of Physical Medicine and Rehabilitation. 2017;98(7):1308-15. doi: 10.1016/j.apmr.2017.02.015. PubMed PMID: WOS:000404322500004.

7416. Cattaruzzi C, Cadelli L, Marcuzzo L, Antonini A, Groppo B, Ros B, et al. Feasibility of a multidisciplinary approach for medical review among elderly patients in four Italian long-term nursing homes. European Journal of Hospital Pharmacy. 2018;25(4):207-9. doi: 10.1136/ejhpharm-2015-000812. PubMed PMID: 138283141. Language: English. Entry Date: 20190829. Revision Date: 20190829. Publication Type: Article.

7417. Caturano B, Martin M. Project SOAR improves inpatient pharmacy at VA hospital: Lean principles used for standardization, optimization, alignment and redesign. ISE: Industrial & Systems Engineering at Work. 2023;55(11):40-7. PubMed PMID: 173132912.

7418. Catz SL, Jack LM, McClure JB, Javitz HS, Deprey M, Zbikowski SM, et al. Adherence to varenicline in the COMPASS smoking cessation intervention trial. Nicotine & tobacco research. 2011;13(5):361-8. doi: 10.1093/ntr/ntr003. PubMed PMID: CN-00801511.

7419. Caudal H, Briend-Godet V, Caroff N, Moret L, Navas D, Huon JF. Vaccine distrust: Investigation of the views and attitudes of parents in regard to vaccination of their children. Annales Pharmaceutiques Françaises. 2020;78(4):294-302. doi: https://doi.org/10.1016/j.pharma.2020.03.003.

7420. Caudell MA, Dorado-Garcia A, Eckford S, Creese C, Byarugaba DK, Afakye K, et al. Towards a bottom-up understanding of antimicrobial use and resistance on the farm: A knowledge, attitudes, and practices survey across livestock systems in five African countries. Plos One. 2020;15(1). doi: 10.1371/journal.pone.0220274. PubMed PMID: WOS:000534599100005.

7421. Caudle KE, Gammal RS, Whirl-Carrillo M, Hoffman JM, Relling MV, Klein TE. Evidence and resources to implement pharmacogenetic knowledge for precision medicine. American journal of health-system pharmacy. 2016;73(23):1977-85.

7422. Caulin C. Historique de l'évaluation des médicaments en vue d'une autorisation de mise sur le marché. Journal Français d'Ophtalmologie. 2008;31(1):71-4. doi: https://doi.org/10.1016/S0181-5512(08)70337-1.

7423. Cavaco A, Roter D. Pharmaceutical consultations in community pharmacies: Utility of the Roter Interaction Analysis System to study pharmacist-patient communication. International Journal of Pharmacy Practice. 2010;18(3):141-8. doi: 10.1211/ijpp/18.03.0003.

7424. Cavaco AM, Arslan M, Şar S. Informing the homeopathic practice for Turkish pharmacists: reviewing the example of Portuguese community pharmacies. Homeopathy. 2017;106(2):93-102. doi: 10.1016/j.homp.2017.02.003.

7425. Cavaco AM, Grilo A, Barros L. Exploring pharmacists' orientation towards patients in Portuguese community pharmacies. Journal of Communication in Healthcare. 2020;13(3):219-27. doi: 10.1080/17538068.2020.1804797.

7426. Cavaco AM, Krookas AA. Community pharmacies automation: any impact on counselling duration and job satisfaction? International journal of clinical pharmacy. 2014;36:325-35.

7427. Cavaco AM, Pereira PF. Pharmacists' counseling protocols for minor ailments: A structure-based analysis. Research in Social and Administrative Pharmacy. 2012;8(1):87-100. doi: 10.1016/j.sapharm.2011.07.004.

7428. Cavaco AM, Sozen-Sahne B, Ulutas-Deniz E, Yegenoglu S. Self-medication and non-prescription drug counseling: Illustrating profession uncertainty within Turkish pharmacy practice. Research in Social and Administrative Pharmacy. 2018;14(8):718-26. doi: https://doi.org/10.1016/j.sapharm.2017.09.006.

7429. Cavaco AMN. Practitioners' and public's perspectives of Portuguese community pharmacy: The role of job satisfaction and professionalism in actual and expected practice [Ph.D.]. England: University of London, University College London (United Kingdom); 2006.

7430. Cavada Carranza I, Garcia-Gonzalez X, Ibanez-Garcia S, Gomez-Costas D, Herranz-Alonso A, Sanjurjo-Saez M. FOUR YEARS OF A PHARMACEUTICAL CARE PROGRAMME IN PATIENTS UNDERGOING CARDIAC SURGERY. European Journal of Hospital Pharmacy. 2023;30(Supplement 1):A140-A1. doi: https://dx.doi.org/10.1136/ejhpharm-2023-eahp.292. PubMed PMID: 640966357.

7431. Cavada Carranza I, Sánchez-Rubio Ferrández J, Alonso Vega GG, Molina García T. Compliance to vaccination recommendations in people living with human immunodeficiency virus: Cross sectional study. Vacunas (English Edition). 2022;23(1):27-31. doi: https://doi.org/10.1016/j.vacune.2022.01.003.

7432. Cavalcante-Santos LM, Guarnieri AC, de Lima Conegundes FS, Giardini MH, Pereira LRL, Varallo

FR. Clinical pharmacy in hospital palliative medicine: non-randomised clinical trial. BMJ Supportive & Palliative Care. 2023.

7433. Cavalcanti AM, Rocha LM, Carillo R, Lima LU, Lugon JR. Effects of homeopathic treatment on pruritus of haemodialysis patients: a randomised placebo-controlled double-blind trial. Homeopathy. 2003;92(4):177-81. doi: 10.1016/j.homp.2003.07.002. PubMed PMID: CN-00465757.

7434. Cavallari LH, Cicali E, Wiisanen K, Fillingim RB, Chakraborty H, Myers RA, et al. Implementing a Pragmatic Clinical Trial to Tailor Opioids for Acute Pain on behalf of the IGNITE ADOPT PGx Investigators. Clinical and translational science. 2022;15(10):2479-92. doi: 10.1111/cts.13376. PubMed PMID: CN-02425773.

7435. Cavallier G, Laudet M, Vayssettes PM, Balayssac D, Chennell P. Hospital – community pharmacy coordination for the dispensing of oral antineoplastic drugs: An observational study in the French county of the Aveyron. Bulletin du Cancer. 2022;109(6):692-706. doi: 10.1016/j.bulcan.2022.02.007.

7436. Cavallier G, Laudet M, Vayssettes PM, Balayssac D, Chennell P. Hospital - community pharmacy coordination for the dispensing of oral antineoplastic drugs: An observational study in the French county of the Aveyron. Bulletin Du Cancer. 2022;109(6):692-706. doi: 10.1016/j.bulcan.2022.02.007. PubMed PMID: WOS:000833560100008.

7437. Cavallo D, Ursini CL, Perniconi B, Francesco AD, Giglio M, Rubino FM, et al. Evaluation of genotoxic effects induced by exposure to antineoplastic drugs in lymphocytes and exfoliated buccal cells of oncology nurses and pharmacy employees. Mutation Research/Genetic Toxicology and Environmental Mutagenesis. 2005;587(1):45-51. doi: https://doi.org/10.1016/j.mrgentox.2005.07.008.

7438. Cavanaugh J, Pinelli N, Eckel S, Gwynne M, Daniels R, Hawes EM. Advancing Pharmacy Practice through an Innovative Ambulatory Care Transitions Program at an Academic Medical Center. Pharmacy. 2020;8(1). doi: 10.3390/pharmacy8010040. PubMed PMID: WOS:000526522600027.

7439. Cavanaugh JJ, Lindsey KN, Shilliday BB, Ratner SP. Pharmacist-coordinated multidisciplinary hospital follow-up visits improve patient outcomes. Journal of Managed Care & Specialty Pharmacy. 2015;21(3):256-60. PubMed PMID: 25726034.

7440. Cavaye D, Lehnbom EC, Laba T-L, El-Boustani E, Joshi R, Webster R. Considering pharmacy workflow in the context of Australian community pharmacy: A pilot time and motion study. Research in Social and Administrative Pharmacy. 2018;14(12):1157-62.

7441. Cavaye D, Lehnbom EC, Laba TL, El-Boustani E, Joshi R, Webster R. Considering pharmacy workflow in the context of Australian community pharmacy: A pilot time and motion study. Research in Social and Administrative Pharmacy. 2018;14(12):1157-62. doi: 10.1016/j.sapharm.2018.01.003.

7442. Cavell GF, Mandaliya D. Magnitude of error: a review of wrong dose medication incidents reported to a UK hospital voluntary incident reporting system. European Journal of Hospital Pharmacy. 2021;28(5):260-5. doi: 10.1136/ejhpharm-2019-001987. PubMed PMID: 152178959. Language: English. Entry Date: 20210903. Revision Date: 20210903. Publication Type: Article.

7443. Caven M, Baiano CX, Robinson EM, Stephens B, Macpherson I, Dillon JF. Hepatitis C reinfection by treatment pathway among people who inject drugs in Tayside, Scotland. Journal of Viral Hepatitis. 2021;28(12):1744-50. doi: 10.1111/jvh.13614.

7444. Caveney B, Fagnano M, Halterman JS, Frey SM. Identifying which children with persistent asthma have preventive medications available at home. Journal of asthma. 2020;57(11):1188-94. doi:

10.1080/02770903.2019.1640734. PubMed PMID: CN-02202246.

7445. Cavero I, Crumb W. Moving towards better predictors of drug-induced Torsade de Pointes: 2–3 November 2005, Crystal City, Virginia, USA. Expert Opinion on Drug Safety. 2006;5(2):335-40.

7446. Cavero Rodrigo E, Climente Martí M, Navarro Fontestad MC, Jiménez Torres NV. Evaluación de la calidad de dos modelos de atención farmacéutica en pacientes onco-hematológicos. Farmacia Hospitalaria. 2007;31(4):231-7. doi: https://doi.org/10.1016/S1130-6343(07)75379-5.

7447. Cavicchi C, Vagnoni E. Sustainable Business Models in Hybrids: A Conceptual Framework for Community Pharmacies' Business Owners. Sustainability. 2020;12(19). doi: 10.3390/su12198125. PubMed PMID: WOS:000587224300001.

7448. Cavrenne P, Spinewine A. Continuity in patient care upon hospital discharge: Evaluation of a clinical pharmacy discharge form for community pharmacists. Journal de Pharmacie de Belgique. 2008;63(3):69-72.

7449. Cavrenne P, Spinewine A. Hospital discharge: Is information transfer from the hospital pharmacist to the community pharmacist useful? Farmaceutisch Tijdschrift voor Belgie. 2008;85(3):78-83.

7450. Cawley MJ, Warning WJ. Pharmacists performing quality spirometry testing: an evidence based review. International Journal of Clinical Pharmacy. 2015;37(5):726-33. doi: 10.1007/s11096-015-0160-x. PubMed PMID: WOS:000362415800011.

7451. Cawley MJ, Warning WJ. A systematic review of pharmacists performing obstructive sleep apnea screening services. International Journal of Clinical Pharmacy. 2016;38(4):752-60. doi: 10.1007/s11096-016-0319-0.

7452. Cawthon C, Walia S, Osborn CY, Niesner KJ, Schnipper JL, Kripalani S. Improving care transitions: the patient perspective. Journal of health communication. 2012;17 Suppl 3(Suppl 3):312-24. doi: 10.1080/10810730.2012.712619. PubMed PMID: CN-00840176.

7453. Cayci AB, Rathbone AP, Lindsey L, editors. Practices and perceptions of community pharmacists in the management of atopic dermatitis: a systematic review and thematic synthesis. Healthcare; 2023: MDPI.

7454. Cayci AB, Rathbone AP, Lindsey L. Practices and Perceptions of Community Pharmacists in the Management of Atopic Dermatitis: A Systematic Review and Thematic Synthesis. Healthcare (Switzerland). 2023;11(15). doi: 10.3390/healthcare11152159.

7455. Caylor S, Aebersold M, Lapham J, Carlson E. The Use of Virtual Simulation and a Modified TeamSTEPPS™ Training for Multiprofessional Education. Clinical Simulation in Nursing. 2015;11(3):163-71. doi: https://doi.org/10.1016/j.ecns.2014.12.003.

7456. Cazarim MD, Cruz-Cazarim ELC, Boyd K, Wu O, Nunes AA. Effect of Medication Therapy Management by Pharmaceutical Care on Blood Pressure and Cardiovascular Risk in Hypertension: A Systematic Review, Meta-Analysis, and Meta-Regression. Pharmaceuticals. 2023;16(6). doi: 10.3390/ph16060845. PubMed PMID: WOS:001017961900001.

7457. Ceballos M, Llano Y, Salazar-Ospina A, Madrigal-Cadavid J, Pino-Marín D, Amariles P. Skills and practices of pharmacy staff for dispensing of drugs with fiscalized substances in drugstores and pharmacies. Revista de Saude Publica. 2021;55:1-9. doi: 10.11606/s1518-8787.2021055003103.

7458. Ceballos M, Salazar-Ospina A, Sabater-Hernández D, Amariles P. Evaluation of the effects of a drug with fiscalized substance dispensation, health education, and pharmacovigilance continuing education

program in Colombia drugstores and drugstores/pharmacies: study protocol of a multicenter, cluster-randomized controlled trial. Trials. 2020;21(1):545. doi: 10.1186/s13063-020-04481-1. PubMed PMID: CN-02139444.

7459. Cecchini D, Alcaide ML, Rodriguez VJ, Mandell LN, Abbamonte JM, Cassetti I, et al. Women of Reproductive Age Living with HIV in Argentina: unique Challenges for Reengagement in Care. Journal of the International Association of Providers of AIDS Care. 2019;18:2325958219883250. doi: 10.1177/2325958219883250. PubMed PMID: CN-02133322.

7460. Cecere Feemster LM, Collins BF, Uman J, Au DH. Factors explaining variation in dyspnea, health-related quality of life, and medication use among veterans with COPD. American journal of respiratory and critical care medicine. 2013;187. PubMed PMID: CN-01107577.

7461. Cecere LM, Slatore CG, Uman JE, Evans LE, Udris EM, Bryson CL, et al. Adherence to long-acting inhaled therapies among patients with chronic obstructive pulmonary disease (COPD). COPD. 2012;9(3):251-8. doi: 10.3109/15412555.2011.650241. PubMed PMID: CN-00834161.

7462. Cecilia MJ, Atucha NM, García-Estañ J. Estilos de salud y hábitos saludables en estudiantes del Grado en Farmacia. Educación Médica. 2018;19:294-305. doi: https://doi.org/10.1016/j.edumed.2017.07.008.

7463. Cecilia MJ, García-Estañ J, Atucha NM. Self-medication in Pharmacy students. Educacion Medica. 2018;19(5):277-82. doi: 10.1016/j.edumed.2017.07.005.

7464. Cederbaum JA, Petering R, Hutchinson MK, He AS, Wilson JP, Jemmott JB, et al. Alcohol outlet density and related use in an urban Black population in Philadelphia public housing communities. Health & Place. 2015;31:31-8. doi: 10.1016/j.healthplace.2014.10.007. PubMed PMID: WOS:000348202100005.

7465. Cederlof C, Tomson G. Private pharmacies and the health sector reform in developing countries - Professional and commercial highlights. Journal of Social and Administrative Pharmacy. 1995;12(3):101-11.

7466. Cedillo-Galindo H, Gracida C. Barriers and Strategies for Taking Medicines in Adult Patients With Renal Transplantation. Transplantation Proceedings. 2011;43(9):3364-6. doi: https://doi.org/10.1016/j.transproceed.2011.09.084.

7467. Celentano AA, Ferruzzi MM, Sesana FF, Scaglione FF. Same initial drug name and pharmacist dispensing errors: a case series. Clinical Toxicology. 2023;61(Supplement 1):102-3. doi: https://dx.doi.org/10.1080/15563650.2023.2192024. PubMed PMID: 642034490.

7468. Çelik E. A pilot study on rational drug use and drug usage habits: Sağlık Bilimleri Enstitüsü; 2011.

7469. Celin A, Seuma J, Ramesh A. Assessment of drug related problems in stroke patients admitted to a South Indian tertiary care teaching hospital. Indian journal of pharmacy practice. 2012;5(4).

7470. Cello R, Conley M, Cooley T, Torre CDl, Dorn M, Ferer DS, et al. ASHP Guidelines on the Safe Use of Automated Dispensing Cabinets. American Journal of Health-System Pharmacy. 2022;79(1):e71-e82. doi: 10.1093/ajhp/zxab325. PubMed PMID: 154264958. Language: English. Entry Date: 20211231. Revision Date: 20230102. Publication Type: Article.

7471. Celum C, Grinsztejn B, Ngure K. Preparing for long-acting PrEP delivery: building on lessons from oral PrEP. Journal of the International AIDS Society. 2023;26(S2). doi: 10.1002/jia2.26103.

7472. Celum C, Hosek S, Tsholwana M, Kassim S, Mukaka S, Dye BJ, et al. PrEP uptake, persistence, adherence, and effect of retrospective drug level feedback on PrEP adherence among young women in

southern Africa: Results from HPTN 082, a randomized controlled trial. PLoS medicine. 2021;18(6):e1003670.

7473. Cen Z, Li J, Hu H, Lei KC, Loi CI, Liang Z, et al. Exploring the implementation of an outreach specialist program for nursing home residents in Macao: A multisite, qualitative study. Frontiers in Public Health. 2022;10. doi: 10.3389/fpubh.2022.950704.

7474. Cen ZF, Li JL, Hu H, Lei KC, Loi CI, Liang ZJ, et al. Exploring the implementation of an outreach specialist program for nursing home residents in Macao: A multisite, qualitative study. Frontiers in Public Health. 2022;10. doi: 10.3389/fpubh.2022.950704. PubMed PMID: WOS:000868069200001.

7475. Cen ZF, Tang PK, Hu H, Cavaco AC, Zeng L, Lei SL, et al. Systematic literature review of adopting eHealth in pharmaceutical care during COVID-19 pandemic: Recommendations for strengthening pharmacy services. BMJ Open. 2022;12(11). doi: 10.1136/bmjopen-2022-066246.

7476. Cena C, Traina S, Parola B, Bo M, Fagiano R, Siviero C. Prescription of proton pump inhibitors in older adults with complex polytherapy. European Journal of Hospital Pharmacy. 2020;27(6):341-5. doi: 10.1136/ejhpharm-2018-001697. PubMed PMID: 146641440. Language: English. Entry Date: 20201030. Revision Date: 20201030. Publication Type: Article.

7477. Centeno PD, Baig M, Lee SEM, Lane CJ, Lagomasino IT. Disclosure of Use and Abuse of Controlled Substances Among Psychiatric Outpatients. Journal of Psychiatric Practice. 2015;21(6):412-8. doi: 10.1097/pra.0000000000000104. PubMed PMID: WOS:000369711600003.

7478. Centers for Disease C, Prevention. Deaths from intravenous colchicine resulting from a compounding pharmacy error--Oregon and Washington, 2007. MMWR - Morbidity & Mortality Weekly Report. 2007;56(40):1050-2. PubMed PMID: 17932481.

7479. Centorrino F, Goren JL, Hennen J, Salvatore P, Kelleher JP, Baldessarini RJ. Multiple versus single antipsychotic agents for hospitalized psychiatric patients: case-control study of risks versus benefits. American Journal of Psychiatry. 2004;161(4):700-6.

7480. Cepeda MS, Farrar JT, Baumgarten M, Boston R, Carr DB, Strom BL. Side effects of opioids during short-term administration: Effect of age, gender, and race. Clinical Pharmacology & Therapeutics. 2003;74(2):102-12.

7481. Cepeda MS, Fife D, Berlin JA, Mastrogiovanni G, Yuan Y. Characteristics of prescribers whose patients shop for opioids: Results from a cohort study. Journal of Opioid Management. 2012;8(5):285-91. doi: 10.5055/jom.2012.0128.

7482. Cepeda MS, Fife D, Berwaerts J, Yuan Y, Mastrogiovanni G. Shopping Behavior for ADHD Drugs: Results of a Cohort Study in a Pharmacy Database. Drugs in R and D. 2014;14(3):205-11. doi: 10.1007/s40268-014-0058-4.

7483. Cepeda MS, Fife D, Chow W, Mastrogiovanni G, Henderson SC. Assessing opioid shopping behaviour: A large cohort study from a medication dispensing database in the US. Drug Safety. 2012;35(4):325-34. doi: 10.2165/11596600-000000000-00000.

7484. Cepeda MS, Fife D, Vo L, Mastrogiovanni G, Yuan Y. Risk of opioid shopping behavior: a comparison of two opioids in a large nationwide prescription database. Regional anesthesia and pain medicine. 2013;38(1). PubMed PMID: CN-01011151.

7485. Cepeda MS, Fife D, Vo L, Mastrogiovanni G, Yuan Y. Comparison of Opioid Doctor Shopping for

Tapentadol and Oxycodone: A Cohort Study. The Journal of Pain. 2013;14(2):158-64. doi: https://doi.org/10.1016/j.jpain.2012.10.012.

7486. Cepeda MS, Kern DM, Seabrook GR, Lovestone S. Comprehensive Real-World Assessment of Marketed Medications to Guide Parkinson's Drug Discovery. Clinical Drug Investigation. 2019;39(11):1067-75. doi: 10.1007/s40261-019-00830-4.

7487. Cerbin-Koczorowska IM, Waszyk-Nowaczyk M, Przymuszala P. Pharmacists' Preparedness to Patients Education at the Time of Pandemic-A Cross-Sectional Study with an Example of SARS-CoV-2 Outbreak in Poland. International Journal of Environmental Research and Public Health. 2020;17(18). doi: 10.3390/ijerph17186659. PubMed PMID: WOS:000580834200001.

7488. Cerbin-Koczorowska M, Przymuszala P, Zielinska-Tomczak L, Wawrzyniak E, Marciniak R. Is there a time and place for health education in chain pharmacies? Perspectives of Polish community pharmacists. Health and Social Care in the Community. 2021;29(5):e56-e66. doi: 10.1111/hsc.13242.

7489. Cerbin-Koczorowska M, Waszyk-Nowaczyk M, Odor K, Michalak M, Piotrowska S, Matschay A, et al. PHARMACEUTICAL CARE AS A CHANCE OF IMPROVING HEALTH CARE IN AREAS WITH AN AVERAGE LEVEL OF URBANIZATION. Farmacia. 2017;65(1):159-66. PubMed PMID: WOS:000394478400025.

7490. Cerbin-Koczorowska M, Zielinska-Tomczak L, Waszyk-Nowaczyk M, Michalak M, Skowron A. As the twig is bent, so is the tree inclined: a survey of student attitudes toward interprofessional collaboration supported with the curricula analysis. Journal of Interprofessional Care. 2019;33(6):636-44. doi: 10.1080/13561820.2019.1572598. PubMed PMID: WOS:000493764900005.

7491. Cercos CA, Catala MTC, Fernandez VJB, Soriano MJR, Monzo FA, Iglesias MCR. Intervention of community pharmacist in the vaccination of adults with respiratory disease. Farmaceuticos Comunitarios. 2018;10(3):5-14. doi: 10.5672/FC.2173-9218.(2018/Vol10).003.02. PubMed PMID: WOS:000445964800002.

7492. Cerda M, Krawczyk N, Keyes K. The future of the united states overdose crisis: challenges and opportunities. The Milbank Quarterly. 2023;101(Suppl 1):478.

7493. Cerfon M-A, Vernaudon J, Gervais F, Morelon E, Coste M-H, Krolak-Salmon P, et al. Drug-related problems in older patients with advanced chronic kidney disease identified during pretransplant comprehensive geriatric assessment. Néphrologie & Thérapeutique. 2022;18(1):45-51. doi: https://doi.org/10.1016/j.nephro.2021.08.006.

7494. Cerio AAP, Mallare NALB, Tolentino RMS. Assessment of the Legibility of the Handwriting in Medical Prescriptions of Doctors from Public and Private Hospitals in Quezon City, Philippines. Procedia Manufacturing. 2015;3:90-7. doi: https://doi.org/10.1016/j.promfg.2015.07.112.

7495. Cernasev A. A Narrative Inquiry of Socio-cultural Influences on Antiretroviral Treatment and Medication Experiences of African-born Persons Living with HIV in Minnesota [Ph.D.]. United States -- Minnesota: University of Minnesota; 2019.

7496. Cernasev A, Hohmeier K, Field C, Gordon AJ, Elliott S, Carlston K, et al. Co-use of Opioid Medications and Alcohol Prevention Study (COAPS). Substance Abuse. 2023;44(3):130-5. doi: 10.1177/08897077231191840.

7497. Cernasev A, Larson WL, Rockwood T, Peden-McAlpine C, Ranelli PL, Okoro O, et al. Narrative experiences of interactions with pharmacists among African-born persons living with HIV: "It's mostly

business.". Research in Social & Administrative Pharmacy. 2020;16(4):529-34. doi: 10.1016/j.sapharm.2019.07.009. PubMed PMID: WOS:000520862600010.

7498. Cernasev A, Melton TC, Jasmin H, Barenie RE. A Qualitative Systematic Literature Review of the Role of U.S. Pharmacists in Prescribing Pre-Exposure Prophylaxis (PrEP). Pharmacy. 2023;11(1). doi: 10.3390/pharmacy11010009. PubMed PMID: WOS:000940089300001.

7499. Cernasev A, Veve MP, Hohmeier KC, Summers NA, Kumar S. Importance of pharmacist-patient relationship in people living with HIV and concomitant opioid use disorder. Exploratory Research in Clinical and Social Pharmacy. 2021;3:100052. doi: https://doi.org/10.1016/j.rcsop.2021.100052.

7500. Cernasev A, Veve MP, Talbott T, Hall EA, Hohmeier KC. Pharmacy Students' Perceptions and Stigma Surrounding Naloxone Use in Patients with Opioid Use Disorder: A Mixed Methods Evaluation. Pharmacy. 2020;8(4). doi: 10.3390/pharmacy8040205. PubMed PMID: WOS:000601805100001.

7501. Cernohous T. A grounded theory approach to determining a patient's decision to use medication therapy management services [Ph.D.]. United States -- Minnesota: University of Minnesota; 2016.

7502. Cerović A, Miletić I, Konić-Ristić A, Baralić I, Djordjević B, Djuricić I, et al. The dry plant extract of common bean seed (Phaseoli vulgari pericarpium) does not have an affect on postprandial glycemia in healthy human subject. Udruzenje basicnih mediciniskih znanosti [Bosnian journal of basic medical sciences]. 2006;6(3):28-33. doi: 10.17305/bjbms.2006.3140. PubMed PMID: CN-00572370.

7503. Cerqueira Santos S, Boaventura TC, Rocha KSS, de Oliveira Filho AD, Onozato T, de Lyra DP. Can we document the practice of dispensing? A systematic review. Journal of Clinical Pharmacy and Therapeutics. 2016;41(6):634-44. doi: 10.1111/jcpt.12462.

7504. Cerqueira-Santos S, Rocha KSS, Araujo D, Santos GA, Menezes PWS, Sanchez JM, et al. Which factors may influence the implementation of drug dispensing in community pharmacies? A qualitative study. Journal of Evaluation in Clinical Practice. doi: 10.1111/jep.13731. PubMed PMID: WOS:000820519100001.

7505. Cerulli C, Cerulli J, Santos EJ, Lu N, He H, Kaukeinen K, et al. Does the health status of intimate partner violence victims warrant pharmacies as portals for public health promotion? Journal of the American Pharmacists Association. 2010;50(2):200-6. doi: 10.1331/JAPhA.2010.09094.

7506. Cerulli C, Cerulli J, Santos EJ, Lu NJ, He H, Kaukeinen K, et al. Does the health status of intimate partner violence victims warrant pharmacies as portals for public health promotion? Journal of the American Pharmacists Association. 2010;50(2):200-6. doi: 10.1331/JAPhA.2010.09094. PubMed PMID: WOS:000276806600015.

7507. Cerulli C, Inoue S, Cerulli J. How to identify, assess, and refer patients experiencing interpersonal violence across the lifespan: the role of US pharmacists in integrated pharmacy research and practice. Integrated Pharmacy Research and Practice. 2019;8:115-25. doi: 10.2147/iprp.S181213. PubMed PMID: WOS:000496756200001.

7508. Cerulli C, Nichols-Hadeed C, Raimondi C, Stone JT, Cerulli J. Facilitating intimate partner violence education among pharmacy students: What do future pharmacists want to know? Currents in Pharmacy Teaching and Learning. 2015;7(3):283-91. doi: https://doi.org/10.1016/j.cptl.2014.12.014.

7509. Cerulli J. Medication therapy management: reflecting on the profession's progress. Journal of the American Pharmacists Association: JAPhA. 2008;48(6):712, 4. PubMed PMID: 19019798.

7510. Cerulli J, Malone M. Women's health promotion within a community advanced pharmacy practice

experience. American journal of pharmaceutical education. 2008;72(2):25. doi: 10.5688/aj720225.

7511. Cerulli J, Zeolla MM. Impact and feasibility of a community pharmacy bone mineral density screening and education program. Journal of the American Pharmacists Association. 2004;44(2):161-7. doi: 10.1331/154434504773062618.

7512. Cerulli J, Zeolla MM, Cottrell J. Drug information questions posed to community pharmacy advanced practice experience students. American Journal of Pharmaceutical Education. 2004;68(3):1-5. doi: 10.5688/aj680368.

7513. Cerutti A, Ledoux T, Vantard N, Cerfon MA, Kimbidima R, Larbre V, et al. Comparing different robots available in the European market for the preparation of injectable chemotherapy and recommendations to users. Journal of Oncology Pharmacy Practice. doi: 10.1177/10781552221143786. PubMed PMID: WOS:000898937400001.

7514. Cerutti A, Ledoux T, Vantard N, Cerfon MA, Kimbidima R, Larbre V, et al. Comparing different robots available in the European market for the preparation of injectable chemotherapy and recommendations to users. Journal of Oncology Pharmacy Practice. 2022. doi: 10.1177/10781552221143786.

7515. Cerutti A, Ledoux T, Vantard N, Cerfon MA, Kimbidima R, Larbre V, et al. Comparing different robots available in the European market for the preparation of injectable chemotherapy and recommendations to users. Journal of Oncology Pharmacy Practice. 2023;29(7):1599-612. doi: 10.1177/10781552221143786.

7516. Cervera Barba EJ, Sagredo Pérez J, Martín González MC, Heras Salvat G, Peña Rodríguez E, Suárez del Villar Acebal T, et al. Oficinas de farmacia y centros de salud: podemos trabajar juntos. Una experiencia de colaboración. SEMERGEN - Medicina de Familia. 2004;30(10):491-7. doi: https://doi.org/10.1016/S1138-3593(04)74358-0.

7517. Cervi A, Diamantouros A, Azzam M, Lane SJ, Sapru H, Verhovsek M. Pharmacy hydroxyurea education materials for patients with sickle cell disease: An environmental scan and assessment of accuracy. Pediatric Blood and Cancer. 2020;67(5). doi: 10.1002/pbc.28179.

7518. Cesarz JL, Steffenhagen AL, Svenson J, Hamedani AG. Emergency Department Discharge Prescription Interventions by Emergency Medicine Pharmacists. Annals of Emergency Medicine. 2013;61(2):209-14.e1. doi: https://doi.org/10.1016/j.annemergmed.2012.04.011.

7519. Cesarz JL, Steffenhagen AL, Svenson J, Hamedani AG, Cesarz JL, Steffenhagen AL, et al. Emergency department discharge prescription interventions by emergency medicine pharmacists. Annals of Emergency Medicine. 2013;61(2):209-14.e1. doi: 10.1016/j.annemergmed.2012.04.011. PubMed PMID: 104294949. Language: English. Entry Date: 20130419. Revision Date: 20211029. Publication Type: journal article.

7520. Ceschi A, Noseda R, Pironi M, Lazzeri N, Eberhardt-Gianella O, Imelli S, et al. Effect of Medication Reconciliation at Hospital Admission on 30-Day Returns to Hospital: A Randomized Clinical Trial. JAMA Network Open. 2021;4(9):e2124672-e. doi: 10.1001/jamanetworkopen.2021.24672. PubMed PMID: 152511003. Language: English. Entry Date: 20210929. Revision Date: 20210929. Publication Type: Article.

7521. Cesen M, Drnovsek VM. The Process of health legislation reform in the Republic of Slovenia. European Journal of Health Law. 2000;7(1):73-84. doi: 10.1163/15718090020523061.

7522. Cespedes AP. A Patient With Acute Abdominal Pain is Referred to a Doctor as a Result of the Drug Indication Service in Costa Rica. Farmaceuticos Comunitarios. 2017;9(3):18-9. doi: 10.5672/FC.2173-9218.(2017/Vol9).003.04. PubMed PMID: WOS:000417277900004.

7523. Cespedes AP, Barrantes CL, Soto LEH. Characterization of Minor Illness Service inquires in 30 metropolitan community pharmacies in Costa Rica. Pharmaceutical Care Espana. 2016;18(1):16-27. PubMed PMID: WOS:000382704700003.

7524. Cessak G, Balkowiec-Iskra E. Evaluation of Pharmacists' Opinion and Knowledge Regarding Generic Medicinal Products in Poland. European Psychiatry. 2015;30:1612. doi: https://doi.org/10.1016/S0924-9338(15)31244-X.

7525. Cessna LG, Goebel LJ. Written Communication From Pharmacy Benefit Managers: Is It Helpful? Cureus Journal of Medical Science. 2023;15(12). doi: 10.7759/cureus.50342. PubMed PMID: WOS:001126862800028.

7526. Cesta A, Bajcar JM, Ong SW, Fernandes OA. The EMITT study: Development and evaluation of a medication information transfer tool. Annals of Pharmacotherapy. 2006;40(6):1074-81. PubMed PMID: 43886964.

7527. Ceulemans M, Liekens S, Van Calsteren K, Allegaert K, Foulon V. Community pharmacists' attitudes, barriers, knowledge and counseling practice with regard to preconception, pregnancy and lactation. Research in social & administrative pharmacy : RSAP. 2020;16(9):1192-200. doi: 10.1016/j.sapharm.2019.12.010. PubMed PMID: CN-02073290.

7528. Ceulemans M, Liekens S, Van Calsteren K, Allegaert K, Foulon V. Impact of a blended learning program on pharmaceutical care during preconception, pregnancy and lactation: results from a randomized controlled trial with comparative design. Reproductive toxicology (Elmsford, NY). 2020;97:7-. doi: 10.1016/j.reprotox.2020.04.037. PubMed PMID: CN-02242957.

7529. Ceulemans M, Liekens S, Van Calsteren K, Allegaert K, Foulon V. Impact of a blended learning program on community pharmacists' barriers, knowledge, and counseling practice with regard to preconception, pregnancy and lactation. Research in social & administrative pharmacy : RSAP. 2021;17(7):1242-9. doi: 10.1016/j.sapharm.2020.09.009. PubMed PMID: CN-02175987.

7530. Cevallos-Cevallos JM, Reyes-De-Corcuera JI. Chapter One - Metabolomics in Food Science. In: Henry J, editor. Advances in Food and Nutrition Research. 67: Academic Press; 2012. p. 1-24.

7531. Ceyhan G, Özpeynirci Ö. A branch and price algorithm for the pharmacy duty scheduling problem. Computers & Operations Research. 2016;72:175-82. doi: https://doi.org/10.1016/j.cor.2016.02.007.

7532. Chaar B, Brien JA, Krass I. Professional ethics in pharmacy: The Australian experience. International Journal of Pharmacy Practice. 2005;13(3):195-204. doi: 10.1211/ijpp.13.3.0005.

7533. Chaar B, Kwong K. Direct-to-consumer advertising: Australian pharmacists' experiences with non-prescription medicines. International Journal of Pharmacy Practice. 2010;18(1):43-50. doi: 10.1211/ijpp.18.01.0008.

7534. Chaar BB, Hanrahan, Jr., Day C. Provision of opioid substitution therapy services in Australian pharmacies. Australasian Medical Journal. 2011;4(4):210-6. doi: 10.4066/AMJ.2011.706.

7535. Chaar BB, Ram S. Professional Ethics in Pharmacy Clinical Governance. In: Babar Z-U-D, editor. Encyclopedia of Pharmacy Practice and Clinical Pharmacy. Oxford: Elsevier; 2019. p. 1-7.

7536. Chaar BB, Wang H, Day CA, Hanrahan JR, Winstock AR, Fois R. Factors influencing pharmacy services in opioid substitution treatment. Drug and Alcohol Review. 2013;32(4):426-34. doi: 10.1111/dar.12032. PubMed PMID: WOS:000321204600013.

7537. Chabikuli N, Schneider H, Blaauw D, Zwi AB, Brugha R. Quality and equity of private sector care for sexually transmitted diseases in South Africa. Health Policy and Planning. 2002;17:40-6. doi: 10.1093/heapol/17.suppl_1.40. PubMed PMID: WOS:000180003700006.

7538. Chabod F, Khalife A, Vanel F, Rondelot G. Choix entre les dispositifs médicaux stériles à usage unique ou réutilisables : un outil d'aide à la décision économique. Le Pharmacien Hospitalier et Clinicien. 2019;54(3):232-40. doi: https://doi.org/10.1016/j.phclin.2019.02.006.

7539. Chabosseau S, Derbré S. Cancer du sein : recommandations sur l'usage de la phytothérapie. Actualités Pharmaceutiques. 2016;55(552):45-9. doi: https://doi.org/10.1016/j.actpha.2015.11.011.

7540. Chabot I, Moisan J, Gregoire JP, Milot A. Pharmacist intervention program for control of hypertension. Annals of pharmacotherapy. 2003;37(9):1186-93. PubMed PMID: CN-00597601.

7541. Chabrier A, Lebel D, Bussières JF. Évolution du cadre juridique et normatif entourant l'exercice de la pharmacie hospitalière et le bon usage des médicaments au Québec. Annales Pharmaceutiques Françaises. 2019;77(6):496-505. doi: https://doi.org/10.1016/j.pharma.2019.08.001.

7542. Chabrier A, Rault P, Atkinson S, Bussieres JF. Conformity of the drug circuit in care units and outpatient clinics: a cross-sectional observational study in a 500-bed university hospital. Canadian Journal of Hospital Pharmacy. 2020;73(2):145-51. PubMed PMID: WOS:000591537400007.

7543. Chaccour C, Casellas A, Hammann F, Ruiz-Castillo P, Nicolas P, Montaña J, et al. BOHEMIA: Broad One Health Endectocide-based Malaria Intervention in Africa—a phase III cluster-randomized, open-label, clinical trial to study the safety and efficacy of ivermectin mass drug administration to reduce malaria transmission in two African settings. Trials. 2023;24(1):128.

7544. Chace Dwyer S, Jain A, Baruwa S, Okafor E, Babajide Oluseyi D, Ubuane O. How do fertility intentions lead to contraceptive continuation among a cohort of family planning users who received services from the private sector in Nigeria. Gates Open Research. 2022;5. doi: 10.12688/gatesopenres.13253.2.

7545. Chackunkal E, Dhanapal Vogel V, Grycki M, Kostoff D. Improving adherence to the Epic Beacon ambulatory workflow. Journal of Oncology Pharmacy Practice. 2017;23(4):273-7. doi: 10.1177/1078155216637215. PubMed PMID: 122523034. Language: English. Entry Date: 20170422. Revision Date: 20170426. Publication Type: Article.

7546. Chadalavada V, Babu S, Balamurugan K. Nonprescription sale of schedule H1 antibiotics in a city of South India. Indian Journal of Pharmacology. 2020;52(6):482-7. doi: 10.4103/IJP.IJP_244_19.

7547. Chadalavada V, Babu SM, Balamurugan K. Nonprescription sale of schedule H1 antibiotics in a city of South India. Indian Journal of Pharmacology. 2020;52(6):482-7. doi: 10.4103/ijp.IJP_244_19. PubMed PMID: WOS:000626097800005.

7548. Chadi A, Thirion DJG, David PM. Vaccine promotion strategies in community pharmacy addressing vulnerable populations: a scoping review. BMC Public Health. 2023;23(1). doi: 10.1186/s12889-023-16601-y.

7549. Chadwick DW, Mundy D, New J. Experiences of using a PKI to access a hospital information system by high street opticians. Computer Communications. 2003;26(16):1893-903. doi: 10.1016/s0140-3664(03)00084-7. PubMed PMID: WOS:000185412800009.

7550. Chae H-W, Kim Y, Suh Y, Lee J, Lee E, Lee E, et al. Prevalence of potentially inappropriate medications based on the STOPPFrail criteria in frail older patients with limited life expectancy: a cross-

sectional study. BMC Geriatrics. 2022;22(1):1-9. doi: 10.1186/s12877-022-03067-7. PubMed PMID: 156547570. Language: English. Entry Date: In Process. Revision Date: 20220717. Publication Type: journal article. Journal Subset: Biomedical.

7551. Chae S, McPherson ML, Walker K. Assessing conversion to transdermal fentanyl or methadone during transition on hospice admission. Journal of pain & palliative care pharmacotherapy. 2020;34(4):164-. doi: 10.1080/15360288.2020.1846416. PubMed PMID: CN-02263407.

7552. Chaffee BW, Lander MJ, Christen C, Redic KA. Surface contamination of counting tools after mock dispensing of cyclophosphamide in a simulated outpatient pharmacy. Journal of Oncology Pharmacy Practice. 2019;25(1):85-93. doi: 10.1177/1078155218764587.

7553. Chafin CC, Tolley EA, George CM, Demirkan K, Kuhl DA, Pugazhenthi M, et al. Gender differences in metered-dose inhaler-spacer device technique. Pharmacotherapy. 2000;20(11):1324-7. doi: 10.1592/phco.20.17.1324.34892. PubMed PMID: CN-01780717.

7554. Chagas MO, de Mendonça Lima T, Rebustini F, Noll M, de Carvalho Queiroz DP, Hernandes JC, et al. Instruments to assess the role of the clinical pharmacist: a systematic review. Systematic Reviews. 2022;11(1). doi: 10.1186/s13643-022-02031-1.

7555. Chagas MO, Lima TD, Rebustini F, Noll M, Queiroz DPD, Hernandes JC, et al. Instruments to assess the role of the clinical pharmacist: a systematic review. Systematic Reviews. 2022;11(1). doi: 10.1186/s13643-022-02031-1. PubMed PMID: WOS:000843136600002.

7556. Chahine B, Al Souheil F. Dispensing patterns of drugs used for neuropathic pain and adherence to NeuPSIG guideline: an observational study. Egyptian Journal of Neurology Psychiatry and Neurosurgery. 2021;57(1). doi: 10.1186/s41983-021-00398-x. PubMed PMID: WOS:000711437600001.

7557. Chahine B, Cherfane M, Sakr F, Safwan J, Dabbous M, Akel M, et al. Community pharmacists' perceptions and role in the management of common dermatological problems in Lebanon: a cross-sectional study. International journal of pharmacy practice. 2021;29(6):573-9. doi: 10.1093/ijpp/riab056. PubMed PMID: CN-02323500.

7558. Chahine B, Souheil FA. Oral contraceptives: knowledge and counselling practices of Lebanese community pharmacists. International Journal of Pharmacy Practice. 2022;30(1):45-51. doi: 10.1093/ijpp/riab069.

7559. Chahwakilian P, Huttner B, Schlemmer B, Harbarth S. Impact of the French campaign to reduce inappropriate ambulatory antibiotic use on the prescription and consultation rates for respiratory tract infections. Journal of Antimicrobial Chemotherapy. 2011;66(12):2872-9. doi: 10.1093/jac/dkr387. PubMed PMID: WOS:000297244100025.

7560. Chai D, Liu Z, Wang L, Duan H, Zhao C, Xu C, et al. Effectiveness of Medication Reconciliation in a Chinese Hospital: A Pilot Randomized Controlled Trial. Journal of Multidisciplinary Healthcare. 2023;16:3641-50. doi: https://dx.doi.org/10.2147/JMDH.S432522. PubMed PMID: 2026759150.

7561. Chaichian Y, Weisman MH, Simard JF. Pulse dose steroid experience among hospitalized patients with systemic lupus erythematosus: a single-center feasibility study. Clinical Rheumatology. 2021;40(4):1317-20. doi: 10.1007/s10067-021-05644-4. PubMed PMID: CN-02492039.

7562. Chaiken BP, Christian CE, Johnson L. Quality and efficiency successes leveraging IT and new processes. Journal of healthcare information management : JHIM. 2007;21(1):48-53.

7563. Chaikomon K, Sirivongrangson P, Lumlertgul N, Peerapornratana S, Praditpornsipa K, Tungsanga K, et al. The outcomes of comprehensive vs standard care in acute kidney injury survivors: a multicenter randomized controlled trial-a 6-month interim analysis. Nephrology (Carlton, Vic). 2020;25(SUPPL 4):29-30. doi: 10.1111/nep.v25.S4. PubMed PMID: CN-02242959.

7564. Chairokanjana K. Development and provision of patient medication records (PMRs) in community pharmacy in Thailand [Ph.D.]. Wales: The University of Wales College of Cardiff (United Kingdom); 1998.

7565. Chaitesipaseut L, Norton JW, Trivelli K, Argon S, Petrie I, Ragueneau-Majlessi I, et al. Evaluating the feasibility of performing pharmacogenetic guided-medication therapy management in a retirement community: A prospective, single arm study. Journal of the American College of Clinical Pharmacy. 2022;5(3):291-301. doi: 10.1002/jac5.1570. PubMed PMID: WOS:000729371600001.

7566. Chaitesipaseut L, Shah N, Truong T-G, Niu F, Shin P, Sharma K, et al. Outcomes of weight-based vs. fixed dose of Pembrolizumab among patients with non-small cell lung cancer. Journal of Oncology Pharmacy Practice. 2023:1. doi: 10.1177/10781552231212926. PubMed PMID: 173840692.

7567. Chaitesipaseut L, Wilson Norton J, Trivelli K, Argon S, Petrie I, Ragueneau-Majlessi I, et al. Evaluating the feasibility of performing pharmacogenetic guided-medication therapy management in a retirement community: A prospective, single arm study. JACCP Journal of the American College of Clinical Pharmacy. 2022;5(3):291-301. doi: 10.1002/jac5.1570.

7568. Chaitha W, Viboonsunti C, Permsuwan U, Awipan R, Karnjanarat P. Confirmatory factor analysis of an instrument to assess quality in pharmacy service of highly-active antiretroviral therapy program. Chiang Mai University Journal of Natural Sciences. 2010;9(1):13-28.

7569. Chaitin C, Velasquez J, Khanfar NM, Chassange S, Perez Torres R, Loan Pham N, et al. Third-year pharmacy students propose an interprofessional prediabetes educational programme: PreDiaMe (Prediabetes + Me). Journal of Interprofessional Care. 2018;32(1):118-22. doi: 10.1080/13561820.2017.1334636.

7570. Chaitin C, Velasquez J, Khanfar NM, Chassange S, Torres RP, Pham NL, et al. Third-year pharmacy students propose an interprofessional prediabetes educational programme: PreDiaMe (Prediabetes plus Me). Journal of Interprofessional Care. 2018;32(1):118-22. doi: 10.1080/13561820.2017.1334636. PubMed PMID: WOS:000416619100020.

7571. Chaitman BR, Pepine CJ, Parker JO, Skopal J, Chumakova G, Kuch J, et al. Effects of ranolazine with atenolol, amlodipine, or diltiazem on exercise tolerance and angina frequency in patients with severe chronic angina: a randomized controlled trial. Jama. 2004;291(3):309-16.

7572. Chaitman BR, Skettino SL, Parker JO, Hanley P, Meluzin J, Kuch J, et al. Anti-ischemic effects and long-term survival during ranolazine monotherapy in patients with chronic severe angina. Journal of the American College of Cardiology. 2004;43(8):1375-82.

7573. Chaitoff A, Strong AT, Bauer SR, Garber A, Landreneau JP, French J, et al. Educational Targets to Reduce Medication Errors by General Surgery Residents. Journal of Surgical Education. 2019;76(6):1612-21. doi: https://doi.org/10.1016/j.jsurg.2019.04.009.

7574. Chaix C, Durand-Zaleski I, Alberti C, Brun-Buisson C. A model to compute the medical cost of patients in intensive care. PharmacoEconomics. 1999;15(6):573-82. doi: 10.2165/00019053-199915060-00005. PubMed PMID: CN-00380767.

7575. Chaiyakunapruk N, Jones SM, Dhippayom T, Sumpradit N. Pharmacy Practice in Thailand. Pharmacy Practice in Developing Countries: Achievements and Challenges2016. p. 3-22.

7576. Chaiyakunapruk N, Laowakul A, Karnchanarat S, Pikulthong N, Ongphiphadhanakul B. Community Pharmacy–Based Implementation and Evaluation of an Osteoporosis Self-Assessment Tool for Asians. Journal of the American Pharmacists Association. 2006;46(3):391-6. doi: https://doi.org/10.1331/154434506777069624.

7577. Chaiyakunapruk N, Laowakul A, Karnchanarat S, Pikulthong N, Ongphiphadhanakul B. Community pharmacy-based implementation and evaluation of an Osteoporosis Self-Assessment Tool for Asians. Journal of the American Pharmacists Association. 2006;46(3):391-6. doi: 10.1331/154434506777069624.

7578. Chaiyakunapruk N, Laowakul A, Karnchanarat S, Pikulthong N, Ongphiphadhanakul B. Community pharmacy-based implementation and evaluation-of an Osteoporosis Self-Assessment Tool for Asians. Journal of the American Pharmacists Association. 2006;46(3):391-6. doi: 10.1331/154434506777069624. PubMed PMID: WOS:000202971700016.

7579. Chakrabarti S, Sara J, Lobo R, Eiring R, Finnes H, Mitchell J, et al. Bolus 5-fluorouracil (5-FU) In Combination With Oxaliplatin Is Safe and Well Tolerated in Patients Who Experienced Coronary Vasospasm With Infusional 5-FU or Capecitabine. Clinical Colorectal Cancer. 2019;18(1):52-7. doi: https://doi.org/10.1016/j.clcc.2018.09.006.

7580. Chakraborti C, Egan J. The Lesser of Two Adverse Reactions. The Joint Commission Journal on Quality and Patient Safety. 2010;36(1):22-7. doi: https://doi.org/10.1016/S1553-7250(10)36004-1.

7581. Chakraborty A, Qato DM, Awadalla SS, Hershow RC, Dworkin MS. Antiretroviral therapy adherence among treatment-naive HIV-infected patients. AIDS. 2020;34(1):127-37. doi: 10.1097/QAD.0000000000002384.

7582. Chakraborty D, Babu Singu H, Kumar Kar A, Biswas W. From fear to faith in the adoption of medicine delivery application: An integration of SOR framework and IRT theory. Journal of Business Research. 2023;166:114140. doi: https://doi.org/10.1016/j.jbusres.2023.114140.

7583. Chakraborty P, Pasupuleti M, Shankar MRJ, Bharat GK, Krishnasamy S, Dasgupta SC, et al. First surveillance of SARS-CoV-2 and organic tracers in community wastewater during post lockdown in Chennai, South India: Methods, occurrence and concurrence. Science of the Total Environment. 2021;778. doi: 10.1016/j.scitotenv.2021.146252. PubMed PMID: WOS:000655718800007.

7584. Chakraborty S, Markatou M, Ball R. Likelihood Ratio Test-Based Drug Safety Assessment using R Package pvLRT. R Journal. 2023;15(1):101-21. doi: 10.32614/rj-2023-027. PubMed PMID: 172749836.

7585. Chakravarthy U, Harding SP, Rogers CA, Downes SM, Lotery AJ, Culliford LA, et al. Alternative treatments to inhibit VEGF in age-related choroidal neovascularisation: 2-year findings of the IVAN randomised controlled trial. The Lancet. 2013;382(9900):1258-67.

7586. Chalasani N, Li Y-J, Dellinger A, Navarro V, Bonkovsky H, Fontana RJ, et al. Clinical features, outcomes, and HLA risk factors associated with nitrofurantoin-induced liver injury. Journal of Hepatology. 2023;78(2):293-300. doi: https://doi.org/10.1016/j.jhep.2022.09.010.

7587. Chalasani N, Li Y-J, Dellinger A, Navarro V, Bonkovsky H, Fontana RJ, et al. Clinical features, outcomes, and HLA risk factors associated with nitrofurantoin-induced liver injury. Journal of Hepatology. 2023;78(2):293-300. doi: https://doi.org/10.1016/j.jhep.2022.09.010.

7588. Chalasani SH, Ramesh M. PMU12 - Stewardship of Medication Errors in A Developing Country's Scenario: A Clinical Pharmacist's Role. Value in Health. 2018;21:S85. doi: https://doi.org/10.1016/j.jval.2018.07.636.

7589. Chalasani SH, Syed J, Ramesh M, Patil V, Kumar TMP. Artificial intelligence in the field of pharmacy practice: A literature review. Exploratory Research in Clinical and Social Pharmacy. 2023;12. doi: 10.1016/j.rcsop.2023.100346. PubMed PMID: WOS:001132477700001.

7590. Chalasani SH, Syed J, Ramesh M, Patil V, Kumar TP. Artificial intelligence in the field of pharmacy practice: A literature review. Exploratory Research in Clinical and Social Pharmacy. 2023;12:100346.

7591. Chalasani SH, Syed J, Ramesh M, Patil V, Pramod Kumar TM. Artificial intelligence in the field of pharmacy practice: A literature review. Exploratory Research in Clinical and Social Pharmacy. 2023;12:100346. doi: https://dx.doi.org/10.1016/j.rcsop.2023.100346. PubMed PMID: 2027919526.

7592. Chalati W, Crilly P, Fletcher J, Kayyali R. A comparative study of the cost and uptake of community pharmacy "Stop Smoking and Emergency Contraception" services from the perspective of the National Health Service. Journal of Research in Pharmacy Practice. 2020;9(2):73-87. doi: 10.4103/jrpp.JRPP_20_35. PubMed PMID: WOS:000546020500003.

7593. Chalker J, Chuc NT, Falkenberg T, Tomson G. Private pharmacies in Hanoi, Vietnam: a randomized trial of a 2-year multi-component intervention on knowledge and stated practice regarding ARI, STD and antibiotic/steroid requests. Tropical medicine & international health. 2002;7(9):803-10. doi: 10.1046/j.1365-3156.2002.00934.x. PubMed PMID: CN-00398035.

7594. Chalker J, Chuc NTK, Falkenberg T, Do NT, Tomson G. STD management by private pharmacies in Hanoi: Practice and knowledge of drug sellers. Sexually Transmitted Infections. 2000;76(4):299-302. doi: 10.1136/sti.76.4.299.

7595. Chalker J, Chuc NTK, Falkenberg T, Tomson G. Private pharmacies in Hanoi, Vietnam: A randomized trial of a 2-year multi-component intervention on knowledge and stated practice regarding ARI, STD and antibiotic/steroid requests. Tropical Medicine and International Health. 2002;7(9):803-10. doi: 10.1046/j.1365-3156.2002.00934.x.

7596. Chalker J, Ratanawijitrasin S, Chuc NT, Petzold M, Tomson G. Effectiveness of a multi-component intervention on dispensing practices at private pharmacies in Vietnam and Thailand--a randomized controlled trial. Social science & medicine (1982). 2005;60(1):131-41. doi: 10.1016/j.socscimed.2004.04.019. PubMed PMID: CN-00505622.

7597. Chalker J, Ratanawijitrasin S, Chuc NTK, Petzold M, Tomson G. Effectiveness of a multi-component intervention on dispensing practices at private pharmacies in Vietnam and Thailand - A randomized controlled trial. Social Science and Medicine. 2005;60(1):131-41. doi: 10.1016/j.socscimed.2004.04.019.

7598. Chalker JC, Vialle-Valentin C, Liana J, Mbwasi R, Semali IA, Kihiyo B, et al. What roles do accredited drug dispensing outlets in Tanzania play in facilitating access to antimicrobials? Results of a multi-method analysis. Antimicrobial Resistance and Infection Control. 2015;4. doi: 10.1186/s13756-015-0075-2. PubMed PMID: WOS:000367369500001.

7599. Chalkidou A, Lambert M, Cordoba G, Taxis K, Hansen MP, Bjerrum L. Misconceptions and Knowledge Gaps on Antibiotic Use and Resistance in Four Healthcare Settings and Five European Countries—A Modified Delphi Study. Antibiotics. 2023;12(9). doi: 10.3390/antibiotics12091435.

7600. Chalkidou A, Lambert M, Cordoba G, Taxis K, Hansen MP, Bjerrum L. Misconceptions and Knowledge Gaps on Antibiotic Use and Resistance in Four Healthcare Settings and Five European Countries-A Modified Delphi Study. Antibiotics-Basel. 2023;12(9). doi: 10.3390/antibiotics12091435. PubMed PMID: WOS:001071118100001.

7601. Challet C, Koutsokera A, Hofer M, Sauty A, Schaad N. Medication review by a clinical pharmacist in an adult cystic fibrosis care center. Le Pharmacien Hospitalier et Clinicien. 2017;52(1):e28. doi: https://doi.org/10.1016/j.phclin.2017.01.071.

7602. Challet C, Koutsokera A, Hofer M, Schaad N, Sauty A. 255 Annual medication review by a clinical pharmacist in an adult cystic fibrosis care center. Journal of Cystic Fibrosis. 2015;14:S123. doi: https://doi.org/10.1016/S1569-1993(15)30430-6.

7603. Chalmers K, Elshaug AG, Larkin S. First steps towards price transparency: comparability of online out-of-pocket tools from Australian private health funds. Australian Health Review. 2020;44(3):347-54. doi: 10.1071/ah19109. PubMed PMID: WOS:000538152000005.

7604. Chalton M. Improving access to novel heart failure agents through a defined multidisciplinary pathway. Heart & Lung. 2018;47(6):658. doi: https://doi.org/10.1016/j.hrtlng.2018.10.018.

7605. Chalupa R, Babica J, Nesměrák K. Pharmacists, chemistry and faculty of science of Charles university. Chemicke Listy. 2020;114(10):635-43.

7606. Cham M, Sefah I, Oppon K, Odikro R, Amenorvi C, Malik A, et al. Availability of Parkinson's disease medicines in Ghana: a national survey. Movement disorder. 2019;34:S34-. PubMed PMID: CN-02095009.

7607. Chambela MDC, Mediano MFF, Carneiro FM, Ferreira RR, Waghabi MC, Mendes VG, et al. Impact of pharmaceutical care on the quality of life of patients with heart failure due to chronic Chagas disease: randomized clinical trial. British journal of clinical pharmacology. 2020;86(1):143-54. doi: 10.1111/bcp.14152. PubMed PMID: CN-02005829.

7608. Chamberlain JM, Kapur J, Shinnar S, Elm J, Holsti M, Babcock L, et al. Efficacy of levetiracetam, fosphenytoin, and valproate for established status epilepticus by age group (ESETT): a double-blind, responsive-adaptive, randomised controlled trial. The Lancet. 2020;395(10231):1217-24. doi: https://doi.org/10.1016/S0140-6736(20)30611-5.

7609. Chamberlain JM, Kapur J, Shinnar S, Elm J, Holsti M, Babcock L, et al. Efficacy of levetiracetam, fosphenytoin, and valproate for established status epilepticus by age group (ESETT): a double-blind, responsive-adaptive, randomised controlled trial. Lancet (london, england). 2020;395(10231):1217-24. doi: 10.1016/S0140-6736(20)30611-5. PubMed PMID: CN-02097054.

7610. Chamberlain MA. The vertically integrated pharmacy department. American Journal of Health-System Pharmacy. 1998;55(7):669-75. doi: 10.1093/ajhp/55.7.669. PubMed PMID: WOS:000072900000010.

7611. Chamberlain R, Huyton J, James D. Pharmacy Technicians' Roles and Responsibilities in the Community Pharmacy Sector: A Welsh Perspective. Pharmacy. 2020;8(2). doi: 10.3390/pharmacy8020097. PubMed PMID: WOS:000552756500016.

7612. Chambers B, Allan J, Webster E, Packer A, Nott S. Feasibility and acceptability of a virtual clinical pharmacy service for elective orthopaedic inpatients in an Australian metropolitan hospital. Journal of Pharmacy Practice & Research. 2023;53(2):79-86. doi: 10.1002/jppr.1853. PubMed PMID: 162971826.

7613. Chambers B, Fleming C, Packer A, Botha L, Hawthorn G, Nott S. Virtual clinical pharmacy services: A model of care to improve medication safety in rural and remote Australian health services. American Journal of Health-System Pharmacy. 2022;79(16):1376-84. PubMed PMID: 35291005.

7614. Chambers JD. Do changes in drug coverage policy point to an increased role for cost-effectiveness analysis in the USA? PharmacoEconomics. 2014;32(8):729-33. doi: 10.1007/s40273-014-0166-6.

7615. Chambers JD, Pope EF, Wilkinson CL, Neumann PJ. Discrepancies between FDA-required labeling and evidence that payers cite in drug coverage policies. Journal of Managed Care and Specialty Pharmacy. 2018;24(12):1240-6A. doi: 10.18553/jmcp.2018.24.12.1240.

7616. Chambers JD, Wilkinson CL, Anderson JE, Chenoweth MD. Variation in Private Payer Coverage of Rheumatoid Arthritis Drugs. Journal of Managed Care & Specialty Pharmacy. 2016;22(10):1176-81. doi: 10.18553/jmcp.2016.22.10.1176. PubMed PMID: WOS:000384393300006.

7617. Chambers K. Looking ahead to this year's CPhl. Pharmaceutical Technology Europe. 2006;18(9):54-7.

7618. Chambers LW, Kaczorowski J, Dolovich L, Karwalajtys T, Hall HL, McDonough B, et al. A community-based program for cardiovascular health awareness. Canadian Journal of Public Health. 2005;96(4):294-8. doi: 10.1007/bf03405169.

7619. Chambers LW, Kaczorowski J, O'Rielly S, Ignagni S, Hearps SJ. Comparison of blood pressure measurements using an automated blood pressure device in community pharmacies and family physicians' offices: a randomized controlled trial. CMAJ open. 2013;1(1):E37-42. doi: 10.9778/cmajo.20130005. PubMed PMID: CN-00997849.

7620. Chambers R. Potential for the abuse of medication for the elderly in residential and nursing homes in the UK. Journal of Elder Abuse & Neglect. 1999;10(1/2):79-89. PubMed PMID: 107201702. Language: English. Entry Date: 19990801. Revision Date: 20150711. Publication Type: Journal Article. Journal Subset: Biomedical.

7621. Chambliss WG, Carroll WA, Kennedy D, Levine D, Moné MA, Douglas Ried L, et al. Role of the pharmacist in preventing distribution of counterfeit medications. Journal of the American Pharmacists Association. 2012;52(2):195-9. doi: https://doi.org/10.1331/JAPhA.2012.11085.

7622. Chambliss WG, Carroll WA, Kennedy D, Levine D, Mone MA, Ried LD, et al. Role of the pharmacist in preventing distribution of counterfeit medications. Journal of the American Pharmacists Association: JAPhA. 2012;52(2):195-9. PubMed PMID: 22370383.

7623. Chambliss WG, Hufford CD, Flagg ML, Glisson JK. Assessment of the Quality of Reference Books on Botanical Dietary Supplements. Journal of the American Pharmaceutical Association (1996). 2002;42(5):723-34. doi: https://doi.org/10.1331/108658002764653504.

7624. Chambrier P, Villeneuve P. Pharmacist self-assessment questionnaire concerning maintenance of the cold chain in the pharmacy. STP Pharma Pratiques. 2013;23(1):9-13.

7625. Chambuso M, Mugoyela V, Kalala W. Consumer survey of malaria fact card: an educational and communication tool in Tanzania. East African journal of public health. 2007;4(2):59-63.

7626. Chamorro M, García-Jiménez E, Amariles P, Chamorro AR, Merino EM, Martínez FM, et al. Effect of pharmacist involvement in adherence to medications in patients with high to moderate cardiovascular risk (Study EMDADER-CV-INCUMPLIMIENTO). Atencion primaria / Sociedad Espanola de Medicina de Familia y Comunitaria. 2011;43(5):245-53. doi: 10.1016/j.aprim.2010.05.006. PubMed PMID: CN-00802015.

7627. Chamorro MA. Effect of pharmacist involvement in adherence to medications in patients with high to moderate cardiovascular risk (EMDADER-CV-INCUMPLIMIENTO). Efecto de la actuacion farmaceutica en la adherencia del tratamiento farmacologico de pacientes ambulatorios con riesgo cardiovascular (emdader-cv-incumplimiento). 2008. PubMed PMID: CN-00991163.

7628. Chamorro MAR, Garcia-Jimenez E, Amariles P, Chamorro AR, Merino EMP, Martinez FM, et al. Effect of pharmacist involvement in adherence to medications in patients with high to moderate cardiovascular risk (Study EMDADER-CV-INCUMPLIMIENTO). Atencion Primaria. 2011;43(5):245-53. doi: 10.1016/j.aprim.2010.05.006. PubMed PMID: WOS:000290844700007.

7629. Chamorro-de-Vega E, Rodríguez-González CG, Giménez-Manzorro Á, Herranz A, Sanjurjo M. Hepatitis C virus infection and the role of a pharmaceutical care program. American Journal of Health-System Pharmacy. 2020;77(6):479-86. doi: 10.1093/ajhp/zxz266. PubMed PMID: 142126239. Language: English. Entry Date: 20200311. Revision Date: 20210315. Publication Type: Article.

7630. Chamorro-de-Vega E, Rodriguez-Gonzalez CG, Gimenez-Manzorro A, Lorenzo-Pinto A, Iglesias-Peinado I, Herranz A, et al. Improving pharmacotherapy outcomes in patients with hepatitis C virus infection treated with direct-acting antivirals: The GRUviC project. International Journal of Clinical Practice. 2017;71(8):n/a-n/a. doi: 10.1111/ijcp.12988. PubMed PMID: 124834620. Corporate Author: GRUviC Study Group. Language: English. Entry Date: 20171129. Revision Date: 20180815. Publication Type: journal article. Journal Subset: Biomedical.

7631. Chamouard V, Freyssenge J, Duport G, Volot F, Varin R, Giraud N, et al. Evaluation of the care pathway in the context of the dispensing of emicizumab (Hemlibra) in community and hospital pharmacies in France: A patient satisfaction survey. Haemophilia. 2023;29(6):1490-8. doi: 10.1111/hae.14857. PubMed PMID: WOS:001069247100001.

7632. Chamouard V, Polosse M, Nageotte A, Claveranne JP, Mercatello A. Le circuit du médicament en anesthésie à l'hôpital Édouard-Herriot. Annales Françaises d'Anesthésie et de Réanimation. 1999;18(3):376-84. doi: https://doi.org/10.1016/S0750-7658(99)80065-3.

7633. Chamoun N, Zeenny R, Mansour H, Chamoun NR. Impact of clinical pharmacy interventions on medication error nodes. International Journal of Clinical Pharmacy. 2016;38(6):1436-44. doi: 10.1007/s11096-016-0384-4. PubMed PMID: 119754944. Language: English. Entry Date: 20180726. Revision Date: 20190708. Publication Type: journal article.

7634. Chamoun NR, Zeenny R, Mansour H. Impact of clinical pharmacy interventions on medication error nodes. International Journal of Clinical Pharmacy. 2016;38(6):1436-44. PubMed PMID: 27770350.

7635. Champagne DR. A Study Assessing Awareness, Attitudes and Interest of Four Non-Dental Medicare Clinical Providers Related to an Alternative Intervention to Improve Oral Health Outcomes in Medical Facilities [M.S.]. United States -- District of Columbia: Howard University; 2014.

7636. Champaigne KD. Ion channel-mediated hydrostatic pressure mechanotransduction in urothelial cells [Ph.D.]. United States -- South Carolina: Clemson University; 2013.

7637. Champaloux SW, Tepper NK, Curtis KM, Zapata LB, Whiteman MK, Marchbanks PA, et al., editors. Contraceptive use among women with medical conditions in a nationwide privately insured population. Obstetrics and Gynecology; 2015.

7638. Champaloux SW, Tepper NK, Curtis KM, Zapata LB, Whiteman MK, Marchbanks PA, et al. Contraceptive Use Among Women With Medical Conditions in a Nationwide Privately Insured Population. Obstetrics and Gynecology. 2015;126(6):1151-9. doi: 10.1097/aog.0000000000001134. PubMed PMID: WOS:000365301300006.

7639. Champaloux SW, Tepper NK, Monsour M, Curtis KM, Whiteman MK, Marchbanks PA, et al. Use of combined hormonal contraceptives among women with migraines and risk of ischemic stroke. American Journal of Obstetrics and Gynecology. 2017;216(5):489.e1-.e7. doi: https://doi.org/10.1016/

j.ajog.2016.12.019.

7640. Champaneria A, Holsinger T, Davagnino J. COACH: Caring for Older Adults and Caregivers at Home. The American Journal of Geriatric Psychiatry. 2022;30(4, Supplement):S17-S8. doi: https://doi.org/10.1016/j.jagp.2022.01.278.

7641. Champenois M. Cass, 1re civ, 14 octobre 2010 : affaire « Catalgine® ». Le Pharmacien Hospitalier. 2011;46(1):42-4. doi: https://doi.org/10.1016/j.phhp.2011.01.018.

7642. Champion C, Sockolow PS, Bowles KH, Potashnik S, Yang Y, Pankok C, et al. Getting to Complete and Accurate Medication Lists During the Transition to Home Health Care. Journal of the American Medical Directors Association. 2021;22(5):1003-8. doi: https://doi.org/10.1016/j.jamda.2020.06.024.

7643. Chan A, Ngai GH, Chung WL, Yeo A, Ng T, Loh KWJ, et al. Practitioners' perspectives on community-based breast cancer survivorship care in Singapore: A focus group study. Health & Social Care in the Community. 2018;26(3):404-11. doi: 10.1111/hsc.12528. PubMed PMID: WOS:000429549900026.

7644. Chan A, Shih V, Chew L. Evolving roles of oncology pharmacists in Singapore: a survey on prescribing patterns of antiemetics for chemotherapy induced nausea and vomiting (CINV) at a cancer centre. Journal of Oncology Pharmacy Practice. 2008;14(1):23-9. PubMed PMID: 18337437.

7645. Chan A, Shih V, Chiang J, Chew L, Tay K, Quek R, et al. Clinical pharmacy services and research for lymphoma patients at a cancer center. Journal of Oncology Pharmacy Practice. 2013;19(1):24-30. PubMed PMID: 22706787.

7646. Chan A, Tan S-H, Wong CM, Yap KY-L, Ko Y. Clinically significant drug—drug interactions between oral anticancer agents and nonanticancer agents: A delphi survey of oncology pharmacists. Clinical Therapeutics. 2009;31:2379-86. doi: https://doi.org/10.1016/j.clinthera.2009.11.008.

7647. Chan AJ, Lebovic G, Wan M, Chen Y, Leung E, Langford BJ, et al. Impact of extended-infusion piperacillin-tazobactam in a Canadian community hospital. Infectious Medicine. 2023;2(1):31-5. doi: https://doi.org/10.1016/j.imj.2023.01.005.

7648. Chan AL. Use of Six Sigma to improve pharmacist dispensing errors at an outpatient clinic. American Journal of Medical Quality. 2004;19(3):128-31.

7649. Chan ALF. Use of six sigma to improve pharmacist dispensing errors at an outpatient clinic. American Journal of Medical Quality. 2004;19(3):128-31. doi: 10.1177/106286060401900306. PubMed PMID: WOS:000221706400006.

7650. Chan AYL, Ma TT, Lau WCY, Ip P, Coghill D, Gao L, et al. Attention-deficit/hyperactivity disorder medication consumption in 64 countries and regions from 2015 to 2019: a longitudinal study. eClinicalMedicine. 2023;58:101780. doi: https://dx.doi.org/10.1016/j.eclinm.2022.101780. PubMed PMID: 2024070892.

7651. Chan B, Edwards ST, Devoe M, Gil R, Mitchell M, Englander H, et al. The SUMMIT ambulatory-ICU primary care model for medically and socially complex patients in an urban federally qualified health center: study design and rationale. Addiction science & clinical practice. 2018;13(1):27. doi: 10.1186/s13722-018-0128-y. PubMed PMID: CN-01925167.

7652. Chan B, Edwards ST, Mitchell M, Kansagara D, Nicolaidis C, Korthuis PT, et al. An ambulatory intensive care unit ("a-ICU") for patients impacted by social determinants of health improved mental health functioning, patient well-being, and outpatient engagement at 6-months: interim results of summit

randomized controlled trial. Journal of general internal medicine. 2020;35(SUPPL 1):S12-. doi: 10.1007/s11606-020-05890-3. PubMed PMID: CN-02257597.

7653. Chan B, Edwards ST, Mitchell M, Nicolaidis C, Kansagara D, Korthuis PT, et al. Can an intensive ambulatory care intervention improve the experience of high-utilizing patients?: 6-month outcomes of the summit randomized controlled trial. Journal of general internal medicine. 2019;34(2):S151-. doi: 10.1007/11606.1525-1497. PubMed PMID: CN-01976812.

7654. Chan B, Edwards ST, Srikanth P, Mitchell M, Devoe M, Nicolaidis C, et al. Ambulatory Intensive Care for Medically Complex Patients at a Health Care Clinic for Individuals Experiencing Homelessness: The SUMMIT Randomized Clinical Trial. JAMA Network Open. 2023;6(11):E2342012. doi: 10.1001/jamanetworkopen.2023.42012.

7655. Chan B, Isenor JE, Kennie-Kaulbach N. Categorization of deprescribing communication tools: a scoping review. Basic & Clinical Pharmacology & Toxicology. 2023;133(6):640-52.

7656. Chan B, Mitchell M, Edwards ST, Saha S, Korthuis PT. Implementation and evaluation of summit, an ambulatory intensive care (A-ICU) model of primary care for high-utilizersata healthcare for the homeless site. Journal of general internal medicine. 2018;33(2):791-2. PubMed PMID: CN-01606174.

7657. Chan B, Reeve E, Matthews S, Carroll PR, Long JC, Held F, et al. Medicine information exchange networks among healthcare professionals and prescribing in geriatric medicine wards. British Journal of Clinical Pharmacology. 2017;83(6):1185-96. PubMed PMID: 28009444.

7658. Chan CC, Tung YC, Lee KT, Chan YH, Chu PH. Clinical outcomes of generic versus brand-name clopidogrel for secondary prevention in patients with acute myocardial infarction: A nationwide cohort study. CTS: Clinical & Translational Science. 2023;16(9):1594-605. doi: 10.1111/cts.13590. PubMed PMID: 171918079.

7659. Chan CM, Shorr AF. Economic and Outcomes Aspects of Venous Thromboembolic Disease. Critical Care Clinics. 2012;28(1):113-+. doi: 10.1016/j.ccc.2011.09.005. PubMed PMID: WOS:000298768000010.

7660. Chan CW, Siu SC, Wong CK, Lee VW. A pharmacist care program: positive impact on cardiac risk in patients with type 2 diabetes. Journal of cardiovascular pharmacology and therapeutics. 2012;17(1):57-64. doi: 10.1177/1074248410396216. PubMed PMID: CN-00897683.

7661. Chan CW, Siu SC, Wong CKW, Lee VWY. A Pharmacist Care Program: Positive Impact on Cardiac Risk in Patients With Type 2 Diabetes. Journal of Cardiovascular Pharmacology and Therapeutics. 2012;17(1):57-64. doi: 10.1177/1074248410396216. PubMed PMID: WOS:000299933100006.

7662. Chan CWE, Tan D, Byers J, Sarvesvaran J, Anderson D. Improving community lung cancer follow-up strategies in South Greater Glasgow and Clyde using 1-year survival data. Lung cancer (Amsterdam, Netherlands). 2022;165:S6-. doi: 10.1016/S0169-5002(22)00058-7. PubMed PMID: CN-02454839.

7663. Chan D, Fan MY, Unützer J. Long-term effectiveness of collaborative depression care in older primary care patients with and without PTSD symptoms. International journal of geriatric psychiatry. 2011;26(7):758-64. doi: 10.1002/gps.2606. PubMed PMID: CN-00794400.

7664. Chan D-C, Chen J-H, Wen C-J, Chiu L-S, Wu S-C. Effectiveness of the medication safety review clinics for older adults prescribed multiple medications. Journal of the Formosan Medical Association. 2014;113(2):106-13. doi: https://doi.org/10.1016/j.jfma.2012.04.013.

7665. Chan DC, Chen JH, Wen CJ, Chiu LS, Wu SC. Effectiveness of the medication safety review clinics

for older adults prescribed multiple medications. Journal of the Formosan Medical Association. 2014;113(2):106-13. doi: 10.1016/j.jfma.2012.04.013. PubMed PMID: WOS:000334015100007.

7666. Chan DM, Fan MY, Unutzer J. Long-term effectiveness of collaborative depression care in older primary care patients with and without PTSD symptoms. International Journal of Geriatric Psychiatry. 2011;26(7):758-64. doi: 10.1002/gps.2606. PubMed PMID: WOS:000289697100012.

7667. Chan ECY. Enhancing pharmaceutical analysis laboratory classes via the measurement of minerals in Berocca® effervescent tablets. Pharmacy Education. 2008;8(1):53-7. doi: 10.1080/15602210801933552.

7668. Chan EW, Taylor SE, Marriott JL, Barger B, Chan EW, Taylor SE, et al. Bringing patients' own medications into an emergency department by ambulance: effect on prescribing accuracy when these patients are admitted to hospital. Medical Journal of Australia. 2009;191(7):374-7. doi: 10.5694/j.1326-5377.2009.tb02842.x. PubMed PMID: 105229482. Language: English. Entry Date: 20100115. Revision Date: 20210103. Publication Type: journal article.

7669. Chan FW, Wong RS, Lau WH, Chan TY, Cheng G, You JH. Management of Chinese patients on warfarin therapy in two models of anticoagulation service - a prospective randomized trial. British journal of clinical pharmacology. 2006;62(5):601-9. doi: 10.1111/j.1365-2125.2006.02693.x. PubMed PMID: CN-00576737.

7670. Chan HK, Aswad E, Ho YE. Influences of pictogram-based instructions in paediatric drug labelling on dosing accuracy among caregivers: a pilot study from Malaysia. Journal of pharmaceutical health services research. 2017;(no pagination). doi: 10.1111/jphs.12169. PubMed PMID: CN-01329041.

7671. Chan HK, Hassali MA. Modified labels for long-term medications: influences on adherence, comprehension and preferences in Malaysia. International journal of clinical pharmacy. 2014;36(5):904-13. doi: 10.1007/s11096-014-0003-1. PubMed PMID: CN-01021553.

7672. Chan JGY, Kwok PCL, Young PM, Chan H-K, Traini D. Mannitol Delivery by Vibrating Mesh Nebulisation for Enhancing Mucociliary Clearance. Journal of Pharmaceutical Sciences. 2011;100(7):2693-702. doi: https://doi.org/10.1002/jps.22494.

7673. Chan JKK, Sleat G, Sharma S, Phoenix G, Graham A. Gastroprotection in trauma patients receiving non-steroidal anti-inflammatory drugs. The Surgeon. 2010;8(4):206-10. doi: https://doi.org/10.1016/j.surge.2009.11.007.

7674. Chan JTN, Nguyen V, Tran TN, Nguyen NV, Do NTT, Doorn HRV, et al. Point-of-care testing in private pharmacy and drug retail settings: a narrative review. Bmc Infectious Diseases. 2023;23(1). doi: 10.1186/s12879-023-08480-w. PubMed PMID: WOS:001053704700001.

7675. Chan JTN, Nguyen V, Tran TN, Nguyen NV, Do NTT, van Doorn HR, et al. Point-of-care testing in private pharmacy and drug retail settings: a narrative review. BMC Infectious Diseases. 2023;23(1):551.

7676. Chan KA, Andrade SE, Boles M, Buist DSM, Chase GA, Donahue JG, et al. Inhibitors of hydroxymethylglutaryl-coenzyme A reductase and risk of fracture among older women. The Lancet. 2000;355(9222):2185-8. doi: https://doi.org/10.1016/S0140-6736(00)02400-4.

7677. Chan KY, Lau CL, Adeeb SM, Somasundaram S, Nasir-Zahari M. A randomized, placebo-controlled, double-blind, prospective clinical trial of silicone gel in prevention of hypertrophic scar development in median sternotomy wound. Plastic and reconstructive surgery. 2005;116(4):1013-20; discussion 21-2. doi: 10.1097/01.prs.0000178397.05852.ce. PubMed PMID: CN-00552227.

7678. Chan LEJ, Soong JL, Lie SA. A cost avoidance study of critical care pharmacists' interventions in a tertiary institution in Singapore. American Journal of Health-System Pharmacy. 2023;80(5):267-83. doi: 10.1093/ajhp/zxac340. PubMed PMID: 162060415.

7679. Chan M, Plakogiannis R, Stefanidis A, Chen M, Saraon T. Pharmacist-Led Deprescribing for Patients With Polypharmacy and Chronic Disease States: A Retrospective Cohort Study. Journal of Pharmacy Practice. 2023;36(5):1192-200. doi: https://dx.doi.org/10.1177/08971900221097246. PubMed PMID: 2016468617.

7680. Chan NY, Choy CC, Chan CK, Siu CW. Effectiveness of a nongovernmental organization-led large-scale community atrial fibrillation screening program using the smartphone electrocardiogram: An observational cohort study. Heart Rhythm. 2018;15(9):1306-11. doi: 10.1016/j.hrthm.2018.06.006. PubMed PMID: WOS:000442908500007.

7681. Chan P, Tomlinson B, Chen YJ, Liu JC, Hsieh MH, Cheng JT. A double-blind placebo-controlled study of the effectiveness and tolerability of oral stevioside in human hypertension. British journal of clinical pharmacology. 2000;50(3):215-20.

7682. Chan S, Babcock L, Geis G, Frey M, Robinson V, Kerrey B. In Situ Simulation to Mitigate Threats to Participation in a Multicenter Clinical Trial in High-Acuity, Low-Frequency Setting. Simulation in healthcare. 2019;14(1):1-9. doi: 10.1097/SIH.0000000000000328. PubMed PMID: CN-01944060.

7683. Chan V, Cave AJ, Banh HL. Self-reported osteoporosis prevention in inhaled corticosteroid users in community pharmacy setting. SAGE Open Medicine. 2015;3. doi: 10.1177/2050312115586912.

7684. Chan V, Tran H. Purchasing Over-the-counter medicines from Australian pharmacy: What do the pharmacy customers value and expect? Pharmacy Practice. 2016;14(3). doi: 10.18549/PharmPract.2016.03.782.

7685. Chan W-JJ, McLachlan AJ, Wheate NJ, Harnett JE. An evaluation of garlic products available in Australian pharmacies–From the label to the laboratory. Journal of Herbal Medicine. 2018;14:61-7. doi: https://doi.org/10.1016/j.hermed.2018.09.004.

7686. Chan-Liston ML, Bourke JL, McWilliams R, Sheehan P, Chapman J, White K, et al. REMS pharmacy tasks: The adoption of an innovative electronic support system. Journal of the American Pharmacists Association. 2019;59(5):704-9. doi: https://doi.org/10.1016/j.japh.2019.04.020.

7687. Chana RC, Bradley H. Sociocultural, economic and regulatory influences on medicine use by consumers in a rural township in Cameroon. Southern Med Review. 2011;4(1):9-16. doi: 10.5655/smr.v4i1.73.

7688. Chanakit T. Thai stakeholder's perceptions of the introduction of the doctor of pharmacy programme [Ph.D.]. England: The University of Nottingham (United Kingdom); 2016.

7689. Chanakit T, Low BY, Wongpoowarak P, Moolasarn S, Anderson C. Hospital pharmacists' perceptions of the suitability of doctor of pharmacy graduates in hospital settings in Thailand. BMC Medical Education. 2015;15(1). doi: 10.1186/s12909-015-0471-6.

7690. Chanasopon S, Saramunee K, Rotjanawanitsalee T, Jitsanguansuk N, Chaiyasong S. Provision of primary care pharmacy operated by hospital pharmacist. Aims Public Health. 2023;10(2):268-80. doi: 10.3934/publichealth.2023020. PubMed PMID: WOS:000982413500001.

7691. Chancy P, Clifton CL, Branham AR, Hayes H, Moose JS, Rhodes LA, et al. Implementation of a

community pharmacy workflow process to identify and follow up with prescription abandonment. Journal of the American Pharmacists Association. 2019;59(4):S129-S35. doi: 10.1016/j.japh.2019.04.015. PubMed PMID: WOS:000475803700024.

7692. Chancy P, Clifton CL, Branham AR, Hayes H, Moose JS, Rhodes LA, et al. Implementation of a community pharmacy workflow process to identify and follow up with prescription abandonment. Journal of the American Pharmacists Association. 2019;59(4):S129-S35. doi: 10.1016/j.japh.2019.04.015.

7693. Chand S, Hiremath S, Shastry CS, Joel JJ, Krishna Bhat CH, Dikkatwar MS. Incidence and types of dispensing errors in the pharmacy of a tertiary care charitable hospital. Clinical Epidemiology and Global Health. 2022;18:101172. doi: https://doi.org/10.1016/j.cegh.2022.101172.

7694. Chand S, Shastry CS, Hiremath S, Joel JJ, Krishnabhat C. Hospital pharmacy management in a tertiary care charitable hospital: Compliance study with quality standards. Clinical Epidemiology and Global Health. 2022;17:101154. doi: https://doi.org/10.1016/j.cegh.2022.101154.

7695. Chanda MM, Ortblad KF, Mwale M, Chongo S, Kanchele C, Kamungoma N, et al. HIV self-testing among female sex workers in Zambia: a cluster randomized controlled trial. PLoS medicine. 2017;14(11):e1002442. doi: 10.1371/journal.pmed.1002442. PubMed PMID: CN-01443042.

7696. Chanda S, Sansgiry SS. Evaluation of facsimile cards for pharmacy-access products containing pseudoephedrine or ephedrine. Journal of the American Pharmacists Association. 2008;48(1):52-7. doi: 10.1331/JAPhA.2008.07009.

7697. Chandana KS, Sathvika K, Sultana SKS. ROBOTICS IN PHARMACY. International Journal of Life Science and Pharma Research. 2020:44-8. PubMed PMID: WOS:000553563000007.

7698. Chandler C, Barriuso P, RozenbergBenDror K, Schmitt B. Pharmacists on a primary care team at a Veterans Affairs Medical Center. American Journal of Health-System Pharmacy. 1997;54(11):1280-7. doi: 10.1093/ajhp/54.11.1280. PubMed PMID: WOS:A1997XC75700010.

7699. Chandler CIR, Hall-Clifford R, Asaph T, Pascal M, Clarke S, Mbonye AK. Introducing malaria rapid diagnostic tests at registered drug shops in Uganda: Limitations of diagnostic testing in the reality of diagnosis. Social Science and Medicine. 2011;72(6):937-44. doi: 10.1016/j.socscimed.2011.01.009.

7700. Chandra A, Cupps S. E-regulation and internet pharmacies: Issues and dilemmas. Clinical Research and Regulatory Affairs. 2002;19(1):67-81. doi: 10.1081/CRP-120004218.

7701. Chandra A, Young JS, Ore CD, Dayani F, Lau D, Wadhwa H, et al. Insurance type impacts the economic burden and survival of patients with newly diagnosed glioblastoma. Journal of Neurosurgery. 2020;133(1):89-99. doi: 10.3171/2019.3.JNS182629.

7702. Chandra T, Hafni L, Novita G. Pharmacy service quality for pharmacy customer satisfaction, trust, and loyalty. Systematic reviews in pharmacy. 2020;11(4):342-7. doi: 10.31838/SRP.2020.4.50. PubMed PMID: CN-02194518.

7703. Chandra-Mouli V, Lenz C, Adebayo E, Lang Lundgren I, Gomez Garbero L, Chatteriee S. A systematic review of the use of adolescent mystery clients in assessing the adolescent friendliness of health services in high, middle, and low-income countries. Global Health Action. 2018;11(1). doi: 10.1080/16549716.2018.1536412.

7704. Chandrakumar A, Ajmal KK, Suriyaprakash TNK, Anusree S. Clinical pharmacist assisted thromboprophylactic optimization in general surgical patients. Le Pharmacien Hospitalier et Clinicien.

2016;51(2):134-40. doi: https://doi.org/10.1016/j.phclin.2015.12.002.

7705. Chandramohan D, Jaffar S, Greenwood B. Use of clinical algorithms for diagnosing malaria 1. Tropical Medicine & International Health. 2002;7(1):45-52.

7706. Chandran A, Bonafede MK, Nigam S, Saltiel-Berzin R, Hirsch LJ, Lahue BJ. Adherence to Insulin Pen Therapy Is Associated with Reduction in Healthcare Costs Among Patients with Type 2 Diabetes Mellitus. American Health and Drug Benefits. 2015;8(3):148-57. PubMed PMID: WOS:000219724800007.

7707. Chandran A, Schaefer C, Ryan K, Baik R, McNett M, Zlateva G. The comparative economic burden of mild, moderate, and severe fibromyalgia: Results from a retrospective chart review and cross-sectional survey of working-age U.S. adults. Journal of Managed Care Pharmacy. 2012;18(6):415-26. doi: 10.18553/jmcp.2012.18.6.415.

7708. Chandrasekhar D, Joseph E, Ghaffoor FA, Thomas HM. Role of pharmacist led home medication review in community setting and the preparation of medication list. Clinical Epidemiology and Global Health. 2019;7(1):66-70. doi: https://doi.org/10.1016/j.cegh.2018.01.002.

7709. Chandy SJ, Mathai E, Thomas K, Faruqui AR, Holloway K, Lundborg CS, editors. Antibiotic use and resistance: perceptions and ethical challenges among doctors, pharmacists and the public in Vellore, South India. IJME FOURTH NATIONAL BIOETHICS CONFERENCE REPORT; 2013.

7710. Chandy SJ, Thomas K, Mathai E, Antonisamy B, Holloway KA, Lundborg CS. Patterns of antibiotic use in the community and challenges of antibiotic surveillance in a lower-middle-income country setting: a repeated cross-sectional study in Vellore, south India. Journal of Antimicrobial Chemotherapy. 2013;68(1):229-36. doi: 10.1093/jac/dks355. PubMed PMID: WOS:000312646300036.

7711. Chandy SJ, Thomas K, Mathai E, Antonisamy B, Holloway KA, Stalsby Lundborg C. Patterns of antibiotic use in the community and challenges of antibiotic surveillance in a lower-middle-income country setting: A repeated cross-sectional study in Vellore, South India. Journal of Antimicrobial Chemotherapy. 2013;68(1):229-36. doi: 10.1093/jac/dks355.

7712. Chanfreau-Coffinier C, Hull LE, Lynch JA, DuVall SL, Damrauer SM, Cunningham FE, et al. Projected Prevalence of Actionable Pharmacogenetic Variants and Level A Drugs Prescribed Among US Veterans Health Administration Pharmacy Users. Jama Network Open. 2019;2(6). doi: 10.1001/jamanetworkopen.2019.5345. PubMed PMID: WOS:000476810700013.

7713. Chang AC, Lincoln J, Lantaff WM, Gernant SA, Jaynes HA, Doucette W, et al. Characterization of actions taken during the delivery of medication therapy management: A time-and-motion approach. Journal of the American Pharmacists Association: JAPhA. 2018;58(1):61-6.e7. PubMed PMID: 29129668.

7714. Chang AK, Bijur P, Barnaby D, Gallagher EJ. A randomized controlled trial of four oral analgesics in the treatment of acute extremity pain. Academic emergency medicine. 2017;24:S21-S2. doi: 10.1111/acem.13203. PubMed PMID: CN-01376353.

7715. Chang AR, Evans M, Yule C, Bohn L, Young A, Lewis M, et al. Using pharmacists to improve risk stratification and management of stage 3A chronic kidney disease: a feasibility study. BMC nephrology. 2016;17(1):168. doi: 10.1186/s12882-016-0383-7. PubMed PMID: CN-01424704.

7716. Chang AY, Tonui EC, Momanyi D, Mills AR, Wasike P, Karwa R, et al. Development of Low-Cost Locally Sourced Two-Component Compression Bandages in Western Kenya. Dermatology and Therapy. 2018;8(3):475-81. doi: 10.1007/s13555-018-0248-z.

7717. Chang C, Schiano T. Drug hepatotoxicity. Alimentary pharmacology & therapeutics. 2007;25(10):1135-51.

7718. Chang CH, Chen HC, Lin JW, Kuo CW, Shau WY, Lai MS. Risk of hospitalization for upper gastrointestinal adverse events associated with nonsteroidal anti-inflammatory drugs: a nationwide case-crossover study in Taiwan. Pharmacoepidemiology and Drug Safety. 2011;20(7):763-71. doi: 10.1002/pds.2140. PubMed PMID: WOS:000293374600012.

7719. Chang CT, Shariff SMM, Abu Bakar NS, Ramzuzzaman NS, Lim CK, Lim EYJ, et al. Polypharmacy and potentially inappropriate medications among hospitalized older adults with COVID-19 in Malaysian tertiary hospitals. Journal of Pharmaceutical Policy and Practice. 2023;16(1). doi: 10.1186/s40545-022-00504-1. PubMed PMID: WOS:000913520700001.

7720. Chang CT, Shariff SMM, Abu Bakar NS, Ramzuzzaman NS, Lim CK, Lim EYJ, et al. Polypharmacy and potentially inappropriate medications among hospitalized older adults with COVID-19 in Malaysian tertiary hospitals. Journal of Pharmaceutical Policy and Practice. 2023;16(1). doi: 10.1186/s40545-022-00504-1. PubMed PMID: WOS:000913520700001.

7721. Chang CT, Teoh SL, Cheah WK, Lee PJ, Azman MA, Ling SH, et al. Impact of deprescribing intervention on potentially inappropriate medications and clinical outcomes among hospitalized older adults in Malaysia: a randomized controlled trial (REVMED RCT) protocol. Journal of Pharmaceutical Policy and Practice. 2023;16(1):113. doi: https://dx.doi.org/10.1186/s40545-023-00621-5. PubMed PMID: 2025856865.

7722. Chang CT, Teoh SL, Rajan P, Lee SWH. Explicit potentially inappropriate medications criteria for older population in Asian countries: a systematic review. Research in Social and Administrative Pharmacy. 2023;19(8):1146-56.

7723. Chang CW, Chu G, Hinz BJ, Greve MDJ. Current use of dietary supplementation in patients with age-related macular degeneration. Canadian Journal of Ophthalmology. 2003;38(1):27-32. doi: 10.1016/S0008-4182(03)80005-4.

7724. Chang CY, Park H, Hincapie-Castillo JM, Heldermon CD, Diaby V, Yang S, et al. Trends, characteristics, race, and ethnicity associated with nonadherence to antidepressants among breast cancer survivors with depression. Journal of Managed Care and Specialty Pharmacy. 2023;29(4):431-45. doi: https://dx.doi.org/10.18553/jmcp.2023.29.4.431. PubMed PMID: 2024127901.

7725. Chang E, Tsang B, Thornley S. Language barriers in the community pharmacy: a survey of northern and western Auckland. Journal of Primary Health Care. 2011;3(2):102-6. PubMed PMID: 104804353. Language: English. Entry Date: 20110819. Revision Date: 20150711. Publication Type: Journal Article.

7726. Chang EH, Doucette WR, Carter BL, Ardery G, Egan BM, James PA. Variable BP control following a physician\pharmacist model for BP: effect of diabetes, chronic kidney disease and racial minorities. Journal of the american society of hypertension. 2014;8(4 SUPPL. 1):e20. doi: 10.1016/j.jash.2014.03.036. PubMed PMID: CN-01042347.

7727. Chang F, Fonseca J, Chang A. Perceived barriers and facilitators to providing methadone maintenance treatment among rural community pharmacists in southwestern Ontario. Canadian pharmacists journal Conference: canadian pharmacists association conference, cpha 2017 Canada. 2017;150(4):S68. doi: 10.1177/1715163517719855. PubMed PMID: CN-01408084.

7728. Chang F, Gupta N. Progress in electronic medical record adoption in Canada. Canadian Family Physician. 2015;61(12):1076-84.

7729. Chang F, O'Connell MB, Mills ME, Hwang JM, Khreizat HS, Garwood CL, et al. Preventive health therapy and behavior outcomes from a brown bag medication review for older adults. Journal of the American Geriatrics Society. 2022;70(11):3202-9. doi: 10.1111/jgs.17982. PubMed PMID: WOS:000833041900001.

7730. Chang F, O'Hare AM, Miao Y, Steinman MA. Use of Renally Inappropriate Medications in Older Veterans: A National Study. Journal of the American Geriatrics Society. 2015;63(11):2290-7. doi: 10.1111/jgs.13790. PubMed PMID: 111218916. Language: English. Entry Date: 20151203. Revision Date: 20191029. Publication Type: Article.

7731. Chang F, Patel T, Schulz ME. The "Rising Tide" of dementia in Canada: What does it mean for pharmacists and the people they care for? Canadian Pharmacists Journal. 2015;148(4):193-9. doi: 10.1177/1715163515588107.

7732. Chang FC, Chi HY, Huang LJ, Lee CH, Yang JL, Yeh MK. Developing school-pharmacist partnerships to enhance correct medication use and pain medication literacy in Taiwan. Journal of the American Pharmacists Association. 2015;55(6):595-602. doi: 10.1331/JAPhA.2015.15053.

7733. Chang H-Y, Kitchen C, Bishop MA, Shermock KM, Gudzune KA, Kharrazi H, et al. Claims-based pharmacy markers for comprehensive medication management program case identification: Validation against concurrent and prospective healthcare costs and utilization. Research in Social and Administrative Pharmacy. 2022;18(10):3800-13. doi: https://doi.org/10.1016/j.sapharm.2022.04.006.

7734. Chang H-Y, Krawczyk N, Schneider KE, Ferris L, Eisenberg M, Richards TM, et al. A predictive risk model for nonfatal opioid overdose in a statewide population of buprenorphine patients. Drug and Alcohol Dependence. 2019;201:127-33. doi: https://doi.org/10.1016/j.drugalcdep.2019.04.016.

7735. Chang HE, Manojlovich M. Clinical nurses' patient safety competency, systems thinking and missed nursing care: A cross-sectional survey. International Journal of Nursing Practice (John Wiley & Sons, Inc). 2023;29(2):1-9. doi: 10.1111/ijn.13130. PubMed PMID: 162878111.

7736. Chang HY, Richards TM, Clark JM, Weiner JP, Segal JB. Change in claims-based diabetes medications is a diabetes improvement indicator. American Journal of Managed Care. 2013;19(7):572-8.

7737. Chang J, Lv B, Zhu S, Yu J, Zhang Y, Ye D, et al. Non-prescription use of antibiotics among children in urban China: a cross-sectional survey of knowledge, attitudes, and practices. Expert Review of Anti-Infective Therapy. 2018;16(2):163-72. doi: 10.1080/14787210.2018.1425616.

7738. Chang J, Lv B, Zhu S, Yu JL, Zhang Y, Ye D, et al. Non-prescription use of antibiotics among children in urban China: a cross-sectional survey of knowledge, attitudes, and practices. Expert Review of Anti-Infective Therapy. 2018;16(2):163-72. doi: 10.1080/14787210.2018.1425616. PubMed PMID: WOS:000423476000008.

7739. Chang J, Mathes DW. Ethical, Financial, and Policy Considerations in Hand Transplantation. Hand Clinics. 2011;27(4):553-60. doi: https://doi.org/10.1016/j.hcl.2011.07.006.

7740. Chang J, Wang Q, Fang Y. Socioeconomic differences in self-medication among middle-aged and older people: data from the China health and retirement longitudinal study. BMJ Open. 2017;7(12):e017306. PubMed PMID: 29259056.

7741. Chang J, Xu S, Zhu S, Li Z, Yu J, Zhang Y, et al. Assessment of non-prescription antibiotic dispensing at community pharmacies in China with simulated clients: a mixed cross-sectional and longitudinal study. Lancet Infectious Diseases. 2019;19(12):1345-54. doi: 10.1016/S1473-3099(19)30324-X. PubMed PMID:

141610876. Language: English. Entry Date: 20200530. Revision Date: 20200621. Publication Type: journal article.

7742. Chang J, Ye D, Lv B, Jiang M, Zhu S, Yan K, et al. Sale of antibiotics without a prescription at community pharmacies in urban China: A multicentre cross-sectional survey. Journal of Antimicrobial Chemotherapy. 2017;72(4):1235-42. doi: 10.1093/jac/dkw519.

7743. Chang J, Ye D, Lv B, Jiang MH, Zhu S, Yan KK, et al. Sale of antibiotics without a prescription at community pharmacies in urban China: a multicentre cross-sectional survey. Journal of Antimicrobial Chemotherapy. 2017;72(4):1235-42. doi: 10.1093/jac/dkw519. PubMed PMID: WOS:000402070800040.

7744. Chang J, Zhao Y, Zhao W, Venkataramanan R, Caritis SN. Quality assessment of compounded 17-hydroxyprogesterone caproate. American Journal of Obstetrics and Gynecology. 2014;210(1):47.e1-.e7. doi: https://doi.org/10.1016/j.ajog.2013.09.039.

7745. Chang LC, Guo JL, Lin HL. Cultural competence education for health professionals from pre-graduation to licensure delivered using facebook: twelve-month follow-up on a randomized control trial. Nurse education today. 2017;59:94-100. doi: 10.1016/j.nedt.2017.09.005. PubMed PMID: CN-01426051.

7746. Chang N-CN, Chanfreau-Coffinier C, Bates J, Tuteja S, Anglin TR, Moore VR, et al. Pharmacogenetic testing and monitoring of complete blood counts among Veterans newly prescribed thiopurine treatments: a retrospective cohort study. Journal of Pharmaceutical Policy & Practice. 2023;16(1):1-7. doi: 10.1186/s40545-023-00657-7. PubMed PMID: 174163677.

7747. Chang TE, Ritchey MD, Park S, Chang A, Odom EC, Durthaler J, et al. National rates of nonadherence to antihypertensive medications among insured adults with hypertension, 2015. Hypertension. 2019;74(6):1324-32. doi: 10.1161/HYPERTENSIONAHA.119.13616.

7748. Chang TI, Desai M, Solomon DH, Winkelmayer WC. Kidney Function and Long-Term Medication Adherence after Myocardial Infarction in the Elderly. Clinical Journal of the American Society of Nephrology. 2011;6(4):864-9. doi: 10.2215/cjn.07290810. PubMed PMID: WOS:000289223600025.

7749. Chang TY, Jacobson M. Going to pot? The impact of dispensary closures on crime. Journal of Urban Economics. 2017;100:120-36. doi: 10.1016/j.jue.2017.04.001. PubMed PMID: WOS:000403629100008.

7750. Chang W, Matambanadzo P, Takaruza A, Hatzold K, Cowan FM, Sibanda E, et al. Effect of Prices, Distribution Strategies, and Marketing on Demand for HIV Self-testing in Zimbabwe: a Randomized Clinical Trial. JAMA network open. 2019;2(8):e199818. doi: 10.1001/jamanetworkopen.2019.9818. PubMed PMID: CN-01990579.

7751. Chang WJ, Li GF, Hu WL, editors. Convenient Community Service System Based on Integrated Service Platform. 6th International Conference on Electronic, Mechanical, Information and Management Society (EMIM); 2016 Apr 01-03; Shenyang, PEOPLES R CHINA2016.

7752. Chang YK, Mark BA. Antecedents of severe and nonsevere medication errors. Journal of Nursing Scholarship. 2009;41(1):70-8.

7753. Chang ZG, Kennedy DT, Holdford DA, Small RE. Pharmacists' knowledge and attitudes toward herbal medicine. Annals of Pharmacotherapy. 2000;34(6):710-5. doi: 10.1345/aph.19263.

7754. Chang ZG, Kennedy DT, Holdford DA, Small RE. Pharmacists' Knowledge and Attitudes Toward Herbal Medicine. Annals of Pharmacotherapy. 2007;41(7-8):1272-6. doi: 10.1345/aph.140062.

7755. Chang ZG, Kennedy DT, Holdford DA, Small RE. Pharmacists' knowledge and attitudes toward herbal medicine (Reprinted from Ann Pharmacother, vol 34, pg 710-5, 2000). Annals of Pharmacotherapy. 2007;41(7-8):1272-6. doi: 10.1345/aph.140062. PubMed PMID: WOS:000248279100025.

7756. Chant C, Dewhurst NF, Friedrich JO. Do we need a pharmacist in the ICU? Intensive Care Medicine. 2015;41(7):1314-20. PubMed PMID: 26077045.

7757. Chantelois EP, Suzuki NT. A pilot program comparing physician- and pharmacist-ordered discharge medications at a Veterans Affairs medical center. American Journal of Health-System Pharmacy. 2003;60(16):1652-6. PubMed PMID: 12966909.

7758. Chanvatik S, Donnua S, Lekagul A, Kaewkhankhaeng W, Vongmongkol V, Athipunyakom P, et al. Antibiotic use in Mandarin production (Citrus reticulata Blanco) in major Mandarin-producing areas in Thailand: A survey assessment. PLoS ONE. 2019;14(11). doi: 10.1371/journal.pone.0225172.

7759. Chao C-C, Yang J-M, Jen W-Y. Determining technology trends and forecasts of RFID by a historical review and bibliometric analysis from 1991 to 2005. Technovation. 2007;27(5):268-79.

7760. Chapet N, Mathieu B, Audurier Y, De Barry G, Jalabert A, Renaudin P, et al. Evaluation of clinical pharmacists' interventions in a cardiology department. European journal of hospital pharmacy. 2017;24:A252-A3. doi: 10.1136/ejhpharm-2017-000640.564. PubMed PMID: CN-01406501.

7761. Chaplin B, Meloni S, Eisen G, Jolayemi T, Banigbe B, Adeola J, et al. Scale-up of networked HIV treatment in Nigeria: creation of an integrated electronic medical records system. International journal of medical informatics. 2015;84(1):58-68.

7762. Chaplin G. Monitoring fluidized bed dryer hydrodynamics using pressure fluctuations and electrical capacitance tomography [Ph.D.]. Canada -- Saskatchewan, CA: The University of Saskatchewan (Canada); 2005.

7763. Chaplin S. Monitoring drug safety: is the Yellow Card Scheme struggling? Prescriber. 2019;30(9):32-4. doi: 10.1002/psb.1789. PubMed PMID: 139252160. Language: English. Entry Date: 20191102. Revision Date: 20200831. Publication Type: Article.

7764. Chapman RH, Liu LZ, Girase PG, Straka RJ. Determining initial and follow-up costs of cardiovascular events in a US managed care population. Bmc Cardiovascular Disorders. 2011;11. doi: 10.1186/1471-2261-11-11. PubMed PMID: WOS:000289206200001.

7765. Chapman S, Jafa K, Longfield K, Vielot N, Buszin J, Ngamkitpaiboon L, et al. Condom social marketing in sub-Saharan Africa and the Total Market Approach. Sexual Health. 2012;9(1):44-50. doi: 10.1071/SH10165.

7766. Chapman-Goetz J, Packham N, Gabb G, Potts C, Yu K, Prosser A, et al. Acceptability and feasibility of the NPS MedicineWise mobile phone application in supporting medication adherence in patients with chronic heart failure: protocol for a pilot study. PloS one. 2022;17(2):e0263284. doi: 10.1371/journal.pone.0263284. PubMed PMID: CN-02363508.

7767. Chappard P. La substitution aux opiacés : le point de vue des usagers. Annales Pharmaceutiques Françaises. 2009;67(5):365-8. doi: https://doi.org/10.1016/j.pharma.2009.06.001.

7768. Chappe M, Corvaisier M, Brangier A, Annweiler C, Spiesser-Robelet L. Impact of the COVID-19 pandemic on drug-related problems and pharmacist interventions in geriatric acute care units. Annales Pharmaceutiques Françaises. 2022;80(5):669-77. doi: https://doi.org/10.1016/j.pharma.2021.12.006.

7769. Chapple A, Hassell K, Nicolson M, Cantrill J. 'You don't really feel you can function normally': women's perceptions and personal management of vaginal thrush. Journal of Reproductive and Infant Psychology. 2000;18(4):309-19. doi: 10.1080/713683045. PubMed PMID: WOS:000165331600004.

7770. Chappuy M, Garcia S, Uhres AC, Janoly-Dumenil A, Dessault J, Chamouard V, et al. Interventions des pharmaciens sur les prescriptions ambulatoires dans le cadre de l'activité de rétrocession d'un centre hospitalier universitaire. Annales Pharmaceutiques Françaises. 2015;73(4):297-306. doi: https://doi.org/10.1016/j.pharma.2014.11.001.

7771. Chappuy M, Garcia S, Uhres AC, Janoly-Dumenil A, Dessault J, Chamouard V, et al. [Pharmacist's interventions on outpatient prescriptions in a university hospital drugs sales service]. Annales Pharmaceutiques Francaises. 2015;73(4):297-306. PubMed PMID: 25558801.

7772. Chapuis C, Chanoine S, Colombet L, Calvino-Gunther S, Tournegros C, Terzi N, et al. Interprofessional safety reporting and review of adverse events and medication errors in critical care. Therapeutics & Clinical Risk Management. 2019;15:549-56. doi: 10.2147/TCRM.S188185. PubMed PMID: 136204187. Language: English. Entry Date: In Process. Revision Date: 20220410. Publication Type: journal article. Journal Subset: Europe.

7773. Chapuis C, Collomp R, Albaladejo L, Terrisse H, Honoré S, Bosson JL, et al. Redistribution of critical drugs in shortage during the first wave of COVID-19 in France: from operating theaters to intensive care units. Journal of Pharmaceutical Policy and Practice. 2022;15(1). doi: 10.1186/s40545-022-00425-z.

7774. Char CWT, Yip AYF, Kee KW, Lee ES, Chua AHL. Effectiveness of pre-consultation medication reconciliation service in reducing medication discrepancies during transition of care from hospital discharge to primary care setting in singapore - A randomised controlled trial. Journal of applied pharmacy. 2017;9(4). doi: 10.21065/1920-4159.1000255. PubMed PMID: CN-01432233.

7775. Charan J, Biswas T. How to calculate sample size for different study designs in medical research? Indian journal of psychological medicine. 2013;35(2):121-6.

7776. Charboneau AL, Shelton PS, Brickley JB, Rich W. Medication errors and penalties in assisted living facilities. Consultant Pharmacist. 2011;26(8):554-65. doi: 10.4140/TCP.n.2011.554. PubMed PMID: 116538213. Language: English. Entry Date: 20160702. Revision Date: 20180711. Publication Type: journal article.

7777. Chareh N. Nutritional Problems and Nutritional Care in Community-Dwelling Older Adults2023.

7778. Chargualaf MJ, Giao TT, Abrahamson AC, Steeb D, Law M, Bates J, et al. Layered learning pharmacy practice model in Ethiopia. Journal of Oncology Pharmacy Practice. 2019;25(7):1699-704. doi: 10.1177/1078155218820105. PubMed PMID: WOS:000482977300022.

7779. Charles F, Castet-Nicolas A, Amouroux C, Moreau J, Werner O, Morin D, et al. Evaluation of the impact of pharmaceutical trainings and tools on the proper use of medicines in pediatrics. Frontiers in Pharmacology. 2023;14:1143974. doi: https://dx.doi.org/10.3389/fphar.2023.1143974. PubMed PMID: 2023127003.

7780. Charlton ME, Levy BT, High RR, Schneider JE, Brooks JM. Effects of Health Savings Account-Eligible Plans on Utilization and Expenditures. American Journal of Managed Care. 2011;17(1):79-86. PubMed PMID: WOS:000286871300007.

7781. Charoo N, Cristofoletti R, Graham A, Lartey P, Abrahamsson B, Groot DW, et al. Biowaiver Monograph for Immediate-Release Solid Oral Dosage Forms: Fluconazole. Journal of Pharmaceutical

Sciences. 2014;103(12):3843-58. doi: https://doi.org/10.1002/jps.24181.

7782. Charoo NA, Cristofoletti R, Khatri AR, Ali AA. Significance of Metabolites in Bioequivalence: Losartan Potassium as a Case Study. Journal of Pharmaceutical Sciences. 2014;103(6):1584-91. doi: https://doi.org/10.1002/jps.23965.

7783. Charoo NA, Shamsher AAA, Lian LY, Abrahamsson B, Cristofoletti R, Groot DW, et al. Biowaiver Monograph for Immediate-Release Solid Oral Dosage Forms: Bisoprolol Fumarate. Journal of Pharmaceutical Sciences. 2014;103(2):378-91. doi: https://doi.org/10.1002/jps.23817.

7784. Charpentier B, François H, Beaudreuil S, Dürrbach A. Necessary but sometimes complicated coordination of healthcare procedures. The example of chronic renal failure. Bulletin de l'Academie Nationale de Medecine. 2013;197(8):1523-30. doi: 10.1016/s0001-4079(19)31199-9.

7785. Charpentier Dr MM, Orr KK, Taveira TH. Improving pharmacist knowledge of oral chemotherapy in the community. Annals of Pharmacotherapy. 2012;46(9):1205-11. doi: 10.1345/aph.1R056.

7786. Charpentier MM, Orr KK, Taveira TH. Improving pharmacist knowledge of oral chemotherapy in the community. Annals of Pharmacotherapy. 2012;46(9):1205-11.

7787. Charpiat B, Bedouch P, Conort O, Rose FX, Juste M, Roubille R, et al. Opportunités d'erreurs médicamenteuses et interventions pharmaceutiques dans le cadre de la prescription informatisée : revue des données publiées par les pharmaciens hospitaliers français. Annales Pharmaceutiques Françaises. 2012;70(2):62-74. doi: https://doi.org/10.1016/j.pharma.2012.02.002.

7788. Charpiat B, Bedouch P, Conort O, Rose FX, Juste M, Roubille R, et al. [Opportunities for medication errors and pharmacist's interventions in the context of computerized prescription order entry: a review of data published by French hospital pharmacists]. Annales Pharmaceutiques Francaises. 2012;70(2):62-74. PubMed PMID: 22500957.

7789. Charpiat B, Bedouch P, Rose FX, Juste M, Roubille R, Conort O, et al. Overdosed paracetamol (acetaminophen) prescriptions and subsequent pharmacist interventions in French hospitals. Annales Pharmaceutiques Françaises. 2013;71(6):410-7. doi: https://doi.org/10.1016/j.pharma.2013.08.010.

7790. Charpiat B, Bedouch P, Tod M, Allenet B. Classifying pharmacists' interventions recorded in observational databases: Are they all necessary and appropriate? Research in Social and Administrative Pharmacy. 2017;13(6):1184-5. doi: https://doi.org/10.1016/j.sapharm.2016.10.020.

7791. Charpiat B, Conort O, Juste M, Rose FX, Roubille R, Bedouch P, et al. Mediator® : analyse du contenu de la base ACT-IP de la Société française de pharmacie clinique. Le Pharmacien Hospitalier et Clinicien. 2014;49(3):192-6. doi: https://doi.org/10.1016/j.phclin.2013.10.073.

7792. Charpiat B, Conort O, Juste M, Rose FX, Roubille R, Bedouch P, et al. The French Society of Clinical Pharmacy ACT-IP© project: Ten years onward, results and prospects. Pharmacien Hospitalier et Clinicien. 2015;50(2):15-24. doi: 10.1016/j.phclin.2014.05.004.

7793. Charpiat B, Conort O, Juste M, Rose FX, Roubille R, Bedouch P, et al. Provision of the instrument of the French Society of Clinical Pharmacy for the documentation of clinical pharmacists' interventions: 10 years after and perspectives. Pharmacien Hospitalier et Clinicien. 2015;50(1):125-35. doi: 10.1016/j.phclin.2014.05.004.

7794. Charpiat B, Derfoufi S, Larger M, Janoly-Dumenil A, Mouchoux C, Allenet B, et al. Identification des lacunes de connaissances des étudiants en filière officine débutant le stage de 5e année : propositions pour

modifier le contenu de l'enseignement. Annales Pharmaceutiques Françaises. 2016;74(5):404-12. doi: https://doi.org/10.1016/j.pharma.2016.01.001.

7795. Charpiat B, Derfoufi S, Larger M, Janoly-Dumenil A, Mouchoux C, Allenet B, et al. Identification of knowledge deficits of pharmacy students at the beginning of the fifth year of pharmacy practice experience: Proposals to change the content of academic programs. Annales Pharmaceutiques Francaises. 2016;74(5):404-12. doi: 10.1016/j.pharma.2016.01.001.

7796. Charpiat B, Goutelle S, Schoeffler M, Aubrun F, Viale JP, Ducerf C, et al. Prescriptions analysis by clinical pharmacists in the post-operative period: a 4-year prospective study. Acta Anaesthesiologica Scandinavica. 2012;56(8):1047-51. PubMed PMID: 22289072.

7797. Charpiat B, Henry A, Leboucher G, Tod M, Allenet B. Overdosed prescription of paracetamol (acetaminophen) in a teaching hospital. Annales Pharmaceutiques Françaises. 2012;70(4):213-8. doi: https://doi.org/10.1016/j.pharma.2012.05.002.

7798. Charpiat B, Mille F, Fombeur P, Machon J, Zawadzki E, Bobay-Madic A. Problèmes rencontrés par les pharmaciens des établissements de santé avec les systèmes d'information hospitaliers : analyse des échanges opérés au sein de réseaux sociaux. Annales Pharmaceutiques Françaises. 2018;76(5):368-81. doi: https://doi.org/10.1016/j.pharma.2018.03.007.

7799. Charriot J, Descamps V, Jankowski R, Maravic M, Bourdin A. Multiple Biologics for Multiple T2 Diseases: A Pharmacoepidemiological Algorithm for Sorting Out Patients by Indication. Journal of Asthma and Allergy. 2023;16:1287-95. doi: 10.2147/jaa.S424152. PubMed PMID: WOS:001113283300001.

7800. Charrois T, Newman S, Sin D, Senthilselvan A, Tsuyuki RT. Improving asthma symptom control in rural communities: the design of the Better Respiratory Education and Asthma Treatment in Hinton and Edson study. Controlled clinical trials. 2004;25(5):502-14. doi: 10.1016/j.cct.2004.07.004. PubMed PMID: CN-00496649.

7801. Charrois TL, Hill RL, Vu D, Foster BC, Boon HS, Cramer K, et al. Community identification of natural health product-drug interactions. Annals of Pharmacotherapy. 2007;41(7-8):1124-9. doi: 10.1345/aph.1H463.

7802. Charrois TL, McAlister FA, Cooney D, Lewanczuk R, Kolber MR, Campbell NR, et al. Improving hypertension management through pharmacist prescribing; the rural Alberta clinical trial in optimizing hypertension (Rural RxACTION): trial design and methods. Implementation science. 2011;6:94. doi: 10.1186/1748-5908-6-94. PubMed PMID: CN-00860784.

7803. Charrois TL, Newman SC, Senthilselvan A, Tsuyuki RT. Improving asthma control in the rural setting: the BREATHE (Better Respiratory Education and Asthma Treatment in Hinton and Edson) study. Canadian pharmacists journal. 2006;139(4):44-50. PubMed PMID: CN-00613563.

7804. Charrois TL, Sewell HD. Clinical decision-making by fourth-year pharmacy students: Towards an understanding of their uncertainty. Currents in Pharmacy Teaching and Learning. 2023;15(9):779-86. doi: https://doi.org/10.1016/j.cptl.2023.07.020.

7805. Charron A, Diamantouros A, Azzam M, Lane S, Sapru H, Verhovsek MM. Pharmacy Hydroxyurea Education Materials for Patients with Sickle Cell Disease: An Environmental Scan and Assessment of Accuracy. Blood. 2017;130:4666. doi: https://doi.org/10.1182/blood.V130.Suppl_1.4666.4666.

7806. Charron E, Okifuji A, Bryan MA, Reese S, Brown JL, Ferguson A, et al. Pain Severity and Interference and Substance Use Among Community Pharmacy Patients Prescribed Opioids: A Secondary Analysis of the

PHARMSCREEN Study. The Journal of Pain. 2022;23(8):1448-59. doi: https://doi.org/10.1016/j.jpain.2022.03.238.

7807. Chartrand M, Guenette L, Brouillette D, Cote S, Huot R, Landry J, et al. Development of Quality Indicators to Assess Oral Anticoagulant Management in Community Pharmacies for Patients with Atrial Fibrillation. Journal of Managed Care & Specialty Pharmacy. 2018;24(4):357-65. PubMed PMID: 29578847.

7808. Chartrand M, Lalonde L, Cantin A, Lahaie A, Odobasic B, Tremblay MP, et al. Anticoagulation management services in community pharmacy: Feasibility of implementing a quality improvement programme through a practice-based research network. Journal of Clinical Pharmacy and Therapeutics. 2018;43(6):877-87. doi: 10.1111/jcpt.12745.

7809. Charuel A, Prevost V. Nutritional counseling in community pharmacies within the framework of the French National Nutrition and Health Program. Annales Pharmaceutiques Francaises. 2014;72(5):337-47. doi: 10.1016/j.pharma.2014.03.003.

7810. Charurat M, Oyegunle M, Benjamin R, Habib A, Eze E, Ele P, et al. Patient retention and adherence to antiretrovirals in a large antiretroviral therapy program in Nigeria: A longitudinal analysis for risk factors. PLoS ONE. 2010;5(5). doi: 10.1371/journal.pone.0010584.

7811. Chase AM, Azimi HA, Forehand CC, Keats K, Taylor A, Wu S, et al. An Evaluation of the Relationship Between Medication Regimen Complexity as Measured by the MRC-ICU to Medication Errors in Critically Ill Patients. Hospital Pharmacy. 2023;58(6):569-74. doi: 10.1177/00185787231170386. PubMed PMID: 173550622.

7812. Chasqueira ICB. Medicamentos Contrafeitos – Revisão Sobre a Venda de Medicamentos Contrafeitos na Internet: Experiência Profissionalizante na Vertente de Farmácia Comunitária e Investigação [M.Sc.]. Portugal: Universidade da Beira Interior (Portugal); 2018.

7813. Chasse MA. Ensuring safe and efficient communication of medication prescriptions in community and ambulatory settings. Alberta RN. 2008;64(1):11-2. PubMed PMID: 18286911.

7814. Chassin MR, Becher EC. The wrong patient. Annals of internal medicine. 2002;136(11):826-33.

7815. Chast F. L'histoire des Annales pharmaceutiques françaises. 1809–2009. Annales Pharmaceutiques Françaises. 2009;67(6):380-90. doi: https://doi.org/10.1016/j.pharma.2009.08.001.

7816. Chastain DB, Ladak A, Curtis J, Tang E, Young HN. Evaluation and predictors of antiretroviral (ART)-related medication errors in hospitalized people living with HIV (PWLH). Open forum infectious diseases. 2019;6:S471-. doi: 10.1093/ofid/ofz360.1169. PubMed PMID: CN-02073074.

7817. Chastang A, Renet S, Corny J, Beaussier H, Petre A, Lillo-Lelouet A, et al. Impact of hospital pharmacist interventions on the combination of citalopram or escitalopram with other QT-prolonging drugs. International Journal of Clinical Pharmacy. 2019;41(1):42-8. PubMed PMID: 30610545.

7818. Chater RW, Moczygemba LR, Lawson KA, Barner JC, Patterson B, Reed B, et al. Building the business model for medication therapy management services. Journal of the American Pharmacists Association: JAPhA. 2008;48(1):16-22. PubMed PMID: 18192126.

7819. Chater RW, Moczygemba LR, Lawson KA, Barrier JC, Patterson B, Reed B, et al. Building the business model for medication therapy management services. Journal of the American Pharmacists Association. 2008;48(1):16-22. doi: 10.1331/JAPhA.2008.08500. PubMed PMID: WOS:000253164700004.

7820. Chatha ZF, Rashid U, Olsen S, Din FU, Khan A, Nawaz K, et al. Pharmacist-led counselling intervention to improve antiretroviral drug adherence in Pakistan: a randomized controlled trial. BMC infectious diseases. 2020;20(1):874. doi: 10.1186/s12879-020-05571-w. PubMed PMID: CN-02200898.

7821. Chatsisvili A, Sapounidis I, Pavlidou G, Zoumpouridou E, Karakousis V-A, Spanakis M, et al. Potential drug–drug interactions in prescriptions dispensed in community pharmacies in Greece. Pharmacy world & science. 2010;32:187-93.

7822. Chatsisvili A, Sapounidis I, Pavlidou G, Zoumpouridou E, Karakousis VA, Spanakis M, et al. Potential drug-drug interactions in prescriptions dispensed in community pharmacies in Greece. Pharmacy World and Science. 2010;32(2):187-93. doi: 10.1007/s11096-010-9365-1.

7823. Chatterjee A. Vaccinophobia and vaccine controversies of the 21st century2013. 1-497 p.

7824. Chatterjee A, Baker T, Rudorf M, Walt G, Stotz C, Martin A, et al. Mobile treatment for opioid use disorder: Implementation of community-based, same-day medication access interventions. Journal of Substance Use & Addiction Treatment. 2024;159. doi: 10.1016/j.josat.2023.209272. PubMed PMID: WOS:001143923600001.

7825. Chatterjee A, Yan S, Lambert A, Morgan JR, Green TC, Jeng PJ, et al. Comparison of a national commercial pharmacy naloxone data source to state and city pharmacy naloxone data sources—Rhode Island, Massachusetts, and New York City, 2013–2019. Health Services Research. 2023;58(5):1141-50. doi: 10.1111/1475-6773.14200.

7826. Chatterjee A, Yan S, Xuan Z, Waye KM, Lambert AM, Green TC, et al. Broadening access to naloxone: Community predictors of standing order naloxone distribution in Massachusetts. Drug and Alcohol Dependence. 2022;230:109190. doi: https://doi.org/10.1016/j.drugalcdep.2021.109190.

7827. Chatterjee A, Yan SP, Lambert A, Morgan JR, Green TC, Jeng PJ, et al. Comparison of a national commercial pharmacy naloxone data source to state and city pharmacy naloxone data sources-Rhode Island, Massachusetts, and New York City, 2013-2019. Health Services Research. 2023;58(5):1141-50. doi: 10.1111/1475-6773.14200. PubMed PMID: WOS:001023652600001.

7828. Chatterjee A, Yan SP, Xuan ZM, Waye KM, Lambert AM, Green TC, et al. Broadening access to naloxone: Community predictors of standing order naloxone distribution in Massachusetts. Drug and Alcohol Dependence. 2022;230. doi: 10.1016/j.drugalcdep.2021.109190. PubMed PMID: WOS:000726987600004.

7829. Chatterjee B. The secure pharmaceutical supply chain: Where does RFID technology fit in and should you be considering it? Pharmaceutical Processing. 2009;24(6):14-6.

7830. Chatterjee C, Mohapatra DP, Estay M. From courts to markets: New evidence on enforcement of pharmaceutical bans in India. Social Science & Medicine. 2019;237:112480. doi: https://doi.org/10.1016/j.socscimed.2019.112480.

7831. Chatterjee S, Carnahan RM, Chen H, Holmes HM, Johnson ML, Aparasu RR. Anticholinergic medication use and risk of pneumonia in elderly adults: a nested case–control study. Journal of the American Geriatrics Society. 2016;64(2):394-400.

7832. Chatterjee S, Hazra A. Overview of pharmacological research activities in North Eastern States of India in last 5 years. Proceedings of the Indian National Science Academy. 2018;84(1):301-8. doi: 10.16943/ptinsa/2018/49307.

7833. Chatterton M, Blenkinsopp A, Pollock K. Statins and the interface between patient and community

pharmacist. International Journal of Pharmacy Practice. 2006;14(4):255-62. doi: 10.1211/ijpp.14.4.0005.

7834. Chattopadhyay SK, Jacob V, Hopkins DP, Lansky A, Elder R, Cuellar AE, et al. Community Guide Methods for Systematic Reviews of Economic Evidence. American Journal of Preventive Medicine. 2022. doi: 10.1016/j.amepre.2022.10.015.

7835. Chattopadhyay SK, Jacob V, Hopkins DP, Lansky A, Elder R, Cuellar AE, et al. Community Guide Methods for Systematic Reviews of Economic Evidence. American Journal of Preventive Medicine. 2023;64(4):569-78. doi: 10.1016/j.amepre.2022.10.015.

7836. Chattopadhyay SK, Jacob V, Hopkins DP, Lansky A, Elder R, Cuellar AE, et al. Community Guide Methods for Systematic Reviews of Economic Evidence. American Journal of Preventive Medicine. 2023;64(4):569-78. doi: 10.1016/j.amepre.2022.10.015. PubMed PMID: WOS:000952501000001.

7837. Chattoraj S, Shi L, Sun CC. Profoundly improving flow properties of a cohesive cellulose powder by surface coating with nano-silica through comilling. Journal of Pharmaceutical Sciences. 2011;100(11):4943-52. doi: https://doi.org/10.1002/jps.22677.

7838. Chaturvedi RR, Etchegaray JM, Raaen L, Jackson J, Friedberg MW. Technology Isn't the Half of It: Integrating Electronic Health Records and Infusion Pumps in a Large Hospital. The Joint Commission Journal on Quality and Patient Safety. 2019;45(10):649-61. doi: https://doi.org/10.1016/j.jcjq.2019.07.006.

7839. Chau N, Chau K, Vauthier JC, Baumann M, Amara ME, Kabuth B, editors. IMPACTS OF SOCIOECONOMIC, FAMILY, SCHOOL, BEHAVIORAL AND MENTAL DIFFICULTIES ON INVOLVEMENT IN VIOLENCE IN BOYS AND GIRLS. International Psychological Applications Conference and Trends; 2013 Apr 26-28; Madrid, SPAIN2013.

7840. Chau S, Jansen A, Ven P, Hoogland P, Elders P, Hugtenburg J, et al. Clinical medication reviews in elderly patients with polypharmacy: a cross-sectional study on drug-related problems in the Netherlands. International Journal of Clinical Pharmacy. 2016;38(1):46-53. doi: 10.1007/s11096-015-0199-8. PubMed PMID: 112692492. Language: English. Entry Date: 20161223. Revision Date: 20190712. Publication Type: journal article.
[truncated: 524,194 more chars]
